# Supplementary material for: Comparative Proteomics and Metabonomics Analysis of Different Diapause Stages Revealed a New Regulation Mechanism of Diapause in Loxostege sticticalis (Lepidoptera: Pyralidae)
Source: Molecules. 2024 Jul 25;29(15):3472. doi: 10.3390/molecules29153472 (PMC11314584; doi:10.3390/molecules29153472)
Supplement: Supplementary file 1 [file molecules-29-03472-s001.zip › analysis process/proteomic/Protein library identification results.pdf]

Accession_id	Description	Peptides	AAs	MW[kDa]	calc.pI	Coverage	PSM	Unique Pe	Protein FD	Abundanc	Abundanc	Abundanc	Abundanc	Abundanc	Abundanc	Abundanc	Abundanc	Abundanc	Abundanc	Abundanc	Abundanc	Abundanc	Abundanc	Abundance_C53_JCZY3
TRINITY_DN248_c0_g1_i1_orf1	unnamed protein product [Chilo suppressalis]	254	9489	1059	6.34	34	375	208	High	1	1.014	1.013	1.014	1.027	1.042	0.977	0.962	0.957	0.91	0.834	0.919	0.502	0.505	0.513
TRINITY_DN1889_c0_g1_i1_orf1	titin isoform X2 [Ostrinia furnacalis]	191	5371	610.3	6.23	43	324	191	High	1	1.001	1.024	1.193	1.204	1.179	1.305	1.267	1.251	1.183	1.094	1.221	0.619	0.612	0.616
TRINITY_DN841_c0_g1_i4_orf1	uncharacterized protein LOC114354070 isoform X3 [Ostrinia furnacalis]	176	7739	888.6	5.58	25	227	1	High	1	1.228	0.987	0.824	0.888	1.385	0.953	1.206	1.235	1.166	1.087	1.061	0.782	0.728	0.762
TRINITY_DN841_c0_g1_i8_orf1	uncharacterized protein LOC114354070 isoform X3 [Ostrinia furnacalis]	176	7902	907.8	5.64	24	227	1	High	1	0.826	0.801	0.865	0.791	0.935	0.822	0.801	0.758	0.599	0.624	0.756	0.794	0.742	0.568
TRINITY_DN1306_c0_g1_i8_orf1	spectrin alpha chain isoform X4 [Pectinophora gossypiella]	106	2421	276.3	5.29	50	184	106	High	1	1.004	0.989	0.999	1.038	1.018	0.976	0.977	0.957	0.894	0.844	0.931	0.789	0.807	0.796
TRINITY_DN5080_c0_g1_i1_orf1	basic juvenile hormone-suppressible protein 2-like [Ostrinia furnacalis]	87	744	89.5	8.84	75	571	46	High	1	1.007	1.018	3.727	3.392	3.421	3.717	3.593	3.822	5.219	6.394	4.724	5.495	5.436	5.734
TRINITY_DN10430_c0_g1_i4_orf1	fatty acid synthase [Ostrinia furnacalis]	76	2396	255.9	6.77	42	133	75	High	1	0.982	1.005	1.262	1.269	1.25	1.446	1.379	1.428	1.094	1.053	1.146	0.908	0.914	0.899
TRINITY_DN501_c0_g1_i5_orf1	hypothetical protein evm_002550 [Chilo suppressalis]	90	17863	1895.1	5.48	8	104	90	High	1	1.016	1.019	0.991	0.989	0.954	0.977	1.024	0.985	0.988	0.869	0.987	2.024	2.082	2.01
TRINITY_DN19662_c0_g2_i1_orf1	storage protein [Ostrinia furnacalis]	51	497	59	8.19	69	385	43	High	1	0.985	1.003	3.132	2.935	2.959	3.189	3.167	3.275	4.398	5.731	3.992	4.232	4.157	4.454
TRINITY_DN4724_c0_g1_i4_orf1	paramyosin, long form isoform X1 [Manduca sexta]	59	876	102.5	5.59	59	135	59	High	1	1.015	1.039	0.923	0.954	0.928	0.897	0.888	0.874	0.857	0.825	0.868	0.492	0.465	0.491
TRINITY_DN1173_c0_g1_i11_orf1	obscurin [Ostrinia furnacalis]	72	3146	355.2	5.83	28	101	26	High	1	1.008	1.007	0.949	0.942	0.966	0.989	0.992	0.988	0.98	0.93	0.996	0.588	0.57	0.598
TRINITY_DN2468_c0_g1_i7_orf1	protein 4.1 homolog isoform X1 [Ostrinia furnacalis]	80	3029	329.3	8.48	34	99	79	High	1	1.018	1.017	1.237	1.3	1.276	1.161	1.211	1.157	1.083	0.984	1.179	0.982	1.018	0.991
TRINITY_DN1211_c0_g1_i10_orf1	spectrin beta chain-like isoform X7 [Spodoptera frugiperda]	68	2532	292.3	5.62	32	101	67	High	1	0.983	0.97	1.016	1.034	1.01	1.004	1.009	1.022	0.949	0.968	0.966	0.771	0.758	0.768
TRINITY_DN11817_c0_g1_i4_orf1	glycogen phosphorylase [Heortia vitessoides]	48	842	96.7	6.04	62	136	48	High	1	1.002	1.007	2.371	2.324	2.264	2.05	2.045	2.086	2.352	2.55	2.292	1.787	1.748	1.845
TRINITY_DN129869_c0_g4_i1_orf1	putative myosin heavy chain, muscle, partial [Cotesia chilonis]	53	1970	225.6	6.04	22	176	42	High	1	1.03	0.998	0.725	0.747	0.754	0.696	0.702	0.676	0.628	0.599	0.654	0.35	0.343	0.35
TRINITY_DN668_c0_g1_i4_orf1	fatty acid synthase-like isoform X1 [Ostrinia furnacalis]	56	2422	268.3	6.67	29	89	55	High	1	1.012	1.022	0.849	0.868	0.833	0.791	0.815	0.807	0.809	0.812	0.751	2.495	2.409	2.477
TRINITY_DN5080_c0_g1_i5_orf1	storage protein [Ostrinia furnacalis]	43	410	49.2	8.4	69	292	3	High	1	0.967	1.018	7.24	6.162	6.547	6.829	6.435	7.145	9.982	8.38	8.115	10.019	10.48	9.896
TRINITY_DN19662_c4_g1_i1_orf1	basic juvenile hormone-suppressible protein 1-like [Ostrinia furnacalis]	31	260	30.7	8.76	82	501	31	High	1	0.999	1.007	2.829	2.756	2.599	2.76	2.758	2.827	3.72	4.354	3.709	3.379	3.333	3.461
TRINITY_DN741_c0_g1_i1_orf1	talin-1 isoform X5 [Ostrinia furnacalis]	51	2719	292.3	6.38	25	75	1	High	1	1.073	1.183	1.254	1.289	1.257	1.292	1.478	1.416	1.096	1.883	1.371	1.158	1.071	1.324
TRINITY_DN741_c0_g1_i10_orf1	talin-1 isoform X13 [Ostrinia furnacalis]	52	3342	365.8	6.46	21	77	3	High	1	1.019	0.978	1.385	1.224	1.367	1.43	1.366	1.307	1.133	1.205	1.321	1.04	0.955	0.915
TRINITY_DN3991_c0_g1_i6_orf1	acetyl-CoA carboxylase isoform X3 [Trichoplusia ni]	56	2383	267.1	6.23	29	88	56	High	1	0.997	0.999	1.358	1.333	1.349	1.716	1.687	1.664	1.386	1.365	1.407	1.062	1.074	1.06
TRINITY_DN416_c0_g1_i1_orf1	unnamed protein product [Diatraea saccharalis]	46	900	103.8	5.73	55	101	42	High	1	1.012	1.005	1.101	1.129	1.09	0.981	0.977	0.957	0.865	0.82	0.909	0.471	0.47	0.489
TRINITY_DN1665_c1_g1_i2_orf1	translation elongation factor 2 [Melitaea cinxia]	40	844	94.5	6.64	52	129	29	High	1	1.003	1.019	0.835	0.773	0.825	0.682	0.664	0.706	0.605	0.569	0.572	0.61	0.601	0.611
TRINITY_DN2254_c0_g1_i4_orf1	vigilin [Ostrinia furnacalis]	52	1269	140.9	6.71	46	81	52	High	1	1.024	0.99	1.053	1.052	1.068	1	0.96	0.984	0.757	0.707	0.777	0.68	0.688	0.656
TRINITY_DN3800_c0_g1_i7_orf1	hypothetical protein evm_004893 [Chilo suppressalis]	42	1359	144.5	6.83	42	78	42	High	1	1.023	0.999	1.274	1.24	1.246	1.2	1.19	1.193	1.107	1.055	1.09	0.844	0.846	0.852
TRINITY_DN100327_c0_g1_i1_orf1	arylphorin subunit alpha-like [Ostrinia furnacalis]	42	693	83.5	6.9	56	159	35	High	1	0.967	0.966	4.166	3.207	3.802	3.009	2.614	3.287	4.999	5.332	3.414	3.338	3.32	3.307
TRINITY_DN511_c0_g2_i1_orf1	pyruvate carboxylase, mitochondrial isoform X1 [Manduca sexta]	44	1200	131.9	6.76	43	84	37	High	1	1.006	1.007	1.609	1.606	1.594	1.55	1.526	1.522	1.53	1.432	1.594	1.134	1.156	1.153
TRINITY_DN235_c0_g1_i2_orf1	unnamed protein product [Parnassius apollo]	29	400	44.3	5.71	70	215	5	High	1	0.976	0.994	0.917	0.925	0.901	0.881	0.921	0.866	0.782	0.747	0.784	0.473	0.476	0.467
TRINITY_DN7336_c0_g1_i13_orf1	PREDICTED: calcium-transporting ATPase sarcoplasmic/endoplasmic reticulum type isoform X2 [Amyelois transitella]	37	1000	109.8	5.41	37	97	37	High	1	1.001	0.999	0.451	0.426	0.465	0.388	0.383	0.41	0.357	0.375	0.345	0.281	0.279	0.281
TRINITY_DN2813_c0_g1_i7_orf1	arylphorin subunit alpha-like [Ostrinia furnacalis]	45	1209	139.8	5.94	36	97	6	High	1	0.948	0.914	5.597	5.919	5.637	4.702	4.772	4.363	2.949	2.478	3.257	4.864	4.959	5.029
TRINITY_DN1287_c0_g1_i5_orf1	probable chitinase 10 isoform X6 [Ostrinia furnacalis]	51	2944	327.2	6.25	24	71	51	High	1	1	1.023	1.681	1.69	1.585	2.057	2.074	1.984	2.186	2.185	2.323	2.373	2.317	2.285
TRINITY_DN41_c0_g1_i3_orf1	uncharacterized protein LOC114359035 isoform X3 [Ostrinia furnacalis]	43	1124	125.9	5.86	46	78	40	High	1	0.958	0.998	2.522	2.608	2.436	2.611	2.586	2.574	2.127	1.946	2.263	3.719	3.764	3.589
TRINITY_DN6325_c0_g1_i9_orf1	fructose-bisphosphate aldolase isoform X2 [Pieris brassicae]	33	390	42.3	8.75	82	133	5	High	1	0.969	0.946	2.072	2.165	1.984	2.462	2.571	2.441	2.254	3.857	2.393	1.553	1.611	1.618
TRINITY_DN41166_c0_g1_i1_orf1	arginine kinase isoform X1 [Ostrinia furnacalis]	34	359	40.2	6.32	73	187	34	High	1	1.002	0.999	0.907	0.95	0.916	0.795	0.809	0.786	0.746	0.715	0.785	0.645	0.645	0.647
TRINITY_DN6587_c0_g1_i3_orf1	phosphoribosylformylglycinamidine synthase isoform X2 [Ostrinia furnacalis]	34	1340	146.1	5.94	36	73	34	High	1	0.983	1.004	1.321	1.332	1.295	1.35	1.322	1.326	1.523	1.432	1.586	1.159	1.181	1.179
TRINITY_DN4434_c0_g1_i7_orf1	V-type proton ATPase catalytic subunit A [Ostrinia furnacalis]	29	617	68.1	5.29	62	84	29	High	1	1.003	0.983	0.399	0.388	0.43	0.425	0.434	0.44	0.443	0.446	0.432	0.419	0.413	0.406
TRINITY_DN1216_c0_g1_i4_orf1	bifunctional purine biosynthesis protein PURH isoform X1 [Ostrinia furnacalis]	29	593	64.4	7.47	56	77	26	High	1	0.962	0.948	1.572	1.6	1.525	1.954	1.934	1.863	1.755	1.688	1.829	1.438	1.439	1.474
TRINITY_DN174_c1_g1_i1_orf1	spectrin beta chain, non-erythrocytic 5-like, partial [Ostrinia furnacalis]	47	2074	234.9	6.19	25	57	47	High	1	0.996	1.014	0.96	0.955	0.961	0.947	0.986	0.983	0.882	0.908	0.91	0.988	0.97	0.982
TRINITY_DN68401_c1_g1_i1_orf1	endoplasmic reticulum chaperone BiP isoform X1 [Ostrinia furnacalis]	40	657	72.5	5.3	57	95	35	High	1	1.008	0.985	1.124	1.173	1.126	0.979	0.992	0.951	0.887	0.871	0.956	0.872	0.881	0.867
TRINITY_DN244_c1_g1_i5_orf1	C-1-tetrahydrofolate synthase, cytoplasmic isoform X3 [Ostrinia furnacalis]	34	929	99.9	7.81	42	80	27	High	1	1.042	1.014	1.613	1.586	1.599	2.263	2.163	2.159	2.048	1.909	2.015	1.74	1.745	1.762
TRINITY_DN812_c2_g1_i1_orf1	1,4-alpha-glucan-branching enzyme [Ostrinia furnacalis]	35	685	79.1	6.44	57	98	35	High	1	1.002	1.031	2.309	2.119	2.202	2.1	2.011	2.099	2.228	2.132	2.063	1.406	1.448	1.421
TRINITY_DN6580_c0_g1_i4_orf1	catalase [Ostrinia furnacalis]	32	550	61.4	8.46	63	117	32	High	1	1.004	0.986	1.741	1.656	1.689	2.291	2.169	2.156	1.989	1.782	1.906	1.604	1.655	1.617
TRINITY_DN9340_c0_g1_i4_orf1	sarcosine dehydrogenase, mitochondrial [Ostrinia furnacalis]	37	912	101.6	7.74	48	78	37	High	1	0.985	1.038	2.004	2.035	1.943	1.649	1.599	1.652	1.77	1.659	1.78	1.332	1.364	1.354
TRINITY_DN2813_c0_g1_i10_orf1	arylphorin subunit alpha-like [Ostrinia furnacalis]	39	1206	139.5	5.87	33	88	1	High	1	0.936	1.009	7.49	7.548	7.186	4.228	4.525	4.334	4.086	3.616	4.277	5.211	5.009	4.803
TRINITY_DN8944_c0_g1_i1_orf1	actin, clone 403 [Trichonephila clavata]	25	384	42.8	5.85	64	171	1	High	1	1.24	1.107	0.534	0.393	0.554	0.488	0.353	0.455	0.472	0.376	0.367	0.881	0.794	0.752
TRINITY_DN2936_c0_g1_i1_orf1	myosin heavy chain, non-muscle isoform X1 [Hyposmocoma kahamanoa]	45	1970	225.4	5.6	25	56	45	High	1	1.005	0.992	0.764	0.791	0.789	0.783	0.805	0.768	0.738	0.759	0.739	1.179	1.185	1.187
TRINITY_DN1173_c0_g1_i12_orf1	obscurin [Ostrinia furnacalis]	46	1303	143	5.34	39	57	1	High	1	0.97	0.851	0.624	0.771	0.754	0.733	0.737	0.744	0.597	0.575	0.755	0.54	0.612	0.506
TRINITY_DN6325_c0_g1_i8_orf1	unnamed protein product [Pieris macdunnoughi]	29	390	42.1	8.5	75	121	1	High	1	0.958	0.997	1.861	1.859	1.784	0.947	0.911	1.012	1.02	0.91	1.034	0.657	0.671	0.574
TRINITY_DN8405_c0_g1_i4_orf1	clathrin heavy chain isoform X1 [Ostrinia furnacalis]	35	1682	191.5	5.66	26	54	35	High	1	1.006	1.032	1.103	1.066	1.073	0.936	0.92	0.972	0.84	0.849	0.824	1.131	1.093	1.099
TRINITY_DN993_c0_g1_i7_orf1	apolipophorins-like [Ostrinia furnacalis]	27	612	66.5	7.27	59	74	27	High	1	0.98	0.991	1.736	1.739	1.677	1.924	1.966	1.939	3.037	3.241	3.069	4.022	4.015	4.062
TRINITY_DN2300_c0_g1_i1_orf1	ATP synthase subunit beta, mitochondrial isoform X4 [Ostrinia furnacalis]	22	516	55.2	5.4	64	91	16	High	1	0.967	0.979	0.658	0.636	0.686	0.643	0.618	0.624	0.657	0.631	0.635	0.571	0.564	0.578
TRINITY_DN3822_c0_g1_i7_orf1	hypothetical protein HF086_002539 [Spodoptera exigua]	28	425	47.1	7.99	60	99	28	High	1	0.99	1.001	2.329	2.286	2.247	2.34	2.246	2.331	2.624	2.418	2.642	2.728	2.73	2.667
TRINITY_DN25341_c0_g1_i1_orf1	heat shock protein 90 [Loxostege sticticalis]	34	718	82.7	5.11	49	82	32	High	1	0.998	0.979	0.526	0.544	0.54	0.513	0.524	0.509	0.489	0.455	0.518	0.676	0.683	0.687
TRINITY_DN32011_c0_g1_i1_orf1	protein disulfide-isomerase [Ostrinia furnacalis]	32	494	55.7	4.81	56	87	32	High	1	1.003	0.987	1.168	1.178	1.195	1.032	1.055	1.027	1.024	0.95	1.026	1.009	1.022	1.003
TRINITY_DN6483_c0_g1_i6_orf1	transketolase-like protein 2 isoform X1 [Ostrinia furnacalis]	29	622	66.8	7.46	56	83	29	High	1	0.971	0.963	1.711	1.704	1.668	1.829	1.776	1.72	1.569	1.553	1.691	1.267	1.307	1.289
TRINITY_DN8915_c0_g1_i3_orf1	filamin-A isoform X1 [Ostrinia furnacalis]	37	923	100.3	6.48	54	51	37	High	1	1.048	1.029	1.049	1.06	1.093	1.141	1.013	1.086	1.028	0.898	1.02	0.529	0.531	0.517
TRINITY_DN2338_c0_g1_i5_orf1	prophenoloxidase PPO1b [Ostrinia furnacalis]	26	684	78.8	7.28	49	62	25	High	1	1.007	1.005	1.659	1.687	1.674	1.6	1.603	1.571	1.843	1.835	1.871	1.467	1.439	1.457
TRINITY_DN235_c0_g3_i1_orf1	actin, muscle-type A2 [Bombyx mori]	27	376	41.8	5.48	73	167	4	High	1	0.952	0.988	0.685	0.701	0.688	0.65	0.645	0.63	0.579	0.546	0.579	0.862	0.863	0.865
TRINITY_DN1587_c0_g1_i4_orf1	spectrin beta chain [Ostrinia furnacalis]	44	2090	237.6	6.92	26	50	43	High	1	1.06	1.045	0.916	0.933	0.945	0.961	0.966	0.935	0.902	0.873	0.91	0.868	0.89	0.878
TRINITY_DN549_c0_g1_i14_orf1	titin-like [Ostrinia furnacalis]	41	2583	293.1	5.4	18	48	41	High	1	1.012	1.045	1.048	1.089	1.074	1.132	1.096	1.118	0.99	0.882	0.954	0.627	0.644	0.632
TRINITY_DN357_c0_g1_i8_orf1	trifunctional enzyme subunit alpha, mitochondrial [Ostrinia furnacalis]	29	763	82.3	9.32	42	67	29	High	1	0.994	1.009	0.927	0.922	0.896	0.808	0.798	0.811	0.835	0.805	0.86	0.765	0.768	0.779
TRINITY_DN8258_c0_g1_i5_orf1	papilin isoform X9 [Ostrinia furnacalis]	39	2624	281.4	4.5	20	53	28	High	1	1.017	0.991	1.449	1.508	1.472	1.418	1.529	1.429	1.34	1.221	1.429	1.151	1.204	1.152
TRINITY_DN1084_c0_g1_i2_orf1	ATP-citrate synthase [Ostrinia furnacalis]	30	668	73.6	8.06	49	58	26	High	1	0.955	0.991	1.318	1.332	1.296	1.455	1.48	1.486	1.584	1.653	1.64	1.18	1.133	1.166
TRINITY_DN2103_c0_g1_i1_orf1	probable aconitate hydratase, mitochondrial isoform X1 [Ostrinia furnacalis]	28	788	85.4	8.43	38	58	24	High	1	1.017	0.988	0.511	0.529	0.542	0.52	0.523	0.521	0.524	0.528	0.554	0.52	0.528	0.527
TRINITY_DN14701_c0_g1_i2_orf1	staphylococcal nuclease domain-containing protein 1 [Ostrinia furnacalis]	34	755	84.3	8.92	45	74	34	High	1	1	0.975	0.855	0.85	0.867	0.824	0.809	0.82	0.774	0.671	0.733	0.689	0.691	0.678
TRINITY_DN2919_c0_g1_i5_orf1	nidogen-1 [Ostrinia furnacalis]	29	1373	151	4.82	26	43	29	High	1	1.022	0.996	0.886	0.856	0.93	0.733	0.718	0.732	0.72	0.668	0.696	0.965	0.951	0.939
TRINITY_DN1772_c1_g2_i1_orf1	aldose reductase-like isoform X2 [Ostrinia furnacalis]	21	320	36.1	6.46	77	79	13	High	1	0.979	1.014	2.774	2.761	2.742	4.541	4.485	4.374	3.712	4.128	3.606	1.434	1.459	1.486
TRINITY_DN15382_c0_g1_i3_orf1	putative aldehyde dehydrogenase family 7 member A1 homolog [Spodoptera	21	536	57.4	6.93	51	54	21	High	1	1.017	1.004	1.542	1.46	1.458	1.273	1.21	1.244	1.527	1.437	1.472	1.411	1.406	1.438
TRINITY_DN29604_c0_g2_i2_orf1	neurofilament heavy polypeptide-like isoform X2 [Ostrinia furnacalis]	28	384	40.5	4.53	77	60	22	High	1	0.952	1.002	1.421	1.473	1.431	1.747	1.796	1.709	1.741	1.518	1.855	5.473	5.714	5.338
TRINITY_DN45598_c0_g1_i2_orf1	heat shock protein 60A-like [Ostrinia furnacalis]	25	572	60.8	5.86	53	46	24	High	1	1.001	1.004	0.512	0.515	0.532	0.564	0.579	0.537	0.512	0.525	0.518	0.491	0.486	0.496
TRINITY_DN5765_c0_g2_i3_orf1	unnamed protein product [Diatraea saccharalis]	37	1933	212.2	5.74	21	45	37	High	1	1.01	0.992	0.786	0.837	0.799	0.759	0.744	0.758	0.724	0.67	0.759	0.631	0.641	0.606
TRINITY_DN15812_c0_g1_i2_orf1	transferrin [Ostrinia furnacalis]	30	681	75.6	7.03	52	45	30	High	1	0.992	0.998	2.066	2.206	2.057	2.01	2.103	1.943	2.173	2.012	2.386	3.328	3.35	3.222
TRINITY_DN21506_c0_g1_i4_orf1	glutamate dehydrogenase, mitochondrial isoform X2 [Ostrinia furnacalis]	22	554	61.3	8.02	49	50	22	High	1	0.999	0.993	0.537	0.544	0.564	0.537	0.54	0.555	0.543	0.537	0.557	0.467	0.471	0.482
TRINITY_DN28654_c0_g1_i2_orf1	acidic juvenile hormone-suppressible protein 1-like [Ostrinia furnacalis]	20	180	21.3	6.96	78	186	10	High	1	0.931	1.002	1.587	1.574	1.516	1.758	1.605	1.779	2.487	6.247	2.56	1.821	1.792	1.939
TRINITY_DN1814_c0_g2_i1_orf1	titin-like, partial [Ostrinia furnacalis]	32	2061	230.9	4.65	21	38	32	High	1	1.001	1.024	1.197	1.255	1.248	1.259	1.219	1.235	1.129	0.978	1.149	0.582	0.588	0.549
TRINITY_DN248_c0_g1_i12_orf1	twitchin-like [Ostrinia furnacalis]	37	1982	226.6	5.5	21	50	3	High	1	0.942	0.962	1.072	1.201	1.22	0.675	0.713	0.713	0.835	0.727	0.871	0.41	0.406	0.448
TRINITY_DN2745_c0_g1_i4_orf1	Tubulin alpha-1 chain [Harpegnathos saltator]	18	472	52.7	5.11	45	51	1	High	1	1.046	0.981	1.6	1.788	1.668	1.414	1.418	1.571	1.475	1.545	1.658	1.492	1.368	1.442
TRINITY_DN41842_c0_g1_i2_orf1	importin-5 [Ostrinia furnacalis]	25	1093	121.8	4.82	30	36	25	High	1	0.973	0.959	0.644	0.654	0.646	0.612	0.624	0.604	0.625	0.624	0.625	0.636	0.626	0.644
TRINITY_DN2873_c0_g1_i7_orf1	transitional endoplasmic reticulum ATPase TER94 [Galleria mellonella]	26	805	89	5.47	37	44	26	High	1	0.984	0.981	1.06	1.097	1.058	0.911	0.92	0.883	0.78	0.757	0.829	0.88	0.873	0.876
TRINITY_DN3219_c0_g1_i6_orf1	non-specific lipid-transfer protein [Ostrinia furnacalis]	20	534	57.6	8.32	48	43	18	High	1	0.994	0.978	1.387	1.369	1.332	1.274	1.303	1.248	0.87	0.883	0.928	1.152	1.154	1.142
TRINITY_DN91_c0_g1_i9_orf1	ribosome-binding protein 1 isoform X8 [Helicoverpa armigera]	24	924	101.1	6.34	33	37	22	High	1	1.011	0.973	0.708	0.77	0.739	0.715	0.735	0.688	0.62	0.565	0.656	0.806	0.805	0.782
TRINITY_DN17759_c0_g1_i5_orf1	hypothetical protein evm_013530 [Chilo suppressalis]	25	992	108.7	8.05	39	43	25	High	1	1.016	0.999	1.718	1.738	1.683	1.496	1.502	1.489	1.512	1.396	1.563	1.041	0.998	1.121
TRINITY_DN592_c0_g1_i6_orf1	PDZ and LIM domain protein Zasp isoform X4 [Pectinophora gossypiella]	21	644	68.6	8.69	52	51	21	High	1	1.016	1.003	1.145	1.195	1.191	1.253	1.282	1.227	0.998	0.949	1.011	0.569	0.555	0.572
TRINITY_DN2803_c4_g1_i1_orf1	ornithine aminotransferase, mitochondrial isoform X2 [Ostrinia furnacalis]	25	408	44.7	7.68	68	81	25	High	1	0.967	0.986	2.223	2.234	2.124	1.807	1.825	1.853	2.214	2.262	2.199	3.068	2.973	3.07
TRINITY_DN40126_c0_g1_i1_orf1	aldehyde dehydrogenase X, mitochondrial-like [Ostrinia furnacalis]	24	424	45.5	5.71	65	52	19	High	1	1.027	1.056	2.548	2.457	2.545	2.646	2.666	2.723	2.472	2.749	2.345	2.102	2.007	2.107
TRINITY_DN3158_c0_g1_i5_orf1	neuroglian [Ostrinia furnacalis]	29	1246	138.8	6.11	31	41	29	High	1	0.968	0.952	1.339	1.308	1.322	1.176	1.16	1.155	1.107	0.985	1.11	1.264	1.308	1.246
TRINITY_DN1422_c0_g1_i4_orf1	unnamed protein product [Chilo suppressalis]	23	731	79.7	6.51	40	31	23	High	1	1.028	1.014	0.51	0.523	0.52	0.484	0.466	0.475	0.484	0.472	0.494	0.47	0.473	0.446
TRINITY_DN7560_c0_g1_i4_orf1	unnamed protein product [Chilo suppressalis]	37	4054	444	5.77	12	42	37	High	1	1.002	1.006	0.824	0.857	0.829	0.718	0.734	0.725	0.712	0.737	0.719	0.936	0.948	0.922
TRINITY_DN3805_c0_g1_i2_orf1	heat shock protein 89 [Glyphodes pyloalis]	31	784	89	5.14	37	54	29	High	1	0.966	0.97	0.924	0.989	0.949	0.871	0.883	0.851	0.732	0.707	0.809	0.838	0.846	0.844
TRINITY_DN280_c0_g1_i12_orf1	tubulin beta-1 chain-like [Leguminivora glycinivorella]	21	460	51.6	4.94	59	70	2	High	1	0.951	1.019	0.897	0.803	0.786	0.79	0.749	0.79	0.797	0.703	0.793	0.816	0.85	0.863
TRINITY_DN7991_c0_g1_i9_orf1	hypothetical protein evm_006720 [Chilo suppressalis]	19	413	47	10.21	45	70	19	High	1	1.033	1.043	0.724	0.73	0.726	0.671	0.644	0.676	0.604	0.565	0.609	0.602	0.591	0.584
TRINITY_DN19659_c1_g1_i1_orf1	elongation factor 1-gamma [Ostrinia furnacalis]	29	423	48	7.43	56	64	29	High	1	1.005	0.993	0.992	1.048	1.009	0.784	0.786	0.782	0.724	0.675	0.763	0.651	0.652	0.649

TRINITY_DN80560_c0_g1_i1_orf1	ATP synthase subunit alpha, mitochondrial [Ostrinia furnacalis]	23	552	59.4	9.11	45	83	23	High	1	1.004	0.994	0.441	0.42	0.472	0.442	0.438	0.451	0.453	0.45	0.443	0.382	0.388	0.394
TRINITY_DN280_c0_g1_i8_orf1	Tubulin beta-1 chain [Papilio xuthus]	20	451	50.6	4.87	53	61	4	High	1	1.018	0.949	0.884	0.894	0.873	0.829	0.881	0.904	0.79	0.932	0.882	2.209	2.277	2.127
TRINITY_DN32509_c0_g1_i3_orf1	heat shock 70 kDa protein 4 isoform X1 [Ostrinia furnacalis]	26	830	92.8	5.94	38	44	26	High	1	1.009	0.981	0.726	0.781	0.741	0.759	0.794	0.774	0.716	0.662	0.777	0.785	0.77	0.771
TRINITY_DN7335_c0_g1_i1_orf1	probable methylmalonate-semialdehyde dehydrogenase [acylating], mitochor	17	522	56.1	7.49	40	43	17	High	1	1.008	0.984	0.793	0.781	0.802	0.746	0.721	0.761	0.811	0.791	0.807	0.66	0.633	0.641
TRINITY_DN2433_c0_g1_i3_orf1	iron regulatory protein 1 [Manduca sexta]	28	891	97	6.51	35	42	27	High	1	0.989	0.972	0.989	1.003	0.971	0.906	0.901	0.914	1.026	0.987	1.06	0.82	0.812	0.828
TRINITY_DN63662_c0_g4_i1_orf1	polyadenylate-binding protein 1 [Ostrinia furnacalis]	21	611	68.2	9.47	40	42	21	High	1	1.01	0.991	0.397	0.407	0.43	0.386	0.4	0.397	0.351	0.363	0.356	0.49	0.495	0.493
TRINITY_DN53238_c1_g1_i5_orf1	isocitrate dehydrogenase [NADP] cytoplasmic [Ostrinia furnacalis]	25	408	46.2	7.21	57	51	24	High	1	0.961	1.001	1.094	1.026	1.048	1.143	1.104	1.202	1.113	1.057	1.002	0.932	0.922	0.943
TRINITY_DN5768_c0_g1_i2_orf1	adenosylhomocysteinase [Ostrinia furnacalis]	18	430	47.3	6.29	40	59	13	High	1	0.984	1.003	1.533	1.481	1.506	1.327	1.295	1.346	1.442	1.411	1.435	0.875	0.876	0.927
TRINITY_DN4998_c0_g1_i21_orf1	phenoloxidase subunit 2-like [Ostrinia furnacalis]	20	693	80	6.05	38	51	16	High	1	1.007	1.019	2.625	2.734	2.547	2.401	2.409	2.286	2.623	2.509	2.68	2.01	2.016	2.048
TRINITY_DN11274_c0_g1_i4_orf1	dipeptidyl peptidase 3 isoform X1 [Ostrinia furnacalis]	29	741	83.5	6.83	47	48	29	High	1	1.033	0.99	1.251	1.226	1.244	1.141	1.116	1.137	1.309	1.269	1.306	1.316	1.339	1.326
TRINITY_DN1209_c0_g1_i9_orf1	NADP-dependent malic enzyme-like isoform X1 [Ostrinia furnacalis]	25	644	70.7	7.39	41	54	25	High	1	0.974	1.028	2.175	2.155	2.12	2.295	2.268	2.273	3.447	3.188	3.597	1.434	1.742	1.481
TRINITY_DN5512_c0_g1_i8_orf1	peroxisomal multifunctional enzyme type 2-like isoform X1 [Ostrinia furnacalis	25	715	77.6	8.72	40	42	25	High	1	0.979	1.018	0.766	0.731	0.753	0.71	0.747	0.753	0.664	0.662	0.674	0.782	0.766	0.758
TRINITY_DN4381_c0_g2_i1_orf1	eukaryotic initiation factor 4A [Glyphodes caesalis]	21	419	47.4	5.4	53	48	20	High	1	0.984	1.022	0.789	0.783	0.792	0.664	0.657	0.67	0.633	0.629	0.63	0.718	0.713	0.724
TRINITY_DN21715_c0_g1_i1_orf1	protein disulfide-isomerase A3 isoform X1 [Ostrinia furnacalis]	22	490	54.9	5.59	47	51	22	High	1	1.044	1.028	1.568	1.567	1.576	1.504	1.496	1.448	1.42	1.236	1.443	1.344	1.411	1.363
TRINITY_DN2719_c1_g1_i6_orf1	unnamed protein product [Chrysodeixis includens]	24	782	85.2	7.23	36	39	24	High	1	1.01	1.018	1.334	1.3	1.294	1.079	1.058	1.092	0.922	0.862	0.912	0.839	0.819	0.812
TRINITY_DN48548_c0_g1_i1_orf1	glutathione S-transferase siama 2 [Heortia vitessoides]	20	203	23.2	8.82	60	102	18	High	1	0.942	0.972	3.205	3.182	3.028	3.223	3.271	3.259	3.156	5.226	3.296	2.824	2.649	3.221
TRINITY_DN24699_c0_g1_i3_orf1	E3 SUMO-protein ligase RanBP2-like [Ostrinia furnacalis]	31	2954	327.8	6.19	14	34	31	High	1	1.009	1.016	0.791	0.76	0.795	0.867	0.853	0.837	0.885	0.901	0.861	0.875	0.868	0.836
TRINITY_DN703_c13_g1_i1_orf1	acidic juvenile hormone-suppressible protein 1-like [Ostrinia furnacalis]	21	489	57.7	6.44	47	92	15	High	1	0.952	1.008	3.241	2.632	2.846	3.63	3.085	4.131	6.059	7.698	4.235	3.745	3.551	4.017
TRINITY_DN16125_c0_g1_i3_orf1	3-ketoacyl-CoA thiolase, mitochondrial [Ostrinia furnacalis]	15	396	41	8.68	53	32	15	High	1	1.033	1.013	0.987	0.936	0.934	0.99	0.944	0.99	1.043	0.988	1.069	0.749	0.786	0.758
TRINITY_DN5266_c0_g1_i1_orf1	malate dehydrogenase, cytoplasmic isoform X2 [Ostrinia furnacalis]	22	337	36.1	8.19	62	58	22	High	1	1.017	0.99	1.658	1.702	1.618	1.833	1.839	1.787	1.639	1.725	1.759	1.236	1.264	1.23
TRINITY_DN9498_c0_g1_i3_orf1	eukaryotic translation initiation factor 4 gamma 3-like isoform X2 [Ostrinia furr	29	1227	137	6.51	29	38	29	High	1	1.016	1.014	0.559	0.583	0.601	0.569	0.577	0.573	0.522	0.515	0.516	0.635	0.64	0.64
TRINITY_DN7394_c0_g2_i1_orf1	peptidyl-prolyl cis-trans isomerase-like [Ostrinia furnacalis]	9	165	17.6	8.29	72	48	9	High	1	1.005	0.995	0.92	0.976	0.918	0.965	0.968	0.924	0.912	0.87	0.99	0.861	0.89	0.874
TRINITY_DN1132_c0_g1_i5_orf1	unnamed protein product [Spodoptera exigua]	21	655	71.6	6.98	41	40	21	High	1	0.987	0.988	0.642	0.643	0.66	0.547	0.534	0.555	0.566	0.515	0.58	0.618	0.631	0.606
TRINITY_DN69_c0_g1_i1_orf1	glycerol-3-phosphate dehydrogenase [NAD(+)], cytoplasmic isoform X1 [Ostri	21	363	39.8	6.25	63	65	21	High	1	0.966	0.974	3.139	2.928	3.02	3.441	3.343	3.474	2.789	2.909	2.646	2.03	2.066	2.098
TRINITY_DN1206_c0_g1_i6_orf1	sorbitol dehydrogenase-like [Spodoptera frugiperda]	12	358	38.7	7.31	46	63	12	High	1	0.982	1.003	2.367	2.154	2.074	2.288	1.89	2.325	2.42	2.221	2.319	1.394	1.443	1.38
TRINITY_DN906_c0_g1_i4_orf1	uncharacterized protein LOC114360441, partial [Ostrinia furnacalis]	22	772	87.8	6.52	38	33	22	High	1	0.991	1.004	1.455	1.444	1.397	1.241	1.274	1.304	1.317	1.297	1.181	4.988	4.946	4.933
TRINITY_DN11178_c0_g1_i1_orf1	hypoxia up-regulated protein 1 [Ostrinia furnacalis]	23	915	102.1	5.35	29	39	23	High	1	1.002	0.994	0.906	0.937	0.924	0.783	0.81	0.818	0.672	0.671	0.749	0.809	0.823	0.793
TRINITY_DN13660_c0_g1_i1_orf1	Aliphatic nitrilase [Operophtera brumata]	17	393	44.5	6.62	50	49	17	High	1	0.989	1.018	2.665	2.704	2.6	2.685	2.654	2.627	3.095	3.144	3.287	1.917	1.884	1.987
TRINITY_DN664_c0_g1_i18_orf1	chitinase-like protein EN03 isoform X2 [Ostrinia furnacalis]	19	433	48	7.81	48	56	19	High	1	0.969	0.955	1.446	1.412	1.414	2.108	2.081	2.003	2.018	1.788	2.096	2.083	2.15	2.048
TRINITY_DN2378_c0_g1_i5_orf1	integrin alpha-8-like isoform X1 [Ostrinia furnacalis]	22	1456	157.6	5.78	21	31	22	High	1	0.959	1.025	1.592	1.691	1.58	1.795	1.825	1.767	1.506	1.463	1.621	1.205	1.221	1.246
TRINITY_DN37372_c0_g1_i1_orf1	laminin subunit beta-1 [Colias croceus]	28	1800	200.1	5.45	19	39	28	High	1	1.003	1.022	1.073	1.08	1.113	1.093	1.091	1.043	1.024	0.955	1.083	1.054	1.078	1.057
TRINITY_DN343_c0_g1_i5_orf1	glutamate synthase [NADH], amyloplastic [Ostrinia furnacalis]	31	2050	225.5	6.74	19	33	31	High	1	0.976	1.012	0.878	0.864	0.907	0.939	0.956	0.946	0.953	0.982	1	0.83	0.81	0.824
TRINITY_DN1366_c0_g1_i5_orf1	unnamed protein product, partial [Iphiclides podalirius]	16	494	54.8	5.4	45	33	16	High	1	0.995	0.983	0.292	0.299	0.337	0.32	0.325	0.317	0.312	0.337	0.315	0.302	0.29	0.314
TRINITY_DN5753_c0_g1_i10_orf1	ryanodine receptor [Ostrinia furnacalis]	31	5116	577.7	5.6	7	35	31	High	1	0.986	0.973	0.784	0.783	0.833	0.819	0.889	0.837	0.79	0.726	0.722	0.625	0.632	0.628
TRINITY_DN9286_c0_g1_i2_orf1	alcohol dehydrogenase class-3 [Ostrinia furnacalis]	16	376	40	7.37	43	47	16	High	1	1.038	1.058	1.126	1.064	1.057	1.368	1.221	1.344	1.226	1.119	1.156	0.671	0.674	0.657
TRINITY_DN7464_c1_g1_i1_orf1	T-complex protein 1 subunit theta [Ostrinia furnacalis]	22	545	59	5.3	45	37	22	High	1	1.018	1.01	0.635	0.632	0.652	0.662	0.635	0.659	0.627	0.606	0.628	0.749	0.764	0.759
TRINITY_DN11467_c0_g1_i5_orf1	27 kDa hemolymph protein-like, partial [Ostrinia furnacalis]	20	233	25.3	5.21	76	69	20	High	1	1.012	0.984	1.492	1.543	1.512	1.644	1.629	1.522	2.145	1.851	2.385	1.636	1.763	1.634
TRINITY_DN1103_c0_g1_i18_orf1	retinal dehydrogenase 1-like [Ostrinia furnacalis]	21	486	51.8	6.8	48	41	1	High	1	0.924	0.951	1.087	1.063	0.816	1.042	1.064	1.027	1.051	1.245	0.912	1.341	1.268	1.146
TRINITY_DN2993_c0_g1_i4_orf1	heat shock 70 kDa protein cognate 5 [Ostrinia furnacalis]	24	686	74.7	6.43	34	41	24	High	1	1.02	0.986	0.436	0.432	0.462	0.421	0.418	0.425	0.416	0.434	0.408	0.464	0.483	0.46
TRINITY_DN4779_c0_g1_i5_orf1	T-complex protein 1 subunit epsilon isoform X1 [Ostrinia furnacalis]	20	542	58.9	6.01	33	35	20	High	1	0.989	0.999	0.6	0.621	0.619	0.561	0.598	0.578	0.533	0.546	0.529	0.667	0.646	0.677
TRINITY_DN1790_c1_g1_i3_orf1	unnamed protein product [Diatraea saccharalis]	24	1037	118	7.44	24	34	24	High	1	0.972	1.002	0.805	0.792	0.822	0.687	0.693	0.699	0.705	0.708	0.721	0.712	0.724	0.727
TRINITY_DN9965_c0_g1_i1_orf1	dihydrolipoyl dehydrogenase [Ostrinia furnacalis]	17	495	52.4	8.76	42	40	17	High	1	0.985	1	0.732	0.691	0.754	0.702	0.688	0.713	0.709	0.668	0.685	0.581	0.59	0.578
TRINITY_DN19651_c0_g1_i1_orf1	cytosolic non-specific dipeptidase [Ostrinia furnacalis]	21	478	53.6	5.95	54	40	21	High	1	1.027	1.027	0.701	0.702	0.729	0.667	0.652	0.666	0.833	0.794	0.882	1.155	1.125	1.104
TRINITY_DN4301_c2_g2_i4_orf1	stress-induced-phosphoprotein 1-like [Ostrinia furnacalis]	24	543	61.7	6.92	48	37	24	High	1	1.038	0.991	0.776	0.782	0.812	0.866	0.878	0.848	0.788	0.712	0.812	0.868	0.905	0.851
TRINITY_DN1103_c0_g1_i12_orf1	retinal dehydrogenase 1-like [Ostrinia furnacalis]	21	486	51.9	6.57	45	40	3	High	1	1.043	1.027	1.212	1.248	1.246	1.673	1.468	1.557	1.491	1.352	1.467	1.333	1.379	1.319
TRINITY_DN1201_c0_g1_i4_orf1	triosephosphate isomerase [Ostrinia furnacalis]	16	248	26.4	5.69	71	66	16	High	1	1.019	1.023	2.784	2.757	2.708	2.577	2.587	2.558	2.604	2.394	2.588	2.148	2.197	2.185
TRINITY_DN36612_c0_g1_i1_orf1	lamin-C isoform X1 [Ostrinia furnacalis]	23	612	69.9	6.89	38	37	23	High	1	1.013	0.985	0.963	1.028	0.975	0.936	0.944	0.93	0.828	0.742	0.871	0.848	0.867	0.836
TRINITY_DN33619_c0_g1_i1_orf1	eukaryotic translation initiation factor 3 subunit C [Ostrinia furnacalis]	21	883	101.6	6.87	24	38	21	High	1	0.984	0.997	0.696	0.695	0.685	0.61	0.617	0.581	0.588	0.579	0.586	0.63	0.64	0.65
TRINITY_DN1404_c0_g1_i6_orf1	uncharacterized protein LOC114363065 [Ostrinia furnacalis]	16	779	81.1	8	40	23	15	High	1	0.98	1.016	1.351	1.284	1.202	1.227	1.216	1.255	1.196	1.248	1.161	0.886	0.841	0.921
TRINITY_DN1175_c1_g1_i2_orf1	methanethiol oxidase [Ostrinia furnacalis]	17	479	53.7	6.25	45	46	10	High	1	1.003	0.995	2.053	2.098	2.049	1.755	1.738	1.723	2.2	1.957	2.251	1.083	1.095	1.107
TRINITY_DN20_c0_g1_i11_orf1	plasma membrane calcium-transporting ATPase 2 [Ostrinia furnacalis]	21	1137	125	7.68	22	37	9	High	1	1.087	1.06	0.62	0.592	0.619	0.571	0.509	0.56	0.551	0.511	0.534	0.511	0.472	0.459
TRINITY_DN14436_c0_g1_i7_orf1	V-type proton ATPase subunit C [Vanessa cardui]	20	385	44.1	8.35	51	33	20	High	1	0.998	0.989	0.339	0.345	0.376	0.357	0.365	0.383	0.359	0.341	0.328	0.434	0.45	0.437
TRINITY_DN315_c0_g1_i1_orf1	tubulin alpha-1B chain [Rattus norvegicus]	12	451	50.1	5.06	34	32	1	High	1	0.859	0.819	1.333	3.155	2.234	2.254	1.6	1.593	1.279	2.403	3.976	1.338	1.634	1.121
TRINITY_DN4080_c0_g1_i8_orf1	AMP deaminase 2 isoform X3 [Ostrinia furnacalis]	21	760	87.3	6.33	35	36	21	High	1	1.016	1.021	1.492	1.453	1.448	2.653	2.474	2.536	1.989	1.836	1.966	1.476	1.491	1.491
TRINITY_DN2745_c0_g1_i2_orf1	PREDICTED: tubulin alpha-1A chain-like [Papilio polytes]	17	450	50.1	5.1	44	42	1	High	1	1.043	0.972	1.115	1.067	0.956	0.693	0.788	0.808	0.886	1	0.8	1.343	1.271	1.386
TRINITY_DN1103_c0_g1_i19_orf1	retinal dehydrogenase 1-like [Ostrinia furnacalis]	20	486	52.4	6.46	47	38	2	High	1	0.975	1.006	1.159	1.13	1.094	0.928	0.959	0.932	0.987	0.94	0.982	0.857	0.845	0.907
TRINITY_DN11409_c0_g1_i4_orf1	nodal modulator 1 [Ostrinia furnacalis]	23	1190	126.7	7.53	30	25	23	High	1	1.023	1.022	0.742	0.754	0.76	0.683	0.688	0.696	0.628	0.662	0.672	0.742	0.74	0.761
TRINITY_DN46173_c0_g3_i1_orf1	Tropomyosin, partial [Cotesia chilonis]	17	289	33.2	4.79	45	54	12	High	1	0.979	0.993	1.205	1.284	1.221	1.087	1.129	1.061	0.993	0.971	1.124	0.577	0.547	0.588
TRINITY_DN12767_c0_g1_i1_orf1	coatomer subunit alpha [Ostrinia furnacalis]	20	971	108.6	7.11	27	33	14	High	1	1.019	1.021	0.834	0.773	0.837	0.886	0.836	0.893	0.785	0.771	0.747	0.776	0.766	0.75
TRINITY_DN5704_c0_g1_i6_orf1	2-oxoglutarate dehydrogenase, mitochondrial isoform X2 [Ostrinia furnacalis]	19	877	98.3	7.17	29	29	18	High	1	0.991	0.99	0.538	0.521	0.539	0.51	0.52	0.524	0.542	0.53	0.549	0.472	0.474	0.494
TRINITY_DN1175_c1_g1_i1_orf1	methanethiol oxidase [Ostrinia furnacalis]	19	479	53.7	6.6	46	36	11	High	1	0.982	1.011	3.037	2.823	3.03	2.809	2.658	2.753	2.827	2.529	2.559	3.816	3.994	3.67
TRINITY_DN848_c0_g1_i1_orf1	actin-interacting protein 1 isoform X2 [Ostrinia furnacalis]	24	607	65.7	6.83	57	29	22	High	1	1.021	0.994	0.825	0.838	0.852	0.777	0.79	0.783	0.713	0.687	0.724	0.727	0.722	0.708
TRINITY_DN1455_c0_g1_i8_orf1	troponin T, skeletal muscle isoform X1 [Galleria mellonella]	23	357	43.1	5.01	41	74	5	High	1	0.977	1.006	1.214	1.321	1.264	1.057	1.13	1.034	0.944	0.872	1.109	0.348	0.355	0.386
TRINITY_DN726_c0_g1_i8_orf1	unnamed protein product [Chilo suppressalis]	19	573	62.3	6.92	38	30	17	High	1	0.968	0.967	1.327	1.424	1.382	1.144	1.274	1.208	1.237	1.206	1.314	1.13	1.102	1.125
TRINITY_DN3159_c0_g1_i4_orf1	uncharacterized protein LOC114362782 [Ostrinia furnacalis]	22	2898	323.1	4.67	10	28	22	High	1	0.992	0.978	0.21	0.214	0.241	0.235	0.251	0.238	0.243	0.24	0.263	0.294	0.286	0.277
TRINITY_DN1262_c0_g1_i2_orf1	alanine aminotransferase 1 isoform X1 [Ostrinia furnacalis]	17	527	58.5	7.61	43	28	17	High	1	1.025	1.02	1.02	1.006	1.029	1.071	1.08	1.106	0.949	1.039	0.928	0.662	0.657	0.685
TRINITY_DN56155_c0_g1_i1_orf1	hypothetical protein evm_004679 [Chilo suppressalis]	19	299	34.1	4.89	58	42	19	High	1	1.001	1.011	0.958	0.856	0.878	0.892	0.954	0.968	0.929	0.858	0.767	14.637	14.883	14.164
TRINITY_DN11948_c0_g1_i8_orf1	cystathionine gamma-lyase [Ostrinia furnacalis]	17	393	43	6.89	56	41	16	High	1	0.999	1.013	2.303	2.225	2.167	2.023	1.988	2.045	2.386	2.236	2.311	1.313	1.271	1.388
TRINITY_DN1161_c0_g1_i2_orf1	fructose-1,6-bisphosphatase 1 [Ostrinia furnacalis]	12	336	36.3	7.69	41	55	12	High	1	0.984	1.018	2.343	2.385	2.268	2.152	2.201	2.216	2.487	2.519	2.531	1.931	1.943	1.976
TRINITY_DN5149_c0_g1_i1_orf1	titin [Ostrinia furnacalis]	26	1892	218.1	6.44	18	35	24	High	1	0.999	0.967	1.103	1.155	1.087	1.014	1.026	1.007	0.998	0.873	1.029	0.866	0.878	0.853
TRINITY_DN3175_c0_g1_i7_orf1	unnamed protein product, partial [Brenthis ino]	18	324	36.2	7.3	70	43	18	High	1	0.988	1.008	1.945	2.072	1.879	2.053	2.173	2.031	1.905	1.974	2.092	1.538	1.551	1.591
TRINITY_DN2648_c0_g1_i8_orf1	dynein heavy chain, cytoplasmic isoform X8 [Ostrinia furnacalis]	27	3325	379.9	6.35	10	29	27	High	1	1.015	1.029	0.992	0.938	0.924	0.959	0.955	0.97	0.976	1.025	0.943	1.018	1.006	1.028
TRINITY_DN5177_c0_g1_i2_orf1	hemolin-like isoform X1 [Ostrinia furnacalis]	22	431	47.3	8.13	58	45	22	High	1	1.013	1.041	2.802	2.811	2.764	6.835	6.665	6.408	5.201	4.704	5.523	3.065	3.132	3.026
TRINITY_DN7854_c0_g1_i4_orf1	failed axon connections [Ostrinia furnacalis]	22	374	42.6	5.62	54	31	22	High	1	0.949	0.989	1.844	1.915	1.799	1.791	1.864	1.753	1.745	1.702	1.853	3.597	3.623	3.628
TRINITY_DN9_c0_g1_i7_orf1	heterogeneous nuclear ribonucleoprotein R isoform X1 [Ostrinia furnacalis]	18	524	58.9	6.81	40	35	2	High	1	0.987	1.036	1.698	1.613	1.605	1.251	1.236	1.293	1.104	1.133	1.016	1.225	1.243	1.219
TRINITY_DN20984_c0_g1_i4_orf1	NADPH--cytochrome P450 reductase isoform X2 [Ostrinia furnacalis]	19	540	61.5	7.15	37	31	19	High	1	0.977	0.986	0.673	0.681	0.701	0.722	0.751	0.745	0.564	0.536	0.59	0.633	0.625	0.608
TRINITY_DN4424_c0_g1_i1_orf1	calreticulin [Ostrinia furnacalis]	17	397	45.7	4.64	48	42	16	High	1	0.986	0.951	1.249	1.244	1.262	1.139	1.131	1.163	1.05	0.999	1.011	1.009	1.032	0.97
TRINITY_DN47389_c0_g1_i2_orf1	non-specific lipid-transfer protein-like [Ostrinia furnacalis]	12	410	43.9	8.13	38	19	10	High	1	1.017	1.005	0.409	0.412	0.422	0.537	0.532	0.513	0.385	0.447	0.409	0.357	0.365	0.387
TRINITY_DN5628_c0_g1_i5_orf1	hypothetical protein O3G_MSEX015036 [Manduca sexta]	16	339	36.3	8.4	55	34	8	High	1	0.974	0.989	1.541	1.616	1.488	1.62	1.505	1.434	1.395	1.214	1.591	0.663	0.629	0.614
TRINITY_DN2049_c1_g1_i3_orf1	luciferin 4-monooxygenase-like [Ostrinia furnacalis]	17	529	59.6	6.89	33	27	1	High	1	0.778	1.308	1.811	1.912	2.115	1.967	1.681	1.848	4.291	5.127	4.211	3.026	2.515	3.092
TRINITY_DN1437_c0_g1_i6_orf1	nucleoprotein TPR isoform X1 [Ostrinia furnacalis]	28	2221	247.3	5.14	14	29	28	High	1	1.017	0.997	0.684	0.683	0.683	0.624	0.647	0.631	0.589	0.552	0.619	0.837	0.858	0.83
TRINITY_DN688_c0_g1_i8_orf1	lysosomal alpha-mannosidase-like [Ostrinia furnacalis]	17	1103	126.4	5.72	20	24	15	High	1	1.015	1.034	1.642	1.654	1.598	1.492	1.558	1.537	1.659	1.6	1.636	2.969	2.979	2.888
TRINITY_DN21000_c0_g1_i1_orf1	elongation factor-1 alpha, partial [Loxostege sticticalis]	16	187	20.4	7.96	69	79	6	High	1	0.988	1.019	1.008	1.036	0.878	0.816	0.72	0.756	0.656	0.819	0.788	0.752	0.69	0.751
TRINITY_DN42269_c2_g1_i1_orf1	probable enoyl-CoA hydratase, mitochondrial [Ostrinia furnacalis]	15	297	32	8.7	50	44	15	High	1	1.002	0.97	0.571	0.585	0.589	0.679	0.69	0.675	0.709	0.686	0.715	0.599	0.626	0.617
TRINITY_DN8659_c0_g1_i1_orf1	ubiquitin-like modifier-activating enzyme 1 [Manduca sexta]	19	678	74.9	5.22	38	32	16	High	1	1.019	1.016	0.836	0.833	0.852	0.772	0.8	0.817	0.793	0.794	0.784	1.09	1.111	1.077
TRINITY_DN1455_c0_g1_i4_orf1	troponin domain-containing protein [Phthorimaea operculella]	20	289	34.6	8.53	48	63	2	High	1	0.945	1.052	1.656	1.668	1.641	1.594	1.669	1.443	1.517	1.353	1.656	1.026	0.972	1.002
TRINITY_DN46367_c0_g1_i2_orf1	T-complex protein 1 subunit zeta [Ostrinia furnacalis]	21	531	57.9	6.86	41	34	20	High	1	1.019	0.988	0.574	0.54	0.58	0.56	0.568	0.562	0.582	0.562	0.583	0.659	0.665	0.652
TRINITY_DN2049_c1_g1_i2_orf1	luciferin 4-monooxygenase-like [Ostrinia furnacalis]	17	529	59.6	6.67	32	26	1	High	1	0.966	0.636	1.464	1.955	1.827	1.05	0.933	1.035	1.291	1.29	0.827	1.367	0.887	1.207
TRINITY_DN20776_c0_g1_i3_orf1	hypothetical protein evm_003273 [Chilo suppressalis]	16	554	59.2	6.57	33	31	16	High	1	0.989	1.016	0.697	0.697	0.709	0.705	0.718	0.723	0.667	0.679	0.676	0.708	0.715	0.729
TRINITY_DN7570_c0_g1_i18_orf1	sodium/potassium-transporting ATPase subunit alpha isoform X4 [Trichoplusia	22	1036	114.6	5.64	27	28	13	High	1	1.005	1.009	1.299	1.412	1.256	0.821	0.888	0.871	0.743	0.734	0.758	0.896	0.896	0.896
TRINITY_DN15136_c0_g1_i2_orf1	alpha-aminoadipic semialdehyde synthase, mitochondrial isoform X3 [Ostrinia	21	931	102.9	7.18	28	33	21	High	1	0.994	1.012	1.202	1.217	1.216	1.124	1.185	1.127	1.121	1.094	1.167	0.843	0.861	0.882
TRINITY_DN2927_c0_g1_i2_orf1	unnamed protein product [Spodoptera littoralis]	19	436	47.6	7.47	44	31	19	High	1	0.968	1.001	0.638	0.658	0.673	0.643	0.667	0.658	0.604	0.621	0.613	0.749	0.709	0.745
TRINITY_DN5129_c0_g3_i3_orf1	probable citrate synthase 2, mitochondrial [Ostrinia furnacalis]	14	466	52	8.46	46	44	14	High	1	0.96	0.978	0.593	0.576	0.575	0.585	0.585	0.605	0.6	0.66	0.568	0.57	0.555	0.583
TRINITY_DN9_c0_g1_i11_orf1	heterogeneous nuclear ribonucleoprotein Q isoform X2 [Galleria mellonella]	18	517	58.5	6.58	41	34	2	High	1	0.957	1	0.976	1.018	0.918	0.809	0.895	0.904	0.969	0.951	0.847	1.54	1.647	1.669

TRINITY_DN211_c1_g1_i10_orf1	protein hu-li tai shao isoform X5 [Galleria mellonella]	19	723	80.1	6.71	36	29	19	High	1	0.995	0.961	0.774	0.764	0.765	0.718	0.72	0.71	0.627	0.659	0.646	0.624	0.628	0.614
TRINITY_DN585_c0_g1_i5_orf1	very low-density lipoprotein receptor isoform X2 [Galleria mellonella]	20	842	94.4	5.34	33	31	2	High	1	0.998	0.867	1.117	1.196	1.2	0.964	0.924	0.986	1.08	1.002	1.221	2.207	2.304	2.109
TRINITY_DN7247_c0_g1_i6_orf1	pyruvate kinase-like isoform X2 [Ostrinia furnacalis]	18	456	48.6	6.79	50	31	10	High	1	1	1.018	0.839	0.837	0.83	1.311	1.223	1.235	0.754	0.776	0.769	0.963	0.922	0.953
TRINITY_DN13368_c0_g1_i1_orf1	isoleucine--tRNA ligase, cytoplasmic [Ostrinia furnacalis]	25	1213	139.5	7.65	25	31	25	High	1	0.988	0.993	0.647	0.663	0.67	0.566	0.607	0.594	0.599	0.588	0.564	0.574	0.603	0.58
TRINITY_DN1173_c1_g1_i9_orf1	hypothetical protein evm_001011 [Chilo suppressalis]	21	1463	160.4	5.62	18	22	1	High	1	0.946	0.928	0.496	0.426	0.475	0.503	0.462	0.442	0.398	0.385	0.423	0.5	0.319	0.297
TRINITY_DN585_c0_g1_i12_orf1	very low-density lipoprotein receptor isoform X3 [Galleria mellonella]	21	903	100.3	5.22	31	33	3	High	1	1.048	0.994	2.298	2.506	2.057	1.99	2.822	2.361	2.246	2.36	2.216	2.918	2.897	2.99
TRINITY_DN20294_c0_g2_i1_orf1	cytochrome b-c1 complex subunit 2, mitochondrial isoform X1 [Ostrinia furnac	17	439	45.7	8.92	43	32	17	High	1	1.003	0.98	0.404	0.408	0.428	0.368	0.363	0.378	0.388	0.366	0.394	0.412	0.416	0.4
TRINITY_DN136358_c0_g1_i1_orf1	acidic juvenile hormone-suppressible protein 1-like [Ostrinia furnacalis]	8	84	9.7	6.52	55	117	2	High	1	1.026	1.049	2.347	2.642	2.419	2.786	2.848	2.675	3.728	7.277	4.406	2.611	2.637	2.638
TRINITY_DN3534_c0_g1_i2_orf1	guanine nucleotide-binding protein subunit beta-like protein [Ostrinia furnaca	17	319	35.9	7.74	66	37	8	High	1	0.951	1.011	0.592	0.537	0.576	0.558	0.61	0.571	0.472	0.473	0.49	0.513	0.507	0.518
TRINITY_DN1233_c0_g2_i1_orf1	unnamed protein product [Spodoptera exigua]	29	4571	505.8	5.31	7	31	29	High	1	1.045	1.066	0.901	0.962	0.936	0.86	0.877	0.874	0.874	0.816	0.878	1.33	1.362	1.309
TRINITY_DN21251_c1_g1_i1_orf1	60S ribosomal protein L4 [Ostrinia furnacalis]	17	417	46.5	11.62	34	45	17	High	1	0.999	0.988	0.703	0.724	0.756	0.698	0.668	0.686	0.585	0.534	0.593	0.598	0.627	0.597
TRINITY_DN1348_c0_g1_i1_orf1	hypothetical protein evm_002665 [Chilo suppressalis]	17	709	80.2	8.54	28	26	17	High	1	1.022	0.999	0.534	0.554	0.572	0.533	0.543	0.531	0.508	0.505	0.527	0.543	0.547	0.55
TRINITY_DN1756_c0_g1_i3_orf1	UTP--glucose-1-phosphate uridylyltransferase isoform X2 [Ostrinia furnacalis]	17	513	57.6	8.06	43	35	17	High	1	1.006	1.019	1.452	1.431	1.442	1.55	1.599	1.622	1.441	1.413	1.372	1.367	1.345	1.381
TRINITY_DN1103_c0_g1_i15_orf1	retinal dehydrogenase 1-like [Ostrinia furnacalis]	21	486	52.7	6.24	51	35	3	High	1	1.004	1.093	1.084	0.934	1.047	0.914	0.983	1.021	1.035	1.019	0.987	0.763	0.855	0.85
TRINITY_DN12865_c0_g1_i1_orf1	synaptic vesicle membrane protein VAT-1 homolog-like [Ostrinia furnacalis]	17	461	50.1	5.99	41	33	17	High	1	0.99	0.993	2.038	1.941	1.901	1.716	1.674	1.808	1.939	1.902	1.947	3.193	3.166	3.149
TRINITY_DN107708_c0_g1_i1_orf1	elongation factor 1-beta' [Pectinophora gossypiella]	15	223	24.5	4.77	73	37	15	High	1	0.988	1.012	0.996	0.993	0.964	0.92	0.935	0.901	0.845	0.827	0.858	0.782	0.774	0.773
TRINITY_DN39813_c0_g1_i1_orf1	nucleoside diphosphate kinase [Ostrinia furnacalis]	11	176	19.8	7.43	74	49	11	High	1	0.987	0.975	0.891	0.833	0.896	0.815	0.809	0.829	0.943	1.085	0.887	0.737	0.736	0.774
TRINITY_DN10889_c0_g1_i8_orf1	four and a half LIM domains protein 2 isoform X7 [Pectinophora gossypiella]	19	344	39.3	7.81	58	39	19	High	1	1.013	1.055	1.751	1.777	1.782	1.953	1.907	1.885	1.656	1.555	1.753	0.65	0.686	0.675
TRINITY_DN5432_c1_g1_i3_orf1	electron transfer flavoprotein-ubiquinone oxidoreductase, mitochondrial [Ostr	18	605	66	6.86	38	24	18	High	1	1.011	0.998	1.202	1.191	1.19	0.897	0.836	0.906	0.876	0.878	0.872	0.829	0.819	0.806
TRINITY_DN1173_c1_g1_i10_orf1	hypothetical protein evm_001011 [Chilo suppressalis]	21	1546	169.8	5.73	17	22	1	High	1	0.941	1.063	0.884	1.085	0.93	0.647	0.77	0.702	0.625	0.524	0.865	0.32	0.352	0.356
TRINITY_DN1868_c0_g1_i1_orf1	protein obstructor-E isoform X1 [Ostrinia furnacalis]	16	276	30.6	5.07	68	40	16	High	1	1.046	1.011	0.422	0.396	0.422	0.482	0.482	0.512	0.591	0.582	0.535	2.199	2.244	2.086
TRINITY_DN6415_c0_g2_i1_orf1	D-arabinitol dehydrogenase 1-like [Ostrinia furnacalis]	18	341	37.5	7.8	61	36	13	High	1	0.995	1.063	2.875	2.605	2.719	4.656	4.266	4.382	3.195	3.091	2.91	1.304	1.267	1.383
TRINITY_DN36788_c0_g1_i2_orf1	isocitrate dehydrogenase [NADP] cytoplasmic-like [Bicyclus anynana]	19	436	49.2	7.71	49	32	18	High	1	0.969	0.974	0.628	0.627	0.628	0.54	0.56	0.553	0.496	0.477	0.496	0.708	0.67	0.717
TRINITY_DN1180_c0_g1_i4_orf1	larval cuticle protein LCP-30-like [Ostrinia furnacalis]	13	307	31	6.11	61	53	13	High	1	1.006	1.015	1.407	1.546	1.399	1.399	1.486	1.345	1.159	1.066	1.423	0.247	0.225	0.246
TRINITY_DN4237_c1_g1_i5_orf1	eukaryotic translation initiation factor 3 subunit A-like isoform X1 [Ostrinia furr	24	1185	138.9	8.98	19	42	24	High	1	1.011	0.98	0.622	0.641	0.645	0.548	0.579	0.554	0.5	0.474	0.536	0.599	0.606	0.61
TRINITY_DN21123_c0_g1_i1_orf1	maternal protein exuperantia [Ostrinia furnacalis]	12	421	45.9	7.21	44	25	12	High	1	0.981	0.986	0.74	0.793	0.737	0.69	0.76	0.696	0.612	0.648	0.67	0.714	0.657	0.761
TRINITY_DN31342_c2_g2_i1_orf1	26S proteasome non-ATPase regulatory subunit 2 isoform X2 [Ostrinia furnaca	19	935	102	5.62	27	23	19	High	1	0.993	0.973	1.207	1.185	1.184	0.943	0.99	0.988	0.958	0.971	0.966	1.159	1.131	1.199
TRINITY_DN5721_c0_g1_i5_orf1	fumarate hydratase, mitochondrial-like isoform X2 [Ostrinia furnacalis]	11	478	51.5	7.44	34	21	11	High	1	1.036	1.004	0.704	0.691	0.725	0.695	0.736	0.717	0.773	0.826	0.764	0.649	0.626	0.637
TRINITY_DN5564_c0_g1_i1_orf1	probable phosphoserine aminotransferase [Ostrinia furnacalis]	15	339	37.1	7.55	53	30	11	High	1	1.055	1.072	1.064	1.053	1.048	1.278	1.227	1.191	1.25	1.278	1.197	1.063	1.064	1.112
TRINITY_DN5756_c0_g1_i4_orf1	leucine--tRNA ligase, cytoplasmic [Ostrinia furnacalis]	22	1176	134.2	7.5	21	26	22	High	1	0.996	1.017	0.781	0.771	0.785	0.692	0.703	0.722	0.664	0.649	0.641	0.667	0.669	0.65
TRINITY_DN1725_c0_g1_i7_orf1	T-complex protein 1 subunit gamma isoform X1 [Ostrinia furnacalis]	17	543	59.3	6.64	38	31	17	High	1	0.971	0.977	0.622	0.633	0.623	0.625	0.645	0.629	0.53	0.547	0.573	0.72	0.689	0.729
TRINITY_DN28299_c0_g1_i1_orf1	adenylosuccinate lyase isoform X1 [Ostrinia furnacalis]	14	492	54.9	7.11	31	24	14	High	1	1.021	1.019	1.578	1.437	1.527	1.77	1.63	1.831	1.962	1.917	1.745	1.242	1.258	1.266
TRINITY_DN11152_c0_g1_i8_orf1	ubiquitin carboxyl-terminal hydrolase 5 [Ostrinia furnacalis]	19	798	88.9	5.63	32	25	19	High	1	1.019	0.965	1.05	1.021	1.08	1.008	0.97	0.982	0.974	0.909	0.881	1.086	1.125	1.093
TRINITY_DN1068_c0_g1_i3_orf1	aspartate aminotransferase, cytoplasmic [Ostrinia furnacalis]	18	409	46.3	6.92	56	41	18	High	1	0.976	0.983	1.278	1.249	1.243	1.263	1.257	1.259	1.261	1.336	1.337	0.938	0.898	0.939
TRINITY_DN18539_c0_g1_i1_orf1	pupal cuticle protein PCP52-like [Ostrinia furnacalis]	13	267	28.3	5.3	54	43	13	High	1	0.983	0.989	0.894	0.855	0.887	0.964	0.997	0.991	1.001	0.994	0.92	6.602	7.003	6.263
TRINITY_DN73923_c0_g1_i1_orf1	protein obstructor-E-like [Ostrinia furnacalis]	15	238	26.5	5.02	65	22	15	High	1	1.037	1.014	0.257	0.26	0.29	0.296	0.311	0.292	0.286	0.263	0.328	1.849	1.855	1.793
TRINITY_DN42753_c0_g1_i2_orf1	26S proteasome regulatory subunit 8 [Ostrinia furnacalis]	20	402	45.3	8.41	61	35	19	High	1	0.974	1.017	1.066	1.065	1.064	0.916	0.977	0.959	0.971	1.012	0.944	1.151	1.13	1.206
TRINITY_DN842_c0_g1_i9_orf1	hypothetical protein evm_011651 [Chilo suppressalis]	19	3279	369.5	6.3	7	19	19	High	1	0.904	1.006	1.538	1.563	1.323	1.171	1.084	1.099	0.992	0.992	0.998	0.957	0.936	0.963
TRINITY_DN58751_c0_g1_i2_orf1	FK506-binding protein 2 isoform X1 [Vanessa tameamea]	13	237	26.2	4.72	56	49	13	High	1	1.009	1.016	1.928	2.005	1.934	1.919	1.838	1.794	1.567	1.43	1.697	1.513	1.558	1.475
TRINITY_DN4410_c0_g1_i1_orf1	rab GDP dissociation inhibitor alpha [Ostrinia furnacalis]	17	443	50	5.43	49	38	17	High	1	1.02	1.041	1.009	1.01	0.991	1.043	1.003	1.037	1.11	1.057	1.075	1.278	1.293	1.293
TRINITY_DN2338_c0_g2_i2_orf1	prophenoloxidase PPO3 [Ostrinia furnacalis]	18	697	79.9	6.79	32	30	2	High	1	1.01	0.978	0.746	0.768	0.763	0.813	0.853	0.797	0.894	0.867	1.003	0.689	0.708	0.741
TRINITY_DN30638_c0_g1_i1_orf1	alanine--tRNA ligase, cytoplasmic [Ostrinia furnacalis]	20	967	107.4	6.02	21	31	19	High	1	0.939	0.94	0.622	0.633	0.646	0.59	0.587	0.589	0.546	0.53	0.555	0.613	0.605	0.602
TRINITY_DN3784_c0_g1_i1_orf1	pancreatic triacylglycerol lipase-like [Ostrinia furnacalis]	14	537	61.9	5.34	30	38	13	High	1	0.989	0.978	0.103	0.1	0.141	0.14	0.154	0.149	0.157	0.159	0.153	0.181	0.188	0.176
TRINITY_DN129_c0_g1_i6_orf1	xanthine dehydrogenase [Ostrinia furnacalis]	18	1354	149.9	7.28	18	22	18	High	1	1.018	1.031	1.004	1.041	0.997	0.79	0.807	0.804	0.898	0.907	1.008	0.913	0.91	0.931
TRINITY_DN12387_c0_g1_i1_orf1	repetitive proline-rich cell wall protein 2-like [Ostrinia furnacalis]	19	368	40.7	9.04	64	41	18	High	1	0.968	0.997	0.972	0.994	0.997	0.914	0.961	0.951	1.004	0.924	0.974	4.822	4.931	4.61
TRINITY_DN89711_c0_g1_i1_orf1	26S proteasome non-ATPase regulatory subunit 1 [Ostrinia furnacalis]	15	1004	110.7	5.45	17	23	15	High	1	0.987	1.002	0.978	0.996	0.982	0.911	0.922	0.891	0.899	0.847	0.9	1.003	1.009	1.028
TRINITY_DN2559_c0_g1_i4_orf1	uricase [Ostrinia furnacalis]	17	340	38.2	7.97	60	33	17	High	1	0.969	1.031	1.672	1.65	1.564	1.345	1.249	1.349	1.219	1.211	1.217	0.789	0.749	0.802
TRINITY_DN5243_c1_g1_i1_orf1	14-3-3 protein epsilon [Maniola hyperantus]	13	262	29.8	4.81	51	40	5	High	1	1.026	1.001	0.918	0.965	0.964	0.918	0.985	0.954	0.848	0.791	0.852	0.939	0.969	1
TRINITY_DN5508_c0_g1_i1_orf1	tubulin beta chain [Plutella xylostella]	16	447	49.9	4.83	38	50	2	High	1	0.967	0.979	0.933	1.035	0.933	1.008	1.059	0.939	0.913	0.915	0.965	1.097	1.155	1.186
TRINITY_DN3985_c0_g2_i1_orf1	hypothetical protein evm_012077 [Chilo suppressalis]	19	299	34.4	9.86	51	57	15	High	1	0.954	0.968	0.726	0.829	0.735	0.684	0.698	0.67	0.61	0.661	0.692	0.635	0.605	0.652
TRINITY_DN1008_c0_g1_i2_orf1	integrin beta-PS [Ostrinia furnacalis]	15	831	91.2	5.76	29	20	15	High	1	0.972	1.041	1.853	1.828	1.878	1.857	1.696	1.8	1.456	1.442	1.445	1.258	1.31	1.187
TRINITY_DN25633_c0_g1_i1_orf1	puromycin-sensitive aminopeptidase isoform X1 [Ostrinia furnacalis]	20	948	107.6	6.46	25	30	20	High	1	1.04	1.02	0.97	0.888	0.96	0.966	0.943	0.99	0.97	0.907	0.851	1.034	1.02	1.007
TRINITY_DN9101_c0_g2_i1_orf1	60S ribosomal protein L7a [Ostrinia furnacalis]	19	268	30.3	10.52	50	47	19	High	1	0.962	0.977	0.666	0.7	0.661	0.586	0.589	0.568	0.523	0.517	0.607	0.552	0.554	0.58
TRINITY_DN30178_c0_g1_i3_orf1	LOW QUALITY PROTEIN: fibrillin-2-like [Bicyclus anynana]	19	3040	326.4	4.91	8	21	19	High	1	1.053	1.018	0.865	1.025	0.832	0.848	1.03	0.873	0.746	0.803	0.778	1.181	1.132	1.125
TRINITY_DN6822_c0_g2_i4_orf1	gelsolin-like [Bicyclus anynana]	14	401	44.3	7.9	45	26	11	High	1	1.013	0.976	1.969	2.01	1.938	1.517	1.562	1.518	1.569	1.394	1.644	1.718	1.766	1.697
TRINITY_DN3442_c0_g1_i4_orf1	UDP-glucose:glycoprotein glucosyltransferase [Ostrinia furnacalis]	22	1567	177.2	5.76	15	32	22	High	1	0.973	0.983	1.089	1.054	1.08	1.064	1.036	1.056	0.996	0.983	0.932	1.027	1.027	0.987
TRINITY_DN9234_c0_g1_i5_orf1	glutathione S-transferase 1-like [Ostrinia furnacalis]	14	215	24.2	7.93	68	35	14	High	1	0.979	0.987	0.883	0.828	0.878	0.803	0.78	0.815	0.925	0.928	0.836	0.867	0.838	0.875
TRINITY_DN9239_c0_g1_i1_orf1	apolipophorins-like [Ostrinia furnacalis]	14	157	18	6.23	78	43	10	High	1	0.917	0.964	1.576	1.36	1.356	1.639	1.581	1.877	3.226	3.1	2.577	4.499	4.313	4.963
TRINITY_DN2807_c0_g1_i4_orf1	FK506-binding protein 59 isoform X1 [Ostrinia furnacalis]	17	473	52.8	5.58	47	21	17	High	1	1.029	1.009	0.496	0.54	0.544	0.448	0.435	0.451	0.433	0.408	0.417	0.592	0.571	0.551
TRINITY_DN655_c0_g1_i3_orf1	moesin/ezrin/radixin homolog 1 isoform X2 [Bombyx mori]	20	579	67.3	6.27	32	42	20	High	1	1.011	1	0.828	0.802	0.832	0.734	0.746	0.768	0.717	0.709	0.722	0.826	0.829	0.823
TRINITY_DN11013_c0_g1_i3_orf1	glutamine:fructose-6-phosphate aminotransferase 1 [Heortia vitessoides]	15	674	75.4	6.57	29	21	15	High	1	0.968	1.021	0.48	0.487	0.514	0.479	0.471	0.493	0.456	0.467	0.395	0.604	0.582	0.592
TRINITY_DN799_c0_g1_i7_orf1	hypothetical protein evm_002571 [Chilo suppressalis]	18	289	30.5	10.8	60	47	18	High	1	0.969	0.969	0.687	0.731	0.708	0.608	0.606	0.605	0.509	0.475	0.535	0.587	0.604	0.589
TRINITY_DN29873_c0_g1_i1_orf1	glyceraldehyde-3-phosphate dehydrogenase 2 [Pectinophora gossypiella]	16	332	35.3	7.93	59	39	16	High	1	0.961	1	1.431	1.533	1.383	1.391	1.411	1.395	1.578	1.601	1.697	1.248	1.169	1.263
TRINITY_DN2442_c0_g1_i2_orf1	digestive cysteine proteinase 2 [Ostrinia furnacalis]	18	552	62.9	6.68	37	35	16	High	1	0.986	1.004	1.824	1.744	1.708	1.431	1.487	1.479	1.474	1.381	1.382	3.36	3.427	3.312
TRINITY_DN276_c0_g1_i1_orf1	protein lethal(2)essential for life-like [Helicoverpa zea]	13	188	21.3	6.54	65	35	5	High	1	0.953	0.98	1.381	1.405	1.34	2.998	2.924	2.835	3.246	2.952	3.73	2.134	2.125	1.96
TRINITY_DN1081_c0_g1_i7_orf1	3-ketoacyl-CoA thiolase, mitochondrial-like [Ostrinia furnacalis]	14	400	41.8	8.06	40	21	14	High	1	1.012	0.975	0.503	0.517	0.543	0.631	0.616	0.632	0.556	0.532	0.581	0.523	0.521	0.503
TRINITY_DN2019_c0_g1_i4_orf1	sushi, von Willebrand factor type A, EGF and pentraxin domain-containing pro	19	3573	389	5.17	8	20	19	High	1	0.979	1.015	0.755	0.75	0.741	0.778	0.758	0.763	0.726	0.78	0.701	0.914	0.907	0.871
TRINITY_DN1880_c0_g1_i4_orf1	serine protease inhibitor dipetalogastin-like [Helicoverpa zea]	14	385	42.6	8.35	52	25	12	High	1	0.998	0.989	3.832	3.761	3.729	3.798	3.587	3.498	4.561	4.117	4.981	1.667	1.631	1.648
TRINITY_DN1831_c0_g1_i3_orf1	hypothetical protein O3G_MSEX014886 [Manduca sexta]	12	425	46.4	5.39	36	27	12	High	1	1.032	1.021	0.834	0.832	0.855	0.798	0.792	0.823	0.826	0.896	0.873	1.053	1.04	0.991
TRINITY_DN12767_c0_g1_i2_orf1	coatomer subunit alpha [Ostrinia furnacalis]	15	669	73.6	6.21	29	23	9	High	1	0.976	0.995	0.858	0.792	0.851	0.887	0.838	0.853	0.755	0.777	0.792	0.969	0.929	0.937
TRINITY_DN948_c0_g1_i1_orf1	mitochondrial-processing peptidase subunit beta [Ostrinia furnacalis]	13	338	37.5	5.68	44	22	13	High	1	0.997	0.984	0.417	0.387	0.435	0.386	0.374	0.382	0.373	0.34	0.379	0.408	0.407	0.404
TRINITY_DN41645_c0_g1_i1_orf1	60S acidic ribosomal protein P2 [Ostrinia furnacalis]	8	92	9.4	4.56	80	32	1	High	1	0.848	0.873	1.138	1.225	1.117	1.3	1.445	1.378	0.761	0.66	0.831	0.817	0.811	0.777
TRINITY_DN5628_c0_g1_i3_orf1	muscle LIM protein Mlp84B isoform X2 [Ostrinia furnacalis]	12	307	33	8.18	57	30	4	High	1	0.974	1.066	1.477	1.538	1.503	1.621	1.65	1.527	1.459	1.311	1.501	0.81	0.876	0.959
TRINITY_DN17913_c0_g1_i8_orf1	fumarylacetoacetase [Ostrinia furnacalis]	13	423	47.4	6.2	41	21	12	High	1	0.991	0.968	1.12	1.079	1.101	1.219	1.194	1.199	1.321	1.336	1.322	1.124	1.138	1.101
TRINITY_DN760_c1_g2_i6_orf1	ADP,ATP carrier protein [Pieris napi]	21	301	33	9.79	57	47	21	High	1	0.912	0.953	0.388	0.385	0.404	0.352	0.363	0.376	0.369	0.534	0.369	0.357	0.34	0.377
TRINITY_DN5262_c0_g1_i7_orf1	T-complex protein 1 subunit beta [Ostrinia furnacalis]	16	536	57.8	6.43	31	23	16	High	1	1.013	1.017	0.626	0.643	0.619	0.553	0.571	0.576	0.543	0.543	0.564	0.688	0.666	0.671
TRINITY_DN4279_c0_g1_i6_orf1	glutathione S-transferase sigma 1 [Ostrinia furnacalis]	14	204	23.1	6.35	54	34	2	High	1	1.074	0.966	1.119	0.871	1.009	1.138	1.081	1.257	1.573	1.658	1.098	1.102	1.063	1.042
TRINITY_DN241_c0_g2_i3_orf1	amidophosphoribosyltransferase-like isoform X2 [Ostrinia furnacalis]	13	504	54.5	8.19	36	27	10	High	1	1	1.012	1.193	1.21	1.145	1.166	1.149	1.158	1.15	1.122	1.161	1.174	1.174	1.142
TRINITY_DN121_c0_g1_i9_orf1	lethal(2) giant larvae protein homolog 1 isoform X1 [Ostrinia furnacalis]	20	1120	122	6.47	23	27	20	High	1	0.986	1.024	0.674	0.702	0.712	0.669	0.668	0.695	0.685	0.696	0.677	0.707	0.686	0.703
TRINITY_DN4866_c0_g1_i2_orf1	actin-binding LIM protein 3 isoform X6 [Ostrinia furnacalis]	17	759	83.4	8.66	33	20	17	High	1	0.995	1.015	1.01	1.035	1.04	1.188	1.198	1.159	1.286	1.2	1.202	0.747	0.76	0.769
TRINITY_DN2173_c0_g1_i1_orf1	heat shock 70 kDa protein cognate 4 [Cephus cinctus]	17	651	70.9	5.58	27	36	8	High	1	0.975	0.958	0.653	0.749	0.65	0.593	0.658	0.568	0.576	0.649	0.709	0.715	0.683	0.757
TRINITY_DN2047_c0_g1_i1_orf1	carboxylesterase CXE17 [Ostrinia furnacalis]	22	707	78.2	4.89	33	33	20	High	1	1.04	1.004	0.666	0.636	0.72	0.628	0.633	0.646	0.665	0.628	0.696	0.666	0.666	0.648
TRINITY_DN2338_c0_g2_i1_orf1	prophenoloxidase PPO3 [Ostrinia furnacalis]	21	697	80.3	6.9	35	27	7	High	1	0.978	1.02	1.238	1.126	1.22	1.024	1.005	1.058	1.617	1.436	1.523	1.132	1.149	1.139
TRINITY_DN4596_c0_g1_i14_orf1	aldehyde dehydrogenase, dimeric NADP-preferring isoform X7 [Ostrinia furna	20	513	56.7	7.74	36	33	18	High	1	0.955	0.981	1.198	1.17	1.146	1.266	1.306	1.309	0.941	1.072	0.944	1.193	1.166	1.248
TRINITY_DN22747_c0_g1_i5_orf1	hypothetical protein HF086_001789 [Spodoptera exigua]	13	514	55.5	8.25	34	29	13	High	1	1.013	0.991	0.877	0.86	0.901	0.861	0.867	0.909	0.789	0.727	0.741	0.728	0.752	0.764
TRINITY_DN6698_c0_g2_i1_orf1	protein mesh isoform X2 [Ostrinia furnacalis]	20	1486	169.6	5.77	17	28	2	High	1	1.01	1.083	1.023	1.229	1.182	1.315	1.339	1.134	1.179	0.923	1.166	0.905	0.85	0.955
TRINITY_DN1982_c0_g1_i24_orf1	uncharacterized protein LOC114361215 isoform X5 [Ostrinia furnacalis]	16	1511	176	4.35	12	18	5	High	1	0.987	0.914	1.066	1.118	1.177	0.895	0.967	0.93	0.8	1.025	0.853	0.567	0.567	0.59
TRINITY_DN2688_c0_g2_i1_orf1	mitochondrial amidoxime reducing component 2 [Galleria mellonella]	15	334	37	8.12	46	25	14	High	1	1.001	0.978	1.105	1.112	1.116	1.286	1.257	1.262	1.111	1.017	1.146	0.712	0.709	0.73
TRINITY_DN15967_c0_g1_i4_orf1	adenylyl cyclase-associated protein 1 isoform X1 [Ostrinia furnacalis]	13	558	61.1	7.78	33	20	13	High	1	0.965	0.999	1.038	1.134	1.014	1.114	1.147	1.081	1.086	1.105	1.204	1.234	1.2	1.228
TRINITY_DN60946_c0_g2_i3_orf1	protein yellow-like [Ostrinia furnacalis]	13	445	49.2	5.77	36	45	13	High	1	0.981	0.994	2.828	2.827	2.701	3.186	3.163	3.039	2.638	2.608	2.816	3.715	3.747	3.672
TRINITY_DN2251_c0_g1_i4_orf1	serine hydroxymethyltransferase, cytosolic isoform X1 [Ostrinia furnacalis]	16	517	57.1	8.37	41	27	14	High	1	0.984	1.031	1.854	2.199	1.808	1.687	1.819	1.573	1.767	1.868	2.213	1.668	1.509	1.719
TRINITY_DN1177_c0_g1_i4_orf1	unnamed protein product [Chilo suppressalis]	18	723	80.6	7.93	29	35	18	High	1	0.994	0.991	1.163	1.209	1.161	1.194	1.171	1.169	1.033	1.025	1.088	0.881	0.868	0.915
TRINITY_DN3959_c1_g2_i1_orf1	PREDICTED: probable isocitrate dehydrogenase [NAD] subunit alpha, mitochor	18	355	38.7	7.93	55	31	16	High	1	1.015	0.995	0.291	0.294	0.345	0.331	0.309	0.318	0.324	0.348	0.308	0.316	0.325	0.331
TRINITY_DN33_c0_g1_i14_orf1	uncharacterized protein CG45076-like isoform X1 [Ostrinia furnacalis]	12	323	37.2	9.77	48	24	1	High	1	0.917	0.992	1.739	1.845	1.801	1.628	1.85	1.717	1.561	1.327	1.562	1.147	1.187	0.998

TRINITY_DN4051_c0_g1_i1_orf1	60S acidic ribosomal protein P2 [Ostrinia furnacalis]	8	111	11.4	4.65	67	33	1	High	1	1.026	0.973	0.858	0.897	0.887	0.822	0.836	0.849	0.916	1.047	1.168	0.903	0.934	0.876
TRINITY_DN12367_c0_g1_i4_orf1	aldose reductase-like isoform X2 [Ostrinia furnacalis]	13	293	32.8	6.32	48	40	3	High	1	0.946	1.006	0.761	0.753	0.755	0.958	0.985	0.963	0.99	0.895	1.014	0.882	0.886	0.85
TRINITY_DN29698_c0_g1_i3_orf1	15-hydroxyprostaglandin dehydrogenase [NAD(+)]-like [Ostrinia furnacalis]	10	277	30.3	5.55	48	20	10	High	1	0.964	0.987	2.18	2.043	2.088	1.822	1.706	1.88	1.926	1.772	1.703	1.084	1.07	1.012
TRINITY_DN4920_c0_g1_i5_orf1	titin homolog [Ostrinia furnacalis]	19	1092	125.1	4.56	26	22	19	High	1	0.966	0.955	0.797	0.858	0.853	0.72	0.753	0.71	0.693	0.615	0.692	0.363	0.385	0.367
TRINITY_DN4279_c0_g1_i4_orf1	glutathione S-transferase sigma 1 [Ostrinia furnacalis]	14	204	23.2	6.35	54	33	2	High	1	1.003	1.016	0.982	0.789	0.921	0.966	1.002	1.052	1.024	1.065	0.865	0.796	0.746	0.774
TRINITY_DN3433_c0_g1_i15_orf1	cytosolic purine 5'-nucleotidase isoform X3 [Ostrinia furnacalis]	18	580	66.4	6.47	36	30	1	High	1	1.056	1.051	1.918	1.942	1.82	2.188	2.155	2.142	1.616	1.721	1.7	1.391	1.464	1.466
TRINITY_DN10222_c0_g1_i2_orf1	glutathione S-transferase sigma 3 [Ostrinia furnacalis]	15	203	22.9	7.53	58	29	14	High	1	0.971	0.935	2.385	2.195	2.212	2.633	2.578	2.661	2.941	3.662	2.69	1.155	1.082	1.289
TRINITY_DN4944_c1_g1_i4_orf1	bifunctional glutamate/proline--tRNA ligase [Ostrinia furnacalis]	20	768	86.3	8.27	29	24	20	High	1	1.002	1.017	0.681	0.687	0.685	0.653	0.654	0.65	0.611	0.581	0.624	0.604	0.583	0.585
TRINITY_DN3433_c0_g1_i6_orf1	cytosolic purine 5'-nucleotidase isoform X2 [Ostrinia furnacalis]	18	607	70.2	7.64	34	30	1	High	1	0.938	0.942	1.617	1.408	1.48	1.285	1.232	1.133	1.305	1.176	1.057	1.039	0.997	1.041
TRINITY_DN1044_c0_g1_i2_orf1	V-type proton ATPase subunit H isoform X3 [Ostrinia furnacalis]	16	476	55.2	6.65	38	28	15	High	1	1.068	1.034	0.232	0.198	0.282	0.259	0.245	0.27	0.259	0.259	0.224	0.257	0.242	0.256
TRINITY_DN3073_c0_g1_i7_orf1	claspin-like [Ostrinia furnacalis]	17	755	81.8	4.27	31	18	17	High	1	0.992	0.993	1.022	1.047	1.041	1.041	1.082	1.033	1.036	0.889	1.002	2.003	1.937	1.936
TRINITY_DN33_c0_g1_i1_orf1	uncharacterized protein CG45076-like isoform X2 [Ostrinia furnacalis]	15	482	54.2	4.93	32	30	5	High	1	0.966	0.965	0.585	0.695	0.601	0.57	0.668	0.562	0.435	0.426	0.528	0.473	0.519	0.518
TRINITY_DN6698_c0_g2_i2_orf1	protein mesh isoform X1 [Ostrinia furnacalis]	19	1351	155	5.3	18	27	1	High	1	0.867	0.721	1.89	1.916	1.874	1.958	1.707	1.916	1.539	1.294	1.539	1.504	1.615	1.462
TRINITY_DN5510_c0_g1_i9_orf1	proteoglycan 4 [Pectinophora gossypiella]	11	573	63.1	7.96	29	16	11	High	1	1.038	1.019	1.147	1.228	1.129	1.247	1.312	1.228	1.084	0.96	1.187	0.701	0.729	0.722
TRINITY_DN32681_c0_g1_i3_orf1	long-chain-fatty-acid--CoA ligase ACSBG2 isoform X2 [Ostrinia furnacalis]	16	679	74.7	7.27	32	21	16	High	1	1.015	0.99	0.503	0.5	0.558	0.593	0.617	0.588	0.525	0.548	0.497	0.434	0.438	0.442
TRINITY_DN1775_c0_g1_i3_orf1	ATP-dependent RNA helicase dbp2-like isoform X1 [Ostrinia furnacalis]	17	829	92.9	9.36	24	21	13	High	1	1.017	1.008	0.277	0.296	0.337	0.336	0.318	0.309	0.345	0.309	0.336	0.575	0.574	0.556
TRINITY_DN801_c0_g1_i2_orf1	cathepsin L [Ostrinia furnacalis]	16	341	38.1	6.58	47	40	16	High	1	0.966	1.011	2.981	3.165	2.951	2.134	2.253	2.122	2.084	1.973	2.367	4.706	4.675	4.73
TRINITY_DN4861_c0_g1_i7_orf1	2-hydroxyacyl-CoA lyase 1 isoform X1 [Ostrinia furnacalis]	15	596	64.5	7.23	30	31	15	High	1	1	0.984	0.655	0.671	0.652	0.667	0.697	0.657	0.633	0.744	0.669	0.645	0.608	0.628
TRINITY_DN5218_c0_g1_i4_orf1	threonine--tRNA ligase, cytoplasmic isoform X1 [Trichoplusia ni]	16	722	83	6.98	27	24	16	High	1	0.994	1.01	0.817	0.799	0.834	0.773	0.797	0.802	0.775	0.808	0.765	0.672	0.668	0.663
TRINITY_DN5099_c0_g1_i3_orf1	trans-1,2-dihydrobenzene-1,2-diol dehydrogenase-like [Ostrinia furnacalis]	17	338	38.1	6.71	55	34	17	High	1	1.01	1.011	2.273	2.161	2.098	2.739	2.494	2.701	2.325	2.18	2.259	1.787	1.793	1.736
TRINITY_DN16187_c0_g1_i1_orf1	eIF-2-alpha kinase activator GCN1 [Colias croceus]	20	1466	158.6	7.23	15	24	20	High	1	1.019	0.998	0.845	0.858	0.862	0.79	0.784	0.798	0.676	0.636	0.686	0.76	0.709	0.715
TRINITY_DN13419_c0_g1_i5_orf1	atrial natriuretic peptide-converting enzyme-like [Ostrinia furnacalis]	15	950	103.6	4.49	22	27	15	High	1	0.996	1.001	1.025	1.054	0.971	1.174	1.206	1.14	0.873	0.707	0.876	1.334	1.366	1.304
TRINITY_DN6358_c0_g1_i5_orf1	histone H1B-like [Ostrinia furnacalis]	18	238	23.7	10.56	54	51	18	High	1	0.98	0.937	1.099	1.115	1.122	1.076	1.098	1.073	0.733	0.637	0.781	0.851	0.869	0.831
TRINITY_DN116_c1_g1_i8_orf1	uncharacterized protein LOC114350057 isoform X2 [Ostrinia furnacalis]	18	325	37.9	9.72	58	35	18	High	1	0.993	1	1.172	1.293	1.197	1.053	1.085	1.044	0.928	1.092	1.034	0.503	0.504	0.519
TRINITY_DN2577_c0_g1_i1_orf1	unnamed protein product [Diatraea saccharalis]	14	706	78.9	5.03	22	27	14	High	1	0.993	0.972	0.786	0.806	0.791	0.731	0.725	0.743	0.565	0.538	0.56	0.626	0.631	0.603
TRINITY_DN21533_c0_g1_i7_orf1	annexin B9 isoform X1 [Ostrinia furnacalis]	18	323	35.9	5.06	46	38	2	High	1	1.031	1.09	1.726	2.016	1.659	1.257	1.453	1.208	1.128	1.162	1.524	1.636	1.552	1.795
TRINITY_DN2515_c0_g1_i6_orf1	chitooligosaccharidolytic beta-N-acetylglucosaminidase isoform X1 [Ostrinia fu	16	614	70.4	5.64	35	22	16	High	1	1.101	1.09	1.59	1.604	1.587	1.663	1.65	1.644	1.564	1.427	1.531	3.334	3.346	3.148
TRINITY_DN8076_c0_g1_i6_orf1	hypothetical protein evm_001812 [Chilo suppressalis]	17	1162	127.3	6.8	16	22	4	High	1	0.993	1	0.9	0.975	0.983	0.846	0.952	0.796	0.794	0.77	0.834	0.791	0.859	0.812
TRINITY_DN2186_c0_g1_i13_orf1	paxillin isoform X2 [Ostrinia furnacalis]	14	595	64.9	7.65	38	18	5	High	1	1.028	1.022	1.077	1.085	1.064	1.081	0.944	0.897	0.99	0.953	0.967	0.854	0.861	0.793
TRINITY_DN1827_c0_g1_i4_orf1	phosphoglycerate mutase 1 [Ostrinia furnacalis]	13	255	28.8	6.1	58	25	13	High	1	1.002	1.002	1.429	1.461	1.409	1.428	1.42	1.391	1.585	1.428	1.764	1.17	1.194	1.229
TRINITY_DN9862_c0_g2_i1_orf1	40S ribosomal protein S4 [Manduca sexta]	12	263	29.6	10.3	44	37	12	High	1	0.939	0.935	0.67	0.722	0.637	0.581	0.579	0.548	0.469	0.502	0.564	0.521	0.487	0.541
TRINITY_DN1125_c0_g1_i4_orf1	hypothetical protein evm_001907 [Chilo suppressalis]	14	422	45.7	8.29	37	29	14	High	1	0.997	0.982	0.859	0.876	0.891	0.934	0.891	0.967	0.755	0.687	0.776	0.603	0.596	0.624
TRINITY_DN2146_c0_g2_i1_orf1	heat shock protein 68-like [Ostrinia furnacalis]	18	639	70.4	5.74	33	26	10	High	1	1.012	1.016	1.437	1.474	1.447	2.599	2.74	2.45	3.313	2.963	3.828	1.936	1.877	1.919
TRINITY_DN1533_c0_g2_i1_orf1	unnamed protein product [Chilo suppressalis]	13	442	48.2	5.97	34	27	13	High	1	0.974	1.003	1.239	1.233	1.261	1.286	1.321	1.234	1.267	1.141	1.305	1.791	1.789	1.762
TRINITY_DN2430_c0_g1_i1_orf1	glutathione S-transferase omega 1 [Ostrinia furnacalis]	10	254	28.8	6.7	50	22	10	High	1	0.995	0.956	0.666	0.627	0.707	0.75	0.69	0.714	0.626	0.605	0.588	0.751	0.781	0.717
TRINITY_DN86090_c0_g1_i1_orf1	ATP synthase subunit b, mitochondrial [Ostrinia furnacalis]	12	242	27.4	8.65	46	29	12	High	1	1.076	1.003	0.517	0.491	0.561	0.487	0.49	0.493	0.504	0.479	0.479	0.442	0.437	0.429
TRINITY_DN3257_c0_g1_i4_orf1	N-acetylneuraminate lyase-like [Ostrinia furnacalis]	10	302	32.5	8.92	44	28	8	High	1	1.022	1.033	1.532	1.539	1.474	1.725	1.766	1.674	1.638	1.602	1.644	1.376	1.401	1.404
TRINITY_DN15160_c0_g1_i1_orf1	tyrosine--tRNA ligase, cytoplasmic [Ostrinia furnacalis]	14	524	58.4	6.58	32	22	14	High	1	0.991	0.966	0.71	0.728	0.75	0.582	0.599	0.612	0.875	0.815	0.861	0.559	0.564	0.581
TRINITY_DN335_c1_g1_i5_orf1	PREDICTED: perilipin-4 isoform X14 [Papilio polytes]	13	147	15.1	7.24	74	21	13	High	1	1.035	1.002	3.089	3.272	3.137	2.853	2.945	2.703	2.072	1.892	2.331	1.234	1.259	1.274
TRINITY_DN42646_c0_g2_i1_orf1	40S ribosomal protein S3 [Helicoverpa armigera]	13	243	26.8	9.6	56	35	13	High	1	0.983	1.047	0.685	0.641	0.644	0.61	0.575	0.613	0.511	0.485	0.484	0.491	0.487	0.494
TRINITY_DN32538_c0_g1_i2_orf1	4-hydroxybutyrate coenzyme A transferase-like [Pectinophora gossypiella]	14	478	52.1	8.21	37	25	14	High	1	0.988	1.008	0.842	0.828	0.839	0.872	0.869	0.853	0.843	0.819	0.862	0.796	0.83	0.81
TRINITY_DN6916_c0_g1_i4_orf1	isovaleryl-CoA dehydrogenase, mitochondrial [Ostrinia furnacalis]	11	415	45.2	7.94	35	17	11	High	1	0.97	0.986	0.818	0.831	0.811	0.762	0.76	0.745	0.784	0.74	0.762	0.608	0.616	0.614
TRINITY_DN78492_c0_g1_i1_orf1	uncharacterized protein LOC114354775 [Ostrinia furnacalis]	14	345	37.8	8.84	52	22	13	High	1	0.976	0.999	0.818	0.812	0.833	0.844	0.864	0.894	0.641	0.659	0.714	0.688	0.623	0.615
TRINITY_DN53684_c0_g1_i1_orf1	eukaryotic translation initiation factor 3 subunit M-like [Ostrinia furnacalis]	17	386	43.9	6.21	42	22	17	High	1	0.961	0.956	0.633	0.647	0.634	0.598	0.617	0.614	0.583	0.605	0.608	0.692	0.663	0.7
TRINITY_DN85412_c0_g1_i1_orf1	unnamed protein product [Diatraea saccharalis]	11	105	12	6.32	87	37	11	High	1	0.993	1.004	1.543	1.578	1.514	1.816	1.742	1.693	2.86	2.679	3.117	4.089	4.099	3.967
TRINITY_DN18230_c1_g1_i1_orf1	glycine dehydrogenase (decarboxylating), mitochondrial isoform X1 [Ostrinia f	11	501	55.3	7.14	32	17	10	High	1	1.042	0.967	1.577	1.635	1.526	1.431	1.465	1.477	1.428	1.37	1.511	1.378	1.52	1.412
TRINITY_DN23429_c0_g2_i1_orf1	muscle-specific protein 20 [Zerene cesonia]	13	184	20.3	8.24	75	32	12	High	1	1	0.986	1.167	1.126	1.148	1.02	1.049	0.979	0.983	0.903	1.014	0.457	0.434	0.449
TRINITY_DN618_c0_g1_i3_orf1	triokinase/FMN cyclase-like isoform X1 [Ostrinia furnacalis]	13	592	62.6	6.76	25	22	12	High	1	1.01	1.037	2.131	2.089	1.972	2.247	2.09	2.216	1.993	1.912	2.068	1.623	1.633	1.544
TRINITY_DN12175_c0_g1_i5_orf1	dolichyl-diphosphooligosaccharide--protein glycosyltransferase subunit 2 isof	14	623	67.6	8.82	25	27	14	High	1	0.984	0.998	1	1.019	1.011	0.98	1.008	0.995	1.018	0.936	1.01	1.024	0.995	1
TRINITY_DN2956_c0_g1_i6_orf1	fructose-bisphosphate aldolase-like isoform X1 [Ostrinia furnacalis]	12	364	39.5	7.44	39	20	9	High	1	1.017	1.012	1.392	1.364	1.408	1.499	1.486	1.475	1.297	1.265	1.237	0.849	0.799	0.844
TRINITY_DN49038_c0_g4_i1_orf1	6-phosphogluconate dehydrogenase, decarboxylating [Ostrinia furnacalis]	15	483	53	7.85	33	30	15	High	1	0.96	0.952	1.497	1.492	1.422	1.435	1.453	1.416	1.415	1.455	1.484	1.167	1.144	1.219
TRINITY_DN23801_c0_g1_i2_orf1	signal recognition particle subunit SRP72 [Pectinophora gossypiella]	12	646	71.7	9.23	21	14	12	High	1	0.949	0.999	0.828	0.84	0.805	0.755	0.765	0.787	0.656	0.67	0.669	0.743	0.76	0.76
TRINITY_DN83005_c0_g1_i1_orf1	ATP synthase subunit O, mitochondrial [Danaus plexippus plexippus]	13	209	22.7	9.66	55	35	13	High	1	1.009	0.988	0.45	0.423	0.483	0.458	0.448	0.46	0.475	0.432	0.441	0.416	0.412	0.403
TRINITY_DN2764_c0_g1_i11_orf1	protein transport protein Sec31A [Ostrinia furnacalis]	15	1241	134.9	6.6	19	17	15	High	1	1.007	0.981	0.901	0.923	0.921	0.926	0.924	0.949	0.842	0.856	0.89	0.88	0.894	0.864
TRINITY_DN18323_c0_g1_i5_orf1	GILT-like protein 1 isoform X1 [Ostrinia furnacalis]	15	223	25	8.53	62	31	15	High	1	0.996	0.975	2.765	2.863	2.716	2.664	2.657	2.536	2.82	2.497	3.157	2.776	2.895	2.798
TRINITY_DN3758_c0_g1_i2_orf1	S-formylglutathione hydrolase isoform X1 [Spodoptera litura]	14	299	33.3	5.97	60	33	14	High	1	1.008	0.982	1.466	1.452	1.414	1.425	1.431	1.408	1.567	1.507	1.648	1.269	1.33	1.309
TRINITY_DN140_c0_g1_i1_orf1	calcyphosin-like protein [Ostrinia furnacalis]	14	232	27.1	6.1	55	38	7	High	1	0.991	1.002	2.143	2.17	2.153	2.485	2.599	2.371	2.907	2.888	3.078	1.903	1.838	1.883
TRINITY_DN23194_c0_g1_i4_orf1	nucleobindin-2 isoform X2 [Ostrinia furnacalis]	13	574	66.2	5.52	28	19	13	High	1	1.037	1.004	1.079	1.145	1.113	1.07	1.064	1.055	0.931	0.846	1.05	1.257	1.28	1.221
TRINITY_DN1982_c0_g1_i17_orf1	unnamed protein product, partial [Iphiclides podalirius]	17	1075	124.3	4.41	17	20	6	High	1	1.007	0.998	0.918	0.94	1.031	0.881	0.951	1.013	0.839	0.772	0.792	0.441	0.395	0.427
TRINITY_DN1572_c0_g1_i6_orf1	eukaryotic translation initiation factor 3 subunit D [Ostrinia furnacalis]	14	547	62	7.42	37	17	14	High	1	0.987	1.001	0.679	0.651	0.712	0.642	0.613	0.65	0.64	0.599	0.622	0.66	0.667	0.643
TRINITY_DN631_c0_g1_i6_orf1	cytosolic 10-formyltetrahydrofolate dehydrogenase isoform X1 [Ostrinia furna(	17	927	101.7	6.1	25	18	17	High	1	1.009	1.001	0.575	0.593	0.589	0.572	0.575	0.548	0.449	0.467	0.498	0.505	0.495	0.519
TRINITY_DN810_c0_g1_i4_orf1	dicer 2 [Ostrinia nubilalis]	18	1511	172.9	7.17	14	26	18	High	1	0.978	1.025	1.215	1.164	1.161	1.586	1.571	1.557	1.235	1.226	1.231	1.416	1.407	1.431
TRINITY_DN63719_c0_g1_i5_orf1	eukaryotic peptide chain release factor GTP-binding subunit ERF3A isoform X2	14	605	66	5.22	30	21	14	High	1	0.979	0.985	0.739	0.771	0.763	0.746	0.798	0.809	0.683	0.697	0.714	0.8	0.735	0.747
TRINITY_DN57900_c0_g1_i2_orf1	hypothetical protein SFRURICE_000634 [Spodoptera frugiperda]	12	265	30.1	7.44	57	31	11	High	1	1.014	1	1.452	1.475	1.506	2.233	2.148	2.102	1.418	1.275	1.544	0.794	0.81	0.794
TRINITY_DN33967_c0_g1_i1_orf1	PREDICTED: elongation factor 1-alpha [Microplitis demolitor]	16	461	50.2	9.03	37	58	4	High	1	1.056	1.047	0.865	0.873	0.899	0.778	0.771	0.791	0.707	0.734	0.69	0.706	0.723	0.785
TRINITY_DN12106_c0_g1_i4_orf1	programmed cell death 6-interacting protein [Ostrinia furnacalis]	17	603	66.1	5.35	31	24	17	High	1	1.013	0.994	1.232	1.218	1.233	1.245	1.232	1.211	1.118	1.03	1.107	1.289	1.308	1.272
TRINITY_DN9492_c0_g1_i7_orf1	aldo-keto reductase AKR2E4-like [Ostrinia furnacalis]	12	336	38.3	7.62	30	36	7	High	1	0.926	0.986	2.13	2.156	1.98	2.479	2.526	2.446	2.281	2.29	2.41	2.007	1.906	2.058
TRINITY_DN1329_c0_g1_i5_orf1	neurogenic locus notch homolog protein 3 [Ostrinia furnacalis]	13	507	54.1	6.3	43	22	13	High	1	0.932	1.024	1.692	1.809	1.741	2.04	1.907	1.903	1.506	1.316	1.668	0.719	0.638	0.624
TRINITY_DN232_c0_g1_i9_orf1	kynurenine--oxoglutarate transaminase 3 [Ostrinia furnacalis]	14	460	51.9	8.59	39	23	13	High	1	1.015	1.009	0.997	0.962	1.037	1.012	0.972	0.982	0.986	0.988	0.985	1.04	1.054	1.035
TRINITY_DN6669_c0_g1_i3_orf1	glutamine synthetase 2 cytoplasmic [Ostrinia furnacalis]	13	382	42.8	6.54	38	24	13	High	1	1.001	0.992	1.15	1.157	1.128	1.268	1.246	1.198	1.096	0.948	1.186	1.223	1.274	1.217
TRINITY_DN26961_c0_g1_i1_orf1	uncharacterized protein LOC120424957 [Culex pipiens pallens]	11	106	12.3	5.1	64	21	5	High	1	0.932	0.964	1.616	2.383	2.025	1.515	1.711	1.14	1.297	1.358	1.868	0.718	0.642	0.623
TRINITY_DN2999_c1_g2_i1_orf1	uncharacterized protein C05D11.1-like [Ostrinia furnacalis]	13	806	90.7	5.21	22	16	13	High	1	1.042	1.029	0.944	0.886	0.938	0.83	0.819	0.839	0.81	0.77	0.76	0.91	0.918	0.854
TRINITY_DN970_c0_g1_i4_orf1	spermine oxidase-like isoform X2 [Ostrinia furnacalis]	16	497	55.5	5.62	32	26	16	High	1	1.038	1.032	1.806	1.836	1.81	2.205	2.15	2.206	2.133	1.884	2.087	1.432	1.488	1.509
TRINITY_DN1423_c0_g1_i4_orf1	hypothetical protein evm_003306 [Chilo suppressalis]	11	231	26.4	6.16	44	35	9	High	1	0.987	0.994	1.666	1.665	1.593	1.744	1.778	1.655	2.015	1.916	2.236	3.095	3.037	3.001
TRINITY_DN55147_c0_g1_i1_orfp1	TRINITY_DN55147_c0_g1_i1_m.59251 TRINITY_DN55147_c0_g1::TRINITY_DN55	7	330	33.7	9.41	50	29	7	High	1	1.026	1.089	2.817	2.935	2.674	2.621	2.485	2.518	2.477	2.296	2.734	0.86	0.847	0.882
TRINITY_DN9715_c0_g1_i1_orf1	V-type proton ATPase subunit E [Manduca sexta]	12	226	26	8.76	44	22	12	High	1	0.98	0.958	0.268	0.257	0.303	0.296	0.283	0.307	0.3	0.286	0.279	0.294	0.288	0.286
TRINITY_DN7228_c0_g1_i6_orf1	neutral alpha-glucosidase AB [Ostrinia furnacalis]	16	871	99	5.99	20	20	16	High	1	0.987	0.988	0.651	0.622	0.652	0.703	0.718	0.714	0.667	0.668	0.658	0.821	0.784	0.802
TRINITY_DN628_c0_g1_i1_orf1	prostamide/prostaglandin F synthase-like [Ostrinia furnacalis]	9	240	26.8	8.73	46	30	9	High	1	0.993	1.002	3.058	2.984	2.836	2.926	2.988	2.933	3.041	3.253	3.023	2.348	2.318	2.563
TRINITY_DN13118_c0_g1_i6_orf1	AP-1 complex subunit beta-1 [Helicoverpa armigera]	15	910	99.8	5.31	20	20	15	High	1	1.011	1.037	0.959	0.883	0.934	0.831	0.853	0.863	0.762	0.792	0.745	1.065	1.06	1.059
TRINITY_DN19251_c0_g1_i8_orf1	succinate--CoA ligase [GDP-forming] subunit beta, mitochondrial [Ostrinia fur	16	421	45.8	6.81	44	22	16	High	1	0.997	1.04	1.312	1.362	1.279	1.115	1.109	1.089	1.108	1.039	1.121	0.863	0.878	0.868
TRINITY_DN1084_c0_g2_i2_orf1	ATP-citrate synthase [Ostrinia furnacalis]	14	474	52.5	7.33	36	28	10	High	1	0.967	1.016	0.963	1.012	0.958	1.094	1.073	1.11	1.137	1.065	1.15	0.759	0.767	0.808
TRINITY_DN16816_c0_g1_i1_orf1	ATP-binding cassette sub-family F member 1 [Ostrinia furnacalis]	16	917	102.4	6.71	19	18	16	High	1	1.015	1.005	0.703	0.714	0.745	0.706	0.668	0.682	0.604	0.564	0.631	0.654	0.641	0.602
TRINITY_DN8406_c0_g1_i2_orf1	titin [Ostrinia furnacalis]	17	1256	141.9	9.5	21	22	15	High	1	0.984	1.011	1.068	1.14	1.066	0.976	0.957	0.924	0.905	0.785	0.996	0.804	0.804	0.797
TRINITY_DN8258_c0_g1_i3_orf1	papilin isoform X7 [Ostrinia furnacalis]	11	749	82	4.87	21	18	3	High	1	1.006	0.948	1.662	1.589	1.561	1.425	1.632	1.595	1.397	1.334	1.352	1.196	1.137	1.093
TRINITY_DN53807_c0_g2_i1_orf1	aminomethyltransferase, mitochondrial [Ostrinia furnacalis]	12	408	44.2	8.46	34	15	12	High	1	0.967	0.986	1.556	1.518	1.471	1.646	1.637	1.632	1.759	1.715	1.812	1.553	1.521	1.583
TRINITY_DN565_c0_g1_i1_orf1	uncharacterized protein LOC114362321 [Ostrinia furnacalis]	10	247	26.4	7.36	55	16	10	High	1	0.982	0.992	1.159	1.208	1.189	1.121	1.137	1.065	1.082	1.223	1.137	0.972	0.992	1.033
TRINITY_DN97138_c0_g1_i2_orf1	tubulin beta chain-like isoform X2 [Ostrinia furnacalis]	12	284	31.2	5.22	52	26	4	High	1	0.979	0.979	1.201	1.236	1.285	1.216	1.342	1.35	1.197	1.462	1.181	2.491	2.513	2.455
TRINITY_DN1423_c0_g1_i8_orf1	ferritin subunit-like [Ostrinia furnacalis]	7	211	23.7	6.64	34	28	6	High	1	0.99	0.986	1.78	1.691	1.81	1.867	1.837	1.768	2.63	2.379	2.39	3.931	4.227	3.827
TRINITY_DN55026_c0_g1_i1_orf1	probable glutamine--tRNA ligase [Ostrinia furnacalis]	19	780	87.4	7.49	27	19	19	High	1	0.976	1.006	0.847	0.853	0.846	0.818	0.83	0.828	0.836	0.865	0.85	0.884	0.857	0.914
TRINITY_DN1196_c0_g1_i5_orf1	glucosamine-6-phosphate isomerase isoform X1 [Ostrinia furnacalis]	8	278	31.2	6.67	45	20	1	High	1	1.037	1.031	1.196	1.156	1.093	1.021	1.03	1.11	1.031	0.794	0.876	2.226	2.108	2.041
TRINITY_DN19942_c0_g1_i2_orf1	ribosomal protein l1p/L10e family domain-containing protein [Phthorimaea op	9	217	24.6	9.72	38	29	9	High	1	1.018	1.001	0.797	0.851	0.827	0.811	0.79	0.764	0.742	0.777	0.846	0.765	0.768	0.745
TRINITY_DN3471_c0_g1_i1_orf1	EH domain-containing protein 3 [Ostrinia furnacalis]	17	532	61	6.62	41	22	17	High	1	0.985	0.989	0.797	0.829	0.815	0.699	0.757	0.695	0.738	0.703	0.782	0.764	0.771	0.786
TRINITY_DN7770_c0_g1_i4_orf1	presequence protease, mitochondrial [Ostrinia furnacalis]	18	1015	116.4	6.8	19	21	18	High	1	1.012	1.038	0.596	0.591	0.614	0.658	0.662	0.714	0.699	0.664	0.646	0.703	0.695	0.679
TRINITY_DN19727_c0_g1_i7_orf1	dihydrolipoyllysine-residue succinyltransferase component of 2-oxoglutarate (	11	464	49.7	9.32	32	22	11	High	1	1.03	0.982	0.552	0.53	0.581	0.632	0.648	0.62	0.649	0.623	0.649	0.558	0.545	0.549

TRINITY_DN24024_c0_g1_i1_orf1	dolichyl-diphosphooligosaccharide--protein glycosyltransferase subunit 1 [Os	17	475	53.8	8.81	40	25	17	High	1	1.04	1.032	0.953	0.942	0.941	0.899	0.794	0.855	0.842	0.772	0.903	0.988	0.957	0.954
TRINITY_DN1750_c1_g1_i5_orf1	lipid droplet localized protein-like [Ostrinia furnacalis]	15	428	47.8	8.91	35	22	15	High	1	1.004	1.032	2.385	2.517	2.27	2.239	2.32	2.163	2.309	2.695	2.541	1.69	1.718	1.783
TRINITY_DN1607_c0_g1_i16_orf1	LOW QUALITY PROTEIN: asparagine--tRNA ligase, cytoplasmic [Ostrinia furna	13	549	62.3	6.21	25	20	13	High	1	1.026	1.03	1.625	1.696	1.577	1.478	1.386	1.438	1.514	1.419	1.534	0.871	0.855	0.868
TRINITY_DN1829_c0_g1_i1_orf1	ubiquitin carboxyl-terminal hydrolase 14 isoform X1 [Ostrinia furnacalis]	13	499	54.6	5.21	32	22	13	High	1	0.99	1.005	1.099	1.086	1.069	1.061	1.056	1.095	1.084	1.08	1.117	1.152	1.149	1.168
TRINITY_DN7579_c1_g3_i1_orf1	peroxiredoxin-5, mitochondrial [Ostrinia furnacalis]	8	189	20.2	8.73	50	22	8	High	1	1.007	0.98	1.661	1.654	1.659	1.597	1.551	1.567	1.706	1.651	1.771	1.81	1.829	1.851
TRINITY_DN45530_c0_g1_i1_orf1	aldose 1-epimerase isoform X1 [Ostrinia furnacalis]	14	378	41.2	6.68	53	19	14	High	1	0.974	0.95	1.523	1.442	1.46	1.64	1.646	1.631	1.887	1.756	1.808	1.544	1.548	1.571
TRINITY_DN4822_c0_g1_i6_orf1	homogentisate 1,2-dioxygenase [Ostrinia furnacalis]	14	438	48.9	6.57	43	16	1	High	1	0.969	0.944	0.938	0.853	0.862	1.371	1.307	1.31	2.32	2.204	2.126	1.285	1.469	1.591
TRINITY_DN49409_c0_g1_i2_orf1	proliferation-associated protein 2G4 [Ostrinia furnacalis]	15	379	42	7.02	39	28	15	High	1	1.022	0.987	0.598	0.597	0.62	0.641	0.601	0.619	0.548	0.539	0.541	0.606	0.621	0.596
TRINITY_DN48851_c0_g1_i2_orf1	translationally-controlled tumor protein homolog [Ostrinia furnacalis]	9	172	19.9	4.86	56	28	9	High	1	1.047	1.006	0.974	0.934	0.978	0.817	0.794	0.814	0.763	0.762	0.74	0.672	0.657	0.693
TRINITY_DN4572_c0_g1_i2_orf1	unnamed protein product [Arctia plantaginis]	13	400	42.5	6.34	36	18	12	High	1	0.934	0.994	0.941	0.986	0.973	1.04	1	1.052	0.972	0.96	1.02	1.275	1.308	1.284
TRINITY_DN825_c8_g1_i5_orf1	ATP-binding cassette sub-family F member 2 [Ostrinia furnacalis]	15	621	70.8	7.17	26	17	15	High	1	1.019	1.044	0.538	0.537	0.586	0.518	0.501	0.5	0.493	0.474	0.461	0.492	0.5	0.486
TRINITY_DN21533_c0_g1_i6_orf1	annexin B9 isoform X2 [Ostrinia furnacalis]	17	323	35.9	5.11	46	37	1	High	1	0.894	0.791	1.482	1.515	1.494	1.182	1.345	1.139	1.272	1.531	1.252	1.416	1.315	1.712
TRINITY_DN6313_c0_g1_i4_orf1	pyruvate dehydrogenase E1 component subunit beta, mitochondrial isoform X	8	366	39.4	6.25	36	15	8	High	1	0.983	0.964	0.722	0.708	0.74	0.733	0.715	0.708	0.743	0.772	0.746	0.67	0.662	0.672
TRINITY_DN21533_c0_g1_i4_orf1	hypothetical protein evm_010931 [Chilo suppressalis]	17	357	39.7	5.54	40	37	1	High	1	1.053	1.034	2.059	1.755	1.864	1.577	1.643	1.64	1.777	1.476	1.559	1.79	1.741	1.608
TRINITY_DN14215_c0_g1_i7_orf1	inner nuclear membrane protein Man1-like [Ostrinia furnacalis]	14	773	86.1	8.69	24	16	14	High	1	0.977	1.004	1.078	0.955	1.039	1.074	0.981	1.074	1.098	0.989	0.925	0.939	0.946	0.922
TRINITY_DN1334_c0_g1_i2_orf1	phosphoenolpyruvate carboxykinase [GTP]-like [Ostrinia furnacalis]	14	638	70.4	7.84	28	20	14	High	1	0.988	0.962	0.768	0.781	0.815	0.704	0.719	0.721	0.783	0.797	0.807	0.809	0.777	0.798
TRINITY_DN12367_c0_g1_i8_orf1	aldose reductase-like isoform X2 [Ostrinia furnacalis]	9	226	25.1	8.21	46	33	1	High	1	0.941	0.958	1.087	1.202	1.121	1.868	1.942	1.923	1.783	2.135	1.922	1.266	1.278	1.329
TRINITY_DN1563_c0_g1_i4_orf1	pupal cuticle protein 36-like [Ostrinia furnacalis]	5	320	30.1	5.34	41	8	5	High	1	0.933	0.975	1.065	1.043	0.911	0.937	0.982	0.988	1.06	1.053	1.164	2.624	2.588	2.429
TRINITY_DN4822_c0_g1_i9_orf1	homogentisate 1,2-dioxygenase [Ostrinia furnacalis]	14	438	48.9	6.57	43	17	1	High	1	0.889	0.962	1.114	1.119	1.095	0.897	0.827	0.979	0.538	0.549	0.521	0.697	0.687	0.594
TRINITY_DN20_c0_g1_i1_orf1	plasma membrane calcium-transporting ATPase 2 [Ostrinia furnacalis]	14	644	71.6	8.53	24	23	2	High	1	1.116	1.173	0.94	0.98	0.96	0.939	0.963	0.833	1.081	1.327	0.972	0.777	0.782	0.963
TRINITY_DN981_c0_g1_i1_orf1	proteasome subunit alpha type-6-like [Ostrinia furnacalis]	12	246	27.2	6.99	52	22	12	High	1	0.941	1.091	1.096	1.13	1.045	1.019	1.016	1.095	1.096	1.002	0.99	1.163	1.116	1.094
TRINITY_DN4540_c0_g1_i9_orf1	dihydropyrimidinase isoform X2 [Manduca sexta]	13	584	63.8	6.95	30	18	13	High	1	1.024	1.003	1.082	1.104	1.089	0.964	0.948	0.97	0.887	0.821	0.905	1.198	1.194	1.132
TRINITY_DN94625_c0_g1_i1_orf1	uncharacterized protein LOC114354112 [Ostrinia furnacalis]	13	464	50.9	8.1	36	18	12	High	1	1.027	1.027	0.48	0.46	0.515	0.493	0.471	0.486	0.472	0.447	0.491	0.465	0.442	0.436
TRINITY_DN48410_c0_g1_i1_orf1	alpha-amylase 1-like [Ostrinia furnacalis]	11	500	56	8.06	29	24	11	High	1	0.979	0.957	0.131	0.145	0.164	0.169	0.196	0.185	0.179	0.192	0.182	0.172	0.169	0.176
TRINITY_DN34455_c0_g1_i1_orf1	sodium/calcium exchanger regulatory protein 1 [Manduca sexta]	11	132	14.9	5.71	81	58	11	High	1	1.011	0.987	1.169	1.163	1.184	1.042	1.052	1.025	1.049	1.006	1.081	0.942	0.97	0.908
TRINITY_DN1669_c0_g1_i6_orf1	14-3-3 protein zeta isoform X1 [Helicoverpa armigera]	12	247	28.1	4.94	50	40	10	High	1	0.997	0.976	0.991	0.956	0.969	1.011	0.991	0.993	0.921	1.11	0.919	0.97	0.968	0.959
TRINITY_DN1444_c1_g1_i5_orf1	spondin-1 isoform X1 [Ostrinia furnacalis]	18	773	85.3	5.72	28	21	18	High	1	1.05	1.024	0.6	0.615	0.652	0.607	0.614	0.591	0.505	0.536	0.536	1.002	1.034	0.97
TRINITY_DN4813_c0_g1_i5_orf1	piwi-like protein Siwi [Ostrinia furnacalis]	18	903	101.4	9.44	20	22	18	High	1	0.992	0.987	1.108	1.096	1.082	1.314	1.281	1.283	0.98	0.954	0.939	1.224	1.194	1.199
TRINITY_DN348_c0_g2_i3_orf1	pancreatic triacylglycerol lipase-like [Ostrinia furnacalis]	11	332	36.1	8.75	45	18	4	High	1	0.998	0.977	0.05	0.052	0.085	0.083	0.091	0.093	0.091	0.084	0.09	0.101	0.094	0.092
TRINITY_DN931_c0_g1_i4_orf1	plastin-2 [Galleria mellonella]	12	717	79.8	5.1	18	17	12	High	1	1.01	1.022	0.911	0.878	0.877	0.952	0.942	0.953	1.04	0.932	0.989	1.076	1.084	1.068
TRINITY_DN703_c0_g1_i2_orf1	acidic juvenile hormone-suppressible protein 1-like [Ostrinia furnacalis]	6	89	10.1	7.08	97	79	6	High	1	0.982	0.974	4.327	4.274	4.287	5.19	5.153	5.078	8.693	7.964	8.697	5.555	5.663	5.766
TRINITY_DN17896_c0_g1_i1_orf1	probable aminopeptidase NPEPL1 isoform X1 [Ostrinia furnacalis]	12	563	59.8	7.75	28	21	12	High	1	0.994	1.01	1.181	1.162	1.171	1.049	1.114	1.032	0.916	0.872	0.977	0.911	0.887	0.894
TRINITY_DN14670_c0_g1_i1_orf1	heat shock protein beta-1 isoform X1 [Helicoverpa armigera]	13	187	21.4	6.15	72	30	13	High	1	1.03	1.02	2.66	2.886	2.54	2.059	2.078	1.996	1.826	2.095	2.123	1.281	1.294	1.293
TRINITY_DN2186_c0_g1_i17_orf1	paxillin isoform X6 [Leguminivora glycinivorella]	11	234	26.4	6.71	65	16	2	High	1	1.06	0.99	0.903	0.959	0.941	0.753	0.781	0.767	0.609	0.614	0.694	0.196	0.215	0.227
TRINITY_DN67716_c0_g1_i1_orf1	unnamed protein product [Spodoptera exigua]	11	340	37.3	7.09	38	21	11	High	1	0.989	0.997	0.711	0.811	0.716	0.731	0.755	0.727	0.75	0.713	0.846	0.726	0.728	0.719
TRINITY_DN391_c0_g1_i4_orf1	phenoloxidase-activating enzyme-like [Ostrinia furnacalis]	11	424	45.8	7.2	39	17	11	High	1	1.031	1.046	1.706	1.731	1.686	1.931	1.936	1.929	1.998	1.888	2.14	3.834	3.988	3.659
TRINITY_DN181_c0_g1_i3_orf1	hypothetical protein evm_003589 [Chilo suppressalis]	14	1222	140.7	6.28	13	21	14	High	1	1.005	1.043	0.881	0.878	0.88	0.799	0.818	0.811	0.737	0.738	0.763	0.922	0.9	0.92
TRINITY_DN5064_c0_g1_i4_orf1	sortilin-related receptor-like [Ostrinia furnacalis]	19	2179	243.8	5.73	10	21	19	High	1	0.959	0.986	1.643	1.578	1.589	1.528	1.538	1.511	1.268	1.439	1.26	2.066	1.962	2.022
TRINITY_DN1447_c0_g1_i5_orf1	PREDICTED: coatomer subunit beta' [Amyelois transitella]	16	1069	119	5.17	17	18	16	High	1	0.998	1.049	0.76	0.721	0.738	0.725	0.692	0.73	0.634	0.603	0.6	0.826	0.793	0.831
TRINITY_DN34479_c0_g1_i2_orf1	PREDICTED: 26S protease regulatory subunit 4 [Amyelois transitella]	10	440	49.2	6.58	32	15	9	High	1	1.007	0.996	0.94	0.964	0.951	0.773	0.751	0.763	0.702	0.672	0.725	0.925	0.937	0.912
TRINITY_DN5439_c0_g1_i2_orf1	uncharacterized protein LOC114353087 [Ostrinia furnacalis]	17	531	61.8	8.15	34	26	16	High	1	1.004	1.019	3.525	3.384	3.356	4.599	4.565	4.571	2.901	3.012	2.929	2.336	2.252	2.405
TRINITY_DN609_c0_g1_i1_orf1	zonadhesin-like isoform X1 [Ostrinia furnacalis]	11	1011	109	6.67	18	13	9	High	1	1.135	1.096	5.377	5.681	5.819	2.803	2.829	2.574	3.268	2.821	3.796	1.117	1.048	1.088
TRINITY_DN1732_c0_g1_i15_orf1	CAD protein isoform X2 [Ostrinia furnacalis]	13	653	72.9	6.06	23	20	1	High	1	0.941	0.93	0.977	1.306	1.137	1.607	1.622	1.59	1.781	2.424	2.128	1.475	1.521	1.767
TRINITY_DN32487_c0_g1_i1_orf1	heat shock protein 75 kDa, mitochondrial [Ostrinia furnacalis]	14	679	77	8.15	27	19	13	High	1	1.06	1.033	0.591	0.579	0.59	0.584	0.589	0.603	0.584	0.608	0.566	0.604	0.59	0.56
TRINITY_DN2097_c1_g2_i2_orf1	serine protease inhibitor 3 [Ostrinia furnacalis]	12	450	50.5	6.15	30	23	12	High	1	1.013	1.026	1.568	1.506	1.538	1.847	1.843	1.918	2.018	2.017	1.861	2.579	2.554	2.631
TRINITY_DN2584_c0_g1_i7_orf1	acylamino-acid-releasing enzyme-like isoform X1 [Ostrinia furnacalis]	13	709	78.1	6.92	21	20	13	High	1	1.011	1.01	0.986	1.033	1.008	1.029	1.094	1.077	1.167	1.079	1.138	1.331	1.303	1.335
TRINITY_DN5976_c0_g1_i1_orf1	ribosomal protein L32 [Bombyx mori]	11	134	16.1	11.56	53	38	11	High	1	1.039	1.059	0.743	0.689	0.739	0.678	0.621	0.662	0.646	0.606	0.633	0.636	0.597	0.605
TRINITY_DN4561_c0_g1_i3_orf1	host cell factor 1 [Ostrinia furnacalis]	15	839	87.3	9.89	21	17	15	High	1	0.975	1.003	0.903	0.919	0.905	0.932	0.928	0.998	0.911	0.869	0.912	1.186	1.196	1.19
TRINITY_DN5029_c0_g1_i1_orf1	ribose-phosphate pyrophosphokinase 2-like [Ostrinia furnacalis]	7	343	37.8	6.87	25	25	7	High	1	0.942	0.976	1.24	1.273	1.239	1.516	1.562	1.458	1.57	1.678	1.719	1.664	1.533	1.681
TRINITY_DN11448_c0_g1_i11_orf1	hypothetical protein B5X24_HaOG201808 [Helicoverpa armigera]	14	262	29.8	9.86	54	32	2	High	1	1.101	1.077	1.629	1.629	1.616	1.166	1.073	1.068	0.885	0.82	1.026	0.672	0.656	0.671
TRINITY_DN21722_c0_g1_i3_orf1	V-type proton ATPase 116 kDa subunit a isoform X1 [Ostrinia furnacalis]	12	837	95.5	6.35	20	14	11	High	1	0.992	0.959	0.223	0.235	0.254	0.27	0.259	0.254	0.245	0.255	0.245	0.253	0.226	0.229
TRINITY_DN80328_c0_g1_i5_orf1	arylphorin subunit alpha-like [Ostrinia furnacalis]	10	104	12.5	6.55	74	123	7	High	1	0.955	0.985	2.23	2.069	2.168	2.621	2.595	2.691	3.643	3.721	3.324	3.017	3.038	3.08
TRINITY_DN12301_c0_g1_i1_orf1	ribose-phosphate pyrophosphokinase 2 [Ostrinia furnacalis]	10	342	37.3	6.79	35	18	6	High	1	1.039	0.974	1.278	1.184	1.255	1.462	1.306	1.332	1.664	1.719	1.565	1.56	1.445	1.615
TRINITY_DN4141_c0_g1_i9_orf1	la-related protein 1 isoform X2 [Ostrinia furnacalis]	15	939	105.5	6.52	19	18	15	High	1	1.011	1.015	0.553	0.664	0.583	0.601	0.605	0.617	0.547	0.556	0.54	0.555	0.563	0.558
TRINITY_DN6415_c0_g1_i1_orf1	D-arabinitol dehydrogenase 1-like [Ostrinia furnacalis]	13	341	37.8	7.91	42	26	6	High	1	0.981	0.96	1.152	1.127	1.266	1.742	1.938	1.719	1.522	1.402	1.354	0.7	0.657	0.681
TRINITY_DN4451_c0_g2_i4_orf1	uncharacterized protein LOC114361986 isoform X1 [Ostrinia furnacalis]	7	180	18.6	9.16	60	15	6	High	1	1.02	0.98	0.655	0.7	0.718	0.597	0.597	0.581	0.696	0.663	0.718	0.463	0.461	0.477
TRINITY_DN9853_c0_g3_i1_orf1	importin-7 isoform X1 [Ostrinia furnacalis]	12	1049	120.6	4.92	13	17	12	High	1	0.991	0.95	0.537	0.511	0.586	0.501	0.442	0.493	0.444	0.444	0.42	0.515	0.496	0.509
TRINITY_DN54612_c0_g1_i3_orf1	hydroxyacid oxidase 1 isoform X1 [Ostrinia furnacalis]	9	367	40	8.69	38	15	9	High	1	0.964	0.95	1.134	1.121	1.129	1.373	1.342	1.317	1	0.999	1.031	0.91	0.947	0.957
TRINITY_DN5337_c0_g1_i6_orf1	epoxide hydrolase 1-like [Ostrinia furnacalis]	11	341	39.7	7.2	39	15	11	High	1	0.999	1.012	2.465	2.089	2.535	3.205	2.931	3.431	3.304	3.025	2.585	2.383	2.349	2.33
TRINITY_DN21555_c0_g1_i4_orf1	uncharacterized protein LOC114351844 [Ostrinia furnacalis]	12	553	60.8	5.71	28	16	12	High	1	0.971	0.972	1.343	1.323	1.279	1.559	1.641	1.606	1.515	1.472	1.448	3.826	3.778	3.724
TRINITY_DN27848_c0_g1_i2_orf1	cystathionine beta-synthase-like [Ostrinia furnacalis]	12	502	54.7	6.33	31	23	12	High	1	1.002	0.987	0.347	0.348	0.385	0.391	0.4	0.392	0.399	0.387	0.394	0.358	0.35	0.343
TRINITY_DN5748_c0_g1_i6_orf1	glycine N-methyltransferase isoform X1 [Ostrinia furnacalis]	9	243	27.4	6.09	48	22	3	High	1	1.045	1.051	3.151	3.112	2.879	2.139	2.195	2.15	2.067	1.787	2.34	1.254	1.388	1.265
TRINITY_DN63536_c0_g1_i1_orf1	adenosylhomocysteinase [Chelonus insularis]	6	436	48	6.71	13	20	1	High	1	0.745	1.077	1.696	1.751	1.642	1.436	1.715	1.636	1.292	1.893	2.334	1.114	1.281	1.333
TRINITY_DN24971_c0_g1_i3_orf1	uncharacterized protein LOC114352370 [Ostrinia furnacalis]	17	549	62.1	5.6	34	21	17	High	1	0.981	1.01	1.548	1.511	1.477	1.665	1.738	1.745	1.592	1.489	1.698	4.175	4.13	3.953
TRINITY_DN7960_c0_g1_i2_orf1	uncharacterized protein LOC114364878 [Ostrinia furnacalis]	13	320	34.5	9.04	50	30	13	High	1	1.038	1.037	2.146	1.949	2.085	2.591	2.329	2.48	2.98	2.679	2.78	1.36	1.361	1.481
TRINITY_DN1294_c0_g1_i3_orf1	46 kDa FK506-binding nuclear protein-like isoform X1 [Ostrinia furnacalis]	12	402	44.7	4.87	32	24	12	High	1	1.013	0.985	0.412	0.409	0.448	0.399	0.424	0.407	0.384	0.392	0.39	0.464	0.47	0.458
TRINITY_DN2069_c1_g1_i8_orf1	lysosomal aspartic protease [Trichoplusia ni]	13	384	41.7	8.31	48	19	13	High	1	0.97	0.993	1.413	1.318	1.362	1.685	1.679	1.642	1.517	1.678	1.459	2.672	2.501	2.789
TRINITY_DN3393_c0_g2_i1_orf1	40S ribosomal protein S8 [Ostrinia furnacalis]	11	210	23.9	10.58	53	20	11	High	1	0.937	0.971	0.668	0.714	0.662	0.597	0.638	0.598	0.547	0.553	0.589	0.589	0.587	0.611
TRINITY_DN7183_c0_g1_i2_orf1	seminal fluid protein CSSFP028 [Chilo suppressalis]	12	509	58.9	6.28	25	16	12	High	1	0.98	1.01	2.128	2.073	1.973	1.621	1.784	1.826	1.947	2.067	1.661	2.854	2.72	2.912
TRINITY_DN18482_c0_g1_i3_orf1	calcyphosin-like protein isoform X3 [Helicoverpa armigera]	11	223	25.6	6.42	56	22	9	High	1	1.022	0.999	1.978	2.014	1.882	2.382	2.308	2.214	2.481	2.337	2.817	1.113	1.085	1.112
TRINITY_DN4592_c0_g1_i1_orf1	hypothetical protein KGM_205563 [Danaus plexippus plexippus]	20	377	42.8	7.55	56	36	17	High	1	0.939	1.017	1.084	1.042	1.039	1.033	1.116	1.028	0.962	1.032	0.934	7.378	7.365	6.858
TRINITY_DN7251_c0_g1_i3_orf1	hypothetical protein evm_008498 [Chilo suppressalis]	9	272	29	10.46	53	15	9	High	1	0.985	1.019	0.573	0.578	0.6	0.543	0.537	0.518	0.486	0.515	0.558	0.801	0.803	0.851
TRINITY_DN120439_c1_g1_i1_orf1	myosin heavy chain variant, partial [Bombyx mori]	13	107	12.3	9.69	65	32	7	High	1	1.016	1.011	0.796	0.88	0.828	0.723	0.797	0.727	0.629	0.594	0.768	0.393	0.369	0.41
TRINITY_DN14286_c0_g1_i5_orf1	insulin-like growth factor 2 mRNA-binding protein 1 isoform X1 [Galleria mell	14	534	58.1	8.66	30	27	14	High	1	1.022	0.996	0.814	0.72	0.791	0.846	0.846	0.835	0.819	0.794	0.754	0.819	0.825	0.836
TRINITY_DN1718_c6_g1_i4_orf1	adenosine kinase [Ostrinia furnacalis]	10	350	38.2	5.52	41	21	10	High	1	1.013	1.024	1.015	1.038	1.011	1.179	1.176	1.209	1.046	1	1.081	0.729	0.746	0.771
TRINITY_DN79000_c1_g1_i1_orf1	AT15141p, partial [Drosophila melanogaster]	8	158	17.8	4.25	47	23	8	High	1	1.071	0.965	0.592	0.64	0.658	0.613	0.588	0.582	0.578	0.516	0.623	0.712	0.765	0.678
TRINITY_DN106156_c1_g1_i1_orf1	arylphorin subunit alpha-like [Ostrinia furnacalis]	12	115	13.5	8.98	59	62	12	High	1	0.96	0.997	3.358	2.86	3.042	3.592	3.317	3.846	6.414	7.048	5.382	4.17	4.005	4.308
TRINITY_DN1196_c0_g1_i4_orf1	glucosamine-6-phosphate isomerase isoform X2 [Ostrinia furnacalis]	8	270	30.4	7.36	45	15	1	High	1	1.167	1.16	1.706	1.513	1.38	1.293	0.931	1.169	1.073	0.915	1.07	2.495	2.195	2.21
TRINITY_DN170_c1_g1_i5_orf1	regulator of chromosome condensation isoform X2 [Helicoverpa zea]	11	608	63.6	4.96	25	13	11	High	1	1.044	0.978	0.701	0.775	0.758	0.623	0.642	0.628	0.612	0.57	0.627	0.783	0.805	0.755
TRINITY_DN7247_c0_g1_i7_orf1	pyruvate kinase-like isoform X3 [Ostrinia furnacalis]	12	288	31.2	8.53	55	22	4	High	1	1.068	1.094	0.704	0.783	0.744	0.761	0.75	0.755	0.649	0.663	0.592	0.906	0.94	0.97
TRINITY_DN32822_c0_g1_i1_orf1	eukaryotic translation initiation factor 2 subunit 1 [Ostrinia furnacalis]	11	331	37	5.05	42	19	11	High	1	1.017	1.005	0.735	0.655	0.722	0.691	0.696	0.744	0.694	0.663	0.599	0.671	0.677	0.685
TRINITY_DN31118_c0_g1_i1_orf1	CPR9 [Ostrinia furnacalis]	8	107	11.4	4.54	78	101	8	High	1	1.008	0.959	1.519	1.6	1.521	1.541	1.591	1.426	1.467	1.357	1.677	0.309	0.285	0.319
TRINITY_DN3840_c0_g1_i1_orf1	probable 2-oxoglutarate dehydrogenase E1 component DHKTD1 homolog, m	16	918	103.3	6.84	16	19	16	High	1	0.944	0.998	0.88	0.84	0.867	0.883	0.847	0.875	0.88	0.874	0.834	0.725	0.736	0.732
TRINITY_DN19079_c0_g1_i5_orf1	unnamed protein product [Euphydryas editha]	11	1301	140.7	7.44	13	13	11	High	1	0.976	0.978	0.788	0.76	0.773	0.695	0.694	0.724	0.677	0.677	0.728	0.846	0.823	0.894
TRINITY_DN11153_c0_g1_i1_orf1	unnamed protein product [Chilo suppressalis]	16	865	100.1	7.88	20	20	16	High	1	0.992	0.956	0.447	0.458	0.489	0.457	0.495	0.476	0.397	0.415	0.383	0.438	0.446	0.459
TRINITY_DN1265_c0_g1_i4_orf1	fumarylacetoacetate hydrolase domain-containing protein 2 isoform X3 [Ostri	12	334	36.6	8.22	42	19	1	High	1	1.018	1.056	0.919	0.9	0.878	0.996	1.379	1.166	1.058	0.969	1.093	0.794	0.831	0.794
TRINITY_DN119797_c0_g1_i1_orf1	hypothetical protein evm_013979 [Chilo suppressalis]	12	261	28.1	10.45	44	35	12	High	1	0.979	1.027	0.968	0.931	0.923	0.879	0.854	0.885	0.816	0.802	0.789	0.829	0.847	0.855
TRINITY_DN4794_c1_g1_i9_orf1	D-3-phosphoglycerate dehydrogenase [Ostrinia furnacalis]	11	327	34.6	7.81	42	23	11	High	1	0.993	1.016	1.242	1.304	1.248	1.122	1.07	1.082	1.447	1.378	1.469	1.154	1.169	1.168
TRINITY_DN8076_c0_g1_i5_orf1	hypothetical protein evm_001812 [Chilo suppressalis]	15	1030	113.1	7.74	15	18	2	High	1	0.922	0.978	0.74	0.66	0.733	0.734	0.741	0.839	0.825	0.775	0.666	0.741	0.799	0.839
TRINITY_DN13385_c0_g1_i4_orf1	putative aminopeptidase W07G4.4 [Ostrinia furnacalis]	13	512	55.7	7.93	28	16	12	High	1	0.964	1.032	1.228	1.188	1.149	1.1	1.084	1.081	0.93	0.867	0.95	0.917	0.908	0.897
TRINITY_DN13576_c0_g1_i1_orf1	uncharacterized protein LOC114350099 [Ostrinia furnacalis]	9	261	29.1	5.92	48	17	9	High	1	1.02	1.02	0.998	0.989	0.972	0.95	0.936	1.02	0.874	0.721	0.735	6.633	6.522	6.228
TRINITY_DN26209_c0_g1_i6_orf1	uncharacterized protein LOC114352357 [Ostrinia furnacalis]	11	256	27.3	7.99	54	25	11	High	1	0.985	1.007	2.679	2.711	2.58	3.723	3.648	3.627	2.921	2.72	3.037	3.018	2.996	2.847
TRINITY_DN8535_c0_g1_i9_orf1	unnamed protein product, partial [Iphiclides podalirius]	7	128	14.5	6.77	48	14	7	High	1	0.988	0.997	0.99	1.055	0.964	0.859	0.919	0.865	0.885	0.877	0.973	0.896	0.846	0.885
TRINITY_DN15420_c0_g3_i2_orf1	elongation factor 1-alpha 2-like [Galleria mellonella]	14	462	50.6	9.01	30	52	3	High	1	1.005	0.934	0.94	0.837	0.989	1.164	1.113	1.119	1.226	1.111	1.02	2.062	2.214	2.084

TRINITY_DN9733_c0_g1_i2_orf1	acidic juvenile hormone-suppressible protein 1-like [Ostrinia furnacalis]	7	104	12.1	6.04	54	54	3	High	1	0.923	0.998	1.732	1.855	1.787	1.471	1.632	1.508	3.948	3.823	4.072	3.122	3.282	2.876
TRINITY_DN4125_c1_g1_i5_orf1	angiotensin-converting enzyme-like isoform X2 [Ostrinia furnacalis]	10	648	74.4	5.25	21	15	10	High	1	0.994	0.993	0.426	0.404	0.448	0.387	0.424	0.44	0.41	0.442	0.404	0.558	0.547	0.554
TRINITY_DN2425_c1_g1_i4_orf1	hypothetical protein evm_005970 [Chilo suppressalis]	16	798	87.6	6.65	21	23	16	High	1	0.982	0.971	0.988	0.884	0.968	0.92	0.925	0.964	0.891	0.869	0.816	0.963	0.963	0.984
TRINITY_DN6291_c0_g1_i4_orf1	succinate-semialdehyde dehydrogenase, mitochondrial [Ostrinia furnacalis]	12	506	54.5	7.97	29	15	12	High	1	0.987	1.011	0.914	0.927	0.914	1.168	1.14	1.158	1.109	1.01	1.073	0.954	0.983	0.965
TRINITY_DN1023_c1_g1_i1_orf1	ras-related protein Rab-7a [Ostrinia furnacalis]	11	208	23.4	5.49	70	12	11	High	1	0.952	1.011	1.326	1.317	1.282	1.325	1.324	1.326	1.249	1.443	1.205	1.387	1.368	1.384
TRINITY_DN48638_c0_g1_i5_orf1	NADH dehydrogenase [ubiquinone] flavoprotein 2, mitochondrial [Ostrinia fur	11	245	27.1	6.4	50	19	11	High	1	1.023	1.021	0.795	0.847	0.835	0.677	0.705	0.641	0.726	0.794	0.74	0.688	0.649	0.684
TRINITY_DN917_c0_g1_i6_orf1	cartilage oligomeric matrix protein [Ostrinia furnacalis]	11	1129	125	4.86	14	21	11	High	1	1.009	1.017	1.19	1.14	1.265	1.144	1.067	1.203	1.117	1.096	1.019	1.146	1.157	1.184
TRINITY_DN19260_c0_g1_i5_orf1	probable 26S proteasome non-ATPase regulatory subunit 3 [Ostrinia furnacalis	16	495	56.4	8.24	36	19	13	High	1	0.986	1.023	0.989	0.985	1.018	0.79	0.794	0.821	0.752	0.724	0.768	1.003	0.981	1.038
TRINITY_DN620_c0_g1_i4_orf1	lysine--tRNA ligase isoform X1 [Ostrinia furnacalis]	15	443	50.6	5.73	42	22	13	High	1	0.989	0.969	0.491	0.521	0.545	0.481	0.493	0.469	0.401	0.383	0.413	0.437	0.434	0.429
TRINITY_DN4152_c0_g1_i1_orf1	importin subunit beta-1 isoform X2 [Ostrinia furnacalis]	14	885	98.1	4.87	20	18	14	High	1	0.957	0.954	0.726	0.719	0.747	0.671	0.696	0.697	0.668	0.767	0.68	0.868	0.889	0.915
TRINITY_DN98538_c0_g1_i1_orf1	ATP synthase subunit d, mitochondrial [Ostrinia furnacalis]	12	173	19.6	5.2	60	25	12	High	1	1.003	0.996	0.523	0.545	0.562	0.518	0.503	0.527	0.514	0.572	0.507	0.497	0.492	0.487
TRINITY_DN11612_c0_g3_i1_orf1	hypothetical protein O3G_MSEX005294 [Manduca sexta]	11	864	97.2	5.86	14	16	10	High	1	1.029	0.988	0.759	0.713	0.742	0.79	0.728	0.76	0.678	0.615	0.712	0.843	0.764	0.791
TRINITY_DN33801_c0_g1_i1_orf1	unnamed protein product [Diatraea saccharalis]	15	403	42.9	7.23	46	23	15	High	1	1.009	1.016	0.594	0.588	0.61	0.581	0.566	0.556	0.493	0.462	0.519	0.664	0.703	0.701
TRINITY_DN5497_c0_g1_i6_orf1	1,2-dihydroxy-3-keto-5-methylthiopentene dioxygenase-like [Ostrinia furnac	12	186	21.9	5.99	67	17	12	High	1	0.982	0.977	1.28	1.31	1.356	1.666	1.708	1.696	1.469	1.474	1.44	1.295	1.357	1.312
TRINITY_DN11177_c0_g1_i4_orf1	calcium-binding mitochondrial carrier protein Aralar1 isoform X1 [Ostrinia furr	12	682	76.1	8.65	23	15	11	High	1	0.961	0.96	1.01	0.931	0.935	1.069	1.079	1.06	0.962	1.095	1.08	0.93	0.891	0.894
TRINITY_DN11448_c0_g1_i15_orf1	unnamed protein product [Chilo suppressalis]	14	244	27	9.6	52	33	2	High	1	1.041	0.992	1.171	1.186	1.164	0.973	1.051	0.958	0.919	0.792	0.999	0.527	0.486	0.468
TRINITY_DN44288_c0_g1_i2_orf1	ATP-dependent RNA helicase p62 [Ostrinia furnacalis]	11	553	60.3	9.23	25	14	8	High	1	0.967	0.989	0.211	0.205	0.256	0.266	0.282	0.285	0.275	0.287	0.267	0.349	0.355	0.356
TRINITY_DN7131_c0_g1_i2_orf1	short/branched chain specific acyl-CoA dehydrogenase, mitochondrial [Ostrini	11	419	46.5	7.39	35	22	11	High	1	1.059	1.027	0.937	0.961	0.96	1.002	0.977	0.984	0.908	0.869	0.938	0.778	0.82	0.796
TRINITY_DN5190_c0_g3_i1_orf1	muscle LIM protein Mlp84B-like isoform X2 [Chelonus insularis]	10	493	53	8.22	23	26	1	High	1	0.966	0.967	1.35	1.357	1.309	1.173	1.434	1.416	1.125	1.019	1.237	1.367	1.367	1.377
TRINITY_DN26688_c0_g1_i2_orf1	myogenesis-regulating glycosidase-like [Ostrinia furnacalis]	11	621	71.1	4.82	21	21	11	High	1	1.018	0.998	0.239	0.221	0.279	0.236	0.232	0.238	0.231	0.223	0.215	0.315	0.309	0.297
TRINITY_DN4744_c0_g1_i7_orf1	glutaryl-CoA dehydrogenase, mitochondrial [Ostrinia furnacalis]	9	424	46.6	8.35	32	14	9	High	1	1.002	0.987	1.072	1.141	1.115	1.144	1.097	1.147	0.962	0.877	0.967	0.827	0.808	0.839
TRINITY_DN10521_c0_g1_i7_orf1	tubulin beta chain-like [Ostrinia furnacalis]	13	410	45.9	6.09	36	22	4	High	1	0.994	1.062	0.615	0.579	0.629	0.619	0.582	0.592	0.6	0.607	0.577	0.54	0.533	0.517
TRINITY_DN8258_c0_g1_i6_orf1	unnamed protein product [Chilo suppressalis]	13	818	89.2	5.06	19	21	2	High	1	1.028	0.944	1.144	1.093	1.135	1.203	1.301	1.015	1.077	0.993	1.141	0.854	0.657	0.706
TRINITY_DN1265_c0_g1_i9_orf1	fumarylacetoacetate hydrolase domain-containing protein 2 isoform X1 [Ostrir	12	337	36.9	8.22	44	19	1	High	1	0.829	1.182	1.211	1.301	1.323	1.489	1.081	1.344	1.228	1.894	1.155	1.079	0.964	1.319
TRINITY_DN934_c2_g1_i7_orf1	ubiquitin-40S ribosomal protein S27a [Ostrinia furnacalis]	10	158	18.2	9.77	49	53	3	High	1	0.947	0.975	0.762	0.891	0.814	0.762	0.801	0.833	0.745	0.768	0.776	0.965	0.981	0.963
TRINITY_DN20279_c0_g1_i1_orf1	NADH dehydrogenase [ubiquinone] iron-sulfur protein 3, mitochondrial [Ostri	11	265	30.2	5.97	51	14	11	High	1	1.031	1.017	0.606	0.625	0.629	0.601	0.616	0.616	0.615	0.675	0.631	0.562	0.6	0.587
TRINITY_DN125140_c0_g1_i1_orf1	glycogen debranching enzyme isoform X2 [Ostrinia furnacalis]	8	423	45.8	6.95	27	13	8	High	1	1.018	1.019	1.847	1.921	1.802	1.544	1.565	1.522	1.794	1.808	1.96	1.75	1.821	1.707
TRINITY_DN1091_c0_g3_i1_orf1	macrophage mannose receptor 1-like [Ostrinia furnacalis]	10	338	38.3	6.35	34	18	9	High	1	0.955	1.007	1.341	1.464	1.408	3.37	3.445	3.032	2.713	2.452	3.125	1.269	1.228	1.314
TRINITY_DN9592_c0_g1_i2_orf1	coatomer subunit delta [Ostrinia furnacalis]	11	506	55.8	6.24	26	14	11	High	1	0.967	1.012	0.889	0.927	0.906	0.998	1.074	1.052	0.871	0.849	0.887	1.126	1.062	1.075
TRINITY_DN136906_c0_g1_i1_orf1	translational elongation factor-1alpha, partial [Ethmia eupostica]	6	105	11.2	7.34	87	24	2	High	1	1.071	1.046	0.932	0.881	0.882	0.792	0.69	0.79	0.722	0.74	0.733	0.622	0.594	0.636
TRINITY_DN779_c0_g1_i3_orf1	uncharacterized protein LOC114351172 isoform X1 [Ostrinia furnacalis]	13	1503	163.2	9.29	13	19	6	High	1	0.985	1.005	1.295	1.45	1.328	1.204	1.202	1.194	1.199	1.027	1.332	0.814	0.808	0.823
TRINITY_DN71840_c0_g1_i1_orf1	60S ribosomal protein L7 [Ostrinia furnacalis]	14	262	30.7	10.52	47	34	14	High	1	0.987	1.011	0.697	0.707	0.686	0.663	0.668	0.668	0.59	0.583	0.592	0.594	0.61	0.625
TRINITY_DN28989_c0_g1_i7_orf1	26S proteasome regulatory subunit 10B [Ostrinia furnacalis]	10	396	44.8	7.8	32	17	10	High	1	1.04	1.021	1.078	1.088	1.07	0.92	0.943	0.984	0.959	0.992	0.895	1.19	1.175	1.192
TRINITY_DN2464_c0_g1_i12_orf1	uncharacterized protein LOC114362996 isoform X1 [Ostrinia furnacalis]	8	335	37.2	5.2	45	16	8	High	1	0.981	0.98	2.932	3.133	2.895	3.34	2.901	2.893	3.02	2.453	3.317	0.966	1.042	0.999
TRINITY_DN2591_c0_g1_i4_orf1	26S proteasome non-ATPase regulatory subunit 13 isoform X1 [Ostrinia furnac	12	357	39.8	6.8	38	15	12	High	1	0.982	0.972	0.957	0.873	0.902	0.776	0.813	0.793	0.844	0.851	0.862	1.15	1.19	1.187
TRINITY_DN8595_c0_g1_i3_orf1	aldose reductase-like isoform X4 [Trichoplusia ni]	5	122	13.9	5.73	67	33	1	High	1	0.951	0.941	3.42	3.375	3.391	4.459	4.406	4.486	3.458	3.615	3.359	1.681	1.631	1.73
TRINITY_DN13718_c0_g1_i7_orf1	immulectin-4 [Ostrinia furnacalis]	10	317	35.4	5.52	50	17	6	High	1	1.013	1.027	0.751	0.682	0.738	0.75	0.783	0.749	0.815	0.804	0.816	0.555	0.52	0.554
TRINITY_DN33249_c0_g1_i1_orf1	eukaryotic translation initiation factor 2 subunit 3-like isoform X2 [Spodoptera	13	468	50.5	8.91	35	16	13	High	1	0.975	1.006	0.655	0.627	0.642	0.621	0.61	0.642	0.633	0.62	0.557	0.703	0.704	0.715
TRINITY_DN8702_c0_g1_i1_orf1	programmed cell death protein 4 isoform X1 [Ostrinia furnacalis]	9	446	49.7	5.67	23	21	9	High	1	1.007	1.031	1.616	1.53	1.59	1.696	1.626	1.631	1.512	1.492	1.457	1.207	1.19	1.191
TRINITY_DN230_c2_g1_i5_orf1	6-pyruvoyl tetrahydrobiopterin synthase [Ostrinia furnacalis]	8	164	18.7	7.36	62	20	8	High	1	1.029	1.011	2.443	2.374	2.34	1.642	1.686	1.646	1.311	1.221	1.414	1.101	1.071	1.105
TRINITY_DN5852_c0_g1_i6_orf1	probable maltase isoform X6 [Ostrinia furnacalis]	11	459	50.8	5.77	25	18	10	High	1	1.033	1.015	1.419	1.465	1.456	0.934	0.958	0.968	1.383	1.241	1.416	1.059	1.079	1.024
TRINITY_DN1355_c0_g1_i7_orf1	larval/pupal rigid cuticle protein 66-like [Ostrinia furnacalis]	8	230	23	7.42	43	19	5	High	1	0.978	1.012	1.19	1.226	1.211	1.246	1.287	1.309	1.416	1.444	1.381	1.452	1.378	1.339
TRINITY_DN5748_c0_g1_i5_orf1	glycine N-methyltransferase isoform X1 [Ostrinia furnacalis]	7	233	26.3	6.02	41	23	1	High	1	0.938	0.986	1.354	1.353	1.442	1.405	1.641	1.562	1.537	1.262	1.304	1.026	1.115	1.048
TRINITY_DN2274_c0_g1_i6_orf1	membrane alanyl aminopeptidase-like [Ostrinia furnacalis]	12	1013	114.7	5.39	15	14	11	High	1	0.982	1.016	0.399	0.382	0.409	0.425	0.471	0.432	0.432	0.49	0.436	0.43	0.41	0.413
TRINITY_DN91989_c0_g1_i1_orf1	protein l(2)37Cc [Pectinophora gossypiella]	9	274	30	6.83	34	15	9	High	1	0.993	0.971	0.561	0.529	0.564	0.505	0.517	0.568	0.583	0.6	0.524	0.595	0.586	0.616
TRINITY_DN28039_c0_g1_i1_orf1	translation elongation factor 2 [Athalia rosae]	12	877	98.2	6.52	16	27	1	High	1	1.025	0.987	1.131	1.145	1.03	0.951	0.819	0.702	0.709	0.782	0.854	0.832	0.748	0.812
TRINITY_DN2365_c0_g1_i6_orf1	carnitine O-acetyltransferase isoform X2 [Ostrinia furnacalis]	13	644	72.4	8.21	23	18	13	High	1	1.044	0.974	0.565	0.616	0.623	0.621	0.672	0.623	0.635	0.601	0.683	0.776	0.738	0.763
TRINITY_DN5740_c0_g1_i4_orf1	unconventional myosin IC isoform X1 [Ostrinia furnacalis]	15	1042	119.7	9.31	15	16	15	High	1	1.033	1.033	0.2	0.191	0.246	0.243	0.249	0.239	0.243	0.273	0.241	0.251	0.244	0.247
TRINITY_DN60787_c0_g1_i5_orf1	probable transaldolase [Ostrinia furnacalis]	13	332	37.1	7.83	39	18	13	High	1	0.951	0.952	1.315	1.297	1.25	1.293	1.315	1.273	1.315	1.348	1.33	0.972	0.983	1.055
TRINITY_DN1443_c0_g1_i4_orf1	ATP-dependent RNA helicase DDX3X isoform X1 [Ostrinia furnacalis]	13	739	82.5	8.06	19	22	12	High	1	1.017	1.018	0.711	0.686	0.772	0.736	0.739	0.761	0.664	0.628	0.631	0.759	0.767	0.699
TRINITY_DN50676_c0_g1_i1_orf1	uncharacterized protein LOC114360659 [Ostrinia furnacalis]	15	566	62.3	7.74	33	18	15	High	1	1.005	1.006	0.774	0.81	0.792	0.884	0.92	0.853	0.673	0.639	0.707	0.555	0.552	0.55
TRINITY_DN98334_c0_g1_i1_orf1	unnamed protein product [Spodoptera littoralis]	8	340	34	9.01	52	20	8	High	1	0.97	1.019	0.944	0.905	0.888	0.912	0.971	0.892	0.895	1.032	0.861	5.053	5.172	4.917
TRINITY_DN44407_c0_g4_i2_orf1	eukaryotic translation initiation factor 5A [Antheraea pernyi]	6	160	17.6	5.4	29	19	3	High	1	1.176	1.089	0.908	0.928	0.87	0.942	0.557	0.737	0.738	0.62	0.826	0.973	0.998	0.863
TRINITY_DN5952_c0_g1_i6_orf1	LOW QUALITY PROTEIN: phosphoacetylglucosamine mutase [Ostrinia furnacal	12	548	59.7	6.32	29	14	12	High	1	0.985	0.967	0.725	0.7	0.742	0.701	0.707	0.713	0.656	0.694	0.658	0.939	0.928	0.896
TRINITY_DN19361_c0_g1_i7_orf1	hydroxylysine kinase [Ostrinia furnacalis]	9	372	42.1	5.83	31	13	9	High	1	1.027	1.011	0.396	0.409	0.44	0.37	0.388	0.394	0.406	0.381	0.41	0.535	0.502	0.514
TRINITY_DN17574_c0_g1_i2_orf1	heat shock protein Hsp-12.2-like [Ostrinia furnacalis]	7	166	19.4	7.05	51	19	7	High	1	0.975	0.97	1.319	1.354	1.31	3.271	3.279	3.046	3.965	3.7	4.121	3.218	3.174	3.13
TRINITY_DN2802_c0_g1_i1_orf1	far upstream element-binding protein 1 isoform X3 [Ostrinia furnacalis]	10	620	65.3	7.27	21	17	10	High	1	1.002	0.991	0.687	0.692	0.732	0.667	0.664	0.629	0.639	0.584	0.616	0.878	0.892	0.847
TRINITY_DN11194_c0_g1_i4_orf1	ATPase family AAA domain-containing protein 3A homolog [Ostrinia furnacalis	13	629	70.3	9.01	21	16	13	High	1	1.009	1.009	0.646	0.654	0.669	0.723	0.69	0.66	0.589	0.575	0.625	0.476	0.477	0.481
TRINITY_DN9242_c0_g1_i1_orf1	electron transfer flavoprotein subunit alpha, mitochondrial [Ostrinia furnacalis]	11	330	34.6	8.43	38	17	11	High	1	0.962	0.975	0.852	0.832	0.86	0.949	0.966	0.926	0.952	0.908	0.941	0.742	0.72	0.755
TRINITY_DN2425_c0_g1_i3_orf1	sialic acid synthase [Ostrinia furnacalis]	12	349	38.8	7.2	38	18	12	High	1	1.042	0.989	1.612	1.608	1.664	1.899	1.898	1.874	1.777	1.635	1.806	2.634	2.833	2.481
TRINITY_DN14684_c0_g2_i1_orf1	cofilin/actin-depolymerizing factor homolog isoform X2 [Helicoverpa armigera	13	148	17	6.57	61	26	13	High	1	0.986	1.012	1.009	1.044	1.023	0.98	1.007	0.971	0.981	1.046	1.035	1.203	1.191	1.269
TRINITY_DN18222_c0_g1_i5_orf1	phosphoglycerate kinase [Maniola hyperantus]	12	398	42.5	6.67	30	26	6	High	1	1.003	1.021	1.097	1.068	1.088	1.116	1.137	1.125	1.272	1.223	1.233	0.893	0.927	0.893
TRINITY_DN471_c0_g1_i6_orf1	ATP-dependent RNA helicase DBP2-A-like [Ostrinia furnacalis]	11	704	76.6	9.73	19	15	7	High	1	0.996	1.06	0.849	0.799	0.842	0.715	0.776	0.746	0.851	0.821	0.836	1.046	1.023	1.048
TRINITY_DN2930_c0_g1_i8_orf1	hypothetical protein evm_000171 [Chilo suppressalis]	13	359	41.5	5.25	35	17	13	High	1	1.014	1.051	0.796	0.73	0.802	0.795	0.734	0.812	0.84	0.815	0.754	0.905	0.894	0.908
TRINITY_DN20676_c0_g1_i6_orf1	aldo-keto reductase AKR2E4-like isoform X1 [Ostrinia furnacalis]	15	357	40.6	6.21	47	19	13	High	1	0.991	1.006	2.503	2.501	2.388	2.879	2.945	2.834	2.674	2.578	2.74	4.258	4.3	4.172
TRINITY_DN2627_c0_g1_i2_orf1	probable cytosolic oligopeptidase A [Ostrinia furnacalis]	13	722	83.4	7.34	20	17	13	High	1	0.998	0.999	0.548	0.53	0.563	0.574	0.59	0.577	0.586	0.623	0.569	0.629	0.595	0.647
TRINITY_DN7161_c0_g1_i7_orf1	ATP-binding cassette sub-family E member 1 [Ostrinia furnacalis]	14	612	69.1	8.09	24	17	14	High	1	1.015	0.979	0.751	0.744	0.802	0.703	0.689	0.713	0.656	0.658	0.646	0.728	0.748	0.748
TRINITY_DN1732_c0_g1_i17_orf1	CAD protein isoform X2 [Ostrinia furnacalis]	12	653	72.9	5.8	22	14	2	High	1	0.958	0.949	0.817	0.877	0.922	1.198	1.372	1.396	1.459	1.431	1.677	1.183	1.109	1.105
TRINITY_DN19375_c0_g1_i2_orf1	vacuolar protein sorting-associated protein 4 [Pararge aegeria]	12	440	49.3	7.02	30	16	12	High	1	1.056	1.043	1.168	1.129	1.114	1.1	1.002	1.021	0.979	0.924	1.095	1.138	1.158	1.1
TRINITY_DN5234_c0_g1_i2_orf1	proline dehydrogenase 1, mitochondrial isoform X2 [Ostrinia furnacalis]	12	577	66.2	9.17	27	14	12	High	1	1.009	1.042	0.643	0.627	0.633	0.594	0.608	0.646	0.708	0.796	0.751	0.602	0.585	0.63
TRINITY_DN1445_c0_g2_i4_orf1	leucine-rich PPR motif-containing protein, mitochondrial [Ostrinia furnacalis]	13	534	60.7	8.1	28	15	2	High	1	1.045	0.995	0.663	0.69	0.697	0.663	0.697	0.584	0.643	0.731	0.663	0.402	0.521	0.513
TRINITY_DN4292_c1_g1_i3_orf1	hypothetical protein evm_004364 [Chilo suppressalis]	11	471	52.6	5.72	28	21	11	High	1	0.957	0.972	0.894	0.895	0.917	0.986	0.999	1.022	0.854	0.843	0.879	0.869	0.869	0.837
TRINITY_DN8343_c0_g1_i2_orf1	multidrug resistance-associated protein 1 isoform X4 [Ostrinia furnacalis]	12	1518	169.3	7.15	10	15	12	High	1	0.976	0.985	0.725	0.736	0.731	0.622	0.591	0.65	0.62	0.646	0.61	0.657	0.638	0.642
TRINITY_DN56459_c0_g1_i2_orf1	aldo-keto reductase AKR2E4-like [Ostrinia furnacalis]	5	74	8.7	5.15	78	30	4	High	1	0.951	0.982	5.649	6.179	5.71	6.25	6.398	5.707	6.173	5.666	7.406	2.237	2.086	2.282
TRINITY_DN5458_c0_g1_i3_orf1	LOW QUALITY PROTEIN: histone H2B-like [Manduca sexta]	9	124	13.8	10.29	58	35	9	High	1	0.989	0.993	1.136	1.151	1.115	1.179	1.113	1.126	1.027	1.137	1.032	1.058	1.065	1.046
TRINITY_DN892_c7_g1_i2_orf1	unnamed protein product [Diatraea saccharalis]	11	432	48.7	7.58	29	33	11	High	1	1.051	1.065	1.984	2.081	1.985	2.276	2.251	2.203	1.66	1.386	1.807	1.965	1.946	1.909
TRINITY_DN37830_c0_g1_i1_orf1	60S ribosomal protein L18a [Galleria mellonella]	14	177	20.9	10.4	55	30	14	High	1	0.996	1.008	0.801	0.783	0.813	0.742	0.714	0.749	0.679	0.733	0.677	0.711	0.711	0.708
TRINITY_DN8466_c0_g1_i1_orf1	26S proteasome regulatory subunit 6A-B [Ostrinia furnacalis]	11	428	47.7	5.22	28	20	10	High	1	0.994	0.993	0.85	0.862	0.889	0.785	0.858	0.85	0.82	0.954	0.777	0.987	0.974	0.974
TRINITY_DN441_c0_g2_i1_orf1	guanine nucleotide-binding protein subunit beta-like protein [Diachasma allo	9	317	35.8	7.74	38	22	1	High	1	1.212	1.303	0.959	0.892	0.738	0.871	0.55	0.784	0.56	0.563	0.562	0.701	0.675	0.68
TRINITY_DN11065_c0_g2_i1_orf1	ribosomal protein s6e domain-containing protein [Phthorimaea operculella]	11	253	29	10.83	37	24	10	High	1	1.01	1.004	0.744	0.817	0.79	0.683	0.7	0.688	0.54	0.629	0.557	0.585	0.579	0.575
TRINITY_DN1820_c0_g1_i6_orf1	3-hydroxyisobutyryl-CoA hydrolase, mitochondrial [Ostrinia furnacalis]	13	367	41.3	8.35	42	17	13	High	1	0.981	1.024	0.63	0.632	0.619	0.663	0.704	0.651	0.625	0.614	0.671	0.665	0.666	0.651
TRINITY_DN768_c0_g1_i7_orf1	neutral ceramidase [Leguminivora glycinivorella]	10	704	77.2	6.8	20	12	10	High	1	0.932	0.928	2.54	2.697	2.49	2.242	2.235	2.196	2.591	2.83	2.991	2.514	2.509	2.556
TRINITY_DN13500_c0_g1_i1_orf1	phosphatidylethanolamine-binding protein homolog F40A3.3-like [Ostrinia fu	10	184	20.5	6.8	64	31	10	High	1	0.977	0.989	1.546	1.532	1.505	1.809	1.737	1.779	2.064	2.304	2.065	1.46	1.439	1.445
TRINITY_DN276_c0_g1_i2_orf1	protein lethal(2)essential for life-like [Ostrinia furnacalis]	9	183	20.8	6.9	43	26	2	High	1	0.991	0.862	1.507	1.433	1.568	3.171	3.258	3.266	5.302	4.195	5.307	11.772	12.626	10.844
TRINITY_DN24970_c0_g1_i4_orf1	pyrroline-5-carboxylate reductase-like isoform X1 [Ostrinia furnacalis]	7	283	29.5	7.78	33	19	7	High	1	0.977	0.992	1.729	1.764	1.736	1.762	1.74	1.723	1.743	1.563	1.724	1.152	1.158	1.155
TRINITY_DN4309_c0_g1_i1_orf1	NEDD8-conjugating enzyme Ubc12, partial [Cotesia chilonis]	8	162	17.9	5.41	40	17	5	High	1	1.029	0.945	0.551	0.604	0.631	0.697	0.657	0.645	0.576	0.487	0.584	0.627	0.711	0.596
TRINITY_DN4341_c0_g1_i4_orf1	uncharacterized protein LOC114354354 [Ostrinia furnacalis]	14	514	59.1	8.34	33	15	14	High	1	1.035	0.99	1.227	1.16	1.256	1.554	1.482	1.567	1.254	1.218	1.16	0.996	1.049	1.038
TRINITY_DN37307_c0_g1_i4_orf1	superoxide dismutase [Mn], mitochondrial [Ostrinia furnacalis]	9	216	24.2	8.82	50	21	9	High	1	1.002	0.998	2.658	2.711	2.466	2.014	1.731	1.89	1.758	1.619	2.045	1.649	1.57	1.593
TRINITY_DN22213_c0_g1_i3_orf1	hypothetical protein evm_003554 [Chilo suppressalis]	10	407	44.1	5.76	37	14	9	High	1	0.963	0.999	0.949	1.098	1.009	0.958	0.999	0.995	0.896	0.807	1.015	1.102	1.126	1.046
TRINITY_DN2205_c0_g1_i3_orf1	probable chitinase 2 [Ostrinia furnacalis]	14	508	57.1	5.99	29	18	14	High	1	0.996	0.987	1.607	1.599	1.645	1.963	1.968	1.964	1.793	1.731	1.747	2.088	2.108	2.046
TRINITY_DN33959_c0_g1_i1_orf1	hypothetical protein evm_002483 [Chilo suppressalis]	12	764	87.9	6.18	18	17	12	High	1	1.021	1.004	0.866	0.855	0.851	0.826	0.868	0.84	0.942	0.803	0.808	0.843	0.812	0.873
TRINITY_DN5122_c0_g1_i3_orf1	calumenin [Ostrinia furnacalis]	10	322	37.7	4.74	37	14	10	High	1	0.99	0.966	1.272	1.305	1.267	1.402	1.425	1.345	1.339	1.307	1.43	1.545	1.514	1.57
TRINITY_DN19639_c0_g2_i1_orf1	basic juvenile hormone-suppressible protein 1-like [Hyposmocoma kahamanc	10	71	8.6	5.9	72	50	2	High	1	1.045	0.905	1.045	0.956	0.977	2.838	2.194	3.154	1.807	2.252	1.193	4.955	4.538	5.504
TRINITY_DN566_c0_g1_i13_orf1	uncharacterized protein LOC114355567 isoform X2 [Ostrinia furnacalis]	5	306	32.5	5.29	23	8	5	High	1	0.999	0.987	1.723	1.563	1.673	1.994	1.763	1.875	1.506	1.49	1.405	1.078	1.161	1.128

TRINITY_DN14443_c0_g1_i1_orf1	SUMO-activating enzyme subunit 2 [Ostrinia furnacalis]	14	610	67	5.35	26	16	14	High	1	0.986	0.98	0.844	0.829	0.85	0.804	0.781	0.813	0.8	0.707	0.753	1.138	1.154	1.132
TRINITY_DN14298_c0_g1_i1_orf1	kinesin heavy chain [Ostrinia furnacalis]	11	710	79.6	5.24	19	15	10	High	1	0.987	1.016	0.831	0.88	0.847	0.783	0.819	0.815	0.766	0.703	0.768	1.037	1.032	1.05
TRINITY_DN1014_c0_g2_i8_orf1	uncharacterized protein LOC114362446 [Ostrinia furnacalis]	9	381	41.1	5.99	31	11	9	High	1	1.007	1.032	1.643	1.352	1.625	1.975	1.913	2.069	1.967	1.73	1.497	1.596	1.656	1.574
TRINITY_DN285_c0_g1_i4_orf1	catalase-like [Ostrinia furnacalis]	9	513	58.6	8.7	23	18	9	High	1	0.898	1.029	2.223	2.165	2.187	2.813	2.773	2.79	2.555	2.392	2.533	1.176	1.105	1.184
TRINITY_DN8224_c0_g1_i7_orf1	hemicentin-1-like isoform X2 [Ostrinia furnacalis]	14	1514	169.3	5.5	11	15	14	High	1	0.928	0.972	1.437	1.514	1.428	1.337	1.388	1.335	1.226	1.255	1.3	1.039	1.032	1.097
TRINITY_DN7630_c0_g2_i1_orf1	flotillin-2 isoform X1 [Ostrinia furnacalis]	12	440	48	5.2	34	17	12	High	1	0.958	0.965	1.626	1.742	1.65	1.683	1.759	1.703	1.53	1.516	1.539	1.255	1.242	1.257
TRINITY_DN2652_c0_g2_i1_orf1	peroxidase-like isoform X1 [Ostrinia furnacalis]	11	793	88.2	7.11	19	13	11	High	1	1.106	0.976	1.353	1.358	1.293	1.204	1.354	1.288	1.408	1.357	1.428	3.432	3.501	3.544
TRINITY_DN6317_c1_g2_i3_orf1	nucleosome assembly protein 1-like 1 isoform X2 [Ostrinia furnacalis]	8	401	45.5	4.75	31	12	8	High	1	1.009	0.969	0.807	0.804	0.832	0.864	0.846	0.857	0.795	0.731	0.766	0.873	0.86	0.901
TRINITY_DN8584_c0_g1_i6_orf1	uncharacterized protein LOC114354070 isoform X3 [Ostrinia furnacalis]	16	3154	355.9	4.77	6	19	16	High	1	0.969	0.947	0.746	0.782	0.777	0.804	0.827	0.769	0.774	0.7	0.804	0.664	0.658	0.65
TRINITY_DN50085_c0_g1_i1_orf1	hypothetical protein evm_013997 [Chilo suppressalis]	13	711	82.4	5.82	22	16	13	High	1	0.983	0.993	0.568	0.495	0.581	0.483	0.459	0.504	0.47	0.456	0.435	0.624	0.583	0.599
TRINITY_DN6308_c0_g1_i6_orf1	myc box-dependent-interacting protein 1 isoform X2 [Ostrinia furnacalis]	10	469	52.4	5.39	33	11	3	High	1	1.011	0.98	0.395	0.46	0.403	0.365	0.408	0.375	0.324	0.362	0.376	0.483	0.512	0.449
TRINITY_DN4816_c0_g2_i3_orf1	15-hydroxyprostaglandin dehydrogenase [NAD(+)]-like [Ostrinia furnacalis]	8	270	29.1	6.81	38	8	7	High	1	0.954	1.116	3.615	3.652	3.316	3.341	3.404	3.4	3.47	3.637	3.702	2.401	2.265	2.426
TRINITY_DN1024_c0_g4_i1_orf1	superoxide dismutase [Cu-Zn]-like [Ostrinia furnacalis]	8	172	18.2	6.51	58	16	8	High	1	1.023	1.003	2.318	2.616	2.346	2.316	2.169	2.156	2.079	2.078	2.393	1.385	1.329	1.254
TRINITY_DN565_c0_g2_i1_orf1	uncharacterized protein LOC114362323 [Ostrinia furnacalis]	7	257	27.4	7.33	34	13	7	High	1	1.015	1.015	0.956	0.99	0.978	1.433	1.493	1.352	0.986	0.99	1.06	0.685	0.671	0.703
TRINITY_DN9239_c0_g2_i2_orf1	apolipophorins-like [Ostrinia furnacalis]	9	67	7.6	5.86	90	19	5	High	1	0.974	1.082	1.446	1.604	1.361	1.439	1.326	1.263	2.042	1.963	2.769	3.18	2.8	3.017
TRINITY_DN465_c0_g1_i3_orf1	26S proteasome regulatory subunit 6B [Ostrinia furnacalis]	8	415	47	5.21	26	15	8	High	1	0.977	1.002	0.997	0.981	0.958	0.864	0.886	0.899	0.905	0.865	0.885	1.046	1.043	1.032
TRINITY_DN7464_c0_g1_i14_orf1	60S ribosomal protein L9 [Nymphalis io]	9	206	23.3	9.89	51	17	9	High	1	0.983	0.993	0.69	0.694	0.719	0.65	0.595	0.629	0.524	0.565	0.539	0.505	0.521	0.507
TRINITY_DN13496_c0_g1_i7_orf1	nucleolar protein 58 [Ostrinia furnacalis]	9	541	60.3	8.29	26	10	9	High	1	0.969	0.958	0.64	0.682	0.67	0.563	0.608	0.567	0.557	0.569	0.539	0.544	0.55	0.539
TRINITY_DN8980_c0_g1_i2_orf1	putative ATP-dependent RNA helicase me31b [Ostrinia furnacalis]	12	443	50.4	8.15	29	17	12	High	1	0.969	1.044	0.699	0.712	0.735	0.686	0.67	0.706	0.744	0.72	0.71	0.843	0.811	0.832
TRINITY_DN4532_c0_g1_i1_orf1	3-hydroxyacyl-CoA dehydrogenase type-2 [Ostrinia furnacalis]	9	255	27	9.07	44	16	9	High	1	0.973	1.01	0.541	0.568	0.547	0.575	0.621	0.564	0.515	0.538	0.547	0.493	0.493	0.496
TRINITY_DN5405_c1_g1_i13_orf1	acyl-CoA synthetase family member 3, mitochondrial [Ostrinia furnacalis]	9	637	70.2	8.43	17	15	9	High	1	1.006	1.015	1.047	1.085	1.062	1.151	1.227	1.192	1.026	1.04	1.059	0.873	0.847	0.848
TRINITY_DN117_c0_g1_i4_orf1	lipase member I-like [Ostrinia furnacalis]	9	152	16.1	7.2	82	18	5	High	1	0.988	0.965	0.036	0.048	0.059	0.071	0.082	0.078	0.067	0.07	0.078	0.172	0.142	0.179
TRINITY_DN891_c5_g1_i5_orf1	unnamed protein product, partial [Brenthis ino]	11	599	66.4	5.3	25	15	11	High	1	0.995	0.977	1.027	1.062	1.016	0.97	0.931	0.996	0.882	0.816	0.903	1.054	1.024	1.058
TRINITY_DN240_c2_g1_i1_orf1	alpha-soluble NSF attachment protein [Ostrinia furnacalis]	11	292	33	6.35	50	14	11	High	1	1.003	1.041	1.225	1.188	1.164	1.195	1.183	1.273	1.188	1.278	1.084	1.184	1.159	1.2
TRINITY_DN62_c0_g1_i7_orf1	tropomodulin-1 isoform X5 [Ostrinia furnacalis]	13	419	47.4	5.15	34	17	13	High	1	0.978	1.005	0.971	1.016	1.002	0.917	0.976	0.964	0.86	0.818	0.898	0.776	0.763	0.743
TRINITY_DN26503_c0_g1_i1_orf1	ruvB-like 2 isoform X1 [Ostrinia furnacalis]	10	466	51.2	5.81	22	14	10	High	1	0.988	1.002	0.777	0.799	0.798	0.721	0.783	0.787	0.704	0.716	0.72	0.842	0.789	0.821
TRINITY_DN64810_c0_g1_i1_orf1	arginine--tRNA ligase, cytoplasmic [Ostrinia furnacalis]	13	684	77.8	6.71	19	17	13	High	1	1.019	0.973	0.724	0.722	0.751	0.667	0.711	0.675	0.671	0.72	0.691	0.657	0.653	0.675
TRINITY_DN17326_c0_g1_i5_orf1	aminoacylase-1-like [Ostrinia furnacalis]	11	399	44.9	6.21	33	16	6	High	1	1.036	1.037	1.192	1.178	1.188	1.712	1.696	1.672	1.563	1.5	1.526	1.478	1.507	1.515
TRINITY_DN4464_c0_g2_i1_orf1	glypican-6 [Pectinophora gossypiella]	11	764	84.3	5.22	20	13	11	High	1	0.93	0.994	1.898	2.014	1.952	1.801	1.715	1.949	1.872	1.849	1.961	2.454	2.278	2.187
TRINITY_DN8724_c0_g1_i5_orf1	vesicle-associated membrane protein/synaptobrevin-binding protein isoform	12	279	31.3	7.47	51	18	3	High	1	1.073	0.951	0.931	0.891	1.012	1.007	0.929	0.9	0.828	0.737	0.798	0.821	0.879	0.806
TRINITY_DN8729_c0_g1_i7_orf1	myosin heavy chain 95F isoform X1 [Ostrinia furnacalis]	15	1243	142.7	8.88	12	17	15	High	1	0.995	0.994	0.676	0.683	0.711	0.738	0.754	0.691	0.771	0.807	0.79	0.763	0.745	0.79
TRINITY_DN359_c0_g1_i5_orf1	eukaryotic translation initiation factor 5 isoform X1 [Ostrinia furnacalis]	13	455	50.3	6.18	30	15	13	High	1	0.973	0.977	1.182	1.168	1.134	1.015	1.092	1.042	0.887	0.834	0.916	1.088	1.073	1.126
TRINITY_DN1445_c0_g2_i3_orf1	leucine-rich PPR motif-containing protein, mitochondrial [Ostrinia furnacalis]	12	523	59.3	8.1	26	14	1	High	1	0.804	0.961	0.756	0.872	0.777	0.74	0.956	0.699	0.626	0.854	0.89	0.671	0.757	0.839
TRINITY_DN1540_c0_g1_i14_orf1	alaserpin-like isoform X1 [Ostrinia furnacalis]	12	397	43.8	5.05	33	17	1	High	1	0.944	0.937	1.703	1.867	1.317	2.409	2.432	2.22	1.967	2.019	2.209	1.247	1.508	1.3
TRINITY_DN1540_c0_g1_i9_orf1	alaserpin-like isoform X9 [Ostrinia furnacalis]	12	396	44.1	5.3	34	16	1	High	1	0.901	0.741	1.088	0.968	1.052	1.428	1.988	1.748	1.57	1.846	1.186	0.985	0.922	0.891
TRINITY_DN1172_c0_g1_i1_orf1	hypothetical protein O3G_MSEX009550 [Manduca sexta]	7	285	28.6	8.46	42	15	6	High	1	1.013	1.051	1.337	1.265	1.21	1.355	1.397	1.368	1.297	1.286	1.331	1.05	1.031	1.072
TRINITY_DN34289_c0_g2_i2_orf1	succinyl-CoA:3-ketoacid coenzyme A transferase 1, mitochondrial [Ostrinia fur	9	523	57.1	7.91	26	15	9	High	1	0.996	1.006	1.041	1.071	1.013	1.051	1.115	1.087	1.121	1.109	1.168	1.019	1.081	1.1
TRINITY_DN9135_c0_g1_i4_orf1	electron transfer flavoprotein subunit beta [Ostrinia furnacalis]	9	253	27.2	8.47	49	13	9	High	1	1.002	0.99	0.917	0.959	0.934	1	1.022	0.981	1.041	0.916	1.099	0.772	0.807	0.839
TRINITY_DN28875_c0_g1_i1_orf1	PREDICTED: ras-related protein Rab-2 [Fopius arisanus]	8	214	23.6	6.39	42	16	8	High	1	0.996	1.001	1.438	1.245	1.355	1.342	1.276	1.456	1.353	1.305	1.132	1.267	1.266	1.351
TRINITY_DN2894_c0_g3_i1_orf1	lactase-phlorizin hydrolase-like [Ostrinia furnacalis]	12	499	57.5	5.02	28	13	10	High	1	1.014	0.982	0.295	0.309	0.326	0.295	0.284	0.292	0.291	0.33	0.29	0.297	0.286	0.303
TRINITY_DN6684_c0_g1_i4_orf1	26S proteasome non-ATPase regulatory subunit 6 isoform X1 [Ostrinia furnaca	11	382	43.4	6.16	31	17	11	High	1	1.012	0.996	1.217	1.213	1.201	0.966	0.954	1.007	0.844	0.886	0.799	1.066	1.024	1.005
TRINITY_DN4501_c0_g2_i1_orf1	methylcrotonoyl-CoA carboxylase subunit alpha, mitochondrial [Ostrinia furna	10	682	75.6	7.05	20	11	9	High	1	1.051	1.058	1.133	1.138	1.081	0.913	0.849	0.907	0.893	0.817	0.891	0.779	0.799	0.791
TRINITY_DN12401_c0_g2_i4_orf1	hydroxysteroid dehydrogenase-like protein 2 [Ostrinia furnacalis]	13	430	46.3	8.87	26	21	13	High	1	1.021	1.019	0.868	0.936	0.892	0.816	0.829	0.81	0.884	0.862	0.951	0.741	0.758	0.785
TRINITY_DN3209_c0_g1_i1_orf1	coatomer subunit beta [Helicoverpa armigera]	10	580	61.9	5.85	22	16	8	High	1	1.002	0.996	0.987	1.138	1.05	1.008	0.96	0.994	0.852	0.817	0.769	1	1.023	1.046
TRINITY_DN2133_c0_g2_i1_orf1	glycogen debranching enzyme isoform X1 [Ostrinia furnacalis]	9	676	75.3	5.99	19	14	9	High	1	1.019	1	1.828	1.941	1.817	1.589	1.585	1.555	1.831	1.755	1.902	1.61	1.629	1.582
TRINITY_DN41952_c0_g1_i4_orf1	protein DDI1 homolog 2 [Ostrinia furnacalis]	9	379	42	5.44	37	12	3	High	1	1.095	1.097	1.148	1.246	1.187	1.211	1.234	1.107	1.009	0.99	1.073	0.944	1.068	0.929
TRINITY_DN2457_c0_g1_i8_orf1	uncharacterized protein LOC114355596 [Ostrinia furnacalis]	7	292	33.4	8.78	27	9	7	High	1	0.94	1.021	6.429	6.946	5.98	3.191	3.569	3.261	2.748	3.484	3.06	1.372	1.286	1.393
TRINITY_DN14235_c0_g1_i1_orf1	maltase A1 [Helicoverpa armigera]	11	581	66.7	4.89	29	11	10	High	1	1.07	1.061	0.501	0.525	0.564	0.433	0.426	0.393	0.378	0.379	0.434	0.402	0.422	0.408
TRINITY_DN11446_c0_g1_i21_orf1	glutathione synthetase-like isoform X1 [Ostrinia furnacalis]	12	493	54.9	6.6	30	14	12	High	1	0.992	1.023	0.954	0.92	0.935	0.86	0.92	0.862	0.938	0.885	0.969	0.934	0.968	0.924
TRINITY_DN7686_c0_g1_i4_orf1	eIF-2-alpha kinase activator GCN1 [Ostrinia furnacalis]	12	1232	134.4	7.21	13	14	12	High	1	1.022	1.02	0.899	0.933	0.923	0.798	0.881	0.79	0.8	0.859	0.801	0.719	0.702	0.728
TRINITY_DN35301_c0_g1_i3_orf1	ATP synthase subunit gamma, mitochondrial-like [Ostrinia furnacalis]	10	296	32.5	9.32	32	26	10	High	1	0.994	1.026	0.523	0.487	0.54	0.529	0.479	0.522	0.527	0.472	0.478	0.433	0.429	0.422
TRINITY_DN56308_c0_g1_i2_orf1	storage protein 1 [Omphisa fuscidentalis]	9	98	11.3	8.79	60	68	9	High	1	0.972	1.026	2.565	2.569	2.449	3.13	3.186	3.097	4	4.357	4.258	3.661	3.492	3.891
TRINITY_DN3913_c0_g1_i6_orf1	protein obstructor-E-like [Ostrinia furnacalis]	9	305	33.8	5.4	46	13	9	High	1	1.01	1.068	1.506	1.582	1.477	1.479	1.574	1.47	1.243	1.122	1.464	0.58	0.561	0.588
TRINITY_DN60903_c0_g1_i1_orf1	26S proteasome non-ATPase regulatory subunit 11 [Ostrinia furnacalis]	10	420	47.2	6.67	28	12	10	High	1	0.995	0.983	1.053	1.062	1.006	0.889	0.909	0.907	0.884	0.84	0.855	1.031	0.991	0.936
TRINITY_DN348_c0_g2_i1_orf1	pancreatic triacylglycerol lipase-like [Ostrinia furnacalis]	8	332	36.4	8.7	37	12	1	High	1	1.027	1.131	0.87	0.788	0.706	0.858	0.903	0.726	0.799	0.761	0.828	0.626	0.59	0.667
TRINITY_DN4533_c0_g1_i1_orf1	neurofilament heavy polypeptide-like isoform X2 [Ostrinia furnacalis]	8	722	76.5	9.82	16	11	8	High	1	0.998	1.023	0.757	0.755	0.775	0.743	0.745	0.694	0.622	0.582	0.657	1.012	1.011	0.926
TRINITY_DN12231_c0_g1_i1_orf1	carbonyl reductase [NADPH] 3-like [Ostrinia furnacalis]	10	284	32.2	7.09	39	15	10	High	1	0.995	1.035	2.36	2.165	2.196	2.265	2.126	2.392	2.371	2.114	2.069	2.525	2.576	2.454
TRINITY_DN18681_c0_g1_i7_orf1	fragile X mental retardation syndrome-related protein 1 isoform X3 [Ostrinia fu	10	464	52.4	6.81	24	16	10	High	1	0.996	1.013	0.748	0.739	0.751	0.678	0.646	0.618	0.608	0.627	0.634	0.896	0.888	0.857
TRINITY_DN120500_c0_g1_i1_orf1	cytochrome P450 6B5-like [Ostrinia furnacalis]	10	523	60.5	8.85	23	15	10	High	1	1.002	0.99	1.067	1.078	1.036	1.778	1.788	1.743	0.992	0.943	1.021	0.634	0.616	0.663
TRINITY_DN2302_c0_g2_i1_orf1	ADP-ribosylation factor 2 [Ostrinia furnacalis]	7	180	20.4	8.57	53	16	5	High	1	0.952	0.985	0.987	0.985	0.962	0.944	0.996	1.001	1.012	1.149	1.061	1.06	0.996	1.066
TRINITY_DN1540_c0_g1_i7_orf1	alaserpin-like isoform X13 [Ostrinia furnacalis]	12	385	42.9	4.88	35	16	1	High	1	1.089	1.005	2.638	2.933	2.715	3.112	3.458	3.482	3.712	3.391	3.932	3.26	3.507	3.588
TRINITY_DN31253_c0_g1_i2_orf1	hypothetical protein evm_009655 [Chilo suppressalis]	13	203	23.4	9.61	53	24	13	High	1	0.949	0.988	0.708	0.674	0.69	0.636	0.648	0.663	0.605	0.627	0.578	0.555	0.532	0.544
TRINITY_DN2338_c0_g1_i3_orf1	phenoloxidase subunit 1-like [Ostrinia furnacalis]	10	682	78.5	6.86	18	18	8	High	1	1.019	1.005	1.027	0.997	1.026	0.797	0.861	0.864	1.375	1.367	1.465	0.917	0.929	0.904
TRINITY_DN45000_c0_g1_i5_orf1	PREDICTED: ATP synthase subunit beta, mitochondrial, partial [Papilio polytes]	7	89	9.5	4.92	88	29	1	High	1	1.094	1.116	0.608	0.665	0.694	0.632	0.601	0.58	0.781	0.574	0.676	0.695	0.676	0.61
TRINITY_DN5914_c1_g1_i9_orf1	unnamed protein product [Chilo suppressalis]	9	289	32.7	6.4	43	17	9	High	1	0.971	0.993	1.88	1.914	1.865	2.045	2.083	1.978	1.751	1.626	1.762	1.382	1.43	1.406
TRINITY_DN5215_c0_g1_i1_orf1	glycogen [starch] synthase isoform X2 [Manduca sexta]	11	690	79.1	6.43	20	19	11	High	1	1.028	1.021	1.055	1.071	1.043	1.032	1.007	1.019	1.138	1.158	1.113	0.942	0.909	0.939
TRINITY_DN15545_c0_g1_i1_orf1	larval cuticle protein LCP-14-like [Ostrinia furnacalis]	3	132	14.6	4.61	48	9	3	High	1	0.934	0.939	3.184	3.538	3.313	3.318	3.222	2.965	2.846	2.35	2.858	0.804	0.829	0.785
TRINITY_DN247_c0_g1_i1_orf1	PREDICTED: ADP-ribosylation factor 1 [Dufourea novaeangliae]	7	186	21.1	6.58	36	15	2	High	1	0.879	1.012	0.983	0.97	0.85	0.95	0.973	1.019	1.084	1.134	0.934	1.104	0.995	1.171
TRINITY_DN63914_c0_g1_i1_orf1	myophilin-like [Ostrinia furnacalis]	9	187	20.7	8.22	51	18	9	High	1	1.031	1.013	0.355	0.371	0.388	0.433	0.423	0.399	0.403	0.409	0.443	0.38	0.387	0.388
TRINITY_DN334_c0_g1_i1_orf1	putative chymotrypsin 12 [Ostrinia nubilalis]	8	202	21.6	9.29	53	17	5	High	1	0.971	1.022	0.173	0.171	0.204	0.213	0.225	0.211	0.22	0.212	0.209	0.197	0.188	0.196
TRINITY_DN96080_c0_g2_i1_orf1	ATP synthase subunit delta, mitochondrial [Ostrinia furnacalis]	6	162	17.2	6	53	20	6	High	1	1.034	0.986	0.453	0.46	0.502	0.423	0.408	0.411	0.452	0.42	0.463	0.359	0.36	0.378
TRINITY_DN27751_c0_g2_i1_orf1	eukaryotic translation initiation factor 3 subunit I [Ostrinia furnacalis]	9	329	36.9	5.78	40	15	9	High	1	1.004	0.985	0.663	0.661	0.661	0.565	0.648	0.571	0.547	0.522	0.559	0.635	0.638	0.634
TRINITY_DN9608_c0_g1_i3_orf1	cytochrome P450 monooxygenase CYP9G18 [Cnaphalocrocis medinalis]	9	503	58.5	7.9	17	14	7	High	1	0.942	0.976	2.453	2.292	2.375	5.455	4.954	5.284	3.184	3.282	3.177	1.765	1.632	1.713
TRINITY_DN2198_c0_g1_i1_orf1	unnamed protein product [Chilo suppressalis]	12	2384	264	6.77	6	12	12	High	1	1.007	1.048	1.031	1.008	1.032	0.981	1.108	1.1	0.949	0.928	0.941	1.102	1.076	1.067
TRINITY_DN28516_c0_g1_i2_orf1	unnamed protein product [Chrysodeixis includens]	11	293	31.6	9.82	33	16	11	High	1	1.032	0.971	0.952	0.896	0.907	1.097	1.065	1.108	1.095	1.309	1.025	0.88	0.919	0.97
TRINITY_DN44083_c0_g1_i2_orf1	putative alpha-ketoglutarate-dependent hypophosphite dioxygenase [Operop	9	264	29.8	6.4	38	21	8	High	1	1.04	1.03	1.682	1.633	1.638	1.738	1.641	1.655	1.563	1.398	1.549	1.139	1.126	1.145
TRINITY_DN15959_c0_g1_i1_orf1	dnaJ homolog subfamily A member 2-like [Ostrinia furnacalis]	10	404	44.9	6.64	33	15	10	High	1	0.997	0.98	0.47	0.505	0.497	0.436	0.475	0.472	0.434	0.446	0.465	0.544	0.547	0.554
TRINITY_DN35666_c0_g1_i1_orf1	phenylalanine--tRNA ligase alpha subunit [Ostrinia furnacalis]	10	491	55.8	6.28	25	15	10	High	1	1.045	1.056	1.078	1.083	1.094	1.071	1.009	1.062	1.012	1.013	0.983	0.948	0.93	0.931
TRINITY_DN380_c0_g2_i2_orf1	chemosensory protein 10 [Ostrinia furnacalis]	8	120	13.6	5.17	54	22	3	High	1	1.005	1.001	3.952	3.971	4.007	4.518	4.732	4.556	5.487	4.745	5.689	2.252	2.512	2.469
TRINITY_DN14996_c0_g1_i2_orf1	40S ribosomal protein S17 [Ostrinia furnacalis]	7	133	15.4	9.7	61	14	7	High	1	0.98	0.997	0.706	0.625	0.711	0.657	0.653	0.7	0.624	0.598	0.542	0.538	0.55	0.531
TRINITY_DN975_c0_g1_i1_orf1	elongation factor 1-alpha 1 [Myotis lucifugus]	9	462	50.1	9.01	20	42	2	High	1	1.251	1.237	1.3	1.867	1.667	2.614	2.424	2.19	2.052	2.005	3.335	1.404	1.411	1.345
TRINITY_DN2627_c0_g2_i1_orf1	phosphotriesterase-related protein [Ostrinia furnacalis]	11	353	39.4	5.47	36	12	11	High	1	1.009	1.002	1.015	1.047	0.997	1.31	1.281	1.33	1.171	1.068	1.214	1.411	1.359	1.265
TRINITY_DN2855_c0_g1_i6_orf1	uncharacterized protein LOC114359301 [Ostrinia furnacalis]	14	424	48.5	8.4	36	17	14	High	1	0.972	0.965	1.223	1.162	1.203	1.141	1.072	1.18	0.969	0.928	0.973	1.204	1.165	1.217
TRINITY_DN33_c0_g1_i8_orf1	uncharacterized protein CG45076-like isoform X2 [Ostrinia furnacalis]	8	226	25.5	9.88	50	14	1	High	1	0.745	1.085	0.525	0.635	0.489	0.473	0.577	0.451	0.596	0.494	0.729	0.596	1.206	0.407
TRINITY_DN3354_c0_g1_i1_orf1	histidine triad nucleotide-binding protein 1-like [Ostrinia furnacalis]	6	128	14	6.64	66	12	6	High	1	1.012	1.018	1.077	1.105	1.015	1.085	1.083	1.077	1.126	1.022	1.091	1.167	1.153	1.089
TRINITY_DN20582_c0_g1_i1_orf1	2-oxoglutarate dehydrogenase, mitochondrial isoform X3 [Ostrinia furnacalis]	8	228	25.6	9.36	32	14	7	High	1	1.056	1.024	0.474	0.462	0.481	0.411	0.423	0.408	0.443	0.429	0.42	0.441	0.469	0.463
TRINITY_DN15362_c0_g1_i1_orf1	probable elongation factor 1-delta isoform X1 [Ostrinia furnacalis]	8	264	29	4.87	33	21	8	High	1	1.006	1.01	0.899	0.885	0.928	0.757	0.754	0.746	0.688	0.648	0.707	0.65	0.667	0.652
TRINITY_DN7040_c0_g2_i1_orf1	uncharacterized protein LOC114353763 [Ostrinia furnacalis]	8	195	22	4.74	32	22	8	High	1	1.003	0.986	3.305	3.078	3.278	3.77	3.742	3.945	4.352	4.548	3.96	4.225	4.268	4.487
TRINITY_DN717_c0_g1_i2_orfp1	TRINITY_DN717_c0_g1_i2_m.67915 TRINITY_DN717_c0_g1::TRINITY_DN717_c0	11	867	90.1	9.64	20	12	8	High	1	1.052	0.975	0.121	0.112	0.161	0.174	0.147	0.167	0.185	0.146	0.197	0.213	0.229	0.238
TRINITY_DN554_c0_g1_i1_orf1	uncharacterized protein LOC114353093 isoform X1 [Ostrinia furnacalis]	10	532	58.7	5.36	25	14	10	High	1	0.984	1.016	1.193	1.189	1.229	1.515	1.492	1.386	1.251	1.406	1.28	0.985	0.959	0.952
TRINITY_DN5118_c0_g1_i1_orf1	AP-2 complex subunit alpha [Ostrinia furnacalis]	13	929	104	7.3	16	16	13	High	1	1.017	1.036	1.084	1.004	1.078	0.952	0.956	0.98	0.892	0.908	0.831	1.245	1.211	1.209
TRINITY_DN4766_c0_g1_i4_orf1	26S proteasome non-ATPase regulatory subunit 12 [Ostrinia furnacalis]	12	450	51.5	6.7	26	19	12	High	1	1.023	1.092	1.041	1.001	0.964	0.865	0.899	0.916	0.862	0.853	0.829	1.044	1.028	1.056
TRINITY_DN2097_c1_g1_i1_orf1	5-oxoprolinase [Ostrinia furnacalis]	11	1291	138.6	6.62	12	12	11	High	1	1.009	1.02	0.948	0.989	0.955	1.184	1.195	1.225	1.209	1.205	1.203	1.525	1.336	1.368
TRINITY_DN467_c3_g1_i5_orf1	lysozyme precursor [Loxostege sticticalis]	8	141	16	8.78	52	22	7	High	1	0.994	0.941	3.077	3.2	3.032	5.068	4.933	4.593	3.945	3.391	4.102	4.795	4.801	4.562

TRINITY_DN7573_c0_g2_i1_orf1	nucleolar protein 56 [Ostrinia furnacalis]	9	458	51	8.9	23	11	9	High	1	0.997	1.003	0.759	0.753	0.749	0.69	0.658	0.711	0.715	0.694	0.684	0.693	0.681	0.707
TRINITY_DN9164_c0_g1_i3_orf1	unnamed protein product [Parnassius apollo]	11	507	58.6	8.69	19	14	11	High	1	0.971	0.965	0.658	0.636	0.668	0.569	0.572	0.594	0.541	0.572	0.472	0.698	0.664	0.732
TRINITY_DN4817_c0_g1_i4_orf1	palmitoyl-protein thioesterase 1 isoform X1 [Ostrinia furnacalis]	8	300	34.6	8.19	30	18	8	High	1	1.02	0.996	1.546	1.572	1.594	1.28	1.355	1.243	1.037	1.037	1.114	1.543	1.523	1.519
TRINITY_DN10264_c1_g1_i5_orf1	probable low-specificity L-threonine aldolase 2 [Ostrinia furnacalis]	8	373	40.4	7.31	29	9	8	High	1	1.109	1.099	1.389	1.178	1.316	1.501	1.297	1.441	1.461	1.659	1.419	1.547	1.522	1.48
TRINITY_DN11259_c0_g1_i1_orf1	uncharacterized protein LOC114357075 [Ostrinia furnacalis]	10	254	26.7	8.09	46	14	6	High	1	0.962	0.961	0.197	0.187	0.24	0.258	0.294	0.28	0.263	0.268	0.236	0.2	0.21	0.217
TRINITY_DN334_c0_g1_i4_orf1	collagenase-like [Ostrinia furnacalis]	7	283	29.8	9.19	39	11	1	High	1	1.068	1.047	0.092	0.072	0.124	0.101	0.182	0.151	0.102	0.089	0.092	0.042	0.07	0.043
TRINITY_DN364_c5_g1_i3_orf1	talin-2-like, partial [Ostrinia furnacalis]	12	979	109.3	6.07	13	13	11	High	1	0.942	0.957	1.404	1.357	1.393	1.326	1.464	1.298	1.265	1.211	1.273	1.475	1.506	1.479
TRINITY_DN1509_c0_g1_i1_orf1	ribosomal protein S15A [Bombyx mori]	9	129	14.7	9.99	64	19	9	High	1	1.021	0.983	0.677	0.616	0.681	0.647	0.628	0.642	0.59	0.551	0.536	0.532	0.523	0.539
TRINITY_DN4343_c0_g1_i2_orf1	uncharacterized protein LOC114365231 isoform X3 [Ostrinia furnacalis]	9	387	43.4	6.32	28	11	9	High	1	0.959	1.088	2.282	2.383	2.27	2.434	2.478	2.48	1.82	1.758	1.915	1.815	1.749	1.725
TRINITY_DN26130_c0_g1_i1_orf1	membrane alanyl aminopeptidase-like [Ostrinia furnacalis]	9	928	104.2	5.38	13	13	9	High	1	1.038	1.01	0.288	0.27	0.308	0.329	0.329	0.323	0.382	0.397	0.366	0.46	0.43	0.479
TRINITY_DN100208_c0_g1_i1_orf1	neurofilament heavy polypeptide-like isoform X2 [Ostrinia furnacalis]	7	81	8.4	4.5	88	10	1	High	1	1.002	1.032	1.439	1.435	1.37	0.461	0.517	0.509	1.144	0.913	1.289	1.776	1.548	1.824
TRINITY_DN30950_c0_g1_i13_orf1	unnamed protein product [Chilo suppressalis]	12	792	90.5	5.96	15	15	12	High	1	1.018	1.058	0.783	0.736	0.83	0.631	0.653	0.712	0.642	0.633	0.639	0.845	0.806	0.831
TRINITY_DN22166_c0_g1_i3_orf1	hypothetical protein evm_010872 [Chilo suppressalis]	10	598	68.1	8.07	22	15	10	High	1	0.959	0.95	1.08	1.026	1.078	1.063	1.054	1.148	1.054	0.941	0.956	0.999	0.993	1.028
TRINITY_DN2823_c0_g1_i6_orf1	proteasome-associated protein ECM29 homolog [Ostrinia furnacalis]	14	1896	211.4	6.9	9	15	14	High	1	0.994	1.03	1.227	1.224	1.251	1.106	1.195	1.141	0.999	1.13	0.986	1.075	1.038	1.066
TRINITY_DN4010_c0_g2_i1_orf1	myophilin [Ostrinia furnacalis]	7	169	19.2	8	47	22	7	High	1	1.039	1.023	1.347	1.348	1.353	1.071	1.079	1.041	0.943	0.907	0.99	0.481	0.478	0.48
TRINITY_DN31609_c0_g1_i3_orf1	sorbitol dehydrogenase-like [Ostrinia furnacalis]	8	351	37	7.36	34	13	8	High	1	1.009	1.016	1.293	1.273	1.235	1.728	1.77	1.73	1.416	1.294	1.358	1.308	1.338	1.276
TRINITY_DN5087_c0_g1_i6_orf1	nascent polypeptide-associated complex subunit alpha [Ostrinia furnacalis]	7	214	22.8	4.89	34	18	7	High	1	0.976	1.023	0.45	0.417	0.5	0.415	0.407	0.427	0.453	0.407	0.41	0.534	0.541	0.513
TRINITY_DN38506_c0_g1_i4_orf1	C-1-tetrahydrofolate synthase, cytoplasmic isoform X1 [Ostrinia furnacalis]	8	152	16.2	5.53	61	11	3	High	1	0.864	0.944	4.646	4.808	4.333	4.685	4.236	4.125	4.298	4.446	5.202	1.12	1.376	1.665
TRINITY_DN3411_c0_g1_i2_orf1	unnamed protein product [Chilo suppressalis]	13	1345	152.1	7.08	12	13	13	High	1	0.989	0.999	0.782	0.823	0.798	0.787	0.816	0.749	0.729	0.783	0.802	0.853	0.805	0.879
TRINITY_DN6308_c0_g1_i3_orf1	myc box-dependent-interacting protein 1 isoform X4 [Pectinophora gossypiel	8	353	40.3	6.64	35	11	1	High	1	0.951	1.029	0.745	0.826	0.826	0.769	0.83	0.801	0.756	0.763	0.742	0.725	0.629	0.75
TRINITY_DN4065_c0_g1_i5_orf1	uncharacterized protein LOC114357176 [Ostrinia furnacalis]	8	649	71	7.06	20	13	8	High	1	0.965	0.967	1.167	1.26	1.148	1.119	1.125	1.128	1.105	1.192	1.218	1.17	1.241	1.181
TRINITY_DN3831_c0_g1_i7_orf1	beta-ureidopropionase-like [Ostrinia furnacalis]	7	389	43.6	6.95	25	9	7	High	1	0.984	0.997	1.638	1.608	1.507	1.87	1.647	1.797	1.738	1.576	1.826	1.39	1.464	1.422
TRINITY_DN114834_c0_g1_i1_orf1	uncharacterized protein LOC115444227 [Manduca sexta]	10	749	81.4	4.97	19	11	10	High	1	1.003	1.021	0.836	0.825	0.802	0.87	0.908	0.882	0.872	0.859	0.877	2.519	2.62	2.458
TRINITY_DN6991_c0_g1_i24_orf1	muscle M-line assembly protein unc-89 isoform X5 [Ostrinia furnacalis]	11	748	83.1	4.61	16	14	11	High	1	0.995	1.041	1.019	1.101	1.108	1.029	1.025	1.035	1.061	1.05	1.178	0.841	0.843	0.818
TRINITY_DN374_c0_g1_i4_orf1	obg-like ATPase 1 [Helicoverpa zea]	10	397	44.5	7.5	32	12	10	High	1	1.009	0.951	0.877	0.89	0.899	0.821	0.854	0.823	0.826	0.851	0.865	0.782	0.818	0.772
TRINITY_DN10747_c0_g1_i5_orf1	unnamed protein product [Plutella xylostella]	11	365	41.8	6.67	36	15	10	High	1	0.96	1.028	1.056	1.046	1.139	1.14	1.14	1.211	1.241	1.257	1.109	4.781	4.915	4.517
TRINITY_DN110231_c0_g1_i1_orf1	protein singed [Ostrinia furnacalis]	11	514	57	6.84	29	14	11	High	1	1.021	1.007	1.753	1.74	1.699	1.279	1.253	1.319	1.359	1.208	1.286	3.206	3.135	3.142
TRINITY_DN12474_c0_g1_i6_orf1	aromatic-L-amino-acid decarboxylase [Ostrinia furnacalis]	10	478	54.3	6.23	20	14	10	High	1	1.018	1.023	0.319	0.273	0.332	0.301	0.301	0.312	0.317	0.314	0.276	0.288	0.295	0.317
TRINITY_DN17738_c0_g1_i2_orf1	unnamed protein product [Diatraea saccharalis]	10	375	43.9	6.89	28	17	10	High	1	1.011	0.959	0.424	0.407	0.455	0.396	0.392	0.396	0.374	0.355	0.362	0.423	0.405	0.399
TRINITY_DN1445_c0_g1_i1_orf1	leucine-rich PPR motif-containing protein, mitochondrial [Ostrinia furnacalis]	9	780	87.6	6.13	14	11	9	High	1	1.017	1.015	0.355	0.391	0.419	0.36	0.384	0.384	0.381	0.377	0.375	0.473	0.449	0.49
TRINITY_DN16673_c0_g1_i1_orf1	myosin heavy chain, partial [Drosophila virilis]	5	67	7.8	9.48	70	25	4	High	1	1.039	1.037	0.819	0.888	0.819	0.763	0.8	0.778	0.705	0.763	0.766	0.552	0.559	0.547
TRINITY_DN31584_c0_g2_i2_orf1	14-3-3 protein epsilon [Gallus gallus]	9	255	29.2	4.74	35	29	1	High	1	0.926	1.031	0.569	0.618	0.624	0.797	0.852	0.859	1.176	1.063	1.265	1.029	1.021	0.963
TRINITY_DN141_c0_g1_i1_orf1	hypothetical protein evm_010402 [Chilo suppressalis]	8	1003	113.8	5.12	11	9	8	High	1	1.025	1.073	0.723	0.711	0.697	0.627	0.593	0.585	0.626	0.557	0.631	0.795	0.741	0.826
TRINITY_DN28661_c0_g1_i1_orf1	cathepsin B [Ostrinia furnacalis]	10	335	37.1	6.29	38	25	10	High	1	0.983	0.986	1.675	1.773	1.709	1.498	1.527	1.573	1.491	1.313	1.51	1.992	2.021	1.963
TRINITY_DN5661_c0_g1_i5_orf1	cytochrome P450 6B7-like [Ostrinia furnacalis]	10	523	60.2	7.96	29	13	10	High	1	0.963	0.965	1.771	1.706	1.764	1.535	1.633	1.589	1.215	1.128	1.241	0.777	0.758	0.866
TRINITY_DN1123_c2_g1_i4_orf1	troponin I isoform X16 [Ostrinia furnacalis]	15	205	24.4	9.77	46	16	8	High	1	1.026	0.889	0.904	0.998	0.948	0.998	0.944	0.916	0.849	0.797	0.904	0.307	0.297	0.34
TRINITY_DN82801_c0_g1_i1_orf1	uncharacterized protein LOC114364712 [Ostrinia furnacalis]	11	539	61.3	5.25	27	16	10	High	1	1.028	0.972	0.167	0.166	0.197	0.216	0.237	0.215	0.214	0.232	0.222	0.695	0.704	0.666
TRINITY_DN2722_c0_g1_i1_orf1	troponin C [Pieris rapae]	9	159	18.1	4.2	58	26	9	High	1	1.033	0.994	1.59	1.661	1.648	1.657	1.624	1.572	1.488	1.446	1.593	0.664	0.666	0.665
TRINITY_DN145227_c0_g1_i1_orf1	26S protease regulatory subunit 7, partial [Cotesia chilonis]	9	436	48.8	6.27	25	16	8	High	1	1.009	1.005	0.948	0.942	0.933	0.844	0.814	0.826	0.871	0.791	0.749	0.939	0.938	0.966
TRINITY_DN6143_c0_g2_i1_orf1	uncharacterized protein LOC114365036 [Ostrinia furnacalis]	8	253	28.8	8.07	41	12	8	High	1	1.009	0.993	0.509	0.49	0.507	0.462	0.475	0.434	0.422	0.408	0.47	0.253	0.3	0.267
TRINITY_DN32997_c0_g1_i8_orf1	RNA-binding protein squid isoform X1 [Ostrinia furnacalis]	8	282	30	9.28	21	12	8	High	1	1.032	1.044	0.473	0.51	0.504	0.439	0.435	0.426	0.433	0.423	0.43	0.592	0.552	0.582
TRINITY_DN19628_c1_g1_i1_orf1	transcription factor BTF3 homolog 4-like [Ostrinia furnacalis]	7	174	19	8.68	50	12	7	High	1	1.089	1.099	0.597	0.623	0.596	0.565	0.533	0.559	0.529	0.515	0.607	0.656	0.635	0.599
TRINITY_DN8716_c0_g1_i3_orf1	aspartate--tRNA ligase, cytoplasmic isoform X1 [Ostrinia furnacalis]	9	534	59.7	7.21	19	13	9	High	1	1.035	1.038	1.29	1.163	1.23	1.294	1.231	1.384	1.18	1.044	0.994	0.999	1.09	1.025
TRINITY_DN9694_c0_g1_i1_orf1	larval cuticle protein LCP-17 [Helicoverpa armigera]	5	152	15.7	4.92	82	26	5	High	1	0.979	0.999	1.89	1.927	1.907	2.118	2.115	2.045	1.922	1.802	1.932	0.78	0.735	0.77
TRINITY_DN987_c0_g1_i3_orf1	unnamed protein product [Chilo suppressalis]	9	196	21.6	7.66	49	17	3	High	1	0.984	0.954	1.109	1.195	1.079	2.065	2.159	2.02	1.904	1.843	2.085	1.323	1.444	1.353
TRINITY_DN15882_c0_g1_i1_orf1	succinate--CoA ligase [ADP-forming] subunit beta, mitochondrial [Ostrinia fur	9	478	51.8	8.65	22	13	9	High	1	1.012	0.987	0.52	0.502	0.555	0.533	0.509	0.534	0.514	0.484	0.51	0.494	0.482	0.469
TRINITY_DN5488_c0_g1_i5_orf1	alpha-amylase-like [Ostrinia furnacalis]	10	500	56.8	6.8	26	12	10	High	1	0.979	0.986	1.832	1.853	1.836	1.623	1.64	1.678	1.888	1.881	1.823	1.874	1.923	1.868
TRINITY_DN257_c0_g1_i7_orf1	zinc finger RNA-binding protein 2 [Ostrinia furnacalis]	9	740	78.7	9.26	14	15	9	High	1	1.014	1.022	0.699	0.725	0.727	0.627	0.645	0.625	0.567	0.53	0.589	0.878	0.864	0.828
TRINITY_DN80328_c0_g1_i9_orf1	arylphorin subunit alpha-like [Ostrinia furnacalis]	7	75	9.1	6.54	84	65	4	High	1	0.969	0.976	2.394	2.135	2.197	2.611	2.522	2.77	3.768	3.626	3.113	3.018	3.062	2.984
TRINITY_DN932_c0_g1_i4_orf1	SXSS-APN2 [Ostrinia furnacalis]	11	941	106.5	5.11	15	13	11	High	1	0.98	0.995	0.565	0.522	0.527	0.59	0.571	0.603	0.561	0.582	0.579	0.549	0.542	0.535
TRINITY_DN18249_c0_g1_i1_orf1	60S ribosomal protein L13 [Pectinophora gossypiella]	11	221	25.1	11.36	48	24	11	High	1	0.963	0.932	0.801	0.929	0.83	0.728	0.749	0.727	0.631	0.573	0.697	0.686	0.675	0.705
TRINITY_DN35694_c0_g1_i1_orf1	glucosidase 2 subunit beta [Ostrinia furnacalis]	8	523	59.4	4.55	17	13	8	High	1	1.035	0.984	0.951	0.98	0.984	1.075	1.079	1.1	1.016	0.886	1.046	1.066	1.083	0.986
TRINITY_DN714_c0_g1_i3_orf1	thymosin beta isoform X4 [Ostrinia furnacalis]	11	170	18.9	5.15	62	25	9	High	1	0.947	1.035	1.056	1.093	1.051	1.172	1.202	1.095	1.008	0.953	1.052	1.939	1.959	1.965
TRINITY_DN36701_c0_g1_i4_orf1	hypothetical protein SFRURICE_004895 [Spodoptera frugiperda]	11	143	16	10.56	62	18	11	High	1	1.037	0.956	0.646	0.712	0.681	0.626	0.613	0.635	0.57	0.566	0.623	0.609	0.612	0.578
TRINITY_DN58207_c0_g1_i1_orf1	60S ribosomal protein L6 [Ostrinia furnacalis]	10	250	28.2	10.99	32	24	9	High	1	1.016	0.95	0.582	0.591	0.611	0.519	0.511	0.525	0.426	0.417	0.46	0.417	0.428	0.404
TRINITY_DN41952_c0_g1_i1_orf1	protein DDI1 homolog 2 [Ostrinia furnacalis]	8	389	43.5	5.38	32	11	2	High	1	1.108	1.095	1.714	1.797	1.797	1.616	1.659	1.555	1.579	1.769	1.778	1.666	1.424	1.495
TRINITY_DN8949_c0_g1_i2_orf1	unnamed protein product [Arctia plantaginis]	7	208	24	10.3	29	20	7	High	1	1.029	0.961	0.631	0.638	0.662	0.583	0.595	0.58	0.451	0.42	0.519	0.536	0.531	0.517
TRINITY_DN12387_c1_g2_i1_orf1	skin secretory protein xP2-like [Ostrinia furnacalis]	13	357	39.7	9.28	50	22	10	High	1	0.954	0.972	0.882	0.946	0.894	0.894	1.007	0.983	0.941	1.092	0.953	4.353	4.602	4.071
TRINITY_DN42542_c0_g1_i1_orf1	sorting nexin lst-4 [Ostrinia furnacalis]	11	557	63.2	5.95	24	13	11	High	1	1.025	1.027	0.849	0.805	0.832	0.867	0.89	0.906	0.911	0.945	0.822	0.903	0.873	0.894
TRINITY_DN5384_c0_g1_i21_orf1	hexokinase type 2 isoform X1 [Ostrinia furnacalis]	10	453	50.1	6.32	25	13	10	High	1	0.963	0.996	0.895	0.876	0.937	0.958	0.927	0.949	0.891	0.93	0.83	0.924	0.875	0.888
TRINITY_DN50517_c0_g1_i5_orf1	hypothetical protein evm_004957 [Chilo suppressalis]	9	208	21.7	7.68	51	15	6	High	1	1.062	1.042	0.865	0.886	0.921	0.997	0.879	0.932	0.895	0.754	0.997	1.325	1.392	1.22
TRINITY_DN31286_c0_g1_i6_orfp1	TRINITY_DN31286_c0_g1_i6_m.28438 TRINITY_DN31286_c0_g1::TRINITY_DN31	5	91	9.4	4.83	71	14	5	High	1	1.037	1.038	2.589	2.738	2.666	3.504	3.355	3.271	2.554	2.375	2.742	1.942	2.084	1.964
TRINITY_DN16077_c0_g1_i13_orf1	dynamin-like 120 kDa protein, mitochondrial [Ostrinia furnacalis]	11	951	108.6	8.68	14	11	10	High	1	0.961	0.992	0.613	0.597	0.595	0.57	0.626	0.56	0.523	0.767	0.583	0.605	0.621	0.603
TRINITY_DN7128_c0_g1_i7_orf1	dystroglycan [Ostrinia furnacalis]	10	1119	124	5.94	11	12	10	High	1	0.994	0.992	1.528	1.609	1.556	1.48	1.457	1.358	1.331	1.293	1.485	1.99	2.052	1.915
TRINITY_DN10476_c0_g1_i1_orf1	prohibitin-2 [Ostrinia furnacalis]	10	299	32.9	9.63	36	18	10	High	1	1.023	1.046	0.539	0.532	0.553	0.508	0.49	0.531	0.481	0.447	0.475	0.489	0.482	0.498
TRINITY_DN19116_c0_g1_i3_orf1	UDP-glucose 4-epimerase isoform X1 [Ostrinia furnacalis]	10	365	40.6	7.3	29	12	10	High	1	0.943	1.034	1.603	1.716	1.572	1.558	1.564	1.578	1.271	1.338	1.342	1.18	1.17	1.24
TRINITY_DN9189_c0_g1_i1_orf1	unnamed protein product, partial [Iphiclides podalirius]	12	454	51	4.82	30	19	5	High	1	1.077	1.007	1.087	1.021	1.067	0.877	0.911	0.932	0.948	0.821	0.873	0.879	0.928	0.905
TRINITY_DN10192_c0_g1_i6_orf1	prolyl endopeptidase isoform X2 [Ostrinia furnacalis]	13	741	83.6	6.07	19	21	13	High	1	0.984	0.98	0.926	0.959	0.949	1.131	1.093	1.043	0.988	0.991	0.89	0.939	0.888	0.888
TRINITY_DN467_c0_g3_i1_orf1	histone-lysine N-methyltransferase 2B-like, partial [Ostrinia furnacalis]	7	523	58.3	6.29	19	8	7	High	1	0.957	0.986	0.768	0.798	0.656	0.727	0.828	0.657	0.635	0.618	0.753	2.413	2.42	2.329
TRINITY_DN4245_c0_g1_i5_orf1	long-chain fatty acid transport protein 4-like [Ostrinia furnacalis]	8	549	60.5	7.12	20	12	7	High	1	0.957	0.985	1.776	1.823	1.769	1.615	1.739	1.676	1.647	1.529	1.701	1.163	1.129	1.174
TRINITY_DN10429_c0_g1_i2_orf1	lon protease homolog, mitochondrial isoform X1 [Ostrinia furnacalis]	12	715	81.5	7.31	17	12	12	High	1	0.98	0.992	0.604	0.605	0.61	0.563	0.566	0.603	0.569	0.556	0.567	0.559	0.539	0.542
TRINITY_DN14328_c0_g1_i12_orf1	larval cuticle protein LCP-30-like [Ostrinia furnacalis]	10	212	24.6	5.02	50	13	10	High	1	1.012	1.086	3.454	3.75	3.555	4.501	4.549	4.098	3.424	2.886	3.723	1.048	0.991	1.11
TRINITY_DN18637_c0_g1_i4_orf1	leucine-rich repeat-containing protein 47-like isoform X1 [Ostrinia furnacalis]	12	531	59.1	8.15	25	15	12	High	1	0.977	0.999	0.936	0.957	0.984	0.94	0.929	0.871	0.903	0.872	0.904	0.91	0.898	0.873
TRINITY_DN334_c0_g1_i3_orf1	chymotrypsin-like serine protease [Ostrinia nubilalis]	6	284	29.6	8.72	35	10	1	High	1	1.002	0.977	0.371	0.391	0.505	0.446	0.394	0.375	0.4	0.406	0.451	0.646	0.634	0.621
TRINITY_DN2267_c0_g1_i1_orf1	hypothetical protein evm_006312 [Chilo suppressalis]	9	378	41.3	8.98	19	25	9	High	1	0.983	0.969	0.323	0.261	0.345	0.275	0.257	0.296	0.315	0.309	0.265	0.281	0.279	0.284
TRINITY_DN91533_c0_g1_i1_orf1	uncharacterized protein LOC114361550 [Ostrinia furnacalis]	10	704	78.9	5.87	18	13	10	High	1	0.96	0.996	1.058	1	1.026	0.995	0.991	1.015	1.015	0.925	0.963	4.006	4.076	3.901
TRINITY_DN31611_c0_g1_i2_orf1	glucose-6-phosphate isomerase-like [Ostrinia furnacalis]	5	133	15	6.58	38	13	5	High	1	1.011	0.992	1.663	1.676	1.58	1.573	1.615	1.587	1.898	1.763	2.003	1.576	1.578	1.668
TRINITY_DN14274_c0_g1_i3_orf1	ATP-dependent RNA helicase dbp2-like isoform X1 [Leguminivora glycinivore	10	770	84.6	10.74	17	13	8	High	1	1.002	0.975	0.864	0.918	0.921	1.001	0.957	0.983	1.154	1.17	1.227	1.358	1.324	1.262
TRINITY_DN10174_c0_g1_i4_orf1	protein D2-like isoform X2 [Aricia agestis]	6	190	20.7	6.18	48	17	2	High	1	0.958	1.002	0.699	0.638	0.748	0.787	0.703	0.796	0.871	0.912	0.707	1.057	1.039	1.067
TRINITY_DN14587_c0_g1_i7_orf1	hypothetical protein evm_001488 [Chilo suppressalis]	9	256	29	6.79	43	11	9	High	1	1.035	0.996	1.211	1.229	1.292	1.413	1.428	1.424	1.426	1.272	1.397	1.458	1.576	1.467
TRINITY_DN2172_c0_g2_i5_orf1	4-hydroxyphenylpyruvate dioxygenase [Ostrinia furnacalis]	9	380	43.6	5.74	26	16	9	High	1	0.992	1.018	0.56	0.513	0.558	0.648	0.603	0.648	0.517	0.512	0.47	0.359	0.357	0.37
TRINITY_DN7808_c0_g1_i1_orf1	probable pyruvate dehydrogenase E1 component subunit alpha, mitochondria	10	401	43.9	8.1	25	13	10	High	1	1.006	0.975	0.654	0.639	0.707	0.701	0.721	0.695	0.686	0.649	0.723	0.504	0.496	0.52
TRINITY_DN7776_c0_g1_i9_orf1	uncharacterized protein LOC114364702 [Ostrinia furnacalis]	9	245	27.1	5.68	41	15	8	High	1	1.023	1.038	1.39	1.374	1.339	1.306	1.236	1.301	1.315	1.246	1.329	1.509	1.43	1.402
TRINITY_DN11799_c0_g1_i4_orf1	V-type proton ATPase 116 kDa subunit a1 isoform X1 [Manduca sexta]	9	851	97.5	6.62	12	16	8	High	1	1.027	1.022	0.431	0.442	0.469	0.418	0.445	0.473	0.497	0.493	0.495	0.567	0.541	0.539
TRINITY_DN1093_c0_g1_i1_orf1	uncharacterized protein LOC114361723 isoform X1 [Ostrinia furnacalis]	9	608	69	6.28	19	12	1	High	1	0.708	0.851	1.119	0.985	1.156	0.879	1.384	1.31	1.031	1.099	1.084	0.888	0.964	0.974
TRINITY_DN1592_c0_g1_i1_orf1	serine protease 7-like isoform X2 [Ostrinia furnacalis]	9	379	41.6	6.18	29	12	9	High	1	1.021	1.037	2.076	2.203	2.054	1.953	2.079	1.906	1.611	1.558	1.752	1.565	1.578	1.598
TRINITY_DN50787_c0_g1_i1_orf1	proteasome subunit alpha type-4 [Ostrinia furnacalis]	8	257	28.6	6.6	32	14	8	High	1	1.001	1.027	1.197	1.167	1.146	1.055	1.063	1.124	1.138	1.097	1.094	1.175	1.133	1.201
TRINITY_DN5982_c0_g1_i3_orf1	coatomer subunit gamma [Ostrinia furnacalis]	11	864	95.2	5.68	14	15	10	High	1	1.042	1.019	0.748	0.773	0.765	0.776	0.744	0.768	0.698	0.72	0.706	0.863	0.89	0.873
TRINITY_DN7626_c0_g1_i1_orf1	NADH dehydrogenase [ubiquinone] flavoprotein 1, mitochondrial isoform X1 [	9	462	50.5	8	24	17	9	High	1	1.018	0.977	0.454	0.481	0.509	0.44	0.438	0.442	0.429	0.436	0.462	0.451	0.454	0.446
TRINITY_DN38720_c0_g1_i3_orf1	transmembrane protein 214 [Ostrinia furnacalis]	13	664	74.8	9.17	23	17	13	High	1	1.08	1.067	1.083	1.014	1.061	0.943	0.914	0.932	0.858	0.819	0.891	0.992	0.921	0.929
TRINITY_DN33146_c0_g1_i1_orf1	adenylate kinase-like [Ostrinia furnacalis]	11	242	27	8.22	42	21	11	High	1	1.036	0.977	0.814	0.863	0.821	0.961	0.965	0.885	0.965	0.904	1.076	0.821	0.811	0.771
TRINITY_DN2062_c0_g1_i9_orf1	uncharacterized protein LOC114350846 [Ostrinia furnacalis]	11	247	25.6	6.79	61	12	6	High	1	0.99	0.98	0.47	0.485	0.482	0.473	0.497	0.483	0.35	0.388	0.361	0.564	0.594	0.577
TRINITY_DN3190_c0_g1_i1_orf1	repetitive proline-rich cell wall protein 1 precursor [Papilio polytes]	18	392	44	9.01	44	22	17	High	1	0.988	1.047	0.979	0.978	0.964	0.95	1.024	1.002	0.999	0.878	0.824	10.242	10.661	9.866
TRINITY_DN2946_c0_g1_i1_orf1	histidine-rich glycoprotein [Ostrinia furnacalis]	9	208	23.4	6.43	28	20	9	High	1	1.051	0.987	0.777	0.803	0.821	0.874	0.912	0.982	0.825	0.742	0.787	6.303	6.359	5.662

TRINITY_DN3476_c0_g1_i5_orf1	maltase A1-like [Ostrinia furnacalis]	9	577	66.3	4.79	22	13	8	High	1	1.053	1.041	0.274	0.262	0.327	0.315	0.308	0.325	0.337	0.356	0.318	0.254	0.249	0.258
TRINITY_DN2813_c0_g1_i3_orf1	arylphorin subunit alpha-like [Ostrinia furnacalis]	13	471	55.6	6.25	27	14	2	High	1	1.014	0.912	1.142	1.037	1.09	0.851	0.722	0.797	0.823	0.939	0.81	0.791	0.894	0.72
TRINITY_DN3321_c0_g1_i3_orf1	peroxidasin [Ostrinia furnacalis]	12	1368	154.8	6.28	10	12	12	High	1	1.012	1.018	0.77	0.775	0.774	0.657	0.734	0.682	0.63	0.604	0.631	0.788	0.787	0.827
TRINITY_DN5893_c0_g1_i7_orf1	jupiter microtubule associated homolog 1-like [Ostrinia furnacalis]	8	146	15.1	9.09	62	11	8	High	1	1.058	1.015	0.957	0.965	0.914	0.897	0.93	0.909	0.946	0.795	1.073	1.817	1.92	1.793
TRINITY_DN8724_c0_g1_i2_orf1	vesicle-associated membrane protein/synaptobrevin-binding protein isoform	10	184	20.7	8.32	68	14	1	High	1	0.996	0.955	1.507	1.639	1.552	1.473	1.311	1.335	1.352	1.483	1.402	1.682	1.512	1.768
TRINITY_DN1421_c0_g1_i1_orf1	uncharacterized protein LOC114352615 [Ostrinia furnacalis]	7	405	44.4	6.35	22	11	7	High	1	0.987	1.016	2.635	2.604	2.514	2.448	2.35	2.471	2.338	2.099	2.458	2.347	2.393	2.346
TRINITY_DN26254_c0_g1_i1_orf1	hypothetical protein evm_003664 [Chilo suppressalis]	3	137	14.7	4.88	36	10	3	High	1	1.034	0.972	1.566	1.577	1.627	1.383	1.387	1.398	1.402	1.144	1.473	0.325	0.304	0.326
TRINITY_DN1212_c0_g1_i8_orf1	extensin isoform X5 [Ostrinia furnacalis]	7	393	44.1	9.39	26	12	7	High	1	1.058	1.064	1.515	1.627	1.56	1.666	1.636	1.502	1.323	1.215	1.572	0.477	0.462	0.509
TRINITY_DN24310_c0_g1_i2_orf1	glucose-6-phosphate 1-dehydrogenase [Ostrinia furnacalis]	11	445	51.3	8.65	25	14	11	High	1	0.974	1.033	1.374	1.329	1.38	1.227	1.24	1.28	1.389	1.363	1.385	1.396	1.39	1.465
TRINITY_DN391_c5_g1_i1_orf1	hypothetical protein B5X24_HaOG210395 [Helicoverpa armigera]	9	466	52.6	6.76	26	18	8	High	1	1.011	1.004	0.485	0.45	0.523	0.446	0.464	0.474	0.468	0.454	0.443	0.483	0.458	0.441
TRINITY_DN1465_c0_g2_i1_orf1	unnamed protein product, partial [Iphiclides podalirius]	8	528	57.9	7.09	23	10	8	High	1	1.001	1.034	0.345	0.358	0.389	0.398	0.398	0.415	0.406	0.506	0.375	0.36	0.352	0.36
TRINITY_DN8637_c0_g1_i1_orf1	superoxide dismutase [Cu-Zn] [Ostrinia furnacalis]	7	154	15.7	6.51	68	14	6	High	1	1.015	1.054	1.514	1.558	1.474	1.948	1.932	1.846	1.778	1.824	1.949	1.729	1.748	1.64
TRINITY_DN19058_c1_g1_i1_orf1	syntaxin-7 [Helicoverpa armigera]	7	264	30	6.81	38	11	7	High	1	0.995	1.048	1.083	1.082	1.084	1.08	1.094	1.097	1.033	1.044	1.054	1.389	1.323	1.293
TRINITY_DN7836_c0_g1_i2_orf1	uncharacterized protein LOC114353624 [Ostrinia furnacalis]	13	1521	168.8	7.53	11	13	13	High	1	1.026	1.004	0.772	0.79	0.838	0.796	0.858	0.898	0.886	0.772	0.751	0.761	0.828	0.798
TRINITY_DN3374_c0_g1_i7_orf1	TPPP family protein CG45057 [Ostrinia furnacalis]	9	203	22.2	9.01	40	14	9	High	1	1.057	0.986	1.476	1.501	1.61	1.493	1.384	1.39	1.335	1.119	1.287	1.049	1.026	0.978
TRINITY_DN5874_c0_g1_i2_orf1	endoplasmic reticulum resident protein 44 isoform X1 [Ostrinia furnacalis]	8	404	46.6	6.29	24	14	8	High	1	0.958	0.931	0.794	0.791	0.859	0.832	0.872	0.822	0.818	0.797	0.842	0.836	0.822	0.862
TRINITY_DN43792_c0_g1_i1_orf1	40S ribosomal protein S3a [Spodoptera litura]	8	207	23.5	8.76	36	16	7	High	1	1.001	1.021	0.729	0.678	0.78	0.732	0.696	0.757	0.631	0.598	0.595	0.682	0.696	0.688
TRINITY_DN2075_c0_g1_i4_orf1	rabankyrin-5 isoform X2 [Ostrinia furnacalis]	11	1183	127.4	5.86	13	11	11	High	1	1.036	1.015	1.064	0.973	1.068	1.128	1.049	1.11	1.013	1.057	0.934	1.064	1.045	1.15
TRINITY_DN2146_c0_g1_i1_orf1	heat shock protein 68-like [Ostrinia furnacalis]	10	636	70.2	5.91	19	16	2	High	1	1.017	0.997	0.908	0.773	0.815	0.899	0.894	0.868	0.852	0.785	0.855	0.657	0.733	0.684
TRINITY_DN2706_c0_g1_i3_orf1	hypothetical protein evm_004793 [Chilo suppressalis]	10	1414	156.7	7.06	9	12	10	High	1	0.997	1.035	1.742	1.723	1.723	1.509	1.525	1.577	1.493	1.598	1.486	1.25	1.311	1.321
TRINITY_DN90321_c0_g2_i1_orf1	pre-mRNA-processing-splicing factor 8 [Apis dorsata]	11	2378	277.6	8.87	5	11	11	High	1	1.011	0.979	0.834	0.797	0.82	0.807	0.89	0.886	0.784	0.79	0.746	0.925	0.928	0.92
TRINITY_DN3029_c4_g1_i1_orf1	proliferation marker protein Ki-67-like, partial [Ostrinia furnacalis]	12	1724	188.8	10.89	8	12	12	High	1	0.963	0.967	0.722	0.817	0.814	0.807	0.874	0.809	0.561	0.632	0.611	0.815	0.817	0.764
TRINITY_DN2072_c0_g1_i1_orf1	dnaJ homolog subfamily C member 3 [Helicoverpa zea]	12	491	56.3	6.64	22	16	12	High	1	0.994	1.012	1.116	1.177	1.048	1.074	1.142	1.08	0.873	0.858	0.893	0.928	0.89	0.915
TRINITY_DN30306_c0_g2_i1_orf1	perilipin-4-like isoform X3 [Ostrinia furnacalis]	6	72	7.2	4.88	64	22	6	High	1	1.032	1.006	4.939	5.428	4.992	4.051	4.213	3.831	3.228	2.791	3.715	1.792	1.881	1.951
TRINITY_DN13972_c0_g1_i5_orf1	myelin expression factor 2-like [Ostrinia furnacalis]	8	518	57.1	9.11	17	13	8	High	1	0.951	1	0.69	0.677	0.688	0.569	0.638	0.596	0.583	0.58	0.598	0.787	0.801	0.773
TRINITY_DN96801_c0_g1_i1_orf1	histone H4 isoform X2 [Gracilinanus agilis]	9	112	12.4	11.39	53	22	2	High	1	1.068	1.123	1.332	1.224	1.374	1.368	1.465	1.464	1.15	1.047	0.915	1.181	1.185	1.161
TRINITY_DN5829_c0_g1_i1_orf1	uncharacterized protein LOC114365758 isoform X3 [Ostrinia furnacalis]	5	207	23.4	6.37	39	10	5	High	1	1.012	1.06	0.957	0.892	0.904	0.936	1.065	0.978	0.956	1.253	0.959	6.162	6.33	6.094
TRINITY_DN15327_c2_g1_i2_orf1	protein lethal(2)essential for life-like [Ostrinia furnacalis]	7	193	22	6.55	44	17	7	High	1	1.032	0.984	2.215	2.268	2.151	2.324	2.394	2.312	2.659	2.355	2.871	0.765	0.736	0.747
TRINITY_DN23183_c0_g1_i2_orf1	nuclear protein localization protein 4 homolog isoform X2 [Ostrinia furnacalis]	8	554	61.1	6.98	18	10	8	High	1	1.04	1.013	1.26	1.246	1.194	1.112	1.126	1.136	1.123	0.938	1.051	1.265	1.209	1.201
TRINITY_DN1090_c0_g1_i1_orf1	RNA helicase aquarius isoform X1 [Ostrinia furnacalis]	12	1440	166.1	6.68	12	12	12	High	1	0.992	0.976	0.9	0.89	0.915	0.896	0.936	0.928	0.853	0.938	0.861	0.917	0.91	0.961
TRINITY_DN9980_c0_g1_i1_orf1	unnamed protein product [Chrysodeixis includens]	10	276	30.3	6.64	36	24	10	High	1	0.992	1.004	1.056	1.004	1.051	0.988	0.963	1.019	0.949	0.897	0.863	1.078	1.132	1.101
TRINITY_DN5562_c1_g1_i3_orf1	cell division cycle and apoptosis regulator protein 1-like [Ostrinia furnacalis]	9	623	70.1	8.82	22	12	9	High	1	1.042	1.054	0.765	0.77	0.771	0.727	0.766	0.705	0.751	0.737	0.732	0.811	0.773	0.75
TRINITY_DN3292_c2_g2_i1_orf1	aldo-keto reductase AKR2E4-like [Ostrinia furnacalis]	7	322	35.8	6.44	32	11	6	High	1	1.03	1.029	1.388	1.36	1.29	1.428	1.475	1.35	1.321	1.193	1.386	1.71	1.635	1.653
TRINITY_DN4469_c0_g1_i2_orf1	metal transporter CNNM4-like [Ostrinia furnacalis]	8	921	102.9	5.11	11	12	8	High	1	1.011	1.022	0.746	0.748	0.74	0.671	0.693	0.656	0.588	0.56	0.571	0.603	0.574	0.6
TRINITY_DN1316_c0_g1_i1_orf1	mitochondrial import receptor subunit TOM70 [Ostrinia furnacalis]	10	548	61.6	6.27	19	13	10	High	1	0.997	1.031	0.575	0.562	0.606	0.59	0.569	0.607	0.676	0.611	0.639	0.626	0.657	0.639
TRINITY_DN5244_c0_g1_i1_orf1	eukaryotic peptide chain release factor GTP-binding subunit-like [Ostrinia furr	6	462	48.5	6.93	20	11	6	High	1	1.001	0.977	0.313	0.319	0.353	0.351	0.346	0.347	0.302	0.28	0.322	0.159	0.148	0.158
TRINITY_DN43881_c0_g1_i2_orf1	estradiol 17-beta-dehydrogenase 8-like [Ostrinia furnacalis]	7	255	26.4	8.09	39	11	7	High	1	0.989	1.077	1.298	1.246	1.107	0.94	0.899	0.984	0.806	0.794	0.786	0.748	0.681	0.79
TRINITY_DN27592_c0_g1_i1_orf1	D-arabinitol dehydrogenase 1-like [Ostrinia furnacalis]	7	133	15.1	8.53	59	17	1	High	1	0.972	0.943	0.851	0.653	0.787	0.125	0.172	0.18	0.223	0.204	0.216	0.155	0.172	0.174
TRINITY_DN3916_c0_g1_i6_orf1	sarcalumenin [Ostrinia furnacalis]	7	827	92.7	4.36	11	11	7	High	1	1.005	1.003	0.989	1.047	1.006	1.043	1.105	1.695	0.932	0.897	0.939	0.883	0.753	0.676
TRINITY_DN2999_c1_g1_i4_orf1	uncharacterized protein C05D11.1-like [Ostrinia furnacalis]	8	273	29.8	6.52	37	10	8	High	1	1.046	1.003	1.06	1.003	0.987	0.933	0.929	0.945	0.893	0.903	0.885	0.893	0.904	0.9
TRINITY_DN2416_c0_g1_i5_orf1	somatomedin-B and thrombospondin type-1 domain-containing protein [Ost	7	276	30.5	7.43	33	13	7	High	1	1.024	0.986	1.356	1.405	1.356	1.605	1.531	1.486	1.202	1.098	1.199	2.069	2.161	1.977
TRINITY_DN14865_c0_g1_i2_orf1	ubiquilin-1 [Ostrinia furnacalis]	7	427	46.2	5.03	27	11	7	High	1	1.024	1.009	1.008	1.089	1.079	0.932	0.96	0.946	0.886	0.822	0.923	1.138	1.238	1.222
TRINITY_DN4524_c0_g1_i2_orf1	sodium/potassium-transporting ATPase subunit alpha isoform X1 [Chelonus in	10	1041	115.5	5.74	12	13	1	High	1	0.895	1.016	0.927	0.991	0.869	1.074	0.986	0.944	0.939	0.937	0.87	1.254	1.084	1.19
TRINITY_DN2407_c0_g1_i6_orf1	uncharacterized protein LOC114366345 isoform X2 [Ostrinia furnacalis]	7	340	37.6	5.54	24	17	5	High	1	0.957	1.073	1.793	1.847	1.73	1.884	1.926	1.923	1.978	1.814	1.927	2.694	2.902	2.528
TRINITY_DN214_c0_g1_i3_orf1	uncharacterized protein LOC114352813 [Ostrinia furnacalis]	6	174	19.4	7.93	38	16	3	High	1	0.946	0.955	2.923	2.285	2.655	1.599	1.685	1.803	2.048	2.074	1.799	0.964	0.943	1.007
TRINITY_DN12964_c0_g1_i1_orf1	dnaJ homolog subfamily A member 1 [Ostrinia furnacalis]	8	403	45.5	6.9	27	10	8	High	1	1.004	0.992	0.591	0.656	0.62	0.687	0.658	0.651	0.612	0.597	0.571	0.7	0.728	0.751
TRINITY_DN4869_c0_g1_i10_orf1	estrogen sulfotransferase-like isoform X1 [Ostrinia furnacalis]	10	343	39.9	7.61	34	11	9	High	1	1.023	0.971	0.117	0.118	0.138	0.136	0.158	0.165	0.151	0.151	0.139	0.14	0.153	0.149
TRINITY_DN53760_c0_g1_i1_orf1	unnamed protein product [Parnassius apollo]	7	625	71.5	7.55	16	8	7	High	1	0.968	1.011	1.001	1.08	1.044	0.972	0.953	0.909	0.839	0.729	0.857	1.041	1.002	0.917
TRINITY_DN3483_c0_g1_i5_orf1	phenoloxidase-activating factor 2-like isoform X1 [Ostrinia furnacalis]	10	407	44.6	6.37	29	12	10	High	1	1.052	1.075	1.297	1.32	1.293	1.641	1.684	1.665	1.743	1.546	1.808	1.714	1.755	1.645
TRINITY_DN13114_c0_g1_i1_orf1	nicalin-1 [Helicoverpa zea]	7	550	60.5	6.71	16	10	7	High	1	1.085	1.027	0.779	0.76	0.795	0.705	0.75	0.768	0.711	0.753	0.726	0.784	0.88	0.774
TRINITY_DN22664_c0_g1_i1_orf1	larval cuticle protein LCP-14-like [Ostrinia furnacalis]	6	181	19.3	5.26	52	9	6	High	1	1.013	1.037	0.454	0.481	0.481	0.334	0.345	0.331	0.215	0.206	0.225	0.156	0.148	0.163
TRINITY_DN5081_c0_g1_i5_orf1	ester hydrolase C11orf54 homolog isoform X1 [Ostrinia furnacalis]	7	369	40.2	8.51	22	11	7	High	1	0.978	0.957	1.306	1.436	1.319	1.603	1.664	1.575	1.469	1.531	1.612	1.265	1.144	1.239
TRINITY_DN2442_c0_g1_i6_orf1	cytochrome P450 6B5-like [Ostrinia furnacalis]	9	494	56.7	7.93	20	10	7	High	1	0.991	1.04	2.015	1.836	1.896	2.236	2.064	2.365	1.74	1.648	1.58	1.613	1.723	1.613
TRINITY_DN12960_c0_g1_i1_orf1	collagen alpha-2(IV) chain-like [Ostrinia furnacalis]	5	327	36	9.5	26	9	5	High	1	1.005	1.023	1.242	1.266	1.094	1.347	1.416	1.273	1.069	1.228	1.132	1.185	1.128	1.194
TRINITY_DN8771_c0_g1_i5_orf1	regucalcin-like [Ostrinia furnacalis]	9	303	33.2	6.58	32	19	7	High	1	0.97	0.975	1.899	2.005	1.882	1.91	2.077	1.885	1.7	1.6	1.815	2.431	2.485	2.309
TRINITY_DN3609_c0_g1_i6_orf1	leukocyte elastase inhibitor-like [Ostrinia furnacalis]	8	516	56.7	7.31	17	10	8	High	1	1.048	1.045	2.372	2.144	2.329	2.944	2.895	2.868	2.179	1.843	1.924	3.275	3.254	3.266
TRINITY_DN11297_c0_g1_i1_orf1	ribosomal protein L13 [Conogethes punctiferalis]	14	204	23.3	10.86	57	30	14	High	1	0.965	0.955	0.666	0.663	0.696	0.672	0.645	0.655	0.51	0.662	0.524	0.539	0.551	0.552
TRINITY_DN2704_c0_g1_i5_orf1	hypothetical protein evm_009002 [Chilo suppressalis]	9	770	84.2	6.02	16	13	9	High	1	1.008	0.989	0.335	0.305	0.351	0.38	0.369	0.369	0.347	0.374	0.441	0.413	0.511	0.408
TRINITY_DN4125_c0_g1_i14_orf1	angiotensin-converting enzyme-like isoform X1 [Ostrinia furnacalis]	7	644	74.3	5.77	12	9	1	High	1	0.993	0.993	0.641	0.679	0.611	0.633	0.641	0.738	0.718	0.435	0.469	3.012	2.976	2.642
TRINITY_DN15834_c0_g1_i2_orf1	ubiquitin carboxyl-terminal hydrolase isozyme L3 [Ostrinia furnacalis]	8	230	25.4	5	45	13	8	High	1	1.012	0.969	1.031	0.981	1.037	0.974	0.876	0.932	0.903	0.834	0.933	0.996	1.031	0.946
TRINITY_DN14572_c0_g1_i1_orf1	ras-related protein Rab-11A [Ostrinia furnacalis]	8	213	24.1	5.53	46	11	8	High	1	0.987	1.001	0.868	0.802	0.85	0.799	0.817	0.88	0.797	0.736	0.731	0.947	0.968	0.949
TRINITY_DN12242_c0_g1_i5_orf1	heterogeneous nuclear ribonucleoprotein 87F-like isoform X1 [Vanessa tamea	8	318	34	9.16	41	12	8	High	1	1.018	1.006	0.406	0.409	0.446	0.357	0.353	0.346	0.384	0.346	0.345	0.685	0.721	0.686
TRINITY_DN3732_c1_g1_i5_orf1	cytochrome P450 6B2-like [Ostrinia furnacalis]	11	480	55.1	7.74	23	15	5	High	1	0.918	0.985	2.086	2.039	2.094	2.472	2.327	2.339	1.195	1.213	1.213	1.212	1.034	1.044
TRINITY_DN448_c0_g1_i20_orf1	probable cytochrome P450 9f2 isoform X1 [Ostrinia furnacalis]	9	524	60.7	6.62	21	13	8	High	1	0.977	1.001	1.947	1.803	1.856	2.517	2.317	2.578	2.035	1.996	1.835	1.316	1.352	1.332
TRINITY_DN43667_c0_g1_i1_orf1	carbonyl reductase [NADPH] 1-like [Ostrinia furnacalis]	8	288	32.5	6.95	32	13	8	High	1	1.002	1.026	1.595	1.48	1.534	2.447	2.326	2.539	2.056	2.08	1.935	1.617	1.669	1.647
TRINITY_DN7226_c0_g1_i2_orf1	chemosensory protein [Conogethes punctiferalis]	5	76	9	7.39	54	15	5	High	1	0.963	1.003	3.905	4.083	3.859	3.074	3.239	3.225	3.278	5.897	3.502	1.916	1.956	2.112
TRINITY_DN5310_c2_g1_i2_orf1	serine protease persephone-like [Ostrinia furnacalis]	9	308	34.8	6.84	34	15	9	High	1	0.977	1.01	2.787	2.728	2.842	3.711	3.568	3.633	2.43	2.417	2.288	2.976	2.906	3.005
TRINITY_DN696_c1_g1_i10_orf1	titin-like [Ostrinia furnacalis]	9	2641	288.8	4.22	5	12	9	High	1	0.991	1.006	0.977	0.981	0.958	1.062	1.124	1.122	1.046	0.997	1.052	3.198	3.28	3.199
TRINITY_DN6147_c0_g1_i2_orf1	uncharacterized protein LOC114352519 [Ostrinia furnacalis]	10	389	44.6	5.19	27	12	10	High	1	0.971	0.969	0.393	0.4	0.415	0.432	0.442	0.45	0.519	0.499	0.491	0.475	0.432	0.435
TRINITY_DN71698_c0_g1_i1_orfp1	TRINITY_DN71698_c0_g1_i1_m.1194 TRINITY_DN71698_c0_g1::TRINITY_DN71€	7	133	15.5	5.81	47	24	6	High	1	1.024	0.974	3.61	3.886	3.499	2.941	2.929	2.893	3.911	3.307	4.434	1.785	1.727	1.744
TRINITY_DN5564_c0_g1_i5_orf1	probable phosphoserine aminotransferase [Ostrinia furnacalis]	5	123	13.8	9.14	56	14	1	High	1	1.11	1.056	1.134	1.031	1.004	1.108	1.113	1.17	1.395	1.589	1.317	0.879	0.952	0.807
TRINITY_DN10138_c0_g1_i1_orf1	storage protein 1 [Omphisa fuscidentalis]	6	85	10.1	5.14	91	40	6	High	1	0.979	0.992	2.068	2.197	2.07	2.189	2.356	2.095	3.411	4.007	3.953	2.856	2.887	2.835
TRINITY_DN7112_c0_g1_i1_orf1	heterogeneous nuclear ribonucleoprotein K isoform X2 [Ostrinia furnacalis]	7	210	22.2	7.24	48	8	7	High	1	1.014	0.95	0.609	0.642	0.641	0.454	0.441	0.452	0.377	0.392	0.419	0.759	0.788	0.728
TRINITY_DN2794_c1_g1_i8_orf1	carboxypeptidase D [Ostrinia furnacalis]	12	1382	155.8	5.77	8	14	12	High	1	1.013	1.052	1.822	1.732	1.803	1.676	1.671	1.742	1.634	1.482	1.463	2.761	2.941	2.726
TRINITY_DN24043_c0_g1_i1_orf1	uncharacterized protein LOC110377964 [Helicoverpa armigera]	7	351	39.4	9.23	23	14	7	High	1	1.017	0.98	0.51	0.51	0.544	0.456	0.447	0.47	0.518	0.486	0.506	0.461	0.473	0.458
TRINITY_DN4731_c0_g1_i1_orf1	gelsolin-like [Ostrinia furnacalis]	7	649	72	4.68	15	8	7	High	1	1.019	0.992	0.199	0.194	0.216	0.248	0.263	0.268	0.217	0.249	0.237	0.208	0.209	0.223
TRINITY_DN3909_c0_g2_i2_orf1	ribosomal protein L24 [Loxostege sticticalis]	8	157	17.7	10.98	40	19	8	High	1	0.982	0.97	0.748	0.761	0.775	0.707	0.719	0.73	0.574	0.546	0.632	0.623	0.659	0.618
TRINITY_DN38685_c0_g1_i4_orf1	unnamed protein product [Euphydryas editha]	8	349	40.2	8.15	34	11	8	High	1	0.997	0.946	1.584	1.695	1.586	1.544	1.535	1.584	1.391	1.361	1.515	1.385	1.354	1.373
TRINITY_DN6595_c1_g1_i6_orf1	methenyltetrahydrofolate synthase domain-containing protein [Ostrinia furnac	11	473	52.3	9.79	23	14	11	High	1	1.009	1.017	1.095	1.254	1.118	1.3	1.395	1.284	1.022	0.994	1.162	1.17	1.192	1.212
TRINITY_DN2089_c0_g1_i5_orf1	eukaryotic translation initiation factor 4B [Ostrinia furnacalis]	10	575	65.7	9.32	17	13	10	High	1	0.981	1.008	0.623	0.637	0.632	0.503	0.506	0.497	0.462	0.404	0.503	0.686	0.669	0.653
TRINITY_DN24_c0_g1_i1_orf1	hypothetical protein evm_007803 [Chilo suppressalis]	10	539	60.2	5.57	19	15	9	High	1	1.002	0.924	3.101	2.868	3.099	2.856	2.835	2.91	2.057	1.707	1.795	1.913	1.992	1.945
TRINITY_DN1552_c0_g1_i3_orf1	casein kinase II subunit alpha isoform X3 [Galleria mellonella]	8	391	46.3	8.22	28	12	8	High	1	0.972	1.049	0.846	0.856	0.849	0.761	0.803	0.796	0.775	0.808	0.814	1.016	0.999	1.001
TRINITY_DN870_c0_g1_i3_orf1	talin-1 isoform X12 [Ostrinia furnacalis]	10	334	37.9	7.49	40	11	9	High	1	1.027	1.047	1.208	1.295	1.235	1.291	1.215	1.26	1.124	0.959	1.15	0.901	0.844	0.847
TRINITY_DN24917_c0_g2_i1_orf1	hypothetical protein L3Q82_022586 [Scortum barcoo]	8	117	13.1	11.36	44	20	1	High	1	1.241	1.155	0.44	0.539	0.468	0.564	0.493	0.635	0.599	1.01	0.636	0.57	0.655	0.721
TRINITY_DN1952_c0_g1_i2_orf1	uncharacterized protein LOC114354403 [Ostrinia furnacalis]	11	608	70.8	5.91	17	14	11	High	1	0.986	1.009	0.627	0.588	0.619	0.696	0.665	0.695	0.654	0.603	0.599	0.386	0.384	0.371
TRINITY_DN4125_c0_g1_i6_orf1	angiotensin-converting enzyme-like isoform X1 [Ostrinia furnacalis]	7	501	58.1	5.97	15	10	1	High	1	0.835	0.85	1.159	1.071	1.235	1.215	1.891	1.532	1.11	1.199	1.096	1.409	1.49	1.666
TRINITY_DN3588_c0_g1_i1_orf1	probable peroxisomal acyl-coenzyme A oxidase 1 [Ostrinia furnacalis]	9	666	74.9	8.76	16	12	9	High	1	0.992	1.007	0.786	0.78	0.76	0.703	0.676	0.758	0.64	0.745	0.676	0.7	0.618	0.614
TRINITY_DN47842_c0_g1_i1_orf1	protein lethal(2)essential for life-like [Helicoverpa armigera]	7	185	20.7	5.27	43	12	7	High	1	1.105	1.126	1.513	1.508	1.513	3.165	2.939	2.899	2.137	2.282	2.38	3.065	2.966	2.875
TRINITY_DN1888_c0_g2_i1_orf1	peptidyl-prolyl cis-trans isomerase FKBP8 [Ostrinia furnacalis]	9	402	44.5	5.85	33	12	9	High	1	0.975	1.022	0.79	0.858	0.825	0.844	0.78	0.793	0.701	0.865	0.718	0.868	0.886	0.858
TRINITY_DN2314_c0_g1_i7_orf1	protein dj-1beta-like isoform X1 [Ostrinia furnacalis]	7	231	24.5	8.51	45	13	4	High	1	1.062	0.995	2.014	2.008	2.021	2.844	2.757	2.622	3.214	2.824	3.143	2.116	2.106	2.105
TRINITY_DN24281_c0_g1_i1_orf1	elongin-B isoform X1 [Maniola jurtina]	9	119	13.4	4.97	80	14	9	High	1	1.014	0.975	0.634	0.594	0.682	0.547	0.523	0.524	0.592	0.556	0.508	0.523	0.531	0.532
TRINITY_DN125427_c0_g1_i1_orf1	heat shock protein 19.8 [Chilo suppressalis]	7	188	21.2	8.12	39	15	3	High	1	1.059	0.953	0.881	0.911	0.954	1.176	1.099	1.21	1.313	1.132	1.327	2.54	2.621	2.484
TRINITY_DN11172_c0_g1_i4_orf1	juvenile hormone epoxide hydrolase-like isoform X1 [Ostrinia furnacalis]	11	461	52.7	8.65	24	17	11	High	1	0.987	0.984	1.048	0.978	1.033	1.277	1.19	1.32	0.81	0.802	0.734	0.794	0.851	0.826
TRINITY_DN1578_c0_g3_i1_orf1	S-adenosylmethionine synthase isoform X1 [Ostrinia furnacalis]	9	253	28.2	5.14	42	12	7	High	1	1.075	1.03	0.381	0.465	0.45	0.424	0.455	0.457	0.456	0.559	0.502	0.416	0.402	0.414

TRINITY_DN41736_c0_g2_i1_orf1	calcyclin-binding protein [Ostrinia furnacalis]	9	235	27.6	8	41	12	9	High	1	1.062	1.028	0.763	0.739	0.755	0.73	0.732	0.763	0.743	0.84	0.709	0.991	0.957	0.989
TRINITY_DN3126_c0_g1_i4_orf1	unnamed protein product, partial [Iphiclides podalirius]	6	87	9.6	9.31	72	13	6	High	1	1.064	1.041	1.153	1.098	1.12	0.992	0.835	0.939	1.238	1.096	1.244	0.934	0.974	0.884
TRINITY_DN115_c0_g1_i6_orf1	basigin [Ostrinia furnacalis]	7	266	29.8	5.16	29	14	7	High	1	0.999	0.971	0.692	0.7	0.75	0.713	0.722	0.73	0.69	0.649	0.721	0.79	0.821	0.785
TRINITY_DN28711_c0_g1_i1_orf1	hypothetical protein evm_000299 [Chilo suppressalis]	7	69	7.5	9.38	83	20	7	High	1	0.956	1.001	1.54	1.571	1.476	1.736	1.793	1.655	2.636	3.504	2.972	3.926	3.962	4.132
TRINITY_DN610_c1_g1_i1_orf1	restin homolog isoform X16 [Ostrinia furnacalis]	10	1392	160.3	5.15	9	10	10	High	1	1.096	1.072	1.008	1.089	1.045	1.026	1.091	1.023	1.015	1.021	1.071	1.077	1.042	1.037
TRINITY_DN3637_c0_g1_i2_orf1	ATP-binding cassette sub-family G member 4 isoform X1 [Ostrinia furnacalis]	8	697	77.8	8.16	16	10	8	High	1	0.991	1.038	1.323	1.323	1.248	1.16	1.132	1.162	1.121	1.261	1.136	1.416	1.421	1.387
TRINITY_DN18869_c0_g1_i1_orf1	unnamed protein product [Parnassius apollo]	7	327	35.5	4.98	33	16	7	High	1	0.988	0.892	0.689	0.648	0.693	0.627	0.629	0.655	0.564	0.679	0.56	0.545	0.537	0.536
TRINITY_DN4497_c2_g1_i3_orf1	uncharacterized protein LOC114353086 [Ostrinia furnacalis]	11	518	59.8	8.46	20	15	9	High	1	1.031	0.985	1.865	1.84	1.845	2.386	2.302	2.37	2.166	2.092	2.132	1.428	1.504	1.49
TRINITY_DN4944_c0_g1_i5_orf1	bifunctional glutamate/proline--tRNA ligase [Ostrinia furnacalis]	9	853	92.4	9.28	14	12	4	High	1	1.03	1.069	0.63	0.674	0.712	0.588	0.531	0.531	0.595	0.584	0.644	0.64	0.661	0.662
TRINITY_DN62_c0_g1_i18_orf1	hypothetical protein evm_002481 [Chilo suppressalis]	7	419	46.7	5.03	25	9	7	High	1	1.008	0.94	0.69	0.695	0.677	0.645	0.588	0.647	0.648	0.593	0.62	0.833	0.84	0.77
TRINITY_DN4944_c0_g1_i2_orf1	bifunctional glutamate/proline--tRNA ligase [Ostrinia furnacalis]	11	450	51	6.92	28	14	6	High	1	0.965	0.972	0.482	0.474	0.472	0.423	0.418	0.413	0.405	0.4	0.418	0.483	0.5	0.469
TRINITY_DN4676_c0_g1_i16_orf1	meiosis-specific nuclear structural protein 1-like isoform X2 [Ostrinia furnacalis	9	291	33.5	7.47	34	12	9	High	1	0.994	1.051	1.543	1.629	1.584	1.018	1.159	1.067	1.237	1.174	1.234	3.712	3.808	3.585
TRINITY_DN4711_c0_g1_i2_orf1	xanthine dehydrogenase-like isoform X1 [Ostrinia furnacalis]	8	1267	138.8	6.74	8	9	8	High	1	0.997	1.012	1.125	1.056	1.133	0.986	1.085	1.013	0.939	0.918	0.885	1.237	1.223	1.283
TRINITY_DN863_c0_g1_i6_orf1	protein henna [Galleria mellonella]	9	456	52.2	5.72	26	9	9	High	1	1.04	0.995	0.629	0.596	0.649	0.839	0.805	0.885	0.753	0.777	0.664	0.564	0.55	0.534
TRINITY_DN20658_c0_g1_i1_orf1	prostaglandin reductase 1-like [Ostrinia furnacalis]	8	158	17.6	8.53	49	17	8	High	1	1.014	1.043	2.546	2.553	2.471	2.505	2.543	2.517	2.276	2.021	2.348	1.661	1.75	1.732
TRINITY_DN2876_c0_g1_i1_orf1	long-chain fatty acid transport protein 4-like [Ostrinia furnacalis]	8	652	72	8.37	16	8	5	High	1	1.032	0.984	1.633	1.632	1.74	1.621	1.63	1.556	1.405	1.614	1.181	1.462	1.303	1.455
TRINITY_DN5382_c0_g2_i1_orf1	protein seele [Ostrinia furnacalis]	7	239	27.3	4.72	28	12	7	High	1	0.934	0.975	0.822	0.861	0.813	0.687	0.714	0.729	0.877	0.782	0.878	0.709	0.686	0.676
TRINITY_DN36538_c0_g1_i2_orf1	xaa-Pro dipeptidase isoform X1 [Ostrinia furnacalis]	9	545	60.8	6.81	18	10	9	High	1	1.028	1.005	0.42	0.432	0.461	0.38	0.389	0.368	0.362	0.424	0.353	0.482	0.472	0.488
TRINITY_DN31637_c0_g1_i3_orf1	protein CDV3 homolog isoform X1 [Ostrinia furnacalis]	8	214	23.3	6.96	63	11	8	High	1	0.993	1.019	0.721	0.699	0.729	0.634	0.699	0.622	0.6	0.585	0.64	0.728	0.754	0.745
TRINITY_DN268_c3_g1_i2_orf1	hypothetical protein evm_003084 [Chilo suppressalis]	8	525	57.1	7.08	20	11	8	High	1	1.012	0.992	1.26	1.299	1.286	1.277	1.208	1.213	1.239	1.136	1.315	1.031	1.055	0.978
TRINITY_DN2682_c0_g1_i4_orf1	40S ribosomal protein S5 [Manduca sexta]	7	228	25.6	9.31	26	21	7	High	1	0.989	0.986	0.737	0.812	0.796	0.652	0.67	0.635	0.53	0.545	0.614	0.603	0.592	0.593
TRINITY_DN1926_c0_g1_i5_orf1	coiled-coil and C2 domain-containing protein 1-like [Ostrinia furnacalis]	9	719	77.6	5.64	19	9	9	High	1	1.062	1.024	1.521	1.529	1.6	1.52	1.424	1.528	1.396	1.283	1.424	1.244	1.318	1.197
TRINITY_DN3588_c0_g1_i4_orf1	probable peroxisomal acyl-coenzyme A oxidase 1 isoform X1 [Ostrinia furnaca	9	669	75.2	7.53	16	10	9	High	1	1.003	1.009	1.22	1.295	1.264	1.002	1.053	1.055	1.002	0.918	0.879	1.124	1.059	1.073
TRINITY_DN14532_c0_g1_i1_orf1	pupal cuticle protein-like [Trichoplusia ni]	4	245	25.9	6.65	26	9	4	High	1	0.953	0.991	0.609	0.614	0.586	0.718	0.782	0.687	0.62	0.604	0.657	2.547	2.647	2.595
TRINITY_DN4572_c0_g3_i1_orf1	putative aminopeptidase W07G4.4 isoform X2 [Ostrinia furnacalis]	4	105	10.6	5.45	45	22	4	High	1	1.033	1.034	0.996	1.003	0.998	1.106	1.101	1.092	1.005	1.055	1.172	1.467	1.483	1.472
TRINITY_DN4320_c0_g1_i1_orf1	selenide, water dikinase [Ostrinia furnacalis]	6	402	44.1	6.74	21	10	6	High	1	0.945	0.954	0.968	1.056	0.917	0.921	0.828	0.91	0.897	0.923	0.917	1.518	1.689	1.735
TRINITY_DN1402_c1_g1_i6_orf1	unnamed protein product [Parnassius apollo]	5	282	32.8	8.54	23	14	5	High	1	0.994	0.962	1.007	0.977	0.999	0.934	0.912	0.855	1.12	1.046	1.199	0.878	0.89	0.898
TRINITY_DN2594_c0_g2_i4_orf1	isocitrate dehydrogenase [NAD] subunit beta, mitochondrial isoform X2 [Ostrir	5	378	41.2	8.35	13	10	3	High	1	1.02	1.04	0.339	0.314	0.347	0.287	0.299	0.293	0.32	0.338	0.325	0.399	0.393	0.379
TRINITY_DN8291_c0_g1_i3_orf1	probable cysteine desulfurase, mitochondrial [Ostrinia furnacalis]	8	454	50.1	8.19	22	8	8	High	1	1.014	1.066	0.858	0.827	0.856	0.868	0.863	0.877	0.865	0.894	0.87	0.869	0.813	0.779
TRINITY_DN11415_c0_g1_i4_orf1	maestro heat-like repeat-containing protein family member 1 [Ostrinia furnac	11	1648	184.8	6.43	8	11	11	High	1	1.043	1.072	1.198	1.066	1.11	1.121	1.127	1.246	1.201	1.139	1.014	1.19	1.182	1.179
TRINITY_DN1718_c1_g1_i5_orf1	gelsolin-like [Ostrinia furnacalis]	8	291	32.8	5.3	27	13	5	High	1	0.98	0.952	1.039	1.028	0.997	1.074	0.976	0.991	0.941	0.812	1.007	0.677	0.694	0.648
TRINITY_DN35147_c0_g1_i1_orf1	collagen alpha-2(IV) chain isoform X2 [Ostrinia furnacalis]	5	269	29	5.97	35	11	5	High	1	0.984	1.022	1.192	1.229	1.15	1.802	1.675	1.592	1.234	1.102	1.295	1.42	1.436	1.364
TRINITY_DN19990_c0_g1_i1_orf1	carboxypeptidase B-like [Ostrinia furnacalis]	7	513	57.6	5.86	16	9	7	High	1	1.003	1.084	1.062	1.065	1.093	1.058	1.109	1.053	1.235	1.214	1.145	3.847	3.886	3.798
TRINITY_DN76815_c0_g1_i3_orf1	5-formyltetrahydrofolate cyclo-ligase [Ostrinia furnacalis]	7	127	14.2	9.2	61	13	1	High	1	0.857	0.899	0.77	0.808	0.887	1.335	1.245	1.189	0.176	0.082	0.116	0.133	0.079	0.081
TRINITY_DN10090_c0_g1_i1_orf1	clotting factor B-like isoform X1 [Ostrinia furnacalis]	9	418	45.8	7.9	28	11	9	High	1	0.999	1.003	1.72	1.743	1.627	1.652	1.762	1.728	1.791	1.681	1.813	2.203	2.248	2.135
TRINITY_DN16539_c0_g1_i7_orf1	dolichyl-diphosphooligosaccharide--protein glycosyltransferase 48 kDa subur	8	435	48.9	6.01	20	13	8	High	1	0.987	1.026	0.952	0.836	0.905	0.914	0.85	0.962	0.852	0.791	0.738	0.874	0.837	0.902
TRINITY_DN95530_c0_g1_i1_orf1	aldose reductase-like isoform X4 [Ostrinia furnacalis]	6	145	16.3	5.55	41	27	1	High	1	0.988	0.912	1.005	1.016	0.874	1.176	1.288	1.175	1.147	0.957	1.141	1.005	0.913	0.899
TRINITY_DN21469_c0_g1_i4_orf1	poly [ADP-ribose] polymerase [Ostrinia furnacalis]	9	998	112.6	8.4	11	10	9	High	1	0.954	0.994	0.979	1.053	1.012	1.058	1.093	1.058	1.052	1.143	1.067	1.048	1.05	1.066
TRINITY_DN126127_c0_g1_i1_orf1	prolow-density lipoprotein receptor-related protein 1, partial [Ostrinia furnaca	9	517	56.4	6.48	25	12	9	High	1	0.972	0.946	1.718	1.696	1.604	1.987	2.098	1.899	1.775	1.725	1.809	2.34	2.323	2.245
TRINITY_DN8367_c0_g1_i1_orf1	uncharacterized protein LOC114357075 [Ostrinia furnacalis]	7	220	23.4	9.63	36	10	3	High	1	1.062	1.009	0.443	0.454	0.482	0.51	0.506	0.539	0.514	0.471	0.537	0.603	0.559	0.618
TRINITY_DN4793_c0_g1_i7_orf1	probable hydroxyacid-oxoacid transhydrogenase, mitochondrial isoform X3 [C	7	462	50.1	6.6	16	10	7	High	1	1.001	0.981	1.331	1.292	1.34	1.378	1.401	1.428	1.048	1.04	1.098	0.872	0.833	0.863
TRINITY_DN1280_c0_g1_i1_orf1	coronin-7 isoform X1 [Ostrinia furnacalis]	11	1247	137.3	6.93	12	11	11	High	1	1.029	0.983	0.799	0.798	0.803	0.758	0.765	0.78	0.784	0.699	0.799	1.315	1.329	1.298
TRINITY_DN5653_c0_g1_i4_orf1	hrp65 protein-like [Ostrinia furnacalis]	10	471	54.7	8.25	26	11	10	High	1	0.986	0.959	0.593	0.615	0.639	0.511	0.535	0.547	0.543	0.559	0.537	0.87	0.799	0.714
TRINITY_DN1400_c0_g1_i21_orf1	pyridoxal-dependent decarboxylase domain-containing protein 1 [Ostrinia fur	8	721	78.4	5.62	14	9	8	High	1	0.978	0.973	0.853	0.909	0.834	0.832	0.868	0.824	0.766	0.889	0.819	0.76	0.783	0.764
TRINITY_DN4628_c0_g1_i1_orf1	guanine nucleotide-binding protein G(q) subunit alpha isoform X1 [Ostrinia fu	9	353	41.5	5.31	35	12	9	High	1	0.976	0.988	1.061	1.03	1.003	1.153	1.238	1.197	0.998	0.969	0.973	1.024	1.047	1.046
TRINITY_DN33763_c0_g1_i1_orf1	uncharacterized protein LOC114355186 [Ostrinia furnacalis]	7	273	29.9	6.55	40	11	7	High	1	0.984	0.98	1.055	1.04	1.043	1.39	1.377	1.344	1.3	1.309	1.309	0.879	0.895	0.913
TRINITY_DN20658_c0_g2_i3_orf1	prostaglandin reductase 1-like [Ostrinia furnacalis]	7	238	25.7	8.34	27	20	7	High	1	0.968	1.003	2.464	2.181	2.507	3.029	2.77	3.079	2.24	2.401	1.876	1.793	1.811	1.815
TRINITY_DN12944_c0_g1_i1_orf1	lachesin-like [Ostrinia furnacalis]	7	460	51.3	5.22	26	8	7	High	1	0.965	1.016	0.971	1.043	1.004	0.926	0.999	0.967	0.86	0.906	0.922	1.024	0.997	1.017
TRINITY_DN6169_c0_g1_i15_orf1	cullin-2 isoform X1 [Ostrinia furnacalis]	9	776	86	7.62	14	9	9	High	1	1.034	0.993	0.957	0.943	0.921	0.812	0.89	0.888	0.821	0.839	0.778	0.913	0.919	0.898
TRINITY_DN9820_c0_g1_i1_orf1	endocuticle structural glycoprotein SgAbd-2-like [Ostrinia furnacalis]	4	147	16	4.88	56	11	4	High	1	1.038	1.021	1.962	2.094	2.024	1.866	1.935	1.857	1.622	1.337	1.621	0.469	0.404	0.472
TRINITY_DN1622_c0_g1_i6_orf1	unnamed protein product [Parnassius apollo]	10	644	73.3	7.37	19	12	10	High	1	1.022	1.002	1.616	1.484	1.526	1.58	1.516	1.657	1.207	1.205	1.167	1.082	1.078	1.041
TRINITY_DN1914_c0_g1_i4_orf1	loricrin-like [Ostrinia furnacalis]	6	591	58.5	6.55	15	7	1	High	1	0.968	0.979	0.501	0.535	0.529	1.261	1.306	1.236	0.624	0.58	0.634	0.366	0.268	0.296
TRINITY_DN3251_c0_g1_i6_orf1	fatty-acid amide hydrolase 2-like [Ostrinia furnacalis]	8	543	59.5	8.22	19	11	8	High	1	0.966	1.025	1.719	1.611	1.663	1.534	1.532	1.642	1.726	1.687	1.528	2.024	1.948	1.97
TRINITY_DN338_c1_g1_i9_orf1	scolexin B-like isoform X2 [Ostrinia furnacalis]	7	360	38.2	6.52	23	10	7	High	1	1.025	1.016	1.733	1.777	1.711	1.626	1.691	1.655	2.135	1.852	1.985	1.988	2.016	2.007
TRINITY_DN107_c0_g1_i1_orf1	dynein light chain 2, cytoplasmic isoform X1 [Pectinophora gossypiella]	3	117	13.5	8.56	38	6	3	High	1	1.112	1.077	0.854	0.796	0.909	0.81	0.862	0.774	0.857	0.743	0.84	0.972	0.902	0.906
TRINITY_DN9770_c0_g1_i1_orf1	flavin reductase (NADPH) [Ostrinia furnacalis]	6	205	22.4	5.49	35	15	6	High	1	0.942	0.938	0.608	0.61	0.64	0.666	0.67	0.688	0.565	0.573	0.569	0.606	0.608	0.615
TRINITY_DN2516_c0_g2_i10_orf1	unnamed protein product, partial [Chilo suppressalis]	9	1132	131.4	7.39	11	9	9	High	1	1.027	1.061	1.687	1.626	1.621	1.534	1.5	1.544	1.49	1.5	1.435	1.44	1.438	1.411
TRINITY_DN53167_c0_g1_i3_orf1	uncharacterized protein LOC114359219 [Ostrinia furnacalis]	6	489	55.9	4.74	16	11	6	High	1	1.023	1.023	0.362	0.398	0.382	0.351	0.355	0.355	0.319	0.39	0.322	0.31	0.313	0.319
TRINITY_DN52944_c0_g1_i1_orf1	apolipophorins-like [Ostrinia furnacalis]	5	100	11	7.24	59	22	5	High	1	0.98	1.086	1.469	1.525	1.443	1.601	1.581	1.616	2.444	2.576	2.371	3.317	3.283	3.15
TRINITY_DN9874_c0_g1_i7_orf1	hypothetical protein evm_013697 [Chilo suppressalis]	11	200	23.3	10.87	44	32	7	High	1	1.053	1.048	0.69	0.627	0.715	0.642	0.613	0.663	0.632	0.565	0.534	0.567	0.569	0.554
TRINITY_DN3053_c0_g1_i2_orf1	prostaglandin reductase 1-like [Ostrinia furnacalis]	9	355	39.1	8.81	28	12	9	High	1	1.005	0.971	0.468	0.468	0.516	0.43	0.453	0.457	0.398	0.386	0.402	0.398	0.388	0.395
TRINITY_DN10502_c0_g1_i4_orf1	interleukin enhancer-binding factor 2 homolog [Ostrinia furnacalis]	9	389	41.8	7.53	29	11	9	High	1	1.025	1.025	0.675	0.679	0.702	0.662	0.674	0.67	0.6	0.673	0.63	0.881	0.863	0.849
TRINITY_DN23978_c0_g1_i2_orf1	insulin-like growth factor-binding protein complex acid labile subunit [Ostrinia	10	856	95.8	4.7	14	11	10	High	1	1.033	1.019	1.17	1.167	1.176	0.924	0.958	0.972	1.477	1.416	1.508	1.412	1.405	1.506
TRINITY_DN13973_c0_g1_i6_orf1	27 kDa glycoprotein-like [Ostrinia furnacalis]	6	261	29.1	5.02	35	8	6	High	1	0.965	1.02	1.229	1.204	1.272	1.867	1.747	1.706	2.017	2.137	2.045	2.787	2.592	2.734
TRINITY_DN28711_c1_g1_i1_orf1	apolipophorins-like [Ostrinia furnacalis]	7	92	9.8	8.51	86	14	7	High	1	0.945	0.993	1.391	1.489	1.36	1.581	1.564	1.643	2.296	2.453	2.392	3.333	3.2	3.229
TRINITY_DN472_c0_g1_i6_orf1	hypothetical protein evm_002904 [Chilo suppressalis]	8	776	84.2	5.62	14	8	8	High	1	1.035	0.978	1.222	1.114	1.241	1.152	1.174	1.198	1.3	1.271	1.138	1.173	1.126	1.16
TRINITY_DN81031_c0_g1_i1_orf1	aldehyde dehydrogenase, partial [Ectropis obliqua]	5	79	8.6	8.46	57	37	5	High	1	0.95	0.946	1.89	2.052	1.849	2.446	2.527	2.342	2.491	2.971	2.853	2.673	2.819	2.789
TRINITY_DN6612_c0_g1_i4_orf1	hypothetical protein O3G_MSEX008151 [Manduca sexta]	8	389	42.7	7.75	24	14	8	High	1	0.992	1.001	1.261	1.284	1.327	1.039	1.026	1.14	0.891	0.852	0.905	0.616	0.58	0.609
TRINITY_DN2038_c0_g1_i2_orf1	tryptophan--tRNA ligase, cytoplasmic-like [Ostrinia furnacalis]	10	400	45.7	6.86	28	14	10	High	1	0.982	0.961	0.821	0.759	0.794	0.709	0.699	0.677	0.79	0.933	0.74	0.697	0.7	0.719
TRINITY_DN52316_c0_g1_i1_orf1	arylphorin subunit alpha-like [Ostrinia furnacalis]	7	113	13.9	5.4	55	25	4	High	1	0.967	1.011	3.005	2.299	2.589	3.324	3.121	4.164	5.461	5.399	3.567	4.327	4.074	4.596
TRINITY_DN3511_c0_g2_i1_orf1	eukaryotic translation initiation factor 2A [Ostrinia furnacalis]	10	570	63.7	9.29	18	11	10	High	1	0.972	0.979	0.895	0.906	0.93	0.843	0.8	0.855	0.753	0.755	0.76	0.768	0.749	0.723
TRINITY_DN2215_c0_g2_i1_orf1	PREDICTED: larval cuticle protein LCP-22-like [Amyelois transitella]	3	156	16.5	5.11	33	29	3	High	1	0.984	0.995	1.543	1.609	1.515	1.465	1.523	1.411	1.248	1.048	1.433	0.264	0.206	0.247
TRINITY_DN740_c0_g1_i1_orf1	GTP-binding nuclear protein Ran [Pieris rapae]	6	213	24.4	7.42	31	11	6	High	1	0.994	1.007	0.596	0.612	0.623	0.621	0.602	0.608	0.568	0.583	0.589	0.676	0.655	0.671
TRINITY_DN11231_c1_g1_i1_orfp1	TRINITY_DN11231_c1_g1_i1_m.13377 TRINITY_DN11231_c1_g1::TRINITY_DN11	4	75	7.6	5.87	89	13	4	High	1	1.001	1.023	1.948	2.025	1.915	1.988	1.895	1.953	1.564	1.401	1.522	1.166	1.177	1.186
TRINITY_DN8030_c0_g1_i2_orf1	chaoptin isoform X1 [Ostrinia furnacalis]	10	1321	150.1	6.6	9	11	10	High	1	0.952	0.987	1.488	1.439	1.351	1.751	1.645	1.778	1.632	1.768	1.605	1.108	1.031	1.086
TRINITY_DN3835_c0_g1_i3_orf1	protein ERGIC-53 isoform X1 [Ostrinia furnacalis]	5	441	49.4	5.85	21	9	4	High	1	0.992	0.975	0.651	0.612	0.676	0.64	0.624	0.646	0.558	0.594	0.572	0.735	0.752	0.802
TRINITY_DN54205_c0_g1_i1_orf1	aldo-keto reductase AKR2E4-like [Ostrinia furnacalis]	7	305	34.5	6.65	32	10	7	High	1	0.985	1.035	1.75	1.742	1.763	2.185	2.063	2.023	2.01	1.997	2.052	1.444	1.408	1.545
TRINITY_DN10792_c0_g2_i5_orf1	uncharacterized protein LOC114366171 [Ostrinia furnacalis]	9	418	48	5.87	20	11	9	High	1	1.011	0.968	0.137	0.135	0.201	0.194	0.202	0.204	0.209	0.209	0.196	0.207	0.191	0.197
TRINITY_DN6235_c0_g1_i5_orf1	rRNA 2'-O-methyltransferase fibrillarin [Vanessa cardui]	10	317	33.1	10.24	35	18	10	High	1	0.992	1.024	0.607	0.644	0.616	0.517	0.515	0.539	0.493	0.516	0.512	0.546	0.528	0.526
TRINITY_DN817_c0_g1_i3_orf1	phenylalanine--tRNA ligase beta subunit [Ostrinia furnacalis]	8	428	48.1	6.34	21	10	8	High	1	0.969	1.009	0.859	0.851	0.884	0.712	0.785	0.758	0.666	0.659	0.67	0.682	0.704	0.689
TRINITY_DN843_c0_g1_i5_orf1	fasciclin-3 isoform X4 [Helicoverpa zea]	10	494	54	5.45	27	13	2	High	1	0.923	0.918	1.01	1.049	1.068	1.002	1.123	1.069	0.969	0.753	0.902	1.28	1.227	1.309
TRINITY_DN25901_c0_g1_i2_orf1	short-chain specific acyl-CoA dehydrogenase, mitochondrial-like isoform X2 [(	10	406	43.9	8.46	27	12	10	High	1	1.008	1.017	0.633	0.66	0.651	0.848	0.794	0.809	0.663	0.62	0.658	0.55	0.601	0.56
TRINITY_DN2688_c0_g1_i3_orf1	mitochondrial amidoxime reducing component 2-like [Ostrinia furnacalis]	8	334	37.4	8.51	24	14	3	High	1	0.993	0.996	1.624	1.673	1.663	1.248	1.286	1.188	1.353	1.325	1.571	1.046	1.032	1.053
TRINITY_DN443_c0_g1_i2_orf1	proteasome subunit beta type-4 [Ostrinia furnacalis]	5	263	28.7	6.3	34	13	5	High	1	0.94	0.94	1.495	1.603	1.467	1.33	1.462	1.331	1.455	1.616	1.59	1.424	1.424	1.462
TRINITY_DN3428_c0_g1_i1_orf1	10 kDa heat shock protein, mitochondrial [Ostrinia furnacalis]	8	103	11.1	8.37	64	13	8	High	1	1.027	0.925	0.457	0.471	0.504	0.42	0.422	0.401	0.401	0.347	0.413	0.484	0.525	0.486
TRINITY_DN1914_c0_g1_i6_orf1	loricrin-like [Ostrinia furnacalis]	6	595	58.9	6.61	15	7	1	High	1	1.119	0.999	0.07	0.074	0.101	0.121	0.127	0.117	0.107	0.106	0.1	0.175	0.211	0.172
TRINITY_DN791_c0_g1_i2_orf1	peroxiredoxin 1 isoform X1 [Maniola jurtina]	8	233	26.5	6.68	32	16	7	High	1	0.987	0.914	0.421	0.379	0.455	0.399	0.359	0.413	0.386	0.36	0.321	0.454	0.455	0.462
TRINITY_DN6108_c0_g1_i5_orf1	myogenesis-regulating glycosidase-like [Ostrinia furnacalis]	11	579	66.3	5.22	20	11	11	High	1	1.019	1.032	1.767	1.738	1.824	1.498	1.69	1.612	2.511	2.282	2.357	1.649	1.797	1.706
TRINITY_DN51050_c0_g1_i3_orf1	GRB10-interacting GYF protein 2 isoform X1 [Ostrinia furnacalis]	10	1614	175.7	7.78	8	10	10	High	1	0.976	0.991	0.755	0.781	0.775	0.768	0.788	0.752	0.706	0.609	0.724	0.864	0.889	0.851
TRINITY_DN81488_c0_g1_i1_orf1	apolipophorins-like [Ostrinia furnacalis]	6	76	8.8	9.28	75	22	6	High	1	1.031	1.05	1.758	1.47	1.648	1.938	1.664	1.871	3.275	2.837	2.526	3.834	4.022	3.512
TRINITY_DN29555_c0_g1_i8_orf1	fasciclin-1 [Ostrinia furnacalis]	9	665	76	7.78	15	11	9	High	1	0.92	0.973	2.553	2.555	2.393	2.274	2.364	2.227	1.881	1.84	1.898	2.009	1.985	2.036
TRINITY_DN140_c0_g1_i5_orf1	calcyphosin-like protein isoform X4 [Helicoverpa armigera]	9	103	12	9.04	73	14	2	High	1	0.936	0.958	2.885	3.088	2.856	2.692	2.711	2.622	0.987	0.87	0.851	0.902	0.967	0.965
TRINITY_DN8241_c0_g1_i3_orf1	transforming growth factor beta-1-induced transcript 1 protein [Ostrinia furna	6	179	19.5	8.02	57	8	6	High	1	0.953	0.964	0.986	1.101	0.978	0.856	0.84	0.789	0.712	0.652	0.826	0.719	0.698	0.726
TRINITY_DN10512_c0_g1_i1_orf1	protein obstructor-E [Ostrinia furnacalis]	8	225	24.8	4.81	57	11	8	High	1	1.029	1.039	1.155	1.227	1.196	1.186	1.211	1.134	1.006	0.923	1.058	0.868	0.882	0.887

TRINITY_DN376_c0_g1_i1_orf1	thioredoxin reductase 1, mitochondrial isoform X4 [Helicoverpa zea]	8	494	53.6	6.76	28	10	8	High	1	0.974	0.984	0.74	0.721	0.747	0.779	0.751	0.795	0.862	0.834	0.811	0.874	0.903	0.903
TRINITY_DN2344_c1_g1_i4_orf1	aldo-keto reductase AKR2E4-like [Ostrinia furnacalis]	6	367	41.3	8.97	20	13	4	High	1	1.002	0.996	0.816	0.782	0.856	0.891	0.932	0.936	0.947	1.011	0.961	0.894	0.919	0.844
TRINITY_DN21357_c0_g1_i5_orf1	40S ribosomal protein S11 isoform X2 [Ostrinia furnacalis]	9	152	17.6	10.49	54	15	9	High	1	0.958	0.959	0.607	0.624	0.637	0.589	0.577	0.564	0.455	0.431	0.504	0.49	0.493	0.488
TRINITY_DN8133_c0_g1_i4_orf1	protein sel-1 homolog 1 isoform X2 [Ostrinia furnacalis]	8	743	81.1	5.99	12	8	8	High	1	0.99	0.937	0.882	0.916	0.969	0.9	0.9	0.884	0.816	0.748	0.824	0.966	0.964	0.98
TRINITY_DN20339_c0_g1_i3_orf1	ecto-NOX disulfide-thiol exchanger 2-like [Ostrinia furnacalis]	9	634	72	7.81	16	9	9	High	1	0.957	1.01	0.785	0.835	0.815	0.667	0.707	0.703	0.65	0.679	0.652	0.904	0.931	0.892
TRINITY_DN1520_c0_g1_i9_orf1	adipocyte plasma membrane-associated protein-like [Ostrinia furnacalis]	11	425	46.2	9.58	28	11	11	High	1	0.965	0.992	0.995	0.976	0.998	1.011	0.948	0.967	0.808	0.764	0.798	0.998	1.037	1.001
TRINITY_DN7241_c0_g2_i2_orf1	40S ribosomal protein S10 [Zerene cesonia]	8	161	18.4	9.99	36	22	8	High	1	1.033	1.053	0.755	0.786	0.743	0.613	0.621	0.604	0.575	0.501	0.621	0.589	0.606	0.566
TRINITY_DN13322_c0_g1_i6_orf1	macrophage mannose receptor 1-like [Ostrinia furnacalis]	6	319	36.3	5.21	25	8	6	High	1	0.945	1.003	1.778	1.703	1.709	2.069	1.951	1.887	1.96	1.725	2.169	1.364	1.381	1.372
TRINITY_DN34536_c0_g1_i6_orf1	clustered mitochondria protein homolog isoform X2 [Ostrinia furnacalis]	9	1129	124.7	6.34	8	12	9	High	1	1.035	1.037	0.3	0.353	0.35	0.349	0.355	0.325	0.306	0.297	0.327	0.37	0.354	0.379
TRINITY_DN21124_c0_g1_i4_orf1	calsyntenin-1 [Ostrinia furnacalis]	10	859	95.1	6.39	14	10	10	High	1	1.002	0.933	0.497	0.517	0.559	0.542	0.579	0.54	0.526	0.569	0.519	1.411	1.481	1.425
TRINITY_DN2507_c0_g1_i7_orf1	uncharacterized protein LOC114357507 isoform X1 [Ostrinia furnacalis]	6	364	38.6	6.29	20	8	6	High	1	0.896	0.956	1.05	1.084	1.079	1.113	1.073	1.05	1.034	1.048	0.995	0.994	0.93	0.957
TRINITY_DN3312_c0_g1_i10_orf1	glycerol-3-phosphate dehydrogenase, mitochondrial-like isoform X3 [Ostrinia	8	720	80.2	8.19	16	10	8	High	1	0.995	0.979	0.555	0.605	0.612	0.496	0.545	0.537	0.601	0.612	0.615	0.573	0.58	0.604
TRINITY_DN4314_c0_g1_i9_orf1	serine proteinase inhibitor 2 [Ostrinia furnacalis]	10	381	41.8	5.53	27	10	10	High	1	1.002	0.987	1.612	1.53	1.494	1.729	1.669	1.706	1.937	1.789	1.855	0.981	1.002	1.007
TRINITY_DN1593_c0_g1_i1_orf1	chemosensory protein csp11 [Helopeltis theivora]	8	137	15.4	7.02	39	24	8	High	1	1.003	0.968	3.177	3.429	3.228	2.2	2.298	2.175	2.257	2.014	2.581	1.048	1.072	1.09
TRINITY_DN53294_c0_g1_i1_orf1	liver carboxylesterase 2-like [Ostrinia furnacalis]	9	664	72.2	7.11	16	11	9	High	1	0.983	0.952	1.124	1.05	1.036	1.077	1.126	1.065	1.536	1.445	1.479	1.328	1.278	1.291
TRINITY_DN493_c0_g1_i4_orf1	ADP-ribosylation factor GTPase-activating protein 3 [Ostrinia furnacalis]	7	499	54.5	6.81	15	9	7	High	1	1.042	1.043	0.797	0.795	0.778	0.703	0.705	0.701	0.684	0.621	0.671	0.878	0.852	0.828
TRINITY_DN2953_c1_g1_i11_orf1	methionine--tRNA ligase, cytoplasmic isoform X2 [Ostrinia furnacalis]	10	887	98.9	7.37	14	10	7	High	1	0.995	1.043	0.737	0.721	0.725	0.699	0.648	0.766	0.812	0.743	0.729	0.836	0.856	0.862
TRINITY_DN351_c14_g1_i2_orf1	calnexin [Ostrinia furnacalis]	10	576	64.6	4.86	15	18	10	High	1	1.003	0.984	0.941	0.98	0.962	0.877	0.891	0.852	0.768	0.715	0.792	0.888	0.906	0.912
TRINITY_DN48610_c0_g1_i2_orf1	hypothetical protein evm_002298 [Chilo suppressalis]	5	174	18.6	4.65	41	10	5	High	1	0.994	1.009	1.26	1.391	1.259	1.025	1.109	1.039	0.897	0.865	1.029	0.435	0.413	0.404
TRINITY_DN2894_c0_g1_i2_orf1	myrosinase 1-like isoform X1 [Ostrinia furnacalis]	7	514	59.3	5.24	17	10	6	High	1	1.042	0.968	0.189	0.196	0.253	0.196	0.225	0.184	0.202	0.2	0.195	0.205	0.22	0.21
TRINITY_DN26168_c0_g1_i1_orf1	ATP-dependent RNA helicase Ddx1-like [Ostrinia furnacalis]	7	728	81.2	6.93	14	8	7	High	1	0.995	1.065	0.774	0.772	0.756	0.745	0.728	0.753	0.68	0.682	0.695	0.871	0.896	0.917
TRINITY_DN4199_c0_g1_i1_orf1	plasminogen activator inhibitor 1 RNA-binding protein-like [Ostrinia furnacalis	11	351	39.3	9.26	29	20	11	High	1	1.005	0.973	0.774	0.815	0.786	0.787	0.816	0.81	0.815	0.827	0.837	0.83	0.815	0.817
TRINITY_DN85319_c0_g1_i1_orf1	cholinesterase 2-like [Ostrinia furnacalis]	9	542	61.4	4.84	18	11	9	High	1	1.025	0.977	0.378	0.357	0.423	0.393	0.388	0.382	0.343	0.319	0.321	0.388	0.394	0.387
TRINITY_DN26818_c0_g1_i1_orf1	PREDICTED: malignant T-cell-amplified sequence 1 homolog [Amyelois transit	5	182	20.5	9	45	8	5	High	1	0.973	0.991	0.93	0.881	0.989	0.976	1.012	0.986	1.02	1.016	0.945	0.818	0.888	0.893
TRINITY_DN843_c0_g1_i2_orf1	unnamed protein product [Diatraea saccharalis]	10	444	48.6	5.45	32	13	2	High	1	1.072	1.161	1.908	1.868	1.445	1.038	1.008	0.914	1.073	1.071	1.235	2.877	2.506	2.338
TRINITY_DN31232_c1_g1_i9_orf1	PREDICTED: elongation factor 1-alpha 1, partial [Haliaeetus albicilla]	6	412	45.4	8.95	14	23	1	High	1	1.182	1.052	0.504	0.469	0.577	0.474	0.574	0.372	0.66	0.534	0.548	0.432	0.392	0.573
TRINITY_DN16349_c0_g1_i10_orf1	protein lingerer-like isoform X1 [Nymphalis io]	5	683	69.3	8.03	14	8	5	High	1	1.012	1.027	0.521	0.519	0.589	0.494	0.552	0.474	0.434	0.4	0.472	0.79	0.765	0.804
TRINITY_DN27771_c0_g1_i1_orf1	glycine--tRNA ligase [Ostrinia furnacalis]	8	533	60	8.16	17	12	8	High	1	0.991	0.964	0.898	0.852	0.882	0.744	0.745	0.786	0.686	0.716	0.665	0.838	0.913	0.873
TRINITY_DN1465_c2_g1_i2_orf1	transcription initiation factor TFIID subunit 1-like [Ostrinia furnacalis]	7	316	36.9	8.19	26	11	7	High	1	0.935	0.991	1.33	1.411	1.334	1.687	1.768	1.756	1.378	1.23	1.334	3.243	3.409	3.126
TRINITY_DN43505_c0_g1_i1_orf1	unnamed protein product [Chilo suppressalis]	7	462	51.4	5.31	27	9	7	High	1	0.986	1.031	1.315	1.347	1.283	1.319	1.381	1.346	1.316	1.603	1.352	1.37	1.372	1.41
TRINITY_DN2922_c0_g1_i1_orf1	uncharacterized protein LOC114354086 [Ostrinia furnacalis]	7	314	33.7	7.08	34	8	7	High	1	0.975	1.008	1.008	1.088	1.011	1.138	1.127	1.05	1.007	0.946	1.063	0.668	0.689	0.654
TRINITY_DN7776_c0_g1_i5_orf1	uncharacterized protein LOC114364702 [Ostrinia furnacalis]	8	1027	116.1	5.22	9	8	7	High	1	1.018	1	1.248	1.402	1.438	1.466	1.55	1.475	1.369	1.184	1.363	1.565	1.591	1.584
TRINITY_DN8367_c0_g2_i2_orf1	uncharacterized protein LOC114357075 [Ostrinia furnacalis]	6	262	27.9	8.06	31	7	3	High	1	1.126	1.05	2.138	2.027	2.12	2.034	2.156	2.114	1.729	1.755	2.096	1.825	1.849	1.968
TRINITY_DN19460_c0_g1_i1_orf1	cuticle protein 3-like [Ostrinia furnacalis]	5	181	19.7	5.12	40	10	5	High	1	0.933	0.971	1.017	1.138	1.089	1.113	1.132	1.078	1.021	0.97	1.115	0.742	0.711	0.679
TRINITY_DN1515_c0_g1_i2_orf1	unnamed protein product [Diatraea saccharalis]	10	1254	141.4	5.48	10	10	10	High	1	0.995	1.03	0.891	0.867	0.913	0.865	0.966	0.959	0.774	0.89	0.773	0.818	0.838	0.833
TRINITY_DN19923_c0_g1_i1_orf1	uncharacterized protein LOC114350958 [Ostrinia furnacalis]	7	146	16.2	4.78	71	14	7	High	1	1.004	1.025	0.801	0.78	0.791	0.833	0.827	0.79	0.888	0.738	0.863	2.055	2.136	1.887
TRINITY_DN642_c0_g1_i6_orf1	reticulon-3-B isoform X5 [Ostrinia furnacalis]	7	214	23.2	9.23	33	10	1	High	1	1.096	1.031	0.74	0.718	0.716	0.637	0.608	0.706	0.608	0.58	0.498	0.564	0.536	0.634
TRINITY_DN130575_c0_g1_i1_orfp	TRINITY_DN130575_c0_g1_i1_m.77798 TRINITY_DN130575_c0_g1::TRINITY_DN	5	69	6.7	6.6	74	18	2	High	1	0.982	0.999	2.471	2.645	2.421	2.666	2.731	2.557	2.148	2.048	2.321	1.702	1.717	1.704
TRINITY_DN15858_c0_g1_i2_orf1	15-hydroxyprostaglandin dehydrogenase [NAD(+)]-like [Ostrinia furnacalis]	5	277	30.1	7.43	30	6	3	High	1	0.975	1.03	2.367	2.285	2.413	1.834	1.854	1.94	2.121	2.203	1.963	1.275	1.217	1.392
TRINITY_DN9717_c0_g2_i1_orf1	proteasome subunit beta type-2 [Ostrinia furnacalis]	7	205	23.2	6.9	29	9	7	High	1	1.001	0.965	1.214	1.277	1.166	1.191	1.233	1.174	1.249	1.295	1.158	1.257	1.192	1.302
TRINITY_DN2043_c0_g1_i11_orf1	phenoloxidase-activating factor 2-like [Ostrinia furnacalis]	7	372	39.8	7.24	21	11	7	High	1	0.95	1.021	1.314	1.376	1.291	1.117	1.101	1.111	1.222	1.086	1.29	1.552	1.635	1.519
TRINITY_DN17406_c0_g1_i1_orf1	uncharacterized protein LOC114356625 [Ostrinia furnacalis]	6	190	20.5	7.2	61	6	6	High	1	1.035	1.023	0.978	0.949	0.854	0.892	0.774	0.892	1.066	1.018	1.082	1.306	1.267	1.161
TRINITY_DN8471_c0_g1_i6_orf1	titin homolog isoform X4 [Ostrinia furnacalis]	9	837	96.1	8.32	12	13	9	High	1	1.026	1.035	1.233	1.165	1.171	1.123	1.346	1.08	1.155	1.162	1.222	0.753	0.808	1.425
TRINITY_DN5354_c0_g1_i4_orf1	NADP-dependent malic enzyme-like [Ostrinia furnacalis]	10	620	68.8	7.05	19	11	10	High	1	0.961	0.96	0.645	0.551	0.653	0.688	0.658	0.713	0.955	0.814	0.715	0.562	0.554	0.584
TRINITY_DN38667_c0_g1_i9_orf1	signal transducer and activator of transcription 5B-like [Melitaea cinxia]	9	771	88.4	6.14	14	10	9	High	1	1.01	1.026	0.886	0.855	0.895	0.787	0.765	0.788	0.817	0.85	0.756	0.989	0.936	0.941
TRINITY_DN143497_c0_g1_i1_orf1	fibroin heavy chain-like [Ostrinia furnacalis]	7	200	19.4	9.82	39	9	7	High	1	0.969	0.998	0.73	0.826	0.797	0.767	0.866	0.801	0.723	0.725	0.739	3.52	3.574	3.43
TRINITY_DN14934_c0_g1_i17_orf1	putative tricarboxylate transport protein, mitochondrial isoform X1 [Ostrinia fu	8	317	34.4	9.63	23	14	8	High	1	0.998	0.999	0.641	0.569	0.634	0.669	0.656	0.68	0.647	0.613	0.582	0.531	0.504	0.52
TRINITY_DN8116_c0_g1_i2_orf1	uncharacterized protein LOC114350845 [Ostrinia furnacalis]	8	251	26.1	8.73	37	12	6	High	1	1.003	0.989	0.176	0.157	0.185	0.229	0.228	0.215	0.189	0.177	0.202	0.206	0.198	0.202
TRINITY_DN21218_c0_g1_i4_orf1	leukotriene A-4 hydrolase isoform X2 [Ostrinia furnacalis]	6	417	46.2	6.04	17	6	6	High	1	0.991	1.015	0.631	0.634	0.636	0.608	0.645	0.642	0.684	0.753	0.667	0.888	0.854	0.908
TRINITY_DN18222_c0_g1_i4_orf1	phosphoglycerate kinase [Manduca sexta]	7	263	27.8	5.69	33	16	1	High	1	0.998	1.048	1.388	1.269	1.025	1.196	0.922	1.187	1.594	1.122	1.769	1.356	1.415	1.178
TRINITY_DN5417_c0_g1_i1_orf1	NADH dehydrogenase [ubiquinone] 1 alpha subcomplex subunit 10, mitochon	9	399	45.9	6.96	22	12	9	High	1	0.98	0.999	0.459	0.486	0.492	0.446	0.451	0.44	0.43	0.445	0.456	0.477	0.442	0.465
TRINITY_DN4451_c0_g1_i1_orf1	uncharacterized protein LOC114361986 isoform X1 [Ostrinia furnacalis]	4	193	20.6	7.84	39	6	3	High	1	1.032	1.077	0.445	0.457	0.432	0.453	0.44	0.444	0.546	0.76	0.525	0.477	0.489	0.461
TRINITY_DN3732_c0_g1_i2_orf1	cytochrome P450 monooxygenase CYP6AB141 [Ostrinia furnacalis]	10	515	59.3	8.91	21	11	7	High	1	0.96	0.942	0.708	0.678	0.674	1.05	1.054	1.091	0.714	0.678	0.744	0.804	0.797	0.789
TRINITY_DN2255_c0_g1_i1_orf1	glutathione S-transferase sigma 3 [Ostrinia furnacalis]	10	201	23	8.16	41	16	9	High	1	0.96	0.991	1.723	1.525	1.675	1.903	1.818	1.921	1.937	1.871	1.604	1.463	1.45	1.54
TRINITY_DN7291_c0_g1_i5_orf1	dynamin-1-like protein isoform X3 [Ostrinia furnacalis]	8	690	77.5	7.27	14	11	1	High	1	1.141	1.216	0.91	1.293	1.053	1.347	1.321	1.326	0.972	0.766	0.802	0.911	0.985	0.902
TRINITY_DN32687_c0_g1_i1_orf1	protein D2-like isoform X2 [Ostrinia furnacalis]	4	105	11.6	6.54	66	8	1	High	1	1.028	1.203	0.828	1.014	0.915	1.195	1.078	1.074	0.85	1.135	0.775	0.928	0.929	0.932
TRINITY_DN625_c2_g2_i2_orf1	L-dopachrome tautomerase yellow-f2-like [Ostrinia furnacalis]	7	401	45.4	6.27	23	10	6	High	1	0.988	1.049	1.538	1.516	1.427	1.633	1.665	1.626	1.882	2.33	1.975	1.7	1.623	1.849
TRINITY_DN4394_c0_g1_i4_orf1	B-cell receptor-associated protein 31 [Ostrinia furnacalis]	7	224	25.5	8.76	22	16	7	High	1	0.989	0.985	1.686	1.796	1.667	1.527	1.601	1.465	1.585	1.337	1.797	1.304	1.377	1.337
TRINITY_DN2835_c0_g1_i6_orf1	probable isoaspartyl peptidase/L-asparaginase GA20639 [Ostrinia furnacalis]	9	313	32.4	6.46	37	12	9	High	1	1.018	1.059	1.385	1.356	1.391	1.715	1.752	1.706	1.933	1.921	2.035	2.425	2.419	2.396
TRINITY_DN3532_c0_g1_i12_orf1	sulfide:quinone oxidoreductase, mitochondrial-like [Ostrinia furnacalis]	9	440	49.4	8.9	21	10	9	High	1	1.01	0.962	0.973	0.956	0.955	0.999	0.967	1.031	0.897	0.88	0.947	0.827	0.778	0.8
TRINITY_DN101_c0_g2_i2_orf1	tyrosine-protein phosphatase non-receptor type 23 [Ostrinia furnacalis]	9	1913	212.3	5.33	7	9	9	High	1	1.029	1.032	1.085	1.09	1.009	1.001	0.987	1.046	1.012	1.207	0.881	1.252	1.244	1.205
TRINITY_DN6522_c0_g1_i6_orf1	proteasome subunit alpha type-2 [Ostrinia furnacalis]	4	234	25.7	6.54	28	7	4	High	1	0.978	0.933	0.962	0.919	0.971	0.96	0.966	0.997	0.973	1.044	0.912	0.888	0.838	0.9
TRINITY_DN1722_c0_g1_i2_orf1	delta(3,5)-Delta(2,4)-dienoyl-CoA isomerase, mitochondrial isoform X1 [Ostrin	8	294	32.4	7.05	30	10	5	High	1	1.043	1.023	1.243	1.219	1.193	1.191	1.227	1.249	1.235	1.147	1.226	1.325	1.38	1.425
TRINITY_DN6103_c0_g1_i6_orf1	sedoheptulokinase-like [Ostrinia furnacalis]	8	459	50.4	6.79	22	9	8	High	1	0.998	0.971	0.752	0.724	0.78	0.785	0.736	0.77	0.728	0.685	0.697	0.885	0.861	0.853
TRINITY_DN1960_c5_g1_i3_orf1	cytochrome P450 monooxygenase CYP9G18 [Cnaphalocrocis medinalis]	6	373	43.4	6.35	21	9	3	High	1	1.043	1.109	1.85	1.8	1.802	2.211	2.133	2.296	2.365	2.197	2.192	1.475	1.384	1.469
TRINITY_DN2171_c0_g1_i1_orf1	probable pterin-4-alpha-carbinolamine dehydratase isoform X1 [Ostrinia furn	6	147	16.5	9.6	41	19	6	High	1	1.036	1.005	1.742	1.665	1.692	1.809	1.769	1.846	1.777	1.543	1.571	2.057	2.06	2.014
TRINITY_DN3332_c0_g1_i2_orf1	glutathione S-transferase sigma3 [Glyphodes pyloalis]	8	180	20.6	5.15	50	20	3	High	1	1.032	1.015	0.245	0.256	0.334	0.313	0.317	0.311	0.291	0.346	0.293	0.321	0.318	0.357
TRINITY_DN7613_c1_g2_i1_orf1	60S ribosomal protein L19 [Helicoverpa armigera]	8	200	23.6	11.22	30	29	8	High	1	0.979	0.97	0.759	0.796	0.784	0.721	0.715	0.727	0.643	0.611	0.666	0.648	0.649	0.657
TRINITY_DN1149_c0_g1_i4_orf1	circadian clock-controlled protein-like [Ostrinia furnacalis]	7	247	27.4	7.33	35	14	7	High	1	0.97	1.102	4.797	4.767	4.589	2.682	2.853	2.713	3.47	3.234	3.696	1.479	1.415	1.483
TRINITY_DN152_c0_g1_i4_orf1	LOW QUALITY PROTEIN: protein tyrosine phosphatase type IVA 3 [Ostrinia furr	5	171	19.4	8.53	28	9	5	High	1	0.972	0.982	0.893	0.945	0.892	0.739	0.78	0.763	0.618	0.613	0.722	0.854	0.852	0.865
TRINITY_DN94248_c0_g2_i3_orf1	uncharacterized protein LOC114357292 isoform X4 [Ostrinia furnacalis]	7	268	28.7	8.68	29	12	7	High	1	0.968	0.965	0.258	0.268	0.301	0.305	0.341	0.329	0.332	0.342	0.355	0.281	0.253	0.279
TRINITY_DN2318_c1_g1_i1_orf1	transcription factor SPT20 homolog [Ostrinia furnacalis]	7	487	54.7	9.23	20	9	7	High	1	1.013	1.023	0.889	0.946	0.992	0.803	0.828	0.812	0.809	0.76	0.856	0.631	0.647	0.643
TRINITY_DN7405_c0_g1_i3_orf1	hexokinase-2-like [Ostrinia furnacalis]	8	501	54.1	6.07	18	10	8	High	1	1.005	0.982	0.499	0.554	0.555	0.594	0.626	0.629	0.554	0.738	0.568	0.548	0.519	0.544
TRINITY_DN12545_c0_g1_i7_orf1	hypothetical protein evm_003491 [Chilo suppressalis]	7	635	71.1	6.61	14	8	7	High	1	1.052	1.135	2.041	1.662	1.914	1.776	1.575	1.809	1.636	1.469	1.34	1.061	0.994	1.104
TRINITY_DN8659_c0_g2_i1_orf1	ubiquitin-like modifier-activating enzyme 1 [Ostrinia furnacalis]	7	437	49.2	5.96	17	13	4	High	1	0.929	0.965	1.038	1.051	1.016	0.828	0.818	0.853	1.026	1.016	0.965	1.408	1.361	1.41
TRINITY_DN14313_c0_g1_i1_orf1	25S rRNA (cytosine-C(5))-methyltransferase nop2 [Ostrinia furnacalis]	9	848	95.6	9.06	12	10	9	High	1	0.979	0.932	0.384	0.402	0.405	0.387	0.406	0.376	0.376	0.374	0.362	0.518	0.467	0.453
TRINITY_DN3551_c0_g1_i4_orf1	carnitine O-palmitoyltransferase 2, mitochondrial [Ostrinia furnacalis]	7	653	74.7	8.68	15	9	7	High	1	0.988	0.97	0.656	0.597	0.632	0.585	0.642	0.6	0.602	0.676	0.612	0.623	0.583	0.556
TRINITY_DN17351_c0_g1_i3_orf1	V-type proton ATPase subunit F [Ostrinia furnacalis]	6	124	13.8	6.18	40	11	6	High	1	1.021	0.993	0.305	0.312	0.351	0.34	0.401	0.33	0.324	0.313	0.352	0.296	0.292	0.301
TRINITY_DN23167_c0_g1_i4_orf1	uncharacterized protein LOC114363065 [Ostrinia furnacalis]	7	687	69.4	6.25	14	8	7	High	1	1.022	0.986	0.807	0.637	0.673	0.538	0.486	0.464	0.45	0.369	0.464	0.314	0.336	0.32
TRINITY_DN5563_c1_g2_i2_orf1	hypothetical protein evm_005765 [Chilo suppressalis]	5	617	68.8	5.76	13	9	5	High	1	1.005	0.955	0.967	1.034	0.951	0.979	0.929	0.905	0.924	0.808	0.957	0.817	0.828	0.767
TRINITY_DN2394_c0_g1_i4_orf1	uncharacterized protein LOC114363116 [Ostrinia furnacalis]	6	166	18.8	8	33	9	6	High	1	1.241	1.161	0.075	0.07	0.107	0.116	0.127	0.103	0.109	0.098	0.117	0.113	0.101	0.109
TRINITY_DN14855_c0_g1_i1_orf1	neurochondrin homolog [Ostrinia furnacalis]	7	739	83.1	5.76	12	9	7	High	1	0.99	1	0.958	0.865	0.977	0.843	0.847	0.873	0.738	0.74	0.658	0.741	0.768	0.76
TRINITY_DN87648_c0_g1_i1_orfp1	TRINITY_DN87648_c0_g1_i1_m.51054 TRINITY_DN87648_c0_g1::TRINITY_DN87	7	179	18	4.23	37	12	7	High	1	1.064	1.011	0.836	0.889	0.882	1.108	1.066	1.043	0.95	0.826	0.962	0.524	0.547	0.535
TRINITY_DN19678_c0_g1_i1_orf1	phosphatidylinositide phosphatase SAC1 [Ostrinia furnacalis]	7	503	57.3	9.31	15	7	6	High	1	1.053	1.038	0.946	0.956	0.937	1.015	0.951	0.968	0.922	0.921	0.868	0.972	0.981	1.073
TRINITY_DN3256_c0_g1_i3_orf1	hypothetical protein evm_007599 [Chilo suppressalis]	7	404	45.6	7.61	21	9	6	High	1	0.964	0.986	1.073	0.996	1.061	1.081	1.083	1.135	1.059	1.09	0.944	1.171	1.09	1.178
TRINITY_DN2461_c0_g1_i5_orf1	secretory phospholipase A2 receptor [Vanessa cardui]	7	283	31.9	5.1	30	13	7	High	1	1.001	1.001	1.965	1.987	2.012	2.119	2.28	2.24	1.957	2.067	2.021	1.184	1.127	1.162
TRINITY_DN2600_c0_g1_i7_orf1	mucin-5AC isoform X2 [Ostrinia furnacalis]	9	912	98	8.44	12	10	9	High	1	1.001	1	0.377	0.466	0.433	0.427	0.455	0.432	0.358	0.351	0.387	1.096	1.145	1
TRINITY_DN51342_c0_g1_i7_orf1	prophenoloxidase [Ostrinia furnacalis]	6	309	36.2	6.9	32	9	6	High	1	1.102	1.022	1	1.023	1.03	1.071	1	1.029	1.21	1.151	1.322	0.912	0.992	0.935
TRINITY_DN2489_c0_g1_i1_orf1	uncharacterized protein LOC114354692 [Ostrinia furnacalis]	5	305	31.8	5.85	24	7	5	High	1	0.977	1.028	1.254	1.208	1.246	1.276	1.269	1.427	1.468	1.456	1.29	3.422	3.296	3.402
TRINITY_DN27136_c0_g3_i1_orf1	WD repeat and FYVE domain-containing protein 2 isoform X1 [Ostrinia furnaca	7	418	46.5	7.64	24	7	7	High	1	0.953	0.95	1.107	1.094	1.126	1.104	1.108	1.042	0.964	1.134	0.981	1.126	1.167	1.105
TRINITY_DN2783_c1_g1_i2_orf1	proline-rich extensin-like protein EPR1 isoform X1 [Ostrinia furnacalis]	9	986	106.7	8.69	14	9	9	High	1	1.002	0.983	0.655	0.669	0.662	0.655	0.705	0.655	0.708	0.67	0.664	0.829	0.773	0.803
TRINITY_DN11108_c0_g1_i4_orf1	peroxisomal leader peptide-processing protease [Ostrinia furnacalis]	6	527	58.1	7.74	17	7	6	High	1	0.977	0.927	2.266	2.083	1.985	1.695	1.756	1.78	1.469	1.39	1.428	1.183	1.257	1.244
TRINITY_DN2818_c0_g1_i2_orf1	proteasome subunit alpha type-3 [Ostrinia furnacalis]	8	255	28.1	5.29	30	12	8	High	1	0.989	0.992	1.397	1.32	1.333	1.234	1.196	1.276	1.283	1.09	1.188	1.34	1.443	1.309
TRINITY_DN2266_c0_g1_i6_orf1	bilin-binding protein-like [Ostrinia furnacalis]	6	205	22.1	5.68	31	10	6	High	1	1.007	0.944	0.337	0.336	0.378	0.346	0.327	0.326	0.616	0.536	0.619	0.356	0.386	0.368

TRINITY_DN1759_c0_g1_i4_orf1	protein PFC0760c-like isoform X1 [Ostrinia furnacalis]	11	890	100.6	4.22	12	13	9	High	1	0.998	1.017	2.039	2.144	2.055	2.202	2.232	2.163	1.452	1.249	1.496	1.879	1.907	1.808
TRINITY_DN2323_c0_g1_i4_orf1	uncharacterized protein LOC114364097 isoform X2 [Ostrinia furnacalis]	8	490	53.5	5	20	10	8	High	1	1.008	1.02	0.843	0.94	0.901	0.886	1.066	0.939	1.052	0.938	1.039	1.75	1.902	1.808
TRINITY_DN15706_c0_g2_i5_orf1	cdc42 homolog [Galleria mellonella]	4	191	21.3	6.55	25	8	3	High	1	1.013	1.012	0.568	0.544	0.542	0.61	0.619	0.56	0.557	0.496	0.579	0.706	0.755	0.721
TRINITY_DN32687_c0_g1_i2_orf1	protein D2-like isoform X2 [Ostrinia furnacalis]	4	105	11.6	6.54	66	12	1	High	1	1.051	1.123	1.264	1.285	1.254	0.567	0.563	0.66	0.717	0.618	0.568	0.575	0.587	0.543
TRINITY_DN19098_c0_g1_i4_orf1	L-xylulose reductase-like [Ostrinia furnacalis]	8	244	26.2	7.02	35	11	8	High	1	1.023	1.054	1.287	1.263	1.306	1.672	1.567	1.598	1.258	1.229	1.21	1.068	1.13	1.064
TRINITY_DN79657_c0_g1_i1_orf1	uncharacterized protein LOC114349955 [Ostrinia furnacalis]	6	299	31.7	10.81	24	8	6	High	1	0.994	1.008	1.912	1.829	1.742	1.9	1.978	1.837	1.611	1.752	1.657	1.823	1.724	1.784
TRINITY_DN38431_c0_g1_i1_orf1	neprilysin-2 isoform X1 [Ostrinia furnacalis]	10	772	87.5	6.65	16	10	10	High	1	0.978	1.015	0.975	0.985	1.006	1.067	1.05	1.04	0.959	1.095	0.953	2.574	2.472	2.48
TRINITY_DN66822_c0_g1_i1_orf1	heterogeneous nuclear ribonucleoprotein 27C isoform X6 [Pieris rapae]	6	279	29.9	7.94	30	13	6	High	1	1.02	1.003	0.632	0.659	0.686	0.612	0.676	0.657	0.572	0.643	0.565	0.673	0.669	0.686
TRINITY_DN5092_c0_g1_i2_orf1	peroxisomal acyl-coenzyme A oxidase 3 isoform X3 [Ostrinia furnacalis]	8	642	72	7.8	15	11	8	High	1	1.057	1.045	0.741	0.754	0.765	0.7	0.808	0.787	0.694	0.684	0.701	0.736	0.721	0.728
TRINITY_DN4891_c0_g1_i4_orf1	xaa-Pro aminopeptidase ApepP-like isoform X1 [Ostrinia furnacalis]	8	665	73.8	6.4	14	9	8	High	1	0.999	1.006	0.749	0.807	0.828	0.849	0.931	0.842	0.883	0.89	0.876	0.933	0.974	0.964
TRINITY_DN9558_c0_g1_i2_orf1	NADH dehydrogenase [ubiquinone] iron-sulfur protein 4, mitochondrial-like [(	5	183	20.8	9.26	48	7	5	High	1	0.999	1.004	0.521	0.55	0.597	0.577	0.549	0.594	0.585	0.871	0.568	0.587	0.591	0.545
TRINITY_DN8046_c0_g1_i4_orf1	tumor protein D54 isoform X3 [Hyposmocoma kahamanoa]	6	210	22.6	5.34	36	9	2	High	1	0.877	1.011	1.139	0.992	1.053	0.821	0.859	0.945	1.058	1.132	0.888	0.986	0.937	0.866
TRINITY_DN1673_c0_g1_i2_orf1	hypothetical protein evm_006080 [Chilo suppressalis]	9	1157	128.5	7.01	10	11	9	High	1	1.028	1.016	0.81	0.861	0.835	0.76	0.822	0.78	0.736	0.8	0.751	0.834	0.838	0.912
TRINITY_DN6199_c2_g1_i3_orf1	uncharacterized protein LOC114352137 [Ostrinia furnacalis]	8	168	19	5.67	65	10	8	High	1	1.073	1.006	1.13	1.111	1.119	1.415	1.401	1.348	1.564	1.412	1.584	1.575	1.67	1.513
TRINITY_DN38075_c0_g1_i1_orf1	60S ribosomal protein L26 [Ostrinia furnacalis]	7	148	17.1	10.7	49	13	7	High	1	0.899	0.959	0.642	0.664	0.639	0.585	0.587	0.597	0.508	0.556	0.565	0.594	0.57	0.613
TRINITY_DN2026_c0_g1_i4_orf1	60S ribosomal protein L35a [Ostrinia furnacalis]	8	159	17.4	11.55	44	18	8	High	1	0.903	0.927	0.619	0.726	0.644	0.581	0.622	0.607	0.523	0.729	0.583	0.54	0.551	0.563
TRINITY_DN40126_c0_g2_i1_orf1	aldehyde dehydrogenase X, mitochondrial-like [Ostrinia furnacalis]	6	143	15.4	7.96	62	10	3	High	1	1.288	1.176	2.472	2.763	2.515	2.28	2.361	2.281	2.133	2.12	2.178	1.443	1.638	1.576
TRINITY_DN4235_c0_g1_i2_orf1	uncharacterized protein LOC114361536 [Ostrinia furnacalis]	6	394	43.7	6.65	16	10	5	High	1	1.053	1.071	2.565	2.317	2.365	2.474	2.368	2.529	2.908	2.887	2.685	2.359	2.174	2.344
TRINITY_DN45973_c0_g1_i4_orf1	6-phosphogluconolactonase [Ostrinia furnacalis]	8	233	25.6	6.9	38	15	8	High	1	0.997	0.997	0.856	0.888	0.907	0.896	0.987	0.91	0.854	0.811	0.896	0.932	0.955	0.978
TRINITY_DN36460_c0_g1_i2_orf1	N-acetylneuraminate lyase-like [Ostrinia furnacalis]	8	299	32.3	7.33	33	15	6	High	1	1.001	0.992	1.299	1.372	1.307	1.253	1.311	1.25	1.237	1.33	1.153	0.978	1.001	1.038
TRINITY_DN14398_c0_g1_i4_orf1	trimethyllysine dioxygenase, mitochondrial [Ostrinia furnacalis]	7	376	43	5.81	22	8	7	High	1	0.973	0.992	1.109	1.058	1.11	1.062	1.042	1.07	1.026	1.109	0.934	0.811	0.82	0.812
TRINITY_DN23349_c0_g1_i9_orf1	COP9 signalosome complex subunit 4 isoform X1 [Ostrinia furnacalis]	10	409	47.3	5.53	26	15	10	High	1	0.963	1.02	0.906	0.892	0.923	0.969	0.926	0.975	0.889	0.99	0.875	1.051	0.995	0.977
TRINITY_DN31163_c1_g1_i4_orf1	phenoloxidase subunit 2-like [Ostrinia furnacalis]	4	112	12.6	4.55	63	7	4	High	1	0.957	0.995	1.204	1.306	1.289	1.242	1.244	1.214	1.488	1.696	1.545	1.045	1.082	1.026
TRINITY_DN51830_c0_g1_i4_orf1	15-hydroxyprostaglandin dehydrogenase [NAD(+)]-like [Ostrinia furnacalis]	7	279	30.2	6.95	33	8	7	High	1	0.955	1.004	0.812	0.809	0.792	1.035	1.021	1.038	0.882	1.004	0.9	0.63	0.587	0.649
TRINITY_DN206_c0_g1_i11_orf1	A-kinase anchor protein 200-like [Ostrinia furnacalis]	6	639	67.6	4.23	12	8	3	High	1	0.964	0.994	1.039	1.12	0.958	1.007	1.134	1.027	0.97	1.067	1.008	2.547	2.697	2.633
TRINITY_DN37048_c0_g1_i9_orf1	microtubule-actin cross-linking factor 1 isoform X15 [Ostrinia furnacalis]	3	129	14.6	6.55	35	8	3	High	1	1.081	1	1.114	1.166	1.099	1.077	1.111	1.095	1.056	1.048	0.998	1.071	0.96	1.028
TRINITY_DN17299_c0_g1_i4_orf1	RNA cytidine acetyltransferase isoform X1 [Ostrinia furnacalis]	10	986	108.8	8.34	9	10	10	High	1	0.995	1.014	0.523	0.511	0.583	0.539	0.59	0.576	0.598	0.488	0.501	0.49	0.499	0.474
TRINITY_DN3582_c0_g1_i2_orf1	uncharacterized protein LOC114357129 [Ostrinia furnacalis]	8	869	96.4	6.39	13	8	8	High	1	1.013	1.009	0.578	0.593	0.632	0.61	0.561	0.581	0.585	0.513	0.553	0.809	0.776	0.797
TRINITY_DN45924_c0_g1_i14_orf1	adenylosuccinate synthetase isoform X1 [Ostrinia furnacalis]	10	452	49.7	6.79	19	14	10	High	1	1.031	1.007	0.524	0.546	0.566	0.467	0.473	0.462	0.427	0.417	0.425	0.506	0.499	0.499
TRINITY_DN11693_c0_g1_i6_orf1	sorting nexin-32 isoform X1 [Ostrinia furnacalis]	8	417	48.2	6.61	21	8	8	High	1	0.999	1.055	1.333	1.352	1.294	1.19	1.129	1.204	0.959	1.021	1.055	1.374	1.433	1.386
TRINITY_DN12_c0_g1_i5_orf1	cAMP-dependent protein kinase type II regulatory subunit isoform X1 [Ostrinia	8	382	43.1	5.05	25	10	8	High	1	1.052	0.932	0.964	0.996	0.941	0.926	0.962	0.934	0.789	0.78	0.848	0.774	0.766	0.771
TRINITY_DN7291_c0_g1_i3_orf1	dynamin-1-like protein isoform X1 [Ostrinia furnacalis]	8	683	76.8	7.42	13	11	1	High	1	1.169	1.235	1.274	1.166	1.3	1.189	1.145	1.209	1.292	1.409	1.249	1.578	1.511	1.554
TRINITY_DN574_c0_g1_i4_orf1	CD63 antigen-like [Ostrinia furnacalis]	7	232	25.2	7.12	34	11	7	High	1	0.999	1.021	1.981	1.974	1.91	1.933	1.901	1.949	1.807	1.772	1.889	1.526	1.637	1.504
TRINITY_DN13718_c0_g1_i4_orf1	immulectin-4 [Ostrinia furnacalis]	6	255	28.6	7.03	36	7	2	High	1	1.123	1.047	1.693	1.446	1.721	2.218	1.602	2.028	1.716	1.552	1.26	1.685	1.409	1.515
TRINITY_DN46_c0_g1_i2_orf1	disks large 1 tumor suppressor protein isoform X12 [Ostrinia furnacalis]	7	745	82.6	7.46	11	7	7	High	1	1.008	1.003	0.833	0.808	0.812	0.744	0.773	0.776	0.809	0.834	0.816	0.968	0.914	0.936
TRINITY_DN15202_c0_g1_i6_orf1	uncharacterized protein LOC114364499 isoform X2 [Ostrinia furnacalis]	3	126	14	5.01	41	6	2	High	1	1.132	0.98	3.951	4.372	4.335	6.923	7.364	7.355	5.555	5.331	6.166	2.318	2.266	2.297
TRINITY_DN2749_c0_g2_i3_orf1	RNA exonuclease 4-like [Ostrinia furnacalis]	7	161	17.8	8.54	45	9	5	High	1	1	0.993	0.348	0.351	0.389	0.947	1.034	0.959	0.469	0.453	0.557	0.459	0.453	0.437
TRINITY_DN20344_c0_g1_i5_orf1	uncharacterized protein LOC114351483 [Ostrinia furnacalis]	6	122	13.5	5	57	10	4	High	1	0.89	1.005	2.836	2.703	2.767	5.11	4.355	4.604	4.017	3.232	3.902	0.935	0.829	0.953
TRINITY_DN8116_c0_g1_i1_orf1	uncharacterized protein LOC114350845 [Ostrinia furnacalis]	6	243	25.5	7.97	26	9	4	High	1	0.971	0.918	0.218	0.209	0.251	0.24	0.226	0.247	0.23	0.225	0.229	0.297	0.291	0.277
TRINITY_DN364_c2_g1_i2_orf1	vinculin-like isoform X3 [Ostrinia furnacalis]	7	639	70.5	6.95	12	8	6	High	1	1.046	1.02	1.395	1.372	1.34	1.138	1.195	1.121	1.201	1.1	1.203	1.323	1.396	1.43
TRINITY_DN9468_c1_g1_i4_orf1	coronin-1C-A isoform X1 [Bombyx mori]	7	520	57.2	6.64	17	9	7	High	1	0.987	1.012	1.189	1.225	1.123	1.07	1.075	1.023	1.018	1.066	1.113	0.81	0.755	0.869
TRINITY_DN15896_c0_g1_i4_orf1	phosphatidylinositol transfer protein alpha isoform [Ostrinia furnacalis]	9	272	31.3	6.43	32	14	9	High	1	1.024	0.976	0.606	0.569	0.63	0.558	0.556	0.563	0.565	0.506	0.533	0.521	0.538	0.513
TRINITY_DN4808_c0_g1_i3_orf1	kinesin light chain [Ostrinia furnacalis]	7	541	61.1	6.71	16	10	7	High	1	0.999	0.994	0.807	0.854	0.802	0.74	0.743	0.734	0.683	0.687	0.786	1.038	1.053	1.008
TRINITY_DN6241_c0_g1_i1_orf1	uncharacterized protein LOC114355531 [Ostrinia furnacalis]	7	84	9.5	7.18	70	20	7	High	1	1.02	0.989	0.581	0.627	0.636	0.955	0.926	0.87	0.974	0.823	1.052	0.499	0.53	0.502
TRINITY_DN14952_c0_g3_i1_orf1	protein DEK isoform X3 [Ostrinia furnacalis]	7	311	34.3	4.64	29	8	7	High	1	0.99	0.94	1.152	1.219	1.089	0.975	1.006	0.953	0.878	0.834	0.973	1.087	1.116	1.125
TRINITY_DN26963_c0_g1_i1_orf1	aminoacyl tRNA synthase complex-interacting multifunctional protein 1 isofori	7	296	32.8	8.95	25	11	7	High	1	1.022	0.966	0.51	0.52	0.513	0.537	0.54	0.512	0.486	0.417	0.461	0.558	0.588	0.567
TRINITY_DN972_c0_g1_i6_orf1	DNA damage-binding protein 1 [Ostrinia furnacalis]	10	654	71.9	5.31	16	12	7	High	1	0.985	1.013	0.907	0.834	0.853	0.82	0.825	0.81	0.815	0.859	0.742	0.942	0.885	0.99
TRINITY_DN39404_c0_g1_i7_orf1	hypothetical protein evm_004736, partial [Chilo suppressalis]	7	2019	227.1	6.81	5	7	7	High	1	1.014	1.064	1.311	1.422	1.329	1.263	1.327	1.259	1.17	1.273	1.256	1.528	1.434	1.613
TRINITY_DN2876_c0_g1_i3_orf1	long-chain fatty acid transport protein 4-like isoform X1 [Ostrinia furnacalis]	6	692	76.8	8.9	11	7	1	High	1	1.09	0.997	0.447	0.716	0.528	0.549	0.519	0.594	0.609	0.773	0.548	0.852	0.651	0.708
TRINITY_DN779_c0_g1_i12_orf1	unnamed protein product [Chilo suppressalis]	8	869	93.6	9.2	16	10	1	High	1	0.933	0.92	1.082	1.16	1.09	1.378	1.275	1.356	1.225	0.931	1.077	0.59	0.513	0.459
TRINITY_DN3601_c0_g1_i5_orf1	Golgi-specific brefeldin A-resistance guanine nucleotide exchange factor 1 [O:	9	1965	217.8	5.66	6	9	9	High	1	1.076	1.038	0.851	0.909	0.913	0.948	0.904	0.866	0.866	0.905	0.834	0.952	0.983	0.962
TRINITY_DN7024_c0_g1_i1_orf1	uncharacterized protein LOC114363116 [Ostrinia furnacalis]	7	134	15.1	6.79	68	15	7	High	1	1.369	1.272	0.02	0.022	0.076	0.054	0.066	0.06	0.063	0.054	0.062	0.07	0.057	0.063
TRINITY_DN8854_c0_g1_i2_orf1	glutathione S-transferase epsilon 2 [Ostrinia furnacalis]	7	218	24.6	4.97	37	10	7	High	1	1.009	0.935	1.174	1.223	1.257	1.335	1.338	1.215	1.445	1.438	1.42	1.102	1.089	1.126
TRINITY_DN2795_c0_g1_i1_orf1	hypothetical protein B5X24_HaOG209451 [Helicoverpa armigera]	10	335	38.3	5.78	30	10	10	High	1	1.021	1.027	0.932	0.796	0.907	0.939	0.862	0.96	0.916	0.796	0.772	0.944	0.966	0.96
TRINITY_DN1921_c1_g1_i5_orf1	hypothetical protein evm_002627 [Chilo suppressalis]	6	446	48	6.79	22	6	6	High	1	1.008	1.047	0.781	0.78	0.743	0.781	0.796	0.771	0.755	0.801	0.811	1.016	1.039	0.997
TRINITY_DN10900_c0_g1_i7_orf1	fatty acid synthase-like [Ostrinia furnacalis]	7	2309	258.9	7.23	4	8	7	High	1	0.937	0.998	1.651	1.642	1.556	1.157	1.253	1.172	1.126	1.223	1.199	1.379	1.343	1.389
TRINITY_DN1771_c0_g2_i1_orf1	eukaryotic peptide chain release factor subunit 1 isoform X1 [Danaus plexippu:	7	437	49.1	5.81	18	11	7	High	1	0.955	0.972	0.681	0.677	0.675	0.703	0.698	0.757	0.794	0.856	0.758	0.813	0.801	0.861
TRINITY_DN2320_c0_g1_i4_orf1	trifunctional enzyme subunit beta, mitochondrial isoform X1 [Ostrinia furnacali	7	473	50.6	9.11	16	16	7	High	1	0.965	0.965	0.942	0.959	0.949	0.816	0.818	0.815	0.852	0.768	0.855	0.8	0.805	0.779
TRINITY_DN11402_c0_g1_i1_orf1	constitutive coactivator of PPAR-gamma-like protein 1 isoform X1 [Ostrinia fur	9	755	84	7.9	16	10	9	High	1	0.985	0.939	0.612	0.62	0.621	0.56	0.606	0.602	0.597	0.616	0.561	0.852	0.844	0.866
TRINITY_DN114890_c0_g1_i4_orf1	chemosensory protein 10 [Ostrinia furnacalis]	6	59	6.9	7.97	76	17	1	High	1	1.077	1.134	3.397	3.519	3.669	3.117	2.914	2.922	5.528	5.02	6.324	1.654	1.691	1.578
TRINITY_DN64769_c0_g1_i3_orf1	procollagen-lysine,2-oxoglutarate 5-dioxygenase isoform X2 [Ostrinia furnaca	8	728	83.9	6.37	13	11	8	High	1	1.013	0.95	0.548	0.562	0.58	0.698	0.692	0.672	0.57	0.544	0.567	1.271	1.248	1.231
TRINITY_DN361_c0_g1_i5_orf1	hexosaminidase [Ostrinia furnacalis]	5	615	70.1	5.83	11	7	5	High	1	1.087	1.005	1.489	1.526	1.446	1.526	1.475	1.463	1.7	1.787	1.649	2.406	2.347	2.365
TRINITY_DN4281_c0_g1_i1_orf1	inosine-5'-monophosphate dehydrogenase [Ostrinia furnacalis]	6	512	55.3	8.1	18	10	6	High	1	0.991	1.02	0.892	0.867	0.854	0.834	0.902	0.821	0.823	0.829	0.898	0.812	0.78	0.799
TRINITY_DN30027_c0_g1_i1_orf1	40S ribosomal protein S14 [Plutella xylostella]	5	151	16.2	10.46	32	13	5	High	1	1.049	1.062	0.825	0.861	0.854	0.818	0.776	0.806	0.682	0.634	0.733	0.805	0.805	0.822
TRINITY_DN107617_c3_g1_i1_orf1	NADH dehydrogenase [ubiquinone] 1 alpha subcomplex subunit 9, mitochond	8	400	45.2	9.17	16	9	8	High	1	0.972	0.98	0.518	0.531	0.539	0.468	0.526	0.521	0.488	0.481	0.479	0.405	0.394	0.439
TRINITY_DN7957_c0_g1_i5_orf1	spermidine synthase [Ostrinia furnacalis]	7	287	32.3	5.85	28	11	7	High	1	1.005	1.066	1.295	1.387	1.323	1.562	1.709	1.605	1.673	1.687	1.753	1.608	1.556	1.651
TRINITY_DN3859_c0_g1_i5_orf1	D-aspartate oxidase [Ostrinia furnacalis]	8	337	37	8.22	21	9	8	High	1	1.045	1.024	1.644	1.535	1.571	1.4	1.37	1.407	1.659	1.585	1.616	1.025	1.023	0.975
TRINITY_DN3732_c0_g1_i6_orf1	cytochrome P450 monooxygenase CYP6AB141 [Ostrinia furnacalis]	9	479	55.5	8.5	20	11	5	High	1	1.007	1.06	1.171	1.193	1.195	0.963	1.064	1.004	0.847	0.988	0.909	0.918	0.839	0.912
TRINITY_DN2302_c0_g1_i1_orf1	enoyl-CoA hydratase domain-containing protein 3, mitochondrial [Ostrinia fur	6	278	30.7	8.28	27	10	6	High	1	0.994	1.024	1.64	1.589	1.539	1.35	1.357	1.324	1.21	1.277	1.211	1.045	1.08	1.083
TRINITY_DN10336_c0_g1_i9_orf1	protein phosphatase methylesterase 1 isoform X1 [Ostrinia furnacalis]	5	409	45.5	5.8	16	7	5	High	1	0.927	1.037	1.149	1.113	1.133	1.205	1.189	1.218	1.139	1.097	1.133	1.334	1.306	1.34
TRINITY_DN7022_c0_g1_i7_orf1	fatty-acid amide hydrolase 2-A-like [Ostrinia furnacalis]	11	537	59.4	7.2	16	12	11	High	1	1.033	1.041	1.45	1.452	1.426	1.086	1.099	1.108	1.264	1.262	1.323	0.976	1.003	0.984
TRINITY_DN294_c0_g1_i2_orf1	chloride intracellular channel isoform 1 [Phthorimaea operculella]	5	281	31.8	6.13	38	5	5	High	1	1.062	1.07	0.996	0.855	0.915	0.737	0.76	0.894	0.774	0.967	0.768	0.903	0.879	0.927
TRINITY_DN6908_c0_g1_i3_orf1	serine--pyruvate aminotransferase, mitochondrial [Ostrinia furnacalis]	5	82	9.8	9.13	52	9	1	High	1	0.996	0.975	1.609	1.591	1.371	1.982	1.842	2.045	1.3	1.453	1.287	1.642	1.47	1.604
TRINITY_DN397_c0_g1_i1_orf1	striatin-3 isoform X1 [Ostrinia furnacalis]	6	477	52.9	5.01	20	7	6	High	1	0.997	0.968	0.744	0.765	0.764	0.708	0.768	0.773	0.699	0.662	0.708	1.044	0.951	0.996
TRINITY_DN21420_c0_g1_i2_orf1	glutathione peroxidase, partial [Ostrinia furnacalis]	5	72	8.3	9	76	12	5	High	1	0.939	1.048	2.487	2.503	2.425	2.321	2.443	2.398	3.269	3.035	3.437	1.937	2.007	2.039
TRINITY_DN109540_c0_g1_i3_orf1	4-coumarate--CoA ligase 1-like isoform X4 [Ostrinia furnacalis]	6	534	58.4	8.48	14	6	6	High	1	1.013	1.012	0.36	0.35	0.384	0.352	0.4	0.415	0.388	0.404	0.369	0.353	0.335	0.357
TRINITY_DN25423_c0_g1_i1_orf1	uncharacterized protein LOC114353174 [Ostrinia furnacalis]	8	468	50.6	6.95	22	11	8	High	1	0.974	1.034	1.317	1.38	1.247	1.19	1.225	1.16	1.099	1.008	1.09	1.073	1.125	1.066
TRINITY_DN658_c1_g1_i6_orf1	protein disulfide-isomerase A5 isoform X1 [Ostrinia furnacalis]	7	646	72.4	7.99	11	9	7	High	1	0.999	0.98	0.878	0.838	0.91	0.861	0.803	0.824	0.843	0.827	0.805	0.936	0.92	0.912
TRINITY_DN6162_c1_g1_i1_orf1	hsc70-interacting protein-like [Ostrinia furnacalis]	8	276	31.1	5.34	36	12	8	High	1	0.998	0.978	1.643	1.541	1.682	1.622	1.495	1.597	1.478	1.301	1.374	1.511	1.594	1.534
TRINITY_DN8458_c0_g2_i1_orf1	poly(U)-specific endoribonuclease homolog [Ostrinia furnacalis]	9	554	61.5	9.48	16	10	9	High	1	0.957	1.009	0.923	0.911	0.955	1.046	1.051	1.095	1.13	1.104	1.088	1.13	1.141	1.199
TRINITY_DN36230_c0_g1_i1_orf1	sorting nexin-2 [Ostrinia furnacalis]	8	180	21.2	7.3	51	9	8	High	1	0.979	1.063	1.371	1.256	1.337	1.179	1.062	1.288	1.038	0.994	0.952	1.158	1.208	1.078
TRINITY_DN40345_c0_g1_i6_orf1	60S ribosomal protein L28 [Ostrinia furnacalis]	9	139	15.9	11.6	33	16	9	High	1	0.987	0.995	0.698	0.766	0.683	0.603	0.637	0.62	0.532	0.577	0.626	0.611	0.59	0.618
TRINITY_DN5182_c0_g1_i5_orf1	rab proteins geranylgeranyltransferase component A 1 isoform X1 [Ostrinia fur	7	532	59.8	4.97	17	9	7	High	1	0.989	1	0.769	0.745	0.807	0.818	0.853	0.807	0.871	0.8	0.831	1.397	1.423	1.401
TRINITY_DN12250_c0_g1_i4_orf1	ubiquinone biosynthesis monooxygenase COQ6, mitochondrial isoform X1 [O:	7	471	51.9	7.83	21	7	7	High	1	1.061	1.055	0.71	0.72	0.782	0.852	0.835	0.838	0.855	0.83	0.78	0.709	0.706	0.73
TRINITY_DN143_c0_g3_i1_orf1	Ubiquitin-60S ribosomal protein L40, partial [Cotesia chilonis]	8	140	16.2	9.96	39	41	1	High	1	1.042	1.032	0.654	0.669	0.724	0.761	0.788	0.721	0.565	0.505	0.62	0.641	0.63	0.647
TRINITY_DN21170_c0_g1_i5_orf1	twitchin-like [Ostrinia furnacalis]	5	155	17	5.1	52	10	1	High	1	0.937	0.958	1.801	2.032	2.033	0.892	0.779	0.884	0.732	0.655	0.875	0.512	0.569	0.566
TRINITY_DN3889_c0_g1_i7_orfp1	TRINITY_DN3889_c0_g1_i7_m.1657 TRINITY_DN3889_c0_g1::TRINITY_DN3889_	5	234	24.3	4.67	38	5	1	High	1	1.745	1.388	0.191	0.208	0.116	0.139	0.22	0.328	0.313	0.157	0.196	0.298	0.292	0.324
TRINITY_DN46173_c0_g3_i2_orf1	tropomyosin-1, isoforms 9A/A/B isoform X33 [Aedes aegypti]	7	249	29	4.7	24	17	2	High	1	1.022	0.981	0.465	0.451	0.487	0.379	0.485	0.442	0.403	0.391	0.412	0.514	0.554	0.544
TRINITY_DN1034_c0_g1_i4_orf1	glycerol kinase isoform X4 [Ostrinia furnacalis]	8	624	69.6	6.42	15	8	7	High	1	0.945	1.064	1.422	1.376	1.299	1.261	1.201	1.338	1.443	1.476	1.326	1.026	0.964	1.09
TRINITY_DN58261_c0_g1_i1_orf1	15-hydroxyprostaglandin dehydrogenase [NAD(+)]-like [Ostrinia furnacalis]	8	268	29.4	5.91	34	12	5	High	1	0.977	0.979	0.824	0.79	0.845	0.686	0.649	0.705	0.555	0.61	0.547	0.678	0.647	0.721
TRINITY_DN1004_c0_g1_i6_orf1	rho GDP-dissociation inhibitor 1 [Papilio machaon]	6	205	23.4	4.93	37	10	6	High	1	1.034	1.141	1.049	1.052	1.031	1.036	1.066	1.228	1.053	0.925	0.981	1.09	1.042	1.119
TRINITY_DN2772_c0_g1_i3_orf1	uncharacterized protein LOC114353284 isoform X4 [Ostrinia furnacalis]	7	257	28.3	6.9	33	9	7	High	1	0.94	0.985	1.362	1.383	1.329	1.427	1.568	1.483	1.704	1.987	1.701	0.979	0.998	1.048
TRINITY_DN2258_c0_g2_i1_orf1	60S ribosomal protein L14 [Ostrinia furnacalis]	6	166	18.8	11.39	32	14	6	High	1	0.971	0.965	0.737	0.778	0.726	0.673	0.655	0.671	0.651	0.719	0.693	0.711	0.707	0.743
TRINITY_DN3229_c0_g1_i1_orf1	uncharacterized protein LOC114358442 isoform X1 [Ostrinia furnacalis]	6	217	23.3	5.29	37	11	6	High	1	1.029	1.103	0.452	0.453	0.513	0.469	0.507	0.475	0.462	0.426	0.453	0.337	0.324	0.333

TRINITY_DN12508_c0_g1_i1_orf1	uncharacterized protein LOC114350091 [Ostrinia furnacalis]	8	549	62.3	9.29	13	10	8	High	1	0.995	1.019	0.954	0.825	0.899	0.894	0.855	0.92	0.73	0.735	0.649	0.595	0.597	0.612
TRINITY_DN10385_c0_g1_i5_orf1	unnamed protein product [Arctia plantaginis]	7	484	55.1	5.92	16	8	7	High	1	1.026	0.928	0.805	0.738	0.832	0.772	0.796	0.781	0.725	0.712	0.625	1.046	0.963	0.982
TRINITY_DN72999_c0_g1_i1_orf1	protein obstructor-E-like isoform X1 [Ostrinia furnacalis]	6	295	32.9	5.47	24	11	6	High	1	1.02	0.997	0.612	0.594	0.62	0.545	0.608	0.577	0.521	0.499	0.545	2.17	2.333	2.2
TRINITY_DN49265_c0_g3_i2_orf1	cytochrome c [Ostrinia furnacalis]	7	108	11.8	9.48	50	13	7	High	1	0.975	0.97	0.376	0.378	0.428	0.318	0.324	0.308	0.374	0.36	0.378	0.352	0.346	0.344
TRINITY_DN4041_c0_g1_i6_orf1	tubulin-folding cofactor B isoform X3 [Ostrinia furnacalis]	7	247	28.1	5.2	23	10	7	High	1	0.974	1.001	1.113	1.163	1.142	1.328	1.284	1.287	1.396	1.158	1.414	1.616	1.693	1.652
TRINITY_DN17446_c0_g1_i1_orf1	eukaryotic translation initiation factor 3 subunit E [Ostrinia furnacalis]	6	445	52	5.81	19	6	6	High	1	1.035	1.09	0.729	0.647	0.728	0.61	0.584	0.67	0.611	0.655	0.582	0.615	0.555	0.653
TRINITY_DN47661_c0_g1_i8_orf1	L-dopachrome tautomerase yellow-f2-like [Ostrinia furnacalis]	4	133	15.1	8.46	45	8	3	High	1	1.037	1.047	0.975	0.968	0.892	0.937	0.984	1.039	1.06	1.093	1.056	1.116	1.064	1.108
TRINITY_DN3378_c0_g1_i4_orf1	squamous cell carcinoma antigen recognized by T-cells 3 [Ostrinia furnacalis]	6	901	103.6	5.64	9	6	6	High	1	1.03	1.083	1.042	1.033	1.006	0.943	0.969	1.021	1.003	1.031	1.059	1.031	1.004	1.031
TRINITY_DN4494_c0_g1_i1_orf1	venom serine carboxypeptidase [Ostrinia furnacalis]	6	485	56.1	8.75	14	8	6	High	1	0.914	0.977	0.828	0.802	0.823	0.632	0.621	0.655	0.686	0.65	0.635	1.042	1.075	1.084
TRINITY_DN124654_c0_g1_i1_orf1	protein lethal(2)essential for life [Manduca sexta]	6	195	22.4	5.17	44	12	6	High	1	0.984	1.039	1.828	1.889	1.749	4.577	4.922	3.863	4.061	3.898	4.496	3.122	3.232	3.181
TRINITY_DN2986_c1_g1_i1_orf1	Troponin C, isoform 1 [Papilio xuthus]	5	181	20.2	4.31	22	15	5	High	1	1.048	0.962	0.363	0.373	0.438	0.258	0.233	0.231	0.16	0.142	0.159	0.103	0.098	0.099
TRINITY_DN12534_c0_g1_i4_orf1	antibacterial protein [Heliothis virescens]	6	104	12	6.07	57	10	5	High	1	1.032	0.978	5.086	5.289	4.852	5.448	5.514	5.199	6.182	5.118	6.438	2.269	2.318	2.369
TRINITY_DN1330_c0_g1_i1_orf1	pancreatic triacylglycerol lipase-like [Ostrinia furnacalis]	8	572	65	5.03	15	18	7	High	1	0.982	1.052	0.318	0.327	0.326	0.356	0.38	0.382	0.425	0.414	0.416	0.429	0.416	0.438
TRINITY_DN2407_c0_g1_i2_orf1	uncharacterized protein LOC114366345 isoform X2 [Ostrinia furnacalis]	6	197	21.5	5.5	36	8	1	High	1	0.892	1.012	7.327	7.724	7.256	1.34	1.409	1.725	2.859	3.274	3.148	1.32	0.99	1.219
TRINITY_DN512_c0_g1_i10_orf1	uncharacterized protein LOC114366781 [Ostrinia furnacalis]	6	146	16.8	7.81	43	12	4	High	1	0.971	1.012	1.661	1.583	1.516	2.076	2.027	2	1.926	1.773	1.843	1.479	1.549	1.398
TRINITY_DN10637_c0_g1_i4_orf1	V-type proton ATPase subunit d [Bombyx mandarina]	7	348	39.6	5	20	13	7	High	1	1.043	1.068	0.377	0.353	0.394	0.358	0.392	0.403	0.385	0.415	0.367	0.371	0.364	0.385
TRINITY_DN452_c9_g1_i1_orf1	epidermal retinol dehydrogenase 2-like [Ostrinia furnacalis]	8	362	39.6	8.57	26	8	8	High	1	1.032	1.027	1.957	1.837	1.839	1.528	1.681	1.645	1.434	1.511	1.391	1.153	1.284	1.3
TRINITY_DN17510_c0_g1_i1_orf1	coatomer subunit gamma-2-like [Ostrinia furnacalis]	8	864	95.1	5.66	11	10	7	High	1	1.006	1.032	0.921	0.834	0.911	1.001	0.916	1.081	0.926	0.915	0.792	1.021	1.021	1.051
TRINITY_DN1268_c0_g1_i1_orf1	nuclear pore complex protein Nup154 [Ostrinia furnacalis]	8	1111	120.6	6.6	11	8	8	High	1	0.98	1.006	0.767	0.747	0.768	0.735	0.792	0.756	0.64	0.8	0.669	0.653	0.658	0.689
TRINITY_DN2490_c0_g2_i1_orfp1	TRINITY_DN2490_c0_g2_i1_m.56872 TRINITY_DN2490_c0_g2::TRINITY_DN249(	7	358	36.3	5.88	34	8	7	High	1	0.923	1.037	0.058	0.057	0.056	0.08	0.091	0.102	0.071	0.074	0.07	0.088	0.107	0.116
TRINITY_DN5684_c0_g1_i4_orf1	Profilin [Operophtera brumata]	7	126	13.7	6.54	59	14	7	High	1	1.013	1.019	0.934	0.89	0.897	0.984	0.91	0.942	0.963	0.903	0.861	1.07	1.059	1.058
TRINITY_DN6994_c0_g1_i3_orf1	C-type mannose receptor 2-like isoform X1 [Ostrinia furnacalis]	6	324	37	5.59	23	10	5	High	1	0.968	1.041	1.666	1.714	1.678	1.197	1.26	1.179	1.273	1.285	1.272	1.091	1.023	0.978
TRINITY_DN128_c0_g1_i5_orf1	PREDICTED: muscle-specific protein 20-like [Amyelois transitella]	7	182	20.4	8.57	41	17	7	High	1	1.002	1.007	0.987	0.974	0.981	1.109	1.139	1.081	1.11	1.157	1.077	0.734	0.765	0.764
TRINITY_DN3348_c0_g1_i1_orf1	larval cuticle protein A2B-like [Ostrinia furnacalis]	5	199	20.3	6.7	42	9	4	High	1	1.01	0.986	1.059	1.007	0.927	1.037	1.079	1.02	0.976	0.967	1.059	1.121	1.088	1.103
TRINITY_DN327_c1_g1_i4_orf1	mitochondrial import receptor subunit TOM40 homolog 1-like [Ostrinia furnac	6	315	34	7.9	26	7	6	High	1	0.982	1.004	0.401	0.395	0.429	0.393	0.407	0.407	0.381	0.359	0.352	0.413	0.418	0.428
TRINITY_DN23586_c0_g1_i3_orf1	myrosinase 1-like isoform X1 [Ostrinia furnacalis]	8	342	39.2	4.94	29	9	5	High	1	1.014	1.035	0.442	0.444	0.429	0.478	0.488	0.462	0.415	0.525	0.505	0.424	0.396	0.443
TRINITY_DN32586_c0_g2_i1_orf1	unnamed protein product [Euphydryas editha]	4	176	20.5	6.39	47	6	4	High	1	0.976	0.959	1.772	1.813	1.729	2.057	2.022	1.94	1.808	1.648	1.909	0.6	0.713	0.684
TRINITY_DN9464_c0_g1_i1_orf1	angio-associated migratory cell protein [Ostrinia furnacalis]	6	404	44.7	4.68	20	7	6	High	1	1.035	1.004	0.333	0.358	0.383	0.349	0.345	0.365	0.309	0.261	0.289	0.337	0.281	0.291
TRINITY_DN2521_c1_g1_i2_orf1	thioredoxin-like protein 1 [Ostrinia furnacalis]	5	286	31.2	5.39	34	6	5	High	1	0.982	0.95	1.19	1.213	1.248	1.057	1.067	1.119	1.022	1.056	0.99	1.16	1.1	1.091
TRINITY_DN4640_c0_g1_i7_orf1	probable 3-hydroxyacyl-CoA dehydrogenase B0272.3 isoform X1 [Ostrinia fur	8	310	34.2	7.88	33	10	7	High	1	0.99	0.951	0.977	1.005	0.952	1.126	1.142	1.076	1.069	1.059	1.151	1.043	1.074	1.098
TRINITY_DN9718_c0_g1_i7_orf1	uncharacterized protein LOC114365184 [Ostrinia furnacalis]	7	741	82.8	9.72	13	8	7	High	1	0.961	1.004	1.624	1.631	1.603	1.599	1.613	1.646	1.715	1.629	1.582	1.6	1.537	1.613
TRINITY_DN935_c0_g1_i3_orf1	carboxylesterase 5A-like [Ostrinia furnacalis]	9	578	64.7	5.68	15	11	7	High	1	0.976	0.986	0.807	0.854	0.84	1.05	1.091	1.099	0.903	1.137	1.026	0.815	0.804	0.788
TRINITY_DN429_c0_g1_i12_orf1	hypothetical protein SFRUCORN_009336 [Spodoptera frugiperda]	6	224	25	6.25	27	9	6	High	1	0.987	0.996	0.784	0.813	0.801	0.826	0.849	0.826	0.833	0.798	0.788	0.947	0.925	0.931
TRINITY_DN7674_c0_g1_i2_orf1	prefoldin subunit 2 [Ostrinia furnacalis]	6	145	16.1	6.8	54	10	6	High	1	0.997	0.958	0.89	0.993	0.97	0.8	0.822	0.812	0.792	0.782	0.762	0.833	0.811	0.838
TRINITY_DN117_c0_g1_i5_orf1	lipase member I-like [Ostrinia furnacalis]	6	421	46.9	4.83	21	8	6	High	1	0.986	0.981	0.244	0.24	0.289	0.298	0.317	0.297	0.269	0.265	0.299	0.233	0.241	0.245
TRINITY_DN41259_c0_g1_i6_orf1	endocuticle structural glycoprotein SgAbd-8 [Ostrinia furnacalis]	7	216	24.7	5.02	44	13	7	High	1	0.995	0.979	0.15	0.144	0.18	0.154	0.174	0.165	0.149	0.156	0.145	1.547	1.639	1.471
TRINITY_DN11113_c0_g1_i1_orf1	glutamate--cysteine ligase [Ostrinia furnacalis]	7	666	75.8	6.89	13	11	7	High	1	1.077	0.993	1.054	1.009	1.006	1.023	1.04	1.134	1.004	0.928	0.933	0.943	0.95	0.957
TRINITY_DN220_c0_g1_i3_orf1	serine-arginine protein 55 isoform X6 [Pieris brassicae]	6	226	26.5	10.26	28	8	6	High	1	0.925	0.942	0.54	0.564	0.581	0.434	0.472	0.419	0.4	0.387	0.41	0.703	0.72	0.755
TRINITY_DN9079_c1_g1_i1_orf1	UDP-glucuronosyltransferase 2B15-like [Ostrinia furnacalis]	8	514	58.4	8.29	16	9	7	High	1	1.021	1.06	1.11	0.944	1.068	1.322	1.248	1.4	1.126	1.165	0.857	0.927	0.882	0.95
TRINITY_DN19043_c0_g2_i1_orf1	hypothetical protein EVAR_60653_1 [Eumeta japonica]	6	187	21	5	32	19	6	High	1	1.014	1.047	1.887	1.931	1.84	1.79	1.859	1.757	1.877	1.672	1.876	5.314	5.657	5.002
TRINITY_DN9100_c0_g1_i5_orf1	microtubule-associated protein futsch-like isoform X6 [Ostrinia furnacalis]	10	2257	250.4	5	5	10	10	High	1	1.02	1.004	0.791	0.821	0.831	0.832	0.934	0.825	0.864	0.79	0.84	1.417	1.338	1.339
TRINITY_DN30311_c0_g1_i4_orf1	ribose-5-phosphate isomerase [Ostrinia furnacalis]	6	268	29.3	8.12	25	8	6	High	1	1.008	1.026	1.088	1.066	1.012	0.934	0.909	0.972	0.865	0.905	0.871	0.885	0.898	0.887
TRINITY_DN3010_c0_g1_i4_orf1	inositol oxygenase-like [Ostrinia furnacalis]	9	291	34	5.66	33	12	9	High	1	1.012	1.036	1.114	1.073	1.115	1.071	1.044	1.088	0.877	0.874	0.858	0.702	0.755	0.725
TRINITY_DN27_c0_g1_i1_orf1	THO complex subunit 4-A [Ostrinia furnacalis]	7	260	27.4	10.89	27	9	7	High	1	1.038	1.029	0.625	0.638	0.685	0.594	0.582	0.56	0.538	0.515	0.545	0.729	0.766	0.732
TRINITY_DN144_c0_g1_i4_orf1	COPII coat assembly protein sec16-like [Ostrinia furnacalis]	9	1628	172.6	4.54	7	9	9	High	1	0.957	1.023	0.915	0.979	0.943	0.988	1.02	0.972	0.913	1.08	1.016	1.406	1.302	1.345
TRINITY_DN5666_c0_g1_i2_orf1	protein ROP isoform X2 [Ostrinia furnacalis]	9	587	66.9	7.15	16	11	9	High	1	1.059	1.04	1.133	1.057	1.131	0.972	1.005	0.984	0.933	0.794	0.926	1.165	1.17	1.179
TRINITY_DN7152_c0_g1_i1_orf1	tissue alpha-L-fucosidase [Ostrinia furnacalis]	10	493	57.2	6.28	19	13	10	High	1	0.945	0.98	1.514	1.484	1.489	1.378	1.379	1.414	1.424	1.365	1.393	1.203	1.267	1.21
TRINITY_DN19092_c0_g1_i2_orf1	eukaryotic translation initiation factor 3 subunit L [Ostrinia furnacalis]	10	544	63.7	6.49	19	12	10	High	1	0.948	0.958	0.573	0.499	0.577	0.505	0.508	0.555	0.452	0.54	0.392	0.522	0.523	0.549
TRINITY_DN13088_c0_g1_i5_orf1	beta-hexosaminidase subunit alpha-like isoform X2 [Ostrinia furnacalis]	9	547	62.8	6.35	17	12	9	High	1	1.002	1.01	1.238	1.046	1.175	1.11	1.117	1.203	1.4	1.337	1.158	2.088	2.056	2.103
TRINITY_DN15234_c0_g1_i3_orf1	60S ribosomal protein L30 [Papilio polytes]	6	113	12.4	9.63	60	11	6	High	1	0.963	1.038	0.819	0.762	0.813	0.769	0.755	0.804	0.711	0.801	0.698	0.631	0.637	0.655
TRINITY_DN7294_c0_g2_i4_orf1	hypothetical protein evm_003455 [Chilo suppressalis]	7	489	55.7	5.88	19	9	7	High	1	0.95	1.01	0.817	0.833	0.86	0.811	0.871	0.803	0.78	0.804	0.788	0.775	0.767	0.769
TRINITY_DN15858_c0_g1_i1_orf1	15-hydroxyprostaglandin dehydrogenase [NAD(+)]-like [Ostrinia furnacalis]	3	271	29.6	6.8	21	5	1	High	1	0.88	0.965	1.672	1.864	1.791	1.898	1.963	1.939	1.967	2.016	2.092	1.152	1.049	1.19
TRINITY_DN30208_c0_g1_i3_orf1	unnamed protein product [Timema cristinae]	4	68	7.7	4.5	57	14	4	High	1	1.016	1.003	1.413	1.441	1.424	1.269	1.308	1.194	1.081	0.933	1.218	0.456	0.461	0.463
TRINITY_DN71699_c0_g1_i1_orf1	apolipophorins-like [Ostrinia furnacalis]	6	72	8.2	8.97	63	19	6	High	1	1.033	1.013	1.444	1.433	1.363	1.555	1.634	1.616	2.454	2.411	2.543	3.235	3.172	3.192
TRINITY_DN6908_c0_g1_i1_orf1	serine--pyruvate aminotransferase, mitochondrial [Ostrinia furnacalis]	5	92	10.7	9.36	53	9	1	High	1	1.232	1.488	1.98	1.782	1.815	2.29	2.525	2.57	1.824	2.976	1.661	2.048	1.93	2.351
TRINITY_DN3878_c0_g1_i4_orf1	eukaryotic translation initiation factor 3 subunit J [Ostrinia furnacalis]	6	242	28.1	4.96	23	10	6	High	1	1.04	1.046	0.632	0.662	0.657	0.581	0.594	0.564	0.582	0.613	0.589	0.616	0.612	0.618
TRINITY_DN3708_c0_g1_i3_orf1	hypothetical protein evm_001632 [Chilo suppressalis]	6	335	37.4	5.27	22	10	6	High	1	0.973	0.973	0.998	0.947	0.956	0.853	0.857	0.896	0.908	0.997	0.9	0.855	0.824	0.879
TRINITY_DN667_c0_g1_i13_orf1	enoyl-CoA hydratase domain-containing protein 2, mitochondrial [Ostrinia fur	8	296	32	8.76	31	8	8	High	1	1.044	0.976	0.765	0.825	0.774	0.872	0.879	0.875	0.81	0.747	0.856	0.751	0.801	0.742
TRINITY_DN6671_c0_g1_i6_orf1	hypothetical protein evm_013656 [Chilo suppressalis]	6	161	17.9	7.33	35	7	6	High	1	0.983	0.954	0.573	0.55	0.589	0.541	0.587	0.563	0.501	0.447	0.48	0.642	0.678	0.646
TRINITY_DN2110_c0_g1_i3_orf1	ribose-phosphate pyrophosphokinase 1 isoform X1 [Chelonus insularis]	5	386	42.2	6.98	13	9	1	High	1	0.923	0.978	1.301	1.277	1.223	1.48	1.504	1.783	1.676	1.723	1.543	1.547	1.399	1.593
TRINITY_DN23564_c0_g1_i7_orf1	cytochrome P450 6B6-like [Ostrinia furnacalis]	10	514	59.4	8.79	17	11	10	High	1	0.914	1.012	1.55	1.626	1.487	1.585	1.611	1.656	1.309	1.243	1.285	1.255	1.218	1.193
TRINITY_DN97378_c0_g1_i2_orf1	dihydropteridine reductase [Galleria mellonella]	5	234	24.7	7.37	32	8	5	High	1	1.026	0.999	1.387	1.375	1.357	1.426	1.518	1.517	1.486	1.563	1.395	1.628	1.5	1.64
TRINITY_DN214_c0_g1_i4_orf1	uncharacterized protein LOC114352813 [Ostrinia furnacalis]	7	262	29.6	8.59	31	11	4	High	1	0.889	0.964	3.272	3.236	3.2	1.838	1.788	1.645	2.277	2.052	2.124	0.869	0.738	0.93
TRINITY_DN7539_c0_g1_i2_orf1	serine protease inhibitor 3/4 [Ostrinia furnacalis]	9	519	58.6	5.66	18	10	9	High	1	0.962	0.955	1.048	1.047	1.022	1.15	1.373	1.289	1.111	1.463	1.033	4.448	4.576	4.464
TRINITY_DN26790_c0_g1_i3_orf1	protein unc-45 homolog B [Ostrinia furnacalis]	8	502	55.9	5.57	18	9	8	High	1	0.986	1.005	0.709	0.714	0.711	0.842	0.877	0.827	0.841	0.984	0.809	0.822	0.842	0.915
TRINITY_DN1369_c0_g2_i3_orf1	ATP-dependent Clp protease ATP-binding subunit clpX-like, mitochondrial isc	8	568	62.1	8.4	15	9	8	High	1	0.992	1.017	0.946	0.961	0.96	0.816	0.828	0.815	0.769	0.74	0.759	0.76	0.711	0.742
TRINITY_DN42824_c0_g1_i5_orf1	prefoldin subunit 3 [Ostrinia furnacalis]	5	186	21.3	5.22	33	9	5	High	1	0.964	1.032	0.625	0.667	0.637	0.59	0.548	0.594	0.608	0.581	0.592	0.766	0.758	0.77
TRINITY_DN1470_c0_g1_i2_orf1	oxidation resistance protein 1 isoform X5 [Ostrinia furnacalis]	7	827	91.2	5.11	12	8	4	High	1	1.08	1.049	1.224	1.218	1.225	1.03	1.115	1.155	1.107	1.061	1.17	0.817	0.875	0.818
TRINITY_DN22678_c0_g1_i4_orf1	NADH-cytochrome b5 reductase 2 isoform X2 [Ostrinia furnacalis]	7	330	37.4	9.35	27	10	7	High	1	0.979	0.947	0.555	0.503	0.541	0.619	0.619	0.63	0.548	0.51	0.502	0.51	0.526	0.492
TRINITY_DN1093_c0_g1_i4_orf1	uncharacterized protein LOC114361723 isoform X4 [Ostrinia furnacalis]	6	238	26.1	5.58	32	8	1	High	1	1.031	0.964	3.332	3.29	2.796	3.636	3.654	3.882	3.613	3.833	3.605	4.207	3.83	4.153
TRINITY_DN51008_c0_g1_i1_orf1	alanine--glyoxylate aminotransferase 2, mitochondrial [Ostrinia furnacalis]	8	488	53.9	7.61	23	9	8	High	1	1.015	1.017	0.969	0.923	0.947	0.976	1.058	1.024	1.015	1.138	1.013	0.97	0.986	0.939
TRINITY_DN6693_c0_g1_i1_orf1	uncharacterized protein LOC114356358 [Ostrinia furnacalis]	7	345	40.4	6.47	24	9	6	High	1	1.008	0.967	0.188	0.17	0.219	0.207	0.195	0.201	0.209	0.217	0.198	0.237	0.254	0.256
TRINITY_DN20957_c0_g1_i1_orf1	adenylate kinase isoenzyme 1 isoform X2 [Ostrinia furnacalis]	6	238	26.2	8.84	30	10	6	High	1	1.004	0.976	0.761	0.753	0.795	0.634	0.62	0.612	0.662	0.601	0.643	0.342	0.333	0.342
TRINITY_DN28759_c0_g1_i1_orf1	innexin inx2 [Ostrinia furnacalis]	7	359	41.6	6.77	19	9	7	High	1	1.004	0.985	0.741	0.69	0.753	0.789	0.742	0.822	0.763	0.716	0.655	1.073	1.066	1.058
TRINITY_DN20347_c0_g1_i6_orf1	venom polypeptide precursor [Doratifera vulnerans]	5	216	24.4	5.19	28	12	5	High	1	1.131	0.984	1.468	1.548	1.435	1.643	1.663	1.655	2.142	2.008	2.275	1.35	1.347	1.325
TRINITY_DN27641_c0_g1_i1_orf1	succinate dehydrogenase [ubiquinone] iron-sulfur subunit, mitochondrial [Ost	6	282	31.8	8.37	30	11	6	High	1	1.015	0.996	0.434	0.444	0.505	0.438	0.443	0.423	0.453	0.471	0.438	0.458	0.427	0.432
TRINITY_DN54410_c0_g2_i1_orf1	lysozyme-like [Ostrinia furnacalis]	5	141	16.2	8.41	43	14	4	High	1	1.09	0.992	0.95	0.981	1.032	1.173	0.984	1.172	1.322	1.226	1.266	0.964	0.982	0.989
TRINITY_DN1491_c0_g1_i8_orf1	GILT-like protein 2 isoform X1 [Ostrinia furnacalis]	5	230	26	5.02	21	11	2	High	1	0.976	1.009	1.145	1.077	1.196	1.233	1.112	1.12	1.16	1.136	1.114	1.865	1.884	1.845
TRINITY_DN12065_c0_g1_i4_orf1	protein DEK isoform X2 [Galleria mellonella]	5	295	31.3	8.9	13	9	5	High	1	0.971	1.044	1.205	1.22	1.201	1.176	1.168	1.146	1.168	1.077	1.263	1.341	1.378	1.377
TRINITY_DN146524_c0_g1_i1_orf1	pupal cuticle protein-like [Ostrinia furnacalis]	4	219	22.2	7.21	30	9	4	High	1	0.954	1.053	1.003	1.052	1.06	1.028	1.039	1.023	0.985	0.912	1.066	1.488	1.501	1.423
TRINITY_DN3366_c0_g1_i6_orf1	eukaryotic translation initiation factor 3 subunit K [Helicoverpa zea]	7	214	24.8	5.81	38	11	7	High	1	1.018	1.004	0.628	0.62	0.679	0.587	0.582	0.592	0.571	0.521	0.551	0.65	0.651	0.672
TRINITY_DN10769_c0_g1_i1_orf1	proteasome subunit beta type-5 [Ostrinia furnacalis]	5	281	31.1	6.42	20	14	5	High	1	0.959	1.021	1.053	0.994	0.999	1.011	0.945	1.047	0.916	0.954	0.892	1.21	1.121	1.143
TRINITY_DN2392_c0_g2_i1_orf1	cytochrome P450 9e2-like [Ostrinia furnacalis]	8	533	61.5	8.38	17	9	6	High	1	0.961	1.012	1.424	1.463	1.332	2.185	2.106	2.131	1.701	1.567	1.869	1.3	1.262	1.273
TRINITY_DN21315_c0_g1_i1_orf1	exportin-7-B isoform X1 [Ostrinia furnacalis]	8	1091	122.4	6.38	9	8	8	High	1	1.027	1.015	1.031	0.998	0.983	1.086	1.1	1.083	1.02	1.012	0.917	1.004	0.928	1.008
TRINITY_DN288_c0_g1_i9_orf1	unnamed protein product [Chilo suppressalis]	8	766	84	6	13	9	8	High	1	0.962	0.99	0.654	0.653	0.681	0.708	0.693	0.668	0.628	0.596	0.645	0.913	0.894	0.913
TRINITY_DN10831_c1_g1_i1_orf1	40S ribosomal protein S16 [Ostrinia furnacalis]	7	151	17	10.11	40	11	5	High	1	1.047	1.085	0.764	0.717	0.761	0.628	0.614	0.658	0.531	0.552	0.53	0.517	0.493	0.529
TRINITY_DN166_c0_g1_i4_orf1	PREDICTED: cryptochrome-1 isoform X1 [Amyelois transitella]	4	555	63.5	6.8	10	6	4	High	1	0.904	0.955	1.218	1.187	1.255	1.375	1.611	1.456	1.654	1.6	1.443	1.874	1.887	1.918
TRINITY_DN12228_c0_g1_i2_orf1	transcriptional activator protein Pur-beta isoform X1 [Ostrinia furnacalis]	5	267	30.2	7.46	25	8	5	High	1	0.967	0.986	1.104	1.079	1.066	1.04	1.027	0.998	0.982	0.926	0.948	1.159	1.151	1.065
TRINITY_DN2283_c0_g2_i1_orf1	H/ACA ribonucleoprotein complex subunit 4 [Ostrinia furnacalis]	9	510	57.3	9.25	16	14	9	High	1	0.986	1.015	0.542	0.523	0.577	0.588	0.608	0.561	0.535	0.477	0.53	0.468	0.471	0.452
TRINITY_DN29009_c0_g2_i2_orf1	juvenile hormone binding protein [Omphisa fuscidentalis]	4	199	22	4.89	23	8	1	High	1	0.942	0.992	1.887	1.91	2.053	1.987	1.903	1.964	2.141	1.793	2.462	1.578	1.701	1.746
TRINITY_DN364_c0_g2_i1_orf1	unnamed protein product [Chilo suppressalis]	6	510	55.5	5.8	14	8	5	High	1	0.987	1.131	1.559	1.655	1.563	1.253	1.375	1.357	1.324	1.197	1.499	1.507	1.555	1.536
TRINITY_DN712_c0_g2_i1_orf1	serine protease inhibitor 77Ba-like [Ostrinia furnacalis]	7	411	46.4	6.96	17	14	7	High	1	1.012	0.983	1.115	1.189	1.132	1.322	1.365	1.299	1.279	1.312	1.199	2.061	2.102	2.018
TRINITY_DN1264_c0_g1_i2_orf1	L-lactate dehydrogenase isoform X1 [Ostrinia furnacalis]	7	348	37.8	6.95	22	10	7	High	1	1.051	0.967	0.963	0.881	0.879	1.217	1.141	1.245	1.096	1.113	0.975	0.807	0.801	0.877
TRINITY_DN17861_c0_g1_i5_orf1	acyl-CoA-binding protein homolog isoform X1 [Ostrinia furnacalis]	4	90	9.6	8.34	47	10	4	High	1	0.925	0.957	0.786	0.777	0.795	0.79	0.788	0.788	0.763	0.773	0.761	0.886	0.842	0.932

TRINITY_DN4748_c0_g1_i5_orf1	unnamed protein product, partial [Brenthis ino]	3	71	7.8	7.88	54	8	3	High	1	0.998	1.069	2.457	2.544	2.393	2.193	2.106	2.149	2.644	2.19	2.556	4.379	5.037	4.465
TRINITY_DN122867_c1_g1_i1_orf1	nuclear migration protein nudC [Ostrinia furnacalis]	7	326	37.7	5.14	22	8	7	High	1	1.025	0.993	0.67	0.648	0.688	0.788	0.734	0.743	0.692	0.686	0.678	0.927	0.928	0.922
TRINITY_DN8245_c0_g1_i4_orf1	uncharacterized protein LOC114357622 [Ostrinia furnacalis]	5	112	12.5	8.57	62	7	2	High	1	0.939	0.884	0.985	1.127	0.993	1.383	1.387	1.555	1.576	1.612	1.55	1.355	1.415	1.358
TRINITY_DN31058_c0_g1_i6_orf1	HIV Tat-specific factor 1 homolog [Ostrinia furnacalis]	7	219	24.7	4.73	30	12	7	High	1	0.988	0.993	0.797	0.848	0.821	0.692	0.755	0.695	0.694	0.711	0.757	0.863	0.83	0.845
TRINITY_DN138481_c0_g1_i5_orf1	hypothetical protein evm_003901 [Chilo suppressalis]	4	217	22.6	8.35	32	7	2	High	1	1.153	1.146	1.384	1.22	1.324	1.529	1.731	1.539	1.763	1.709	1.953	7.965	7.389	6.862
TRINITY_DN31118_c0_g2_i1_orf1	unnamed protein product [Spodoptera exigua]	2	122	12.9	4.92	53	9	2	High	1	1	0.957	1.326	1.443	1.379	1.599	1.532	1.46	1.582	1.267	1.696	0.252	0.212	0.243
TRINITY_DN1993_c0_g1_i1_orf1	6-phosphofructo-2-kinase/fructose-2,6-bisphosphatase isoform X1 [Ostrinia f	6	513	58.6	7.78	13	12	6	High	1	1.008	1.058	1.442	1.378	1.355	1.138	1.078	1.138	1.143	1.009	1.075	0.892	0.851	0.875
TRINITY_DN1080_c0_g1_i1_orf1	bleomycin hydrolase [Ostrinia furnacalis]	8	485	55.6	7.18	21	8	8	High	1	0.994	1.005	1.465	1.573	1.568	1.327	1.375	1.318	1.433	1.344	1.415	1.451	1.462	1.449
TRINITY_DN26994_c1_g1_i6_orf1	FGGY carbohydrate kinase domain-containing protein [Ostrinia furnacalis]	8	547	60.1	6.52	15	12	8	High	1	1.053	1.05	1.037	0.98	0.983	1.25	1.188	1.268	1.14	1.116	1.069	1.135	1.148	1.17
TRINITY_DN695_c0_g1_i5_orf1	uncharacterized protein LOC114363574 isoform X1 [Ostrinia furnacalis]	5	253	27.6	5.6	28	7	3	High	1	1.071	1.038	1.368	1.436	1.467	1.294	1.471	1.292	1.269	1.321	1.63	1.048	0.892	1.139
TRINITY_DN2947_c0_g1_i4_orf1	ras-related protein Rab-39B [Spodoptera litura]	6	239	27.1	5.36	29	9	5	High	1	0.974	0.941	1.655	1.416	1.561	1.763	1.591	1.897	1.729	1.659	1.494	1.455	1.533	1.378
TRINITY_DN120979_c0_g1_i1_orf1	la-related protein 1-like isoform X2 [Ostrinia furnacalis]	7	324	34.8	9.16	27	9	7	High	1	1.027	1.016	0.614	0.7	0.626	0.538	0.581	0.559	0.526	0.543	0.638	0.705	0.675	0.682
TRINITY_DN2684_c0_g2_i3_orf1	glutamate decarboxylase 1-like isoform X1 [Ostrinia furnacalis]	6	481	54.5	6.83	18	6	6	High	1	1.021	1.115	1.215	1.171	1.154	1.384	1.546	1.496	1.721	1.637	1.517	2.662	2.852	2.618
TRINITY_DN60949_c0_g1_i4_orf1	aldo-keto reductase AKR2E4-like [Galleria mellonella]	4	123	13.7	9.5	24	18	1	High	1	0.97	0.927	1.562	1.539	1.402	1.044	0.961	1.015	0.935	0.829	0.855	1.428	1.433	1.442
TRINITY_DN142376_c0_g1_i1_orf1	ubiquitin-conjugating enzyme E2 N [Ostrinia furnacalis]	3	151	17.2	6.57	28	7	3	High	1	1.057	1.064	1.117	1.105	1.141	1.136	1.138	1.176	1.338	1.179	1.646	1.142	1.175	1.191
TRINITY_DN629_c0_g1_i6_orf1	annexin B9-like isoform X1 [Ostrinia furnacalis]	6	495	54.7	6.32	16	9	6	High	1	1.066	1.022	1.05	0.976	1.057	0.722	0.71	0.786	0.886	0.763	0.809	1.111	1.098	1.073
TRINITY_DN4956_c0_g1_i6_orf1	nucleolar GTP-binding protein 1 [Ostrinia furnacalis]	6	655	75.1	9.66	10	7	6	High	1	1.039	1.008	0.429	0.475	0.424	0.377	0.475	0.468	0.488	0.457	0.467	0.468	0.438	0.442
TRINITY_DN9302_c0_g1_i1_orf1	DEAD-box helicase Dbp80 [Ostrinia furnacalis]	7	465	51.6	6.47	19	7	7	High	1	0.999	1.004	1.093	1.068	1.094	1.035	1.042	0.989	0.987	0.961	0.982	1.261	1.31	1.312
TRINITY_DN1384_c0_g1_i5_orf1	vacuolar protein sorting-associated protein VTA1 homolog [Ostrinia furnacalis	7	316	34.6	5.39	22	8	7	High	1	0.953	0.96	1.152	1.109	1.17	1.193	1.236	1.145	1.182	1.208	1.145	1.302	1.317	1.403
TRINITY_DN66671_c0_g1_i1_orf1	phosphoglucomutase-like [Ostrinia furnacalis]	2	87	9.2	9.41	43	6	2	High	1	0.961	1.023	1.361	1.337	1.242	1.343	1.23	1.381	1.477	1.322	1.424	1.451	1.401	1.259
TRINITY_DN1282_c1_g1_i4_orf1	uncharacterized protein LOC114360659 [Ostrinia furnacalis]	6	580	64.2	8.76	13	9	6	High	1	1.05	1.047	1.054	1.093	1.117	1.075	1.136	1.113	1.162	1.175	1.159	1.087	1.035	1.067
TRINITY_DN2694_c0_g1_i3_orf1	cubilin homolog [Ostrinia furnacalis]	9	1742	196.5	5.49	6	10	9	High	1	1.015	1.064	1.291	1.29	1.279	0.844	0.925	0.932	0.97	0.914	0.898	0.84	0.924	0.91
TRINITY_DN18773_c0_g1_i3_orf1	keratin, type II cytoskeletal 68 kDa, component IB-like [Ostrinia furnacalis]	3	148	13.9	11.84	47	6	3	High	1	0.824	0.93	0.4	0.371	0.405	0.361	0.393	0.383	0.272	0.292	0.417	0.235	0.247	0.286
TRINITY_DN5019_c0_g1_i2_orf1	RRP12-like protein isoform X4 [Ostrinia furnacalis]	8	1382	153.7	9	6	8	8	High	1	1.018	1.006	0.437	0.427	0.44	0.438	0.44	0.426	0.419	0.372	0.393	0.423	0.453	0.453
TRINITY_DN3896_c0_g1_i1_orf1	glyoxalase domain-containing protein 4 [Ostrinia furnacalis]	6	285	31.7	6.18	22	9	6	High	1	1	1.093	1.276	1.255	1.273	1.416	1.387	1.377	1.403	1.388	1.374	1.752	1.713	1.786
TRINITY_DN64892_c0_g1_i1_orf1	aldehyde dehydrogenase X, mitochondrial [Manduca sexta]	5	54	5.7	7.42	59	17	3	High	1	0.917	0.955	1.459	1.615	1.421	1.732	1.967	1.758	1.854	3.234	2.043	1.995	1.786	2.166
TRINITY_DN38274_c0_g1_i1_orf1	uncharacterized protein LOC114360402 [Ostrinia furnacalis]	5	261	29.3	6.43	34	5	5	High	1	0.98	1.086	1.107	0.97	0.858	1.034	1.034	0.953	0.932	0.931	0.87	6.44	6.555	6.064
TRINITY_DN2175_c0_g1_i4_orf1	uncharacterized protein LOC114353827 [Ostrinia furnacalis]	5	160	17.4	7.74	45	11	5	High	1	0.962	1.011	1.694	1.664	1.609	1.379	1.281	1.188	1.41	1.253	1.624	0.89	0.882	0.899
TRINITY_DN1191_c0_g1_i4_orf1	interferon-inducible double-stranded RNA-dependent protein kinase activato	5	381	42.1	8.97	16	11	5	High	1	1.003	0.972	0.505	0.554	0.568	0.565	0.579	0.568	0.52	0.515	0.562	0.56	0.575	0.545
TRINITY_DN8544_c12_g1_i2_orf1	mannosyl-oligosaccharide 1,2-alpha-mannosidase IA-like isoform X1 [Ostrinia	7	668	75	6.2	13	9	6	High	1	0.96	0.97	1.016	1.075	1.014	0.978	1.177	1.088	1.004	1.068	0.902	0.928	0.91	0.976
TRINITY_DN7512_c0_g1_i1_orf1	hypothetical protein evm_010529 [Chilo suppressalis]	7	200	22.1	9.13	50	8	5	High	1	0.974	1.002	0.458	0.425	0.477	0.527	0.52	0.53	0.508	0.536	0.504	0.412	0.409	0.444
TRINITY_DN102260_c0_g1_i1_orf1	unnamed protein product [Diatraea saccharalis]	5	88	9.6	9.86	64	8	3	High	1	1.115	1.052	1.115	1.076	0.971	0.848	0.699	0.746	0.734	0.678	0.832	0.754	0.747	0.692
TRINITY_DN25779_c0_g1_i6_orf1	aldo-keto reductase AKR2E4-like [Ostrinia furnacalis]	5	322	35.9	7.14	23	5	5	High	1	1.021	0.961	0.311	0.328	0.347	0.273	0.277	0.268	0.287	0.319	0.286	0.645	0.698	0.653
TRINITY_DN1093_c0_g1_i6_orf1	uncharacterized protein LOC114361723 isoform X4 [Ostrinia furnacalis]	7	414	47.1	6.14	20	8	4	High	1	1.028	0.997	1.725	1.601	1.891	1.857	1.905	1.993	2.219	2.082	1.808	2.096	2.079	1.995
TRINITY_DN2570_c0_g1_i1_orf1	PREDICTED: pyruvate carboxylase, mitochondrial isoform X1 [Microplitis demo	8	1193	131.4	6.55	7	15	1	High	1	0.899	0.982	1.47	1.486	1.501	1.371	1.808	1.627	1.304	1.352	2.105	1.158	1.117	1.204
TRINITY_DN381_c0_g1_i1_orf1	cuticle protein 8-like [Ostrinia furnacalis]	4	230	25	6.86	22	7	4	High	1	0.945	0.952	0.936	0.996	0.868	0.929	0.952	0.909	0.863	0.671	0.741	9.122	9.341	7.971
TRINITY_DN14987_c0_g1_i3_orf1	hypothetical protein evm_009121 [Chilo suppressalis]	9	2239	248.9	7.02	5	9	9	High	1	0.998	1.048	0.898	0.941	0.891	0.826	0.847	0.819	0.735	0.806	0.783	1.067	1.073	1.074
TRINITY_DN5559_c0_g1_i1_orf1	ferrochelatase, mitochondrial isoform X2 [Ostrinia furnacalis]	10	401	45.2	8.03	22	11	10	High	1	0.945	0.977	0.616	0.618	0.666	0.695	0.687	0.689	0.681	0.712	0.689	0.631	0.672	0.653
TRINITY_DN1957_c0_g1_i4_orf1	NAD kinase 2, mitochondrial [Ostrinia furnacalis]	7	435	49.1	7.83	21	7	7	High	1	0.994	1.028	1.684	1.485	1.58	2.096	2.05	2.109	2.376	2.309	2.226	1.927	1.879	1.894
TRINITY_DN11374_c0_g1_i4_orf1	unnamed protein product [Chilo suppressalis]	9	1207	143.9	7.18	9	9	9	High	1	1.014	0.979	1.103	1.114	1.125	1.1	1.172	1.155	0.95	1.105	0.951	0.967	0.913	0.993
TRINITY_DN56795_c1_g1_i1_orf1	uncharacterized protein LOC114365476 [Ostrinia furnacalis]	5	254	27.1	8.82	27	9	5	High	1	1.015	1.024	0.756	0.803	0.756	1.068	1.062	1.019	0.927	0.907	1.032	0.672	0.727	0.728
TRINITY_DN1266_c6_g1_i1_orf1	transmembrane 9 superfamily member 2 [Ostrinia furnacalis]	7	674	76.1	7.72	11	10	7	High	1	0.977	0.982	0.835	0.919	0.865	0.822	0.889	0.876	0.784	0.81	0.797	0.812	0.77	0.817
TRINITY_DN2861_c0_g2_i1_orf1	proteasome subunit alpha type-5 [Ostrinia furnacalis]	6	224	24.6	5.01	42	14	6	High	1	0.96	0.97	1.337	1.252	1.281	1.287	1.338	1.322	1.329	1.381	1.327	1.486	1.429	1.569
TRINITY_DN59422_c0_g1_i2_orf1	larval cuticle protein LCP-22-like isoform X2 [Pectinophora gossypiella]	4	182	19.5	9.45	38	5	4	High	1	1.019	1.111	2.21	2.314	2.167	2.267	2.203	2.108	1.87	1.612	2.103	0.393	0.386	0.431
TRINITY_DN874_c2_g1_i1_orf1	uncharacterized protein LOC114356358 [Ostrinia furnacalis]	6	394	46.2	7.5	19	8	6	High	1	0.994	0.959	1.813	1.845	1.91	2.181	2.004	2.058	1.681	1.932	1.757	1.584	1.662	1.556
TRINITY_DN582_c0_g1_i5_orf1	hypothetical protein evm_007348 [Chilo suppressalis]	5	1319	146.3	6.18	7	6	3	High	1	1.08	1.097	1.5	1.454	1.439	1.623	1.555	1.627	1.496	1.351	1.563	1.587	1.379	1.232
TRINITY_DN6015_c1_g1_i3_orf1	probable salivary secreted peptide [Ostrinia furnacalis]	7	110	12.3	9.07	61	9	6	High	1	1.008	1.045	1.167	1.248	1.162	1.296	1.358	1.326	1.416	1.478	1.346	1.488	1.457	1.516
TRINITY_DN147676_c0_g1_i1_orf1	PREDICTED: 60S ribosomal protein L23 [Microplitis demolitor]	7	140	14.8	10.58	64	18	4	High	1	0.969	0.944	0.768	0.841	0.827	0.737	0.769	0.747	0.605	0.688	0.641	0.581	0.596	0.606
TRINITY_DN542_c0_g2_i1_orf1	uncharacterized protein LOC114364889 [Ostrinia furnacalis]	4	388	44.5	5.07	16	9	4	High	1	1.012	0.937	0.167	0.157	0.184	0.203	0.207	0.207	0.197	0.218	0.177	0.211	0.205	0.215
TRINITY_DN6423_c0_g1_i6_orf1	phenoloxidase-activating factor 2-like isoform X1 [Ostrinia furnacalis]	5	509	55.7	6.18	13	6	3	High	1	1.07	1.084	2.802	2.997	2.584	2.999	3.184	2.971	2.926	2.759	3.377	5.771	6.222	5.405
TRINITY_DN20710_c0_g1_i2_orf1	plexin A3 [Ostrinia furnacalis]	7	1898	211.9	6.65	5	7	7	High	1	1.04	1.052	0.992	0.988	0.953	1.007	0.995	0.98	1.017	0.966	1.023	1.345	1.337	1.303
TRINITY_DN21559_c0_g2_i1_orf1	protein bicaudal D isoform X3 [Galleria mellonella]	6	548	59.4	5.55	15	7	6	High	1	1.025	0.987	0.733	0.724	0.74	0.765	0.781	0.762	0.758	0.762	0.784	1.037	0.99	1.01
TRINITY_DN1285_c0_g1_i6_orf1	bifunctional 3'-phosphoadenosine 5'-phosphosulfate synthase isoform X3 [Os	5	390	43.5	7.71	16	9	5	High	1	0.931	0.956	0.436	0.433	0.457	0.397	0.433	0.399	0.353	0.367	0.338	0.429	0.446	0.418
TRINITY_DN3769_c0_g1_i1_orf1	uncharacterized protein LOC114360562 [Ostrinia furnacalis]	8	384	45.3	10.4	20	10	8	High	1	0.967	1.016	1.011	1.022	0.983	1.064	1.105	1.072	0.959	0.921	0.961	0.796	0.823	0.849
TRINITY_DN42903_c0_g1_i4_orf1	rab GTPase-binding effector protein 1 isoform X1 [Ostrinia furnacalis]	7	597	66.8	5.36	15	7	7	High	1	0.985	1.02	0.863	0.861	0.795	0.785	0.862	0.814	0.807	0.741	0.82	0.934	0.896	0.875
TRINITY_DN3332_c0_g1_i11_orf1	glutathione S-transferase sigma3 [Glyphodes pyloalis]	8	200	23.1	6.23	45	20	2	High	1	0.961	1.014	0.263	0.221	0.27	0.273	0.279	0.282	0.28	0.436	0.271	0.354	0.316	0.341
TRINITY_DN5867_c0_g1_i1_orf1	NADH dehydrogenase [ubiquinone] 1 alpha subcomplex subunit 7-like [Ostrin	6	110	12.3	9.92	60	9	6	High	1	1.019	0.995	0.373	0.412	0.439	0.396	0.409	0.403	0.395	0.364	0.42	0.48	0.519	0.484
TRINITY_DN120593_c0_g1_i1_orf1	SUMO-activating enzyme subunit 1 [Ostrinia furnacalis]	6	339	38.1	5.57	20	9	6	High	1	0.97	1.005	0.848	0.89	0.888	0.84	0.876	0.888	0.835	0.803	0.813	1.155	1.114	1.149
TRINITY_DN2177_c0_g1_i1_orf1	uncharacterized protein LOC114349824 isoform X1 [Ostrinia furnacalis]	4	831	89.7	6.39	6	9	4	High	1	1.012	1.012	0.694	0.682	0.744	0.73	0.701	0.808	0.855	0.817	0.804	0.672	0.675	0.677
TRINITY_DN10716_c1_g1_i1_orf1	apoptosis-inducing factor 3-like [Ostrinia furnacalis]	8	602	65.4	8.4	15	9	8	High	1	0.963	0.973	0.804	0.81	0.764	0.773	0.808	0.771	0.767	0.827	0.713	0.735	0.719	0.796
TRINITY_DN13746_c0_g1_i5_orf1	unnamed protein product [Chilo suppressalis]	6	1464	158.8	8.91	6	7	6	High	1	1.006	0.977	0.986	1.028	1.071	1.082	1.109	1.103	1.01	1.142	1.11	1.008	1.064	1.019
TRINITY_DN8651_c0_g1_i18_orf1	glutathione S-transferase theta 2 [Conogethes punctiferalis]	5	144	16.8	7.62	39	6	2	High	1	1.039	1.111	1.497	1.562	1.556	1.347	1.322	1.399	0.962	0.996	1.008	0.594	0.602	0.619
TRINITY_DN3235_c0_g1_i1_orf1	SPARC [Trichoplusia ni]	6	317	36.9	5.01	24	8	6	High	1	1.039	0.964	0.246	0.23	0.27	0.25	0.279	0.273	0.218	0.224	0.215	0.758	0.795	0.717
TRINITY_DN3836_c0_g1_i4_orf1	2-oxoisovalerate dehydrogenase subunit alpha, mitochondrial [Ostrinia furnac	8	432	48.9	6.86	21	9	8	High	1	1.012	0.973	0.546	0.527	0.563	0.628	0.64	0.618	0.464	0.445	0.484	0.426	0.429	0.397
TRINITY_DN5950_c0_g1_i4_orf1	retinol dehydrogenase 14-like [Ostrinia furnacalis]	7	337	38	9.52	28	8	7	High	1	0.993	1.033	0.941	0.907	0.878	1.011	0.972	1.103	0.936	0.887	0.9	0.925	0.853	0.867
TRINITY_DN805_c0_g1_i5_orf1	serine protease snake-like [Ostrinia furnacalis]	6	400	43.9	5.53	23	6	6	High	1	1.035	1.129	2.198	2.101	2.034	2.401	2.212	2.255	2.41	2.261	2.467	2.508	2.836	2.293
TRINITY_DN2170_c1_g1_i3_orf1	beta-1,3-glucan-binding protein-like [Ostrinia furnacalis]	4	191	21.5	6.2	30	5	4	High	1	1.063	0.942	1.497	1.453	1.454	1.516	1.621	1.528	1.758	1.749	1.549	2.459	2.601	2.455
TRINITY_DN8245_c0_g1_i3_orf1	uncharacterized protein LOC114357622 [Ostrinia furnacalis]	5	112	12.5	8.9	62	6	2	High	1	1.01	1	1.772	1.883	1.645	1.765	1.657	1.825	2.027	1.858	2.002	1.914	2.015	1.996
TRINITY_DN1376_c0_g1_i4_orf1	phosphatidylinositol 4-kinase type 2-beta [Ostrinia furnacalis]	6	452	51.7	6.93	17	7	6	High	1	0.976	0.945	0.995	0.951	0.911	0.913	0.943	0.996	0.977	0.974	0.897	0.98	1.01	0.995
TRINITY_DN13615_c0_g1_i3_orf1	calcium-binding protein P-like [Ostrinia furnacalis]	6	269	28.8	7.97	30	8	6	High	1	0.963	1.081	1.32	1.447	1.425	1.488	1.282	1.435	1.506	1.274	1.462	1.433	1.461	1.468
TRINITY_DN829_c0_g1_i8_orf1	cytochrome P450 6B6-like [Ostrinia furnacalis]	7	515	58.5	8.09	16	9	5	High	1	0.974	0.988	1.316	1.227	1.231	1.605	1.586	1.655	1.201	1.275	1.265	0.844	0.791	0.84
TRINITY_DN43942_c0_g1_i1_orf1	LOW QUALITY PROTEIN: caprin homolog [Ostrinia furnacalis]	6	413	45.3	5.16	20	8	6	High	1	1.039	1.027	0.482	0.511	0.532	0.471	0.426	0.425	0.434	0.702	0.425	0.615	0.584	0.586
TRINITY_DN4272_c0_g1_i1_orf1	MICOS complex subunit MIC19-like [Ostrinia furnacalis]	6	167	18.8	7.68	48	10	6	High	1	1	0.964	0.759	0.748	0.772	0.803	0.803	0.738	0.652	0.648	0.71	0.726	0.729	0.674
TRINITY_DN19034_c0_g1_i1_orf1	tRNA-splicing ligase RtcB homolog [Ostrinia furnacalis]	6	506	55.7	8.53	15	8	6	High	1	0.98	1.037	1.096	1.142	1.124	1.059	1.087	1.078	0.928	0.974	1.003	1.206	1.22	1.151
TRINITY_DN64015_c0_g1_i1_orf1	asparagine synthetase [glutamine-hydrolyzing] [Ostrinia furnacalis]	8	551	62.5	6.71	17	10	8	High	1	0.973	0.956	0.846	0.872	0.896	0.855	0.82	0.798	0.822	0.835	0.855	0.913	0.899	0.858
TRINITY_DN4069_c0_g1_i5_orf1	putative sulfiredoxin [Ostrinia furnacalis]	4	129	14.6	8.62	47	7	4	High	1	0.974	1.007	2.146	2.129	2.092	2.243	2.022	2.219	1.618	1.519	1.578	1.326	1.326	1.273
TRINITY_DN23444_c0_g1_i11_orf1	serrate RNA effector molecule homolog isoform X3 [Ostrinia furnacalis]	10	619	70.5	6.2	16	11	5	High	1	1.039	1.178	0.792	0.953	1.029	0.749	0.708	1	0.654	0.894	0.566	0.728	1.573	0.738
TRINITY_DN307_c1_g1_i1_orf1	uncharacterized protein LOC114356704 [Ostrinia furnacalis]	3	96	10.4	8.66	35	15	3	High	1	1.068	1.02	1.24	1.213	1.258	3.168	3.022	2.803	2.225	1.825	2.344	2.106	2.113	1.935
TRINITY_DN14487_c0_g1_i4_orf1	hypothetical protein HW555_009956 [Spodoptera exigua]	6	501	54.6	6.96	14	8	6	High	1	1.011	1.002	0.546	0.558	0.577	0.523	0.522	0.546	0.561	0.617	0.602	0.751	0.708	0.738
TRINITY_DN3616_c0_g1_i4_orf1	conotoxin ArMKLT2-032-like [Ostrinia furnacalis]	3	56	6.1	5.29	68	8	1	High	1	0.952	0.836	2.349	2.202	2.043	1.731	1.364	1.423	3.646	2.701	4.155	6.181	5.815	4.584
TRINITY_DN13312_c0_g2_i1_orf1	von Willebrand factor A domain-containing protein 8 [Trichoplusia ni]	9	884	99.5	8.34	12	9	9	High	1	0.941	0.976	0.938	0.991	0.881	0.877	0.866	0.858	0.893	0.983	0.875	0.651	0.644	0.728
TRINITY_DN1391_c0_g1_i29_orfp1	TRINITY_DN1391_c0_g1_i29_m.70767 TRINITY_DN1391_c0_g1::TRINITY_DN139	6	494	52.3	8.06	14	9	5	High	1	1.035	1.101	3.833	3.989	3.713	4.05	2.942	3.47	3.497	2.784	3.912	2.562	2.551	2.298
TRINITY_DN95665_c0_g1_i1_orf1	hypothetical protein HW555_003264 [Spodoptera exigua]	3	122	13.9	8.28	30	15	3	High	1	1.025	1.011	0.588	0.613	0.63	0.538	0.585	0.54	0.656	0.58	0.654	0.625	0.622	0.616
TRINITY_DN4456_c0_g1_i1_orf1	uncharacterized protein LOC114353382 isoform X1 [Ostrinia furnacalis]	6	374	41	6.46	20	8	6	High	1	0.992	0.983	0.894	0.989	0.971	1.035	1.172	1.147	0.905	0.878	0.826	1.237	1.139	1.144
TRINITY_DN135_c0_g1_i1_orf1	60S ribosomal protein L11 [Nymphalis io]	5	201	23.1	10.26	20	9	4	High	1	0.914	0.966	0.683	0.702	0.679	0.677	0.696	0.692	0.632	0.722	0.679	0.531	0.481	0.568
TRINITY_DN38301_c0_g1_i2_orf1	gamma-taxilin [Ostrinia furnacalis]	7	629	71.1	5.48	12	7	7	High	1	0.999	0.985	0.437	0.432	0.502	0.484	0.453	0.499	0.485	0.464	0.474	0.593	0.567	0.566
TRINITY_DN11973_c0_g1_i1_orf1	nuclear pore complex protein Nup93-like [Ostrinia furnacalis]	7	824	93.3	6.42	11	7	7	High	1	1.019	1.106	0.84	0.89	0.861	0.847	0.812	0.83	0.812	0.748	0.794	0.83	0.869	0.807
TRINITY_DN23204_c0_g1_i1_orf1	LOW QUALITY PROTEIN: uncharacterized protein LOC114350452 [Ostrinia furr	5	280	30.4	5.43	21	6	5	High	1	1.01	0.963	0.713	0.713	0.745	0.846	0.831	0.795	0.845	0.763	0.852	0.562	0.686	0.634
TRINITY_DN36893_c0_g1_i1_orf1	40S ribosomal protein S15 [Bicyclus anynana]	5	147	17	10.43	59	7	5	High	1	0.9	0.941	0.812	0.815	0.795	0.626	0.609	0.643	0.574	0.593	0.584	0.608	0.61	0.618
TRINITY_DN1386_c0_g1_i6_orf1	ras-related protein Rab-36 [Ostrinia furnacalis]	7	495	54.1	6.42	13	8	7	High	1	0.973	0.97	0.447	0.44	0.487	0.538	0.507	0.571	0.478	0.497	0.431	0.645	0.604	0.62
TRINITY_DN1947_c0_g1_i6_orf1	insulin-degrading enzyme [Ostrinia furnacalis]	10	1008	114.3	6.46	10	11	10	High	1	1.016	0.994	0.732	0.749	0.802	0.779	0.809	0.758	0.739	0.716	0.709	0.812	0.799	0.829
TRINITY_DN29018_c0_g1_i4_orf1	prostaglandin reductase 1-like isoform X1 [Ostrinia furnacalis]	7	335	36.5	6.89	24	7	7	High	1	0.977	0.949	0.154	0.19	0.211	0.171	0.172	0.182	0.241	0.248	0.222	0.146	0.151	0.156
TRINITY_DN28806_c0_g1_i1_orf1	ATP-dependent RNA helicase WM6 [Ostrinia furnacalis]	5	426	48.7	5.64	15	7	5	High	1	0.975	0.975	0.574	0.604	0.65	0.617	0.606	0.699	0.578	0.566	0.537	0.791	0.611	0.625
TRINITY_DN18593_c0_g1_i1_orf1	60S ribosomal protein L22-like [Ostrinia furnacalis]	7	147	16.8	9.82	48	12	7	High	1	1.006	0.997	0.877	0.953	0.815	0.747	0.745	0.713	0.668	0.639	0.777	0.735	0.685	0.684

TRINITY_DN50225_c0_g1_i1_orf1	SRSF protein kinase 3 [Galleria mellonella]	4	605	68.5	6.19	6	4	4	High	1	1.013	0.99	0.792	0.766	0.787	0.716	0.697	0.775	0.698	0.878	0.686	1.016	0.963	0.975
TRINITY_DN32_c0_g1_i4_orf1	epidermal growth factor receptor substrate 15 homolog [Ostrinia furnacalis]	6	309	34.9	4.93	23	7	6	High	1	0.952	0.997	0.724	0.834	0.725	1.166	1.228	1.139	0.941	0.941	0.887	0.648	0.631	0.632
TRINITY_DN8306_c0_g1_i4_orf1	NAD(P) transhydrogenase, mitochondrial-like [Ostrinia furnacalis]	6	834	86.1	5.68	10	7	6	High	1	1.029	0.937	1.048	1.144	1.023	1.141	1.036	1.077	0.906	0.893	0.846	1.457	1.432	1.344
TRINITY_DN246_c1_g1_i5_orf1	lachesin isoform X1 [Ostrinia furnacalis]	6	368	41	6.92	22	7	6	High	1	0.926	0.949	1.025	1.037	1.048	1.099	1.064	1.199	1.176	1.141	1.138	1.442	1.492	1.361
TRINITY_DN9435_c0_g1_i7_orf1	uncharacterized protein LOC114350197 [Ostrinia furnacalis]	5	154	16.8	6.32	34	6	5	High	1	0.941	0.949	1.604	1.723	1.506	1.673	1.811	1.57	2.164	2.245	2.716	1.145	1.14	1.307
TRINITY_DN14073_c0_g1_i1_orf1	cytochrome c oxidase subunit 4 isoform 1, mitochondrial-like [Ostrinia furnaca	6	174	20.2	9.79	47	7	6	High	1	1.036	0.997	0.476	0.451	0.501	0.524	0.482	0.533	0.644	0.66	0.585	0.523	0.53	0.535
TRINITY_DN1073_c0_g1_i4_orf1	carboxylesterase [Loxostege sticticalis]	6	589	65.7	4.91	14	8	5	High	1	0.969	0.925	0.273	0.288	0.341	0.335	0.373	0.345	0.306	0.331	0.298	0.308	0.32	0.314
TRINITY_DN13186_c0_g1_i1_orf1	NADH dehydrogenase [ubiquinone] iron-sulfur protein 5-like [Bicyclus anynan	3	100	11.7	8.48	48	4	3	High	1	0.973	0.973	0.613	0.595	0.622	0.539	0.599	0.558	0.675	0.738	0.726	0.588	0.567	0.572
TRINITY_DN21035_c0_g1_i14_orf1	mitochondrial amidoxime reducing component 2-like [Ostrinia furnacalis]	6	138	15.6	7.78	41	9	1	High	1	0.953	0.915	0.791	0.785	0.893	0.595	0.603	0.646	0.277	0.226	0.239	0.248	0.244	0.268
TRINITY_DN21909_c0_g1_i1_orf1	complement component 1 Q subcomponent-binding protein, mitochondrial [(	6	288	31.9	5.2	24	7	6	High	1	1.039	1.011	0.39	0.389	0.417	0.381	0.378	0.374	0.395	0.351	0.363	0.39	0.423	0.389
TRINITY_DN13093_c0_g1_i2_orf1	enhancer of mRNA-decapping protein 4 [Ostrinia furnacalis]	8	1001	107	5.2	7	9	8	High	1	0.987	1.013	0.813	0.809	0.851	0.876	0.896	0.84	0.726	0.689	0.761	0.874	0.905	0.919
TRINITY_DN48410_c0_g2_i1_orf1	alpha-amylase 2-like isoform X3 [Ostrinia furnacalis]	8	502	56.5	6.68	15	9	8	High	1	1.006	0.944	0.193	0.192	0.233	0.24	0.242	0.234	0.248	0.242	0.238	0.215	0.21	0.222
TRINITY_DN35635_c0_g1_i1_orf1	probable NADH dehydrogenase [ubiquinone] 1 alpha subcomplex subunit 12 [	5	143	16.9	9.09	44	11	5	High	1	0.987	0.976	0.429	0.424	0.459	0.345	0.335	0.322	0.397	0.384	0.419	0.501	0.483	0.471
TRINITY_DN64230_c0_g1_i1_orf1	serine/threonine-protein phosphatase 2B catalytic subunit 3-like [Ostrinia furn	9	495	55.9	6.43	20	9	9	High	1	0.999	1.022	0.941	0.917	0.983	0.969	1.014	0.997	0.929	0.866	0.913	0.927	0.942	0.91
TRINITY_DN2193_c0_g1_i7_orf1	long-chain-fatty-acid--CoA ligase 5 isoform X1 [Ostrinia furnacalis]	9	692	77.2	8	14	11	9	High	1	0.984	0.984	1.358	1.397	1.338	1.297	1.385	1.346	1.101	1.057	1.055	0.824	0.796	0.824
TRINITY_DN18374_c0_g1_i1_orf1	L-2-hydroxyglutarate dehydrogenase, mitochondrial [Ostrinia furnacalis]	8	445	49.4	8.85	22	10	8	High	1	1.039	1.002	1.525	1.443	1.498	1.509	1.486	1.521	1.633	1.791	1.439	1.403	1.394	1.387
TRINITY_DN12193_c0_g1_i2_orf1	carbonyl reductase [NADPH] 1-like [Ostrinia furnacalis]	7	274	29.9	7.53	34	9	3	High	1	1.052	0.986	0.836	0.839	0.883	0.877	0.986	0.94	0.947	0.819	0.942	0.937	0.899	0.894
TRINITY_DN13799_c0_g1_i1_orf1	uncharacterized protein LOC116345248 [Contarinia nasturtii]	6	142	15.8	6.3	44	11	6	High	1	1.008	0.988	1.908	1.902	1.795	3.639	3.786	3.567	4.597	4.151	4.857	3.059	3.191	3.092
TRINITY_DN18502_c0_g1_i1_orf1	uncharacterized protein LOC114359515 [Ostrinia furnacalis]	6	554	59.7	4.87	15	8	6	High	1	1	0.922	0.497	0.537	0.537	0.504	0.562	0.512	0.476	0.489	0.484	3.119	3.18	2.846
TRINITY_DN8317_c1_g2_i8_orf1	PREDICTED: guanine nucleotide-binding protein G(I)/G(S)/G(T) subunit beta-1	7	340	37.2	6.28	26	12	7	High	1	0.987	1.009	1.039	1.01	1.024	1.049	1.094	1.036	0.968	0.902	0.974	1.074	1.156	1.153
TRINITY_DN757_c3_g1_i2_orf1	PREDICTED: galectin-4-like [Amyelois transitella]	6	236	24.8	5.06	28	16	6	High	1	0.98	0.958	1.402	1.456	1.441	1.446	1.498	1.444	1.357	1.353	1.355	1.078	1.119	1.114
TRINITY_DN3906_c0_g1_i5_orf1	ejaculatory bulb-specific protein 3-like [Ostrinia furnacalis]	7	119	13.2	5.85	44	9	7	High	1	0.966	0.986	1.191	1.268	1.187	1.02	1.041	0.965	0.738	0.733	0.866	0.578	0.552	0.586
TRINITY_DN27670_c0_g1_i4_orf1	dynactin subunit 2 [Ostrinia furnacalis]	6	369	41.1	5.14	19	6	6	High	1	1.002	0.981	1.01	1.019	1.048	0.992	0.999	0.962	0.958	0.927	0.969	1.094	1.101	1.101
TRINITY_DN120089_c0_g1_i1_orf1	phosphoglucomutase [Ostrinia furnacalis]	6	123	13.3	5.82	66	8	6	High	1	0.954	1.02	1.258	1.255	1.2	1.294	1.34	1.204	1.146	1.25	1.232	0.903	0.897	0.882
TRINITY_DN11464_c0_g1_i3_orf1	unnamed protein product [Spodoptera littoralis]	7	507	56.4	5.2	15	8	7	High	1	1.017	1.034	0.784	0.929	0.81	0.855	0.786	0.842	0.805	0.77	0.87	1.008	1.042	1.054
TRINITY_DN2040_c0_g1_i6_orf1	trypsin-like serine proteinase T26 protein, partial [Chilo infuscatellus]	5	340	37.1	7.06	17	8	2	High	1	1.016	1.129	2.635	2.557	2.636	3.027	2.935	3.048	3.374	2.625	2.919	1.632	1.554	1.491
TRINITY_DN15318_c0_g1_i1_orf1	hepatoma-derived growth factor-related protein 2-like [Ostrinia furnacalis]	7	330	36.9	9.09	27	11	7	High	1	1.054	0.981	0.95	1.049	1.043	0.898	0.963	0.9	0.763	0.69	0.771	0.714	0.735	0.713
TRINITY_DN59852_c0_g1_i1_orf1	hypothetical protein evm_008421 [Chilo suppressalis]	4	100	11.3	7.18	45	6	4	High	1	0.965	0.988	1.314	1.453	1.319	1.318	1.368	1.258	1.257	1.042	1.288	1.072	1.066	0.992
TRINITY_DN4408_c6_g1_i1_orf1	polyprotein, partial [Bemisia tabaci]	6	674	76.2	6.64	10	7	6	High	1	1.058	1.025	1.097	1.047	1.064	1.125	1.106	1.05	0.56	0.531	0.595	0.429	0.368	0.385
TRINITY_DN57536_c0_g1_i14_orf1	hypothetical protein O3G_MSEX012216 [Manduca sexta]	7	145	15.8	6.92	38	10	5	High	1	1.033	1.025	1.173	1.107	1.209	1.249	1.13	1.291	1.233	1.123	1.072	1.254	1.289	1.194
TRINITY_DN5495_c0_g1_i5_orf1	multiple coagulation factor deficiency protein 2 homolog isoform X1 [Ostrinia	6	226	26.2	6.06	31	6	6	High	1	1.033	1.064	1.232	1.21	1.113	1.246	1.169	1.186	1.142	0.993	1.162	1.472	1.415	1.342
TRINITY_DN1935_c0_g1_i1_orf1	adult-specific cuticular protein ACP-22-like [Ostrinia furnacalis]	7	165	19.3	5.73	38	10	6	High	1	0.959	1.023	0.857	0.811	0.795	0.763	0.772	0.728	0.748	0.818	0.731	4.49	4.522	4.258
TRINITY_DN2574_c0_g1_i5_orf1	prion-like-(Q/N-rich) domain-bearing protein 25 [Ostrinia furnacalis]	4	326	34.9	5.95	20	4	4	High	1	1.021	0.966	0.314	0.308	0.348	0.363	0.436	0.361	0.357	0.286	0.381	0.294	0.305	0.286
TRINITY_DN1491_c0_g1_i4_orf1	GILT-like protein 2 isoform X1 [Ostrinia furnacalis]	4	238	26.5	6.87	16	9	1	High	1	0.806	0.849	2.169	2.038	2.308	1.027	0.626	0.882	1.462	1.404	1.64	2.968	3.015	3.247
TRINITY_DN35245_c0_g1_i1_orf1	ras GTPase-activating protein-binding protein 2 isoform X1 [Nymphalis io]	7	188	20.7	6.8	36	8	7	High	1	0.995	1.02	0.801	0.78	0.786	0.637	0.671	0.658	0.674	0.699	0.724	0.812	0.824	0.818
TRINITY_DN36144_c0_g1_i3_orf1	nicotinate phosphoribosyltransferase isoform X1 [Ostrinia furnacalis]	8	599	66.9	7.24	16	8	8	High	1	1.014	1.031	1.361	1.493	1.271	1.5	1.693	1.601	2.121	2.414	2.426	1.899	1.539	1.86
TRINITY_DN8511_c0_g1_i1_orf1	NADH dehydrogenase [ubiquinone] 1 beta subcomplex subunit 10 [Ostrinia fu	5	158	19.1	7.36	41	6	5	High	1	1.015	0.984	0.556	0.622	0.559	0.522	0.521	0.549	0.548	0.492	0.551	0.523	0.508	0.495
TRINITY_DN22046_c1_g1_i5_orf1	uncharacterized protein LOC114351208 [Ostrinia furnacalis]	6	428	49.1	8.15	17	7	6	High	1	0.956	0.957	1.217	1.24	1.248	1.639	1.615	1.565	1.191	1.174	1.248	1.065	1.071	1.083
TRINITY_DN679_c0_g1_i2_orf1	cytochrome b-c1 complex subunit 7-like [Ostrinia furnacalis]	6	108	13.2	7.94	44	12	6	High	1	1.015	0.961	0.356	0.349	0.39	0.318	0.311	0.32	0.328	0.335	0.313	0.326	0.363	0.327
TRINITY_DN1194_c0_g1_i5_orf1	sequestosome-1-like isoform X4 [Ostrinia furnacalis]	6	590	64.8	6.09	12	9	6	High	1	0.965	1.015	1.765	1.612	1.695	1.151	1.198	1.198	1.151	1.055	1.343	1.867	1.95	2.193
TRINITY_DN9079_c0_g1_i5_orf1	UDP-glucuronosyltransferase-like [Ostrinia furnacalis]	5	428	48.5	9.03	14	6	5	High	1	0.902	0.837	3.507	3.266	3.212	3.272	3.355	3.378	3.61	3.719	3.313	2.067	2.135	2.386
TRINITY_DN2438_c0_g1_i1_orf1	dystrophin, isoforms A/C/F/G/H-like [Ostrinia furnacalis]	8	2756	308.9	5.47	3	11	5	High	1	0.987	0.952	0.886	0.88	0.829	1.052	1.148	1.068	0.883	0.901	0.806	1.314	1.279	1.222
TRINITY_DN82_c0_g1_i1_orf1	vacuolar protein sorting-associated protein 26B-like [Ostrinia furnacalis]	5	436	49.6	6.44	13	7	5	High	1	0.934	0.998	1.173	1.148	1.172	0.948	0.975	1.05	0.936	0.912	0.871	1.07	1.032	1.082
TRINITY_DN2876_c0_g1_i5_orf1	long-chain fatty acid transport protein 4-like isoform X1 [Ostrinia furnacalis]	4	680	75.7	8.75	7	5	1	High	1	1.014	1.065	0.735	0.801	0.8	0.596	0.771	0.726	0.84	0.704	0.911	0.736	0.716	0.754
TRINITY_DN2783_c0_g1_i22_orf1	methionine aminopeptidase 1-like [Pectinophora gossypiella]	5	381	42.2	6.71	17	7	5	High	1	0.969	0.947	0.728	0.771	0.803	0.762	0.764	0.791	0.668	0.657	0.612	0.699	0.668	0.645
TRINITY_DN1355_c0_g1_i5_orf1	hypothetical protein evm_006664 [Chilo suppressalis]	4	209	21.1	8.21	17	17	3	High	1	0.985	1.035	1.157	1.191	1.143	1.063	1.104	1.082	0.982	0.927	1.114	0.824	0.839	0.807
TRINITY_DN23740_c0_g1_i3_orf1	ras-related protein Rab-14 [Ostrinia furnacalis]	5	213	23.7	7.36	38	8	4	High	1	0.976	0.994	1.146	1.215	1.15	1.071	1.168	1.153	1.093	1.061	1.073	1.208	1.287	1.287
TRINITY_DN1814_c0_g1_i11_orf1	titin-like, partial [Ostrinia furnacalis]	5	584	66.6	5.64	15	6	4	High	1	1.08	1.095	1.097	1.054	0.976	1.045	0.94	1.016	0.834	0.714	0.907	0.515	0.506	0.546
TRINITY_DN5873_c0_g4_i1_orf1	hypothetical protein evm_003048 [Chilo suppressalis]	5	169	19.1	10.77	38	9	5	High	1	1.001	1.01	0.806	0.818	0.76	0.766	0.672	0.747	0.623	0.594	0.658	0.621	0.658	0.568
TRINITY_DN1391_c1_g2_i2_orf1	uncharacterized protein LOC119837640 isoform X2 [Zerene cesonia]	6	312	35.1	9.29	24	7	6	High	1	0.971	1.033	1.19	1.238	1.182	1.118	1.172	1.106	0.995	0.888	1.113	0.566	0.558	0.608
TRINITY_DN1503_c0_g1_i6_orf1	ecdysteroid-regulated 16 kDa protein [Ostrinia furnacalis]	6	157	17.1	7.61	38	12	6	High	1	0.976	0.948	2.437	2.641	2.355	1.811	2.009	1.901	2.106	1.978	2.281	1.639	1.697	1.726
TRINITY_DN1741_c0_g1_i5_orf1	gephyrin isoform X1 [Ostrinia furnacalis]	6	616	66.4	6.54	14	6	6	High	1	0.933	0.953	1.207	1.252	1.204	1.131	1.203	1.192	1.219	1.036	1.164	1.004	0.976	0.959
TRINITY_DN53115_c0_g1_i1_orf1	small glutamine-rich tetratricopeptide repeat-containing protein beta-like [Os	3	289	31.3	5.43	17	4	3	High	1	0.994	1.006	0.763	0.749	0.684	0.661	0.714	0.713	0.737	0.709	0.645	0.837	0.884	0.845
TRINITY_DN3260_c0_g1_i6_orf1	vesicular integral-membrane protein VIP36 isoform X1 [Ostrinia furnacalis]	4	327	37.4	6.99	17	6	3	High	1	1.046	1.043	1.002	0.963	0.949	0.827	0.871	0.8	0.745	0.767	0.778	0.877	0.815	0.937
TRINITY_DN3600_c0_g1_i1_orf1	unnamed protein product [Chilo suppressalis]	6	369	41.5	6.73	21	6	6	High	1	1.053	1.058	1.258	1.232	1.325	1.087	1.194	1.113	1.271	1.198	1.36	1.437	1.434	1.464
TRINITY_DN14094_c0_g1_i1_orfp1	TRINITY_DN14094_c0_g1_i1_m.76391 TRINITY_DN14094_c0_g1::TRINITY_DN14	5	119	13.5	8.95	48	7	5	High	1	0.924	1.001	0.638	0.789	0.656	0.744	0.83	0.697	0.882	0.888	1.233	0.4	0.4	0.468
TRINITY_DN18273_c0_g1_i4_orf1	venom protease-like [Ostrinia furnacalis]	7	432	48.3	7.15	22	8	7	High	1	1.019	1.089	1.198	1.17	1.213	1.24	1.439	1.381	1.77	1.797	1.562	1.455	1.339	1.44
TRINITY_DN29100_c0_g1_i2_orf1	endocuticle structural glycoprotein ABD-5-like [Galleria mellonella]	3	104	11.2	4.83	61	5	3	High	1	1.001	0.964	0.907	0.952	0.897	1.039	1.041	0.974	0.938	0.779	0.935	0.422	0.378	0.494
TRINITY_DN101658_c0_g1_i1_orf1	uncharacterized protein LOC114349955 [Ostrinia furnacalis]	6	401	43.4	6.55	16	8	6	High	1	0.924	0.981	1.183	1.049	1.143	1.163	1.103	1.234	1.184	1.286	1.048	1.03	0.966	0.962
TRINITY_DN7919_c0_g1_i4_orf1	UDP-N-acetylglucosamine--peptide N-acetylglucosaminyltransferase 110 kDa	6	1058	117.7	6.81	7	7	5	High	1	0.949	0.977	0.862	0.956	0.908	0.905	0.985	0.942	0.865	0.96	0.783	0.81	0.77	0.799
TRINITY_DN126648_c0_g1_i1_orf1	elongation factor 1 alpha, partial [Spodoptera exigua]	3	100	10.9	8.68	44	9	2	High	1	0.993	1.002	0.875	0.756	0.798	0.818	0.792	0.797	0.598	0.551	0.605	0.675	0.661	0.593
TRINITY_DN50517_c0_g1_i3_orf1	PREDICTED: larval cuticle protein A2B-like [Amyelois transitella]	5	182	18.7	6.92	54	7	2	High	1	0.916	1.041	0.879	1.038	0.874	0.913	1.077	1.036	1.08	1.189	0.997	1.185	1.266	1.303
TRINITY_DN1816_c0_g1_i5_orf1	nuclear pore complex protein Nup205 [Ostrinia furnacalis]	7	1457	159.1	7.49	6	7	7	High	1	0.925	1.181	0.801	0.926	0.875	0.88	0.845	0.696	0.731	0.79	0.856	0.799	0.868	0.812
TRINITY_DN364_c1_g1_i2_orf1	talin-2-like, partial [Ostrinia furnacalis]	7	766	81.6	5.22	9	7	7	High	1	1.04	0.979	2.758	2.918	2.836	1.909	1.921	1.926	2.17	1.885	2.083	2.637	2.689	2.593
TRINITY_DN644_c0_g1_i1_orf1	cuticle protein 19-like [Ostrinia furnacalis]	4	131	14.8	6.84	34	8	4	High	1	0.989	0.934	0.647	0.545	0.539	0.606	0.716	0.621	0.564	0.436	0.42	7.811	8.425	7.211
TRINITY_DN83150_c0_g1_i1_orf1	fructose-bisphosphate aldolase-like isoform X1 [Ostrinia furnacalis]	4	90	10	4.84	51	7	1	High	1	0.916	0.968	1.003	0.866	1.01	1.279	1.273	1.308	1.087	0.971	1.028	1.13	1.125	1.233
TRINITY_DN11637_c0_g1_i1_orf1	ribosome-binding protein 1 isoform X4 [Colias croceus]	2	90	10	4.75	49	3	1	High	1	1.017	0.962	0.856	1.02	0.995	0.815	0.913	0.921	0.537	0.782	0.638	0.817	0.735	0.728
TRINITY_DN35099_c0_g1_i1_orf1	transmembrane emp24 domain-containing protein 2 [Ostrinia furnacalis]	5	206	23.3	5.3	30	8	5	High	1	0.947	0.979	1.001	1.002	0.946	0.872	0.884	0.901	0.839	0.772	0.764	1.02	1.019	1.059
TRINITY_DN972_c0_g2_i1_orf1	DNA damage-binding protein 1 [Ostrinia furnacalis]	8	529	58.5	5.77	16	9	5	High	1	0.976	0.991	0.686	0.647	0.728	0.67	0.609	0.835	0.675	0.592	0.578	0.867	0.779	0.836
TRINITY_DN994_c0_g1_i2_orf1	extensin-like isoform X2 [Ostrinia furnacalis]	6	723	81.2	9.8	14	8	6	High	1	1.004	0.971	0.908	0.963	0.926	0.911	0.869	0.937	0.726	1.098	0.845	0.882	0.885	0.847
TRINITY_DN3970_c0_g1_i1_orf1	hypothetical protein evm_002369 [Chilo suppressalis]	5	118	12.3	5.8	45	9	5	High	1	1.021	1.048	0.903	0.907	0.899	0.778	0.843	0.835	0.749	0.732	0.739	0.664	0.686	0.666
TRINITY_DN12661_c0_g1_i3_orf1	T-complex protein 11-like protein 1 [Ostrinia furnacalis]	8	492	55.5	5.59	17	10	8	High	1	1.015	1.056	1.691	2.142	1.689	1.37	1.37	1.473	1.315	1.241	1.451	1.812	1.657	1.588
TRINITY_DN10994_c0_g1_i4_orf1	trypsin inhibitor-like [Ostrinia furnacalis]	2	73	8.1	7.55	49	6	2	High	1	0.988	1.087	1.948	1.908	2.041	2.289	2.017	2.154	2.419	2.074	2.198	1.327	1.346	1.311
TRINITY_DN1956_c1_g1_i5_orf1	uncharacterized protein LOC114356377 [Ostrinia furnacalis]	8	763	87	7.03	12	9	8	High	1	0.987	0.959	0.605	0.618	0.665	0.573	0.621	0.599	0.52	0.574	0.523	0.603	0.564	0.614
TRINITY_DN6248_c0_g1_i1_orf1	DNA topoisomerase I, mitochondrial [Ostrinia furnacalis]	8	947	110.1	8.94	9	8	8	High	1	1.027	1	0.377	0.356	0.429	0.373	0.383	0.381	0.372	0.416	0.341	0.623	0.599	0.586
TRINITY_DN364_c0_g1_i2_orf1	uncharacterized protein LOC114366657, partial [Ostrinia furnacalis]	6	528	58.1	5.44	13	7	6	High	1	0.997	1.07	1.731	1.753	1.758	1.439	1.423	1.413	1.423	1.298	1.438	1.661	1.652	1.543
TRINITY_DN95713_c0_g1_i1_orf1	SH3 domain-binding glutamic acid-rich protein homolog [Zerene cesonia]	7	285	31.5	4.51	19	10	7	High	1	0.993	1.008	0.872	0.91	0.889	0.738	0.761	0.787	0.845	0.819	0.839	0.804	0.826	0.834
TRINITY_DN8046_c0_g1_i5_orf1	tumor protein D53 homolog isoform X2 [Ostrinia furnacalis]	5	310	34.2	4.98	18	8	1	High	1	1.008	1.061	0.967	1.194	0.971	0.916	0.817	0.914	0.836	0.863	0.771	0.87	0.848	1.097
TRINITY_DN4394_c0_g2_i1_orf1	carboxylesterase [Ostrinia furnacalis]	4	445	48.8	8.91	20	5	4	High	1	0.903	0.983	1.409	1.413	1.5	1.251	1.184	1.395	1.902	2.318	1.73	1.832	1.647	1.874
TRINITY_DN10548_c0_g2_i1_orf1	uridine 5'-monophosphate synthase-like [Ostrinia furnacalis]	7	474	52.6	7.39	17	8	7	High	1	0.982	1.005	0.772	0.815	0.795	0.664	0.704	0.711	0.593	0.615	0.614	0.811	0.821	0.821
TRINITY_DN280_c4_g1_i5_orf1	fibroin light chain [Haritalodes derogata]	5	263	27.2	8.6	24	8	5	High	1	0.973	0.977	0.716	0.741	0.752	0.902	0.969	0.938	1.074	1.079	1.122	0.54	0.553	0.529
TRINITY_DN26649_c0_g1_i2_orf1	ATP synthase-coupling factor 6, mitochondrial [Ostrinia furnacalis]	5	95	10.5	8.88	62	11	5	High	1	0.991	0.977	0.462	0.485	0.507	0.444	0.472	0.449	0.468	0.484	0.487	0.437	0.453	0.446
TRINITY_DN37141_c0_g1_i2_orf1	dnaJ homolog shv [Ostrinia furnacalis]	6	353	39.8	5.87	18	6	6	High	1	1.019	0.921	0.711	0.797	0.807	0.813	0.785	0.74	0.747	0.681	0.774	0.872	0.923	0.843
TRINITY_DN19939_c0_g1_i4_orf1	unnamed protein product [Chilo suppressalis]	9	1070	121.1	5.94	10	9	9	High	1	1.039	1.031	1.041	0.955	1.145	0.67	0.795	0.751	0.796	0.833	0.724	0.754	0.759	0.778
TRINITY_DN53281_c0_g1_i11_orf1	larval cuticle protein LCP-17-like [Ostrinia furnacalis]	4	132	14.5	4.67	32	9	4	High	1	1.02	1.029	1.405	1.509	1.397	1.517	1.67	1.549	1.177	0.948	1.341	1.25	1.334	1.264
TRINITY_DN2927_c0_g1_i6_orf1	T-complex protein 1 subunit eta [Ostrinia furnacalis]	5	181	19.4	6.54	38	6	5	High	1	0.947	0.985	0.517	0.519	0.537	0.473	0.518	0.521	0.495	0.518	0.487	0.593	0.563	0.595
TRINITY_DN6063_c1_g2_i1_orf1	short-chain specific acyl-CoA dehydrogenase, mitochondrial [Ostrinia furnacal	6	408	43.9	8	16	8	6	High	1	1.002	1.002	0.812	0.83	0.784	0.709	0.69	0.707	0.721	0.685	0.737	0.75	0.775	0.773
TRINITY_DN3332_c0_g1_i10_orf1	glutathione S-transferase sigma3 [Glyphodes pyloalis]	6	200	23.2	6.57	33	22	1	High	1	1.015	1.006	0.91	0.934	0.996	0.944	1.017	0.975	1.034	1.027	1.081	1.083	1.063	1.167
TRINITY_DN2953_c1_g1_i10_orf1	methionine--tRNA ligase, cytoplasmic isoform X2 [Ostrinia furnacalis]	5	166	17.6	9.39	57	6	2	High	1	0.9	1.113	1.173	1.289	1.075	1.425	1.445	1.383	1.242	1.122	1.506	2.887	3.215	2.729
TRINITY_DN875_c0_g1_i3_orf1	secernin-3 [Ostrinia furnacalis]	5	399	44.7	5.95	15	7	5	High	1	1.016	0.972	1.347	1.346	1.277	1.183	1.208	1.144	1.597	1.349	1.609	2.213	2.309	2.07
TRINITY_DN24789_c0_g1_i9_orfp1	TRINITY_DN24789_c0_g1_i9_m.25888 TRINITY_DN24789_c0_g1::TRINITY_DN24	7	113	13.2	6.52	46	8	5	High	1	1.102	0.909	5.517	6.069	5.687	2.05	2.128	2.156	2.19	1.948	2.507	0.976	1.014	1.003
TRINITY_DN3492_c0_g1_i1_orf1	larval cuticle protein A2B-like [Ostrinia furnacalis]	5	148	15.8	6.52	48	10	5	High	1	1.007	1.015	1.122	1.134	1.183	1.073	1.142	1.08	1.069	1.103	1.082	0.948	0.892	0.932
TRINITY_DN79210_c0_g1_i1_orf1	V-type proton ATPase 16 kDa proteolipid subunit [Frieseomelitta varia]	1	158	16	9.04	11	19	1	High	1	0.939	0.936	0.298	0.272	0.295	0.283	0.29	0.309	0.302	0.361	0.28	0.31	0.286	0.325
TRINITY_DN701_c0_g1_i1_orf1	venom protease-like isoform X3 [Ostrinia furnacalis]	5	238	27.1	6.4	30	6	5	High	1	0.979	0.964	1.801	1.644	1.859	2.281	2.025	2.258	1.812	1.673	1.559	1.092	1.024	1.116

TRINITY_DN86149_c0_g1_i1_orf1	NADH dehydrogenase [ubiquinone] 1 alpha subcomplex subunit 8 [Galleria m	6	176	20.1	5.97	42	7	6	High	1	1.014	0.99	0.433	0.46	0.486	0.389	0.387	0.397	0.49	0.483	0.471	0.465	0.478	0.48
TRINITY_DN57074_c0_g2_i1_orf1	ribosomal protein l36e domain-containing protein [Phthorimaea operculella]	4	120	13.8	11.62	27	19	4	High	1	0.989	0.977	0.708	0.696	0.7	0.637	0.65	0.615	0.596	0.614	0.621	0.622	0.592	0.593
TRINITY_DN1097_c0_g1_i1_orf1	40S ribosomal protein S28 [Bombyx mori]	4	65	7.3	10.37	55	9	4	High	1	0.958	0.988	0.784	0.841	0.761	0.847	0.906	0.788	0.845	0.831	0.926	0.758	0.701	0.797
TRINITY_DN1766_c0_g1_i6_orf1	epsin-2 isoform X1 [Ostrinia furnacalis]	6	547	59.4	6.42	20	6	6	High	1	0.94	0.934	0.916	0.961	0.919	0.909	0.916	0.887	0.878	0.842	0.876	0.969	0.961	0.975
TRINITY_DN1154_c0_g1_i1_orf1	calexcitin-1-like [Ostrinia furnacalis]	5	182	20.6	4.86	35	6	5	High	1	0.945	0.941	0.238	0.232	0.296	0.301	0.334	0.359	0.481	0.325	0.304	0.229	0.245	0.233
TRINITY_DN3343_c0_g1_i4_orf1	AFG3-like protein 2 [Ostrinia furnacalis]	7	657	72.3	9.23	12	8	7	High	1	1.024	1.005	0.618	0.64	0.633	0.643	0.614	0.611	0.605	0.632	0.597	0.605	0.65	0.606
TRINITY_DN1328_c0_g1_i6_orf1	fungal protease inhibitor-1-like [Ostrinia furnacalis]	5	105	11.2	8.31	54	17	5	High	1	0.973	1.024	1.298	1.386	1.361	1.444	1.537	1.412	1.61	1.515	1.751	0.994	1.025	1.042
TRINITY_DN104_c0_g1_i4_orf1	PREDICTED: heparan-alpha-glucosaminide N-acetyltransferase [Amyelois tran	5	580	64.2	8.1	11	6	4	High	1	0.95	1.005	1.669	1.537	1.591	1.496	1.386	1.424	1.196	1.158	1.188	2.437	2.366	2.401
TRINITY_DN3464_c0_g1_i1_orf1	putative mitochondrial aconitate hydratase isoform X1-likeprotein, partial [Cot	5	819	89.4	8.57	6	12	1	High	1	1.125	1.019	1.126	1.09	1.06	1.354	1.183	1.138	2.19	1.883	1.846	2.905	2.59	2.875
TRINITY_DN23042_c0_g1_i1_orf1	protoporphyrinogen oxidase [Ostrinia furnacalis]	5	472	52.8	8.28	14	6	5	High	1	0.971	1.014	1.133	1.096	1.019	0.999	1.098	1.047	0.952	0.951	0.934	1.163	1.166	1.288
TRINITY_DN34423_c0_g1_i3_orf1	THAP domain-containing protein 4-like [Ostrinia furnacalis]	2	107	12.2	8.46	23	12	1	High	1	0.628	0.469	6.247	6.327	6.312	7.049	5.623	5.476	6.697	5.946	8.228	3.877	4.02	4.069
TRINITY_DN2942_c0_g1_i6_orf1	microtubule-associated protein RP/EB family member 1 [Ostrinia furnacalis]	6	278	30.9	7.42	24	8	6	High	1	1.037	1.009	1.019	0.984	1.037	0.961	0.983	0.935	0.897	0.792	0.922	1.176	1.239	1.154
TRINITY_DN14458_c0_g1_i2_orf1	spermatogenesis-associated protein 20 isoform X1 [Ostrinia furnacalis]	5	834	93.7	6.24	9	5	5	High	1	0.933	1.038	1.213	1.295	1.228	1.155	1.153	1.139	1.217	1.25	1.301	1.757	1.772	1.749
TRINITY_DN1363_c0_g1_i11_orf1	cytochrome P450 CYP12A2-like isoform X1 [Ostrinia furnacalis]	8	503	57.7	8.4	17	9	8	High	1	1.004	1.024	3.255	3.539	3.349	2.962	3.018	2.878	2.676	2.679	2.908	1.91	1.91	2.033
TRINITY_DN79734_c0_g2_i3_orf1	60S ribosomal protein L27a [Ostrinia furnacalis]	7	154	17.1	10.61	39	18	7	High	1	1.009	0.97	0.633	0.586	0.691	0.633	0.616	0.65	0.535	0.457	0.492	0.539	0.576	0.504
TRINITY_DN517_c0_g1_i5_orf1	putative pre-mRNA-splicing factor ATP-dependent RNA helicase PRP1 [Helicc	8	736	83	7.33	13	9	8	High	1	0.957	1.001	0.6	0.674	0.67	0.667	0.645	0.617	0.63	0.63	0.57	0.647	0.659	0.697
TRINITY_DN3300_c0_g2_i1_orf1	annexin B10 isoform X9 [Ostrinia furnacalis]	6	319	35.5	4.89	21	6	6	High	1	1.038	0.955	1.584	1.515	1.557	1.352	1.25	1.298	1.3	1.13	1.267	1.366	1.417	1.314
TRINITY_DN3411_c0_g2_i1_orf1	putative U5 small nuclear ribonucleoprotein 200 kDa helicase, partial [Ostrinia	5	911	102.6	5.88	7	5	5	High	1	0.971	1.036	0.768	0.762	0.796	0.794	0.891	0.896	0.703	0.674	0.673	0.943	0.834	0.943
TRINITY_DN39975_c0_g1_i4_orf1	hsp90 co-chaperone Cdc37 [Ostrinia furnacalis]	7	379	44.7	5.14	21	7	7	High	1	0.998	1.004	0.873	0.907	0.912	0.956	0.995	1.012	0.916	0.926	0.967	1.143	1.138	1.063
TRINITY_DN31676_c0_g1_i4_orf1	N-acetylgalactosaminyltransferase 7 isoform X1 [Ostrinia furnacalis]	7	589	67.8	7.96	12	11	7	High	1	1.033	1.022	1.337	1.331	1.301	1.39	1.395	1.449	1.58	1.592	1.402	1.706	1.637	1.713
TRINITY_DN9085_c0_g1_i1_orf1	golgin subfamily A member 2-like [Ostrinia furnacalis]	6	690	80	5.02	10	6	6	High	1	0.996	0.976	0.767	0.775	0.836	0.785	0.743	0.804	0.724	0.7	0.72	0.764	0.799	0.766
TRINITY_DN17326_c0_g1_i8_orf1	aminoacylase-1-like [Ostrinia furnacalis]	6	185	20.8	5.24	33	8	1	High	1	1.031	0.97	1.975	1.844	1.81	2.048	1.949	2.136	1.937	1.7	1.996	1.416	1.437	1.357
TRINITY_DN4247_c0_g1_i4_orf1	unnamed protein product [Spodoptera exigua]	6	566	61.5	7.31	12	8	6	High	1	0.948	1.021	0.864	0.852	0.851	1.059	1.009	1.057	0.91	1.008	0.927	0.935	0.936	0.948
TRINITY_DN2896_c0_g1_i2_orf1	general odorant-binding protein 56d-like isoform X2 [Ostrinia furnacalis]	7	133	15	8.66	46	10	7	High	1	1.022	1.034	1.106	1.119	1.086	1.03	1.026	1.035	0.941	0.865	0.99	3.481	3.674	3.246
TRINITY_DN1277_c4_g1_i5_orf1	GTP cyclohydrolase 1 isoform X1 [Ostrinia furnacalis]	5	291	32.6	7.01	19	10	5	High	1	1.032	1.015	0.275	0.307	0.329	0.354	0.386	0.352	0.301	0.309	0.32	0.334	0.324	0.343
TRINITY_DN2914_c0_g1_i1_orf1	U1 small nuclear ribonucleoprotein A [Ostrinia furnacalis]	5	223	25.4	9.76	25	8	5	High	1	1.031	1.021	0.753	0.673	0.718	0.621	0.632	0.714	0.581	0.577	0.641	0.9	0.806	0.772
TRINITY_DN2782_c0_g1_i7_orf1	CDK5 regulatory subunit-associated protein 3 [Ostrinia furnacalis]	7	507	56.6	5.03	14	7	7	High	1	1.013	0.981	1.011	1.099	1.065	0.964	1.036	0.971	0.834	0.719	0.875	1.028	1.11	1.078
TRINITY_DN1666_c0_g1_i2_orf1	putative defense protein Hdd11 [Ostrinia furnacalis]	6	165	17.7	8.72	52	7	6	High	1	1.031	1.006	0.378	0.382	0.406	0.679	0.632	0.657	0.559	0.51	0.584	0.387	0.384	0.426
TRINITY_DN18136_c0_g1_i1_orf1	proteoglycan 4-like [Ostrinia furnacalis]	6	1349	149.3	4.45	6	6	6	High	1	0.965	1.007	1.36	1.368	1.267	0.933	1.033	1.009	0.912	1.09	0.984	1.381	1.341	1.383
TRINITY_DN1609_c0_g1_i3_orf1	senecionine N-oxygenase-like [Ostrinia furnacalis]	5	457	52.2	7.08	14	5	5	High	1	1	0.949	0.994	0.966	0.96	1.019	1.125	0.996	0.947	0.982	0.914	0.846	0.859	0.916
TRINITY_DN960_c1_g1_i6_orf1	hypothetical protein evm_007130 [Chilo suppressalis]	5	145	15.5	7.65	38	9	5	High	1	1.048	0.968	2.746	2.695	2.757	2.279	2.273	2.323	2.231	1.995	2.3	2.466	2.472	2.315
TRINITY_DN1134_c0_g1_i4_orf1	cytochrome P450 6B5-like [Ostrinia furnacalis]	6	494	56.4	8.63	16	6	3	High	1	1.013	1.006	1.342	1.3	1.26	1.424	1.421	1.448	1.298	1.259	1.281	0.654	0.617	0.584
TRINITY_DN29009_c0_g2_i3_orf1	juvenile hormone binding protein [Omphisa fuscidentalis]	5	154	17.3	5.68	31	8	2	High	1	1.002	1.1	0.864	0.855	0.899	1.124	1.259	0.992	1.294	1.296	1.017	0.925	0.886	1.008
TRINITY_DN1661_c0_g1_i1_orf1	NAD(P) transhydrogenase, mitochondrial-like [Ostrinia furnacalis]	4	221	23.7	9.47	23	7	4	High	1	0.941	0.971	0.76	0.803	0.758	0.748	0.736	0.834	0.719	0.7	0.693	0.996	0.958	0.954
TRINITY_DN2488_c0_g1_i4_orf1	NEDD8-activating enzyme E1 regulatory subunit [Ostrinia furnacalis]	6	535	59.9	5.54	17	6	6	High	1	0.984	1.002	0.98	1.061	1.04	0.996	1.012	0.985	0.947	1.078	0.921	0.995	0.96	0.986
TRINITY_DN106476_c0_g1_i3_orf1	mitochondrial import inner membrane translocase subunit TIM44 [Ostrinia furr	7	436	49.7	7.58	18	7	7	High	1	0.999	1.025	0.527	0.537	0.544	0.516	0.513	0.511	0.485	0.405	0.412	0.448	0.469	0.476
TRINITY_DN74538_c0_g1_i1_orf1	tetra-peptide repeat homeobox protein 1-like [Ostrinia furnacalis]	3	226	23.1	9.55	32	6	3	High	1	1.026	1.038	1.141	1.026	0.951	0.972	1.016	0.941	1.075	1.086	0.904	7.562	6.96	7.444
TRINITY_DN11176_c0_g1_i1_orf1	peroxiredoxin-6 [Ostrinia furnacalis]	4	223	25	5.49	27	7	4	High	1	0.953	0.999	1.054	1.069	1.106	1.059	1.109	1.075	1.022	1.053	1.016	0.98	0.955	0.981
TRINITY_DN99020_c0_g1_i1_orf1	uncharacterized protein LOC114357292 isoform X4 [Ostrinia furnacalis]	5	255	27.2	7.24	22	7	5	High	1	0.932	0.989	0.83	0.867	0.831	0.982	0.968	0.971	0.837	0.79	0.888	1.429	1.377	1.381
TRINITY_DN4757_c0_g1_i3_orf1	melanotransferrin isoform X1 [Ostrinia furnacalis]	6	857	94.6	6.71	9	6	6	High	1	0.983	0.998	0.592	0.604	0.654	0.695	0.618	0.634	0.649	0.578	0.634	1.02	1.039	0.966
TRINITY_DN43420_c0_g2_i1_orf1	collagenase-like [Ostrinia furnacalis]	4	285	29.8	9.6	24	4	4	High	1	0.998	0.933	0.322	0.332	0.386	0.32	0.32	0.346	0.343	0.305	0.311	0.34	0.376	0.325
TRINITY_DN3749_c0_g1_i1_orf1	cytochrome c oxidase subunit 6B1 [Ostrinia furnacalis]	5	82	9.9	8.73	43	11	5	High	1	0.991	1.057	0.396	0.414	0.427	0.362	0.357	0.361	0.397	0.358	0.43	0.405	0.395	0.4
TRINITY_DN31314_c0_g1_i4_orf1	uncharacterized protein LOC114355976 [Ostrinia furnacalis]	7	764	86.8	9.85	9	7	7	High	1	0.984	1.046	0.988	0.917	1.051	0.98	1.026	0.984	1.132	1.018	0.917	3.774	3.82	3.675
TRINITY_DN344_c1_g1_i1_orf1	chymotrypsin-like serine protease 16 [Ostrinia nubilalis]	5	286	30.7	8.22	29	5	5	High	1	0.99	1.019	0.223	0.228	0.302	0.253	0.269	0.259	0.647	0.598	0.689	0.2	0.172	0.195
TRINITY_DN2844_c0_g1_i2_orf1	glutathione S-transferase 9 [Streltzoviella insularis]	5	199	22	5.07	31	13	5	High	1	0.916	0.952	1.254	1.278	1.291	1.369	1.464	1.326	1.143	1.047	1.107	0.978	0.929	0.953
TRINITY_DN3209_c0_g2_i6_orf1	coatomer subunit beta, partial [Ostrinia furnacalis]	5	536	59.2	6.34	11	6	3	High	1	0.985	1.019	0.95	1.027	0.921	1.219	1.217	1.215	0.901	1.133	0.975	1.063	1.095	1.181
TRINITY_DN413_c0_g1_i11_orf1	regulator of nonsense transcripts 1 [Helicoverpa armigera]	4	1042	115.9	7.33	6	5	4	High	1	0.949	0.973	0.504	0.571	0.59	0.566	0.556	0.51	0.527	0.649	0.544	0.641	0.608	0.642
TRINITY_DN53358_c0_g1_i3_orf1	arylphorin subunit alpha-like [Ostrinia furnacalis]	4	108	13.3	6.93	39	17	4	High	1	0.941	1.012	2.078	1.536	1.952	2.356	2.3	3.005	3.351	6.957	1.818	2.297	2.078	2.65
TRINITY_DN9132_c0_g1_i5_orf1	ubiquitin-like-conjugating enzyme ATG3 [Spodoptera frugiperda]	5	324	36.1	4.69	18	6	5	High	1	0.959	0.977	2.121	2.15	2.124	2.628	2.487	2.475	2.489	3.587	2.578	1.898	1.835	1.854
TRINITY_DN3132_c0_g1_i10_orf1	oxysterol-binding protein 1-like [Ostrinia furnacalis]	4	523	58.4	5.4	10	5	4	High	1	0.973	1.008	0.934	0.938	0.911	0.874	0.881	0.873	0.781	0.755	0.747	0.837	0.868	0.876
TRINITY_DN50724_c0_g2_i1_orf1	40S ribosomal protein S13 [Papilio polytes]	5	151	17.2	10.45	30	19	3	High	1	1.026	1.02	0.743	0.721	0.734	0.663	0.663	0.701	0.689	0.66	0.579	0.655	0.656	0.665
TRINITY_DN4030_c0_g2_i1_orf1	putative trypsin 6 [Ostrinia nubilalis]	3	264	28.7	10.3	20	6	3	High	1	0.992	1.035	0.282	0.247	0.259	0.315	0.332	0.346	0.396	0.425	0.369	0.325	0.329	0.35
TRINITY_DN2126_c0_g1_i2_orf1	ubiquitin-conjugating enzyme E2-22 kDa [Ostrinia furnacalis]	4	202	22.6	5.3	22	6	4	High	1	0.993	0.996	1.21	1.041	1.181	1.121	1.078	1.17	1.149	1.05	0.954	1.187	1.205	1.183
TRINITY_DN51096_c0_g1_i1_orf1	L-galactose dehydrogenase-like [Ostrinia furnacalis]	6	328	37	5.81	20	6	6	High	1	1.05	1.123	1.164	1.105	1.128	1.121	1.105	1.239	1.097	1.163	1.07	1.004	0.953	0.952
TRINITY_DN14597_c0_g1_i5_orf1	UDP-glucuronosyltransferase 2B1-like isoform X3 [Ostrinia furnacalis]	6	518	58.6	8.6	14	7	5	High	1	0.98	0.974	0.647	0.599	0.671	0.755	0.759	0.78	0.589	0.674	0.554	0.482	0.47	0.494
TRINITY_DN19998_c0_g1_i1_orf1	ubiquitin-conjugating enzyme E2 variant 2 [Helicoverpa armigera]	4	144	16.4	7.14	33	6	4	High	1	1.069	1.023	1.053	1.293	1.16	1.122	1.095	1.07	1.079	1.054	1.221	1.547	1.529	1.549
TRINITY_DN13098_c2_g1_i2_orf1	unnamed protein product [Arctia plantaginis]	6	195	22.9	5.45	41	6	6	High	1	1.088	1	1.941	1.99	1.895	2.135	2.093	2.148	2.433	2.171	2.347	2.037	1.932	2.006
TRINITY_DN4429_c0_g1_i5_orf1	FACT complex subunit Ssrp1 isoform X1 [Ostrinia furnacalis]	7	719	81.1	7.21	11	7	7	High	1	0.989	0.982	0.646	0.591	0.643	0.654	0.652	0.683	0.727	0.709	0.669	0.819	0.773	0.754
TRINITY_DN8655_c0_g4_i1_orf1	serine/threonine-protein phosphatase 2A 56 kDa regulatory subunit epsilon is	5	461	53.6	7.5	13	6	5	High	1	1.042	1.067	0.99	0.964	0.949	0.983	1.019	1.031	0.932	0.846	1.036	0.933	0.935	0.95
TRINITY_DN726_c0_g1_i2_orf1	vinculin [Ostrinia furnacalis]	4	360	39.4	6.01	15	5	4	High	1	1.015	0.998	1.379	1.638	1.458	1.107	1.426	1.265	1.397	1.364	1.399	1.307	1.297	1.231
TRINITY_DN3403_c0_g1_i6_orf1	ralBP1-associated Eps domain-containing protein 1 isoform X5 [Ostrinia furna	6	703	77.7	6.1	10	6	6	High	1	1.029	0.973	1.141	1.219	1.189	1.137	1.186	1.165	1.084	1.122	1.113	1.093	1.237	1.132
TRINITY_DN5111_c0_g1_i2_orf1	uncharacterized protein LOC126368598 [Pectinophora gossypiella]	2	131	14.3	9.36	29	10	2	High	1	0.974	0.969	0.661	0.716	0.701	0.677	0.698	0.707	0.77	0.788	0.737	0.611	0.591	0.617
TRINITY_DN9457_c0_g1_i9_orf1	plexin domain-containing protein 2 [Spodoptera litura]	5	569	62.7	6.65	10	7	5	High	1	1.027	0.982	1.534	1.339	1.535	1.571	1.475	1.487	1.287	1.213	1.129	1.631	1.608	1.765
TRINITY_DN650_c0_g1_i3_orf1	chitinase 7 [Glyphodes pyloalis]	7	987	111.3	6.61	9	7	7	High	1	0.97	0.975	0.631	0.601	0.611	0.735	0.816	0.779	0.64	0.717	0.642	1.759	1.77	1.666
TRINITY_DN49530_c0_g1_i1_orf1	ommochrome-binding protein-like [Ostrinia furnacalis]	3	121	13.6	6.32	50	6	3	High	1	0.925	0.976	1.918	1.732	1.887	2.038	2.289	2.352	2.302	2.544	2.016	1.609	1.445	1.512
TRINITY_DN4068_c1_g2_i1_orf1	larval cuticle protein LCP-17 [Helicoverpa armigera]	4	160	17	5.82	64	6	4	High	1	1.077	0.95	2.592	2.613	2.597	1.785	1.889	1.726	1.923	1.719	1.925	0.722	0.924	0.945
TRINITY_DN2847_c0_g1_i20_orf1	uncharacterized protein LOC114352221 [Ostrinia furnacalis]	5	260	29.1	6.06	28	6	5	High	1	1.022	0.977	1.298	1.263	1.41	1.848	1.912	1.83	1.549	1.521	1.556	1.177	1.271	1.257
TRINITY_DN104663_c1_g1_i2_orf1	PREDICTED: gelsolin-like [Amyelois transitella]	3	112	12.4	4.86	53	4	1	High	1	1.027	1.036	2.302	2.097	2.448	2.186	2.346	2.361	3.394	3.011	2.372	2.753	2.642	3.133
TRINITY_DN20560_c0_g1_i6_orf1	pupal cuticle protein C1B-like [Ostrinia furnacalis]	3	229	22.6	9.29	30	7	3	High	1	1.077	1.176	3.608	3.692	3.471	3.575	3.588	3.294	2.755	2.53	2.957	5.338	5.399	5.392
TRINITY_DN4710_c0_g1_i1_orf1	hypothetical protein evm_000671 [Chilo suppressalis]	5	967	110.7	8.16	8	5	5	High	1	0.848	0.863	0.462	0.509	0.471	0.544	0.522	0.47	0.482	0.494	0.55	0.535	0.547	0.524
TRINITY_DN40650_c0_g1_i1_orf1	60S ribosomal protein L12 [Zerene cesonia]	5	164	17.5	9.72	45	10	5	High	1	0.983	0.947	0.65	0.714	0.671	0.585	0.654	0.58	0.503	0.485	0.55	0.512	0.504	0.534
TRINITY_DN12526_c0_g1_i5_orf1	uncharacterized protein LOC114359035 isoform X3 [Ostrinia furnacalis]	4	136	15.2	5.33	37	8	2	High	1	0.895	1.059	2.156	2.093	2.248	4.838	5.169	4.582	3.032	3.071	3.995	6.359	5.993	6.823
TRINITY_DN40669_c0_g2_i1_orf1	uncharacterized protein LOC114355531 [Ostrinia furnacalis]	6	82	9.5	7.18	59	13	6	High	1	1.048	1.029	1	0.913	0.905	0.992	1.003	0.98	0.948	0.755	0.729	20.114	21.28	19.295
TRINITY_DN2668_c0_g1_i7_orf1	unnamed protein product [Chrysodeixis includens]	6	346	38.5	9.31	26	7	6	High	1	1.023	1.058	0.371	0.389	0.372	0.362	0.417	0.419	0.379	0.327	0.399	0.243	0.258	0.239
TRINITY_DN5578_c0_g1_i4_orf1	chromatin modification-related protein eaf-1-like [Ostrinia furnacalis]	7	1374	150.2	6.48	6	8	5	High	1	1.018	0.881	0.454	0.431	0.577	0.453	0.43	0.419	0.4	0.316	0.362	0.6	0.63	0.557
TRINITY_DN16490_c0_g2_i1_orf1	dnaJ protein homolog 1 [Ostrinia furnacalis]	5	352	38.9	9.26	14	5	5	High	1	1.017	0.993	0.869	0.923	0.85	0.888	0.919	0.851	0.901	0.861	0.929	0.894	0.861	0.995
TRINITY_DN34432_c0_g1_i1_orf1	39S ribosomal protein L44, mitochondrial [Ostrinia furnacalis]	6	325	36.7	7.85	22	7	6	High	1	0.977	0.935	0.522	0.516	0.562	0.53	0.557	0.565	0.54	0.572	0.498	0.686	0.695	0.742
TRINITY_DN23732_c0_g1_i1_orf1	glutathione S-transferase 1-like [Ostrinia furnacalis]	5	215	24.2	6.07	29	6	5	High	1	0.983	1.007	1.005	0.983	1.036	1.075	1.047	0.997	1.304	1.197	1.2	1.47	1.42	1.427
TRINITY_DN117_c0_g1_i6_orf1	lipase member I-like [Ostrinia furnacalis]	4	372	42.3	5.08	11	10	1	High	1	1.022	0.984	0.059	0.071	0.102	0.105	0.121	0.142	0.155	0.149	0.129	0.192	0.178	0.186
TRINITY_DN6881_c0_g1_i1_orf1	putative protein TPRXL [Ostrinia furnacalis]	5	214	22.4	4.73	23	9	5	High	1	1.011	0.987	0.861	0.941	0.911	1.051	1.065	1.042	1.178	1	1.222	0.186	0.15	0.208
TRINITY_DN8654_c0_g1_i1_orf1	argininosuccinate lyase [Ostrinia furnacalis]	5	475	53.5	6.49	14	6	5	High	1	0.993	1.07	1.155	1.145	1.197	1.078	1.06	1.053	1.132	1.335	1.239	1.195	1.096	1.111
TRINITY_DN10774_c0_g2_i3_orf1	uncharacterized protein LOC114362157, partial [Ostrinia furnacalis]	5	589	64.7	8.16	11	5	5	High	1	0.947	0.978	1.217	1.397	1.261	1.491	1.44	1.575	1.206	1.329	1.217	1.015	1.027	1.059
TRINITY_DN5475_c0_g1_i3_orf1	traB domain-containing protein-like isoform X1 [Ostrinia furnacalis]	6	393	44	5.99	16	8	6	High	1	0.985	0.965	0.997	0.955	0.962	0.971	0.943	0.976	0.868	0.733	0.79	1.063	1.08	1.058
TRINITY_DN25136_c0_g1_i1_orf1	jg9843 [Pararge aegeria aegeria]	3	131	15	7.27	38	5	3	High	1	1.001	1.03	0.809	0.793	0.725	0.763	0.824	0.8	0.915	0.892	0.817	0.892	0.851	0.9
TRINITY_DN225_c0_g1_i6_orf1	glutathione S-transferase delta 3 [Ostrinia furnacalis]	6	219	24.2	5.44	30	10	6	High	1	0.975	0.949	1.046	1.044	1.135	1.497	1.688	1.545	1.172	1.098	1.139	1.317	1.328	1.321
TRINITY_DN21451_c0_g1_i3_orf1	gelsolin-like [Ostrinia furnacalis]	5	118	13.4	5.45	47	6	2	High	1	0.932	0.924	0.621	0.655	0.675	0.663	0.827	0.754	0.721	0.689	0.704	0.54	0.526	0.561
TRINITY_DN59291_c0_g1_i1_orf1	ATP-dependent RNA helicase vasa [Ostrinia furnacalis]	5	617	67.4	5.68	8	7	5	High	1	1.025	1.031	0.811	0.859	0.824	0.787	0.782	0.791	0.688	0.65	0.694	1.033	0.995	0.956
TRINITY_DN4835_c0_g1_i2_orf1	ribonucleoside-diphosphate reductase large subunit [Ostrinia furnacalis]	7	798	90.5	7.37	10	8	7	High	1	1.012	0.99	0.729	0.745	0.753	0.591	0.597	0.614	0.55	0.573	0.548	1.01	0.96	1.046
TRINITY_DN195_c0_g3_i6_orf1	E3 ubiquitin-protein ligase SIAH1-like [Ostrinia furnacalis]	6	343	38	8.87	17	7	6	High	1	0.938	0.974	1.129	1.22	1.174	1.216	1.357	1.233	1.159	1.173	1.141	1.29	1.255	1.233
TRINITY_DN2200_c0_g1_i4_orf1	uncharacterized protein LOC114363443 [Ostrinia furnacalis]	4	142	16.2	5.4	30	8	4	High	1	0.961	0.947	0.872	0.918	0.899	0.747	0.753	0.692	0.862	0.822	0.921	0.509	0.489	0.474
TRINITY_DN35763_c0_g1_i2_orf1	probable methylthioribulose-1-phosphate dehydratase [Helicoverpa armigera	4	226	25.4	6.61	26	4	4	High	1	0.96	0.916	1.248	1.211	1.268	1.533	1.331	1.471	1.353	1.302	1.232	0.932	1.014	0.984
TRINITY_DN12320_c0_g1_i1_orf1	brefeldin A-inhibited guanine nucleotide-exchange protein 1 [Ostrinia furnaca	8	1713	193.1	6.27	5	8	8	High	1	0.986	1.067	0.848	0.837	0.807	0.822	0.782	0.818	0.731	0.777	0.807	0.922	0.928	0.93
TRINITY_DN34784_c0_g1_i1_orf1	F-actin-capping protein subunit alpha [Ostrinia furnacalis]	5	288	32.3	5.85	20	7	5	High	1	0.992	1.001	1.172	1.23	1.136	1.034	1.053	0.999	0.934	0.998	1.135	1.087	1.072	1.05
TRINITY_DN4189_c0_g2_i1_orf1	unnamed protein product [Chilo suppressalis]	6	1122	126.1	6.23	6	7	6	High	1	1.021	1.087	1.646	1.591	1.487	1.346	1.258	1.321	1.23	1.135	1.297	2.743	2.573	2.618

TRINITY_DN125967_c0_g1_i1_orf1	26S proteasome non-ATPase regulatory subunit 14 [Ostrinia furnacalis]	6	311	34.6	6.29	20	8	6	High	1	0.97	0.965	1.048	1.048	1.047	0.986	0.979	1.027	0.889	0.99	0.905	1.097	1.178	1.146
TRINITY_DN83948_c0_g1_i3_orf1	carbonyl reductase [NADPH] 1-like [Ostrinia furnacalis]	4	324	36.4	7.87	19	4	4	High	1	1.095	1.055	0.597	0.565	0.535	0.539	0.633	0.542	0.57	0.778	0.612	0.518	0.47	0.472
TRINITY_DN1054_c0_g1_i8_orf1	tubulin-specific chaperone D [Ostrinia furnacalis]	6	1171	130.1	6.11	7	6	5	High	1	0.99	1.109	1.596	1.76	1.574	1.576	1.535	1.538	1.285	1.327	1.444	1.478	1.357	1.599
TRINITY_DN31503_c0_g1_i4_orf1	hypothetical protein evm_001345 [Chilo suppressalis]	5	405	46.2	5.85	12	10	4	High	1	1.017	1.036	0.599	0.543	0.622	0.607	0.557	0.578	0.614	0.647	0.56	0.775	0.796	0.841
TRINITY_DN22242_c0_g2_i1_orf1	juvenile hormone epoxide hydrolase-like [Ostrinia furnacalis]	5	337	38.1	7.97	16	10	5	High	1	1.005	0.976	1.829	1.804	1.76	2.123	2.093	2.052	1.692	1.906	1.603	1.766	1.696	1.849
TRINITY_DN231_c1_g2_i1_orf1	G protein-activated inward rectifier potassium channel 3-like isoform X5 [Ostri	8	484	55.4	6.64	17	8	8	High	1	1.095	1.143	0.902	0.931	0.933	1.163	1.126	1.061	1.137	0.922	1.089	0.942	0.998	0.898
TRINITY_DN64627_c0_g1_i1_orf1	probable 3-hydroxyacyl-CoA dehydrogenase B0272.3 [Ostrinia furnacalis]	6	310	33.5	9.11	24	9	5	High	1	1.005	1.001	0.833	0.875	0.806	0.783	0.798	0.802	0.815	0.869	0.9	0.931	0.939	0.952
TRINITY_DN38835_c0_g3_i1_orf1	protein transport protein Sec61 subunit alpha [Spodoptera litura]	5	418	46.1	8.73	11	8	3	High	1	1.103	1.026	0.873	0.559	0.902	0.798	0.688	0.925	0.842	0.683	0.423	0.508	0.496	0.451
TRINITY_DN1406_c0_g2_i2_orf1	uncharacterized protein LOC114366644 [Ostrinia furnacalis]	6	841	93.2	6.83	8	6	6	High	1	0.995	1.052	0.947	0.905	1.037	1.026	1.1	1.061	0.979	0.908	1.079	0.851	0.863	0.842
TRINITY_DN12997_c0_g2_i1_orf1	cytochrome P450 monooxygenase CYP6AE28 [Cnaphalocrocis medinalis]	5	523	60.3	7.46	12	6	5	High	1	0.999	1.037	0.948	0.961	0.97	0.998	0.956	1.05	0.969	0.974	0.93	0.768	0.782	0.852
TRINITY_DN5132_c0_g1_i4_orf1	small heat shock protein Hsp24.2 [Ostrinia furnacalis]	5	224	24.7	4.75	21	10	5	High	1	1.059	1.023	1.663	1.346	1.634	2.458	2.303	2.516	2.577	2.487	1.968	2.325	2.384	2.329
TRINITY_DN11639_c0_g1_i1_orf1	cysteine--tRNA ligase, cytoplasmic isoform X1 [Ostrinia furnacalis]	5	723	81.9	6.99	6	5	5	High	1	1.006	1.057	0.665	0.669	0.664	0.657	0.679	0.648	0.673	0.673	0.684	0.693	0.715	0.723
TRINITY_DN51045_c0_g1_i1_orf1	cell growth-regulating nucleolar protein [Ostrinia furnacalis]	4	421	48.3	8.02	13	5	4	High	1	1.028	1.05	0.318	0.374	0.368	0.339	0.386	0.368	0.362	0.342	0.368	0.451	0.46	0.434
TRINITY_DN6871_c0_g1_i3_orf1	1-acyl-sn-glycerol-3-phosphate acyltransferase gamma-like [Ostrinia furnaca	5	383	44.5	8.94	15	6	5	High	1	0.991	1.03	0.732	0.692	0.721	0.694	0.598	0.686	0.643	1.153	0.588	0.861	0.85	0.832
TRINITY_DN6247_c0_g1_i2_orf1	innexin inx3 [Ostrinia furnacalis]	5	386	44	7.97	14	5	5	High	1	1.024	1.024	0.765	0.71	0.803	0.853	0.833	0.839	0.823	0.731	0.763	0.792	0.812	0.822
TRINITY_DN4070_c0_g1_i4_orf1	alpha-N-acetylgalactosaminidase isoform X3 [Ostrinia furnacalis]	5	440	49.7	5.63	15	5	5	High	1	0.972	0.955	0.541	0.519	0.557	0.449	0.488	0.457	0.424	0.449	0.393	1.195	1.204	1.139
TRINITY_DN184_c0_g1_i10_orf1	C-type mannose receptor 2-like isoform X1 [Leguminivora glycinivorella]	6	278	30.8	6.42	27	6	1	High	1	0.716	0.681	2.313	2.579	2.494	1.301	1.647	1.256	1.365	1.868	1.273	1.935	1.804	2.122
TRINITY_DN18300_c0_g1_i17_orf1	RNA-binding protein lark isoform X1 [Helicoverpa armigera]	5	326	36.6	8.85	17	6	5	High	1	1.006	1.104	0.799	0.808	0.776	0.729	0.751	0.658	0.725	0.743	0.671	0.762	0.754	0.767
TRINITY_DN537_c0_g1_i1_orf1	pupal cuticle protein C1B-like precursor [Papilio xuthus]	4	282	27.7	9.36	32	4	4	High	1	1.016	0.991	1.544	2.084	1.585	1.233	1.371	1.494	1.347	1.21	1.332	2.635	2.359	2.076
TRINITY_DN132043_c0_g1_i1_orf1	ankyrin repeat and MYND domain-containing protein 2 [Ostrinia furnacalis]	4	395	43.9	7.3	14	6	4	High	1	0.974	0.952	0.727	0.72	0.731	0.696	0.696	0.67	0.686	0.656	0.667	0.827	0.854	0.827
TRINITY_DN1255_c0_g1_i17_orf1	LIM and senescent cell antigen-like-containing domain protein 1 isoform X1 [I	5	384	44.2	8.27	18	5	5	High	1	0.96	0.994	1.273	1.253	1.205	1.253	1.355	1.28	1.296	1.275	1.136	1.143	1.105	1.099
TRINITY_DN745_c7_g1_i1_orf1	uncharacterized protein LOC114358822 [Ostrinia furnacalis]	4	298	33.1	5.15	17	5	4	High	1	1.021	1.063	1.574	1.526	1.505	1.699	1.72	1.754	2.349	2.345	2.045	1.996	1.945	2.062
TRINITY_DN4289_c0_g1_i5_orf1	translocon-associated protein subunit beta [Ostrinia furnacalis]	3	190	20.9	8.29	26	7	3	High	1	0.98	1.012	1.272	1.186	1.241	1.2	1.154	1.145	1.058	1.397	1.096	0.956	0.968	1.06
TRINITY_DN3616_c0_g2_i1_orf1	conotoxin ArMKLT2-032-like [Ostrinia furnacalis]	4	80	8.7	5.41	64	8	2	High	1	0.909	1.058	1.542	1.696	1.686	1.444	1.453	1.511	1.43	1.232	1.436	4.093	4.37	4.028
TRINITY_DN5032_c0_g1_i1_orf1	protein windpipe [Ostrinia furnacalis]	5	705	77.5	5.29	9	6	5	High	1	0.972	0.972	1.067	1.13	1.079	1.029	1.061	1.039	0.967	0.86	0.993	1.037	1.067	1.057
TRINITY_DN2593_c0_g1_i1_orf1	midgut carboxypeptidase [Loxostege sticticalis]	5	434	49.1	7.52	14	6	4	High	1	0.985	1.005	0.468	0.454	0.51	0.547	0.599	0.626	0.55	0.665	0.531	0.532	0.53	0.54
TRINITY_DN1965_c0_g1_i7_orf1	CTP synthase isoform X1 [Ostrinia furnacalis]	6	610	68.1	7.08	10	6	6	High	1	0.98	1.013	0.51	0.542	0.59	0.452	0.506	0.496	0.544	0.552	0.533	0.556	0.571	0.561
TRINITY_DN31851_c0_g1_i2_orf1	CDGSH iron-sulfur domain-containing protein 2 homolog [Helicoverpa armig	5	132	14.7	8.85	38	10	5	High	1	1.022	0.997	0.602	0.607	0.65	0.486	0.495	0.498	0.461	1.029	0.493	0.659	0.645	0.687
TRINITY_DN21852_c0_g1_i1_orf1	glutaredoxin-3 [Ostrinia furnacalis]	7	220	24.1	5.27	34	8	7	High	1	0.965	1.025	0.938	0.972	0.887	0.878	0.948	0.881	0.889	0.876	0.9	0.77	0.804	0.796
TRINITY_DN8703_c0_g1_i2_orf1	beta-glucuronidase-like isoform X1 [Ostrinia furnacalis]	6	646	73.5	6.8	10	6	6	High	1	1.024	1.034	1.466	1.477	1.428	1.519	1.448	1.457	2.028	2.03	1.866	2.067	1.994	1.84
TRINITY_DN3597_c0_g1_i10_orf1	unnamed protein product [Diatraea saccharalis]	3	412	47.5	4.92	12	4	3	High	1	1.069	1.032	0.903	0.918	0.908	0.787	0.883	0.772	0.74	0.57	0.773	0.991	0.956	0.897
TRINITY_DN17049_c0_g1_i6_orf1	unnamed protein product [Arctia plantaginis]	5	339	38.7	6.02	16	7	5	High	1	1.065	1.025	0.529	0.534	0.564	0.481	0.491	0.505	0.485	0.429	0.472	0.487	0.534	0.532
TRINITY_DN8846_c0_g1_i1_orf1	PREDICTED: synapse-associated protein of 47 kDa-like isoform X2 [Papilio xutl	6	409	44.2	4.6	19	6	6	High	1	1.065	1.059	0.87	0.846	0.866	1.045	1.222	1.027	0.937	0.73	0.8	1.097	1.117	0.925
TRINITY_DN1298_c0_g1_i3_orf1	ras GTPase-activating protein-binding protein 2-like, partial [Ostrinia furnacali	3	144	16.1	10.07	24	5	3	High	1	0.965	1.014	0.789	0.799	0.762	0.674	0.716	0.696	0.598	0.501	0.619	0.82	0.873	0.742
TRINITY_DN11370_c0_g1_i6_orf1	protein MEMO1 [Hyposmocoma kahamanoa]	6	295	33.4	6.71	19	9	6	High	1	0.987	1.034	1.744	1.61	1.75	1.345	1.324	1.568	1.405	1.296	1.287	1.469	1.509	1.546
TRINITY_DN6715_c0_g1_i3_orf1	hypothetical protein evm_010226 [Chilo suppressalis]	6	970	107.9	5.63	8	6	6	High	1	0.996	0.993	0.916	0.908	0.908	0.892	0.898	1.025	0.909	1.234	1.009	1.057	0.972	1.012
TRINITY_DN21971_c0_g1_i4_orf1	40S ribosomal protein S26 [Nymphalis io]	2	119	13.7	11.28	18	5	2	High	1	0.745	0.948	0.672	0.63	0.577	0.549	0.485	0.666	0.499	0.461	0.523	0.647	0.608	0.581
TRINITY_DN16525_c0_g1_i4_orf1	clavesin-1 [Ostrinia furnacalis]	5	292	34.2	8.69	27	8	5	High	1	0.92	0.956	1.052	0.998	0.96	1.004	1.054	1.06	1.025	1.106	1.097	1.184	1.059	1.107
TRINITY_DN26301_c0_g1_i1_orf1	uncharacterized protein LOC114359193 [Ostrinia furnacalis]	3	259	28.5	5.26	16	5	3	High	1	1.035	0.991	1.436	1.429	1.354	1.517	1.569	1.508	1.49	1.419	1.365	3.708	3.642	3.518
TRINITY_DN1783_c0_g1_i2_orf1	uncharacterized protein LOC114352783 [Ostrinia furnacalis]	7	1325	150.6	6.44	5	9	7	High	1	0.975	1.039	1.048	1.11	1.069	1.075	1.07	1.091	1.357	1.205	1.227	1.177	1.157	1.241
TRINITY_DN9316_c1_g1_i1_orf1	peptidyl-tRNA hydrolase 2, mitochondrial-like [Ostrinia furnacalis]	4	161	17.2	9.48	35	4	4	High	1	0.949	1.007	0.571	0.586	0.553	0.614	0.559	0.614	0.596	0.537	0.589	0.537	0.532	0.548
TRINITY_DN24163_c0_g1_i1_orf1	luciferin 4-monooxygenase-like [Ostrinia furnacalis]	6	562	62.7	7.12	11	7	6	High	1	1.016	1.01	0.522	0.469	0.528	0.536	0.53	0.604	0.507	0.493	0.458	0.69	0.623	0.674
TRINITY_DN1498_c0_g1_i2_orf1	ran GTPase-activating protein 1 [Ostrinia furnacalis]	5	563	60.2	5.14	12	5	5	High	1	1.087	1.017	0.759	0.728	0.76	0.743	0.809	0.798	0.789	0.83	0.735	0.928	0.834	0.833
TRINITY_DN16931_c0_g1_i1_orf1	pancreatic triacylglycerol lipase-like [Ostrinia furnacalis]	5	304	32.3	6.51	26	5	5	High	1	0.986	0.999	0.126	0.127	0.181	0.266	0.28	0.281	0.209	0.215	0.203	0.171	0.163	0.167
TRINITY_DN48096_c0_g2_i2_orf1	eukaryotic translation initiation factor 4E-like [Ostrinia furnacalis]	8	210	24.4	6.28	33	12	8	High	1	0.991	0.967	0.879	0.856	0.875	0.81	0.792	0.801	0.712	0.652	0.713	0.824	0.814	0.76
TRINITY_DN7123_c0_g1_i1_orf1	activating signal cointegrator 1 complex subunit 2 homolog isoform X1 [Ostrin	5	155	17.6	9.57	46	6	5	High	1	0.953	0.993	0.89	0.871	0.858	0.86	0.887	0.903	0.894	0.761	0.88	3.068	3.135	2.981
TRINITY_DN12969_c0_g1_i3_orf1	queuosine salvage protein [Ostrinia furnacalis]	5	336	38.6	5.31	17	6	5	High	1	0.947	0.985	2.949	2.816	2.747	1.816	1.664	1.866	1.351	1.201	1.296	1.47	1.576	1.475
TRINITY_DN5558_c0_g1_i4_orf1	unnamed protein product [Parnassius apollo]	4	217	24.9	8.62	31	5	4	High	1	1.068	1.012	1.375	1.641	1.505	1.407	1.415	1.261	1.227	1.129	1.202	1.099	1.18	1.287
TRINITY_DN47930_c0_g1_i4_orf1	uncharacterized protein LOC114362634 [Ostrinia furnacalis]	3	95	10.4	4.65	59	6	3	High	1	1.013	1.027	1.283	1.354	1.301	1.286	1.342	1.266	1.183	1.199	1.363	0.817	0.837	0.939
TRINITY_DN2170_c4_g1_i2_orf1	beta-1,3-glucan-binding protein-like isoform X1 [Ostrinia furnacalis]	5	392	43.8	5.71	16	5	5	High	1	1.025	0.974	1.542	1.67	1.507	1.556	1.656	1.506	1.654	1.491	1.768	1.603	1.597	1.611
TRINITY_DN3325_c0_g1_i1_orf1	histone H2A.V [Plutella xylostella]	3	129	13.4	10.55	26	10	2	High	1	0.975	0.991	1.144	1.174	1.152	1.259	1.29	1.278	1.163	0.995	1.23	1.305	1.333	1.243
TRINITY_DN3616_c0_g2_i2_orf1	conotoxin ArMKLT2-032-like [Ostrinia furnacalis]	4	80	8.7	5.11	64	7	1	High	1	1.013	0.923	1.676	1.852	2.052	2.292	2.546	2.011	1.801	1.675	1.747	1.132	0.833	0.983
TRINITY_DN24121_c1_g1_i6_orf1	serine protease persephone-like [Ostrinia furnacalis]	7	255	28.9	6.67	29	10	7	High	1	0.952	0.974	2.741	3.086	2.695	2.651	2.788	2.615	1.194	1.206	1.458	0.809	0.791	0.859
TRINITY_DN27852_c0_g1_i1_orf1	baculoviral IAP repeat-containing protein 6-like [Ostrinia furnacalis]	6	1339	147.1	5.55	6	6	6	High	1	1.005	0.966	0.536	0.605	0.62	0.492	0.507	0.519	0.585	0.627	0.617	0.735	0.717	0.68
TRINITY_DN5344_c0_g1_i7_orf1	ADP-ribosylation factor 1 [Eumeta japonica]	4	104	11.9	4.79	29	8	1	High	1	0.872	0.98	0.856	0.798	1.014	0.911	1.056	1.072	0.921	0.865	0.78	0.942	0.948	1.048
TRINITY_DN57137_c0_g1_i1_orfp1	TRINITY_DN57137_c0_g1_i1_m.46420 TRINITY_DN57137_c0_g1::TRINITY_DN57	3	55	5.8	8.19	33	6	3	High	1	0.936	0.911	8.355	9.766	8.288	9.1	9.833	8.288	6.167	5.286	8.108	3.461	3.667	3.975
TRINITY_DN136031_c0_g1_i7_orf1	ferritin, lower subunit isoform X3 [Spodoptera litura]	3	97	11.1	8	28	15	1	High	1	0.913	0.935	6.291	5.855	6.865	3.199	3.354	3.328	0.717	0.729	0.76	0.878	0.656	0.907
TRINITY_DN17906_c0_g1_i1_orf1	phosphoglucose-6-isomerase [Ostrinia furnacalis]	6	108	12	7.5	49	8	5	High	1	1.037	0.991	1.2	0.914	1.193	1.239	1.082	1.278	1.467	1.278	0.988	1.042	1.109	0.986
TRINITY_DN17071_c0_g1_i6_orf1	serine/arginine-rich splicing factor 7-like [Ostrinia furnacalis]	4	176	20.1	11.43	33	7	3	High	1	0.98	1.018	0.937	0.945	0.92	0.807	0.824	1.039	0.704	0.805	0.865	0.919	0.845	0.965
TRINITY_DN896_c0_g1_i2_orf1	uncharacterized protein LOC114356314 isoform X2 [Ostrinia furnacalis]	4	230	24.4	9.35	22	5	4	High	1	0.959	1.053	1.755	1.895	1.853	1.348	1.367	1.297	1.093	1.03	1.034	1.871	1.994	1.861
TRINITY_DN59388_c0_g1_i1_orf1	uncharacterized protein LOC114353759 [Ostrinia furnacalis]	5	187	20.8	7.03	36	8	5	High	1	0.97	1.012	2.289	2.441	2.168	1.472	1.495	1.419	1.793	1.739	1.983	3.681	3.653	3.62
TRINITY_DN452_c0_g1_i4_orf1	protein CREG1 [Ostrinia furnacalis]	7	247	28.4	7.56	19	8	7	High	1	1.033	1.08	1.857	1.905	1.989	1.585	1.824	1.694	1.241	1.243	1.221	1.33	1.295	1.216
TRINITY_DN9593_c0_g1_i2_orf1	uncharacterized protein LOC113518937 [Galleria mellonella]	3	136	15	5.58	32	4	3	High	1	1.113	1.108	1.491	1.57	1.43	2.096	2.204	2.144	2.828	2.865	3.002	2.307	2.329	2.205
TRINITY_DN1123_c2_g1_i3_orf1	troponin I isoform X8 [Ostrinia furnacalis]	7	88	10.3	9.76	55	8	3	High	1	1.026	1	1.15	1.212	1.21	1.009	1.031	1.01	0.911	0.733	0.982	0.299	0.36	0.323
TRINITY_DN1310_c0_g1_i4_orf1	trypsin-like isoform X1 [Ostrinia furnacalis]	3	254	26.7	4.32	26	7	3	High	1	1.032	0.99	0.145	0.165	0.175	0.194	0.203	0.199	0.208	0.242	0.187	0.222	0.184	0.212
TRINITY_DN20363_c1_g1_i1_orf1	probable phosphoserine aminotransferase [Galleria mellonella]	2	61	6.8	6.54	51	7	1	High	1	0.995	0.9	1.018	0.873	0.995	0.883	0.862	0.882	0.915	0.965	0.946	0.973	1.032	1.033
TRINITY_DN4954_c0_g1_i5_orf1	glycogen debranching enzyme [Trichoplusia ni]	5	423	46.6	6.87	17	9	5	High	1	1.007	0.993	1.432	1.347	1.442	1.223	1.174	1.202	1.387	1.308	1.327	1.316	1.346	1.322
TRINITY_DN3924_c0_g1_i5_orf1	SH3 domain-containing kinase-binding protein 1-like isoform X1 [Ostrinia furi	7	740	81.1	7.58	11	8	7	High	1	1.01	0.996	0.789	0.784	0.824	0.771	0.747	0.756	0.737	0.595	0.72	0.814	0.813	0.805
TRINITY_DN47114_c0_g1_i5_orf1	nucleolar protein dao-5 isoform X2 [Ostrinia furnacalis]	7	684	72.7	8.94	10	10	7	High	1	1.044	0.993	0.306	0.31	0.343	0.281	0.283	0.289	0.22	0.221	0.219	0.275	0.271	0.281
TRINITY_DN108051_c0_g1_i2_orf1	uncharacterized protein LOC114351921 [Ostrinia furnacalis]	4	161	18.7	9.85	24	5	4	High	1	1.006	0.986	0.494	0.442	0.5	0.488	0.444	0.453	0.46	0.504	0.443	0.472	0.512	0.491
TRINITY_DN1767_c0_g2_i15_orf1	fasciclin-2 isoform X3 [Ostrinia furnacalis]	6	835	92	6.24	9	6	6	High	1	0.988	1.021	1.446	1.483	1.503	1.235	1.457	1.389	1.497	1.428	1.368	1.932	1.906	1.912
TRINITY_DN36699_c0_g1_i3_orf1	pyridoxal phosphate homeostasis protein [Ostrinia furnacalis]	5	254	28.3	6.79	24	7	5	High	1	1.011	1.035	0.971	0.967	0.891	0.822	0.846	0.83	0.839	0.921	0.826	0.805	0.797	0.803
TRINITY_DN17031_c0_g1_i1_orf1	arginase, hepatic [Ostrinia furnacalis]	5	341	37.4	6.6	18	7	5	High	1	0.991	1.012	1.515	1.418	1.43	1.651	1.561	1.732	1.698	1.522	1.499	1.319	1.373	1.276
TRINITY_DN10007_c0_g1_i1_orf1	28S ribosomal protein S31, mitochondrial [Ostrinia furnacalis]	5	366	42.1	9.17	20	8	5	High	1	0.997	0.978	0.514	0.546	0.56	0.632	0.655	0.575	0.578	0.855	0.613	0.537	0.521	0.53
TRINITY_DN3619_c0_g2_i1_orf1	transport and Golgi organization protein 11 [Ostrinia furnacalis]	4	225	25.3	6.96	32	4	4	High	1	0.978	1.006	0.681	0.75	0.791	0.685	0.73	0.716	0.685	0.761	0.663	0.854	0.787	0.823
TRINITY_DN30510_c0_g1_i6_orf1	spodomicin-like [Ostrinia furnacalis]	3	62	6.7	6.73	60	23	3	High	1	0.961	0.967	8.65	9.33	8.505	6.132	6.3	6.102	4.197	3.59	5.068	1.233	1.131	1.257
TRINITY_DN6044_c0_g1_i4_orf1	acyl-CoA-binding protein-like [Ostrinia furnacalis]	5	85	9.5	9.14	54	7	5	High	1	1.05	0.974	0.103	0.103	0.144	0.115	0.121	0.118	0.136	0.124	0.138	0.129	0.135	0.131
TRINITY_DN2921_c1_g1_i4_orf1	uncharacterized protein LOC114352340 isoform X2 [Ostrinia furnacalis]	6	209	23.6	5.86	35	6	5	High	1	0.966	1.172	1.57	1.913	1.66	2.015	1.731	1.505	1.518	1.608	1.578	1.571	1.783	1.616
TRINITY_DN13221_c0_g1_i3_orf1	fasciclin-3-like [Ostrinia furnacalis]	6	173	19	5.87	46	11	1	High	1	0.941	1.029	0.31	0.315	0.312	0.742	0.794	0.744	1.213	1.525	1.515	3.076	3.04	3.089
TRINITY_DN1601_c0_g1_i4_orf1	cytoplasmic dynein 1 intermediate chain isoform X4 [Ostrinia furnacalis]	8	678	74.9	5.52	14	9	8	High	1	0.999	1.026	0.74	0.82	0.786	0.829	0.826	0.798	0.78	0.817	0.906	1.072	1.069	1.071
TRINITY_DN479_c6_g1_i2_orf1	beta-1,3-glucan-binding protein-like [Ostrinia furnacalis]	5	480	53.4	6.21	11	5	5	High	1	1.04	1.035	1.744	1.685	1.783	1.466	1.472	1.556	1.405	1.581	1.568	2.434	2.485	2.504
TRINITY_DN2331_c0_g1_i1_orf1	heat shock 70 kDa protein cognate 4-like, partial [Zerene cesonia]	4	113	12.7	5.01	22	15	2	High	1	0.95	0.991	0.791	1.021	0.791	0.787	0.926	0.699	0.865	0.921	1.097	0.817	0.706	0.892
TRINITY_DN35051_c0_g1_i1_orf1	uncharacterized protein LOC114364307 [Ostrinia furnacalis]	5	128	14.8	4.63	30	8	5	High	1	1.023	0.982	0.102	0.108	0.119	0.15	0.153	0.161	0.127	0.127	0.129	0.131	0.125	0.132
TRINITY_DN5731_c0_g1_i13_orf1	unnamed protein product [Parnassius apollo]	6	475	54.3	8.62	16	7	6	High	1	0.976	0.999	1.047	1.032	1.002	1.03	1.116	1.02	0.974	1.154	1.077	1.135	1.049	1.167
TRINITY_DN15339_c0_g1_i6_orf1	nuclear pore glycoprotein p62 isoform X3 [Ostrinia furnacalis]	4	498	51.1	4.77	9	5	4	High	1	0.989	0.954	0.694	0.672	0.727	0.752	0.852	0.882	0.618	0.61	0.653	0.725	0.775	0.697
TRINITY_DN1424_c0_g1_i5_orf1	insect group I lytic polysaccharide monooxygenase [Ostrinia furnacalis]	6	343	39	7.03	21	8	6	High	1	0.934	0.994	2.169	2.066	2.107	2.48	2.361	2.514	2.404	2.271	2.34	2.029	2.009	2.105
TRINITY_DN2082_c0_g1_i2_orf1	choline-phosphate cytidylyltransferase B-like isoform X1 [Ostrinia furnacalis]	5	403	45.3	5.6	17	5	5	High	1	0.985	0.977	0.411	0.453	0.476	0.49	0.482	0.469	0.441	0.409	0.424	0.547	0.528	0.556
TRINITY_DN4304_c0_g1_i3_orf1	TOM1-like protein 2 isoform X4 [Galleria mellonella]	6	493	52.9	5	22	6	6	High	1	0.99	0.98	1.128	1.236	1.268	1.219	1.353	1.293	1.224	1.184	1.175	1.158	1.082	1.138
TRINITY_DN31399_c0_g1_i3_orf1	ATP-dependent zinc metalloprotease YME1 homolog [Ostrinia furnacalis]	7	777	85.3	8.92	11	8	7	High	1	0.994	1.068	0.737	0.847	0.793	0.798	0.878	0.768	0.687	0.759	0.824	0.829	0.816	0.823
TRINITY_DN15755_c0_g1_i1_orf1	cytochrome P450 monooxygenase CYP6AB141 [Ostrinia furnacalis]	6	238	27.4	5.86	28	7	3	High	1	1.129	1.072	2.559	2.437	2.47	2.72	2.847	3.031	1.439	1.753	1.639	1.052	1.048	1.025
TRINITY_DN5453_c0_g1_i2_orf1	PREDICTED: MOB kinase activator-like 1 [Papilio polytes]	4	219	25.2	7.01	17	5	4	High	1	0.848	0.775	1.132	1.237	1.079	0.929	1.064	1.03	1.076	1.042	1.004	0.951	0.939	0.983
TRINITY_DN22242_c0_g1_i1_orf1	juvenile hormone epoxide hydrolase-like [Ostrinia furnacalis]	7	198	22.9	7.47	26	10	7	High	1	1.019	1.019	1.6	1.417	1.514	1.765	1.606	1.794	1.442	1.264	1.29	1.344	1.428	1.359
TRINITY_DN7839_c0_g1_i4_orf1	elongin-C [Ostrinia furnacalis]	3	150	15.9	4.74	28	6	3	High	1	1.005	1.014	0.823	0.779	0.803	0.735	0.759	0.794	0.746	0.667	0.662	0.824	0.793	0.77
TRINITY_DN12497_c0_g1_i1_orf1	probable N-acetyltransferase san [Ostrinia furnacalis]	5	177	20.2	8.24	36	5	5	High	1	1.007	0.997	0.363	0.314	0.378	0.328	0.353	0.376	0.326	0.328	0.295	0.418	0.405	0.393

TRINITY_DN9109_c0_g1_i1_orf1	unnamed protein product [Chrysodeixis includens]	5	393	42.3	6.71	14	5	5	High	1	0.975	0.996	0.905	1.03	0.953	1.271	1.208	1.154	0.986	0.9	0.954	1.368	1.355	1.414
TRINITY_DN50517_c0_g1_i1_orf1	cuticle protein 21-like [Ostrinia furnacalis]	4	197	20.4	7.01	36	5	2	High	1	0.918	1.041	1.642	1.726	1.623	1.625	1.547	1.561	1.501	1.393	1.769	1.807	1.733	1.781
TRINITY_DN15458_c0_g1_i3_orf1	integrin alpha-PS1 isoform X1 [Ostrinia furnacalis]	6	1124	124.4	6.54	7	6	6	High	1	0.951	1.014	0.856	0.868	0.898	0.858	0.834	0.882	0.826	0.732	0.767	0.974	0.959	0.84
TRINITY_DN76333_c0_g1_i2_orf1	larval cuticle protein 65Ag1-like [Ostrinia furnacalis]	4	139	15.6	6.05	43	10	4	High	1	1.051	0.957	1.194	1.254	1.213	1.366	1.377	1.249	1.18	1.094	1.353	0.274	0.252	0.26
TRINITY_DN4008_c0_g1_i7_orf1	nuclear export mediator factor NEMF homolog isoform X1 [Ostrinia furnacalis]	5	1024	116.2	6.7	5	5	5	High	1	1.088	0.963	0.714	0.77	0.691	0.625	0.541	0.597	0.554	0.472	0.546	0.601	0.531	0.525
TRINITY_DN23360_c0_g1_i3_orf1	protein PTCD3 homolog, mitochondrial [Ostrinia furnacalis]	5	714	78.8	5.15	8	5	5	High	1	0.992	1.024	0.52	0.479	0.489	0.492	0.511	0.484	0.498	0.454	0.476	0.702	0.629	0.639
TRINITY_DN11826_c0_g1_i4_orf1	aldehyde dehydrogenase X, mitochondrial-like [Ostrinia furnacalis]	3	113	11.9	6.57	48	6	3	High	1	1.014	0.961	2.553	1.947	2.225	3.176	2.731	3.592	4.064	3.888	2.852	3.504	3.447	3.534
TRINITY_DN463_c0_g1_i3_orf1	supervillin isoform X2 [Ostrinia furnacalis]	6	1458	164.1	6.11	5	6	6	High	1	0.937	0.937	0.938	0.941	0.92	0.929	0.947	0.91	1.012	0.833	1	1.076	1.079	1.018
TRINITY_DN82008_c0_g1_i1_orf1	GMP reductase 1-like [Ostrinia furnacalis]	6	346	37.6	7.21	23	6	6	High	1	1.004	1.039	0.602	0.629	0.657	0.626	0.646	0.661	0.59	0.559	0.535	0.604	0.561	0.569
TRINITY_DN22422_c0_g1_i4_orf1	2-hydroxyacylsphingosine 1-beta-galactosyltransferase-like [Ostrinia furnacali	6	473	53.8	8.75	13	6	2	High	1	0.996	1.063	0.899	0.796	0.812	0.803	0.921	0.894	0.96	1.05	0.833	0.978	0.868	1
TRINITY_DN9742_c0_g1_i5_orf1	ADP-ribosylation factor-like protein 1 [Bombyx mori]	4	180	20.1	6.57	34	4	4	High	1	0.999	1.063	1.096	1.025	1.104	1.063	1.196	1.216	1.126	1.284	0.945	1.179	1.085	1.203
TRINITY_DN2848_c0_g1_i2_orf1	glyceraldehyde-3-phosphate dehydrogenase isoform 1 [Homo sapiens]	3	335	36	8.44	16	5	2	High	1	0.942	0.934	1.481	1.62	1.613	1.601	1.722	1.664	1.969	1.896	2.029	1.819	1.866	1.707
TRINITY_DN98091_c0_g1_i3_orf1	UDP-glycosyltransferase UGT40AP2, partial [Ostrinia furnacalis]	4	511	57.4	8.81	11	5	4	High	1	0.923	0.915	0.221	0.227	0.25	0.249	0.263	0.271	0.321	0.311	0.282	0.302	0.285	0.282
TRINITY_DN12331_c0_g1_i5_orf1	septin-7 isoform X1 [Ostrinia furnacalis]	3	697	75.8	9.03	7	4	3	High	1	1.076	0.967	0.966	0.979	1.148	1.109	1.01	1.054	1.054	0.992	1.095	1.76	1.66	1.635
TRINITY_DN37599_c0_g1_i1_orf1	bmp-2 protein isoform X3 [Bombyx mori]	4	90	9.8	9.99	50	7	4	High	1	1.002	1.041	0.637	0.677	0.702	0.597	0.613	0.565	0.571	0.517	0.604	0.784	0.818	0.801
TRINITY_DN14298_c0_g3_i1_orf1	kinesin heavy chain [Ostrinia furnacalis]	5	253	28.7	9.58	22	7	2	High	1	1.049	0.987	0.799	0.861	0.811	0.792	0.711	0.671	0.599	0.552	0.679	1.077	1.142	1.048
TRINITY_DN10530_c0_g1_i1_orf1	cytochrome c oxidase subunit NDUFA4 [Ostrinia furnacalis]	5	81	9.2	9.58	48	13	5	High	1	0.986	0.977	0.374	0.37	0.428	0.359	0.359	0.354	0.37	0.373	0.374	0.394	0.389	0.369
TRINITY_DN101358_c0_g2_i1_orf1	glycylpeptide N-tetradecanoyltransferase 2 [Ostrinia furnacalis]	7	470	54.1	8.31	14	8	7	High	1	0.949	0.993	0.843	0.834	0.819	0.717	0.718	0.754	0.717	0.724	0.826	0.85	0.882	0.911
TRINITY_DN8261_c0_g1_i1_orf1	UDP-N-acetylhexosamine pyrophosphorylase-like protein 1 [Ostrinia furnacali	5	487	54.6	6.09	11	5	5	High	1	0.954	0.941	0.43	0.396	0.469	0.431	0.41	0.445	0.429	0.43	0.415	0.578	0.541	0.536
TRINITY_DN940_c0_g1_i4_orf1	uncharacterized protein LOC114357075 [Ostrinia furnacalis]	5	255	27	7.27	24	5	2	High	1	0.871	0.953	0.494	0.608	0.586	0.655	0.622	0.781	0.743	1.038	0.703	0.623	0.549	0.592
TRINITY_DN147517_c0_g1_i1_orf1	eukaryotic translation initiation factor 4 gamma 3-like isoform X5 [Ostrinia furr	4	482	51.1	9.19	13	5	4	High	1	1.06	0.988	0.699	0.695	0.725	0.669	0.713	0.654	0.604	0.536	0.641	0.64	0.682	0.629
TRINITY_DN23640_c0_g1_i5_orf1	proteasomal ATPase-associated factor 1-like [Ostrinia furnacalis]	4	396	43.3	6.52	12	4	4	High	1	1.036	0.987	1.26	1.153	1.232	1.263	1.352	1.4	1.317	1.277	1.374	1.218	1.367	1.321
TRINITY_DN3066_c0_g1_i5_orf1	thioredoxin domain-containing protein [Ostrinia furnacalis]	5	315	35.6	8.35	19	7	5	High	1	0.988	1.029	0.874	0.786	0.838	0.888	0.929	0.915	1.027	1.077	0.815	0.989	1.06	1.042
TRINITY_DN49570_c0_g1_i1_orf1	transmembrane emp24 domain-containing protein bai [Ostrinia furnacalis]	5	205	23.7	5.72	19	7	5	High	1	1.02	0.945	0.952	0.978	1.015	0.918	0.923	0.981	0.899	0.839	0.917	1.044	1.02	1.109
TRINITY_DN3310_c0_g1_i1_orf1	hypothetical protein evm_010516 [Chilo suppressalis]	5	358	40.4	8.72	17	5	5	High	1	0.967	1.026	0.642	0.617	0.647	0.705	0.618	0.66	0.706	0.626	0.647	5.56	5.902	5.226
TRINITY_DN35377_c0_g1_i3_orf1	unnamed protein product [Chilo suppressalis]	2	149	16.5	7.87	21	6	2	High	1	1.018	1.002	0.578	0.58	0.632	0.696	0.711	0.693	0.794	0.769	0.71	0.861	0.908	0.899
TRINITY_DN18388_c0_g1_i6_orf1	serine protease [Ostrinia furnacalis]	4	426	47	7.78	13	6	4	High	1	0.955	0.961	1.949	1.959	1.955	1.893	1.8	1.803	2.427	2.054	2.541	2.676	2.613	2.412
TRINITY_DN15786_c0_g1_i1_orf1	echinoderm microtubule-associated protein-like 2 isoform X3 [Ostrinia furnaca	5	874	95.5	7.2	7	5	5	High	1	0.99	1.033	0.972	1.065	1.017	0.925	0.907	0.921	0.83	0.856	0.86	0.891	0.91	0.977
TRINITY_DN40028_c0_g1_i1_orf1	signal recognition particle receptor subunit alpha homolog [Ostrinia furnacalis	6	600	66.5	7.49	9	6	6	High	1	1.006	0.965	0.836	0.809	0.884	0.826	0.743	0.837	0.742	0.711	0.661	0.798	0.8	0.777
TRINITY_DN4036_c0_g2_i1_orf1	microvitellogenin-like [Ostrinia furnacalis]	5	289	33.5	6.24	20	8	5	High	1	1.014	0.978	0.517	0.51	0.548	0.553	0.573	0.576	0.317	0.267	0.324	0.484	0.463	0.437
TRINITY_DN11746_c0_g2_i1_orf1	splicing factor 3B subunit 1 isoform X1 [Diprion similis]	5	1315	147.7	7.01	4	5	5	High	1	1.068	1.039	0.753	0.755	0.845	0.815	0.976	0.923	0.818	0.715	0.655	0.732	0.741	0.723
TRINITY_DN17772_c0_g1_i4_orf1	S-methyl-5'-thioadenosine phosphorylase [Galleria mellonella]	4	279	30.4	6.54	18	5	4	High	1	0.988	0.994	0.973	0.995	1.012	1.128	1.167	1.157	1.15	1.165	1.048	1.001	0.998	0.956
TRINITY_DN10257_c0_g1_i2_orf1	prefoldin subunit domain-containing protein [Phthorimaea operculella]	5	128	14.8	9.11	41	9	5	High	1	0.98	0.977	0.72	0.689	0.748	0.755	0.728	0.735	0.846	0.733	0.704	0.813	0.798	0.829
TRINITY_DN6510_c1_g1_i1_orf1	hypothetical protein evm_008080 [Chilo suppressalis]	5	1123	127.9	6.74	7	5	5	High	1	0.928	1.022	1.341	1.416	1.286	1.318	1.381	1.239	1.217	1.539	1.168	1.632	1.56	1.651
TRINITY_DN7633_c0_g1_i1_orf1	prolow-density lipoprotein receptor-related protein 1, partial [Ostrinia furnaca	6	579	62.4	5.88	13	6	6	High	1	1.098	1.043	1.867	1.701	1.646	2.144	2.024	2.053	1.932	1.841	1.859	2.28	2.081	2.315
TRINITY_DN56998_c0_g1_i2_orf1	glycerate kinase [Ostrinia furnacalis]	4	511	55.6	6.64	10	4	4	High	1	1.051	0.984	1.07	0.947	1.06	1.257	1.152	1.282	0.928	0.965	0.888	0.939	0.98	0.866
TRINITY_DN3113_c1_g2_i1_orf1	short-chain dehydrogenase/reductase family 16C member 6-like [Ostrinia furr	5	330	37.1	7.39	18	9	5	High	1	1.05	1.038	0.785	0.703	0.765	0.692	0.675	0.713	0.681	0.684	0.606	0.841	0.871	0.84
TRINITY_DN5556_c0_g1_i3_orf1	hypothetical protein evm_006874 [Chilo suppressalis]	4	456	50.4	5.22	11	5	4	High	1	0.986	1.054	1.216	1.289	1.226	1.341	1.419	1.425	1.412	1.352	1.403	1.41	1.413	1.414
TRINITY_DN1481_c0_g1_i4_orf1	G protein-coupled receptor kinase 1 isoform X2 [Helicoverpa armigera]	6	701	80.2	7.17	10	6	6	High	1	1.103	1.112	1.101	1.229	1.077	1.1	1.096	1.036	0.987	1.573	1.05	1.675	1.497	1.625
TRINITY_DN18027_c0_g2_i1_orf1	vanin-like protein 2 isoform X2 [Ostrinia furnacalis]	5	523	59.5	6.34	12	6	3	High	1	0.93	0.951	1.196	1.121	1.201	1.169	1.135	1.388	1.443	1.218	1.243	1.735	1.715	1.654
TRINITY_DN3539_c0_g1_i7_orf1	transcription elongation regulator 1-like [Ostrinia furnacalis]	5	186	22.1	9.2	27	6	5	High	1	0.973	0.932	0.624	0.703	0.66	0.544	0.625	0.574	0.549	0.525	0.59	0.767	0.747	0.729
TRINITY_DN8095_c0_g1_i3_orf1	circadian clock-controlled protein-like [Ostrinia furnacalis]	4	246	27.1	9.06	18	4	4	High	1	1.109	1.033	2.235	2.198	2.245	2.177	2.009	2.014	2.274	2.325	1.967	1.037	0.83	0.949
TRINITY_DN354_c0_g1_i2_orf1	COP9 signalosome complex subunit 1 [Ostrinia furnacalis]	4	482	54.3	6.61	13	4	4	High	1	0.932	1.055	0.947	0.988	0.955	0.847	0.98	0.833	0.989	1.092	0.9	0.992	0.908	0.921
TRINITY_DN3454_c0_g1_i1_orf1	MICOS complex subunit MIC13 homolog QIL1 [Ostrinia furnacalis]	4	121	13.3	8.27	55	5	4	High	1	1.051	0.984	0.621	0.607	0.592	0.631	0.611	0.63	0.664	0.803	0.646	0.533	0.567	0.611
TRINITY_DN48250_c0_g1_i1_orf1	larval/pupal rigid cuticle protein 66-like [Hyposmocoma kahamanoa]	3	105	11	5.83	33	6	1	High	1	1.006	0.939	1.468	1.174	1.397	1.615	1.583	1.515	1.856	1.706	1.713	1.721	1.786	1.691
TRINITY_DN1772_c7_g1_i7_orf1	sulfotransferase family cytosolic 1B member 1-like [Ostrinia furnacalis]	4	238	27.6	7.28	20	6	2	High	1	1.018	1.038	0.564	0.488	0.539	0.629	0.628	0.664	0.667	0.638	0.537	0.66	0.617	0.633
TRINITY_DN10373_c0_g1_i1_orf1	homocysteine S-methyltransferase 1-like [Ostrinia furnacalis]	4	341	38.2	5.41	16	5	4	High	1	0.948	0.953	2.059	1.956	1.942	1.573	1.51	1.523	1.211	1.048	1.276	1.831	1.773	1.77
TRINITY_DN36682_c0_g1_i1_orf1	uncharacterized protein DDB_G0287625-like [Ostrinia furnacalis]	5	393	42.7	12.48	16	5	5	High	1	0.919	1.012	0.661	0.809	0.753	0.748	0.809	0.733	0.706	0.654	0.72	0.735	0.739	0.625
TRINITY_DN23783_c0_g2_i1_orf1	cytochrome b5 [Ostrinia furnacalis]	4	133	14.8	5.36	36	7	4	High	1	1.019	0.968	0.619	0.609	0.628	0.781	0.723	0.757	0.632	0.557	0.623	0.816	0.826	0.763
TRINITY_DN3980_c0_g2_i1_orf1	beta-parvin [Ostrinia furnacalis]	6	360	41.1	6	20	7	6	High	1	1	0.949	1.089	0.991	1.06	1.049	0.999	1.053	1.085	1.08	0.959	0.947	0.941	0.882
TRINITY_DN12757_c0_g1_i1_orf1	proteasome subunit alpha type-7-1-like [Ostrinia furnacalis]	4	249	28	8.21	16	9	4	High	1	1.024	1.022	1.037	0.987	1.017	0.961	0.965	0.999	0.957	0.896	0.904	1.125	1.164	1.1
TRINITY_DN45220_c0_g1_i1_orf1	delta(3,5)-Delta(2,4)-dienoyl-CoA isomerase, mitochondrial isoform X1 [Ostrin	4	68	7.4	8.75	59	4	1	High	1	1.245	1.295	1.844	2.226	1.976	2.555	2.754	2.678	3.525	2.517	3.043	4.459	4.564	4.138
TRINITY_DN147427_c0_g1_i1_orf1	importin subunit alpha-4 [Ostrinia furnacalis]	5	514	56.6	5.22	12	7	5	High	1	0.969	1.002	0.758	0.823	0.743	0.8	0.823	0.774	0.78	0.692	0.892	0.868	0.872	0.888
TRINITY_DN12671_c0_g1_i4_orf1	hemicentin-1-like isoform X1 [Ostrinia furnacalis]	5	792	89.1	4.91	9	5	5	High	1	1	0.984	1.213	1.264	1.325	1.31	1.17	1.312	1.302	1.312	1.206	4.081	3.938	3.756
TRINITY_DN5238_c0_g1_i2_orf1	DNA-(apurinic or apyrimidinic site) lyase [Ostrinia furnacalis]	5	656	73.1	6.06	11	5	5	High	1	1.025	0.985	0.39	0.36	0.399	0.352	0.28	0.296	0.235	0.261	0.228	0.517	0.512	0.487
TRINITY_DN2663_c0_g1_i12_orf1	WW domain-binding protein 2 isoform X1 [Bombyx mori]	5	303	31.6	8.21	19	7	5	High	1	1.017	1.052	1.1	0.944	1.077	1.078	1.067	1.147	1.343	1.392	1.257	1.164	1.1	1.188
TRINITY_DN29969_c0_g1_i5_orf1	twitchin-like [Ostrinia furnacalis]	5	143	16.3	8.07	38	6	1	High	1	0.746	0.639	1.302	2.069	1.538	0.762	0.604	0.995	0.685	0.905	1.043	0.461	0.478	0.575
TRINITY_DN82324_c0_g1_i4_orf1	hypothetical protein evm_001824 [Chilo suppressalis]	4	123	13.7	10.13	37	9	4	High	1	1.081	1.051	0.692	0.712	0.721	0.601	0.529	0.602	0.485	0.517	0.529	0.54	0.558	0.536
TRINITY_DN7754_c0_g1_i2_orf1	carboxypeptidase E-like isoform X1 [Ostrinia furnacalis]	4	480	54	6.32	11	6	4	High	1	1.014	1.136	1.697	1.722	1.704	1.565	1.508	1.645	1.688	1.485	1.553	1.687	1.72	1.617
TRINITY_DN18036_c0_g1_i7_orf1	pentatricopeptide repeat-containing protein 2, mitochondrial-like [Ostrinia fu	6	379	44.3	8.81	16	6	6	High	1	1.025	1.02	0.36	0.337	0.418	0.347	0.358	0.366	0.348	0.32	0.348	0.423	0.427	0.428
TRINITY_DN132_c0_g2_i2_orf1	alpha-tocopherol transfer protein-like isoform X1 [Ostrinia furnacalis]	6	322	37.7	7.72	20	6	6	High	1	0.948	0.927	1.729	1.825	1.72	1.102	1.208	1.083	1.028	1.102	1.117	1.578	1.566	1.61
TRINITY_DN2749_c4_g1_i2_orf1	RNA exonuclease 4-like [Ostrinia furnacalis]	4	87	9.2	7.88	53	7	3	High	1	1.007	0.975	0.339	0.572	0.393	1.052	1.061	0.939	1.422	1.588	1.804	0.522	0.572	0.554
TRINITY_DN496_c0_g1_i7_orf1	unnamed protein product [Diatraea saccharalis]	4	888	98.1	8	6	6	4	High	1	1.03	1.037	0.985	1.041	0.854	0.849	0.806	0.829	0.761	0.686	0.738	0.658	0.706	0.605
TRINITY_DN115082_c0_g1_i5_orf1	protein dj-1beta-like isoform X2 [Ostrinia furnacalis]	4	107	11.4	9.01	45	8	1	High	1	1.027	0.96	1.164	1.082	1.063	0.413	0.525	0.482	0.615	0.509	0.559	0.32	0.322	0.318
TRINITY_DN8454_c0_g1_i4_orf1	translocon-associated protein subunit alpha [Ostrinia furnacalis]	4	280	31.2	4.61	16	9	4	High	1	0.987	1	1.002	1.077	0.999	0.887	0.955	0.904	0.809	0.721	0.897	0.674	0.672	0.692
TRINITY_DN2836_c0_g1_i4_orf1	protein Diedel [Drosophila sechellia]	4	87	9.6	6.47	61	6	4	High	1	0.968	0.923	1.257	1.286	1.27	1.138	1.067	1.035	1.084	1.015	1.29	1.33	1.317	1.302
TRINITY_DN27728_c0_g1_i1_orf1	hypothetical protein evm_008152 [Chilo suppressalis]	6	555	63	6.35	13	6	6	High	1	1.01	1.061	0.887	0.78	0.907	0.89	0.916	0.984	1.008	0.892	0.841	0.985	0.982	0.928
TRINITY_DN4898_c0_g1_i7_orf1	annulin-like isoform X3 [Ostrinia furnacalis]	7	762	86.1	6.27	9	7	7	High	1	0.99	1.061	0.8	0.806	0.77	0.806	0.766	0.721	0.739	0.77	0.73	1.643	1.696	1.589
TRINITY_DN5562_c1_g2_i1_orf1	cell division cycle and apoptosis regulator protein 1-like [Ostrinia furnacalis]	4	476	54	5.19	13	5	4	High	1	1.028	1.041	0.776	0.683	0.75	0.695	0.776	0.765	0.806	0.843	0.687	0.814	0.832	0.803
TRINITY_DN23364_c0_g1_i1_orf1	PREDICTED: uncharacterized protein LOC106134920 [Amyelois transitella]	3	151	15.7	9.2	34	7	3	High	1	1.07	0.999	1.429	1.737	1.261	1.545	1.429	1.486	1.628	1.475	1.68	2.682	2.934	2.197
TRINITY_DN295_c5_g1_i2_orf1	unnamed protein product [Chilo suppressalis]	5	177	19.9	6.79	24	10	5	High	1	0.997	1.107	2.987	3.074	2.972	2.729	2.969	2.666	4.008	3.932	4.309	1.949	2.04	1.844
TRINITY_DN646_c0_g1_i5_orf1	unnamed protein product [Diatraea saccharalis]	5	724	79.9	9.52	8	5	5	High	1	1.013	0.988	0.528	0.54	0.536	0.544	0.543	0.564	0.514	0.514	0.501	0.612	0.656	0.66
TRINITY_DN1768_c0_g1_i2_orf1	low-density lipoprotein receptor-related protein 1B-like [Ostrinia furnacalis]	4	530	55.3	6.4	9	5	4	High	1	1.033	1.078	1.046	1.015	1.044	1.104	1.264	1.263	1.038	1.112	1.103	1.375	1.352	1.242
TRINITY_DN23398_c0_g1_i1_orf1	cytochrome P450 6B7-like [Ostrinia furnacalis]	6	500	57.2	8.53	12	6	6	High	1	1.146	1.124	1.916	1.857	2.014	2.387	2.429	2.458	2.347	2.333	2.003	2.584	2.524	2.425
TRINITY_DN4688_c0_g1_i2_orf1	uncharacterized protein LOC114359411 [Ostrinia furnacalis]	6	629	71.4	8.18	10	7	6	High	1	0.916	1.028	1.18	1.258	1.211	1.234	1.362	1.276	1.162	1.044	1.12	2.531	2.678	2.64
TRINITY_DN8037_c0_g2_i1_orf1	2-oxo-4-hydroxy-4-carboxy-5-ureidoimidazoline decarboxylase-like [Ostrini	4	173	19.9	6.79	24	9	4	High	1	0.957	1.024	2.212	2.2	2.087	1.625	1.739	1.63	1.843	1.86	1.992	1.913	1.928	1.864
TRINITY_DN3355_c0_g2_i4_orf1	UDP-glycosyltransferase UGT33AL1 [Ostrinia furnacalis]	6	530	60.5	8.03	13	7	4	High	1	1.049	0.999	0.412	0.441	0.443	0.512	0.527	0.567	0.464	0.59	0.579	0.357	0.378	0.405
TRINITY_DN3472_c0_g1_i6_orf1	unnamed protein product [Euphydryas editha]	5	267	29.4	5.74	20	8	5	High	1	0.99	0.954	0.803	0.804	0.818	0.945	0.966	0.889	1.036	0.971	1.087	0.819	0.83	0.83
TRINITY_DN2065_c1_g2_i1_orf1	2-amino-3-ketobutyrate coenzyme A ligase, mitochondrial [Ostrinia furnacalis	4	425	46.2	8.12	13	4	4	High	1	1.052	1.023	0.362	0.42	0.43	0.414	0.407	0.44	0.431	0.401	0.423	0.685	0.724	0.701
TRINITY_DN134810_c0_g1_i1_orf1	pancreatic triacylglycerol lipase-like [Ostrinia furnacalis]	2	88	9.7	4.88	51	5	1	High	1	1.089	1.025	1.023	1.515	1.323	1.384	1.486	1.08	0.942	2.014	1.441	0.934	0.965	1.215
TRINITY_DN5112_c0_g1_i1_orf1	unnamed protein product, partial [Iphiclides podalirius]	6	212	25	11.78	28	14	6	High	1	0.99	0.978	0.657	0.657	0.707	0.697	0.708	0.713	0.567	0.527	0.528	0.693	0.678	0.695
TRINITY_DN14389_c0_g1_i4_orf1	DE-cadherin [Ostrinia furnacalis]	5	1491	166.2	4.78	5	5	5	High	1	0.973	1.051	1.088	1.08	1.072	0.969	0.983	0.989	0.983	1.061	1.021	1.299	1.266	1.204
TRINITY_DN3076_c0_g1_i1_orf1	alpha-mannosidase 2 [Ostrinia furnacalis]	5	1169	133.5	7.18	5	6	5	High	1	1.014	1.015	0.915	0.919	0.952	1.038	1.047	1.079	0.952	0.985	0.855	1.061	1.094	1.171
TRINITY_DN4631_c0_g1_i7_orf1	uncharacterized protein LOC114366284 [Ostrinia furnacalis]	5	752	84.8	5.15	10	7	5	High	1	1.002	0.979	0.467	0.488	0.506	0.519	0.539	0.571	0.554	0.583	0.521	0.577	0.526	0.539
TRINITY_DN1466_c0_g1_i4_orf1	insecticyanin-A-like [Ostrinia furnacalis]	5	219	25.4	8.9	23	5	5	High	1	1.035	1.072	2.106	2.018	2.111	1.44	1.345	1.359	2.325	1.984	2.156	1.856	1.938	1.821
TRINITY_DN1824_c0_g2_i2_orf1	branched-chain-amino-acid aminotransferase, cytosolic [Manduca sexta]	4	429	48.1	7.65	11	4	4	High	1	0.981	1.047	0.552	0.537	0.613	0.726	0.729	0.696	0.675	0.66	0.602	0.65	0.606	0.708
TRINITY_DN103475_c0_g1_i4_orf1	lipid storage droplets surface-binding protein 1 isoform X3 [Ostrinia furnacalis	3	162	18	8.13	23	9	3	High	1	0.943	1.016	1.99	2.097	1.855	1.904	1.858	1.821	1.732	1.704	1.738	1.286	1.124	1.085
TRINITY_DN139335_c0_g2_i1_orf1	unnamed protein product [Homo sapiens]	5	196	22.2	7.9	21	9	1	High	1	1.103	1.105	1.033	1.263	1.053	0.99	1.089	1.233	0.776	0.689	1.032	2.111	1.462	1.357
TRINITY_DN15720_c0_g1_i2_orf1	signal peptidase complex subunit 2 [Ostrinia furnacalis]	4	184	20.7	8.48	21	8	4	High	1	0.943	1.029	1.136	1.172	1.086	0.977	1.049	1.054	0.991	0.974	0.978	1.083	1.06	1.111
TRINITY_DN3332_c0_g1_i9_orf1	glutathione S-transferase sigma3 [Glyphodes pyloalis]	4	200	23.1	6.57	30	14	3	High	1	1.073	1.042	1.001	0.972	1.053	1.096	1.055	1.289	0.762	0.956	0.646	0.577	0.542	0.523
TRINITY_DN2022_c0_g1_i7_orf1	hypothetical protein evm_000891 [Chilo suppressalis]	4	216	24.3	5.43	23	4	4	High	1	1.026	0.944	1.058	1.082	1.035	1.12	1.159	1.203	1.102	1.051	1.158	1.134	1.203	1.16
TRINITY_DN206_c0_g1_i8_orf1	A-kinase anchor protein 200-like [Ostrinia furnacalis]	4	320	34.3	4.5	15	5	1	High	1	1.153	1.024	0.887	0.988	0.896	0.97	1.041	0.932	0.723	0.702	0.838	2.823	3.265	2.663
TRINITY_DN901_c0_g1_i8_orf1	serine protease persephone-like [Ostrinia furnacalis]	6	357	39.4	6.38	22	7	6	High	1	0.992	0.995	0.941	0.935	1.014	1.105	1.066	1.107	1.036	1.031	1.038	1.152	1.104	1.134
TRINITY_DN8783_c0_g1_i9_orf1	luciferin 4-monooxygenase-like [Ostrinia furnacalis]	5	523	58	6.54	12	6	3	High	1	1.093	1.034	0.719	0.744	0.752	1.384	1.282	1.301	1.436	0.854	0.886	0.82	0.845	0.86

TRINITY_DN1494_c0_g1_i3_orf1	dihydropyrimidine dehydrogenase [NADP(+)] [Ostrinia furnacalis]	4	696	75.2	5.63	6	6	4	High	1	1.031	1.021	0.739	0.745	0.793	0.683	0.75	0.721	0.734	0.712	0.708	0.736	0.767	0.766
TRINITY_DN5149_c0_g1_i12_orfp1	TRINITY_DN5149_c0_g1_i12_m.8808 TRINITY_DN5149_c0_g1::TRINITY_DN5149	6	253	30.6	6.57	55	8	2	High	1	1.015	1.179	1.284	1.334	1.125	1.043	1.056	0.977	1.076	0.857	1.065	0.81	0.783	0.856
TRINITY_DN4228_c0_g1_i5_orf1	phenoloxidase-activating enzyme-like [Ostrinia furnacalis]	3	422	47.5	8.05	16	4	3	High	1	1.011	1.047	1.85	1.908	1.644	1.82	2.115	1.972	1.729	1.802	1.914	2.727	2.625	2.744
TRINITY_DN4950_c0_g1_i2_orf1	unnamed protein product [Diatraea saccharalis]	5	1273	141.2	7.2	5	5	5	High	1	1.091	1.02	0.621	0.618	0.707	0.611	0.672	0.636	0.576	0.576	0.532	0.857	0.861	0.865
TRINITY_DN117412_c0_g1_i1_orf1	eukaryotic translation initiation factor 3 subunit F [Ostrinia furnacalis]	3	277	30.3	5.8	13	7	3	High	1	0.974	1.003	1.178	1.125	1.051	1.089	1.11	1.167	1.176	1.15	1.14	1.064	1.058	1.105
TRINITY_DN23946_c0_g1_i1_orf1	ubiquitin-conjugating enzyme E2L [Bombyx mori]	5	154	17.8	8.56	38	6	5	High	1	0.994	1.013	0.75	0.793	0.79	0.815	0.748	0.782	0.784	0.752	0.832	0.972	1.016	0.994
TRINITY_DN65681_c0_g1_i1_orf1	ferritin subunit-like [Ostrinia furnacalis]	2	72	8.1	5.08	31	5	1	High	1	0.89	0.974	0.646	0.643	0.791	2.081	1.737	1.708	2.009	1.664	1.415	5.099	4.851	4.808
TRINITY_DN6621_c0_g1_i1_orf1	translocon-associated protein subunit delta [Ostrinia furnacalis]	4	131	14.3	5.48	36	8	4	High	1	0.979	0.961	1.161	1.143	1.186	1.036	1.045	1.132	1.034	0.927	1.008	0.673	0.738	0.694
TRINITY_DN350_c0_g1_i10_orf1	microtubule-associated protein tau-like isoform X5 [Ostrinia furnacalis]	5	276	29.5	9.76	19	10	2	High	1	1.092	1.053	0.996	1.005	1.11	1.056	1.071	0.996	0.857	0.835	0.941	0.788	0.689	0.757
TRINITY_DN2650_c0_g1_i1_orf1	hypothetical protein HW555_002849 [Spodoptera exigua]	5	284	31.9	7.87	16	6	5	High	1	0.887	0.964	1.906	1.736	1.873	2.681	2.738	2.923	1.937	1.979	2.012	1.974	1.937	2.138
TRINITY_DN61711_c0_g1_i1_orf1	mitochondrial carnitine/acylcarnitine translocase [Loxostege sticticalis]	4	299	32.2	9.25	20	7	4	High	1	0.962	1.031	0.91	0.882	0.848	0.748	0.776	0.806	0.745	0.699	0.743	0.767	0.774	0.767
TRINITY_DN77425_c0_g1_i2_orf1	anosmin-1 [Ostrinia furnacalis]	5	418	47.5	9.98	16	6	5	High	1	1	1.043	1.174	1.044	1.087	1.334	1.227	1.254	1.208	1.141	1.212	5.038	5.061	4.827
TRINITY_DN3203_c0_g2_i1_orf1	unnamed protein product [Parnassius apollo]	5	1203	139.5	8.25	5	5	5	High	1	1.007	1.087	0.949	0.979	1.044	0.93	0.985	0.936	0.882	1.161	0.947	0.884	0.983	1.023
TRINITY_DN7002_c0_g1_i4_orf1	uncharacterized protein LOC114349917 [Ostrinia furnacalis]	5	377	42.3	5.62	15	5	5	High	1	0.997	0.957	0.911	0.967	0.969	0.956	1.046	0.992	0.863	0.936	0.887	0.878	1.015	0.926
TRINITY_DN138481_c0_g1_i2_orf1	cuticle protein 8-like isoform X2 [Vanessa tameamea]	4	233	24.2	8.88	27	9	2	High	1	0.933	1	0.891	0.815	0.791	0.912	0.992	0.957	0.887	1.066	1.082	4.769	4.874	4.696
TRINITY_DN41321_c1_g1_i3_orf1	uncharacterized protein LOC114357706 isoform X1 [Ostrinia furnacalis]	5	612	71.3	6.54	11	5	5	High	1	0.916	1.059	1.096	1.077	0.983	1.082	1.187	1.116	1.027	1.039	1.081	1.348	1.253	1.337
TRINITY_DN28729_c0_g1_i7_orf1	serine/threonine-protein kinase mig-15 isoform X7 [Ostrinia furnacalis]	5	1000	111.1	8.37	7	5	1	High	1	0.943	1.087	1.556	1.259	1.124	1.081	0.734	0.942	1.007	0.959	0.899	1.617	1.482	1.128
TRINITY_DN62_c1_g1_i3_orf1	D-2-hydroxyglutarate dehydrogenase, mitochondrial-like [Ostrinia furnacalis]	4	506	55.8	6.76	10	5	3	High	1	1.045	0.976	1.061	1.121	1.117	1.258	1.37	1.203	1.453	1.297	1.328	0.836	0.736	0.839
TRINITY_DN19411_c0_g1_i1_orf1	myrosinase 1-like [Ostrinia furnacalis]	5	516	59.2	5.07	10	5	4	High	1	1.01	1.015	0.76	0.934	0.721	0.849	0.922	0.906	0.816	1.002	1.092	0.753	0.642	0.838
TRINITY_DN22928_c0_g1_i6_orf1	hypothetical protein B5X24_HaOG209714 [Helicoverpa armigera]	4	393	42.4	4.78	13	5	4	High	1	0.892	1.017	1.289	1.271	1.363	1.285	1.433	1.136	1.088	1.014	1.117	1.362	1.369	1.533
TRINITY_DN32448_c0_g1_i1_orf1	unnamed protein product [Arctia plantaginis]	5	376	42.6	7.02	19	5	5	High	1	1.03	1.07	1.166	1.153	1.167	1.234	1.255	1.352	1.532	1.831	1.509	1.451	1.428	1.518
TRINITY_DN53866_c0_g1_i1_orf1	larval/pupal cuticle protein H1C-like [Ostrinia furnacalis]	2	220	21.8	6.83	22	2	2	High	1	1.138	1.221	1.476	1.716	1.457	1.534	1.408	1.287	1.359	1.106	1.262	3.571	3.625	3.447
TRINITY_DN164_c0_g1_i11_orf1	hypothetical protein evm_000323 [Chilo suppressalis]	5	770	86.9	7.02	8	5	5	High	1	0.987	0.992	0.732	0.77	0.726	0.687	0.739	0.711	0.7	0.735	0.79	0.816	0.786	0.797
TRINITY_DN22956_c0_g1_i1_orf1	lipoamide acyltransferase component of branched-chain alpha-keto acid dehy	6	475	52	8.44	12	6	6	High	1	0.993	1.004	0.667	0.628	0.686	0.797	0.761	0.799	0.738	0.68	0.676	0.497	0.502	0.513
TRINITY_DN146217_c0_g1_i1_orf1	60S acidic ribosomal protein P0 [Bombus bifarius]	4	327	35.4	7.05	14	9	2	High	1	0.946	0.985	1.027	0.983	0.995	0.875	0.855	0.88	0.853	0.831	0.896	0.608	0.614	0.603
TRINITY_DN11670_c0_g1_i1_orf1	teneurin-m isoform X1 [Ostrinia furnacalis]	4	3096	344.5	6.2	2	4	4	High	1	0.989	1.021	1.406	1.344	1.433	1.219	1.344	1.298	1.085	1.047	1.153	2.76	2.728	2.769
TRINITY_DN12064_c0_g2_i1_orf1	ras-related protein Rab-35 [Ostrinia furnacalis]	4	200	23	8.9	25	6	2	High	1	1.039	0.872	0.946	0.901	0.794	0.787	0.705	0.818	0.804	0.894	0.927	0.933	0.959	0.982
TRINITY_DN399_c9_g1_i1_orf1	4-coumarate--CoA ligase 1-like [Ostrinia furnacalis]	6	548	60	8.69	12	6	6	High	1	1.101	1.188	0.795	1.126	0.854	1.125	1.05	1.617	1.201	1.045	1.082	0.978	1.19	0.979
TRINITY_DN32769_c1_g1_i5_orf1	large subunit GTPase 1 homolog [Ostrinia furnacalis]	7	639	72.4	6.99	9	8	7	High	1	1.008	0.983	0.549	0.64	0.647	0.634	0.655	0.609	0.571	0.573	0.556	0.506	0.497	0.522
TRINITY_DN7451_c0_g1_i10_orf1	huntingtin-interacting protein 1 isoform X4 [Pectinophora gossypiella]	7	1065	117.6	5.67	7	7	7	High	1	0.957	0.981	0.848	0.865	0.82	0.779	0.79	0.743	0.728	0.698	0.703	0.889	0.96	0.879
TRINITY_DN27035_c0_g1_i1_orf1	glucose-6-phosphate isomerase-like [Ostrinia furnacalis]	3	122	13.3	9.09	39	8	3	High	1	1.065	1.113	1.976	2.01	1.902	2.88	2.713	2.63	2.988	2.619	3.707	2.674	2.788	2.632
TRINITY_DN2264_c0_g1_i1_orf1	cytochrome P450 6B6-like [Ostrinia furnacalis]	5	514	59.1	8.46	12	6	3	High	1	0.969	1.116	1.599	1.424	1.357	1.733	1.873	2.037	1.865	1.578	1.739	1.795	1.546	1.657
TRINITY_DN11808_c0_g1_i8_orf1	unnamed protein product [Diatraea saccharalis]	5	199	22.3	8.25	28	6	5	High	1	0.969	0.996	0.94	0.895	0.966	1.023	0.968	1.064	1.252	1.175	1.178	0.928	0.954	0.931
TRINITY_DN7341_c0_g1_i8_orf1	LOW QUALITY PROTEIN: proteasome activator complex subunit 4-like [Ostrini	4	1959	219.6	6.52	3	4	4	High	1	0.954	0.982	0.964	0.859	0.885	0.767	0.867	0.852	0.831	0.902	0.808	0.712	0.66	0.727
TRINITY_DN1047_c0_g1_i6_orf1	mitochondrial genome maintenance exonuclease 1-like [Ostrinia furnacalis]	4	398	45.6	9.52	12	7	4	High	1	1.01	0.97	1.671	1.597	1.6	1.556	1.712	1.621	1.641	1.461	1.534	1.265	1.242	1.235
TRINITY_DN14774_c0_g1_i4_orf1	aminopeptidase N-like [Ostrinia furnacalis]	6	933	106.7	6.4	7	6	6	High	1	1.032	1.018	1.362	1.359	1.379	0.905	0.965	1.004	0.871	0.916	1.03	1.98	1.759	1.961
TRINITY_DN8979_c0_g1_i5_orf1	ras-related protein Rab-5B [Vanessa cardui]	5	212	22.8	8.13	27	5	5	High	1	1.047	1	1.338	1.364	1.364	1.355	1.309	1.467	1.179	1.116	1.103	1.487	1.502	1.531
TRINITY_DN21218_c0_g2_i3_orf1	leukotriene A-4 hydrolase isoform X2 [Ostrinia furnacalis]	5	255	29.7	5.59	20	5	5	High	1	0.996	0.988	0.664	0.758	0.708	0.65	0.71	0.661	0.684	0.626	0.77	0.808	0.797	0.836
TRINITY_DN32572_c0_g1_i1_orf1	signal peptidase complex subunit 3 [Ostrinia furnacalis]	5	190	21.7	8.66	26	9	5	High	1	1.023	1.02	1.109	0.857	1.058	0.964	0.881	1.035	0.956	0.866	0.75	0.959	1.046	0.898
TRINITY_DN8019_c0_g1_i4_orf1	deoxyhypusine hydroxylase [Ostrinia furnacalis]	5	305	34.1	4.96	19	5	5	High	1	1.003	0.944	0.423	0.458	0.48	0.405	0.45	0.425	0.405	0.387	0.437	0.464	0.493	0.504
TRINITY_DN8651_c0_g1_i16_orf1	glutathione S-transferase theta 2 [Conogethes punctiferalis]	5	227	26.5	8.94	24	6	2	High	1	0.956	0.907	1.18	1.569	1.395	1.965	1.637	1.817	1.27	1.192	1.454	1.089	1.064	1.084
TRINITY_DN5962_c0_g1_i1_orf1	tRNA (cytosine(34)-C(5))-methyltransferase [Ostrinia furnacalis]	6	733	83.8	7.61	10	6	6	High	1	0.999	0.975	0.541	0.533	0.571	1.33	1.183	1.232	0.611	0.599	0.589	0.482	0.483	0.467
TRINITY_DN4081_c0_g1_i1_orf1	unnamed protein product, partial [Iphiclides podalirius]	2	248	26.9	5	18	5	2	High	1	0.987	0.991	0.816	0.855	0.738	1.027	1.13	0.869	0.944	0.852	1.028	0.824	0.877	0.897
TRINITY_DN17215_c0_g1_i4_orf1	28S ribosomal protein S29, mitochondrial [Ostrinia furnacalis]	4	371	42.8	8.56	11	4	4	High	1	0.931	0.956	0.628	0.583	0.659	0.631	0.561	0.619	0.597	0.704	0.604	0.55	0.577	0.626
TRINITY_DN7655_c0_g1_i3_orf1	hypothetical protein evm_001118 [Chilo suppressalis]	4	521	60.7	5.44	10	5	4	High	1	0.999	0.995	0.66	0.672	0.7	0.667	0.698	0.689	0.647	0.618	0.664	0.761	0.796	0.724
TRINITY_DN1326_c0_g1_i2_orf1	cuticle protein 7 [Plutella xylostella]	4	170	18.5	6.96	24	9	1	High	1	0.919	0.962	0.825	1.037	0.954	0.996	1.495	1.709	0.963	0.873	0.89	3.647	3.979	3.131
TRINITY_DN112120_c0_g1_i1_orf1	juvenile hormone esterase-like [Ostrinia furnacalis]	5	535	60.2	5.25	17	5	5	High	1	0.95	0.984	0.474	0.442	0.545	0.518	0.448	0.552	0.573	0.619	0.496	0.516	0.487	0.486
TRINITY_DN1063_c0_g1_i16_orf1	hypothetical protein evm_000854 [Chilo suppressalis]	5	411	46.6	5.11	14	7	5	High	1	0.977	1.04	1.153	1.206	1.221	1.449	1.392	1.318	1.234	1.232	1.331	1.22	1.267	1.273
TRINITY_DN130778_c0_g1_i1_orf1	26S proteasome non-ATPase regulatory subunit 8 [Ostrinia furnacalis]	4	268	30.1	6.54	19	5	4	High	1	1.033	0.999	0.987	0.929	1.023	0.87	0.826	0.85	0.767	0.698	0.762	0.985	1.004	0.973
TRINITY_DN12690_c0_g1_i1_orf1	ELAV-like protein 1 [Ostrinia furnacalis]	6	376	40.5	9.06	18	6	6	High	1	1.003	1.049	1.09	1.258	1.118	1.128	1.199	1.072	1.04	0.833	0.874	0.653	0.688	0.64
TRINITY_DN25975_c0_g3_i2_orf1	V-type proton ATPase subunit D isoform X2 [Ostrinia furnacalis]	7	246	27.4	9.64	20	9	7	High	1	0.99	1.026	0.299	0.259	0.32	0.325	0.341	0.353	0.324	0.31	0.318	0.376	0.417	0.408
TRINITY_DN4245_c0_g2_i1_orf1	long-chain fatty acid transport protein 4-like [Ostrinia furnacalis]	3	177	19.7	9.91	21	5	2	High	1	0.966	1.003	2.691	2.487	2.55	2.708	2.555	2.883	2.485	2.323	2.317	1.94	1.827	1.867
TRINITY_DN13648_c0_g1_i6_orf1	neurexin-4 [Ostrinia furnacalis]	5	1275	142.6	6.13	4	5	5	High	1	1.033	1.009	1.024	0.98	0.99	0.995	1.08	0.99	0.954	0.824	0.862	2.153	2.056	2.235
TRINITY_DN135077_c0_g1_i1_orf1	hypothetical protein KR038_001662 [Drosophila bunnanda]	5	72	8.5	9.45	64	9	4	High	1	1.092	0.946	0.955	1.039	1.03	1.263	1.348	1.296	1.031	0.891	1.117	0.545	0.642	0.601
TRINITY_DN36199_c0_g1_i1_orf1	fatty acid-binding protein 1-like [Ostrinia furnacalis]	5	133	15.2	5.15	48	7	5	High	1	1.015	0.976	0.04	0.046	0.07	0.039	0.062	0.055	0.059	0.074	0.067	0.057	0.06	0.065
TRINITY_DN1370_c0_g1_i2_orf1	hypothetical protein evm_000756 [Chilo suppressalis]	2	231	23	9.29	35	4	2	High	1	1.06	1.236	4.269	3.988	4.136	4.533	4.23	4.839	5.736	5.899	4.696	8.119	7.905	7.546
TRINITY_DN399_c3_g2_i6_orf1	proline-rich extensin-like protein EPR1 [Ostrinia furnacalis]	3	175	18.8	9.07	33	5	3	High	1	0.94	0.987	1.791	1.832	1.552	2.08	2.111	1.972	2.318	2.109	2.621	2.203	2.148	2.083
TRINITY_DN31001_c0_g1_i1_orf1	endocuticle structural glycoprotein ABD-5-like [Ostrinia furnacalis]	3	111	12.2	4.48	41	4	3	High	1	1.025	0.939	0.881	0.924	0.899	1.078	1.124	1.005	1.018	0.819	1.03	0.332	0.371	0.318
TRINITY_DN662_c0_g1_i1_orf1	PREDICTED: actin-related protein 2 [Amyelois transitella]	4	394	44.8	5.97	13	6	4	High	1	1.043	0.991	0.83	0.831	0.835	0.755	0.79	0.796	0.821	0.805	0.936	0.947	0.976	0.958
TRINITY_DN1383_c0_g1_i2_orf1	uncharacterized protein LOC114353133 isoform X1 [Ostrinia furnacalis]	4	318	34.4	7.71	24	6	4	High	1	1.017	0.95	0.539	0.537	0.565	0.688	0.722	0.627	0.712	0.686	0.844	0.544	0.517	0.522
TRINITY_DN13997_c0_g1_i5_orf1	28 kDa heat- and acid-stable phosphoprotein [Ostrinia furnacalis]	5	173	19.8	9.28	32	6	5	High	1	1.019	1.029	1.455	1.509	1.595	1.534	1.448	1.485	1.764	1.637	1.699	1.683	1.787	1.751
TRINITY_DN139438_c0_g1_i1_orf1	T-complex protein 1 subunit delta [Ostrinia furnacalis]	3	118	12.1	6.35	53	4	3	High	1	1.158	1.158	0.798	0.77	0.834	0.885	0.911	0.798	0.739	0.761	0.832	0.898	0.832	0.834
TRINITY_DN3194_c0_g1_i6_orf1	uncharacterized protein LOC114361386 [Ostrinia furnacalis]	5	964	109.4	5.86	7	6	4	High	1	0.977	1.052	0.161	0.14	0.185	0.208	0.222	0.203	0.181	0.161	0.179	0.201	0.227	0.216
TRINITY_DN3231_c0_g1_i12_orf1	integrin-linked protein kinase [Pectinophora gossypiella]	5	452	50.9	7.99	14	5	5	High	1	1.034	1.007	1.366	1.356	1.33	1.523	1.463	1.48	1.223	1.229	1.259	0.765	0.742	0.755
TRINITY_DN1226_c0_g1_i11_orfp1	TRINITY_DN1226_c0_g1_i11_m.52385 TRINITY_DN1226_c0_g1::TRINITY_DN122	4	91	9.1	4.96	53	11	2	High	1	1.077	1.011	2.261	2.383	2.216	3.063	2.76	2.751	1.914	1.724	1.965	1.087	1.058	1.039
TRINITY_DN10745_c0_g1_i14_orf1	septin-1 [Ostrinia furnacalis]	3	374	42.6	5.78	15	3	3	High	1	1.063	1.178	0.787	0.823	0.773	0.901	0.791	0.858	0.842	0.782	0.818	1.357	1.275	1.334
TRINITY_DN2172_c0_g2_i8_orf1	hypothetical protein evm_003685 [Chilo suppressalis]	4	372	42.4	8.05	11	5	4	High	1	1.021	0.973	0.931	0.87	0.894	0.806	0.838	0.796	0.732	0.725	0.687	1.055	1.07	1.017
TRINITY_DN22018_c0_g1_i3_orf1	hypothetical protein evm_009110 [Chilo suppressalis]	5	809	93.3	8.1	8	6	5	High	1	1.051	1.037	0.899	0.877	0.885	1.063	1.075	1.088	1.141	1.079	1.106	1.257	1.328	1.22
TRINITY_DN19000_c0_g1_i4_orf1	NADH dehydrogenase [ubiquinone] 1 beta subcomplex subunit 9 [Ostrinia furı	6	144	17.6	9.44	43	7	6	High	1	0.956	0.95	0.465	0.518	0.502	0.456	0.467	0.504	0.44	0.435	0.456	0.474	0.462	0.472
TRINITY_DN12193_c0_g1_i6_orf1	carbonyl reductase [NADPH] 1-like [Ostrinia furnacalis]	5	276	30.3	7.18	24	6	1	High	1	1.057	0.785	1.118	1.06	1.264	1.278	1.241	1.081	1.382	1.736	1.493	1.531	1.271	1.474
TRINITY_DN21743_c0_g1_i2_orf1	uncharacterized protein LOC114357426 [Ostrinia furnacalis]	4	125	13.9	5.12	36	5	1	High	1	0.926	1.105	1.008	0.818	0.856	1.203	1.174	1.038	1.21	1.009	1.083	0.893	1.062	0.883
TRINITY_DN3335_c0_g1_i1_orf1	unnamed protein product [Pieris macdunnoughi]	3	136	15.3	9.07	18	9	3	High	1	0.986	1.016	1.084	1.167	1.108	0.736	0.761	0.751	0.63	0.566	0.744	1.073	1.103	1.047
TRINITY_DN23167_c0_g2_i1_orf1	hypothetical protein evm_003712 [Chilo suppressalis]	4	238	25.2	8.68	14	7	4	High	1	1.038	1.086	1.556	1.424	1.362	1.701	1.613	1.545	1.572	1.419	1.791	2.208	2.401	2.253
TRINITY_DN24322_c0_g1_i4_orf1	unnamed protein product, partial [Brenthis ino]	3	232	22.5	11.43	20	4	3	High	1	1.037	0.993	1.281	1.337	1.326	1.027	1.034	1.009	1.142	0.951	1.199	0.518	0.597	0.549
TRINITY_DN43076_c0_g1_i6_orf1	protein argonaute-2 isoform X2 [Pectinophora gossypiella]	5	863	97.5	9.11	7	5	5	High	1	1.011	0.978	0.827	0.783	0.801	0.712	0.723	0.79	0.781	0.692	0.613	0.964	0.945	0.956
TRINITY_DN9732_c0_g1_i7_orf1	CD109 antigen [Ostrinia furnacalis]	7	1833	209	6.86	4	7	7	High	1	0.903	1.001	0.975	0.929	0.989	0.984	0.951	0.978	0.914	0.937	0.928	1.357	1.385	1.412
TRINITY_DN5383_c0_g1_i4_orf1	vacuolar protein sorting-associated protein 35 [Ostrinia furnacalis]	5	501	56.5	5.83	11	6	3	High	1	0.987	1.062	1.744	1.647	1.675	1.646	1.486	1.59	1.525	1.405	1.383	1.591	1.752	1.716
TRINITY_DN8596_c0_g1_i2_orf1	SWI/SNF-related matrix-associated actin-dependent regulator of chromatin su	5	573	62.9	6.79	8	5	5	High	1	0.902	0.89	0.574	0.716	0.653	0.695	0.779	0.784	0.77	0.664	0.748	0.977	1.028	0.866
TRINITY_DN2140_c0_g1_i1_orf1	uncharacterized protein LOC126367764 [Pectinophora gossypiella]	4	762	84	6.84	7	4	4	High	1	1.037	1.073	1.269	1.425	1.169	1.089	1.118	1.144	1.233	1.105	1.14	3.658	4.102	3.92
TRINITY_DN4502_c0_g1_i3_orf1	kynurenine formamidase isoform X1 [Ostrinia furnacalis]	6	352	40.1	6.52	17	7	6	High	1	1.003	1.01	2.071	1.785	1.887	1.889	1.736	1.984	1.759	1.826	1.568	1.354	1.306	1.356
TRINITY_DN1543_c0_g2_i2_orf1	regulation of enolase protein 1-like isoform X3 [Ostrinia furnacalis]	4	180	20.9	5.21	31	4	4	High	1	0.91	0.961	1.209	1.094	1.148	1.82	1.67	1.811	1.239	1.196	0.976	1.193	1.032	1.207
TRINITY_DN1459_c1_g1_i1_orf1	reticulon-1 isoform X2 [Ostrinia furnacalis]	4	139	14.6	4.5	33	4	4	High	1	1.096	1.011	0.69	0.722	0.738	0.621	0.642	0.589	0.692	0.684	0.658	0.839	0.898	0.803
TRINITY_DN11044_c0_g1_i4_orf1	protein Skeletor, isoforms B/C isoform X3 [Ostrinia furnacalis]	5	668	73.8	6.05	11	5	5	High	1	1.104	1.135	1.289	1.303	1.269	1.246	1.201	1.295	1.315	1.396	1.206	1.445	1.345	1.392
TRINITY_DN20323_c0_g1_i1_orf1	cuticle protein 7 [Ostrinia furnacalis]	4	187	20.6	6.15	28	11	3	High	1	1.038	1.032	0.94	0.952	1.003	0.916	0.927	0.902	0.799	0.667	0.863	0.939	0.91	0.888
TRINITY_DN48413_c1_g1_i2_orf1	probable protein phosphatase 2C 11 isoform X1 [Manduca sexta]	6	662	70.5	4.45	13	7	6	High	1	1.037	0.983	0.532	0.499	0.507	0.454	0.45	0.448	0.469	0.455	0.453	0.839	0.816	0.82
TRINITY_DN4159_c1_g1_i1_orf1	F-actin-capping protein subunit beta [Ostrinia furnacalis]	4	275	31	5.55	16	5	4	High	1	1.022	1.117	0.86	0.925	0.883	0.784	0.754	0.787	0.775	0.685	0.844	1.042	0.996	1.026
TRINITY_DN1870_c0_g1_i6_orf1	programmed cell death protein 5 [Ostrinia furnacalis]	3	130	14.8	5.63	24	4	3	High	1	0.961	0.997	1.279	1.447	1.296	1.438	1.416	1.336	1.576	1.275	1.767	1.726	1.774	1.637
TRINITY_DN2793_c0_g2_i1_orf1	PREDICTED: ras-related protein Rab-4B [Amyelois transitella]	4	214	23.5	8.63	24	5	3	High	1	1.007	1.009	1.337	1.336	1.302	1.473	1.298	1.289	1.402	1.882	1.34	1.763	1.576	1.788
TRINITY_DN4183_c0_g1_i8_orf1	histidine--tRNA ligase, cytoplasmic isoform X3 [Ostrinia furnacalis]	5	521	58.6	7.74	9	5	5	High	1	1.05	1.018	0.647	0.678	0.66	0.619	0.66	0.626	0.627	0.597	0.624	0.566	0.609	0.561
TRINITY_DN144956_c0_g1_i1_orf1	40S ribosomal protein S18 [Cotesia glomerata]	4	152	17.6	10.4	22	11	3	High	1	0.993	0.956	0.625	0.664	0.67	0.633	0.631	0.593	0.552	0.437	0.567	0.509	0.567	0.559
TRINITY_DN28729_c0_g1_i9_orf1	serine/threonine-protein kinase mig-15 isoform X2 [Ostrinia furnacalis]	6	845	94.4	8.56	9	6	2	High	1	1.212	1.076	1.309	1.342	1.446	1.449	2.065	1.512	1.704	1.952	1.655	1.756	1.81	1.889
TRINITY_DN2472_c2_g1_i7_orf1	serine/threonine-protein kinase Tao isoform X1 [Vanessa cardui]	7	1007	117.4	8.22	7	7	7	High	1	1.083	1.03	1.082	1.103	1.006	0.909	1.018	0.994	0.96	0.9	1.001	1.062	1.009	1.049
TRINITY_DN33926_c0_g1_i1_orf1	ribosomal protein S9 [Ailuropoda melanoleuca]	5	194	22.6	10.65	21	9	1	High	1	1.012	0.899	0.769	0.496	0.556	0.661	0.5	0.574	0.63	0.534	0.521	0.52	0.417	0.47
TRINITY_DN69707_c0_g1_i1_orf1	titin-like, partial [Ostrinia furnacalis]	3	74	8.4	5.5	49	5	2	High	1	1.078	1.031	1.213	1.201	1.284	1.328	1.269	1.227	1.281	1.118	1.262	0.595	0.671	0.601

TRINITY_DN27833_c0_g2_i1_orf1	uncharacterized protein LOC114359161 [Ostrinia furnacalis]	4	372	41	8.16	13	5	3	High	1	0.872	0.841	1.168	1.224	1.199	1.695	1.591	1.646	1.212	1.14	1.19	1.308	1.229	1.235
TRINITY_DN636_c1_g1_i9_orf1	secretory phospholipase A2 receptor-like [Ostrinia furnacalis]	4	271	31	5.69	18	4	2	High	1	0.931	0.899	4.093	3.337	4.034	2.668	2.588	3.281	6.816	6.119	5.379	5.192	5.546	5.372
TRINITY_DN3472_c1_g1_i4_orf1	Krueppel homolog 2-like [Ostrinia furnacalis]	4	257	28.7	9.58	16	7	4	High	1	1	0.971	0.799	0.747	0.814	0.755	0.829	0.82	0.667	0.721	0.663	0.986	0.97	0.968
TRINITY_DN16122_c0_g1_i4_orf1	cytochrome P450 6k1-like [Ostrinia furnacalis]	5	498	56.9	8.65	12	7	5	High	1	0.938	0.975	0.414	0.417	0.45	0.541	0.549	0.53	0.48	0.59	0.497	0.458	0.44	0.473
TRINITY_DN661_c0_g3_i5_orf1	cuticle protein 18.6-like [Ostrinia furnacalis]	5	212	22.3	6.95	28	5	3	High	1	1.092	1.103	0.999	1.044	1.032	1.233	1.129	1.096	0.929	1.148	0.96	2.079	2.036	1.925
TRINITY_DN21782_c0_g1_i8_orf1	atypical kinase COQ8B, mitochondrial [Ostrinia furnacalis]	6	647	73.1	6.99	11	7	6	High	1	0.906	0.907	0.665	0.655	0.662	0.638	0.689	0.646	0.585	0.709	0.653	0.724	0.655	0.775
TRINITY_DN10644_c0_g1_i2_orf1	carboxylesterase [Cnaphalocrocis medinalis]	8	488	55	5.8	17	10	6	High	1	1.036	1.055	0.416	0.403	0.45	0.435	0.466	0.457	0.426	0.542	0.447	0.629	0.687	0.581
TRINITY_DN4380_c0_g1_i9_orf1	hypothetical protein evm_012370 [Chilo suppressalis]	4	760	83.6	6.24	6	4	4	High	1	1.018	1.043	0.738	0.709	0.691	0.62	0.575	0.652	0.62	0.526	0.616	0.818	0.766	0.699
TRINITY_DN7908_c0_g1_i5_orf1	protein transport protein Sec24A [Helicoverpa zea]	5	1192	129.5	8.02	6	5	5	High	1	0.99	1.008	0.72	0.786	0.77	0.73	0.804	0.77	0.832	0.895	0.883	0.669	0.671	0.742
TRINITY_DN12584_c0_g1_i1_orf1	carnitine O-palmitoyltransferase 1, liver isoform [Ostrinia furnacalis]	5	776	89.4	8.53	8	6	5	High	1	1.007	0.991	0.657	0.637	0.663	0.713	0.712	0.709	0.636	0.629	0.659	0.572	0.597	0.591
TRINITY_DN2438_c0_g1_i4_orf1	dystrophin, isoforms A/C/F/G/H isoform X2 [Manduca sexta]	5	869	97.4	7.71	6	5	2	High	1	1.027	1.075	0.972	0.995	0.856	0.848	0.897	0.837	0.814	0.7	0.754	1.019	0.98	1.079
TRINITY_DN140212_c0_g1_i1_orf1	macrophage mannose receptor 1-like [Ostrinia furnacalis]	3	92	10.3	5.15	59	6	2	High	1	0.944	1.038	1.225	1.23	1.311	2.295	2.238	2.041	1.993	1.535	1.917	2.044	2.013	1.562
TRINITY_DN109503_c0_g1_i4_orf1	uncharacterized protein LOC114366345 isoform X2 [Ostrinia furnacalis]	4	166	18.1	6.49	45	4	1	High	1	1.069	1.147	0.797	0.914	0.875	1.076	1.206	1.089	1.813	1.593	1.929	0.938	0.881	0.834
TRINITY_DN11620_c0_g1_i2_orf1	serine/threonine-protein kinase PAK 3 [Ostrinia furnacalis]	4	497	55.8	5.8	13	4	4	High	1	1.013	1.126	1.008	1.036	1.132	0.976	1.013	1.194	1.24	1.671	1.041	1.309	1.36	1.335
TRINITY_DN1318_c0_g1_i5_orf1	uncharacterized protein LOC114360956 [Ostrinia furnacalis]	5	450	47.9	4.5	21	6	5	High	1	1.012	0.938	0.316	0.308	0.361	0.285	0.31	0.303	0.293	0.304	0.316	0.253	0.252	0.272
TRINITY_DN130069_c0_g6_i1_orf1	hypothetical protein HPG69_006179 [Diceros bicornis minor]	3	216	23.6	5.77	19	5	1	High	1	1.132	1.069	1.242	1.198	1.222	1.317	1.151	1.375	1.07	1.303	1.003	1.554	1.25	1.461
TRINITY_DN2735_c0_g1_i4_orf1	hypothetical protein evm_008546 [Chilo suppressalis]	3	485	52	6.07	9	5	3	High	1	0.96	1.03	1.053	1.083	1.046	1.263	1.183	1.192	1.05	1.258	1.075	0.917	0.895	0.849
TRINITY_DN17172_c0_g1_i5_orf1	omega-amidase NIT2 isoform X1 [Zerene cesonia]	6	299	33.2	7.96	21	9	6	High	1	0.942	0.979	1.403	1.403	1.326	1.494	1.517	1.598	1.314	1.404	1.366	1.204	1.232	1.231
TRINITY_DN21743_c0_g1_i1_orf1	uncharacterized protein LOC114357426 [Ostrinia furnacalis]	4	106	11.7	6.07	36	7	1	High	1	0.977	0.945	1.194	1.111	1.136	1.63	1.479	1.583	2.211	1.937	2.004	1.829	1.995	1.788
TRINITY_DN332_c0_g1_i6_orf1	dolichyl-diphosphooligosaccharide--protein glycosyltransferase subunit STT3I	6	770	86.3	8.98	8	6	6	High	1	0.981	0.979	0.783	0.701	0.772	0.794	0.81	0.849	0.733	0.683	0.636	0.701	0.741	0.728
TRINITY_DN68725_c0_g1_i1_orf1	hydroxymethylglutaryl-CoA synthase 1 [Ostrinia furnacalis]	5	456	50.8	6.55	13	6	5	High	1	1.034	1.089	0.863	0.808	0.879	0.811	0.796	0.836	0.825	0.828	0.776	0.96	0.993	0.947
TRINITY_DN1326_c0_g1_i1_orf1	cuticle protein 7-like [Ostrinia furnacalis]	3	128	13.9	6.4	27	5	1	High	1	1.017	0.987	0.688	0.571	0.515	0.458	0.447	0.509	0.521	0.678	0.779	9.675	9.612	6.994
TRINITY_DN6855_c1_g1_i3_orf1	developmentally-regulated GTP-binding protein 2 [Ostrinia furnacalis]	5	364	40.8	8.73	15	5	5	High	1	0.998	1.004	0.813	0.752	0.789	0.764	0.791	0.843	0.961	0.916	0.683	0.795	0.772	0.814
TRINITY_DN52553_c0_g1_i1_orf1	hemocyte protein-glutamine gamma-glutamyltransferase-like [Ostrinia furnac	4	693	80	6.05	8	4	4	High	1	0.899	0.978	1.019	1.04	1.043	0.986	1.047	1.08	0.922	0.927	0.88	3.703	3.489	3.555
TRINITY_DN13119_c0_g1_i4_orf1	endocuticle structural glycoprotein ABD-5-like [Bicyclus anynana]	3	114	12.3	4.54	49	6	3	High	1	0.97	0.967	1.478	1.487	1.502	1.588	1.58	1.477	1.222	1.088	1.28	0.266	0.289	0.309
TRINITY_DN76633_c0_g1_i1_orfp1	TRINITY_DN76633_c0_g1_i1_m.53394 TRINITY_DN76633_c0_g1::TRINITY_DN76	4	163	19	6.34	31	7	3	High	1	1.028	1.1	1.93	2.061	1.856	1.297	1.551	1.379	2.115	1.782	2.488	0.899	0.925	0.887
TRINITY_DN142485_c0_g1_i1_orf1	uncharacterized protein CG16817-like [Ostrinia furnacalis]	4	172	19.2	4.64	31	5	4	High	1	1.055	0.967	0.748	0.768	0.794	0.608	0.627	0.622	0.593	0.571	0.657	0.702	0.711	0.764
TRINITY_DN14565_c0_g1_i11_orf1	4-aminobutyrate aminotransferase, mitochondrial [Galleria mellonella]	6	467	52.2	7.65	18	7	6	High	1	0.975	1.014	0.441	0.44	0.437	0.521	0.587	0.522	0.476	0.484	0.48	0.592	0.582	0.588
TRINITY_DN5462_c0_g2_i1_orf1	acyl-protein thioesterase 1 [Ostrinia furnacalis]	2	220	23.8	7.05	16	3	2	High	1	1.127	1.1	1.428	1.431	1.329	1.27	0.854	0.984	1.258	1.326	1.37	1.441	1.561	1.243
TRINITY_DN1352_c0_g1_i5_orf1	uncharacterized protein LOC113491815 [Trichoplusia ni]	3	152	16.5	5.48	28	6	3	High	1	0.906	0.944	2.085	2.271	2.128	2.299	2.329	2.052	2.443	2.177	2.881	1.723	1.588	1.69
TRINITY_DN131603_c0_g1_i4_orfp	TRINITY_DN131603_c0_g1_i4_m.86149 TRINITY_DN131603_c0_g1::TRINITY_DN	3	111	12.9	4.91	22	5	1	High	1	0.855	0.955	1.289	1.394	1.416	13.197	13.229	11.052	0.647	0.717	0.685	0.749	0.586	0.36
TRINITY_DN1752_c0_g1_i18_orf1	titin isoform X1 [Ostrinia furnacalis]	6	1789	204	5.43	4	6	6	High	1	1.027	0.992	0.708	0.835	0.786	0.749	0.819	0.772	0.675	0.729	0.757	0.591	0.552	0.561
TRINITY_DN5907_c0_g1_i4_orf1	hypothetical protein evm_010265 [Chilo suppressalis]	5	251	28.1	6.4	23	5	5	High	1	1.028	1.048	1.081	1.16	1.117	1.194	1.204	1.206	1.244	1.215	1.19	3.893	3.869	3.983
TRINITY_DN9059_c0_g1_i1_orf1	sulfotransferase family cytosolic 1B member 1-like [Ostrinia furnacalis]	3	183	20.7	6.58	22	5	1	High	1	0.985	1.005	0.393	0.496	0.591	0.418	0.56	0.485	0.539	0.533	0.475	0.56	0.62	0.581
TRINITY_DN7776_c0_g1_i1_orf1	uncharacterized protein LOC114364702 [Ostrinia furnacalis]	3	154	17.5	5.78	34	6	3	High	1	0.971	1.045	1.28	1.434	1.369	1.253	1.304	1.274	1.454	1.426	1.605	1.566	1.65	1.465
TRINITY_DN2101_c0_g1_i6_orf1	protein obstructor-E-like [Ostrinia furnacalis]	5	248	28.1	6	20	11	5	High	1	1.033	1.008	0.769	0.74	0.746	0.669	0.673	0.767	0.658	0.499	0.56	8.411	8.523	7.546
TRINITY_DN2062_c0_g1_i11_orf1	uncharacterized protein LOC114350846 [Ostrinia furnacalis]	6	147	15.4	6.79	61	7	1	High	1	1.078	1.026	0.55	0.44	0.542	0.589	0.499	0.562	0.454	0.393	0.428	0.564	0.512	0.573
TRINITY_DN2350_c0_g1_i6_orf1	protein yellow-like isoform X2 [Ostrinia furnacalis]	4	485	55	6.93	12	5	4	High	1	1.005	0.961	1.093	1.044	0.998	1.451	1.525	1.456	1.187	1.029	1.166	0.619	0.682	0.639
TRINITY_DN15624_c0_g1_i1_orf1	LOW QUALITY PROTEIN: V-type proton ATPase subunit S1-like [Ostrinia furna	3	402	45.2	4.93	9	4	3	High	1	1.118	1.107	0.237	0.31	0.293	0.261	0.234	0.268	0.318	0.321	0.384	0.467	0.525	0.385
TRINITY_DN8079_c0_g1_i2_orf1	hypothetical protein evm_000040 [Chilo suppressalis]	5	153	18.2	10.58	42	7	5	High	1	0.809	0.877	0.615	0.749	0.592	0.574	0.585	0.599	0.533	1.003	0.664	0.563	0.574	0.676
TRINITY_DN98313_c0_g1_i1_orf1	tetrahydrofolate synthase, partial [Plutella xylostella]	3	81	8.6	4.42	38	8	1	High	1	0.951	0.965	1.22	1.463	1.085	1.358	1.377	1.554	1.716	1.584	1.709	1.772	1.707	1.66
TRINITY_DN3797_c0_g2_i3_orf1	3'(2'),5'-bisphosphate nucleotidase 1 isoform X2 [Ostrinia furnacalis]	5	311	33.3	5.57	16	5	5	High	1	1.042	1.05	1.108	1.09	1.117	0.858	0.875	1.002	0.924	0.855	0.859	0.914	0.945	0.89
TRINITY_DN185_c0_g1_i6_orf1	eukaryotic translation initiation factor 4E-binding protein Mextli isoform X2 [O	5	456	49.9	8.54	11	5	5	High	1	0.981	1.097	0.987	0.985	0.989	0.869	0.908	0.898	0.857	0.778	0.938	1.045	1.164	1.103
TRINITY_DN2304_c0_g1_i4_orf1	clustered mitochondria protein homolog isoform X2 [Ostrinia furnacalis]	4	428	48	8.47	11	4	4	High	1	0.993	0.966	0.328	0.36	0.406	0.422	0.489	0.457	0.387	0.734	0.363	0.385	0.377	0.368
TRINITY_DN12336_c0_g1_i1_orfp1	TRINITY_DN12336_c0_g1_i1_m.30792 TRINITY_DN12336_c0_g1::TRINITY_DN12	4	85	9.3	8.44	51	7	4	High	1	1.05	1.047	2.009	2.161	2.007	2.262	2.187	2.167	1.171	1.244	1.345	0.93	0.824	0.907
TRINITY_DN14498_c0_g1_i1_orf1	eukaryotic translation initiation factor 2 subunit 2 [Ostrinia furnacalis]	5	306	34.8	5.9	18	6	5	High	1	0.997	1.057	0.791	0.773	0.782	0.685	0.64	0.714	0.657	0.584	0.645	0.601	0.604	0.587
TRINITY_DN4977_c0_g1_i2_orf1	manganese-transporting ATPase 13A1 [Ostrinia furnacalis]	6	1165	130.6	7.09	6	6	6	High	1	0.921	0.957	0.47	0.504	0.506	0.459	0.446	0.485	0.437	0.486	0.453	0.545	0.547	0.564
TRINITY_DN2054_c0_g1_i1_orf1	macrophage mannose receptor 1-like [Ostrinia furnacalis]	5	317	35.5	5.81	17	5	5	High	1	1.059	0.979	0.877	0.928	0.935	0.712	0.686	0.737	0.729	0.706	0.806	1.307	1.282	1.249
TRINITY_DN8226_c0_g1_i1_orf1	myosin heavy chain, muscle isoform X16 [Helicoverpa armigera]	3	59	6.3	5.36	56	37	1	High	1	1.013	1.022	1.022	1.105	1.085	1.049	1.103	1.027	0.941	0.856	1.004	0.456	0.452	0.459
TRINITY_DN22589_c0_g1_i6_orfp1	TRINITY_DN22589_c0_g1_i6_m.19386 TRINITY_DN22589_c0_g1::TRINITY_DN22	2	182	17.5	6.51	30	2	1	High	1	1.427	1.09	1.199	1.07	1.051	2.078	1.927	2.319	1.788	1.78	1.942	0.981	1.083	0.934
TRINITY_DN3976_c0_g1_i6_orf1	grpE protein homolog, mitochondrial [Ostrinia furnacalis]	3	223	24.4	7.4	23	3	3	High	1	1.037	1.013	0.56	0.531	0.572	0.552	0.604	0.556	0.563	0.622	0.605	0.48	0.49	0.505
TRINITY_DN13303_c0_g1_i6_orf1	membrane-associated progesterone receptor component 1-like [Ostrinia furn	4	197	21.9	4.78	27	5	4	High	1	0.992	0.972	1.724	1.573	1.734	1.493	1.544	1.531	1.659	1.585	1.437	1.713	1.64	1.71
TRINITY_DN7233_c0_g2_i1_orf1	protein transport protein Sec61 subunit beta [Ostrinia furnacalis]	3	97	10.1	11.8	37	7	3	High	1	1.065	0.96	1.049	0.918	1.103	0.907	0.95	0.981	0.869	0.95	0.835	0.762	0.803	0.792
TRINITY_DN13901_c0_g1_i4_orf1	vesicle-associated membrane protein 7-like [Ostrinia furnacalis]	5	219	24.7	8.81	32	5	5	High	1	0.948	1.126	1.328	1.207	1.237	1.323	1.237	1.335	1.252	1.237	1.242	1.298	1.283	1.41
TRINITY_DN5562_c0_g1_i3_orf1	cell division cycle and apoptosis regulator protein 1-like [Ostrinia furnacalis]	4	171	18	4.96	30	4	4	High	1	1.014	0.93	0.684	0.629	0.664	0.708	0.629	0.684	0.699	0.637	0.654	0.843	0.848	0.802
TRINITY_DN51776_c0_g2_i1_orf1	cuticle protein CP14.6-like [Ostrinia furnacalis]	3	116	12.4	4.35	47	3	3	High	1	0.973	0.904	0.972	0.93	0.962	1.446	1.325	1.177	1.174	0.958	1.244	0.693	0.762	0.792
TRINITY_DN116972_c0_g1_i1_orf1	phosphofructokinase domain-containing protein [Phthorimaea operculella]	4	106	11.7	7.33	46	8	4	High	1	1.017	1.023	1.474	1.512	1.344	1.317	1.373	1.322	1.345	1.271	1.386	1.024	0.929	0.977
TRINITY_DN4403_c0_g1_i3_orf1	AP-1 complex subunit gamma-1 [Ostrinia furnacalis]	5	865	95.7	6.42	6	5	5	High	1	1.01	1.041	0.725	0.676	0.76	0.714	0.698	0.73	0.817	0.732	0.645	0.884	0.95	1.013
TRINITY_DN26488_c0_g1_i6_orf1	phosphatidate phosphatase LPIN2 isoform X1 [Ostrinia furnacalis]	5	983	108.8	5.35	8	5	5	High	1	0.911	0.933	1.192	1.297	1.204	0.998	1.032	0.998	0.923	0.911	1.025	1.112	1.196	1.124
TRINITY_DN15513_c0_g1_i6_orf1	uncharacterized protein LOC114350859 [Ostrinia furnacalis]	4	247	25.8	7.46	22	4	3	High	1	1.021	1.076	0.506	0.521	0.511	0.665	0.705	0.752	0.544	0.573	0.564	0.415	0.371	0.394
TRINITY_DN535_c1_g1_i2_orf1	protein tramtrack, beta isoform isoform X24 [Bicyclus anynana]	4	427	47.4	6.01	18	5	4	High	1	0.946	0.994	0.851	0.882	0.829	0.692	0.718	0.7	0.658	0.611	0.839	1.393	1.293	1.357
TRINITY_DN18569_c0_g2_i1_orf1	stomatin-like protein 2, mitochondrial [Ostrinia furnacalis]	5	408	45.6	6.68	13	5	5	High	1	1.027	1.007	0.601	0.658	0.669	0.601	0.636	0.652	0.564	0.564	0.587	0.74	0.648	0.635
TRINITY_DN13055_c0_g1_i5_orf1	116 kDa U5 small nuclear ribonucleoprotein component isoform X1 [Ostrinia fu	6	706	79.1	6.01	8	7	6	High	1	0.763	1.057	0.496	0.624	0.487	0.445	0.405	0.408	0.368	0.376	0.394	0.606	0.622	0.595
TRINITY_DN46715_c0_g1_i1_orf1	hypothetical protein evm_000885 [Chilo suppressalis]	4	347	39.3	7.72	15	5	4	High	1	1.051	1.145	0.907	0.9	0.916	1.282	1.281	1.148	1.232	1.287	1.216	1.008	0.97	1.021
TRINITY_DN11655_c0_g1_i1_orf1	ribosome biogenesis protein BRX1 homolog [Ostrinia furnacalis]	5	368	42.7	9.8	16	6	5	High	1	0.905	0.961	0.51	0.493	0.537	0.506	0.535	0.527	0.529	0.552	0.539	0.458	0.475	0.486
TRINITY_DN51523_c0_g1_i4_orf1	macrophage migration inhibitory factor homolog [Ostrinia furnacalis]	3	119	12.9	6.77	29	6	3	High	1	1.01	1.083	0.999	0.998	0.973	1.197	1.228	1.202	1.245	1.576	1.152	1.054	1.169	1.057
TRINITY_DN5628_c0_g2_i1_orf1	selenoprotein F [Ostrinia furnacalis]	5	165	18.7	5.59	34	5	5	High	1	0.976	0.983	1.112	1.224	1.174	1.041	1.252	1.119	1.058	1.164	1.071	1.096	1.114	1.122
TRINITY_DN16886_c0_g1_i4_orf1	ER membrane protein complex subunit 1 [Ostrinia furnacalis]	5	915	100.9	6.7	7	6	5	High	1	1.019	0.956	0.711	0.713	0.68	0.664	0.679	0.673	0.651	0.637	0.702	0.655	0.62	0.657
TRINITY_DN8783_c0_g1_i5_orf1	luciferin 4-monooxygenase-like [Ostrinia furnacalis]	3	523	58.1	6.38	8	3	1	High	1	0.809	0.865	2.132	1.943	1.984	1.44	1.421	1.082	1.017	0.836	1.304	1.05	1.317	1.092
TRINITY_DN1407_c0_g1_i5_orf1	unnamed protein product [Chrysodeixis includens]	4	153	17	9.58	26	32	4	High	1	0.974	0.999	1.287	1.353	1.299	1.171	1.247	1.174	1.522	1.354	1.597	2.233	2.351	2.217
TRINITY_DN3263_c0_g1_i2_orf1	serine hydroxymethyltransferase, cytosolic isoform X1 [Diachasma alloeum]	3	523	58.2	8.6	5	5	1	High	1	1.002	0.988	1.835	1.566	1.705	1.812	1.597	1.95	1.816	2.389	1.279	1.147	1.101	1.231
TRINITY_DN85476_c0_g1_i1_orf1	iron-sulfur protein NUBPL-like [Ostrinia furnacalis]	4	319	34.7	8.09	11	6	4	High	1	0.927	0.931	1.442	1.453	1.367	1.259	1.303	1.232	1.009	0.966	1.066	0.905	0.884	0.9
TRINITY_DN34830_c0_g1_i1_orf1	ubiquitin-like domain-containing CTD phosphatase 1 [Ostrinia furnacalis]	3	344	40.1	6.29	13	3	3	High	1	1.022	0.97	1.071	1.13	1.131	0.953	1.012	1.002	0.864	0.93	0.964	1.373	1.437	1.406
TRINITY_DN25582_c0_g1_i3_orf1	uroporphyrinogen decarboxylase [Ostrinia furnacalis]	4	358	40.2	6.55	15	5	4	High	1	0.958	0.949	1.289	1.247	1.274	1.249	1.305	1.287	1.27	1.283	1.248	1.15	1.153	1.249
TRINITY_DN29034_c0_g1_i1_orf1	trypsin-like serine protease [Ostrinia nubilalis]	2	255	27.5	9.7	16	4	2	High	1	1.076	1.068	0.477	0.515	0.495	0.503	0.568	0.666	0.636	0.554	0.56	0.814	0.896	0.758
TRINITY_DN1470_c0_g1_i8_orf1	oxidation resistance protein 1 isoform X5 [Ostrinia furnacalis]	6	576	63.7	5.44	12	6	3	High	1	0.925	0.972	1.753	1.892	1.627	1.516	1.641	1.375	1.44	1.362	1.622	0.965	0.965	0.986
TRINITY_DN661_c1_g2_i1_orf1	larval/pupal cuticle protein H1C-like [Ostrinia furnacalis]	4	237	25.4	7.08	18	5	4	High	1	1.024	1.023	0.779	0.854	0.776	0.791	0.797	0.765	0.643	0.663	0.68	2.408	2.54	2.314
TRINITY_DN64446_c0_g1_i1_orf1	uncharacterized protein LOC114364307 [Ostrinia furnacalis]	3	206	23.7	4.26	15	7	3	High	1	0.99	0.969	0.152	0.133	0.194	0.182	0.193	0.192	0.124	0.141	0.115	0.2	0.198	0.217
TRINITY_DN9794_c0_g2_i8_orf1	acetyl-coenzyme A synthetase [Ostrinia furnacalis]	4	665	74.7	6.38	7	4	4	High	1	1.049	1.033	0.947	1.005	0.976	1.206	1.242	1.102	1.08	1.108	1.186	1.264	1.248	1.317
TRINITY_DN56690_c0_g1_i4_orf1	hypothetical protein evm_002209, partial [Chilo suppressalis]	4	430	48.3	6.95	14	4	4	High	1	0.9	0.972	1.314	1.248	1.206	0.995	0.938	0.992	1.468	1.354	1.379	1.57	1.542	1.496
TRINITY_DN1124_c0_g1_i7_orf1	PREDICTED: cuticle protein 18.6, isoform B [Amyelois transitella]	4	235	27.2	7.62	18	9	4	High	1	1.062	1.011	0.933	0.833	0.875	0.861	0.894	0.868	0.715	0.469	0.466	12.45	13.19	11.979
TRINITY_DN14831_c0_g1_i9_orf1	probable protein phosphatase 2C T23F11.1 isoform X1 [Ostrinia furnacalis]	4	336	37.3	4.89	13	5	4	High	1	1.019	1.023	1.363	1.341	1.288	1.171	1.238	1.334	1.226	1.169	1.314	1.215	1.206	1.22
TRINITY_DN11886_c0_g1_i1_orf1	glycerophosphodiester phosphodiesterase GDPD6-like [Ostrinia furnacalis]	5	369	40.7	5.29	17	6	5	High	1	0.988	1.006	0.758	0.768	0.816	0.621	0.657	0.667	0.694	0.599	0.626	0.655	0.664	0.644
TRINITY_DN69713_c0_g1_i1_orf1	membrane-bound alkaline phosphatase-like [Ostrinia furnacalis]	3	526	57.6	5.74	7	3	3	High	1	0.987	0.958	0.202	0.199	0.217	0.245	0.259	0.292	0.262	0.25	0.219	0.183	0.149	0.159
TRINITY_DN4068_c0_g2_i4_orf1	larval cuticle protein LCP-17-like precursor [Papilio polytes]	3	124	13.4	5.39	56	7	2	High	1	0.988	1.037	1.912	2.147	2.125	3.937	3.914	3.825	3.27	2.708	3.348	0.707	0.695	0.559
TRINITY_DN130075_c1_g2_i1_orf1	60S ribosomal protein L23 [Microtus ochrogaster]	4	140	14.9	10.51	43	10	1	High	1	0.849	0.917	0.361	0.425	0.409	0.386	0.418	0.341	0.428	0.642	0.401	0.36	0.303	0.449
TRINITY_DN7246_c0_g1_i7_orf1	maternal protein tudor-like isoform X2 [Ostrinia furnacalis]	6	984	111.2	7.5	7	6	6	High	1	0.961	0.969	1.003	0.939	0.956	1.011	1.018	1.077	1.005	1.034	1.021	1.178	1.172	1.147
TRINITY_DN20680_c0_g1_i5_orf1	tsukushin isoform X2 [Ostrinia furnacalis]	5	356	40.6	8.19	15	5	5	High	1	1.006	1.025	2.009	1.767	2.015	2.198	2.012	2.128	1.721	1.865	1.637	3.135	3.005	3.122
TRINITY_DN2100_c0_g1_i2_orf1	uncharacterized protein LOC114362418 [Ostrinia furnacalis]	4	260	30.1	7.31	21	5	4	High	1	0.981	0.965	0.886	0.935	0.878	0.856	0.931	0.929	0.967	0.967	0.86	1.21	1.116	1.237
TRINITY_DN9003_c0_g1_i20_orf1	RNA-binding protein Nova-2 isoform X4 [Ostrinia furnacalis]	4	407	42.2	6.68	11	5	4	High	1	0.914	0.991	0.405	0.44	0.41	0.31	0.328	0.301	0.269	0.256	0.29	0.3	0.339	0.35
TRINITY_DN20321_c0_g1_i5_orf1	uncharacterized protein LOC114350467 isoform X3 [Ostrinia furnacalis]	5	420	48.4	5.63	15	5	5	High	1	1.02	1.05	0.54	0.571	0.634	0.716	0.731	0.762	0.802	0.667	0.77	0.587	0.626	0.646
TRINITY_DN40945_c0_g1_i1_orf1	RNA exonuclease 4-like [Ostrinia furnacalis]	4	61	6.6	5.94	67	4	2	High	1	1.002	1.017	1.259	1.324	1.299	1.434	1.591	1.374	1.078	0.965	1.002	1.21	1.274	1.382
TRINITY_DN20215_c0_g2_i1_orf1	unnamed protein product [Spodoptera littoralis]	3	493	54.5	9.5	11	4	3	High	1	1.09	1.051	0.725	0.856	0.801	0.731	0.75	0.736	0.701	0.901	0.881	1.149	1.093	1.087
TRINITY_DN13063_c0_g1_i1_orf1	protein unzipped [Ostrinia furnacalis]	3	454	50.3	4.94	9	5	3	High	1	1.006	0.961	1.01	1.054	1.063	0.804	0.913	0.934	1.087	1	1.081	1.257	1.283	1.211

TRINITY_DN136028_c0_g2_i1_orf1	cytochrome c oxidase subunit 5A, mitochondrial [Ostrinia furnacalis]	6	152	17	5.43	27	7	6	High	1	0.987	0.919	0.513	0.539	0.58	0.56	0.64	0.618	0.573	0.572	0.547	0.674	0.688	0.686
TRINITY_DN63030_c0_g1_i5_orf1	uncharacterized protein LOC114358571 [Ostrinia furnacalis]	3	263	28.8	7.25	20	3	3	High	1	1.05	1.077	1.369	1.392	1.221	1.31	1.378	1.342	1.163	1.29	1.323	0.912	0.904	1.041
TRINITY_DN5943_c0_g1_i6_orf1	N-acetylglucosamine-6-phosphate deacetylase [Ostrinia furnacalis]	4	402	43.2	6.58	13	6	4	High	1	0.973	0.985	1.113	1.076	0.945	1.064	1.042	1.089	1.098	1.118	1.089	1.093	1.142	1.147
TRINITY_DN11230_c0_g1_i4_orf1	peptidyl-prolyl cis-trans isomerase FKBP1A-like [Ostrinia furnacalis]	3	108	11.7	8.15	41	5	3	High	1	1.026	0.959	0.956	0.992	0.994	0.952	0.958	0.93	0.966	0.83	1.051	0.962	1.024	0.93
TRINITY_DN35277_c0_g1_i1_orf1	luciferin 4-monooxygenase-like, partial [Ostrinia furnacalis]	5	551	61.2	7.03	10	5	5	High	1	0.987	1.04	0.489	0.44	0.496	0.561	0.617	0.603	0.561	0.532	0.532	0.525	0.472	0.468
TRINITY_DN1436_c0_g1_i3_orf1	vacuolar protein sorting-associated protein 27-like [Trichoplusia ni]	5	289	32.8	5.86	26	6	1	High	1	0.782	1.419	0.719	1.058	1.11	0.752	0.991	1.101	0.823	0.861	0.835	1.835	1.758	1.6
TRINITY_DN2716_c0_g2_i1_orf1	eukaryotic translation initiation factor 1A, X-chromosomal [Ostrinia furnacalis]	3	148	16.9	5.35	21	4	3	High	1	0.999	0.945	0.621	0.595	0.635	0.522	0.522	0.538	0.44	0.394	0.509	0.603	0.665	0.599
TRINITY_DN6992_c0_g1_i6_orf1	UDP-glucuronosyltransferase 2B15-like [Ostrinia furnacalis]	4	525	58.8	8.69	10	5	4	High	1	0.959	0.973	0.699	0.694	0.67	0.996	0.98	1.002	0.895	0.915	0.854	0.894	0.857	0.926
TRINITY_DN452_c1_g1_i3_orf1	ruvB-like helicase 1 [Colias croceus]	4	456	49.7	7.15	9	5	4	High	1	1.004	1.047	0.696	0.66	0.685	0.696	0.671	0.73	0.67	0.652	0.541	0.743	0.784	0.764
TRINITY_DN556_c0_g2_i1_orf1	serine protease inhibitor dipetalogastin-like [Ostrinia furnacalis]	3	288	30.5	5.07	19	4	1	High	1	0.978	1.114	0.995	0.87	0.91	0.558	0.344	0.272	0.609	0.609	0.68	1.036	0.806	0.818
TRINITY_DN10558_c0_g1_i4_orf1	unnamed protein product [Chrysodeixis includens]	3	178	18.3	6.96	44	4	1	High	1	0.956	0.908	0.872	0.686	0.838	0.487	0.328	0.588	0.371	0.318	0.663	1.135	1.106	0.731
TRINITY_DN20796_c0_g1_i4_orf1	probable low-specificity L-threonine aldolase 2 [Ostrinia furnacalis]	4	374	40.7	7.78	14	4	4	High	1	0.929	0.994	1.108	1.172	1.103	1.256	1.323	1.286	1.193	1.197	1.243	1.686	1.642	1.786
TRINITY_DN4131_c0_g2_i1_orf1	hypothetical protein evm_005631 [Chilo suppressalis]	4	160	17.5	4.84	28	5	4	High	1	1.094	0.999	0.918	0.943	1.089	0.939	0.947	0.935	0.995	0.838	0.89	0.918	0.946	0.826
TRINITY_DN542_c0_g1_i4_orf1	uncharacterized protein LOC114364889 [Ostrinia furnacalis]	5	392	44.8	5.67	14	6	5	High	1	0.925	0.973	0.323	0.301	0.373	0.324	0.329	0.327	0.331	0.335	0.333	0.265	0.269	0.269
TRINITY_DN6144_c0_g1_i2_orf1	tumor suppressor candidate 3 [Ostrinia furnacalis]	6	331	37.5	9.69	15	9	6	High	1	0.908	0.915	0.915	1.037	0.902	0.903	0.93	0.945	0.88	0.977	0.963	0.868	0.864	0.928
TRINITY_DN15737_c0_g1_i7_orf1	UPF0160 protein C27H6.8 [Ostrinia furnacalis]	4	363	42	6.34	14	4	4	High	1	0.996	1.024	0.765	0.658	0.794	0.689	0.605	0.677	0.516	0.506	0.465	0.552	0.57	0.543
TRINITY_DN11383_c0_g2_i4_orf1	aminoacylase-1A-like [Ostrinia furnacalis]	5	401	45	5.96	14	5	5	High	1	1.003	1.005	1.545	1.585	1.663	1.907	2.016	1.83	1.84	1.95	1.751	1.21	1.289	1.367
TRINITY_DN428_c0_g1_i8_orf1	phenoloxidase-activating factor 2-like isoform X1 [Ostrinia furnacalis]	5	434	46.8	7.97	13	10	4	High	1	0.984	1.064	1.843	1.915	1.862	1.84	1.928	1.821	2.128	1.963	2.229	2.053	1.954	2.02
TRINITY_DN6994_c0_g1_i4_orf1	C-type mannose receptor 2-like isoform X1 [Ostrinia furnacalis]	5	328	37.3	5.36	18	5	4	High	1	0.987	1.004	0.65	0.675	0.645	0.914	0.834	0.803	0.672	0.633	0.69	0.617	0.588	0.667
TRINITY_DN10680_c0_g1_i5_orf1	cGMP-dependent protein kinase, isozyme 2 forms cD4/T1/T3A/T3B-like isofor	5	744	84.5	5.63	7	5	5	High	1	1.012	1.043	1.274	1.216	1.321	1.262	1.262	1.335	1.376	1.389	1.554	1.675	1.717	1.618
TRINITY_DN14298_c0_g1_i3_orf1	kinesin heavy chain [Galleria mellonella]	5	184	21	5.71	25	7	1	High	1	1.021	1.092	1	0.944	0.778	1.126	1.047	1.088	1.072	1	1.12	1.288	1.324	1.096
TRINITY_DN578_c0_g1_i3_orf1	charged multivesicular body protein 7 [Ostrinia furnacalis]	3	622	69.9	5.63	7	3	2	High	1	1.089	1.007	1.132	1.236	1.216	1.134	1.087	1.244	0.794	0.844	0.895	1.323	1.269	1.052
TRINITY_DN10415_c0_g1_i5_orf1	hypothetical protein evm_000184 [Chilo suppressalis]	5	746	80.1	6.81	10	6	5	High	1	0.887	1.061	0.85	0.897	0.903	0.971	1.059	1.055	0.795	0.73	0.801	1.125	1.088	0.998
TRINITY_DN22842_c0_g1_i4_orf1	MICOS complex subunit MIC27-like [Ostrinia furnacalis]	5	246	27.1	8.03	20	6	5	High	1	0.993	0.997	0.601	0.608	0.554	0.564	0.55	0.544	0.568	0.594	0.537	0.547	0.602	0.624
TRINITY_DN6239_c0_g1_i1_orf1	eukaryotic translation initiation factor 6 [Ostrinia furnacalis]	4	245	26.4	4.77	24	5	4	High	1	0.986	0.931	0.685	0.686	0.722	0.723	0.674	0.676	0.79	0.706	0.783	0.999	0.995	0.967
TRINITY_DN17133_c0_g1_i1_orf1	unnamed protein product [Chrysodeixis includens]	4	279	29.4	8.44	23	5	4	High	1	0.994	0.937	1.178	1.14	1.092	1.082	1.061	1.127	1.318	1.137	1.153	1.299	1.3	1.389
TRINITY_DN11616_c0_g1_i3_orf1	coiled-coil domain-containing protein 6-like [Ostrinia furnacalis]	5	416	47.2	7.15	14	6	5	High	1	1.065	1.019	0.644	0.693	0.671	0.693	0.793	0.677	0.634	0.639	0.676	0.842	0.89	0.821
TRINITY_DN295_c3_g1_i1_orfp1	TRINITY_DN295_c3_g1_i1_m.18839 TRINITY_DN295_c3_g1::TRINITY_DN295_c3	3	117	12.6	7.12	49	6	3	High	1	0.975	0.908	10.965	10.796	11.508	12.279	12.471	11.14	13.568	11.097	15.86	5.686	5.496	5.398
TRINITY_DN9647_c0_g1_i1_orf1	cytochrome P450 6B2-like [Ostrinia furnacalis]	5	515	59.3	8.6	11	5	5	High	1	0.92	0.969	0.498	0.489	0.506	0.541	0.492	0.502	0.462	0.474	0.479	0.407	0.371	0.411
TRINITY_DN114344_c0_g1_i4_orf1	microtubule-actin cross-linking factor 1 isoform X15 [Ostrinia furnacalis]	4	125	14.2	5.68	30	4	4	High	1	0.974	1.006	1.126	1.166	1.1	1.031	0.936	0.966	0.889	0.851	0.89	0.65	0.612	0.654
TRINITY_DN52395_c0_g2_i2_orf1	twitchin isoform X20 [Zerene cesonia]	5	90	10.1	8.12	73	6	1	High	1	1.042	1.142	1.663	1.701	1.777	1.178	1.175	1.083	1.292	1.052	1.09	0.414	0.579	0.505
TRINITY_DN13598_c1_g1_i1_orf1	transport and Golgi organization protein 1-like [Ostrinia furnacalis]	4	1074	117.9	4.46	5	4	4	High	1	1.058	1.07	0.927	0.962	0.946	0.881	0.912	0.888	0.811	0.828	0.852	0.816	0.841	0.843
TRINITY_DN72056_c0_g1_i1_orf1	protein PBDC1 [Ostrinia furnacalis]	5	163	19.1	5.44	39	6	5	High	1	0.995	0.973	0.676	0.625	0.7	0.625	0.633	0.632	0.645	0.59	0.556	0.709	0.705	0.633
TRINITY_DN120_c0_g1_i2_orf1	PREDICTED: myosin light chain alkali-like [Amyelois transitella]	3	86	9.8	4.34	41	4	1	High	1	1.081	0.977	1.32	1.433	1.418	1.321	1.336	1.432	1.142	1.369	1.216	0.693	0.708	0.698
TRINITY_DN661_c0_g1_i1_orf1	hypothetical protein evm_002822 [Chilo suppressalis]	3	160	17.6	6.76	22	4	1	High	1	1.121	0.999	1.729	1.673	1.606	1.362	1.264	1.28	1.028	0.855	1.131	6.613	6.396	5.217
TRINITY_DN10110_c1_g2_i1_orf1	venom allergen 3-like [Ostrinia furnacalis]	4	319	35.6	7.06	18	4	4	High	1	0.982	1.021	1.088	1.02	1.059	0.809	0.82	0.847	0.693	0.68	0.732	1.071	1.122	1.03
TRINITY_DN47219_c0_g1_i3_orf1	protein windbeutel [Ostrinia furnacalis]	5	252	28.6	8.35	21	6	5	High	1	1.045	1.056	0.881	0.902	0.86	0.87	0.889	0.913	1.005	0.957	0.965	1.312	1.371	1.273
TRINITY_DN14501_c0_g1_i1_orf1	28S ribosomal protein S28, mitochondrial [Ostrinia furnacalis]	3	188	20.8	9.29	22	3	3	High	1	0.993	0.977	0.54	0.521	0.617	0.555	0.546	0.597	0.662	0.575	0.613	0.747	0.764	0.754
TRINITY_DN1493_c0_g1_i5_orf1	uncharacterized protein LOC114350869 [Ostrinia furnacalis]	5	186	20.6	6.39	30	6	4	High	1	1.073	1.083	1.042	1.074	1.124	1.893	1.99	1.658	1.523	1.193	1.535	1.302	1.383	1.191
TRINITY_DN64141_c0_g1_i4_orf1	probable salivary secreted peptide [Ostrinia furnacalis]	3	118	13.3	9.35	33	4	3	High	1	1.056	1.002	1.562	1.987	1.571	2.068	2.251	1.978	2.078	3.623	2.632	2.723	2.443	3.143
TRINITY_DN7785_c0_g1_i1_orf1	uncharacterized protein LOC114364098 [Ostrinia furnacalis]	2	264	29.9	8.57	16	2	2	High	1	0.968	0.982	0.693	0.763	0.727	0.683	0.581	0.649	0.484	0.816	0.535	3.602	3.897	3.72
TRINITY_DN2340_c0_g1_i4_orf1	unnamed protein product [Diatraea saccharalis]	5	371	42.8	5.68	16	5	5	High	1	1.01	0.991	0.895	0.891	0.949	1.001	0.901	0.841	0.93	0.93	0.82	0.884	0.902	0.914
TRINITY_DN2207_c0_g1_i6_orf1	methionine-R-sulfoxide reductase B1 isoform X2 [Ostrinia furnacalis]	3	166	19	8.88	28	4	2	High	1	0.869	0.912	1.235	1.213	1.149	1.145	1.146	1.22	1.404	1.255	1.372	0.966	1.015	0.928
TRINITY_DN2542_c0_g2_i1_orf1	peroxiredoxin-2-like [Ostrinia furnacalis]	2	227	25.1	7.85	13	7	2	High	1	1.039	0.979	0.63	0.667	0.605	0.733	0.785	0.802	0.834	0.737	0.847	0.925	0.888	0.866
TRINITY_DN11050_c0_g1_i8_orf1	uncharacterized protein LOC114360965, partial [Ostrinia furnacalis]	5	957	106.3	8.6	7	5	5	High	1	0.993	0.996	0.784	0.797	0.788	0.816	0.901	0.873	0.764	0.762	0.759	0.676	0.616	0.655
TRINITY_DN2848_c0_g1_i1_orf1	glyceraldehyde-3-phosphate dehydrogenase isoform 2 [Mus musculus]	2	333	35.8	8.25	12	3	1	High	1	0.918	1.019	1.284	1.29	1.573	1.466	1.35	1.703	5.856	1.145	1.507	1.22	1.045	1.055
TRINITY_DN24707_c0_g1_i2_orf1	tether containing UBX domain for GLUT4 [Ostrinia furnacalis]	5	507	58	6.06	12	5	5	High	1	0.957	0.969	1.072	1.075	1.06	1.099	1.195	1.187	1.143	1.266	1.087	1.033	1.12	1.111
TRINITY_DN14935_c0_g1_i1_orf1	kynurenine/alpha-aminoadipate aminotransferase, mitochondrial [Ostrinia furi	4	482	54.4	7.74	12	5	4	High	1	0.885	0.939	0.677	0.671	0.73	0.878	0.952	0.944	0.732	0.8	0.761	0.653	0.638	0.685
TRINITY_DN3015_c0_g1_i7_orf1	glycine-rich protein DOT1-like [Ostrinia furnacalis]	4	298	27.9	9.22	16	5	4	High	1	0.89	0.919	1.761	1.827	1.761	1.751	1.639	1.7	1.736	1.605	1.656	1.424	1.322	1.402
TRINITY_DN2277_c0_g1_i11_orf1	solute carrier family 12 member 4 isoform X1 [Ostrinia furnacalis]	5	1085	119.4	6.89	6	5	5	High	1	1	0.903	1.218	0.941	0.976	0.983	0.934	0.941	0.918	1.025	1.101	1.206	1.319	1.369
TRINITY_DN1305_c0_g1_i6_orf1	glutathione S-transferase sigma 3 [Ostrinia furnacalis]	3	168	19.4	5.66	26	5	2	High	1	0.915	0.976	1.06	1.004	1.019	1.999	2.031	2.172	2.245	2.204	1.801	1.031	1.043	1.009
TRINITY_DN482_c0_g1_i1_orf1	fatty acyl reductase 7 [Maruca vitrata]	4	510	57.9	8.47	8	7	4	High	1	0.936	1.055	0.947	0.89	0.874	0.868	0.886	0.917	0.951	0.895	0.832	1.202	1.179	1.146
TRINITY_DN24476_c0_g1_i1_orf1	ensconsin-like isoform X1 [Ostrinia furnacalis]	5	825	92	8.76	6	5	4	High	1	1.027	0.958	0.318	0.268	0.362	0.241	0.244	0.216	0.278	0.252	0.251	0.375	0.411	0.374
TRINITY_DN6696_c0_g1_i4_orf1	mitochondrial 2-oxodicarboxylate carrier [Ostrinia furnacalis]	5	307	33.9	9.67	16	5	5	High	1	0.975	0.911	0.836	0.689	0.859	0.683	0.68	0.763	0.807	0.739	0.669	0.812	0.808	0.766
TRINITY_DN20878_c0_g4_i2_orf1	GTP 3',8-cyclase, mitochondrial isoform X1 [Ostrinia furnacalis]	3	365	38.1	10.81	13	3	3	High	1	1.029	1.036	0.943	0.977	0.954	0.801	0.742	0.885	0.874	0.93	0.867	0.884	0.913	0.835
TRINITY_DN1497_c0_g2_i6_orf1	unnamed protein product [Chilo suppressalis]	3	609	66	8.28	7	3	3	High	1	0.912	0.939	1.609	1.802	1.52	1.242	1.291	1.3	1.375	1.377	1.395	1.414	1.395	1.48
TRINITY_DN1507_c0_g1_i5_orf1	27 kDa hemolymph protein-like, partial [Ostrinia furnacalis]	6	288	31.9	4.86	23	7	6	High	1	1.015	0.984	1.731	1.81	1.767	1.521	1.577	1.543	1.536	1.401	1.533	2.126	2.294	1.944
TRINITY_DN3521_c0_g2_i1_orf1	barrier-to-autointegration factor [Ostrinia furnacalis]	4	96	10.9	7.75	52	6	4	High	1	1.038	0.899	0.876	0.913	0.973	1.082	1.076	1.021	0.933	0.799	0.941	1.208	1.254	1.192
TRINITY_DN21943_c1_g1_i1_orf1	myosin light chain alkali isoform X2 [Ostrinia furnacalis]	4	119	13.2	4.83	34	13	2	High	1	1.018	0.975	0.818	0.838	0.864	0.83	0.888	0.889	0.876	0.838	0.862	0.622	0.637	0.607
TRINITY_DN2714_c0_g1_i3_orf1	monocarboxylate transporter 9-like [Ostrinia furnacalis]	4	551	60.9	8.97	12	4	4	High	1	1.078	0.944	1.142	0.982	1.008	1.117	1.086	1.168	1.213	1.273	0.949	1.025	1.061	1.048
TRINITY_DN2624_c0_g1_i6_orf1	unnamed protein product [Danaus chrysippus]	2	94	10.2	5.11	35	4	2	High	1	0.99	1.072	1.72	1.654	1.576	1.65	1.599	1.568	1.481	1.531	1.398	1.297	1.406	1.346
TRINITY_DN42856_c0_g1_i1_orf1	GSCOCG00007769001-RA-CDS [Cotesia congregata]	2	989	110.7	7.4	4	3	1	High	1	0.873	0.991	1.923	2.192	1.939	1.764	1.993	1.731	1.505	1.489	1.823	1.709	1.848	1.807
TRINITY_DN6588_c0_g1_i4_orf1	uncharacterized protein LOC114350845 [Ostrinia furnacalis]	4	251	26.1	8.56	22	4	3	High	1	0.922	2.496	0.915	0.696	0.904	1.099	1.117	1.037	0.735	0.738	0.927	1.041	1.072	1.028
TRINITY_DN4012_c0_g4_i2_orf1	uncharacterized protein LOC114362418 [Ostrinia furnacalis]	3	228	25.2	6.57	16	5	3	High	1	1.035	0.959	0.719	0.681	0.727	0.802	0.846	0.778	0.707	0.886	0.745	0.96	0.925	0.906
TRINITY_DN2478_c0_g1_i1_orf1	apoptosis inhibitor 5 [Ostrinia furnacalis]	5	566	63.8	6.58	10	5	5	High	1	0.993	1.014	0.776	0.785	0.878	0.793	0.817	0.88	0.793	0.779	0.772	1.031	0.88	0.83
TRINITY_DN28638_c0_g1_i1_orf1	uncharacterized protein LOC114364075 [Ostrinia furnacalis]	7	305	34.8	6.96	25	9	7	High	1	0.996	0.97	0.396	0.413	0.466	0.489	0.525	0.483	0.411	0.456	0.426	0.362	0.35	0.332
TRINITY_DN2472_c0_g1_i6_orf1	programmed cell death protein 6 isoform X1 [Colias croceus]	3	179	21	5.24	22	4	3	High	1	0.936	0.961	2.138	2.143	2.18	2.143	2.122	2.087	2.84	2.618	2.753	1.968	2.032	2.067
TRINITY_DN8143_c0_g1_i6_orf1	gamma-soluble NSF attachment protein-like [Galleria mellonella]	3	336	36.6	5.07	17	3	3	High	1	0.935	1.012	1.203	1.161	1.203	1.1	1.104	1.159	1.125	1.198	1.258	1.325	1.229	1.308
TRINITY_DN6545_c0_g1_i6_orf1	organic cation transporter protein-like [Ostrinia furnacalis]	4	507	55.7	7.03	10	5	4	High	1	0.978	1.014	0.725	0.714	0.696	0.615	0.622	0.694	0.781	0.795	0.781	0.816	0.81	0.789
TRINITY_DN46022_c0_g1_i1_orf1	mRNA-decapping enzyme 1A [Ostrinia furnacalis]	4	429	47.2	6.89	13	6	4	High	1	1.064	1.005	0.683	0.708	0.691	0.777	0.731	0.644	0.692	0.627	0.679	0.856	0.897	0.799
TRINITY_DN21150_c0_g1_i4_orf1	RNA-binding protein cabeza-like isoform X2 [Bicyclus anynana]	3	127	13.2	6.07	31	5	3	High	1	1.006	0.988	0.51	0.519	0.563	0.469	0.458	0.46	0.42	0.367	0.414	0.616	0.644	0.599
TRINITY_DN97042_c0_g1_i6_orf1	apolipophorins-like [Ostrinia furnacalis]	2	120	12.7	5.2	29	8	2	High	1	0.981	1.007	1.628	1.517	1.558	1.651	1.822	1.795	2.728	2.85	2.733	3.539	3.427	3.428
TRINITY_DN5312_c4_g1_i2_orf1	unnamed protein product [Chilo suppressalis]	5	707	80.4	6.6	9	6	5	High	1	0.944	1.018	0.772	0.795	0.777	0.845	0.818	0.776	0.781	0.869	0.815	0.81	0.78	0.806
TRINITY_DN7580_c0_g1_i1_orf1	cytochrome P450 monooxygenase CYP6AB141 [Ostrinia furnacalis]	6	449	52.1	8.68	14	7	4	High	1	1.006	0.946	0.839	0.844	0.919	0.982	1.048	1.03	0.643	0.593	0.599	0.407	0.432	0.455
TRINITY_DN11121_c0_g1_i5_orf1	unnamed protein product [Chilo suppressalis]	5	1648	177.9	6.61	4	5	5	High	1	0.992	1.047	0.781	0.74	0.729	0.653	0.733	0.716	0.608	0.644	0.695	0.634	0.628	0.66
TRINITY_DN2343_c1_g1_i2_orf1	receptor expression-enhancing protein 5-like isoform X1 [Ostrinia furnacalis]	6	194	22.8	8.53	26	6	2	High	1	0.923	1.003	2.397	2.431	2.379	2.505	2.496	2.237	0.853	0.871	1.029	1.761	1.637	1.701
TRINITY_DN21872_c0_g1_i2_orf1	facilitated trehalose transporter Tret1-2 homolog [Ostrinia furnacalis]	5	580	63.7	6.09	9	5	5	High	1	0.991	1.009	0.831	0.877	0.806	0.748	0.72	0.681	0.806	0.68	0.823	0.572	0.618	0.587
TRINITY_DN1785_c0_g1_i5_orf1	beta-mannosidase [Ostrinia furnacalis]	5	884	101.9	6.95	7	6	5	High	1	0.988	1.009	0.848	0.786	0.841	0.969	0.934	0.964	1.314	1.227	1.25	0.934	0.876	0.836
TRINITY_DN2638_c0_g1_i7_orf1	structural maintenance of chromosomes protein 1A [Trichoplusia ni]	5	1222	140.3	7.33	5	6	5	High	1	1.078	1.034	0.959	0.947	0.936	0.884	0.855	0.84	0.812	0.827	0.911	1.213	1.21	1.18
TRINITY_DN97589_c0_g1_i3_orf1	ribosomal protein L37A [Bombyx mori]	5	107	12	10.05	31	8	5	High	1	0.975	1.035	0.701	0.696	0.693	0.679	0.637	0.647	0.538	0.455	0.524	0.464	0.471	0.463
TRINITY_DN3223_c0_g1_i4_orf1	ras-related protein Rac1 isoform X1 [Plutella xylostella]	2	192	21.4	8.15	13	4	1	High	1	0.851	0.949	1.081	1.083	1.021	0.988	0.955	0.914	0.992	1.031	0.93	1.123	1.21	1.09
TRINITY_DN2035_c0_g1_i1_orf1	uncharacterized protein LOC114356683 [Ostrinia furnacalis]	4	109	12.2	7.88	64	5	4	High	1	0.921	1.033	1.752	1.861	1.703	1.533	1.731	1.601	1.623	1.62	1.836	1.795	1.788	1.96
TRINITY_DN30713_c0_g1_i3_orf1	phosphoglucomutase [Ostrinia furnacalis]	1	86	9.6	5.41	21	6	1	High	1	1.045	1.09	1.083	1.089	1.083	1.077	1.179	1.126	1.599	1.746	1.468	0.918	0.831	0.894
TRINITY_DN124171_c0_g1_i4_orf1	dystonin isoform X27 [Trichoplusia ni]	4	111	13.1	8.97	41	4	4	High	1	0.986	0.981	1.344	1.334	1.297	1.208	1.216	1.317	1.24	1.172	1.157	0.943	1.037	0.866
TRINITY_DN13018_c0_g1_i1_orf1	uncharacterized protein LOC114354768 isoform X2 [Ostrinia furnacalis]	2	290	32.7	7.3	14	3	1	High	1	1.012	0.974	0.7	0.66	0.639	0.751	0.66	0.85	0.87	0.677	0.861	0.866	0.901	0.871
TRINITY_DN21984_c0_g1_i6_orf1	venom serine protease 34-like [Ostrinia furnacalis]	4	412	44.3	6.8	18	4	3	High	1	1.017	0.976	0.489	0.404	0.493	0.576	0.582	0.578	0.599	0.655	0.532	0.524	0.547	0.526
TRINITY_DN22422_c0_g1_i3_orf1	2-hydroxyacylsphingosine 1-beta-galactosyltransferase-like [Ostrinia furnacali	5	378	43.1	6.54	13	5	1	High	1	0.928	0.95	0.951	0.879	1.053	1.121	0.944	1.119	1.013	1.104	0.909	1.061	0.895	0.956
TRINITY_DN745_c5_g1_i2_orf1	facilitated trehalose transporter Tret1-like isoform X2 [Ostrinia furnacalis]	4	509	55.5	9.13	9	4	4	High	1	1.158	1.102	1.476	1.461	1.464	1.377	1.441	1.535	1.475	1.328	1.367	1.299	1.147	1.228
TRINITY_DN26040_c0_g1_i11_orf1	ran-binding protein 9 isoform X9 [Helicoverpa zea]	5	524	57	7.05	11	5	5	High	1	0.984	0.975	0.944	1.007	0.905	0.985	1.033	0.985	0.901	0.959	0.927	0.979	1.027	0.872
TRINITY_DN24689_c0_g1_i1_orf1	thioredoxin domain-containing protein 17-like [Ostrinia furnacalis]	4	126	14.5	5.01	36	5	4	High	1	1.054	0.965	0.834	0.782	0.84	1.156	1.073	1.155	1.259	1.261	1.205	1.236	1.273	1.225
TRINITY_DN21411_c0_g1_i1_orf1	hypothetical protein evm_012759 [Chilo suppressalis]	3	190	21.6	7.83	18	3	3	High	1	1.066	0.941	0.996	1.021	0.9	1.1	1.264	1.117	1.171	1.071	1.19	1.088	1.179	1.134
TRINITY_DN59429_c0_g1_i6_orf1	uncharacterized protein LOC114366345 isoform X2 [Ostrinia furnacalis]	2	107	12.3	8.24	22	6	2	High	1	1.074	1.002	5.774	5.269	5.355	3.533	3.322	3.777	4.759	4.875	4.861	1.763	1.484	1.698
TRINITY_DN29579_c0_g1_i1_orf1	heterogeneous nuclear ribonucleoprotein A1, A2/B1 homolog [Ostrinia furnaca	2	333	35.7	8.66	15	6	2	High	1	0.978	0.921	1.031	1.06	1.103	0.929	0.952	0.955	0.93	0.958	0.933	1.068	1.146	1.215
TRINITY_DN37900_c0_g1_i1_orf1	replication protein A 70 kDa DNA-binding subunit [Ostrinia furnacalis]	6	606	67	7.05	12	6	6	High	1	1.004	1.024	0.822	0.864	0.892	0.869	0.935	0.861	0.954	1.013	0.901	0.928	0.982	0.951

TRINITY_DN9325_c0_g1_i1_orf1	protein takeout-like [Ostrinia furnacalis]	4	236	26	5.68	25	7	4	High	1	1.069	0.998	1.832	1.634	1.778	3.177	2.581	2.963	3.001	2.534	2.407	1.947	1.979	1.859
TRINITY_DN1436_c0_g1_i5_orf1	vacuolar protein sorting-associated protein 27-like [Trichoplusia ni]	5	288	32.6	5.86	26	6	1	High	1	0.778	0.923	0.827	1.118	0.81	0.693	0.745	0.644	0.825	1.03	0.687	1.027	1.092	1.194
TRINITY_DN18291_c0_g1_i1_orf1	hydroxyacylglutathione hydrolase, mitochondrial isoform X1 [Ostrinia furnacali	5	300	33.8	7.36	18	7	5	High	1	0.996	0.998	1.193	1.2	1.207	1.246	1.351	1.163	1.184	1.094	1.364	1.209	1.181	1.205
TRINITY_DN11117_c0_g1_i1_orf1	venom carboxylesterase-6-like [Ostrinia furnacalis]	5	568	63.8	5.4	11	7	4	High	1	1.03	0.986	0.419	0.412	0.448	0.494	0.496	0.49	0.451	0.514	0.448	0.412	0.428	0.387
TRINITY_DN23444_c0_g1_i10_orf1	serrate RNA effector molecule homolog isoform X2 [Ostrinia furnacalis]	6	538	61.1	6.47	10	6	1	High	1	0.968	1.044	0.748	0.802	0.607	0.603	0.684	0.665	0.583	0.574	0.694	0.72	0.734	0.795
TRINITY_DN18242_c0_g1_i3_orf1	CCHC-type zinc finger protein CG3800 [Papilio xuthus]	4	164	18.3	8.22	27	7	4	High	1	0.988	1.066	0.697	0.687	0.631	0.49	0.51	0.528	0.488	0.42	0.554	0.675	0.655	0.707
TRINITY_DN1656_c2_g1_i5_orf1	15-hydroxyprostaglandin dehydrogenase [NAD(+)]-like [Ostrinia furnacalis]	3	275	29.5	5.34	17	5	3	High	1	0.971	0.98	0.482	0.448	0.533	0.811	0.758	0.782	0.509	0.526	0.477	0.353	0.375	0.369
TRINITY_DN2816_c1_g1_i3_orf1	nuclear pore membrane glycoprotein 210 [Ostrinia furnacalis]	6	1709	184.4	6.34	4	6	6	High	1	1.009	1.055	1.033	1.133	1.128	1.11	1.209	1.144	1.021	1.086	1.086	1.051	0.986	1.063
TRINITY_DN135188_c0_g1_i2_orf1	proteasome inhibitor PI31 subunit [Ostrinia furnacalis]	5	280	31.1	5.35	23	8	5	High	1	0.915	1.021	1.497	1.593	1.553	1.511	1.632	1.597	1.497	1.723	1.524	1.287	1.251	1.297
TRINITY_DN27960_c0_g1_i1_orf1	ATP synthase mitochondrial F1 complex assembly factor 1 [Ostrinia furnacalis]	4	294	34.2	7.39	13	5	4	High	1	1.066	1.042	0.552	0.522	0.537	0.559	0.547	0.535	0.565	0.548	0.535	0.68	0.671	0.684
TRINITY_DN17559_c0_g1_i4_orf1	GDP-mannose 4,6 dehydratase isoform X2 [Ostrinia furnacalis]	4	361	40.5	6.86	16	4	4	High	1	1.05	0.988	0.368	0.423	0.419	0.359	0.413	0.389	0.395	0.479	0.395	0.427	0.42	0.415
TRINITY_DN5852_c0_g1_i13_orf1	uncharacterized protein LOC114364714 isoform X3 [Ostrinia furnacalis]	6	367	41.7	6.02	17	7	5	High	1	0.996	0.935	1.136	1.15	1.115	0.945	0.989	1.01	1.209	1.123	1.122	0.922	0.85	0.89
TRINITY_DN1707_c0_g1_i1_orf1	inositol oxygenase-like [Ostrinia furnacalis]	4	291	34.5	5.63	21	4	4	High	1	1.063	1.003	0.169	0.185	0.251	0.252	0.287	0.318	0.299	0.363	0.33	0.252	0.216	0.263
TRINITY_DN3584_c0_g1_i3_orf1	paired amphipathic helix protein Sin3b [Ostrinia furnacalis]	6	1334	148.8	9.32	5	6	6	High	1	0.945	0.948	0.836	0.867	0.872	0.792	0.889	0.831	0.692	0.74	0.837	0.874	0.895	0.858
TRINITY_DN77480_c0_g1_i2_orf1	hypothetical protein evm_008422 [Chilo suppressalis]	4	80	8.8	6.02	48	5	3	High	1	1.06	0.982	1.137	1.235	1.278	1.109	1.157	1.071	0.992	0.745	1.015	0.76	0.812	0.796
TRINITY_DN350_c0_g1_i4_orf1	microtubule-associated protein tau-like isoform X6 [Ostrinia furnacalis]	4	220	23.2	9.88	20	7	1	High	1	0.915	0.86	1.653	1.818	1.665	1.819	1.754	1.568	1.562	1.527	1.556	1.081	0.996	1.135
TRINITY_DN5383_c0_g1_i1_orf1	vacuolar protein sorting-associated protein 35 [Ostrinia furnacalis]	5	435	50.2	6.35	12	8	3	High	1	0.868	0.897	1.015	1.14	0.957	0.98	1.077	0.99	0.912	0.997	1.008	0.867	0.875	0.975
TRINITY_DN14396_c0_g1_i4_orf1	COP9 signalosome complex subunit 7b [Ostrinia furnacalis]	4	273	30.5	6.01	15	5	4	High	1	0.978	0.942	1.044	0.969	0.959	0.998	1.025	1.092	1.047	1.078	0.966	1.209	1.163	1.123
TRINITY_DN35809_c0_g1_i1_orf1	spodomicin-like [Ostrinia furnacalis]	3	62	6.8	6.47	60	7	3	High	1	0.978	0.935	8.035	8.781	7.918	5.366	5.391	5.388	7.27	5.828	8.361	2.392	2.294	2.233
TRINITY_DN6842_c0_g1_i1_orf1	rab3 GTPase-activating protein regulatory subunit [Ostrinia furnacalis]	5	1337	152	6.33	4	5	5	High	1	0.911	0.969	0.87	0.911	1.068	1.064	1.002	0.932	0.915	0.973	0.903	0.857	0.826	0.867
TRINITY_DN3434_c0_g1_i1_orf1	coiled-coil domain-containing protein 47 [Ostrinia furnacalis]	4	422	48.2	6.73	11	5	4	High	1	1.023	1.037	0.802	0.743	0.762	0.759	0.724	0.792	0.707	0.741	0.63	0.764	0.672	0.586
TRINITY_DN13347_c0_g1_i1_orf1	endothelial differentiation-related factor 1 homolog [Ostrinia furnacalis]	6	146	16.1	10.04	36	8	6	High	1	0.972	0.978	0.725	0.739	0.763	0.719	0.795	0.701	0.709	0.646	0.764	0.715	0.711	0.675
TRINITY_DN3952_c0_g1_i3_orf1	protein Skeletor, isoforms D/E-like isoform X1 [Ostrinia furnacalis]	4	1456	162.2	5.85	4	5	4	High	1	0.969	0.998	0.99	1.033	1.014	1.41	1.356	1.297	1.554	1.641	1.885	2.638	2.474	2.534
TRINITY_DN3245_c2_g1_i4_orf1	membrane-associated progesterone receptor component 1-like [Ostrinia furn	4	175	19.4	4.74	29	4	4	High	1	1.029	0.894	0.75	0.837	0.848	0.785	0.816	0.769	0.789	0.712	0.789	0.741	0.728	0.707
TRINITY_DN9920_c0_g1_i1_orf1	uncharacterized protein LOC114351526 [Ostrinia furnacalis]	5	528	57.9	6.54	9	5	5	High	1	0.946	0.928	1.136	1.15	1.101	1.437	1.524	1.548	1.492	1.392	1.348	2.37	2.268	2.492
TRINITY_DN98016_c0_g1_i1_orf1	methanethiol oxidase [Ostrinia furnacalis]	3	109	12.1	7.02	34	6	1	High	1	0.95	0.94	1.258	1.398	1.323	1.525	1.564	1.443	1.151	1.266	1.272	2.017	2.021	2.186
TRINITY_DN304_c2_g1_i1_orf1	unnamed protein product [Chilo suppressalis]	5	2398	270.7	6.87	2	5	5	High	1	1.001	1.021	1.008	0.987	1.041	1.145	1.277	1.161	1.083	1.024	1.002	1.172	1.089	1.054
TRINITY_DN6087_c0_g1_i7_orf1	uncharacterized protein LOC114355564 [Ostrinia furnacalis]	5	555	61.5	5.58	9	6	5	High	1	0.914	0.938	0.286	0.316	0.34	0.319	0.356	0.373	0.32	0.36	0.37	0.41	0.34	0.412
TRINITY_DN19155_c0_g1_i1_orf1	cleavage and polyadenylation specificity factor 73 [Ostrinia furnacalis]	5	686	77.1	6.51	8	5	5	High	1	0.974	1.058	0.714	0.696	0.752	0.627	0.647	0.685	0.632	0.814	0.627	0.872	0.853	0.79
TRINITY_DN391_c1_g2_i1_orf1	NADH dehydrogenase [ubiquinone] 1 alpha subcomplex subunit 13 [Ostrinia f	3	153	18.1	9.35	33	6	3	High	1	1.026	1.02	0.576	0.591	0.605	0.534	0.512	0.562	0.555	0.612	0.523	0.556	0.561	0.528
TRINITY_DN4790_c0_g1_i6_orf1	ADP-ribosylation factor-like protein 8 [Ostrinia furnacalis]	3	187	21.4	7.37	20	4	3	High	1	0.918	0.999	1.328	1.337	1.409	1.171	1.295	1.25	1.177	1.195	1.209	1.499	1.41	1.508
TRINITY_DN38180_c0_g1_i3_orf1	guanine deaminase [Ostrinia furnacalis]	6	443	49.4	6.19	12	7	6	High	1	0.943	0.954	1.183	1.238	1.144	1.434	1.525	1.365	1.277	1.313	1.465	1.141	1.163	1.157
TRINITY_DN30687_c0_g1_i1_orf1	glucose-6-phosphate isomerase [Chelonus insularis]	2	557	62.7	7.01	5	10	1	High	1	0.986	0.956	1.111	1.01	1.048	1.025	0.985	1.009	1.194	1.241	1.064	1.01	1.009	1.109
TRINITY_DN18592_c0_g2_i1_orf1	GATOR complex protein MIOS [Ostrinia furnacalis]	4	372	40.3	7.06	13	4	3	High	1	0.888	0.946	1.211	1.273	1.23	1.325	1.19	1.154	1.176	1.834	1.289	1.261	1.196	1.323
TRINITY_DN27979_c0_g1_i2_orf1	zinc finger CCCH domain-containing protein 15 homolog [Ostrinia furnacalis]	5	381	43.1	6.4	13	5	5	High	1	1.018	1.002	0.433	0.437	0.464	0.443	0.462	0.43	0.436	0.444	0.411	0.497	0.488	0.507
TRINITY_DN9562_c0_g1_i3_orf1	cullin-3 isoform X1 [Ostrinia furnacalis]	5	570	65.8	7.53	10	5	3	High	1	0.906	0.994	1.416	1.428	1.519	1.333	1.534	1.418	1.586	2.31	1.467	1.257	1.298	1.377
TRINITY_DN7861_c0_g1_i5_orf1	cytochrome b5-related protein-like [Ostrinia furnacalis]	3	451	53.3	7.52	9	5	3	High	1	1.043	1.032	0.909	0.863	0.895	1.393	1.301	1.305	1.144	1.046	1.12	0.809	0.754	0.78
TRINITY_DN18648_c0_g1_i1_orf1	PREDICTED: protein Mo25 [Papilio xuthus]	3	335	39.1	7.47	9	4	3	High	1	1.009	0.953	0.843	0.789	0.849	0.939	0.852	0.868	0.943	0.868	0.892	1.006	0.979	0.97
TRINITY_DN5126_c0_g1_i3_orf1	cytochrome P450 monooxygenase CYP4L47 [Ostrinia furnacalis]	4	492	55.5	7.33	10	4	4	High	1	1.031	0.963	1.207	1.095	1.054	1.407	1.37	1.484	1.536	1.555	1.442	0.974	0.906	0.973
TRINITY_DN19702_c0_g1_i4_orf1	AP-3 complex subunit mu-1 [Ostrinia furnacalis]	4	416	46.4	7.87	11	4	4	High	1	1.021	1.097	0.94	1.04	0.994	0.842	1.15	0.972	0.9	0.973	0.883	1.08	1.15	1.129
TRINITY_DN1166_c0_g3_i4_orf1	pleckstrin homology domain-containing family F member 2 isoform X1 [Ostrin	3	288	32.2	7.58	14	4	3	High	1	0.943	1.006	1.05	1.142	1.026	1.063	1.054	1.072	1.087	1.41	1.277	1.223	1.144	1.237
TRINITY_DN98147_c0_g2_i1_orf1	hypothetical protein evm_002297 [Chilo suppressalis]	3	293	31.2	5.52	18	4	3	High	1	1.303	1.262	1.264	1.103	1.174	1.448	1.476	1.402	1.075	0.856	0.999	6.353	6.353	5.842
TRINITY_DN38435_c0_g1_i1_orf1	UDP-glucuronosyltransferase 2B20-like [Ostrinia furnacalis]	5	523	60.4	7.84	11	6	5	High	1	1.021	1.051	0.831	0.826	0.86	0.7	0.694	0.705	0.73	0.719	0.749	0.439	0.433	0.459
TRINITY_DN11124_c0_g1_i4_orf1	hypothetical protein O3G_MSEX014253 [Manduca sexta]	5	302	34.2	5.5	19	5	5	High	1	1.007	1.017	0.663	0.657	0.72	0.669	0.646	0.638	0.658	0.737	0.624	0.707	0.735	0.679
TRINITY_DN1694_c0_g1_i1_orf1	cuticle protein 16.5-like [Ostrinia furnacalis]	3	119	12	9.52	52	4	3	High	1	1.102	0.99	1.496	1.751	1.477	1.384	1.641	1.632	1.534	1.953	1.947	1.528	1.441	1.667
TRINITY_DN1866_c0_g1_i4_orf1	unnamed protein product [Chrysodeixis includens]	5	257	29.5	7.12	20	7	5	High	1	1.038	1.041	0.714	0.727	0.673	0.832	0.834	0.784	0.764	0.764	0.902	0.765	0.764	0.763
TRINITY_DN12567_c0_g1_i1_orf1	PREDICTED: mannose-1-phosphate guanyltransferase beta-like [Amyelois trar	5	369	40.6	6.44	13	7	5	High	1	1.037	1.056	1.035	0.878	1.018	0.972	0.943	1.02	0.945	0.873	0.807	0.909	0.959	0.938
TRINITY_DN34534_c0_g2_i1_orf1	hypothetical protein evm_008316 [Chilo suppressalis]	4	110	11.8	5.11	53	5	4	High	1	1.012	1.003	1.12	1.36	1.183	1.041	1.286	1.082	1.043	1.092	1.146	1.263	1.247	1.359
TRINITY_DN9311_c0_g1_i1_orf1	cuticle protein 8-like [Ostrinia furnacalis]	4	141	15	6.84	52	5	4	High	1	1.032	1.017	0.854	0.91	0.875	0.627	0.699	0.76	0.619	0.626	0.629	6.733	7.248	5.997
TRINITY_DN1034_c0_g2_i1_orf1	glycerol kinase isoform X7 [Ostrinia furnacalis]	3	188	21.6	5.87	21	3	2	High	1	0.907	0.924	1.631	1.227	1.415	1.377	1.76	1.333	1.707	1.448	1.482	1.195	1.195	1.35
TRINITY_DN2202_c0_g1_i9_orf1	vascular endothelial growth factor receptor 1 isoform X3 [Ostrinia furnacalis]	4	1363	153.5	6.52	4	4	4	High	1	0.995	0.936	1.083	1.154	1.138	1.212	1.255	1.304	1.297	1.282	1.219	1.328	1.388	1.306
TRINITY_DN7828_c0_g1_i2_orf1	alpha-N-acetylgalactosaminidase-like isoform X1 [Ostrinia furnacalis]	4	423	48.4	5.31	12	5	4	High	1	0.967	1	0.69	0.692	0.665	0.674	0.693	0.719	0.669	0.651	0.662	1.023	0.954	0.923
TRINITY_DN45953_c0_g1_i1_orf1	UPF0587 protein GA18326-like [Ostrinia furnacalis]	4	161	18.6	5.44	35	4	4	High	1	0.961	0.91	0.762	0.791	0.76	0.997	0.944	0.952	0.919	0.886	0.886	0.862	0.926	0.935
TRINITY_DN389_c0_g1_i2_orf1	uncharacterized protein LOC118068293 isoform X2 [Chelonus insularis]	5	533	60.2	5.25	11	8	4	High	1	1.052	1.026	0.325	0.383	0.364	0.347	0.369	0.34	0.338	0.385	0.367	0.846	0.838	0.854
TRINITY_DN6161_c0_g1_i1_orf1	intersectin-2 isoform X2 [Ostrinia furnacalis]	5	1233	132.4	5.43	5	5	5	High	1	0.952	0.946	0.891	0.92	0.895	0.956	1.14	0.893	0.894	0.981	0.92	1.085	1.072	1.171
TRINITY_DN350_c0_g1_i5_orf1	tau-like protein isoform X6 [Bombyx mori]	4	252	26.7	9.94	16	6	1	High	1	0.957	1.111	2.472	2.882	2.66	2.051	2.305	1.916	2.108	2.066	2.523	1.307	1.511	1.499
TRINITY_DN23069_c0_g2_i3_orf1	uncharacterized protein LOC114364799 [Ostrinia furnacalis]	5	357	38.9	5.9	18	6	5	High	1	1.095	1.104	1.357	1.365	1.277	1.378	1.506	1.485	1.36	1.425	1.385	1.015	1.027	1.036
TRINITY_DN1814_c0_g2_i4_orfp1	TRINITY_DN1814_c0_g2_i4_m.63284 TRINITY_DN1814_c0_g2::TRINITY_DN1814	3	257	30.5	5.6	34	5	3	High	1	1.106	1.047	1.135	1.235	1.22	1.392	1.434	1.315	1.26	1.059	1.284	0.415	0.382	0.399
TRINITY_DN16052_c0_g2_i1_orf1	histone H3, putative [Brugia malayi]	6	150	16.8	11.41	28	8	6	High	1	0.969	1.065	1.172	1.206	1.167	1.152	1.139	1.205	1.087	1.591	1.141	1.151	1.216	1.181
TRINITY_DN28622_c0_g1_i1_orf1	actin-related protein 3 [Ostrinia furnacalis]	5	418	47	6.27	14	6	5	High	1	1.035	0.949	0.781	0.735	0.842	0.735	0.819	0.751	0.678	0.761	0.695	1.01	1.051	1.035
TRINITY_DN36581_c0_g1_i5_orf1	enoyl-CoA delta isomerase 1, mitochondrial-like isoform X1 [Ostrinia furnacali	5	279	30.8	8.51	20	7	5	High	1	0.99	0.955	1.369	1.346	1.356	0.926	0.96	0.955	1.14	1.071	1.119	0.904	0.932	0.907
TRINITY_DN1473_c0_g1_i8_orf1	probable E3 ubiquitin-protein ligase HERC4 isoform X2 [Ostrinia furnacalis]	4	1042	117.7	6.34	4	4	4	High	1	0.913	0.967	0.86	0.887	0.926	0.911	1.073	1.002	0.886	0.89	0.863	0.927	0.925	0.949
TRINITY_DN2751_c0_g1_i3_orf1	unnamed protein product [Chilo suppressalis]	4	306	34.4	7.75	13	5	4	High	1	1.015	0.996	1.035	1.171	0.995	0.946	1.014	0.981	1.079	1.08	1.13	1.134	1.112	1.013
TRINITY_DN28503_c0_g1_i6_orf1	uncharacterized protein LOC114363584 [Ostrinia furnacalis]	5	116	12.8	6.8	42	5	5	High	1	0.903	1.024	2.717	2.955	2.52	1.286	1.4	1.47	1.203	1.113	1.422	0.656	0.583	0.583
TRINITY_DN7740_c0_g1_i2_orf1	D-arabinitol dehydrogenase 1 [Eumeta japonica]	4	83	9.3	8.78	69	6	1	High	1	0.974	1.044	2.343	2.544	2.428	2.711	2.729	2.735	2.163	2.007	2.4	1.371	1.338	1.368
TRINITY_DN146126_c0_g1_i1_orf1	malate dehydrogenase, mitochondrial [Chelonus insularis]	2	340	36	9.42	6	7	2	High	1	0.99	0.989	0.749	0.774	0.796	0.838	0.83	0.766	0.777	0.713	0.862	0.568	0.596	0.586
TRINITY_DN16091_c0_g1_i1_orfp1	TRINITY_DN16091_c0_g1_i1_m.64010 TRINITY_DN16091_c0_g1::TRINITY_DN16	3	123	14.3	7.44	43	4	2	High	1	1.171	1.183	2.342	2.524	2.292	2.868	1.668	2.686	6.238	4.908	7.431	2.228	1.806	2.154
TRINITY_DN25534_c0_g1_i1_orf1	venom serine carboxypeptidase-like [Ostrinia furnacalis]	4	494	55.9	5.44	12	6	4	High	1	0.997	1.141	0.8	0.789	0.789	0.847	0.858	0.838	1.409	0.889	0.709	4.655	4.691	4.714
TRINITY_DN12256_c0_g1_i1_orf1	lysosome membrane protein 2-like [Ostrinia furnacalis]	3	153	17.6	5.05	22	4	3	High	1	0.993	1.009	2.081	2.012	2.022	1.995	1.957	1.951	1.788	1.744	1.659	2.175	2.21	2.202
TRINITY_DN146119_c0_g1_i1_orf1	protein SEC13 homolog [Ostrinia furnacalis]	4	313	34.6	5	16	5	4	High	1	0.983	0.997	0.612	0.637	0.654	0.733	0.752	0.673	0.575	0.528	0.559	0.862	0.881	0.864
TRINITY_DN4135_c0_g1_i5_orf1	probable small nuclear ribonucleoprotein Sm D2 [Manduca sexta]	3	119	13.3	9.83	24	6	3	High	1	1.003	0.931	0.615	0.547	0.639	0.561	0.546	0.577	0.484	0.458	0.513	0.613	0.619	0.632
TRINITY_DN44335_c0_g1_i7_orf1	hypothetical protein SFRURICE_000584 [Spodoptera frugiperda]	3	510	56.5	8.84	9	4	3	High	1	1.074	1.001	0.793	0.735	0.779	0.757	0.797	0.743	0.733	0.722	0.693	0.942	0.967	0.87
TRINITY_DN101991_c0_g1_i5_orf1	hypothetical protein O3G_MSEX015044, partial [Manduca sexta]	3	133	14.7	8	33	3	3	High	1	0.958	1.043	1.063	1.21	1.22	1.208	1.181	1.203	1.218	1.064	1.147	0.696	0.664	0.787
TRINITY_DN556_c0_g2_i2_orf1	serine protease inhibitor dipetalogastin-like [Ostrinia furnacalis]	3	282	29.8	4.98	18	4	1	High	1	0.881	0.924	1.074	0.92	1.036	0.815	0.928	0.636	1.077	0.93	0.9	0.852	0.825	0.881
TRINITY_DN3673_c0_g1_i10_orf1	hypothetical protein evm_008955 [Chilo suppressalis]	4	395	42.6	5.53	14	4	4	High	1	0.896	0.903	0.532	0.588	0.557	0.525	0.525	0.501	0.579	0.612	0.61	0.687	0.702	0.706
TRINITY_DN9090_c0_g1_i9_orf1	CD63 antigen [Ostrinia furnacalis]	3	238	25.6	5.49	13	6	3	High	1	0.95	0.978	2.402	2.417	2.474	2.829	2.691	2.515	3.214	2.752	3.347	2.277	2.397	2.345
TRINITY_DN2943_c2_g3_i1_orf1	RUN and FYVE domain-containing protein 2 isoform X2 [Ostrinia furnacalis]	5	660	75.1	6.44	8	5	5	High	1	0.963	0.906	1.028	1.054	1.183	1.027	1.401	1.244	1.11	1.095	1.067	0.999	0.998	1.025
TRINITY_DN4014_c0_g1_i1_orf1	cyclin-Q [Ostrinia furnacalis]	4	251	28.4	6.99	20	4	4	High	1	0.997	1.036	0.957	1.006	1.046	0.766	0.818	0.864	0.907	0.995	0.861	0.83	0.839	0.843
TRINITY_DN2374_c0_g1_i1_orf1	uncharacterized protein LOC114357127 [Ostrinia furnacalis]	4	389	43.6	10.21	13	4	4	High	1	1.055	0.973	0.709	0.735	0.795	0.643	0.547	0.569	0.582	0.522	0.583	0.8	0.783	0.759
TRINITY_DN21531_c0_g1_i1_orf1	viral IAP-associated factor homolog [Ostrinia furnacalis]	4	229	26.4	4.89	21	4	4	High	1	1.001	0.975	0.367	0.413	0.404	0.35	0.351	0.348	0.396	0.383	0.458	0.608	0.627	0.608
TRINITY_DN6153_c0_g1_i6_orf1	uncharacterized protein LOC114354884 [Ostrinia furnacalis]	2	191	21.4	7.9	19	3	2	High	1	0.951	0.989	0.68	0.751	0.824	0.945	0.982	0.962	0.836	0.891	0.907	0.834	0.862	0.809
TRINITY_DN698_c0_g1_i5_orf1	PREDICTED: small nuclear ribonucleoprotein Sm D3 [Amyelois transitella]	3	131	14.1	10.51	31	4	3	High	1	0.979	1	0.507	0.459	0.497	0.46	0.442	0.436	0.429	0.461	0.383	0.549	0.561	0.556
TRINITY_DN7688_c0_g1_i2_orf1	uncharacterized protein LOC114352518 [Ostrinia furnacalis]	4	412	47.9	5.11	10	4	1	High	1	1.062	0.969	0.06	0.062	0.128	0.176	0.098	0.128	0.12	0.127	0.143	0.091	0.076	0.128
TRINITY_DN119557_c0_g1_i3_orf1	eukaryotic translation initiation factor 4 gamma 2 [Ostrinia furnacalis]	2	369	38.5	11.12	8	4	2	High	1	0.87	0.931	0.914	0.942	0.986	0.979	1.063	1.008	0.94	0.99	0.869	1.02	1.079	1.108
TRINITY_DN430_c0_g1_i5_orf1	hypothetical protein NE865_02252 [Phthorimaea operculella]	5	730	83.6	7.58	8	6	5	High	1	0.998	1.025	0.448	0.486	0.479	0.468	0.435	0.41	0.319	0.308	0.342	0.41	0.41	0.351
TRINITY_DN31047_c0_g1_i4_orf1	4-coumarate--CoA ligase 1-like isoform X1 [Ostrinia furnacalis]	4	546	59.4	8.06	8	5	4	High	1	0.968	0.911	0.431	0.413	0.472	0.368	0.352	0.379	0.343	0.341	0.325	0.337	0.313	0.345
TRINITY_DN15993_c0_g1_i1_orf1	carbonyl reductase [NADPH] 1-like [Ostrinia furnacalis]	5	277	31	6.73	22	6	5	High	1	0.953	0.997	1.044	0.97	1.038	1.039	1.082	1.085	1.042	0.935	0.952	1.063	1.059	0.994
TRINITY_DN3664_c0_g1_i8_orf1	hypothetical protein evm_011332 [Chilo suppressalis]	3	202	23	7.46	23	4	3	High	1	1.044	1.037	0.923	0.888	0.915	0.858	0.867	0.811	0.822	0.793	0.87	0.902	0.921	0.879
TRINITY_DN78_c0_g1_i5_orf1	dolichyl-diphosphooligosaccharide--protein glycosyltransferase subunit STT3/	5	707	79.6	7.17	7	6	5	High	1	0.894	0.997	0.935	1.012	0.947	0.884	0.942	0.969	0.859	0.919	0.942	1.01	0.791	0.946
TRINITY_DN27771_c0_g2_i1_orf1	glycine--tRNA ligase [Ostrinia furnacalis]	4	260	28.6	5.76	20	6	4	High	1	0.913	0.953	1.122	1.186	1.146	1.21	1.266	1.203	1.181	1.352	1.243	1.125	1.097	1.099
TRINITY_DN43350_c0_g3_i1_orf1	uncharacterized protein LOC114355190 [Ostrinia furnacalis]	3	229	25.8	7.17	25	4	3	High	1	1.011	1.047	2.898	2.79	2.676	3.953	3.805	3.526	3.431	3.124	3.496	1.143	1.202	1.283
TRINITY_DN12885_c0_g1_i1_orf1	prolow-density lipoprotein receptor-related protein 1, partial [Ostrinia furnaca	3	880	95.4	6.09	5	3	3	High	1	0.909	0.956	1.312	1.605	1.313	1.28	1.303	1.364	1.355	1.434	1.626	1.644	1.819	1.596
TRINITY_DN6988_c0_g1_i3_orf1	cuticle protein 1-like [Ostrinia furnacalis]	3	91	10.1	6.73	59	5	3	High	1	0.937	0.934	1.584	1.735	1.557	1.655	1.717	1.802	2.184	2.009	2.242	2.073	2.201	2.182
TRINITY_DN1083_c0_g1_i4_orf1	latent-transforming growth factor beta-binding protein 4-like [Ostrinia furnaca	5	1364	151.1	5.2	5	5	5	High	1	1.066	0.995	0.888	0.991	0.901	1.022	1.038	1.005	1.192	1.087	1.185	0.877	0.857	0.907

TRINITY_DN27958_c0_g1_i1_orf1	UV excision repair protein RAD23 homolog A [Ostrinia furnacalis]	3	325	36	4.6	14	4	3	High	1	0.928	0.93	0.96	0.973	0.944	0.825	0.768	0.797	0.867	0.675	0.828	0.835	0.886	0.845
TRINITY_DN45859_c0_g1_i1_orf1	nuclear valosin-containing protein-like [Ostrinia furnacalis]	4	865	94.6	5.31	6	4	4	High	1	0.995	0.951	0.691	0.724	0.719	0.803	0.848	0.728	0.755	0.842	0.784	0.878	0.92	0.857
TRINITY_DN2271_c0_g1_i12_orf1	plasminogen activator inhibitor 1-like [Ostrinia furnacalis]	3	452	50.7	6.29	8	3	3	High	1	1.062	1.029	1.38	1.586	1.443	1.331	1.4	1.31	1.527	1.753	1.892	1.623	1.544	1.795
TRINITY_DN80660_c0_g1_i1_orf1	probable phospholipid hydroperoxide glutathione peroxidase isoform X1 [Pier	4	86	9.5	7.87	52	6	4	High	1	0.97	1.017	1.656	1.543	1.524	1.452	1.32	1.353	1.867	1.78	2.013	1.332	1.287	1.302
TRINITY_DN3439_c0_g2_i2_orf1	histone H2A.Z-specific chaperone CHZ1-like [Ostrinia furnacalis]	4	289	31.9	4.4	14	5	4	High	1	0.884	0.877	4.486	4.35	4.303	5.157	5.169	4.793	3.173	2.864	3.175	1.32	1.384	1.392
TRINITY_DN619_c0_g1_i1_orf1	putative uncharacterized protein DDB_G0271606 [Ostrinia furnacalis]	3	330	39.1	4.94	16	6	3	High	1	0.985	1.028	0.667	0.766	0.608	0.518	0.647	0.521	0.483	0.496	0.479	6.655	6.873	6.177
TRINITY_DN3929_c0_g1_i1_orf1	glutathione S-transferase 1-1-like [Ostrinia furnacalis]	5	166	18.9	6.34	23	5	3	High	1	0.974	1.002	0.468	0.379	0.467	0.509	0.486	0.541	0.516	0.521	0.452	0.413	0.433	0.4
TRINITY_DN3860_c0_g1_i5_orf1	nucleoplasmin-like protein isoform X1 [Hyposmocoma kahamanoa]	4	188	20.9	4.83	30	5	4	High	1	0.972	0.987	0.741	0.791	0.813	0.541	0.515	0.521	0.505	0.471	0.587	0.846	0.753	0.712
TRINITY_DN14584_c0_g1_i1_orf1	ATP-binding cassette sub-family G member 4 [Ostrinia furnacalis]	4	635	70	7.28	7	4	4	High	1	0.982	0.995	0.931	0.899	1.015	0.911	0.946	0.985	0.796	0.906	0.803	0.964	1.024	0.98
TRINITY_DN103107_c0_g1_i2_orf1	superoxide dismutase [Cu-Zn] [Ostrinia furnacalis]	2	79	8.5	7.88	47	3	1	High	1	1.205	0.904	4.549	5.113	4.56	0.554	0.753	0.895	0.773	0.663	0.676	1.026	0.975	0.859
TRINITY_DN8625_c0_g1_i1_orf1	GDP-L-fucose synthase [Ostrinia furnacalis]	5	320	35.9	7.02	18	5	5	High	1	1.037	1.035	1.444	1.231	1.338	1.311	1.256	1.397	2.025	1.655	1.465	1.136	1.02	1.073
TRINITY_DN98249_c0_g1_i1_orf1	laminin subunit alpha-like [Ostrinia furnacalis]	2	111	11.7	4.82	19	3	2	High	1	1.014	0.954	1.026	0.905	1.074	0.941	0.923	0.888	0.933	0.783	0.947	0.909	0.974	0.968
TRINITY_DN6642_c0_g2_i1_orf1	protein purity of essence [Ostrinia furnacalis]	6	828	89.4	8.51	7	6	6	High	1	1.036	0.989	0.857	0.898	0.845	0.745	0.777	0.834	0.801	0.78	0.754	0.926	0.972	0.886
TRINITY_DN33365_c0_g1_i1_orf1	mucin-5AC isoform X1 [Ostrinia furnacalis]	3	1812	204.2	5.87	2	4	3	High	1	0.949	1.029	1.091	1.173	1.014	1.014	1.088	1.064	1.005	1.001	0.942	2.946	2.986	2.918
TRINITY_DN4108_c0_g1_i6_orf1	chromobox protein homolog 1-like [Ostrinia furnacalis]	4	280	32.2	9.11	19	6	4	High	1	0.991	0.987	1.408	1.324	1.42	1.33	1.296	1.269	1.046	1.071	1.118	1.475	1.502	1.443
TRINITY_DN9979_c0_g1_i1_orf1	ADP-dependent glucokinase [Ostrinia furnacalis]	4	490	54.9	6.49	8	4	4	High	1	0.978	0.965	1.21	1.214	1.196	0.914	0.978	0.974	0.812	0.81	0.818	0.864	0.925	0.912
TRINITY_DN8766_c0_g1_i1_orf1	prolow-density lipoprotein receptor-related protein 1, partial [Ostrinia furnaca	4	671	73	6.2	7	4	4	High	1	0.897	0.99	1.549	1.706	1.529	1.87	1.833	1.676	1.476	1.253	1.536	1.887	1.789	1.816
TRINITY_DN582_c0_g1_i2_orf1	unnamed protein product [Chilo suppressalis]	4	578	64	7.97	10	5	2	High	1	0.97	0.985	1.274	1.262	1.434	0.906	1.178	1.14	1.393	1.241	1.157	1.262	1.466	1.144
TRINITY_DN9541_c0_g1_i5_orf1	probable ubiquitin carboxyl-terminal hydrolase FAF-X [Ostrinia furnacalis]	4	968	109.2	6.61	5	4	4	High	1	0.916	0.947	1.003	0.839	0.94	0.946	0.907	1.007	0.934	0.811	0.837	1.079	1.02	0.995
TRINITY_DN142588_c0_g1_i1_orf1	peptidyl-prolyl cis-trans isomerase [Cotesia flavipes]	3	201	22.1	9.11	13	4	3	High	1	1.063	0.958	0.615	0.556	0.633	0.545	0.535	0.53	0.545	0.483	0.516	0.885	0.971	0.87
TRINITY_DN20130_c0_g1_i1_orf1	uncharacterized protein LOC114354518 isoform X1 [Ostrinia furnacalis]	5	994	109.5	9.61	6	5	5	High	1	1.008	0.998	0.719	0.706	0.723	0.692	0.67	0.668	0.638	0.506	0.594	0.686	0.653	0.598
TRINITY_DN12973_c0_g1_i1_orf1	mitochondrial-processing peptidase subunit alpha [Ostrinia furnacalis]	5	538	59.2	6.8	10	5	5	High	1	0.953	0.941	0.431	0.463	0.456	0.455	0.435	0.407	0.394	0.549	0.397	0.47	0.458	0.487
TRINITY_DN827_c1_g1_i1_orf1	peptidoglycan recognition protein 4-like isoform X1 [Ostrinia furnacalis]	4	301	34.6	7.08	15	6	4	High	1	0.996	1.036	1.834	1.765	1.726	1.478	1.494	1.337	1.328	1.361	1.239	1.446	1.469	1.366
TRINITY_DN11798_c0_g2_i1_orf1	N-acetylglucosamine-6-sulfatase-like isoform X2 [Ostrinia furnacalis]	4	496	56.2	5.44	11	5	4	High	1	1.025	0.984	1.566	1.691	1.616	1.273	1.421	1.269	1.595	1.541	1.688	1.203	1.231	1.269
TRINITY_DN20369_c0_g1_i2_orf1	uncharacterized protein LOC114366225 [Ostrinia furnacalis]	4	438	51.4	5.29	11	4	4	High	1	1.06	1.03	0.473	0.485	0.476	0.435	0.464	0.467	0.453	0.398	0.431	0.554	0.567	0.565
TRINITY_DN5037_c0_g1_i3_orf1	glycerol kinase-like isoform X1 [Ostrinia furnacalis]	4	514	55.9	5.68	9	4	4	High	1	1.013	1.095	1.107	1.09	1.042	0.889	0.899	0.942	0.959	1.023	0.848	0.937	0.893	0.947
TRINITY_DN7574_c0_g1_i10_orf1	proteasome activator complex subunit 3 isoform X2 [Ostrinia furnacalis]	3	247	28.3	7.11	14	5	3	High	1	1.158	1.103	0.682	0.623	0.646	0.52	0.48	0.521	0.462	0.408	0.444	0.671	0.632	0.635
TRINITY_DN30_c0_g1_i6_orf1	casein kinase I-like isoform X1 [Hyposmocoma kahamanoa]	4	374	43.4	9.57	11	5	1	High	1	1.031	1.093	0.443	0.444	0.454	0.446	0.508	0.535	0.411	0.439	0.374	0.655	0.603	0.672
TRINITY_DN5170_c0_g1_i5_orf1	hemolymph lipopolysaccharide-binding protein-like isoform X2 [Leguminivora	4	88	10	7.44	57	5	2	High	1	1.178	1.205	2.086	2.174	2.021	1.99	2.144	1.835	0.943	1.135	1.171	1.871	1.804	1.932
TRINITY_DN38650_c0_g1_i2_orf1	elongator complex protein 1 [Ostrinia furnacalis]	5	1269	144.9	6.05	5	5	5	High	1	1.07	1.005	0.711	0.696	0.72	0.761	0.698	0.831	0.804	0.724	0.683	0.796	0.786	0.785
TRINITY_DN21545_c0_g1_i2_orf1	sterile alpha and TIR motif-containing protein 1 isoform X1 [Ostrinia furnacalis	5	1177	129.1	7.05	6	5	5	High	1	1.083	1.044	1.275	1.315	1.182	1.358	1.296	1.278	1.241	1.194	1.319	1.075	1.082	1.073
TRINITY_DN12003_c0_g2_i1_orf1	lactoylglutathione lyase [Ostrinia furnacalis]	4	131	14.8	6.16	37	7	2	High	1	0.94	1.026	1.665	1.528	1.544	1.356	1.434	1.43	1.484	1.413	1.433	1.411	1.306	1.371
TRINITY_DN2083_c0_g1_i4_orf1	uncharacterized protein LOC114359113 [Ostrinia furnacalis]	3	224	24.2	4.65	17	3	3	High	1	1.064	0.989	0.197	0.161	0.224	0.248	0.23	0.233	0.265	0.403	0.238	0.268	0.306	0.297
TRINITY_DN1322_c0_g1_i4_orf1	putative histone-binding protein Caf1 [Papilio machaon]	3	432	48.6	4.93	9	7	3	High	1	1.034	1.075	0.825	0.792	0.806	0.658	0.683	0.747	0.746	1.621	0.766	1.112	1.16	1.069
TRINITY_DN7062_c0_g1_i1_orf1	ribosome maturation protein SBDS [Ostrinia furnacalis]	4	251	28.7	8.88	16	4	4	High	1	1.043	1.04	1.294	1.449	1.435	1.431	1.442	1.384	1.123	1.07	1.342	1.642	1.661	1.687
TRINITY_DN14262_c0_g1_i5_orf1	cytochrome P450 monooxygenase CYP9G18 [Cnaphalocrocis medinalis]	3	114	12.9	4.91	39	4	1	High	1	1.18	1.163	2.215	2.418	2.463	1.917	1.875	1.814	2.247	2.346	2.451	1.666	1.756	1.767
TRINITY_DN7711_c1_g1_i3_orf1	long-chain fatty acid transport protein 1-like [Ostrinia furnacalis]	5	661	73.6	8.91	8	5	5	High	1	0.919	0.927	1.332	1.433	1.265	1.028	1.041	1.074	1.002	0.943	0.923	1.948	1.906	1.931
TRINITY_DN4497_c0_g1_i4_orf1	cytochrome P450 9e2-like [Ostrinia furnacalis]	3	266	31.2	8.81	15	4	1	High	1	1.2	1.18	0.473	0.486	0.442	0.684	0.708	0.656	0.404	0.405	0.526	0.502	0.459	0.528
TRINITY_DN1123_c2_g1_i5_orf1	troponin I isoform X4 [Leguminivora glycinivorella]	5	136	16.3	9.7	40	5	2	High	1	0.903	0.919	0.837	0.902	0.831	0.599	0.687	0.645	0.677	0.606	0.623	0.358	0.347	0.401
TRINITY_DN10810_c0_g1_i4_orf1	arrestin domain-containing protein 17 [Galleria mellonella]	5	425	46.9	6.64	11	7	5	High	1	0.915	1.046	1.24	0.892	1.149	0.992	0.958	1.24	1.229	1.065	0.845	1.118	1.162	1.147
TRINITY_DN3461_c0_g1_i1_orf1	protein SCO1 homolog, mitochondrial [Ostrinia furnacalis]	4	251	28.2	8.92	25	4	4	High	1	0.983	1.006	0.63	0.606	0.606	0.641	0.656	0.631	0.599	0.615	0.579	0.462	0.414	0.442
TRINITY_DN569_c0_g3_i12_orf1	prominin-like protein isoform X2 [Ostrinia furnacalis]	4	930	103.1	7.65	6	5	4	High	1	0.969	0.979	1.162	1.196	1.18	1.193	1.446	1.261	1.201	1.265	1.339	1.619	1.417	1.621
TRINITY_DN649_c1_g1_i13_orf1	U1 small nuclear ribonucleoprotein 70 kDa isoform X2 [Ostrinia furnacalis]	5	294	34.8	10.65	16	6	5	High	1	0.955	0.929	0.755	0.776	0.805	0.717	0.666	0.73	0.717	0.626	0.701	0.816	0.758	0.745
TRINITY_DN3324_c0_g1_i3_orf1	uncharacterized protein LOC114363957 isoform X2 [Ostrinia furnacalis]	4	580	64	6.34	9	5	4	High	1	1.014	1.065	1.328	1.256	1.229	1.028	0.918	1.169	1.003	1.067	0.902	1.107	1.058	1.099
TRINITY_DN2227_c0_g1_i5_orf1	protein 60A [Ostrinia furnacalis]	4	416	46.3	6.86	11	5	4	High	1	0.953	0.964	1.728	1.87	1.694	1.725	1.983	1.583	1.275	1.542	1.444	2.422	2.452	2.366
TRINITY_DN21719_c0_g2_i4_orf1	chymotrypsin-2-like [Ostrinia furnacalis]	3	125	13.6	7.85	42	3	3	High	1	1.071	1.057	1.132	1.042	1.054	1.008	1.068	1.12	0.992	1.134	0.91	1.987	1.779	1.979
TRINITY_DN62707_c0_g1_i1_orf1	uncharacterized protein LOC114362831 [Ostrinia furnacalis]	3	130	14.3	7.43	29	3	2	High	1	0.898	0.868	2.004	1.767	1.707	1.27	1.156	1.426	1.447	1.555	1.309	0.916	0.924	1.01
TRINITY_DN121047_c0_g1_i3_orf1	unnamed protein product [Parnassius apollo]	2	151	16.2	5.12	18	5	1	High	1	1.029	0.945	0.769	0.799	0.793	0.777	0.739	0.779	0.838	0.755	0.87	0.923	0.892	0.859
TRINITY_DN490_c0_g1_i1_orf1	40S ribosomal protein S16-like [Cotesia glomerata]	3	148	16.6	10.13	18	5	1	High	1	1.132	1.268	1.051	1.217	1.071	1.063	1.159	1.124	0.954	1.27	1.008	1.014	0.902	1.02
TRINITY_DN7388_c0_g1_i7_orf1	ras-related protein Rab-18A isoform X1 [Ostrinia furnacalis]	4	203	22.8	7.21	19	5	4	High	1	0.897	0.904	1.072	1.144	1.124	1.337	1.33	1.557	1.079	1.048	1.04	1.278	1.291	1.27
TRINITY_DN3377_c0_g1_i1_orf1	calcyphosin-like protein isoform X3 [Helicoverpa armigera]	6	231	26.8	5.69	19	7	4	High	1	0.973	0.981	1.481	1.548	1.525	1.273	1.098	1.158	1.539	1.217	1.562	1.128	1.091	0.97
TRINITY_DN5675_c0_g1_i6_orf1	probable ATP-dependent RNA helicase DDX43 [Ostrinia furnacalis]	4	659	73.9	6.25	8	5	3	High	1	0.934	0.972	1.134	1.118	1.074	1.082	1.12	1.168	0.945	1.145	0.996	1.258	1.271	1.304
TRINITY_DN41_c0_g1_i5_orf1	putative phospholipase B-like 2 [Ostrinia furnacalis]	5	560	63.9	7.11	10	8	5	High	1	0.953	0.978	1.387	1.235	1.173	1.118	1.176	1.195	1.197	1.384	1.072	1.873	1.806	2.006
TRINITY_DN2941_c0_g1_i1_orf1	FAS-associated factor 1 [Ostrinia furnacalis]	4	641	72	5.25	7	4	4	High	1	0.978	0.868	1.183	1.264	1.209	1.126	1.098	1.094	1.043	0.937	1.044	1.064	1.109	1.072
TRINITY_DN1557_c0_g1_i9_orf1	carboxylesterase CXE18 [Ostrinia furnacalis]	4	564	63.7	5.88	7	4	4	High	1	1.024	1.004	0.485	0.442	0.514	0.378	0.409	0.42	0.416	0.368	0.325	0.311	0.335	0.371
TRINITY_DN42461_c0_g1_i4_orf1	obscurin [Ostrinia furnacalis]	3	97	11.1	5.54	37	5	1	High	1	1.023	1.092	1.003	1.101	1.016	0.859	0.886	0.869	0.744	0.653	0.769	0.276	0.336	0.317
TRINITY_DN1197_c0_g1_i6_orf1	tensin-2-like isoform X6 [Ostrinia furnacalis]	5	1211	129.9	8.46	5	5	5	High	1	0.986	1.011	0.979	1.026	1.057	0.888	0.875	0.891	0.843	0.769	0.882	0.812	0.749	0.745
TRINITY_DN7867_c0_g1_i1_orf1	putative inorganic phosphate cotransporter [Ostrinia furnacalis]	5	472	52	7.68	8	7	5	High	1	1.098	1.051	1.385	1.416	1.392	1.679	1.718	1.6	1.609	1.627	1.659	1.789	1.619	1.785
TRINITY_DN3008_c0_g1_i12_orf1	reticulon-4-interacting protein 1 homolog, mitochondrial [Ostrinia furnacalis]	2	375	39	8.21	9	2	2	High	1	0.978	1.008	0.505	0.552	0.524	0.502	0.553	0.485	0.471	0.728	0.535	0.438	0.438	0.496
TRINITY_DN140423_c0_g1_i2_orf1	obscurin [Ostrinia furnacalis]	2	92	11	6.37	28	4	1	High	1	0.951	0.946	1.018	1.026	1.051	0.93	0.966	0.999	0.883	0.803	0.861	0.96	0.884	0.766
TRINITY_DN15811_c0_g1_i7_orf1	mitochondrial import inner membrane translocase subunit Tim10-like [Ostrinia	4	94	10.7	6.76	48	5	4	High	1	0.952	1.012	0.749	0.947	0.759	0.607	0.736	0.61	0.625	0.648	0.842	0.585	0.571	0.671
TRINITY_DN15865_c0_g1_i1_orf1	carboxylesterase, partial [Ostrinia furnacalis]	4	348	38	8.6	14	5	4	High	1	0.996	0.96	0.685	0.765	0.71	0.809	0.868	0.831	0.778	0.708	0.659	1.759	1.733	1.674
TRINITY_DN25492_c0_g1_i1_orf1	PREDICTED: myrosinase 1-like [Amyelois transitella]	4	506	58.4	5.35	11	5	4	High	1	0.977	1.028	0.857	0.885	0.828	0.783	0.789	0.727	0.79	0.745	0.824	0.533	0.463	0.546
TRINITY_DN1617_c0_g1_i5_orf1	hypothetical protein evm_009822 [Chilo suppressalis]	3	468	51.5	8.47	7	3	3	High	1	0.98	0.988	0.343	0.394	0.368	0.493	0.443	0.492	0.425	0.436	0.436	0.416	0.398	0.413
TRINITY_DN2514_c1_g1_i13_orf1	seroin transcript 1A2 [Ostrinia nubilalis]	4	151	16.6	9.57	39	6	2	High	1	0.991	1.084	1.643	2.14	1.794	2.023	2.149	1.856	1.534	2.204	1.623	0.967	1.037	1.046
TRINITY_DN3712_c0_g1_i1_orf1	ribonuclease Oy [Ostrinia furnacalis]	3	271	31.5	6.15	15	4	3	High	1	0.975	0.829	2.193	1.961	2.027	1.669	1.479	1.66	1.454	1.313	1.437	1.691	1.553	1.429
TRINITY_DN16234_c0_g2_i3_orf1	uncharacterized protein LOC114363370 [Ostrinia furnacalis]	3	72	7.4	8.05	51	6	3	High	1	1.031	1.064	1.561	1.7	1.684	4.11	4.07	3.679	4.371	3.565	4.901	1.907	1.945	1.896
TRINITY_DN36434_c0_g2_i3_orf1	clotting factor B isoform X1 [Ostrinia furnacalis]	3	439	48.8	7.52	9	4	3	High	1	0.926	0.942	1.82	1.907	2.091	2.556	3.157	2.692	2.234	1.993	1.912	1.349	1.157	1.358
TRINITY_DN17376_c0_g1_i2_orf1	E3 UFM1-protein ligase 1 homolog [Ostrinia furnacalis]	5	766	86.2	6.06	6	6	5	High	1	0.964	0.951	0.696	0.687	0.694	0.639	0.676	0.674	0.553	0.535	0.581	0.666	0.618	0.634
TRINITY_DN4301_c0_g1_i5_orf1	protein phosphatase 1 regulatory subunit 12A isoform X1 [Ostrinia furnacalis]	5	877	95.8	6.14	7	5	5	High	1	0.974	0.999	0.815	0.822	0.812	0.813	0.858	0.856	0.855	0.827	0.861	0.945	0.972	0.901
TRINITY_DN6535_c0_g1_i3_orf1	mRNA export factor [Ostrinia furnacalis]	3	350	39.1	7.61	10	4	3	High	1	1.003	1.085	0.764	0.777	0.741	0.663	0.673	0.629	0.609	0.582	0.667	0.697	0.643	0.676
TRINITY_DN500_c0_g1_i1_orf1	splicing factor U2AF 50 kDa subunit isoform X2 [Manduca sexta]	4	418	46.7	8.95	15	5	4	High	1	0.981	0.971	0.719	0.734	0.686	0.567	0.646	0.621	0.745	0.84	0.743	0.704	0.626	0.687
TRINITY_DN25856_c0_g1_i1_orf1	myrosinase 1-like [Ostrinia furnacalis]	4	504	57.7	4.91	9	4	3	High	1	1.033	0.987	0.409	0.392	0.417	0.375	0.383	0.372	0.384	0.361	0.341	0.347	0.317	0.335
TRINITY_DN10639_c0_g1_i6_orf1	protein-glutamate O-methyltransferase-like isoform X1 [Ostrinia furnacalis]	6	478	52.3	7.74	12	6	6	High	1	1.053	1.046	0.932	0.912	0.965	0.936	0.897	0.953	0.948	0.935	0.849	0.84	0.892	0.818
TRINITY_DN4233_c0_g2_i2_orf1	actin-related protein 2/3 complex subunit 5-B [Ostrinia furnacalis]	4	150	16.6	5.19	33	5	4	High	1	0.98	0.946	0.701	0.686	0.761	0.688	0.672	0.748	0.698	0.636	0.598	0.892	0.874	0.902
TRINITY_DN8407_c0_g1_i2_orf1	dihydrolipoyllysine-residue acetyltransferase component of pyruvate dehydrog	3	194	20.6	9.36	25	4	3	High	1	0.954	0.931	1.087	1.146	1.191	1.259	1.232	1.182	1.259	1.507	1.168	1.119	1.133	1.099
TRINITY_DN5162_c0_g1_i3_orf1	steroidogenic acute regulatory protein-like [Ostrinia furnacalis]	5	471	53.3	7.99	11	5	5	High	1	1.015	1.01	0.98	1.034	1.051	0.845	0.863	0.866	0.846	0.83	0.844	1.082	1.056	1.053
TRINITY_DN5174_c0_g3_i1_orf1	protein odr-4 homolog [Ostrinia furnacalis]	3	467	52.4	5.07	8	3	3	High	1	1.012	1.018	1.122	1.112	1.188	1.636	1.744	1.366	1.284	1.283	1.126	1.082	1.099	1.102
TRINITY_DN65518_c0_g1_i1_orf1	unc-112-related protein-like, partial [Ostrinia furnacalis]	2	70	7.8	6.25	70	2	2	High	1	0.938	1.021	1.556	1.543	1.39	1.675	1.492	1.558	1.575	1.602	1.654	1.059	1.137	1.095
TRINITY_DN5675_c0_g1_i1_orf1	unnamed protein product [Danaus chrysippus]	4	71	7.4	10.51	61	5	4	High	1	0.983	0.963	1.116	1.065	1.135	0.964	0.897	0.997	1.181	1.084	1.064	1.04	1.054	1.068
TRINITY_DN3861_c0_g3_i2_orf1	glutamyl aminopeptidase-like isoform X4 [Ostrinia furnacalis]	5	918	105.6	6.52	6	5	5	High	1	1.023	1.016	1.504	1.451	1.494	1.657	1.735	1.646	1.442	1.594	1.533	1.49	1.638	1.542
TRINITY_DN35800_c0_g1_i6_orf1	ommochrome-binding protein-like [Ostrinia furnacalis]	6	281	31.8	4.88	20	6	6	High	1	0.965	0.916	1.121	1.138	1.166	1.205	1.172	1.102	1.35	1.219	1.281	0.952	0.974	0.969
TRINITY_DN31815_c0_g1_i4_orf1	E3 ubiquitin-protein ligase listerin-like [Ostrinia furnacalis]	4	1570	175.5	6.49	3	4	4	High	1	1.011	0.993	0.799	0.877	0.776	0.767	0.824	0.793	0.818	0.769	0.703	0.784	0.82	0.731
TRINITY_DN7516_c0_g2_i1_orf1	serine/threonine-protein kinase 10-like, partial [Ostrinia furnacalis]	4	961	109.9	7.39	4	4	4	High	1	1.036	1.111	0.801	0.847	0.983	0.967	0.956	0.963	0.899	0.9	0.961	1.207	1.133	1.04
TRINITY_DN50787_c0_g2_i2_orf1	40S ribosomal protein S29 [Hyposmocoma kahamanoa]	2	56	6.6	9.91	38	3	2	High	1	0.964	0.912	0.378	0.428	0.48	0.369	0.365	0.37	0.243	0.206	0.243	0.318	0.346	0.341
TRINITY_DN7325_c0_g1_i1_orf1	collagenase-like [Ostrinia furnacalis]	3	144	15.5	7.97	33	4	1	High	1	0.83	0.968	0.518	0.568	0.686	0.71	0.708	0.709	0.706	0.836	0.654	0.509	0.492	0.675
TRINITY_DN42337_c0_g1_i6_orf1	cuticle protein 8-like [Leguminivora glycinivorella]	4	143	15.3	6.58	36	4	1	High	1	0.658	0.891	0.393	0.534	0.568	0.585	0.571	0.581	0.447	0.298	0.581	1.464	1.822	1.408
TRINITY_DN9376_c1_g1_i3_orf1	RB1-inducible coiled-coil protein 1 isoform X4 [Ostrinia furnacalis]	6	1298	147.6	5.44	4	6	6	High	1	1.031	1.079	1.356	1.343	1.366	1.435	1.454	1.465	1.296	1.158	1.239	1.224	1.295	1.173
TRINITY_DN1585_c0_g1_i1_orf1	aldose reductase-like isoform X2 [Ostrinia furnacalis]	4	316	35.7	7.33	9	7	2	High	1	1.11	1.082	0.839	0.859	0.879	1.234	1.268	1.237	1.119	0.921	1.074	0.536	0.568	0.566
TRINITY_DN267_c0_g1_i1_orf1	keratin, type I cytoskeletal 9-like [Ostrinia furnacalis]	3	533	52.5	8.76	9	3	3	High	1	0.904	0.909	0.927	0.955	0.897	0.761	0.818	0.795	0.652	0.713	0.716	3.027	3.37	3.237
TRINITY_DN955_c0_g1_i2_orf1	gloverin-like [Ostrinia furnacalis]	3	194	21.1	6.96	20	5	2	High	1	1.057	0.927	2.521	2.766	2.529	3.106	3.242	2.878	1.358	1.312	1.356	2.532	2.605	2.5
TRINITY_DN394_c0_g1_i2_orf1	uncharacterized protein LOC114351483 [Ostrinia furnacalis]	3	109	12	5.14	28	4	1	High	1	0.883	0.935	3.247	3.352	3.092	4.597	4.633	3.972	2.358	2.151	2.378	0.782	0.826	0.894
TRINITY_DN6586_c0_g1_i1_orf1	fatty acyl-CoA reductase wat-like isoform X1 [Ostrinia furnacalis]	3	626	71.4	7.14	7	3	3	High	1	1.024	1.017	0.59	0.6	0.622	0.563	0.567	0.644	0.607	0.531	0.592	1.738	1.896	1.637
TRINITY_DN50593_c0_g1_i1_orf1	uncharacterized protein LOC114361588 isoform X14 [Ostrinia furnacalis]	3	203	22.4	8.92	17	4	3	High	1	1.093	1.108	1.346	1.318	1.354	1.534	1.573	1.48	1.388	1.532	1.445	0.933	0.861	0.997

TRINITY_DN32359_c0_g2_i1_orf1	PREDICTED: 26S proteasome non-ATPase regulatory subunit 4 isoform X2 [Fo	2	379	41.8	4.81	6	5	2	High	1	1.062	1.044	1.052	0.972	1.033	0.853	0.782	0.878	0.819	0.729	0.764	1.086	1.142	1.084
TRINITY_DN17935_c0_g1_i1_orf1	NEDD8-conjugating enzyme Ubc12 [Ostrinia furnacalis]	4	183	20.8	7.27	23	4	4	High	1	1.005	0.987	0.702	0.714	0.71	0.782	0.717	0.783	0.714	0.714	0.694	0.906	0.918	0.938
TRINITY_DN1048_c0_g1_i6_orf1	uncharacterized protein LOC114360661 [Ostrinia furnacalis]	4	111	12.1	6.35	39	7	4	High	1	0.898	0.864	2.149	2.483	2.22	1.873	1.956	1.811	3.084	2.74	3.593	2.29	2.389	2.296
TRINITY_DN15000_c0_g1_i4_orf1	15-hydroxyprostaglandin dehydrogenase [NAD(+)]-like [Ostrinia furnacalis]	5	249	27.4	6.93	18	6	5	High	1	1.123	1.018	0.91	0.851	0.982	1.973	1.835	2.116	1.018	1.012	0.853	0.686	0.64	0.656
TRINITY_DN14030_c0_g1_i1_orf1	ribosome-recycling factor, mitochondrial [Ostrinia furnacalis]	4	253	28.3	9.5	24	5	4	High	1	0.964	1.032	0.786	0.756	0.815	0.872	0.812	0.84	0.817	0.991	0.783	0.713	0.761	0.694
TRINITY_DN60680_c0_g1_i2_orf1	unnamed protein product [Euphydryas editha]	3	137	15.7	6.68	28	7	3	High	1	1.005	0.989	0.704	0.694	0.671	0.615	0.633	0.619	0.593	0.56	0.602	0.627	0.664	0.605
TRINITY_DN3488_c0_g1_i2_orf1	hsp70-binding protein 1 isoform X1 [Ostrinia furnacalis]	4	350	38.9	4.78	13	4	4	High	1	1.047	0.993	0.646	0.764	0.662	0.556	0.674	0.544	0.664	0.628	0.663	0.736	0.748	0.745
TRINITY_DN14743_c0_g1_i4_orf1	pseudouridine-5'-phosphatase-like [Ostrinia furnacalis]	4	232	26.3	5.66	21	5	4	High	1	1.003	0.931	0.845	0.775	0.864	0.947	0.969	0.921	0.912	0.8	0.824	0.76	0.74	0.745
TRINITY_DN14460_c0_g1_i6_orf1	scavenger receptor class B member 1-like [Ostrinia furnacalis]	3	555	62.9	5.69	6	3	3	High	1	1.061	1.131	2.208	2.089	2.099	2.323	2.296	2.346	2.282	2.113	2.311	2.468	2.577	2.257
TRINITY_DN5074_c0_g1_i7_orf1	zonadhesin-like [Ostrinia furnacalis]	4	230	25.8	5.36	21	4	4	High	1	0.976	0.977	0.972	0.972	0.978	0.888	0.979	0.9	0.737	0.742	0.696	2.21	2.265	2.218
TRINITY_DN4040_c0_g1_i10_orf1	hypothetical protein evm_007488 [Chilo suppressalis]	5	1162	128.9	6.33	6	5	5	High	1	0.969	0.966	0.405	0.408	0.43	0.428	0.494	0.468	0.495	0.463	0.469	0.441	0.494	0.471
TRINITY_DN24399_c0_g1_i1_orf1	retinol-binding protein pinta-like [Ostrinia furnacalis]	4	307	35.4	6.44	15	4	4	High	1	1.071	1.024	0.454	0.44	0.494	0.272	0.299	0.318	0.366	0.316	0.374	0.287	0.335	0.29
TRINITY_DN34423_c0_g1_i2_orf1	THAP domain-containing protein 4-like [Ostrinia furnacalis]	2	87	9.9	8.75	24	7	1	High	1	0.992	1.041	2.188	1.914	1.932	2.109	1.877	1.746	2.25	2.307	2.195	1.647	1.684	1.669
TRINITY_DN4324_c0_g1_i1_orf1	uncharacterized protein LOC114354985 isoform X1 [Ostrinia furnacalis]	4	143	16	9.52	20	4	4	High	1	0.981	0.97	0.995	0.99	0.937	1.045	1.21	1.179	1.088	0.995	0.988	3.384	3.473	3.508
TRINITY_DN2107_c0_g2_i3_orf1	LIM and SH3 domain protein Lasp [Ostrinia furnacalis]	5	432	49.4	7.37	12	5	5	High	1	0.961	1.009	1.228	1.131	1.253	1.229	1.115	1.194	1.43	1.05	1.09	1.785	1.819	1.778
TRINITY_DN135679_c0_g1_i2_orfp	TRINITY_DN135679_c0_g1_i2_m.85525 TRINITY_DN135679_c0_g1::TRINITY_DN	3	54	6.1	8.54	46	9	1	High	1	0.883	0.962	1.602	1.651	1.529	1.493	1.584	1.522	1.969	1.85	2.024	0.859	0.84	0.913
TRINITY_DN18148_c0_g2_i1_orf1	nudC domain-containing protein 1 [Ostrinia furnacalis]	4	575	65.8	6.11	7	5	4	High	1	0.938	0.953	0.987	0.995	0.997	1.106	1.153	1.107	1.282	1.336	1.286	1.083	1.098	1.198
TRINITY_DN28509_c0_g1_i1_orf1	39S ribosomal protein L43, mitochondrial [Ostrinia furnacalis]	5	185	21.2	9.39	35	5	5	High	1	1.01	0.955	0.541	0.548	0.535	0.595	0.616	0.626	0.661	0.738	0.636	0.702	0.665	0.654
TRINITY_DN2064_c1_g1_i1_orf1	hypothetical protein evm_007509 [Chilo suppressalis]	3	182	20.8	6.86	21	3	3	High	1	1.005	1.041	0.537	0.563	0.571	0.499	0.564	0.591	0.482	0.693	0.496	0.48	0.419	0.518
TRINITY_DN13371_c0_g1_i4_orf1	ATP synthase mitochondrial F1 complex assembly factor 2 [Ostrinia furnacalis]	3	284	32.3	7.14	15	3	3	High	1	0.975	1.035	0.734	0.787	0.792	0.731	0.706	0.711	0.685	0.667	0.787	0.629	0.62	0.608
TRINITY_DN15400_c0_g1_i1_orf1	uncharacterized protein LOC114366781 [Ostrinia furnacalis]	3	91	10.3	6.89	36	5	1	High	1	1.106	1.038	4.242	4.568	4.731	4.133	3.865	3.994	1.341	1.199	1.557	6.464	7.869	5.792
TRINITY_DN33893_c0_g1_i1_orf1	high mobility group protein I-like [Ostrinia furnacalis]	5	118	12.4	9.66	33	9	5	High	1	0.928	1.016	0.901	0.953	0.958	0.881	0.882	0.87	0.714	0.657	0.776	1.306	1.279	1.249
TRINITY_DN14755_c0_g1_i4_orf1	aldehyde dehydrogenase, dimeric NADP-preferring isoform X6 [Ostrinia furna	3	93	9.9	4.65	34	5	1	High	1	0.966	0.896	0.844	1.052	1.117	0.919	1.14	1.018	1.115	1.081	0.99	0.782	0.953	0.818
TRINITY_DN9146_c0_g1_i1_orf1	drebrin-like protein [Ostrinia furnacalis]	4	558	62.4	4.94	10	4	4	High	1	0.986	1.083	0.772	0.816	0.77	0.792	0.762	0.757	0.786	0.717	0.757	1.091	0.906	1.067
TRINITY_DN2473_c0_g1_i2_orf1	translation initiation factor eIF-2B subunit delta [Ostrinia furnacalis]	6	662	72.3	9.38	9	6	6	High	1	0.983	0.934	0.855	0.928	0.877	0.907	1.052	0.898	0.715	0.881	0.737	0.779	0.768	0.815
TRINITY_DN10646_c0_g1_i2_orf1	tyrosine-protein phosphatase non-receptor type 61F-like isoform X1 [Vanessa	4	459	52.2	5.78	11	4	4	High	1	0.978	1.066	1.011	0.989	1.067	1.12	1.108	1.172	1.238	1.16	1.183	1.336	1.335	1.318
TRINITY_DN6426_c0_g1_i2_orf1	ras-related protein Rap1 [Ostrinia furnacalis]	2	184	20.9	8.12	13	3	2	High	1	0.995	0.971	1.102	1.086	1.071	1.156	1.265	1.201	1.19	1.129	1.115	1.223	1.247	1.176
TRINITY_DN5682_c0_g1_i6_orf1	spodomicin-like [Ostrinia furnacalis]	2	63	7	6.73	40	10	2	High	1	1.065	1.069	2.277	2.548	2.285	2.053	1.761	1.995	2.008	1.577	2.078	1.795	1.908	1.661
TRINITY_DN11970_c0_g1_i4_orf1	myb-like protein AA [Ostrinia furnacalis]	3	1125	126.8	6.43	5	3	3	High	1	0.944	0.93	0.841	0.856	0.856	0.901	1.324	1.003	1.036	1.226	1.057	2.887	3.101	2.895
TRINITY_DN69871_c0_g1_i1_orf1	translocon-associated protein subunit gamma [Venturia canescens]	2	182	20.7	9.48	8	4	2	High	1	0.929	1.027	1.182	1.138	1.056	1.014	0.938	1.048	0.964	1.07	0.883	0.799	0.83	0.853
TRINITY_DN47257_c0_g1_i4_orf1	PREDICTED: microtubule-actin cross-linking factor 1-like, partial [Amyelois tra	4	101	11.5	5.86	38	6	2	High	1	1.081	1.064	0.97	0.933	0.917	0.932	0.871	0.988	0.834	0.808	0.842	0.694	0.7	0.677
TRINITY_DN6876_c0_g2_i1_orf1	serine/threonine-protein phosphatase 5 [Spodoptera litura]	5	490	56	6.7	12	5	5	High	1	0.921	1.027	0.739	0.749	0.785	0.714	0.722	0.697	0.697	0.831	0.713	0.689	0.674	0.687
TRINITY_DN2013_c0_g1_i15_orf1	high mobility group protein DSP1-like isoform X1 [Ostrinia furnacalis]	4	325	38.1	8.47	18	5	4	High	1	0.932	0.948	1.205	1.074	1.16	1.096	1.291	1.118	1.166	1.324	1.228	1.062	1.213	1.128
TRINITY_DN2114_c0_g1_i5_orf1	vegetative cell wall protein gp1-like isoform X1 [Ostrinia furnacalis]	4	236	25.5	7.42	16	5	4	High	1	0.98	0.953	0.152	0.16	0.217	0.23	0.204	0.211	0.189	0.195	0.214	0.144	0.142	0.146
TRINITY_DN840_c5_g1_i11_orf1	unnamed protein product [Diatraea saccharalis]	4	1450	158.8	7.18	4	4	4	High	1	0.983	0.992	0.667	0.701	0.665	0.728	0.784	0.719	0.732	1.759	0.744	0.751	0.701	0.755
TRINITY_DN9156_c0_g1_i1_orf1	GTP:AMP phosphotransferase AK3, mitochondrial [Ostrinia furnacalis]	5	253	29	9.44	19	5	5	High	1	1.038	1.077	0.535	0.507	0.55	0.634	0.696	0.629	0.65	0.674	0.579	0.611	0.654	0.622
TRINITY_DN2290_c0_g1_i2_orfp1	TRINITY_DN2290_c0_g1_i2_m.69732 TRINITY_DN2290_c0_g1::TRINITY_DN229C	2	233	26.1	6.62	16	2	2	High	1	0.911	0.941	1.412	1.525	1.399	0.682	0.879	0.972	0.919	0.739	0.984	3.127	2.877	2.382
TRINITY_DN7942_c0_g1_i1_orf1	hypothetical protein evm_012160 [Chilo suppressalis]	3	122	14.2	8.73	25	3	3	High	1	1.077	1.008	0.62	0.652	0.725	0.651	0.694	0.707	0.644	0.692	0.723	0.776	0.803	0.802
TRINITY_DN49786_c0_g1_i1_orf1	UDP-glucuronosyltransferase 2B15-like isoform X1 [Ostrinia furnacalis]	4	517	58.8	8.69	9	4	4	High	1	1	0.973	0.774	0.718	0.815	1.082	1.012	1.062	0.949	0.985	0.837	0.752	0.805	0.76
TRINITY_DN4401_c0_g2_i1_orf1	hypothetical protein evm_003554 [Chilo suppressalis]	2	83	9.1	7.27	48	2	1	High	1	0.972	0.857	0.741	0.764	0.712	0.899	1.057	1.025	0.733	0.875	0.791	1.226	1.059	1.01
TRINITY_DN3307_c1_g1_i2_orf1	BTB/POZ domain-containing protein 2-like [Ostrinia furnacalis]	3	420	47	6.28	7	3	2	High	1	1.178	1.004	1.387	1.335	1.194	0.644	0.546	0.627	0.694	0.769	0.737	0.883	0.801	0.806
TRINITY_DN26013_c0_g1_i1_orf1	E3 ubiquitin-protein ligase CHIP [Ostrinia furnacalis]	5	287	33.8	5.96	20	5	5	High	1	0.969	0.964	1.297	1.348	1.295	1.193	1.143	1.148	1.27	1.218	1.281	1.395	1.374	1.398
TRINITY_DN31118_c1_g1_i1_orf1	endocuticle structural glycoprotein ABD-4-like [Ostrinia furnacalis]	1	112	12	4.41	23	3	1	High	1	0.992	0.964	0.809	0.902	0.84	0.94	0.932	0.879	0.811	0.66	0.912	0.193	0.137	0.182
TRINITY_DN16343_c0_g1_i6_orf1	aminopeptidase N6 [Ostrinia nubilalis]	5	878	100.2	5.81	7	5	5	High	1	1.026	1.015	0.456	0.425	0.482	0.453	0.478	0.467	0.446	0.474	0.491	0.436	0.436	0.407
TRINITY_DN28695_c0_g1_i1_orf1	proteasome subunit beta type-3 [Ostrinia furnacalis]	3	205	23.1	5.12	17	6	3	High	1	0.986	0.994	0.988	0.967	1.005	0.939	1.039	0.958	0.97	1.044	0.924	0.93	0.921	1
TRINITY_DN2881_c0_g1_i7_orf1	5'-3' exoribonuclease 1 [Ostrinia furnacalis]	4	1529	175.1	6.92	3	4	4	High	1	0.892	1.014	0.938	1.023	1.072	0.955	1.182	1.1	0.896	0.904	0.782	0.94	1.036	1.092
TRINITY_DN272_c0_g1_i1_orf1	vacuolar protein sorting-associated protein 11 homolog [Ostrinia furnacalis]	4	863	98.4	6.87	7	4	4	High	1	1.084	1.054	1.318	1.444	1.312	1.253	1.321	1.407	1.502	1.436	1.764	2.002	1.44	1.547
TRINITY_DN53747_c0_g3_i1_orf1	acidic leucine-rich nuclear phosphoprotein 32 family member A isoform X3 [H	4	258	29.2	4.32	21	4	4	High	1	0.976	1.096	0.882	0.88	0.901	0.91	0.937	0.923	0.872	0.93	1.007	0.905	0.921	0.968
TRINITY_DN184_c0_g1_i1_orf1	macrophage mannose receptor 1-like isoform X1 [Maniola jurtina]	3	322	35.7	6.06	14	3	1	High	1	0.995	0.997	3.042	2.844	3.001	0.823	0.839	1.303	1.647	1.61	1.688	1.05	1.72	1.568
TRINITY_DN15046_c0_g1_i8_orf1	epidermal retinol dehydrogenase 2-like isoform X1 [Ostrinia furnacalis]	3	309	34.7	6.93	13	4	3	High	1	1.003	0.972	0.205	0.216	0.235	0.277	0.299	0.302	0.326	0.394	0.329	0.243	0.206	0.262
TRINITY_DN2859_c0_g1_i7_orf1	cleavage and polyadenylation specificity factor subunit 5 [Ostrinia furnacalis]	4	227	25.9	8.88	15	4	4	High	1	1.041	0.954	0.724	0.678	0.776	0.715	0.713	0.714	0.715	0.681	0.748	0.804	0.702	0.684
TRINITY_DN18863_c0_g1_i3_orf1	splicing factor 3A subunit 1 isoform X1 [Ostrinia furnacalis]	4	527	58.8	5.01	11	4	4	High	1	1.019	1.127	0.883	0.846	0.857	0.772	0.751	0.847	0.729	0.805	0.809	0.911	0.874	0.853
TRINITY_DN19122_c0_g1_i7_orf1	phosphatidylserine decarboxylase proenzyme, mitochondrial [Ostrinia furnacal	4	379	43.4	9.5	12	4	4	High	1	0.972	1.036	0.52	0.49	0.56	0.607	0.59	0.604	0.574	0.624	0.569	0.548	0.537	0.554
TRINITY_DN7787_c0_g1_i1_orf1	trimeric intracellular cation channel type 1B.1 [Manduca sexta]	3	275	30.3	9.2	11	3	3	High	1	1.009	0.96	0.536	0.478	0.533	0.599	0.554	0.626	0.514	0.494	0.481	0.627	0.642	0.635
TRINITY_DN6482_c0_g1_i1_orf1	endocuticle structural glycoprotein SgAbd-5-like [Ostrinia furnacalis]	3	103	11.3	4.81	46	3	3	High	1	0.966	0.993	1.001	1.103	1.102	1.316	1.323	1.26	1.192	1.019	1.3	0.564	0.486	0.538
TRINITY_DN13941_c0_g1_i6_orf1	tryptophan 2,3-dioxygenase [Ostrinia furnacalis]	4	399	46.1	6.3	11	4	4	High	1	0.943	0.99	0.849	0.776	0.913	0.814	0.708	0.853	0.904	0.866	0.86	0.905	0.855	0.813
TRINITY_DN795_c0_g1_i6_orf1	ubiquitin carboxyl-terminal hydrolase 45 [Ostrinia furnacalis]	5	996	110.2	6.19	5	6	5	High	1	0.976	1.017	0.868	0.872	0.914	0.945	0.944	0.905	0.879	0.849	0.92	0.997	1.088	1.009
TRINITY_DN26947_c0_g1_i1_orf1	probable 39S ribosomal protein L24, mitochondrial [Ostrinia furnacalis]	4	253	29.4	9.42	23	4	4	High	1	0.977	1.014	0.825	0.79	0.862	0.811	0.925	0.85	0.899	1.067	0.954	0.777	0.694	0.766
TRINITY_DN21792_c0_g1_i1_orf1	probable 28S ribosomal protein S25, mitochondrial [Ostrinia furnacalis]	4	167	18.7	9.11	23	4	4	High	1	0.981	0.982	0.493	0.44	0.458	0.51	0.498	0.509	0.543	0.555	0.488	0.548	0.512	0.553
TRINITY_DN5133_c0_g1_i7_orf1	epimerase family protein SDR39U1 [Ostrinia furnacalis]	4	305	33.8	9.07	14	6	4	High	1	1.029	1.092	0.64	0.635	0.665	0.746	0.83	0.748	0.659	0.662	0.733	0.763	0.731	0.713
TRINITY_DN26439_c0_g1_i2_orf1	uncharacterized protein LOC114351853 [Ostrinia furnacalis]	3	101	10.9	6.83	50	3	3	High	1	0.951	1.081	2.138	2.413	2.41	2.135	1.943	1.956	1.923	1.525	2.103	1.226	1.233	1.138
TRINITY_DN50151_c0_g1_i1_orf1	heat shock 70 kDa protein 14 [Ostrinia furnacalis]	6	473	51.7	5.12	14	7	6	High	1	0.978	1.041	0.561	0.54	0.606	0.553	0.554	0.545	0.51	0.509	0.494	0.59	0.57	0.612
TRINITY_DN4770_c0_g1_i4_orf1	transportin-3 isoform X1 [Ostrinia furnacalis]	3	920	102.4	5.96	5	3	3	High	1	1.099	1.115	0.858	0.882	0.865	0.746	0.871	0.807	0.768	0.736	0.723	0.951	0.881	0.914
TRINITY_DN38783_c0_g1_i1_orf1	regucalcin-like [Ostrinia furnacalis]	4	163	18.3	5.5	34	5	2	High	1	0.925	0.91	1.428	1.772	1.66	2.013	2.34	2.18	1.644	2.777	1.377	1.135	1.149	1.151
TRINITY_DN7682_c0_g1_i2_orf1	myogenesis-regulating glycosidase isoform X1 [Ostrinia furnacalis]	5	965	109.6	6.05	5	5	5	High	1	0.954	1.077	1.133	1.07	1.086	1.035	1.06	1.039	0.973	0.889	0.916	1.305	1.279	1.387
TRINITY_DN1408_c0_g1_i10_orf1	protein diaphanous [Ostrinia furnacalis]	5	1091	121.5	7.66	6	5	5	High	1	1.036	0.969	1.158	1.044	1.168	1.181	1.062	1.129	1.28	1.266	1.283	1.272	1.324	1.319
TRINITY_DN886_c0_g1_i1_orf1	collagenase-like isoform X1 [Ostrinia furnacalis]	3	280	29.3	7.23	14	4	3	High	1	0.939	1.03	0.353	0.416	0.406	0.462	0.493	0.467	0.483	0.48	0.532	0.487	0.5	0.482
TRINITY_DN2212_c0_g1_i1_orf1	uncharacterized protein LOC114356884 [Ostrinia furnacalis]	4	401	43.8	7.23	13	4	4	High	1	0.913	1.073	0.941	0.908	0.933	0.924	1.033	0.964	1.07	1.134	0.981	0.912	0.931	0.939
TRINITY_DN4695_c0_g1_i4_orf1	glutathione S-transferase epsilon 3 [Ostrinia furnacalis]	4	234	27	6.64	18	5	4	High	1	0.983	0.976	1.248	1.225	1.251	1.398	1.467	1.398	1.218	1.434	1.139	0.983	0.979	1.034
TRINITY_DN41065_c0_g1_i5_orf1	uncharacterized protein LOC114362544 [Ostrinia furnacalis]	3	223	25.1	6.14	17	4	3	High	1	1.049	0.976	1.115	1.072	1.041	1.074	1.153	1.091	1.293	1.056	1.25	1.195	1.193	1.216
TRINITY_DN85290_c0_g2_i1_orf1	unnamed protein product, partial [Brenthis ino]	3	68	7.6	8.7	56	3	1	High	1	1.025	0.933	3.707	3.706	3.768	1.444	1.221	1.393	3.913	4.42	4.533	1.771	1.858	1.907
TRINITY_DN10079_c0_g1_i1_orf1	PREDICTED: ubiquitin-conjugating enzyme E2 H [Amyelois transitella]	3	204	22.4	4.97	17	4	3	High	1	1.036	1.079	1.448	1.438	1.428	1.217	1.19	1.178	1.193	1.183	1.223	1.29	1.18	1.139
TRINITY_DN66287_c0_g1_i1_orfp1	tenascin-like isoform X4 [Trichoplusia ni]	2	107	11.9	5.47	25	4	2	High	1	1.177	1.077	3.548	3.879	3.053	0.875	0.933	0.899	0.859	0.943	1.044	0.8	0.837	0.999
TRINITY_DN3647_c1_g1_i5_orf1	unnamed protein product, partial [Iphiclides podalirius]	4	312	33.9	4.87	24	4	4	High	1	1.022	1.058	1.963	2.154	2.114	1.696	1.56	1.537	1.424	1.346	1.492	2.216	2.262	2.081
TRINITY_DN1477_c0_g1_i5_orf1	mitochondrial import inner membrane translocase subunit Tim9 [Ostrinia furna	3	91	10.7	6.52	35	3	3	High	1	1.048	1.001	0.537	0.498	0.587	0.507	0.5	0.462	0.516	0.418	0.508	0.509	0.54	0.504
TRINITY_DN3984_c0_g1_i4_orf1	uncharacterized protein LOC114360871 isoform X1 [Ostrinia furnacalis]	4	1311	145.6	6.77	4	4	4	High	1	0.869	0.785	0.776	0.713	0.688	0.723	0.727	0.629	0.757	0.747	0.77	0.819	0.823	0.887
TRINITY_DN7688_c0_g1_i10_orf1	uncharacterized protein LOC114352518 [Ostrinia furnacalis]	4	413	47.9	5.11	10	4	1	High	1	1.059	0.955	0.119	0.081	0.19	0.162	0.228	0.181	0.134	0.172	0.19	0.136	0.149	0.163
TRINITY_DN8406_c0_g1_i3_orfp1	TRINITY_DN8406_c0_g1_i3_m.76210 TRINITY_DN8406_c0_g1::TRINITY_DN8406	4	153	18.5	6.98	46	6	1	High	1	0.936	0.979	0.866	0.948	0.969	0.878	0.991	0.906	0.832	0.669	0.853	0.886	0.872	0.797
TRINITY_DN13259_c0_g1_i2_orf1	mitotic spindle assembly checkpoint protein MAD1 [Pectinophora gossypiella]	4	719	82.8	6.76	7	4	4	High	1	0.99	1.088	0.784	0.828	0.816	0.733	0.852	0.803	0.691	0.679	0.735	0.798	0.741	0.707
TRINITY_DN18172_c0_g1_i6_orf1	digestive cysteine proteinase 2-like [Ostrinia furnacalis]	3	506	57.6	5.29	7	5	3	High	1	0.999	1.015	0.226	0.218	0.242	0.275	0.276	0.276	0.263	0.286	0.247	0.315	0.327	0.324
TRINITY_DN6307_c0_g1_i5_orf1	putative helicase mov-10-B.1 [Ostrinia furnacalis]	4	895	103.2	8.69	6	4	4	High	1	1.045	0.996	0.676	0.693	0.684	0.626	0.586	0.642	0.691	0.765	0.593	0.653	0.648	0.603
TRINITY_DN3177_c0_g1_i1_orf1	Pupal cuticle protein PCP52 [Papilio xuthus]	1	170	17.4	6.87	8	5	1	High	1	0.913	0.939	0.927	1.02	0.918	1.055	1.167	1.018	0.836	0.862	0.907	1.316	1.354	1.33
TRINITY_DN17726_c0_g1_i1_orf1	ubiquitin conjugation factor E4 A isoform X1 [Ostrinia furnacalis]	3	1038	116.5	5.57	4	3	3	High	1	0.942	0.942	0.769	0.84	0.754	0.697	0.791	0.726	0.793	0.731	0.647	0.803	0.85	0.868
TRINITY_DN446_c0_g1_i20_orf1	uncharacterized protein LOC114356043 [Ostrinia furnacalis]	3	1422	159.1	9.57	3	3	3	High	1	1.051	1.044	0.748	0.788	0.775	0.688	0.806	0.804	0.981	0.921	0.75	0.845	0.857	0.852
TRINITY_DN18538_c0_g3_i1_orf1	poly(A)-specific ribonuclease PARN-like [Ostrinia furnacalis]	4	517	60.1	7.09	8	4	4	High	1	1.16	1.057	1.317	1.316	1.419	1.654	1.628	1.564	1.476	1.314	1.638	1.563	1.514	1.665
TRINITY_DN8359_c0_g1_i5_orf1	zinc finger protein 622 [Ostrinia furnacalis]	4	394	45.5	6.77	12	4	4	High	1	1.09	1.121	0.926	0.909	0.878	0.956	0.962	1.045	0.888	0.949	0.922	0.986	0.911	0.911
TRINITY_DN12514_c0_g2_i1_orf1	peroxidase isoform X3 [Ostrinia furnacalis]	3	699	77.4	6.23	7	3	3	High	1	0.854	0.951	1.356	1.348	1.193	1.11	1.392	1.479	1.595	1.985	1.437	1.47	1.576	1.542
TRINITY_DN2997_c0_g1_i6_orf1	titin-like [Ostrinia furnacalis]	5	2113	231.4	5.01	3	5	5	High	1	0.98	1.045	0.666	0.873	0.645	0.566	0.573	0.542	0.613	0.492	0.624	0.73	0.713	0.688
TRINITY_DN58261_c0_g1_i2_orf1	15-hydroxyprostaglandin dehydrogenase [NAD(+)]-like [Ostrinia furnacalis]	4	192	21.1	6.9	25	6	1	High	1	1.008	0.943	1.266	1.307	1.287	1.541	1.41	1.296	1.376	1.319	1.149	0.782	0.801	0.74
TRINITY_DN3430_c0_g1_i1_orf1	protein HGV2-like isoform X2 [Ostrinia furnacalis]	4	346	37.7	4.27	14	5	4	High	1	1.083	1.079	0.944	0.943	0.965	0.964	0.927	0.909	1.059	0.921	0.916	1.305	1.412	1.393
TRINITY_DN59829_c0_g1_i1_orf1	putative mediator of RNA polymerase II transcription subunit 12 [Ostrinia furna	4	583	67.1	9.2	10	5	4	High	1	0.956	1.058	0.544	0.564	0.59	0.512	0.488	0.482	0.533	0.472	0.466	1.417	1.416	1.369
TRINITY_DN1020_c0_g1_i3_orf1	beta-arrestin-1 isoform X2 [Ostrinia furnacalis]	3	411	45.7	6.54	10	3	3	High	1	1.033	0.986	0.967	1.019	0.959	1.031	1.212	1.255	1.123	1.245	1.047	1.113	1.004	1.095
TRINITY_DN1589_c0_g1_i7_orf1	unnamed protein product [Chilo suppressalis]	4	1184	132.9	9.04	4	4	4	High	1	0.907	1.006	0.918	0.878	0.904	0.93	0.945	0.954	0.773	0.911	0.777	0.877	0.872	0.898
TRINITY_DN695_c0_g1_i12_orf1	seroin transcript 2A, partial [Ostrinia nubilalis]	3	141	15.1	8.46	30	4	1	High	1	1.068	1.056	1.141	1.218	1.017	1.136	1.195	0.94	0.923	0.858	1.082	0.361	0.424	0.46

TRINITY_DN72707_c0_g1_i1_orf1	uncharacterized protein LOC114357549 [Ostrinia furnacalis]	3	535	60.3	6.23	7	3	3	High	1	0.989	0.992	0.259	0.259	0.278	0.268	0.253	0.272	0.301	0.296	0.321	0.258	0.245	0.257
TRINITY_DN22597_c0_g1_i4_orf1	uncharacterized protein LOC114361588 isoform X16 [Ostrinia furnacalis]	3	109	12.4	4.7	29	4	3	High	1	1.06	0.991	1.713	1.903	1.658	1.587	1.667	1.575	1.399	1.302	1.668	0.892	0.783	0.903
TRINITY_DN2977_c0_g1_i3_orf1	transmembrane 9 superfamily member 3 [Ostrinia furnacalis]	3	575	66.6	6.87	6	3	3	High	1	1.143	1.025	0.622	0.53	0.627	0.598	0.556	0.581	0.568	0.57	0.51	0.854	0.888	0.863
TRINITY_DN2732_c0_g1_i4_orf1	hypothetical protein evm_010211 [Chilo suppressalis]	5	457	52.2	6.64	11	6	4	High	1	1.051	1.073	1.114	1.187	1.154	1.196	1.106	1.248	1.06	1.072	1.034	1.267	1.165	1.041
TRINITY_DN483_c0_g1_i6_orf1	uncharacterized protein LOC114354803 isoform X1 [Ostrinia furnacalis]	3	799	90.7	6.95	4	3	3	High	1	1.038	0.969	1.079	1.158	1.288	1.584	1.788	1.467	1.127	0.882	1.013	1.157	1.155	1.219
TRINITY_DN38562_c0_g1_i3_orf1	persulfide dioxygenase ETHE1, mitochondrial isoform X1 [Ostrinia furnacalis]	3	265	29	7.33	13	5	3	High	1	0.985	1.005	0.634	0.641	0.66	0.796	0.77	0.737	0.776	0.727	0.92	0.531	0.541	0.526
TRINITY_DN41761_c0_g1_i4_orf1	transmembrane protease serine 9 [Ostrinia furnacalis]	3	539	58.5	8.56	10	4	3	High	1	0.916	1.065	1.112	1.194	1.091	1.206	1.238	1.119	1.21	1.518	1.126	2.232	2.175	1.966
TRINITY_DN19830_c0_g1_i1_orf1	macrophage migration inhibitory factor-like [Ostrinia furnacalis]	4	120	13.2	6.62	27	5	4	High	1	1.017	1.017	0.692	0.739	0.717	0.745	0.772	0.665	0.758	0.727	0.863	0.495	0.522	0.525
TRINITY_DN29743_c0_g1_i9_orf1	polyadenylate-binding protein 2 isoform X1 [Ostrinia furnacalis]	3	225	24.9	6.57	19	3	3	High	1	0.944	0.937	0.775	0.73	0.806	0.707	0.676	0.684	0.702	0.618	0.641	0.931	0.922	0.875
TRINITY_DN11060_c0_g1_i6_orf1	extracellular matrix protein A-like isoform X3 [Ostrinia furnacalis]	3	1073	105.7	7.05	4	3	3	High	1	0.86	0.936	2.498	2.628	2.312	1.889	1.655	1.714	1.719	1.643	1.792	2.477	2.535	2.371
TRINITY_DN139326_c0_g1_i1_orf1	40S ribosomal protein S13 [Gallus gallus]	3	150	17.1	10.54	17	7	1	High	1	0.931	0.971	0.879	0.871	0.93	0.875	0.804	0.841	0.806	0.68	0.836	0.743	0.75	0.767
TRINITY_DN3856_c0_g1_i7_orf1	uncharacterized protein LOC114355702 [Ostrinia furnacalis]	4	753	81.5	5.14	7	4	4	High	1	1.067	0.986	0.675	0.613	0.74	0.692	0.707	0.657	0.658	0.572	0.58	0.828	0.825	0.817
TRINITY_DN49785_c1_g1_i3_orf1	uncharacterized protein LOC114365444, partial [Ostrinia furnacalis]	1	237	26.6	7.34	6	2	1	High	1	1.003	1.095	1.206	1.241	1.292	1.265	1.213	1.246	1.098	0.907	1.004	4.168	4.359	4.242
TRINITY_DN28626_c0_g1_i5_orf1	3-ketodihydrosphingosine reductase [Ostrinia furnacalis]	3	327	35.3	8.51	10	4	3	High	1	1.174	1.091	1.256	1.175	1.036	1.04	0.898	0.931	1.188	1.097	1.348	1.13	1.071	0.955
TRINITY_DN2224_c0_g1_i1_orf1	serine--tRNA ligase, cytoplasmic [Ostrinia furnacalis]	4	261	29.2	6.19	17	4	4	High	1	0.975	1.045	0.769	0.903	0.878	0.729	0.867	0.719	0.732	0.683	0.763	0.574	0.595	0.59
TRINITY_DN31417_c0_g1_i3_orf1	titin-like [Ostrinia furnacalis]	2	233	25.3	5.31	17	2	2	High	1	0.811	0.836	1.157	1.252	1.186	1.164	1.321	1.326	1.311	1.325	1.237	3.336	3.149	2.682
TRINITY_DN108200_c0_g1_i1_orf1	uncharacterized protein LOC114350842 [Ostrinia furnacalis]	3	249	26	8	16	3	3	High	1	0.929	1.03	0.485	0.47	0.518	0.486	0.575	0.527	0.478	0.467	0.526	0.396	0.454	0.451
TRINITY_DN35633_c0_g2_i1_orf1	uncharacterized protein LOC114353024 [Ostrinia furnacalis]	4	411	45.2	5.73	12	5	4	High	1	1.076	1.096	0.644	0.665	0.708	0.678	0.707	0.714	0.697	0.756	0.705	1.419	1.478	1.456
TRINITY_DN19746_c0_g1_i5_orf1	hypothetical protein evm_004480 [Chilo suppressalis]	3	127	14.3	6.79	17	4	3	High	1	0.916	0.899	0.902	1.023	0.989	1.042	1.145	1.06	0.894	0.932	1.069	1.262	1.308	1.157
TRINITY_DN20185_c0_g1_i6_orf1	zinc finger protein on ecdysone puffs [Ostrinia furnacalis]	5	581	65.7	7.72	10	5	5	High	1	0.987	0.96	0.762	0.736	0.713	0.61	0.648	0.705	0.66	0.631	0.668	0.981	0.902	0.846
TRINITY_DN5442_c0_g1_i4_orf1	hypothetical protein evm_004688 [Chilo suppressalis]	6	400	45.5	8.62	11	8	6	High	1	1.053	1.005	0.658	0.695	0.788	0.676	0.679	0.672	0.719	0.604	0.772	0.942	0.959	0.891
TRINITY_DN12476_c0_g1_i4_orf1	guanine nucleotide-binding protein-like 3 homolog [Ostrinia furnacalis]	4	577	64.8	9.6	7	4	4	High	1	1.043	1.023	0.428	0.433	0.498	0.481	0.664	0.547	0.766	0.634	0.449	0.474	0.383	0.444
TRINITY_DN8717_c0_g1_i5_orf1	hypothetical protein evm_006607 [Chilo suppressalis]	4	188	19.6	11.08	24	4	4	High	1	1.01	0.963	0.657	0.635	0.721	0.736	0.857	0.828	0.678	0.658	0.736	0.63	0.659	0.657
TRINITY_DN578_c0_g1_i5_orf1	charged multivesicular body protein 7 [Ostrinia furnacalis]	2	385	43.1	5.35	10	2	1	High	1	1.173	1.282	1.873	2.08	2.229	1.981	2.017	2.088	2.592	2.269	2.655	2.445	2.455	2.585
TRINITY_DN1012_c0_g1_i2_orf1	teneurin-a isoform X1 [Ostrinia furnacalis]	4	2024	222.3	6.4	2	4	4	High	1	1.161	1.053	2.203	2.234	1.825	2.519	2.531	2.123	1.345	1.396	1.678	3.117	3.55	3.514
TRINITY_DN97472_c0_g1_i5_orf1	microtubule-actin cross-linking factor 1 isoform X15 [Ostrinia furnacalis]	3	154	17.9	8.85	19	6	1	High	1	1.007	0.982	1.42	1.659	1.549	1.437	1.381	1.276	1.383	1.251	1.427	0.877	0.983	0.888
TRINITY_DN4257_c0_g1_i2_orf1	dynactin subunit 1 [Ostrinia furnacalis]	4	894	99.4	5.31	6	4	4	High	1	0.967	0.998	0.935	1.007	0.929	1.058	0.988	0.982	0.817	0.833	0.749	1.053	0.959	0.948
TRINITY_DN20793_c0_g2_i1_orf1	mucin-2-like [Ostrinia furnacalis]	4	481	52.5	9.25	9	5	4	High	1	1.087	1.027	0.787	0.748	0.724	0.712	0.707	0.673	0.769	0.776	0.655	3.644	3.632	3.444
TRINITY_DN50725_c0_g1_i6_orf1	BTB/POZ domain-containing protein 2-like [Ostrinia furnacalis]	3	403	45.4	6.07	9	4	3	High	1	1.029	1.015	2.846	2.556	2.007	0.736	0.867	0.779	0.92	0.979	0.942	0.932	0.833	0.91
TRINITY_DN492_c0_g1_i4_orf1	hypothetical protein evm_000589 [Chilo suppressalis]	4	793	86.5	5.43	5	4	4	High	1	1	0.924	0.56	0.531	0.658	0.565	0.485	0.518	0.555	0.599	0.44	0.601	0.534	0.488
TRINITY_DN2043_c0_g1_i3_orf1	phenoloxidase-activating factor 2-like [Ostrinia furnacalis]	3	389	41.6	6.99	9	4	3	High	1	0.966	0.928	0.954	0.953	0.98	1.001	0.956	1.001	0.909	0.887	0.842	1.462	1.408	1.402
TRINITY_DN97097_c0_g1_i4_orf1	plectin-like, partial [Ostrinia furnacalis]	3	118	13.9	5.6	24	5	3	High	1	1.039	1.118	1.266	1.289	1.425	1.478	1.452	1.398	1.577	1.399	1.702	2.018	2.289	2.224
TRINITY_DN920_c0_g1_i6_orf1	glutathione S-transferase omega 2 [Ostrinia furnacalis]	3	282	32.3	5.76	11	5	1	High	1	1.219	1.143	0.656	0.649	0.671	1.69	1.635	1.532	1.036	0.873	0.936	0.65	0.591	0.637
TRINITY_DN10520_c0_g1_i2_orf1	probable 39S ribosomal protein L45, mitochondrial [Ostrinia furnacalis]	4	259	30	9.79	16	5	4	High	1	1	1.139	0.488	0.421	0.496	0.417	0.446	0.457	0.454	0.499	0.416	0.517	0.524	0.486
TRINITY_DN10455_c0_g2_i1_orf1	ubiquitin fusion degradation protein 1 homolog [Ostrinia furnacalis]	4	305	34.3	5.48	16	5	4	High	1	0.949	1.034	1.277	1.199	1.206	1.193	1.228	1.296	1.164	1.409	1.188	1.193	1.091	1.262
TRINITY_DN2238_c0_g2_i1_orf1	mitochondrial import inner membrane translocase subunit Tim8 [Ostrinia furna	4	90	10.2	5	44	6	4	High	1	1.01	0.899	0.363	0.357	0.408	0.41	0.422	0.406	0.435	0.546	0.442	0.438	0.449	0.449
TRINITY_DN18391_c0_g2_i8_orf1	protein suppressor of forked [Helicoverpa zea]	5	728	83.8	7.87	6	5	5	High	1	0.988	1.04	0.987	0.822	0.853	0.843	0.888	0.922	0.912	0.905	0.963	1.071	1.13	1.092
TRINITY_DN3292_c2_g1_i4_orf1	ribosome biogenesis regulatory protein homolog [Ostrinia furnacalis]	4	352	40	10.08	12	6	4	High	1	0.951	0.988	0.381	0.379	0.378	0.47	0.474	0.458	0.467	0.453	0.442	0.455	0.402	0.398
TRINITY_DN100479_c0_g1_i3_orf1	transmembrane emp24 domain-containing protein eca [Ostrinia furnacalis]	3	217	25.2	6.73	19	4	3	High	1	0.917	0.969	0.982	0.934	0.848	0.873	0.785	0.901	0.804	0.812	0.837	0.994	0.912	0.958
TRINITY_DN3562_c0_g1_i4_orf1	peroxisomal membrane protein PEX14-like isoform X2 [Ostrinia furnacalis]	3	248	28	8.06	13	4	3	High	1	1.039	1.003	0.719	0.666	0.736	0.745	0.733	0.703	0.869	0.799	0.794	0.797	0.797	0.733
TRINITY_DN54477_c0_g1_i1_orf1	rho-associated protein kinase 2 isoform X11 [Ostrinia furnacalis]	4	781	88.8	6.14	6	4	4	High	1	1.032	1.055	1.032	1.058	0.942	0.899	0.967	0.927	0.792	0.755	0.824	0.969	0.922	0.92
TRINITY_DN42337_c0_g1_i5_orf1	hypothetical protein evm_002829 [Chilo suppressalis]	4	165	17.6	6.8	31	4	4	High	1	0.915	0.995	0.801	0.987	0.754	1.023	0.869	1.102	0.853	0.666	1.084	2.975	3.124	2.637
TRINITY_DN14754_c0_g1_i6_orf1	cathepsin L [Papilio xuthus]	3	113	12.8	8.28	41	4	1	High	1	0.685	0.747	6.27	5.26	5.183	2.306	2.174	2.09	1.274	1.045	1.5	0.569	0.567	0.795
TRINITY_DN48590_c0_g1_i1_orf1	acyl-CoA Delta(11) desaturase isoform X1 [Ostrinia furnacalis]	4	324	36.9	7.58	14	4	4	High	1	0.829	1.02	0.584	0.564	0.555	0.589	0.552	0.597	0.528	0.538	0.533	2.331	2.426	2.226
TRINITY_DN13923_c0_g2_i1_orf1	sideroflexin-2 [Zerene cesonia]	2	327	36.9	9.58	9	3	2	High	1	0.934	0.998	0.727	0.676	0.681	0.746	0.66	0.679	0.899	1.029	0.833	0.837	0.727	0.796
TRINITY_DN1494_c0_g2_i1_orf1	dihydropyrimidine dehydrogenase [NADP(+)] [Ostrinia furnacalis]	4	325	35.2	8.68	11	4	4	High	1	1.033	0.973	0.662	0.636	0.721	0.715	0.597	0.629	0.693	0.677	0.643	0.711	0.671	0.713
TRINITY_DN4725_c0_g1_i4_orf1	uncharacterized protein LOC114354375 [Ostrinia furnacalis]	5	423	49.1	5.69	15	5	5	High	1	0.982	0.996	0.474	0.461	0.475	0.522	0.542	0.541	0.531	0.583	0.519	0.551	0.53	0.576
TRINITY_DN61222_c0_g1_i1_orf1	60S ribosomal protein L38 [Bicyclus anynana]	3	104	12.1	9.72	29	6	3	High	1	0.984	0.984	0.656	0.62	0.653	0.548	0.569	0.553	0.502	0.478	0.52	0.547	0.552	0.582
TRINITY_DN84883_c0_g1_i1_orf1	protein yellow [Ostrinia furnacalis]	3	465	52.5	6.46	9	3	3	High	1	0.972	0.944	0.884	0.888	0.964	0.958	0.913	0.787	0.932	0.939	0.876	1.092	1.062	1.061
TRINITY_DN75746_c0_g1_i1_orfp1	TRINITY_DN75746_c0_g1_i1_m.54871 TRINITY_DN75746_c0_g1::TRINITY_DN75	2	82	9.6	7.61	29	4	2	High	1	1.294	1.174	0.225	0.213	0.278	0.266	0.306	0.269	0.286	0.244	0.261	0.279	0.288	0.271
TRINITY_DN13384_c0_g1_i1_orf1	26S proteasome regulatory subunit S3 [Aphelinus abdominalis]	4	498	57	8.41	7	4	1	High	1	0.982	1.026	0.922	0.854	0.918	0.831	0.982	0.992	0.84	0.759	0.751	0.888	1.005	0.956
TRINITY_DN1252_c0_g1_i3_orf1	unnamed protein product [Chilo suppressalis]	3	895	98.6	6.62	4	3	2	High	1	0.975	1.258	1.041	1.339	1.179	0.901	0.917	1.025	1.22	1.101	1.144	1.943	2.068	2.037
TRINITY_DN6014_c1_g1_i2_orf1	putative hydroxypyruvate isomerase [Ostrinia furnacalis]	4	260	29.1	7.47	18	5	4	High	1	0.966	0.956	0.988	0.968	0.973	1.027	1.16	1.014	0.941	1.033	0.967	1.367	1.27	1.376
TRINITY_DN1393_c0_g1_i2_orf1	uncharacterized protein LOC114357114 isoform X1 [Ostrinia furnacalis]	4	1121	130.4	7.55	5	4	4	High	1	0.965	0.966	0.985	1.116	1.12	1.269	1.136	1.227	0.99	1.041	1.117	1.443	1.254	1.392
TRINITY_DN11159_c0_g1_i5_orf1	sphingosine-1-phosphate lyase isoform X2 [Ostrinia furnacalis]	3	52	5.8	4.26	65	3	3	High	1	0.946	0.929	0.96	0.915	0.928	1.086	1.204	1.097	1.229	1.177	1.287	1.174	1.191	1.267
TRINITY_DN3029_c1_g2_i1_orf1	unnamed protein product [Plutella xylostella]	3	204	22.5	9.23	13	3	3	High	1	1.035	1.219	1.982	2.25	1.942	1.604	1.445	1.529	1.677	1.622	1.619	1.327	1.467	1.368
TRINITY_DN5296_c0_g2_i1_orf1	RNA-binding protein Rsf1 [Ostrinia furnacalis]	3	159	16.5	10.05	19	4	3	High	1	0.964	1.075	1.033	1.285	1.026	0.826	1.034	0.873	0.762	0.856	0.99	1.284	1.056	1.286
TRINITY_DN2070_c1_g1_i1_orf1	mitochondrial carrier homolog 2-like [Helicoverpa zea]	3	295	33.1	8.62	12	3	3	High	1	0.95	1.086	0.563	0.485	0.543	0.499	0.502	0.511	0.465	0.564	0.44	0.491	0.462	0.509
TRINITY_DN27321_c0_g1_i1_orf1	ras-related protein Rap-2c [Bicyclus anynana]	4	181	20.3	5.43	20	4	4	High	1	0.95	0.941	0.774	0.769	0.853	0.827	0.783	0.826	0.694	0.614	0.76	1.319	1.301	1.268
TRINITY_DN4785_c0_g2_i1_orf1	methylthioribose-1-phosphate isomerase [Ostrinia furnacalis]	4	351	38.5	6.8	10	6	4	High	1	1.011	0.987	0.931	0.987	1.043	1.093	1.253	1.122	1.017	0.932	1.058	1.336	1.412	1.225
TRINITY_DN71863_c0_g1_i2_orf1	unnamed protein product [Diatraea saccharalis]	3	155	17.8	9.54	25	3	3	High	1	1.02	1.032	1.894	1.899	1.831	2.383	2.326	2.261	2.787	2.987	2.481	2.326	2.175	2.314
TRINITY_DN2668_c0_g1_i6_orf1	pancreatic triacylglycerol lipase-like [Spodoptera litura]	3	313	34.7	8.62	11	3	1	High	1	1.042	0.934	0.662	0.417	0.578	0.448	0.642	0.675	0.51	0.686	0.639	0.724	0.551	0.659
TRINITY_DN8908_c0_g1_i1_orf1	unnamed protein product [Spodoptera littoralis]	3	337	37.5	8.15	12	3	3	High	1	1.024	0.924	0.808	0.831	0.818	0.639	0.734	0.753	0.695	0.781	0.659	0.681	0.635	0.692
TRINITY_DN2743_c0_g1_i5_orf1	regucalcin-like [Ostrinia furnacalis]	2	89	10	6.28	29	2	2	High	1	0.813	0.924	1.573	1.584	1.624	1.961	3.553	2.379	1.371	1.359	1.184	1.507	1.546	1.656
TRINITY_DN2376_c0_g1_i6_orf1	dmX-like protein 2 isoform X3 [Pectinophora gossypiella]	5	3184	350.5	6.35	2	6	5	High	1	0.987	0.994	0.977	1.011	0.97	0.945	1.001	0.959	0.846	0.991	0.929	0.958	0.866	0.905
TRINITY_DN26824_c0_g1_i1_orf1	60S ribosomal protein L6 [Hyposmocoma kahamanoa]	3	81	8.6	10.64	38	5	2	High	1	1.027	1.023	0.645	0.763	0.701	0.774	0.831	0.735	0.456	0.388	0.553	0.591	0.656	0.605
TRINITY_DN40586_c0_g1_i4_orf1	inosine triphosphate pyrophosphatase [Ostrinia furnacalis]	3	190	21.7	7.15	17	3	3	High	1	1.016	0.975	0.763	0.804	0.81	0.882	0.877	0.779	0.852	0.783	0.853	0.752	0.865	0.848
TRINITY_DN3037_c0_g1_i1_orf1	coiled-coil domain-containing protein 58-like [Spodoptera frugiperda]	3	136	15.7	7.74	32	3	3	High	1	0.963	0.903	0.825	0.92	0.868	0.871	0.902	0.9	1.055	1.033	1.062	0.742	0.669	0.771
TRINITY_DN10824_c0_g1_i3_orf1	endochitinase isoform X2 [Ostrinia furnacalis]	4	562	62.8	5.47	9	4	4	High	1	0.914	1.137	1.525	1.592	1.539	1.101	1.121	1.229	1.09	1.093	1.128	7.558	7.789	7.698
TRINITY_DN13233_c0_g1_i3_orf1	60S ribosomal protein L31 [Galleria mellonella]	4	124	14.3	10.58	32	6	4	High	1	1.019	1.049	0.801	0.823	0.782	0.686	0.653	0.683	0.615	0.592	0.697	0.615	0.62	0.582
TRINITY_DN3179_c0_g1_i1_orf1	diamine acetyltransferase 2-like [Ostrinia furnacalis]	3	173	19.4	6.43	20	3	3	High	1	1.037	1.088	0.761	0.781	0.79	0.656	0.709	0.811	0.727	0.678	0.659	0.952	0.898	0.894
TRINITY_DN42759_c0_g2_i1_orf1	fatty acid synthase-like [Ostrinia furnacalis]	5	1474	166.4	6.93	3	5	5	High	1	1.015	0.924	0.281	0.273	0.298	0.375	0.461	0.389	0.337	0.335	0.317	0.347	0.324	0.344
TRINITY_DN4501_c0_g1_i3_orf1	methylcrotonoyl-CoA carboxylase subunit alpha, mitochondrial [Ostrinia furna	2	147	15.5	5.01	18	2	1	High	1	1.003	0.931	0.783	0.75	0.73	0.672	0.651	0.744	0.668	0.528	0.53	0.423	0.409	0.351
TRINITY_DN29879_c0_g1_i3_orf1	uncharacterized protein LOC114350556 isoform X1 [Ostrinia furnacalis]	3	321	36.4	9.23	9	3	3	High	1	1.098	1.057	0.946	0.978	0.988	1.376	1.364	1.33	0.958	0.858	1.051	1.759	1.795	1.76
TRINITY_DN3512_c0_g1_i2_orf1	probable tRNA (guanine(26)-N(2))-dimethyltransferase [Ostrinia furnacalis]	2	463	51.5	8.41	6	2	2	High	1	0.885	1.099	0.739	0.918	0.898	0.792	1.043	0.994	0.781	1.042	0.959	1.001	0.944	0.857
TRINITY_DN57918_c0_g1_i1_orf1	PREDICTED: serine--tRNA ligase, cytoplasmic [Fopius arisanus]	3	494	56.4	6.38	7	3	3	High	1	0.97	0.986	0.837	0.75	0.811	0.689	0.696	0.751	0.659	0.706	0.662	0.513	0.48	0.485
TRINITY_DN52788_c0_g1_i1_orf1	putative fatty acyl-CoA reductase CG5065 isoform X1 [Ostrinia furnacalis]	5	523	60	7.43	12	5	5	High	1	1.016	0.997	1.017	1.042	0.992	0.993	0.959	1.001	1.017	1.021	0.954	1.941	1.805	1.994
TRINITY_DN8691_c0_g1_i3_orf1	nucleolin-like [Melitaea cinxia]	2	136	14.4	4.58	15	3	2	High	1	1.036	0.993	0.259	0.309	0.313	0.222	0.195	0.198	0.255	0.196	0.212	0.54	0.496	0.462
TRINITY_DN47304_c0_g1_i3_orf1	ubiquitin-fold modifier 1 isoform X2 [Ostrinia furnacalis]	3	116	13	9.58	44	4	3	High	1	0.917	0.944	0.985	1.004	0.959	1.067	1.002	0.98	1.036	1.237	1.075	1.114	1.083	1.136
TRINITY_DN2207_c0_g1_i4_orf1	methionine-R-sulfoxide reductase B1 isoform X4 [Pectinophora gossypiella]	2	48	5.3	8.35	67	3	1	High	1	0.987	0.892	1.355	1.468	1.451	2.114	2.277	2.17	1.478	1.236	1.494	0.669	0.662	0.825
TRINITY_DN26149_c0_g1_i5_orf1	thymosin beta isoform X3 [Ostrinia furnacalis]	3	93	10.4	5.22	35	8	1	High	1	1.031	0.96	1.521	1.544	1.542	1.847	1.738	1.735	1.655	1.79	1.685	3.434	3.414	3.372
TRINITY_DN3062_c0_g1_i1_orf1	HEAT repeat-containing protein 1 [Ostrinia furnacalis]	4	2102	240.8	6.28	2	4	4	High	1	1.007	1.02	0.337	0.343	0.407	0.321	0.361	0.326	0.262	0.277	0.293	0.444	0.411	0.385
TRINITY_DN7938_c0_g1_i3_orf1	protein halfway isoform X1 [Ostrinia furnacalis]	3	511	58	6.64	8	3	3	High	1	0.993	1.022	1.494	1.453	1.463	1.582	1.582	1.467	1.278	1.446	1.515	1.614	1.51	1.508
TRINITY_DN38482_c0_g1_i4_orf1	pyruvate dehydrogenase (acetyl-transferring) kinase, mitochondrial [Ostrinia fu	3	419	47.1	6.95	9	3	3	High	1	0.991	0.904	0.914	0.969	0.985	0.899	0.983	1.092	1.036	1.266	1.039	0.808	0.784	0.892
TRINITY_DN4767_c0_g1_i4_orf1	cysteine protease XCP2-like [Ostrinia furnacalis]	6	100	11.7	7.12	38	11	6	High	1	0.912	0.99	4.639	4.937	4.676	4.682	4.662	4.429	6.492	5.274	7.009	6.643	7.051	6.247
TRINITY_DN15607_c0_g1_i6_orf1	protein artichoke-like [Ostrinia furnacalis]	5	957	109	6.61	6	5	5	High	1	1.008	0.965	0.473	0.494	0.518	0.407	0.397	0.385	0.373	0.374	0.37	0.402	0.418	0.43
TRINITY_DN24932_c0_g1_i1_orf1	PREDICTED: neurocalcin homolog [Microplitis demolitor]	3	192	22	5.25	18	4	3	High	1	0.963	0.966	0.903	0.808	0.839	0.879	0.9	0.893	0.835	0.776	0.873	1.008	1.049	0.913
TRINITY_DN400_c0_g1_i1_orf1	serine/threonine-protein phosphatase 2A activator-like isoform X2 [Ostrinia fu	4	333	38.2	6.65	15	4	4	High	1	0.91	0.985	1.483	1.385	1.442	1.491	1.569	1.525	1.622	1.531	1.582	1.303	1.283	1.428
TRINITY_DN3058_c0_g1_i1_orf1	mesencephalic astrocyte-derived neurotrophic factor homolog [Ostrinia furnac	4	173	19.9	8.19	18	5	4	High	1	0.981	1.053	1.205	1.263	1.193	0.818	0.881	0.86	1.081	0.952	1.27	0.843	0.794	0.914
TRINITY_DN286_c0_g1_i2_orf1	uncharacterized protein LOC114361329 [Ostrinia furnacalis]	4	1329	150.3	6.79	3	4	4	High	1	1.021	0.991	0.579	0.574	0.702	0.698	0.74	0.722	0.69	0.718	0.676	1.418	1.452	1.378
TRINITY_DN114960_c0_g1_i4_orf1	zinc finger protein on ecdysone puffs-like [Ostrinia furnacalis]	3	82	9.4	5.45	38	5	3	High	1	0.992	0.995	1.074	1.214	1.087	0.989	1.083	1.201	1.031	0.937	1.154	1.491	1.576	1.611
TRINITY_DN14944_c0_g1_i9_orf1	casein kinase I isoform X1 [Ostrinia furnacalis]	2	485	54.5	9.2	7	3	2	High	1	1.346	1.257	1.598	1.185	1.385	1.363	1.51	1.624	1.402	1.402	1.01	2.128	2.077	1.996
TRINITY_DN6535_c0_g2_i1_orf1	NADH dehydrogenase [ubiquinone] 1 beta subcomplex subunit 3 [Ostrinia furi	4	99	11	9.13	39	4	4	High	1	1.006	0.948	0.401	0.456	0.486	0.429	0.421	0.376	0.424	0.413	0.447	0.489	0.495	0.48
TRINITY_DN2953_c1_g1_i2_orf1	methionine--tRNA ligase, cytoplasmic isoform X6 [Ostrinia furnacalis]	4	97	10.3	9.2	43	4	1	High	1	1.183	1.062	0.174	0.065	0.22	0.195	0.249	0.163	0.215	0.183	0.183	0.378	0.437	0.385

TRINITY_DN418_c1_g1_i3_orf1	hypothetical protein evm_003996 [Chilo suppressalis]	3	76	8.8	6.51	46	4	1	High	1	0.995	1.123	2.696	3.098	2.759	2.188	1.988	1.87	4.661	3.851	4.931	3.283	3.265	4.051
TRINITY_DN48460_c0_g1_i1_orf1	PREDICTED: signal recognition particle 54 kDa protein [Fopius arisanus]	4	504	55.5	8.76	6	6	4	High	1	1.021	1	0.82	0.848	0.863	0.838	0.813	0.803	0.758	0.664	0.752	0.923	0.955	0.927
TRINITY_DN84938_c0_g1_i4_orf1	vinculin-like isoform X2 [Ostrinia furnacalis]	3	145	15.7	5.38	23	4	1	High	1	1.239	1.379	2.88	2.629	3.057	2.163	2.305	2.409	1.677	1.606	1.204	1.082	1.299	1.877
TRINITY_DN3715_c0_g1_i2_orf1	uncharacterized protein LOC114356437 isoform X1 [Ostrinia furnacalis]	3	171	19.1	6.05	19	4	3	High	1	1.018	1.061	0.752	0.695	0.717	0.869	0.888	0.91	1.384	1.175	1.128	2.845	3.143	2.77
TRINITY_DN14477_c0_g1_i12_orf1	PREDICTED: phosphoribosyl pyrophosphate synthase-associated protein 2 isof	3	377	41.8	8.05	10	3	3	High	1	0.962	1.006	0.7	0.705	0.739	0.788	0.776	0.759	0.744	0.887	0.698	0.992	0.98	1.018
TRINITY_DN69236_c0_g1_i1_orf1	peroxiredoxin [Ostrinia furnacalis]	3	246	27.8	6.54	14	4	2	High	1	0.996	1.003	0.489	0.476	0.461	0.558	0.479	0.538	0.376	0.329	0.354	0.794	0.781	0.735
TRINITY_DN610_c0_g1_i1_orf1	CAP-Gly domain-containing linker protein 1 isoform X10 [Ostrinia furnacalis]	3	356	37.8	10.33	10	3	3	High	1	0.915	0.912	1.652	1.569	1.672	1.305	1.438	1.504	1.703	1.68	1.581	0.911	0.868	1.101
TRINITY_DN3598_c0_g1_i1_orf1	esterase FE4-like [Ostrinia furnacalis]	5	543	61.6	7.33	10	6	4	High	1	1.007	1.008	0.36	0.36	0.417	0.392	0.451	0.436	0.404	0.455	0.413	0.379	0.369	0.389
TRINITY_DN2758_c0_g1_i7_orf1	hypothetical protein evm_012952 [Chilo suppressalis]	3	174	19.4	5.95	26	3	1	High	1	0.908	0.958	1.182	1.151	1.132	1.431	1.279	1.358	1.049	0.948	1.129	1.259	1.35	1.164
TRINITY_DN10290_c0_g1_i7_orf1	aquaporin AQPAe.a [Ostrinia furnacalis]	1	269	27	6.77	6	7	1	High	1	0.979	0.968	1.228	1.191	1.136	1.096	1.136	1.132	1.328	1.321	1.22	1.388	1.433	1.517
TRINITY_DN8406_c0_g1_i4_orf1	titin [Ostrinia furnacalis]	3	356	39.2	9.58	11	3	1	High	1	0.968	1.043	0.862	1.059	1.1	0.804	0.848	0.845	0.833	0.711	0.914	0.704	0.687	0.64
TRINITY_DN26195_c0_g1_i6_orf1	bifunctional coenzyme A synthase isoform X1 [Ostrinia furnacalis]	5	540	61.1	7.75	11	5	5	High	1	0.981	1.034	1.002	0.977	0.989	0.946	1.072	0.958	0.966	1.169	1.016	0.866	0.82	0.895
TRINITY_DN20499_c0_g1_i1_orf1	exosome RNA helicase MTR4 isoform X2 [Ostrinia furnacalis]	3	685	77.7	6.05	5	3	2	High	1	1.057	0.973	0.622	0.672	0.668	0.682	0.624	0.646	0.643	0.689	0.6	0.65	0.683	0.706
TRINITY_DN5433_c0_g1_i5_orf1	uncharacterized protein LOC114351067 [Ostrinia furnacalis]	4	304	34.7	5.25	14	4	4	High	1	0.943	0.943	1.441	1.556	1.62	1.24	1.225	1.192	1.575	1.468	1.578	1.37	1.498	1.361
TRINITY_DN48554_c0_g1_i1_orf1	39S ribosomal protein L39, mitochondrial [Ostrinia furnacalis]	3	340	38.6	8.29	10	3	3	High	1	0.982	0.997	0.587	0.537	0.581	0.535	0.481	0.538	0.593	0.533	0.54	0.534	0.534	0.547
TRINITY_DN8824_c0_g2_i1_orf1	60S ribosomal protein L34-like [Ostrinia furnacalis]	5	119	13.9	11.34	29	7	5	High	1	0.991	1.044	0.696	0.662	0.733	0.686	0.623	0.66	0.549	0.473	0.501	0.566	0.569	0.565
TRINITY_DN5525_c0_g1_i4_orf1	probable glucosamine 6-phosphate N-acetyltransferase [Ostrinia furnacalis]	4	199	22.5	8.7	23	7	4	High	1	0.956	0.989	0.745	0.774	0.734	0.768	0.694	0.731	0.8	0.77	0.781	0.874	0.877	0.885
TRINITY_DN2372_c0_g1_i5_orf1	ATP-binding cassette sub-family B member 7, mitochondrial isoform X1 [Ostri	3	697	76	9.42	6	3	3	High	1	0.938	0.984	1.135	1.134	1.069	1.034	1.084	1.054	1.102	1.067	1.047	1.031	1.08	1.102
TRINITY_DN33488_c0_g1_i2_orf1	semaphorin-1A isoform X3 [Trichoplusia ni]	5	752	83.1	6.67	7	5	5	High	1	0.961	0.948	1.984	2.191	1.999	1.841	1.87	1.881	1.87	2.052	1.865	1.438	1.303	1.461
TRINITY_DN19115_c0_g1_i1_orf1	putative ATP synthase subunit f, mitochondrial [Ostrinia furnacalis]	4	107	12.6	9.86	34	4	4	High	1	0.824	0.902	0.526	0.582	0.514	0.433	0.495	0.461	0.442	0.62	0.581	0.548	0.564	0.648
TRINITY_DN43431_c0_g1_i1_orf1	glycine dehydrogenase (decarboxylating), mitochondrial isoform X1 [Ostrinia f	2	84	9.7	8.53	42	2	2	High	1	0.993	1.063	1.846	1.914	1.52	1.609	1.461	1.431	1.464	1.454	1.641	1.949	2.08	1.987
TRINITY_DN14922_c0_g3_i2_orf1	probable pseudouridine-5'-phosphatase [Ostrinia furnacalis]	3	228	25.8	4.92	19	4	3	High	1	0.941	0.948	1.465	1.609	1.491	1.34	1.283	1.269	1.32	1.288	1.23	0.665	0.728	0.715
TRINITY_DN23432_c0_g1_i1_orf1	7-methylguanosine phosphate-specific 5'-nucleotidase-like isoform X2 [Ostrir	3	296	33.5	5.64	11	3	3	High	1	1.069	1.043	0.751	0.824	0.782	0.722	0.82	0.772	0.773	0.788	0.657	0.771	0.747	0.702
TRINITY_DN17845_c0_g1_i3_orf1	ataxin-10-like [Ostrinia furnacalis]	4	801	89.6	4.98	7	4	4	High	1	0.967	1.057	1.585	1.688	1.469	1.315	1.417	1.325	1.311	1.182	1.457	1.201	1.165	1.257
TRINITY_DN25916_c0_g1_i1_orf1	uncharacterized protein LOC125063950 [Vanessa atalanta]	2	66	7.7	4.54	30	7	2	High	1	0.963	0.963	0.875	0.887	0.87	0.681	0.655	0.667	0.575	0.571	0.611	0.871	0.81	0.819
TRINITY_DN21215_c0_g1_i7_orf1	phytanoyl-CoA dioxygenase, peroxisomal-like [Ostrinia furnacalis]	3	304	34.7	8.28	12	3	3	High	1	0.956	0.955	1.003	0.894	0.942	0.779	0.82	0.898	0.579	0.538	0.591	0.74	0.664	0.665
TRINITY_DN61786_c0_g1_i1_orf1	PREDICTED: ras-related protein Rab-10 [Fopius arisanus]	3	200	22.9	7.37	15	5	1	High	1	0.849	0.788	0.809	0.576	0.876	0.788	0.792	0.796	0.773	0.751	0.527	0.983	0.853	0.917
TRINITY_DN19810_c1_g1_i7_orf1	RNA-binding protein spenito [Ostrinia furnacalis]	3	750	85.4	9.5	5	3	3	High	1	1.043	0.926	0.724	0.777	0.867	0.737	0.83	0.668	0.588	0.677	0.616	1.031	1.038	0.98
TRINITY_DN2343_c1_g1_i8_orf1	receptor expression-enhancing protein 5-like isoform X3 [Ostrinia furnacalis]	5	192	22.4	7.75	21	5	1	High	1	1.095	0.998	0.134	0.137	0.212	0.217	0.231	0.252	0.197	0.177	0.195	0.169	0.155	0.186
TRINITY_DN2529_c0_g1_i3_orf1	collagen alpha-1(X) chain-like [Ostrinia furnacalis]	3	829	76.9	6.77	5	5	3	High	1	1.045	0.892	1.166	1.287	1.341	1.446	1.399	1.272	1.289	1.102	1.213	1.031	1.003	1.013
TRINITY_DN1073_c0_g1_i3_orf1	carboxylesterase [Loxostege sticticalis]	5	615	68.1	4.56	7	9	3	High	1	1.193	1.078	0.995	1.044	0.955	1.362	1.169	1.183	1.103	0.952	1.248	0.44	0.393	0.518
TRINITY_DN52761_c0_g1_i2_orf1	atlastin isoform X4 [Ostrinia furnacalis]	2	108	12.4	7.44	31	2	2	High	1	0.982	0.981	2.325	2.135	2.325	2.438	2.372	2.176	1.475	1.688	1.355	1.705	1.812	1.744
TRINITY_DN2456_c0_g1_i2_orf1	glycerol-3-phosphate phosphatase isoform X1 [Ostrinia furnacalis]	3	348	38.2	8.4	13	3	3	High	1	0.95	1.023	0.574	0.544	0.619	0.579	0.56	0.61	0.617	0.565	0.572	0.671	0.706	0.687
TRINITY_DN512_c1_g1_i4_orf1	protein argonaute-2 [Ostrinia furnacalis]	5	854	97.1	9.32	6	5	5	High	1	1.027	0.992	1.134	1.04	1.16	1.427	1.352	1.412	1.322	1.378	1.29	1.255	1.166	1.232
TRINITY_DN1285_c0_g2_i1_orf1	bifunctional 3'-phosphoadenosine 5'-phosphosulfate synthase isoform X3 [Ost	3	295	32.9	5.74	14	3	3	High	1	0.901	0.986	0.369	0.334	0.411	0.351	0.361	0.403	0.327	0.363	0.348	0.379	0.336	0.359
TRINITY_DN3893_c0_g2_i3_orf1	cleavage and polyadenylation specificity factor subunit CG7185 isoform X2 [Os	4	633	69.6	10.3	8	4	4	High	1	0.968	1.011	0.589	0.646	0.662	0.519	0.554	0.573	0.475	0.415	0.516	0.825	0.837	0.836
TRINITY_DN6004_c0_g1_i1_orf1	endocuticle structural glycoprotein ABD-4-like [Ostrinia furnacalis]	2	154	16.7	4.82	23	3	2	High	1	0.951	1.024	1.309	1.275	1.252	1.359	1.292	1.399	1.267	1.325	1.271	3.515	3.705	3.706
TRINITY_DN19092_c2_g1_i1_orf1	mitochondrial fission 1 protein isoform X1 [Ostrinia furnacalis]	4	149	16.8	8.57	30	6	4	High	1	1.115	1.07	1.583	1.183	1.534	1.477	1.305	1.488	1.588	1.494	1.219	1.205	1.196	1.213
TRINITY_DN38835_c0_g2_i1_orf1	sec61alpha [Papilio xuthus]	3	128	13.9	6.35	27	4	1	High	1	0.881	0.987	0.997	0.924	0.89	1.151	1.11	1.205	0.947	1.439	1.146	1.08	0.994	1.104
TRINITY_DN24318_c0_g1_i1_orf1	60S ribosomal protein L29 [Ostrinia furnacalis]	4	73	8.5	11.82	38	7	4	High	1	1.064	1.054	0.788	0.791	0.722	0.703	0.586	0.694	0.532	0.438	0.519	0.578	0.562	0.524
TRINITY_DN3017_c0_g1_i6_orf1	uncharacterized protein LOC114364875 isoform X1 [Ostrinia furnacalis]	2	670	73	6.24	5	2	2	High	1	1.001	0.924	1.618	1.571	1.325	1.471	1.263	1.339	1.332	1.226	1.997	1.989	2.489	1.566
TRINITY_DN661_c0_g2_i2_orf1	cuticle protein 7-like [Ostrinia furnacalis]	2	182	19.4	7.08	19	5	2	High	1	0.998	1.062	0.886	0.845	0.836	0.974	1.515	1.132	1.209	1.336	1.183	3.929	3.8	3.687
TRINITY_DN6309_c0_g1_i7_orf1	apolipoprotein D-like isoform X1 [Ostrinia furnacalis]	3	268	29.9	7.28	10	3	3	High	1	0.866	1.047	1.627	1.67	1.465	1.457	1.637	1.521	1.292	1.976	1.306	1.349	1.362	1.552
TRINITY_DN11204_c0_g1_i3_orf1	spermosin-like [Ostrinia furnacalis]	4	603	66.4	8.12	6	5	4	High	1	0.948	0.964	1.29	1.235	1.2	0.847	0.89	0.943	1.012	0.918	0.978	1.313	1.313	1.314
TRINITY_DN135780_c0_g1_i1_orf1	flotillin-1 isoform X1 [Pectinophora gossypiella]	3	113	12.8	5.71	27	3	2	High	1	0.906	0.902	3.304	3.328	3.321	3.418	3.526	3.16	2.521	2.291	2.841	2.184	2.147	2.178
TRINITY_DN1986_c0_g1_i1_orf1	serine protease inhibitor 77Ba-like [Ostrinia furnacalis]	3	395	44.7	5.78	8	3	3	High	1	0.995	1.007	1.497	1.351	1.454	1.357	1.542	1.557	1.552	1.253	1.503	2.507	2.66	2.478
TRINITY_DN13287_c0_g1_i5_orf1	dystrophin-like, partial [Ostrinia furnacalis]	2	691	77.2	6.7	5	2	2	High	1	1.08	1.06	0.86	0.789	0.822	0.95	0.905	1.011	0.881	0.696	0.835	0.988	0.99	1.014
TRINITY_DN4025_c0_g1_i1_orf1	unnamed protein product [Chilo suppressalis]	4	438	49.5	9.47	9	4	4	High	1	1.002	1.025	0.631	0.706	0.718	0.695	0.646	0.667	0.608	0.585	0.555	0.698	0.711	0.674
TRINITY_DN117844_c0_g1_i1_orf1	ATP-citrate synthase [Cotesia glomerata]	1	1095	120.7	7.17	1	3	1	High	1	0.957	0.961	1.602	1.714	1.664	1.767	1.865	1.814	1.804	1.683	1.947	1.048	1.031	1.067
TRINITY_DN20007_c0_g1_i1_orf1	hypothetical protein evm_011958 [Chilo suppressalis]	3	164	18.7	8.85	18	3	3	High	1	0.912	1.022	0.589	0.646	0.621	0.569	0.655	0.605	0.599	0.622	0.553	0.78	0.723	0.769
TRINITY_DN20527_c0_g1_i1_orf1	dihydrofolate reductase [Ostrinia furnacalis]	4	185	21	7.42	21	4	4	High	1	0.981	0.956	0.72	0.711	0.699	0.983	0.987	0.983	0.846	0.823	0.762	0.78	0.774	0.799
TRINITY_DN2343_c1_g1_i12_orf1	receptor expression-enhancing protein 5-like isoform X3 [Ostrinia furnacalis]	5	166	19.4	7.77	25	6	1	High	1	1.008	0.984	1.598	1.419	1.53	1.141	1.146	1.12	1.046	0.912	0.954	1.009	1.068	1.026
TRINITY_DN108_c0_g1_i1_orf1	cyclic GMP-AMP synthase-like [Ostrinia furnacalis]	4	523	60.6	9.23	9	4	4	High	1	0.996	1.087	1.189	1.103	1.159	1.386	1.365	1.376	1.334	1.385	1.186	1.206	1.144	1.186
TRINITY_DN50471_c0_g1_i4_orf1	hypothetical protein evm_008982 [Chilo suppressalis]	3	513	58.8	7.44	8	3	3	High	1	1.02	1.122	0.931	0.968	1.026	0.962	1.023	1.016	1.104	1.036	1.173	1.712	1.607	1.633
TRINITY_DN15685_c0_g1_i5_orf1	uncharacterized protein LOC114352354 [Ostrinia furnacalis]	3	255	29.1	7.14	14	3	3	High	1	0.905	0.84	3.22	3.382	3.063	2.588	2.592	2.554	2.35	2.466	2.541	0.778	0.848	0.868
TRINITY_DN753_c0_g1_i4_orf1	venom dipeptidyl peptidase 4-like isoform X2 [Ostrinia furnacalis]	3	843	92.6	4.82	5	4	3	High	1	1.006	1.022	0.376	0.401	0.419	0.362	0.434	0.463	0.319	0.296	0.35	0.394	0.431	0.429
TRINITY_DN7170_c0_g1_i11_orf1	sulfite oxidase isoform X1 [Ostrinia furnacalis]	3	456	51	7.05	12	3	3	High	1	1.003	0.976	1.225	1.342	1.156	1.109	1.158	1.144	1.049	0.875	1.073	1.159	1.232	1.106
TRINITY_DN146841_c0_g1_i1_orf1	muscle-specific protein 20 [Temnothorax curvispinosus]	2	184	20.6	8.69	10	4	1	High	1	0.9	0.961	0.903	0.93	0.873	0.986	1.143	0.911	0.906	1.027	1.273	0.211	0.236	0.162
TRINITY_DN64222_c0_g1_i1_orf1	heat shock protein 20.2 [Glyphodes pyloalis]	2	176	20	6.55	18	2	1	High	1	1.016	0.837	0.878	0.913	0.944	0.88	0.916	1.042	1.032	0.944	0.923	1.301	1.406	1.332
TRINITY_DN2286_c2_g1_i1_orf1	coatomer subunit zeta-1 isoform X1 [Ostrinia furnacalis]	2	184	20.6	5.25	21	2	2	High	1	0.962	1.073	2.166	1.894	2.063	2.229	2.316	2.495	2.28	4.412	2.039	1.829	1.781	1.946
TRINITY_DN27488_c0_g1_i9_orf1	hypothetical protein evm_006422 [Chilo suppressalis]	3	209	24.4	5.81	19	3	3	High	1	1.042	0.964	0.872	0.885	0.898	0.986	1.156	1.065	0.928	0.96	1.008	1.002	1.018	0.962
TRINITY_DN54366_c0_g1_i1_orf1	protein obstructor-E-like [Ostrinia furnacalis]	6	239	26.9	5	28	6	6	High	1	1.071	1.068	0.648	0.65	0.65	0.677	0.654	0.741	0.682	0.594	0.613	3.04	3.29	3.184
TRINITY_DN17828_c0_g1_i1_orf1	ER membrane protein complex subunit 8/9 homolog [Ostrinia furnacalis]	4	215	24.3	6.34	18	5	4	High	1	1	1.051	0.75	0.747	0.774	0.583	0.666	0.65	0.605	1.364	0.604	0.838	0.763	0.875
TRINITY_DN30131_c0_g1_i1_orf1	PREDICTED: 60S ribosomal protein L44 [Amyelois transitella]	3	104	12.3	10.52	28	6	3	High	1	1.003	0.968	0.541	0.6	0.591	0.565	0.537	0.558	0.395	0.519	0.411	0.408	0.428	0.416
TRINITY_DN6497_c0_g1_i1_orf1	ommochrome-binding protein-like [Ostrinia furnacalis]	2	152	17	6.3	15	4	2	High	1	1.109	1.068	2.066	2.06	2.294	2.42	2.37	2.223	3.015	2.771	3.148	2.187	2.357	2.26
TRINITY_DN74020_c0_g1_i2_orf1	unnamed protein product [Euphydryas editha]	2	98	10.4	4.74	21	4	2	High	1	1.083	1.272	1.224	1.408	1.091	1.556	1.65	1.645	1.371	1.339	1.391	1.443	1.57	1.493
TRINITY_DN106038_c0_g1_i1_orf1	ankyrin-3-like isoform X1 [Galleria mellonella]	3	457	47.2	7.99	8	3	3	High	1	1.07	1.024	0.487	0.537	0.537	0.546	0.519	0.478	0.549	0.438	0.497	0.696	0.694	0.704
TRINITY_DN2967_c0_g1_i4_orf1	UDP-glycosyltransferase UGT41G1 [Ostrinia furnacalis]	3	194	21.7	6.8	13	4	3	High	1	1.013	1.02	0.523	0.552	0.551	0.654	0.63	0.71	0.615	0.654	0.691	0.573	0.606	0.54
TRINITY_DN57111_c0_g1_i1_orf1	trypsin-like serine proteinase T26 protein, partial [Chilo infuscatellus]	2	73	7.9	4.56	26	5	1	High	1	0.961	0.907	3.679	3.857	3.751	1.788	1.973	1.656	1.94	1.591	2.09	0.41	0.458	0.512
TRINITY_DN461_c0_g1_i5_orf1	cullin-1 isoform X1 [Ostrinia furnacalis]	6	789	91	8.21	6	6	6	High	1	0.994	1.004	0.91	0.876	0.921	0.817	0.808	0.796	0.855	0.886	0.756	0.939	0.945	0.939
TRINITY_DN41506_c0_g1_i4_orf1	60S ribosome subunit biogenesis protein NIP7 homolog [Ostrinia furnacalis]	2	180	20.6	9.25	22	3	2	High	1	1.015	1.021	0.926	0.901	0.961	0.974	0.848	0.86	0.938	1.147	0.907	0.81	0.859	0.808
TRINITY_DN323_c0_g2_i5_orf1	oxygen-dependent coproporphyrinogen-III oxidase isoform X1 [Ostrinia furna	3	391	45.4	6.76	9	4	3	High	1	1.013	1.045	0.829	0.855	0.884	0.96	1.112	1.163	1.041	1.037	0.854	1.066	1.006	1.083
TRINITY_DN4621_c0_g1_i4_orf1	uncharacterized protein LOC114358242 isoform X3 [Ostrinia furnacalis]	3	481	56.1	6.29	8	3	3	High	1	0.994	1.196	1.748	1.695	1.912	1.773	1.877	2.032	2.019	1.981	1.705	1.409	1.352	1.274
TRINITY_DN1853_c0_g1_i3_orf1	trans-Golgi network integral membrane protein TGN38-like isoform X1 [Ostrir	3	514	56	5.01	7	3	3	High	1	0.935	0.987	0.835	0.959	0.757	0.732	0.926	0.807	0.668	0.613	0.824	1.235	1.145	1.353
TRINITY_DN14922_c0_g1_i4_orf1	pseudouridine-5'-phosphatase-like [Ostrinia furnacalis]	3	228	25.5	6.44	18	4	3	High	1	1.113	1.063	1.043	1.098	1.01	0.974	1.069	1.013	1.059	1.056	1.287	0.862	0.841	0.924
TRINITY_DN9555_c0_g1_i1_orf1	pyridoxal kinase [Ostrinia furnacalis]	4	301	33.1	6.24	13	4	4	High	1	1.067	1.114	0.781	0.696	0.76	0.811	0.852	0.811	0.865	0.867	0.759	0.888	0.809	0.874
TRINITY_DN41108_c0_g1_i1_orf1	coatomer subunit epsilon-like [Ostrinia furnacalis]	3	223	24.3	5.12	18	5	3	High	1	1.03	1.038	0.804	0.781	0.801	0.818	0.969	0.899	0.849	0.84	0.737	1.027	0.982	1.005
TRINITY_DN34857_c0_g1_i6_orf1	uridine diphosphate glucose pyrophosphatase-like [Ostrinia furnacalis]	2	211	24.1	5.24	12	4	2	High	1	0.969	1.031	0.859	0.886	0.766	0.859	0.844	0.825	1.035	0.86	1.026	0.799	0.763	0.858
TRINITY_DN9938_c0_g2_i1_orf1	hypothetical protein E2986_04423 [Frieseomelitta varia]	2	180	21.4	11.66	11	6	2	High	1	1.018	0.99	0.684	0.693	0.748	0.672	0.682	0.679	0.633	0.547	0.585	0.882	0.832	0.8
TRINITY_DN33967_c2_g2_i1_orf1	titin homolog [Ostrinia furnacalis]	5	886	94.8	4.56	7	5	5	High	1	0.803	0.826	0.646	0.875	0.708	0.751	0.75	0.646	0.767	0.86	0.89	1.248	1.198	1.285
TRINITY_DN10484_c0_g1_i8_orf1	hypothetical protein evm_010330 [Chilo suppressalis]	4	1389	152.9	5.69	5	4	4	High	1	0.943	0.933	1.109	1.188	1.111	1.401	1.464	1.422	1.167	1.204	1.337	1.169	1.097	1.032
TRINITY_DN63561_c1_g2_i2_orf1	unnamed protein product [Diatraea saccharalis]	5	803	90.8	7.23	7	5	5	High	1	0.958	0.977	0.741	0.758	0.727	0.672	0.75	0.734	0.649	0.699	0.681	0.872	0.843	0.796
TRINITY_DN8158_c0_g1_i2_orf1	hypothetical protein G9C98_000136 [Cotesia typhae]	2	735	88	7.17	1	11	2	High	1	1.036	1.03	4.062	1.825	3.32	5.546	3.634	6.947	10.985	12.334	3.016	4.103	3.541	3.496
TRINITY_DN14306_c0_g1_i1_orf1	prostaglandin E synthase 2 [Galleria mellonella]	4	386	44	8.97	11	4	4	High	1	1.059	1.032	0.886	1.01	0.907	0.811	0.859	0.758	0.729	0.726	1.063	0.706	0.708	0.809
TRINITY_DN21758_c0_g1_i1_orf1	RNA transcription, translation and transport factor protein [Ostrinia furnacalis]	3	223	25.1	6.42	23	3	3	High	1	1.059	0.991	0.916	0.947	0.961	1.007	1.055	1.011	1.027	1.086	1.048	1.027	0.97	0.978
TRINITY_DN18933_c0_g1_i3_orf1	PREDICTED: protein BUD31 homolog [Papilio xuthus]	4	144	17.1	8.59	28	4	4	High	1	0.977	0.991	0.685	0.688	0.739	0.607	0.675	0.703	0.564	0.462	0.546	0.933	0.981	0.975
TRINITY_DN5346_c0_g1_i5_orf1	syntaxin-1A isoform X2 [Pectinophora gossypiella]	2	291	33.2	5.34	8	2	2	High	1	0.978	1.017	0.802	1.029	0.897	0.703	0.668	0.685	0.736	0.612	0.705	0.976	1.084	0.847
TRINITY_DN36718_c0_g1_i1_orf1	unnamed protein product [Chilo suppressalis]	3	472	52	7.55	8	3	3	High	1	0.877	0.982	0.538	0.48	0.522	0.528	0.513	0.465	0.531	0.544	0.462	0.52	0.51	0.526
TRINITY_DN16147_c0_g1_i4_orf1	aldo-keto reductase AKR2E4-like isoform X1 [Ostrinia furnacalis]	3	354	40.5	7.49	12	4	3	High	1	1.045	1.078	1.625	1.716	1.617	1.335	1.384	1.337	1.547	1.555	1.71	1.437	1.402	1.33
TRINITY_DN27885_c0_g1_i3_orf1	ubiquinone biosynthesis protein COQ9-B, mitochondrial-like isoform X2 [Ostri	3	278	31	8.78	13	3	3	High	1	1.084	1.036	0.754	0.745	0.786	0.808	0.863	0.799	0.948	0.86	0.805	0.726	0.738	0.652
TRINITY_DN19731_c0_g1_i1_orf1	allergen Tha p 1-like [Ostrinia furnacalis]	3	129	14.9	7.3	23	5	3	High	1	1.02	1.035	2.495	2.632	2.764	3.491	3.355	3.351	3.473	3.147	3.856	2.091	2.327	1.979
TRINITY_DN15175_c0_g1_i1_orf1	zinc carboxypeptidase-like [Ostrinia furnacalis]	3	399	45.8	5.34	7	3	3	High	1	1.056	1.085	1.08	1.062	1.181	1.451	1.405	1.22	1.675	1.72	1.822	8.516	8.539	8.363
TRINITY_DN2983_c0_g1_i6_orf1	hypothetical protein evm_002448 [Chilo suppressalis]	2	1383	156.5	6.24	2	2	2	High	1	0.957	1.143	1.37	1.463	1.483	1.681	1.343	1.529	1.305	1.738	1.371	1.206	1.196	1.172
TRINITY_DN103118_c0_g1_i4_orf1	hypothetical protein evm_006930 [Chilo suppressalis]	3	310	35.2	9.38	13	3	3	High	1	0.982	1.058	0.897	0.806	0.798	0.959	0.858	0.974	0.715	0.687	0.656	0.983	0.888	1.18

TRINITY_DN1450_c0_g2_i1_orf1	death-associated protein 1 [Ostrinia furnacalis]	2	101	10.7	9.52	37	4	2	High	1	1.209	1.062	3.045	3.118	3.234	3.101	2.819	2.698	2.599	2.113	3.095	1.592	1.798	1.351
TRINITY_DN9637_c0_g1_i14_orf1	zinc finger protein swm isoform X3 [Ostrinia furnacalis]	3	726	83.4	9.64	5	3	3	High	1	0.993	1.072	0.632	0.606	0.654	0.584	0.67	0.542	0.656	0.518	0.627	0.956	0.956	0.85
TRINITY_DN48536_c0_g1_i3_orf1	unnamed protein product [Chilo suppressalis]	4	524	59.5	5.54	13	4	4	High	1	1.125	1.151	0.708	0.673	0.654	0.697	0.701	0.716	0.762	0.758	0.727	0.778	0.784	0.806
TRINITY_DN2201_c0_g1_i1_orf1	pleiotropic regulator 1 [Ostrinia furnacalis]	3	449	49.6	8.76	6	5	2	High	1	1.012	0.963	0.626	0.669	0.693	1.155	0.979	1.09	0.757	0.833	0.746	1.273	1.201	1.303
TRINITY_DN4681_c0_g2_i2_orf1	regulator of nonsense transcripts 2 isoform X2 [Ostrinia furnacalis]	4	1122	128.6	5.29	4	4	4	High	1	0.973	1.011	0.765	0.694	0.807	0.83	1.03	0.925	0.807	0.849	0.83	0.87	0.855	0.823
TRINITY_DN3406_c0_g1_i17_orf1	uncharacterized protein ZK1073.1-like isoform X6 [Spodoptera frugiperda]	4	395	44	8.56	10	5	4	High	1	1.02	0.948	1.536	1.532	1.492	1.344	1.524	1.535	1.348	1.259	1.339	1.284	1.255	1.254
TRINITY_DN6365_c0_g1_i4_orf1	40S ribosomal protein S21 [Helicoverpa armigera]	3	83	9.1	7.8	42	4	3	High	1	1.059	1.033	0.836	0.83	0.865	0.934	0.875	0.865	0.716	0.738	0.747	0.588	0.617	0.564
TRINITY_DN79083_c0_g1_i2_orf1	unnamed protein product [Arctia plantaginis]	4	385	44.8	5.27	12	4	4	High	1	1.042	0.987	0.734	0.712	0.838	0.944	1.013	0.968	1.083	0.868	0.974	0.758	0.74	0.8
TRINITY_DN11159_c0_g2_i1_orf1	sphingosine-1-phosphate lyase isoform X1 [Ostrinia furnacalis]	2	481	52.7	8.02	7	2	2	High	1	1.148	1.104	0.957	0.834	0.86	0.829	0.812	0.92	0.946	0.869	0.824	0.901	0.94	0.858
TRINITY_DN54524_c0_g1_i6_orf1	serine protease inhibitor dipetalogastin [Ostrinia furnacalis]	3	502	55.3	8.05	7	3	3	High	1	0.973	1.1	0.924	0.81	0.96	0.82	0.975	0.918	0.814	0.843	0.835	4.171	4.274	4.487
TRINITY_DN18230_c1_g2_i1_orf1	hypothetical protein O3G_MSEX004459 [Manduca sexta]	2	84	9.5	6.11	36	4	2	High	1	1.096	1.056	1.773	1.763	1.777	1.913	1.837	1.903	2.069	1.877	1.873	1.944	2	1.994
TRINITY_DN25210_c0_g1_i1_orf1	mitochondrial import receptor subunit TOM22 homolog [Ostrinia furnacalis]	3	159	17.5	4.49	26	3	3	High	1	1.022	0.992	0.694	0.636	0.697	0.62	0.715	0.582	0.566	0.551	0.65	0.735	0.679	0.715
TRINITY_DN11981_c0_g1_i7_orf1	luciferin 4-monooxygenase-like isoform X2 [Ostrinia furnacalis]	3	556	62.4	6.62	7	3	3	High	1	0.898	0.962	3.753	3.79	3.356	2.284	2.417	2.588	2.977	2.588	2.913	1.698	1.578	1.517
TRINITY_DN45949_c0_g1_i1_orf1	uncharacterized protein LOC114355167 [Ostrinia furnacalis]	3	199	22.7	6.01	18	3	3	High	1	0.976	0.943	1.435	1.589	1.612	1.181	1.217	1.194	1.48	1.384	1.632	0.423	0.409	0.446
TRINITY_DN1427_c0_g1_i7_orf1	SAFB-like transcription modulator isoform X1 [Ostrinia furnacalis]	3	510	58.8	9.77	9	4	3	High	1	0.969	0.98	0.675	0.739	0.766	0.684	0.7	0.688	0.547	0.537	0.582	0.69	0.785	0.696
TRINITY_DN28577_c0_g1_i6_orf1	delta-1-pyrroline-5-carboxylate dehydrogenase, mitochondrial [Nymphalis io	3	104	11.3	9.28	34	7	3	High	1	0.961	0.976	0.295	0.289	0.34	0.324	0.321	0.336	0.309	0.295	0.303	0.332	0.334	0.358
TRINITY_DN13324_c0_g1_i3_orf1	aldehyde dehydrogenase, partial [Dioryctria abietella]	4	114	12.5	4.77	44	7	4	High	1	0.987	0.998	0.981	0.846	1.003	1.109	1.035	1.146	1.017	0.988	0.919	1.041	1.02	1.035
TRINITY_DN6231_c0_g1_i6_orf1	ran-binding protein 3 isoform X1 [Ostrinia furnacalis]	4	452	48.6	5.07	11	4	4	High	1	0.937	0.991	0.468	0.482	0.498	0.441	0.442	0.424	0.342	0.344	0.379	0.531	0.541	0.528
TRINITY_DN14301_c0_g1_i1_orf1	apoptosis-inducing factor 1, mitochondrial-like [Ostrinia furnacalis]	4	350	36.6	5.12	11	4	4	High	1	0.969	1.029	0.627	0.732	0.622	0.762	0.756	0.779	0.628	0.829	0.688	0.597	0.677	0.624
TRINITY_DN1641_c0_g1_i6_orf1	rhodanese domain-containing protein CG4456-like [Ostrinia furnacalis]	3	166	18.7	9.1	22	4	3	High	1	0.984	1.026	0.636	0.657	0.672	0.863	0.89	0.803	0.823	0.765	0.809	0.75	0.822	0.781
TRINITY_DN1249_c0_g1_i10_orf1	venom carboxylesterase-6-like [Ostrinia furnacalis]	3	508	55.8	4.75	6	4	1	High	1	0.995	0.943	0.15	0.161	0.241	0.298	0.284	0.277	0.299	0.305	0.285	0.2	0.185	0.193
TRINITY_DN3649_c0_g1_i6_orf1	unnamed protein product [Chilo suppressalis]	3	943	103.8	5.64	3	3	3	High	1	1.01	0.977	0.572	0.637	0.676	0.605	0.797	0.812	0.487	0.373	0.47	0.889	0.874	0.912
TRINITY_DN3835_c0_g1_i4_orf1	protein ERGIC-53 isoform X1 [Ostrinia furnacalis]	2	146	15.5	8.5	25	3	1	High	1	1.02	1.054	0.945	0.882	0.923	0.762	0.689	0.78	0.89	0.662	0.742	0.937	0.847	0.801
TRINITY_DN54554_c0_g1_i1_orf1	LOW QUALITY PROTEIN: signal transducing adapter molecule 2 [Ostrinia furna	3	154	16.8	6.68	20	3	3	High	1	1	0.927	1.101	1.321	1.09	1.122	1.133	1.098	1.055	1.291	1.206	1.193	1.227	1.307
TRINITY_DN74654_c0_g1_i4_orf1	limulus clotting factor C-like isoform X4 [Ostrinia furnacalis]	3	100	11.4	9.41	28	6	3	High	1	1.005	1.036	1.936	1.609	1.895	1.873	1.623	1.925	2.245	1.854	1.879	2.012	1.855	1.876
TRINITY_DN22443_c0_g2_i3_orf1	protein eiger [Ostrinia furnacalis]	3	488	54.7	5.62	7	4	3	High	1	0.954	0.959	1.108	1.163	1.213	1.049	1.238	1.207	1.365	1.252	1.055	1.775	1.701	1.696
TRINITY_DN2401_c0_g2_i1_orf1	DNA-directed RNA polymerase I subunit RPA2 [Ostrinia furnacalis]	4	1105	123.9	8.56	4	4	4	High	1	0.952	1.005	0.91	0.902	0.929	0.92	0.943	0.966	0.927	0.94	0.954	0.764	0.778	0.795
TRINITY_DN2182_c0_g1_i4_orf1	growth-blocking peptide, long form-like isoform X1 [Ostrinia furnacalis]	4	167	18.8	7.94	22	4	4	High	1	1.048	0.994	1.592	1.696	1.54	1.477	1.394	1.363	1.646	1.413	1.649	1.23	1.368	1.173
TRINITY_DN2348_c0_g1_i1_orfp1	TRINITY_DN2348_c0_g1_i1_m.39060 TRINITY_DN2348_c0_g1::TRINITY_DN2348	2	148	16.6	6.43	29	3	2	High	1	1.045	1.065	1.283	1.321	1.308	1.245	1.377	1.136	1.401	1.229	1.611	1.991	1.928	1.873
TRINITY_DN1789_c0_g1_i5_orf1	uncharacterized protein LOC114365543 [Ostrinia furnacalis]	5	1146	131.2	8.85	5	5	5	High	1	0.938	1.001	1.551	1.597	1.589	1.072	1.118	1.113	1.364	1.28	1.387	1.117	1.123	1.042
TRINITY_DN1232_c0_g1_i1_orf1	acanthoscurrin-2-like isoform X1 [Ostrinia furnacalis]	2	171	16.6	8.56	15	3	2	High	1	0.937	0.966	0.908	0.937	0.841	0.717	0.795	0.854	0.7	0.679	0.629	0.411	0.453	0.422
TRINITY_DN15265_c0_g1_i1_orf1	cullin-5 [Ostrinia furnacalis]	3	779	90.3	7.66	3	4	3	High	1	0.977	1.023	1.038	0.97	1.025	0.843	0.793	0.9	0.853	0.755	0.811	0.962	0.899	0.934
TRINITY_DN73_c0_g1_i6_orf1	autophagy-related protein 16-1 isoform X1 [Vanessa cardui]	4	570	63.1	7.43	8	4	4	High	1	0.982	0.918	1.42	1.521	1.367	1.506	1.608	1.51	1.427	1.611	1.468	1.374	1.219	1.343
TRINITY_DN11347_c0_g1_i1_orf1	N(4)-(Beta-N-acetylglucosaminyl)-L-asparaginase-like [Ostrinia furnacalis]	4	341	37	6.09	13	5	4	High	1	1.077	0.996	0.209	0.201	0.294	0.272	0.267	0.31	0.341	0.305	0.3	0.357	0.316	0.308
TRINITY_DN5149_c0_g1_i14_orfp1	TRINITY_DN5149_c0_g1_i14_m.8804 TRINITY_DN5149_c0_g1::TRINITY_DN5149	3	87	10.6	6.55	54	5	1	High	1	0.92	1.067	1.121	0.997	1.242	1.323	1.821	1.424	1.287	0.948	1.181	0.782	0.8	0.989
TRINITY_DN14920_c0_g1_i1_orf1	anamorsin homolog [Ostrinia furnacalis]	2	250	27.1	5.29	8	4	2	High	1	1.033	1.026	0.495	0.49	0.536	0.432	0.432	0.431	0.474	0.412	0.474	0.661	0.67	0.686
TRINITY_DN108573_c0_g1_i1_orf1	uncharacterized protein LOC114366171 [Ostrinia furnacalis]	3	416	47.8	5.47	7	3	3	High	1	0.958	0.968	0.221	0.225	0.254	0.256	0.232	0.266	0.297	0.275	0.295	0.277	0.28	0.294
TRINITY_DN625_c9_g1_i7_orf1	ecdysone 20-monooxygenase [Ostrinia furnacalis]	3	517	58.2	8.72	8	3	3	High	1	1.07	1.146	1.53	1.503	1.515	1.6	1.637	1.763	1.485	1.797	1.462	1.306	1.235	1.184
TRINITY_DN69535_c0_g1_i2_orf1	juvenile hormone esterase-like [Ostrinia furnacalis]	4	545	61.3	5	9	4	4	High	1	0.955	1.033	0.754	0.717	0.694	0.768	0.778	0.826	0.82	0.859	0.835	0.757	0.748	0.776
TRINITY_DN1716_c0_g1_i14_orf1	putative gamma-glutamylcyclotransferase CG2811 isoform X3 [Ostrinia furnac	3	184	21.4	7.56	16	3	3	High	1	0.995	1.067	0.786	0.755	0.759	0.804	0.843	0.791	0.999	0.783	0.932	1.411	1.408	1.326
TRINITY_DN10441_c0_g1_i3_orf1	zonadhesin-like [Ostrinia furnacalis]	3	1092	119.6	6.83	4	3	3	High	1	0.961	1.059	1.697	1.859	1.939	1.461	1.426	1.351	1.521	1.177	1.497	0.855	0.858	0.797
TRINITY_DN9000_c0_g2_i1_orf1	uncharacterized protein LOC114356585 [Ostrinia furnacalis]	5	533	59.8	6.52	14	6	5	High	1	1.029	0.981	0.808	0.736	0.823	0.823	0.828	0.842	0.785	0.834	0.768	1.392	1.376	1.363
TRINITY_DN40015_c0_g1_i2_orf1	PREDICTED: 60S ribosomal protein L18 [Amyelois transitella]	4	184	21	11.87	20	12	4	High	1	0.918	1.092	0.753	0.736	0.714	0.642	0.687	0.695	0.63	0.564	0.631	0.619	0.627	0.617
TRINITY_DN7226_c0_g1_i5_orf1	chemosensory protein [Dioryctria abietella]	4	76	8.8	8.82	28	8	4	High	1	0.854	0.86	3.845	4.12	3.8	2.87	3.195	2.986	3.458	3.515	3.594	2.025	1.853	2.017
TRINITY_DN2345_c0_g1_i4_orf1	chromobox protein homolog 3-like [Ostrinia furnacalis]	2	179	20.3	5.15	16	3	2	High	1	1.021	0.85	0.907	0.844	0.99	0.826	0.824	0.8	0.815	0.754	0.788	1.205	1.12	1.168
TRINITY_DN1710_c0_g2_i2_orf1	relish [Ostrinia furnacalis]	3	864	96.4	6.27	3	3	3	High	1	0.997	1.006	1.161	1.142	1.227	0.9	0.848	0.895	0.858	0.679	0.809	1.01	0.961	0.947
TRINITY_DN4276_c0_g1_i11_orf1	arylsulfatase B [Ostrinia furnacalis]	3	411	45.9	5.4	10	3	2	High	1	1.141	1.047	1.673	1.484	1.399	1.395	1.563	1.389	1.791	1.438	1.461	1.867	1.709	1.448
TRINITY_DN11133_c0_g1_i5_orf1	UHRF1-binding protein 1-like isoform X6 [Helicoverpa zea]	4	1254	136.8	6.18	3	4	4	High	1	0.99	1.006	1.211	1.218	1.372	1.11	1.122	1.249	1.058	1.092	0.993	1.142	1.124	1.128
TRINITY_DN16451_c0_g1_i7_orf1	diphthine--ammonia ligase-like [Ostrinia furnacalis]	4	752	83.4	5.35	6	5	4	High	1	1.026	1.032	0.902	0.947	0.966	1.159	1.081	1.109	1.004	0.964	1.034	0.927	0.876	0.843
TRINITY_DN18918_c0_g1_i3_orf1	myrosinase 1-like isoform X2 [Ostrinia furnacalis]	4	284	32.6	5.19	15	4	2	High	1	0.997	1.1	0.662	0.692	0.637	0.592	0.648	0.617	0.848	0.897	0.872	0.634	0.696	0.644
TRINITY_DN77005_c0_g2_i1_orf1	RNA polymerase II degradation factor 1-like [Ostrinia furnacalis]	2	153	15.7	4.92	21	2	2	High	1	0.942	0.954	0.915	1.057	0.937	0.938	0.951	0.91	1.128	1.174	0.966	1.227	1.127	1.042
TRINITY_DN105506_c0_g1_i8_orf1	microtubule-actin cross-linking factor 1 isoform X15 [Ostrinia furnacalis]	4	129	14.5	7.53	27	5	3	High	1	1.088	1.025	1.37	1.345	1.348	1.545	1.384	1.348	1.27	1.296	1.25	0.813	0.831	0.827
TRINITY_DN61112_c0_g1_i4_orfp1	TRINITY_DN61112_c0_g1_i4_m.53012 TRINITY_DN61112_c0_g1::TRINITY_DN61	3	77	9.2	7.2	39	3	3	High	1	0.971	0.965	0.16	0.156	0.193	0.175	0.181	0.21	0.209	0.2	0.183	0.204	0.181	0.198
TRINITY_DN12003_c0_g1_i1_orf1	hypothetical protein, partial [Ostrinia furnacalis]	3	101	11.7	5.41	34	6	1	High	1	0.944	0.957	1.282	1.387	1.377	1.09	1.05	1.069	1.124	1.07	1.274	1.075	1.17	1.191
TRINITY_DN12009_c0_g1_i1_orf1	uncharacterized protein LOC114365631 [Ostrinia furnacalis]	2	70	8.2	6.54	17	5	2	High	1	0.946	0.923	3.376	2.868	3.394	5.649	4.911	5.463	3.352	3.343	2.911	5.886	6.932	5.323
TRINITY_DN4938_c0_g1_i13_orf1	peroxisomal biogenesis factor 19 [Ostrinia furnacalis]	4	290	31.8	4.28	11	4	4	High	1	0.957	0.923	0.604	0.614	0.623	0.481	0.463	0.473	0.491	0.457	0.509	0.426	0.45	0.402
TRINITY_DN640_c0_g1_i5_orf1	pancreatic triacylglycerol lipase-like [Ostrinia furnacalis]	3	335	37.2	8.48	13	4	3	High	1	1.059	1.049	0.721	0.703	0.814	0.804	0.799	0.941	0.844	0.869	0.711	0.612	0.667	0.675
TRINITY_DN14475_c0_g1_i1_orf1	NPC intracellular cholesterol transporter 1 homolog 1b-like [Ostrinia furnacalis	3	1245	138.1	4.92	3	4	3	High	1	0.893	0.959	0.642	0.578	0.723	0.686	0.749	0.83	0.673	0.749	0.711	0.651	0.634	0.704
TRINITY_DN35725_c0_g1_i1_orf1	mitochondrial import inner membrane translocase subunit Tim13-like [Bicyclus	3	95	10.9	8.16	39	3	3	High	1	1.024	0.958	0.279	0.259	0.353	0.33	0.324	0.299	0.309	0.29	0.337	0.468	0.476	0.457
TRINITY_DN12579_c0_g1_i1_orf1	uncharacterized protein LOC114354070 isoform X3 [Ostrinia furnacalis]	3	107	11.9	4.41	40	3	3	High	1	1.085	0.994	0.64	0.663	0.622	0.561	0.696	0.643	0.494	0.572	0.535	0.566	0.63	0.504
TRINITY_DN3826_c0_g1_i1_orf1	39S ribosomal protein L18, mitochondrial [Ostrinia furnacalis]	1	170	19.6	9.52	11	3	1	High	1	0.969	0.992	1.556	1.487	1.591	1.367	1.503	1.464	1.856	2.193	2.113	0.924	0.855	1.206
TRINITY_DN6016_c0_g1_i8_orf1	hypothetical protein evm_010883 [Chilo suppressalis]	4	328	37.7	6.3	14	4	4	High	1	0.972	1.004	0.89	0.923	0.962	0.534	0.667	0.601	0.768	0.693	0.738	0.812	0.883	0.959
TRINITY_DN16258_c0_g1_i2_orf1	uncharacterized protein LOC114359911 [Ostrinia furnacalis]	3	252	27.3	8.46	15	5	3	High	1	1.054	1.002	0.518	0.557	0.602	0.469	0.463	0.476	0.492	0.388	0.535	0.65	0.709	0.685
TRINITY_DN2826_c0_g1_i7_orf1	ATP-binding cassette subfamily D member 1 [Chilo suppressalis]	4	671	76.8	9.33	7	4	4	High	1	0.906	0.967	0.701	0.621	0.66	0.797	0.804	0.783	0.7	0.681	0.787	0.798	0.806	0.807
TRINITY_DN8985_c0_g1_i4_orf1	cytochrome P450 6B6-like [Ostrinia furnacalis]	4	516	59.5	8.28	8	4	3	High	1	1.037	1.128	1.508	1.419	1.501	2.015	2.051	1.892	2.182	2.154	2.289	2.298	2.349	2.404
TRINITY_DN5956_c1_g1_i5_orf1	uncharacterized protein DDB_G0286299-like [Ostrinia furnacalis]	3	591	66.4	9.61	7	3	3	High	1	1.06	1.025	0.327	0.312	0.341	0.363	0.427	0.333	0.329	0.285	0.35	0.316	0.32	0.284
TRINITY_DN7037_c0_g1_i4_orf1	unnamed protein product [Chilo suppressalis]	3	158	18	9.22	22	3	3	High	1	1.039	0.977	0.523	0.493	0.536	0.611	0.626	0.584	0.614	0.538	0.687	0.815	0.819	0.793
TRINITY_DN9437_c0_g1_i1_orf1	glyoxylate reductase/hydroxypyruvate reductase-like [Ostrinia furnacalis]	4	356	38.3	7.96	11	4	4	High	1	1.009	1.031	0.66	0.717	0.656	0.739	0.744	0.733	0.6	0.881	0.647	0.798	0.719	0.802
TRINITY_DN10581_c0_g1_i5_orf1	facilitated trehalose transporter Tret1-like isoform X1 [Ostrinia furnacalis]	2	495	53.9	8.66	5	5	2	High	1	1.031	1.093	1.342	1.474	1.321	1.346	1.485	1.349	1.228	1.212	1.295	1.027	1.095	1.07
TRINITY_DN143496_c0_g1_i1_orf1	cullin-3 [Diachasma alloeum]	3	782	90.6	8.6	4	3	1	High	1	1.05	1.124	0.807	0.945	1.113	0.866	0.909	0.906	0.722	0.624	0.625	0.866	0.798	0.915
TRINITY_DN3809_c0_g1_i7_orf1	hypothetical protein O3G_MSEX012108 [Manduca sexta]	3	258	29.3	8.43	17	3	3	High	1	0.956	0.923	0.979	0.805	0.871	0.96	0.841	1.039	0.937	0.917	0.674	0.877	0.849	0.935
TRINITY_DN4959_c0_g1_i1_orf1	pancreatic triacylglycerol lipase-like [Ostrinia furnacalis]	2	338	37.1	9	7	2	1	High	1	0.937	0.911	0.026	0.01	0.073	0.104	0.129	0.12	0.128	0.115	0.082	0.123	0.135	0.129
TRINITY_DN57636_c0_g1_i4_orf1	PREDICTED: plectin-like, partial [Papilio polytes]	4	157	18.6	7.4	25	4	3	High	1	0.981	1.008	0.975	1.032	1.021	0.969	0.943	1.064	0.872	1.001	1.064	0.826	0.78	0.836
TRINITY_DN3244_c0_g1_i4_orf1	protein ABHD16A isoform X1 [Ostrinia furnacalis]	4	495	56	8.47	9	4	4	High	1	1.039	0.994	0.725	0.774	0.712	0.807	0.79	0.84	0.806	0.783	0.826	0.921	0.857	0.936
TRINITY_DN3298_c0_g2_i4_orf1	macrophage mannose receptor 1-like [Ostrinia furnacalis]	3	227	25.9	6.6	19	3	3	High	1	1.018	1.085	1.207	1.208	1.14	1.508	1.517	1.476	1.184	0.998	1.188	0.85	0.952	0.869
TRINITY_DN5274_c0_g2_i2_orf1	lopap-like [Ostrinia furnacalis]	3	211	24.4	5.63	14	4	2	High	1	0.98	1.044	2.518	2.556	2.359	2.294	1.89	2.126	2.511	2.253	2.295	1.649	1.608	1.553
TRINITY_DN4242_c0_g1_i6_orf1	fibrohexamerin-like [Ostrinia furnacalis]	3	222	25.5	7.83	14	3	3	High	1	0.966	1.036	1.322	1.271	1.286	2.355	2.196	2.337	2.48	2.338	2.425	0.631	0.587	0.516
TRINITY_DN42205_c0_g1_i4_orf1	eukaryotic translation initiation factor 4H [Ostrinia furnacalis]	5	197	20.9	8.69	29	6	5	High	1	1.005	1.005	0.711	0.738	0.756	0.734	0.765	0.798	0.757	0.746	0.703	0.978	0.906	0.885
TRINITY_DN18338_c0_g1_i6_orf1	aquaporin AQPAn.G isoform X1 [Ostrinia furnacalis]	1	279	29	7.34	8	2	1	High	1	1.113	0.965	2.841	2.064	3.387	2.921	2.466	2.889	1.907	2.057	2.196	6.357	5.495	5.89
TRINITY_DN18918_c0_g1_i2_orf1	myrosinase 1-like isoform X2 [Ostrinia furnacalis]	3	310	35.9	5.11	13	3	1	High	1	0.862	1.024	0.503	0.584	0.592	0.538	0.631	0.57	0.592	1.234	0.615	0.437	0.52	0.512
TRINITY_DN4476_c0_g1_i5_orf1	trypsin, alkaline C-like isoform X1 [Ostrinia furnacalis]	4	246	26.3	10.11	22	4	1	High	1	0.913	0.916	0.323	0.422	0.372	0.41	0.376	0.401	0.285	0.331	0.382	0.368	0.382	0.504
TRINITY_DN7918_c0_g1_i1_orf1	charged multivesicular body protein 2a [Ostrinia furnacalis]	3	207	23.3	7.27	12	5	3	High	1	0.934	0.931	0.856	0.888	0.894	0.854	0.884	0.903	0.961	0.908	0.895	1.014	1.107	1.024
TRINITY_DN46452_c0_g1_i1_orf1	hypothetical protein HELRODRAFT_157839, partial [Cotesia chilonis]	3	91	10.4	9	32	5	3	High	1	0.979	0.994	1.1	1.176	1.141	1.028	1.032	1.008	1.116	0.978	1.17	1.109	1.079	1.004
TRINITY_DN11215_c0_g1_i1_orf1	dnaJ homolog subfamily C member 2 [Ostrinia furnacalis]	5	623	72.5	8.51	8	5	5	High	1	1.02	0.984	0.594	0.608	0.618	0.573	0.613	0.625	0.577	0.672	0.631	0.7	0.728	0.719
TRINITY_DN4255_c0_g1_i10_orf1	LOW QUALITY PROTEIN: lebocin-4-like [Ostrinia furnacalis]	3	199	22.7	10.77	21	3	2	High	1	0.715	0.86	2.185	2.532	1.89	2.281	2.085	2.064	1.831	1.727	1.827	1.458	1.592	1.71
TRINITY_DN135449_c0_g1_i5_orf1	larval cuticle protein LCP-17-like [Galleria mellonella]	2	79	8.6	7.52	68	6	1	High	1	1.086	1.125	3.208	2.841	3.034	4.004	3.758	4.337	5.005	4.36	3.975	3.799	3.452	3.032
TRINITY_DN145224_c0_g1_i1_orf1	skin secretory protein xP2-like [Ostrinia furnacalis]	3	225	22.5	11.66	16	5	3	High	1	1.035	1.045	1.054	1.167	0.999	1.059	0.994	1.051	1.024	1.012	1.063	0.912	0.939	0.917
TRINITY_DN821_c0_g1_i8_orf1	nuclear pore complex protein Nup153 isoform X2 [Ostrinia furnacalis]	4	1349	142.8	9.07	3	4	4	High	1	0.925	1.037	0.68	0.664	0.752	0.708	0.647	0.634	0.67	0.807	0.604	0.697	0.698	0.747
TRINITY_DN9868_c0_g1_i6_orf1	sister chromatid cohesion protein PDS5 homolog B-B isoform X1 [Ostrinia furr	4	1192	135.9	7.28	4	4	4	High	1	1.028	1.033	0.943	0.933	0.931	0.893	0.909	1.016	1.241	0.861	0.804	0.882	0.898	0.906
TRINITY_DN13395_c0_g1_i1_orf1	cytoplasmic dynein 1 light intermediate chain 2 [Galleria mellonella]	5	429	47.8	5.41	14	5	5	High	1	1.017	0.989	0.768	0.824	0.786	0.711	0.75	0.706	0.731	0.693	0.803	1.409	1.472	1.385
TRINITY_DN3478_c0_g1_i10_orf1	acyl-CoA dehydrogenase family member 9, mitochondrial [Ostrinia furnacalis]	4	608	68.9	6.86	8	4	4	High	1	0.963	1.035	0.694	0.684	0.677	0.817	0.819	0.822	0.979	0.964	0.959	0.949	0.888	0.955
TRINITY_DN5487_c0_g1_i2_orf1	forkhead box protein K1 [Ostrinia furnacalis]	3	528	57.2	8.82	8	3	3	High	1	0.959	1	1.074	1.023	0.947	0.95	0.931	1.07	1.11	1.115	1.065	0.977	0.934	0.995
TRINITY_DN4859_c0_g1_i5_orf1	importin-11, partial [Ostrinia furnacalis]	2	1004	112.9	6.02	3	2	2	High	1	0.996	1.045	0.861	0.86	0.778	0.807	0.831	0.841	0.72	0.692	0.725	0.889	0.951	0.934
TRINITY_DN5603_c0_g1_i1_orf1	translin [Ostrinia furnacalis]	3	238	27.8	5.69	15	4	3	High	1	0.962	0.949	1.266	1.189	1.285	1.115	1.136	1.373	1.172	1.229	1.035	1.188	1.29	1.279

TRINITY_DN4538_c0_g1_i4_orf1	2-acylglycerol O-acyltransferase 1-like [Ostrinia furnacalis]	3	357	40.5	9.14	14	3	3	High	1	1.032	1.049	0.749	0.711	0.751	0.713	0.821	0.829	0.807	0.769	0.693	0.651	0.689	0.769
TRINITY_DN13018_c0_g1_i2_orf1	uncharacterized protein LOC114354768 isoform X1 [Ostrinia furnacalis]	2	290	32.8	6.2	14	2	1	High	1	1.017	0.985	0.8	0.854	0.975	0.777	0.774	0.997	0.944	1.058	0.837	0.902	0.89	0.939
TRINITY_DN11825_c0_g1_i4_orf1	39S ribosomal protein L22, mitochondrial [Ostrinia furnacalis]	4	203	23.7	9.85	27	4	4	High	1	1.002	1.068	0.607	0.559	0.594	0.55	0.588	0.629	0.546	0.524	0.536	0.55	0.551	0.555
TRINITY_DN18329_c0_g1_i2_orf1	bromodomain-containing protein 3 [Ostrinia furnacalis]	3	595	63.3	9.38	5	3	3	High	1	1.006	1.025	0.844	0.813	0.855	0.739	0.953	0.845	0.765	0.673	0.74	0.924	0.951	0.9
TRINITY_DN1354_c0_g1_i6_orf1	elongator complex protein 2 isoform X1 [Pectinophora gossypiella]	4	775	85.9	6.28	4	4	4	High	1	1.056	1.021	0.779	0.726	0.774	0.855	0.816	0.845	0.775	0.827	0.75	0.773	0.771	0.798
TRINITY_DN21619_c0_g1_i1_orf1	28S ribosomal protein S15, mitochondrial [Ostrinia furnacalis]	3	276	32.8	9.33	14	3	3	High	1	1.029	1.036	0.476	0.524	0.5	0.643	0.689	0.624	0.485	0.479	0.521	0.573	0.56	0.526
TRINITY_DN1749_c0_g2_i2_orf1	putative GPI-anchored protein pfl2 isoform X1 [Ostrinia furnacalis]	4	1058	116.8	5.15	5	4	4	High	1	1.114	1.093	1.058	1.018	1.052	1.098	1.085	0.986	0.868	0.806	0.914	3.828	3.95	3.767
TRINITY_DN52887_c0_g1_i1_orf1	cytochrome P450 6B5-like [Ostrinia furnacalis]	4	306	34.9	8.31	13	4	2	High	1	0.839	0.894	0.99	0.846	0.895	0.697	0.733	0.769	1.09	1.207	0.939	0.822	0.774	0.861
TRINITY_DN53166_c0_g1_i1_orf1	mannose-6-phosphate isomerase isoform X2 [Ostrinia furnacalis]	1	100	11.1	6.14	11	3	1	High	1	0.957	0.959	0.89	0.861	0.821	0.884	0.966	0.831	0.914	0.932	0.809	0.84	0.878	0.844
TRINITY_DN17130_c0_g2_i1_orf1	protein maelstrom homolog [Ostrinia furnacalis]	3	436	49.5	7.03	7	3	3	High	1	1.022	1.01	1.038	0.95	1.047	1.04	0.927	1.043	0.996	0.916	0.863	0.986	1.026	0.924
TRINITY_DN2187_c0_g1_i1_orf1	flotillin-1 [Chelonus insularis]	4	426	46.9	5.39	10	4	3	High	1	1.003	1.001	2.719	2.851	2.626	2.825	2.694	2.689	2.406	2.131	2.527	1.908	1.84	1.864
TRINITY_DN9544_c0_g1_i1_orf1	collagen type IV alpha-3-binding protein isoform X1 [Ostrinia furnacalis]	3	309	34.6	5.31	14	3	3	High	1	0.971	0.962	1.24	1.235	1.292	1.262	1.399	1.246	1.138	1.072	1.158	1.182	1.372	1.311
TRINITY_DN4367_c0_g1_i1_orf1	heat shock protein 21.7c [Chilo suppressalis]	3	187	21.6	6.96	19	8	1	High	1	1.021	0.768	0.972	0.939	0.833	1.134	1.136	1.465	1.27	1.306	1.277	3.254	3.022	3.034
TRINITY_DN5568_c0_g2_i2_orf1	carboxypeptidase D isoform X5 [Ostrinia furnacalis]	4	481	53.7	6.98	8	5	4	High	1	0.968	1.016	0.718	0.786	0.75	0.737	0.794	0.924	0.642	0.809	0.661	1.708	1.551	1.518
TRINITY_DN107261_c0_g1_i1_orf1	ATP synthase subunit g, mitochondrial [Ostrinia furnacalis]	3	98	10.7	9.94	30	4	3	High	1	0.902	0.93	0.447	0.445	0.466	0.432	0.42	0.394	0.453	0.537	0.482	0.365	0.361	0.429
TRINITY_DN109943_c0_g1_i1_orf1	uncharacterized protein LOC114361588 isoform X14 [Ostrinia furnacalis]	3	116	12.5	4.98	25	5	3	High	1	0.937	0.946	1.091	1.158	1.156	1.147	1.099	1.127	1.165	0.958	1.019	0.767	0.751	0.728
TRINITY_DN14904_c0_g1_i1_orf1	attacin [Ostrinia furnacalis]	3	157	17.3	10.4	26	3	3	High	1	1.089	1.107	2.871	2.981	2.948	8.823	9.278	8.223	1.937	1.792	2.282	2.209	2.181	2.076
TRINITY_DN2825_c0_g1_i3_orf1	carbonic anhydrase 2-like [Ostrinia furnacalis]	2	274	30.6	6.39	8	2	2	High	1	1.067	1.043	0.228	0.239	0.276	0.25	0.212	0.235	0.216	0.276	0.278	0.166	0.192	0.209
TRINITY_DN10650_c0_g1_i1_orf1	protein Skeletor, isoforms B/C-like [Ostrinia furnacalis]	4	683	75.4	6.9	6	5	4	High	1	0.933	0.975	1.43	1.412	1.272	1.182	1.268	1.191	1.073	0.968	1.143	1.22	1.177	1.167
TRINITY_DN13411_c0_g1_i4_orf1	facilitated trehalose transporter Tret1-like [Ostrinia furnacalis]	2	535	58.8	8.56	5	3	2	High	1	0.953	0.995	1.834	1.81	1.731	1.81	1.755	1.791	1.718	1.529	1.656	1.75	1.76	1.609
TRINITY_DN51441_c0_g1_i5_orf1	uncharacterized protein LOC114353529 isoform X2 [Ostrinia furnacalis]	4	666	73.7	9.11	6	6	4	High	1	1.077	1.05	0.883	0.913	0.939	0.887	0.936	0.88	0.878	0.877	0.835	1.048	1.072	0.985
TRINITY_DN20499_c0_g3_i1_orf1	exosome RNA helicase MTR4 isoform X2 [Ostrinia furnacalis]	3	476	54.4	7.55	7	3	2	High	1	0.991	0.969	0.642	0.589	0.633	0.519	0.534	0.564	0.455	0.539	0.515	0.644	0.633	0.636
TRINITY_DN4420_c0_g1_i1_orf1	guanine nucleotide-binding protein subunit beta-like protein 1 [Ostrinia furna	4	322	36.2	7.43	16	4	4	High	1	0.949	0.916	0.956	0.975	0.981	1.018	0.985	1.021	1.032	0.822	0.96	1.034	1.124	0.964
TRINITY_DN31390_c0_g1_i2_orf1	UDP-glucuronosyltransferase 2B20-like [Ostrinia furnacalis]	2	524	59.4	8.78	6	2	2	High	1	0.963	1.059	1.309	1.252	1.466	0.947	1.084	1.096	1.344	1.462	1.175	0.807	0.762	0.797
TRINITY_DN3653_c0_g1_i4_orf1	lysine-specific demethylase lid isoform X1 [Ostrinia furnacalis]	3	838	94.6	7.87	5	3	3	High	1	0.957	1.092	0.884	0.943	0.824	0.898	0.773	0.863	0.892	0.997	0.774	1.049	0.951	1.032
TRINITY_DN1215_c0_g1_i2_orf1	PI-stichotoxin-She2a-like [Ostrinia furnacalis]	2	80	8.6	4.51	31	3	2	High	1	0.948	0.862	0.775	0.925	0.848	1.186	1.281	1.137	1.055	1.052	1.252	0.427	0.44	0.445
TRINITY_DN94337_c0_g1_i1_orf1	hypothetical protein evm_006136 [Chilo suppressalis]	2	408	47.2	8.51	7	2	2	High	1	1.095	0.983	0.527	0.413	0.53	0.632	0.511	0.595	0.639	0.55	0.48	0.444	0.478	0.407
TRINITY_DN129808_c0_g1_i1_orf1	uncharacterized protein LOC114354070 isoform X3 [Ostrinia furnacalis]	3	133	14.7	5.21	25	3	3	High	1	0.967	0.98	0.882	0.923	0.884	0.847	0.83	0.804	0.759	0.725	0.715	0.718	0.751	0.712
TRINITY_DN25373_c0_g1_i1_orf1	epidermal growth factor receptor substrate 15-like 1 [Ostrinia furnacalis]	2	540	57.3	5	6	2	2	High	1	1.021	1.045	0.512	0.56	0.535	0.556	0.52	0.634	0.557	0.502	0.561	0.73	0.708	0.623
TRINITY_DN1425_c0_g1_i4_orf1	fibulin-2-like [Ostrinia furnacalis]	4	1434	158.5	5.02	3	4	4	High	1	0.961	1.002	0.758	0.782	0.759	0.771	0.831	0.717	0.614	0.641	0.635	0.937	0.982	1.031
TRINITY_DN25273_c0_g1_i1_orf1	skin secretory protein xP2-like [Ostrinia furnacalis]	3	283	29	9.74	16	3	3	High	1	1.04	1.125	1.292	1.445	1.399	1.466	1.517	1.615	1.535	1.486	1.62	4.83	4.817	4.516
TRINITY_DN12495_c0_g1_i2_orf1	probable ATP-dependent RNA helicase pitchoune [Manduca sexta]	2	609	69	9.22	3	3	2	High	1	0.973	0.969	0.429	0.401	0.466	0.393	0.402	0.403	0.389	0.385	0.368	0.365	0.384	0.398
TRINITY_DN10940_c0_g1_i10_orfp	TRINITY_DN10940_c0_g1_i10_m.52163 TRINITY_DN10940_c0_g1::TRINITY_DN1	3	247	22.7	4.6	14	3	1	High	1	1.091	1.05	0.2	0.186	0.2	0.195	0.181	0.227	0.193	0.194	0.219	0.085	0.085	0.068
TRINITY_DN1597_c0_g1_i5_orfp1	TRINITY_DN1597_c0_g1_i5_m.57494 TRINITY_DN1597_c0_g1::TRINITY_DN1597	2	85	9.2	6.73	27	2	2	High	1	0.991	0.966	2.84	2.934	2.77	5.528	4.987	4.635	4.186	3.869	5.182	3.041	3.157	2.891
TRINITY_DN2880_c0_g1_i2_orf1	sialomucin core protein 24 [Pectinophora gossypiella]	3	218	22.5	8.68	11	4	3	High	1	0.998	1.006	2.145	2.3	2.345	1.904	1.99	1.691	1.683	1.515	1.984	2.222	2.342	2.272
TRINITY_DN12823_c0_g1_i1_orf1	malectin-A [Ostrinia furnacalis]	3	264	29.7	5.4	13	3	3	High	1	0.994	1.121	0.86	0.881	0.904	0.845	0.867	0.8	0.897	0.762	0.852	1.033	0.895	1.081
TRINITY_DN12134_c0_g1_i4_orf1	glutathione S-transferase 1-1 [Ostrinia furnacalis]	2	233	26.7	6.74	16	2	2	High	1	1.022	0.96	0.644	0.66	0.681	0.681	0.694	0.683	0.445	0.498	0.542	1.318	1.283	1.209
TRINITY_DN36928_c0_g1_i5_orf1	actin-interacting protein 1 isoform X2 [Ostrinia furnacalis]	3	94	10.3	8.19	38	4	1	High	1	1.04	1.027	0.426	0.583	0.433	0.903	0.876	0.987	0.918	0.94	0.953	0.634	0.706	0.645
TRINITY_DN6391_c0_g1_i1_orf1	secretory carrier-associated membrane protein 1 [Ostrinia furnacalis]	2	342	38.1	7.39	11	2	2	High	1	1.026	1.06	1.154	1.19	1.16	1.25	1.522	1.252	1.374	1.238	1.282	1.1	1.091	1.154
TRINITY_DN9740_c0_g1_i4_orf1	aldehyde dehydrogenase, mitochondrial [Bicyclus anynana]	2	148	16.4	9.35	18	2	2	High	1	0.871	0.885	1.32	1.846	1.215	1.465	1.512	1.46	1.065	1.141	1.63	1.733	1.603	1.889
TRINITY_DN26853_c0_g1_i1_orf1	astacin-like metalloprotease toxin 5 [Ostrinia furnacalis]	2	319	37	4.89	8	2	2	High	1	0.911	1.014	0.79	0.796	0.799	0.777	0.993	0.828	0.791	0.907	0.82	0.703	0.661	0.697
TRINITY_DN76283_c0_g6_i1_orf1	fatty acid synthase-like [Ostrinia furnacalis]	1	851	95	6.62	2	2	1	High	1	0.972	1.073	0.231	0.266	0.277	0.249	0.335	0.272	0.251	0.27	0.272	0.256	0.259	0.26
TRINITY_DN1616_c0_g1_i3_orf1	U4/U6 small nuclear ribonucleoprotein Prp3 isoform X1 [Ostrinia furnacalis]	3	719	81.1	9.61	4	3	3	High	1	1.061	0.892	0.717	0.826	0.854	0.775	0.704	0.664	0.891	0.742	0.726	1.279	1.165	1.137
TRINITY_DN143532_c0_g1_i1_orf1	3-oxoacyl-[acyl-carrier-protein] reductase FabG-like [Aphidius gifuensis]	1	253	27.2	8.44	5	3	1	High	1	0.962	0.949	0.995	1.174	1.009	1.63	2.169	2.055	4.21	3.593	3.407	0.818	0.847	1.049
TRINITY_DN2497_c0_g1_i2_orf1	protein stunted-like isoform X1 [Colias croceus]	2	61	6.9	11	48	2	1	High	1	1.063	1.067	0.645	0.71	0.649	0.455	0.441	0.483	0.568	0.499	0.616	0.381	0.456	0.45
TRINITY_DN4262_c0_g1_i16_orf1	sperm-associated antigen 7 homolog [Ostrinia furnacalis]	3	236	27	9.04	13	3	3	High	1	0.978	1.028	0.614	0.656	0.704	0.548	0.567	0.618	0.478	0.458	0.457	0.861	0.909	0.777
TRINITY_DN2615_c0_g1_i1_orf1	14 kDa phosphohistidine phosphatase-like [Ostrinia furnacalis]	3	157	17.6	7.4	20	3	3	High	1	1.015	0.972	0.877	0.901	0.931	0.799	0.897	0.914	0.842	0.683	0.897	0.905	1.039	0.858
TRINITY_DN1249_c0_g1_i6_orf1	venom carboxylesterase-6-like [Ostrinia furnacalis]	3	585	64.7	4.63	6	4	1	High	1	0.908	0.866	0.194	0.238	0.149	0.172	0.102	0.16	0.159	0.166	0.048	0.543	0.519	0.533
TRINITY_DN783_c0_g1_i7_orf1	microtubule-associated protein Jupiter isoform X4 [Helicoverpa armigera]	2	228	24	9.66	12	3	2	High	1	0.981	1.072	0.513	0.499	0.488	0.467	0.49	0.525	0.419	0.448	0.47	1.148	1.021	1.049
TRINITY_DN10636_c0_g1_i1_orf1	unnamed protein product [Arctia plantaginis]	3	337	38.3	9.2	9	3	3	High	1	0.843	0.892	0.496	0.495	0.605	0.35	0.572	0.465	0.388	0.584	0.387	0.435	0.447	0.442
TRINITY_DN3456_c0_g1_i1_orf1	protein purity of essence [Ostrinia furnacalis]	4	1257	135.5	6.1	5	4	4	High	1	0.944	0.999	0.819	0.847	0.881	0.876	0.875	0.827	0.867	1.004	0.908	1.036	0.92	1.035
TRINITY_DN67623_c0_g1_i1_orf1	maltase A1-like [Ostrinia furnacalis]	4	604	68.6	5.11	9	5	4	High	1	1.024	1.01	0.18	0.178	0.192	0.189	0.223	0.208	0.189	0.172	0.206	0.186	0.188	0.192
TRINITY_DN3456_c0_g2_i1_orf1	protein purity of essence [Ostrinia furnacalis]	3	712	76.4	5.15	4	4	3	High	1	0.949	1.024	0.688	0.779	0.86	0.757	0.764	0.732	1.087	0.91	0.991	0.855	0.843	0.817
TRINITY_DN1027_c0_g1_i8_orf1	phosphatidylinositol 3-kinase catalytic subunit type 3 isoform X1 [Ostrinia furn	3	917	104.4	7.06	4	3	3	High	1	0.693	0.788	0.722	0.733	0.721	0.72	0.902	0.861	0.789	0.816	0.761	0.763	0.851	0.83
TRINITY_DN4767_c0_g1_i6_orf1	cysteine protease XCP2-like [Ostrinia furnacalis]	2	45	5.3	8.5	44	3	2	High	1	1.013	0.971	2.985	3.311	3.033	4.81	5.06	4.562	6.573	5.483	7.085	5.013	5.367	5.031
TRINITY_DN9489_c0_g1_i5_orf1	interferon-related developmental regulator 2 [Ostrinia furnacalis]	3	434	47.1	6.57	10	3	3	High	1	0.94	1.076	0.88	0.928	1	0.859	1.097	0.991	1.031	1.102	1.03	0.862	0.852	1.011
TRINITY_DN6747_c0_g1_i7_orf1	retinol dehydrogenase 12-like [Ostrinia furnacalis]	3	313	34.2	8.88	8	3	3	High	1	0.964	0.979	0.368	0.365	0.418	0.312	0.29	0.296	0.25	0.262	0.28	0.343	0.354	0.348
TRINITY_DN3975_c0_g1_i10_orf1	modular serine protease-like [Ostrinia furnacalis]	2	274	29.8	7.91	10	3	2	High	1	1.046	1.15	1.244	1.422	1.097	1.484	1.37	1.303	1.268	1.281	1.383	1.503	1.463	1.161
TRINITY_DN1924_c0_g1_i3_orf1	type 1 phosphatidylinositol 4,5-bisphosphate 4-phosphatase isoform X1 [Ostri	3	235	25.3	8.7	18	3	3	High	1	0.923	0.979	1.014	0.922	0.909	1.064	1.072	1.099	1.072	1.242	1.03	1.065	1.124	1.09
TRINITY_DN57462_c0_g1_i1_orf1	glutathione S transferase-S5 [Glyphodes pyloalis]	2	80	9.2	5.45	28	12	1	High	1	1.231	0.942	0.443	0.399	0.505	0.579	0.645	0.659	0.586	0.787	0.469	0.641	0.744	0.757
TRINITY_DN4449_c0_g2_i1_orf1	calcium/calmodulin-dependent protein kinase type 1 isoform X3 [Cephus cinct	3	432	49	7.02	10	3	3	High	1	1.058	0.998	1.739	2.075	1.803	1.967	2.22	1.961	2.007	1.918	2.068	1.918	2.06	2.044
TRINITY_DN9280_c0_g1_i1_orf1	stromal cell-derived factor 2 [Ostrinia furnacalis]	3	225	25	7.17	23	3	3	High	1	0.879	0.906	0.943	0.832	0.922	0.705	0.79	0.745	0.849	0.76	0.896	0.747	0.797	0.753
TRINITY_DN3929_c0_g3_i3_orf1	Glutathione S-transferase 1, isoform D [Papilio machaon]	2	107	12.3	5.91	23	2	1	High	1	1.034	1.02	0.369	0.381	0.409	0.538	0.512	0.502	0.303	0.32	0.334	0.289	0.246	0.289
TRINITY_DN9724_c0_g1_i4_orf1	ras family domain-containing protein [Phthorimaea operculella]	2	224	25.5	8.09	9	3	2	High	1	0.961	0.991	0.942	0.976	1.001	0.854	0.865	0.915	0.757	0.807	0.803	0.839	0.853	0.878
TRINITY_DN70236_c0_g1_i1_orf1	NAD-dependent protein deacetylase sirtuin-2-like [Ostrinia furnacalis]	4	408	46.2	5.33	11	4	4	High	1	1.059	0.992	1.01	1.124	1.081	1.066	1.136	0.963	0.796	0.777	0.838	0.914	0.827	0.897
TRINITY_DN1749_c0_g1_i1_orf1	putative GPI-anchored protein pfl2 isoform X2 [Ostrinia furnacalis]	2	534	59	4.87	6	2	2	High	1	1.069	0.891	1.104	1.029	1.051	1.14	1.078	1.002	0.971	0.802	0.943	3.429	3.875	3.545
TRINITY_DN3753_c0_g1_i7_orf1	very-long-chain 3-oxoacyl-CoA reductase isoform X2 [Ostrinia furnacalis]	3	313	34.9	9.39	11	4	3	High	1	1.08	1.045	0.686	0.675	0.695	0.633	0.655	0.645	0.688	0.741	0.691	0.788	0.753	0.784
TRINITY_DN69307_c0_g1_i6_orf1	hypothetical protein evm_010738 [Chilo suppressalis]	2	87	9.5	5.45	29	5	1	High	1	0.97	0.977	1.573	1.765	1.74	2.356	2.447	2.461	2.504	2.357	2.762	1.563	1.757	1.721
TRINITY_DN27721_c1_g1_i2_orf1	mitochondrial import receptor subunit TOM20 homolog [Ostrinia furnacalis]	2	154	17.2	8.02	15	2	2	High	1	0.783	0.816	0.338	0.337	0.378	0.373	0.342	0.339	0.31	0.277	0.356	0.373	0.405	0.426
TRINITY_DN4217_c0_g1_i2_orf1	hypothetical protein evm_006611 [Chilo suppressalis]	3	1479	168	6.51	2	3	3	High	1	1.019	1.096	1.261	1.255	1.255	1.198	1.263	1.271	0.991	1.362	1.18	1.89	1.992	1.926
TRINITY_DN40_c0_g1_i3_orf1	trypsin CFT-1-like [Ostrinia furnacalis]	4	260	28.1	8.18	18	4	4	High	1	0.983	1.019	0.229	0.286	0.324	0.289	0.321	0.293	0.332	0.328	0.281	0.329	0.324	0.331
TRINITY_DN9542_c0_g1_i4_orf1	NAD(P)H-hydrate epimerase [Ostrinia furnacalis]	3	267	28.9	7.66	16	3	3	High	1	1.106	1.068	1.706	1.775	1.692	1.813	1.905	1.869	1.728	1.867	1.782	1.852	1.751	1.776
TRINITY_DN10701_c0_g2_i2_orf1	synaptosomal-associated protein 29 [Ostrinia furnacalis]	3	271	29.9	5.74	11	5	3	High	1	0.883	0.937	0.594	0.594	0.597	0.53	0.479	0.494	0.509	0.404	0.549	0.673	0.714	0.636
TRINITY_DN38366_c0_g1_i4_orfp1	TRINITY_DN38366_c0_g1_i4_m.10666 TRINITY_DN38366_c0_g1::TRINITY_DN38	1	142	13.5	3.68	8	4	1	High	1	1.149	0.99	0.947	1.058	0.865	0.96	0.997	0.939	0.652	0.567	0.758	0.28	0.335	0.328
TRINITY_DN472_c1_g1_i3_orf1	IST1 homolog isoform X1 [Ostrinia furnacalis]	5	380	42.5	6.15	10	6	5	High	1	0.951	1.004	1.739	1.5	1.532	1.548	1.301	1.6	1.451	1.335	1.256	1.343	1.357	1.432
TRINITY_DN23502_c0_g1_i1_orf1	small nuclear ribonucleoprotein F [Ostrinia furnacalis]	4	88	9.7	4.81	31	5	4	High	1	0.992	1.013	0.723	0.771	0.741	0.567	0.563	0.551	0.464	0.478	0.494	0.786	0.747	0.887
TRINITY_DN21341_c0_g1_i1_orf1	FAST kinase domain-containing protein 4 isoform X6 [Ostrinia furnacalis]	2	392	42.8	9.48	8	2	2	High	1	0.925	1.007	0.98	0.882	0.938	0.932	0.93	0.92	1.694	2.214	1.914	1.035	0.951	1.046
TRINITY_DN16972_c0_g1_i1_orf1	aldo-keto reductase family 1 member A1-like [Ostrinia furnacalis]	3	324	35.7	7.27	10	3	3	High	1	0.959	1.012	1.027	1.073	1.053	0.966	1.175	1.056	0.889	1.49	0.903	1.203	1.244	1.165
TRINITY_DN30070_c0_g1_i6_orf1	uncharacterized protein LOC114361440 isoform X1 [Ostrinia furnacalis]	2	485	55	8.82	7	2	2	High	1	1.04	1.096	0.464	0.405	0.482	0.514	0.573	0.611	0.661	0.576	0.513	0.511	0.53	0.634
TRINITY_DN7868_c0_g1_i8_orf1	uncharacterized protein LOC114353432 isoform X4 [Ostrinia furnacalis]	2	486	55.8	6.8	5	2	2	High	1	0.939	0.934	0.677	0.707	0.716	0.661	0.678	0.737	0.592	0.558	0.482	0.668	0.659	0.75
TRINITY_DN4708_c0_g1_i5_orf1	GTP-binding protein 1 [Ostrinia furnacalis]	3	608	66.9	8.68	6	4	3	High	1	0.968	0.925	0.989	0.954	0.93	0.995	1.071	1.05	0.956	1.704	0.944	1.222	1.197	1.33
TRINITY_DN5444_c0_g2_i1_orf1	phenoloxidase-activating factor 2-like [Ostrinia furnacalis]	3	252	27.9	6.84	14	3	3	High	1	1.004	1.061	1.434	1.28	1.33	1.388	1.281	1.469	1.244	1.09	1.25	3.511	3.521	3.365
TRINITY_DN9324_c1_g2_i2_orf1	kinesin-like protein Klp10A isoform X4 [Spodoptera frugiperda]	4	770	85.6	8.65	5	4	4	High	1	1.084	1.095	0.888	1.042	0.919	0.938	0.912	0.875	0.967	1.012	1.043	1.332	1.537	1.245
TRINITY_DN34426_c0_g1_i1_orf1	laminin subunit alpha-like, partial [Ostrinia furnacalis]	2	78	9.1	7.91	47	2	2	High	1	0.894	0.924	0.887	0.907	0.924	0.977	0.891	1.053	1.331	1.144	1.177	1.205	1.165	1.32
TRINITY_DN4602_c0_g1_i4_orf1	2-iminobutanoate/2-iminopropanoate deaminase [Ostrinia furnacalis]	2	173	18.2	6.54	22	2	2	High	1	1.034	1.07	1.406	1.517	1.504	1.9	1.911	1.786	1.882	1.735	1.645	2.218	1.911	2.216
TRINITY_DN25680_c0_g1_i1_orf1	signal peptidase complex subunit 1 [Papilio machaon]	3	93	10.6	9.74	20	4	3	High	1	1.045	1.117	1.127	1.099	1.229	1.111	1.156	1.073	1.006	0.972	1.056	1.058	1.105	1.005
TRINITY_DN172_c1_g1_i3_orf1	galactose oxidase, central domain-containing protein [Phthorimaea operculell	3	491	54.2	5.6	6	3	3	High	1	0.788	0.907	0.561	0.7	0.676	0.754	0.693	0.682	0.646	0.705	0.683	0.883	0.727	0.838
TRINITY_DN13167_c0_g1_i1_orf1	selenoprotein M-like [Ostrinia furnacalis]	2	110	12.4	6.64	26	2	2	High	1	1.064	0.987	0.858	0.907	0.908	1.12	1.169	1.086	0.888	0.79	0.886	1.42	1.596	1.39
TRINITY_DN5595_c0_g1_i1_orf1	keratin, type I cytoskeletal 10-like [Ostrinia furnacalis]	1	170	16.6	7.12	14	1	1	High	1	0.946	0.961	1.171	0.981	0.794	0.903	1.156	0.857	0.819	0.836	0.731	3.938	4.345	3.971
TRINITY_DN135679_c0_g1_i1_orfp	TRINITY_DN135679_c0_g1_i1_m.85524 TRINITY_DN135679_c0_g1::TRINITY_DN	3	54	6.1	8.54	46	8	1	High	1	0.972	1.109	0.808	0.888	0.855	1.044	1.079	1.031	0.559	0.622	0.569	0.79	0.731	0.634
TRINITY_DN29717_c0_g1_i2_orf1	myotubularin-related protein 4-like isoform X1 [Spodoptera frugiperda]	3	854	95.1	6.42	6	3	3	High	1	1.016	1.08	0.843	0.917	0.913	1.121	1.036	0.924	0.866	0.944	0.867	0.987	1.045	0.976
TRINITY_DN5022_c0_g1_i4_orf1	syntenin-1-like [Ostrinia furnacalis]	4	286	30.8	8.09	20	4	4	High	1	1.15	1.144	2.222	2.168	2.054	2.225	2.079	2.331	1.888	1.706	1.932	2.492	2.632	2.22
TRINITY_DN18728_c0_g1_i2_orf1	H/ACA ribonucleoprotein complex subunit 3 [Galleria mellonella]	2	64	7.6	9.7	53	4	2	High	1	1.04	1.014	0.616	0.648	0.67	0.615	0.622	0.644	0.564	0.595	0.596	0.626	0.623	0.553
TRINITY_DN71610_c0_g1_i1_orf1	GPI transamidase component PIG-T [Ostrinia furnacalis]	3	555	62.3	8.25	5	3	3	High	1	0.958	0.943	0.965	1.071	1.02	0.989	1.051	1.101	0.933	0.874	0.914	1.134	1.15	1.17

TRINITY_DN8714_c0_g1_i6_orf1	stimulator of interferon genes protein isoform X1 [Nymphalis io]	3	355	40.6	6.62	9	3	3	High	1	1.074	1.141	1.337	1.244	1.341	1.287	1.203	1.158	1.263	1.196	1.088	1.344	1.192	1.316
TRINITY_DN16084_c0_g1_i4_orf1	synaptobrevin homolog YKT6 [Ostrinia furnacalis]	2	198	22.6	7.53	15	2	2	High	1	1.018	1.06	0.901	0.887	0.901	0.848	0.846	0.845	0.897	1.02	1.065	0.952	0.907	0.925
TRINITY_DN558_c0_g1_i4_orf1	nucleoporin SEH1 isoform X1 [Ostrinia furnacalis]	3	374	41.2	7.25	10	4	3	High	1	1.045	1.039	0.98	0.944	0.962	1.086	1.267	1.058	0.999	1.072	0.901	0.913	0.836	0.842
TRINITY_DN2718_c0_g1_i6_orf1	cleavage stimulation factor subunit 2 isoform X1 [Ostrinia furnacalis]	3	387	42.8	6.42	8	4	3	High	1	1.016	1.064	0.571	0.578	0.618	0.486	0.514	0.534	0.496	0.445	0.471	0.867	0.88	0.853
TRINITY_DN3733_c0_g1_i1_orf1	60S ribosomal protein L37, partial [Papilio machaon]	4	99	11.4	11.66	30	5	4	High	1	1.079	0.955	0.379	0.379	0.405	0.486	0.411	0.417	0.248	0.211	0.31	0.426	0.406	0.392
TRINITY_DN15157_c0_g1_i1_orf1	UDP-glycosyltransferase UGT40AM2 [Ostrinia furnacalis]	3	528	60.1	8.76	7	3	3	High	1	0.862	0.903	1.522	1.281	1.301	1.815	1.561	1.751	1.275	1.346	1.178	1.128	1.183	1.177
TRINITY_DN6098_c1_g1_i5_orf1	unnamed protein product, partial [Iphiclides podalirius]	2	72	7.7	5.07	28	8	2	High	1	1.023	0.985	2.741	2.901	2.686	3.381	2.409	2.858	2.962	2.529	3.354	1.621	1.774	1.574
TRINITY_DN24164_c0_g1_i1_orf1	PREDICTED: ras-related protein Rab6 [Microplitis demolitor]	2	209	23.6	5.53	11	5	1	High	1	0.978	1.006	0.721	0.748	0.738	0.814	0.8	0.784	0.732	0.791	0.593	0.645	0.776	0.708
TRINITY_DN9765_c0_g1_i6_orf1	hypothetical protein evm_005049 [Chilo suppressalis]	2	406	46.1	6.67	7	3	2	High	1	0.915	0.92	0.758	0.85	0.867	0.707	0.805	0.842	0.724	0.777	0.719	0.995	0.886	1.036
TRINITY_DN2778_c0_g1_i5_orf1	hypothetical protein evm_001346 [Chilo suppressalis]	3	766	85	5.54	4	3	3	High	1	1.026	1.12	0.762	0.727	0.732	0.721	0.687	0.639	0.652	0.694	0.626	0.769	0.812	0.682
TRINITY_DN3383_c0_g1_i5_orf1	uncharacterized protein LOC114357426 [Ostrinia furnacalis]	4	320	35.5	4.97	14	7	4	High	1	0.983	1.032	0.961	0.926	0.899	1.618	1.723	1.567	2.419	2.141	2.426	2.105	2.036	2.094
TRINITY_DN2708_c0_g1_i6_orf1	calpain-A isoform X2 [Nymphalis io]	4	848	95.7	4.78	6	4	4	High	1	1.049	1.02	0.976	1.147	1.027	0.994	0.968	1.035	1.04	1.047	0.95	0.987	1.109	0.951
TRINITY_DN59335_c0_g1_i2_orf1	peroxisomal acyl-coenzyme A oxidase 3 [Ostrinia furnacalis]	3	689	77.6	8.05	6	3	3	High	1	0.931	0.963	0.512	0.592	0.564	0.552	0.556	0.639	0.539	0.59	0.594	0.508	0.512	0.472
TRINITY_DN50237_c0_g1_i8_orf1	LOW QUALITY PROTEIN: uncharacterized protein LOC114361080 [Ostrinia furr	3	109	12.7	9.52	35	3	3	High	1	0.885	1.013	1.339	1.38	1.408	1.251	1.439	1.339	1.647	2.122	1.728	1.582	1.527	1.708
TRINITY_DN104244_c0_g1_i1_orf1	putative GMP synthetase, partial [Cydia pomonella]	2	143	15.7	7.11	17	3	2	High	1	1.013	0.952	1.004	1.024	1.032	0.872	0.851	0.846	0.946	0.805	0.897	1.019	0.896	0.855
TRINITY_DN84478_c0_g1_i8_orf1	uncharacterized protein LOC114359035 isoform X1 [Ostrinia furnacalis]	2	52	5.9	8.51	52	2	1	High	1	0.936	1.078	14.06	14.464	14.167	10.233	10.051	10.087	20.042	16.81	21.746	13.599	14.927	12.827
TRINITY_DN14843_c0_g1_i1_orf1	myotubularin-related protein 13 [Ostrinia furnacalis]	2	1187	131.3	8.02	2	2	2	High	1	1.124	1.204	0.95	1.049	0.976	0.999	0.818	0.882	0.887	0.75	0.947	1.06	0.945	0.977
TRINITY_DN18620_c0_g1_i5_orf1	hypothetical protein evm_011254 [Chilo suppressalis]	2	995	111.2	8.1	3	3	2	High	1	1.069	1.096	1.026	1.008	1.1	1.107	1.179	1.119	1.007	0.863	1.131	1.311	1.432	1.271
TRINITY_DN5140_c0_g1_i1_orf1	ubiquitin thioesterase OTU1 [Ostrinia furnacalis]	4	314	34.6	5.25	12	5	4	High	1	1.024	1.021	0.903	0.824	0.867	0.956	0.976	0.997	0.994	0.903	0.899	0.948	0.925	0.9
TRINITY_DN8676_c0_g1_i1_orf1	probable rRNA-processing protein EBP2 homolog [Ostrinia furnacalis]	3	314	36	10.07	10	3	3	High	1	1.016	0.951	0.45	0.459	0.501	0.491	0.485	0.506	0.488	0.447	0.524	0.465	0.521	0.531
TRINITY_DN1865_c1_g1_i3_orf1	exosome complex exonuclease RRP44 isoform X1 [Ostrinia furnacalis]	3	975	109.7	6.74	3	3	3	High	1	0.827	0.909	0.782	0.817	0.916	0.972	1.058	0.992	1.01	1.041	0.851	0.883	0.84	0.91
TRINITY_DN6532_c2_g1_i1_orf1	nuclear receptor coactivator 5 isoform X1 [Ostrinia furnacalis]	2	433	48	6	6	3	2	High	1	0.976	1.022	1.078	1.134	1.082	1.278	1.176	1.242	1.319	1.392	1.242	1.223	1.169	1.291
TRINITY_DN10785_c0_g1_i4_orf1	alkylglycerol monooxygenase-like [Ostrinia furnacalis]	2	456	53	7.62	7	2	2	High	1	1.212	1.195	0.885	0.806	0.82	0.731	0.754	0.748	0.677	0.737	0.661	0.641	0.578	0.636
TRINITY_DN8958_c0_g1_i1_orf1	nuclear cap-binding protein subunit 1 [Galleria mellonella]	2	799	92.5	6.51	3	2	2	High	1	1.067	1.07	0.734	0.692	0.713	0.6	0.71	0.825	0.559	0.497	0.487	0.867	0.865	0.907
TRINITY_DN70_c6_g1_i1_orf1	optineurin isoform X1 [Ostrinia furnacalis]	3	668	75.3	5.26	6	3	3	High	1	1.009	1.026	1.487	1.449	1.454	1.241	1.38	1.492	1.712	1.639	1.765	1.68	1.66	1.602
TRINITY_DN2673_c0_g3_i1_orf1	uncharacterized protein LOC114361372 [Ostrinia furnacalis]	2	1014	115.7	5.49	2	3	1	High	1	0.937	0.889	0.518	0.508	0.497	0.454	0.68	0.629	0.465	0.503	0.325	0.616	0.588	0.578
TRINITY_DN7040_c0_g1_i4_orf1	uncharacterized protein LOC114353763 [Ostrinia furnacalis]	2	64	7.4	4.59	39	2	2	High	1	0.878	0.955	2.457	2.251	2.373	3.213	2.999	2.806	3.263	2.665	3.396	3	3.445	2.674
TRINITY_DN38341_c0_g2_i2_orf1	delta(24)-sterol reductase-like [Ostrinia furnacalis]	3	342	39.6	8.51	11	3	3	High	1	0.996	1.003	0.664	0.559	0.729	0.609	0.628	0.63	0.681	0.696	0.604	0.582	0.626	0.626
TRINITY_DN33008_c0_g1_i1_orf1	double-stranded RNA-binding protein Staufen homolog 2 isoform X3 [Helicov	2	285	30	9.82	11	2	2	High	1	0.898	0.876	0.73	0.821	0.661	0.65	0.91	0.805	0.774	0.807	0.692	1	0.991	1.198
TRINITY_DN5648_c0_g1_i5_orf1	protein tumorous imaginal discs, mitochondrial-like isoform X2 [Ostrinia furna	3	491	54.3	8.72	7	3	3	High	1	1.023	1.024	0.524	0.579	0.586	0.558	0.56	0.607	0.56	0.484	0.613	0.69	0.692	0.659
TRINITY_DN14874_c0_g1_i6_orf1	uncharacterized protein LOC114358148 [Ostrinia furnacalis]	2	464	52.5	4.92	5	3	2	High	1	1.052	1.02	1.976	1.969	1.92	1.086	1.214	1.163	1.541	1.539	1.338	1.301	1.265	1.23
TRINITY_DN27984_c0_g2_i1_orf1	unnamed protein product [Psylliodes chrysocephala]	2	154	17.6	7.83	19	3	2	High	1	1.067	1.041	1.243	0.993	1.111	1.102	1.14	1.141	1.314	1.248	0.992	1.233	1.293	1.271
TRINITY_DN45633_c0_g1_i1_orf1	ubiquitin thioesterase otubain-like [Ostrinia furnacalis]	3	274	31.4	4.88	11	5	3	High	1	1.026	1.06	0.628	0.485	0.547	0.567	0.586	0.634	0.548	0.536	0.463	0.81	0.868	0.89
TRINITY_DN18922_c0_g1_i1_orf1	LOW QUALITY PROTEIN: CCR4-NOT transcription complex subunit 6 [Ostrinia	3	542	61.5	6.9	6	3	3	High	1	0.932	0.965	0.994	0.972	1.055	0.836	0.946	0.796	0.797	0.786	0.808	1.153	1.268	0.973
TRINITY_DN5880_c0_g2_i2_orf1	macrophage mannose receptor 1 [Bombyx mori]	3	340	38.3	6.29	11	4	3	High	1	0.933	1.003	1.659	1.706	1.56	2.178	2.266	1.958	1.312	1.242	1.414	1.013	1.029	0.984
TRINITY_DN33953_c0_g1_i4_orf1	leucine carboxyl methyltransferase 1 [Ostrinia furnacalis]	3	318	36	5.72	13	3	3	High	1	1.075	1.203	1.286	1.322	1.199	1.302	1.415	1.409	1.324	1.217	1.158	1.103	1.138	1.124
TRINITY_DN6185_c0_g1_i12_orf1	mitogen-activated protein kinase 1 [Ostrinia furnacalis]	4	363	41.8	6.54	10	5	4	High	1	0.887	0.863	0.609	0.625	0.627	0.596	0.67	0.674	0.614	0.674	0.75	0.805	0.805	0.874
TRINITY_DN41311_c0_g2_i3_orf1	ras-related protein Rab-8A isoform X2 [Ostrinia furnacalis]	2	151	17.4	8.82	13	2	2	High	1	1.128	1.041	0.513	0.581	0.638	0.581	0.609	0.55	0.51	0.534	0.556	0.736	0.804	0.857
TRINITY_DN92153_c0_g2_i2_orf1	methylenetetrahydrofolate reductase [Ostrinia furnacalis]	3	272	30.7	6.43	10	3	3	High	1	1.014	0.987	0.967	0.871	0.969	0.614	0.682	0.674	0.667	0.596	0.538	0.836	0.842	0.804
TRINITY_DN13139_c0_g1_i1_orf1	AP-1 complex subunit mu-1 [Ostrinia furnacalis]	3	422	48.5	6.81	10	3	3	High	1	0.998	1.068	0.741	0.712	0.761	0.788	0.76	0.809	0.757	0.815	0.711	0.982	0.912	0.918
TRINITY_DN2579_c0_g1_i7_orf1	aminopeptidase N5 [Ostrinia nubilalis]	3	952	108	5.62	3	3	3	High	1	0.973	0.979	0.348	0.36	0.453	0.366	0.386	0.383	0.357	0.365	0.402	0.377	0.431	0.36
TRINITY_DN2618_c0_g1_i3_orf1	CDP-diacylglycerol--inositol 3-phosphatidyltransferase [Ostrinia furnacalis]	2	220	24.6	7.78	8	4	2	High	1	0.874	0.887	1.493	1.484	1.443	1.988	1.882	1.956	1.885	1.733	1.694	1.382	1.274	1.393
TRINITY_DN49143_c0_g1_i1_orf1	uncharacterized protein LOC107270465 [Cephus cinctus]	5	634	69.1	7.02	7	5	5	High	1	1.045	0.986	1.05	1.062	1.051	1.06	1.064	1.039	1.131	1.118	0.971	3.687	3.705	3.836
TRINITY_DN7434_c0_g1_i1_orf1	bifunctional lysine-specific demethylase and histidyl-hydroxylase NO66 [Ostrir	3	597	68.6	6.47	6	3	3	High	1	0.969	1.036	0.835	0.7	0.822	0.817	0.819	0.878	0.92	1.092	0.817	0.909	0.888	0.97
TRINITY_DN321_c0_g1_i1_orf1	uncharacterized protein LOC126371336 [Pectinophora gossypiella]	2	228	25.8	4.89	15	3	2	High	1	1.097	0.985	1.107	1.141	1.152	1.046	1.42	1.225	0.995	1.071	1.093	1.982	2.028	2.094
TRINITY_DN590_c0_g1_i4_orf1	trypsin inhibitor-like [Ostrinia furnacalis]	2	94	10.4	7.88	31	3	2	High	1	1.015	0.884	0.887	1.087	0.948	0.815	1.136	1.119	1.009	0.882	1.116	1.384	1.481	1.222
TRINITY_DN26789_c0_g1_i2_orf1	D-2-hydroxyglutarate dehydrogenase, mitochondrial-like [Ostrinia furnacalis]	3	490	54.2	7.37	8	3	2	High	1	1.108	1.144	1.251	1.345	1.291	1.374	1.308	1.301	1.78	1.66	1.635	1.255	1.234	1.284
TRINITY_DN1074_c0_g1_i7_orf1	eukaryotic translation initiation factor 4E type 2 [Ostrinia furnacalis]	2	248	27.8	7.66	11	3	2	High	1	0.922	1	0.643	0.582	0.698	0.648	0.655	0.704	0.586	0.562	0.572	1.262	1.194	1.27
TRINITY_DN11376_c0_g2_i1_orf1	cathepsin K-like [Ostrinia furnacalis]	2	472	53.7	5.25	6	3	2	High	1	0.968	0.909	0.191	0.19	0.247	0.247	0.236	0.246	0.223	0.298	0.237	0.239	0.236	0.248
TRINITY_DN2170_c0_g1_i2_orf1	beta-1,3-glucan-binding protein-like [Ostrinia furnacalis]	1	72	8.2	9.72	21	2	1	High	1	0.971	1.076	1.239	1.393	1.235	1.201	1.168	0.999	1.372	1.332	1.312	1.391	1.37	1.535
TRINITY_DN55160_c0_g1_i1_orf1	esterase FE4-like isoform X2 [Ostrinia furnacalis]	2	293	34	6.58	8	4	2	High	1	0.956	0.903	0.531	0.553	0.558	0.566	0.672	0.6	0.542	0.658	0.579	0.484	0.418	0.504
TRINITY_DN48619_c0_g1_i1_orf1	PREDICTED: lysine--tRNA ligase isoform X2 [Fopius arisanus]	3	583	66.7	6.34	5	5	1	High	1	1.001	1.007	0.617	0.641	0.689	0.637	0.486	0.589	0.475	0.453	0.417	0.431	0.431	0.426
TRINITY_DN19821_c0_g2_i4_orf1	uncharacterized protein LOC114359393 isoform X1 [Ostrinia furnacalis]	4	247	29.1	9.44	17	4	4	High	1	0.976	0.917	1.341	1.36	1.326	1.613	1.463	1.557	1.63	3.358	1.533	2.14	2.036	2.161
TRINITY_DN22674_c0_g1_i2_orf1	protein arginine N-methyltransferase 7 isoform X1 [Ostrinia furnacalis]	3	725	81.8	5.49	5	3	3	High	1	1.051	0.986	1.223	0.999	1.086	1.304	1.272	1.423	1.585	1.808	1.317	1.515	1.396	1.398
TRINITY_DN37538_c0_g2_i1_orf1	esterase FE4-like [Ostrinia furnacalis]	3	130	15.9	8.7	31	3	3	High	1	1.059	1.114	0.252	0.274	0.276	0.285	0.327	0.274	0.317	0.34	0.27	0.343	0.351	0.345
TRINITY_DN2076_c0_g2_i1_orf1	macrophage mannose receptor 1-like isoform X1 [Maniola jurtina]	2	314	35.6	6.05	8	2	2	High	1	0.976	1.052	1.615	1.602	1.612	1.754	1.917	1.751	2.562	1.925	1.747	1.543	1.564	1.333
TRINITY_DN2257_c0_g1_i4_orf1	protein phosphatase 1 catalytic subunit [Bombyx mori]	2	328	37.5	6.19	9	2	2	High	1	0.948	0.901	0.561	0.489	0.622	0.601	0.518	0.604	0.768	0.921	0.587	0.674	0.683	0.71
TRINITY_DN49047_c0_g1_i2_orf1	unnamed protein product [Parnassius apollo]	2	74	8.4	10.39	31	4	1	High	1	0.978	0.974	0.93	0.887	0.937	0.769	0.725	0.765	0.606	0.693	0.587	0.891	0.97	0.93
TRINITY_DN13236_c0_g1_i4_orf1	zonadhesin-like isoform X4 [Ostrinia furnacalis]	3	89	10.2	7.36	48	3	2	High	1	1.016	0.94	2.806	3.167	3.105	2.108	1.862	1.757	1.778	1.491	2.032	0.78	0.868	0.816
TRINITY_DN51776_c0_g1_i1_orf1	unnamed protein product, partial [Iphiclides podalirius]	2	111	11.9	4.53	18	3	2	High	1	0.986	0.921	1.203	1.498	1.274	1.699	1.746	1.55	1.597	1.303	1.528	1.008	1.178	0.964
TRINITY_DN971_c0_g1_i10_orfp1	TRINITY_DN971_c0_g1_i10_m.54268 TRINITY_DN971_c0_g1::TRINITY_DN971_c	2	186	20.5	6.87	20	2	2	High	1	0.834	0.503	31.231	35.761	31.133	25.204	24.144	24.205	17.9	15.374	19.162	8.668	8.635	8.256
TRINITY_DN37856_c0_g1_i5_orf1	spermine synthase [Ostrinia furnacalis]	3	372	42.3	5.03	7	3	3	High	1	1.008	1.042	0.848	0.851	0.848	0.706	0.696	0.789	0.711	0.647	0.657	0.683	0.712	0.728
TRINITY_DN2958_c0_g1_i2_orf1	uncharacterized protein LOC114356495 [Ostrinia furnacalis]	3	326	36	5.06	17	3	3	High	1	1.093	1.031	1.176	1.186	1.214	0.927	0.937	1.048	0.786	0.911	1.061	0.749	0.784	0.724
TRINITY_DN9661_c0_g1_i1_orf1	tetratricopeptide repeat protein 37 [Ostrinia furnacalis]	4	1270	145.9	8.16	3	4	4	High	1	1.092	1.114	0.891	0.811	0.967	0.917	1.047	1.608	0.907	0.905	0.737	1.152	0.992	1.148
TRINITY_DN55154_c0_g2_i1_orf1	glycosyl transferase family 8 domain-containing protein [Phthorimaea opercul	1	73	8.3	4.56	26	2	1	High	1	1.003	1.093	1.103	0.89	0.915	1.415	1.262	1.347	1.673	1.565	1.619	0.882	0.818	0.889
TRINITY_DN19690_c0_g1_i1_orf1	unnamed protein product [Chilo suppressalis]	2	84	9.3	8.76	36	2	2	High	1	1.041	1.019	1.522	1.567	1.777	1.822	1.66	2.005	1.656	1.767	1.529	1.776	1.604	1.811
TRINITY_DN9062_c0_g2_i3_orf1	ubiquitin conjugation factor E4 B isoform X2 [Ostrinia furnacalis]	2	423	48.7	6.95	8	2	2	High	1	1.08	1.074	0.704	0.638	0.668	0.674	0.708	0.627	0.675	0.554	0.677	1.066	1.065	1.001
TRINITY_DN17657_c0_g1_i1_orf1	alcohol dehydrogenase 18, partial [Helicoverpa assulta]	3	54	5.7	5.17	61	3	2	High	1	0.973	1.043	1.568	1.443	1.463	1.202	0.854	1.115	1.758	1.392	1.21	0.663	0.66	0.631
TRINITY_DN23774_c0_g1_i1_orf1	hypothetical protein evm_002234 [Chilo suppressalis]	3	387	42	6.33	8	3	3	High	1	1.06	1.047	0.893	0.819	0.865	0.875	0.938	0.945	0.84	0.908	0.839	1.001	0.945	1.02
TRINITY_DN29563_c0_g1_i5_orf1	N-terminal kinase-like protein [Trichoplusia ni]	4	811	90.3	6.57	4	4	4	High	1	0.969	0.97	0.91	0.978	0.952	0.863	0.812	0.819	0.852	0.857	0.883	1.166	1.063	1.066
TRINITY_DN44777_c0_g1_i2_orf1	probable very-long-chain enoyl-CoA reductase art-1 [Ostrinia furnacalis]	2	300	34.2	9.51	7	4	2	High	1	0.839	0.951	1.08	1.364	1.073	0.909	1.036	0.881	0.793	0.969	1.085	1.282	1.121	1.495
TRINITY_DN27556_c0_g1_i1_orf1	bystin [Ostrinia furnacalis]	2	428	49.1	8.09	6	3	2	High	1	0.928	1	0.561	0.557	0.579	0.539	0.554	0.556	0.525	0.62	0.602	0.688	0.703	0.707
TRINITY_DN61048_c0_g1_i2_orf1	PREDICTED: protein THEM6-like [Amyelois transitella]	3	195	22.7	9.01	13	3	3	High	1	0.882	1.008	1.502	1.519	1.392	1.373	1.338	1.317	0.973	1.09	1.16	1.477	1.36	1.51
TRINITY_DN39490_c0_g1_i1_orf1	unnamed protein product [Parnassius apollo]	2	253	26.7	8.13	11	2	2	High	1	1.009	1.091	0.961	0.873	0.859	0.841	0.886	0.781	0.759	0.751	0.84	0.833	0.81	0.838
TRINITY_DN133474_c0_g2_i2_orf1	enoyl-[acyl-carrier-protein] reductase, mitochondrial [Ostrinia furnacalis]	3	362	40	8.98	10	3	3	High	1	1.055	1.066	0.975	0.994	0.988	1.173	1.143	1.263	1.152	1.2	1.39	0.965	0.907	0.951
TRINITY_DN486_c0_g1_i5_orf1	adaptor complexes medium subunit family domain-containing protein [Phthor	3	440	49.9	9.48	8	4	3	High	1	1.069	1.005	1.029	0.981	0.942	0.846	0.815	0.882	0.724	0.726	0.71	1.234	1.162	1.203
TRINITY_DN17045_c0_g2_i3_orf1	unnamed protein product [Diatraea saccharalis]	3	280	30.9	8.1	14	5	3	High	1	0.995	0.962	0.662	0.63	0.692	0.564	0.538	0.549	0.526	0.534	0.462	0.579	0.565	0.667
TRINITY_DN1841_c0_g1_i2_orf1	carboxylesterase [Cnaphalocrocis medinalis]	2	530	58.4	5.96	7	2	2	High	1	0.957	1.108	1.128	1.227	1.221	1.692	1.423	1.5	1.586	1.816	1.922	1.466	1.253	1.281
TRINITY_DN547_c0_g1_i1_orf1	WD repeat-containing protein 43 [Ostrinia furnacalis]	3	447	49.2	9.26	8	3	3	High	1	1	1.018	0.564	0.558	0.596	0.684	0.745	0.683	0.676	0.666	0.708	0.634	0.633	0.632
TRINITY_DN21341_c0_g1_i4_orf1	FAST kinase domain-containing protein 4 isoform X6 [Ostrinia furnacalis]	2	132	15.1	9.14	16	2	2	High	1	0.964	0.901	0.49	0.507	0.508	0.514	0.48	0.585	0.489	0.51	0.506	0.619	0.567	0.515
TRINITY_DN2967_c0_g1_i7_orf1	UDP-glucuronosyltransferase 1-7C-like isoform X2 [Ostrinia furnacalis]	3	406	45.6	8.87	9	3	3	High	1	0.885	0.952	0.637	0.666	0.671	0.814	0.884	0.83	0.708	0.907	0.818	0.705	0.649	0.71
TRINITY_DN95850_c0_g1_i1_orf1	alpha,alpha-trehalose-phosphate synthase [UDP-forming] isoform X2 [Chelon	2	803	90.3	6.67	2	4	2	High	1	1.136	1.022	1.374	1.316	1.423	1.547	1.559	1.569	1.024	0.931	1.047	1.59	1.553	1.651
TRINITY_DN6275_c0_g1_i3_orf1	CTL-like protein 1 isoform X1 [Galleria mellonella]	2	666	75.3	8.24	4	2	2	High	1	0.908	0.923	1.34	1.388	1.482	1.515	1.398	1.534	1.361	1.427	1.245	1.656	1.741	1.767
TRINITY_DN58413_c0_g1_i4_orf1	cysteine and histidine-rich protein 1 isoform X1 [Ostrinia furnacalis]	2	299	34.8	6.6	7	3	2	High	1	1.055	1.114	0.448	0.41	0.415	0.413	0.45	0.448	0.382	0.45	0.419	0.398	0.342	0.371
TRINITY_DN24317_c0_g1_i7_orf1	peptidyl-tRNA hydrolase ICT1, mitochondrial [Ostrinia furnacalis]	3	201	22.9	10.07	16	3	3	High	1	0.956	0.923	0.414	0.419	0.472	0.43	0.523	0.464	0.391	0.388	0.417	0.452	0.488	0.541
TRINITY_DN5472_c0_g1_i1_orf1	serine/threonine-protein kinase 26 isoform X3 [Ostrinia furnacalis]	3	546	61.5	6.89	7	3	3	High	1	0.859	0.891	0.7	0.757	0.741	0.645	0.833	0.855	0.724	0.652	0.744	0.89	0.834	0.836
TRINITY_DN10364_c0_g1_i5_orf1	uncharacterized protein LOC114352615 [Ostrinia furnacalis]	3	430	46.8	5.53	6	4	3	High	1	0.969	0.995	0.996	0.965	0.946	1.137	1.153	1.154	0.997	1.174	1.096	2.142	2.221	2.196
TRINITY_DN5235_c0_g1_i7_orf1	peptidoglycan-recognition protein SA-like [Ostrinia furnacalis]	2	185	20.7	5.53	16	3	2	High	1	0.976	0.992	0.655	0.66	0.651	0.738	0.791	0.765	0.73	0.709	0.668	1.399	1.469	1.385
TRINITY_DN52893_c0_g1_i1_orf1	growth arrest and DNA damage-inducible proteins-interacting protein 1 [Galle	3	251	29.1	9.45	13	3	3	High	1	1.151	1.065	0.561	0.56	0.538	0.543	0.541	0.438	0.471	0.446	0.476	0.582	0.612	0.552
TRINITY_DN69334_c0_g1_i1_orf1	PREDICTED: 15-hydroxyprostaglandin dehydrogenase [NAD(+)]-like [Papilio x	3	268	29.1	7.42	11	3	3	High	1	1.043	1.003	1.276	1.199	1.345	0.421	0.461	0.547	0.43	0.531	0.417	0.493	0.522	0.531
TRINITY_DN6423_c0_g1_i5_orf1	phenoloxidase-activating factor 2-like isoform X2 [Ostrinia furnacalis]	3	229	25.1	5.26	14	3	1	High	1	0.958	1.008	1.296	1.282	1.41	1.375	1.545	1.548	1.498	1.478	1.43	2.358	2.178	2.235
TRINITY_DN28366_c0_g1_i1_orf1	aldehyde dehydrogenase, cytosolic 2-like [Hyposmocoma kahamanoa]	3	89	9.8	5.48	53	3	2	High	1	0.963	0.849	0.761	0.819	0.782	0.96	0.967	0.911	0.832	0.812	0.907	0.825	0.797	0.891
TRINITY_DN81926_c0_g1_i1_orf1	membrane-bound alkaline phosphatase-like isoform X3 [Ostrinia furnacalis]	1	541	59.1	6.18	4	1	1	High	1	1.065	1.101	0.508	0.494	0.521	0.663	0.53	0.687	0.494	0.5	0.427	0.463	0.527	0.387
TRINITY_DN29190_c0_g1_i4_orf1	gloverin-like [Ostrinia furnacalis]	2	109	11.5	9.09	28	3	1	High	1	0.831	1.018	0.754	0.799	0.732	2.872	2.966	2.732	0.813	0.868	0.839	1.381	1.333	1.12

TRINITY_DN21278_c0_g2_i2_orf1	mannose-1-phosphate guanyltransferase alpha-A [Ostrinia furnacalis]	3	444	49.4	9.31	8	5	3	High	1	0.883	0.937	1.527	1.608	1.42	1.687	1.531	1.658	1.49	1.384	1.404	1.234	1.323	1.277
TRINITY_DN15591_c0_g1_i3_orf1	c-Jun NH2-terminal kinase [Danaus plexippus plexippus]	3	445	50.5	8.31	7	3	3	High	1	1.028	1.053	0.967	1.038	0.973	0.954	1.075	1.05	0.935	0.866	0.966	1.195	1.13	1.148
TRINITY_DN10766_c0_g1_i1_orf1	hypothetical protein evm_008559 [Chilo suppressalis]	3	494	56.2	7.46	6	3	3	High	1	1.027	1.012	1.367	1.272	1.235	1.265	1.318	1.239	1.105	1.047	1.03	2.41	2.477	2.297
TRINITY_DN7106_c0_g1_i5_orf1	TBC1 domain family member 15 isoform X5 [Helicoverpa zea]	3	644	73.8	5.34	6	3	3	High	1	1.081	1.047	1.274	1.465	1.329	1.332	1.339	1.352	1.265	2.105	1.301	1.028	1.111	1.141
TRINITY_DN1144_c0_g1_i10_orf1	TIL [Ostrinia furnacalis]	2	91	10.1	5.08	55	2	2	High	1	0.903	0.98	1.397	1.437	1.568	1.781	1.83	1.804	2.195	2.018	2.499	1.691	1.887	1.871
TRINITY_DN17003_c1_g1_i1_orf1	unnamed protein product [Chilo suppressalis]	3	2377	264.9	5.31	2	4	3	High	1	0.995	0.996	0.977	0.97	0.95	0.969	1.154	1.055	0.984	0.912	1.061	1.524	1.592	1.442
TRINITY_DN607_c0_g1_i16_orf1	protein muscleblind isoform X1 [Ostrinia furnacalis]	3	347	38.1	9.6	7	4	3	High	1	1.021	1.013	0.734	0.751	0.702	0.711	0.701	0.74	0.664	0.609	0.704	0.816	0.867	0.841
TRINITY_DN14009_c0_g1_i1_orf1	proline-rich extensin-like protein EPR1 [Manduca sexta]	3	251	26.8	9.26	12	3	3	High	1	1.053	1.018	1.005	0.856	0.995	0.858	0.947	0.944	0.916	0.804	0.778	2.069	2.113	2.157
TRINITY_DN616_c1_g1_i6_orf1	esterase B1-like isoform X1 [Ostrinia furnacalis]	3	589	65.8	6.92	6	3	2	High	1	0.983	0.951	0.923	1.131	1.12	1.222	1.339	1.299	1.251	1.474	1.301	0.677	0.763	0.801
TRINITY_DN17137_c0_g1_i2_orf1	unnamed protein product [Diatraea saccharalis]	3	93	10.5	5.01	34	4	3	High	1	0.959	0.942	1.102	1.186	1.062	1.115	1.195	1.062	0.987	0.88	1.038	0.68	0.761	0.69
TRINITY_DN2709_c0_g1_i4_orf1	ATP-dependent RNA helicase dbp2-like [Ostrinia furnacalis]	2	516	58.7	9.35	4	3	1	High	1	0.903	0.792	0.413	0.544	0.484	0.413	0.526	0.509	0.479	0.546	0.601	0.363	0.332	0.338
TRINITY_DN2012_c0_g1_i3_orf1	contactin [Ostrinia furnacalis]	4	1312	149.7	6.46	3	5	4	High	1	0.952	1.019	1.067	1.047	1.12	1.028	1.109	1.063	1	0.896	1.033	1.402	1.389	1.404
TRINITY_DN2780_c0_g1_i5_orf1	probable ATP-dependent RNA helicase DDX27 [Ostrinia furnacalis]	4	804	90.9	9.58	7	4	4	High	1	1.005	1.025	0.421	0.4	0.383	0.336	0.497	0.396	0.453	0.428	0.453	0.422	0.431	0.476
TRINITY_DN12586_c0_g1_i4_orf1	zonadhesin-like isoform X4 [Ostrinia furnacalis]	2	135	14.3	5.47	26	2	1	High	1	0.873	0.833	3.785	3.513	3.905	2.436	2.324	2.264	3.797	2.813	3.656	0.682	0.853	0.928
TRINITY_DN4121_c0_g1_i1_orf1	uncharacterized protein LOC114358001, partial [Ostrinia furnacalis]	3	933	104.2	8.91	4	3	3	High	1	0.994	0.977	0.56	0.599	0.571	0.476	0.491	0.543	0.422	0.515	0.493	0.431	0.439	0.462
TRINITY_DN52553_c0_g2_i1_orf1	hemocyte protein-glutamine gamma-glutamyltransferase-like [Ostrinia furnac	2	219	25.2	9.94	11	4	2	High	1	1.064	1.232	1.109	0.864	0.911	1.181	1.05	1.153	0.95	1.012	0.834	9.519	9.05	8.031
TRINITY_DN40508_c0_g1_i1_orf1	mRNA turnover protein 4 homolog [Ostrinia furnacalis]	3	244	28.2	7.85	16	4	3	High	1	1.034	0.97	0.499	0.527	0.549	0.63	0.604	0.654	0.605	0.815	0.538	0.676	0.634	0.553
TRINITY_DN7169_c0_g2_i6_orf1	PREDICTED: protein GDAP2 homolog [Amyelois transitella]	2	304	34.6	6.74	8	2	2	High	1	0.967	0.977	1.262	0.956	1.172	1.076	1.1	1.023	1.089	1.059	1.002	1.072	1.123	0.994
TRINITY_DN38211_c0_g1_i1_orf1	Golgi reassembly-stacking protein 2 [Ostrinia furnacalis]	2	448	48.5	5	9	2	2	High	1	1.006	0.99	0.682	0.854	0.805	0.775	0.814	0.968	0.766	0.793	0.739	1.022	1.015	1.115
TRINITY_DN12820_c0_g1_i1_orf1	chromodomain-helicase-DNA-binding protein 7 [Ostrinia furnacalis]	4	2875	323.5	6.79	2	4	4	High	1	1.055	1.03	0.671	0.771	0.765	0.753	0.828	0.784	0.669	0.677	0.713	0.957	0.969	0.886
TRINITY_DN44633_c0_g1_i4_orf1	LDLR chaperone boca [Ostrinia furnacalis]	2	187	21.8	4.89	11	4	2	High	1	1.014	1.029	0.764	0.848	0.798	1.157	1.086	1.086	0.96	0.85	0.96	1.255	1.358	1.139
TRINITY_DN6967_c0_g1_i3_orf1	furin-like protease 2 isoform X2 [Manduca sexta]	3	1293	141.2	6.62	4	3	3	High	1	1.045	0.949	0.967	0.865	0.952	0.979	0.895	0.879	0.959	1.08	1.046	1.118	1.161	1.369
TRINITY_DN11680_c0_g1_i1_orf1	uncharacterized protein LOC114355414 [Ostrinia furnacalis]	3	110	12.5	9.29	25	4	2	High	1	0.946	0.996	1.595	1.433	1.426	1.77	1.701	1.912	1.591	1.612	1.352	1.344	1.314	1.304
TRINITY_DN129863_c0_g1_i1_orf1	protein PFC0760c-like isoform X2 [Ostrinia furnacalis]	3	168	19.3	4.26	21	4	1	High	1	0.995	1.094	4.378	5.136	4.038	2.382	3.081	2.694	2.69	2.561	4.135	3.143	2.883	3.338
TRINITY_DN36928_c0_g1_i2_orf1	actin-interacting protein 1 isoform X2 [Ostrinia furnacalis]	3	94	10.3	8.19	38	4	1	High	1	0.977	0.956	0.793	0.92	0.813	0.72	0.663	0.667	0.641	0.513	0.595	0.692	0.643	0.672
TRINITY_DN76216_c0_g2_i3_orf1	lysosomal alpha-mannosidase isoform X1 [Pieris rapae]	3	105	12	4.75	25	3	1	High	1	0.796	1.097	1.591	1.337	1.776	2.055	1.611	1.758	1.88	3.524	1.666	3.404	3.862	3.633
TRINITY_DN19917_c0_g1_i1_orf1	synaptic vesicle glycoprotein 2B-like isoform X4 [Ostrinia furnacalis]	1	519	57.3	6.16	2	3	1	High	1	0.951	0.952	0.055	0.07	0.063	0.066	0.078	0.065	0.06	0.063	0.062	0.082	0.071	0.076
TRINITY_DN146957_c0_g1_i1_orf1	26S proteasome non-ATPase regulatory subunit 7 [Apis florea]	2	328	37.3	6.76	11	2	2	High	1	0.965	1.093	1.388	1.506	1.406	1.264	1.431	1.206	1.27	1.398	1.587	1.52	1.387	1.531
TRINITY_DN43611_c0_g1_i1_orf1	39S ribosomal protein L41, mitochondrial [Ostrinia furnacalis]	2	162	18.2	9.1	25	2	2	High	1	0.943	0.968	0.265	0.318	0.35	0.282	0.273	0.283	0.271	0.242	0.254	0.293	0.383	0.284
TRINITY_DN3056_c0_g1_i1_orf1	spidroin-2-like [Ostrinia furnacalis]	1	775	76.6	8.13	4	2	1	High	1	1.157	0.98	1.067	1.092	1.208	0.991	1.124	1.371	0.996	0.782	1.02	3.678	3.743	3.658
TRINITY_DN35983_c0_g1_i2_orf1	LIM domain-containing protein jub [Ostrinia furnacalis]	3	717	78.1	6.99	6	3	3	High	1	1.095	0.959	0.944	0.92	0.801	0.815	0.936	0.659	0.677	0.764	0.936	0.852	0.97	0.95
TRINITY_DN2224_c0_g2_i1_orf1	tumor susceptibility gene 101 protein [Ostrinia furnacalis]	3	400	45.3	7.01	10	3	3	High	1	0.978	0.964	0.879	0.85	0.809	0.794	0.858	0.77	0.682	0.645	0.675	0.848	0.878	0.899
TRINITY_DN6470_c0_g3_i2_orf1	trypsin CFT-1-like [Ostrinia furnacalis]	1	255	27.4	8.25	5	2	1	High	1	0.859	0.899	0.73	0.706	0.715	0.85	1.065	1.038	0.963	0.908	0.989	3.514	3.948	3.683
TRINITY_DN32896_c0_g3_i1_orf1	PREDICTED: calcium-binding mitochondrial carrier protein Aralar1 isoform X1	2	670	74.7	8.56	3	2	1	High	1	0.946	0.979	0.721	0.791	0.783	0.85	0.858	0.835	0.829	0.714	0.703	0.698	0.629	0.594
TRINITY_DN2830_c0_g1_i9_orf1	facilitated trehalose transporter Tret1 isoform X3 [Ostrinia furnacalis]	2	524	56	5.6	5	3	2	High	1	0.88	0.959	0.751	0.722	0.807	0.826	0.863	0.895	0.886	0.858	0.815	0.791	0.808	0.729
TRINITY_DN27045_c0_g1_i1_orf1	cytochrome P450 6B5-like [Galleria mellonella]	4	502	57.9	7.55	7	4	4	High	1	1.053	1.059	0.819	0.859	0.916	1.074	0.976	1.098	0.838	0.779	0.684	0.54	0.515	0.561
TRINITY_DN338_c2_g1_i2_orf1	alpha-tocopherol transfer protein-like isoform X1 [Ostrinia furnacalis]	3	312	35.6	7.36	14	4	2	High	1	1.002	0.99	0.543	0.516	0.54	0.492	0.47	0.518	0.558	0.527	0.527	0.629	0.615	0.554
TRINITY_DN2887_c0_g1_i1_orf1	F-box/LRR-repeat protein 4-like isoform X1 [Ostrinia furnacalis]	3	614	69.9	6.35	5	3	3	High	1	1.024	1.102	1.287	1.344	1.352	1.293	1.302	1.421	1.177	1.023	1.124	0.936	0.974	0.987
TRINITY_DN755_c0_g1_i3_orf1	uncharacterized protein LOC114358844 [Ostrinia furnacalis]	2	62	7	9.47	37	2	2	High	1	1.049	1.014	0.465	0.498	0.462	0.512	0.473	0.546	0.478	0.541	0.485	0.569	0.503	0.546
TRINITY_DN817_c0_g2_i4_orf1	phenylalanine--tRNA ligase beta subunit-like [Ostrinia furnacalis]	3	197	21.7	8.02	17	4	3	High	1	0.973	0.908	0.907	0.977	1.01	1.001	0.882	1.04	1.039	1.07	0.876	0.935	0.883	0.993
TRINITY_DN3127_c0_g1_i9_orf1	RNA-binding protein 1 isoform X1 [Galleria mellonella]	3	159	18	9.07	18	5	2	High	1	0.917	0.964	0.536	0.555	0.562	0.485	0.415	0.439	0.476	0.511	0.493	0.578	0.615	0.613
TRINITY_DN4752_c0_g1_i3_orf1	thioredoxin domain-containing protein 9 [Ostrinia furnacalis]	3	212	24.5	6.89	25	3	3	High	1	0.965	1.008	0.681	0.663	0.757	0.77	0.744	0.764	0.737	0.718	0.697	0.766	0.772	0.728
TRINITY_DN20682_c0_g1_i2_orf1	hypothetical protein B5X24_HaOG200252 [Helicoverpa armigera]	3	99	11	4.72	40	3	2	High	1	0.964	0.98	0.606	0.599	0.537	0.526	0.566	0.566	0.493	0.507	0.499	0.427	0.378	0.354
TRINITY_DN34727_c0_g1_i3_orf1	tyrosine-protein kinase Src42A isoform X2 [Trichoplusia ni]	3	507	58.2	6.61	8	3	3	High	1	1.069	1.052	1.068	1.05	1.058	1.063	1.097	1.027	1.046	0.877	0.925	1.684	1.587	1.499
TRINITY_DN37532_c0_g1_i1_orf1	transcription elongation factor S-II [Ostrinia furnacalis]	2	291	32.9	8.78	8	2	1	High	1	0.951	0.919	0.714	0.646	0.711	0.603	0.645	0.705	0.635	0.514	0.542	0.913	0.917	0.852
TRINITY_DN2450_c0_g1_i6_orf1	oxysterol-binding protein-related protein 9 [Manduca sexta]	2	705	79.7	6.57	3	2	2	High	1	0.999	1.025	0.782	0.746	0.737	0.702	0.795	0.755	0.721	0.77	0.727	1.15	1.223	1.208
TRINITY_DN17189_c0_g1_i2_orf1	fibroin heavy chain [Haritalodes derogata]	1	285	28.4	9.14	8	1	1	High	1	1.241	1.233	1.111	0.934	0.97	0.79	0.741	0.798	0.774	0.755	0.809	0.663	0.827	0.681
TRINITY_DN5176_c0_g1_i2_orf1	uncharacterized protein LOC114361931 [Ostrinia furnacalis]	3	423	49.6	8.35	9	3	3	High	1	1.076	1.172	1.133	1.007	1.086	1.073	1.065	1.113	0.958	1.023	0.972	0.919	0.806	0.935
TRINITY_DN11612_c0_g2_i1_orf1	eukaryotic translation initiation factor 5B [Manduca sexta]	2	377	42.8	7.23	8	3	1	High	1	0.847	0.995	1.161	1.233	1.528	0.865	0.728	0.734	0.63	0.658	0.794	0.897	0.853	0.924
TRINITY_DN25896_c0_g1_i6_orf1	60S ribosomal export protein NMD3 [Ostrinia furnacalis]	2	503	57.5	6.74	6	2	2	High	1	0.95	0.977	0.579	0.533	0.511	0.536	0.519	0.469	0.531	0.676	0.519	0.487	0.566	0.572
TRINITY_DN12777_c0_g1_i5_orf1	clathrin light chain isoform X2 [Ostrinia furnacalis]	4	205	23.2	4.94	18	4	4	High	1	0.946	1.031	1.025	1.096	1.027	0.819	0.847	0.809	0.639	0.633	0.8	1.186	1.192	1.164
TRINITY_DN1337_c0_g2_i1_orf1	minor histocompatibility antigen H13 [Ostrinia furnacalis]	2	366	40.4	6.86	7	2	2	High	1	1.012	0.947	0.795	0.686	0.8	0.729	0.7	0.821	0.666	0.63	0.6	0.769	0.754	0.735
TRINITY_DN2232_c1_g1_i3_orf1	protein FAM98A-like [Ostrinia furnacalis]	1	202	21	9.91	13	2	1	High	1	0.897	1.016	0.698	0.859	0.767	0.525	0.569	0.639	0.638	0.465	0.565	0.999	0.862	0.771
TRINITY_DN10095_c0_g1_i5_orf1	uncharacterized protein LOC114361222 [Ostrinia furnacalis]	3	1153	129.6	7.93	3	3	3	High	1	1.039	1.003	1.023	0.993	0.888	1.089	1.038	0.941	0.88	0.837	0.882	1.338	1.297	1.264
TRINITY_DN3667_c0_g1_i4_orf1	coronin-2B-like isoform X2 [Ostrinia furnacalis]	2	348	39.6	8.4	8	2	2	High	1	1.025	0.953	1.101	1.23	1.233	0.526	0.633	0.948	0.796	0.668	0.915	1.112	1.113	1.023
TRINITY_DN18563_c2_g1_i1_orf1	protein held out wings isoform X2 [Diachasma alloeum]	4	338	37	7.23	12	4	4	High	1	1.043	1.04	0.935	0.916	1.016	0.94	0.928	0.967	1.024	1.1	0.924	1.195	1.316	1.287
TRINITY_DN5841_c0_g1_i2_orf1	hypothetical protein evm_011295 [Chilo suppressalis]	2	875	97.6	8.34	3	2	2	High	1	0.905	0.844	0.564	0.632	0.602	0.589	0.625	0.596	0.469	0.498	0.481	0.823	0.707	0.81
TRINITY_DN14856_c0_g1_i1_orf1	upstream activation factor subunit spp27 [Ostrinia furnacalis]	3	219	25.1	5.71	12	4	3	High	1	1.039	1.015	1.359	1.296	1.377	1.284	1.297	1.242	1.171	1.027	1.125	2.093	2.186	2.116
TRINITY_DN4134_c2_g1_i2_orf1	prostatic acid phosphatase-like [Ostrinia furnacalis]	4	404	46.8	6.47	11	4	4	High	1	0.962	1.037	1.041	0.988	1.089	0.961	1.062	0.969	0.957	0.947	0.944	1.393	1.366	1.418
TRINITY_DN2432_c0_g1_i1_orf1	unconventional myosin-XV-like [Ostrinia furnacalis]	3	751	85.4	8.07	5	4	3	High	1	0.973	1.152	1.214	1.459	1.244	1.093	1.266	0.988	1.101	1.092	1.004	1.308	1.491	1.352
TRINITY_DN60821_c0_g1_i1_orf1	nucleolar GTP-binding protein 2 [Ostrinia furnacalis]	2	704	79.9	8.69	3	2	2	High	1	1.052	1.073	0.474	0.37	0.559	0.623	0.651	0.658	0.85	0.833	0.649	0.782	0.84	0.827
TRINITY_DN1826_c0_g2_i4_orf1	glutaminyl-peptide cyclotransferase-like [Ostrinia furnacalis]	3	381	43.7	7.62	8	3	3	High	1	0.925	0.981	0.749	0.691	0.693	0.657	0.687	0.728	0.802	0.754	0.589	0.749	0.755	0.745
TRINITY_DN1884_c0_g2_i2_orf1	phosphotriesterase-related protein [Ostrinia furnacalis]	2	186	20.9	6.15	20	2	2	High	1	1.035	0.978	0.437	0.447	0.542	0.708	0.668	0.59	0.639	0.653	0.642	0.538	0.54	0.466
TRINITY_DN10796_c0_g2_i1_orf1	F-BAR domain only protein 2 [Ostrinia furnacalis]	3	893	99.2	7.9	3	3	3	High	1	0.973	0.99	0.464	0.42	0.539	0.524	0.532	0.48	0.508	0.444	0.506	0.553	0.491	0.54
TRINITY_DN3135_c0_g1_i6_orf1	acanthoscurrin-1-like [Ostrinia furnacalis]	1	334	25	9.79	7	1	1	High	1	1.059	0.917	0.461	0.42	0.444	0.501	0.49	0.49	0.41	0.371	0.501	0.145	0.192	0.184
TRINITY_DN64126_c0_g1_i1_orf1	senecionine N-oxygenase isoform X2 [Galleria mellonella]	2	49	5.4	8.05	51	2	2	High	1	0.906	0.972	1.499	1.386	1.196	1.502	1.574	1.669	1.445	1.512	1.401	0.986	1.077	1.081
TRINITY_DN8310_c0_g2_i1_orf1	uncharacterized protein LOC116773294 [Danaus plexippus plexippus]	3	78	9.3	5.01	46	4	3	High	1	1.018	1.035	1.406	1.446	1.257	1.621	1.6	1.539	1.687	1.78	1.769	0.571	0.677	0.666
TRINITY_DN21609_c0_g2_i1_orf1	translation initiation factor eIF-2B subunit epsilon [Ostrinia furnacalis]	3	359	40.1	4.56	8	3	3	High	1	0.995	0.953	0.616	0.594	0.636	0.566	0.579	0.54	0.453	0.431	0.496	0.638	0.627	0.659
TRINITY_DN10871_c0_g1_i3_orf1	hypothetical protein HF086_013701 [Spodoptera exigua]	2	331	36.6	9.23	6	3	2	High	1	0.966	1.002	0.843	0.946	0.87	0.746	0.799	0.767	0.756	0.813	0.712	0.897	0.982	0.872
TRINITY_DN29369_c0_g1_i1_orf1	uncharacterized protein LOC114351747 [Ostrinia furnacalis]	2	542	61.3	7.59	5	2	2	High	1	1.095	0.999	1.411	1.364	1.336	1.351	1.174	1.254	1.149	1.26	1.348	1.228	1.232	1.231
TRINITY_DN3174_c0_g1_i2_orf1	deoxyribose-phosphate aldolase [Ostrinia furnacalis]	3	315	34.6	8.9	10	3	3	High	1	0.95	0.957	1.06	0.94	0.962	1.03	0.883	0.985	0.931	0.838	0.979	0.995	0.96	0.997
TRINITY_DN41546_c0_g1_i15_orf1	monocarboxylate transporter 12 isoform X8 [Ostrinia furnacalis]	3	769	82.3	5.58	6	3	3	High	1	1.31	0.985	0.639	0.711	0.607	0.66	0.856	0.679	0.657	0.683	0.647	0.589	0.72	0.747
TRINITY_DN4270_c0_g1_i1_orf1	cytochrome b-c1 complex subunit 8-like [Ostrinia furnacalis]	3	82	9.5	9.6	34	3	3	High	1	1.084	1.064	0.993	0.884	1.033	1.165	1.1	1.509	1.421	1.239	1.556	1.496	1.619	1.506
TRINITY_DN83374_c0_g1_i1_orf1	uncharacterized protein LOC114350302, partial [Ostrinia furnacalis]	3	547	63	8.43	7	4	2	High	1	1.037	1.027	0.841	0.812	0.862	0.839	0.927	0.824	0.967	0.811	0.88	2.415	2.282	2.173
TRINITY_DN106_c0_g1_i3_orf1	cytochrome b5-like heme/Steroid binding domain-containing protein [Phthor	2	132	14.9	5.05	23	3	2	High	1	0.952	0.964	0.869	0.843	0.888	1.064	1.103	1.074	1.112	0.982	1.046	0.994	1.113	1.155
TRINITY_DN143852_c0_g1_i1_orf1	60S ribosomal protein L10 [Cotesia glomerata]	4	219	25.2	9.95	11	7	4	High	1	0.903	0.856	0.747	0.907	0.782	0.695	0.731	0.651	0.523	0.588	0.674	0.606	0.563	0.632
TRINITY_DN107288_c0_g1_i2_orf1	methionine-tRNA synthetase, partial [Papilio xuthus]	3	71	7.7	10.07	44	3	1	High	1	0.918	0.922	0.51	0.519	0.606	0.42	0.396	0.44	0.621	0.535	0.749	0.39	0.473	0.359
TRINITY_DN147458_c0_g1_i1_orf1	60S ribosomal protein L5, partial [Cotesia chilonis]	5	321	37	9.86	13	10	1	High	1	1.075	1.143	0.821	0.83	0.759	0.72	0.654	0.726	0.542	0.416	0.587	2.323	2.447	2.271
TRINITY_DN43391_c0_g1_i5_orf1	myrosinase 1-like isoform X2 [Ostrinia furnacalis]	4	514	59.3	6.02	6	4	3	High	1	0.965	1.023	0.241	0.261	0.317	0.277	0.337	0.337	0.346	0.33	0.305	0.273	0.315	0.299
TRINITY_DN22983_c0_g1_i2_orfp1	TRINITY_DN22983_c0_g1_i2_m.10495 TRINITY_DN22983_c0_g1::TRINITY_DN22	3	78	9.2	5.69	21	10	3	High	1	0.973	0.977	0.187	0.188	0.229	0.175	0.184	0.187	0.16	0.155	0.177	0.205	0.206	0.206
TRINITY_DN51480_c0_g1_i1_orf1	uncharacterized protein LOC114361588 isoform X14 [Ostrinia furnacalis]	3	117	12.6	5.48	27	4	3	High	1	1.067	1.104	1.786	1.766	1.923	2.229	2.332	2.421	2.551	2.287	2	1.446	1.452	1.877
TRINITY_DN33248_c0_g1_i1_orf1	elongation factor Ts, mitochondrial isoform X3 [Ostrinia furnacalis]	2	322	35.6	7.37	7	2	2	High	1	1.004	1.005	0.564	0.593	0.622	0.533	0.528	0.581	0.454	0.475	0.485	0.519	0.49	0.504
TRINITY_DN28741_c0_g1_i3_orf1	uncharacterized protein LOC114351652 [Ostrinia furnacalis]	3	1083	123.5	5.16	4	3	3	High	1	1.105	0.99	2.078	2.092	2.231	1.208	1.147	1.18	1.198	1.322	1.222	1.572	1.581	1.465
TRINITY_DN28501_c0_g1_i2_orfp1	TRINITY_DN28501_c0_g1_i2_m.58934 TRINITY_DN28501_c0_g1::TRINITY_DN28	3	97	10.9	8.43	30	4	1	High	1	0.911	1.033	8.157	8.692	7.971	3.149	2.994	3.141	2.822	2.508	2.891	0.765	0.688	0.702
TRINITY_DN2959_c0_g1_i2_orf1	equilibrative nucleoside transporter 1 isoform X1 [Ostrinia furnacalis]	2	487	54.5	7.37	5	2	2	High	1	1.144	1.084	1.07	0.807	1.026	1.101	1.075	1.258	1.044	0.894	0.68	1.109	1.073	1.011
TRINITY_DN38230_c0_g1_i4_orf1	hypothetical protein evm_007803 [Chilo suppressalis]	2	109	12.1	8.5	17	3	1	High	1	1.127	0.986	3.151	2.326	3.452	2.427	1.6	2.122	1.937	1.654	1.294	0.75	0.669	0.894
TRINITY_DN53136_c0_g1_i1_orf1	glutathione S transferase-E4 [Glyphodes pyloalis]	3	218	25.1	6.4	15	4	3	High	1	1.015	1.013	0.528	0.428	0.513	0.414	0.445	0.388	0.461	0.503	0.422	0.4	0.446	0.415
TRINITY_DN37336_c1_g1_i1_orf1	actin-related protein 2/3 complex subunit 2 [Ostrinia furnacalis]	3	285	33	7.66	10	3	3	High	1	1.081	1.094	1.046	0.899	0.935	0.806	0.815	0.978	0.988	0.932	0.836	0.956	0.879	0.934
TRINITY_DN37654_c0_g1_i5_orf1	unnamed protein product [Pieris macdunnoughi]	2	207	22.5	7.11	10	2	2	High	1	1.083	0.998	1.443	1.339	1.46	1.617	1.631	1.786	1.837	1.924	1.202	1.723	1.743	2.026
TRINITY_DN4810_c0_g1_i3_orf1	clathrin interactor 1 isoform X2 [Maniola jurtina]	4	613	66.6	5.85	6	5	4	High	1	1.106	1.018	0.714	0.759	0.674	0.655	0.752	0.676	0.645	0.62	0.67	0.752	0.764	0.705
TRINITY_DN13856_c0_g1_i1_orf1	angiotensin-converting enzyme-like [Ostrinia furnacalis]	3	711	81.3	5.47	5	3	3	High	1	1.106	1.108	1.254	1.304	1.221	1.081	1.199	1.275	1.554	1.482	1.314	1.688	1.701	1.547
TRINITY_DN16911_c0_g1_i3_orf1	conserved oligomeric Golgi complex subunit 3 isoform X1 [Ostrinia furnacalis]	3	479	53.8	7.01	6	3	3	High	1	0.939	0.97	0.92	0.862	0.961	0.811	1.031	0.939	0.899	0.947	0.884	1.022	1.008	0.97
TRINITY_DN48020_c0_g1_i1_orf1	aminopeptidase N4 [Cnaphalocrocis medinalis]	2	136	15.7	8.65	21	3	1	High	1	0.877	0.931	0.262	0.218	0.316	0.335	0.388	0.374	0.297	0.303	0.278	0.27	0.263	0.295
TRINITY_DN8473_c0_g1_i6_orf1	serine/threonine-protein phosphatase 6 regulatory subunit 1 [Ostrinia furnacal	2	1008	110.3	4.7	3	2	1	High	1	0.985	1.113	1.566	1.624	1.62	1.84	2.533	2.047	1.958	2.732	1.909	1.184	1.101	1.394

TRINITY_DN6933_c1_g1_i1_orf1	Chlorophyll a-b binding protein 40, chloroplastic [Trichinella nelsoni]	3	222	24	5.64	8	7	2	High	1	0.929	1.062	0.403	0.409	0.469	0.413	0.401	0.378	0.421	0.417	0.435	0.456	0.482	0.503
TRINITY_DN2701_c1_g1_i6_orf1	protein ELYS-like isoform X10 [Ostrinia furnacalis]	5	2582	286.9	5.41	2	5	5	High	1	0.94	0.935	1.002	1.071	0.973	1.03	1.092	1.048	0.961	1.111	1.216	1.205	1.212	1.204
TRINITY_DN9072_c0_g1_i1_orf1	SET and MYND domain-containing protein 4-like [Ostrinia furnacalis]	3	681	77.6	8.24	5	3	3	High	1	0.975	0.977	0.761	0.71	0.713	0.798	0.832	0.853	0.972	1.029	0.787	1.279	1.181	1.288
TRINITY_DN4002_c0_g1_i1_orf1	activating signal cointegrator 1 complex subunit 3 [Ostrinia furnacalis]	2	2182	249.7	6.93	1	2	2	High	1	0.971	1.018	0.759	0.689	0.679	0.78	0.896	0.876	0.855	0.804	0.782	0.773	0.749	0.715
TRINITY_DN39725_c0_g1_i4_orf1	lysosome membrane protein 2-like [Ostrinia furnacalis]	2	124	13.7	6.77	25	2	2	High	1	1.003	1.001	1.043	1.154	0.913	1.236	1.346	1.112	0.809	0.745	1.071	1.197	1.027	1.234
TRINITY_DN6310_c0_g2_i10_orf1	BAG family molecular chaperone regulator 2 isoform X1 [Ostrinia furnacalis]	3	200	22.6	4.83	23	3	3	High	1	1.029	1.055	1.09	1.298	1.263	1.24	1.208	1.149	1.401	1.48	1.267	1.277	1.164	1.455
TRINITY_DN3022_c0_g1_i1_orf1	uncharacterized protein LOC114360532 [Ostrinia furnacalis]	2	248	27.9	5.43	10	2	2	High	1	1.05	0.867	1.048	1.181	1.005	1.205	1.171	1.163	1.124	1.192	1.154	0.959	1.069	0.961
TRINITY_DN4026_c0_g1_i4_orf1	serine protease [Ostrinia furnacalis]	2	422	46.5	7.34	6	2	2	High	1	1.196	0.911	0.677	0.749	0.714	0.668	0.734	0.655	0.696	0.639	0.731	0.698	0.862	0.936
TRINITY_DN6685_c0_g1_i8_orf1	cleft lip and palate transmembrane protein 1 homolog [Ostrinia furnacalis]	3	625	72.4	6.16	7	3	3	High	1	1.004	1.016	0.465	0.372	0.499	0.364	0.367	0.332	0.351	0.319	0.32	0.5	0.493	0.506
TRINITY_DN4425_c0_g1_i4_orf1	CKLF-like MARVEL transmembrane domain-containing protein 4 [Ostrinia furn	2	183	20.5	9.23	10	3	1	High	1	1.005	1.207	1.446	0.945	1.242	1.204	1.051	1.171	1.147	0.888	0.989	0.939	0.916	0.8
TRINITY_DN4952_c0_g1_i1_orf1	mitogen-activated protein kinase kinase kinase 4 [Ostrinia furnacalis]	3	1165	131.3	6.52	3	4	3	High	1	1.091	0.836	0.936	0.969	1.027	1.003	1.056	1.099	0.935	0.908	1.079	0.898	0.768	0.664
TRINITY_DN17409_c0_g1_i5_orf1	integrin beta-6-like [Ostrinia furnacalis]	2	175	19.6	4.75	14	3	2	High	1	1.186	1.188	0.965	1.034	1.157	1.188	1.247	1.412	1.015	0.87	1.02	0.949	1.016	0.996
TRINITY_DN9406_c0_g1_i5_orf1	proton-coupled amino acid transporter-like protein pathetic [Ostrinia furnacal	2	470	52	7.58	8	2	2	High	1	0.876	0.965	0.973	0.974	0.95	1.041	1.249	1.182	1.115	1.406	0.948	0.744	0.745	0.91
TRINITY_DN2024_c0_g1_i12_orfp1	unnamed protein product, partial [Brenthis ino]	2	406	46	5.92	6	2	2	High	1	0.954	0.961	2.263	2.348	2.277	1.639	1.722	1.814	2.258	1.842	2.461	3.275	3.508	3.25
TRINITY_DN825_c23_g1_i5_orf1	methionine-tRNA synthetase, partial [Papilio xuthus]	3	86	9.5	9.7	36	3	1	High	1	0.738	0.919	1.429	1.31	1.569	0.784	0.926	1.084	0.819	0.608	0.645	0.892	0.667	0.911
TRINITY_DN2497_c0_g1_i1_orf1	protein stunted-like isoform X2 [Vanessa tameamea]	2	57	6.6	10.58	49	2	1	High	1	0.907	0.929	0.586	0.486	0.556	0.796	0.794	0.72	0.74	0.71	0.712	0.737	0.873	0.901
TRINITY_DN133760_c0_g1_i1_orf1	THO complex subunit 7 homolog [Ostrinia furnacalis]	2	204	22.8	4.93	12	2	2	High	1	1.021	0.979	0.684	0.661	0.674	0.797	0.638	0.627	0.71	0.742	0.679	0.847	0.784	0.81
TRINITY_DN7267_c1_g1_i4_orf1	probable pseudouridine-5'-phosphatase [Ostrinia furnacalis]	3	247	27.6	8.68	11	3	3	High	1	1.041	0.99	0.694	0.713	0.733	0.721	0.753	0.734	0.673	0.624	0.645	0.559	0.536	0.562
TRINITY_DN1079_c0_g1_i4_orf1	CD109 antigen-like [Ostrinia furnacalis]	3	1348	146.3	6.39	2	4	3	High	1	1.014	1.032	0.344	0.354	0.375	0.415	0.392	0.43	0.395	0.318	0.393	0.388	0.407	0.401
TRINITY_DN38412_c0_g1_i1_orf1	translation initiation factor eIF-2B subunit alpha [Ostrinia furnacalis]	3	303	33.6	5.99	13	3	3	High	1	1.04	1.114	1.43	1.301	1.413	1.46	1.384	1.394	1.607	1.522	1.581	1.803	1.863	1.894
TRINITY_DN905_c0_g1_i4_orf1	(11Z)-hexadec-11-enoyl-CoA conjugase-like [Ostrinia furnacalis]	2	365	41.9	7.47	8	2	2	High	1	1.161	1.004	0.427	0.42	0.409	0.386	0.323	0.389	0.426	0.367	0.382	0.486	0.437	0.437
TRINITY_DN659_c0_g2_i1_orf1	unnamed protein product [Diatraea saccharalis]	2	58	6	6.52	33	4	2	High	1	1.04	1.019	0.926	1.043	0.986	0.822	0.889	0.86	0.819	0.777	0.786	0.769	0.704	0.744
TRINITY_DN3134_c0_g1_i1_orf1	cytochrome c oxidase subunit 6C-1 isoform X1 [Hyposmocoma kahamanoa]	3	100	11.6	9.1	21	5	3	High	1	1.025	1.052	0.347	0.299	0.36	0.294	0.271	0.302	0.339	0.299	0.297	0.303	0.311	0.339
TRINITY_DN11657_c0_g1_i2_orf1	trehalase-1 [Omphisa fuscidentalis]	3	582	67.2	5.5	5	3	2	High	1	0.958	0.989	0.4	0.408	0.431	0.332	0.37	0.353	0.384	0.447	0.413	0.798	0.781	0.74
TRINITY_DN942_c0_g1_i1_orf1	probable nuclear transport factor 2 isoform X1 [Ostrinia furnacalis]	2	130	14.6	5.15	18	2	2	High	1	1.003	0.966	0.763	0.634	0.751	0.719	0.714	0.753	0.744	0.636	0.617	1.033	1.002	1.007
TRINITY_DN41086_c0_g1_i4_orf1	collagenase-like [Pectinophora gossypiella]	3	327	36.1	6.35	10	3	3	High	1	0.994	0.959	0.051	0.051	0.115	0.111	0.131	0.138	0.096	0.103	0.098	0.095	0.092	0.092
TRINITY_DN4707_c0_g1_i1_orf1	PREDICTED: DNA-directed RNA polymerases I, II, and III subunit RPABC1 [Papil	3	210	24.4	6.15	12	4	3	High	1	0.962	1.099	0.627	0.585	0.623	0.575	0.605	0.629	0.605	0.529	0.542	0.691	0.734	0.762
TRINITY_DN1860_c0_g1_i2_orf1	26S proteasome non-ATPase regulatory subunit 5 [Ostrinia furnacalis]	3	505	56.1	5.41	8	3	2	High	1	0.911	0.932	0.658	0.629	0.594	0.804	0.744	0.837	0.717	0.932	0.665	0.771	0.681	0.751
TRINITY_DN95414_c0_g1_i1_orf1	protein arginine N-methyltransferase 5 [Ostrinia furnacalis]	3	653	73.4	6.54	4	3	3	High	1	1.011	1.002	0.794	0.809	0.815	0.728	0.703	0.77	0.657	0.544	0.6	0.599	0.595	0.607
TRINITY_DN104596_c0_g1_i1_orf1	unnamed protein product [Diatraea saccharalis]	1	102	11.1	4.65	16	3	1	High	1	1.074	1.059	1.41	1.438	1.344	1.505	1.545	1.649	1.703	1.923	1.66	1.504	1.617	1.679
TRINITY_DN6203_c0_g1_i1_orfp1	TRINITY_DN6203_c0_g1_i1_m.72736 TRINITY_DN6203_c0_g1::TRINITY_DN6203	2	92	10.8	4.01	32	2	2	High	1	1.056	1.039	0.799	0.819	0.785	0.807	0.757	0.854	1.765	1.596	1.602	1.268	1.192	1.071
TRINITY_DN2040_c0_g1_i15_orfp1	TRINITY_DN2040_c0_g1_i15_m.4150 TRINITY_DN2040_c0_g1::TRINITY_DN2040	4	279	30.3	9.47	16	5	2	High	1	0.904	0.994	1.246	1.299	1.181	1.268	1.462	1.087	1.231	1.263	1.287	0.458	0.459	0.556
TRINITY_DN2483_c0_g1_i1_orf1	UDP-glucuronosyltransferase 2B10-like [Ostrinia furnacalis]	3	525	59.5	7.17	6	3	3	High	1	0.955	0.972	1.84	1.257	1.662	1.714	1.515	1.885	2.03	1.875	1.531	1.561	1.517	1.533
TRINITY_DN886_c0_g2_i4_orf1	collagenase-like [Ostrinia furnacalis]	2	284	30.5	8.02	8	4	2	High	1	0.977	1.01	0.438	0.456	0.516	0.455	0.51	0.481	0.414	0.416	0.483	0.599	0.605	0.637
TRINITY_DN5375_c0_g1_i1_orf1	DNA topoisomerase 3-beta-1 [Pectinophora gossypiella]	2	651	73.2	7.62	5	2	2	High	1	1.009	0.997	0.84	0.794	0.875	0.84	0.74	0.925	0.925	0.866	0.757	0.972	0.777	0.832
TRINITY_DN46202_c0_g1_i1_orf1	alpha-tubulin N-acetyltransferase 1-like isoform X2 [Ostrinia furnacalis]	3	250	27.6	8.94	12	3	3	High	1	0.892	0.919	1.03	1.043	1.18	0.893	0.982	0.924	0.878	0.908	0.891	1.096	1.094	1.114
TRINITY_DN52649_c0_g1_i6_orf1	twinfilin [Ostrinia furnacalis]	3	343	39.1	7.2	11	3	3	High	1	0.983	0.969	1.182	1.206	1.114	1.279	1.364	1.273	1.255	1.136	1.41	1.127	0.997	1.101
TRINITY_DN13511_c0_g1_i4_orf1	alpha-2-macroglobulin receptor-associated protein isoform X1 [Ostrinia furna	2	360	41.9	8.18	7	2	2	High	1	1.111	1.049	2.45	2.212	1.96	1.974	0.882	1.388	1.784	1.254	2.418	2.614	2.442	1.948
TRINITY_DN13602_c0_g1_i4_orf1	NEDD8 ultimate buster 1-like [Ostrinia furnacalis]	2	564	62.9	5.77	4	2	2	High	1	0.805	1.034	1.27	1.181	1.109	1.132	1.097	1.178	1.385	1.344	1.122	1.041	0.958	1.031
TRINITY_DN920_c0_g1_i4_orf1	glutathione S-transferase omega 2 [Ostrinia furnacalis]	3	283	32.4	6.8	11	4	1	High	1	1.205	1.058	1.576	1.442	1.589	1.025	1.112	0.999	0.701	0.658	0.615	0.711	0.598	0.607
TRINITY_DN9872_c0_g1_i2_orf1	serine protease inhibitor 88Ea-like [Ostrinia furnacalis]	3	412	46.4	5.97	8	3	3	High	1	0.976	1.073	0.877	0.878	0.874	0.891	0.821	0.891	0.957	0.887	0.861	1.42	1.481	1.335
TRINITY_DN3283_c0_g2_i1_orf1	dipeptidyl peptidase 9 isoform X2 [Manduca sexta]	2	659	73.3	5.54	3	3	2	High	1	1.059	1.065	0.886	1.008	0.99	0.805	0.821	0.847	0.812	0.687	0.725	0.794	0.866	0.891
TRINITY_DN8685_c0_g1_i5_orf1	macrophage mannose receptor 1-like [Zerene cesonia]	2	371	40.6	5.85	6	2	2	High	1	1.043	0.959	3.088	3.273	3.251	2.053	1.979	1.912	2.255	2.202	2.567	1.405	1.202	1.321
TRINITY_DN7603_c0_g1_i5_orf1	tetratricopeptide repeat protein 1-like [Ostrinia furnacalis]	2	285	32.7	4.88	8	2	2	High	1	0.994	0.997	0.791	0.853	0.769	0.706	0.813	0.713	0.712	0.729	0.694	1.041	1.119	1.145
TRINITY_DN1116_c0_g1_i6_orf1	RNA exonuclease 4-like [Ostrinia furnacalis]	3	190	21.5	8.24	17	3	3	High	1	1.05	1.066	1.307	1.308	1.339	1.79	1.83	1.784	1	0.999	1.034	1.235	1.214	1.166
TRINITY_DN39_c0_g1_i1_orf1	protein phosphatase PP2A 55 kDa regulatory subunit isoform X5 [Colias croceu	1	393	45.1	5.88	4	2	1	High	1	0.906	0.985	0.993	1.05	0.939	0.932	1.048	1.179	1.096	1.007	0.915	1.054	1.016	0.979
TRINITY_DN125071_c0_g1_i1_orf1	ensconsin-like isoform X4 [Ostrinia furnacalis]	2	98	9.8	12.15	34	2	1	High	1	0.769	0.915	0.594	0.669	0.465	0.618	0.782	0.756	0.606	0.605	0.904	0.715	0.659	0.81
TRINITY_DN1110_c1_g1_i9_orf1	MD-2-related lipid-recognition protein-like [Ostrinia furnacalis]	2	152	17.2	7.4	17	2	2	High	1	0.915	0.993	1.03	0.951	1.072	1.434	1.284	1.413	1.852	1.768	1.879	3.694	3.702	3.818
TRINITY_DN34726_c0_g2_i1_orf1	heat shock factor-binding protein 1 [Ostrinia furnacalis]	1	83	9.5	4.21	13	2	1	High	1	0.983	1.039	0.553	0.585	0.57	0.645	0.65	0.606	0.583	0.516	0.562	0.736	0.738	0.791
TRINITY_DN28311_c0_g1_i2_orf1	RNA polymerase II-associated protein 3-like isoform X1 [Ostrinia furnacalis]	4	516	58.2	8.5	10	4	4	High	1	1.022	1.028	1.222	1.362	1.199	1.108	1.155	1.085	1.175	1.057	1.236	1.021	1.013	1.053
TRINITY_DN1245_c0_g1_i4_orf1	nuclear RNA export factor 1 [Ostrinia furnacalis]	2	614	68.7	9.23	6	2	2	High	1	0.992	1.154	1.261	1.255	1.247	1.448	1.744	1.493	1.221	1.501	1.224	1.016	1.014	1.015
TRINITY_DN3953_c0_g1_i2_orf1	TP53-binding protein 1-like [Ostrinia furnacalis]	3	1234	134.7	6.24	1	3	3	High	1	1.005	0.897	0.673	0.642	0.689	0.735	0.58	0.669	0.673	0.784	0.727	0.954	1.058	1.139
TRINITY_DN22604_c0_g1_i3_orf1	cytochrome P450 6B5-like [Ostrinia furnacalis]	2	94	11	6.55	24	2	1	High	1	0.966	0.858	1.114	1.113	1.035	1.241	1.3	1.263	1.055	1.155	1.287	1.103	1.096	1.225
TRINITY_DN143792_c0_g1_i1_orf1	ras-related GTP-binding protein C [Venturia canescens]	3	392	43.9	4.78	8	3	3	High	1	0.923	0.995	1.275	1.155	1.15	1.049	1.089	1.072	1.302	1.28	1.071	1.301	1.225	1.248
TRINITY_DN5933_c0_g1_i1_orf1	peroxidase-like [Ostrinia furnacalis]	3	652	74.9	7.01	5	4	2	High	1	0.962	1.1	1.388	1.337	1.223	1.437	1.472	1.421	1.424	1.431	1.305	0.991	0.843	0.879
TRINITY_DN291_c0_g1_i2_orf1	DNA replication licensing factor Mcm7 [Helicoverpa armigera]	2	719	81.1	6.95	4	2	2	High	1	0.924	1.08	1.024	0.998	1.084	1.153	1.209	0.943	1.185	1.129	1.291	1.42	1.398	1.562
TRINITY_DN21214_c0_g2_i1_orf1	heat shock protein family A (Hsp70) member 1A [Homo sapiens]	2	413	45.4	5.64	5	5	1	High	1	1.07	1.008	0.583	0.529	0.567	0.488	0.499	0.494	0.549	0.428	0.605	0.574	0.595	0.579
TRINITY_DN25251_c0_g2_i1_orf1	dolichyl-phosphate beta-glucosyltransferase [Ostrinia furnacalis]	2	335	38.2	6.79	7	2	2	High	1	1.061	1.171	1.154	1.114	1.169	0.863	0.948	0.991	0.919	0.908	0.897	0.998	1.126	1.117
TRINITY_DN3627_c0_g1_i7_orf1	spermine oxidase-like isoform X2 [Ostrinia furnacalis]	2	465	51.6	6.28	5	2	2	High	1	0.982	0.989	1.41	1.547	1.508	1.06	0.989	1.224	1.059	1.031	1.034	1.118	1.207	1.179
TRINITY_DN4565_c0_g2_i1_orf1	acid phosphatase type 7 isoform X2 [Ostrinia furnacalis]	2	189	21.7	5.08	15	3	2	High	1	1.015	0.863	2.309	2.149	2.182	1.599	1.713	1.801	1.733	1.939	1.734	1.866	1.899	1.835
TRINITY_DN72859_c0_g1_i1_orf1	hypothetical protein evm_010574 [Chilo suppressalis]	2	446	50.4	8.75	8	2	2	High	1	1.073	1.063	0.823	0.839	0.743	0.801	0.781	0.848	0.669	0.731	0.749	0.936	0.92	1.002
TRINITY_DN63568_c0_g1_i1_orf1	chromatin-remodeling complex ATPase chain Iswi isoform X1 [Ostrinia furnaca	4	1023	118.5	8.5	5	4	4	High	1	0.964	0.964	0.914	0.976	0.98	0.953	1.088	0.974	1	1.014	1.013	1.165	1.144	1.141
TRINITY_DN17312_c0_g1_i1_orf1	mRNA cap guanine-N7 methyltransferase [Ostrinia furnacalis]	2	409	47.5	6.37	5	2	2	High	1	0.989	0.936	0.488	0.548	0.533	0.413	0.43	0.398	0.508	0.392	0.346	0.656	0.7	0.562
TRINITY_DN9575_c0_g1_i1_orf1	uncharacterized protein LOC114351119 [Ostrinia furnacalis]	2	452	49.8	8.43	5	2	1	High	1	0.987	1.058	0.624	0.619	0.873	0.582	0.708	0.733	0.548	0.551	0.438	0.925	0.811	0.833
TRINITY_DN52859_c0_g1_i4_orf1	uncharacterized protein LOC114351423 [Ostrinia furnacalis]	2	193	21.7	5.47	14	2	2	High	1	0.924	1.102	0.675	0.634	0.754	0.905	0.776	1	0.898	1.752	0.698	0.66	0.588	0.755
TRINITY_DN259_c0_g1_i8_orf1	hypothetical protein evm_000095 [Chilo suppressalis]	3	340	39.2	5.71	10	3	3	High	1	0.796	0.818	0.515	0.55	0.567	0.516	0.544	0.547	0.495	0.507	0.431	0.642	0.564	0.6
TRINITY_DN8580_c0_g1_i12_orf1	peroxisomal N(1)-acetyl-spermine/spermidine oxidase-like isoform X1 [Ostrin	3	462	51.3	6.18	8	3	2	High	1	1.03	0.982	1.195	1.148	1.313	1.701	1.464	1.792	1.876	1.603	1.465	1.767	1.965	1.786
TRINITY_DN57454_c0_g1_i4_orf1	translation machinery-associated protein 7 homolog [Zerene cesonia]	2	64	7	10.05	30	5	2	High	1	1.011	1.001	0.521	0.547	0.563	0.545	0.586	0.622	0.657	0.562	0.662	0.688	0.735	0.672
TRINITY_DN36476_c1_g1_i1_orfp1	TRINITY_DN36476_c1_g1_i1_m.70910 TRINITY_DN36476_c1_g1::TRINITY_DN36	2	87	9.5	9.55	21	10	2	High	1	0.942	0.935	8.606	8.881	8.151	7.571	7.45	7.313	5.254	4.346	6.112	2.061	2.092	2.198
TRINITY_DN325_c0_g1_i15_orf1	protein draper-like [Ostrinia furnacalis]	2	473	51.6	5.15	16	5	2	High	1	0.857	0.989	2.765	3.108	2.804	1.085	1.265	1.197	0.896	0.91	1.109	2.003	1.892	1.88
TRINITY_DN4720_c0_g2_i1_orf1	cystathionine gamma-lyase [Ostrinia furnacalis]	2	94	9.9	8.19	32	3	1	High	1	0.852	0.855	0.956	0.849	0.817	0.755	0.906	0.811	0.875	0.814	0.893	0.846	0.933	0.709
TRINITY_DN18091_c0_g1_i5_orf1	probable glutamine-dependent NAD(+) synthetase [Ostrinia furnacalis]	2	741	83.5	6.79	3	2	2	High	1	1.089	1.011	1.016	0.908	1.066	0.919	1.021	1.017	1.023	0.928	0.945	0.967	0.942	1.032
TRINITY_DN19261_c0_g1_i3_orf1	UDP-glucuronic acid decarboxylase 1 isoform X1 [Papilio machaon]	4	502	57.2	8.53	8	4	4	High	1	1.025	1.023	1.462	1.401	1.415	1.383	1.418	1.549	1.404	1.36	1.146	1.216	1.349	1.128
TRINITY_DN3370_c0_g1_i5_orf1	unnamed protein product, partial [Brenthis ino]	4	610	70.3	7.74	6	4	4	High	1	0.958	0.94	0.805	0.823	0.764	0.523	0.566	0.583	0.55	0.526	0.577	0.602	0.7	0.6
TRINITY_DN14220_c0_g1_i1_orf1	U3 small nucleolar ribonucleoprotein protein IMP4 [Ostrinia furnacalis]	2	309	35.6	8.66	9	2	2	High	1	0.97	0.952	0.685	0.723	0.682	0.714	0.83	0.697	0.796	0.852	0.674	0.662	0.692	0.797
TRINITY_DN5001_c0_g1_i4_orf1	uncharacterized protein LOC114356665 [Ostrinia furnacalis]	2	439	48.3	7.36	7	2	2	High	1	1.078	1.151	1.475	1.338	1.42	1.718	2.11	1.829	1.609	2.041	1.687	0.987	0.921	1.179
TRINITY_DN1213_c0_g1_i5_orf1	CAAX prenyl protease 1 homolog [Ostrinia furnacalis]	2	440	50.7	6.7	5	2	2	High	1	0.973	0.981	1.095	1.018	1.198	1.194	1.076	1.405	1.28	1.393	1.018	1.182	1.13	1.02
TRINITY_DN1405_c0_g1_i1_orf1	cyclin-dependent kinase 10 isoform X1 [Ostrinia furnacalis]	3	404	45.2	8.7	8	3	3	High	1	0.984	0.95	0.787	0.936	0.856	0.794	0.782	0.851	0.786	0.764	0.789	1.098	1.152	0.902
TRINITY_DN2352_c0_g1_i15_orf1	polyadenylate-binding protein 1-B-like [Ostrinia furnacalis]	2	142	16.2	4.58	19	2	2	High	1	1.02	1.079	1.21	1.1	1.124	1.046	1.177	1.09	0.959	1.167	1.091	1.548	1.333	1.334
TRINITY_DN4276_c0_g1_i6_orf1	arylsulfatase B [Ostrinia furnacalis]	3	538	59.9	6.58	6	3	2	High	1	1.036	0.999	1.386	1.511	1.475	1.09	1.215	1.239	1.393	1.123	1.656	1.958	1.883	1.672
TRINITY_DN6933_c0_g1_i2_orf1	Chlorophyll a-b binding protein 40, chloroplastic [Trichinella nelsoni]	3	267	28.3	5.3	7	7	2	High	1	0.901	0.963	0.088	0.085	0.121	0.114	0.125	0.101	0.117	0.116	0.139	0.104	0.121	0.127
TRINITY_DN57998_c1_g3_i1_orf1	uncharacterized protein LOC114363305 isoform X2 [Ostrinia furnacalis]	2	204	23.2	7.94	17	6	2	High	1	1.004	1.059	1.321	1.246	1.223	1.334	1.254	1.369	1.37	1.399	1.313	5.008	5.241	4.905
TRINITY_DN67649_c0_g1_i1_orf1	proliferating cell nuclear antigen [Ostrinia furnacalis]	3	260	29	4.78	13	4	3	High	1	1.05	0.911	0.74	0.743	0.741	0.657	0.661	0.745	0.729	0.703	0.669	1.098	1.15	1.152
TRINITY_DN6556_c0_g1_i7_orf1	NFX1-type zinc finger-containing protein 1-like isoform X1 [Ostrinia furnacalis	3	2049	234.4	7.78	2	3	3	High	1	0.958	0.961	0.581	0.556	0.591	0.724	0.721	0.692	0.554	0.549	0.537	0.617	0.599	0.659
TRINITY_DN48023_c1_g1_i1_orf1	carboxylesterase [Ostrinia furnacalis]	3	533	59.4	5.72	8	3	3	High	1	1.003	0.936	1.032	1.025	1.011	1.18	1.205	1.128	1.043	1.213	1.082	1.373	1.352	1.405
TRINITY_DN51429_c1_g1_i1_orf1	ER degradation-enhancing alpha-mannosidase-like protein 3 isoform X1 [Ostı	3	863	96.2	6.18	4	3	3	High	1	1.029	0.953	1.203	1.237	1.271	1.031	1.131	1.233	1.296	1.445	1.3	1.217	1.296	1.342
TRINITY_DN3005_c0_g1_i7_orf1	lachesin-like isoform X3 [Ostrinia furnacalis]	3	430	48.5	5.85	7	4	3	High	1	0.995	1.026	1.2	1.167	1.162	1.272	1.327	1.246	1.35	1.341	1.475	2.224	2.215	2.178
TRINITY_DN2563_c0_g1_i4_orf1	trypsin, alkaline B-like [Ostrinia furnacalis]	2	267	29.5	5.22	13	2	2	High	1	0.866	1.052	0.553	0.56	0.542	0.595	0.638	0.613	0.573	0.86	0.649	0.666	0.59	0.645
TRINITY_DN10455_c0_g1_i2_orf1	actin-related protein 2/3 complex subunit 4 [Plutella xylostella]	2	168	19.8	9.01	13	2	2	High	1	0.972	1.009	0.779	0.713	0.724	0.61	0.616	0.609	0.668	0.607	0.522	0.778	0.77	0.719
TRINITY_DN8366_c0_g1_i4_orf1	luciferin 4-monooxygenase-like [Ostrinia furnacalis]	3	543	61.5	6.8	5	4	3	High	1	0.873	0.917	0.4	0.36	0.363	0.467	0.488	0.473	0.45	0.423	0.448	0.407	0.395	0.386
TRINITY_DN47784_c0_g2_i1_orfp1	arylphorin subunit alpha-like [Ostrinia furnacalis]	3	86	10.1	6.8	27	3	2	High	1	1.037	1.131	6.794	7.224	6.844	4.255	4.407	4.097	2.818	2.386	3.297	2.635	2.79	2.568
TRINITY_DN53311_c0_g2_i1_orf1	transcription elongation factor S-II [Chelonus insularis]	2	313	34.9	8.82	8	2	1	High	1	1.129	1.087	0.464	0.602	0.637	0.594	0.559	0.84	0.582	0.471	0.551	1.103	1.233	1.063
TRINITY_DN29156_c0_g1_i1_orf1	protein FAM136A [Ostrinia furnacalis]	3	147	16.6	7.24	20	4	3	High	1	1.043	1.026	0.657	0.645	0.642	0.579	0.615	0.55	0.524	0.568	0.575	0.519	0.52	0.548
TRINITY_DN5004_c0_g1_i2_orf1	MOXD1 homolog 1-like [Ostrinia furnacalis]	2	729	82.2	6.33	4	2	2	High	1	0.935	0.991	0.753	0.81	0.813	0.912	0.87	0.854	0.891	0.793	0.901	1.056	0.976	1.095

TRINITY_DN22824_c0_g1_i4_orf1	LIM domain and actin-binding protein 1 [Ostrinia furnacalis]	2	85	9.8	8.44	34	2	2	High	1	0.958	0.943	1.476	1.506	1.574	1.172	1.228	1.203	0.889	0.833	0.939	1.042	1.099	0.895
TRINITY_DN1005_c0_g2_i1_orf1	hypothetical protein ILUMI_24512 [Ignelater luminosus]	2	120	13.5	8.51	21	2	1	High	1	0.927	0.97	1.023	0.975	0.974	1.009	0.723	0.963	0.872	0.882	0.726	0.947	1.004	1.042
TRINITY_DN17271_c0_g1_i1_orf1	uncharacterized protein LOC114350693 [Ostrinia furnacalis]	2	139	15.3	4.51	14	2	2	High	1	1.059	1.005	0.59	0.576	0.64	0.569	0.478	0.519	0.444	0.491	0.463	0.965	1.03	0.98
TRINITY_DN2596_c0_g1_i6_orf1	unnamed protein product [Arctia plantaginis]	3	1121	126	8.34	3	3	2	High	1	1.033	0.931	1.248	1.37	1.33	1.248	2.359	1.508	1.476	1.928	1.374	1.061	0.926	0.939
TRINITY_DN19_c0_g1_i8_orf1	protein misato [Ostrinia furnacalis]	3	543	61.9	5.11	7	3	3	High	1	1.002	0.991	0.995	0.874	0.872	0.979	1.061	1.008	0.905	1.072	0.945	1.047	1.046	1.06
TRINITY_DN3838_c0_g1_i8_orf1	ER membrane protein complex subunit 2-like isoform X1 [Ostrinia furnacalis]	2	290	33.3	7.05	8	2	2	High	1	1.003	1.011	0.832	0.799	0.812	0.725	0.652	0.686	0.646	0.621	0.603	0.954	0.87	0.952
TRINITY_DN125521_c0_g2_i1_orf1	seroin transcript 1A2 [Ostrinia nubilalis]	2	72	8.1	7.42	35	4	1	High	1	0.916	1.131	2.444	2.245	2.116	3.607	2.698	2.786	2.581	2.461	3.151	0.74	0.769	0.969
TRINITY_DN2975_c0_g1_i4_orf1	ubiquitin-like protein 4A [Ostrinia furnacalis]	2	149	17	5.47	15	2	2	High	1	1.072	1.06	0.516	0.568	0.558	0.51	0.585	0.641	0.526	0.373	0.511	0.582	0.637	0.614
TRINITY_DN277_c1_g1_i1_orf1	uncharacterized protein LOC114363802 isoform X4 [Ostrinia furnacalis]	2	726	78.1	7.94	3	2	2	High	1	0.991	1.035	0.902	0.812	0.843	0.88	0.874	0.884	0.658	0.588	0.702	1.17	1.137	1.049
TRINITY_DN19186_c0_g1_i1_orf1	39S ribosomal protein L9, mitochondrial [Ostrinia furnacalis]	2	253	29.2	9.6	10	2	2	High	1	0.933	0.978	0.439	0.464	0.483	0.481	0.526	0.542	0.457	0.495	0.455	0.591	0.527	0.547
TRINITY_DN6262_c0_g2_i1_orf1	thrombospondin type-1 domain-containing protein 4-like [Ostrinia furnacalis]	2	623	67.7	8.02	4	2	2	High	1	0.971	1.11	0.896	0.937	1.02	0.73	0.924	0.897	0.744	0.787	0.716	0.866	0.939	0.827
TRINITY_DN23824_c0_g1_i1_orf1	28S ribosomal protein S30, mitochondrial [Ostrinia furnacalis]	2	553	65.3	7.44	3	2	2	High	1	1.13	1.026	0.488	0.455	0.543	0.559	0.568	0.541	0.654	0.52	0.595	0.539	0.539	0.525
TRINITY_DN21609_c0_g1_i1_orf1	translation initiation factor eIF-2B subunit epsilon [Ostrinia furnacalis]	2	340	38.2	6.18	7	2	2	High	1	1.045	1.019	1.231	1.349	1.244	1.276	1.511	1.307	1.179	1.29	1.317	1.266	1.276	1.257
TRINITY_DN45293_c0_g1_i1_orf1	signal peptidase complex catalytic subunit SEC11A [Plutella xylostella]	3	178	20.2	9.13	17	3	3	High	1	1.023	0.945	1.095	0.791	1.052	0.854	1.079	0.909	0.925	0.841	0.707	0.853	0.978	0.986
TRINITY_DN12594_c0_g1_i1_orf1	DNA-directed RNA polymerases I and III subunit RPAC1 [Ostrinia furnacalis]	1	331	37.5	6.55	8	1	1	High	1	1.239	1.158	1.146	1.224	0.973	1.514	1.164	1.065	1.696	1.292	1.543	1.413	0.915	1.231
TRINITY_DN103_c0_g1_i1_orf1	unnamed protein product [Diatraea saccharalis]	2	164	17.8	8.92	18	2	2	High	1	0.951	0.932	1.163	1.048	0.971	1.169	1.421	1.424	1.264	1.094	1.119	1.374	1.374	1.262
TRINITY_DN42964_c0_g1_i1_orf1	protein lethal(2)essential for life-like [Galleria mellonella]	1	207	23.2	6.3	5	2	1	High	1	1.028	1.058	0.729	0.665	0.788	1.9	1.884	1.698	1.062	0.956	0.986	2.447	2.519	2.281
TRINITY_DN892_c0_g1_i9_orf1	calcyphosin-like protein isoform X2 [Ostrinia furnacalis]	2	251	28.2	5.03	10	3	2	High	1	1.04	1.022	0.983	1.03	1.058	0.956	0.936	0.927	0.829	0.782	0.835	1.099	1.081	1.144
TRINITY_DN3301_c0_g1_i2_orf1	hemicentin-2-like isoform X1 [Ostrinia furnacalis]	3	1162	129.9	6.76	3	3	3	High	1	0.943	0.938	1.949	2.169	1.832	2.209	2.176	2.018	2.854	3.352	3.156	2.652	2.71	2.729
TRINITY_DN2840_c0_g1_i5_orf1	hypothetical protein evm_002181 [Chilo suppressalis]	3	152	16.5	8.19	23	4	3	High	1	1.037	0.908	0.474	0.462	0.522	0.552	0.491	0.558	0.462	0.485	0.547	1.553	1.578	1.509
TRINITY_DN1641_c0_g1_i8_orf1	rhodanese domain-containing protein CG4456-like isoform X1 [Ostrinia furna	3	165	18.7	9.22	15	3	3	High	1	1.061	1.058	0.811	0.871	0.859	0.85	0.86	0.764	0.913	0.841	0.905	0.963	0.947	0.978
TRINITY_DN17947_c0_g1_i4_orf1	unnamed protein product [Parnassius apollo]	2	381	42.5	5.25	8	2	2	High	1	1.19	1.036	1.023	1.107	1.411	1.189	1.533	1.46	1.197	0.94	0.928	1.103	1.123	1.236
TRINITY_DN84669_c0_g1_i1_orf1	PREDICTED: microtubule-actin cross-linking factor 1, isoforms 1/2/3/5 [Amyelo	2	100	11.8	9.54	15	4	2	High	1	0.928	0.912	0.873	0.921	0.874	0.907	0.945	0.93	0.691	0.717	0.868	1.056	0.854	0.827
TRINITY_DN3566_c0_g1_i4_orf1	probable nucleoporin Nup54 isoform X2 [Ostrinia furnacalis]	2	572	59.7	9.25	5	2	2	High	1	1.006	1.01	0.978	0.962	1.04	0.976	1.038	0.983	1	1.073	0.769	1.027	1.124	0.942
TRINITY_DN5680_c0_g1_i3_orf1	ribulose-phosphate 3-epimerase [Ostrinia furnacalis]	2	224	24.4	5.83	11	4	2	High	1	0.976	0.944	1.148	1.108	1.176	1.013	0.976	1.046	1.085	0.989	1.135	1.121	1.061	1.115
TRINITY_DN2265_c0_g1_i5_orf1	elongation factor G, mitochondrial [Ostrinia furnacalis]	3	635	71.3	7.15	5	3	3	High	1	1.052	0.973	0.49	0.427	0.543	0.67	0.644	0.731	0.811	0.748	0.524	0.53	0.53	0.525
TRINITY_DN5507_c0_g1_i1_orf1	PREDICTED: protein mago nashi [Amyelois transitella]	2	146	17.3	6.52	21	2	2	High	1	0.971	0.955	0.855	0.751	0.808	1.108	1.414	1.228	1.181	1.395	1.03	0.899	0.885	0.865
TRINITY_DN3027_c0_g1_i4_orf1	ribosome biogenesis protein BOP1 homolog [Ostrinia furnacalis]	4	842	95.8	5.76	6	4	4	High	1	1.158	1.073	0.532	0.534	0.544	0.505	0.539	0.503	0.518	0.507	0.521	0.485	0.472	0.491
TRINITY_DN7920_c0_g1_i2_orf1	uncharacterized protein LOC114357268 [Ostrinia furnacalis]	3	198	22.3	5.12	14	3	3	High	1	0.953	0.985	0.253	0.283	0.303	0.381	0.415	0.452	0.377	0.423	0.378	0.43	0.373	0.349
TRINITY_DN987_c0_g1_i11_orf1	macrophage mannose receptor 1-like [Pieris napi]	2	325	36.1	5.4	8	2	2	High	1	1.021	1.057	0.857	0.826	0.843	0.647	0.647	0.686	0.888	0.947	1.011	0.863	1.016	0.904
TRINITY_DN101325_c0_g1_i4_orf1	endonuclease G, mitochondrial [Ostrinia furnacalis]	2	307	34.5	9.32	9	2	2	High	1	1.044	1.125	0.573	0.685	0.731	0.488	0.628	0.525	0.608	0.683	0.726	0.519	0.705	0.568
TRINITY_DN1612_c0_g1_i3_orf1	immunoglobulin-binding protein 1b [Ostrinia furnacalis]	3	342	38.7	5.25	9	3	3	High	1	1.038	1.013	1.238	1.324	1.372	1.541	1.439	1.364	1.683	1.432	1.463	1.602	1.662	1.582
TRINITY_DN3747_c1_g2_i1_orf1	exportin-5-like, partial [Ostrinia furnacalis]	2	200	22.5	6.33	13	2	2	High	1	0.99	1.123	0.72	0.739	0.885	0.868	1.177	0.812	0.855	0.998	0.917	0.97	0.916	0.972
TRINITY_DN2109_c0_g1_i4_orf1	mucin-2-like isoform X2 [Ostrinia furnacalis]	3	2217	236.3	8.43	2	3	3	High	1	1.008	0.969	0.717	0.769	0.779	0.788	0.83	0.815	1.149	1.321	1.525	2.072	2.172	2.156
TRINITY_DN5954_c0_g1_i2_orf1	myosin-VIIa [Ostrinia furnacalis]	5	2166	249.6	9	2	5	5	High	1	0.892	0.997	0.785	0.805	0.754	0.732	0.761	0.737	0.77	0.744	0.762	1.789	1.561	1.653
TRINITY_DN5478_c0_g2_i2_orf1	venom protease-like isoform X4 [Ostrinia furnacalis]	1	390	42.8	6.34	5	1	1	High	1	0.998	0.894	1.173	1.433	1.035	1.271	1.532	0.977	1.052	0.766	1.016	0.884	1.12	1.03
TRINITY_DN85161_c0_g1_i2_orf1	hypothetical protein evm_013813 [Chilo suppressalis]	2	108	12.2	6.34	29	2	2	High	1	0.949	1.073	1.212	1.332	1.129	1.001	1.028	1.104	1.201	0.985	1.155	0.914	1.097	0.899
TRINITY_DN1763_c0_g3_i2_orf1	heterogeneous nuclear ribonucleoprotein H-like isoform X2 [Ostrinia furnacali	2	357	39.5	6.92	8	3	2	High	1	0.98	0.999	0.633	0.557	0.609	0.738	0.823	0.791	0.492	0.61	0.505	0.969	0.95	0.801
TRINITY_DN2642_c0_g1_i5_orf1	protein LSM12 homolog [Ostrinia furnacalis]	3	186	21.1	7.46	17	3	3	High	1	0.96	1.018	0.778	0.785	0.828	0.7	0.75	0.827	0.638	0.622	0.824	0.697	0.641	0.674
TRINITY_DN23164_c0_g1_i4_orf1	uncharacterized protein LOC114365928 isoform X1 [Ostrinia furnacalis]	2	326	36.1	7.88	7	2	2	High	1	0.998	0.989	1.176	1.129	1.1	1.189	1.19	1.234	1.175	1.284	0.996	0.88	0.913	0.985
TRINITY_DN45477_c0_g1_i1_orf1	putative E3 ubiquitin-protein ligase UBR7 [Ostrinia furnacalis]	2	721	80.9	4.45	4	2	2	High	1	1.058	1.178	0.354	0.32	0.318	0.412	0.463	0.429	0.42	0.324	0.34	0.572	0.5	0.562
TRINITY_DN46369_c0_g1_i3_orf1	unnamed protein product [Chilo suppressalis]	2	407	47.2	5.62	5	2	2	High	1	0.91	0.934	0.932	0.928	0.93	0.91	0.963	0.879	0.861	0.743	0.789	0.816	0.861	0.789
TRINITY_DN22299_c0_g1_i1_orf1	tafazzin homolog [Ostrinia furnacalis]	2	308	34.3	8.53	7	2	2	High	1	1.075	1.078	0.865	1.063	0.979	0.782	1.091	1.07	0.935	1.845	0.835	1.009	0.959	1.122
TRINITY_DN10694_c1_g2_i1_orf1	hsp70-Hsp90 organizing protein 3-like [Ostrinia furnacalis]	3	241	26.9	8.66	15	3	3	High	1	1.018	0.927	0.923	0.904	0.836	0.846	0.817	0.832	0.821	1.031	0.818	1.177	1.223	1.268
TRINITY_DN59042_c1_g1_i1_orf1	nuclear pore complex protein Nup50 [Ostrinia furnacalis]	2	606	64.3	8.47	4	2	2	High	1	0.985	0.978	0.603	0.658	0.611	0.673	0.733	0.637	0.6	0.591	0.591	0.726	0.732	0.74
TRINITY_DN44119_c0_g1_i1_orf1	PREDICTED: GTP-binding protein 128up [Fopius arisanus]	2	367	41	8.78	6	2	2	High	1	0.988	1.112	0.559	0.569	0.594	0.609	0.595	0.56	0.552	0.743	0.532	0.552	0.489	0.541
TRINITY_DN15448_c0_g1_i1_orf1	ragulator complex protein LAMTOR1-like [Ostrinia furnacalis]	1	123	13.4	4.82	20	1	1	High	1	0.989	1.039	0.58	0.517	0.571	0.591	0.494	0.544	0.558	0.578	0.644	0.608	0.632	0.555
TRINITY_DN16011_c0_g1_i3_orf1	hypothetical protein evm_002694 [Chilo suppressalis]	2	200	22.8	6.8	11	2	2	High	1	0.932	0.916	0.939	0.918	0.934	0.829	0.883	0.822	0.781	0.809	0.774	1.146	1.191	1.136
TRINITY_DN8012_c0_g1_i3_orf1	uncharacterized protein LOC114354053 [Ostrinia furnacalis]	2	648	70.4	4.53	4	2	2	High	1	1.05	1.101	0.529	0.566	0.614	0.707	0.582	0.625	0.585	0.788	0.446	0.67	0.697	0.753
TRINITY_DN7277_c0_g1_i1_orf1	uncharacterized protein LOC114363024 [Ostrinia furnacalis]	2	192	22.1	6.92	15	2	2	High	1	0.995	0.998	0.83	0.803	0.818	0.86	0.966	0.819	0.954	0.851	1.024	1.028	1.096	0.974
TRINITY_DN1833_c0_g1_i5_orf1	uncharacterized protein LOC114356866 isoform X3 [Ostrinia furnacalis]	2	116	12.7	5.58	28	2	2	High	1	0.989	1.014	1.09	1.121	1.123	0.799	0.936	1.228	1.614	1.589	1.352	1.465	1.394	1.421
TRINITY_DN33883_c0_g1_i1_orf1	probable 28S ribosomal protein S6, mitochondrial [Ostrinia furnacalis]	2	151	17.7	8.87	17	2	2	High	1	1.039	1.126	0.486	0.461	0.484	0.509	0.525	0.525	0.514	0.599	0.526	0.61	0.565	0.624
TRINITY_DN2338_c3_g2_i3_orf1	charged multivesicular body protein 4b isoform X1 [Ostrinia furnacalis]	2	222	25.2	5.05	11	2	2	High	1	1.043	1.009	0.984	1.013	0.984	1.041	1.115	1.048	1.054	1.046	0.994	0.875	0.874	0.834
TRINITY_DN6668_c0_g1_i4_orf1	UBX domain-containing protein 4 isoform X1 [Ostrinia furnacalis]	2	528	58.1	6.29	4	3	2	High	1	0.913	0.908	0.7	0.701	0.634	0.601	0.665	0.563	0.709	0.662	0.81	0.582	0.6	0.59
TRINITY_DN37165_c0_g1_i4_orf1	pyridoxine-5'-phosphate oxidase-like [Ostrinia furnacalis]	2	254	29.5	8.66	9	2	2	High	1	1.008	1.017	0.52	0.551	0.555	0.618	0.599	0.644	0.486	0.488	0.484	0.594	0.584	0.592
TRINITY_DN34689_c0_g1_i4_orf1	uncharacterized protein DDB_G0283357 isoform X13 [Helicoverpa armigera]	2	582	66	9.23	5	2	2	High	1	1.081	1.012	0.666	0.752	0.658	0.67	0.691	0.645	0.688	0.602	0.673	0.876	0.852	0.856
TRINITY_DN9759_c0_g1_i1_orf1	iroquois-class homeodomain protein IRX-1-like isoform X1 [Ostrinia furnacalis	3	353	39.4	6.18	9	4	3	High	1	0.921	0.982	0.772	0.803	0.814	0.794	0.727	0.745	0.683	0.557	0.624	0.821	0.784	0.7
TRINITY_DN3063_c0_g1_i5_orf1	mini-chromosome maintenance complex-binding protein [Ostrinia furnacalis]	3	404	46.4	5.35	10	3	3	High	1	1.052	0.997	0.711	0.733	0.788	0.643	0.661	0.641	0.546	0.579	0.634	0.692	0.688	0.663
TRINITY_DN33452_c0_g1_i1_orf1	lethal(2) giant larvae protein isoform X8 [Ostrinia furnacalis]	1	123	13.7	10.07	14	1	1	High	1	0.955	0.955	0.076	0.091	0.161	0.538	0.611	0.467	0.327	0.364	0.407	0.211	0.129	0.189
TRINITY_DN2798_c0_g1_i5_orf1	arylsulfatase B-like isoform X1 [Ostrinia furnacalis]	3	597	66.8	6.42	7	3	3	High	1	0.915	0.932	2.155	2.077	1.981	1.803	1.944	1.815	2.231	2.217	2.406	2.431	2.259	2.279
TRINITY_DN11514_c0_g1_i1_orf1	uncharacterized protein LOC114350079 [Ostrinia furnacalis]	4	423	47.7	8.05	9	4	4	High	1	1.157	1.121	1.888	1.894	1.897	1.845	1.908	1.839	1.695	1.539	1.553	2.143	2.041	2.223
TRINITY_DN9316_c0_g3_i1_orf1	cytochrome P450 6k1-like isoform X1 [Ostrinia furnacalis]	3	498	58	8.53	6	3	3	High	1	1.012	1.017	1.528	1.393	1.443	1.278	1.164	1.343	1.157	1.099	1.192	1.223	1.225	1.353
TRINITY_DN12576_c0_g1_i2_orf1	eukaryotic translation initiation factor 4E transporter-like isoform X5 [Hyposmo	2	730	81.9	9.47	4	2	2	High	1	1.075	1.135	0.799	0.902	0.717	0.804	0.921	0.741	0.676	0.803	0.844	1.102	1.116	1.109
TRINITY_DN518_c0_g1_i1_orf1	unnamed protein product [Arctia plantaginis]	3	842	95.9	5.6	5	4	3	High	1	0.976	0.952	0.756	0.765	0.85	0.78	0.792	0.754	0.741	0.789	0.713	0.956	0.963	0.981
TRINITY_DN99063_c0_g1_i1_orf1	microtubule-associated protein futsch isoform X4 [Ostrinia furnacalis]	1	79	9.1	7.34	18	2	1	High	1	0.952	0.949	1.505	1.429	1.406	1.327	1.201	1.329	1.612	0.799	1.047	0.771	0.682	0.617
TRINITY_DN57765_c0_g1_i1_orf1	cytochrome P450 6B6-like [Ostrinia furnacalis]	3	510	58.8	8.5	5	4	1	High	1	1.116	1.166	0.486	0.56	0.53	0.758	0.811	0.694	0.71	0.749	0.71	0.582	0.668	0.63
TRINITY_DN4742_c0_g1_i1_orf1	ribosomal protein S6 kinase beta-1 [Ostrinia furnacalis]	2	452	50.8	6.07	6	2	2	High	1	0.947	0.953	1.05	1.164	0.967	0.946	0.994	0.841	0.756	0.808	0.777	0.916	0.951	0.933
TRINITY_DN3433_c2_g1_i2_orf1	cytosolic purine 5'-nucleotidase isoform X4 [Ostrinia furnacalis]	2	97	10.9	6.8	32	3	2	High	1	0.96	0.818	1.726	1.609	2.126	1.855	1.512	1.457	1.614	1.344	1.146	1.102	1.053	0.996
TRINITY_DN12873_c0_g1_i3_orf1	proteoglycan 4-like [Ostrinia furnacalis]	2	668	74.7	6.21	5	3	2	High	1	1.11	1.044	1.365	1.467	1.514	1.494	1.681	1.721	1.74	2.018	1.504	1.194	1.105	1.241
TRINITY_DN63152_c0_g1_i7_orf1	microtubule-actin cross-linking factor 1 isoform X21 [Manduca sexta]	2	137	15.8	8.72	13	5	2	High	1	0.969	1.009	1.229	1.273	1.193	1.282	1.337	1.34	1.186	1.176	1.189	0.938	1.017	0.994
TRINITY_DN82017_c0_g1_i5_orf1	carboxylesterase CXE17 [Ostrinia furnacalis]	2	109	11.7	4.28	41	3	1	High	1	1.122	0.893	1.298	1.058	1.002	0.546	0.548	0.902	0.689	0.896	0.723	0.806	0.58	0.807
TRINITY_DN30037_c0_g1_i5_orf1	cytoglobin-1-like isoform X2 [Ostrinia furnacalis]	3	201	23.2	9.57	15	3	3	High	1	0.962	0.965	0.692	0.694	0.785	1.288	1.107	1.332	1.026	1.069	0.927	0.862	0.92	0.924
TRINITY_DN10304_c0_g2_i1_orf1	glycine-rich cell wall structural protein [Ostrinia furnacalis]	2	300	27.6	9.04	16	2	2	High	1	0.909	0.894	0.932	0.992	0.835	0.825	0.979	0.871	0.968	1.207	0.939	2.562	2.642	2.378
TRINITY_DN1738_c0_g1_i5_orf1	baculoviral IAP repeat-containing protein 6-like [Ostrinia furnacalis]	2	758	82.4	5.67	4	2	2	High	1	0.83	0.956	1.016	1.287	1.098	1.379	1.229	1.351	1.481	1.308	1.148	1.335	1.321	1.32
TRINITY_DN2973_c1_g1_i9_orf1	unconventional myosin ID [Ostrinia furnacalis]	3	995	111.9	9.6	3	4	3	High	1	1.007	1.086	0.681	0.632	0.696	0.646	0.694	0.65	0.7	0.723	0.62	0.641	0.632	0.626
TRINITY_DN3699_c1_g1_i1_orf1	surfeit locus protein 4 homolog [Ostrinia furnacalis]	2	270	30.5	8.66	9	3	2	High	1	0.902	1.02	0.94	0.806	0.96	0.876	0.915	1.057	0.952	1.074	0.877	1.064	0.993	1.097
TRINITY_DN4619_c0_g1_i1_orf1	hypothetical protein evm_004860 [Chilo suppressalis]	2	327	37.3	5.19	7	2	2	High	1	0.947	0.921	0.976	0.873	0.994	0.88	0.806	0.805	0.886	0.856	0.787	1.071	0.872	0.939
TRINITY_DN13350_c0_g1_i4_orf1	cap-specific mRNA (nucleoside-2'-O-)-methyltransferase 1 [Ostrinia furnacalis	2	913	103.3	6.19	3	2	2	High	1	1.012	0.967	1.104	1.156	1.044	0.856	0.921	1.03	0.961	1.043	1.028	0.66	0.673	0.627
TRINITY_DN10403_c0_g1_i3_orf1	hypothetical protein evm_000264 [Chilo suppressalis]	2	400	43.9	6.13	6	2	1	High	1	0.857	0.936	0.635	0.735	0.777	0.801	0.932	0.695	0.817	1.047	1.237	1.161	1.373	1.341
TRINITY_DN100_c0_g1_i13_orf1	hypothetical protein O3G_MSEX015273 [Manduca sexta]	2	318	36.8	8.79	6	2	2	High	1	0.986	0.98	0.889	0.868	0.91	0.898	0.893	0.881	0.955	0.849	0.821	0.551	0.509	0.569
TRINITY_DN19537_c0_g1_i1_orf1	disintegrin and metalloproteinase domain-containing protein 10 isoform X1 [C	3	838	93.3	7.43	5	3	3	High	1	1.113	1.11	1.134	1.207	1.261	1.115	1.247	1.248	0.991	1.02	1.051	1.571	1.53	1.432
TRINITY_DN2852_c0_g1_i9_orf1	golgin subfamily A member 4-like [Ostrinia furnacalis]	4	1351	155.2	5.24	3	4	4	High	1	0.94	0.934	0.411	0.441	0.494	0.417	0.392	0.383	0.372	0.364	0.357	0.473	0.469	0.441
TRINITY_DN42705_c0_g1_i3_orf1	multiple inositol polyphosphate phosphatase 1 isoform X1 [Ostrinia furnacalis]	4	484	55.3	6.06	7	4	4	High	1	0.991	1.051	0.869	0.983	0.894	0.716	0.658	0.747	0.705	0.69	0.709	0.76	0.783	0.78
TRINITY_DN10662_c0_g1_i4_orf1	HD domain-containing protein 2 [Ostrinia furnacalis]	2	206	24.1	5.52	10	4	2	High	1	1.037	1.002	0.462	0.482	0.54	0.467	0.525	0.503	0.396	0.386	0.43	0.664	0.66	0.602
TRINITY_DN19521_c0_g1_i1_orf1	sodium/potassium-transporting ATPase subunit beta-1-like isoform X1 [Ostrir	2	296	33.3	5.92	8	2	2	High	1	0.919	0.906	1.578	1.666	1.549	1.32	1.562	1.33	1.227	1.372	1.251	1.264	1.286	1.287
TRINITY_DN24323_c0_g1_i3_orf1	ubiquitin-protein ligase E3A [Ostrinia furnacalis]	3	919	103	5.02	4	3	3	High	1	0.94	0.922	0.818	0.938	0.861	0.757	0.804	0.914	0.781	0.68	0.684	0.901	0.914	0.926
TRINITY_DN6074_c0_g1_i1_orf1	uncharacterized protein C1683.06c-like isoform X1 [Ostrinia furnacalis]	2	348	38.9	6.24	5	2	2	High	1	0.989	0.963	0.18	0.185	0.214	0.172	0.202	0.2	0.237	0.225	0.218	0.279	0.243	0.226
TRINITY_DN756_c0_g1_i11_orf1	calcium-binding protein E63-1 isoform X1 [Ostrinia furnacalis]	2	243	26.2	4.82	12	2	2	High	1	0.946	1.017	0.664	0.696	0.871	0.549	0.752	0.788	0.552	0.623	0.657	0.657	0.624	0.578
TRINITY_DN18794_c0_g1_i5_orf1	hypothetical protein evm_012380 [Chilo suppressalis]	3	798	90.2	6.42	4	3	3	High	1	0.933	0.971	0.68	0.741	0.708	0.6	0.586	0.658	0.727	0.55	0.667	0.815	0.803	0.697
TRINITY_DN72934_c0_g1_i1_orf1	carboxylesterase 8 [Streltzoviella insularis]	2	542	61.5	6.32	5	2	1	High	1	1.291	1.183	0.419	0.483	0.417	0.463	0.42	0.425	0.429	0.555	0.414	0.455	0.518	0.5
TRINITY_DN57348_c0_g1_i4_orf1	facilitated trehalose transporter Tret1-like isoform X1 [Ostrinia furnacalis]	3	634	69.3	8.81	5	3	3	High	1	0.92	1.028	1.237	1.113	1.121	1.283	1.074	1.244	1.036	1.078	1.025	1.455	1.534	1.38
TRINITY_DN63533_c0_g1_i2_orf1	glutathione S-transferase sigma3 [Glyphodes pyloalis]	2	76	8.8	4.88	20	3	2	High	1	0.924	0.905	0.805	0.882	0.855	0.905	0.882	0.91	0.764	0.821	0.58	0.522	0.458	0.587
TRINITY_DN36632_c0_g1_i1_orf1	hypothetical protein evm_011159, partial [Chilo suppressalis]	2	310	34.2	8.46	8	2	2	High	1	1.069	1.198	1.537	1.634	1.504	1.645	1.588	1.602	1.152	1.254	1.209	1.146	1.179	1.24
TRINITY_DN41922_c0_g3_i1_orf1	influenza virus NS1A-binding protein-like [Ostrinia furnacalis]	3	333	34.6	8.95	14	3	3	High	1	0.91	0.972	0.632	0.66	0.689	0.588	0.589	0.647	0.598	0.581	0.615	0.526	0.604	0.595

TRINITY_DN938_c0_g1_i7_orf1	protein ultraspiracle homolog isoform X2 [Ostrinia furnacalis]	3	465	52.2	7.99	6	3	3	High	1	1.043	1.077	1.6	1.485	1.443	1.395	1.341	1.62	1.513	1.347	1.27	1.323	1.348	1.387
TRINITY_DN2769_c0_g1_i1_orf1	pseudouridylate synthase 7 homolog [Ostrinia furnacalis]	2	711	80.9	7.71	4	2	2	High	1	0.971	1.034	0.622	0.604	0.607	0.556	0.524	0.548	0.477	0.486	0.554	0.674	0.719	0.661
TRINITY_DN10403_c0_g1_i1_orf1	hypothetical protein evm_000264 [Chilo suppressalis]	2	254	28.7	7.87	9	2	1	High	1	1.173	1.076	1.337	1.473	1.523	1.852	1.58	1.758	1.522	1.38	1.633	1.191	1.227	1.149
TRINITY_DN3687_c0_g1_i1_orf1	acyl-CoA-binding domain-containing protein 5 isoform X2 [Manduca sexta]	2	270	30.4	6.44	10	4	2	High	1	1	1.093	1.41	1.347	1.377	1.303	1.131	1.125	1.022	0.98	0.961	1.048	1.134	1.231
TRINITY_DN34056_c0_g1_i4_orf1	hypothetical protein evm_001944 [Chilo suppressalis]	3	146	16.1	9.04	33	3	3	High	1	1.029	1.044	0.827	0.807	0.723	0.794	0.749	0.816	0.832	1.656	0.838	0.735	0.653	0.732
TRINITY_DN5031_c0_g1_i1_orf1	PREDICTED: 40S ribosomal protein S12 [Trachymyrmex septentrionalis]	2	159	17.4	6.51	6	4	2	High	1	0.984	0.948	0.693	0.721	0.74	0.645	0.611	0.672	0.553	0.522	0.576	0.532	0.581	0.559
TRINITY_DN62192_c0_g1_i2_orf1	ELAV-like protein 1 [Ostrinia furnacalis]	2	360	38.9	8.7	8	2	2	High	1	0.956	0.816	0.942	0.954	1.085	1.03	1.262	1.21	1.261	1.565	1.183	1.103	1.015	1.163
TRINITY_DN10398_c0_g1_i12_orf1	zinc finger protein ZPR1 isoform X2 [Ostrinia furnacalis]	2	456	51	4.7	6	2	2	High	1	1.051	0.999	0.883	0.942	0.926	0.871	0.986	0.968	1.076	1.029	1.132	0.824	0.715	0.76
TRINITY_DN63492_c0_g1_i1_orf1	unnamed protein product [Chilo suppressalis]	2	357	40.9	8.91	6	2	2	High	1	1.046	0.939	1.001	1.075	1.08	0.986	1.023	0.948	0.864	0.871	0.843	1.003	0.885	0.928
TRINITY_DN19293_c0_g1_i4_orf1	carboxylesterase [Ostrinia furnacalis]	2	543	61.2	6.01	5	2	2	High	1	1.019	0.984	0.956	0.929	1.034	1.288	1.266	1.371	0.954	1.3	0.8	0.891	0.776	0.886
TRINITY_DN31598_c0_g1_i1_orf1	39S ribosomal protein L28, mitochondrial [Ostrinia furnacalis]	3	282	32.6	9.57	16	3	3	High	1	0.868	0.989	0.933	0.848	0.902	0.87	0.981	0.965	1.035	1.39	0.961	1.1	1.04	1.1
TRINITY_DN4747_c0_g1_i4_orf1	transcription factor A, mitochondrial [Ostrinia furnacalis]	3	248	29.3	9.86	9	3	3	High	1	0.895	1.034	0.64	0.567	0.545	0.592	0.614	0.58	0.589	0.457	0.534	0.683	0.713	0.594
TRINITY_DN1664_c0_g1_i4_orf1	uncharacterized protein LOC114355246 [Ostrinia furnacalis]	3	568	65.1	8.78	6	3	3	High	1	1.064	1.057	1.714	1.837	1.835	1.575	1.518	1.446	1.58	1.423	1.672	1.895	1.987	1.946
TRINITY_DN1469_c0_g1_i1_orf1	kynurenine--oxoglutarate transaminase 3 isoform X4 [Orussus abietinus]	2	448	51.2	7.55	4	2	1	High	1	0.98	0.942	0.992	0.846	0.858	0.774	0.826	0.837	0.807	0.779	0.684	0.835	0.828	0.943
TRINITY_DN21924_c0_g1_i3_orf1	uncharacterized protein LOC114358821 [Ostrinia furnacalis]	2	304	33.8	5.95	9	2	2	High	1	0.956	1.108	0.854	0.809	0.958	1.103	0.978	0.939	1.038	1.417	0.938	0.836	0.815	0.933
TRINITY_DN18756_c0_g1_i6_orf1	COP9 signalosome complex subunit 6 [Ostrinia furnacalis]	2	349	38.1	6.32	5	2	2	High	1	1.039	0.99	1.068	1.225	1.014	0.929	0.98	1.076	0.891	0.918	0.966	1.152	1.271	1.272
TRINITY_DN30185_c0_g1_i3_orf1	organic cation transporter protein [Ostrinia furnacalis]	2	404	43.8	8.19	7	3	2	High	1	0.978	1.111	1.224	0.907	1.016	0.962	0.838	1.023	1.393	1.344	1.16	1.003	1.055	1.071
TRINITY_DN108818_c0_g1_i5_orf1	hypothetical protein O3G_MSEX012842 [Manduca sexta]	2	108	11.7	5.06	33	3	1	High	1	1.05	0.971	0.815	0.807	0.689	0.684	0.589	0.682	0.619	0.528	0.717	0.782	0.822	0.687
TRINITY_DN10630_c0_g1_i2_orf1	J domain-containing protein [Ostrinia furnacalis]	2	169	19	5.66	14	2	2	High	1	0.933	0.853	0.775	0.735	0.796	1.163	1.073	1.095	0.758	0.625	0.776	1.409	1.501	1.252
TRINITY_DN10396_c0_g1_i1_orf1	charged multivesicular body protein 3 isoform X1 [Ostrinia furnacalis]	2	218	24.6	5.33	12	3	2	High	1	0.895	0.983	1.103	1.236	1.194	1.297	1.225	1.158	1.146	1.329	1.364	1.118	1.171	1.213
TRINITY_DN14250_c0_g1_i1_orf1	apolipophorins-like [Ostrinia furnacalis]	2	85	9.7	5.1	22	3	2	High	1	0.941	0.894	0.997	1.101	1.031	1.411	1.524	1.413	2.305	2.188	2.72	2.32	2.343	2.237
TRINITY_DN64297_c0_g1_i1_orf1	vanin-like protein 2 isoform X2 [Ostrinia furnacalis]	3	70	7.8	9.47	57	3	1	High	1	1.417	1.336	5.983	6.562	4.619	2.526	3.369	2.411	7.733	8.118	7.036	13.563	14.15	14.772
TRINITY_DN295_c2_g1_i2_orf1	phosphoglycolate phosphatase 1A, chloroplastic [Manduca sexta]	4	128	13.9	9.42	28	4	4	High	1	1.002	1.008	3.068	2.883	2.791	2.435	2.675	2.581	4.436	3.582	4.048	2.948	3.139	2.767
TRINITY_DN478_c0_g1_i16_orf1	lipid storage droplets surface-binding protein 2 isoform X1 [Ostrinia furnacalis	3	322	33.9	7.05	9	3	3	High	1	1	0.964	0.59	0.533	0.614	0.353	0.374	0.385	0.449	0.456	0.451	0.548	0.536	0.562
TRINITY_DN29035_c0_g1_i5_orf1	2-oxoisovalerate dehydrogenase subunit beta, mitochondrial [Ostrinia furnaca	3	368	40.4	6.05	10	4	3	High	1	0.968	1.007	0.961	0.926	0.999	1.054	1.12	1.045	1.082	1.157	1.055	0.906	0.925	0.905
TRINITY_DN61_c0_g2_i3_orf1	mitochondrial dicarboxylate carrier [Ostrinia furnacalis]	2	291	31.8	9.88	5	3	2	High	1	0.988	1.154	0.862	0.735	0.743	0.829	0.788	0.838	0.804	0.776	0.646	0.655	0.683	0.658
TRINITY_DN11375_c0_g1_i4_orf1	uncharacterized protein LOC114363514 isoform X3 [Ostrinia furnacalis]	2	1460	163.7	5.96	2	3	2	High	1	0.885	0.977	0.783	0.826	0.832	0.859	0.9	0.988	0.879	0.958	0.927	1.064	0.998	1.167
TRINITY_DN579_c1_g1_i1_orf1	trafficking protein particle complex subunit 8 [Ostrinia furnacalis]	2	968	107.5	5.67	3	2	2	High	1	0.991	1.083	0.85	0.938	0.973	1.109	1.093	0.957	0.943	0.942	1.088	1.004	0.878	0.897
TRINITY_DN96738_c0_g2_i1_orf1	hCG1996054, isoform CRA_d [Homo sapiens]	2	80	9.1	9.09	28	3	1	High	1	1.023	1.169	0.951	0.846	0.92	1.052	0.954	1.14	0.903	0.893	0.961	1.21	1.299	0.912
TRINITY_DN31663_c0_g1_i2_orf1	PHD finger-like domain-containing protein 5A [Nasonia vitripennis]	2	110	12.5	8.41	18	2	2	High	1	1.032	1.084	0.512	0.54	0.55	0.51	0.521	0.489	0.452	0.447	0.434	0.622	0.729	0.6
TRINITY_DN16924_c0_g1_i1_orf1	peroxiredoxin-1 [Homo sapiens]	3	199	22.1	8.13	15	4	2	High	1	1.1	1.043	0.954	1.194	1.197	1.279	1.117	1.138	1.086	1.055	1.088	1.212	1.293	1.345
TRINITY_DN4439_c0_g2_i1_orf1	unnamed protein product, partial [Brenthis ino]	3	650	75	6.86	5	4	3	High	1	0.98	0.983	0.734	0.739	0.719	0.722	0.715	0.707	0.639	0.603	0.715	1.078	1.026	1.051
TRINITY_DN4213_c0_g1_i4_orf1	nardilysin-like isoform X1 [Ostrinia furnacalis]	3	1140	131.4	6.43	3	3	3	High	1	1.042	1.066	0.425	0.431	0.431	0.399	0.481	0.396	0.431	0.44	0.435	0.682	0.731	0.671
TRINITY_DN2571_c0_g2_i1_orf1	PREDICTED: huntingtin-interacting protein K isoform X1 [Amyelois transitella]	3	120	13.5	4.92	28	3	3	High	1	1.04	1.006	0.73	0.639	0.706	0.599	0.663	0.616	0.676	0.659	0.685	0.631	0.633	0.622
TRINITY_DN12555_c0_g1_i1_orf1	uncharacterized protein LOC114350746 [Ostrinia furnacalis]	2	163	17.3	5.33	15	3	2	High	1	0.941	0.903	1.136	1.018	1.076	1.148	1.253	1.019	1.17	1.748	1.114	1.763	1.882	1.616
TRINITY_DN1353_c0_g1_i1_orf1	UDP-glucose 4-epimerase-like [Ostrinia furnacalis]	2	361	40.3	6.4	8	2	2	High	1	0.962	0.896	0.26	0.234	0.277	0.336	0.269	0.271	0.318	0.283	0.282	0.46	0.458	0.456
TRINITY_DN4820_c0_g2_i2_orf1	tudor and KH domain-containing protein homolog isoform X1 [Ostrinia furnac	3	527	58.3	6.49	7	3	3	High	1	1.058	1.05	0.972	0.993	1.034	1.008	1.009	1.113	1.195	1.062	1.134	1.282	1.33	1.162
TRINITY_DN2630_c0_g3_i3_orf1	eukaryotic translation initiation factor 4E-binding protein 2 [Ostrinia furnacalis	2	115	12.4	9.38	18	3	2	High	1	0.975	0.903	0.555	0.646	0.624	0.533	0.571	0.526	0.725	0.627	0.747	0.57	0.702	0.616
TRINITY_DN1237_c0_g1_i4_orf1	armadillo-like helical domain-containing protein 3 [Ostrinia furnacalis]	2	684	76.7	6.65	4	2	2	High	1	0.893	0.927	1.036	0.971	1.12	0.791	0.768	0.96	0.852	1.109	0.878	0.821	0.661	0.765
TRINITY_DN20710_c0_g2_i2_orf1	acyl-CoA:lysophosphatidylglycerol acyltransferase 1-like isoform X2 [Manduca	2	373	43.3	8.28	6	2	2	High	1	1.131	0.967	1.124	1.399	0.992	1.234	1.332	1.274	1.056	1.11	1.794	0.906	1.006	0.961
TRINITY_DN113327_c0_g1_i2_orf1	proteasome subunit beta type-6 [Helicoverpa armigera]	2	99	10.5	8.41	23	3	2	High	1	0.92	1.045	1.092	1.014	0.955	0.984	0.985	1.024	1.273	1.234	1.169	1.054	0.994	0.999
TRINITY_DN6914_c0_g1_i2_orf1	atypical protein kinase C isoform X2 [Spodoptera litura]	2	680	76.4	6.79	4	2	2	High	1	1.036	1.101	0.828	0.736	0.87	0.822	0.785	0.886	0.787	1.054	0.732	1.079	0.978	0.989
TRINITY_DN11448_c0_g1_i4_orf1	uncharacterized protein LOC114364760 isoform X5 [Ostrinia furnacalis]	2	124	13.9	9.41	23	3	2	High	1	0.977	0.98	0.795	0.76	0.812	0.777	0.732	0.764	0.678	0.709	0.752	0.544	0.481	0.473
TRINITY_DN1978_c0_g1_i4_orf1	RNA exonuclease 4-like [Ostrinia furnacalis]	2	122	13.6	10.62	22	2	2	High	1	0.88	0.941	1.21	1.05	1.214	1.257	1.402	1.372	0.834	0.777	0.938	1.115	1.164	1.138
TRINITY_DN16824_c0_g1_i7_orf1	insulin receptor substrate 1 [Ostrinia furnacalis]	2	698	76	8.59	5	3	2	High	1	0.983	0.862	1.086	1.072	1.187	1.269	1.172	1.159	1.151	1.223	1.254	1.057	0.997	0.928
TRINITY_DN7778_c0_g1_i1_orf1	peroxiredoxin-2 [Cotesia glomerata]	2	250	28.2	6.73	7	3	1	High	1	0.975	0.85	1.004	1.042	1.029	0.866	0.879	0.854	0.744	0.726	0.872	0.532	0.486	0.587
TRINITY_DN6788_c0_g1_i1_orf1	unnamed protein product [Chilo suppressalis]	2	511	57.9	8.12	4	2	2	High	1	0.835	0.799	0.96	0.97	0.983	0.905	0.842	0.885	0.978	0.914	0.798	1.046	0.989	1.117
TRINITY_DN2566_c0_g1_i5_orf1	uncharacterized protein LOC114349936 [Ostrinia furnacalis]	2	112	12.4	5.57	21	2	2	High	1	1.045	1.035	0.928	0.893	1.179	1.17	1.394	1.274	1.78	1.472	1.938	2.292	2.344	2.16
TRINITY_DN3832_c0_g1_i1_orf1	serine-threonine kinase receptor-associated protein [Galleria mellonella]	2	324	35.9	6.92	7	2	2	High	1	1.027	0.956	0.58	0.649	0.55	0.579	0.571	0.548	0.566	0.579	0.666	0.73	0.718	0.839
TRINITY_DN5191_c0_g2_i1_orf1	CD151 antigen-like [Ostrinia furnacalis]	3	254	28.7	6.33	12	3	3	High	1	1.12	1.02	1.578	1.219	1.345	1.939	1.593	1.937	1.894	2.014	1.589	0.867	0.63	0.994
TRINITY_DN114982_c0_g1_i1_orf1	uncharacterized protein LOC114357708 [Ostrinia furnacalis]	2	195	22.6	9.8	13	2	2	High	1	0.938	0.961	0.915	1.434	0.897	0.847	1.027	0.716	1.027	1.483	1.986	2.678	2.019	2.508
TRINITY_DN549_c0_g1_i7_orf1	titin-like, partial [Ostrinia furnacalis]	2	428	48.1	5.52	4	4	2	High	1	1.076	1.075	1.51	1.615	1.47	1.513	1.183	1.282	1.285	1.065	1.385	0.395	0.436	0.452
TRINITY_DN4207_c0_g1_i1_orf1	mitochondrial import inner membrane translocase subunit Tim21 [Ostrinia furr	3	223	25.3	9.23	13	3	3	High	1	0.991	0.963	0.586	0.559	0.623	0.818	0.812	0.859	0.766	0.782	0.672	0.775	0.799	0.748
TRINITY_DN2879_c0_g1_i4_orf1	nucleoporin Nup35 [Ostrinia furnacalis]	2	290	31.5	8.09	11	2	2	High	1	0.895	0.982	0.685	0.822	0.737	0.71	0.555	0.598	0.464	0.507	0.61	0.95	1.04	0.913
TRINITY_DN28428_c0_g1_i2_orf1	unnamed protein product [Chrysodeixis includens]	3	587	65.6	6.86	4	3	3	High	1	0.989	1.027	0.876	0.865	0.82	0.771	0.848	0.855	0.735	0.675	0.743	1.041	1.043	1.074
TRINITY_DN4384_c0_g1_i5_orf1	chemosensory protein 5 [Conogethes punctiferalis]	2	147	16.9	9.44	14	3	2	High	1	0.969	1.031	1.321	1.399	1.267	1.286	1.216	1.164	1.51	1.239	1.646	4.778	4.807	4.602
TRINITY_DN705_c0_g1_i1_orf1	protein unc-13 homolog 4B isoform X3 [Ostrinia furnacalis]	3	1453	165.4	6.44	3	4	3	High	1	0.968	0.995	0.817	0.763	0.872	0.851	0.913	0.817	0.828	0.847	0.926	0.899	0.862	0.877
TRINITY_DN119265_c0_g2_i1_orf1	PREDICTED: sorting nexin-12 [Fopius arisanus]	3	163	19.1	8.92	17	4	3	High	1	0.915	0.968	1.294	1.365	1.399	1.123	1.17	1.044	1.189	1.076	1.102	1.368	1.424	1.473
TRINITY_DN1091_c0_g2_i10_orf1	macrophage mannose receptor 1-like isoform X2 [Maniola hyperantus]	3	320	35.4	5.78	8	4	2	High	1	0.984	1.069	1.281	1.348	1.346	0.906	0.952	1.011	1.264	1.241	1.336	1.559	1.697	1.482
TRINITY_DN3869_c0_g1_i4_orf1	glycosylphosphatidylinositol anchor attachment 1 protein [Galleria mellonella]	2	752	84.7	7.14	3	2	2	High	1	0.95	1.234	0.631	0.77	0.758	0.946	1.027	0.709	0.899	1.197	0.628	0.698	0.908	0.946
TRINITY_DN12608_c0_g1_i1_orf1	centrosome-associated zinc finger protein CP190 [Ostrinia furnacalis]	3	1370	155.8	4.88	2	3	3	High	1	1.01	1.065	0.765	0.947	0.948	0.844	0.826	0.833	0.784	0.802	0.672	0.842	0.819	0.841
TRINITY_DN57904_c0_g2_i1_orf1	cuticle protein 19 [Plutella xylostella]	2	129	14.1	6.6	16	3	1	High	1	0.982	0.848	0.246	0.237	0.248	0.217	0.272	0.243	0.243	0.165	0.26	1.356	1.522	1.351
TRINITY_DN14372_c0_g2_i1_orf1	12 kDa FK506-binding protein-like [Ostrinia furnacalis]	2	108	11.7	8.75	19	2	2	High	1	1.068	1.012	0.551	0.602	0.606	0.493	0.563	0.496	0.518	0.455	0.507	0.597	0.677	0.598
TRINITY_DN605_c0_g1_i4_orf1	dnaJ homolog subfamily C member 7 [Ostrinia furnacalis]	3	495	56.2	7.85	7	3	3	High	1	0.967	1.049	0.663	0.669	0.705	0.611	0.747	0.63	0.624	0.764	0.657	0.739	0.686	0.752
TRINITY_DN5458_c1_g1_i9_orf1	histone H2A-like [Aedes aegypti]	2	154	17.1	10.64	21	2	1	High	1	0.878	0.753	1.385	1.484	1.7	1.25	1.426	1.364	1.122	1.339	1.267	1.233	1.424	1.627
TRINITY_DN2593_c0_g3_i1_orf1	midgut carboxypeptidase [Loxostege sticticalis]	3	438	49.1	6.16	7	3	3	High	1	0.951	0.984	0.635	0.593	0.634	0.669	0.666	0.662	0.648	0.634	0.665	0.57	0.627	0.577
TRINITY_DN1004_c0_g2_i1_orf1	E3 ubiquitin-protein ligase NEDD4 isoform X6 [Ostrinia furnacalis]	2	769	88.3	5.76	4	2	2	High	1	1.02	0.951	1.157	1.127	1.113	1.286	1.137	1.265	1.395	1.425	1.42	1.167	1.162	1.384
TRINITY_DN146006_c0_g1_i1_orf1	unnamed protein product [Chrysodeixis includens]	1	105	10.9	5.58	17	1	1	High	1	1.664	1.552	1.272	1.115	0.921	1.101	0.652	0.888	0.963	0.865	1.051	0.515	0.676	0.689
TRINITY_DN6163_c0_g1_i4_orf1	CTD nuclear envelope phosphatase 1 homolog [Ostrinia furnacalis]	3	243	28.4	9.44	10	3	3	High	1	0.991	1.022	0.91	0.824	0.894	0.908	0.88	0.92	0.828	0.851	0.805	1.046	1.045	0.939
TRINITY_DN26337_c0_g1_i3_orf1	lysosome membrane protein 2-like [Ostrinia furnacalis]	3	738	84.8	5.5	4	3	3	High	1	1.069	1.079	1.436	1.262	1.228	1.519	1.784	2.035	1.615	1.34	1.476	2.289	2.254	2.251
TRINITY_DN7075_c0_g2_i1_orf1	retinal dehydrogenase 1-like [Ostrinia furnacalis]	2	92	10.2	5.48	24	4	2	High	1	0.942	0.996	1.363	1.362	1.259	1.74	1.721	1.593	1.683	1.585	1.534	1.23	1.255	1.234
TRINITY_DN13887_c0_g1_i5_orf1	transmembrane protein 184B isoform X3 [Ostrinia furnacalis]	2	408	46	7.23	7	3	2	High	1	0.999	1.057	1.211	1.189	1.065	1.128	1.34	1.239	1.224	1.162	1.284	2.131	2.021	2.033
TRINITY_DN26882_c0_g1_i1_orf1	uncharacterized protein LOC114349648 [Ostrinia furnacalis]	2	263	28.6	4.74	8	2	2	High	1	0.922	1.003	0.914	0.956	0.937	0.951	0.926	0.971	1.038	0.911	0.879	5.81	6.028	5.496
TRINITY_DN67231_c0_g1_i1_orf1	endocuticle structural glycoprotein SgAbd-8-like [Ostrinia furnacalis]	1	130	13.9	4.59	20	2	1	High	1	1.049	0.884	0.996	1.101	1.027	1.118	1.167	1.097	0.954	0.72	0.998	0.024	0.02	0.032
TRINITY_DN334_c0_g1_i2_orf1	chymotrypsin-like serine protease, partial [Ostrinia nubilalis]	2	131	13.2	9.44	18	4	1	High	1	1.076	1.047	0.181	0.176	0.203	0.253	0.204	0.206	0.191	0.251	0.182	0.211	0.156	0.187
TRINITY_DN86621_c0_g1_i2_orf1	protein sly1 homolog isoform X1 [Ostrinia furnacalis]	2	359	39.6	8.32	9	2	2	High	1	0.991	1.19	0.928	1.062	1.129	0.989	0.998	1.006	0.827	0.746	0.957	1.574	1.484	1.568
TRINITY_DN21981_c0_g1_i8_orf1	dihydroorotate dehydrogenase (quinone), mitochondrial [Ostrinia furnacalis]	2	390	42.8	9.16	5	2	2	High	1	1.028	1.015	0.544	0.635	0.666	0.581	0.621	0.625	0.506	0.668	0.521	0.655	0.648	0.694
TRINITY_DN24350_c0_g1_i1_orf1	zinc finger protein 771-like [Ostrinia furnacalis]	2	300	32.5	6.15	9	2	2	High	1	0.867	1.06	0.79	1.048	0.954	0.902	0.954	0.932	0.835	0.825	0.97	1.075	1.03	1.103
TRINITY_DN3949_c0_g1_i1_orf1	probable cytochrome P450 304a1 [Ostrinia furnacalis]	3	510	59	8.76	5	3	3	High	1	0.998	1.003	1.904	1.82	1.645	1.925	2.073	2.112	2.122	2.136	2.188	3.011	3.024	3.038
TRINITY_DN123184_c0_g1_i1_orf1	double-strand break repair protein MRE11 [Ostrinia furnacalis]	3	562	63.1	5.52	4	3	3	High	1	0.985	0.95	0.883	0.968	0.948	0.782	0.83	0.765	0.751	0.65	0.804	1.184	1.249	1.226
TRINITY_DN1108_c1_g2_i1_orfp1	TRINITY_DN1108_c1_g2_i1_m.5565 TRINITY_DN1108_c1_g2::TRINITY_DN1108_	2	204	22.5	5.87	11	2	2	High	1	1.223	1.061	2.445	2.655	2.577	1.724	1.904	1.826	1.646	1.67	2.068	1.461	1.58	1.382
TRINITY_DN28802_c0_g1_i1_orf1	apolipoprotein D-like [Ostrinia furnacalis]	2	231	26	6.21	11	2	2	High	1	1.033	0.931	1.288	1.286	1.333	1.452	1.577	1.522	1.388	1.178	1.157	1.615	1.562	1.578
TRINITY_DN131471_c0_g1_i1_orf1	basement membrane-specific heparan sulfate proteoglycan core protein isofo	1	69	7.5	5.26	19	3	1	High	1	0.999	1.029	1.3	1.158	1.213	1.675	1.737	1.617	1.629	1.531	1.374	1.501	1.592	1.592
TRINITY_DN838_c0_g1_i18_orf1	hypothetical protein evm_003399, partial [Chilo suppressalis]	4	808	91	9.29	6	4	4	High	1	0.986	0.97	0.979	0.991	1.007	0.911	0.898	0.859	0.841	0.799	0.874	0.797	0.798	0.754
TRINITY_DN18118_c0_g2_i10_orf1	clavesin-1-like [Ostrinia furnacalis]	1	301	35.6	6.86	5	1	1	High	1	1.058	1.098	1.47	1.121	1.23	1.239	1.011	1.317	1.343	1.13	1.241	1.362	1.439	1.395
TRINITY_DN22515_c0_g1_i10_orf1	hypothetical protein KGM_202621 [Danaus plexippus plexippus]	2	69	7.7	4.63	29	2	2	High	1	1.004	1.035	5.191	5.317	5.382	6.182	5.679	4.813	6.216	4.671	6.317	5.775	6.062	5.103
TRINITY_DN38225_c0_g2_i1_orf1	uncharacterized protein LOC114354273 [Ostrinia furnacalis]	2	407	46.6	8.81	4	3	2	High	1	0.969	0.936	2.244	2.041	2.109	1.6	1.723	1.709	1.618	1.546	1.534	1.039	1.158	1.113
TRINITY_DN18031_c0_g1_i1_orf1	63 kDa chaperonin, mitochondrial-like [Ostrinia furnacalis]	2	563	60.7	7.52	5	2	1	High	1	1.46	1.179	1.385	2.442	2.009	2.611	2.589	2.842	2.089	2.222	9.037	1.965	1.95	1.85
TRINITY_DN8940_c0_g1_i4_orf1	probable ATP-dependent RNA helicase DDX23 [Ostrinia furnacalis]	3	824	96	9.66	3	3	2	High	1	0.944	1.106	0.763	0.866	0.811	0.823	0.888	0.892	0.784	0.913	0.749	0.699	0.836	0.749
TRINITY_DN9465_c0_g1_i4_orf1	prisilkin-39-like [Ostrinia furnacalis]	2	429	43.9	9.41	8	3	2	High	1	1.156	1.075	0.988	1.073	0.936	1.04	0.962	0.991	1.065	1.37	0.862	1.263	1.29	1.211
TRINITY_DN5686_c0_g1_i4_orf1	FACT complex subunit spt16 isoform X2 [Ostrinia furnacalis]	3	1120	127	6.35	3	3	3	High	1	0.963	1.057	0.512	0.506	0.557	0.566	0.62	0.56	0.458	0.424	0.462	0.573	0.542	0.566
TRINITY_DN3055_c0_g1_i9_orf1	WAP four-disulfide core domain protein 2-like [Ostrinia furnacalis]	1	132	13.9	7.37	13	2	1	High	1	0.98	0.954	1.617	1.553	1.392	1.436	1.053	1.173	1.011	0.944	1.081	1.089	0.919	0.909
TRINITY_DN72816_c0_g1_i2_orf1	Golgi apparatus protein 1 [Ostrinia furnacalis]	3	1097	126.2	7.34	3	3	3	High	1	0.941	0.973	0.753	0.83	0.764	0.706	0.745	0.784	0.612	0.607	0.583	1.278	1.291	1.186
TRINITY_DN4321_c0_g1_i1_orf1	acyl-CoA Delta(11) desaturase isoform X1 [Ostrinia furnacalis]	2	362	41.7	8.87	5	2	2	High	1	0.975	1.103	0.899	0.9	0.886	1.275	1.382	1.395	0.978	0.801	0.947	0.83	0.843	0.843

TRINITY_DN14937_c0_g1_i7_orf1	multidrug resistance protein homolog 49-like [Ostrinia furnacalis]	3	1307	143.2	7.5	2	3	3	High	1	0.943	0.975	0.379	0.34	0.408	0.499	0.511	0.556	0.441	0.385	0.384	0.43	0.456	0.439
TRINITY_DN33178_c0_g1_i1_orf1	synaptojanin-1 [Ostrinia furnacalis]	2	1073	118.9	6.51	2	2	2	High	1	0.952	1.098	0.763	0.748	0.732	0.686	0.736	0.733	0.636	0.71	0.663	0.832	0.736	0.816
TRINITY_DN34786_c0_g1_i1_orf1	small heat shock protein Hsp29.7 [Ostrinia furnacalis]	2	250	28.2	6.19	8	2	2	High	1	0.808	0.928	0.739	0.747	0.646	0.746	0.87	0.679	0.826	0.811	0.755	2.081	2.052	2.162
TRINITY_DN3747_c1_g1_i3_orf1	unnamed protein product, partial [Iphiclides podalirius]	3	649	71.6	7.91	4	3	3	High	1	0.814	0.896	0.609	0.598	0.581	0.636	0.673	0.619	0.508	0.496	0.569	0.575	0.568	0.608
TRINITY_DN146758_c0_g1_i1_orf1	PREDICTED: mitochondrial import inner membrane translocase subunit Tim16-	2	127	14	9.55	16	2	2	High	1	1.031	1.015	0.538	0.56	0.677	0.64	0.695	0.655	0.66	0.564	0.852	0.675	0.548	0.521
TRINITY_DN4156_c0_g1_i2_orf1	calcium channel flower [Ostrinia furnacalis]	1	193	20.9	7.02	10	1	1	High	1	1.11	1.255	0.741	0.677	0.743	0.656	0.663	0.609	0.455	0.543	0.444	1.277	1.425	1.429
TRINITY_DN56121_c0_g1_i4_orf1	uncharacterized protein LOC114361939 [Ostrinia furnacalis]	2	106	12.6	9.99	21	2	2	High	1	1.158	1.089	0.782	0.684	0.856	0.757	0.693	0.791	0.827	0.855	0.819	0.867	0.8	0.852
TRINITY_DN14285_c0_g1_i6_orf1	RNA-binding protein 39 [Ostrinia furnacalis]	3	496	56.6	9.72	7	3	3	High	1	1.032	1.108	1.104	1.148	1.111	1.095	1.017	1.054	1.196	1.039	1.09	1.113	1.165	1.067
TRINITY_DN14524_c0_g1_i1_orf1	calcineurin subunit B type 2 isoform X1 [Nasonia vitripennis]	2	171	19.4	4.69	13	2	2	High	1	1.007	0.876	1.062	0.979	1.181	1.237	1.168	1.276	1.321	1.186	0.895	1.09	1.268	0.972
TRINITY_DN22815_c0_g1_i2_orf1	acyl carrier protein, mitochondrial isoform X1 [Ostrinia furnacalis]	2	154	17.3	6.54	15	2	2	High	1	0.928	0.911	0.443	0.498	0.471	0.442	0.437	0.441	0.397	0.432	0.488	0.414	0.423	0.41
TRINITY_DN3847_c1_g1_i1_orf1	ribosome production factor 2 homolog [Ostrinia furnacalis]	2	313	35.9	10.01	7	2	2	High	1	0.937	1.025	0.287	0.348	0.304	0.338	0.387	0.358	0.378	0.372	0.388	0.347	0.419	0.366
TRINITY_DN65299_c0_g4_i1_orf1	LOW QUALITY PROTEIN: signal transducing adapter molecule 2 [Ostrinia furna	1	140	14.6	4.28	19	1	1	High	1	1.019	1.034	1.732	1.81	1.524	1.378	1.328	1.47	1.318	1.231	1.477	1.641	1.715	1.653
TRINITY_DN64788_c0_g1_i1_orf1	uncharacterized protein LOC114365187 isoform X3 [Ostrinia furnacalis]	2	86	9.1	6.73	33	2	1	High	1	0.885	0.894	1.486	1.671	1.323	1.4	1.024	1.13	1.302	0.898	1.117	1.244	1.233	1.113
TRINITY_DN140_c1_g1_i2_orf1	modular serine protease-like isoform X1 [Ostrinia furnacalis]	3	765	83	5.27	5	3	3	High	1	0.828	0.978	1.53	1.506	1.468	1.107	1.28	1.154	1.073	1.209	1.273	1.91	1.857	1.983
TRINITY_DN1362_c0_g1_i4_orf1	heparan-alpha-glucosaminide N-acetyltransferase [Helicoverpa armigera]	2	575	63.5	9.44	5	2	2	High	1	1.054	0.937	1.15	1.137	1.275	0.986	0.917	1.007	1.05	1.077	1.03	1.56	1.528	1.4
TRINITY_DN3670_c0_g1_i2_orf1	WD repeat domain phosphoinositide-interacting protein 2 isoform X7 [Ostrinia	3	329	35	7.59	9	3	3	High	1	1.024	1.098	1.037	1.005	1.001	0.957	0.987	0.957	0.938	0.938	1.027	1.035	0.908	1.126
TRINITY_DN2160_c0_g1_i13_orf1	unnamed protein product [Spodoptera exigua]	2	294	34.5	5.36	8	2	2	High	1	1.002	1.117	0.759	0.753	0.783	0.779	0.813	0.839	0.683	0.802	0.783	1.132	0.906	0.98
TRINITY_DN10266_c0_g1_i5_orf1	armadillo repeat-containing protein 6 homolog [Ostrinia furnacalis]	2	469	52.3	5.47	5	2	2	High	1	1.073	0.965	0.865	0.782	0.884	0.729	0.776	0.826	0.807	0.584	0.618	0.807	0.8	0.823
TRINITY_DN1407_c0_g1_i12_orf1	hypothetical protein evm_012298 [Chilo suppressalis]	3	72	8	6.52	29	5	3	High	1	0.946	0.88	1.207	1.324	1.198	1.02	1.134	1.129	1.579	2.656	1.679	2.674	2.861	2.728
TRINITY_DN46409_c0_g1_i1_orf1	unnamed protein product [Heterotrigona itama]	2	511	54.5	5.33	7	2	2	High	1	1.107	0.923	0.226	0.251	0.294	0.283	0.235	0.26	0.344	0.357	0.337	0.392	0.395	0.358
TRINITY_DN42333_c0_g1_i5_orf1	regucalcin-like [Ostrinia furnacalis]	3	206	22.7	5.5	17	4	1	High	1	1.202	1.077	1.055	0.965	1.127	1.194	1.206	1.151	1.005	1.423	1.268	1.916	1.43	1.568
TRINITY_DN38540_c0_g1_i1_orf1	GSCOCG00000129001-RA-CDS [Cotesia congregata]	2	327	38.6	9.6	9	3	2	High	1	1.121	1.005	0.469	0.524	0.52	0.457	0.558	0.488	0.405	0.419	0.439	0.69	0.756	0.611
TRINITY_DN22941_c0_g1_i1_orf1	protein CWC15 homolog [Ostrinia furnacalis]	2	217	24.7	8.03	12	2	2	High	1	0.971	0.936	0.696	0.756	0.783	0.616	0.672	0.86	0.689	0.683	0.736	0.688	0.671	0.625
TRINITY_DN116951_c0_g3_i2_orf1	spermine oxidase-like isoform X2 [Ostrinia furnacalis]	2	81	8.6	5.21	38	2	2	High	1	1.004	0.923	0.719	0.698	0.719	0.792	0.791	0.806	0.646	0.584	0.762	0.281	0.262	0.315
TRINITY_DN98723_c1_g1_i1_orf1	uncharacterized protein LOC114362777 [Ostrinia furnacalis]	2	582	66.2	6.65	3	4	1	High	1	1.021	0.934	0.739	0.789	0.815	0.782	0.746	0.706	0.663	0.604	0.725	0.666	0.697	0.594
TRINITY_DN38644_c0_g1_i1_orf1	unnamed protein product, partial [Iphiclides podalirius]	2	328	35.8	6.73	7	3	2	High	1	1.001	0.972	0.804	0.831	0.804	0.854	0.983	0.883	0.877	0.806	0.796	1.021	1.035	1.1
TRINITY_DN4908_c1_g1_i5_orf1	DNA topoisomerase 2 isoform X1 [Ostrinia furnacalis]	3	1564	177.2	8.78	2	3	3	High	1	0.932	0.954	0.572	0.572	0.618	0.609	0.537	0.612	0.592	0.564	0.516	0.97	1.102	0.913
TRINITY_DN101995_c0_g1_i1_orf1	microtubule-actin cross-linking factor 1 isoform X15 [Ostrinia furnacalis]	1	74	8.3	4.5	16	1	1	High	1	0.939	0.939	1.174	1.05	1.125	1.23	1.199	1.208	1.015	0.796	0.98	0.588	0.607	0.571
TRINITY_DN16400_c0_g2_i1_orf1	superoxide dismutase [Cu-Zn]-like isoform X1 [Ostrinia furnacalis]	2	242	25.9	6.68	10	2	2	High	1	0.957	1.04	1.817	1.868	1.804	1.346	1.306	1.282	1.324	1.159	1.403	0.765	0.718	0.831
TRINITY_DN76283_c0_g2_i1_orf1	fatty acid synthase-like [Ostrinia furnacalis]	3	284	32	9.39	8	3	3	High	1	1.029	0.993	0.395	0.362	0.373	0.425	0.515	0.512	0.426	0.467	0.37	0.317	0.332	0.358
TRINITY_DN5925_c0_g1_i5_orf1	isocitrate dehydrogenase [NAD] subunit gamma, mitochondrial-like isoform X	2	388	42.4	7.2	6	2	2	High	1	1.042	0.985	0.442	0.449	0.501	0.37	0.351	0.408	0.392	0.329	0.428	0.387	0.404	0.373
TRINITY_DN22_c0_g1_i3_orf1	uncharacterized protein LOC114362831 [Ostrinia furnacalis]	2	107	11.9	5.9	20	2	1	High	1	1.032	0.995	0.837	0.749	0.816	1.184	1.093	1.248	0.724	0.914	0.745	0.999	0.836	0.837
TRINITY_DN72285_c1_g1_i1_orf1	hypothetical protein HF086_001747 [Spodoptera exigua]	1	111	12.8	7.43	16	1	1	High	1	1.117	1.09	1.023	0.691	0.95	1.032	0.906	1.123	0.94	0.744	0.677	0.761	0.872	0.855
TRINITY_DN15667_c0_g1_i2_orf1	coiled-coil domain-containing protein 25 [Ostrinia furnacalis]	2	207	24.3	6.58	10	2	2	High	1	1.104	1.013	0.458	0.502	0.499	0.438	0.497	0.413	0.494	0.491	0.469	0.577	0.564	0.543
TRINITY_DN47123_c0_g1_i1_orf1	WD40 repeat-containing protein SMU1 [Ostrinia furnacalis]	2	512	56.4	7.96	4	2	2	High	1	0.987	1.036	0.505	0.543	0.433	0.399	0.395	0.439	0.42	0.368	0.375	0.55	0.514	0.545
TRINITY_DN93566_c0_g2_i1_orf1	60S acidic ribosomal protein P1 [Manduca sexta]	2	111	11.3	4.21	22	6	2	High	1	0.977	0.999	0.777	0.82	0.82	0.75	0.724	0.772	0.707	0.714	0.703	0.674	0.753	0.641
TRINITY_DN2924_c0_g1_i2_orf1	cuticular protein RR-2 [Spodoptera litura]	2	115	11.6	8.73	14	3	2	High	1	0.975	0.987	0.582	0.527	0.548	0.819	0.8	0.683	0.478	0.446	0.55	0.295	0.31	0.343
TRINITY_DN2808_c0_g1_i8_orf1	uncharacterized protein LOC114353011 isoform X2 [Ostrinia furnacalis]	2	407	45.8	8.18	5	2	2	High	1	1.01	1.033	0.908	0.928	0.938	0.905	0.999	0.856	0.788	0.788	0.794	1.074	1.054	1.189
TRINITY_DN8536_c0_g1_i2_orf1	PC4 and SFRS1-interacting protein isoform X4 [Galleria mellonella]	2	273	31.1	5.52	6	2	2	High	1	1.017	0.912	0.515	0.506	0.551	0.515	0.442	0.487	0.373	0.361	0.413	0.702	0.755	0.781
TRINITY_DN1038_c0_g1_i4_orf1	gastric triacylglycerol lipase-like [Ostrinia furnacalis]	1	516	58.7	6.49	3	2	1	High	1	0.891	0.971	1.251	1.501	1.337	1.21	1.245	1.41	0.949	1.54	1.185	1.636	1.498	1.578
TRINITY_DN29402_c0_g1_i1_orf1	hypothetical protein evm_000391 [Chilo suppressalis]	1	138	15.6	5.4	9	1	1	High	1	0.877	0.949	1.186	1.19	1.072	1.11	1.133	1.155	0.94	1.146	1.02	1.356	1.313	1.145
TRINITY_DN44094_c0_g1_i1_orf1	myotrophin-like [Ostrinia furnacalis]	2	68	7.4	5.35	38	5	2	High	1	1.004	0.916	0.803	0.734	0.861	0.985	0.998	0.958	0.946	0.848	0.934	1.331	1.472	1.249
TRINITY_DN47605_c0_g2_i1_orf1	hypothetical protein evm_002030 [Chilo suppressalis]	3	618	71	8.22	5	3	3	High	1	0.958	0.976	0.728	0.836	0.814	0.819	0.778	0.809	0.8	0.782	0.727	0.763	0.72	0.767
TRINITY_DN1720_c0_g1_i3_orf1	monocarboxylate transporter 12 [Ostrinia furnacalis]	2	540	58.3	8.07	6	2	2	High	1	1.053	0.918	1.36	1.334	1.372	1.628	1.86	1.661	1.67	1.506	1.753	1.766	1.592	1.631
TRINITY_DN107840_c1_g1_i1_orf1	HEAT repeat-containing protein 3 [Ostrinia furnacalis]	3	624	68.8	4.75	4	3	3	High	1	0.938	1.004	0.487	0.573	0.562	0.511	0.629	0.538	0.504	0.633	0.501	0.589	0.6	0.663
TRINITY_DN8887_c0_g1_i1_orf1	vacuolar-sorting protein SNF8 [Ostrinia furnacalis]	2	249	28.1	8.5	9	3	2	High	1	0.901	0.89	1.01	1.007	1.039	0.991	1.197	1.063	0.931	0.903	0.975	1.058	1.007	1.004
TRINITY_DN25285_c0_g1_i1_orf1	pancreatic triacylglycerol lipase-like [Ostrinia furnacalis]	2	473	53.3	7.27	5	2	2	High	1	0.854	0.869	0.703	0.63	0.785	0.819	0.868	0.702	0.773	0.774	0.667	0.884	0.871	0.779
TRINITY_DN10045_c0_g1_i1_orf1	methylosome protein 50-like [Ostrinia furnacalis]	4	334	37	5.34	10	4	4	High	1	0.95	0.96	0.934	0.919	0.901	0.86	0.907	0.981	0.992	0.942	0.699	1.038	0.971	0.971
TRINITY_DN25997_c1_g2_i4_orf1	ribokinase-like [Ostrinia furnacalis]	2	316	33.9	6.27	8	2	2	High	1	0.874	0.918	0.5	0.52	0.501	0.647	0.822	0.813	0.628	0.539	0.578	0.707	0.792	0.725
TRINITY_DN35351_c0_g1_i3_orf1	adult-specific cuticular protein ACP-20-like [Ostrinia furnacalis]	2	184	19.2	6.89	16	2	2	High	1	1.166	1.149	1.205	1.026	1.102	1.2	1.272	1.299	1.132	1.265	0.961	5.556	6.377	5.849
TRINITY_DN41848_c0_g1_i4_orf1	vacuolar protein sorting-associated protein 28 homolog [Ostrinia furnacalis]	2	210	24.8	5.33	13	4	2	High	1	0.96	1.025	1.625	1.438	1.496	1.603	1.493	1.544	1.961	1.797	1.452	1.388	1.341	1.395
TRINITY_DN76036_c0_g1_i1_orf1	cytochrome c oxidase subunit 6A1, mitochondrial-like [Ostrinia furnacalis]	2	107	12	9.7	24	3	2	High	1	1.034	1.072	0.442	0.444	0.448	0.358	0.358	0.366	0.526	0.383	0.426	0.448	0.505	0.455
TRINITY_DN105901_c0_g1_i2_orfp	contactin-like [Pectinophora gossypiella]	2	148	16.7	10.27	16	3	2	High	1	0.875	0.984	0.33	0.383	0.33	0.335	0.384	0.346	0.413	0.535	0.413	0.333	0.286	0.327
TRINITY_DN14217_c0_g1_i1_orf1	putative serine protease K12H4.7 [Ostrinia furnacalis]	1	481	54.9	5.2	3	1	1	High	1	1.304	1.428	0.14	0.058	0.159	0.133	0.136	0.262	0.097	0.169	2.496	1.769	0.924	1.948
TRINITY_DN2894_c0_g2_i3_orf1	myrosinase 1-like isoform X1 [Ostrinia furnacalis]	3	434	50.1	6.21	6	3	1	High	1	0.946	0.929	0.498	0.536	0.571	0.331	0.299	0.368	0.233	0.243	0.278	0.899	0.661	0.929
TRINITY_DN8369_c0_g1_i1_orf1	39S ribosomal protein L37, mitochondrial [Ostrinia furnacalis]	3	410	47.1	6.68	6	3	3	High	1	0.968	0.979	0.252	0.271	0.3	0.316	0.309	0.336	0.299	0.34	0.322	0.413	0.411	0.408
TRINITY_DN24539_c0_g1_i4_orf1	low molecular weight phosphotyrosine protein phosphatase 1-like isoform X2	2	160	18.3	6.77	13	2	2	High	1	1.019	1	0.851	0.871	0.818	0.756	0.763	0.717	0.793	0.737	0.867	1.029	1.112	0.951
TRINITY_DN2749_c0_g1_i4_orf1	RNA exonuclease 4-like [Ostrinia furnacalis]	3	109	12.4	6.93	27	4	2	High	1	0.884	0.91	0.42	0.48	0.351	0.731	0.689	0.615	0.752	0.696	0.798	0.486	0.46	0.476
TRINITY_DN72369_c0_g1_i1_orf1	microsomal triacylglycerol transfer protein [Vanessa cardui]	1	95	9.9	5.95	14	3	1	High	1	0.879	0.941	1.533	1.51	1.447	1.581	1.57	1.544	1.343	1.743	1.555	1.787	1.545	1.71
TRINITY_DN15682_c0_g1_i4_orf1	seroin transcript 1B [Ostrinia nubilalis]	2	55	5.9	5.73	36	2	2	High	1	1.106	0.954	2.21	2.297	2.156	1.07	1.441	1.312	3.112	3.795	3.052	1.266	1.029	1.596
TRINITY_DN121802_c0_g1_i6_orfp	TRINITY_DN121802_c0_g1_i6_m.78506 TRINITY_DN121802_c0_g1::TRINITY_DN	1	111	12.2	6.04	32	1	1	High	1	1.043	1.124	2.659	3.142	2.817	1.471	1.4	1.593	1.8	3.7	1.901	1.113	0.998	1.287
TRINITY_DN4550_c1_g1_i19_orf1	titin homolog [Ostrinia furnacalis]	4	1776	202.9	7.3	2	4	4	High	1	0.97	0.999	1.052	1.173	1.028	0.93	0.962	1.053	1.35	1.39	1.498	1.353	1.325	1.366
TRINITY_DN13866_c0_g1_i4_orf1	synembryn-A [Ostrinia furnacalis]	2	551	61.9	5.53	5	2	2	High	1	0.943	1.015	0.876	0.966	0.972	0.984	0.947	1.009	1.005	1.035	1.085	1.085	0.889	1.18
TRINITY_DN371_c0_g1_i6_orf1	trypsin beta-like [Ostrinia furnacalis]	2	310	32.9	8.81	11	2	2	High	1	0.801	0.991	0.672	0.667	0.685	0.708	0.671	0.678	0.754	0.702	0.688	0.729	0.559	0.672
TRINITY_DN20426_c0_g2_i1_orf1	prolyl 3-hydroxylase sudestada1 [Ostrinia furnacalis]	3	617	68.8	4.96	5	3	3	High	1	0.924	0.977	0.988	0.982	0.934	0.853	0.854	1.011	0.784	0.778	0.791	0.891	0.878	0.982
TRINITY_DN9916_c0_g1_i1_orf1	PREDICTED: dynein light chain Tctex-type [Amyelois transitella]	1	116	12.9	5.41	16	1	1	High	1	0.962	0.912	0.594	0.711	0.73	0.649	0.753	0.751	0.604	0.619	0.567	1.358	1.444	1.179
TRINITY_DN535_c3_g2_i1_orf1	PSME3-interacting protein isoform X2 [Ostrinia furnacalis]	3	248	28.2	5.05	15	4	3	High	1	1.007	1.066	0.796	0.832	0.808	0.707	0.708	0.671	0.77	0.655	0.817	0.79	0.775	0.798
TRINITY_DN1901_c0_g1_i6_orf1	mitochondrial intermembrane space import and assembly protein 40 [Ostrinia	2	143	15	4.34	13	2	2	High	1	1.029	1.025	0.327	0.256	0.343	0.266	0.297	0.332	0.334	0.273	0.309	0.374	0.312	0.369
TRINITY_DN18279_c0_g1_i1_orf1	solute carrier family 12 member 9 isoform X1 [Hyposmocoma kahamanoa]	2	933	101.4	8.12	2	2	2	High	1	0.838	0.875	1.194	1.126	1.079	1.063	1.062	1.09	1.048	1.238	1.158	1.008	1.028	1.16
TRINITY_DN3791_c0_g1_i2_orf1	transmembrane protein 19 [Ostrinia furnacalis]	2	345	37.5	8.6	8	2	2	High	1	1.101	1.224	0.648	0.616	0.66	0.631	0.618	0.734	0.567	0.584	0.683	0.678	0.739	0.764
TRINITY_DN3513_c0_g1_i5_orf1	vacuolar protein sorting-associated protein 16 homolog [Ostrinia furnacalis]	2	835	94.7	8.43	3	2	2	High	1	1.03	1.058	1.755	1.647	1.444	1.502	1.476	1.697	1.641	1.686	1.852	2.023	1.823	1.852
TRINITY_DN146364_c0_g1_i1_orf1	pupal cuticle protein 20-like [Ostrinia furnacalis]	1	149	15.9	6.04	12	1	1	High	1	1.187	0.989	0.973	1.041	0.933	0.776	0.742	0.731	0.905	0.883	0.873	0.95	0.987	0.81
TRINITY_DN2802_c1_g1_i1_orf1	psi [Ostrinia furnacalis]	1	119	12.1	5.87	10	2	1	High	1	0.957	1.052	0.757	0.813	0.786	0.639	0.638	0.636	0.636	0.621	0.731	0.992	0.903	0.972
TRINITY_DN4654_c0_g1_i6_orf1	vam6/Vps39-like protein [Ostrinia furnacalis]	4	865	98.4	7.03	5	4	4	High	1	1.01	0.982	0.981	1.049	0.962	0.938	1.02	0.929	1.024	1.247	1.292	1.881	1.354	1.26
TRINITY_DN9506_c0_g1_i2_orf1	glutathione S-transferase sigma 4 [Conogethes punctiferalis]	3	201	23	5.71	22	3	3	High	1	0.985	1.06	0.912	0.795	0.863	0.684	0.727	0.807	0.964	1.082	0.708	0.561	0.623	0.628
TRINITY_DN251_c0_g1_i2_orf1	hypothetical protein evm_008466 [Chilo suppressalis]	2	278	29.1	6.29	9	2	2	High	1	1.028	0.845	1.298	1.239	1.01	1.04	1.186	1.047	1.598	1.422	1.775	1.439	1.443	1.645
TRINITY_DN51968_c0_g1_i1_orf1	splicing factor U2af 38 kDa subunit [Aphidius gifuensis]	1	246	28.6	9.1	6	1	1	High	1	1.111	1.007	0.744	0.665	0.621	0.487	0.451	0.478	0.458	0.406	0.442	0.794	0.759	0.741
TRINITY_DN83622_c0_g1_i2_orf1	uncharacterized protein LOC114350344 [Ostrinia furnacalis]	1	45	4.9	5.02	40	2	1	High	1	0.917	0.956	1.224	1.264	1.259	1.309	1.33	1.421	1.384	1.469	2.252	0.904	1.037	1.244
TRINITY_DN93764_c0_g1_i1_orf1	microtubule-associated protein futsch isoform X4 [Ostrinia furnacalis]	3	145	16.4	9	22	3	3	High	1	1.156	1.197	1.475	1.549	1.471	1.29	1.21	1.394	1.471	1.261	1.48	1.121	1.165	1.178
TRINITY_DN18937_c0_g1_i1_orf1	uncharacterized protein LOC114351683 isoform X7 [Ostrinia furnacalis]	2	179	20.8	5.83	10	2	2	High	1	0.837	0.944	0.804	0.81	0.805	0.66	0.671	0.653	0.887	0.834	0.742	0.85	0.908	0.804
TRINITY_DN100821_c0_g1_i1_orf1	putative GMP synthase, partial [Operophtera brumata]	2	66	7.3	6.77	32	2	2	High	1	0.982	0.991	0.553	0.587	0.547	0.455	0.493	0.493	0.403	0.369	0.405	0.483	0.498	0.446
TRINITY_DN913_c0_g1_i6_orf1	uncharacterized protein LOC114354578 [Ostrinia furnacalis]	2	105	11.5	4.92	28	2	2	High	1	0.913	1.058	1.63	1.654	1.508	1.707	1.535	1.554	1.72	1.618	1.808	1.779	1.79	1.706
TRINITY_DN41280_c0_g1_i2_orf1	unnamed protein product [Plutella xylostella]	2	71	8	6.52	35	2	1	High	1	0.98	1.285	0.769	0.938	0.78	1.877	1.561	1.552	1.074	0.957	1.263	0.809	0.734	0.624
TRINITY_DN10066_c0_g2_i2_orf1	inositol polyphosphate 5-phosphatase K isoform X2 [Manduca sexta]	3	487	55.6	6.44	8	3	3	High	1	1.04	1.004	0.798	0.841	0.852	0.773	0.758	0.767	0.906	0.864	0.811	0.754	0.684	0.75
TRINITY_DN10118_c0_g1_i4_orf1	glutaredoxin-C4-like [Ostrinia furnacalis]	3	116	12.6	8.25	22	4	3	High	1	1.005	1.014	1.232	1.203	1.164	0.984	1.117	1.044	1.126	1.061	1.117	0.889	0.886	0.914
TRINITY_DN18624_c0_g1_i5_orf1	uncharacterized protein LOC114353135 [Ostrinia furnacalis]	1	301	34.8	7.36	5	1	1	High	1	1.06	0.974	1.23	1.066	1.077	1.433	1.292	1.1	0.953	1.513	1.111	10.956	10.681	10.262
TRINITY_DN6563_c0_g1_i1_orf1	cytochrome c oxidase assembly protein COX15 homolog [Ostrinia furnacalis]	3	429	47.4	9.92	7	3	3	High	1	0.994	1.019	0.432	0.4	0.511	0.436	0.442	0.503	0.478	0.437	0.393	0.671	0.633	0.6
TRINITY_DN5857_c0_g1_i13_orf1	uncharacterized protein LOC114353981 isoform X1 [Ostrinia furnacalis]	3	286	32.8	8.63	9	3	3	High	1	1.049	1.029	0.645	0.616	0.695	0.595	0.61	0.615	0.537	0.481	0.539	0.512	0.507	0.52
TRINITY_DN2356_c2_g1_i6_orf1	ER membrane protein complex subunit 3 [Ostrinia furnacalis]	1	250	28.6	9.07	5	1	1	High	1	1.09	1.09	0.919	0.779	0.873	0.574	0.563	0.704	0.632	0.636	0.537	0.717	0.707	0.778
TRINITY_DN2192_c1_g1_i1_orf1	unnamed protein product [Chrysodeixis includens]	3	492	54.6	8.22	6	3	3	High	1	0.946	0.953	0.843	0.892	0.923	0.974	0.892	0.919	0.93	1.15	0.982	0.825	0.828	0.945
TRINITY_DN1293_c0_g1_i4_orf1	putative fatty acyl-CoA reductase CG5065 [Ostrinia furnacalis]	3	521	59.2	7.42	7	3	3	High	1	1.007	1.043	0.987	0.952	0.905	0.856	0.807	0.796	0.752	0.713	0.863	1.889	1.891	1.862
TRINITY_DN13174_c0_g1_i4_orf1	N-alpha-acetyltransferase 35, NatC auxiliary subunit [Ostrinia furnacalis]	2	725	81.8	7.14	3	2	2	High	1	1.001	1.01	0.561	0.606	0.596	0.506	0.554	0.544	0.622	0.58	0.557	0.499	0.555	0.525
TRINITY_DN1902_c0_g1_i4_orf1	chemosensory protein 10 [Conogethes pinicolalis]	2	106	11.8	9.09	20	2	2	High	1	0.812	0.925	1.054	1.087	1.068	0.89	0.989	0.843	0.988	1.086	0.827	3.218	3.358	3.193
TRINITY_DN54269_c0_g1_i3_orf1	lopap-like [Ostrinia furnacalis]	2	78	9.2	8.43	26	3	1	High	1	1.122	1.169	1.068	0.919	0.996	1.74	1.424	1.404	1.464	1.649	1.4	2.725	2.619	2.457

TRINITY_DN154_c0_g1_i4_orf1	ER membrane protein complex subunit 4 [Ostrinia furnacalis]	2	172	19.1	9.31	10	2	2	High	1	1.017	0.963	0.603	0.598	0.595	0.537	0.545	0.588	0.547	0.566	0.445	0.768	0.705	0.678
TRINITY_DN4929_c1_g2_i5_orf1	guanylate kinase isoform X2 [Ostrinia furnacalis]	2	201	22.7	8.54	12	2	2	High	1	1.011	0.999	0.621	0.62	0.707	0.49	0.502	0.458	0.441	0.402	0.398	0.369	0.348	0.306
TRINITY_DN1506_c0_g1_i6_orfp1	TRINITY_DN1506_c0_g1_i6_m.57691 TRINITY_DN1506_c0_g1::TRINITY_DN1506	1	172	19	9.95	7	8	1	High	1	0.807	1.058	13.483	13.775	12.871	18.374	17.674	15.873	16.062	13.514	19.654	0.75	0.552	0.815
TRINITY_DN90289_c0_g1_i5_orf1	40S ribosomal protein S25 [Eumeta japonica]	2	79	9.1	10.21	33	4	2	High	1	0.912	0.997	0.615	0.673	0.676	0.636	0.629	0.547	0.664	0.656	0.643	0.681	0.679	0.687
TRINITY_DN129207_c0_g1_i1_orf1	U3 small nucleolar RNA-interacting protein 2 [Ostrinia furnacalis]	2	448	50.6	8.69	5	2	2	High	1	1.034	0.954	0.683	0.724	0.766	0.614	0.723	0.728	0.67	0.788	0.752	0.798	0.781	0.733
TRINITY_DN36592_c0_g1_i1_orf1	uncharacterized protein LOC114359903 [Ostrinia furnacalis]	2	294	33.4	8.31	8	2	2	High	1	0.982	0.911	0.751	0.838	0.771	0.758	0.726	0.738	0.744	0.705	0.777	0.703	0.752	0.647
TRINITY_DN3028_c0_g1_i1_orf1	pre-rRNA processing protein FTSJ3 [Ostrinia furnacalis]	2	868	99	9.14	3	2	2	High	1	0.932	0.967	0.459	0.492	0.415	0.495	0.552	0.501	0.499	0.418	0.387	0.385	0.466	0.442
TRINITY_DN782_c0_g1_i5_orf1	regulator of gene activity isoform X3 [Ostrinia furnacalis]	2	428	46.2	6.61	7	2	2	High	1	1.187	0.837	0.889	0.743	0.842	0.775	0.891	0.849	0.837	0.968	0.82	1.098	1.179	1.007
TRINITY_DN10619_c0_g5_i7_orf1	protein bunched, class 2/F/G isoform-like isoform X2 [Ostrinia furnacalis]	2	990	107.5	5.35	2	2	2	High	1	0.989	0.948	0.809	0.788	0.868	1.107	1.001	0.892	0.851	0.79	0.844	1.164	1.203	1.172
TRINITY_DN7488_c0_g1_i1_orf1	28S ribosomal protein S17, mitochondrial [Ostrinia furnacalis]	3	161	18.6	8.69	20	3	3	High	1	1.034	0.985	0.744	0.889	0.794	1.176	0.799	0.749	0.777	1.015	0.732	0.608	0.75	0.791
TRINITY_DN21435_c0_g1_i2_orf1	glycogen-binding subunit 76A isoform X1 [Ostrinia furnacalis]	1	562	62.5	4.89	4	1	1	High	1	0.922	1.09	1.826	1.934	1.786	1.654	1.961	1.411	1.599	1.551	1.855	1.538	1.441	1.195
TRINITY_DN5578_c0_g1_i10_orf1	unnamed protein product [Chilo suppressalis]	3	573	62.9	6.42	4	3	1	High	1	0.961	0.948	0.117	0.155	0.184	0.184	0.184	0.203	0.227	0.239	0.245	0.362	0.307	0.319
TRINITY_DN442_c0_g1_i10_orf1	tuberin isoform X4 [Helicoverpa armigera]	3	1174	127.3	7.66	4	3	3	High	1	1.072	0.967	0.886	0.916	0.794	0.887	0.868	1.028	0.72	0.757	0.826	1.148	0.881	0.872
TRINITY_DN4144_c0_g1_i7_orf1	uncharacterized protein LOC114350172 [Ostrinia furnacalis]	2	376	42	6.13	7	2	2	High	1	0.96	0.968	0.621	0.801	0.791	0.684	0.734	0.651	0.836	0.779	0.661	2.053	1.377	1.849
TRINITY_DN5421_c0_g1_i1_orf1	arylphorin subunit alpha-like [Ostrinia furnacalis]	4	147	17.2	9.04	21	5	1	High	1	1.021	1.077	2.264	2.384	2.33	2.174	2.231	2.096	2.667	2.417	3.079	2.065	2.263	2.29
TRINITY_DN10131_c0_g1_i7_orf1	aldo-keto reductase AKR2E4-like [Ostrinia furnacalis]	3	336	38	6.55	7	3	2	High	1	0.984	0.871	0.565	0.607	0.621	0.541	0.603	0.655	0.494	0.478	0.649	1.112	1.05	0.958
TRINITY_DN17838_c0_g1_i4_orf1	mitogen-activated protein kinase kinase kinase 4 [Ostrinia furnacalis]	2	354	39.3	6.83	7	2	2	High	1	0.75	0.998	1.347	1.346	1.303	1.316	1.342	1.489	1.384	2.011	1.518	1.53	1.42	1.481
TRINITY_DN130_c0_g1_i7_orf1	RNA-binding protein fusilli isoform X1 [Bombyx mori]	3	726	79.8	7.31	3	3	3	High	1	0.949	0.96	0.687	0.727	0.747	0.649	0.735	0.632	0.641	0.506	0.569	0.718	0.721	0.763
TRINITY_DN747_c0_g1_i1_orf1	trypsin, alkaline C-like [Ostrinia furnacalis]	1	274	30.5	7.97	6	1	1	High	1	1.061	1.24	0.554	0.68	0.551	0.516	0.454	0.527	0.883	0.682	0.899	0.549	0.482	0.543
TRINITY_DN51995_c0_g3_i1_orf1	circadian clock-controlled protein-like [Ostrinia furnacalis]	3	260	29.2	6.93	10	3	3	High	1	1.02	1.042	2.467	2.121	2.37	3.866	3.606	3.75	17.669	15.542	15.853	1.003	1.365	1.389
TRINITY_DN51568_c0_g1_i1_orf1	splicing factor 3A subunit 2 [Ostrinia furnacalis]	3	261	30	9.57	13	3	3	High	1	0.887	1.029	0.609	0.655	0.637	0.544	0.608	0.567	0.533	0.5	0.516	0.609	0.612	0.66
TRINITY_DN73224_c0_g4_i2_orf1	PREDICTED: poly(rC)-binding protein 3 isoform X2 [Vollenhovia emeryi]	2	553	57.5	9.04	5	2	2	High	1	0.934	1.042	0.691	0.578	0.66	0.665	0.736	0.707	0.689	0.646	0.632	1.327	1.445	1.251
TRINITY_DN14183_c0_g1_i3_orf1	multiple epidermal growth factor-like domains protein 6 [Ostrinia furnacalis]	2	373	40.5	6.07	9	2	2	High	1	1.034	1.056	0.69	0.737	0.743	0.756	0.817	0.825	0.724	0.812	0.789	0.678	0.916	0.814
TRINITY_DN104297_c0_g1_i1_orf1	tubulin-specific chaperone D [Ostrinia furnacalis]	2	98	11	5.81	29	2	1	High	1	0.929	0.984	0.902	0.93	1.034	1.702	1.874	1.541	1.31	1.574	1.384	1.29	1.023	1.19
TRINITY_DN37055_c0_g1_i1_orf1	ras GTPase-activating protein-binding protein 2-like, partial [Ostrinia furnacali	1	96	10.1	5.11	20	2	1	High	1	0.926	0.888	1.046	1.027	1.062	1.288	1.222	1.195	1.178	1.438	1.157	0.937	0.863	0.958
TRINITY_DN27300_c0_g1_i7_orfp1	TRINITY_DN27300_c0_g1_i7_m.71141 TRINITY_DN27300_c0_g1::TRINITY_DN27	3	81	9.2	9.48	35	4	2	High	1	0.968	1.032	3.72	3.899	3.403	1.771	1.822	2.133	2.397	1.954	2.64	1.508	1.509	1.599
TRINITY_DN11125_c0_g1_i1_orf1	LOW QUALITY PROTEIN: nuclear receptor-binding protein homolog [Ostrinia f	2	391	44.2	6.52	7	2	2	High	1	0.702	0.971	0.86	0.851	0.717	0.804	0.845	0.76	0.639	0.618	0.729	0.874	0.82	0.814
TRINITY_DN14953_c0_g1_i5_orf1	EEF1A lysine methyltransferase 2 [Ostrinia furnacalis]	2	216	24.1	4.78	11	2	2	High	1	0.936	0.945	0.549	0.618	0.608	0.567	0.505	0.515	0.56	0.565	0.547	0.595	0.525	0.473
TRINITY_DN9569_c1_g1_i7_orf1	V-set and immunoglobulin domain-containing protein 1-like isoform X1 [Ostr	1	286	31.9	4.34	4	2	1	High	1	0.998	0.929	1.301	1.437	1.436	1.346	1.42	1.287	1.334	1.395	1.479	0.941	0.936	0.956
TRINITY_DN6771_c0_g2_i1_orf1	putative endoplasmin, partial [Cotesia chilonis]	2	805	91.8	4.91	2	3	1	High	1	0.899	0.922	1.032	1.351	1.249	1.455	1.489	1.591	1.344	1.519	1.177	1.438	1.259	1.329
TRINITY_DN26993_c1_g1_i8_orf1	endocuticle structural glycoprotein ABD-4-like [Ostrinia furnacalis]	1	123	13.5	4.67	10	2	1	High	1	1.014	1.122	1.417	1.46	1.56	1.364	1.348	1.215	1.196	1.24	1.148	1.056	0.915	1.003
TRINITY_DN20009_c0_g1_i1_orf1	vimentin [Homo sapiens]	2	453	52.1	5.02	5	2	1	High	1	0.912	1.365	1.601	1.413	1.447	1.642	2.146	1.881	3.281	2.156	4.325	1.921	2.644	2.229
TRINITY_DN21719_c0_g1_i2_orf1	chymotrypsin-2-like [Ostrinia furnacalis]	2	144	15.6	4.82	14	3	2	High	1	1.048	1.036	0.912	0.893	0.878	0.995	0.891	0.907	1.13	0.976	1.049	6.673	6.89	6.331
TRINITY_DN1534_c0_g1_i3_orf1	peptidoglycan recognition protein-like [Ostrinia furnacalis]	2	99	11.1	7.66	23	3	2	High	1	1.002	1.119	1.251	1.366	1.344	1.932	1.961	1.728	3.301	3.095	3.401	1.316	1.154	1.252
TRINITY_DN1860_c0_g1_i1_orf1	26S proteasome non-ATPase regulatory subunit 5 [Ostrinia furnacalis]	2	509	57.3	6.54	5	2	1	High	1	1.065	1.22	1.229	1.28	1.241	0.896	0.823	1.17	1.006	0.994	1.019	1.077	1.147	1.051
TRINITY_DN95850_c0_g4_i3_orf1	calreticulin [Cotesia glomerata]	2	378	43.9	4.54	8	7	1	High	1	1.002	0.955	1.554	1.684	1.684	1.699	1.432	1.584	1.51	1.94	1.295	1.39	1.322	1.618
TRINITY_DN64772_c0_g1_i1_orf1	aldehyde dehydrogenase, partial [Mythimna separata]	2	53	5.9	5.87	42	6	2	High	1	1.023	1.044	2.242	2.091	2.194	2.979	3.093	2.921	3.415	2.921	3.251	3.406	3.264	3.208
TRINITY_DN5581_c0_g1_i4_orf1	activating signal cointegrator 1 [Ostrinia furnacalis]	2	309	35.5	7.9	7	2	2	High	1	0.933	0.826	1.193	1.289	1.016	0.997	1.076	1.055	0.936	1.17	1.013	0.885	0.846	1.185
TRINITY_DN110376_c0_g1_i1_orf1	nucleolysin TIAR [Osmia bicornis bicornis]	2	391	43.5	7.93	6	2	2	High	1	0.95	0.915	0.857	0.824	0.921	0.844	0.855	0.946	0.83	0.826	0.693	0.759	0.784	0.726
TRINITY_DN8394_c1_g1_i9_orf1	uncharacterized protein LOC114364294 [Ostrinia furnacalis]	3	262	30	9.74	9	3	3	High	1	0.949	0.935	0.849	0.934	0.952	0.888	0.912	0.867	0.818	0.73	0.857	0.722	0.77	0.718
TRINITY_DN30663_c0_g1_i1_orf1	surfeit locus protein 6 homolog [Ostrinia furnacalis]	2	288	33.5	10.15	6	2	2	High	1	1.002	0.951	0.231	0.304	0.291	0.225	0.205	0.198	0.296	0.259	0.306	0.176	0.242	0.228
TRINITY_DN27111_c0_g1_i1_orf1	copper transport protein ATOX1 [Helicoverpa armigera]	2	72	7.7	7.99	32	4	2	High	1	0.919	0.95	0.617	0.869	0.68	0.721	0.961	0.707	0.821	1.539	1.599	1.478	1.004	2.016
TRINITY_DN883_c0_g1_i8_orf1	diacylglycerol O-acyltransferase 1 isoform X1 [Ostrinia furnacalis]	2	491	57.1	9.38	5	2	2	High	1	0.874	0.957	0.579	0.591	0.529	0.577	0.758	0.665	0.413	0.512	0.518	0.487	0.521	0.609
TRINITY_DN38693_c0_g1_i4_orf1	protein RER1 [Ostrinia furnacalis]	2	195	23	9.54	10	3	2	High	1	1.116	1.034	0.767	0.656	0.684	0.671	0.707	0.796	0.643	0.749	0.585	0.838	0.757	0.718
TRINITY_DN13330_c0_g1_i4_orf1	carboxylesterase [Cnaphalocrocis medinalis]	2	559	62.7	7.83	4	2	2	High	1	0.95	0.912	1.04	1.183	0.942	1.673	1.623	1.342	1.22	0.879	1.275	1.7	1.577	1.487
TRINITY_DN27968_c0_g2_i2_orf1	nucleolin-like [Ostrinia furnacalis]	2	299	33.3	10.39	8	2	2	High	1	0.847	0.865	0.875	0.768	0.794	0.866	0.829	0.96	0.821	0.864	0.937	3.39	3.652	3.496
TRINITY_DN1005_c0_g1_i5_orf1	hypothetical protein evm_008839 [Chilo suppressalis]	2	375	41.2	6.47	6	2	1	High	1	0.907	0.819	0.749	0.799	0.794	1.114	0.936	0.778	0.81	0.407	0.677	1.68	1.785	1.858
TRINITY_DN8646_c0_g1_i2_orf1	chromatin accessibility complex protein 1 [Ostrinia furnacalis]	2	135	15.3	4.96	21	2	2	High	1	1.01	1.113	0.773	0.782	0.907	0.989	0.925	0.921	0.963	1.124	0.978	0.927	0.969	1.018
TRINITY_DN44070_c0_g2_i2_orf1	protein Gawky isoform X2 [Ostrinia furnacalis]	2	1301	139.9	6.96	2	2	2	High	1	0.957	0.926	0.679	0.699	0.815	0.76	0.85	0.805	0.757	1.443	0.649	1.313	1.208	1.342
TRINITY_DN9904_c0_g1_i1_orf1	unnamed protein product [Chrysodeixis includens]	2	673	74.7	6	4	2	2	High	1	0.969	1.007	1.031	0.977	1.133	1.016	1.212	1.077	1.244	1.819	1.229	0.853	0.926	0.95
TRINITY_DN90327_c0_g1_i1_orf1	hypothetical protein HF086_000910, partial [Spodoptera exigua]	2	69	7.1	5.74	36	2	2	High	1	1.112	1.111	1.383	1.293	1.294	1.254	1.157	1.042	1.147	1.095	1.085	1.491	1.406	1.426
TRINITY_DN16894_c0_g1_i5_orf1	dnaJ homolog subfamily C member 5 isoform X1 [Colias croceus]	1	204	22.2	6.19	10	1	1	High	1	0.891	0.881	0.399	0.491	0.504	0.687	0.377	0.447	0.377	0.328	0.467	0.661	0.635	0.515
TRINITY_DN4018_c0_g1_i4_orf1	pyrimidodiazepine synthase-like [Ostrinia furnacalis]	2	240	28.6	7.11	10	2	2	High	1	0.952	0.955	0.866	0.99	1.012	0.844	0.929	0.94	1.059	0.9	0.907	0.911	0.888	1.043
TRINITY_DN37699_c0_g1_i4_orfp1	TRINITY_DN37699_c0_g1_i4_m.58777 TRINITY_DN37699_c0_g1::TRINITY_DN37	1	121	12.7	8.76	18	1	1	High	1	1.097	0.984	0.581	0.522	0.478	0.537	0.458	0.275	0.184	0.369	0.243	0.278	0.301	0.333
TRINITY_DN26251_c0_g1_i1_orf1	serine/arginine-rich splicing factor 1A [Neodiprion lecontei]	3	247	28.3	9.94	11	4	3	High	1	0.973	0.92	0.696	0.754	0.708	0.61	0.626	0.623	0.548	0.523	0.576	0.767	0.764	0.779
TRINITY_DN20346_c0_g1_i1_orf1	NADH dehydrogenase [ubiquinone] 1 alpha subcomplex subunit 6 [Ostrinia fu	2	125	14.8	9.67	12	3	2	High	1	1.057	1.006	0.33	0.314	0.353	0.361	0.419	0.373	0.349	0.368	0.349	0.398	0.438	0.375
TRINITY_DN15291_c0_g1_i11_orf1	uncharacterized protein LOC114353772 [Ostrinia furnacalis]	2	190	21.5	6.52	10	3	2	High	1	0.952	0.995	1.204	1.061	1.067	1.274	1.362	1.372	1.985	1.91	1.625	1.954	1.993	2.09
TRINITY_DN5840_c0_g1_i6_orf1	catenin alpha isoform X2 [Ostrinia furnacalis]	3	919	101.6	6.46	4	3	3	High	1	1.04	1.03	0.829	0.802	0.907	0.76	0.733	0.835	0.893	0.713	0.799	1.218	1.37	1.38
TRINITY_DN4836_c0_g1_i4_orf1	hypothetical protein O3G_MSEX014157 [Manduca sexta]	2	186	21.4	5.01	11	2	2	High	1	0.957	0.901	0.753	0.79	0.798	0.597	0.633	0.685	0.584	0.69	0.59	0.753	0.773	0.744
TRINITY_DN141381_c0_g1_i1_orf1	very long-chain specific acyl-CoA dehydrogenase, mitochondrial [Chelonus in:	2	629	69	7.83	4	3	2	High	1	0.948	1.027	0.924	0.553	0.841	0.789	0.621	0.926	0.852	0.869	0.501	0.434	0.364	0.423
TRINITY_DN2593_c0_g2_i1_orf1	midgut carboxypeptidase [Loxostege sticticalis]	2	435	48.9	6.55	6	2	1	High	1	1.05	0.914	0.447	0.314	0.554	0.519	0.472	0.494	0.337	0.642	0.545	0.413	0.413	0.466
TRINITY_DN24751_c0_g1_i1_orf1	NADH dehydrogenase [ubiquinone] 1 beta subcomplex subunit 7-like [Ostrinia	3	118	14.3	8.16	30	4	3	High	1	1.076	1.019	0.482	0.519	0.568	0.517	0.551	0.558	0.496	0.438	0.466	0.511	0.552	0.491
TRINITY_DN8833_c0_g1_i1_orf1	nucleolar protein 16 [Ostrinia furnacalis]	2	201	24.1	9.66	8	2	2	High	1	1.04	1.026	0.325	0.377	0.361	0.41	0.403	0.423	0.371	0.337	0.292	0.334	0.408	0.369
TRINITY_DN5585_c0_g1_i4_orf1	calpain-7-like isoform X3 [Ostrinia furnacalis]	4	796	90.8	7.2	4	4	4	High	1	1.038	1.121	1.193	1.423	1.393	1.587	1.296	1.213	1.123	1.068	1.147	1.044	1.141	1.245
TRINITY_DN855_c0_g1_i5_orf1	hypothetical protein HF086_009970 [Spodoptera exigua]	1	123	13.3	4.13	12	2	1	High	1	0.994	0.981	1.327	1.425	1.304	1.231	1.185	1.056	1.004	0.872	1.074	1.298	1.24	1.335
TRINITY_DN66453_c0_g1_i4_orfp1	TRINITY_DN66453_c0_g1_i4_m.7345 TRINITY_DN66453_c0_g1::TRINITY_DN664	2	92	9.7	8.65	21	3	1	High	1	0.989	1.054	2.306	2.301	2.249	2.584	2.862	2.537	1.502	1.588	1.856	1.044	1.008	1.029
TRINITY_DN3019_c0_g1_i1_orf1	uncharacterized protein LOC114355530 isoform X1 [Ostrinia furnacalis]	1	95	10.6	7.99	18	1	1	High	1	1.047	0.97	0.732	0.862	0.775	1.045	1.098	1.026	0.906	1.096	0.999	0.618	0.718	0.643
TRINITY_DN4679_c0_g2_i13_orf1	GATOR complex protein MIOS [Ostrinia furnacalis]	2	268	28.7	7.43	8	2	2	High	1	0.903	0.886	1.282	1.233	1.241	1.189	1.241	1.289	1.076	0.909	1.061	1.292	1.134	1.055
TRINITY_DN2796_c0_g1_i28_orf1	phosphofurin acidic cluster sorting protein 2 isoform X1 [Pectinophora gossypi	3	576	62.4	8.91	6	3	3	High	1	1.008	0.994	1.425	1.705	1.516	1.701	1.611	1.569	1.695	1.443	1.396	1.596	1.865	1.604
TRINITY_DN43841_c0_g1_i1_orf1	39S ribosomal protein L4, mitochondrial [Ostrinia furnacalis]	2	284	32.2	9.58	8	3	2	High	1	0.906	0.905	0.731	0.749	0.703	0.781	0.78	0.837	0.736	0.86	0.802	0.86	0.84	0.818
TRINITY_DN702_c0_g1_i15_orf1	NF-kappa-B inhibitor cactus [Ostrinia furnacalis]	1	339	37.1	5.31	4	1	1	High	1	1.237	1.322	0.862	1.081	0.588	0.856	0.862	1.129	0.87	1.183	0.723	1.04	1.056	0.834
TRINITY_DN47575_c0_g1_i1_orf1	PREDICTED: splicing factor 1-like [Fopius arisanus]	2	620	66.9	9.09	2	2	2	High	1	0.826	0.945	0.786	0.729	0.569	0.593	0.517	0.526	0.393	0.512	0.523	0.521	0.483	0.54
TRINITY_DN6875_c0_g1_i2_orf1	hypothetical protein evm_002936 [Chilo suppressalis]	2	289	31.8	5.27	7	2	2	High	1	1.121	1.078	1.034	0.957	1.1	1.227	1.088	1.114	1.016	1.332	0.958	1.179	1.225	1.076
TRINITY_DN26411_c0_g1_i2_orfp1	TRINITY_DN26411_c0_g1_i2_m.24123 TRINITY_DN26411_c0_g1::TRINITY_DN26	3	114	10.9	4.88	46	3	1	High	1	1.086	0.979	0.098	0.037	0.158	0.377	0.472	0.611	0.102	0.05	0.092	0.103	0.118	0.08
TRINITY_DN11823_c1_g1_i2_orf1	LOW QUALITY PROTEIN: uncharacterized protein LOC114362902 [Ostrinia furr	2	360	38.4	7.68	6	2	2	High	1	1.014	1.116	1.042	1.258	1.224	1.119	1.189	1.211	1.075	1.337	1.157	0.941	0.828	0.964
TRINITY_DN15059_c0_g1_i9_orf1	heterogeneous nuclear ribonucleoprotein K isoform X3 [Danaus plexippus ple›	1	148	14.9	11.09	12	1	1	High	1	0.969	1.034	0.979	1.061	1.053	1.009	1.173	1.003	0.956	0.803	0.881	0.873	0.995	1.095
TRINITY_DN122321_c0_g1_i1_orf1	uncharacterized protein LOC114356271 [Ostrinia furnacalis]	1	68	7.6	4.59	32	1	1	High	1	1.141	0.906	2.603	1.706	2.135	2.592	2.33	3.234	2.681	2.47	1.981	1.824	1.567	1.649
TRINITY_DN2748_c0_g1_i6_orf1	uncharacterized protein LOC114352811 [Ostrinia furnacalis]	3	419	48	9.06	8	3	3	High	1	1.072	1.019	0.544	0.679	0.53	0.426	0.449	0.511	0.585	0.527	0.571	0.43	0.422	0.436
TRINITY_DN124300_c0_g1_i2_orf1	protein transport protein Sec23A isoform X1 [Venturia canescens]	2	775	86.4	6.87	3	2	2	High	1	1.069	0.985	0.65	0.755	0.698	0.648	0.622	0.7	0.536	0.63	0.69	0.687	0.642	0.71
TRINITY_DN35662_c0_g1_i5_orf1	hypothetical protein evm_006436 [Chilo suppressalis]	2	295	32.9	6.27	6	2	2	High	1	1.044	1.015	0.217	0.221	0.304	0.245	0.269	0.231	0.277	0.251	0.241	0.313	0.33	0.306
TRINITY_DN46140_c0_g1_i1_orf1	protein PRRC2A-like isoform X4 [Ostrinia furnacalis]	1	238	24.1	9.92	10	1	1	High	1	1.145	1.016	0.522	0.515	0.522	0.739	0.328	0.478	0.662	0.536	0.55	0.961	0.903	0.678
TRINITY_DN18909_c0_g1_i6_orf1	unnamed protein product [Euphydryas editha]	2	537	60.8	4.93	4	2	1	High	1	1.048	0.993	0.503	0.578	0.58	0.728	0.988	0.718	0.65	0.9	0.531	0.557	0.501	0.641
TRINITY_DN3111_c0_g1_i5_orf1	CCAAT/enhancer-binding protein zeta-like [Ostrinia furnacalis]	2	1010	114.2	6.32	3	2	2	High	1	1.056	1.097	0.71	0.686	0.73	0.582	0.755	0.662	0.709	0.816	0.644	0.827	0.74	0.803
TRINITY_DN19295_c0_g1_i1_orf1	ubiquitin carboxyl-terminal hydrolase 30 homolog [Galleria mellonella]	2	234	24.4	8.41	12	2	2	High	1	0.896	0.863	0.771	0.815	0.831	0.881	0.822	0.841	0.876	0.828	0.717	0.929	0.915	0.958
TRINITY_DN51737_c0_g1_i3_orf1	N-alpha-acetyltransferase 40 [Ostrinia furnacalis]	1	242	27.5	8.05	10	1	1	High	1	0.95	1.015	0.645	0.719	0.898	0.808	0.704	0.807	0.773	0.686	0.788	1.123	1.112	0.94
TRINITY_DN7534_c0_g1_i15_orf1	protein-glucosylgalactosylhydroxylysine glucosidase isoform X2 [Ostrinia furna	2	423	48.7	5.11	5	2	2	High	1	1.11	1.118	2.881	2.767	2.754	2.536	2.348	2.394	1.857	1.899	2.06	2.774	2.433	2.628
TRINITY_DN12932_c0_g1_i1_orf1	COP9 signalosome complex subunit 3 [Ostrinia furnacalis]	2	423	47.9	6.52	6	2	2	High	1	0.98	1.017	1.306	1.209	1.175	1.097	1.175	1.215	1.066	1.128	1.026	0.987	1.004	1.09
TRINITY_DN4025_c0_g1_i13_orf1	tetratricopeptide repeat protein 14 homolog isoform X2 [Ostrinia furnacalis]	2	316	35.8	9.94	8	2	2	High	1	1.054	1.148	0.67	0.631	0.709	0.649	0.568	0.54	0.503	0.326	0.364	0.698	0.722	0.569
TRINITY_DN4182_c0_g1_i6_orf1	tetratricopeptide repeat protein 19 homolog, mitochondrial [Ostrinia furnacalis	2	327	37.4	7.94	7	3	2	High	1	0.938	0.85	0.809	0.787	0.82	0.861	0.858	0.947	0.862	0.818	0.892	0.754	0.722	0.718
TRINITY_DN88640_c0_g1_i1_orf1	tetratricopeptide repeat protein 27-like [Ostrinia furnacalis]	2	781	90.3	5.92	3	3	2	High	1	0.982	1.022	0.637	0.643	0.662	0.708	0.759	0.814	0.739	0.725	0.563	0.699	0.701	0.67
TRINITY_DN4795_c0_g1_i2_orf1	D-amino-acid oxidase isoform X1 [Ostrinia furnacalis]	1	176	19.6	8.6	8	1	1	High	1	0.887	0.877	0.577	0.618	0.639	0.73	0.854	0.653	0.611	0.618	0.437	0.677	0.623	0.446
TRINITY_DN81791_c0_g2_i2_orf1	UDP-N-acetylglucosamine--peptide N-acetylglucosaminyltransferase 110 kDa	2	1057	118.1	6.86	2	3	1	High	1	1.02	0.972	1.127	1.157	0.994	0.935	0.897	0.898	0.917	0.855	0.938	1.08	1.061	1.184
TRINITY_DN268_c1_g1_i7_orf1	unnamed protein product [Spodoptera littoralis]	2	586	62.2	8.98	4	2	2	High	1	1.031	1.046	1.172	1.147	1.092	1.246	1.074	1.187	1.201	1.248	1.2	2.675	2.66	2.698
TRINITY_DN111985_c0_g1_i1_orf1	Parkinson disease protein 7 [Homo sapiens]	1	189	19.9	6.79	13	1	1	High	1	1.502	1.478	1.332	2.097	2.2	2.364	2.286	2.338	2.896	2.023	5.894	2.048	2.096	1.801

TRINITY_DN13285_c0_g1_i9_orf1	E3 ubiquitin-protein ligase RNF13 isoform X1 [Ostrinia furnacalis]	2	441	48.6	6.93	5	2	2	High	1	0.935	1.042	1.36	1.366	1.366	1.199	1.215	1.368	1.243	1.169	1.314	1.505	1.454	1.42
TRINITY_DN64118_c0_g1_i4_orf1	RNA-binding protein with serine-rich domain 1-A-like [Ostrinia furnacalis]	2	232	25.5	10.77	13	2	2	High	1	1.02	1.196	1.044	1.079	1.274	1.261	1.148	1.202	0.976	1.293	1.402	1.137	1.193	1.006
TRINITY_DN6688_c0_g1_i5_orf1	hypothetical protein evm_011309 [Chilo suppressalis]	2	238	27.5	4.4	8	2	2	High	1	0.984	1.004	0.951	1.008	0.993	0.876	1.115	0.993	0.935	0.888	0.908	0.942	0.924	0.849
TRINITY_DN42759_c0_g3_i1_orf1	fatty acid synthase-like [Ostrinia furnacalis]	2	768	84.3	7.01	3	2	2	High	1	0.978	1	0.305	0.277	0.335	0.315	0.312	0.296	0.321	0.336	0.337	0.301	0.223	0.255
TRINITY_DN86844_c0_g2_i1_orf1	spermine oxidase-like isoform X1 [Ostrinia furnacalis]	2	419	46.8	5.19	5	2	1	High	1	0.97	0.96	0.423	0.495	0.482	0.294	0.48	0.378	0.376	0.409	0.426	0.373	0.358	0.313
TRINITY_DN1231_c0_g1_i4_orf1	AN1-type zinc finger protein 6 isoform X1 [Galleria mellonella]	2	255	28.5	9.45	7	2	2	High	1	1.001	0.903	0.675	0.657	0.714	0.678	0.771	0.872	0.696	0.583	0.624	1.245	1.328	1.305
TRINITY_DN31225_c0_g1_i1_orf1	ribosome biogenesis protein BMS1 homolog [Ostrinia furnacalis]	2	1089	124.6	8.41	2	2	2	High	1	1.024	0.947	2.208	2.167	2.111	2.084	2.119	2.088	1.456	1.256	1.556	0.792	0.897	0.828
TRINITY_DN54543_c0_g5_i2_orf1	reactive oxygen species modulator 1 [Papilio machaon]	1	79	8.2	9.54	23	1	1	High	1	0.95	1.082	0.352	0.251	0.357	0.367	0.328	0.376	0.37	0.317	0.344	0.368	0.406	0.38
TRINITY_DN2423_c0_g2_i2_orf1	serine palmitoyltransferase 3 [Ostrinia furnacalis]	2	568	62.3	9.8	4	2	2	High	1	0.97	1.047	1.02	0.976	0.935	0.982	1.017	1.059	0.925	1.252	0.895	0.9	0.952	0.94
TRINITY_DN1030_c0_g1_i6_orf1	gamma-glutamyl hydrolase A-like isoform X1 [Ostrinia furnacalis]	2	421	48.4	6.47	6	2	2	High	1	1.127	1.02	1.926	1.933	1.891	2.129	2.554	2.339	2.065	2.014	2.017	2.017	2.141	2.227
TRINITY_DN27723_c0_g1_i1_orf1	putative uncharacterized protein DDB_G0282133 isoform X1 [Ostrinia furnacali	2	170	19.3	4.86	14	2	1	High	1	0.969	0.927	0.731	0.692	0.738	0.797	0.821	0.796	0.68	0.691	0.631	1.352	1.326	1.3
TRINITY_DN56877_c0_g1_i4_orf1	programmed cell death protein 2-like [Ostrinia furnacalis]	2	401	43.5	4.58	6	2	2	High	1	0.92	1.044	0.913	0.826	0.888	1.024	1.051	1.196	0.823	1.085	0.859	0.801	0.833	0.897
TRINITY_DN14185_c0_g1_i1_orf1	uncharacterized protein LOC114358675 [Ostrinia furnacalis]	2	159	18.4	4.78	19	2	2	High	1	1.076	1.119	2.572	2.833	2.602	2.116	2.124	1.944	2.565	2.423	2.881	1.356	1.378	1.308
TRINITY_DN66302_c0_g1_i1_orf1	carboxypeptidase B-like [Ostrinia furnacalis]	1	422	47.1	6.07	3	1	1	High	1	1.089	1.06	0.219	0.2	0.229	0.293	0.343	0.3	0.237	0.314	0.225	0.194	0.272	0.305
TRINITY_DN34399_c0_g1_i1_orf1	cysteine synthase-like [Ostrinia furnacalis]	3	326	34.6	5.81	9	3	3	High	1	0.943	0.915	0.802	0.875	0.84	0.719	0.794	0.815	0.695	0.664	0.727	0.635	0.643	0.623
TRINITY_DN1882_c0_g1_i4_orf1	zinc transporter ZIP13 homolog [Ostrinia furnacalis]	2	394	42.2	5.47	7	2	2	High	1	0.88	0.91	0.681	0.909	0.806	0.695	0.724	0.639	0.449	0.367	0.528	0.562	0.722	0.643
TRINITY_DN3431_c0_g1_i1_orf1	3-hydroxyacyl-CoA dehydrogenase type-2-like [Ostrinia furnacalis]	1	256	27.5	7.88	7	1	1	High	1	0.991	1.05	1.13	1.256	1.263	1.049	1.425	1.255	1.627	1.31	1.346	1.342	1.197	1.193
TRINITY_DN57856_c0_g2_i1_orf1	cytochrome P450 6B2-like [Ostrinia furnacalis]	2	68	7.2	9.83	38	2	2	High	1	0.934	0.986	1.323	1.291	1.44	1.568	1.383	1.423	0.923	0.919	0.859	0.676	0.588	0.676
TRINITY_DN5055_c0_g1_i12_orf1	probable peroxisomal acyl-coenzyme A oxidase 1 [Ostrinia furnacalis]	2	665	74.6	7.23	5	2	2	High	1	0.891	0.935	1.262	1.211	1.242	1.182	1.155	1.464	0.991	0.78	0.957	1.14	1.11	1.112
TRINITY_DN7064_c0_g1_i20_orfp1	TRINITY_DN7064_c0_g1_i20_m.53649 TRINITY_DN7064_c0_g1::TRINITY_DN706	2	255	28.2	7.28	9	2	1	High	1	0.991	1.013	1.177	1.382	1.275	1.565	1.884	1.55	1.559	3.137	1.914	0.908	0.725	1.131
TRINITY_DN128231_c0_g1_i5_orf1	glutathione S-transferase sigma3 [Glyphodes pyloalis]	1	85	9.5	5.9	14	2	1	High	1	0.907	0.928	0.928	1.021	0.926	0.715	0.823	0.75	2.495	2.503	2.889	1.042	1.04	1.059
TRINITY_DN18804_c0_g1_i5_orf1	zinc finger protein Xfin-like [Ostrinia furnacalis]	3	1583	179.5	5.85	2	3	3	High	1	0.998	1.161	1.181	1.265	1.277	1.166	1.143	1.049	1.607	1.298	1.725	2.892	2.91	2.75
TRINITY_DN9560_c0_g1_i5_orf1	uncharacterized protein LOC114357350 [Ostrinia furnacalis]	2	245	27.1	5.48	11	2	1	High	1	0.985	1.128	2.071	2.312	2.118	1.753	1.441	1.676	1.673	1.351	1.833	0.629	0.538	0.423
TRINITY_DN7976_c0_g1_i4_orf1	unnamed protein product [Chilo suppressalis]	3	1396	161.6	8.66	2	3	3	High	1	0.995	1.076	1.053	1.163	1.041	0.906	1.189	1.033	0.901	0.807	1.006	0.877	0.913	0.874
TRINITY_DN11962_c0_g1_i2_orf1	BTB/POZ domain-containing protein 2-like [Ostrinia furnacalis]	3	429	48.6	7.09	7	3	2	High	1	0.981	1.033	1.43	1.35	1.435	0.891	0.911	0.886	0.896	0.973	0.957	0.847	0.926	0.928
TRINITY_DN394_c0_g1_i4_orf1	uncharacterized protein LOC114351483 [Ostrinia furnacalis]	2	114	12.6	5.86	19	2	1	High	1	1.117	1.053	1.622	1.851	1.785	2.67	2.684	2.237	2.331	2.597	2.447	0.901	0.989	1.277
TRINITY_DN11263_c0_g1_i5_orf1	SET domain-containing protein SmydA-8 [Ostrinia furnacalis]	2	494	54.9	5.83	5	4	2	High	1	0.893	0.901	1.455	1.524	1.408	1.055	1.294	1.183	1.221	1.298	1.02	1.087	1.085	1.197
TRINITY_DN5467_c0_g1_i5_orf1	synaptic vesicle glycoprotein 2B-like isoform X2 [Ostrinia furnacalis]	1	535	57.3	7.8	2	3	1	High	1	1.057	1.114	1.816	1.645	1.674	1.592	1.793	1.785	1.587	1.32	1.61	3.026	3.013	2.909
TRINITY_DN5169_c0_g1_i5_orf1	ero1-like protein isoform X1 [Ostrinia furnacalis]	2	524	59.7	6.02	6	2	2	High	1	1.058	0.885	0.71	0.662	0.696	0.688	0.705	0.649	0.61	0.657	0.734	0.716	0.629	0.656
TRINITY_DN4916_c0_g2_i1_orf1	uncharacterized protein LOC114357135, partial [Ostrinia furnacalis]	2	1251	141	8.57	2	2	2	High	1	0.945	1.014	0.932	1.089	1.03	1.265	1.142	1.296	1.205	1.108	1.156	0.973	0.859	0.908
TRINITY_DN117707_c0_g1_i3_orf1	acyl-CoA synthetase family member 2, mitochondrial isoform X1 [Ostrinia furn	2	580	63.6	7.2	4	2	2	High	1	1.09	1.104	0.691	0.751	0.779	0.788	0.794	0.732	0.809	0.793	0.693	0.716	0.61	0.615
TRINITY_DN16912_c0_g1_i1_orf1	COP9 signalosome complex subunit 5 [Ostrinia furnacalis]	2	348	38.8	6.37	7	2	2	High	1	1.1	1.025	0.908	1.042	1.003	1.097	0.986	1.228	1.031	1.416	1.002	1.185	0.919	1.057
TRINITY_DN22609_c0_g2_i3_orf1	THO complex subunit 5 homolog [Ostrinia furnacalis]	3	691	77.9	8.31	5	3	3	High	1	1.065	1.039	0.925	0.975	0.959	0.899	0.987	0.839	0.953	0.929	0.972	0.805	1.014	0.975
TRINITY_DN45271_c0_g1_i1_orf1	double-strand break repair protein MRE11 [Ostrinia furnacalis]	1	45	5.1	8.98	27	2	1	High	1	1.14	1.097	1.51	1.425	1.472	1.408	1.492	1.335	1.433	1.288	1.542	1.926	1.746	1.75
TRINITY_DN23416_c1_g1_i2_orf1	ran-specific GTPase-activating protein-like [Ostrinia furnacalis]	3	225	25.8	4.94	12	3	3	High	1	0.984	0.924	1.096	1.053	1.187	1.087	1.112	1.096	0.906	0.943	1.027	1.252	1.185	1.158
TRINITY_DN36817_c0_g1_i1_orf1	uncharacterized protein LOC114357350 [Ostrinia furnacalis]	1	73	7.6	4.94	29	1	1	High	1	1.025	0.987	0.726	0.823	0.729	0.656	0.663	0.674	0.79	0.622	0.584	0.232	0.385	0.352
TRINITY_DN1304_c0_g1_i6_orf1	uncharacterized protein LOC114359545 [Ostrinia furnacalis]	1	81	8.8	6.47	14	4	1	High	1	0.981	1.05	2.085	2.107	2.004	2.233	1.689	1.722	1.822	2.022	2.047	2.209	2.139	2.116
TRINITY_DN12826_c0_g1_i1_orf1	uncharacterized protein LOC114363296 [Ostrinia furnacalis]	2	188	20.4	5.11	10	2	2	High	1	0.958	0.894	0.669	0.697	0.745	0.771	0.746	0.768	0.655	0.588	0.722	0.747	0.725	0.735
TRINITY_DN12683_c0_g1_i3_orf1	sulfated surface glycoprotein 185-like [Ostrinia furnacalis]	2	250	27.1	6.99	9	2	2	High	1	1.002	0.988	0.478	0.481	0.466	0.479	0.479	0.447	0.457	0.425	0.453	0.36	0.389	0.371
TRINITY_DN44557_c0_g1_i4_orf1	serine hydrolase-like protein [Ostrinia furnacalis]	2	92	10.4	9.57	16	2	2	High	1	0.965	0.949	0.566	0.714	0.651	0.475	0.691	0.541	0.45	0.516	0.48	0.457	0.531	0.445
TRINITY_DN648_c0_g1_i5_orf1	uncharacterized protein LOC114360051 [Ostrinia furnacalis]	2	660	75	9.45	4	2	2	High	1	0.854	0.917	1.229	1.233	1.207	1.128	1.345	0.965	0.936	1.274	1.229	1.309	1.043	1.266
TRINITY_DN4798_c0_g1_i3_orf1	unnamed protein product [Spodoptera exigua]	3	487	54.7	5.48	7	3	3	High	1	0.925	1.069	1.063	1.221	1.166	0.736	0.815	0.784	0.915	0.823	0.88	1.226	1.226	1.258
TRINITY_DN24325_c0_g1_i12_orf1	MICOS complex subunit MIC10-like [Ostrinia furnacalis]	1	77	8.3	8.47	14	2	1	High	1	1.044	1.041	0.714	0.642	0.61	0.648	0.729	0.638	0.741	0.69	0.658	0.581	0.589	0.624
TRINITY_DN1252_c0_g1_i1_orf1	WD repeat-containing protein 47 isoform X1 [Ostrinia furnacalis]	2	577	63.4	8.81	3	2	1	High	1	1.495	1.64	1.834	2.087	2.409	2.141	1.561	1.925	2.213	1.608	2.207	1.05	1.554	1.078
TRINITY_DN45446_c0_g1_i2_orf1	peptide transporter family 1-like isoform X1 [Ostrinia furnacalis]	2	697	78.5	6.25	3	3	2	High	1	1.049	0.916	0.454	0.51	0.567	0.491	0.528	0.554	0.532	0.565	0.624	0.433	0.48	0.464
TRINITY_DN8411_c1_g1_i1_orf1	CCA tRNA nucleotidyltransferase 1, mitochondrial [Ostrinia furnacalis]	2	343	40	7.21	6	2	2	High	1	0.856	1.019	0.724	0.877	0.802	0.871	0.815	0.897	0.907	1.09	0.753	0.813	0.795	0.849
TRINITY_DN43355_c0_g1_i1_orf1	uncharacterized protein CG16817-like [Ostrinia furnacalis]	1	164	18.9	4.68	10	1	1	High	1	1.031	1.076	0.631	0.77	0.69	0.731	0.691	0.65	0.569	0.78	0.581	0.722	0.869	0.873
TRINITY_DN9926_c1_g1_i1_orf1	rab GTPase-activating protein 1-like isoform X6 [Ostrinia furnacalis]	2	1176	131.1	5.76	2	2	2	High	1	1.043	1.091	1.499	1.464	1.339	1.633	1.707	1.387	0.945	0.989	1.09	1.479	1.411	1.362
TRINITY_DN2943_c2_g2_i1_orf1	protein phosphatase inhibitor 2-like [Ostrinia furnacalis]	1	164	18.8	4.88	9	1	1	High	1	0.773	0.948	1.927	1.888	1.899	1.727	2.073	1.729	1.725	1.581	2.145	1.919	1.853	1.912
TRINITY_DN65974_c0_g1_i2_orf1	uncharacterized protein LOC114362364 [Ostrinia furnacalis]	2	117	13	8.19	26	2	2	High	1	0.989	1.033	0.919	0.935	0.806	1.909	1.684	1.987	1.592	1.43	1.638	1.184	1.005	1.213
TRINITY_DN30012_c1_g1_i1_orf1	ubiquitin domain-containing protein UBFD1-like [Ostrinia furnacalis]	2	292	32.3	6.44	6	3	2	High	1	0.927	0.993	1.53	1.439	1.376	1.542	1.565	1.592	1.344	1.289	1.632	1.146	1.083	1.142
TRINITY_DN18558_c0_g1_i7_orf1	cytosolic Fe-S cluster assembly factor NUBP2 homolog [Ostrinia furnacalis]	2	263	28.4	5.47	7	2	2	High	1	1.04	0.966	0.778	0.738	0.744	0.793	0.751	0.782	0.779	0.626	0.851	0.829	0.935	0.818
TRINITY_DN1133_c0_g1_i6_orf1	zinc finger protein 391-like [Ostrinia furnacalis]	2	539	59.6	8.78	5	2	2	High	1	1	0.956	0.699	0.948	0.838	0.787	0.979	0.835	0.766	0.864	0.717	1.241	1.179	1.349
TRINITY_DN45449_c0_g1_i1_orf1	ATP-dependent helicase brm [Ostrinia furnacalis]	3	146	15.3	8	20	3	3	High	1	0.99	0.991	0.47	0.466	0.483	0.515	0.6	0.914	0.411	0.428	0.438	0.738	0.707	0.698
TRINITY_DN18909_c0_g1_i8_orf1	unnamed protein product [Euphydryas editha]	2	537	60.6	4.93	4	2	1	High	1	1.029	0.894	0.291	0.251	0.278	0.432	0.398	0.356	0.267	0.376	0.318	0.286	0.309	0.299
TRINITY_DN36410_c0_g1_i4_orf1	cytochrome P450 6a2-like [Ostrinia furnacalis]	2	496	56.9	8.98	5	2	2	High	1	0.981	0.991	1.034	1.311	1.222	1.149	1.123	1.139	1.048	1.202	1.107	1.09	0.989	1.123
TRINITY_DN8780_c0_g1_i3_orf1	uncharacterized protein LOC114363370 [Ostrinia furnacalis]	2	116	12.2	8.66	32	2	2	High	1	1.004	1.019	1.548	1.774	1.549	2.01	1.684	2.02	2.302	2.12	2.517	1.69	1.806	1.684
TRINITY_DN2962_c0_g1_i7_orf1	glucose transporter type 1-like isoform X1 [Ostrinia furnacalis]	2	480	51.8	7.91	4	2	2	High	1	0.907	0.982	1.225	1.057	1.2	1.02	0.946	0.954	0.863	1.077	0.96	1.013	0.96	0.993
TRINITY_DN3166_c1_g1_i6_orf1	hypothetical protein evm_013813 [Chilo suppressalis]	1	94	10.5	8.24	13	3	1	High	1	0.958	1.014	4.917	4.751	4.975	9.892	9.487	9.167	5.488	4.497	6.112	2.99	2.731	3.083
TRINITY_DN4255_c0_g1_i11_orf1	LOW QUALITY PROTEIN: lebocin-4-like [Ostrinia furnacalis]	2	198	22.2	10.04	13	2	1	High	1	1.03	1.084	3.355	3.274	3.365	3.493	3.27	3.198	2.711	2.676	3.2	5.024	5.162	4.67
TRINITY_DN5256_c0_g1_i1_orf1	uncharacterized protein LOC114359599 [Ostrinia furnacalis]	2	93	9.7	11.44	23	2	1	High	1	1.105	1.125	0.997	0.827	0.876	1.023	1.094	1.053	1.341	0.982	1.273	1.281	1.254	1.271
TRINITY_DN6680_c0_g1_i1_orf1	hypothetical protein evm_009571 [Chilo suppressalis]	2	335	38.3	7.9	7	2	2	High	1	1.02	0.941	1.241	1.253	1.097	1.638	1.736	1.735	1.698	1.725	1.935	1.164	1.162	1.313
TRINITY_DN85490_c0_g2_i1_orf1	venom carboxylesterase-6-like [Ostrinia furnacalis]	2	209	22.7	5.2	10	4	1	High	1	0.902	0.797	0.512	0.581	0.645	0.641	0.73	0.632	0.695	0.769	0.596	0.643	0.555	0.675
TRINITY_DN69170_c0_g2_i1_orf1	stromal membrane-associated protein 1-like [Pectinophora gossypiella]	2	209	23.7	9.54	10	2	2	High	1	1.027	0.991	0.645	0.726	0.664	0.585	0.718	0.641	0.568	0.604	0.633	0.765	0.809	0.835
TRINITY_DN53427_c0_g1_i2_orf1	heparanase-like [Ostrinia furnacalis]	1	514	58.8	8.7	3	1	1	High	1	0.996	1.213	1.552	1.481	1.401	1.185	1.24	1.162	1.271	1.137	1.177	1.945	1.885	1.8
TRINITY_DN23941_c0_g1_i5_orf1	dystonin isoform X11 [Galleria mellonella]	2	99	11.2	5.58	18	2	2	High	1	1.021	0.929	1.024	1.185	1.085	1.015	1.028	1.022	1.022	0.849	0.867	0.668	0.683	0.661
TRINITY_DN103511_c0_g1_i4_orf1	probable salivary secreted peptide [Ostrinia furnacalis]	2	87	9.6	7.42	45	3	2	High	1	0.892	1.066	1.485	1.604	1.464	1.761	1.954	1.977	1.887	3.011	1.613	1.47	1.286	1.603
TRINITY_DN137_c0_g1_i1_orf1	60S ribosomal protein L8 [Cotesia glomerata]	4	257	28.1	10.78	9	4	3	High	1	0.979	0.972	0.638	0.708	0.605	0.485	0.494	0.496	0.441	0.419	0.476	0.464	0.528	0.493
TRINITY_DN11396_c0_g1_i1_orf1	uncharacterized protein LOC114352414 isoform X1 [Ostrinia furnacalis]	2	242	27.3	4.97	9	2	2	High	1	1.044	0.977	0.53	0.56	0.558	0.564	0.587	0.608	0.611	0.629	0.536	0.709	0.695	0.677
TRINITY_DN52244_c1_g1_i1_orf1	triokinase/FMN cyclase-like isoform X2 [Ostrinia furnacalis]	2	72	8.2	5.73	22	3	1	High	1	0.963	0.952	1.597	1.715	1.634	0.439	0.462	0.575	0.923	0.883	0.935	1.167	0.91	1.269
TRINITY_DN122423_c0_g4_i1_orf1	dynein heavy chain, cytoplasmic isoform X2 [Chelonus insularis]	2	866	97.5	6.38	3	2	2	High	1	1.077	1.024	0.911	1.013	1.076	1.047	1.073	1.129	0.946	0.881	0.929	0.996	0.939	0.907
TRINITY_DN36324_c0_g1_i12_orf1	motile sperm domain-containing protein 1-like [Ostrinia furnacalis]	1	213	24.1	7.47	7	1	1	High	1	1.063	1.065	1.094	1.255	1.109	1.049	1.235	1.22	0.958	0.923	0.961	1.295	1.276	1.26
TRINITY_DN5664_c0_g1_i1_orf1	CDGSH iron-sulfur domain-containing protein 3, mitochondrial-like [Ostrinia f	1	137	15.7	9.64	12	1	1	High	1	0.858	0.809	0.602	0.577	0.452	0.848	1.026	0.718	0.878	0.742	0.56	3.621	3.511	3.454
TRINITY_DN17208_c0_g1_i2_orf1	integrator complex subunit 11 [Ostrinia furnacalis]	3	596	67.5	7.75	5	3	3	High	1	1.01	0.957	0.634	0.751	0.689	0.635	0.565	0.869	0.525	0.606	0.654	0.758	0.637	0.644
TRINITY_DN2299_c0_g1_i3_orf1	DNA-directed RNA polymerase II subunit RPB1 [Ostrinia furnacalis]	2	1901	209.5	7.4	1	2	2	High	1	1.008	1.012	1.188	1.127	1.185	1.437	1.483	1.351	0.861	0.847	0.775	0.793	0.675	0.754
TRINITY_DN1066_c0_g1_i4_orf1	ribosome biogenesis protein WDR12 homolog [Ostrinia furnacalis]	3	420	46.5	5.74	6	3	3	High	1	0.973	0.995	0.495	0.512	0.528	0.511	0.444	0.462	0.445	0.452	0.453	0.498	0.54	0.486
TRINITY_DN1772_c1_g3_i1_orf1	protein Red isoform X1 [Ostrinia furnacalis]	1	509	57.2	6.34	3	2	1	High	1	0.966	1.111	0.746	0.912	0.761	0.658	0.647	0.748	0.636	0.642	0.623	0.689	0.763	0.731
TRINITY_DN82944_c0_g1_i4_orf1	senecionine N-oxygenase isoform X2 [Ostrinia furnacalis]	2	159	17.9	5.47	20	2	2	High	1	0.929	1.03	1.761	1.841	1.811	2.101	2.002	2.01	1.991	2.063	2.019	1.522	1.372	1.514
TRINITY_DN17212_c0_g1_i6_orf1	putative deoxyribonuclease TATDN1 [Ostrinia furnacalis]	2	305	34.4	6.54	7	2	2	High	1	1.06	0.962	0.641	0.546	0.724	0.731	0.646	0.63	0.671	0.66	0.628	0.799	0.842	0.692
TRINITY_DN36006_c0_g1_i5_orf1	pro-resilin-like [Ostrinia furnacalis]	1	107	11.5	4.72	14	2	1	High	1	0.996	1.013	0.65	0.735	0.774	0.801	0.864	0.746	0.454	0.608	0.692	0.892	0.894	0.922
TRINITY_DN7414_c0_g1_i1_orf1	uncharacterized protein LOC114357447 [Ostrinia furnacalis]	2	270	29.8	10.02	6	2	2	High	1	0.953	0.964	0.792	0.71	0.793	0.757	0.767	0.717	0.632	0.603	0.701	0.572	0.573	0.617
TRINITY_DN7964_c0_g1_i6_orfp1	TRINITY_DN7964_c0_g1_i6_m.23478 TRINITY_DN7964_c0_g1::TRINITY_DN7964	2	86	9	10.3	30	4	2	High	1	1.071	0.999	0.276	0.261	0.245	0.258	0.226	0.265	0.364	0.268	0.322	0.172	0.184	0.239
TRINITY_DN23740_c1_g1_i1_orf1	NADH dehydrogenase [ubiquinone] iron-sulfur protein 6, mitochondrial isofor	1	121	13.7	8.56	12	2	1	High	1	1.067	0.974	0.711	0.707	0.802	0.619	0.54	0.69	0.844	0.687	0.92	0.482	0.542	0.486
TRINITY_DN3865_c0_g1_i5_orf1	trypsin, alkaline B-like [Ostrinia furnacalis]	1	261	27.7	8.78	6	1	1	High	1	1.125	1.139	0.942	0.704	1.106	1.002	1.396	0.857	1.111	1.04	1.04	0.814	0.874	1.252
TRINITY_DN8754_c0_g1_i2_orf1	dnaJ homolog subfamily C member 11 [Ostrinia furnacalis]	2	575	65.1	7.96	3	2	2	High	1	1.064	1.013	0.385	0.428	0.455	0.425	0.424	0.486	0.43	0.44	0.439	0.382	0.468	0.438
TRINITY_DN112921_c0_g1_i1_orf1	2-oxoglutarate and iron-dependent oxygenase domain-containing protein 3-	1	314	35.7	8.84	3	2	1	High	1	0.95	1.142	0.978	1.038	1.205	0.955	1.188	1.043	0.959	1.26	1.026	1.139	1.144	1.106
TRINITY_DN34676_c1_g1_i3_orf1	tRNA (uracil-5-)-methyltransferase homolog A [Ostrinia furnacalis]	2	633	71.1	7.14	5	2	2	High	1	0.968	1.062	1.162	1.22	1.15	0.967	1.225	1.186	1.294	1.771	1.28	0.975	0.869	0.974
TRINITY_DN8953_c0_g1_i4_orf1	gonadotropin-releasing hormone receptor [Ostrinia furnacalis]	2	397	45	9.73	6	2	2	High	1	0.889	0.915	3.167	2.748	2.571	2.307	2.504	2.588	1.845	1.937	2.491	1.782	1.894	2.064
TRINITY_DN45097_c0_g1_i5_orf1	ras-related protein Rab-24-like [Ostrinia furnacalis]	2	204	23.2	7.3	9	2	2	High	1	0.889	0.963	1.96	1.957	1.896	1.825	1.649	1.699	1.795	1.619	1.889	1.814	1.998	1.963
TRINITY_DN51424_c0_g2_i1_orf1	ras suppressor protein 1 [Helicoverpa zea]	3	286	32.1	7.59	9	4	3	High	1	0.968	0.983	1.26	1.364	1.281	1.198	1.141	1.166	1.268	1.192	1.326	1.064	1.012	0.988
TRINITY_DN18592_c0_g1_i4_orf1	GATOR complex protein MIOS [Ostrinia furnacalis]	2	280	31.1	8.47	8	2	1	High	1	0.928	0.918	1.323	0.836	1.033	1.347	1.523	1.89	1.809	1.689	1.081	1.728	1.612	1.876
TRINITY_DN29448_c0_g1_i1_orf1	28S ribosomal protein S9, mitochondrial [Ostrinia furnacalis]	2	400	45.9	9.17	5	3	2	High	1	0.982	0.96	0.477	0.464	0.5	0.478	0.467	0.471	0.485	0.415	0.444	0.627	0.577	0.642
TRINITY_DN4571_c0_g1_i4_orf1	PREDICTED: nuclear factor NF-kappa-B p105 subunit [Microplitis demolitor]	1	149	17	7.4	9	1	1	High	1	1.171	1.193	0.879	0.774	0.75	1.135	0.865	1.195	0.781	0.567	0.728	1.753	1.779	1.364
TRINITY_DN49221_c0_g1_i1_orf1	Similar to ND-23: NADH dehydrogenase (ubiquinone) 23 kDa subunit (Drosop	1	211	24.2	6.13	9	1	1	High	1	1.056	1.025	1.145	1.073	1.098	0.822	0.764	0.807	0.873	0.974	0.764	0.902	0.966	1.041

TRINITY_DN40176_c0_g1_i1_orf1	transcription elongation factor 1 homolog [Plutella xylostella]	1	82	9.6	8.27	22	1	1	High	1	1.051	0.95	1.158	1.249	1.065	0.995	0.906	0.977	0.764	0.726	0.986	1.094	1.099	1.052
TRINITY_DN9991_c0_g1_i4_orf1	unnamed protein product [Parnassius apollo]	1	151	16.8	6.37	15	1	1	High	1	1.148	1.058	1.268	1.279	1.373	1.662	1.843	1.791	1.415	1.402	1.961	1.123	1.057	1.119
TRINITY_DN142442_c0_g1_i1_orf1	60S ribosomal protein L38 [Homo sapiens]	1	70	8.2	10.1	19	1	1	High	1	1.278	1.347	1.354	1.656	1.737	2.203	2.059	1.937	2.466	2.154	3.147	1.538	1.797	1.708
TRINITY_DN61135_c0_g1_i1_orf1	uncharacterized protein LOC114362571 [Ostrinia furnacalis]	2	78	9.1	5.25	26	2	2	High	1	0.767	0.875	1.112	1.195	1.088	1.243	1.298	1.097	1.184	1.603	1.263	1.41	1.382	1.489
TRINITY_DN44219_c0_g1_i1_orf1	mitochondrial import inner membrane translocase subunit TIM50-C-like [Ostri	1	386	44.9	7.81	3	1	1	High	1	1.188	1.106	0.435	0.379	0.432	0.416	0.216	0.377	0.4	0.221	0.364	0.538	0.512	0.465
TRINITY_DN22044_c0_g2_i1_orf1	derlin-1 [Ostrinia furnacalis]	1	252	28.9	8.54	6	2	1	High	1	1.029	0.945	1.185	1.344	1.349	1.321	1.319	1.175	1.001	0.992	1.195	0.796	0.873	0.921
TRINITY_DN51197_c0_g1_i3_orf1	microtubule-actin cross-linking factor 1 isoform X15 [Ostrinia furnacalis]	2	107	12	4.88	19	3	2	High	1	0.958	0.898	1.072	1.193	1.11	1.207	1.184	1.198	0.953	0.84	1.007	1.074	1.168	0.938
TRINITY_DN6945_c0_g1_i5_orf1	unconventional myosin-IXa-like isoform X3 [Ostrinia furnacalis]	2	662	74.4	8.88	2	2	2	High	1	0.975	0.936	0.881	1.042	0.917	0.968	0.93	1.092	0.991	0.842	0.883	0.996	1.026	0.95
TRINITY_DN6653_c0_g1_i1_orf1	NEDD8-activating enzyme E1 catalytic subunit [Ostrinia furnacalis]	1	450	50.1	6.46	4	1	1	High	1	0.945	1.032	1.064	1.085	1.066	1.191	0.839	0.952	0.932	0.927	0.862	0.825	0.823	0.768
TRINITY_DN1803_c0_g1_i3_orf1	translocator protein-like isoform X1 [Ostrinia furnacalis]	3	191	21.3	7.15	12	4	3	High	1	0.959	0.906	0.434	0.446	0.529	0.494	0.455	0.476	0.485	0.535	0.404	0.603	0.605	0.648
TRINITY_DN28018_c0_g6_i1_orf1	microtubule-associated protein futsch-like isoform X6 [Ostrinia furnacalis]	2	554	62.4	5.95	3	2	2	High	1	0.999	0.996	0.924	0.9	0.916	0.924	0.929	0.931	1.049	0.949	0.91	1.475	1.612	1.515
TRINITY_DN26985_c0_g1_i5_orf1	secretory phospholipase A2 receptor-like [Helicoverpa zea]	2	91	9.8	6.92	26	2	2	High	1	0.852	0.941	2.233	2.269	2.207	0.759	0.666	0.678	0.774	0.783	1.004	2.692	2.669	2.683
TRINITY_DN1368_c0_g1_i6_orf1	hypothetical protein SFRUCORN_009408 [Spodoptera frugiperda]	1	251	27.8	5.24	5	2	1	High	1	1.001	0.978	0.698	0.677	0.744	0.833	0.718	0.752	0.669	0.621	0.725	0.814	0.772	0.787
TRINITY_DN15743_c0_g1_i5_orf1	uncharacterized protein LOC114351683 isoform X8 [Ostrinia furnacalis]	1	115	12.9	4.94	17	1	1	High	1	1.014	0.959	0.894	0.776	0.858	0.813	0.625	0.89	0.687	0.926	0.827	0.934	0.952	0.812
TRINITY_DN84322_c0_g2_i1_orf1	alanyl-tRNA synthetase 1 [Homo sapiens]	2	111	12.3	5.03	14	5	1	High	1	0.996	0.968	0.58	0.632	0.601	0.496	0.52	0.496	0.439	0.405	0.422	0.515	0.566	0.569
TRINITY_DN25865_c0_g1_i2_orf1	pseudouridine-metabolizing bifunctional protein C1861.05 [Spodoptera litura]	2	707	75.7	8.06	3	2	2	High	1	0.939	0.766	0.871	0.784	0.896	0.908	0.858	0.905	0.876	1.059	0.974	0.745	0.843	0.833
TRINITY_DN667_c0_g1_i5_orf1	unnamed protein product [Arctia plantaginis]	2	514	58.3	6.61	5	2	2	High	1	1.014	1.072	1.057	0.932	0.996	1.027	1.071	1.021	0.728	0.863	0.85	1.321	1.272	1.16
TRINITY_DN22175_c0_g1_i1_orf1	low-density lipoprotein receptor-related protein 1B-like [Ostrinia furnacalis]	2	126	14.2	10.48	14	2	2	High	1	0.994	1.094	1.506	1.45	1.268	1.543	1.451	1.461	1.306	1.334	1.407	1.462	1.37	1.366
TRINITY_DN31585_c0_g1_i1_orf1	transcription elongation factor SPT5 [Ostrinia furnacalis]	2	824	91.7	6.87	3	2	2	High	1	0.968	1.011	0.647	0.65	0.656	0.65	0.567	0.721	0.604	0.573	0.676	0.807	0.798	0.783
TRINITY_DN12806_c0_g2_i1_orf1	inactive pancreatic lipase-related protein 1-like isoform X2 [Ostrinia furnacalis	1	332	37.1	8.22	6	1	1	High	1	1.021	0.996	0.87	0.709	0.877	0.719	0.651	0.657	0.656	0.52	0.528	0.622	0.603	0.537
TRINITY_DN1760_c0_g1_i4_orf1	uncharacterized protein LOC114357676 [Ostrinia furnacalis]	2	287	31	7.96	8	3	2	High	1	1.012	1.039	0.942	0.994	0.903	0.712	0.693	0.715	1.005	0.849	0.981	0.888	0.876	0.882
TRINITY_DN33867_c0_g1_i9_orf1	uncharacterized protein LOC114357513 [Ostrinia furnacalis]	2	136	14.4	6.52	15	3	2	High	1	1.074	1	0.733	0.707	0.77	0.649	0.673	0.696	0.844	0.765	0.778	0.653	0.728	0.62
TRINITY_DN10399_c0_g1_i2_orf1	unnamed protein product [Chilo suppressalis]	2	313	35.9	6.19	6	2	2	High	1	1.116	1.074	0.693	0.738	0.7	0.683	0.681	0.673	0.572	0.64	0.658	0.727	0.687	0.693
TRINITY_DN774_c0_g1_i9_orf1	sequestosome-1-like isoform X3 [Ostrinia furnacalis]	1	503	54.6	6.35	4	1	1	High	1	1.01	0.9	1.029	1.306	1.014	0.956	1.148	0.95	1.017	1.148	1.116	1.053	0.848	0.914
TRINITY_DN122423_c0_g5_i1_orf1	PREDICTED: dynein heavy chain, cytoplasmic isoform X3 [Fopius arisanus]	2	332	37.9	5.63	5	2	2	High	1	0.918	0.881	0.664	0.769	0.766	0.723	0.826	0.78	0.648	0.623	0.674	0.848	0.756	0.766
TRINITY_DN49872_c0_g2_i1_orf1	NIF3-like protein 1 [Ostrinia furnacalis]	2	298	32.5	7.85	5	2	2	High	1	0.943	1.001	0.733	0.686	0.659	0.749	0.778	0.857	0.58	0.52	0.581	0.826	0.736	0.718
TRINITY_DN9931_c0_g1_i1_orf1	syntaxin-18 [Ostrinia furnacalis]	2	323	37.3	5.12	5	2	2	High	1	1.007	0.958	0.702	0.627	0.7	0.726	0.691	0.709	0.638	0.552	0.574	0.736	0.677	0.73
TRINITY_DN2184_c0_g1_i4_orf1	uncharacterized protein LOC114359356 [Ostrinia furnacalis]	2	255	28	4.68	8	2	2	High	1	0.976	1.008	0.353	0.331	0.362	0.569	0.548	0.488	0.504	0.486	0.44	0.389	0.388	0.409
TRINITY_DN27398_c0_g1_i3_orf1	lissencephaly-1 homolog [Helicoverpa armigera]	1	411	46.2	7.4	4	1	1	High	1	1.131	0.934	1.126	0.965	0.809	0.774	0.716	0.573	0.67	0.661	0.802	1.074	0.986	0.912
TRINITY_DN8694_c1_g1_i4_orf1	sodium/potassium-transporting ATPase subunit beta-2-like isoform X2 [Ostrir	2	319	37.3	7.34	6	2	2	High	1	0.959	0.914	1.237	1.337	1.383	1.454	1.764	1.566	1.181	1.081	1.055	1.687	1.665	1.601
TRINITY_DN73230_c0_g1_i1_orf1	cysteine and histidine-rich domain-containing protein [Ostrinia furnacalis]	2	351	39	7.08	9	2	2	High	1	0.862	0.923	0.731	0.707	0.812	0.821	0.853	0.84	0.879	0.932	0.808	0.931	0.832	0.848
TRINITY_DN527_c0_g1_i5_orf1	uncharacterized protein LOC114366638 [Ostrinia furnacalis]	2	285	32.2	9.5	13	2	2	High	1	1.017	0.949	0.935	0.992	0.829	0.771	0.825	0.966	0.917	0.855	0.897	1.034	0.985	0.961
TRINITY_DN139212_c0_g1_i4_orf1	uncharacterized protein LOC114350112 [Ostrinia furnacalis]	2	254	27.3	8.12	8	2	2	High	1	1.072	1.007	0.388	0.405	0.448	0.273	0.235	0.191	0.227	0.216	0.226	0.671	0.629	0.539
TRINITY_DN12293_c0_g1_i1_orf1	hypothetical protein evm_011848 [Chilo suppressalis]	3	210	23.9	7.97	12	3	3	High	1	1.057	1.085	0.795	0.862	0.772	1.009	1.059	0.983	0.744	0.754	0.705	0.645	0.744	0.69
TRINITY_DN5919_c0_g1_i4_orf1	esterase FE4-like [Ostrinia furnacalis]	2	571	63.7	6.46	5	2	2	High	1	1	0.977	0.562	0.63	0.55	0.598	0.623	0.654	0.538	0.558	0.54	0.605	0.498	0.624
TRINITY_DN27300_c0_g1_i1_orfp1	TRINITY_DN27300_c0_g1_i1_m.71142 TRINITY_DN27300_c0_g1::TRINITY_DN27	2	81	9.2	9.66	21	4	1	High	1	0.998	1.164	11.271	11.811	11.118	2.949	3.043	3.496	3.742	3.053	3.418	1.448	1.132	1.425
TRINITY_DN14301_c0_g2_i1_orf1	unnamed protein product [Chrysodeixis includens]	2	179	19.5	9.07	11	2	2	High	1	0.995	1.086	0.47	0.466	0.448	0.581	0.592	0.536	0.544	0.517	0.54	0.532	0.543	0.534
TRINITY_DN39904_c0_g1_i1_orf1	Keratin 8 [Mus musculus]	2	439	49.2	5.17	4	3	1	High	1	0.953	2.125	0.398	1.434	0.508	1.16	0.801	1.843	2.194	1.663	0.67	1.992	1.172	0.255
TRINITY_DN31303_c0_g1_i4_orf1	tRNA pseudouridine synthase A isoform X1 [Ostrinia furnacalis]	2	445	51.7	6.57	6	2	2	High	1	1.029	0.972	0.649	0.753	0.677	0.634	0.652	0.629	0.711	0.684	0.586	0.752	0.708	0.66
TRINITY_DN9412_c0_g1_i1_orf1	maspardin-like [Ostrinia furnacalis]	1	321	36.1	6.4	5	1	1	High	1	0.985	1.052	1.345	1.374	1.251	1.089	1.204	1.199	1.392	1.517	1.511	1.958	1.775	1.752
TRINITY_DN48983_c0_g1_i2_orf1	E3 ubiquitin-protein ligase Su(dx) [Ostrinia furnacalis]	1	883	100.6	7.17	1	1	1	High	1	1.055	1.012	0.661	0.779	0.789	0.671	0.858	1.009	0.652	0.642	0.522	1	0.775	0.838
TRINITY_DN4133_c0_g1_i2_orfp2	unnamed protein product [Spodoptera exigua]	2	62	6.9	8.66	40	2	2	High	1	0.995	0.928	1.167	1.22	1.094	1.088	1.124	0.959	0.504	0.594	0.671	0.59	0.516	0.512
TRINITY_DN26089_c0_g1_i1_orf1	putative neuropeptide precursor protein isoform X1 [Ostrinia furnacalis]	2	381	43	8.03	5	2	2	High	1	1.11	1.108	1.046	1.286	0.91	1.501	1.531	1.41	1.072	0.993	1.213	1.363	1.464	1.402
TRINITY_DN70409_c0_g1_i3_orf1	tyrosine-protein phosphatase non-receptor type 9 isoform X3 [Ostrinia furnac	1	368	40.5	6.6	4	1	1	High	1	1.022	1.173	0.911	1.066	0.936	0.836	0.888	0.884	0.803	0.711	0.862	0.927	0.965	1.06
TRINITY_DN15478_c0_g1_i1_orf1	STE20/SPS1-related proline-alanine-rich protein kinase [Vanessa tameamea]	1	491	54.1	5.5	3	1	1	High	1	1.051	1.07	1.246	1.579	1.302	1.558	1.582	2.053	1.747	1.925	1.768	1.452	1.226	1.508
TRINITY_DN9510_c0_g2_i1_orf1	RNA polymerase II transcriptional coactivator [Ostrinia furnacalis]	3	106	12.1	8.9	29	3	3	High	1	1.026	0.98	1.079	1.034	1.102	0.777	0.803	0.856	0.848	0.72	0.789	0.851	0.838	0.853
TRINITY_DN64719_c0_g1_i2_orfp1	TRINITY_DN64719_c0_g1_i2_m.37745 TRINITY_DN64719_c0_g1::TRINITY_DN64	1	90	8.4	10.4	14	2	1	High	1	1.031	1.121	4.16	3.927	3.475	2.176	1.818	2.053	1.832	1.553	1.956	0.866	0.641	0.933
TRINITY_DN8136_c0_g1_i1_orf1	HIG1 domain family member 2A, mitochondrial [Ostrinia furnacalis]	1	98	10.9	10.26	14	2	1	High	1	0.966	0.984	1.136	1.097	1.131	0.775	0.804	0.746	0.965	0.779	0.913	1.028	0.918	0.897
TRINITY_DN8027_c0_g1_i3_orf1	protein MON2 homolog isoform X3 [Ostrinia furnacalis]	1	862	94.1	7.2	1	2	1	High	1	0.934	0.944	0.914	0.935	0.898	0.91	1.023	0.977	0.884	1.083	0.877	1.017	1.077	0.988
TRINITY_DN536_c0_g1_i7_orf1	polymerase delta-interacting protein 2 isoform X3 [Ostrinia furnacalis]	2	344	39.3	8.05	12	2	2	High	1	0.973	0.917	0.546	0.496	0.467	0.372	0.499	0.503	0.454	0.468	0.511	0.437	0.414	0.435
TRINITY_DN2084_c0_g1_i1_orf1	40S ribosomal protein S24 [Helicoverpa armigera]	2	132	15.2	10.9	13	2	2	High	1	1.026	0.984	0.498	0.542	0.495	0.494	0.603	0.566	0.495	0.518	0.522	0.468	0.451	0.467
TRINITY_DN4076_c1_g2_i2_orf1	vacuole membrane protein 1 [Ostrinia furnacalis]	1	398	44.2	9.55	4	1	1	High	1	0.904	0.885	1.454	1.801	1.706	2.019	2.43	1.881	2.335	2.265	2.136	2.033	2.004	2.398
TRINITY_DN3647_c2_g1_i3_orf1	unnamed protein product, partial [Iphiclides podalirius]	1	89	9.5	9.45	13	1	1	High	1	0.958	0.977	0.447	0.394	0.503	0.515	0.49	0.512	0.382	0.354	0.45	0.689	0.737	0.826
TRINITY_DN1480_c0_g1_i5_orf1	carboxypeptidase N subunit 2-like [Ostrinia furnacalis]	2	744	83.3	5.24	3	2	2	High	1	0.98	1.139	1.09	1.297	1.159	1.165	1.571	1.25	1.175	0.994	1.305	1.69	1.661	1.611
TRINITY_DN3545_c0_g1_i6_orf1	group XV phospholipase A2-like [Ostrinia furnacalis]	2	409	45.6	8.13	6	2	2	High	1	0.976	0.902	1.417	1.515	1.24	1.25	1.472	1.367	1.224	1.484	1.215	1.749	1.678	1.731
TRINITY_DN17061_c0_g1_i1_orf1	uncharacterized protein LOC113511282 isoform X2 [Galleria mellonella]	1	241	26.8	9.63	9	1	1	High	1	1.014	0.989	1.178	1.262	1.175	1.147	1.204	1.311	1.085	1.06	1.193	0.851	0.722	0.873
TRINITY_DN2406_c0_g1_i6_orf1	uncharacterized protein LOC114361672 [Ostrinia furnacalis]	2	526	61.1	4.41	4	2	2	High	1	1.098	1.274	3.42	3.919	3.378	1.626	1.802	1.714	2.065	1.82	2.144	2.597	2.278	2.239
TRINITY_DN37986_c0_g1_i2_orf1	unnamed protein product [Diatraea saccharalis]	2	318	34.8	10.14	7	2	2	High	1	1.033	1.034	0.893	0.871	0.944	0.857	0.798	0.796	0.757	0.681	0.739	0.71	0.786	0.629
TRINITY_DN14507_c0_g1_i5_orf1	PTB domain-containing adapter protein ced-6 [Ostrinia furnacalis]	1	519	57	7.5	3	1	1	High	1	1.127	1.061	0.839	0.709	0.658	0.544	0.305	0.35	0.432	0.576	0.346	1.397	1.49	0.796
TRINITY_DN3450_c0_g1_i3_orf1	hypothetical protein evm_008214 [Chilo suppressalis]	2	812	90.2	5.15	3	2	2	High	1	1	1.055	0.713	0.671	0.679	0.717	0.754	0.832	0.559	0.587	0.633	0.816	0.853	0.83
TRINITY_DN39200_c0_g1_i5_orf1	juvenile hormone epoxide hydrolase-like [Ostrinia furnacalis]	1	497	56.8	8.57	4	1	1	High	1	0.962	0.997	0.08	0.044	0.127	0.053	0.081	0.1	0.086	0.093	0.083	0.105	0.087	0.093
TRINITY_DN3675_c0_g1_i1_orf1	unnamed protein product [Spodoptera exigua]	2	541	59.9	8.48	5	2	2	High	1	0.945	0.967	1.13	1.22	1.278	1.075	1.15	1.217	1.064	1.374	1.1	1.455	1.645	1.751
TRINITY_DN5349_c0_g1_i1_orf1	glutamate-rich WD repeat-containing protein 1 [Galleria mellonella]	2	447	50	4.6	6	3	2	High	1	0.975	1.02	0.595	0.582	0.608	0.484	0.604	0.574	0.548	0.551	0.519	0.487	0.563	0.551
TRINITY_DN117362_c0_g1_i5_orf1	uncharacterized protein LOC114354191 [Ostrinia furnacalis]	1	442	48.5	4.58	5	1	1	High	1	0.901	0.917	0.447	0.517	0.41	0.57	0.431	0.713	0.503	0.832	0.425	0.413	0.375	0.496
TRINITY_DN21494_c0_g1_i2_orf1	pancreatic triacylglycerol lipase-like [Ostrinia furnacalis]	1	330	35.8	6.9	4	1	1	High	1	1.055	0.965	0.465	0.279	0.431	0.352	0.364	0.363	0.354	0.372	0.308	0.459	0.418	0.375
TRINITY_DN6023_c0_g1_i7_orf1	exocyst complex component 7 [Ostrinia furnacalis]	2	685	75.5	7.62	4	2	2	High	1	1.038	1.106	1.079	1.104	1.058	1.206	1.15	1.247	1.157	0.99	1.071	0.989	1.049	1.064
TRINITY_DN210_c0_g1_i9_orf1	glycine-rich cell wall structural protein-like [Ostrinia furnacalis]	2	152	15.2	9.55	14	3	2	High	1	1.173	1.015	1.142	1.256	1.205	1.083	1.054	0.981	0.957	0.829	0.816	1.25	1.234	1.327
TRINITY_DN7316_c0_g2_i1_orf1	ubiquitin-protein ligase E3C [Ostrinia furnacalis]	3	642	73	7.17	5	3	3	High	1	1.087	1.276	1.406	1.416	1.383	1.033	1.198	1.143	0.98	1.112	1.107	1.232	1.485	1.545
TRINITY_DN115498_c0_g1_i1_orf1	fatty acid synthase-like [Ostrinia furnacalis]	2	91	10.1	8.5	24	3	2	High	1	1.057	1.081	0.537	0.567	0.674	0.75	0.625	0.597	0.529	0.675	0.604	0.542	0.538	0.552
TRINITY_DN6058_c0_g1_i3_orf1	NHL repeat-containing protein 2 isoform X2 [Ostrinia furnacalis]	2	754	83.7	6.34	4	2	2	High	1	0.991	1.031	1.301	1.283	1.396	1.278	1.448	1.297	1.272	1.292	1.307	1.108	1.15	1.175
TRINITY_DN230_c1_g1_i3_orf1	protein artichoke-like [Ostrinia furnacalis]	2	1144	129.4	5.59	2	2	2	High	1	1.059	1.168	1.624	1.69	1.689	1.225	1.536	1.273	1.313	1.333	1.375	1.34	1.397	1.388
TRINITY_DN467_c4_g1_i2_orf1	GRIP and coiled-coil domain-containing protein 1 [Ostrinia furnacalis]	2	625	72.1	6.35	3	2	2	High	1	0.957	0.942	0.606	0.574	0.62	0.65	0.657	1.111	0.677	0.563	0.733	1.128	1.192	1.251
TRINITY_DN40058_c0_g2_i1_orf1	host cell factor [Chelonus insularis]	1	1492	157.7	5.24	1	2	1	High	1	0.902	0.983	0.917	0.898	1.068	0.844	1.021	0.89	0.977	1.043	0.951	1.01	0.933	1.039
TRINITY_DN4695_c0_g1_i3_orf1	glutathione S-transferase epsilon 3 [Ostrinia furnacalis]	2	218	24.8	6.87	9	2	2	High	1	0.991	1.061	1.236	1.163	1.179	2.436	1.999	2.193	2.505	2.235	2.578	0.574	0.535	0.528
TRINITY_DN436_c0_g2_i5_orfp1	TRINITY_DN436_c0_g2_i5_m.4776 TRINITY_DN436_c0_g2::TRINITY_DN436_c0_	2	150	15.7	11.53	18	3	2	High	1	1.22	1.027	0.985	0.966	1.116	1.478	1.486	1.4	1.044	1.022	1.081	0.896	0.918	0.923
TRINITY_DN27332_c0_g2_i1_orf1	Alpha-D-phosphohexomutase alpha/beta/alpha domain III, partial [Trinorches	2	63	7	5.17	43	2	2	High	1	1.124	1.113	1.133	1.097	1.176	1.078	0.971	1.122	1.076	0.896	1.1	0.909	0.951	0.874
TRINITY_DN6122_c0_g1_i6_orf1	deubiquitinase DESI2 isoform X1 [Helicoverpa armigera]	1	211	23.8	8.32	8	1	1	High	1	1.182	1.188	2.744	2.333	3.099	3.579	3.35	3.171	4.963	4.129	3.911	3.819	4.518	4.112
TRINITY_DN4085_c0_g1_i1_orf1	Protein TSSC1 [Papilio machaon]	1	373	41.9	5.69	3	1	1	High	1	0.858	0.703	0.728	0.666	0.661	0.542	0.654	0.585	0.602	0.629	0.502	0.69	0.946	1.027
TRINITY_DN18035_c0_g1_i7_orf1	mitochondrial ornithine transporter 1 [Ostrinia furnacalis]	2	219	24.3	9.16	11	2	2	High	1	1.073	1.146	0.793	0.856	0.763	0.636	0.782	0.888	0.791	0.934	0.717	0.837	0.704	0.716
TRINITY_DN3418_c0_g1_i3_orf1	proto-oncogene tyrosine-protein kinase ROS [Ostrinia furnacalis]	2	1383	155.2	8.09	2	2	2	High	1	0.903	0.982	1.176	1.262	1.057	1.239	1.275	1.391	1.287	1.388	1.257	1.295	1.324	1.296
TRINITY_DN195_c4_g1_i1_orf1	beta-1,3-glucan-binding protein 1 [Ostrinia furnacalis]	1	122	14.1	5.27	19	1	1	High	1	0.845	0.86	1.212	1.031	0.838	0.258	0.369	0.314	0.372	0.26	0.289	0.335	0.292	0.272
TRINITY_DN19669_c0_g1_i1_orf1	uncharacterized protein LOC114350331 [Ostrinia furnacalis]	1	132	14.5	7.72	8	1	1	High	1	0.995	0.918	1.153	1.262	1.122	1.05	1.272	1.017	0.932	0.881	1.237	1.029	1.045	1.203
TRINITY_DN1109_c0_g1_i6_orf1	1-phosphatidylinositol phosphodiesterase-like [Cotesia glomerata]	2	301	34.7	8.68	8	2	2	High	1	0.979	1.003	0.32	0.278	0.394	0.349	0.441	0.433	0.433	0.59	0.383	0.579	0.606	0.61
TRINITY_DN33867_c0_g1_i8_orf1	uncharacterized protein LOC114357513 [Ostrinia furnacalis]	1	359	37.8	5.54	5	1	1	High	1	1.033	1.036	1.247	1.346	1.178	1.04	1.044	1.089	1.324	1.207	1.383	1.189	1.216	1.301
TRINITY_DN3059_c0_g1_i1_orf1	isopentenyl-diphosphate Delta-isomerase 1 [Ostrinia furnacalis]	2	253	29.7	6.68	9	2	2	High	1	1.008	0.971	0.924	0.928	1.029	1.003	0.895	0.907	0.919	1.258	0.889	0.937	0.997	1.296
TRINITY_DN45948_c1_g1_i1_orf1	unnamed protein product [Leptidea sinapis]	2	34	3.7	8.18	68	3	2	High	1	1.032	1.079	3.749	3.39	3.625	3.07	2.802	3.234	2.471	2.206	2.543	4.77	5.53	4.727
TRINITY_DN31119_c0_g1_i1_orf1	transforming acidic coiled-coil-containing protein 3-like [Ostrinia furnacalis]	2	319	36	5.94	6	2	2	High	1	0.975	0.986	0.748	0.754	0.81	0.714	0.82	0.798	0.6	0.581	0.658	0.811	0.813	0.789
TRINITY_DN4589_c0_g1_i1_orf1	protein penguin [Ostrinia furnacalis]	2	630	70.9	9.44	3	2	2	High	1	1.059	1.035	0.512	0.511	0.629	0.544	0.505	0.566	0.514	0.543	0.403	0.513	0.478	0.522
TRINITY_DN5834_c0_g1_i2_orf1	CD2 antigen cytoplasmic tail-binding protein 2 homolog [Ostrinia furnacalis]	2	332	38.3	4.84	6	2	2	High	1	1.156	1.039	0.744	0.745	0.736	0.722	0.934	0.797	0.748	0.766	0.815	0.956	0.945	1.019
TRINITY_DN2770_c0_g2_i4_orf1	phosphatidylinositol 4-phosphate 3-kinase C2 domain-containing subunit alp	2	1800	200.6	6.54	1	2	2	High	1	0.868	0.896	1.131	1.162	0.985	0.928	1.044	0.858	0.726	0.743	0.747	0.836	0.869	0.867
TRINITY_DN761_c0_g1_i3_orf1	nudix hydrolase 8-like [Ostrinia furnacalis]	2	259	29.8	6.33	7	2	2	High	1	1.062	1.134	0.937	0.824	0.949	0.993	1.18	1.015	0.988	1.058	0.807	0.995	0.934	0.887
TRINITY_DN38608_c0_g1_i1_orf1	LOW QUALITY PROTEIN: protein hook [Manduca sexta]	2	90	10.3	5.99	23	2	2	High	1	0.889	0.893	0.879	0.982	0.86	0.951	1.035	1.221	0.866	0.98	0.97	0.933	1.023	0.998
TRINITY_DN89483_c0_g1_i1_orf1	mitochondrial enolase superfamily member 1-like isoform X2 [Maniola jurtina]	2	73	8.3	9.09	21	4	2	High	1	1.013	0.993	1.514	1.371	1.515	1.991	1.73	2.038	1.483	1.314	1.284	1.087	1.07	1.03
TRINITY_DN15904_c0_g1_i1_orf1	papilin isoform X8 [Ostrinia furnacalis]	1	91	9.9	4.31	31	1	1	High	1	1.158	1.092	0.728	0.932	0.859	0.932	0.884	0.819	0.832	0.558	0.744	0.941	0.982	0.972

TRINITY_DN15870_c0_g1_i3_orf1	PREDICTED: mitochondrial import inner membrane translocase subunit Tim23	1	208	22.1	9.32	5	1	1	High	1	0.998	0.943	0.314	0.216	0.292	0.237	0.252	0.321	0.248	0.283	0.264	0.321	0.287	0.343
TRINITY_DN53400_c0_g1_i1_orf1	hypothetical protein evm_004547 [Chilo suppressalis]	2	607	66.9	7.8	4	2	2	High	1	0.958	0.972	0.775	0.603	0.755	0.728	0.828	0.764	0.732	0.828	0.733	0.757	0.71	0.854
TRINITY_DN27264_c0_g1_i1_orf1	uncharacterized protein LOC114353424 [Ostrinia furnacalis]	2	257	29.3	8.95	6	2	2	High	1	0.916	0.903	1.067	0.96	1.232	1.216	1.034	1.157	2.271	1.958	2.255	4.421	4.857	4.142
TRINITY_DN1781_c0_g1_i8_orf1	transportin-1 [Pectinophora gossypiella]	2	902	101.4	5.35	2	2	2	High	1	0.941	0.969	0.991	0.947	0.935	0.72	0.721	0.745	0.748	0.816	0.738	1.042	1.074	1.069
TRINITY_DN71308_c0_g1_i4_orf1	uncharacterized protein LOC114361536 [Ostrinia furnacalis]	2	84	9.4	8.19	25	2	1	High	1	1.323	1.213	2.364	2.189	2.007	2.29	1.872	2.329	2.387	2.719	1.653	2.838	2.858	2.879
TRINITY_DN54586_c1_g1_i1_orf1	protein YIPF5 [Ostrinia furnacalis]	1	264	28.8	4.34	5	1	1	High	1	0.959	1.015	0.932	1.041	0.981	0.793	0.738	0.727	0.725	0.609	0.846	1.127	1.155	1.004
TRINITY_DN3057_c0_g2_i1_orf1	chromodomain-helicase-DNA-binding protein Mi-2 homolog isoform X3 [Ch	2	1973	222.7	5.72	1	2	2	High	1	1.007	0.997	0.616	0.646	0.517	0.619	0.516	0.749	0.587	0.517	0.662	0.772	0.764	0.815
TRINITY_DN8553_c0_g1_i4_orf1	coiled-coil-helix-coiled-coil-helix domain-containing protein 7 isoform X2 [O	2	86	10.4	8.87	27	2	2	High	1	1.082	1.068	0.874	0.803	0.804	0.838	0.912	0.799	0.915	0.703	0.813	1.001	1.088	0.96
TRINITY_DN11327_c0_g1_i1_orf1	PREDICTED: trithorax group protein osa-like [Papilio xuthus]	2	198	21.4	4.91	12	2	2	High	1	1.042	1.019	1.124	1.162	1.122	1.192	1.536	1.425	1.168	1.262	1.105	1.098	1.191	1.138
TRINITY_DN101544_c0_g1_i1_orf1	unnamed protein product [Diatraea saccharalis]	2	670	77.4	8.07	3	2	2	High	1	0.861	0.845	0.806	0.867	0.987	0.838	0.945	0.925	0.917	0.861	0.863	0.948	0.879	0.931
TRINITY_DN96170_c0_g1_i1_orf1	uncharacterized protein LOC114355569 [Ostrinia furnacalis]	3	802	87.6	5.99	4	3	3	High	1	0.941	1.076	0.369	0.346	0.43	0.459	0.367	0.434	0.328	0.335	0.443	0.381	0.347	0.352
TRINITY_DN14046_c0_g1_i1_orf1	retinol dehydrogenase 14 [Ostrinia furnacalis]	2	338	37.5	8.85	7	2	2	High	1	0.855	0.91	0.619	0.709	0.675	0.659	0.63	0.649	0.581	0.832	0.622	1.255	1.187	1.275
TRINITY_DN3133_c0_g1_i6_orf1	max-like protein X [Ostrinia furnacalis]	2	209	24.2	6.8	11	2	2	High	1	1.107	1.182	0.997	0.786	0.834	0.904	1.071	1.045	1.042	1.267	1.025	0.726	0.912	1.002
TRINITY_DN68770_c0_g1_i1_orf1	seroin transcript 1A2 [Ostrinia nubilalis]	2	81	8.7	9.39	30	2	1	High	1	1.167	1.269	1.448	1.221	1.496	1.749	1.622	1.987	2.898	2.468	2.352	1.039	0.967	0.848
TRINITY_DN141352_c0_g1_i1_orf1	carboxy-terminal domain RNA polymerase II polypeptide A small phosphatase	2	293	32.2	6.74	7	2	2	High	1	0.983	0.924	0.502	0.577	0.597	0.602	0.62	0.658	0.508	0.67	0.546	0.624	0.585	0.607
TRINITY_DN4385_c0_g2_i1_orf1	LOW QUALITY PROTEIN: carbonic anhydrase 1-like [Ostrinia furnacalis]	2	265	29.4	5.96	6	2	2	High	1	1.05	0.971	0.776	0.905	0.825	0.729	0.779	0.729	0.574	0.68	0.7	0.636	0.566	0.497
TRINITY_DN5692_c0_g1_i4_orf1	E3 ubiquitin-protein ligase MARCH6 [Ostrinia furnacalis]	2	861	95.5	7.74	5	2	2	High	1	1.149	1.303	1.12	1.388	1.207	1.303	1.459	1.295	1.296	1.242	1.237	1.084	1.1	1.048
TRINITY_DN4300_c0_g1_i5_orf1	replication factor C subunit 3 [Ostrinia furnacalis]	2	355	40.6	8.92	8	2	2	High	1	0.972	1.089	0.778	0.777	0.642	0.692	0.718	0.767	0.8	0.659	0.653	0.764	0.782	0.695
TRINITY_DN9282_c0_g1_i2_orf1	uncharacterized protein LOC114363102 isoform X2 [Ostrinia furnacalis]	1	147	15.1	4.5	10	1	1	High	1	0.816	0.933	0.619	0.624	0.552	0.592	0.521	0.69	0.484	0.419	0.454	5.158	5.07	5.268
TRINITY_DN802_c0_g1_i2_orf1	active breakpoint cluster region-related protein [Ostrinia furnacalis]	2	1267	138.9	6.74	1	2	2	High	1	1.06	1.195	0.791	0.697	0.785	0.673	0.542	0.654	0.751	0.589	0.63	1.056	1.067	0.989
TRINITY_DN700_c0_g1_i3_orf1	V-type proton ATPase subunit H isoform X1 [Chelonus insularis]	2	518	59.4	6.06	3	2	1	High	1	1.029	1.02	0.211	0.14	0.257	0.24	0.204	0.245	0.257	0.266	0.225	0.251	0.271	0.262
TRINITY_DN1039_c0_g1_i5_orf1	WD repeat-containing protein 26 [Ostrinia furnacalis]	2	344	38.5	6.74	7	2	2	High	1	0.964	1.011	0.843	0.954	1.065	1.055	1.214	1.228	0.941	0.911	0.962	0.926	0.903	0.998
TRINITY_DN4886_c0_g1_i6_orf1	uncharacterized protein LOC114349567 [Ostrinia furnacalis]	2	314	35.6	5.67	6	2	2	High	1	0.924	1.02	1.222	1.373	1.039	1.123	1.107	1.27	1.32	1.079	1.383	1.871	1.83	1.862
TRINITY_DN13686_c0_g2_i1_orf1	transmembrane protease serine 9-like [Ostrinia furnacalis]	2	397	43.9	7.11	6	2	2	High	1	0.844	1.013	1.357	1.357	1.496	1.948	1.738	1.577	1.639	1.606	1.459	4.372	4.394	4.249
TRINITY_DN3459_c0_g1_i4_orf1	PREDICTED: probable small nuclear ribonucleoprotein G [Papilio polytes]	2	76	8.5	8.84	26	2	2	High	1	0.936	1.011	0.908	1.02	0.89	0.911	1.072	1.052	1.151	1.136	1.107	1.363	1.243	1.354
TRINITY_DN40439_c0_g1_i5_orf1	ommochrome binding protein 1, partial [Ostrinia nubilalis]	1	137	15.4	7.33	8	2	1	High	1	1.07	1.137	2.1	1.856	2.147	2.071	1.985	2.19	2.962	3.152	2.023	2.186	2.086	2.478
TRINITY_DN14967_c0_g2_i1_orf1	glyceraldehyde-3-phosphate dehydrogenase 2 [Holotrichia oblita]	2	332	35.5	7.43	6	2	2	High	1	0.982	1.048	0.272	0.293	0.408	0.355	0.335	0.386	0.389	0.386	0.338	0.355	0.347	0.333
TRINITY_DN53167_c0_g1_i2_orf1	uncharacterized protein LOC114359219 [Ostrinia furnacalis]	2	521	59.8	5.21	5	2	2	High	1	0.984	0.965	0.41	0.353	0.427	0.394	0.459	0.385	0.506	0.458	0.384	0.454	0.465	0.44
TRINITY_DN27723_c0_g1_i2_orf1	putative uncharacterized protein DDB_G0282133 isoform X1 [Ostrinia furnacali	2	156	17.7	4.73	15	2	1	High	1	1.178	1.017	0.779	0.698	0.787	0.775	0.737	0.999	0.736	0.864	0.824	0.836	0.751	0.77
TRINITY_DN2968_c0_g1_i3_orf1	uncharacterized protein LOC114357587 [Ostrinia furnacalis]	2	390	45	6.04	6	2	2	High	1	1.084	1.032	1.005	0.988	1.012	1.146	1.076	1.208	0.997	1.052	1.067	1.228	1.263	1.311
TRINITY_DN19262_c0_g1_i1_orf1	tRNA (guanine-N(7)-)-methyltransferase non-catalytic subunit wdr4 [Ostrinia f	1	375	42.3	6.54	5	1	1	High	1	1.068	1.054	0.813	0.707	0.852	0.898	0.786	0.822	0.739	0.684	0.835	0.674	0.772	0.743
TRINITY_DN58125_c0_g1_i1_orf1	protein yellow-like [Ostrinia furnacalis]	1	173	19.2	5.05	9	1	1	High	1	0.98	1.074	1.124	1.523	1.135	1.16	1.423	1.102	0.993	1.128	1.188	3.532	3.371	3.219
TRINITY_DN12671_c0_g1_i6_orf1	hemicentin-1-like isoform X1 [Ostrinia furnacalis]	1	91	10.3	5.24	13	1	1	High	1	0.904	1.021	1.518	1.537	1.311	1.258	1.355	1.341	1.857	1.735	1.872	4.951	5.396	5.058
TRINITY_DN2621_c0_g1_i1_orf1	GPN-loop GTPase 1 [Ostrinia furnacalis]	2	374	41.1	4.89	6	2	2	High	1	1.018	1.011	1.085	1.005	1.262	0.892	0.957	0.885	0.892	0.839	0.892	0.807	0.785	0.818
TRINITY_DN5161_c0_g1_i5_orf1	glyoxylate reductase/hydroxypyruvate reductase-like isoform X1 [Ostrinia furn	2	324	35.4	8.37	6	3	2	High	1	0.988	0.986	0.762	0.855	0.743	0.931	0.909	0.982	0.67	0.743	0.646	0.684	0.687	0.559
TRINITY_DN78686_c0_g1_i1_orf1	myosin-2 essential light chain isoform X2 [Harpegnathos saltator]	1	156	17.5	4.74	8	1	1	High	1	0.967	0.856	0.62	0.66	0.775	0.687	0.77	0.676	0.602	0.449	0.584	1.073	0.999	0.951
TRINITY_DN13898_c0_g1_i2_orf1	protein PRRC2A-like isoform X2 [Ostrinia furnacalis]	3	1788	198.3	9.31	1	3	3	High	1	1.013	0.956	0.933	1.041	0.95	0.78	0.957	0.875	1.088	0.751	0.93	1.27	1.362	1.181
TRINITY_DN103457_c0_g1_i1_orf1	28S ribosomal protein S22, mitochondrial [Ostrinia furnacalis]	2	379	43.6	8.32	7	2	2	High	1	0.987	0.995	0.459	0.46	0.412	0.487	0.544	0.488	0.484	0.541	0.574	0.48	0.553	0.545
TRINITY_DN1099_c1_g1_i4_orf1	syntaxin-12 isoform X1 [Ostrinia furnacalis]	2	276	30.5	6.1	9	3	2	High	1	1.042	1.107	1.152	1.08	1.067	0.969	1.135	1.122	1.131	0.946	1.041	1.099	1.109	1.061
TRINITY_DN3119_c0_g1_i7_orf1	unnamed protein product [Chilo suppressalis]	1	201	22.5	9.01	6	1	1	High	1	0.915	0.936	0.821	0.792	0.711	0.897	0.985	0.931	0.85	0.772	1.003	1.182	1.49	1.291
TRINITY_DN15411_c0_g1_i4_orf1	uncharacterized protein LOC114362040 isoform X1 [Ostrinia furnacalis]	2	284	32.7	6.84	9	2	2	High	1	1.045	1.029	1.24	1.273	1.219	1.136	1.155	1.139	1.206	1.336	1.268	1.655	1.587	1.518
TRINITY_DN5891_c0_g2_i4_orf1	amino acid transporter AVT1A-like [Ostrinia furnacalis]	1	451	48.1	7.85	2	1	1	High	1	0.996	1.064	0.18	0.135	0.226	0.253	0.202	0.212	0.232	0.244	0.179	0.201	0.176	0.218
TRINITY_DN9492_c1_g1_i1_orf1	aldo-keto reductase AKR2E4-like [Galleria mellonella]	3	107	12.3	6.71	32	3	3	High	1	0.941	1.117	1.592	1.548	1.752	1.97	2.064	2.078	1.891	1.948	1.758	1.372	1.369	1.602
TRINITY_DN22053_c0_g1_i13_orf1	uncharacterized protein LOC114355104 [Ostrinia furnacalis]	2	540	61.5	8.16	3	2	2	High	1	0.963	0.98	1.502	1.402	1.449	1.549	1.504	1.548	1.564	1.809	1.584	2.256	2.07	2.236
TRINITY_DN29291_c0_g1_i1_orf1	carboxylesterase [Ostrinia furnacalis]	2	223	26.3	8.41	8	3	1	High	1	1.001	1.053	0.288	0.496	0.416	0.39	0.435	0.421	0.438	0.624	0.458	0.471	0.425	0.5
TRINITY_DN79673_c0_g1_i1_orf1	thioredoxin, mitochondrial-like [Ostrinia furnacalis]	2	150	16.7	8.57	16	2	2	High	1	1.045	0.937	0.683	0.737	0.739	0.83	0.789	0.806	0.81	0.673	0.738	0.526	0.547	0.468
TRINITY_DN8502_c0_g1_i2_orf1	unnamed protein product [Chilo suppressalis]	1	512	56.8	7.27	3	1	1	High	1	0.995	0.85	0.774	0.872	0.667	0.864	0.886	0.713	0.811	1.168	0.792	0.774	0.808	0.84
TRINITY_DN1920_c0_g1_i4_orf1	conserved oligomeric Golgi complex subunit 1 isoform X1 [Ostrinia furnacalis]	2	937	107	6.19	2	2	2	High	1	0.958	1	0.949	0.966	1.079	0.888	1.233	1.055	1.041	1.194	0.956	0.943	0.96	0.871
TRINITY_DN19250_c0_g2_i2_orf1	uncharacterized protein LOC114351683 isoform X8 [Ostrinia furnacalis]	2	182	22.2	7.37	9	2	2	High	1	1.106	0.999	0.696	0.629	0.705	0.908	0.844	0.888	0.806	0.78	0.656	0.878	0.784	0.834
TRINITY_DN10812_c0_g1_i3_orf1	sodium/potassium-transporting ATPase subunit beta-2-like [Ostrinia furnacali:	2	279	32.5	7.4	6	2	2	High	1	0.941	1.007	0.797	0.866	0.949	0.994	1.096	1.001	1.053	0.954	1.11	0.91	0.912	0.89
TRINITY_DN10325_c0_g1_i4_orf1	uncharacterized protein LOC114351977 isoform X1 [Ostrinia furnacalis]	2	972	107.2	4.98	2	2	2	High	1	1.039	0.917	0.918	1.142	1.042	1.004	1.106	1.007	1.042	0.813	1.147	0.911	0.885	0.902
TRINITY_DN34751_c0_g1_i1_orf1	peroxisomal acyl-coenzyme A oxidase 3 isoform X3 [Ostrinia furnacalis]	1	91	9.8	4.88	13	2	1	High	1	1.074	1.096	0.962	0.983	1.021	1.041	1.067	1.012	1.089	0.982	1.096	1.267	1.241	1.267
TRINITY_DN6901_c0_g1_i4_orf1	(E3-independent) E2 ubiquitin-conjugating enzyme isoform X1 [Ostrinia furna	3	836	91.9	5.45	3	3	3	High	1	0.894	0.901	0.778	0.784	0.843	0.829	0.935	0.873	0.824	0.67	0.805	0.853	0.882	0.808
TRINITY_DN1628_c0_g2_i3_orf1	uncharacterized protein LOC114363979 [Ostrinia furnacalis]	1	218	24.7	5.25	7	1	1	High	1	0.919	1.014	1.014	1.085	0.956	0.86	1.01	0.871	1.575	1.426	1.282	1.05	1.102	0.999
TRINITY_DN105157_c0_g1_i1_orf1	potential E3 ubiquitin-protein ligase ariadne-2 [Ostrinia furnacalis]	2	487	56.5	6.05	4	2	2	High	1	0.819	0.946	0.935	0.977	0.956	1.005	1.033	0.972	0.931	0.967	0.888	0.898	0.68	0.805
TRINITY_DN338_c0_g1_i1_orf1	chorion protein S36-like [Ostrinia furnacalis]	1	490	50.9	8.56	3	1	1	High	1	1.019	1.068	1.085	1.401	1.089	0.918	1.088	1.045	1.086	0.969	1.033	4.819	5.114	4.849
TRINITY_DN12087_c0_g1_i2_orf1	unnamed protein product [Euphydryas editha]	3	115	12.6	9.66	31	4	3	High	1	0.935	0.982	0.739	0.688	0.695	0.774	0.966	0.804	0.824	0.866	0.871	0.723	0.778	0.747
TRINITY_DN40281_c0_g1_i1_orf1	thioredoxin domain-containing protein 15 [Ostrinia furnacalis]	1	295	33.4	5.47	6	1	1	High	1	1.166	1.082	1.571	1.594	1.456	1.642	1.443	1.492	1.467	2.677	1.497	1.144	1.072	1.13
TRINITY_DN15965_c0_g1_i1_orf1	U3 small nucleolar RNA-associated protein 15 homolog [Ostrinia furnacalis]	2	524	57.9	7.62	4	2	2	High	1	0.991	0.98	0.385	0.398	0.502	0.51	0.47	0.453	0.446	0.464	0.394	0.386	0.431	0.459
TRINITY_DN12527_c0_g1_i4_orf1	DNA-directed RNA polymerase III subunit RPC4 isoform X1 [Ostrinia furnacalis	1	372	40.9	7.44	3	1	1	High	1	0.829	0.983	0.621	0.679	0.54	0.622	0.748	0.625	0.455	0.561	0.71	0.724	0.752	0.793
TRINITY_DN754_c1_g1_i6_orf1	28S ribosomal protein S2, mitochondrial [Ostrinia furnacalis]	1	250	28.4	9.03	5	1	1	High	1	1.174	1.154	0.344	0.372	0.425	0.459	0.435	0.43	0.322	0.239	0.429	0.474	0.372	0.376
TRINITY_DN1505_c0_g1_i1_orf1	uncharacterized protein LOC114362816 isoform X1 [Ostrinia furnacalis]	2	500	56	8.69	4	2	2	High	1	1.008	0.984	0.29	0.345	0.411	0.293	0.41	0.337	0.354	0.254	0.412	0.386	0.375	0.432
TRINITY_DN57150_c0_g2_i1_orf1	vacuolar protein-sorting-associated protein 25-like isoform X2 [Ostrinia furna	1	175	20.3	4.92	9	2	1	High	1	1.099	1.091	0.94	0.957	0.883	0.871	0.968	0.873	0.868	0.735	0.83	0.806	0.98	0.793
TRINITY_DN37366_c0_g1_i7_orf1	juvenile hormone epoxide hydrolase-like [Ostrinia furnacalis]	1	460	52	8.02	2	1	1	High	1	0.948	1.013	0.749	0.582	0.753	0.737	0.819	0.815	0.812	0.623	0.632	0.701	0.824	0.783
TRINITY_DN14018_c0_g1_i4_orf1	chitobiosyldiphosphodolichol beta-mannosyltransferase [Ostrinia furnacalis]	2	435	49.8	7.87	6	2	2	High	1	0.899	0.896	0.552	0.535	0.558	0.525	0.501	0.498	0.504	0.564	0.532	0.669	0.61	0.68
TRINITY_DN24909_c0_g1_i5_orf1	exportin-5-like [Ostrinia furnacalis]	2	408	44.6	6.98	6	2	2	High	1	1.036	1.041	0.862	0.786	0.966	0.9	0.989	0.821	0.905	0.94	0.985	0.881	0.91	0.898
TRINITY_DN754_c1_g1_i8_orf1	lysophospholipid acyltransferase 5 [Ostrinia furnacalis]	2	473	55.3	8.53	3	2	2	High	1	0.934	1.018	0.488	0.476	0.458	0.513	0.48	0.562	0.51	0.512	0.431	0.636	0.637	0.648
TRINITY_DN18338_c0_g1_i7_orf1	aquaporin AQPAn.G isoform X2 [Ostrinia furnacalis]	1	259	27	6.9	5	2	1	High	1	1.068	1.025	0.931	0.784	0.931	0.879	0.897	0.854	0.857	0.883	0.715	0.739	0.792	0.686
TRINITY_DN5513_c0_g1_i1_orf1	GDP-Man:Man(3)GlcNAc(2)-PP-Dol alpha-1,2-mannosyltransferase [Ostrinia f	2	486	55.6	8.56	5	2	2	High	1	0.933	0.997	1.021	1.047	1.145	0.949	1.233	1.048	0.891	0.91	0.933	0.756	0.769	0.849
TRINITY_DN22049_c0_g1_i1_orf1	synaptobrevin-1-like [Trichoplusia ni]	1	122	13.3	9.13	14	1	1	High	1	1.011	1.042	0.88	0.927	0.923	0.998	0.915	0.936	1.042	0.869	0.636	1.028	1.157	1.076
TRINITY_DN213_c0_g1_i3_orf1	unnamed protein product [Chilo suppressalis]	2	1477	161.3	5.68	2	2	2	High	1	0.927	1.057	1.019	1.055	0.989	1.058	1.252	1.318	1.288	1.33	1.168	1.157	1.253	1.217
TRINITY_DN37923_c0_g1_i1_orf1	hypothetical protein NE865_05974 [Phthorimaea operculella]	2	168	19.3	4.64	13	3	2	High	1	0.918	0.946	0.711	0.717	0.755	0.646	0.664	0.652	0.63	0.56	0.684	0.913	0.953	0.85
TRINITY_DN22797_c0_g1_i5_orf1	phenoloxidase-activating factor 2-like isoform X1 [Ostrinia furnacalis]	2	119	13.5	5.78	14	4	1	High	1	0.974	1.15	1.683	1.509	1.333	1.242	1.122	1.209	1.211	1.209	1.315	0.754	0.957	0.723
TRINITY_DN46372_c0_g1_i1_orf1	unnamed protein product [Chilo suppressalis]	1	164	18.1	9.01	9	4	1	High	1	1.052	1.057	1.651	1.702	1.785	1.249	1.251	1.246	1.202	1.124	1.31	0.671	0.64	0.703
TRINITY_DN5630_c4_g1_i2_orf1	PREDICTED: stress-associated endoplasmic reticulum protein 2 [Amyelois trans	1	64	7.2	9.99	13	3	1	High	1	0.975	0.972	0.703	0.752	0.714	0.57	0.583	0.534	0.635	0.529	0.636	0.672	0.674	0.664
TRINITY_DN72541_c0_g1_i2_orf1	xaa-Pro aminopeptidase ApepP-like isoform X2 [Ostrinia furnacalis]	1	102	11.6	6.54	24	1	1	High	1	1.123	1.175	0.896	0.945	0.833	0.75	0.62	0.824	0.832	0.73	0.726	0.544	0.546	0.581
TRINITY_DN56250_c0_g1_i7_orf1	sex-lethal homolog isoform X3 [Ostrinia furnacalis]	1	370	41.5	9.22	4	1	1	High	1	0.867	0.932	0.645	0.653	0.692	0.759	0.932	0.816	0.75	0.857	0.684	0.47	0.638	0.647
TRINITY_DN2885_c1_g1_i2_orf1	ubiquitin-like-specific protease ESD4 [Ostrinia furnacalis]	2	699	79.7	8.69	3	2	2	High	1	1.011	0.935	0.603	0.739	0.679	0.547	0.547	0.521	0.553	0.452	0.463	0.831	0.846	0.887
TRINITY_DN14239_c0_g1_i5_orf1	uncharacterized protein LOC114352770 [Ostrinia furnacalis]	1	286	33.6	7.4	5	1	1	High	1	0.793	1.015	1.078	1.227	1.11	1.69	1.771	1.835	1.151	1.38	1.235	1.259	1.12	1.257
TRINITY_DN23175_c0_g1_i6_orf1	myb-binding protein 1A-like protein [Ostrinia furnacalis]	1	1143	130.2	7.18	1	1	1	High	1	0.916	1.008	0.225	0.245	0.261	0.236	0.271	0.247	0.27	0.248	0.223	0.311	0.311	0.316
TRINITY_DN1333_c0_g1_i6_orf1	uncharacterized protein LOC114362563 [Ostrinia furnacalis]	1	320	35.2	4.91	6	1	1	High	1	1.025	0.896	1.282	1.145	1.216	1.167	1.376	1.059	1.159	1.152	0.953	0.762	0.896	0.773
TRINITY_DN13157_c0_g1_i1_orf1	TBC1 domain family member 23 [Ostrinia furnacalis]	2	679	76.8	5.4	4	2	2	High	1	0.977	1.001	1.001	1.027	0.903	1.056	1.215	1.013	0.965	1.109	0.872	0.843	0.883	0.891
TRINITY_DN33430_c0_g1_i5_orf1	NADH dehydrogenase [ubiquinone] iron-sulfur protein 2, mitochondrial [Ostri	2	191	21.9	5.27	11	3	1	High	1	0.953	1.055	0.998	0.79	0.921	1.381	1.247	1.323	1.83	1.58	0.926	1.334	1.675	1.647
TRINITY_DN84016_c0_g1_i1_orf1	multidrug resistance-associated protein 4-like isoform X1 [Ostrinia furnacalis]	2	1339	150	7.53	2	2	2	High	1	0.989	1.132	0.886	0.902	0.992	0.993	1.018	1.077	1.298	0.903	0.776	0.871	0.876	0.863
TRINITY_DN8390_c0_g1_i2_orf1	tubulin gamma-1 chain-like isoform X1 [Ostrinia furnacalis]	2	456	51.4	6.07	5	2	2	High	1	1.004	0.947	1.076	1.114	1.065	0.987	0.986	1.039	0.96	1.052	1.024	1.539	1.545	1.486
TRINITY_DN34816_c0_g1_i4_orf1	uncharacterized protein LOC114355576 [Ostrinia furnacalis]	1	337	38.1	5.94	5	1	1	High	1	0.994	1.052	1.143	1.116	1.194	1.449	1.247	1.28	1.072	1.249	1.36	1.224	1.298	1.456
TRINITY_DN1639_c0_g2_i2_orf1	longitudinals lacking protein-like [Plutella xylostella]	2	128	14.6	7.9	17	2	2	High	1	1.131	1.153	0.86	0.673	0.703	0.8	0.77	0.787	0.807	0.735	0.663	0.897	0.888	0.941
TRINITY_DN12432_c0_g1_i2_orf1	sorting nexin-8 isoform X3 [Ostrinia furnacalis]	1	354	39.7	7.39	5	1	1	High	1	1.002	1.229	1.449	1.446	1.363	1.015	0.888	1.08	1.163	1.247	1.068	1.456	1.232	1.14
TRINITY_DN11894_c1_g1_i5_orf1	39S ribosomal protein L33, mitochondrial [Ostrinia furnacalis]	1	67	7.9	10.23	18	1	1	High	1	1.104	0.946	0.488	0.607	0.443	0.537	0.588	0.507	0.336	0.449	0.477	0.561	0.431	0.468
TRINITY_DN311_c0_g1_i8_orfp1	TRINITY_DN311_c0_g1_i8_m.65152 TRINITY_DN311_c0_g1::TRINITY_DN311_c0	1	137	15	4.83	18	2	1	High	1	1.215	1.218	2.518	2.014	2.202	0.972	0.882	1.007	2.647	2.322	2.915	1.036	1.047	1.119
TRINITY_DN4694_c0_g2_i1_orf1	uncharacterized protein LOC114362122 [Ostrinia furnacalis]	2	651	76	6.73	4	2	2	High	1	0.998	1.033	0.946	0.809	0.86	0.93	0.889	0.747	0.938	1.028	0.851	1.541	1.55	1.582
TRINITY_DN14019_c0_g1_i5_orf1	hypothetical protein evm_009768 [Chilo suppressalis]	1	105	10.8	9.41	14	1	1	High	1	0.929	0.935	0.491	0.438	0.456	0.458	0.829	1.081	0.621	0.617	0.542	1.063	1.157	1.241
TRINITY_DN3833_c0_g1_i4_orf1	division abnormally delayed protein [Ostrinia furnacalis]	2	550	60	5.64	3	2	2	High	1	0.909	1.044	0.958	0.943	0.989	1.018	1.074	0.914	1.033	0.913	1.128	2.397	2.283	2.29
TRINITY_DN6567_c0_g1_i5_orf1	dymeclin isoform X1 [Ostrinia furnacalis]	3	714	79.8	6.49	4	3	3	High	1	1.005	0.968	0.826	0.896	0.897	0.932	0.771	0.806	0.768	0.657	0.686	0.889	0.95	0.864

TRINITY_DN8812_c0_g1_i1_orf1	nuclear pore complex protein Nup160 homolog isoform X3 [Ostrinia furnacalis	2	496	54.3	8.12	4	2	2	High	1	0.926	0.86	1.229	1.138	1.222	1.104	1.037	1.15	1.316	1.205	1.093	1.322	1.218	1.285
TRINITY_DN6418_c0_g1_i28_orf1	peritrophic membrane chitin binding protein [Loxostege sticticalis]	1	446	48	4.37	3	1	1	High	1	1.072	0.992	0.252	0.345	0.25	0.267	0.283	0.286	0.312	0.271	0.305	0.247	0.209	0.206
TRINITY_DN68397_c0_g1_i2_orf1	clavesin-1-like [Ostrinia furnacalis]	2	298	34.6	7.33	8	2	2	High	1	0.935	0.991	1.003	0.909	0.825	0.838	0.729	0.924	0.936	1.127	0.971	3.897	3.908	4.124
TRINITY_DN16933_c0_g1_i10_orf1	uridine phosphorylase 1-like [Ostrinia furnacalis]	2	392	43.7	6.38	6	2	2	High	1	0.957	1.028	0.719	0.705	0.775	0.727	0.804	0.825	0.735	0.68	0.637	0.685	0.77	0.798
TRINITY_DN87522_c0_g2_i1_orf1	regucalcin-like [Ostrinia furnacalis]	2	67	7.7	5.1	31	2	1	High	1	1.005	1.018	1.02	0.997	1.066	1.327	0.902	1.022	0.91	0.941	0.879	1.146	1.201	1.085
TRINITY_DN17772_c0_g2_i3_orf1	uncharacterized protein LOC114351684 [Ostrinia furnacalis]	2	244	28	6.74	10	2	2	High	1	1.022	1.044	1.19	0.841	1.171	1.006	0.926	1.22	1.506	1.454	1.073	1.444	1.322	1.494
TRINITY_DN7212_c0_g1_i4_orf1	peptidylglycine alpha-hydroxylating monooxygenase [Ostrinia furnacalis]	2	336	38.1	6.6	8	2	2	High	1	1.01	1.07	1.443	1.206	1.235	1.578	1.666	1.722	2.025	2.004	1.59	1.461	1.383	1.471
TRINITY_DN73900_c0_g1_i1_orf1	carbonic anhydrase 7 [Ostrinia furnacalis]	1	320	36.5	8.48	3	4	1	High	1	0.914	1.012	2.427	2.668	2.523	2.503	2.058	2.37	3.04	2.445	3.134	1.349	1.152	1.05
TRINITY_DN22772_c0_g1_i5_orf1	probable ATP-dependent RNA helicase DDX46 isoform X2 [Ostrinia furnacalis]	2	956	107.1	9.07	3	2	2	High	1	1.025	1.052	1.028	1.06	0.837	1.12	1.055	1.097	1.048	1.193	1.187	1.166	0.998	1.063
TRINITY_DN64510_c0_g1_i1_orf1	39S ribosomal protein L15, mitochondrial [Ostrinia furnacalis]	2	294	33.9	9.23	7	2	2	High	1	0.983	0.955	0.363	0.387	0.426	0.417	0.422	0.366	0.346	0.419	0.324	0.412	0.383	0.412
TRINITY_DN14391_c1_g1_i2_orf1	pre-rRNA-processing protein TSR1 homolog [Ostrinia furnacalis]	2	437	50.6	6.89	6	2	2	High	1	0.976	0.862	0.886	0.718	0.855	0.917	0.896	1.019	1.425	1.156	1.297	1.289	1.268	1.235
TRINITY_DN66040_c0_g1_i2_orf1	serine protease inhibitor dipetalogastin-like isoform X2 [Ostrinia furnacalis]	1	53	6	6.28	21	3	1	High	1	1.191	1.028	4.317	3.809	3.793	3.08	3.842	2.709	7.698	6.297	7.564	19.572	19.656	18.675
TRINITY_DN29440_c1_g1_i4_orf1	neutral lipase [Helicoverpa armigera]	1	105	11.7	9.96	10	2	1	High	1	0.93	0.92	0.199	0.408	0.362	0.275	0.452	0.25	0.361	0.355	0.454	0.459	0.442	0.463
TRINITY_DN122393_c0_g1_i1_orf1	microtubule-associated protein futsch isoform X4 [Ostrinia furnacalis]	2	86	9.5	9.57	29	3	2	High	1	1.078	1.02	1.381	1.481	1.277	1.291	0.957	1.062	0.985	0.899	1.066	0.535	0.732	0.582
TRINITY_DN3499_c0_g1_i8_orf1	modular serine protease-like [Ostrinia furnacalis]	2	669	73.2	5.3	6	2	2	High	1	1.11	1.107	0.967	0.913	0.96	0.887	0.739	0.801	0.931	0.873	0.991	1.294	1.27	1.211
TRINITY_DN8641_c0_g1_i1_orf1	uncharacterized protein LOC114357057 [Ostrinia furnacalis]	2	474	52.6	6.7	6	2	2	High	1	0.991	1.092	1.01	1.039	1.112	1.179	1.255	1.053	1.167	1.216	1.116	1.847	1.75	1.794
TRINITY_DN20749_c0_g1_i3_orf1	protein arginine N-methyltransferase 1-like [Ostrinia furnacalis]	2	522	59.5	5.1	4	2	2	High	1	0.946	0.898	0.503	0.524	0.576	0.467	0.488	0.539	0.505	0.403	0.401	0.486	0.447	0.501
TRINITY_DN5086_c0_g1_i1_orf1	unnamed protein product [Diatraea saccharalis]	1	77	8.1	10.37	23	3	1	High	1	0.962	0.989	0.691	0.756	0.741	0.633	0.576	0.595	0.559	0.496	0.684	0.709	0.701	0.777
TRINITY_DN3343_c0_g2_i1_orf1	AFG3-like protein 2 [Ostrinia furnacalis]	2	200	22.3	5.05	14	2	2	High	1	0.994	1.069	0.605	0.705	0.601	0.636	0.702	0.694	0.585	0.504	0.563	0.764	0.825	0.797
TRINITY_DN121893_c0_g1_i1_orf1	hypothetical protein, partial [Ostrinia furnacalis]	2	76	8.5	9.45	24	2	1	High	1	0.902	0.862	0.693	0.666	0.633	0.744	0.646	0.497	0.45	0.418	0.427	0.561	0.584	0.425
TRINITY_DN8555_c0_g1_i1_orf1	epoxide hydrolase 4-like [Ostrinia furnacalis]	2	359	39.5	9	6	2	2	High	1	0.918	0.979	1.112	1.235	1.077	1.282	1.218	1.514	1.357	1.349	1.423	1.608	1.305	1.261
TRINITY_DN8107_c0_g1_i1_orf1	protein pelota [Ostrinia furnacalis]	2	389	43.7	6.18	5	2	2	High	1	0.977	0.979	1.022	0.998	0.99	1.083	0.955	0.927	0.733	0.842	0.9	0.934	0.86	0.806
TRINITY_DN1254_c0_g1_i1_orf1	lysophospholipid acyltransferase 7-like [Ostrinia furnacalis]	2	488	56.5	9.14	4	2	2	High	1	1.132	0.99	1.214	1.319	1.351	1.054	1.162	1.083	1.078	0.771	1.122	1.406	1.453	1.405
TRINITY_DN34465_c0_g1_i1_orf1	putative peptidyl-tRNA hydrolase PTRHD1 [Ostrinia furnacalis]	2	128	14.6	7.4	16	2	2	High	1	1.071	0.966	0.846	0.853	0.742	1.68	1.616	1.534	0.978	0.916	1.026	1.098	1.143	1.114
TRINITY_DN43293_c0_g1_i2_orf1	egl nine homolog 1 isoform X2 [Helicoverpa armigera]	2	483	54	8.18	5	2	2	High	1	1.044	0.998	0.603	0.579	0.486	0.661	0.743	0.924	0.487	0.394	0.428	0.907	0.708	0.754
TRINITY_DN62557_c0_g1_i1_orf1	6-phosphofructokinase [Operophtera brumata]	2	64	7	8.94	38	2	2	High	1	0.983	0.965	1.264	1.105	1.189	1.226	1.079	1.17	1.367	1.279	1.234	0.832	0.903	0.948
TRINITY_DN3759_c0_g1_i1_orf1	uncharacterized protein LOC114350416 [Ostrinia furnacalis]	2	1131	132.4	7.61	2	2	2	High	1	1.005	0.956	0.367	0.409	0.382	0.405	0.41	0.389	0.326	0.292	0.329	0.714	0.83	0.69
TRINITY_DN3591_c0_g1_i1_orf1	sodium/potassium-transporting ATPase subunit beta-2-like [Ostrinia furnacalis	1	325	36.7	5.58	6	1	1	High	1	1.211	1.321	0.969	1.15	1.272	1.432	1.162	1.481	1.011	1.654	1.419	1.183	0.904	1.211
TRINITY_DN24469_c0_g2_i2_orf1	unnamed protein product, partial [Brenthis ino]	2	90	10.6	9.19	13	2	2	High	1	0.982	1.002	0.648	0.644	0.677	0.738	0.741	0.675	0.675	0.575	0.699	1.098	1.135	1.192
TRINITY_DN5503_c0_g1_i5_orf1	protein PRRC1-like isoform X1 [Ostrinia furnacalis]	2	364	38.2	5.39	5	2	2	High	1	0.871	1	1.241	0.987	1.01	1.056	1.191	0.994	1.122	0.892	1.037	1.131	1.22	1.232
TRINITY_DN33452_c0_g1_i3_orf1	lethal(2) giant larvae protein isoform X8 [Ostrinia furnacalis]	1	97	10.7	8.47	18	1	1	High	1	1.045	0.985	1.029	1.072	1.204	0.3	0.439	0.378	0.531	0.51	0.56	0.624	0.625	0.636
TRINITY_DN34166_c0_g1_i1_orf1	hypothetical protein EVAR_80688_1 [Eumeta japonica]	2	91	10.6	8.44	24	2	2	High	1	1.054	0.976	1.494	1.609	1.563	1.608	1.678	1.456	1.621	1.425	1.592	1.612	1.475	1.654
TRINITY_DN25360_c0_g1_i2_orf1	segment polarity protein dishevelled homolog DVL-3 [Vanessa atalanta]	1	621	67.1	6.67	3	1	1	High	1	0.779	0.723	0.819	0.74	0.832	0.833	0.934	0.941	0.766	1.048	0.826	1.076	1.035	1.04
TRINITY_DN19980_c0_g1_i4_orf1	hypothetical protein evm_012507 [Chilo suppressalis]	1	341	37.3	6.25	4	1	1	High	1	1.007	1.119	2.501	2.949	2.126	1.733	2.43	2.539	1.92	1.938	1.996	3.164	2.995	2.986
TRINITY_DN7803_c0_g1_i2_orf1	membrane-associated protein Hem [Ostrinia furnacalis]	2	1125	129.5	6.8	2	3	2	High	1	1.025	1.012	0.792	0.752	0.793	0.906	0.975	0.863	0.76	0.731	0.782	1.751	1.644	1.667
TRINITY_DN39266_c0_g1_i1_orf1	PREDICTED: NECAP-like protein CG9132 [Microplitis demolitor]	1	258	28.7	8.53	6	1	1	High	1	1.013	1.016	2.183	1.856	2.003	2.008	2.067	2.054	2.621	2.206	2.31	2.17	2.192	2.633
TRINITY_DN8543_c0_g1_i1_orf1	39S ribosomal protein L38, mitochondrial [Ostrinia furnacalis]	1	394	46.4	9.13	4	1	1	High	1	1.007	0.97	0.429	0.438	0.49	0.334	0.31	0.299	0.296	0.254	0.293	0.364	0.33	0.36
TRINITY_DN5460_c0_g1_i5_orf1	unnamed protein product [Parnassius apollo]	1	1006	111.5	8.62	1	1	1	High	1	0.985	0.844	0.942	1.027	1.015	0.762	0.838	0.834	0.81	0.832	0.879	0.993	0.895	0.989
TRINITY_DN5105_c0_g1_i10_orf1	poly(U)-binding-splicing factor half pint [Ostrinia furnacalis]	2	623	66.1	6.95	4	2	2	High	1	0.902	0.957	0.743	0.809	0.73	0.745	0.786	0.776	0.591	1.05	0.614	0.833	0.701	0.777
TRINITY_DN19748_c0_g1_i4_orf1	PREDICTED: cysteine-rich hydrophobic domain-containing protein 2 [Amyelois	2	165	18.9	6.3	15	2	2	High	1	1.144	1.188	1.683	1.761	1.937	1.647	1.871	1.809	1.812	1.734	1.544	1.842	2.179	2.135
TRINITY_DN2784_c0_g1_i3_orf1	toll-like receptor 6 [Ostrinia furnacalis]	2	913	103.3	7.2	2	2	2	High	1	0.988	1.016	1.578	1.461	1.493	1.625	1.405	1.857	1.929	1.955	1.35	2.016	1.731	1.591
TRINITY_DN2623_c0_g1_i3_orf1	unnamed protein product [Chilo suppressalis]	2	1915	214.6	7.18	1	2	2	High	1	1.097	1.045	0.622	0.629	0.626	0.468	0.518	0.589	0.524	0.535	0.518	0.83	0.774	0.777
TRINITY_DN2822_c0_g1_i4_orf1	uncharacterized protein LOC114354271 isoform X1 [Ostrinia furnacalis]	2	523	56	6.68	4	3	2	High	1	0.98	0.972	0.901	0.939	0.96	0.972	0.955	1.047	0.867	0.907	0.812	0.726	0.783	0.803
TRINITY_DN36987_c0_g1_i1_orf1	basement membrane-specific heparan sulfate proteoglycan core protein isofo	1	73	7.7	4.61	19	3	1	High	1	1.249	1.231	1.035	1.049	1.099	1.238	1.201	1.297	0.961	0.739	0.943	1.435	1.4	1.334
TRINITY_DN44557_c0_g2_i1_orf1	serine hydrolase-like protein [Ostrinia furnacalis]	1	283	32.3	7.91	5	1	1	High	1	0.961	0.86	0.535	0.585	0.505	0.511	0.503	0.573	0.435	0.393	0.508	0.626	0.586	0.574
TRINITY_DN16749_c0_g1_i1_orf1	uncharacterized protein LOC114353228 [Ostrinia furnacalis]	2	191	20.8	10.05	7	2	2	High	1	0.901	0.96	0.561	0.494	0.637	0.502	0.488	0.502	0.485	0.491	0.526	0.478	0.495	0.526
TRINITY_DN1701_c0_g1_i4_orf1	protein farnesyltransferase/geranylgeranyltransferase type-1 subunit alpha [Os	2	335	39.4	5.91	7	2	2	High	1	0.981	1.058	1.032	1.046	0.999	1.017	1.134	0.947	1.077	0.884	0.974	1.009	0.962	1.002
TRINITY_DN8473_c0_g1_i5_orf1	serine/threonine-protein phosphatase 6 regulatory subunit 1 [Ostrinia furnaca	2	346	36.5	5.88	6	2	1	High	1	0.958	0.979	1.388	1.334	1.353	1.235	1.273	1.401	1.52	1.678	1.604	1.42	1.457	1.329
TRINITY_DN53531_c0_g1_i1_orf1	uncharacterized aarF domain-containing protein kinase 1 [Ostrinia furnacalis]	2	513	59.8	7.94	4	2	2	High	1	0.913	1.077	0.879	0.917	0.941	0.985	0.963	1.015	0.957	0.983	0.844	1.06	1.013	1.002
TRINITY_DN28592_c0_g1_i2_orf1	UDP-glucuronosyltransferase 2B14-like isoform X1 [Ostrinia furnacalis]	2	514	58.6	7.64	4	2	1	High	1	1.017	0.893	0.316	0.311	0.325	0.333	0.312	0.329	0.271	0.227	0.279	0.349	0.36	0.387
TRINITY_DN1554_c0_g1_i9_orf1	LOW QUALITY PROTEIN: puff-specific protein Bx42 [Ostrinia furnacalis]	1	554	62.3	9.28	3	1	1	High	1	1.004	1.024	0.679	0.608	0.736	0.685	0.715	0.685	0.848	0.69	0.686	0.95	1.078	1.019
TRINITY_DN54134_c0_g1_i1_orf1	NFU1 iron-sulfur cluster scaffold homolog, mitochondrial-like [Ostrinia furnaca	2	267	30.1	5.31	7	2	2	High	1	0.962	1.03	0.718	0.711	0.727	0.63	0.64	0.627	0.639	0.653	0.794	0.853	0.798	0.77
TRINITY_DN2996_c0_g1_i1_orf1	uroporphyrinogen-III synthase-like [Ostrinia furnacalis]	1	273	30.5	7.24	6	1	1	High	1	0.919	1.012	1.136	1.023	1.085	1.182	1.029	1.06	1.121	0.913	1.183	0.999	0.953	0.975
TRINITY_DN33183_c0_g1_i4_orf1	glutamate--cysteine ligase regulatory subunit [Ostrinia furnacalis]	2	334	36.3	6.52	6	2	2	High	1	0.994	1.003	0.568	0.611	0.612	0.635	0.657	0.618	0.614	0.633	0.594	0.71	0.72	0.701
TRINITY_DN2596_c0_g1_i2_orf1	rho guanine nucleotide exchange factor 11-like isoform X2 [Ostrinia furnacalis	2	482	52.2	5.14	3	2	1	High	1	0.963	0.999	0.775	0.952	0.929	1.039	0.81	0.87	0.882	0.865	0.731	1.026	0.944	1.005
TRINITY_DN5491_c0_g1_i4_orf1	bis(5'-nucleosyl)-tetraphosphatase [asymmetrical] [Ostrinia furnacalis]	1	141	16.3	5.38	13	1	1	High	1	0.766	0.937	0.923	1.02	1.123	0.852	0.983	1.002	1.222	1.042	1.138	0.997	1.113	1.144
TRINITY_DN30673_c0_g1_i5_orf1	protein phosphatase 1 regulatory subunit 21 [Ostrinia furnacalis]	1	435	49.9	5.96	3	1	1	High	1	0.9	0.918	1.042	1.026	1.032	0.785	0.814	0.816	0.745	0.96	0.912	1.057	1.146	1.129
TRINITY_DN2265_c0_g2_i1_orf1	LOW QUALITY PROTEIN: elongation factor G, mitochondrial-like [Leguminivor	1	162	18.1	5.57	7	2	1	High	1	1.071	1.006	0.392	0.399	0.457	0.387	0.552	0.46	0.503	0.443	0.405	0.571	0.603	0.515
TRINITY_DN3109_c0_g1_i5_orf1	protein takeout isoform X2 [Ostrinia furnacalis]	2	247	27.6	6.55	11	2	2	High	1	0.853	0.845	1.767	1.78	1.93	1.496	1.529	1.411	1.363	1.669	1.358	2.007	2.029	1.928
TRINITY_DN30300_c0_g2_i1_orf1	60S acidic ribosomal protein P2 isoform X2 [Ovis aries]	2	115	11.7	4.54	17	6	1	High	1	0.868	0.881	1.068	1.136	1.057	1.095	1.354	1.26	1.354	1.468	1.245	1.223	1.072	1.024
TRINITY_DN3276_c0_g1_i4_orf1	hypothetical protein B5X24_HaOG201803 [Helicoverpa armigera]	2	77	8.4	6.89	13	2	2	High	1	0.972	0.932	1.225	1.23	1.141	1.359	1.318	1.277	1.07	0.842	1.141	1.245	1.384	1.247
TRINITY_DN1029_c0_g1_i1_orfp1	TRINITY_DN1029_c0_g1_i1_m.64408 TRINITY_DN1029_c0_g1::TRINITY_DN1029	1	54	6.1	10.17	56	1	1	High	1	1.121	1.166	1.384	1.374	1.323	1.296	1.159	1.285	1.22	1.024	1.486	0.668	0.555	0.455
TRINITY_DN1492_c0_g1_i4_orf1	sarcoplasmic calcium-binding protein isoform X2 [Ostrinia furnacalis]	2	280	32.3	6.86	6	3	2	High	1	1.045	1.082	1.173	1.106	1.195	1.087	1.085	1.134	0.824	0.686	0.805	1.261	1.223	1.189
TRINITY_DN74086_c0_g1_i1_orf1	UPF0489 protein C5orf22 homolog [Ostrinia furnacalis]	1	375	42.6	4.75	4	1	1	High	1	1.101	1.114	1.275	1.405	1.646	1.414	1.358	1.421	1.228	0.959	1.271	3.193	3.122	3.417
TRINITY_DN15244_c0_g1_i5_orf1	titin homolog [Ostrinia furnacalis]	2	1188	135.2	4.69	2	2	2	High	1	1.035	1.127	0.706	0.665	0.748	0.591	0.524	0.686	0.627	0.504	0.613	0.717	0.65	0.758
TRINITY_DN6972_c0_g1_i3_orf1	adapter molecule Crk [Ostrinia furnacalis]	2	292	33	6.57	10	2	2	High	1	1.264	0.938	1.23	1.268	1.101	1.119	1.283	1.117	1.325	1.296	1.245	1.163	1.167	1.218
TRINITY_DN261_c0_g1_i5_orfp1	TRINITY_DN261_c0_g1_i5_m.18559 TRINITY_DN261_c0_g1::TRINITY_DN261_c0	2	189	18.7	9.1	11	2	2	High	1	0.899	1.343	1.362	1.461	1.441	1.017	1.041	1.149	1.208	1.241	1.673	2.4	2.555	2.002
TRINITY_DN19885_c0_g1_i1_orf1	transmembrane emp24 domain-containing protein 5 [Ostrinia furnacalis]	1	234	27.1	6.68	6	1	1	High	1	0.944	0.946	0.836	0.985	0.865	1.147	0.98	0.797	0.882	0.885	0.802	1.341	1.236	1.174
TRINITY_DN9517_c0_g1_i7_orf1	transcriptional repressor p66-alpha [Ostrinia furnacalis]	2	488	51.8	9.99	8	2	2	High	1	0.943	1.106	0.939	1.092	0.898	0.945	0.908	0.906	0.78	0.996	0.903	1.096	1.06	1.132
TRINITY_DN146181_c0_g1_i1_orf1	vesicular integral-membrane protein VIP36 [Diachasma alloeum]	2	329	37.3	6.83	7	3	2	High	1	0.826	0.873	1.205	1.312	1.3	1.478	1.4	1.367	1.214	1.542	1.12	1.232	1.166	1.095
TRINITY_DN79319_c0_g1_i8_orfp1	TRINITY_DN79319_c0_g1_i8_m.49956 TRINITY_DN79319_c0_g1::TRINITY_DN79	2	83	9.6	8.78	20	2	2	High	1	0.993	0.971	0.22	0.217	0.269	0.284	0.295	0.334	0.306	0.332	0.28	0.259	0.251	0.238
TRINITY_DN1114_c0_g1_i4_orf1	vesicle-associated membrane protein 2 isoform X1 [Helicoverpa armigera]	1	115	12.5	5.19	14	1	1	High	1	0.951	1.084	1.233	1.028	1.16	1.201	1.035	1.087	1.154	1.241	1.237	1.027	1.037	0.898
TRINITY_DN40211_c0_g1_i1_orf1	rho GTPase-activating protein 44-like [Ostrinia furnacalis]	2	690	76.9	7.62	3	2	1	High	1	0.988	1.1	0.871	0.757	0.839	0.75	0.733	0.803	0.774	0.876	0.775	0.926	0.871	0.972
TRINITY_DN3193_c0_g1_i3_orf1	LOW QUALITY PROTEIN: DENN domain-containing protein Crag [Ostrinia furn	2	724	78.7	6.1	3	2	2	High	1	1.041	1.292	1.085	1.25	1.089	1.198	1.273	1.334	1.438	1.28	1.118	1.026	1.21	1.202
TRINITY_DN7966_c0_g1_i4_orf1	leucine-rich repeat neuronal protein 1-like [Ostrinia furnacalis]	2	500	57.2	5.72	4	2	2	High	1	0.94	1.009	0.305	0.283	0.354	0.311	0.322	0.306	0.317	0.29	0.279	0.346	0.361	0.33
TRINITY_DN42120_c0_g1_i2_orf1	FAM172 family protein homolog CG10038 [Ostrinia furnacalis]	2	327	36.6	7.25	9	2	2	High	1	0.885	0.881	1.203	1.306	1.254	0.965	1.088	1.129	1.051	1.146	1.104	0.939	0.825	0.906
TRINITY_DN4064_c0_g2_i1_orf1	disintegrin and metalloproteinase domain-containing protein 12 isoform X1 [C	2	1568	172.2	8.79	2	2	2	High	1	1.041	0.963	1.153	1.178	1.161	1.349	1.519	1.397	1.167	1.192	0.953	1.268	1.038	1.141
TRINITY_DN969_c0_g1_i3_orf1	protein UBASH3A homolog isoform X3 [Ostrinia furnacalis]	2	687	77	6.6	3	2	2	High	1	0.914	1.06	0.511	0.484	0.477	0.437	0.425	0.508	0.388	0.373	0.466	0.674	0.682	0.781
TRINITY_DN1073_c0_g1_i1_orf1	carboxylesterase [Loxostege sticticalis]	2	278	30.1	6.32	8	4	1	High	1	1.05	1.093	0.442	0.391	0.439	0.444	0.425	0.447	0.41	0.946	0.363	0.447	0.371	0.367
TRINITY_DN82426_c0_g1_i6_orfp1	lysosome-associated membrane glycoprotein 1-like isoform X4 [Ostrinia furna	1	121	12.6	4.49	12	2	1	High	1	0.994	0.945	0.996	0.95	0.91	1.205	1.385	1.252	0.978	1.075	1.188	2.734	2.66	2.941
TRINITY_DN66_c10_g1_i1_orf1	COP9 signalosome complex subunit 2 [Ostrinia furnacalis]	2	448	51.4	5.45	6	2	2	High	1	1.049	0.94	1.167	0.986	0.978	1.055	1.001	1.14	1.233	1.202	0.927	1.007	1.016	1.152
TRINITY_DN2614_c0_g2_i3_orf1	uncharacterized protein LOC114360661 [Ostrinia furnacalis]	1	113	12.7	6.27	18	1	1	High	1	0.951	1.192	1.631	1.383	1.794	1.696	1.746	2.046	1.954	2.555	1.924	1.589	1.327	1.737
TRINITY_DN79868_c0_g1_i1_orf1	lethal(2)neighbour of Tid protein [Ostrinia furnacalis]	2	494	57.7	9.17	3	2	2	High	1	0.902	1.024	0.746	0.755	0.746	0.609	0.629	0.686	0.572	0.523	0.546	0.861	0.806	0.789
TRINITY_DN8561_c0_g4_i1_orf1	dynactin subunit 4 [Ostrinia furnacalis]	2	328	36.9	8.75	6	2	2	High	1	0.862	0.92	0.639	0.63	0.639	0.67	0.794	0.631	0.594	0.612	0.569	0.747	0.716	0.805
TRINITY_DN3949_c1_g1_i1_orf1	probable cytochrome P450 304a1 isoform X2 [Ostrinia furnacalis]	2	149	16.8	8.29	17	2	2	High	1	1.031	1.118	0.416	0.48	0.418	0.719	0.827	0.752	0.375	0.49	0.496	0.41	0.422	0.379
TRINITY_DN121439_c0_g1_i1_orf1	unnamed protein product [Chilo suppressalis]	3	872	98	6.6	4	3	3	High	1	0.983	0.969	0.919	1.091	1.004	1.175	1.134	0.991	1.052	1.229	1.03	1.207	1.18	1.18
TRINITY_DN4299_c0_g1_i6_orf1	sterol O-acyltransferase 2 [Ostrinia furnacalis]	1	471	54.4	9.16	3	1	1	High	1	0.867	0.892	1.089	1.029	0.964	1.056	1.201	0.854	1.388	2.461	1.538	1.124	1.006	1.025
TRINITY_DN3665_c0_g1_i2_orf1	regucalcin-like isoform X2 [Ostrinia furnacalis]	1	62	6.9	6.24	16	1	1	High	1	1.23	1.033	0.359	0.394	0.425	0.379	0.341	0.348	0.289	0.342	0.386	0.372	0.358	0.39
TRINITY_DN95_c0_g1_i5_orf1	protein VAC14 homolog isoform X3 [Ostrinia furnacalis]	2	698	77.5	6.06	3	2	2	High	1	0.933	1.127	1.189	1.413	1.14	1.288	1.099	1.11	1.199	1.747	1.338	1.494	1.308	1.66
TRINITY_DN119893_c0_g2_i3_orf1	ATP-binding cassette sub-family F member 3 isoform X1 [Ostrinia furnacalis]	1	706	79.2	6.19	2	1	1	High	1	1.025	1.069	1.172	1.013	1.154	0.918	0.935	0.868	0.608	0.868	0.869	0.758	0.695	0.693
TRINITY_DN12222_c0_g1_i1_orf1	unnamed protein product [Chilo suppressalis]	1	1254	139.6	6.29	1	1	1	High	1	0.93	1.009	0.808	0.871	0.989	0.916	0.945	0.949	0.693	0.842	0.661	1.32	1.741	1.393
TRINITY_DN132857_c0_g1_i1_orf1	hypothetical protein evm_000559 [Chilo suppressalis]	3	250	28.9	8.78	12	3	3	High	1	0.941	0.943	0.889	0.995	0.924	1.016	0.967	0.958	0.871	0.979	0.905	1.124	1.158	1.173
TRINITY_DN49936_c0_g2_i1_orf1	39S ribosomal protein L20, mitochondrial [Ostrinia furnacalis]	2	159	18.6	9.79	12	2	2	High	1	0.916	0.927	0.403	0.393	0.456	0.498	0.524	0.542	0.556	0.551	0.448	0.598	0.538	0.509
TRINITY_DN11942_c0_g1_i1_orf1	hypothetical protein B5X24_HaOG213660 [Helicoverpa armigera]	1	129	13.6	5.03	8	2	1	High	1	1.097	1.104	1.375	1.265	1.222	1.22	1.102	1.128	0.827	0.738	0.872	1.107	1.208	1.119

TRINITY_DN11772_c0_g1_i1_orf1	conserved oligomeric Golgi complex subunit 2 [Ostrinia furnacalis]	2	692	80.1	7.93	4	2	2	High	1	1.07	1.176	1.29	1.129	1.121	1.218	1.243	1.286	0.97	1.155	1.229	2.304	1.955	1.831
TRINITY_DN437_c0_g1_i1_orf1	LOW QUALITY PROTEIN: fibronectin type-III domain-containing protein 3A-lik	1	626	68.1	6.93	2	1	1	High	1	0.913	0.868	0.849	0.716	0.902	0.71	0.703	0.8	0.745	0.693	0.818	0.896	0.78	0.706
TRINITY_DN15247_c0_g1_i2_orf1	probable G-protein coupled receptor Mth-like 3 isoform X1 [Ostrinia furnacali	1	410	46.6	6.87	2	1	1	High	1	1.037	0.996	3.426	2.998	3.032	3.494	3.28	3.557	2.64	2.428	2.093	3.933	4.273	4.164
TRINITY_DN43228_c0_g1_i1_orf1	enoyl-CoA delta isomerase 2, mitochondrial [Ostrinia furnacalis]	2	244	27.5	6.27	9	2	2	High	1	0.968	0.964	0.906	0.858	0.858	1.071	0.99	1.124	0.903	0.977	0.816	1.02	0.959	1.052
TRINITY_DN61777_c0_g1_i4_orf1	exocyst complex component 2 [Ostrinia furnacalis]	2	877	97.9	6.25	3	2	2	High	1	0.792	1.102	1.511	1.504	1.736	1.545	1.748	1.723	1.782	1.924	1.68	1.679	1.653	1.76
TRINITY_DN3766_c0_g1_i10_orf1	circadian clock-controlled protein-like [Ostrinia furnacalis]	2	238	25.9	8.38	6	2	2	High	1	1.028	0.966	1.814	1.755	1.543	1.653	1.602	1.428	1.418	1.251	1.502	1.464	1.369	1.315
TRINITY_DN125150_c0_g1_i1_orf1	aldehyde dehydrogenase, dimeric NADP-preferring isoform X5 [Ostrinia furna	2	77	8.3	5.78	22	3	2	High	1	0.952	0.778	1.748	1.826	1.814	2.261	2.052	1.981	1.786	1.547	1.678	2.143	2.095	2.358
TRINITY_DN24631_c0_g2_i1_orf1	O-GlcNAc hydrolase [Ostrinia furnacalis]	2	242	26.5	5.35	7	2	2	High	1	0.927	1.027	0.93	1.061	0.999	1.065	0.963	0.995	0.862	0.995	0.925	1.385	1.341	1.355
TRINITY_DN66442_c0_g2_i3_orf1	ubiquitin-conjugating enzyme E2 G1 [Papilio polytes]	2	168	19.3	5.27	11	4	2	High	1	1	0.95	0.855	1.148	1.094	0.947	0.815	0.817	0.728	0.886	0.714	0.994	0.97	0.947
TRINITY_DN1672_c0_g1_i6_orf1	cystinosin homolog isoform X1 [Ostrinia furnacalis]	1	430	48.7	6.52	2	1	1	High	1	0.979	1.126	2.147	1.843	1.971	2.437	2.497	2.554	1.796	1.696	1.861	2.234	2.088	2.25
TRINITY_DN4132_c0_g1_i14_orf1	thyroid receptor-interacting protein 11-like isoform X1 [Ostrinia furnacalis]	2	1527	176.3	5.16	2	2	2	High	1	0.923	0.974	0.708	0.794	0.765	0.77	0.844	0.715	0.555	0.505	0.535	0.744	0.829	0.645
TRINITY_DN43412_c0_g1_i2_orf1	U1 small nuclear ribonucleoprotein C [Ostrinia furnacalis]	1	163	17.6	9.42	6	1	1	High	1	0.955	0.916	0.523	0.586	0.569	0.496	0.408	0.356	0.362	0.314	0.393	0.694	0.738	0.588
TRINITY_DN8660_c0_g1_i1_orf1	chondroitin sulfate synthase 2 [Trichoplusia ni]	2	747	83.3	8.13	3	3	2	High	1	1.171	1.076	1.071	1.429	1.222	1.286	1.415	1.355	1.015	0.841	1.177	1.559	1.408	1.405
TRINITY_DN35162_c0_g1_i4_orf1	mannosyl-oligosaccharide glucosidase [Ostrinia furnacalis]	1	540	62	8.44	3	1	1	High	1	0.797	0.948	0.981	1.052	1.178	1.057	0.963	0.937	0.804	1.052	0.636	0.788	0.893	0.924
TRINITY_DN19829_c0_g1_i1_orf1	28S ribosomal protein S35, mitochondrial [Ostrinia furnacalis]	2	236	27.2	9.48	8	2	2	High	1	1	0.953	0.524	0.423	0.695	0.513	0.67	0.51	0.514	0.409	0.474	0.443	0.472	0.452
TRINITY_DN39170_c0_g1_i4_orf1	unnamed protein product [Arctia plantaginis]	1	57	6.2	4.37	32	1	1	High	1	1.054	0.971	0.667	0.714	0.683	0.653	0.726	0.67	0.877	0.679	1.004	0.873	0.82	1.005
TRINITY_DN27994_c0_g1_i1_orf1	uncharacterized protein LOC114364076 [Ostrinia furnacalis]	2	304	35	7.24	8	2	2	High	1	0.958	0.898	0.433	0.54	0.548	0.396	0.512	0.484	0.452	0.553	0.519	0.621	0.592	0.679
TRINITY_DN18568_c0_g1_i2_orfp1	TRINITY_DN18568_c0_g1_i2_m.13844 TRINITY_DN18568_c0_g1::TRINITY_DN18	1	76	8.5	8.19	13	1	1	High	1	0.659	0.781	0.828	0.759	1.084	4.39	3.7	4.065	5.237	5.981	3.741	0.458	0.979	1.187
TRINITY_DN19995_c0_g1_i2_orf1	E3 ubiquitin-protein ligase ZNF598 [Ostrinia furnacalis]	2	826	91.7	8.66	3	2	2	High	1	1.06	0.942	1.051	1.017	1.093	0.82	0.743	0.831	0.925	0.77	0.851	0.845	0.9	0.771
TRINITY_DN3401_c0_g1_i1_orf1	28S ribosomal protein S5, mitochondrial [Ostrinia furnacalis]	2	429	49	9.99	6	2	2	High	1	0.946	0.932	0.451	0.323	0.445	0.413	0.547	0.432	0.419	0.471	0.378	0.402	0.427	0.431
TRINITY_DN2168_c0_g1_i2_orf1	protein arginine methyltransferase NDUFAF7 homolog, mitochondrial [Ostrinia	1	437	49.6	6.87	5	1	1	High	1	1.076	1.163	0.501	0.66	0.663	0.664	0.68	0.594	0.518	0.515	0.501	0.502	0.607	0.54
TRINITY_DN21539_c0_g1_i1_orf1	probable phenylalanine--tRNA ligase, mitochondrial [Ostrinia furnacalis]	1	441	51	7.02	3	1	1	High	1	0.867	1.108	0.517	0.469	0.388	0.52	0.389	0.506	0.386	0.42	0.416	0.664	0.615	0.72
TRINITY_DN22875_c0_g1_i6_orf1	microtubule-actin cross-linking factor 1 isoform X15 [Ostrinia furnacalis]	1	98	11.1	4.97	13	1	1	High	1	0.99	1.19	2.222	1.903	2.121	2.23	1.912	1.824	1.818	1.629	1.662	1.231	1.465	1.436
TRINITY_DN701_c1_g1_i4_orf1	venom serine protease Bi-VSP-like [Ostrinia furnacalis]	2	183	20.4	5.87	10	2	2	High	1	1.17	0.803	1.162	1.392	1.873	1.404	1.534	1.417	0.999	0.766	1.068	1.146	1.194	1.142
TRINITY_DN2120_c0_g1_i2_orf1	cullin-4A [Ostrinia furnacalis]	2	789	91.1	8.73	2	2	2	High	1	0.95	0.972	0.804	0.639	0.791	0.892	0.754	0.928	0.743	0.733	0.77	0.736	0.676	0.682
TRINITY_DN16868_c0_g2_i1_orf1	gamma-glutamylcyclotransferase-like isoform X1 [Ostrinia furnacalis]	1	179	20.7	7.74	7	1	1	High	1	0.885	0.953	0.968	0.889	0.955	1.255	1.321	1.213	1.198	1.339	1.307	1.3	1.274	1.25
TRINITY_DN3355_c0_g1_i1_orf1	UDP-glucuronosyltransferase 2B2-like [Ostrinia furnacalis]	2	523	60.2	8.81	4	2	1	High	1	1.073	0.971	0.515	0.552	0.327	0.548	0.585	0.584	0.587	0.831	0.623	0.507	0.652	0.56
TRINITY_DN19493_c0_g1_i5_orf1	zinc finger MYM-type protein 3 isoform X1 [Ostrinia furnacalis]	2	1281	142.6	4.79	1	2	2	High	1	1.048	1.043	0.591	0.497	0.725	0.629	0.646	0.594	0.544	0.451	0.516	0.937	1.051	0.912
TRINITY_DN81248_c0_g1_i1_orf1	39S ribosomal protein L12, mitochondrial [Ostrinia furnacalis]	1	181	19.8	8.03	7	2	1	High	1	1.057	1.015	0.411	0.512	0.537	0.487	0.544	0.532	0.47	0.915	0.512	0.56	0.504	0.568
TRINITY_DN11069_c0_g2_i1_orf1	fat storage-inducing transmembrane protein [Ostrinia furnacalis]	2	363	42.2	9.25	6	2	2	High	1	0.965	1.099	0.319	0.337	0.32	0.307	0.313	0.302	0.211	0.205	0.255	0.371	0.313	0.311
TRINITY_DN17437_c0_g1_i1_orf1	phospholipase A1 VesT1.02-like [Ostrinia furnacalis]	2	337	37.8	7.62	6	2	2	High	1	0.878	1.015	1.039	0.994	1.018	1.161	1.14	1.324	1.159	0.952	0.994	1.582	1.476	1.54
TRINITY_DN1091_c0_g1_i1_orf1	macrophage mannose receptor 1-like [Pararge aegeria]	2	304	33.2	5.74	6	3	1	High	1	0.861	0.729	1.811	1.993	1.644	1.001	1.247	1.413	1.108	1.305	1.089	2.308	2.564	2.252
TRINITY_DN15865_c0_g2_i2_orf1	carboxylesterase [Cnaphalocrocis medinalis]	2	234	26.8	7.03	10	2	2	High	1	1.11	1.358	1.273	1.142	1.289	1.506	1.325	1.448	1.354	1.395	1.411	1.985	2.061	1.989
TRINITY_DN4895_c0_g1_i2_orf1	coiled-coil domain-containing protein 86 [Ostrinia furnacalis]	1	166	19.7	10.13	8	1	1	High	1	1.017	1	0.374	0.424	0.355	0.348	0.405	0.393	0.358	0.394	0.331	0.321	0.267	0.34
TRINITY_DN9002_c0_g1_i1_orf1	membrane magnesium transporter 1 [Ostrinia furnacalis]	1	117	13.1	8.56	8	1	1	High	1	0.943	1.151	0.617	0.51	0.567	0.401	0.459	0.535	0.38	0.403	0.427	0.466	0.471	0.447
TRINITY_DN2170_c0_g2_i1_orf1	beta-1,3-glucan-binding protein-like [Ostrinia furnacalis]	1	169	19.1	6.8	8	2	1	High	1	1.003	0.977	1.453	1.492	1.403	1.589	1.674	1.297	2.04	1.982	1.951	1.633	1.458	1.369
TRINITY_DN6503_c0_g1_i8_orf1	uncharacterized protein LOC114354432 [Ostrinia furnacalis]	1	944	108.9	6.16	2	1	1	High	1	1.208	1.12	0.861	0.884	0.838	0.746	0.693	0.648	0.696	0.729	0.797	0.766	0.789	0.789
TRINITY_DN94475_c0_g1_i1_orf1	mannosyl-oligosaccharide alpha-1,2-mannosidase IA-like isoform X2 [Ostrinia	2	95	10.8	10.15	26	3	1	High	1	0.885	0.999	0.828	0.822	0.69	0.778	0.949	0.978	0.73	1.039	0.792	0.775	0.722	0.767
TRINITY_DN56430_c0_g1_i1_orf1	unnamed protein product, partial [Iphiclides podalirius]	1	68	8	8.57	21	2	1	High	1	0.949	1.051	1.3	1.552	1.279	1.111	1.282	1.175	0.955	0.949	1.293	1.665	1.71	1.784
TRINITY_DN6660_c0_g1_i5_orf1	pre-mRNA-splicing factor SYF1 [Ostrinia furnacalis]	2	848	99.3	5.99	3	2	2	High	1	1.006	0.941	0.738	0.658	0.7	0.782	0.704	0.814	0.685	1.307	0.669	0.735	0.715	0.658
TRINITY_DN108819_c0_g1_i1_orf1	NADH dehydrogenase [ubiquinone] 1 beta subcomplex subunit 8, mitochondr	2	173	20	8.32	15	2	2	High	1	1.032	0.974	0.565	0.601	0.597	0.561	0.551	0.584	0.56	0.494	0.488	0.608	0.626	0.591
TRINITY_DN6262_c0_g1_i2_orf1	ADAMTS-like protein 4 isoform X2 [Ostrinia furnacalis]	2	632	68.6	8.35	3	2	2	High	1	1.061	1.051	0.943	1.062	0.86	0.782	0.867	0.876	0.84	0.717	0.869	1.052	0.909	0.893
TRINITY_DN51252_c0_g2_i1_orf1	peroxidase-like [Ostrinia furnacalis]	2	166	18.9	8.31	13	3	1	High	1	1.017	1.035	0.446	0.275	0.404	0.526	0.439	0.494	0.41	0.451	0.439	0.592	0.747	0.533
TRINITY_DN37218_c0_g1_i12_orf1	protein white [Ostrinia furnacalis]	2	689	75.8	8.1	4	3	2	High	1	0.871	0.91	0.669	0.637	0.547	0.627	0.731	0.589	0.601	0.59	0.624	0.68	0.657	0.697
TRINITY_DN2971_c0_g1_i1_orf1	uncharacterized protein LOC114364864 [Ostrinia furnacalis]	1	988	110.7	4.98	1	1	1	High	1	1.02	1.172	0.91	0.811	0.867	0.99	1.06	0.959	1.098	1.056	1.12	1.37	1.459	1.444
TRINITY_DN1703_c0_g1_i6_orf1	leucine-rich repeat-containing protein 15-like [Ostrinia furnacalis]	2	419	47.7	6.2	4	2	2	High	1	0.882	1.053	1.151	1.235	1.134	1.362	1.294	1.246	1.352	1.131	1.47	2.408	2.186	2.123
TRINITY_DN37699_c0_g1_i3_orfp1	TRINITY_DN37699_c0_g1_i3_m.58788 TRINITY_DN37699_c0_g1::TRINITY_DN37	1	121	12.6	8.72	12	1	1	High	1	1.333	1.215	0.406	0.446	0.437	0.181	0.207	0.296	0.209	0.219	0.202	0.114	0.156	0.168
TRINITY_DN36494_c0_g1_i1_orf1	MKI67 FHA domain-interacting nucleolar phosphoprotein-like [Ostrinia furnac	2	263	30.6	9.88	6	2	2	High	1	1.024	0.981	0.275	0.299	0.354	0.265	0.243	0.265	0.231	0.214	0.22	0.193	0.222	0.193
TRINITY_DN5828_c0_g1_i5_orf1	unnamed protein product [Diatraea saccharalis]	1	341	36.7	8.46	4	1	1	High	1	0.894	1.026	0.791	0.877	0.913	0.795	0.777	0.747	0.852	0.993	0.85	0.804	0.825	0.916
TRINITY_DN92_c1_g2_i1_orf1	SID1 transmembrane family member 1-like isoform X3 [Ostrinia furnacalis]	2	647	73	5.81	4	2	2	High	1	0.99	0.814	1.486	1.749	1.602	1.593	1.556	1.386	1.417	1.281	1.596	1.555	1.638	1.628
TRINITY_DN50743_c0_g1_i1_orf1	cytochrome P450 monooxygenase CYP321F7 [Ostrinia furnacalis]	2	497	57.6	8.65	4	2	2	High	1	0.938	0.974	1.072	1.012	1.163	1.398	1.527	1.556	1.303	1.55	1.466	0.866	1.206	0.878
TRINITY_DN86127_c0_g1_i6_orf1	uncharacterized protein LOC114361588 isoform X14 [Ostrinia furnacalis]	1	156	17.5	6.57	10	1	1	High	1	0.963	0.824	0.973	0.972	1.019	0.874	0.463	0.632	0.797	0.65	1.022	1.005	0.946	0.776
TRINITY_DN16145_c0_g1_i12_orf1	Down syndrome cell adhesion molecule-like protein Dscam2 isoform X16 [Ost	2	234	25.8	5.08	9	2	2	High	1	1.141	0.983	1.1	1.049	0.971	1.053	1.151	1.3	1.041	1.499	1.022	1.553	1.421	1.459
TRINITY_DN3131_c0_g1_i5_orf1	senecionine N-oxygenase-like isoform X1 [Ostrinia furnacalis]	2	454	52.1	6.33	5	2	2	High	1	1.042	1.087	0.959	0.735	0.803	0.91	0.989	0.958	1.188	1.099	1.149	1.19	1.265	1.213
TRINITY_DN13747_c1_g2_i8_orf1	UPF0518 protein GH13096 isoform X1 [Ostrinia furnacalis]	1	904	101.5	5.94	1	1	1	High	1	1.021	1.124	0.891	0.86	0.873	0.782	0.795	1.02	0.874	0.986	0.925	0.808	0.816	1.094
TRINITY_DN9044_c0_g1_i1_orf1	hypothetical protein SFRURICE_005818, partial [Spodoptera frugiperda]	1	118	13.6	5.29	13	1	1	High	1	0.89	1.091	1.13	0.98	1.31	1.542	1.595	1.538	1.488	1.547	1.439	1.286	1.263	1.204
TRINITY_DN81312_c0_g1_i1_orf1	atlastin isoform X4 [Ostrinia furnacalis]	1	56	6.1	6.51	21	1	1	High	1	0.97	1.064	0.808	0.809	0.792	0.716	0.766	0.711	0.61	0.52	0.594	0.546	0.701	0.618
TRINITY_DN14365_c0_g1_i2_orf1	leucine-rich repeat-containing protein 57-like [Colias croceus]	1	238	27.1	7.49	6	1	1	High	1	0.794	0.741	0.609	0.655	0.557	0.729	0.747	0.654	0.547	0.679	0.666	0.805	0.763	0.798
TRINITY_DN32306_c0_g1_i3_orf1	acetyl-coenzyme A transporter 1 [Ostrinia furnacalis]	1	517	57	7.77	4	1	1	High	1	0.936	0.904	0.577	0.56	0.619	0.492	0.471	0.557	0.282	0.361	0.475	0.569	0.606	0.495
TRINITY_DN49942_c0_g1_i2_orf1	uncharacterized protein LOC114356866 isoform X3 [Ostrinia furnacalis]	1	66	7.2	8.53	30	1	1	High	1	1.028	0.958	0.703	0.523	0.651	0.803	0.557	0.861	1.093	0.773	0.4	0.569	0.558	0.431
TRINITY_DN16316_c0_g1_i7_orf1	exocyst complex component 6 isoform X1 [Ostrinia furnacalis]	1	761	85.9	5.87	2	1	1	High	1	0.967	1.125	1.782	1.604	1.344	1.024	1.278	1.173	1.977	1.437	1.267	1.666	1.69	1.395
TRINITY_DN14107_c0_g1_i4_orf1	bifunctional methylenetetrahydrofolate dehydrogenase/cyclohydrolase, mitocl	2	334	36.5	6.73	5	2	2	High	1	0.896	0.998	0.448	0.468	0.488	0.475	0.573	0.457	0.561	0.584	0.476	0.531	0.544	0.62
TRINITY_DN172_c8_g2_i1_orf1	sialin [Ostrinia furnacalis]	2	503	54.5	6.44	4	2	2	High	1	0.938	1.122	1.166	0.969	1.05	0.839	0.701	0.874	0.916	0.661	0.844	1.313	1.251	1.364
TRINITY_DN4003_c0_g1_i2_orf1	hypothetical protein evm_007498 [Chilo suppressalis]	2	630	70.1	6.15	2	2	2	High	1	0.962	0.806	0.72	0.666	0.868	0.939	0.915	0.82	0.753	0.697	0.852	0.565	0.752	0.754
TRINITY_DN43637_c0_g1_i1_orf1	sarcolemmal membrane-associated protein [Ostrinia furnacalis]	1	799	89.4	5.22	2	1	1	High	1	1.013	1.021	0.936	0.743	0.967	0.897	0.895	0.895	0.699	0.821	0.929	1.119	0.862	1.003
TRINITY_DN7749_c1_g1_i2_orf1	uncharacterized protein LOC114364356 isoform X1 [Ostrinia furnacalis]	2	569	62.1	7.74	4	2	2	High	1	0.885	0.93	1.221	1.1	1.208	0.989	1.154	1.116	0.945	1.078	1.158	1.28	1.102	1.351
TRINITY_DN38424_c0_g1_i1_orf1	glucose dehydrogenase [FAD, quinone]-like [Ostrinia furnacalis]	1	597	66.3	6.84	2	1	1	High	1	1.004	1.081	0.952	0.806	0.761	0.628	0.611	0.58	0.487	0.453	0.264	0.954	1.077	0.847
TRINITY_DN15836_c0_g1_i1_orf1	DNA-directed RNA polymerase I subunit RPA49-like [Ostrinia furnacalis]	1	330	37.7	9.26	4	1	1	High	1	0.938	1.054	0.65	0.76	0.72	0.621	0.76	0.671	0.563	0.925	0.715	0.829	0.91	0.746
TRINITY_DN29166_c0_g1_i1_orf1	THO complex subunit 6 [Ostrinia furnacalis]	2	325	35.9	6.34	6	2	2	High	1	1.078	0.994	1.05	1.233	0.945	1.001	0.895	0.998	0.884	0.975	1.096	1.177	0.887	0.869
TRINITY_DN657_c0_g1_i2_orf1	cytochrome c-type heme lyase [Ostrinia furnacalis]	2	274	31.6	6.73	7	2	2	High	1	1.02	1.015	0.269	0.255	0.261	0.325	0.315	0.3	0.238	0.238	0.196	0.228	0.239	0.238
TRINITY_DN125565_c1_g1_i1_orf1	uncharacterized protein LOC114352340 isoform X2 [Ostrinia furnacalis]	2	94	10.7	4.7	31	2	1	High	1	0.846	0.845	1.762	1.678	1.385	1.82	1.919	1.55	1.562	3.936	2.038	1.588	1.149	1.577
TRINITY_DN5813_c0_g1_i9_orf1	alpha-(1,6)-fucosyltransferase [Ostrinia furnacalis]	2	580	67	8.29	3	2	2	High	1	0.969	0.985	1.543	1.658	1.593	1.407	1.488	1.329	1.41	1.39	1.491	1.375	1.304	1.323
TRINITY_DN1528_c0_g1_i4_orf1	uncharacterized protein LOC114353202 [Ostrinia furnacalis]	2	407	46	5.72	5	2	2	High	1	0.814	0.969	0.725	0.736	0.812	0.645	0.804	0.97	0.628	0.808	0.618	1.008	0.937	1.158
TRINITY_DN61744_c0_g1_i1_orf1	diphosphoinositol polyphosphate phosphohydrolase 2 [Spodoptera litura]	1	156	17.9	6.93	7	1	1	High	1	1.011	1.028	0.869	0.966	0.878	0.735	0.801	0.73	0.779	0.816	0.954	0.952	0.883	0.869
TRINITY_DN39509_c0_g1_i1_orf1	Membrane alanyl aminopeptidase [Papilio machaon]	1	87	9.7	5.36	22	1	1	High	1	0.864	0.886	0.884	0.912	0.848	0.793	1.08	0.761	0.957	2.427	1.09	0.716	0.705	0.819
TRINITY_DN1375_c0_g1_i5_orf1	glutaminase [Chilo suppressalis]	2	639	71.8	7.34	3	2	2	High	1	0.942	1.036	0.504	0.487	0.476	0.493	0.529	0.645	0.629	0.477	0.493	0.591	0.498	0.399
TRINITY_DN1448_c0_g1_i3_orf1	RNA polymerase II-associated protein 1 [Ostrinia furnacalis]	2	1216	138.9	5.81	2	2	2	High	1	1.087	0.965	1.05	0.984	1.083	1.016	1.076	1.041	1.176	1.166	1.055	1.022	1.019	1.058
TRINITY_DN5065_c0_g1_i1_orf1	protein FAM8A1 [Ostrinia furnacalis]	1	309	35.2	5.94	5	1	1	High	1	0.986	1.237	0.884	0.948	0.973	0.909	0.898	1.173	0.958	0.997	0.859	0.974	0.965	1.051
TRINITY_DN27087_c0_g1_i1_orf1	2',5'-phosphodiesterase 12 [Ostrinia furnacalis]	2	590	67.3	7.02	3	2	2	High	1	0.972	0.925	0.482	0.457	0.502	0.513	0.515	0.549	0.41	0.406	0.381	0.45	0.431	0.407
TRINITY_DN62184_c1_g1_i1_orf1	uncharacterized protein LOC114362189 [Ostrinia furnacalis]	1	1105	123.3	7.87	2	1	1	High	1	1.107	1.17	1.498	1.296	1.199	1.604	1.77	1.536	2.14	1.447	1.678	1.789	1.436	1.546
TRINITY_DN41179_c0_g1_i1_orf1	RNA-binding protein NOB1 [Ostrinia furnacalis]	1	423	47.8	5.85	4	1	1	High	1	1.016	0.988	0.535	0.452	0.543	0.618	0.611	0.632	0.564	0.569	0.64	0.638	0.613	0.652
TRINITY_DN146236_c0_g1_i1_orf1	vesicle-fusing ATPase 1-like [Chelonus insularis]	1	741	81.8	7.24	2	1	1	High	1	1.037	1.036	1.162	1.01	1.142	0.933	0.924	0.87	0.824	0.831	0.812	0.646	0.807	0.704
TRINITY_DN959_c0_g1_i7_orf1	Golgi to ER traffic protein 4 homolog [Ostrinia furnacalis]	2	317	36.5	7.9	5	2	2	High	1	0.885	0.946	0.509	0.463	0.468	0.485	0.512	0.532	0.547	0.561	0.552	0.71	0.567	0.619
TRINITY_DN49527_c0_g1_i1_orf1	Protein lin-7 homolog B [Eumeta japonica]	2	198	21.8	8.34	11	2	2	High	1	1.041	1.1	0.696	0.707	0.772	0.707	0.671	0.73	0.727	0.69	0.662	0.718	0.661	0.767
TRINITY_DN7234_c0_g1_i10_orf1	GPI transamidase component PIG-S isoform X2 [Helicoverpa armigera]	2	520	58.4	5.94	3	2	2	High	1	0.929	1.277	1.124	1.341	1.147	1.158	0.923	1.065	1.085	0.949	1.08	1.266	1.25	1.054
TRINITY_DN2676_c0_g1_i2_orf1	probable cytochrome P450 303a1 [Ostrinia furnacalis]	2	498	57.1	8	5	2	2	High	1	0.909	0.964	0.971	0.907	0.961	0.904	0.914	0.868	1.016	1.262	1.342	1.426	1.323	1.364
TRINITY_DN25234_c0_g1_i1_orf1	uncharacterized protein LOC114353853 [Ostrinia furnacalis]	1	87	10	7.12	15	1	1	High	1	0.705	0.91	4.515	4.122	4.739	1.88	1.954	2.216	2.661	2.883	2.495	2.686	2.631	2.636
TRINITY_DN2167_c0_g1_i6_orf1	retinol dehydrogenase 13-like [Ostrinia furnacalis]	1	330	36.7	8.48	4	1	1	High	1	0.927	1.042	0.543	0.496	0.569	0.589	0.504	0.581	0.606	0.507	0.554	0.511	0.49	0.522
TRINITY_DN12442_c0_g1_i4_orf1	midasin-like [Ostrinia furnacalis]	2	2376	270.7	4.93	1	2	2	High	1	0.973	1.012	0.377	0.446	0.439	0.481	0.536	0.859	0.444	0.366	0.37	0.468	0.513	0.395
TRINITY_DN119291_c0_g1_i1_orf1	cyclophilin A, partial [Cotesia chilonis]	1	44	4.5	8.21	20	1	1	High	1	0.915	0.858	0.897	0.797	0.981	1.286	1.263	1.187	1.334	1.03	0.995	0.961	1.285	0.939
TRINITY_DN77572_c0_g1_i1_orf1	steroid receptor RNA activator 1 [Ostrinia furnacalis]	2	203	22.5	6.8	11	3	2	High	1	0.879	0.862	0.854	0.937	0.851	0.838	0.87	0.824	0.682	0.669	0.656	0.874	0.757	0.784
TRINITY_DN5531_c0_g3_i3_orf1	hypothetical protein evm_013868 [Chilo suppressalis]	2	2163	240.3	5.71	1	2	2	High	1	1.059	1.057	1.101	1.062	0.932	1.17	1.073	1.189	1.083	1.067	1.181	0.652	0.751	0.638
TRINITY_DN62729_c0_g1_i13_orf1	G protein-coupled receptor kinase 2 isoform X3 [Manduca sexta]	1	600	67.4	8.21	3	1	1	High	1	0.87	0.961	0.876	1.137	1.029	1.018	1.022	1.081	0.824	0.858	0.811	1.126	1.054	1.234
TRINITY_DN7493_c0_g1_i1_orf1	cofilin-1 [Homo sapiens]	1	166	18.5	8.09	10	1	1	High	1	1.167	1.205	1.353	2.109	1.664	2.14	1.768	1.809	1.958	2.174	4.47	1.777	1.579	1.899

TRINITY_DN25686_c0_g1_i4_orf1	exocyst complex component 3 [Ostrinia furnacalis]	2	749	86.4	6.21	3	2	2	High	1	0.908	1.05	0.776	0.824	0.827	0.777	0.851	0.849	0.819	0.852	0.765	0.948	1.066	0.968
TRINITY_DN10231_c0_g2_i1_orf1	uncharacterized protein LOC114361472 [Ostrinia furnacalis]	1	70	7.7	4.75	14	1	1	High	1	0.858	0.84	1.633	1.247	1.67	2.012	2.152	1.757	1.064	1.205	0.923	2.176	2.292	2.495
TRINITY_DN96566_c0_g1_i1_orf1	NADH-ubiquinone oxidoreductase subunit 8-like [Ostrinia furnacalis]	1	251	28.6	6.9	4	1	1	High	1	0.906	0.88	0.286	0.202	0.386	0.381	0.329	0.369	0.45	0.395	0.302	0.282	0.311	0.299
TRINITY_DN4732_c0_g1_i2_orf1	reversion-inducing cysteine-rich protein with Kazal motifs [Ostrinia furnacalis]	1	947	104.1	7.47	1	1	1	High	1	0.987	0.969	0.583	0.686	0.799	0.842	0.994	1.137	0.837	0.856	0.707	1.076	1.053	0.945
TRINITY_DN12673_c3_g1_i2_orf1	unnamed protein product [Chilo suppressalis]	2	415	46.5	6.39	5	2	2	High	1	0.976	0.94	1.258	1.32	1.248	1.062	0.954	1.094	1.146	0.959	1.135	1.986	1.815	1.942
TRINITY_DN12424_c0_g1_i2_orf1	uncharacterized protein LOC114350608 [Ostrinia furnacalis]	2	1159	137.1	7.94	2	2	2	High	1	0.885	1.028	1.465	1.513	1.535	1.561	1.458	1.373	1.314	1.308	1.361	1.124	1.323	1.299
TRINITY_DN7329_c0_g1_i6_orf1	serine hydrolase-like protein 2 isoform X2 [Ostrinia furnacalis]	2	294	33.6	8.84	7	2	2	High	1	1.037	0.971	0.504	0.549	0.663	0.801	0.8	0.777	0.559	0.705	0.622	0.615	0.605	0.65
TRINITY_DN10030_c0_g1_i2_orf1	uncharacterized protein LOC114360702 [Ostrinia furnacalis]	2	210	23.5	9.54	11	3	2	High	1	0.995	0.999	0.568	0.599	0.574	0.549	0.629	0.641	0.525	0.668	0.56	0.625	0.64	0.611
TRINITY_DN3821_c1_g1_i7_orf1	mitochondrial carrier protein Rim2 isoform X1 [Ostrinia furnacalis]	1	365	40.2	9.64	4	1	1	High	1	0.936	1.083	2.564	2.466	2.491	3.291	2.876	2.77	3.03	2.954	3.588	1.955	1.82	1.83
TRINITY_DN59028_c0_g1_i1_orf1	15-hydroxyprostaglandin dehydrogenase [NAD(+)]-like [Ostrinia furnacalis]	1	273	29.5	6.77	5	1	1	High	1	1.037	0.969	1.27	1.55	1.436	1.755	1.509	1.713	1.553	1.65	1.892	1.616	1.63	1.541
TRINITY_DN4360_c0_g1_i4_orf1	glucose-6-phosphate isomerase-like, partial [Bicyclus anynana]	1	45	4.9	4.72	60	2	1	High	1	1.537	1.43	2.772	2.941	2.49	1.931	0.688	1.312	2.273	1.999	3.044	2.107	1.545	1.067
TRINITY_DN43942_c0_g2_i1_orf1	LOW QUALITY PROTEIN: caprin homolog [Ostrinia furnacalis]	1	206	22.7	9.51	5	1	1	High	1	1.077	1.019	0.198	0.191	0.265	0.242	0.224	0.204	0.126	0.197	0.154	0.402	0.419	0.348
TRINITY_DN344_c0_g1_i1_orf1	chymotrypsin-like serine protease 16 [Ostrinia nubilalis]	1	268	28.1	7.46	4	2	1	High	1	0.988	1.011	0.593	0.565	0.624	0.53	0.646	0.635	0.632	0.547	0.544	0.588	0.548	0.646
TRINITY_DN14677_c0_g2_i3_orf1	AP-3 complex subunit beta-2 [Ostrinia furnacalis]	2	398	42.3	5.06	4	2	2	High	1	0.998	0.971	0.955	1.051	1.021	0.769	0.85	0.806	0.777	0.66	0.711	1.1	1.111	1.074
TRINITY_DN131662_c0_g1_i4_orf1	splicing factor 3A subunit 1 isoform X1 [Ostrinia furnacalis]	1	176	18.9	9.31	8	1	1	High	1	1.273	1.041	0.863	0.811	0.761	0.652	0.752	0.754	0.758	0.644	0.7	0.916	0.714	0.866
TRINITY_DN41697_c0_g1_i1_orf1	5-formyltetrahydrofolate cyclo-ligase [Ostrinia furnacalis]	2	39	4.5	9.88	38	2	1	High	1	0.809	0.867	0.274	0.342	0.29	0.221	0.175	0.225	0.486	0.506	0.607	0.563	0.557	0.592
TRINITY_DN11118_c0_g2_i1_orf1	putative tRNA pseudouridine synthase Pus10 [Ostrinia furnacalis]	1	338	37.5	6.42	4	1	1	High	1	1.288	1.239	0.919	1.261	1.147	1.29	1.087	1.174	1.191	1.115	1.212	1.189	0.999	1.21
TRINITY_DN14611_c0_g1_i5_orf1	hsc70-interacting protein-like [Galleria mellonella]	1	117	11.5	4.56	28	2	1	High	1	0.819	1.025	1.94	2.227	2.064	1.623	1.786	1.759	1.732	1.47	1.676	1.265	1.514	1.305
TRINITY_DN25976_c0_g1_i4_orf1	hypothetical protein B566_EDAN014657 [Ephemera danica]	1	81	8.4	4.89	30	1	1	High	1	1.082	1.379	2.461	2.321	2.477	2.768	1.946	2.137	2.341	2.42	2.248	3.118	3.264	2.941
TRINITY_DN22944_c0_g3_i1_orf1	transmembrane protein 115 [Ostrinia furnacalis]	2	372	41.3	9.19	5	2	2	High	1	0.891	0.982	1.243	1.227	1.081	1.213	1.409	1.244	1.082	1.327	1.188	1.269	1.228	1.174
TRINITY_DN5829_c0_g2_i1_orf1	uncharacterized protein LOC114365758 isoform X2 [Ostrinia furnacalis]	1	142	15.1	5.25	8	2	1	High	1	0.991	0.965	0.701	0.709	0.761	0.807	0.985	0.964	0.758	0.595	0.668	1.425	1.304	1.197
TRINITY_DN112409_c1_g1_i1_orf1	uncharacterized protein LOC114360748 [Ostrinia furnacalis]	2	309	35.8	4.97	6	2	2	High	1	0.914	0.893	0.965	0.874	0.982	0.845	0.88	0.943	0.829	0.911	0.989	0.825	0.873	0.894
TRINITY_DN48602_c0_g1_i6_orf1	amidophosphoribosyltransferase-like isoform X1 [Ostrinia furnacalis]	2	96	10.4	8.92	18	2	1	High	1	1.013	1.162	1.676	1.701	1.523	1.168	1.374	1.224	1.494	1.694	1.499	1.472	1.324	1.572
TRINITY_DN5678_c0_g2_i3_orf1	coiled-coil domain-containing protein 115 [Ostrinia furnacalis]	1	156	17.6	5.99	9	1	1	High	1	1.09	0.981	0.389	0.411	0.449	0.533	0.485	0.489	0.447	0.428	0.467	0.503	0.465	0.442
TRINITY_DN19377_c0_g1_i4_orf1	uncharacterized protein LOC114353316 [Ostrinia furnacalis]	1	277	29.7	9.19	5	1	1	High	1	1.088	0.967	1.404	1.31	1.444	1.585	1.463	1.768	1.708	1.489	1.503	1.367	1.426	1.311
TRINITY_DN4514_c0_g1_i1_orf1	enoyl-CoA delta isomerase 1, mitochondrial-like isoform X1 [Ostrinia furnacali	1	281	31	8.94	6	1	1	High	1	0.935	1.018	0.671	0.767	0.613	0.441	0.435	0.402	0.553	0.512	0.635	0.345	0.36	0.364
TRINITY_DN749_c0_g1_i1_orf1	serine/threonine-protein phosphatase 4 regulatory subunit 3 isoform X3 [Ostri	2	805	91.5	5.12	2	2	1	High	1	0.882	0.935	0.652	0.749	0.817	0.747	0.765	0.771	0.706	0.749	0.704	0.97	0.918	0.93
TRINITY_DN16390_c0_g1_i4_orf1	unnamed protein product, partial [Brenthis ino]	1	879	96.5	11.56	2	1	1	High	1	0.984	0.935	0.794	0.993	0.973	0.902	0.929	1.096	0.93	0.77	0.899	1.381	1.173	1.272
TRINITY_DN48713_c0_g1_i1_orf1	sphingomyelin phosphodiesterase 4 [Ostrinia furnacalis]	1	768	88.6	7.37	2	1	1	High	1	1.063	0.94	0.623	0.799	0.683	0.69	0.862	0.783	0.919	0.861	0.841	0.817	0.864	0.925
TRINITY_DN13025_c0_g1_i8_orf1	phosphorylase b kinase gamma catalytic chain, skeletal muscle/heart isoform is	2	419	48.1	7.3	4	2	2	High	1	1.028	1.02	0.893	0.917	0.903	0.862	0.959	0.979	0.942	1.025	0.843	0.896	0.933	0.84
TRINITY_DN37729_c0_g1_i8_orf1	adenylyltransferase and sulfurtransferase MOCS3 isoform X1 [Ostrinia furnacali	1	633	71.6	8.91	2	1	1	High	1	0.822	1.023	0.546	0.666	0.891	0.773	0.732	0.547	0.739	0.579	0.556	0.721	0.701	0.746
TRINITY_DN19187_c0_g1_i1_orf1	fumarylacetoacetase [Chelonus insularis]	2	418	46.6	6.54	4	2	1	High	1	1.003	0.93	0.651	0.617	0.626	0.606	0.606	0.658	0.505	0.471	0.5	0.697	0.631	0.583
TRINITY_DN36045_c0_g1_i2_orf1	hypothetical protein evm_012355 [Chilo suppressalis]	2	192	22.7	9.55	11	2	2	High	1	0.958	0.96	0.556	0.527	0.49	0.602	0.575	0.619	0.756	0.536	0.566	0.562	0.629	0.672
TRINITY_DN2821_c0_g1_i1_orf1	uncharacterized protein LOC114356423 [Ostrinia furnacalis]	1	151	17.3	6.77	10	1	1	High	1	0.895	0.99	0.813	0.709	0.864	1.042	1.299	1.279	1.135	1.456	1.024	0.911	0.774	0.973
TRINITY_DN31348_c0_g1_i1_orf1	protein lethal(2)essential for life [Bombyx mori]	1	63	7.2	8.68	22	1	1	High	1	0.911	0.967	3.13	3.268	3.22	3.287	3.361	3.213	4.909	4.758	5.486	1.794	1.717	1.868
TRINITY_DN69049_c0_g1_i2_orf1	membrane alanyl aminopeptidase-like [Ostrinia furnacalis]	1	451	51.3	4.63	2	1	1	High	1	1.028	0.962	0.246	0.219	0.291	0.223	0.251	0.239	0.218	0.188	0.203	0.224	0.2	0.221
TRINITY_DN919_c0_g1_i7_orf1	facilitated trehalose transporter Tret1-like [Ostrinia furnacalis]	2	494	54.6	8.95	3	2	2	High	1	0.988	1.039	1.851	1.981	1.865	0.994	1.014	1.016	0.906	0.934	1.122	1.355	1.336	1.326
TRINITY_DN71465_c0_g1_i1_orf1	adenylate kinase isoenzyme 6 [Ostrinia furnacalis]	1	176	20.6	4.65	9	1	1	High	1	1.192	1.032	0.466	0.517	0.471	0.578	0.533	0.497	0.487	0.475	0.48	0.479	0.524	0.517
TRINITY_DN36496_c0_g1_i1_orf1	unnamed protein product [Parnassius apollo]	1	318	35.3	5.67	3	1	1	High	1	0.981	0.991	0.626	0.53	0.611	0.994	1.2	0.801	0.582	0.628	0.541	0.702	0.768	0.796
TRINITY_DN113778_c0_g2_i1_orf1	metastasis-associated protein MTA3 [Galleria mellonella]	1	589	65.4	8.4	2	1	1	High	1	0.898	1.123	0.914	0.847	0.843	0.808	0.765	0.669	0.616	0.703	0.555	1.169	1.089	1.053
TRINITY_DN5749_c0_g1_i4_orf1	hypothetical protein SFRURICE_000329 [Spodoptera frugiperda]	2	376	42.7	7.18	5	2	2	High	1	1.028	1.054	1.021	1.112	1.124	1.127	1.051	0.967	0.855	0.987	0.918	0.986	0.961	0.995
TRINITY_DN32780_c0_g1_i2_orf1	renin receptor [Ostrinia furnacalis]	1	339	38.1	5.92	4	2	1	High	1	1.033	1.125	0.851	0.887	0.901	0.707	0.806	0.843	1.07	1.231	1.008	1.16	1.142	1.199
TRINITY_DN6436_c0_g1_i1_orf1	serine/threonine-protein kinase PAK 3 isoform X1 [Ostrinia furnacalis]	1	582	63.7	7.08	2	1	1	High	1	0.971	0.878	0.69	0.606	0.722	0.565	0.567	0.656	0.636	0.598	0.71	1.198	1.264	1.314
TRINITY_DN47949_c0_g1_i4_orf1	high affinity copper uptake protein 1-like isoform X1 [Ostrinia furnacalis]	1	252	28.6	6.25	6	1	1	High	1	1.152	1.168	1.31	1.08	1.12	1.247	1.116	1.387	1.297	1.082	1.191	1.015	1.059	1.109
TRINITY_DN41609_c0_g1_i1_orf1	39S ribosomal protein L42, mitochondrial [Ostrinia furnacalis]	1	111	13.1	9.54	11	1	1	High	1	1.052	1.016	1.12	0.924	1.129	0.862	1.035	1.188	0.964	0.967	1.032	0.949	1.059	0.944
TRINITY_DN28922_c0_g1_i2_orf1	uncharacterized protein LOC119829283 isoform X2 [Zerene cesonia]	1	117	12.5	8.47	10	1	1	High	1	0.963	1.076	1.513	1.145	1.475	1.241	1.174	1.335	1.59	1.861	1.237	5.421	5.665	4.902
TRINITY_DN2303_c0_g1_i1_orf1	tubulin-specific chaperone cofactor E-like protein [Ostrinia furnacalis]	2	464	52.5	5.88	6	2	2	High	1	1.077	1.015	0.936	0.956	0.905	0.902	0.903	0.996	0.883	1.047	0.831	0.907	0.83	0.918
TRINITY_DN28439_c0_g1_i7_orf1	liprin-alpha-1 isoform X2 [Pectinophora gossypiella]	2	1122	125.5	6.2	2	2	2	High	1	1.074	1.036	0.86	0.992	0.764	0.886	0.942	0.946	0.876	0.857	0.915	0.996	0.927	0.877
TRINITY_DN7750_c0_g1_i1_orf1	lipoyl synthase, mitochondrial [Ostrinia furnacalis]	2	367	41.6	8.57	8	2	2	High	1	0.969	0.989	1.041	1.006	0.914	1.11	1.049	0.888	1.018	1.1	0.881	1.083	0.924	0.912
TRINITY_DN33009_c0_g1_i2_orfp1	TRINITY_DN33009_c0_g1_i2_m.55538 TRINITY_DN33009_c0_g1::TRINITY_DN33	1	154	17.5	5.96	8	2	1	High	1	0.858	0.946	0.554	0.716	0.502	0.608	0.722	0.524	0.59	0.932	1.289	0.755	0.633	0.9
TRINITY_DN7549_c0_g1_i1_orf1	uncharacterized protein LOC114355006 [Ostrinia furnacalis]	2	185	20	4.87	14	2	2	High	1	0.872	1.013	0.729	0.811	0.827	0.674	0.778	0.935	0.663	0.635	0.63	4.355	4.649	4.262
TRINITY_DN11584_c0_g1_i2_orf1	L-threonine 3-dehydrogenase, mitochondrial [Ostrinia furnacalis]	1	342	39.3	8.34	4	1	1	High	1	0.967	1.105	0.487	0.468	0.522	0.473	0.439	0.574	0.591	0.58	0.569	0.562	0.444	0.571
TRINITY_DN1099_c1_g1_i2_orf1	dehydrogenase/reductase SDR family protein 7-like [Ostrinia furnacalis]	2	317	34.8	9.52	6	2	2	High	1	1.207	1.14	1.202	1.117	1.261	1.14	0.98	1.153	0.892	0.763	1.017	1.418	1.15	1.277
TRINITY_DN18396_c0_g1_i1_orf1	uncharacterized protein LOC114359424 [Ostrinia furnacalis]	1	365	39.7	4.77	4	1	1	High	1	1.047	1.009	0.725	0.717	0.707	0.484	0.537	0.57	0.427	0.462	0.444	0.277	0.286	0.292
TRINITY_DN16939_c0_g1_i4_orf1	39S ribosomal protein L17, mitochondrial [Ostrinia furnacalis]	2	207	24	9.57	7	2	2	High	1	0.946	0.927	0.321	0.296	0.317	0.279	0.284	0.289	0.259	0.227	0.277	0.27	0.301	0.26
TRINITY_DN143509_c0_g1_i1_orf1	ATP-dependent 6-phosphofructokinase isoform X3 [Diachasma alloeum]	1	426	46.3	7.64	2	3	1	High	1	1.103	1.156	1.182	1.147	1.19	1.377	1.191	1.365	1.24	1.133	0.974	0.85	0.732	0.782
TRINITY_DN53847_c0_g1_i7_orf1	carboxylesterase [Ostrinia furnacalis]	1	290	31.5	8.06	6	1	1	High	1	1.047	0.994	0.788	1.024	0.878	1.011	0.888	0.993	0.858	0.989	1.042	1.078	1.202	1.086
TRINITY_DN41349_c0_g3_i1_orf1	blastoderm-specific protein 25D [Ostrinia furnacalis]	1	137	15.3	4.56	9	1	1	High	1	1.056	1.183	1.01	1.042	1.148	0.984	1.067	1.042	1.099	1.141	1.171	1.28	1.099	1.092
TRINITY_DN27021_c0_g1_i1_orf1	rab3 GTPase-activating protein catalytic subunit isoform X1 [Ostrinia furnacalis	1	799	89.6	5.17	1	1	1	High	1	1.069	1.263	2.659	2.838	2.265	1.386	1.201	1.521	3.311	1.443	1.4	1.345	1.261	1.285
TRINITY_DN2117_c0_g1_i1_orf1	BUB3-interacting and GLEBS motif-containing protein ZNF207 [Chelonus insul	2	485	52.5	9.64	4	2	2	High	1	0.941	0.959	0.517	0.554	0.535	0.415	0.456	0.438	0.414	0.417	0.401	0.637	0.616	0.609
TRINITY_DN30273_c1_g1_i1_orf1	uncharacterized protein LOC114358591 isoform X2 [Ostrinia furnacalis]	2	1195	135.4	9	2	2	2	High	1	1.113	1.185	1.019	1.076	1.057	0.997	0.987	0.934	0.903	0.756	0.937	1.417	1.377	1.42
TRINITY_DN111110_c0_g1_i1_orf1	NAD-dependent protein deacylase-like [Ostrinia furnacalis]	1	289	31.5	8.06	5	1	1	High	1	1.035	0.969	1.053	1.023	0.999	0.968	0.951	0.913	0.677	0.765	0.698	0.548	0.552	0.539
TRINITY_DN17394_c0_g1_i1_orf1	monocarboxylate transporter 14-like [Ostrinia furnacalis]	2	806	89.3	7.65	2	2	2	High	1	0.986	0.968	0.682	0.704	0.733	0.487	0.476	0.559	0.496	0.427	0.511	0.582	0.553	0.639
TRINITY_DN96739_c0_g1_i1_orf1	annexin A6, isoform CRA_b [Homo sapiens]	1	150	16.9	5.27	7	1	1	High	1	1.031	0.937	1.613	1.473	1.681	1.495	1.709	1.622	1.275	1.056	1.258	1.71	1.972	1.77
TRINITY_DN2738_c1_g1_i3_orf1	uridine-cytidine kinase isoform X1 [Helicoverpa zea]	1	299	34	8.07	6	1	1	High	1	1.017	1.068	0.654	0.679	0.637	0.627	0.696	0.602	0.609	0.704	0.559	0.781	0.78	0.791
TRINITY_DN61536_c0_g3_i1_orf1	cubilin homolog [Ostrinia furnacalis]	1	161	18	7.77	9	1	1	High	1	0.779	0.797	1.158	1.083	1.037	0.769	0.774	0.774	0.784	0.859	0.883	0.908	0.728	0.833
TRINITY_DN5581_c0_g1_i1_orf1	mucin-5AC-like [Ostrinia furnacalis]	2	1136	128.1	9.5	2	2	2	High	1	0.926	0.999	1.019	0.819	0.992	1.332	1.251	1.207	1.108	1.059	1.093	6.404	6.695	6.071
TRINITY_DN825_c2_g1_i5_orf1	hypothetical protein SFRURICE_018365 [Spodoptera frugiperda]	1	70	7.5	7.65	20	2	1	High	1	1.005	1.007	0.447	0.561	0.478	0.505	0.466	0.45	0.5	0.568	0.595	0.545	0.536	0.563
TRINITY_DN1753_c1_g1_i8_orf1	serine/threonine-protein kinase WNK1-like isoform X15 [Ostrinia furnacalis]	2	1818	203.2	5.94	0	3	2	High	1	0.893	1.007	0.737	0.799	0.806	0.852	1.007	0.992	0.801	0.74	0.777	0.697	0.742	0.788
TRINITY_DN5300_c0_g1_i3_orf1	valacyclovir hydrolase [Ostrinia furnacalis]	1	132	15.5	7.11	10	1	1	High	1	0.918	0.855	1.045	1.025	1.013	1.088	1.042	1.055	0.958	0.921	0.917	0.884	0.8	0.682
TRINITY_DN2710_c0_g1_i4_orf1	translin-associated protein X [Ostrinia furnacalis]	2	286	32	7.02	8	2	2	High	1	1.026	1.117	1.29	1.571	1.32	1.528	1.678	1.59	1.335	1.533	1.94	1.308	1.307	1.351
TRINITY_DN90108_c0_g1_i1_orf1	uncharacterized protein LOC114356879 [Ostrinia furnacalis]	1	287	33.6	7.44	5	1	1	High	1	1.089	0.679	0.597	0.759	0.631	0.757	1.046	0.658	0.852	1.534	1.197	0.701	0.607	0.933
TRINITY_DN23020_c0_g1_i1_orf1	actin-related protein 2/3 complex subunit 1A-A [Ostrinia furnacalis]	1	73	8.4	7.12	16	1	1	High	1	0.947	1.021	1.156	1.194	1.065	1.091	0.863	1.159	0.873	0.809	0.935	1.169	1.248	1.239
TRINITY_DN7473_c0_g1_i1_orf1	zinc finger protein 664-like [Ostrinia furnacalis]	1	344	39.4	7.96	4	1	1	High	1	1.058	1.109	0.987	1.079	0.907	0.838	0.955	0.872	0.915	0.898	1.029	1.113	1	1.096
TRINITY_DN31433_c0_g1_i1_orf1	notchless protein homolog 1 [Ostrinia furnacalis]	2	473	52.5	7.34	5	2	2	High	1	1.063	1.034	0.584	0.587	0.578	0.686	0.636	0.737	0.628	0.604	0.611	0.659	0.647	0.629
TRINITY_DN14673_c0_g1_i3_orf1	charged multivesicular body protein 1b [Hyposmocoma kahamanoa]	2	241	26.7	8.72	6	3	2	High	1	0.992	0.968	1.021	1.257	1.174	1.211	1.153	1.382	1.142	1.046	1.19	1.178	1.14	1.039
TRINITY_DN4189_c0_g1_i4_orf1	tolloid-like protein 1 [Ostrinia furnacalis]	1	327	36.2	5.2	5	1	1	High	1	0.864	0.837	0.889	0.982	0.931	0.924	1.048	0.853	0.958	1.409	1.076	1.051	1.154	1.217
TRINITY_DN10195_c0_g1_i8_orf1	charged multivesicular body protein 6-A [Ostrinia furnacalis]	1	206	23.9	5.29	7	1	1	High	1	1.008	1.078	1.421	1.292	1.468	1.441	1.385	1.34	1.482	1.456	1.703	1.491	1.391	1.304
TRINITY_DN6136_c0_g1_i1_orf1	uncharacterized protein LOC114354455 [Ostrinia furnacalis]	1	293	33.2	8.31	5	1	1	High	1	0.7	0.94	1.277	1.056	1.224	1.688	1.522	1.669	1.648	2.91	1.516	1.445	1.498	1.307
TRINITY_DN811_c0_g1_i15_orf1	uncharacterized protein LOC114364160 [Ostrinia furnacalis]	1	333	37.7	7.5	4	1	1	High	1	0.853	0.998	2.431	1.977	1.888	1.699	1.304	1.552	1.745	1.924	1.685	1.824	1.638	1.663
TRINITY_DN110888_c0_g1_i2_orf1	uncharacterized protein LOC114364502 [Ostrinia furnacalis]	1	302	33.8	5.66	4	4	1	High	1	0.94	1.014	0.343	0.358	0.321	0.376	0.37	0.391	0.45	0.761	0.468	0.322	0.308	0.347
TRINITY_DN44658_c0_g1_i2_orf1	lipase 3-like [Ostrinia furnacalis]	1	462	51	5.4	3	1	1	High	1	0.889	0.958	0.8	0.815	0.981	0.688	0.872	0.846	0.701	0.695	0.62	1.189	1.208	1.083
TRINITY_DN77005_c0_g3_i1_orf1	RNA polymerase II degradation factor 1-like [Ostrinia furnacalis]	1	388	41.8	4.68	3	1	1	High	1	0.923	0.84	0.729	0.695	0.628	0.714	0.824	0.805	0.836	0.755	0.916	1.276	1.227	1.155
TRINITY_DN5408_c0_g1_i5_orf1	uncharacterized protein LOC114359912 [Ostrinia furnacalis]	1	261	30.4	7.39	5	1	1	High	1	0.907	1.097	2.448	2.305	2.486	3.671	3.353	3.711	1.919	1.353	1.722	6.785	7.3	5.879
TRINITY_DN81803_c0_g2_i1_orf1	cathepsin K-like [Ostrinia furnacalis]	1	117	13.3	3.98	8	2	1	High	1	0.975	0.996	0.081	0.053	0.111	0.122	0.119	0.127	0.116	0.119	0.117	0.159	0.174	0.158
TRINITY_DN928_c0_g2_i1_orf1	fasciclin-2-like [Ostrinia furnacalis]	1	661	74.8	5.59	3	1	1	High	1	0.996	1.124	1.373	1.68	1.134	1.28	1.714	1.468	1.216	1.428	1.486	1.068	0.98	0.811
TRINITY_DN104139_c0_g1_i1_orf1	cytochrome c oxidase assembly factor 7 homolog isoform X2 [Ostrinia furnacal	2	250	28.4	8.25	10	2	2	High	1	0.9	0.956	0.691	0.737	0.718	0.784	0.738	0.734	0.774	0.868	0.773	0.588	0.619	0.745
TRINITY_DN15597_c0_g1_i1_orf1	microsomal glutathione S-transferase 1-like [Ostrinia furnacalis]	1	146	16.4	9.6	7	2	1	High	1	0.917	1	1.898	1.836	2.073	2.452	2.38	2.32	1.863	2.023	1.607	2.269	2.316	2.327
TRINITY_DN34703_c0_g1_i4_orf1	gamma-tubulin complex component 3 homolog [Ostrinia furnacalis]	1	835	93.4	8.53	1	1	1	High	1	1.137	0.825	0.82	0.748	0.997	0.988	0.914	0.724	0.561	0.487	0.804	1.379	1.606	1.318
TRINITY_DN22272_c0_g1_i1_orf1	28S ribosomal protein S33, mitochondrial [Galleria mellonella]	1	115	13.8	10.3	9	1	1	High	1	0.942	1.116	0.717	0.835	0.832	0.995	1.324	1.172	1.192	1.233	1.122	0.998	1.051	1.141
TRINITY_DN68477_c0_g1_i1_orf1	rap1 GTPase-GDP dissociation stimulator 1-B [Ostrinia furnacalis]	2	472	51.8	5.02	7	2	2	High	1	0.992	0.977	0.995	1.038	1.044	1.049	0.933	1.176	1.206	1.259	1.042	1.26	1.177	1.062
TRINITY_DN22951_c0_g1_i1_orf1	TAR DNA-binding protein 43-like [Ostrinia furnacalis]	2	301	33.1	7.39	6	2	2	High	1	1.048	0.981	0.733	0.678	0.768	0.767	1.101	0.691	0.556	0.649	0.623	0.781	0.804	0.833
TRINITY_DN3275_c0_g1_i4_orf1	calmodulin-like protein 4 [Ostrinia furnacalis]	1	149	16.8	5.6	9	1	1	High	1	1.182	1.064	0.946	1.049	1.091	1.088	1.363	1.16	1.334	1.604	1.488	1.367	1.231	1.757
TRINITY_DN15370_c0_g1_i4_orf1	DNA replication licensing factor Mcm5 [Spodoptera litura]	2	730	81.3	8.27	3	3	2	High	1	1.047	0.975	0.431	0.475	0.508	0.497	0.542	0.571	0.524	0.555	0.507	0.621	0.539	0.581

TRINITY_DN18366_c0_g1_i1_orf1	rabenosyn-5 isoform X1 [Ostrinia furnacalis]	1	472	53.8	7.44	3	1	1	High	1	0.994	1.052	1.051	0.971	0.783	0.798	0.975	0.922	0.91	0.83	0.984	0.808	0.888	0.974
TRINITY_DN32583_c0_g1_i4_orf1	ATP-binding cassette sub-family B member 8, mitochondrial-like [Ostrinia furr	2	696	76.2	9.03	3	2	2	High	1	1.013	0.946	0.831	0.841	0.882	0.885	0.851	0.918	0.951	1.574	0.89	0.719	0.764	0.762
TRINITY_DN112234_c0_g1_i6_orf1	putative peptidyl-prolyl cis-trans isomerase dodo [Ostrinia furnacalis]	2	48	5.4	9.54	19	2	2	High	1	0.955	0.889	0.756	0.864	0.809	0.936	1.087	0.918	0.925	0.929	1.039	0.874	0.965	0.947
TRINITY_DN1532_c0_g1_i6_orf1	mRNA (2'-O-methyladenosine-N(6)-)-methyltransferase [Ostrinia furnacalis]	1	384	44.6	8.32	3	1	1	High	1	1.05	0.71	1.22	1.489	1.227	1.16	1.637	1.462	1.453	1.969	1.632	1.42	1.247	1.673
TRINITY_DN18860_c0_g1_i1_orf1	DDB1- and CUL4-associated factor 13 [Ostrinia furnacalis]	2	447	51.5	9.55	3	2	2	High	1	0.972	0.991	0.393	0.445	0.439	0.441	0.397	0.458	0.476	0.454	0.427	0.475	0.513	0.492
TRINITY_DN1260_c0_g2_i1_orf1	vegetative cell wall protein gp1 [Ostrinia furnacalis]	1	295	31.3	6.05	5	1	1	High	1	0.944	0.958	0.842	0.791	0.823	1.091	1.314	1.142	0.725	0.841	0.96	1.149	1.124	1.221
TRINITY_DN638_c0_g2_i9_orf1	zinc finger protein 629-like isoform X1 [Ostrinia furnacalis]	2	588	67.5	9.09	3	2	1	High	1	0.892	1.192	0.713	1.057	1.013	0.903	0.919	0.955	0.775	0.649	1.007	0.943	0.763	0.869
TRINITY_DN460_c0_g1_i3_orf1	ribonuclease P protein subunit p30 isoform X2 [Ostrinia furnacalis]	1	262	29.9	8.9	6	1	1	High	1	1.043	1.133	1.025	1.268	1.179	1.442	1.035	1.221	1.638	1.604	1.006	1.652	1.526	1.304
TRINITY_DN3116_c0_g1_i2_orf1	esterase AAEL000016 [Ostrinia furnacalis]	1	243	26.8	6.16	4	1	1	High	1	1.042	1.125	1.07	1.222	1.175	1.148	1.495	1.341	1.138	1.264	1.235	1.297	1.23	1.075
TRINITY_DN12181_c0_g2_i1_orf1	85/88 kDa calcium-independent phospholipase A2 [Ostrinia furnacalis]	2	811	88.4	6.8	2	2	2	High	1	0.96	1.079	1.125	0.856	1.178	1.213	0.999	1.147	1.09	1.147	0.913	1.204	1.05	1.116
TRINITY_DN1710_c0_g1_i1_orf1	nuclear factor NF-kappa-B p105 subunit-like isoform X2 [Ostrinia furnacalis]	2	188	20.8	4.6	13	2	2	High	1	0.96	1.051	2	1.778	1.748	1.732	1.852	2.045	2.476	2.043	1.998	2.136	1.879	1.828
TRINITY_DN23474_c1_g1_i1_orf1	unnamed protein product [Chrysodeixis includens]	1	109	11.4	5.9	17	1	1	High	1	0.923	0.91	1.328	1.343	1.576	0.844	0.945	0.818	0.681	0.59	0.647	0.504	0.605	0.495
TRINITY_DN14112_c0_g1_i3_orf1	uncharacterized protein LOC114350956 [Ostrinia furnacalis]	2	419	48.5	7.43	5	2	2	High	1	1.005	1.001	1.25	1.441	1.299	1.45	1.533	1.574	1.441	1.298	1.349	1.111	0.965	1.121
TRINITY_DN8692_c0_g1_i2_orf1	caspase-1-like [Ostrinia furnacalis]	2	298	34	6.65	8	2	2	High	1	0.984	0.974	1.114	1.025	1.058	1.124	1.09	1.154	0.868	1.047	1.037	1.807	1.723	1.815
TRINITY_DN3457_c0_g1_i4_orf1	aryl hydrocarbon receptor nuclear translocator homolog [Ostrinia furnacalis]	1	535	58.6	7.09	2	1	1	High	1	1.218	0.885	0.504	0.808	0.627	0.468	0.456	0.551	0.529	0.181	0.39	0.739	0.61	0.701
TRINITY_DN4814_c0_g1_i6_orf1	vesicle transport protein GOT1B [Pectinophora gossypiella]	1	144	15.6	9.83	6	2	1	High	1	1.014	1.039	0.593	0.528	0.628	0.727	0.766	0.804	0.75	0.618	0.747	0.751	0.805	0.755
TRINITY_DN198_c2_g1_i2_orf1	solute carrier organic anion transporter family member 5A1-like isoform X1 [O	1	757	81.6	6.67	1	2	1	High	1	1.125	1.083	1.767	1.641	1.614	1.73	1.838	1.756	1.528	1.656	1.493	2.265	2.207	2.258
TRINITY_DN5593_c0_g1_i1_orf1	PREDICTED: leucine-rich repeat-containing protein 47-like [Fopius arisanus]	1	530	60.3	8.37	2	1	1	High	1	1.059	0.952	0.75	0.828	0.75	0.565	0.627	0.506	0.704	0.67	0.694	0.738	0.655	0.684
TRINITY_DN9044_c0_g1_i2_orf1	unnamed protein product [Euphydryas editha]	1	99	11.4	9.5	10	2	1	High	1	1.005	1.144	5.23	5.411	5.325	1.952	1.819	2.15	2.961	2.658	3.059	3.02	2.981	2.959
TRINITY_DN3971_c0_g1_i1_orf1	L-asparaginase-like isoform X1 [Ostrinia furnacalis]	2	375	42.2	8.07	6	2	2	High	1	1.17	1.091	0.658	0.778	0.714	0.755	0.77	0.771	0.789	0.938	0.671	0.879	0.675	0.681
TRINITY_DN15154_c0_g1_i5_orf1	motile sperm domain-containing protein 2-like [Ostrinia furnacalis]	1	103	11.9	8.43	13	1	1	High	1	1.048	1.051	0.861	0.886	0.971	0.79	0.858	0.825	0.698	0.76	0.985	1.011	0.856	0.896
TRINITY_DN20133_c0_g1_i1_orf1	fructose-bisphosphate aldolase A isoform X2 [Microcebus murinus]	1	364	39.3	8.09	4	1	1	High	1	0.957	0.973	1.89	1.992	2.152	1.966	2.117	1.691	6.202	1.32	1.386	1.59	1.365	1.209
TRINITY_DN26569_c0_g1_i4_orf1	hypothetical protein evm_009797 [Chilo suppressalis]	1	182	20.6	7.01	9	1	1	High	1	1.019	1.152	1.4	1.184	1.477	1.257	1.182	1.445	1.376	3.142	1.198	1.342	1.167	1.444
TRINITY_DN8652_c0_g1_i8_orf1	glucocorticoid-induced transcript 1 protein-like [Ostrinia furnacalis]	1	328	35.2	10.07	4	1	1	High	1	1.008	0.977	1.287	1.253	1.246	1.115	1.12	1.108	1.067	0.993	1.225	1.155	1.296	1.34
TRINITY_DN11188_c0_g1_i2_orf1	testin [Ostrinia furnacalis]	3	709	78.5	7.27	5	3	3	High	1	1.131	1.039	0.941	1.043	0.822	0.883	0.834	0.867	0.831	0.808	0.799	1.145	1.009	0.927
TRINITY_DN29017_c0_g1_i4_orf1	cysteine protease ATG4B [Ostrinia furnacalis]	2	382	43.3	4.88	7	2	2	High	1	1.005	1.061	1.243	1.494	1.354	1.249	1.464	1.397	1.504	1.551	1.44	1.353	1.402	1.605
TRINITY_DN15762_c0_g1_i2_orf1	YTH domain-containing family protein 3 isoform X3 [Maniola hyperantus]	2	316	37.3	8.53	6	2	2	High	1	1.046	1.026	0.745	0.974	0.929	0.752	0.712	0.757	0.72	0.802	0.653	1.007	0.89	0.816
TRINITY_DN1475_c0_g1_i6_orf1	uncharacterized protein LOC113226757 isoform X2 [Hyposmocoma kahamanc	2	717	82	6.95	3	2	2	High	1	1.026	1.037	1.266	1.201	1.135	1.216	1.157	1.26	1.056	1.212	1.291	0.948	1.017	0.897
TRINITY_DN14168_c0_g1_i1_orf1	transmembrane 7 superfamily member 3-like [Ostrinia furnacalis]	1	552	62.2	7.56	3	1	1	High	1	1.041	1.212	0.557	0.536	0.422	0.442	0.438	0.548	0.466	0.494	0.333	0.498	0.447	0.426
TRINITY_DN6312_c0_g1_i1_orf1	cytochrome c oxidase assembly protein COX19 [Ostrinia furnacalis]	1	91	10.5	7.8	14	1	1	High	1	1.011	1.05	0.8	0.723	0.861	0.871	0.886	0.875	0.713	0.861	0.675	1.07	1.246	1.119
TRINITY_DN2812_c0_g1_i5_orf1	myotubularin-related protein 2 [Ostrinia furnacalis]	2	625	71	8.5	3	2	2	High	1	0.955	0.96	0.792	0.675	0.857	0.691	0.728	0.752	0.825	0.775	0.691	0.964	0.929	0.889
TRINITY_DN552_c0_g1_i3_orf1	patronin isoform X9 [Ostrinia furnacalis]	1	1254	140.3	7.61	1	1	1	High	1	1.521	1.289	1.227	1.024	1.099	1.001	0.861	1.009	0.889	0.945	0.903	1.442	1.586	1.294
TRINITY_DN1757_c0_g1_i4_orf1	F-box/LRR-repeat protein 2 isoform X1 [Ostrinia furnacalis]	1	516	59.1	6.21	2	1	1	High	1	1.206	1.134	0.586	0.431	0.618	0.58	0.635	0.678	0.664	0.608	0.423	0.435	0.431	0.29
TRINITY_DN2058_c0_g1_i2_orf1	proteasomal ubiquitin receptor ADRM1 [Ostrinia furnacalis]	1	133	13.8	4.82	10	2	1	High	1	0.974	0.917	1.24	1.267	1.148	1.089	0.767	0.931	1.299	1.047	1.557	2.531	2.14	1.945
TRINITY_DN26408_c0_g1_i7_orf1	venom carboxylesterase-6-like [Ostrinia furnacalis]	2	580	64.2	5.27	2	4	1	High	1	0.916	0.991	0.224	0.199	0.237	0.13	0.203	0.252	0.169	0.242	0.115	0.234	0.184	0.16
TRINITY_DN5208_c0_g1_i7_orf1	WD repeat-containing protein 48 homolog isoform X1 [Ostrinia furnacalis]	1	651	72.5	6.99	2	1	1	High	1	0.927	0.928	0.808	0.904	0.752	0.803	0.986	0.873	0.903	1.117	0.913	1.178	1.004	1.073
TRINITY_DN43656_c0_g1_i1_orf1	GPI ethanolamine phosphate transferase 3 isoform X2 [Ostrinia furnacalis]	2	626	70	5.47	5	2	2	High	1	0.961	0.986	0.718	0.679	0.647	0.612	0.579	0.667	0.627	0.59	0.681	0.563	0.64	0.617
TRINITY_DN8973_c0_g1_i3_orf1	pancreatic triacylglycerol lipase-like [Vanessa tameamea]	2	324	35.2	8.57	9	2	2	High	1	0.937	0.988	0.871	1.102	0.999	0.864	1.225	0.841	1.007	0.972	1.075	1.029	1.403	1.317
TRINITY_DN106730_c0_g1_i1_orf1	Photosystem I reaction center subunit II, chloroplastic, partial [Trichinella zimba	2	209	22.6	9.73	11	2	2	High	1	1.039	1.006	0.317	0.328	0.422	0.441	0.445	0.531	0.412	0.355	0.439	0.366	0.41	0.334
TRINITY_DN1292_c0_g1_i3_orf1	uncharacterized protein LOC114360660 [Ostrinia furnacalis]	1	132	14.2	4.67	11	1	1	High	1	0.969	1.092	0.776	0.877	0.862	1.435	1.479	1.403	0.96	0.973	1.242	0.688	0.692	0.644
TRINITY_DN27500_c0_g1_i4_orf1	hemicentin-1-like [Ostrinia furnacalis]	1	507	57.4	4.78	2	1	1	High	1	0.991	1.093	1.434	1.564	1.564	0.998	0.994	0.951	0.574	0.559	0.74	0.266	0.297	0.302
TRINITY_DN143628_c0_g1_i1_orf1	probable elongator complex protein 3 [Diachasma alloeum]	1	515	58.9	7.68	3	1	1	High	1	0.893	0.979	0.929	1.032	1.007	0.861	1.012	1.141	1.067	1.01	1.109	1.131	1.07	0.994
TRINITY_DN7739_c0_g1_i2_orf1	erlin-2-like [Ostrinia furnacalis]	1	338	38.2	6.65	4	1	1	High	1	1.025	1.148	1.387	1.328	1.39	1.38	1.311	1.268	1.311	1.225	1.245	1.431	1.428	1.49
TRINITY_DN13783_c0_g4_i2_orf1	hypothetical protein evm_010131 [Chilo suppressalis]	1	97	11.2	8.81	10	1	1	High	1	1.018	0.955	0.293	0.277	0.288	0.323	0.309	0.328	0.268	0.231	0.256	0.271	0.297	0.281
TRINITY_DN29026_c0_g1_i4_orf1	TIL [Ostrinia furnacalis]	1	91	9.9	6.47	14	1	1	High	1	1.003	1.396	2.456	3.106	2.903	4.572	5.979	4.626	3.107	3.441	3.078	2.129	2.506	2.441
TRINITY_DN32532_c0_g1_i1_orf1	fatty acyl-CoA hydrolase precursor, medium chain [Ostrinia furnacalis]	2	79	8.6	5.36	33	3	2	High	1	1.001	1.12	2.123	2.028	2.137	1.837	1.967	2.138	2.411	2.054	2.214	2.659	2.64	2.883
TRINITY_DN4004_c0_g1_i1_orf1	protein FAM114A2 isoform X1 [Ostrinia furnacalis]	1	569	62.7	4.72	2	1	1	High	1	0.793	0.948	0.883	1.182	0.918	1.442	1.405	1.548	1.272	1.295	1.465	0.779	0.813	0.892
TRINITY_DN95558_c0_g3_i1_orf1	cytochrome P450 monooxygenase CYP9G19 [Cnaphalocrocis medinalis]	1	69	8	8.88	16	1	1	High	1	1.01	1.128	2.653	2.637	2.298	2.761	2.468	2.86	2.295	2.011	2.207	2.475	2.305	2.397
TRINITY_DN57475_c0_g1_i1_orf1	myotubularin-related protein 8 isoform X2 [Pectinophora gossypiella]	2	398	45.6	7.75	5	2	2	High	1	0.996	1.113	0.923	0.975	1.004	0.884	0.94	0.901	0.934	1.023	0.825	1.013	0.896	0.859
TRINITY_DN10722_c0_g3_i1_orf1	inositol-3-phosphate synthase [Ostrinia furnacalis]	1	498	55.4	6.76	2	1	1	High	1	0.919	0.928	0.597	0.689	0.62	0.509	0.528	0.596	0.39	0.393	0.618	0.818	0.724	0.779
TRINITY_DN32601_c0_g1_i2_orf1	uncharacterized protein LOC114363197 [Ostrinia furnacalis]	1	424	48.2	6.24	3	1	1	High	1	1.042	1.008	0.358	0.346	0.452	0.47	0.382	0.372	0.364	0.259	0.393	1.138	1.165	1.172
TRINITY_DN31327_c0_g2_i1_orf1	multidrug resistance protein 1A isoform X1 [Ostrinia furnacalis]	2	1260	137.8	6.7	2	2	2	High	1	1.11	1.127	0.562	0.552	0.57	0.614	0.617	0.524	0.417	0.442	0.494	0.579	0.541	0.551
TRINITY_DN7556_c0_g1_i3_orf1	jg27436 [Pararge aegeria aegeria]	1	75	8.1	6.58	28	1	1	High	1	1.083	0.966	1.244	1.139	1.35	1.358	0.961	1.181	1.432	1.68	1.456	1.317	1.067	1.201
TRINITY_DN37418_c0_g1_i4_orf1	NADH dehydrogenase [ubiquinone] 1 alpha subcomplex assembly factor 3 iso	1	187	20.8	6.57	6	1	1	High	1	1.013	0.777	1.159	1.187	1.214	0.917	0.921	0.992	1.031	1	1.197	0.893	0.926	1.037
TRINITY_DN5811_c0_g1_i4_orf1	uridine-cytidine kinase-like 1 isoform X1 [Ostrinia furnacalis]	2	541	61.1	7.15	5	2	2	High	1	1.021	1.054	0.687	0.663	0.767	0.834	0.93	0.913	0.888	0.801	1.006	1.119	1.087	0.884
TRINITY_DN28844_c0_g1_i10_orf1	uncharacterized protein LOC114361502 [Ostrinia furnacalis]	1	127	13.4	7.44	11	1	1	High	1	0.933	0.949	1.082	1.217	1.153	1.177	1.135	1.081	1.166	1.035	1.251	1.137	1.079	1.034
TRINITY_DN35002_c0_g2_i2_orf1	sulfite oxidase, mitochondrial isoform X3 [Leguminivora glycinivorella]	1	67	7	6.51	16	1	1	High	1	0.948	1.023	1.336	1.286	1.307	0.839	0.814	0.827	0.789	0.8	0.907	0.963	0.781	0.994
TRINITY_DN26805_c0_g2_i3_orf1	phosphopantothenate--cysteine ligase isoform X1 [Ostrinia furnacalis]	2	322	35.7	6.93	5	2	2	High	1	0.967	0.934	1.115	1.097	1.154	1.159	1.271	1.262	1.234	1.267	1.188	1.374	1.238	1.229
TRINITY_DN15930_c0_g1_i5_orf1	ubiquitin-like modifier-activating enzyme ATG7 [Ostrinia furnacalis]	2	686	75.8	6.15	3	2	2	High	1	1.088	1.108	1.179	1.03	0.928	1.188	1.462	1.199	1.23	0.852	1.112	1.266	1.363	1.297
TRINITY_DN29229_c0_g1_i5_orfp1	TRINITY_DN29229_c0_g1_i5_m.11187 TRINITY_DN29229_c0_g1::TRINITY_DN29	1	132	14.2	4.7	10	1	1	High	1	0.867	0.905	0.373	0.495	0.42	0.423	0.585	0.423	0.387	0.31	0.356	0.328	0.413	0.201
TRINITY_DN23746_c0_g1_i2_orf1	protein 4.1 homolog isoform X1 [Ostrinia furnacalis]	2	343	39.5	9.25	8	2	1	High	1	1.025	1.1	1.597	1.57	1.451	1.365	1.555	1.428	2.104	1.846	1.859	2.183	1.872	2.244
TRINITY_DN10652_c0_g1_i4_orf1	uncharacterized protein LOC114364166 [Ostrinia furnacalis]	2	245	28.5	6.9	7	2	2	High	1	0.916	1.021	1.515	1.52	1.587	1.133	1.146	1.109	1.144	1.341	1.008	1.006	1.008	0.956
TRINITY_DN44110_c0_g1_i4_orf1	putative phosphatidate phosphatase [Ostrinia furnacalis]	1	287	32.9	7.88	8	1	1	High	1	1.176	1.079	1.848	1.811	1.793	1.397	1.648	1.44	1.489	1.183	1.116	1.413	1.278	1.124
TRINITY_DN11820_c0_g1_i1_orf1	hypothetical protein evm_000341 [Chilo suppressalis]	1	329	36.9	5.19	5	1	1	High	1	1.019	1.041	0.799	0.653	0.635	0.718	0.559	0.654	0.416	0.441	0.519	1.035	0.857	0.891
TRINITY_DN825_c0_g1_i18_orfp1	TRINITY_DN825_c0_g1_i18_m.8360 TRINITY_DN825_c0_g1::TRINITY_DN825_c0	1	396	44.7	8.13	5	2	1	High	1	1.098	1.026	1.229	1.06	1.268	0.938	1.06	0.88	1.051	0.907	0.985	0.674	0.607	0.596
TRINITY_DN18696_c0_g1_i1_orf1	rap guanine nucleotide exchange factor 2 [Ostrinia furnacalis]	1	1180	132.4	6.71	1	1	1	High	1	0.983	1.423	0.664	0.673	0.496	0.484	0.47	0.542	0.46	0.535	0.591	0.456	0.562	0.448
TRINITY_DN2402_c1_g1_i8_orf1	interferon regulatory factor 2-binding protein 1 [Ostrinia furnacalis]	2	431	45.8	8.54	4	2	2	High	1	0.931	0.724	0.691	0.911	0.821	0.63	0.741	0.641	0.634	0.722	0.886	0.793	0.926	0.815
TRINITY_DN8922_c0_g1_i3_orf1	protein sidekick isoform X1 [Ostrinia furnacalis]	1	2215	245.2	6.13	1	1	1	High	1	0.909	0.667	1.458	1.205	1.51	1.24	1.351	1.386	1.747	2.587	1.618	1.388	1.48	1.52
TRINITY_DN4820_c0_g1_i1_orf1	tudor and KH domain-containing protein homolog [Galleria mellonella]	1	146	16.2	6.89	10	1	1	High	1	1.107	0.978	0.608	0.63	0.655	0.58	0.523	0.579	0.468	0.466	0.538	0.529	0.457	0.608
TRINITY_DN8926_c0_g1_i4_orf1	run domain Beclin-1-interacting and cysteine-rich domain-containing protein	2	703	79	5.54	3	2	2	High	1	1.047	0.949	1.225	1.413	1.406	1.439	1.363	1.18	1.022	1.522	1.302	1.417	1.379	1.359
TRINITY_DN19951_c0_g1_i5_orf1	protein croquemort-like [Ostrinia furnacalis]	1	504	56.6	5.03	3	1	1	High	1	0.904	1.047	1.113	1.309	1.191	0.983	1.497	1.205	1.208	1.326	1.327	2.263	2.085	1.821
TRINITY_DN11566_c0_g1_i6_orf1	lens fiber major intrinsic protein-like isoform X1 [Ostrinia furnacalis]	1	249	25.6	6.79	4	1	1	High	1	0.981	1.05	0.777	0.678	0.862	1.105	1.079	1.061	0.866	0.777	0.842	1.156	1.297	1.042
TRINITY_DN85004_c0_g1_i1_orf1	uncharacterized protein LOC114357684 [Ostrinia furnacalis]	1	173	18.1	9.91	9	1	1	High	1	0.977	0.826	1.014	1.121	1.046	1.007	1.002	0.963	1.078	1.036	1.071	0.589	0.519	0.45
TRINITY_DN31967_c0_g1_i5_orf1	N-acetylgalactosamine kinase [Ostrinia furnacalis]	1	487	54.5	6.74	2	1	1	High	1	0.976	1.013	0.617	0.636	0.582	0.672	0.528	0.727	0.717	0.64	0.546	0.964	0.912	0.941
TRINITY_DN9836_c0_g1_i2_orf1	STAM-binding protein-like A isoform X2 [Ostrinia furnacalis]	1	282	31.7	6.76	5	1	1	High	1	1.156	1.035	0.997	0.941	1.073	1.258	1.498	1.313	1.215	1.286	1.251	1.08	1.105	1.378
TRINITY_DN14209_c0_g1_i1_orf1	unnamed protein product [Diatraea saccharalis]	1	72	8.2	4.67	13	1	1	High	1	1.044	1.096	0.994	1.01	1.12	0.948	1.024	0.866	0.835	0.633	0.904	0.799	0.846	0.779
TRINITY_DN3801_c0_g1_i9_orf1	claspin-like isoform X2 [Ostrinia furnacalis]	1	1307	146.4	6.05	1	1	1	High	1	1.027	1.133	0.76	0.799	0.736	0.645	0.75	0.795	0.626	0.713	0.78	0.8	0.945	0.896
TRINITY_DN27704_c0_g1_i1_orf1	PREDICTED: tRNA (guanine-N(7)-)-methyltransferase [Amyelois transitella]	2	236	28	7.3	7	2	2	High	1	1.01	0.948	0.469	0.449	0.516	0.551	0.504	0.453	0.441	0.443	0.445	0.487	0.583	0.528
TRINITY_DN14973_c0_g1_i1_orf1	hypothetical protein evm_003718 [Chilo suppressalis]	1	511	55.4	5.74	5	1	1	High	1	0.966	1.04	1.103	1.105	0.897	1.062	0.97	0.967	1.099	0.99	0.933	0.69	0.949	1.005
TRINITY_DN91946_c0_g1_i1_orf1	protein catecholamines up [Ostrinia furnacalis]	1	353	38	6.93	4	1	1	High	1	0.898	1.074	1.947	1.866	1.798	2.081	1.903	2.044	1.609	1.493	1.461	1.203	1.301	1.076
TRINITY_DN6330_c0_g1_i1_orfp1	TRINITY_DN6330_c0_g1_i1_m.42332 TRINITY_DN6330_c0_g1::TRINITY_DN6330	1	50	5.3	6.15	26	1	1	High	1	1.304	1.186	6.649	7.491	6.992	5.909	7.901	6.646	5.385	4.417	5.118	2.914	2.949	2.319
TRINITY_DN868_c0_g1_i4_orf1	uncharacterized protein LOC114359357 isoform X1 [Ostrinia furnacalis]	1	202	22.6	4.86	5	1	1	High	1	1.021	0.985	0.215	0.221	0.326	0.519	0.532	0.46	0.211	0.187	0.201	0.207	0.175	0.221
TRINITY_DN17995_c0_g4_i1_orf1	dynein light chain roadblock-type 2 [Bombyx mori]	1	97	10.9	6.8	16	1	1	High	1	1.013	0.928	0.861	1.014	1.048	0.969	0.985	1.017	1.295	1.193	0.994	1.436	1.296	1.231
TRINITY_DN42719_c0_g1_i1_orf1	inter-alpha-trypsin inhibitor heavy chain H4-like isoform X11 [Ostrinia furnaca	1	71	7.4	4.42	23	1	1	High	1	1.29	1.036	1.389	1.501	1.066	1.228	1.388	1.278	1.566	2.052	1.945	4.229	4.658	3.78
TRINITY_DN4356_c0_g1_i6_orf1	mulatexin-like [Ostrinia furnacalis]	1	185	20.1	7.97	5	1	1	High	1	0.961	0.964	0.181	0.182	0.299	0.538	0.496	0.628	0.785	0.585	0.678	0.461	0.395	0.47
TRINITY_DN131264_c0_g1_i2_orf1	unnamed protein product [Parnassius apollo]	2	109	11.7	9.91	24	2	2	High	1	0.724	1.104	1.063	0.892	0.948	0.563	0.838	0.614	0.798	0.916	0.769	1.323	1.592	1.525
TRINITY_DN747_c0_g1_i4_orf1	trypsin, alkaline C-like [Ostrinia furnacalis]	1	252	28	7.36	5	1	1	High	1	0.974	1.113	0.29	0.338	0.333	0.422	0.396	0.373	0.318	0.303	0.294	0.291	0.313	0.286
TRINITY_DN5363_c0_g1_i1_orf1	cytochrome c oxidase assembly factor 6 homolog [Ostrinia furnacalis]	1	82	10	8.46	20	1	1	High	1	0.937	1.055	0.952	0.825	0.789	0.921	1.007	0.929	0.916	1.097	0.967	0.703	0.832	0.826
TRINITY_DN1575_c0_g1_i10_orf1	uncharacterized protein LOC114359245 [Ostrinia furnacalis]	1	417	45.9	5.44	2	1	1	High	1	0.716	0.989	3.377	4.233	3.675	1.028	1.09	1.266	3.025	2.493	3.372	2.461	2.759	2.165
TRINITY_DN56708_c0_g3_i1_orfp1	TRINITY_DN56708_c0_g3_i1_m.56611 TRINITY_DN56708_c0_g3::TRINITY_DN56	1	68	7.1	5.66	18	1	1	High	1	0.968	1.064	1.998	1.984	1.778	1.358	1.502	1.536	1.463	1.202	1.484	0.847	0.954	0.843
TRINITY_DN1630_c0_g1_i6_orf1	major facilitator superfamily domain-containing protein 1-like [Ostrinia furnac	1	513	57.8	5.53	3	1	1	High	1	0.958	1.177	1.37	1.53	1.221	1.186	1.328	1.452	1.482	1.49	1.336	2.249	2.097	2.042
TRINITY_DN42159_c0_g1_i6_orf1	regucalcin-like [Nymphalis io]	2	116	12.3	9.2	22	3	1	High	1	0.473	0.729	1.099	0.875	1.062	0.919	0.929	1.394	1.427	3.546	1.289	1.438	1.495	1.769
TRINITY_DN3254_c0_g1_i1_orf1	ATP-dependent (S)-NAD(P)H-hydrate dehydratase [Ostrinia furnacalis]	1	324	35.1	8.43	4	1	1	High	1	0.947	1.115	0.668	0.773	0.743	0.844	0.839	0.733	0.879	0.867	0.839	0.907	0.847	0.784
TRINITY_DN84_c0_g1_i4_orf1	aspartyl/asparaginyl beta-hydroxylase isoform X2 [Ostrinia furnacalis]	1	683	78	4.96	2	2	1	High	1	1.309	1.066	0.733	0.668	0.786	0.871	0.776	0.707	0.679	0.768	0.572	0.603	0.717	0.632

TRINITY_DN8361_c0_g1_i4_orf1	activin receptor type-2A-like isoform X1 [Ostrinia furnacalis]	1	548	60.4	6.37	2	1	1	High	1	1.135	1.073	1.119	1.067	1.048	1.14	1.137	0.964	0.842	0.842	1.125	1.218	1.005	1.097
TRINITY_DN76529_c0_g1_i1_orfp1	TRINITY_DN76529_c0_g1_i1_m.64079 TRINITY_DN76529_c0_g1::TRINITY_DN76	1	69	7.7	11.53	23	1	1	High	1	1.044	1.104	1.677	1.889	1.852	0.973	1.069	0.979	0.719	0.771	0.924	0.499	0.469	0.481
TRINITY_DN29351_c0_g1_i1_orfp1	TRINITY_DN29351_c0_g1_i1_m.58077 TRINITY_DN29351_c0_g1::TRINITY_DN29	1	327	37.7	8.65	4	1	1	High	1	0.901	0.94	1.125	1.097	1.01	1.099	1.266	1.143	1.247	1.49	1.139	1.096	1.232	1.425
TRINITY_DN8044_c0_g1_i2_orf1	protein FRG1 homolog [Pectinophora gossypiella]	2	258	28.5	7.43	7	2	2	High	1	1.02	0.964	0.963	1.078	1.038	0.97	0.9	0.908	0.89	0.752	0.869	1.009	1.036	1.013
TRINITY_DN3504_c0_g1_i4_orfp1	TRINITY_DN3504_c0_g1_i4_m.43930 TRINITY_DN3504_c0_g1::TRINITY_DN3504	1	195	21.5	5.19	8	1	1	High	1	0.888	0.953	0.272	0.334	0.28	0.312	0.284	0.418	0.285	0.197	0.277	0.121	0.053	0.118
TRINITY_DN4280_c0_g1_i8_orf1	uncharacterized protein LOC114354853 [Ostrinia furnacalis]	1	295	32.5	8.94	5	1	1	High	1	1.023	0.931	0.833	0.783	0.912	0.661	0.812	0.826	0.674	1	0.84	0.818	0.681	0.908
TRINITY_DN83574_c0_g1_i1_orf1	glutathione S-transferase omega 4 [Ostrinia furnacalis]	2	274	31.8	8.63	7	2	2	High	1	1.096	1.037	1.006	0.884	0.821	0.873	0.908	0.897	1.034	0.895	0.898	1.066	1.026	1.001
TRINITY_DN67026_c0_g1_i6_orf1	hypothetical protein O3G_MSEX011964 [Manduca sexta]	1	67	7	5.87	27	1	1	High	1	1.016	1.1	0.922	0.897	1.122	1.326	1.281	1.154	1.689	1.371	1.457	0.987	1.533	1.173
TRINITY_DN51658_c0_g1_i1_orf1	E3 ubiquitin-protein ligase synoviolin B [Ostrinia furnacalis]	1	153	17	5.43	14	1	1	High	1	0.948	1.149	1.338	1.176	1.578	0.923	0.772	1.042	1.079	0.784	0.81	1.1	1.163	1.103
TRINITY_DN39673_c0_g1_i1_orf1	uncharacterized protein LOC114359357 isoform X1 [Ostrinia furnacalis]	1	255	27.9	4.49	4	3	1	High	1	0.948	0.905	0.299	0.296	0.315	0.44	0.447	0.386	0.321	0.355	0.376	0.338	0.32	0.319
TRINITY_DN15114_c0_g2_i1_orf1	uncharacterized protein LOC114359552 [Ostrinia furnacalis]	1	226	25.6	5.01	6	1	1	High	1	1.037	0.893	1.145	1.303	1.316	1.205	0.945	1.088	1.026	1.044	1.011	1.329	1.53	1.51
TRINITY_DN1267_c0_g2_i10_orf1	secretory phospholipase A2 receptor-like [Ostrinia furnacalis]	2	297	34.1	5.41	5	2	2	High	1	0.803	0.908	1.893	1.803	1.73	1.107	1.037	1.23	1.405	1.105	1.434	0.674	0.588	0.65
TRINITY_DN51836_c0_g3_i1_orf1	KRT17 isoform 1 [Pan troglodytes]	1	132	15.3	4.77	10	1	1	High	1	0.792	1.463	0.863	1.222	0.784	0.654	0.708	0.905	1.233	1.346	0.881	1.588	1.6	0.99
TRINITY_DN44517_c0_g1_i4_orf1	regucalcin-like [Ostrinia furnacalis]	1	335	36.9	6.32	4	1	1	High	1	0.897	1.094	2.452	2.533	2.194	2.243	2.403	2.507	2.177	2.625	1.964	1.871	1.817	1.654
TRINITY_DN1576_c0_g1_i4_orf1	elongator complex protein 3 [Manduca sexta]	1	345	39	8.76	4	1	1	High	1	0.923	0.961	1.355	1.11	1.045	1.028	1.317	1.179	1.094	1.956	1.149	1.168	1.145	1.176
TRINITY_DN36648_c0_g1_i1_orf1	UMP-CMP kinase [Ostrinia furnacalis]	2	213	23.9	7.75	9	2	2	High	1	0.998	0.988	0.578	0.573	0.655	0.651	0.578	0.561	0.59	0.501	0.598	0.626	0.598	0.566
TRINITY_DN19866_c0_g1_i4_orf1	lys-63-specific deubiquitinase BRCC36-like [Ostrinia furnacalis]	1	264	29.6	5.62	5	1	1	High	1	1.009	1.064	1.033	1.008	0.898	1.031	1.076	1.1	1.393	1.807	1.467	1.009	1.199	0.962
TRINITY_DN44261_c0_g1_i1_orf1	neural Wiskott-Aldrich syndrome protein-like [Colias croceus]	1	315	35.4	9.2	4	1	1	High	1	0.833	0.796	0.649	0.719	0.687	0.635	0.637	0.781	0.805	0.395	0.671	0.595	0.542	0.549
TRINITY_DN9383_c0_g1_i3_orf1	uncharacterized protein LOC114361502 [Ostrinia furnacalis]	1	173	19.3	7.14	5	2	1	High	1	1.036	0.938	1.926	2.031	1.8	1.236	1.299	1.213	1.399	1.096	1.68	2.206	2.333	2.287
TRINITY_DN7122_c0_g1_i1_orf1	hypothetical protein evm_003965 [Chilo suppressalis]	2	873	96.4	7.4	3	2	2	High	1	0.903	0.764	0.575	0.597	0.595	0.629	0.779	0.828	0.535	0.555	0.652	0.678	0.831	0.816
TRINITY_DN27276_c0_g1_i5_orf1	probable small nuclear ribonucleoprotein Sm D1 [Ostrinia furnacalis]	1	125	13.6	11.53	10	1	1	High	1	1.026	1.036	0.829	0.738	0.956	0.804	1.249	0.951	0.623	0.643	0.556	0.512	0.497	0.526
TRINITY_DN9475_c0_g1_i6_orf1	uncharacterized protein LOC114358636 [Ostrinia furnacalis]	1	160	18	6.51	10	1	1	High	1	0.914	0.878	0.88	0.885	0.925	1.049	1.169	0.982	0.851	0.854	1.106	1.492	1.577	1.497
TRINITY_DN1956_c7_g1_i1_orf1	polyprenol reductase [Ostrinia furnacalis]	1	305	35.3	9.06	4	1	1	High	1	0.938	0.908	0.731	0.934	0.794	0.814	0.887	0.889	0.85	0.634	0.809	0.881	0.885	0.8
TRINITY_DN31980_c0_g1_i1_orf1	E3 ubiquitin-protein ligase TRIP12 isoform X2 [Ostrinia furnacalis]	2	1057	116.6	6.96	1	2	2	High	1	1.014	0.999	1.083	0.824	1.028	0.91	0.995	0.921	1.156	1.055	1.022	1.188	1.275	1.075
TRINITY_DN123396_c0_g1_i1_orf1	PREDICTED: delta-1-pyrroline-5-carboxylate dehydrogenase, mitochondrial is	1	568	63.5	8.34	1	2	2	High	1	1.009	0.92	0.249	0.279	0.311	0.215	0.227	0.248	0.255	0.231	0.261	0.282	0.26	0.276
TRINITY_DN8771_c0_g2_i1_orf1	regucalcin-like [Ostrinia furnacalis]	2	172	19.2	4.92	11	3	1	High	1	1.19	0.928	0.524	0.571	0.523	0.264	0.499	0.388	2.035	1.589	2.134	0.559	0.642	0.607
TRINITY_DN8838_c0_g1_i1_orf1	mannose-P-dolichol utilization defect 1 protein homolog [Ostrinia furnacalis]	1	247	26.8	9.23	4	1	1	High	1	0.832	0.859	0.419	0.266	0.48	0.433	0.401	0.639	0.432	0.379	0.228	0.412	0.477	0.531
TRINITY_DN2508_c0_g1_i2_orf1	uncharacterized protein LOC114361845 [Ostrinia furnacalis]	1	188	20.8	5.07	5	1	1	High	1	0.991	1.208	1.359	1.637	1.426	1.54	1.666	1.474	1.304	1.567	1.549	1.87	1.858	1.857
TRINITY_DN28981_c0_g1_i1_orf1	uncharacterized protein C6orf203 homolog [Ostrinia furnacalis]	1	199	22.6	9.57	5	1	1	High	1	0.996	1.113	0.44	0.384	0.419	0.456	0.443	0.497	0.491	0.375	0.472	0.546	0.52	0.504
TRINITY_DN4905_c0_g1_i6_orf1	uncharacterized protein LOC114351759 [Ostrinia furnacalis]	1	606	62.4	4.78	4	1	1	High	1	1.159	1.119	0.25	0.402	0.383	0.403	0.355	0.435	0.521	0.511	0.315	0.34	0.307	0.382
TRINITY_DN11665_c0_g1_i4_orf1	TBC1 domain family member 9 isoform X1 [Ostrinia furnacalis]	2	1139	128.5	5.8	1	2	2	High	1	0.924	1.043	0.83	0.988	0.989	1.04	0.974	1.042	1.001	0.913	0.934	1.379	1.283	1.497
TRINITY_DN28938_c0_g1_i1_orf1	uncharacterized protein LOC114354070 isoform X3 [Ostrinia furnacalis]	1	88	9.6	9.51	11	1	1	High	1	1.261	1.065	0.964	0.937	1.039	0.796	0.91	0.946	0.795	0.772	0.834	1.077	0.951	0.88
TRINITY_DN227_c0_g1_i1_orf1	double-stranded ribonuclease 2 [Ostrinia nubilalis]	1	448	50.9	8.94	3	1	1	High	1	0.98	1.175	0.502	0.474	0.529	0.533	0.603	0.533	0.795	0.609	0.787	0.702	0.718	0.721
TRINITY_DN32420_c0_g1_i2_orf1	PREDICTED: plectin-like, partial [Papilio polytes]	2	104	12.2	6.05	18	2	1	High	1	1.004	0.953	0.801	0.646	0.626	0.966	0.935	0.81	0.765	0.778	0.741	0.674	0.582	0.574
TRINITY_DN2474_c0_g1_i5_orf1	glucose-6-phosphate 1-epimerase [Galleria mellonella]	1	277	32	7.14	4	1	1	High	1	0.886	1.011	0.747	0.833	0.831	0.706	0.764	0.683	0.876	0.872	0.82	1.227	1.04	1.264
TRINITY_DN18782_c0_g1_i4_orf1	putative riboflavin kinase [Ostrinia furnacalis]	1	162	18.2	6.68	7	1	1	High	1	0.948	1.06	1.246	1.205	1.137	1.575	2.046	1.835	1.624	1.82	1.621	0.709	0.659	0.875
TRINITY_DN2827_c3_g1_i3_orf1	CBP80/20-dependent translation initiation factor isoform X3 [Helicoverpa zea]	2	469	49.7	9.04	5	2	2	High	1	1.094	1.078	0.976	0.956	1.101	1.121	0.959	1.054	1.028	1.084	0.717	0.849	0.757	0.963
TRINITY_DN25345_c0_g1_i1_orf1	chromodomain-helicase-DNA-binding protein 1 isoform X3 [Ostrinia furnacali	2	1816	208.3	8.16	1	2	2	High	1	0.93	0.933	0.697	0.746	0.689	0.698	0.825	0.781	0.891	1.041	1.481	2.244	2.516	2.089
TRINITY_DN62091_c0_g1_i1_orf1	protein NipSnap [Venturia canescens]	1	284	33.2	9.35	4	2	1	High	1	0.966	0.947	1.659	1.458	1.598	1.906	1.706	2.023	1.841	1.916	1.426	1.239	1.207	1.414
TRINITY_DN10297_c0_g1_i1_orf1	polyglutamine-binding protein 1 [Ostrinia furnacalis]	1	279	31.7	6.3	4	1	1	High	1	0.907	0.861	0.603	0.67	0.667	0.599	0.613	0.655	0.659	0.765	0.703	1.056	0.825	0.802
TRINITY_DN16123_c0_g1_i1_orf1	39S ribosomal protein L51, mitochondrial [Ostrinia furnacalis]	1	156	18.5	10.18	13	1	1	High	1	1.052	0.978	1.085	1.266	1.018	0.835	1.023	0.83	1.144	1.783	1.46	0.852	1.013	1.192
TRINITY_DN1968_c0_g1_i3_orf1	protein ABHD4 isoform X3 [Ostrinia furnacalis]	1	378	42.2	8.35	4	1	1	High	1	0.948	1.07	1.098	1.019	1.168	0.806	0.958	0.91	1.086	1.141	0.981	0.964	0.746	0.912
TRINITY_DN57496_c0_g1_i1_orf1	NADPH:adrenodoxin oxidoreductase, mitochondrial [Ostrinia furnacalis]	2	472	52.8	8.29	3	2	2	High	1	1	1.025	0.441	0.47	0.486	0.689	0.719	0.701	0.537	0.527	0.582	0.714	0.761	0.687
TRINITY_DN120144_c0_g1_i1_orf1	pre-mRNA-splicing factor SPF27 [Ostrinia furnacalis]	1	245	28	5.99	4	1	1	High	1	0.976	0.926	0.913	1.02	0.909	0.746	0.923	0.781	0.88	0.719	0.862	1.112	1.183	1.121
TRINITY_DN63389_c0_g1_i4_orf1	retinol dehydrogenase 14-like [Ostrinia furnacalis]	2	310	34.7	8.54	13	2	2	High	1	1.178	1.138	1.141	1.157	1.094	1.644	1.752	1.606	1.606	1.436	1.534	1.562	1.381	1.659
TRINITY_DN16487_c0_g1_i1_orf1	p21-activated protein kinase-interacting protein 1-like [Ostrinia furnacalis]	1	385	42.5	8.41	2	1	1	High	1	0.928	0.899	0.363	0.465	0.408	0.579	0.607	0.499	0.506	0.571	0.739	0.493	0.427	0.508
TRINITY_DN7047_c0_g1_i1_orf1	hypothetical protein G9C98_004728 [Cotesia typhae]	1	208	24	9.41	4	1	1	High	1	1.001	0.95	0.184	0.171	0.258	0.226	0.262	0.219	0.228	0.18	0.189	0.254	0.294	0.295
TRINITY_DN51498_c0_g1_i1_orf1	delta-aminolevulinic acid dehydratase isoform X3 [Ostrinia furnacalis]	2	333	36.3	6.92	7	5	2	High	1	0.977	1.077	1.004	1.102	1.049	1.289	1.591	1.455	1.095	1.016	1.012	1.133	1.174	1.128
TRINITY_DN32514_c0_g2_i1_orf1	mucin-5AC-like [Ostrinia furnacalis]	1	131	14.6	4.49	9	1	1	High	1	1.062	1.194	1.076	1.543	1.175	0.797	0.961	1.404	0.918	1.23	0.786	21.41	23.006	18.79
TRINITY_DN42337_c0_g1_i4_orf1	insect cuticle protein domain-containing protein [Phthorimaea operculella]	2	84	9	6.67	39	2	1	High	1	1.03	1.001	1.146	1.123	1.277	1.072	0.92	1.052	1.14	1.101	1.271	1.213	1.206	1.03
TRINITY_DN7618_c0_g1_i4_orf1	uncharacterized protein LOC114366712 isoform X1 [Ostrinia furnacalis]	1	341	36.6	8.53	4	1	1	High	1	1.206	0.979	1.673	1.703	1.684	1.909	2.482	1.997	1.925	1.618	2.154	2.838	3.313	2.993
TRINITY_DN54711_c0_g1_i1_orf1	39S ribosomal protein L50, mitochondrial [Ostrinia furnacalis]	1	187	21.2	9.44	9	1	1	High	1	1.016	1.052	0.673	0.805	0.723	0.86	0.701	0.652	0.648	0.723	0.62	0.653	0.643	0.726
TRINITY_DN7583_c0_g1_i1_orf1	39S ribosomal protein L21, mitochondrial [Ostrinia furnacalis]	1	177	19.8	10.24	5	1	1	High	1	1	1.003	0.44	0.362	0.417	0.428	0.292	0.354	0.402	0.377	0.447	0.57	0.563	0.607
TRINITY_DN5757_c0_g1_i1_orf1	ATP-dependent DNA helicase 2 subunit 1 [Ostrinia furnacalis]	1	594	66.8	6.05	2	1	1	High	1	0.947	1.096	1.226	0.996	1.051	1.026	1.126	1.026	1.101	1.261	1.079	2.133	2.329	2.953
TRINITY_DN23089_c0_g1_i1_orf1	integrator complex subunit 3 homolog [Ostrinia furnacalis]	1	999	114.4	5.6	1	1	1	High	1	1.026	0.968	0.828	0.884	0.836	0.752	0.663	0.711	0.56	0.897	0.612	0.797	0.681	0.934
TRINITY_DN14601_c0_g1_i2_orf1	unnamed protein product [Chilo suppressalis]	2	682	76.4	8.9	2	2	2	High	1	0.993	1.01	0.416	0.472	0.476	0.478	0.549	0.46	0.393	0.496	0.354	0.47	0.701	0.497
TRINITY_DN45708_c1_g1_i1_orf1	uncharacterized protein LOC114356993 [Ostrinia furnacalis]	1	467	53.8	8.44	2	1	1	High	1	1.268	1.41	1.12	1.215	1.225	0.937	0.897	1.301	1.039	1.124	1.19	1.136	1.309	1.257
TRINITY_DN9198_c0_g1_i4_orf1	4-coumarate--CoA ligase 1-like isoform X1 [Ostrinia furnacalis]	2	442	49.1	8.46	4	2	2	High	1	1.006	1.068	0.831	0.81	0.746	1.163	1.164	0.856	0.894	0.762	0.862	0.662	0.655	0.632
TRINITY_DN10379_c0_g1_i3_orf1	succinate dehydrogenase [ubiquinone] cytochrome b small subunit, mitochonc	2	181	19.8	9.99	9	3	2	High	1	0.971	0.979	0.521	0.508	0.482	0.531	0.482	0.522	0.448	0.758	0.432	0.553	0.488	0.517
TRINITY_DN13435_c0_g1_i1_orf1	putative mediator of RNA polymerase II transcription subunit 26 [Ostrinia furna	1	594	67.4	7.21	2	1	1	High	1	1.264	1.229	1.029	1.068	1.201	0.946	1.352	1.09	1.298	1.57	1.462	1.522	1.202	1.374
TRINITY_DN3529_c0_g1_i7_orf1	putative fatty acyl-CoA reductase CG5065 [Ostrinia furnacalis]	1	517	59.3	8.18	3	1	1	High	1	1.09	1.103	0.914	1.108	1.019	1.044	1.102	0.908	0.762	0.646	0.819	0.674	0.74	0.658
TRINITY_DN23343_c0_g1_i9_orf1	pre-mRNA-processing factor 6 isoform X1 [Ostrinia furnacalis]	1	929	104.7	8	1	1	1	High	1	1.022	0.921	0.379	0.472	0.471	0.471	0.427	0.384	0.338	0.275	0.385	0.519	0.45	0.453
TRINITY_DN81084_c0_g3_i1_orf1	unnamed protein product [Diatraea saccharalis]	2	235	25.4	8.98	12	2	2	High	1	1.022	0.991	0.91	1.04	0.866	0.759	0.944	0.829	0.7	0.89	0.803	0.816	0.86	0.944
TRINITY_DN29034_c0_g1_i2_orf1	trypsin-like serine protease [Ostrinia nubilalis]	1	142	15.3	8.41	8	1	1	High	1	1.011	0.996	0.263	0.257	0.315	0.278	0.257	0.242	0.255	0.228	0.232	0.197	0.209	0.173
TRINITY_DN4243_c0_g1_i6_orf1	SET and MYND domain-containing protein 4-like [Ostrinia furnacalis]	1	591	68.1	7.94	2	1	1	High	1	1.633	1.717	0.701	1.047	0.794	0.723	0.999	0.797	0.831	0.665	0.985	0.782	0.836	0.841
TRINITY_DN6716_c0_g1_i1_orf1	probable deoxyhypusine synthase [Ostrinia furnacalis]	1	370	40.9	6.57	3	1	1	High	1	1.028	0.948	1.144	1.025	1.034	1.226	1.052	1.147	1.169	1.173	0.876	1.081	1.003	1.185
TRINITY_DN19244_c0_g1_i7_orf1	uncharacterized protein LOC114350218 [Ostrinia furnacalis]	1	215	25.6	10.8	4	1	1	High	1	0.946	0.946	0.245	0.34	0.347	0.255	0.252	0.226	0.177	0.159	0.205	0.491	0.434	0.36
TRINITY_DN15373_c0_g1_i2_orf1	SET domain-containing protein SmydA-8-like isoform X2 [Ostrinia furnacalis]	1	423	47.5	6.27	3	1	1	High	1	0.974	0.962	1.361	1.234	1.242	1.274	1.26	1.13	1.27	1.413	1.113	0.86	0.801	0.845
TRINITY_DN12775_c0_g1_i10_orfp	TRINITY_DN12775_c0_g1_i10_m.21238 TRINITY_DN12775_c0_g1::TRINITY_DN1	2	66	7.7	9.54	14	2	2	High	1	1.071	0.957	2.043	1.791	1.88	2.61	2.517	2.673	2.755	2.479	2.263	1.611	1.614	1.646
TRINITY_DN581_c3_g2_i1_orf1	uncharacterized protein LOC114364499 isoform X3 [Ostrinia furnacalis]	1	59	6.7	9.88	17	1	1	High	1	1.128	1.295	2.123	2.089	1.966	3.75	4.059	3.983	2.969	2.678	2.902	2.023	1.799	1.602
TRINITY_DN20763_c0_g1_i2_orf1	uncharacterized protein LOC114355848 [Ostrinia furnacalis]	1	170	18.5	8.16	11	1	1	High	1	0.924	0.897	0.757	1.056	0.789	0.735	0.774	0.844	0.713	0.801	0.743	0.739	0.602	0.649
TRINITY_DN440_c0_g1_i11_orf1	hypothetical protein B5X24_HaOG200304 [Helicoverpa armigera]	1	1369	153	7.21	1	1	1	High	1	0.852	0.94	0.873	0.849	0.831	0.791	0.959	0.796	0.886	1.171	0.979	0.996	0.918	0.965
TRINITY_DN2897_c0_g2_i1_orf1	gem-associated protein 5-like [Ostrinia furnacalis]	1	911	100.8	6.67	2	1	1	High	1	1.007	0.991	1.114	1.161	1.084	1.294	1.38	1.285	1.497	1.642	1.724	1.732	1.762	1.771
TRINITY_DN8682_c0_g1_i4_orf1	protein SYS1 homolog [Ostrinia furnacalis]	1	163	18.4	6.86	9	1	1	High	1	0.97	0.917	0.913	0.827	0.804	0.784	0.936	0.905	0.913	1.041	0.83	1.133	1.229	1.321
TRINITY_DN2712_c0_g1_i2_orf1	mitochondrial intermediate peptidase [Hyposmocoma kahamanoa]	1	697	78	6.81	2	1	1	High	1	0.93	0.897	0.848	0.859	0.853	0.751	0.932	1.009	0.896	1.807	0.876	0.966	0.922	1.127
TRINITY_DN3486_c0_g1_i5_orf1	uncharacterized protein LOC114360519 [Ostrinia furnacalis]	1	104	12.1	8.47	12	1	1	High	1	1.076	1.012	1.827	1.988	1.804	1.813	1.912	1.685	1.246	0.954	1.227	1.107	0.999	1.012
TRINITY_DN23714_c0_g1_i4_orf1	DNA-directed RNA polymerases I, II, and III subunit RPABC4 [Ostrinia furnacalis	1	64	7.7	9.31	19	1	1	High	1	1.113	1.159	0.892	0.774	0.754	0.867	0.795	0.761	0.886	1.034	0.79	0.987	0.902	0.955
TRINITY_DN131924_c0_g1_i2_orf1	protein FAM151A isoform X2 [Ostrinia furnacalis]	1	299	32.5	6.86	5	1	1	High	1	0.928	0.952	1.136	1.267	1.153	1.188	1.267	1.26	0.831	1.089	2.36	1.524	0.922	0.986
TRINITY_DN14154_c0_g1_i1_orf1	uncharacterized protein LOC114360857, partial [Ostrinia furnacalis]	1	463	51	6.21	2	1	1	High	1	0.962	0.932	1.083	1.217	0.972	0.973	1.15	1.271	1.147	1.464	1.203	1.276	1.365	1.376
TRINITY_DN3047_c0_g2_i6_orf1	uncharacterized protein LOC114362414 isoform X1 [Ostrinia furnacalis]	2	382	43.9	6.38	4	2	2	High	1	1.099	1.035	0.904	1.152	0.929	0.98	1.074	1.076	1.008	0.9	0.866	1.017	0.941	1.093
TRINITY_DN27214_c0_g1_i4_orf1	ankyrin repeat domain-containing protein 49 [Manduca sexta]	1	240	26.1	5.1	4	1	1	High	1	0.992	1.178	0.92	1.068	1.07	0.942	0.933	0.941	0.807	0.87	0.983	1.183	0.978	1.17
TRINITY_DN46124_c0_g1_i1_orf1	protein NipSnap [Plutella xylostella]	1	74	8.3	7.08	12	2	1	High	1	1.076	1.242	1.015	1	0.944	1.166	0.837	0.91	0.951	0.908	0.907	0.861	0.793	0.8
TRINITY_DN3087_c0_g1_i1_orf1	uncharacterized protein LOC114360174 [Ostrinia furnacalis]	1	1736	197.3	6.24	1	1	1	High	1	0.908	1.117	1.357	1.324	1.451	1.313	1.269	1.426	1.046	1.708	1.03	1.223	1.114	1.173
TRINITY_DN798_c1_g1_i3_orf1	protein goliath isoform X1 [Ostrinia furnacalis]	1	424	47	7.87	3	1	1	High	1	1.18	1.089	1.942	2.367	2.323	2.388	2.088	2.575	2.105	2.161	2.607	2.318	2.239	2.511
TRINITY_DN56993_c0_g1_i4_orf1	polypyrimidine tract-binding protein 1 isoform X11 [Helicoverpa zea]	1	540	58.2	9.25	3	1	1	High	1	1.031	0.929	0.62	0.728	0.857	0.875	0.807	0.92	0.831	0.736	0.683	1.232	1.434	1.069
TRINITY_DN925_c0_g1_i5_orf1	glutathione hydrolase 1 proenzyme-like [Helicoverpa zea]	1	576	62.3	6.35	3	1	1	High	1	0.94	0.994	0.82	0.903	0.907	1.063	1.035	0.918	1.071	0.886	0.918	0.895	0.753	0.738
TRINITY_DN2535_c0_g1_i4_orf1	ATP-dependent RNA helicase DDX24 [Ostrinia furnacalis]	1	1038	116.3	6.3	2	1	1	High	1	0.78	0.848	0.422	0.603	0.655	0.548	0.539	0.582	0.648	0.592	0.603	0.545	0.536	0.529
TRINITY_DN30476_c0_g1_i1_orf1	unnamed protein product [Arctia plantaginis]	1	99	11.1	5.15	8	1	1	High	1	0.89	0.876	1.204	1.203	1.203	1.159	1.126	1.157	0.911	0.861	1.142	0.86	0.91	0.881
TRINITY_DN12286_c1_g1_i2_orf1	sideroflexin-1-3 [Galleria mellonella]	1	318	35.3	9.28	4	2	1	High	1	1.003	1.021	1.182	1.229	1.175	1.124	1.299	1.227	1.418	1.56	1.614	2.32	1.928	1.903
TRINITY_DN18128_c0_g1_i4_orf1	arylsulfatase B [Ostrinia furnacalis]	1	87	9.5	6.27	14	1	1	High	1	0.996	1.036	1.827	1.947	1.79	2.577	2.287	2.246	2.807	3.134	3.167	2.485	2.622	2.538
TRINITY_DN468_c0_g1_i3_orf1	transmembrane protein 41 homolog isoform X2 [Ostrinia furnacalis]	1	271	30.6	9.28	7	1	1	High	1	0.977	0.971	0.193	0.184	0.261	0.298	0.301	0.325	0.284	0.282	0.268	0.285	0.281	0.256
TRINITY_DN10371_c0_g1_i2_orf1	heterogeneous nuclear ribonucleoprotein L isoform X2 [Vanessa cardui]	2	476	52.6	8.5	5	2	2	High	1	0.654	1.257	1.552	1.386	1.85	1.766	1.906	2.432	2.007	1.305	1.15	2.002	1.794	1.686
TRINITY_DN11368_c0_g1_i1_orf1	dystonin isoform X24 [Pieris brassicae]	2	84	10	6.07	23	3	2	High	1	0.929	1.007	1.052	1.038	0.988	1.03	1.068	1.017	1.074	0.87	1.039	0.897	0.897	0.932
TRINITY_DN25870_c0_g2_i6_orf1	homeobox protein extradenticle isoform X3 [Ostrinia furnacalis]	1	390	43.1	7.27	3	1	1	High	1	1.159	0.945	0.944	0.957	1.042	0.827	0.797	0.894	0.806	0.675	0.82	1.271	1.217	1.322

TRINITY_DN96884_c0_g1_i1_orf1	hypothetical protein evm_003360 [Chilo suppressalis]	2	265	30.6	5.62	6	3	2	High	1	1.025	1.089	0.921	0.716	0.99	0.873	0.802	0.971	0.888	0.916	0.75	0.943	0.797	0.824
TRINITY_DN1381_c0_g1_i5_orf1	CKLF-like MARVEL transmembrane domain-containing protein 4 isoform X1 [(	2	178	19.5	9.26	10	2	1	High	1	1.245	1.276	1.718	1.607	1.709	1.707	1.59	1.742	1.484	1.608	1.12	1.021	1.077	0.958
TRINITY_DN7064_c0_g1_i6_orf1	unnamed protein product [Chilo suppressalis]	2	233	26.3	5.54	10	2	1	High	1	0.946	0.999	0.753	0.904	0.72	0.823	0.83	0.837	0.81	0.687	0.801	0.76	0.953	0.789
TRINITY_DN5070_c0_g1_i1_orf1	ATP-dependent (S)-NAD(P)H-hydrate dehydratase-like [Ostrinia furnacalis]	1	327	35.5	8.29	2	1	1	High	1	1.002	1.233	1.413	1.418	1.533	1.247	1.251	1.347	1.533	1.46	1.779	2.22	1.737	1.737
TRINITY_DN42240_c0_g2_i1_orf1	unnamed protein product [Chilo suppressalis]	1	511	57.1	8.95	2	1	1	High	1	1.056	0.921	1.05	0.883	0.93	1.081	1.131	1.145	1.039	0.783	1.132	1.013	1.012	0.977
TRINITY_DN2135_c0_g1_i2_orf1	casein kinase I isoform X1 [Ostrinia furnacalis]	1	341	39.8	9.48	4	1	1	High	1	1.077	0.925	0.84	0.751	0.945	1.121	1.004	0.953	0.843	1.009	0.933	0.925	0.982	1.088
TRINITY_DN34040_c0_g2_i1_orf1	uncharacterized protein LOC114352849 [Ostrinia furnacalis]	1	208	24	4.42	6	1	1	High	1	1.025	0.953	0.359	0.337	0.417	0.461	0.485	0.481	0.449	0.419	0.461	0.258	0.275	0.248
TRINITY_DN3593_c0_g1_i3_orfp1	TRINITY_DN3593_c0_g1_i3_m.43968 TRINITY_DN3593_c0_g1::TRINITY_DN3593	1	71	7.5	8.73	15	1	1	High	1	1.038	1.087	1.157	1.126	2.323	2.693	2.935	2.874	1.175	1.168	0.887	0.806	0.793	0.644
TRINITY_DN2196_c0_g1_i2_orf1	HIRA-interacting protein 3-like [Ostrinia furnacalis]	1	646	70.4	9.23	2	1	1	High	1	1.033	0.879	0.498	0.553	0.558	0.55	0.5	0.512	0.496	0.607	0.634	0.733	0.804	0.776
TRINITY_DN97883_c0_g1_i2_orf1	talin-2-like, partial [Ostrinia furnacalis]	1	94	10.1	5.03	10	1	1	High	1	0.932	1.158	2.248	2.536	2.282	1.337	1.35	1.337	1.714	1.63	1.954	1.966	1.965	2.057
TRINITY_DN1895_c0_g1_i2_orf1	unnamed protein product [Chrysodeixis includens]	1	213	23.6	7.52	9	1	1	High	1	0.89	1.032	1.124	1.184	1.229	1.039	1.18	1.208	1.049	0.82	0.91	0.6	0.572	0.552
TRINITY_DN3975_c0_g1_i7_orf1	low-density lipoprotein receptor domain class A domain-containing protein [F	1	97	10.6	5.82	13	1	1	High	1	0.974	1.13	1.467	1.901	1.71	1.209	1.203	1.201	1.495	1.041	1.366	1.272	1.207	1.331
TRINITY_DN91198_c0_g2_i1_orf1	unc-112-related protein-like [Pectinophora gossypiella]	1	138	14.8	5.21	12	2	1	High	1	1.157	1.145	0.821	0.998	1.129	0.893	0.871	0.955	0.692	0.853	0.907	0.961	1.025	0.789
TRINITY_DN16516_c0_g1_i1_orf1	sulfotransferase 1E1 [Galleria mellonella]	1	330	38.4	6.4	4	2	1	High	1	0.986	1.087	0.865	0.892	0.874	1.125	1.097	0.939	1.116	1.196	0.857	1.278	1.386	1.308
TRINITY_DN54387_c0_g1_i1_orf1	catalase-like [Pectinophora gossypiella]	1	79	9.3	9.7	22	2	1	High	1	1.177	0.994	1.219	1.096	1.045	1.363	1.359	1.421	1.273	1.068	1.328	1.372	1.344	1.355
TRINITY_DN1569_c0_g1_i6_orf1	uncharacterized protein LOC114350603 [Ostrinia furnacalis]	1	323	37	5.92	7	1	1	High	1	1.52	1.125	1.613	1.055	1.18	1.576	1.754	1.243	2.247	2.171	2.25	1.953	1.768	1.809
TRINITY_DN5848_c0_g1_i6_orf1	brain tumor protein isoform X1 [Ostrinia furnacalis]	2	871	95.7	7.21	2	2	2	High	1	1.123	0.924	0.822	0.895	1.18	1.215	1.492	1.697	1.805	0.981	1.046	1.804	2.263	1.811
TRINITY_DN7868_c0_g1_i2_orf1	uncharacterized protein LOC114353432 isoform X4 [Ostrinia furnacalis]	1	317	36.1	6.24	5	1	1	High	1	0.954	0.822	1.668	1.573	1.618	1.348	1.63	1.568	1.819	2.106	1.742	1.862	1.712	2.344
TRINITY_DN1170_c0_g1_i8_orf1	titin homolog [Trichoplusia ni]	1	472	53	4.58	2	1	1	High	1	0.923	1.012	0.751	0.711	0.861	0.818	0.666	0.637	0.75	0.758	0.586	0.781	0.805	0.965
TRINITY_DN19303_c0_g1_i5_orf1	lipopolysaccharide-induced tumor necrosis factor-alpha factor-like [Ostrinia fu	1	139	14.4	8.13	11	1	1	High	1	0.958	1.047	1.11	1.233	1.122	0.93	0.796	0.842	0.685	0.731	0.664	1.162	1.276	1.295
TRINITY_DN40281_c0_g2_i1_orf1	glyoxylate reductase/hydroxypyruvate reductase [Ostrinia furnacalis]	1	185	20	8.28	5	1	1	High	1	1.029	1.037	0.574	0.718	0.646	0.725	0.856	0.73	0.654	0.627	0.682	0.681	0.643	0.589
TRINITY_DN14826_c0_g1_i1_orf1	uncharacterized protein LOC114350939 [Ostrinia furnacalis]	1	76	8.6	9.44	16	3	1	High	1	0.962	0.962	0.503	0.506	0.546	0.433	0.531	0.444	0.445	0.406	0.461	0.468	0.475	0.471
TRINITY_DN2031_c11_g1_i2_orfp1	TRINITY_DN2031_c11_g1_i2_m.4044 TRINITY_DN2031_c11_g1::TRINITY_DN203	1	148	16.3	6.23	10	1	1	High	1	1.182	1.106	0.916	1.017	1.446	1.295	0.64	1.051	0.547	0.545	0.601	0.55	0.726	0.437
TRINITY_DN95056_c0_g2_i2_orf1	40S ribosomal protein S18 [Halotydeus destructor]	2	152	17.6	10.54	9	3	1	High	1	0.848	1.042	0.934	0.981	0.99	0.904	1.006	0.918	0.819	0.752	0.795	0.894	1.019	0.931
TRINITY_DN11322_c0_g1_i2_orf1	CRAL-TRIO domain-containing protein C3H8.02 [Ostrinia furnacalis]	1	224	26	5.95	5	1	1	High	1	1.059	0.959	0.618	0.613	0.746	0.718	0.606	0.73	0.777	0.8	0.696	0.768	0.723	0.764
TRINITY_DN98995_c0_g1_i2_orf1	hypothetical protein HF086_008399, partial [Spodoptera exigua]	1	709	74.1	4.86	3	1	1	High	1	1.037	1.243	1.516	1.542	1.451	1.5	1.512	1.664	1.273	1.072	1.022	0.867	0.589	0.822
TRINITY_DN12920_c0_g3_i1_orf1	zonadhesin-like isoform X4 [Ostrinia furnacalis]	1	82	8.8	6.68	13	1	1	High	1	0.916	0.823	2.962	3.478	2.851	1.242	1.171	1.265	0.876	1.116	1.057	1.199	1.131	1.253
TRINITY_DN7565_c0_g1_i3_orf1	acylphosphatase-2-like [Ostrinia furnacalis]	1	121	13.7	8.79	9	1	1	High	1	1.035	1.087	0.794	0.736	0.672	1.009	1.051	0.94	0.99	0.875	0.915	2.064	2.478	2.087
TRINITY_DN27247_c0_g2_i1_orfp1	TRINITY_DN27247_c0_g2_i1_m.23157 TRINITY_DN27247_c0_g2::TRINITY_DN27	1	69	7	11.22	20	1	1	High	1	0.868	1.256	1.123	1.189	1.161	2.2	2.09	2.212	1.367	1.895	1.316	1.202	0.778	1.037
TRINITY_DN5987_c1_g1_i1_orf1	DNA-directed RNA polymerase II subunit RPB2 [Ostrinia furnacalis]	1	1176	133.8	6.95	1	1	1	High	1	0.853	0.815	1.09	1.113	0.879	1.199	0.957	1.251	1.057	2.716	1.116	1.113	0.853	1.169
TRINITY_DN5233_c0_g1_i1_orf1	pre-mRNA-splicing factor 38-like [Ostrinia furnacalis]	1	293	36.1	9.01	3	1	1	High	1	0.903	1.033	0.674	0.647	0.674	0.578	0.946	0.696	0.675	0.604	0.619	0.749	0.691	0.803
TRINITY_DN41113_c0_g1_i7_orf1	exocyst complex component 4-like [Ostrinia furnacalis]	1	572	63.2	7.56	2	1	1	High	1	1.115	0.785	0.729	0.893	0.616	0.674	0.715	0.77	0.748	0.432	0.733	0.705	0.892	0.674
TRINITY_DN35757_c0_g1_i1_orf1	ADP-ribosylation factor-like protein 6-interacting protein 1 [Ostrinia furnacalis	2	194	22.2	7.75	11	2	2	High	1	0.989	0.87	1.18	1.104	0.743	1.593	1.65	1.332	1.345	1.053	1.163	1.309	1.279	1.361
TRINITY_DN3755_c0_g1_i3_orf1	caspase-1-like isoform X2 [Ostrinia furnacalis]	2	324	36.7	6.29	6	2	2	High	1	0.842	0.922	0.626	0.652	0.736	0.668	0.8	0.744	0.738	0.902	0.763	0.766	0.643	0.843
TRINITY_DN48054_c0_g1_i7_orf1	CCR4-NOT transcription complex subunit 11 [Bombyx mori]	1	478	53.9	5.91	2	1	1	High	1	1.403	1.273	1.52	1.009	1.092	1.244	0.59	1.412	0.823	0.801	1.034	0.785	0.97	0.914
TRINITY_DN18839_c0_g1_i4_orf1	unnamed protein product [Chilo suppressalis]	2	967	109.2	5.85	2	2	2	High	1	0.998	1.018	0.994	0.853	1.091	0.994	0.975	1.085	0.927	0.845	0.801	0.837	0.839	0.84
TRINITY_DN2954_c0_g1_i1_orf1	unnamed protein product [Diatraea saccharalis]	2	1449	162.1	5.92	1	2	2	High	1	1.1	1.181	0.742	0.78	0.718	0.669	0.636	0.65	0.563	0.46	0.591	0.542	0.657	0.579
TRINITY_DN129226_c0_g1_i2_orf1	hypothetical protein evm_000268 [Chilo suppressalis]	1	269	28.7	5.38	3	1	1	High	1	1.065	0.92	0.506	0.605	0.434	0.572	0.502	0.581	0.367	0.442	0.366	0.853	0.906	0.792
TRINITY_DN15376_c0_g1_i1_orf1	peptidyl-prolyl cis-trans isomerase isoform X1 [Ostrinia furnacalis]	2	305	33.7	6.02	9	2	2	High	1	0.913	0.947	0.641	0.692	0.711	0.615	0.646	0.675	0.698	0.611	0.66	0.659	0.707	0.635
TRINITY_DN21367_c0_g1_i1_orf1	40S ribosomal protein S15 isoform 2 [Homo sapiens]	1	145	17	10.39	19	1	1	High	1	1.358	1.783	1.74	2.237	2.057	1.976	1.956	2.177	2.657	2.691	4.055	1.588	1.93	1.478
TRINITY_DN1155_c0_g1_i9_orf1	carboxypeptidase B-like [Ostrinia furnacalis]	1	427	48.3	5.07	3	1	1	High	1	0.897	0.916	0.799	0.905	0.902	0.869	1.11	0.974	0.835	1.159	0.814	0.768	0.864	0.817
TRINITY_DN35669_c0_g1_i1_orf1	unnamed protein product [Diatraea saccharalis]	2	706	79.8	8.75	2	2	2	High	1	1.029	1.032	0.563	0.601	0.576	0.467	0.571	0.53	0.478	0.541	0.529	0.76	0.766	0.746
TRINITY_DN17905_c0_g3_i1_orf1	zinc finger protein 706-like [Ostrinia furnacalis]	2	77	8.7	9.79	18	3	2	High	1	1.021	0.954	0.323	0.368	0.373	0.321	0.373	0.357	0.348	0.336	0.401	0.528	0.529	0.469
TRINITY_DN47_c0_g1_i2_orf1	uncharacterized protein LOC114356437 isoform X1 [Ostrinia furnacalis]	1	153	17.9	5.49	8	2	1	High	1	0.964	0.974	0.251	0.286	0.319	0.475	0.38	0.352	0.389	0.401	0.425	0.294	0.28	0.283
TRINITY_DN10538_c0_g1_i7_orf1	protein YIF1B [Ostrinia furnacalis]	1	377	41.2	9.23	3	1	1	High	1	1.039	1.212	1.325	0.878	0.902	0.982	0.903	0.867	0.761	0.798	1.345	0.92	0.743	0.895
TRINITY_DN198_c0_g1_i2_orf1	retinol dehydrogenase 13-like [Ostrinia furnacalis]	1	321	35.3	7.81	4	1	1	High	1	1.01	1.16	1.275	1.325	1.162	0.531	0.532	0.527	0.475	0.476	0.63	1.152	1.068	1.198
TRINITY_DN143603_c0_g1_i1_orf1	hypothetical protein KR044_005587 [Drosophila immigrans]	1	247	26.9	6	5	1	1	High	1	1.108	1.202	0.39	0.326	0.422	0.47	0.472	0.41	0.337	0.397	0.428	0.389	0.362	0.309
TRINITY_DN20118_c0_g1_i4_orfp1	hypothetical protein HF086_007571 [Spodoptera exigua]	2	282	29.7	11.27	9	2	2	High	1	0.944	0.886	0.545	0.671	0.603	0.451	0.452	0.886	0.368	0.438	0.552	0.894	0.725	0.817
TRINITY_DN4439_c0_g1_i2_orf1	cytoplasmic FMR1-interacting protein isoform X1 [Ostrinia furnacalis]	1	577	65.9	7.93	1	1	1	High	1	1.025	1.142	0.843	0.792	0.682	0.647	0.659	0.652	0.661	0.661	0.678	1.028	1.072	0.99
TRINITY_DN245_c0_g1_i4_orf1	ER lumen protein-retaining receptor [Ostrinia furnacalis]	1	212	24.4	8.82	9	1	1	High	1	1.086	1.076	1.565	1.637	1.72	1.96	1.911	1.787	1.882	2.344	1.558	0.902	1.005	0.793
TRINITY_DN2396_c0_g1_i9_orfp1	TRINITY_DN2396_c0_g1_i9_m.39038 TRINITY_DN2396_c0_g1::TRINITY_DN2396	1	180	19.3	7.68	6	1	1	High	1	0.759	1.526	30.425	35.527	32.211	24.391	26.632	22.885	29.569	24.594	34.927	10.922	13.343	11.434
TRINITY_DN17373_c0_g1_i1_orf1	thioredoxin domain-containing protein 11 isoform X3 [Ostrinia furnacalis]	1	180	20.5	9.88	6	1	1	High	1	0.89	1.096	1.13	1.367	1.176	0.969	1.194	1.135	1.119	1.188	0.859	1.198	0.994	1.176
TRINITY_DN9444_c0_g1_i3_orf1	hypothetical protein evm_007126 [Chilo suppressalis]	1	1726	196.2	6.55	1	1	1	High	1	0.676	0.965	0.712	0.654	0.524	0.721	0.628	0.567	0.515	0.523	0.683	0.825	0.723	0.642
TRINITY_DN1691_c0_g1_i3_orf1	uncharacterized protein LOC114354192 isoform X2 [Ostrinia furnacalis]	2	747	82.2	5.9	3	2	2	High	1	1.043	0.885	0.825	0.839	0.693	0.792	0.768	0.831	0.767	0.672	0.912	0.984	1.019	0.881
TRINITY_DN52051_c0_g1_i3_orf1	nischarin-like [Ostrinia furnacalis]	2	465	53.3	6.58	5	2	2	High	1	0.949	1.032	1.025	0.887	1.049	1.128	1.192	1.075	1.072	1.009	0.958	1.049	1.01	1.039
TRINITY_DN46090_c0_g2_i1_orf1	inactive tyrosine-protein kinase 7-like, partial [Ostrinia furnacalis]	1	148	16.7	4.79	6	1	1	High	1	1.155	0.95	0.92	0.895	1.002	0.856	0.9	0.833	0.805	0.759	0.811	2.187	1.76	1.58
TRINITY_DN108354_c0_g1_i1_orf1	WD repeat-containing protein 61-like [Ostrinia furnacalis]	1	327	35.8	5.34	2	1	1	High	1	1.024	1.004	0.558	0.595	0.551	0.608	0.679	0.617	0.539	0.426	0.5	0.627	0.677	0.611
TRINITY_DN18164_c0_g1_i7_orf1	uncharacterized protein LOC114366518 isoform X5 [Ostrinia furnacalis]	1	585	64.7	7.91	2	1	1	High	1	0.939	1.034	0.664	0.377	0.603	0.503	0.296	0.308	0.377	0.429	0.404	1.137	1.202	0.82
TRINITY_DN55148_c0_g1_i1_orf1	60S ribosomal protein L11 isoform 1 [Homo sapiens]	2	179	20.3	9.6	12	3	1	High	1	1.076	1.115	1.604	1.463	1.517	2.451	2.553	2.213	1.805	2.009	2.603	1.761	1.487	1.401
TRINITY_DN4194_c0_g1_i1_orf1	hornerin-like [Ostrinia furnacalis]	2	1807	184.6	6.8	1	2	2	High	1	1.111	0.877	0.387	0.324	0.497	0.554	0.637	0.835	0.339	0.313	0.412	1.01	1.112	0.866
TRINITY_DN135781_c0_g1_i1_orf1	vitamin K epoxide reductase complex subunit 1-like protein 1 [Ostrinia furnaca	1	155	17.2	8.34	5	1	1	High	1	0.943	0.979	1.181	1.129	1.219	1.285	1.257	1.308	1.123	1.076	1.24	0.944	0.958	0.97
TRINITY_DN346_c0_g1_i7_orf1	CDK-activating kinase assembly factor MAT1 [Ostrinia furnacalis]	1	311	35.7	5.38	5	1	1	High	1	1.415	1.082	1.941	1.769	1.749	2.05	2.046	2.063	2.247	2.352	2.714	1.749	1.698	1.538
TRINITY_DN3845_c1_g1_i4_orf1	protein jagunal [Ostrinia furnacalis]	1	197	23.2	6.9	6	1	1	High	1	0.777	0.879	1.032	0.859	0.923	0.885	1.011	1.027	0.943	1.32	0.747	0.816	0.809	0.905
TRINITY_DN21570_c0_g1_i1_orf1	ceramide synthase 5-like [Ostrinia furnacalis]	3	370	43.3	8.84	9	3	3	High	1	1.001	1.009	0.905	0.841	0.979	0.931	0.897	0.954	0.933	1.013	0.875	0.606	0.75	0.77
TRINITY_DN6244_c0_g1_i4_orf1	uncharacterized protein CG3556 [Ostrinia furnacalis]	1	471	52.3	6.8	2	1	1	High	1	1.194	1.443	1.516	1.63	1.604	1.547	1.561	1.48	1.326	1.448	1.74	2.082	2.129	1.628
TRINITY_DN65247_c1_g1_i1_orf1	beta-1,3-glucan-binding protein [Manduca sexta]	1	86	9.9	9.86	14	1	1	High	1	0.908	0.939	1.264	1.262	1.213	1.091	1.168	1.156	1.393	1.215	1.201	1.214	1.159	1.324
TRINITY_DN138086_c0_g1_i1_orf1	hypothetical protein evm_000614 [Chilo suppressalis]	2	110	12.8	6.29	22	2	2	High	1	1.109	1.138	1.792	2.252	2.018	1.824	2.143	1.562	1.538	1.652	2.003	1.121	1.253	1.241
TRINITY_DN9410_c0_g1_i4_orf1	protein RRP5 homolog [Ostrinia furnacalis]	1	520	60.2	7.43	3	1	1	High	1	0.893	0.914	0.644	0.676	0.559	0.556	0.656	0.52	0.523	0.698	0.568	0.554	0.505	0.452
TRINITY_DN136187_c0_g1_i2_orf1	U4/U6 small nuclear ribonucleoprotein Prp4-like [Ostrinia furnacalis]	1	71	7.8	6.35	17	1	1	High	1	0.998	0.888	1.077	0.903	0.939	0.99	1.111	1.06	0.909	1.284	0.843	1	0.871	0.842
TRINITY_DN44935_c0_g1_i1_orf1	unnamed protein product [Diatraea saccharalis]	1	103	11.5	5.2	14	1	1	High	1	0.87	0.888	1.021	0.94	1.03	0.87	1.04	1.135	0.925	1.525	1.081	1.013	1.042	1.034
TRINITY_DN57998_c1_g1_i1_orf1	uncharacterized protein LOC113509309, partial [Galleria mellonella]	1	77	9.1	5.5	14	2	1	High	1	1.146	1.128	1.261	1.371	1.319	1.232	1.031	1.089	1.558	1.257	1.635	5.526	6.395	4.866
TRINITY_DN24668_c0_g1_i8_orf1	uncharacterized protein LOC114364067 isoform X3 [Ostrinia furnacalis]	1	814	86.4	6.61	2	1	1	High	1	1.121	0.985	1.706	1.666	1.829	1.275	1.227	1.018	1.38	1.26	1.16	1.554	1.69	1.755
TRINITY_DN11823_c3_g2_i1_orf1	probable cytochrome P450 301a1, mitochondrial [Ostrinia furnacalis]	1	458	52.2	8.1	3	1	1	High	1	1.196	0.996	0.788	0.897	1.171	1.287	1.106	1.088	1.084	1.059	0.977	0.855	1.115	0.846
TRINITY_DN867_c0_g1_i1_orf1	hemicentin-2-like isoform X1 [Ostrinia furnacalis]	1	1247	138.3	6.49	1	1	1	High	1	0.946	1.054	0.908	0.734	0.971	0.742	0.716	0.689	0.818	0.935	0.996	2.101	1.95	1.884
TRINITY_DN8640_c0_g1_i4_orf1	glutathione S-transferase E14-like isoform X1 [Ostrinia furnacalis]	1	238	27.4	8.57	5	1	1	High	1	0.96	1.136	0.839	0.739	0.801	0.894	0.74	0.756	0.82	0.891	0.776	0.838	0.838	0.809
TRINITY_DN3773_c0_g1_i4_orf1	peptidyl-prolyl cis-trans isomerase G isoform X2 [Ostrinia furnacalis]	2	792	93.9	9.92	3	2	2	High	1	1.075	1	0.515	0.617	0.586	0.544	0.536	0.517	0.456	0.338	0.509	0.663	0.599	0.578
TRINITY_DN3227_c0_g1_i5_orf1	transmembrane emp24 domain-containing protein 5-like isoform X1 [Ostrinia	1	229	26.6	5.1	5	1	1	High	1	1.392	1.615	1.032	0.894	1.185	1.176	1.494	1.909	1.692	1.475	1.458	1.552	1.7	1.95
TRINITY_DN16965_c0_g2_i1_orf1	hypothetical protein evm_007405 [Chilo suppressalis]	1	273	27	9.95	3	2	1	High	1	0.932	1.019	0.538	0.57	0.503	0.506	0.609	0.537	0.415	0.435	0.446	0.731	0.739	0.721
TRINITY_DN6189_c0_g1_i1_orf1	optic atrophy 3 protein homolog isoform X2 [Ostrinia furnacalis]	1	262	29.7	8.7	3	1	1	High	1	1.006	0.926	0.458	0.536	0.482	0.535	0.617	0.531	0.522	0.586	0.567	0.767	0.886	0.811
TRINITY_DN6380_c0_g1_i1_orf1	THAP domain-containing protein 1-like isoform X1 [Ostrinia furnacalis]	1	342	39.4	5.25	3	1	1	High	1	1.137	0.91	0.732	0.8	0.703	0.668	0.689	0.735	0.811	0.633	0.575	0.94	0.842	0.912
TRINITY_DN86355_c0_g1_i1_orf1	uncharacterized protein LOC114358088 isoform X2 [Ostrinia furnacalis]	1	69	7.6	5.01	20	1	1	High	1	0.869	0.944	0.91	0.797	0.835	0.854	0.901	0.737	0.898	0.838	0.911	0.747	0.753	0.735
TRINITY_DN2803_c2_g1_i8_orf1	trans-1,2-dihydrobenzene-1,2-diol dehydrogenase-like isoform X1 [Ostrinia f	1	347	38.6	6.64	3	1	1	High	1	1.057	1.005	0.6	0.459	0.517	0.526	0.58	0.705	0.508	0.445	0.56	0.478	0.507	0.496
TRINITY_DN46132_c0_g2_i2_orf1	hypothetical protein evm_003834 [Chilo suppressalis]	1	1339	151	6.32	1	1	1	High	1	0.928	1.144	1.612	1.701	1.625	1.289	1.466	1.235	1.56	1.117	1.111	1.055	0.91	0.861
TRINITY_DN124950_c0_g2_i1_orf1	TATA box-binding protein-like protein 1 [Ostrinia furnacalis]	1	290	31.8	7.77	4	1	1	High	1	1.164	1.142	0.78	0.862	0.495	0.362	0.438	0.345	0.402	0.377	0.497	0.608	0.718	0.632
TRINITY_DN1380_c0_g1_i5_orf1	ubiquitin-fold modifier-conjugating enzyme 1 [Ostrinia furnacalis]	1	127	14.4	6.79	6	2	1	High	1	0.953	0.9	1.29	1.329	1.281	0.726	0.776	0.842	1.149	1.009	1.226	0.88	0.914	0.893
TRINITY_DN17671_c1_g2_i3_orf1	endoplasmic reticulum junction formation protein lunapark-A isoform X2 [Ostr	2	355	39.9	9.32	6	2	2	High	1	0.882	0.964	0.909	0.912	0.873	1.088	1.002	0.956	0.95	1.055	0.883	1.056	0.997	0.943
TRINITY_DN31751_c0_g1_i5_orf1	THO complex subunit 3 [Ostrinia furnacalis]	2	328	36.6	6.11	8	3	2	High	1	0.926	0.946	0.701	0.758	0.714	0.673	0.719	0.715	0.723	0.72	0.712	0.804	0.718	0.808
TRINITY_DN5997_c0_g1_i4_orf1	NEDD4 family-interacting protein 1-like isoform X1 [Ostrinia furnacalis]	1	219	24.6	5.69	5	2	1	High	1	0.857	0.954	0.911	0.969	0.958	0.817	0.892	0.841	0.865	0.796	0.896	1.037	0.949	0.952
TRINITY_DN21474_c0_g1_i1_orf1	motile sperm domain-containing protein 2-like [Ostrinia furnacalis]	1	93	10.9	6.58	13	1	1	High	1	0.8	0.967	0.938	0.797	0.822	1.177	0.887	1.083	1.323	1.449	0.814	1.046	0.936	0.954
TRINITY_DN26663_c0_g1_i4_orf1	phagocyte signaling-impaired protein [Ostrinia furnacalis]	2	592	67	7.17	4	2	2	High	1	0.863	0.936	1.051	1.038	0.912	1.065	0.951	1.061	0.959	0.931	0.882	0.809	0.719	0.837
TRINITY_DN2044_c0_g1_i5_orfp1	TRINITY_DN2044_c0_g1_i5_m.4210 TRINITY_DN2044_c0_g1::TRINITY_DN2044_	1	150	16.2	6.67	5	2	1	High	1	1.451	0.861	5.83	6.958	7.022	9.64	6.863	7.274	10.897	8.213	12.841	3.451	3.585	2.921
TRINITY_DN56110_c0_g1_i1_orf1	pescadillo homolog [Ostrinia furnacalis]	2	607	71.4	8.59	3	2	2	High	1	1.041	1.011	0.318	0.328	0.371	0.342	0.39	0.399	0.33	0.35	0.283	0.342	0.311	0.33
TRINITY_DN23570_c0_g1_i2_orf1	putative trypsin 6 [Ostrinia nubilalis]	1	240	26.1	7.31	4	1	1	High	1	0.891	1.007	0.285	0.22	0.269	0.212	0.24	0.244	0.256	0.194	0.247	0.278	0.252	0.281
TRINITY_DN125441_c0_g1_i5_orf1	KH domain-containing, RNA-binding, signal transduction-associated protein 2	2	85	9.8	6.15	24	2	2	High	1	1.022	1.004	2.016	2.162	1.891	1.723	1.534	1.886	1.738	1.765	1.785	2.153	2.119	2.231

TRINITY_DN1875_c0_g1_i1_orf1	uncharacterized protein LOC114366320 isoform X1 [Ostrinia furnacalis]	2	441	49.3	5.57	7	2	2	High	1	1.069	1.058	0.697	0.67	0.694	0.842	0.679	0.861	0.516	0.514	0.573	0.752	0.841	0.799
TRINITY_DN747_c0_g2_i1_orf1	trypsin, alkaline C-like [Ostrinia furnacalis]	1	272	30.5	8.29	5	1	1	High	1	0.843	0.842	0.317	0.449	0.447	0.573	0.514	0.428	0.337	1.06	0.475	0.511	0.487	0.471
TRINITY_DN23229_c0_g1_i2_orf1	uncharacterized protein LOC114362553 [Ostrinia furnacalis]	1	509	56.8	6.74	2	1	1	High	1	1.1	0.996	1.292	1.238	1.147	1.745	1.684	1.456	1.725	1.367	1.17	2.191	2.347	2.297
TRINITY_DN13563_c0_g1_i1_orf1	Golgi resident protein GCP60 isoform X1 [Ostrinia furnacalis]	1	482	55.3	5.05	4	1	1	High	1	1.031	1.044	1.448	1.585	1.438	1.534	1.447	1.432	1.112	1.039	1.063	1.118	0.983	1.019
TRINITY_DN42506_c0_g1_i1_orf1	28S ribosomal protein S7, mitochondrial [Ostrinia furnacalis]	1	234	27.3	9.66	4	1	1	High	1	1.085	1.046	0.393	0.346	0.436	0.425	0.441	0.49	0.486	0.363	0.32	0.579	0.476	0.576
TRINITY_DN6656_c0_g1_i1_orf1	sorting and assembly machinery component 50 homolog isoform X9 [Ostrinia	2	464	49.8	6.93	3	2	2	High	1	1.05	1.036	0.886	0.937	0.814	0.892	0.968	0.918	0.897	0.755	0.773	1.518	1.535	1.55
TRINITY_DN240_c0_g1_i4_orf1	unnamed protein product [Chilo suppressalis]	1	2364	260.4	6.61	1	1	1	High	1	1.023	1.162	0.866	1.077	0.983	0.77	0.965	0.987	0.792	0.859	0.664	0.946	0.767	0.847
TRINITY_DN12771_c0_g1_i1_orf1	histone acetyltransferase type B catalytic subunit [Ostrinia furnacalis]	2	404	46.4	6.02	3	2	2	High	1	0.987	1.057	0.722	0.806	0.815	0.705	0.752	0.703	0.964	0.832	0.881	1.052	1.257	1.117
TRINITY_DN7964_c0_g1_i1_orfp1	TRINITY_DN7964_c0_g1_i1_m.23483 TRINITY_DN7964_c0_g1::TRINITY_DN7964	2	78	8.7	6.04	17	2	2	High	1	1.067	1.033	0.337	0.286	0.351	0.468	0.347	0.423	0.431	0.342	0.37	0.404	0.392	0.302
TRINITY_DN277_c0_g1_i5_orf1	uncharacterized protein LOC114363802 isoform X2 [Ostrinia furnacalis]	1	312	34.4	9.52	4	3	1	High	1	0.986	0.964	0.839	0.988	0.831	0.801	0.766	0.869	0.76	1.012	0.8	0.811	0.755	0.876
TRINITY_DN119919_c0_g2_i1_orf1	PREDICTED: rho-associated protein kinase 2 isoform X3 [Fopius arisanus]	1	100	11.1	5.45	13	1	1	High	1	0.982	1.133	1.033	1.062	1.143	1.053	1.213	1.095	1.129	1.644	1.079	1.248	1.154	1.21
TRINITY_DN19814_c0_g1_i4_orf1	general odorant-binding protein 28a-like [Ostrinia furnacalis]	1	153	16.7	7.9	5	1	1	High	1	1.054	0.976	0.555	0.617	0.622	0.451	0.528	0.513	0.363	0.348	0.334	0.296	0.262	0.254
TRINITY_DN24391_c1_g1_i1_orf1	ER membrane protein complex subunit 7 [Ostrinia furnacalis]	1	228	26	8.88	8	1	1	High	1	0.992	0.984	0.838	0.828	0.81	0.837	0.89	0.805	0.879	0.883	0.988	1.004	1.083	1.192
TRINITY_DN9094_c0_g1_i1_orf1	uncharacterized protein LOC114356316 [Ostrinia furnacalis]	1	525	60.2	6.86	3	1	1	High	1	0.883	0.971	0.548	0.646	0.651	0.684	0.453	0.551	0.55	0.67	0.593	0.494	0.563	0.606
TRINITY_DN67243_c0_g1_i1_orf1	39S ribosomal protein L3, mitochondrial [Ostrinia furnacalis]	1	357	40.4	9.69	4	1	1	High	1	0.93	0.715	1.385	1.595	1.86	1.484	1.953	1.573	1.483	1.865	1.619	1.202	1.251	1.203
TRINITY_DN1230_c1_g1_i5_orf1	uncharacterized protein LOC114353440 [Ostrinia furnacalis]	1	313	35.6	8.37	3	1	1	High	1	0.942	0.806	2.169	2.224	2.066	2.299	2.287	2.321	1.875	1.786	1.872	1.046	0.898	1.204
TRINITY_DN34821_c0_g1_i4_orf1	acetylcholine receptor subunit alpha-L1-like [Ostrinia furnacalis]	1	415	47.7	6.74	3	1	1	High	1	0.924	1.034	0.321	0.303	0.315	0.403	0.443	0.435	0.495	0.401	0.424	0.945	1.047	0.949
TRINITY_DN15578_c0_g2_i1_orfp1	uncharacterized protein LOC125235519 [Leguminivora glycinivorella]	1	106	10.9	8.19	9	1	1	High	1	1.012	1.26	4.536	4.278	4.56	2.717	2.574	2.876	3.919	3.397	3.544	3.739	4.226	4.577
TRINITY_DN7881_c1_g1_i5_orf1	8-oxo-dGDP phosphatase NUDT18 [Ostrinia furnacalis]	1	333	36.9	6.38	4	1	1	High	1	1.102	0.946	0.977	1.259	1.042	0.92	0.949	1.102	0.981	1.022	1.045	1.127	1.115	1.11
TRINITY_DN2311_c0_g3_i1_orf1	uncharacterized protein LOC114364231 isoform X1 [Ostrinia furnacalis]	1	366	41.4	5.92	2	2	1	High	1	0.979	0.921	1.395	1.314	1.533	2.267	2.218	1.953	1.415	1.612	1.498	4.518	4.914	4.253
TRINITY_DN6351_c0_g1_i4_orf1	cytochrome P450 CYP12A2-like [Ostrinia furnacalis]	2	503	57.5	8.76	4	2	2	High	1	1.009	0.969	0.475	0.499	0.518	0.545	0.451	0.481	0.584	0.382	0.536	0.371	0.395	0.37
TRINITY_DN1675_c0_g1_i1_orf1	DNA-directed RNA polymerase II subunit Rpb4 [Trichoplusia ni]	1	138	15.9	4.68	7	1	1	High	1	0.942	1.009	1.098	1.008	0.934	1.033	0.902	0.91	0.97	0.884	1.034	1.085	1.187	1.166
TRINITY_DN1567_c0_g1_i15_orf1	probable dual specificity protein kinase madd-3 isoform X1 [Ostrinia furnacalis	1	1012	114.1	5.14	1	1	1	High	1	1.123	1.168	0.681	0.646	0.655	0.594	0.593	0.532	0.485	0.641	0.724	0.661	0.734	0.52
TRINITY_DN20614_c0_g1_i1_orf1	hypothetical protein evm_003552 [Chilo suppressalis]	2	1301	143	8.24	1	2	2	High	1	0.681	0.68	0.865	0.765	1.041	0.94	1.985	1.622	1.874	2.013	0.868	1.936	1.611	2.49
TRINITY_DN57749_c0_g1_i4_orf1	LOW QUALITY PROTEIN: DENN domain-containing protein Crag [Ostrinia furn	1	912	101.8	8.07	1	1	1	High	1	0.888	0.975	0.934	1.089	0.934	1.206	1.263	1.268	0.818	0.904	0.751	0.945	0.844	0.972
TRINITY_DN2400_c0_g1_i1_orf1	uncharacterized protein LOC114351021 [Ostrinia furnacalis]	1	113	12.9	8.27	11	1	1	High	1	1.095	1.009	1.18	1.243	1.519	1.038	1.368	1.326	1.111	1.217	1.116	5.975	5.801	5.651
TRINITY_DN895_c0_g2_i1_orf1	protein N-terminal asparagine amidohydrolase [Cotesia glomerata]	1	314	34.8	5.72	3	1	1	High	1	0.991	0.869	1.474	1.222	1.446	1.125	1.12	1.366	1.157	1.299	1.003	1.901	1.824	1.898
TRINITY_DN19687_c0_g1_i1_orf1	probable ribosome production factor 1 [Ostrinia furnacalis]	1	280	33	9.7	4	1	1	High	1	0.927	0.978	0.539	0.494	0.564	0.703	0.621	0.686	0.84	0.771	0.593	0.735	0.642	0.673
TRINITY_DN12392_c0_g1_i3_orf1	protein KIAA0100 [Pectinophora gossypiella]	1	1670	187.5	7.39	1	1	1	High	1	0.953	1.174	1.118	1.137	1.212	1.161	1.351	1.409	1.088	1.529	1.3	0.946	1.107	1.065
TRINITY_DN142217_c0_g1_i1_orf1	aldehyde dehydrogenase, mitochondrial [Cotesia glomerata]	1	510	55.5	6.76	2	1	1	High	1	1.024	1.007	1.06	1.024	1.051	1.151	1.198	1.238	0.895	1.24	0.678	0.976	1.219	0.983
TRINITY_DN14057_c0_g1_i3_orf1	bumetanide-sensitive sodium-(potassium)-chloride cotransporter-like [Ostrini	1	1160	127.3	7.91	1	1	1	High	1	0.981	0.969	0.972	1.047	1.338	0.859	1.039	1.098	0.981	1.107	1.143	0.968	1.104	0.945
TRINITY_DN5422_c0_g1_i1_orf1	nitrilase and fragile histidine triad fusion protein NitFhit isoform X1 [Ostrinia fu	1	304	34.2	8.69	3	1	1	High	1	1.256	1.154	1.241	1.236	1.216	1.248	1.363	1.486	0.875	0.749	0.827	1.387	1.563	1.445
TRINITY_DN5406_c0_g2_i1_orf1	uncharacterized protein LOC114350326 [Ostrinia furnacalis]	1	138	15.6	7.06	9	1	1	High	1	1.108	1.289	5.612	5.023	4.742	3.659	3.486	2.898	3.927	3.624	3.321	5.994	5.387	5.781
TRINITY_DN17329_c0_g2_i3_orf1	uncharacterized protein LOC114354338 isoform X1 [Ostrinia furnacalis]	2	1333	150.8	7.96	2	2	2	High	1	1.115	1.033	0.974	0.984	0.97	1.025	1.011	0.971	1.35	1.237	1.467	1.735	1.49	1.552
TRINITY_DN16830_c0_g1_i5_orf1	adrenodoxin [Ostrinia furnacalis]	1	158	17.6	5.14	6	1	1	High	1	1.089	0.963	0.324	0.342	0.38	0.366	0.397	0.332	0.256	0.319	0.342	0.396	0.4	0.349
TRINITY_DN1038_c1_g1_i3_orf1	uncharacterized protein LOC114363583 [Ostrinia furnacalis]	1	100	11	4.79	11	2	1	High	1	1.011	1.175	1.911	2.141	1.902	1.787	1.657	2.055	1.879	1.472	1.55	1.001	0.997	0.903
TRINITY_DN31_c0_g1_i3_orfp1	TRINITY_DN31_c0_g1_i3_m.1394 TRINITY_DN31_c0_g1::TRINITY_DN31_c0_g1_i	2	117	12.6	5	15	3	2	High	1	0.9	0.983	1.016	0.738	0.79	1.754	1.168	1.374	1.238	1.087	1.042	4.916	4.655	4.741
TRINITY_DN6388_c0_g1_i1_orf1	arf-GAP with SH3 domain, ANK repeat and PH domain-containing protein 1-li	1	193	21.2	4.98	8	1	1	High	1	0.993	1.011	1.137	1.157	1.135	0.981	1.073	0.955	1.044	1.163	1.025	1.014	1.034	1.04
TRINITY_DN129226_c0_g4_i1_orf1	hypothetical protein evm_000268 [Chilo suppressalis]	1	356	39.3	5.16	4	1	1	High	1	0.891	0.943	0.558	0.507	0.59	0.742	0.603	0.704	0.926	0.712	0.611	0.733	0.9	0.769
TRINITY_DN110519_c0_g1_i1_orf1	uncharacterized protein LOC114366601 [Ostrinia furnacalis]	1	202	20	9.63	5	1	1	High	1	0.949	0.985	0.824	0.751	0.748	0.875	0.883	1.091	0.984	0.709	0.825	2.724	3.151	3.161
TRINITY_DN4802_c0_g1_i4_orf1	uncharacterized protein LOC114366345 isoform X2 [Ostrinia furnacalis]	1	90	9.9	8.03	12	1	1	High	1	0.921	0.918	2.81	2.864	3.02	5.277	5.325	5.385	2.145	2.093	2.505	1.69	1.86	1.441
TRINITY_DN19807_c0_g1_i1_orf1	DNA-directed RNA polymerase I subunit RPA1 [Ostrinia furnacalis]	2	1654	186.7	7.91	1	2	2	High	1	1.066	1.105	1.262	1.359	1.367	1.308	1.26	1.447	1.466	1.313	1.275	1.168	1.073	1.14
TRINITY_DN3702_c0_g1_i1_orf1	U4/U6.U5 tri-snRNP-associated protein 2 [Ostrinia furnacalis]	1	483	56	8.21	2	1	1	High	1	1.024	0.95	0.739	0.871	0.791	0.69	0.741	0.679	0.465	0.444	0.491	0.8	0.846	0.752
TRINITY_DN54150_c0_g1_i1_orf1	uncharacterized protein LOC114351648 [Ostrinia furnacalis]	1	231	25.9	5.58	6	1	1	High	1	0.933	1.013	0.43	0.494	0.548	0.463	0.572	0.591	0.583	0.557	0.573	0.599	0.472	0.599
TRINITY_DN5046_c0_g3_i1_orf1	uncharacterized protein LOC114358520 [Ostrinia furnacalis]	2	1239	139.7	5.25	2	2	2	High	1	0.924	0.966	0.48	0.596	0.534	0.583	0.607	0.558	0.541	0.532	0.465	0.832	0.913	0.762
TRINITY_DN46372_c0_g2_i1_orf1	basic salivary proline-rich protein 1 isoform X2 [Ostrinia furnacalis]	1	224	23.9	7.37	5	1	1	High	1	1.205	1.198	1.099	1.285	1.008	0.961	0.985	0.751	0.79	0.781	0.887	0.648	0.687	0.521
TRINITY_DN43328_c0_g1_i1_orf1	tubulin--tyrosine ligase-like protein 12 [Ostrinia furnacalis]	1	613	71.9	5.07	2	1	1	High	1	1.112	0.969	0.382	0.446	0.591	0.604	0.485	0.345	0.429	0.391	0.414	1.081	1.068	1.107
TRINITY_DN1273_c0_g1_i4_orf1	hypothetical protein evm_006077 [Chilo suppressalis]	2	685	78.5	8.35	2	2	2	High	1	1.018	1.078	0.882	0.923	0.861	0.949	0.678	0.92	0.867	0.84	0.756	0.987	0.904	0.88
TRINITY_DN2611_c0_g1_i10_orf1	ancient ubiquitous protein 1-like [Hyposmocoma kahamanoa]	2	397	44.4	9.47	5	2	2	High	1	0.977	0.966	0.8	0.952	0.898	0.773	0.864	0.844	0.747	0.661	0.866	0.926	0.825	0.872
TRINITY_DN466_c0_g1_i5_orf1	transformer-2 protein homolog beta isoform X13 [Ostrinia furnacalis]	1	274	32.2	11.17	5	1	1	High	1	0.983	1.068	0.744	0.809	0.854	0.736	0.887	0.974	0.908	0.89	0.771	0.683	0.621	0.862
TRINITY_DN4686_c0_g2_i1_orf1	lysophospholipase-like protein 1 [Ostrinia furnacalis]	2	239	26.7	6.92	9	2	2	High	1	0.974	1.003	0.689	0.703	0.673	0.669	0.706	0.689	0.673	0.685	0.541	0.969	0.949	1.014
TRINITY_DN20067_c0_g1_i6_orf1	hypothetical protein evm_010712 [Chilo suppressalis]	1	143	17	5.1	6	1	1	High	1	1.108	0.974	0.763	0.681	0.773	0.803	0.826	0.757	0.802	0.751	0.721	1.087	1.098	1.113
TRINITY_DN2691_c0_g1_i1_orf1	WD repeat-containing protein 18 [Ostrinia furnacalis]	1	444	49.4	6.76	4	1	1	High	1	0.985	1.041	0.485	0.493	0.575	0.582	0.476	0.544	0.579	0.534	0.452	0.525	0.449	0.463
TRINITY_DN10458_c0_g1_i1_orf1	V-type proton ATPase 21 kDa proteolipid subunit [Ostrinia furnacalis]	1	205	21.6	8.73	5	1	1	High	1	1.029	1.13	0.464	0.359	0.465	0.467	0.579	0.606	0.519	0.499	0.48	0.347	0.437	0.429
TRINITY_DN5569_c0_g1_i1_orf1	PREDICTED: chromatin complexes subunit BAP18 isoform X1 [Eufriesea mexica	1	156	17	5.07	6	1	1	High	1	0.791	0.793	0.976	0.941	0.873	0.807	0.823	1.174	0.906	0.855	0.766	1.069	1.065	1.083
TRINITY_DN115210_c0_g4_i1_orf1	PREDICTED: CAD protein [Microplitis demolitor]	1	1596	177.3	6.9	1	1	1	High	1	1.028	1.292	1.131	0.984	1.117	1.449	1.369	1.327	1.068	1.009	1.071	1.346	1.351	1.253
TRINITY_DN52761_c0_g2_i1_orf1	atlastin-like isoform X4 [Ostrinia furnacalis]	1	77	8.8	6.39	12	1	1	High	1	0.908	0.909	1.485	1.586	1.659	1.793	1.817	1.708	1.217	1.081	1.084	1.286	1.218	1.207
TRINITY_DN34153_c0_g2_i2_orf1	uncharacterized protein LOC114351567 [Ostrinia furnacalis]	1	151	17.4	5.24	5	1	1	High	1	1.086	1.069	1.26	1.199	1.36	1.342	1.444	1.273	1.163	1.169	1.139	1.067	1.06	1.147
TRINITY_DN65604_c0_g1_i2_orf1	LOW QUALITY PROTEIN: ankyrin repeat domain-containing protein 17 [Ostrini	2	866	92.8	9.35	3	2	2	High	1	0.993	1.001	0.675	0.674	0.727	0.756	0.744	0.696	0.766	0.733	0.754	0.727	0.779	0.755
TRINITY_DN12858_c0_g1_i5_orf1	unnamed protein product, partial [Iphiclides podalirius]	1	258	28	5.82	5	1	1	High	1	1.129	0.932	0.807	0.715	0.855	0.757	0.694	0.702	0.653	0.735	0.659	1.171	1.047	1.057
TRINITY_DN37538_c0_g4_i1_orf1	esterase FE4-like [Ostrinia furnacalis]	1	110	12.7	4.46	12	1	1	High	1	1.009	0.98	0.404	0.355	0.337	0.393	0.414	0.384	0.417	0.292	0.407	0.495	0.522	0.519
TRINITY_DN14242_c0_g1_i2_orfp1	TRINITY_DN14242_c0_g1_i2_m.18449 TRINITY_DN14242_c0_g1::TRINITY_DN14	1	147	16	5.15	8	1	1	High	1	1.386	1.618	23.089	24.022	23.09	12.447	8.38	9.395	15.959	17.239	22.679	9.949	8.446	10.902
TRINITY_DN1313_c0_g1_i2_orf1	39S ribosomal protein L40, mitochondrial [Ostrinia furnacalis]	1	203	23.8	9.48	11	1	1	High	1	1.019	1.099	0.481	0.469	0.413	0.405	0.418	0.446	0.554	0.595	0.584	1.561	1.419	1.363
TRINITY_DN63943_c0_g1_i5_orf1	uncharacterized protein LOC114357144 [Ostrinia furnacalis]	1	316	34.9	5.01	3	1	1	High	1	1.186	1.082	1.417	1.636	1.305	1.47	1.722	1.498	1.804	1.308	1.117	1.72	1.632	1.795
TRINITY_DN29934_c0_g1_i6_orf1	sodium/potassium-transporting ATPase subunit beta-2-like [Ostrinia furnacali:	1	326	37.5	8.62	4	1	1	High	1	1.223	1.117	0.636	0.611	0.542	0.296	0.255	0.349	0.254	0.288	0.32	0.632	0.581	0.554
TRINITY_DN109144_c0_g1_i5_orf1	uncharacterized protein LOC126369165 [Pectinophora gossypiella]	1	87	10.3	9.57	11	1	1	High	1	0.833	0.835	0.467	0.476	0.54	0.606	0.63	0.541	0.507	0.574	0.561	0.568	0.525	0.523
TRINITY_DN2441_c0_g1_i1_orf1	protein RFT1 homolog [Ostrinia furnacalis]	2	560	63.8	8.7	3	2	1	High	1	1.267	1.177	0.866	0.713	0.949	0.898	0.9	0.965	0.751	0.78	0.692	0.868	0.896	0.903
TRINITY_DN23264_c0_g1_i1_orf1	U5 small nuclear ribonucleoprotein 40 kDa protein [Ostrinia furnacalis]	1	348	38.8	8.09	3	1	1	High	1	1.043	1.026	0.8	0.698	0.792	0.612	0.573	0.572	0.548	0.48	0.549	0.735	0.68	0.708
TRINITY_DN4622_c0_g1_i1_orf1	keratin-associated protein 19-2-like [Ostrinia furnacalis]	1	218	22.1	8.13	6	1	1	High	1	1.034	0.977	0.632	0.656	0.728	0.576	0.628	0.56	0.582	0.577	0.664	0.407	0.398	0.475
TRINITY_DN7258_c0_g1_i2_orf1	mitochondrial glutamate carrier 1-like [Ostrinia furnacalis]	1	314	34.1	9.66	4	1	1	High	1	1.092	0.884	0.854	0.812	0.779	1.042	0.9	0.816	0.937	0.923	0.674	0.929	0.788	0.877
TRINITY_DN628_c0_g1_i7_orf1	prostamide/prostaglandin F synthase-like [Ostrinia furnacalis]	2	236	26.6	9.42	8	2	2	High	1	0.908	0.931	0.343	0.349	0.361	0.309	0.312	0.319	0.37	0.392	0.368	0.473	0.487	0.483
TRINITY_DN11388_c0_g1_i4_orf1	limbic system-associated membrane protein-like, partial [Ostrinia furnacalis]	1	458	51.3	5.68	2	1	1	High	1	1.108	1.166	1.487	1.413	1.424	1.438	1.472	1.447	1.649	1.462	1.935	0.828	0.801	0.726
TRINITY_DN26355_c0_g1_i4_orf1	small integral membrane protein 12 [Ostrinia furnacalis]	1	87	10	6.71	14	1	1	High	1	1.094	1.012	0.612	0.539	0.52	0.557	0.604	0.456	0.463	0.501	0.623	0.865	0.72	0.7
TRINITY_DN34413_c0_g1_i1_orf1	LOW QUALITY PROTEIN: probable RNA methyltransferase bin3 [Ostrinia furnac	2	782	87.4	9.42	2	2	2	High	1	0.997	0.879	0.886	0.964	0.88	0.864	0.89	0.782	0.752	0.555	0.787	0.775	0.799	0.717
TRINITY_DN8644_c0_g1_i3_orf1	SEC14-like protein 2 [Ostrinia furnacalis]	1	413	46.9	7.44	2	1	1	High	1	1.044	1.006	1.659	1.906	1.806	1.204	1.426	1.175	1.128	0.879	1.49	1.443	1.426	1.362
TRINITY_DN11596_c0_g1_i1_orf1	probable ATP-dependent RNA helicase DDX47 [Ostrinia furnacalis]	1	462	51.6	7.15	3	1	1	High	1	1.066	0.835	0.734	0.875	0.87	0.796	0.961	0.882	1.043	0.966	1.023	0.883	0.823	0.752
TRINITY_DN29541_c0_g1_i1_orf1	kinesin-like protein KIF23 [Ostrinia furnacalis]	1	871	98	9.09	1	1	1	High	1	1.126	1.01	1.414	1.542	1.738	1.959	8.105	2.681	1.989	3.599	1.795	2.247	2.915	5.149
TRINITY_DN2655_c0_g2_i1_orf1	DNA fragmentation factor subunit alpha [Ostrinia furnacalis]	1	197	22	4.7	5	1	1	High	1	1.055	0.881	0.831	0.867	0.869	0.993	1.443	1.158	1.148	1.18	1.357	1.071	0.952	0.999
TRINITY_DN27033_c1_g1_i3_orfp1	poly(U)-specific endoribonuclease homolog [Ostrinia furnacalis]	1	51	5.8	9.7	18	1	1	High	1	0.945	0.944	0.982	0.661	1.069	1.379	1.365	1.51	1.434	1.98	0.922	1.246	0.956	1.158
TRINITY_DN7992_c0_g1_i4_orf1	uncharacterized protein LOC114362579 [Ostrinia furnacalis]	2	730	80.6	6.8	2	2	2	High	1	1.031	1.02	1.318	1.478	1.343	1.294	1.34	1.368	1.328	1.167	1.249	1.233	1.313	1.294
TRINITY_DN47914_c0_g2_i1_orf1	UBX domain-containing protein 1-A-like [Ostrinia furnacalis]	1	233	26	5.52	3	1	1	High	1	1.064	0.969	0.852	0.823	0.89	0.732	0.735	0.791	0.803	0.742	0.767	1.076	1.169	1.226
TRINITY_DN16900_c0_g2_i1_orf1	uncharacterized oxidoreductase dhs-27-like [Ostrinia furnacalis]	1	271	32.1	6.11	3	1	1	High	1	1.191	1.064	0.318	0.237	0.213	0.371	0.359	0.364	0.391	0.332	0.316	0.395	0.263	0.344
TRINITY_DN17615_c0_g1_i3_orf1	hypothetical protein SFRUCORN_008858 [Spodoptera frugiperda]	1	75	8.4	5.97	20	1	1	High	1	1.076	1.192	4.47	4.759	5.087	5.854	6.225	5.409	4.501	4.73	5.712	2.773	3.617	3.589
TRINITY_DN65988_c0_g1_i4_orf1	uncharacterized protein LOC114354070 isoform X3 [Ostrinia furnacalis]	1	96	10.6	4.78	9	1	1	High	1	1.078	1.031	0.791	0.776	0.799	0.706	0.746	0.697	0.655	0.601	0.689	0.75	0.761	0.686
TRINITY_DN6439_c0_g1_i1_orf1	GPI mannosyltransferase 3 isoform X4 [Ostrinia furnacalis]	1	504	57.2	9.31	3	1	1	High	1	0.958	0.928	0.592	0.613	0.391	0.537	0.372	0.548	0.484	0.412	0.337	0.728	0.624	0.742
TRINITY_DN44792_c0_g1_i1_orf1	parafibromin [Ostrinia furnacalis]	1	549	61.5	9.72	3	1	1	High	1	0.957	0.942	0.74	0.772	0.662	0.721	0.857	0.687	0.83	1.052	0.817	1.132	1.029	1.31
TRINITY_DN38471_c0_g2_i1_orf1	cytochrome c oxidase assembly protein COX20, mitochondrial [Ostrinia furnaci	1	122	13.9	8.4	9	1	1	High	1	0.881	0.859	0.72	0.605	0.56	0.672	0.837	0.623	0.655	1.525	0.63	0.658	0.674	0.688
TRINITY_DN16840_c1_g1_i1_orf1	attacin-like [Ostrinia furnacalis]	1	204	22.2	9.99	6	1	1	High	1	1.173	1.089	2.113	1.96	2.019	2.815	2.573	2.48	1.366	1.337	1.668	2.134	2.124	1.923
TRINITY_DN862_c0_g1_i4_orf1	uncharacterized protein LOC114359380 isoform X1 [Ostrinia furnacalis]	1	230	26.3	9.11	8	1	1	High	1	0.928	1.043	1.07	1.382	1.219	1.043	1.147	1.386	1.161	1.479	1.557	2.126	1.625	1.71
TRINITY_DN86580_c0_g1_i1_orf1	microtubule-actin cross-linking factor 1 isoform X15 [Ostrinia furnacalis]	1	130	14.6	5.05	7	1	1	High	1	0.953	0.949	0.822	0.892	0.813	0.852	0.947	0.849	0.844	0.763	0.76	0.837	0.984	0.899
TRINITY_DN34406_c0_g2_i9_orfp1	TRINITY_DN34406_c0_g2_i9_m.33755 TRINITY_DN34406_c0_g2::TRINITY_DN34	1	81	9.3	9.09	11	1	1	High	1	0.702	0.624	7.68	8.685	6.786	3.269	3.114	3.555	1.091	1.308	0.779	5.226	4.98	4.67
TRINITY_DN40823_c0_g1_i1_orf1	nucleolar protein 10 [Ostrinia furnacalis]	1	663	76.2	8.06	2	1	1	High	1	1.178	1.125	0.762	0.82	0.827	0.655	0.824	0.829	0.713	0.79	0.702	0.911	0.877	0.826
TRINITY_DN141462_c0_g1_i1_orf1	mitochondrial-processing peptidase subunit beta [Diachasma alloeum]	1	477	53.3	6.42	2	3	1	High	1	1.018	0.998	0.28	0.251	0.319	0.265	0.225	0.268	0.247	0.231	0.239	0.244	0.244	0.231
TRINITY_DN18196_c0_g1_i4_orf1	uncharacterized protein LOC114363471 isoform X3 [Ostrinia furnacalis]	1	354	38.5	7.66	4	1	1	High	1	0.921	0.781	1.83	1.975	1.581	1.564	1.715	1.589	1.765	1.867	1.404	1.385	1.209	0.912

TRINITY_DN9207_c0_g1_i1_orf1	RNA polymerases N / 8 kDa subunit domain-containing protein [Phthorimaea	1	75	8.7	8.47	12	1	1	High	1	1.078	0.958	0.617	0.505	0.577	0.658	0.683	0.759	0.833	0.821	0.489	0.59	0.537	0.64
TRINITY_DN1391_c1_g2_i4_orf1	hypothetical protein SFRURICE_002236 [Spodoptera frugiperda]	2	450	50.6	8.15	5	2	2	High	1	1.008	1.046	0.925	0.704	0.852	0.809	0.844	0.804	0.788	0.646	0.681	0.704	0.745	0.735
TRINITY_DN41573_c0_g1_i1_orf1	BRISC and BRCA1-A complex member 2-like [Ostrinia furnacalis]	1	371	42.2	4.97	4	1	1	High	1	1.245	1.282	1.632	2.147	2.59	2.338	2.658	0.986	0.97	1.642	3.374	0.87	0.826	0.625
TRINITY_DN2606_c0_g1_i5_orf1	galectin-4-like isoform X1 [Ostrinia furnacalis]	1	369	41.6	5.22	4	1	1	High	1	1.031	1.125	1.554	1.784	1.702	2.01	1.688	1.703	1.556	2.169	1.915	1.921	1.762	1.984
TRINITY_DN24218_c0_g1_i1_orf1	uncharacterized protein LOC114362624 [Ostrinia furnacalis]	1	496	56.4	7.4	2	1	1	High	1	0.946	0.935	1.009	0.902	1.048	0.777	0.799	0.806	0.688	0.76	0.579	1.184	1.218	1.304
TRINITY_DN32022_c0_g1_i1_orf1	striatin isoform X1 [Diachasma alloeum]	1	723	80	5.21	1	1	1	High	1	0.88	0.985	0.619	0.66	0.578	0.536	0.44	0.565	0.46	0.398	0.489	0.648	0.673	0.586
TRINITY_DN2907_c0_g2_i4_orf1	nucleoporin NUP188 homolog isoform X1 [Ostrinia furnacalis]	1	1832	207.9	6.9	1	1	1	High	1	0.953	1.049	0.865	0.98	0.859	0.85	0.919	0.938	0.771	0.678	0.844	0.918	0.829	0.837
TRINITY_DN6205_c0_g1_i4_orfp1	TRINITY_DN6205_c0_g1_i4_m.72677 TRINITY_DN6205_c0_g1::TRINITY_DN6205	1	67	7.2	8.53	13	1	1	High	1	1.037	1.059	1.493	1.527	1.597	1.071	0.813	1.061	0.944	0.862	1.036	1.738	1.706	1.564
TRINITY_DN40_c0_g2_i1_orf1	trypsin CFT-1-like [Ostrinia furnacalis]	1	262	28.5	7.44	3	1	1	High	1	1.01	1.06	0.313	0.261	0.351	0.312	0.36	0.306	0.295	0.297	0.304	0.242	0.219	0.287
TRINITY_DN7647_c0_g1_i4_orf1	E3 ubiquitin-protein ligase Bre1 isoform X6 [Ostrinia furnacalis]	1	921	105.9	6.84	1	1	1	High	1	1.144	1.284	0.774	0.805	0.692	0.609	0.637	0.462	0.591	0.457	0.585	0.833	0.733	0.6
TRINITY_DN6084_c0_g1_i4_orf1	unconventional myosin-Va isoform X1 [Manduca sexta]	2	1612	184.4	9.13	2	2	2	High	1	0.858	1.025	0.776	0.649	0.669	0.783	0.787	0.829	0.704	0.776	0.75	0.825	0.895	0.754
TRINITY_DN8432_c0_g2_i1_orf1	unnamed protein product [Chrysodeixis includens]	1	572	62.7	5.74	3	1	1	High	1	0.851	0.86	0.57	0.614	0.576	0.39	0.636	0.543	0.52	0.842	0.495	0.895	0.884	0.716
TRINITY_DN4021_c0_g1_i1_orf1	leech-derived tryptase inhibitor C-like [Ostrinia furnacalis]	1	66	7.2	8.73	11	1	1	High	1	1.08	1.055	1.014	1.091	1.253	0.846	1.267	1.076	0.836	0.623	0.7	7.663	8.807	7.751
TRINITY_DN356_c2_g1_i3_orf1	scavenger receptor class B member 1 isoform X2 [Pectinophora gossypiella]	1	515	57.7	6.14	3	1	1	High	1	0.831	1.078	1.38	1.898	1.526	1.489	1.764	1.45	1.46	1.838	1.388	1.266	0.999	1.196
TRINITY_DN7289_c0_g1_i1_orf1	nuclear cap-binding protein subunit 2 [Ostrinia furnacalis]	1	161	18.8	8.51	9	1	1	High	1	1.029	1.125	0.696	0.852	0.605	0.429	0.484	0.598	0.508	0.572	0.437	0.611	0.607	0.481
TRINITY_DN16899_c0_g2_i1_orf1	serine/threonine-protein kinase GA29083 [Ostrinia furnacalis]	1	678	74.4	9.16	2	1	1	High	1	0.983	0.937	0.89	0.901	0.815	0.848	0.949	1.056	0.759	0.78	0.852	0.842	0.969	0.88
TRINITY_DN18216_c0_g1_i4_orf1	golgin subfamily A member 7 [Ostrinia furnacalis]	1	161	18.5	6.67	6	1	1	High	1	1.108	1.009	1.37	1.255	1.249	1.085	1.164	1.163	1.091	0.975	0.91	1.017	1.09	1.109
TRINITY_DN7238_c0_g1_i7_orf1	vacuolar protein-sorting-associated protein 36 isoform X3 [Ostrinia furnacalis]	2	402	44.7	6.57	5	2	2	High	1	0.988	1.177	1.122	1.53	0.979	1.025	1.239	1.259	0.733	0.841	1.139	1.099	1.114	1.026
TRINITY_DN11698_c0_g1_i1_orf1	hypothetical protein evm_015129 [Chilo suppressalis]	2	164	17.3	10.01	13	3	2	High	1	1.003	1.096	1.653	1.727	1.474	1.928	1.867	1.946	1.785	1.735	1.762	1.179	1.207	1.271
TRINITY_DN15900_c0_g1_i6_orf1	unnamed protein product [Diatraea saccharalis]	2	950	111.5	10.35	2	2	2	High	1	1.1	0.958	0.246	0.31	0.38	0.302	0.355	0.335	0.259	0.243	0.263	0.477	0.429	0.408
TRINITY_DN7590_c0_g1_i4_orf1	innexin inx1-like [Pectinophora gossypiella]	1	361	42.1	8.62	3	1	1	High	1	0.847	0.77	1.401	1.257	1.312	1.637	1.492	1.723	1.701	1.42	1.38	1.947	1.799	2.135
TRINITY_DN7909_c0_g2_i1_orf1	aldehyde oxidase 3 [Ostrinia furnacalis]	2	1050	116.9	5.83	2	3	2	High	1	0.907	0.942	0.878	0.94	0.895	0.742	0.766	0.758	0.615	0.543	0.634	0.664	0.658	0.622
TRINITY_DN3241_c0_g1_i1_orf1	cilia- and flagella-associated protein 99-like [Ostrinia furnacalis]	2	593	69.5	9.32	3	2	2	High	1	1.147	0.925	0.966	0.99	1.134	1.415	1.414	1.373	1.352	1.084	1.159	1.31	1.374	1.144
TRINITY_DN12769_c0_g1_i5_orf1	uncharacterized protein LOC114365633 [Ostrinia furnacalis]	1	184	19.3	4.79	6	1	1	High	1	1.16	1.091	0.871	1.024	0.822	0.954	0.804	1.018	0.828	0.737	0.905	1.106	1.229	1.127
TRINITY_DN218_c0_g1_i1_orf1	altered inheritance of mitochondria protein 3-like isoform X2 [Ostrinia furnaca	2	164	18	7.12	10	3	2	High	1	1.062	0.952	0.83	0.898	0.848	0.877	1.095	0.798	0.87	0.789	0.889	2.577	2.732	2.349
TRINITY_DN96170_c0_g2_i1_orf1	uncharacterized protein LOC114355569 [Ostrinia furnacalis]	1	285	31.7	8.85	4	1	1	High	1	0.927	0.978	0.337	0.325	0.3	0.378	0.404	0.383	0.36	0.333	0.328	0.38	0.404	0.357
TRINITY_DN146544_c0_g1_i1_orf1	UPF0047 protein YjbQ [Aphidius gifuensis]	1	150	16.6	6.4	9	1	1	High	1	0.888	0.895	0.407	0.431	0.37	0.412	0.353	0.385	0.416	0.332	0.424	0.469	0.338	0.406
TRINITY_DN4390_c0_g1_i4_orf1	GILT-like protein 1 [Ostrinia furnacalis]	1	139	16.1	8.19	9	1	1	High	1	0.951	1.03	0.876	0.863	0.975	0.724	0.966	0.951	1.248	1.315	1.011	0.823	0.999	1.071
TRINITY_DN105055_c0_g1_i1_orfp	unnamed protein product [Euphydryas editha]	2	133	15.2	7.74	15	2	2	High	1	1.02	1.049	0.536	0.622	0.625	0.643	0.661	0.658	0.653	0.697	0.611	0.484	0.559	0.542
TRINITY_DN42373_c0_g4_i1_orf1	unnamed protein product [Spodoptera exigua]	1	127	14.9	9.31	6	2	1	High	1	1.069	0.971	0.834	0.882	0.8	0.694	0.702	0.631	0.559	0.459	0.633	0.543	0.626	0.533
TRINITY_DN44073_c0_g1_i3_orf1	inter-alpha-trypsin inhibitor heavy chain H4-like isoform X11 [Ostrinia furnaca	2	78	8.9	6.74	18	4	2	High	1	1.039	1.132	1.923	1.976	2.005	1.549	1.677	1.49	2.155	1.733	2.071	2.816	2.822	2.558
TRINITY_DN31520_c1_g1_i1_orf1	probable DNA-directed RNA polymerase III subunit RPC6 [Ostrinia furnacalis]	1	296	33	7.52	4	1	1	High	1	0.868	1.072	0.736	0.654	0.696	0.658	0.715	0.831	0.6	0.584	0.735	0.81	0.704	0.944
TRINITY_DN3255_c0_g1_i1_orf1	uncharacterized protein LOC114351042 [Ostrinia furnacalis]	1	125	13.9	8.6	6	1	1	High	1	0.931	0.988	0.589	0.664	0.743	0.58	0.724	0.681	0.622	0.497	0.576	3.035	3.37	3.258
TRINITY_DN279_c0_g1_i10_orf1	RE1-silencing transcription factor-like isoform X1 [Ostrinia furnacalis]	1	1077	120.9	6.84	1	1	1	High	1	1.086	1.073	0.443	0.515	0.471	0.38	0.443	0.409	0.27	0.332	0.321	1.073	1.168	1.179
TRINITY_DN2700_c2_g1_i1_orf1	mitotic checkpoint protein BUB3 [Ostrinia furnacalis]	1	332	37.6	8.09	4	1	1	High	1	0.819	0.938	1.24	1.27	1.718	1.23	0.993	1.096	1.168	0.967	1.318	0.772	1.037	0.981
TRINITY_DN659_c0_g1_i3_orf1	probable malonyl-CoA-acyl carrier protein transacylase, mitochondrial [Ostrin	1	309	33	9.17	3	1	1	High	1	1.156	1.164	0.46	0.539	0.548	0.609	0.654	0.593	0.562	0.503	0.575	0.671	0.559	0.58
TRINITY_DN61674_c0_g1_i2_orf1	fatty acid-binding protein 1-like [Ostrinia furnacalis]	2	125	14.2	5.97	12	2	2	High	1	0.985	1.079	0.099	0.115	0.125	0.137	0.148	0.133	0.13	0.135	0.158	0.14	0.149	0.15
TRINITY_DN16128_c0_g1_i5_orf1	probable prefoldin subunit 4 [Ostrinia furnacalis]	2	135	15.4	4.68	13	2	2	High	1	0.981	1.03	0.746	0.856	0.788	0.702	0.726	0.694	0.65	0.535	0.654	0.743	0.716	0.766
TRINITY_DN10933_c0_g2_i1_orf1	uncharacterized protein LOC114357588 [Ostrinia furnacalis]	1	402	46	6.71	2	1	1	High	1	0.969	0.996	1.559	1.419	1.554	1.306	1.39	1.349	1.644	1.73	1.373	1.436	1.336	1.594
TRINITY_DN116874_c0_g1_i1_orfp	TRINITY_DN116874_c0_g1_i1_m.85176 TRINITY_DN116874_c0_g1::TRINITY_DN	2	94	10.4	8.85	27	2	2	High	1	0.908	0.866	0.884	0.73	0.919	1.131	1.116	1.335	1.832	1.933	1.474	1.479	1.556	1.583
TRINITY_DN21930_c0_g1_i1_orf1	coactosin-like protein isoform X2 [Trichoplusia ni]	1	264	29.8	5.21	5	1	1	High	1	0.85	0.883	0.8	0.91	0.86	0.817	1.056	0.788	0.679	1.002	0.772	1.72	1.805	1.804
TRINITY_DN5153_c1_g1_i1_orf1	nose resistant to fluoxetine protein 6-like isoform X1 [Ostrinia furnacalis]	1	634	71.6	8.28	1	1	1	High	1	0.83	1.18	3.781	3.662	3.4	2.84	2.459	2.981	2.91	2.466	2.292	1.597	1.658	1.625
TRINITY_DN207_c0_g2_i3_orf1	JNK-interacting protein 3 isoform X2 [Ostrinia furnacalis]	1	1228	135.5	5.6	1	1	1	High	1	1.148	1.069	0.563	0.516	0.545	0.613	0.448	0.558	0.592	0.521	0.514	0.572	0.571	0.603
TRINITY_DN64171_c0_g1_i1_orf1	uncharacterized protein LOC114358253 [Ostrinia furnacalis]	1	409	46.7	9.25	3	1	1	High	1	1.199	1.283	0.852	1.042	0.938	0.863	0.896	1.294	1.242	1.164	0.898	0.865	0.788	0.758
TRINITY_DN52768_c0_g1_i1_orf1	carboxypeptidase Q-like isoform X2 [Ostrinia furnacalis]	1	469	52	4.86	2	1	1	High	1	1.026	1.047	0.22	0.259	0.291	0.285	0.341	0.271	0.256	0.247	0.324	0.298	0.268	0.255
TRINITY_DN5560_c0_g1_i5_orf1	uncharacterized protein LOC114354348 isoform X1 [Ostrinia furnacalis]	1	250	28.5	5.64	6	1	1	High	1	1.141	0.959	1.051	1.197	0.892	1.22	1.156	1.177	1.109	1.723	1.281	1.485	1.271	1.294
TRINITY_DN1108_c3_g1_i1_orfp1	TRINITY_DN1108_c3_g1_i1_m.5561 TRINITY_DN1108_c3_g1::TRINITY_DN1108_	1	112	12.3	6.37	11	1	1	High	1	2.486	0.447	14.8	13.607	9.364	8.19	5.161	7.93	12.943	11.131	14.914	8.895	8.082	5.474
TRINITY_DN35865_c0_g1_i1_orf1	uncharacterized protein LOC114354496 isoform X1 [Ostrinia furnacalis]	1	405	45.2	6.06	5	1	1	High	1	1.049	0.915	0.93	0.983	1.116	0.943	0.89	0.814	0.796	0.671	0.652	1.224	0.87	0.94
TRINITY_DN699_c0_g2_i1_orf1	TPA_exp: putative parasitoid killing factor [Trichoplusia ni]	1	388	43.9	6.57	3	4	1	High	1	1.014	1.028	1.457	1.602	1.504	1.779	1.809	1.756	2.695	2.552	2.864	3.807	4.104	3.554
TRINITY_DN39530_c0_g1_i1_orf1	NHP2-like protein 1 [Ostrinia furnacalis]	1	130	14.2	7.85	9	1	1	High	1	0.961	0.979	0.848	0.804	0.887	0.926	0.87	0.791	0.854	0.804	0.877	0.943	0.972	0.893
TRINITY_DN37538_c0_g1_i1_orf1	esterase FE4-like [Ostrinia furnacalis]	1	210	22.5	7.33	4	1	1	High	1	0.957	0.929	0.08	0.077	0.114	0.12	0.094	0.137	0.12	0.116	0.139	0.121	0.094	0.115
TRINITY_DN98814_c0_g1_i2_orf1	PREDICTED: chaoptin [Amyelois transitella]	1	1324	146.9	6.4	1	1	1	High	1	0.896	0.932	0.662	0.794	0.77	0.718	0.702	0.676	0.832	0.851	0.701	1.135	1.247	1.161
TRINITY_DN33885_c0_g1_i1_orf1	inhibitor of nuclear factor kappa-B kinase subunit alpha [Ostrinia furnacalis]	1	779	89.6	7.75	2	1	1	High	1	0.876	0.943	2.18	3.026	2.528	1.866	2.509	1.642	1.433	2.242	3.097	1.151	0.769	1.244
TRINITY_DN27456_c0_g2_i1_orf1	organic cation transporter-like protein [Ostrinia furnacalis]	1	502	55.1	8.19	2	1	1	High	1	0.949	0.92	0.963	0.893	1.155	0.992	1.062	1.154	0.709	0.815	0.759	1.539	1.364	1.3
TRINITY_DN56379_c0_g1_i1_orf1	alpha-tocopherol transfer protein-like [Ostrinia furnacalis]	1	301	35.2	6.73	4	1	1	High	1	1.087	1.078	0.699	0.773	0.768	0.801	0.862	0.93	0.857	0.827	0.848	0.827	0.881	0.949
TRINITY_DN4911_c0_g1_i6_orf1	ATP-binding cassette sub-family G member 1-like isoform X2 [Ostrinia furnaca	1	602	66.9	8.35	2	1	1	High	1	0.899	1.015	1.159	1.221	1.185	1.357	1.315	1.745	1.201	1.28	1.183	1.081	1.32	1.35
TRINITY_DN6974_c0_g2_i1_orf1	mucolipin-3-like [Ostrinia furnacalis]	1	600	69	6.84	2	1	1	High	1	1.318	1.25	1.389	1.298	1.228	1.137	0.978	1.204	1.022	1.228	1.054	1.669	1.682	1.759
TRINITY_DN9536_c0_g1_i4_orf1	adrenodoxin-like protein, mitochondrial isoform X1 [Ostrinia furnacalis]	1	167	19	6.73	8	1	1	High	1	1.049	0.947	0.841	0.835	0.909	0.765	0.854	0.784	0.878	0.861	0.92	1.398	1.709	1.466
TRINITY_DN9062_c0_g2_i1_orf1	ubiquitin conjugation factor E4 B isoform X2 [Ostrinia furnacalis]	1	788	87.6	6.21	2	1	1	High	1	1.099	0.901	0.794	0.831	0.86	1.003	0.952	0.895	0.75	0.837	0.951	1.437	0.981	1.043
TRINITY_DN8701_c0_g1_i3_orf1	putative uncharacterized protein DDB_G0282133 [Ostrinia furnacalis]	1	365	41.2	5.82	4	1	1	High	1	1.107	0.974	0.827	0.769	0.88	0.926	0.981	0.769	0.655	0.691	0.721	1.175	1.183	1.013
TRINITY_DN11552_c0_g1_i4_orf1	glutathione hydrolase 1 proenzyme-like isoform X3 [Ostrinia furnacalis]	1	613	67	8.43	2	1	1	High	1	1.126	1.162	0.816	0.697	0.559	0.691	0.832	0.686	0.68	0.572	0.769	0.709	0.749	0.512
TRINITY_DN4731_c0_g2_i1_orf1	gelsolin-like [Ostrinia furnacalis]	1	176	19.7	4.77	6	1	1	High	1	0.85	0.95	0.55	0.482	0.512	0.592	0.645	0.755	0.635	0.769	0.583	0.612	0.532	0.58
TRINITY_DN1999_c0_g1_i9_orf1	acyl-CoA Delta(11) desaturase-like [Ostrinia furnacalis]	1	352	40.8	8.22	3	1	1	High	1	1.002	1.049	0.536	0.499	0.511	0.863	0.906	0.907	0.536	0.464	0.509	0.589	0.655	0.616
TRINITY_DN3651_c0_g1_i5_orf1	probable tRNA N6-adenosine threonylcarbamoyltransferase [Ostrinia furnacali	1	334	36.8	5.67	4	1	1	High	1	1.011	1.013	0.811	0.822	0.874	0.95	1.005	1.092	0.809	0.944	0.902	0.901	0.943	0.874
TRINITY_DN1427_c0_g1_i9_orf1	SAFB-like transcription modulator isoform X3 [Ostrinia furnacalis]	1	624	71.9	5.52	2	1	1	High	1	0.997	1.015	0.498	0.524	0.645	0.321	0.414	0.357	0.249	0.324	0.365	0.608	0.658	0.544
TRINITY_DN5008_c0_g1_i1_orf1	LOW QUALITY PROTEIN: integrator complex subunit 14 [Ostrinia furnacalis]	1	519	56	6.96	3	1	1	High	1	1.113	1.167	1.05	1.038	0.905	0.801	1.099	0.74	0.679	0.705	0.844	0.889	0.97	0.718
TRINITY_DN14734_c0_g1_i2_orf1	SET and MYND domain-containing protein 5 isoform X1 [Ostrinia furnacalis]	1	385	43.2	4.81	5	1	1	High	1	0.939	1.031	0.617	1.014	0.543	0.718	0.59	0.648	0.529	0.943	1.003	1.117	0.883	0.812
TRINITY_DN4558_c0_g2_i1_orf1	hypothetical protein evm_005814 [Chilo suppressalis]	1	449	49.8	7.17	3	1	1	High	1	0.993	1.132	1.189	1.095	1.082	1.085	1.229	1.406	1.324	1.366	1.218	1.464	1.149	1.176
TRINITY_DN9458_c0_g1_i4_orf1	uncharacterized protein LOC114363583 [Ostrinia furnacalis]	1	86	9.3	4.88	13	1	1	High	1	0.863	0.979	0.699	0.757	0.925	0.905	0.973	0.882	2.054	1.846	2.222	0.801	0.792	0.75
TRINITY_DN6243_c0_g1_i5_orf1	sorting nexin-20 [Ostrinia furnacalis]	1	255	28.8	8.05	5	1	1	High	1	0.985	0.891	2.165	2.053	1.836	1.75	1.83	1.863	1.61	1.896	1.712	2.217	2.325	2.111
TRINITY_DN7134_c0_g1_i1_orf1	phosphatidylglycerophosphatase and protein-tyrosine phosphatase 1 [Ostrinia	1	196	22.6	8.94	6	1	1	High	1	1.011	0.933	0.58	0.65	0.614	0.751	0.593	0.636	0.615	0.687	0.758	0.514	0.579	0.561
TRINITY_DN109931_c0_g1_i1_orf1	hydroxymethylglutaryl-CoA lyase, mitochondrial isoform X1 [Ostrinia furnacalis	1	52	5.9	8.97	15	1	1	High	1	1.056	0.993	1.821	2.021	1.87	1.864	1.597	1.519	1.274	1.293	1.687	0.424	0.379	0.382
TRINITY_DN4053_c0_g1_i5_orf1	uncharacterized protein LOC114358355 [Ostrinia furnacalis]	1	199	21.5	9.79	6	1	1	High	1	0.869	0.915	0.925	1.104	1.058	1.315	1.075	1.391	0.931	0.891	1.033	0.657	0.603	0.608
TRINITY_DN48765_c0_g1_i7_orf1	uncharacterized protein LOC114352307 [Ostrinia furnacalis]	1	197	22	6.16	8	1	1	High	1	1.031	1.102	1.679	1.625	1.584	1.368	0.978	1.385	1.366	1.626	1.547	1.867	1.618	1.813
TRINITY_DN31431_c0_g1_i1_orf1	carnosine N-methyltransferase [Ostrinia furnacalis]	1	383	44.3	5.9	3	1	1	High	1	0.939	0.946	0.805	0.879	0.904	0.758	0.742	0.84	0.507	0.659	0.664	0.759	0.947	0.907
TRINITY_DN533_c3_g1_i2_orf1	hypothetical protein evm_012530 [Chilo suppressalis]	1	306	35.5	7.96	5	1	1	High	1	0.854	0.859	0.994	0.887	0.901	0.877	0.942	0.88	0.861	0.826	0.934	0.947	1.008	1.084
TRINITY_DN401_c0_g1_i15_orf1	cholinephosphotransferase 1 isoform X2 [Ostrinia furnacalis]	1	432	47.9	6.86	2	1	1	High	1	1.009	0.932	0.648	0.634	0.639	0.704	0.58	0.607	0.605	0.562	0.681	0.618	0.635	0.638
TRINITY_DN699_c0_g1_i5_orf1	TPA_exp: putative parasitoid killing factor [Trichoplusia ni]	1	395	44.9	6.55	3	1	1	High	1	1.01	0.852	1.478	1.738	1.541	1.162	1.319	1.25	1.354	2.249	1.469	1.059	1.222	1.113
TRINITY_DN23968_c0_g1_i2_orf1	peptide-N(4)-(N-acetyl-beta-glucosaminyl)asparagine amidase isoform X1 [C	1	620	71.1	8.15	2	1	1	High	1	1.105	0.959	0.996	0.887	1.239	1.001	1.064	1.064	1.208	1.285	1.076	0.978	1.222	1.201
TRINITY_DN971_c0_g1_i5_orfp1	TRINITY_DN971_c0_g1_i5_m.54249 TRINITY_DN971_c0_g1::TRINITY_DN971_c0	1	107	11.5	7.08	9	1	1	High	1	0.708	0.989	12.061	10.866	10.683	13.238	13.875	11.997	20.606	17.52	24.873	4.683	3.422	4.314
TRINITY_DN9554_c0_g1_i1_orf1	zinc transporter 1 [Chelonus insularis]	1	448	50.3	6.93	2	2	1	High	1	0.796	0.894	0.663	0.791	0.836	0.931	1.487	1.154	0.907	0.881	0.814	0.99	1.047	1.032
TRINITY_DN3355_c0_g3_i1_orf1	UDP-glucuronosyltransferase 2B14-like isoform X1 [Ostrinia furnacalis]	2	366	41.7	5.35	5	2	1	High	1	0.918	1.104	0.883	0.928	0.781	0.801	0.858	0.915	0.869	1.025	1.008	1.005	0.887	1.086
TRINITY_DN16905_c0_g1_i1_orf1	unnamed protein product [Leptidea sinapis]	1	362	40.3	8.95	2	1	1	High	1	1.428	1.018	3.187	2.875	2.392	2.723	2.019	2.737	2.946	2.69	2.534	2.908	1.721	3.01
TRINITY_DN8621_c0_g1_i4_orf1	aminopeptidase N-like isoform X2 [Ostrinia furnacalis]	2	871	102.3	8.24	3	2	2	High	1	1.01	0.958	1.847	2.123	1.765	1.446	1.536	1.69	1.219	1.146	1.304	1.061	1.104	1.254
TRINITY_DN12101_c0_g1_i2_orf1	UPF0545 protein C22orf39 homolog [Ostrinia furnacalis]	1	161	19.2	5.4	6	1	1	High	1	0.94	0.927	0.515	0.44	0.566	0.496	0.524	0.542	0.635	0.591	0.615	0.663	0.729	0.648
TRINITY_DN13361_c0_g1_i1_orf1	LOW QUALITY PROTEIN: baculoviral IAP repeat-containing protein 6-like, part	1	401	44.3	5.85	2	1	1	High	1	0.783	1.006	0.984	0.94	0.946	0.878	0.956	0.945	0.847	0.77	0.89	0.89	0.797	0.746
TRINITY_DN26293_c0_g1_i4_orf1	UDP-glucose 6-dehydrogenase [Ostrinia furnacalis]	1	479	52.9	6.65	3	1	1	High	1	1.056	0.934	0.677	0.698	0.824	0.863	0.766	0.719	0.843	0.799	0.92	1.377	1.186	1.323
TRINITY_DN48970_c0_g1_i1_orf1	uncharacterized protein LOC114356431 isoform X2 [Ostrinia furnacalis]	1	68	7.8	6.27	19	1	1	High	1	0.902	1.108	0.735	0.76	0.733	0.627	0.71	0.766	0.58	0.605	0.697	0.992	0.991	0.887
TRINITY_DN4056_c0_g1_i8_orf1	uncharacterized protein LOC114349672 [Ostrinia furnacalis]	1	737	82.7	9.25	1	1	1	High	1	1.013	0.898	0.7	0.834	0.67	0.565	0.565	0.561	0.45	0.383	0.573	0.748	0.649	0.626
TRINITY_DN1012_c0_g2_i1_orf1	teneurin-a isoform X1 [Ostrinia furnacalis]	1	737	84.6	8.97	3	1	1	High	1	1.102	1.147	1.66	1.262	1.497	1.428	2.318	2.782	2.158	1.619	1.46	1.32	1.341	1.431
TRINITY_DN87803_c0_g1_i2_orf1	hypothetical protein NE865_05903 [Phthorimaea operculella]	2	87	9.7	4.72	23	2	1	High	1	1.718	1.581	1.837	1.609	1.35	1.037	1.506	1.812	1.937	1.029	1.289	10.94	10.257	10.691
TRINITY_DN834_c0_g1_i1_orf1	tissue inhibitor of metalloproteinase [Ostrinia furnacalis]	1	201	23.2	9.48	7	1	1	High	1	0.941	1.073	0.534	0.736	0.694	0.65	0.679	0.703	0.86	1.014	0.735	0.874	0.657	0.831
TRINITY_DN29229_c0_g1_i4_orf1	uncharacterized protein LOC114351433 isoform X1 [Ostrinia furnacalis]	1	610	69.3	8.69	2	1	1	High	1	1.242	1.005	0.8	0.983	0.787	0.604	0.595	0.642	0.523	0.537	0.645	0.491	0.46	0.537
TRINITY_DN28221_c0_g2_i1_orf1	unnamed protein product [Chilo suppressalis]	1	89	10.1	4.7	9	2	1	High	1	0.935	0.946	2.657	2.671	2.642	2.79	2.951	2.641	3.376	2.863	3.67	3.099	3.343	3.301

TRINITY_DN2395_c0_g1_i7_orf1	uncharacterized protein LOC114352963 [Ostrinia furnacalis]	1	414	45.8	7.83	3	1	1	High	1	1.102	1.052	1.388	1.316	1.188	1.51	1.83	1.309	1.454	2.001	1.679	1.774	1.611	1.906
TRINITY_DN31645_c0_g1_i3_orf1	dystonin isoform X43 [Helicoverpa armigera]	1	141	15.8	9.85	6	1	1	High	1	1.088	0.944	1.388	1.545	1.378	1.308	1.363	1.231	1.153	0.974	1.232	0.682	0.501	0.547
TRINITY_DN25017_c0_g1_i1_orf1	peroxisomal acyl-coenzyme A oxidase 3-like [Zerene cesonia]	1	26	3.3	8.66	46	1	1	High	1	0.984	0.948	0.915	0.887	0.925	0.872	0.912	1.112	1.081	1.033	0.867	0.928	0.864	0.936
TRINITY_DN29120_c0_g1_i6_orf1	putative inorganic phosphate cotransporter [Ostrinia furnacalis]	1	246	27.3	8.66	4	1	1	High	1	1.036	0.969	0.119	0.13	0.155	0.204	0.166	0.15	0.149	0.138	0.158	0.3	0.321	0.274
TRINITY_DN8700_c9_g1_i1_orf1	uncharacterized protein LOC114351392 isoform X1 [Ostrinia furnacalis]	2	235	26.3	8.54	10	2	2	High	1	1.049	1.028	0.993	1.061	1.07	1.08	1.085	1.073	1.124	1.115	1.23	1.367	1.28	1.433
TRINITY_DN71832_c0_g1_i1_orf1	basement membrane-specific heparan sulfate proteoglycan core protein isofo	1	76	8.3	5.1	12	2	1	High	1	1.05	1.22	1.27	1.281	0.877	1.345	1.52	1.322	0.694	0.546	0.622	1.778	2.457	1.816
TRINITY_DN2695_c0_g1_i8_orfp1	TRINITY_DN2695_c0_g1_i8_m.44478 TRINITY_DN2695_c0_g1::TRINITY_DN2695	1	498	56.2	5.33	2	1	1	High	1	0.684	1.005	0.209	0.215	0.252	0.372	0.353	0.229	0.763	0.777	0.987	0.185	0.231	0.137
TRINITY_DN3299_c0_g1_i2_orf1	metaxin-1 isoform X3 [Ostrinia furnacalis]	1	316	36.5	6.55	4	1	1	High	1	1.353	1.16	0.676	0.583	0.617	0.516	0.345	0.417	0.49	0.435	0.593	0.54	0.517	0.448
TRINITY_DN11569_c0_g1_i1_orf1	hypothetical protein evm_008488 [Chilo suppressalis]	1	425	45.7	6.73	3	1	1	High	1	0.815	0.702	1.302	1.316	1.356	1.533	1.24	1.755	1.581	6.189	1.321	1.156	1.175	1.616
TRINITY_DN27725_c0_g1_i2_orf1	BRISC complex subunit FAM175B-like [Ostrinia furnacalis]	1	394	43.9	6.35	3	1	1	High	1	0.563	0.838	0.638	0.668	0.424	0.3	0.421	0.5	0.391	0.452	0.461	0.896	0.892	0.598
TRINITY_DN2589_c0_g1_i1_orf1	sterol regulatory element-binding protein 1 [Ostrinia furnacalis]	1	1063	117.4	8.69	1	1	1	High	1	0.859	0.88	0.969	0.967	0.949	0.94	1.001	0.863	0.664	0.828	1.013	0.938	0.926	0.941
TRINITY_DN39933_c0_g1_i2_orf1	thioredoxin domain-containing protein 11 isoform X4 [Ostrinia furnacalis]	1	727	83.6	6.4	2	1	1	High	1	0.976	1.134	1.815	1.659	1.725	1.388	1.425	1.314	1.147	1.94	1.404	1.124	0.899	0.933
TRINITY_DN30704_c0_g1_i1_orf1	cytochrome P450 monooxygenase CYP6AE134v2 [Ostrinia furnacalis]	1	532	61.1	8.27	2	1	1	High	1	0.973	1.066	0.086	0.129	0.27	0.23	0.271	0.273	0.209	0.223	0.161	0.117	0.083	0.058
TRINITY_DN81181_c0_g1_i6_orfp1	C-type lectin domain family 4 member E [Pieris rapae]	1	88	9.7	9.29	14	1	1	High	1	0.851	0.844	1.454	1.439	1.589	0.545	0.808	0.775	0.895	1.251	0.85	0.738	0.807	0.668
TRINITY_DN1652_c0_g1_i12_orf1	synaptotagmin 1 isoform X1 [Ostrinia furnacalis]	1	429	48.1	6.34	3	1	1	High	1	1.171	1.107	0.808	0.78	0.947	0.991	0.877	0.955	0.681	0.756	0.84	0.815	0.853	0.87
TRINITY_DN28152_c0_g1_i1_orf1	mitochondrial import inner membrane translocase subunit Tim29 [Ostrinia furr	1	186	21.8	7.77	4	1	1	High	1	1.044	0.999	0.367	0.308	0.324	0.346	0.342	0.422	0.363	0.353	0.316	0.308	0.413	0.389
TRINITY_DN1571_c0_g1_i9_orf1	conserved oligomeric Golgi complex subunit 4 [Ostrinia furnacalis]	1	715	77.1	6.57	2	1	1	High	1	1.07	0.976	0.867	0.758	0.735	1.05	0.831	1.064	0.747	0.917	0.865	0.782	0.759	0.963
TRINITY_DN1227_c0_g1_i1_orf1	uncharacterized protein LOC114356076 [Ostrinia furnacalis]	2	390	45.5	9.44	5	2	2	High	1	1.022	0.981	0.656	0.616	0.69	0.635	0.62	0.712	0.727	0.773	0.594	0.818	0.731	0.732
TRINITY_DN130439_c0_g1_i1_orf1	DDRGK domain-containing protein 1-like [Ostrinia furnacalis]	1	291	33.3	5.25	3	1	1	High	1	1.186	1.037	1.724	1.986	1.632	1.273	1.468	1.382	1.244	0.855	1.679	1.891	1.575	1.819
TRINITY_DN9248_c0_g1_i10_orf1	unnamed protein product [Arctia plantaginis]	2	347	38.7	8.34	5	2	2	High	1	1.049	1.045	0.789	0.746	0.799	0.773	0.719	0.678	0.693	0.662	0.726	0.545	0.546	0.58
TRINITY_DN6870_c0_g1_i5_orf1	uncharacterized protein LOC114365835 [Ostrinia furnacalis]	1	163	17.8	5.87	7	1	1	High	1	1.067	1.147	0.795	1.243	0.942	1.075	1.412	1.348	1.025	0.785	1.114	1.501	1.448	1.768
TRINITY_DN8252_c0_g1_i6_orf1	scavenger receptor class B member 1 [Ostrinia furnacalis]	1	626	71.4	8.38	2	1	1	High	1	0.871	1.202	1.59	1.587	1.504	1.604	1.805	1.955	1.364	3.181	1.633	1.232	1.22	1.507
TRINITY_DN30271_c0_g1_i4_orf1	splicing factor 3B subunit 3 isoform X1 [Ostrinia furnacalis]	1	135	14.9	5.24	12	1	1	High	1	1.139	0.966	0.98	1.143	1.101	0.96	1.016	1.077	1.155	0.99	1.287	0.957	0.942	0.924
TRINITY_DN101682_c0_g1_i1_orf1	cysteine-rich with EGF-like domain protein 2 [Ostrinia furnacalis]	1	360	40.4	6	2	1	1	High	1	1.213	1.019	0.714	0.725	0.623	0.72	0.621	0.612	0.47	0.26	0.524	0.675	0.532	0.503
TRINITY_DN49147_c0_g2_i1_orf1	glutenin, high molecular weight subunit PW212-like [Ostrinia furnacalis]	1	288	31.6	8.57	5	2	1	High	1	1.106	1.114	1.421	1.1	1.487	1.734	1.843	1.892	1.964	2.167	1.648	4.613	4.572	4.779
TRINITY_DN125_c0_g1_i2_orf1	PREDICTED: purine nucleoside phosphorylase isoform X1 [Microplitis demolito	1	331	37.1	6.46	3	1	1	High	1	0.969	0.922	1.279	1.104	1.371	1.314	1.503	1.377	1.56	2.025	1.571	1.238	1.181	1.296
TRINITY_DN7635_c0_g1_i1_orf1	dynactin subunit 1 [Ostrinia furnacalis]	1	229	25.6	8.28	5	1	1	High	1	0.945	0.848	1.061	1.113	0.909	1.054	1.125	1.047	0.94	0.956	0.897	1.178	1.219	0.885
TRINITY_DN35991_c0_g1_i2_orf1	serine/threonine-protein kinase D1 isoform X1 [Ostrinia furnacalis]	1	321	36.2	7.01	4	1	1	High	1	0.904	1.017	1.414	1.15	1.088	1.111	1.356	1.342	1.141	1.53	1.323	1.053	1.157	1.184
TRINITY_DN37585_c0_g2_i1_orf1	cuticle protein 19.8-like [Ostrinia furnacalis]	1	138	14.7	6.27	6	1	1	High	1	1.105	1.128	0.808	0.944	0.943	0.987	1.08	0.92	0.774	0.901	0.762	2.184	2.528	1.865
TRINITY_DN29414_c1_g2_i1_orf1	serine protease 44-like isoform X2 [Ostrinia furnacalis]	1	78	8.8	7.84	15	1	1	High	1	0.911	0.996	3.277	2.3	2.468	3.612	2.95	3.985	5.225	5.152	2.865	3.995	3.191	4.012
TRINITY_DN53233_c0_g1_i1_orf1	unnamed protein product, partial [Iphiclides podalirius]	1	140	15.6	10.26	7	2	1	High	1	1.095	1.145	1.094	1.07	1.181	1.4	1.071	1.177	1.367	1.458	1.188	1.415	1.481	1.291
TRINITY_DN17255_c0_g1_i9_orf1	focal adhesion kinase 1 isoform X2 [Galleria mellonella]	1	1350	150.4	7.85	1	1	1	High	1	1.082	1.097	0.827	0.92	0.76	0.868	1.103	1.07	0.809	1.195	1.02	1.095	0.949	1.051
TRINITY_DN12024_c0_g1_i4_orf1	pancreatic lipase-related protein 2 isoform X1 [Ostrinia furnacalis]	1	282	31.2	6.9	5	1	1	High	1	0.953	0.862	1.1	1.326	1.253	1.264	1.328	1.544	1.446	1.401	1.629	1.571	1.655	1.751
TRINITY_DN5420_c0_g1_i2_orf1	DNA-directed RNA polymerase II subunit RPB1-like [Ostrinia furnacalis]	1	514	58	9.39	2	1	1	High	1	1.05	1.299	1.916	1.794	1.539	1.482	1.67	1.713	1.765	1.86	1.74	2.613	2.37	2.842
TRINITY_DN169_c0_g1_i3_orf1	ankyrin repeat domain-containing protein 11-like isoform X4 [Vanessa atalant	1	743	79.8	9.41	2	1	1	High	1	1.512	1.266	1.047	1.836	1.248	1.122	0.749	1.63	1.097	0.951	1.212	0.942	1.034	0.898
TRINITY_DN6202_c0_g1_i2_orf1	PREDICTED: serine/threonine-protein phosphatase PP1-beta catalytic subunit	1	380	43.7	6.33	3	1	1	High	1	0.925	1.017	0.807	0.859	0.804	0.848	0.792	0.795	0.913	0.847	0.819	1.026	0.965	1.026
TRINITY_DN14721_c0_g1_i2_orf1	protein masquerade-like isoform X2 [Ostrinia furnacalis]	1	356	38	9.04	3	1	1	High	1	0.999	1.129	0.603	0.807	0.57	0.577	0.7	0.579	0.587	0.661	0.768	2.27	1.973	2.033
TRINITY_DN116467_c0_g1_i1_orf1	probable small nuclear ribonucleoprotein E [Ostrinia furnacalis]	1	95	11.1	9.57	17	2	1	High	1	0.918	1.036	1.197	1.159	1.278	1.034	1.206	1.195	1.152	1.367	1.173	1.357	1.497	1.525
TRINITY_DN3887_c0_g1_i1_orf1	formin-like protein isoform X3 [Ostrinia furnacalis]	1	507	57.8	9.09	3	1	1	High	1	1.129	0.998	0.891	1.191	1.117	0.959	0.982	0.978	0.957	0.865	0.984	1.538	1.624	1.83
TRINITY_DN7735_c0_g1_i4_orf1	calphotin-like [Ostrinia furnacalis]	1	226	22.5	9.41	3	1	1	High	1	0.88	1.129	2.044	2.505	1.646	2.194	1.949	1.899	2.057	1.987	1.732	0.156	0.339	0.321
TRINITY_DN1706_c0_g1_i7_orf1	LOW QUALITY PROTEIN: RNA polymerase-associated protein CTR9 homolog [	1	1197	136.5	9.95	1	1	1	High	1	0.914	0.892	0.503	0.447	0.46	0.446	0.413	0.43	0.356	0.352	0.359	0.64	0.606	0.557
TRINITY_DN4145_c0_g1_i1_orf1	uncharacterized protein LOC114353175 isoform X1 [Ostrinia furnacalis]	1	672	73.6	5.94	2	1	1	High	1	0.949	0.856	0.661	0.65	0.652	0.631	0.537	0.701	0.604	0.558	0.625	0.39	0.293	0.416
TRINITY_DN80245_c0_g1_i1_orf1	peroxisomal membrane protein 2 [Ostrinia furnacalis]	1	185	20.9	9.77	4	1	1	High	1	0.907	0.975	0.793	0.535	0.721	0.704	0.654	0.827	0.618	0.677	0.636	0.547	0.485	0.468
TRINITY_DN5198_c0_g1_i5_orfp1	TRINITY_DN5198_c0_g1_i5_m.8637 TRINITY_DN5198_c0_g1::TRINITY_DN5198_	1	222	24.7	5.06	5	1	1	High	1	0.638	0.733	3.805	4.19	4.153	2.735	2.042	3.145	2.485	2.106	2.312	1.041	1.018	0.992
TRINITY_DN29956_c1_g1_i1_orf1	PREDICTED: dual specificity mitogen-activated protein kinase kinase dSOR1 isc	1	414	45.6	6.05	3	1	1	High	1	0.9	1.034	0.935	0.987	1.223	1.346	1.464	1.461	1.319	1.701	1.197	1.324	1.153	1.277
TRINITY_DN70485_c0_g1_i2_orf1	serine/threonine-protein kinase Genghis Khan-like [Ostrinia furnacalis]	1	411	45.1	8.76	4	1	1	High	1	1.137	1.041	1.302	1.497	1.712	1.355	1.396	1.373	1.425	1.5	1.575	1.767	2.121	2.153
TRINITY_DN10071_c0_g1_i2_orf1	trypsin, alkaline C-like [Ostrinia furnacalis]	1	257	26.7	5.2	8	1	1	High	1	1.049	1.213	0.702	0.898	0.803	0.709	0.722	0.672	0.702	1.092	0.898	0.751	0.481	0.719
TRINITY_DN3536_c0_g1_i1_orf1	partner of Y14 and mago [Ostrinia furnacalis]	1	193	21.5	9.64	8	1	1	High	1	0.897	0.938	0.851	0.822	0.794	0.799	0.867	0.853	0.881	0.736	0.907	0.927	0.981	0.884
TRINITY_DN1153_c1_g1_i1_orf1	gamma-butyrobetaine dioxygenase [Ostrinia furnacalis]	2	402	46.7	8.05	4	2	2	High	1	1.038	1.08	0.795	0.729	0.788	1.055	1.055	1.025	0.774	0.682	0.862	0.827	0.756	0.809
TRINITY_DN122423_c0_g1_i1_orf1	hypothetical protein TSAR_001625, partial [Trichomalopsis sarcophagae]	1	114	12.9	4.97	7	1	1	High	1	1.189	1.206	1.062	0.993	1.224	1.049	0.904	0.974	1.092	0.824	0.8	1.259	1.22	1.15
TRINITY_DN409_c0_g1_i5_orf1	ecto-NOX disulfide-thiol exchanger 2-like isoform X1 [Ostrinia furnacalis]	1	714	82.4	6.89	2	1	1	High	1	0.81	0.929	1.024	0.821	1.048	0.946	0.912	0.86	1.112	0.969	0.926	1.109	1.04	0.848
TRINITY_DN72_c0_g1_i16_orf1	protein groucho-like [Ostrinia furnacalis]	2	711	76.5	7.61	4	2	2	High	1	0.871	0.845	0.937	0.902	0.979	0.892	0.999	0.999	0.843	0.898	1.043	1.183	1.208	1.136
TRINITY_DN16179_c0_g1_i1_orf1	calexcitin-1 [Ostrinia furnacalis]	1	184	21.2	5.07	5	1	1	High	1	0.97	1.071	0.873	0.726	0.994	0.967	0.901	0.996	0.874	0.988	0.824	0.883	1.021	1.127
TRINITY_DN2270_c0_g2_i1_orf1	integrin beta-nu [Ostrinia furnacalis]	2	813	89	5.08	3	2	2	High	1	0.988	1.007	0.303	0.381	0.412	0.372	0.274	0.306	0.375	0.302	0.303	0.314	0.357	0.357
TRINITY_DN48497_c0_g1_i1_orf1	unnamed protein product [Chrysodeixis includens]	1	100	10	4.83	12	1	1	High	1	1.087	1.044	0.677	0.85	0.706	1.797	1.742	1.55	0.81	0.739	0.995	1.089	1.247	1.344
TRINITY_DN7622_c0_g2_i2_orf1	uncharacterized protein LOC114351301 [Ostrinia furnacalis]	1	653	75	8.4	2	1	1	High	1	0.926	0.771	1.216	1.479	1.095	0.999	1.139	0.933	1.213	1.839	1.466	1.116	1.114	1.268
TRINITY_DN18218_c0_g1_i7_orf1	inhibin beta B chain [Ostrinia furnacalis]	1	360	41.4	7.96	4	1	1	High	1	0.846	0.999	1.44	1.487	1.256	1.828	2.12	1.773	1.642	3.497	7.431	8.375	5.186	8.689
TRINITY_DN4567_c0_g1_i5_orf1	3-ketoacyl-CoA thiolase, mitochondrial-like [Ostrinia furnacalis]	1	398	42.1	6.25	3	1	1	High	1	0.893	0.925	0.728	0.934	0.789	0.75	0.869	1.05	0.754	0.913	0.734	0.598	0.709	0.647
TRINITY_DN127151_c0_g1_i1_orf1	3-oxoacyl-[acyl-carrier-protein] synthase, mitochondrial [Ostrinia furnacalis]	1	431	45.9	7.21	3	1	1	High	1	0.989	0.981	0.569	0.496	0.555	0.576	0.566	0.491	0.509	0.654	0.587	0.467	0.547	0.527
TRINITY_DN10058_c0_g1_i1_orf1	dolichyl-diphosphooligosaccharide--protein glycosyltransferase subunit DAD1	1	112	12.6	8.29	7	1	1	High	1	1.093	1.094	0.728	0.48	0.752	0.727	0.524	0.818	0.738	0.66	0.408	0.556	0.64	0.621
TRINITY_DN4204_c0_g1_i1_orf1	uncharacterized protein LOC114359352 [Ostrinia furnacalis]	1	281	32.1	6.79	4	1	1	High	1	1.345	1.244	1.345	1.419	1.322	4.197	3.714	3.875	6.148	5.377	6.285	0.686	0.819	0.774
TRINITY_DN40028_c0_g2_i3_orf1	hypothetical protein HF086_005631 [Spodoptera exigua]	1	34	3.6	8.68	38	1	1	High	1	0.852	0.828	0.728	0.758	0.934	0.854	1.055	0.988	0.932	1.069	0.898	0.91	0.814	1.02
TRINITY_DN98692_c0_g3_i1_orf1	fatty acyl-CoA hydrolase precursor, medium chain [Ostrinia furnacalis]	1	63	7.2	6.67	13	3	1	High	1	0.983	1.015	3.794	3.909	3.604	3.053	3.087	2.694	3.119	2.613	3.207	4.115	4.34	3.897
TRINITY_DN15845_c0_g1_i1_orf1	probable ATP-dependent RNA helicase DDX28 isoform X1 [Ostrinia furnacalis]	2	499	56.1	9.09	5	2	2	High	1	0.876	1.19	0.606	0.803	0.69	0.694	0.617	0.669	0.549	0.563	0.688	0.665	0.767	0.877
TRINITY_DN24528_c0_g1_i1_orf1	uncharacterized protein CG1161-like [Ostrinia furnacalis]	2	191	21.8	8.79	7	4	2	High	1	1.017	0.899	1.241	1.336	1.857	1.414	2.072	2.06	1.502	0.881	1.109	1.327	1.401	1.439
TRINITY_DN1293_c1_g1_i4_orf1	putative fatty acyl-CoA reductase CG5065 [Ostrinia furnacalis]	1	497	56.9	7.93	3	1	1	High	1	1.129	0.931	2.126	1.975	1.912	0.758	0.762	1.214	1.297	0.932	0.73	1.585	1.486	1.468
TRINITY_DN24266_c0_g2_i2_orf1	chromobox-like protein 5 [Helicoverpa armigera]	1	192	21.9	5.95	6	1	1	High	1	1.272	1.153	1.042	1.173	0.949	0.67	0.806	0.683	0.649	0.631	0.719	1.208	1.396	1.289
TRINITY_DN858_c0_g1_i3_orf1	uncharacterized protein LOC114351944 [Ostrinia furnacalis]	2	179	20.2	5.6	9	4	2	High	1	1.02	1.015	2.289	2.577	2.464	2.046	1.888	1.986	1.19	0.968	1.087	3.076	3.069	2.737
TRINITY_DN3322_c0_g1_i2_orf1	myrosinase 1-like isoform X2 [Ostrinia furnacalis]	1	554	63.5	7.49	2	1	1	High	1	1.028	1.164	0.667	0.733	0.74	0.796	0.836	0.791	1.138	1.008	0.882	1.096	0.985	0.978
TRINITY_DN16643_c0_g2_i4_orf1	heterogeneous nuclear ribonucleoprotein R isoform X6 [Danaus plexippus ple>	1	88	10	12.31	9	7	1	High	1	0.944	0.971	0.969	1.076	1.013	1.066	1.099	1.132	0.969	0.943	1.095	1.463	1.465	1.449
TRINITY_DN778_c0_g1_i1_orf1	uncharacterized protein LOC114363281 [Ostrinia furnacalis]	1	1039	115.4	9.09	1	1	1	High	1	0.944	1.115	0.656	0.731	0.715	0.857	0.712	0.813	0.534	0.475	0.648	5.326	4.936	5.483
TRINITY_DN9615_c0_g1_i1_orf1	uncharacterized protein LOC114352730 [Ostrinia furnacalis]	1	711	77.3	5.81	2	1	1	High	1	1.056	1.174	1.442	1.541	1.47	1.454	1.586	1.512	1.513	1.483	1.893	1.998	1.838	1.78
TRINITY_DN1651_c0_g2_i1_orf1	UBX domain-containing protein 6 [Ostrinia furnacalis]	1	189	22	5.76	9	1	1	High	1	1.076	1.218	1.902	1.705	1.699	1.811	1.601	1.717	1.567	2.294	2.081	2.2	1.927	1.963
TRINITY_DN14464_c0_g1_i1_orf1	GMP synthase [glutamine-hydrolyzing] [Chelonus insularis]	1	714	78.7	6.8	2	1	1	High	1	0.96	1.094	0.726	0.72	0.683	0.57	0.656	0.676	0.535	0.568	0.647	0.56	0.606	0.647
TRINITY_DN1848_c0_g1_i2_orf1	uncharacterized protein LOC114351337 [Ostrinia furnacalis]	1	385	40.5	6.99	2	1	1	High	1	0.786	0.946	1.164	1.175	1.252	1.583	1.793	1.043	1.286	1.439	1.365	1.14	1.263	1.418
TRINITY_DN15736_c0_g1_i2_orf1	uncharacterized protein LOC114355289 isoform X3 [Ostrinia furnacalis]	2	522	58	8.6	4	2	2	High	1	1.104	0.836	0.543	0.772	0.722	1.05	0.784	0.97	0.828	0.699	0.865	1.034	1.136	1.023
TRINITY_DN313_c0_g1_i5_orf1	collagen alpha-1(X) chain-like [Ostrinia furnacalis]	1	356	36.1	8.48	2	1	1	High	1	0.966	0.962	0.477	0.556	0.507	0.529	0.563	0.461	0.436	0.334	0.424	0.309	0.244	0.341
TRINITY_DN19110_c0_g1_i2_orf1	peroxidase [Ostrinia furnacalis]	1	750	84.9	6.57	2	1	1	High	1	1.008	1.028	1.389	1.359	1.163	1.32	1.137	1.514	1.681	1.946	2.053	2.502	2.955	2.639
TRINITY_DN4463_c0_g1_i2_orf1	uncharacterized protein LOC113521486 isoform X3 [Galleria mellonella]	1	1408	153.3	5.67	1	1	1	High	1	0.929	0.981	0.763	0.684	0.746	0.76	0.877	0.973	0.761	0.679	0.562	0.862	0.903	0.657
TRINITY_DN13094_c0_g1_i1_orf1	probable ATP-dependent RNA helicase DDX56 [Ostrinia furnacalis]	1	567	64.1	9.16	2	1	1	High	1	1.016	0.945	0.615	0.573	0.51	0.798	0.641	0.703	0.717	1.048	0.683	0.562	0.592	0.548
TRINITY_DN108122_c0_g1_i9_orf1	hypothetical protein SFRUCORN_003152 [Spodoptera frugiperda]	2	91	10.8	9.25	20	2	2	High	1	1.004	0.997	0.341	0.338	0.62	0.622	0.327	0.371	0.368	0.286	0.345	0.409	0.379	0.347
TRINITY_DN33418_c0_g1_i1_orf1	homer protein homolog 2-like [Ostrinia furnacalis]	1	192	20.5	8.43	7	1	1	High	1	4.164	0.696	1.47	1.029	0.922	0.733	0.883	0.8	0.695	0.566	0.737	1.474	1.36	1.817
TRINITY_DN1989_c0_g1_i1_orf1	sarcoplasmic calcium-binding protein 1 isoform X1 [Ostrinia furnacalis]	1	195	22.6	4.7	8	1	1	High	1	1.127	0.951	1.001	0.822	0.872	1.193	1.044	1.11	1.025	1.698	1.154	0.66	0.633	0.721
TRINITY_DN2371_c0_g1_i10_orf1	ileal sodium/bile acid cotransporter-like isoform X1 [Ostrinia furnacalis]	1	465	50.7	8.43	2	1	1	High	1	0.825	0.942	0.873	0.824	1.069	1.046	1.069	0.977	0.946	0.94	0.961	0.923	1.206	1.084
TRINITY_DN17655_c0_g1_i1_orf1	BRISC and BRCA1-A complex member 1-like [Ostrinia furnacalis]	1	296	34.2	5.66	3	1	1	High	1	0.928	0.88	0.769	0.861	0.901	0.714	0.67	0.681	0.851	0.748	0.707	1.321	1.107	1.19
TRINITY_DN34134_c0_g2_i1_orf1	THUMP domain-containing protein 1 homolog [Ostrinia furnacalis]	1	289	33.2	8.48	4	1	1	High	1	0.963	1.079	1.807	1.61	1.52	2.079	1.695	1.94	1.517	1.51	1.44	1.222	1.173	1.286
TRINITY_DN51766_c0_g1_i2_orf1	facilitated trehalose transporter Tret1-like [Ostrinia furnacalis]	1	460	49.7	6.54	3	1	1	High	1	1.041	1.024	0.153	0.174	0.202	0.173	0.184	0.175	0.164	0.146	0.169	0.305	0.342	0.264
TRINITY_DN9455_c0_g1_i6_orf1	uncharacterized protein LOC114360866 isoform X4 [Ostrinia furnacalis]	1	1469	158.2	5.17	1	1	1	High	1	1.164	1.163	0.602	0.578	0.837	0.68	0.937	0.738	0.745	0.601	0.976	1.878	1.914	1.782
TRINITY_DN86127_c1_g1_i2_orfp1	TRINITY_DN86127_c1_g1_i2_m.43062 TRINITY_DN86127_c1_g1::TRINITY_DN86	1	68	7.8	9.52	16	1	1	High	1	1.039	0.978	0.759	0.828	0.715	1.03	1.431	1.348	1.279	1.204	1.376	0.5	0.498	0.549
TRINITY_DN19413_c0_g1_i2_orf1	U4/U6 small nuclear ribonucleoprotein Prp4 [Papilio xuthus]	1	126	14.9	9.14	10	1	1	High	1	0.94	1.011	0.658	0.725	0.714	0.714	0.798	0.719	0.732	0.692	0.608	0.739	0.68	0.619
TRINITY_DN8598_c0_g1_i2_orf1	tyrosine--tRNA ligase, mitochondrial [Ostrinia furnacalis]	2	462	52.3	8.73	5	2	2	High	1	0.917	1.048	0.671	0.592	0.654	0.61	0.652	0.636	0.699	0.65	0.568	0.709	0.647	0.635
TRINITY_DN22572_c0_g1_i1_orf1	eukaryotic translation elongation factor 1 epsilon-1 [Ostrinia furnacalis]	1	173	19.3	8.25	5	1	1	High	1	0.955	0.914	0.666	0.599	0.688	0.733	0.705	0.62	0.471	0.496	0.537	0.685	0.739	0.72
TRINITY_DN8440_c0_g1_i9_orf1	protein FAM177A1-like [Ostrinia furnacalis]	2	143	15.9	4.61	16	2	2	High	1	0.919	0.973	1.491	1.543	1.574	1.601	1.839	1.811	1.495	1.727	1.441	1.301	1.447	1.444
TRINITY_DN57202_c0_g1_i1_orf1	PREDICTED: U4/U6 small nuclear ribonucleoprotein Prp31 [Amyelois transitella	1	499	55.5	8.78	3	1	1	High	1	0.908	0.901	0.256	0.274	0.255	0.29	0.305	0.299	0.282	0.267	0.254	0.264	0.323	0.219

TRINITY_DN4689_c0_g1_i5_orf1	pericentriolar material 1 protein-like isoform X3 [Ostrinia furnacalis]	2	1732	193.5	6.01	1	2	2	High	1	0.824	0.925	0.828	0.804	0.82	1.015	0.829	0.941	0.966	0.939	1.639	2.373	2.059	2.789
TRINITY_DN26375_c0_g1_i1_orf1	hypothetical protein O3G_MSEX007366 [Manduca sexta]	1	1193	129.6	4.64	2	1	1	High	1	0.949	0.903	0.256	0.301	0.28	0.346	0.345	0.333	0.301	0.271	0.34	0.444	0.362	0.354
TRINITY_DN4937_c0_g1_i2_orf1	zinc finger protein 778-like [Ostrinia furnacalis]	1	446	50.8	7.2	3	1	1	High	1	0.972	0.932	0.903	1.048	0.863	0.872	0.812	0.804	0.629	0.762	0.733	1.053	0.94	1.316
TRINITY_DN3176_c0_g1_i2_orf1	dnaJ homolog subfamily B member 6 isoform X2 [Ostrinia furnacalis]	1	283	32.1	9.63	6	1	1	High	1	0.994	1.114	0.725	0.546	0.617	0.645	0.654	0.551	0.631	0.539	0.661	0.875	0.843	0.859
TRINITY_DN11928_c0_g1_i3_orf1	leucyl-cystinyl aminopeptidase-like isoform X4 [Ostrinia furnacalis]	2	1065	120.7	5.54	1	2	2	High	1	0.915	1.018	0.508	0.582	0.551	0.538	0.581	0.585	0.58	0.575	0.583	0.673	0.68	0.625
TRINITY_DN22871_c0_g2_i1_orf1	mitochondrial import inner membrane translocase subunit Tim17-B [Ostrinia fu	1	165	17.6	6.54	5	1	1	High	1	0.953	1.053	0.622	0.54	0.626	0.709	0.597	0.74	0.735	0.869	0.684	0.658	0.71	0.654
TRINITY_DN7073_c0_g1_i1_orf1	unnamed protein product, partial [Brenthis ino]	2	156	17.7	8.28	10	2	2	High	1	1.063	1.006	0.428	0.392	0.403	0.446	0.326	0.383	0.318	0.295	0.361	0.339	0.354	0.308
TRINITY_DN17368_c0_g1_i6_orf1	protein bark beetle [Ostrinia furnacalis]	1	3018	341.9	6.35	0	1	1	High	1	1.126	1.062	1.3	1.475	1.066	1.118	1.517	1.52	1.665	2.294	1.495	1.655	1.518	1.416
TRINITY_DN13515_c0_g1_i1_orf1	low-density lipoprotein receptor-related protein 4-like isoform X2 [Ostrinia fu	2	1925	216.2	5.94	1	2	2	High	1	1.089	1.06	1.36	1.322	1.335	1.466	1.543	1.173	1.445	1.58	1.328	1.406	1.341	1.372
TRINITY_DN16978_c0_g1_i1_orf1	la-related protein 7 [Helicoverpa armigera]	2	476	54.5	8.78	5	2	2	High	1	0.971	1.081	0.941	1.005	0.901	0.912	0.959	0.89	1.161	1.389	1.055	0.762	0.773	0.796
TRINITY_DN11986_c0_g1_i1_orf1	DNA replication licensing factor Mcm2 [Ostrinia furnacalis]	1	884	100.3	5.44	2	1	1	High	1	1.231	1.012	1.182	1.002	1.242	1.36	1.16	1.208	1.194	1.192	0.611	0.781	0.658	0.67
TRINITY_DN17417_c0_g1_i11_orf1	sodium/hydrogen exchanger 9B2-like isoform X4 [Ostrinia furnacalis]	1	692	75.4	7.69	3	1	1	High	1	1.069	1.076	0.362	0.569	0.541	0.566	0.636	0.576	0.54	0.518	0.586	0.427	0.426	0.439
TRINITY_DN127056_c0_g1_i1_orf1	pantothenate kinase 4 [Ostrinia furnacalis]	1	356	40	5.31	3	1	1	High	1	1.353	1.552	1.015	1.298	1.093	0.882	1.113	1.228	0.811	1.024	0.904	1.223	1.099	1.163
TRINITY_DN16408_c0_g1_i1_orf1	ABC transporter G family member 20 isoform X1 [Ostrinia furnacalis]	1	784	87.5	6.92	2	1	1	High	1	0.961	0.903	0.737	0.725	0.796	0.71	0.693	0.671	0.648	0.57	0.605	0.706	0.712	0.585
TRINITY_DN19584_c0_g1_i2_orf1	protein NDUFAF4 homolog [Ostrinia furnacalis]	1	218	25.1	8.18	6	2	1	High	1	1.296	1.355	0.808	0.656	0.684	0.923	0.806	1.32	0.677	0.82	0.725	1.262	0.953	1.203
TRINITY_DN26429_c0_g1_i4_orf1	zinc transporter 9 [Ostrinia furnacalis]	2	580	65.5	8.32	3	2	2	High	1	1.004	1.038	0.663	0.75	0.735	0.731	0.696	0.643	0.662	0.628	0.705	0.794	0.838	0.818
TRINITY_DN28660_c0_g1_i4_orf1	iron-sulfur cluster assembly 1 homolog, mitochondrial [Ostrinia furnacalis]	1	131	14.2	9.04	9	1	1	High	1	1.052	1.337	0.784	0.892	0.994	1.005	1.128	1.051	1.012	1.059	0.985	1.023	1.106	1.18
TRINITY_DN6059_c0_g1_i1_orf1	brachyurin-like [Ostrinia furnacalis]	1	290	30.1	7.75	4	1	1	High	1	0.962	1.115	0.254	0.206	0.233	0.208	0.245	0.279	0.247	0.221	0.238	0.176	0.144	0.222
TRINITY_DN37585_c0_g1_i1_orf1	cuticle protein 19.8-like [Ostrinia furnacalis]	1	175	17.7	7.75	10	1	1	High	1	0.968	0.987	0.841	1.039	1.254	0.902	1.311	1.121	1.228	1.58	1.151	2.305	2.349	2.024
TRINITY_DN42177_c0_g1_i4_orf1	androgen-dependent TFPI-regulating protein-like [Ostrinia furnacalis]	2	234	27.1	9.28	6	3	2	High	1	0.967	0.975	0.977	1.013	1.04	0.858	0.86	0.892	0.824	0.745	0.827	0.751	0.812	0.694
TRINITY_DN40999_c0_g1_i1_orf1	nucleoporin GLE1 [Ostrinia furnacalis]	1	646	73.9	7.5	2	1	1	High	1	1.044	1.121	1.129	1.141	1.138	1.07	1.117	1.385	1.075	1.051	1.221	1.141	1.126	1.128
TRINITY_DN8083_c0_g1_i1_orf1	solute carrier family 35 member F6 [Ostrinia furnacalis]	1	365	39.9	6.87	2	1	1	High	1	1.019	0.993	1.946	1.714	1.811	1.505	1.342	1.45	1.368	1.25	1.427	2.102	2.131	2.187
TRINITY_DN25987_c0_g1_i5_orf1	GILT-like protein 2 isoform X1 [Ostrinia furnacalis]	1	92	10.3	6.27	12	2	1	High	1	1.035	1.032	1.237	1.407	1.56	1.034	1.209	1.12	0.925	1.325	0.842	2.153	2.229	2.068
TRINITY_DN19043_c0_g3_i2_orf1	hypothetical protein EVAR_60654_1 [Eumeta japonica]	2	255	26.5	9.45	8	2	2	High	1	0.936	0.953	0.991	1.152	1.049	1.394	1.526	1.445	1.476	1.282	1.17	1.806	1.895	1.724
TRINITY_DN114198_c0_g1_i1_orf1	catalase isozyme 1, partial [Sturnira hondurensis]	1	492	56.9	7.14	3	1	1	High	1	0.96	0.881	0.401	0.4	0.491	0.758	0.865	0.518	0.538	0.525	0.444	0.427	0.511	0.518
TRINITY_DN33728_c0_g2_i1_orf1	uncharacterized protein LOC114350200 [Ostrinia furnacalis]	1	198	23	6.87	4	1	1	High	1	1.058	1.31	1.208	1.063	1.587	1.509	1.522	1.472	1.912	1.525	1.774	2.886	2.993	2.334
TRINITY_DN7064_c0_g1_i19_orf1	unnamed protein product [Chilo suppressalis]	1	652	73	7.52	2	1	1	High	1	1.915	1.219	5	5.356	4.848	7.966	9.01	8.353	6.914	5.701	8.225	1.553	1.212	1.597
TRINITY_DN10939_c0_g1_i5_orf1	glia maturation factor beta [Ostrinia furnacalis]	1	141	16.3	5.21	7	1	1	High	1	0.896	1.019	0.758	1.264	1.097	0.856	1.037	0.926	1.105	0.979	0.923	1.064	0.876	1.178
TRINITY_DN640_c0_g1_i2_orf1	pancreatic triacylglycerol lipase-like [Ostrinia furnacalis]	1	211	23.2	10.73	6	1	1	High	1	1.073	1.073	0.874	0.805	0.839	0.907	0.927	0.858	0.756	0.976	0.821	0.918	0.649	0.707
TRINITY_DN7391_c0_g1_i2_orf1	hypothetical protein evm_000945 [Chilo suppressalis]	1	419	44.9	8.05	2	1	1	High	1	0.984	0.96	0.694	0.669	0.742	0.88	0.757	0.87	0.791	0.862	0.697	1.056	0.818	1.068
TRINITY_DN22654_c0_g2_i4_orf1	protein EFR3 homolog cmp44E isoform X1 [Ostrinia furnacalis]	1	836	92.8	6.8	2	1	1	High	1	0.876	0.888	0.567	0.625	0.615	0.575	0.61	0.564	0.539	0.538	0.597	0.713	0.735	0.772
TRINITY_DN146718_c0_g1_i1_orf1	40S ribosomal protein S6 [Diachasma alloeum]	2	251	28.7	10.62	8	7	1	High	1	1.048	0.946	0.647	0.683	0.658	0.744	0.78	0.719	0.594	0.52	0.565	0.649	0.621	0.639
TRINITY_DN14730_c0_g1_i7_orf1	titin homolog [Ostrinia furnacalis]	1	2941	324.7	4.68	0	1	1	High	1	1.18	1.201	0.509	0.56	0.539	0.408	0.554	0.523	0.443	0.459	0.503	0.489	0.565	0.414
TRINITY_DN12372_c0_g1_i4_orf1	WD repeat-containing protein 44 isoform X4 [Ostrinia furnacalis]	1	1026	112.1	7.23	1	1	1	High	1	0.872	0.953	0.749	0.889	0.814	0.723	0.881	0.763	0.756	0.611	0.614	0.733	0.665	0.56
TRINITY_DN22375_c0_g1_i4_orf1	venom carboxylesterase-6-like [Ostrinia furnacalis]	1	105	11.6	6.52	7	1	1	High	1	1.189	1.14	1.216	1.599	1.564	1.942	1.89	2.075	2.186	1.935	2.061	1.529	1.829	1.521
TRINITY_DN23790_c0_g1_i1_orf1	wiskott-Aldrich syndrome protein family member 2 [Ostrinia furnacalis]	1	321	36.3	9.73	2	1	1	High	1	1.069	1.023	0.731	0.773	0.842	0.872	0.744	0.705	0.595	0.777	0.642	0.965	0.971	0.934
TRINITY_DN5767_c0_g1_i4_orf1	cell division cycle 5-like protein [Helicoverpa armigera]	2	334	38.6	9.48	8	2	2	High	1	0.995	0.925	0.578	0.473	0.558	0.543	0.576	0.501	0.56	0.59	0.449	0.633	0.693	0.512
TRINITY_DN9871_c0_g1_i11_orf1	PEST proteolytic signal-containing nuclear protein-like [Ostrinia furnacalis]	1	217	24.7	10.2	6	2	1	High	1	1.154	0.992	1.329	1.477	1.423	1.099	1.167	1.011	1.01	0.861	1.124	0.417	0.471	0.418
TRINITY_DN15961_c0_g1_i1_orf1	uncharacterized protein LOC113522423 [Galleria mellonella]	1	103	12.2	9.64	8	1	1	High	1	1.031	1.124	1.402	1.675	2.09	2.238	1.766	1.691	1.896	1.958	2.045	2.16	2.139	2.232
TRINITY_DN1380_c0_g1_i6_orf1	ubiquitin-fold modifier-conjugating enzyme 1 [Ostrinia furnacalis]	1	90	10.8	7.18	12	1	1	High	1	0.951	0.957	0.955	1.048	1.006	0.948	0.971	1.005	1.011	0.967	1.023	1.008	0.834	0.986
TRINITY_DN55160_c0_g2_i1_orf1	esterase FE4-like isoform X2 [Ostrinia furnacalis]	1	305	34.3	5.59	4	1	1	High	1	1.217	1.213	0.129	0.055	0.189	0.165	0.169	0.225	0.304	0.275	0.288	0.185	0.26	0.202
TRINITY_DN41708_c0_g1_i1_orf1	facilitated trehalose transporter Tret1-like [Ostrinia furnacalis]	1	501	55.1	9.5	2	1	1	High	1	1.027	1.021	0.831	0.786	1.049	1.134	1.278	1.207	1.015	0.921	0.811	0.575	0.639	0.598
TRINITY_DN5538_c0_g1_i1_orf1	hypothetical protein evm_010760 [Chilo suppressalis]	1	126	13.7	4.98	9	2	1	High	1	1.062	0.887	1.325	1.055	1.135	1.304	1.231	1.506	1.757	1.792	1.11	1.62	1.458	1.589
TRINITY_DN34745_c0_g2_i1_orf1	GSK3-beta interaction protein-like [Galleria mellonella]	1	118	13.3	5.07	8	1	1	High	1	1.038	0.826	0.653	0.742	0.795	0.792	0.727	0.731	0.702	0.609	0.768	1.122	1.094	1.083
TRINITY_DN5042_c0_g1_i4_orf1	mediator of RNA polymerase II transcription subunit 25-like isoform X2 [Ostrin	1	704	75.7	8.34	2	1	1	High	1	0.869	0.918	0.875	0.983	1.027	0.96	1.155	1.136	0.986	1.02	0.844	0.936	1.115	1.064
TRINITY_DN5045_c0_g1_i6_orf1	mitochondrial chaperone BCS1 [Ostrinia furnacalis]	1	424	48.4	8.16	4	1	1	High	1	0.874	1.043	0.797	1.033	0.726	1.186	0.994	1.202	1.041	1.394	1.039	1.035	0.942	0.931
TRINITY_DN10183_c0_g2_i3_orf1	uncharacterized protein LOC114360370 isoform X1 [Ostrinia furnacalis]	1	220	24.4	8.34	3	1	1	High	1	0.932	1.151	0.896	1.131	1.052	1.109	0.881	0.901	0.764	0.655	0.685	1.158	1.244	1.144
TRINITY_DN445_c0_g1_i2_orf1	sorting nexin-17 [Ostrinia furnacalis]	1	479	54.7	6.54	3	1	1	High	1	0.979	1.046	1.356	1.341	1.244	0.956	1.288	1.275	1.131	1.177	1.277	1.594	1.885	1.504
TRINITY_DN14063_c0_g1_i7_orf1	probable phosphorylase b kinase regulatory subunit beta isoform X1 [Ostrinia	1	1185	134.3	7.43	1	1	1	High	1	0.966	1.058	0.43	0.481	0.393	0.504	0.484	0.533	0.466	0.37	0.381	0.423	0.371	0.399
TRINITY_DN38392_c0_g1_i1_orf1	enoyl-CoA hydratase domain-containing protein 3 [Agrotis segetum]	1	29	3.4	9.54	41	1	1	High	1	1.038	1.268	0.894	1.053	1.064	2.098	1.875	2.027	1.295	1.183	1.437	1.731	1.591	1.571
TRINITY_DN5200_c0_g1_i2_orf1	uncharacterized protein LOC114351644 [Ostrinia furnacalis]	1	139	15.5	9.16	9	1	1	High	1	0.93	1.06	0.879	0.933	1.038	0.964	0.862	0.797	0.808	0.639	0.853	1.103	1.141	1.343
TRINITY_DN2890_c0_g1_i2_orf1	alanine aminotransferase 1 [Chelonus insularis]	1	270	30.1	8.75	3	1	1	High	1	1.167	1.034	1.202	1.073	1.044	1.187	0.985	1.078	0.966	0.96	0.892	0.693	0.738	0.652
TRINITY_DN639_c0_g1_i10_orf1	unnamed protein product, partial [Iphiclides podalirius]	1	191	21.3	8.29	7	1	1	High	1	1.07	0.879	0.661	1.033	0.832	0.697	0.812	0.714	0.754	0.735	0.583	0.72	0.61	0.758
TRINITY_DN34015_c0_g1_i7_orf1	nitrate reductase [NADPH]-like isoform X1 [Manduca sexta]	1	133	14.8	9.89	7	2	1	High	1	1.093	1.037	0.737	0.801	0.849	0.87	0.885	0.854	0.943	0.821	0.964	0.83	0.976	0.859
TRINITY_DN26010_c0_g1_i2_orf1	cytochrome b-c1 complex subunit 10-like [Ostrinia furnacalis]	1	58	6.3	8.53	14	3	1	High	1	0.965	0.903	0.227	0.2	0.256	0.202	0.198	0.212	0.245	0.19	0.237	0.201	0.208	0.211
TRINITY_DN44491_c0_g1_i12_orf1	unnamed protein product [Chrysodeixis includens]	1	524	57.7	8.5	2	1	1	High	1	0.948	0.929	1.355	2.178	1.847	2.123	1.732	1.5	1.278	1.713	1.4	1.007	1.06	0.983
TRINITY_DN5597_c0_g1_i2_orf1	monocarboxylate transporter 9-like [Ostrinia furnacalis]	1	518	56.7	9.29	2	1	1	High	1	0.959	1	0.521	0.629	0.562	0.645	0.691	0.615	0.684	0.677	0.611	0.555	0.546	0.472
TRINITY_DN14035_c0_g1_i1_orf1	protein takeout-like [Ostrinia furnacalis]	1	242	26.7	8.34	4	1	1	High	1	1.077	1.081	0.567	0.601	0.583	0.512	0.651	0.655	0.594	0.527	0.568	0.537	0.522	0.524
TRINITY_DN6424_c0_g1_i2_orf1	protein tweety-like [Ostrinia furnacalis]	1	561	57.9	9.35	5	1	1	High	1	0.904	1.124	1.304	1.352	1.253	1.291	1.645	1.464	1.241	3.935	1.787	1.471	1.513	1.657
TRINITY_DN5300_c0_g1_i2_orf1	valacyclovir hydrolase [Ostrinia furnacalis]	1	76	9.1	5.41	16	1	1	High	1	1.147	1.269	1.68	1.682	1.806	1.616	1.807	1.832	1.766	1.638	1.789	1.559	1.448	1.622
TRINITY_DN112706_c0_g1_i2_orf1	alaserpin-like isoform X4 [Ostrinia furnacalis]	1	64	7	5.19	25	1	1	High	1	0.935	1.058	0.88	1.188	1.029	0.825	0.932	0.889	0.774	0.703	0.887	0.929	0.701	0.794
TRINITY_DN82810_c0_g1_i1_orf1	putative carbonic anhydrase 3 [Ostrinia furnacalis]	1	320	35.3	7.36	3	1	1	High	1	0.971	1.18	0.606	0.519	0.602	0.782	0.544	0.712	0.517	0.722	0.384	0.74	0.72	0.754
TRINITY_DN81719_c0_g1_i1_orf1	tyrosine 3-monooxygenase isoform X1 [Ostrinia furnacalis]	1	560	63.5	5.53	2	1	1	High	1	1.11	1.111	0.595	0.506	0.564	0.522	0.612	0.665	0.627	0.725	0.566	0.625	0.658	0.711
TRINITY_DN25681_c0_g1_i5_orf1	hypothetical protein evm_005766 [Chilo suppressalis]	1	941	108.4	5.94	2	1	1	High	1	0.967	0.955	0.606	0.711	0.527	0.718	0.635	0.574	0.736	0.868	0.746	0.51	0.478	0.587
TRINITY_DN46447_c0_g1_i5_orf1	calmodulin-A-like [Ostrinia furnacalis]	1	181	20.8	4.53	5	1	1	High	1	0.908	0.943	0.762	0.794	0.867	0.708	0.872	1	0.922	0.601	0.73	0.925	0.787	0.669
TRINITY_DN133414_c0_g1_i1_orf1	protein strawberry notch-like isoform X2 [Venturia canescens]	1	1370	151	8.12	1	1	1	High	1	1.014	1.136	1.266	1.469	1.117	1.103	1.31	1.222	1.055	1.048	1.206	1.039	1.168	0.997
TRINITY_DN5281_c0_g2_i3_orf1	serine/threonine-protein kinase RIO2 isoform X2 [Ostrinia furnacalis]	1	454	51.7	5.69	3	1	1	High	1	0.975	0.841	0.748	0.993	0.761	0.701	0.786	0.784	0.647	0.85	0.719	0.572	0.517	0.608
TRINITY_DN8569_c1_g2_i7_orf1	furin-like protease 1, partial [Ostrinia furnacalis]	1	1066	116.2	7.39	1	1	1	High	1	1.069	1.129	1.086	1.151	1.206	1.099	1.14	0.983	1.032	0.845	0.914	1.633	1.847	1.674
TRINITY_DN3474_c1_g2_i7_orf1	LOW QUALITY PROTEIN: endoplasmic reticulum metallopeptidase 1-like [Ostri	1	478	53.3	5.83	2	1	1	High	1	0.916	0.992	0.554	0.545	0.592	0.565	0.697	0.636	0.569	0.561	0.473	0.622	0.755	0.623
TRINITY_DN13944_c0_g1_i1_orf1	vacuolar protein sorting-associated protein 52 homolog [Ostrinia furnacalis]	1	664	76.8	6.29	3	1	1	High	1	1.094	1.145	0.6	0.585	0.556	0.653	0.67	0.642	0.565	0.814	0.552	0.608	0.641	0.625
TRINITY_DN41997_c0_g1_i2_orf1	39S ribosomal protein L23, mitochondrial [Ostrinia furnacalis]	1	150	18	9.28	8	1	1	High	1	1.072	1.149	0.681	0.743	0.87	0.803	0.675	0.787	0.679	0.8	0.751	0.807	0.732	0.793
TRINITY_DN17423_c0_g1_i2_orf1	Heterogeneous nuclear ribonucleoproteins C1/C2 variant, partial [Homo sapie	1	300	33.1	5.03	4	1	1	High	1	0.849	0.762	0.8	1.129	1.026	0.985	1.392	1.292	1.219	1.231	1.616	0.886	1.036	1.021
TRINITY_DN133228_c0_g1_i3_orf1	microtubule-actin cross-linking factor 1 isoform X15 [Ostrinia furnacalis]	1	103	11.5	11.09	8	2	1	High	1	0.951	0.876	0.695	0.747	0.806	0.773	0.82	0.819	0.759	0.77	0.74	0.614	0.606	0.66
TRINITY_DN670_c0_g1_i15_orf1	hypothetical protein evm_002669 [Chilo suppressalis]	1	488	55.1	8.09	3	1	1	High	1	1.189	1.276	1.176	1.096	1.018	1.096	1.119	0.888	0.87	0.705	0.965	1.056	0.954	1.131
TRINITY_DN113272_c0_g1_i1_orf1	altered inheritance of mitochondria protein 3-like [Ostrinia furnacalis]	1	296	33	7.5	5	1	1	High	1	1.03	0.97	0.621	0.672	0.566	0.599	0.681	0.696	0.575	0.434	0.597	3.34	2.966	2.924
TRINITY_DN3503_c0_g1_i1_orfp1	uncharacterized protein LOC114356429 [Ostrinia furnacalis]	1	127	14.4	5.5	9	1	1	High	1	0.883	0.956	0.396	0.45	0.4	0.499	0.486	0.469	0.486	0.405	0.411	0.412	0.493	0.532
TRINITY_DN40197_c0_g1_i1_orf1	UDP-N-acetylglucosamine--dolichyl-phosphate N-acetylglucosaminephosph	1	395	44.7	7.34	3	2	1	High	1	0.968	1.063	0.742	0.726	0.8	0.694	0.689	0.828	0.778	0.68	0.639	0.866	0.867	0.843
TRINITY_DN23838_c0_g1_i4_orf1	ubiquitin domain-containing protein 2 isoform X1 [Ostrinia furnacalis]	2	235	26.1	5.94	7	2	2	High	1	0.944	1.024	0.669	0.719	0.804	0.682	0.706	0.747	0.608	0.68	1.71	0.922	1.099	0.931
TRINITY_DN33837_c0_g1_i6_orf1	bolA-like protein DDB_G0274169 [Ostrinia furnacalis]	1	130	14.6	9.5	6	1	1	High	1	0.839	0.927	0.833	0.898	0.941	0.812	0.873	0.887	0.889	0.822	0.819	1.045	1.114	1.076
TRINITY_DN105574_c0_g1_i1_orf1	prolow-density lipoprotein receptor-related protein 1, partial [Ostrinia furnaca	1	193	20.1	4.36	6	1	1	High	1	1.118	1.028	2.058	2.243	1.803	1.963	2.06	2.022	1.901	1.911	2.41	2.334	2.349	2.607
TRINITY_DN12014_c0_g1_i2_orf1	unnamed protein product [Chilo suppressalis]	1	908	99.8	8.5	1	1	1	High	1	1.186	1.173	2.436	2.204	2.085	1.396	1.692	1.473	1.409	1.464	1.518	2.066	1.876	2.004
TRINITY_DN1354_c5_g1_i1_orf1	PREDICTED: probable elongator complex protein 2 [Papilio xuthus]	1	67	7.5	6.51	18	1	1	High	1	1.117	1.262	1.266	1.279	1.168	1.207	1.3	1.255	1.186	1.479	1.519	1.692	1.289	1.659
TRINITY_DN21596_c0_g1_i1_orf1	peptidyl-prolyl cis-trans isomerase NIMA-interacting 4 [Zerene cesonia]	1	135	14.1	9.63	9	1	1	High	1	1.036	0.995	0.939	1.018	0.919	0.939	0.948	0.909	0.846	0.799	0.847	0.803	0.748	0.805
TRINITY_DN4248_c0_g1_i4_orf1	hypothetical protein SFRURICE_003580 [Spodoptera frugiperda]	1	590	66.9	9	2	1	1	High	1	0.944	1.038	1.182	1.025	1.104	1.037	1.042	1.214	0.944	1.255	0.841	0.928	0.915	0.885
TRINITY_DN50571_c1_g1_i1_orf1	WD repeat-containing protein 46 [Ostrinia furnacalis]	1	574	65.3	9.51	2	1	1	High	1	0.989	0.93	0.335	0.35	0.379	0.418	0.502	0.403	0.464	0.457	0.354	0.64	0.628	0.706
TRINITY_DN6406_c0_g1_i1_orf1	protein FAM98A-like [Ostrinia furnacalis]	1	337	38.3	7.33	4	1	1	High	1	0.956	1.075	0.531	0.455	0.602	0.595	0.599	0.803	0.542	0.647	0.566	0.556	0.553	0.484
TRINITY_DN2874_c0_g1_i4_orf1	ATP-binding cassette sub-family G member 1-like [Ostrinia furnacalis]	1	619	69.6	8.47	1	1	1	High	1	1.255	1.078	0.778	0.908	0.845	0.774	0.857	1.048	0.868	0.801	0.906	0.961	1.086	1.098
TRINITY_DN48973_c0_g1_i5_orf1	uncharacterized protein LOC114351683 isoform X8 [Ostrinia furnacalis]	1	137	15.8	5.39	6	1	1	High	1	1.007	1.04	0.784	0.818	0.748	0.753	0.629	0.676	0.839	0.884	0.839	0.928	0.907	0.88
TRINITY_DN2475_c0_g2_i1_orf1	uncharacterized protein LOC114363767 [Ostrinia furnacalis]	1	358	40.2	8.46	4	1	1	High	1	0.904	0.954	0.713	0.847	0.697	0.692	0.841	0.79	0.826	0.772	0.788	0.876	1.015	0.933
TRINITY_DN27110_c0_g1_i4_orf1	uncharacterized protein LOC114366000 [Ostrinia furnacalis]	1	252	27.5	5.8	5	1	1	High	1	0.969	1.067	0.873	0.871	0.92	1.04	1.109	1.195	0.892	1.066	0.842	1.051	0.986	1.16
TRINITY_DN383_c0_g1_i1_orf1	probable Golgi SNAP receptor complex member 2 [Ostrinia furnacalis]	1	209	24.1	8.05	6	1	1	High	1	1.114	0.936	0.701	0.859	0.63	0.745	0.807	0.769	0.581	0.6	0.855	1.077	0.961	0.859
TRINITY_DN39532_c0_g1_i1_orf1	hypothetical protein evm_009649 [Chilo suppressalis]	1	311	35.1	5.21	2	1	1	High	1	1.036	0.898	0.5	0.539	0.432	0.502	0.573	0.48	0.483	0.361	0.361	0.617	0.736	0.587
TRINITY_DN9831_c0_g1_i1_orf1	hypothetical protein evm_002135 [Chilo suppressalis]	1	85	9.2	5.35	12	1	1	High	1	0.931	1.228	0.866	0.802	0.947	1.041	0.854	0.773	0.809	0.86	0.72	0.963	0.936	0.801
TRINITY_DN3407_c0_g1_i9_orf1	uncharacterized protein YJR142W [Ostrinia furnacalis]	1	329	37.7	5.69	4	1	1	High	1	0.937	0.973	0.937	1.147	1.277	1.009	1.019	1.124	1.336	1.143	1.231	1.274	1.252	1.319

TRINITY_DN2102_c0_g1_i11_orf1	prenylated Rab acceptor protein 1 isoform X4 [Ostrinia furnacalis]	2	167	18.6	9.17	11	2	2	High	1	0.848	0.838	0.857	0.852	0.875	0.946	1.071	1.012	0.818	0.966	0.913	1.197	1.253	1.163
TRINITY_DN2828_c0_g1_i4_orf1	CKLF-like MARVEL transmembrane domain-containing protein 4 [Ostrinia furn	1	173	19	7.37	4	2	1	High	1	1.002	1.001	1.135	0.755	1.007	1.199	0.981	1.651	1.556	1.245	0.778	0.95	0.917	0.795
TRINITY_DN53810_c0_g1_i1_orf1	39S ribosomal protein L53, mitochondrial [Pectinophora gossypiella]	1	142	15.6	9.95	8	1	1	High	1	1.03	1.022	0.476	0.478	0.633	0.614	0.565	0.489	0.503	0.516	0.713	0.718	0.639	0.675
TRINITY_DN47666_c0_g1_i4_orf1	PREDICTED: probable splicing factor 3B subunit 5 [Amyelois transitella]	1	85	9.9	6.35	15	1	1	High	1	1.088	0.992	0.747	0.814	0.798	0.673	0.78	0.825	0.817	0.591	0.708	0.915	1.022	0.807
TRINITY_DN21567_c0_g1_i7_orf1	transcription initiation factor TFIID subunit 4 isoform X1 [Ostrinia furnacalis]	1	829	88	9.38	2	1	1	High	1	1.262	0.974	1.276	1.306	1.158	1.183	1.155	0.858	1.257	1.419	1.168	1.05	1.097	0.973
TRINITY_DN1351_c0_g1_i1_orf1	PREDICTED: flavin reductase (NADPH) [Microplitis demolitor]	1	202	22.4	7.15	4	1	1	High	1	1.011	1.096	0.882	0.809	0.778	0.841	0.9	0.775	0.638	0.532	0.696	0.755	0.666	0.661
TRINITY_DN4979_c0_g2_i9_orf1	arf-GAP with dual PH domain-containing protein 1-like isoform X2 [Ostrinia fu	1	384	44.5	8.66	4	1	1	High	1	1.082	0.936	1.204	1.224	1.179	1.311	1.057	1.108	0.983	1.192	1.337	1.373	1.327	1.539
TRINITY_DN23119_c0_g1_i3_orf1	double-strand-break repair protein rad21 homolog isoform X1 [Ostrinia furna	1	944	99.9	5.29	1	1	1	High	1	1.074	1.068	0.978	0.978	1.067	0.89	1.068	1.027	1.072	1.147	1.197	0.944	0.89	1.25
TRINITY_DN29633_c0_g1_i8_orf1	transmembrane protein 87A isoform X1 [Ostrinia furnacalis]	1	527	59.4	6.27	2	1	1	High	1	1.083	1.09	0.992	1.15	1.162	0.887	0.971	0.801	0.699	0.972	0.878	1.459	1.381	1.228
TRINITY_DN89829_c0_g1_i1_orf1	PREDICTED: ubiquitin-conjugating enzyme E2 T [Microplitis demolitor]	1	144	16.4	7.28	5	2	1	High	1	1.028	1.058	0.836	0.757	0.864	0.775	0.748	0.8	0.785	0.741	0.683	1.008	0.959	0.993
TRINITY_DN5012_c0_g1_i6_orf1	putative serine protease K12H4.7 [Ostrinia furnacalis]	1	494	55	5.05	3	1	1	High	1	0.881	0.803	0.513	0.471	0.45	0.529	0.534	0.566	0.425	0.622	0.545	0.36	0.411	0.365
TRINITY_DN34347_c0_g1_i1_orf1	nesprin-1-like isoform X8 [Bombyx mandarina]	1	79	8.6	4.59	18	1	1	High	1	0.936	0.96	0.984	0.929	0.773	0.679	0.835	0.728	0.628	0.61	0.792	0.65	0.552	0.664
TRINITY_DN61536_c0_g7_i1_orf1	cubilin homolog [Ostrinia furnacalis]	1	150	17	6.25	5	1	1	High	1	0.868	1.033	1.219	1.289	1.007	1.067	1.089	1.416	1.345	1.221	1.053	1.61	1.354	1.301
TRINITY_DN213_c0_g1_i5_orf1	protein lap4-like [Ostrinia furnacalis]	1	760	83.1	5.27	2	1	1	High	1	1.141	1.327	1.295	1.516	1.469	1.429	1.601	1.718	1.167	1.246	1.447	1.633	1.392	1.603
TRINITY_DN7794_c0_g1_i1_orf1	laminin subunit gamma-1-like [Ostrinia furnacalis]	1	79	9	5.45	15	1	1	High	1	0.932	0.886	0.858	0.828	0.848	0.99	1.175	1.022	0.934	0.906	0.819	0.775	0.787	0.825
TRINITY_DN24132_c0_g1_i2_orf1	palmitoyltransferase ZDHHC5 isoform X1 [Ostrinia furnacalis]	1	489	54.7	9.39	2	1	1	High	1	0.928	1.006	1.002	1.029	1.168	1.19	1.005	0.985	1.162	0.757	1.016	1.483	1.237	1.229
TRINITY_DN879_c0_g1_i2_orf1	DNA-directed RNA polymerase I subunit RPA12 [Ostrinia furnacalis]	1	120	13.4	7.85	10	1	1	High	1	1.116	1.039	0.81	0.691	0.767	0.829	0.863	0.702	1.04	0.885	0.659	1.042	0.943	0.947
TRINITY_DN32479_c0_g1_i8_orf1	hypothetical protein evm_009815 [Chilo suppressalis]	1	516	56.4	6.13	2	1	1	High	1	1.047	1.06	0.477	0.436	0.477	0.48	0.483	0.488	0.372	0.325	0.392	0.29	0.307	0.177
TRINITY_DN6105_c0_g1_i1_orf1	plasminogen receptor (KT) isoform X2 [Ostrinia furnacalis]	1	151	17.8	8.43	5	1	1	High	1	1.003	1.319	1.19	1.012	1.149	1.078	1.127	1.142	1.311	1.108	1.084	1.12	1.143	0.948
TRINITY_DN766_c0_g1_i1_orf1	nucleolar complex protein 3 homolog [Ostrinia furnacalis]	2	298	32.6	9.17	6	2	2	High	1	0.926	1.067	0.891	0.88	0.875	0.773	0.867	0.765	0.737	0.867	0.786	0.733	0.915	0.913
TRINITY_DN45400_c0_g1_i1_orf1	nuclear pore complex protein Nup107 [Ostrinia furnacalis]	1	841	95.5	5.73	2	1	1	High	1	0.756	0.963	1.038	0.589	0.61	0.763	0.782	0.694	0.546	0.778	1.127	0.99	1.079	1.03
TRINITY_DN24693_c1_g1_i1_orf1	ubiquitin-conjugating enzyme E2 G2 isoform X2 [Ostrinia furnacalis]	1	165	18.4	4.73	9	1	1	High	1	0.824	1.013	0.549	0.577	0.604	0.615	0.623	0.639	0.608	0.627	0.57	0.646	0.618	0.53
TRINITY_DN13651_c0_g1_i2_orf1	40S ribosomal protein S12, mitochondrial [Ostrinia furnacalis]	1	160	17.4	11	6	1	1	High	1	0.992	0.962	0.233	0.241	0.304	0.22	0.253	0.242	0.193	0.186	0.204	0.273	0.338	0.333
TRINITY_DN23183_c1_g1_i2_orf1	myotubularin-related protein 9 [Ostrinia furnacalis]	1	560	64.3	6.61	2	1	1	High	1	1.07	1.256	1.228	1.274	1.244	1.226	1.132	1.102	0.987	0.876	0.922	2.05	1.893	1.524
TRINITY_DN1659_c0_g1_i3_orf1	beta-catenin-like protein 1 [Ostrinia furnacalis]	1	557	63.8	5.17	3	1	1	High	1	1.056	1.231	1.645	1.451	1.964	1.885	1.671	1.82	1.789	1.775	1.649	1.637	1.932	1.736
TRINITY_DN36856_c0_g1_i1_orf1	protein enhancer of sevenless 2B isoform X2 [Formica exsecta]	1	211	24.6	5.8	7	1	1	High	1	1.076	0.967	1.366	1.465	1.407	1.161	1.276	1.161	1.244	1.295	1.448	1.804	1.81	1.879
TRINITY_DN77830_c0_g2_i2_orf1	prostaglandin reductase 1-like [Leguminivora glycinivorella]	1	233	25	8.21	4	2	1	High	1	1.076	1.178	1.119	1.321	1.389	0.951	0.922	0.96	1.067	0.953	1.008	0.823	0.845	0.83
TRINITY_DN2695_c0_g1_i14_orfp1	TRINITY_DN2695_c0_g1_i14_m.44485 TRINITY_DN2695_c0_g1::TRINITY_DN269	2	664	73.5	7.96	5	2	2	High	1	1.089	0.948	0.316	0.3	0.346	0.496	0.573	0.496	0.301	0.284	0.329	0.177	0.229	0.219
TRINITY_DN25625_c0_g2_i1_orf1	keratin, type I cytoskeletal 18 [Mus musculus]	1	358	41	4.98	2	2	1	High	1	0.898	2.404	0.784	1.731	0.894	1.351	1.021	2.056	3.167	2.143	0.843	2.828	1.107	0.512
TRINITY_DN2181_c1_g1_i8_orf1	vacuolar protein sorting-associated protein 37B [Ostrinia furnacalis]	1	215	24.4	4.83	4	1	1	High	1	0.899	0.898	0.565	0.622	0.658	0.707	0.584	0.752	0.624	0.514	0.633	0.711	1.033	0.769
TRINITY_DN3110_c0_g1_i4_orf1	hypothetical protein B5X24_HaOG214278 [Helicoverpa armigera]	1	376	42.4	8.9	2	1	1	High	1	0.917	0.955	1.213	0.819	0.863	1.479	1.136	1.221	1.121	1.208	0.888	0.827	0.918	0.748
TRINITY_DN50875_c0_g1_i3_orf1	conserved oligomeric Golgi complex subunit 8 [Ostrinia furnacalis]	1	552	63.7	5.53	2	1	1	High	1	0.835	0.833	0.655	0.69	0.684	0.639	0.65	0.756	0.663	0.532	0.612	0.87	0.926	0.887
TRINITY_DN17907_c0_g1_i13_orf1	androgen-induced gene 1 protein-like isoform X1 [Galleria mellonella]	1	155	18.2	8.73	8	1	1	High	1	0.877	0.984	1.084	0.929	0.932	1.303	1.08	1.4	1.226	1.137	1.192	0.699	0.755	0.87
TRINITY_DN31377_c0_g2_i1_orf1	phosphatidate cytidylyltransferase, mitochondrial [Ostrinia furnacalis]	1	336	38.8	9.06	3	1	1	High	1	0.914	0.959	0.771	0.676	0.73	0.796	0.966	0.868	1.447	1.556	1.209	1.2	1.237	1.437
TRINITY_DN104507_c0_g1_i2_orf1	replication protein A 32 kDa subunit [Ostrinia furnacalis]	1	265	29.8	8.24	4	1	1	High	1	1.026	1.057	0.632	0.668	0.646	0.625	0.517	0.531	0.538	0.37	0.465	0.78	0.893	0.914
TRINITY_DN12700_c0_g1_i7_orf1	CWF19-like protein 1 [Galleria mellonella]	1	535	60.6	6.34	2	1	1	High	1	1.048	1.403	1.065	0.916	0.844	1.137	1.406	1.214	0.841	1.155	1.14	1.02	1.304	0.978
TRINITY_DN4291_c1_g2_i1_orf1	cleft lip and palate transmembrane protein 1-like protein [Ostrinia furnacalis]	1	539	61.9	8.95	2	1	1	High	1	0.791	0.937	1.079	0.858	0.838	0.954	1.016	1.119	1.052	2.047	0.947	1.083	0.831	1.135
TRINITY_DN40562_c0_g2_i1_orf1	dual specificity protein phosphatase 23-like isoform X2 [Ostrinia furnacalis]	1	238	27.1	5.43	5	1	1	High	1	1.024	0.963	0.486	0.516	0.565	0.58	0.611	0.61	0.534	0.612	0.511	0.811	0.75	0.704
TRINITY_DN47591_c1_g1_i1_orf1	uncharacterized protein LOC114364828 [Ostrinia furnacalis]	1	216	24.9	8.79	5	1	1	High	1	0.819	0.995	0.588	0.494	0.635	0.63	0.512	0.523	0.493	0.664	0.46	0.666	0.494	0.68
TRINITY_DN157_c0_g1_i4_orf1	ATP-binding cassette sub-family A member 1-like [Ostrinia furnacalis]	1	1784	200.3	5.6	0	1	1	High	1	1.203	1.028	0.583	0.622	0.5	0.725	0.56	0.644	0.491	0.596	0.596	0.561	0.638	0.417
TRINITY_DN11172_c1_g1_i1_orf1	juvenile hormone epoxide hydrolase-like isoform X1 [Ostrinia furnacalis]	1	291	33.1	7.62	6	1	1	High	1	1.009	1.027	1.332	1.09	1.323	1.298	1.35	1.503	1.538	2.628	1.213	0.965	1.141	1.125
TRINITY_DN64759_c0_g1_i1_orf1	mitochondrial inner membrane protein OXA1L-like [Ostrinia furnacalis]	1	396	44.1	9.16	2	1	1	High	1	0.993	1.071	0.642	0.871	0.867	0.935	0.771	0.786	0.869	0.899	0.6	0.643	0.57	0.575
TRINITY_DN6472_c0_g1_i5_orf1	unnamed protein product [Chilo suppressalis]	1	504	57.2	6.95	2	1	1	High	1	1.044	1.068	1.298	1.567	1.467	1.657	1.635	1.461	1.309	1.38	1.574	1.561	1.611	1.444
TRINITY_DN98242_c0_g1_i1_orf1	adenosine deaminase 2-A-like [Galleria mellonella]	1	498	57.4	6.48	1	1	1	High	1	1.07	1.069	0.785	0.778	0.816	0.567	0.617	0.627	0.61	0.504	0.529	2.335	2.707	2.61
TRINITY_DN42082_c0_g2_i2_orfp1	TRINITY_DN42082_c0_g2_i2_m.7835 TRINITY_DN42082_c0_g2::TRINITY_DN420	1	132	13.9	12.6	7	1	1	High	1	0.951	0.925	0.636	0.639	0.622	0.563	0.557	0.526	0.464	0.45	0.421	0.516	0.475	0.524
TRINITY_DN37538_c0_g3_i1_orf1	esterase FE4-like [Ostrinia furnacalis]	1	115	13.4	8.76	13	1	1	High	1	0.836	0.931	0.365	0.296	0.376	0.334	0.438	0.327	0.371	0.485	0.349	0.307	0.291	0.33
TRINITY_DN48846_c0_g1_i1_orf1	tudor domain-containing protein 7 isoform X3 [Ostrinia furnacalis]	1	116	12.3	10.59	10	1	1	High	1	0.991	0.938	1.569	1.516	1.416	1.462	1.393	1.478	1.417	1.444	1.48	1.302	1.454	1.184
TRINITY_DN115373_c0_g1_i1_orf1	protein mini spindles [Ostrinia furnacalis]	1	80	8.3	8.22	16	1	1	High	1	0.899	0.95	0.759	0.827	0.971	1.111	0.97	1.165	1.012	0.84	1.123	1.061	0.94	0.739
TRINITY_DN25783_c0_g1_i2_orf1	SET and MYND domain-containing protein 4 [Ostrinia furnacalis]	1	747	84	7.74	1	1	1	High	1	0.911	0.983	0.545	0.751	0.446	0.472	0.495	0.585	0.339	0.354	0.433	0.71	0.561	0.723
TRINITY_DN27300_c0_g1_i6_orfp1	TRINITY_DN27300_c0_g1_i6_m.71140 TRINITY_DN27300_c0_g1::TRINITY_DN27	1	128	14.4	8.29	9	1	1	High	1	1.013	1.153	2.295	2.149	2.122	0.932	0.862	0.969	0.877	0.736	0.818	0.764	0.718	0.634
TRINITY_DN12013_c0_g1_i6_orf1	tudor domain-containing protein 7 isoform X3 [Ostrinia furnacalis]	1	306	33.4	5.4	7	1	1	High	1	1.206	1.076	2.337	2.253	2.504	1.571	1.249	1.484	1.398	1.497	1.545	1.314	0.965	1.143
TRINITY_DN48404_c0_g1_i2_orf1	OCIA domain-containing protein 1-like isoform X1 [Ostrinia furnacalis]	1	246	26.6	6.35	6	1	1	High	1	0.986	1.008	1.102	0.941	0.97	0.984	1.124	1.074	1.3	1.322	1.029	1.153	1.046	0.922
TRINITY_DN6813_c1_g1_i1_orf1	pantothenate kinase 3 isoform X2 [Ostrinia furnacalis]	1	473	52.1	8.4	3	1	1	High	1	0.943	0.989	1.553	1.391	1.334	1.701	1.644	1.713	1.818	2.128	1.543	1.125	1.037	1.189
TRINITY_DN2675_c0_g1_i1_orf1	mitochondrial folate transporter/carrier [Ostrinia furnacalis]	1	320	35.9	9.88	4	1	1	High	1	0.834	0.923	0.841	0.935	0.831	0.952	1.123	1.073	1.088	1.169	0.691	1.312	0.721	0.687
TRINITY_DN921_c3_g3_i1_orf1	dual serine/threonine and tyrosine protein kinase-like [Ostrinia furnacalis]	1	140	15.7	4.97	6	1	1	High	1	1.124	1.089	1.11	0.97	1.052	1.305	1.081	1.153	0.84	1.067	1.042	0.976	0.986	0.995
TRINITY_DN42171_c0_g1_i1_orf1	amyloid-beta-like protein isoform X1 [Manduca sexta]	1	751	82.6	5	2	1	1	High	1	0.909	1.082	0.646	0.774	0.646	0.732	0.582	0.68	0.749	0.534	0.582	1.002	1.184	1.077
TRINITY_DN4062_c0_g2_i1_orf1	venom peptide BmKAPI-like isoform X2 [Ostrinia furnacalis]	1	97	10.4	6.47	9	1	1	High	1	0.957	0.902	2.174	2.299	2.272	1.901	1.754	1.87	1.639	1.555	1.875	1.522	1.652	1.393
TRINITY_DN3698_c0_g1_i4_orf1	neuroblastoma-amplified sequence-like [Ostrinia furnacalis]	1	1864	210.1	6.57	1	1	1	High	1	1.252	1.066	1.075	1.18	1.204	1.088	0.953	0.992	0.819	0.746	1.03	0.766	1.001	0.857
TRINITY_DN27114_c0_g1_i1_orf1	putative inorganic phosphate cotransporter [Ostrinia furnacalis]	1	63	7.1	8.88	17	1	1	High	1	0.865	1.041	0.9	1.001	0.916	0.802	0.985	0.878	0.936	1.162	0.923	1.274	1.297	1.228
TRINITY_DN1856_c0_g1_i3_orf1	hypothetical protein evm_006253 [Chilo suppressalis]	2	1001	111.1	9.67	1	2	2	High	1	1.029	1.067	0.799	0.786	0.766	0.76	0.857	0.74	0.751	0.654	0.773	0.937	0.906	0.935
TRINITY_DN95971_c0_g5_i1_orf1	exportin-1 [Diachasma alloeum]	1	540	62.1	6.16	1	1	1	High	1	0.839	0.879	0.9	1.027	0.981	0.856	0.87	0.898	0.874	0.748	0.869	0.615	0.768	0.687
TRINITY_DN3978_c0_g2_i1_orf1	nicastrin [Ostrinia furnacalis]	1	722	79.5	5.64	1	1	1	High	1	1.028	1.024	1.1	1.17	1.071	0.988	1.07	1.168	1.1	1.413	0.827	1.698	1.753	1.467
TRINITY_DN2918_c0_g1_i1_orf1	28S ribosomal protein S10, mitochondrial [Ostrinia furnacalis]	1	172	19.9	8.94	5	1	1	High	1	0.964	0.918	0.286	0.3	0.367	0.286	0.326	0.328	0.309	0.341	0.326	0.47	0.487	0.484
TRINITY_DN9019_c0_g1_i5_orf1	exosome complex component RRP46 [Ostrinia furnacalis]	1	213	23	5.16	5	1	1	High	1	0.808	0.933	1.134	1.041	1.171	1.018	1.529	1.555	1.393	1.604	1.061	1.109	1.104	1.241
TRINITY_DN117042_c0_g1_i2_orf1	UBX domain-containing protein 6 [Ostrinia furnacalis]	1	71	7.8	6.64	14	1	1	High	1	1.034	0.899	1.182	1.339	1.162	1.374	1.471	1.451	1.574	1.307	1.295	1.364	1.292	1.234
TRINITY_DN6785_c0_g1_i1_orf1	Similar to CG4038: Probable H/ACA ribonucleoprotein complex subunit 1 (Dro	1	153	16.4	9.92	6	1	1	High	1	0.993	0.995	0.616	0.736	0.668	0.615	0.726	0.99	0.787	0.533	0.605	0.896	0.528	0.592
TRINITY_DN2326_c0_g1_i1_orf1	transmembrane protein 256 homolog isoform X1 [Ostrinia furnacalis]	1	204	22.5	8.87	7	1	1	High	1	0.923	0.949	1.497	1.253	1.186	1.084	1.226	1.169	1.094	0.873	0.903	1.392	1.451	1.305
TRINITY_DN8155_c0_g1_i1_orf1	peroxisomal targeting signal 1 receptor-like [Ostrinia furnacalis]	1	535	59.6	4.97	3	1	1	High	1	1.134	1.115	1.164	1.508	1.294	1.096	1.059	0.881	1.076	1.016	1.287	1.054	0.894	0.869
TRINITY_DN23616_c0_g1_i4_orf1	ribosome biogenesis protein NSA2 homolog [Ostrinia furnacalis]	1	259	30	10.3	4	1	1	High	1	1.055	1.064	0.735	0.672	0.688	0.729	0.605	0.783	0.955	0.922	0.63	0.869	0.836	0.884
TRINITY_DN3520_c0_g1_i4_orf1	protein KRTCAP2 homolog [Ostrinia furnacalis]	1	131	14.4	9.94	10	1	1	High	1	0.915	0.86	0.871	0.813	0.834	0.868	0.748	0.828	0.799	0.724	0.588	0.848	0.868	0.869
TRINITY_DN57_c0_g2_i3_orf1	transcriptional repressor CTCF-like [Ostrinia furnacalis]	1	677	77.8	6.3	1	1	1	High	1	1.003	1.039	0.968	0.872	1.087	0.814	0.849	0.915	0.817	0.681	0.727	0.955	0.88	0.967
TRINITY_DN4900_c0_g1_i6_orf1	hypothetical protein evm_001963 [Chilo suppressalis]	1	757	82.1	5.45	2	1	1	High	1	1.102	1.128	1.557	1.774	1.433	1.483	1.341	1.752	1.696	1.672	1.632	1.453	1.486	1.795
TRINITY_DN88876_c0_g1_i1_orf1	Photosystem I reaction center subunit III, chloroplastic, partial [Trichinella zimb	1	220	24.1	9.63	5	1	1	High	1	0.853	0.827	0.951	0.923	0.979	1.572	1.224	1.426	1.258	1.493	1.432	1.813	1.54	1.933
TRINITY_DN6381_c0_g1_i2_orf1	solute carrier family 12 member 8 [Ostrinia furnacalis]	1	661	72.9	8.22	1	2	1	High	1	1.018	1.164	0.944	0.906	0.939	0.808	0.657	0.795	0.719	0.824	0.611	0.788	0.963	0.737
TRINITY_DN35582_c0_g1_i1_orf1	uncharacterized protein LOC114364680 [Ostrinia furnacalis]	1	232	27.1	6.07	5	1	1	High	1	0.859	0.932	0.997	0.988	0.847	0.779	0.756	0.916	0.791	0.794	0.871	0.658	0.658	0.585
TRINITY_DN865_c0_g1_i4_orf1	PREDICTED: uncharacterized protein LOC106130957 [Amyelois transitella]	1	874	98.7	6.28	2	1	1	High	1	1.224	1.017	0.835	0.795	0.998	1.072	0.782	0.787	0.961	0.938	0.826	1.042	1.102	0.94
TRINITY_DN71549_c0_g1_i1_orf1	alpha-N-acetylglucosaminidase [Ostrinia furnacalis]	1	88	10.6	9.8	11	1	1	High	1	1.035	1.089	1.14	1.097	1.211	1.137	0.933	1.163	1.222	1.188	1.192	1.015	1.192	1.203
TRINITY_DN51938_c0_g3_i1_orf1	unnamed protein product [Mus musculus]	1	353	40.6	6.29	4	1	1	High	1	0.977	1.023	0.646	0.729	0.643	1.36	1.499	1.266	0.778	0.687	1.006	1.063	0.845	0.954
TRINITY_DN75086_c0_g1_i5_orf1	lysosome membrane protein 2-like [Ostrinia furnacalis]	1	72	7.7	5.91	11	1	1	High	1	0.959	0.944	4.113	3.581	4.359	4.446	4.398	4.089	3.71	3.508	2.937	4.81	5.043	4.441
TRINITY_DN22156_c0_g1_i1_orf1	trafficking protein particle complex subunit 8 [Ostrinia furnacalis]	1	386	43.2	8.21	3	1	1	High	1	0.899	1.086	0.767	0.612	0.597	0.711	0.869	0.753	0.688	0.62	0.633	0.914	0.842	0.934
TRINITY_DN723_c0_g1_i2_orf1	uncharacterized protein LOC114358970, partial [Ostrinia furnacalis]	1	673	76	4.65	2	1	1	High	1	0.934	0.854	0.703	0.762	0.897	0.895	0.909	0.905	0.755	0.696	0.858	0.862	0.791	0.714
TRINITY_DN47591_c0_g1_i2_orf1	39S ribosomal protein L30, mitochondrial [Ostrinia furnacalis]	1	178	20.7	9.6	12	1	1	High	1	0.993	1.024	0.863	0.656	0.762	0.891	1.12	0.804	0.786	1.224	0.575	0.611	0.599	0.631
TRINITY_DN237_c1_g1_i1_orf1	PREDICTED: cytoplasmic protein NCK1 isoform X1 [Microplitis demolitor]	2	392	44.1	7.84	3	2	2	High	1	1.035	1.027	0.332	0.458	0.499	0.374	0.367	0.386	0.458	0.561	0.316	0.626	0.708	0.775
TRINITY_DN10360_c0_g1_i16_orf1	H(+)/Cl(-) exchange transporter 3 isoform X1 [Ostrinia furnacalis]	1	837	91.8	7.44	1	1	1	High	1	1.154	1.269	1.215	1.679	1.617	1.518	1.672	1.746	1.408	1.647	1.399	1.111	1.258	1.005
TRINITY_DN11457_c0_g1_i5_orf1	uncharacterized protein LOC114352268 [Ostrinia furnacalis]	1	123	14.3	8.84	13	1	1	High	1	1.226	1.091	0.504	0.652	0.563	0.565	0.566	0.422	0.476	0.445	0.561	0.623	0.661	0.57
TRINITY_DN24873_c0_g1_i4_orf1	uncharacterized protein LOC114365742 [Ostrinia furnacalis]	2	514	59.3	8.82	3	2	2	High	1	1.029	1.042	0.432	0.365	0.439	0.433	0.397	0.415	0.335	0.311	0.342	0.335	0.342	0.317
TRINITY_DN11492_c0_g1_i8_orf1	unnamed protein product [Chilo suppressalis]	1	146	16.4	9.41	5	3	1	High	1	1.032	0.885	1.465	1.244	1.655	1.54	1.47	1.646	1.66	1.372	1.223	1.478	1.639	1.273
TRINITY_DN42273_c0_g1_i1_orf1	uncharacterized protein LOC114349567 [Ostrinia furnacalis]	1	554	62.4	6.8	2	1	1	High	1	0.746	0.902	0.966	1.023	0.916	0.894	0.945	0.972	1.316	1.164	1.03	1.095	1.154	1.011
TRINITY_DN3039_c0_g2_i1_orf1	uncharacterized protein LOC114353136 [Ostrinia furnacalis]	1	249	26.3	5.3	6	1	1	High	1	0.957	1.249	1.779	2.041	1.673	2.009	2.134	1.719	2.036	1.768	2.44	1.933	1.815	1.812
TRINITY_DN30509_c0_g1_i9_orf1	prostatic acid phosphatase [Ostrinia furnacalis]	1	417	47.8	8.63	3	1	1	High	1	1.044	1.006	1.665	1.28	1.629	1.051	0.934	0.975	1.142	0.901	0.891	0.813	1.075	0.936
TRINITY_DN131642_c0_g1_i2_orf1	uncharacterized protein LOC114361588 isoform X14 [Ostrinia furnacalis]	1	76	8.4	4.3	30	1	1	High	1	0.962	0.968	1.259	0.989	1.301	1.048	1.533	1.426	1.072	0.995	1.314	1.302	0.977	0.896
TRINITY_DN8024_c0_g1_i6_orf1	zinc finger CCHC domain-containing protein 24-like isoform X2 [Pararge aege	1	94	10.9	9.67	11	1	1	High	1	0.881	0.926	0.986	0.857	0.949	0.851	0.882	0.787	0.78	0.723	0.763	0.834	0.663	0.773
TRINITY_DN2505_c0_g1_i1_orf1	uncharacterized protein LOC114349853 [Ostrinia furnacalis]	1	252	29.1	5.3	3	1	1	High	1	1.013	0.961	0.54	0.526	0.586	0.545	0.442	0.542	0.571	0.457	0.61	0.456	0.488	0.474
TRINITY_DN21623_c0_g2_i1_orf1	Chlorophyll a-b binding protein, chloroplastic [Trichinella nelsoni]	1	246	26.5	8.32	3	1	1	High	1	1.25	1.321	0.832	1.06	0.722	1.115	1.018	1.063	0.909	0.852	1.024	1.223	1.29	1.337

TRINITY_DN4443_c0_g1_i4_orf1	lysosome-associated membrane glycoprotein 1-like isoform X4 [Ostrinia furna	1	232	24.7	5.12	3	2	1	High	1	0.974	1.02	2.481	2.545	2.455	1.914	1.828	1.856	2.049	1.789	2.19	2.194	2.151	2.222
TRINITY_DN79804_c0_g1_i1_orf1	zinc finger protein on ecdysone puffs-like [Ostrinia furnacalis]	1	77	8.9	4.91	21	1	1	High	1	1.153	0.886	0.407	0.349	0.466	0.433	0.326	0.393	0.41	0.305	0.279	0.526	0.559	0.413
TRINITY_DN96_c0_g1_i1_orf1	collagenase-like [Ostrinia furnacalis]	1	404	44.4	4.73	3	1	1	High	1	0.925	1.089	0.132	0.164	0.192	0.168	0.188	0.253	0.2	0.202	0.22	0.172	0.144	0.161
TRINITY_DN13067_c0_g1_i6_orf1	diphosphomevalonate decarboxylase [Ostrinia furnacalis]	1	391	42.6	6.79	2	2	1	High	1	1.026	1.169	0.906	0.903	0.982	1.135	1.189	1.169	1.179	1.19	1.013	1.612	1.703	1.725
TRINITY_DN70382_c0_g1_i10_orf1	TGF-beta receptor type-1 isoform X4 [Ostrinia furnacalis]	1	516	58.3	8.15	3	1	1	High	1	0.978	1.207	0.769	0.978	0.732	0.565	0.923	0.665	0.558	0.359	0.702	0.645	0.615	0.588
TRINITY_DN20558_c0_g1_i2_orf1	Transient receptor potential channel pyrexia [Operophtera brumata]	1	76	8.5	5.11	14	1	1	High	1	0.963	1.083	2.149	2.027	1.963	3.091	2.94	3.027	1.683	1.724	1.5	1.586	1.64	1.806
TRINITY_DN1309_c0_g2_i1_orf1	chymotrypsin-1-like [Ostrinia furnacalis]	1	286	31	7.24	4	1	1	High	1	1.213	1.103	0.97	0.78	0.904	1.026	1.133	0.916	0.883	1.096	0.607	0.683	0.693	0.693
TRINITY_DN42719_c0_g2_i1_orf1	inter-alpha-trypsin inhibitor heavy chain H4-like isoform X11 [Ostrinia furnaca	1	116	12.1	4.97	12	1	1	High	1	1.043	1.172	3.828	3.576	3.176	2.444	3.087	2.659	4.255	4.284	4.236	4.674	4.756	4.817
TRINITY_DN30932_c0_g1_i2_orf1	delta(24)-sterol reductase-like isoform X2 [Ostrinia furnacalis]	1	511	59.2	8.31	2	1	1	High	1	0.93	0.896	0.174	0.128	0.224	0.267	0.194	0.22	0.267	0.219	0.213	0.377	0.307	0.363
TRINITY_DN57798_c0_g1_i1_orf1	ubiquitin carboxyl-terminal hydrolase 36 [Ostrinia furnacalis]	1	713	79	9.09	2	1	1	High	1	1.346	1.412	0.324	0.242	0.45	0.459	0.372	0.365	0.403	0.326	0.341	0.563	0.664	0.686
TRINITY_DN48838_c0_g1_i6_orf1	merlin-like [Ostrinia furnacalis]	1	657	75.3	6.02	2	1	1	High	1	1.095	1.069	0.666	0.693	0.723	0.705	0.717	0.581	0.704	0.542	0.603	0.876	0.746	0.809
TRINITY_DN18650_c0_g1_i1_orf1	bombyxin B-9-like [Ostrinia furnacalis]	1	82	9.3	5.8	10	1	1	High	1	0.89	0.924	1.056	1.039	1.101	1.375	1.307	1.222	1.398	1.659	1.399	1.736	1.894	1.991
TRINITY_DN33408_c0_g1_i1_orf1	hypothetical protein HF086_017664 [Spodoptera exigua]	1	70	8.1	11.75	11	1	1	High	1	1.168	1.253	0.983	0.934	1.274	0.782	0.921	0.605	1.427	1.462	2.105	1.379	1.115	1.236
TRINITY_DN104597_c0_g1_i2_orf1	hemicentin-2-like isoform X1 [Ostrinia furnacalis]	1	522	58.3	8.5	2	1	1	High	1	0.833	0.909	0.869	0.782	0.706	0.942	0.912	0.941	1.152	1.066	0.962	0.8	0.711	0.924
TRINITY_DN14705_c0_g2_i1_orf1	coiled-coil-helix-coiled-coil-helix domain-containing protein 10, mitochondri	1	146	15.2	8.56	7	1	1	High	1	0.938	1.015	0.444	0.534	0.446	0.416	0.552	0.419	0.568	0.834	0.65	0.718	0.51	0.61
TRINITY_DN11015_c0_g1_i8_orf1	nicotinamide riboside kinase 1 [Ostrinia furnacalis]	1	208	23.9	6.64	7	2	1	High	1	1.054	1.078	0.886	0.974	0.92	1.037	1.267	1.153	1.116	1.113	0.866	0.925	0.902	0.849
TRINITY_DN48237_c0_g1_i5_orf1	myogenesis-regulating glycosidase-like [Ostrinia furnacalis]	1	673	76.4	4.93	1	1	1	High	1	1.108	0.965	0.192	0.233	0.26	0.312	0.283	0.253	0.188	0.246	0.248	0.304	0.289	0.29
TRINITY_DN44877_c0_g1_i2_orf1	U6 snRNA-associated Sm-like protein LSm7 [Diachasma alloeum]	1	135	15.5	6.58	6	1	1	High	1	0.939	0.899	0.795	0.669	0.743	0.693	0.657	0.679	0.68	0.579	0.655	0.904	0.865	0.811
TRINITY_DN36061_c0_g4_i2_orf1	putative GPI-anchored protein pfl2 [Ostrinia furnacalis]	1	836	91.7	4.91	1	1	1	High	1	0.929	1.032	0.202	0.283	0.211	0.243	0.26	0.235	0.288	0.178	0.306	1.396	1.496	1.504
TRINITY_DN80424_c0_g1_i1_orf1	PREDICTED: cytoplasmic FMR1-interacting protein [Dufourea novaeangliae]	1	600	69.2	7.77	2	1	1	High	1	1.025	0.95	2.076	2.114	2.083	1.171	0.743	0.923	0.747	1.041	0.779	1.411	1.126	1.743
TRINITY_DN27491_c0_g1_i1_orf1	prolactin regulatory element-binding protein [Galleria mellonella]	1	423	47	8.94	4	1	1	High	1	0.904	0.882	1.124	1.255	1.165	1.131	1.054	0.985	0.925	0.862	1.015	0.841	0.798	0.727
TRINITY_DN78873_c0_g1_i4_orf1	hypothetical protein evm_008224 [Chilo suppressalis]	1	134	14.6	8.85	6	1	1	High	1	1.073	0.99	0.553	0.772	0.644	0.487	0.531	0.604	0.302	0.468	0.492	0.577	0.574	0.553
TRINITY_DN33995_c0_g1_i5_orf1	unnamed protein product [Spodoptera exigua]	1	340	37.8	9.06	4	1	1	High	1	1.111	0.811	2.401	2.468	2.539	1.14	1.692	1.69	0.939	0.842	0.755	0.692	0.781	0.734
TRINITY_DN21380_c0_g1_i1_orf1	ankyrin repeat domain-containing protein 13C [Ostrinia furnacalis]	1	443	50.5	6.3	3	1	1	High	1	0.923	0.879	0.842	0.818	0.887	0.723	0.853	0.728	1.02	1.041	0.971	1.331	0.926	1.129
TRINITY_DN14944_c0_g1_i7_orf1	alpha-tocopherol transfer protein-like isoform X1 [Ostrinia furnacalis]	1	251	29.3	6.33	7	1	1	High	1	1.024	0.97	0.956	1.031	1.037	0.788	0.79	0.846	0.818	0.85	0.832	1.094	1.085	1.009
TRINITY_DN556_c0_g1_i4_orf1	Brasiliensin [Operophtera brumata]	1	383	41.5	5.22	7	1	1	High	1	0.83	0.926	0.494	0.489	0.519	0.591	0.933	0.767	0.631	1.144	0.667	0.613	0.493	0.455
TRINITY_DN4550_c1_g1_i5_orfp2	TRINITY_DN4550_c1_g1_i5_m.14710 TRINITY_DN4550_c1_g1::TRINITY_DN455C	1	167	20.9	9.06	7	1	1	High	1	0.935	0.958	0.426	0.462	0.446	0.418	0.354	0.404	0.356	0.283	0.389	0.314	0.367	0.341
TRINITY_DN5275_c0_g1_i1_orf1	paraplegin [Ostrinia furnacalis]	1	789	87.8	8.91	1	1	1	High	1	1.005	0.907	0.646	0.594	0.56	0.545	0.527	0.505	0.472	0.439	0.488	0.723	0.703	0.737
TRINITY_DN86699_c0_g4_i1_orf1	protein unc-45 homolog B [Diachasma alloeum]	1	147	15.9	8.85	8	1	1	High	1	0.913	0.951	1.023	0.926	0.852	0.936	1.038	0.923	1.083	1.087	1.179	1.065	1.056	0.965
TRINITY_DN2908_c0_g1_i1_orf1	uncharacterized protein LOC114361337 [Ostrinia furnacalis]	1	554	61.6	7.23	2	1	1	High	1	0.806	0.728	0.837	0.672	0.703	0.719	0.813	0.64	0.58	0.705	0.466	6.058	5.658	5.216
TRINITY_DN809_c0_g1_i9_orf1	receptor-type tyrosine-protein phosphatase N2 isoform X6 [Ostrinia furnacalis	1	1009	114.3	5.29	1	1	1	High	1	0.653	0.958	1.137	1.421	1.078	0.992	1.216	1.382	1.045	0.835	1.387	1.371	1.237	0.992
TRINITY_DN111_c0_g2_i2_orf1	hypothetical protein O3G_MSEX007696 [Manduca sexta]	2	450	48.3	8.12	3	2	2	High	1	1.1	1.13	0.744	0.829	0.886	0.859	1.796	0.866	0.611	0.664	0.7	1.122	1.05	0.892
TRINITY_DN77318_c0_g2_i1_orf1	uncharacterized protein LOC114351191 [Ostrinia furnacalis]	1	185	20.5	8.29	4	1	1	High	1	0.953	0.903	1.631	1.704	1.668	1.436	1.601	1.426	0.999	0.841	1.098	0.95	1.174	1.13
TRINITY_DN5998_c0_g2_i1_orf1	protein archease-like [Ostrinia furnacalis]	1	159	18.4	4.44	8	1	1	High	1	0.958	0.935	1.335	1.294	1.226	1.148	1.045	1.176	1.012	1.16	1.163	1.038	0.983	0.908
TRINITY_DN5125_c0_g1_i6_orf1	protein furry-like [Ostrinia furnacalis]	1	1695	188.7	6.64	0	1	1	High	1	0.883	0.866	0.943	0.738	0.89	0.692	0.854	0.803	0.887	0.806	0.785	0.956	0.955	0.877
TRINITY_DN2544_c1_g1_i2_orf1	hypothetical protein evm_004833 [Chilo suppressalis]	1	251	28.2	5.29	4	1	1	High	1	0.945	1.019	1.223	1.009	1.019	1.105	1.163	1.196	1.063	1.023	1.049	0.777	0.702	0.987
TRINITY_DN106479_c1_g1_i1_orf1	secretory phospholipase A2 receptor-like [Ostrinia furnacalis]	1	66	7.8	5.08	20	1	1	High	1	0.807	0.833	1.79	1.859	1.816	1.438	1.417	1.428	1.668	2.169	1.408	1.811	1.446	1.8
TRINITY_DN51239_c0_g1_i5_orf1	regulatory-associated protein of mTOR [Ostrinia furnacalis]	1	751	83.9	6.96	1	1	1	High	1	0.843	0.905	0.615	0.624	0.626	0.695	0.686	0.787	0.607	0.585	0.529	0.79	0.562	0.541
TRINITY_DN10234_c0_g1_i1_orf1	39S ribosomal protein L16, mitochondrial [Ostrinia furnacalis]	1	242	28.1	9.88	3	1	1	High	1	0.965	1.006	0.767	0.769	0.712	0.908	0.888	0.828	0.879	0.941	0.953	0.911	0.915	0.931
TRINITY_DN8173_c0_g1_i3_orf1	dihydroceramide fatty acyl 2-hydroxylase FAH1 [Ostrinia furnacalis]	1	338	39.3	8.46	4	1	1	High	1	0.985	1.238	0.336	0.265	0.356	0.393	0.423	0.564	0.31	0.414	0.382	0.405	0.345	0.31
TRINITY_DN2141_c0_g1_i1_orf1	low density lipoprotein receptor adapter protein 1-like [Ostrinia furnacalis]	1	215	23.7	7.5	6	1	1	High	1	1.056	1.195	1.964	2.242	1.792	1.377	1.266	1.44	1.586	1.602	1.367	1.952	1.915	1.702
TRINITY_DN8610_c0_g1_i4_orf1	PREDICTED: arrestin domain-containing protein 4 [Amyelois transitella]	1	329	36.4	8.51	4	1	1	High	1	0.841	1.029	0.859	1.011	0.783	0.8	0.883	0.952	0.865	0.938	0.772	0.757	0.751	0.919
TRINITY_DN5437_c0_g1_i1_orf1	unnamed protein product [Chilo suppressalis]	1	1216	133.2	8.06	1	1	1	High	1	0.975	1.007	1.002	1.255	1.182	1.007	1.053	1.094	0.918	0.803	1.013	0.772	0.856	0.788
TRINITY_DN43369_c0_g2_i1_orf1	cytochrome P450 monooxygenase 304 [Glyphodes pyloalis]	1	114	13	6.68	8	1	1	High	1	0.917	0.939	0.338	0.302	0.359	0.692	0.586	0.835	0.455	0.481	0.365	0.65	0.695	0.601
TRINITY_DN4151_c1_g1_i4_orf1	5-methylcytosine rRNA methyltransferase NSUN4 isoform X1 [Ostrinia furnaca	1	512	58.3	6.9	3	1	1	High	1	1.037	0.87	0.754	0.682	0.616	0.742	0.697	0.772	0.707	0.513	0.749	0.807	0.811	0.77
TRINITY_DN41129_c0_g1_i1_orf1	histone deacetylase 6 [Ostrinia furnacalis]	1	734	82.1	7.25	1	1	1	High	1	0.998	0.924	0.892	0.784	0.923	0.853	1.061	0.932	1.277	1.096	1.098	0.898	1.058	1.025
TRINITY_DN33038_c0_g1_i1_orf1	39S ribosomal protein L46, mitochondrial [Ostrinia furnacalis]	1	260	29.9	7.01	5	1	1	High	1	1.016	1.09	0.431	0.358	0.49	0.514	0.522	0.548	0.522	0.75	0.502	0.429	0.504	0.495
TRINITY_DN1955_c0_g1_i5_orf1	ultraviolet-B receptor UVR8-like [Ostrinia furnacalis]	1	384	42.4	6.9	3	1	1	High	1	1.125	1.063	0.951	0.755	0.987	1.062	1.062	1.194	1.379	1.261	0.844	0.888	0.886	0.846
TRINITY_DN15380_c0_g1_i1_orf1	39S ribosomal protein L32, mitochondrial [Ostrinia furnacalis]	1	194	22.6	9.51	5	1	1	High	1	0.916	1.051	0.26	0.265	0.269	0.255	0.251	0.226	0.266	0.242	0.231	0.266	0.321	0.269
TRINITY_DN139537_c0_g1_i1_orf1	uncharacterized protein LOC114351440 [Ostrinia furnacalis]	1	202	23.3	9.32	5	1	1	High	1	0.424	0.912	1.29	1.331	0.898	1.216	1.701	1.001	1.873	2	1.142	2.458	3.257	3.229
TRINITY_DN10229_c0_g1_i6_orf1	autophagy protein 5 isoform X2 [Ostrinia furnacalis]	1	264	31.2	6.39	4	1	1	High	1	0.974	0.923	1.322	1.382	1.456	1.289	1.374	1.207	1.224	1.202	1.371	1.547	1.513	1.461
TRINITY_DN3814_c1_g1_i1_orf1	39S ribosomal protein L11, mitochondrial [Ostrinia furnacalis]	1	196	22	9.5	5	1	1	High	1	1.373	1.035	0.514	0.441	0.485	0.528	0.536	0.405	0.417	0.392	0.424	0.438	0.458	0.482
TRINITY_DN18159_c0_g1_i6_orf1	zinc carboxypeptidase-like [Ostrinia furnacalis]	1	413	46.8	5.73	3	1	1	High	1	0.905	0.925	0.681	0.864	0.831	0.592	0.698	0.8	0.644	0.493	0.576	0.66	0.506	0.643
TRINITY_DN976_c0_g1_i5_orf1	filaggrin-2-like [Ostrinia furnacalis]	1	715	74.5	9.38	2	1	1	High	1	0.985	0.922	1.377	1.309	1.175	1.444	1.488	1.415	1.326	1.469	0.914	1.293	1.231	1.295
TRINITY_DN5298_c0_g1_i3_orf1	uncharacterized protein LOC114352519 [Ostrinia furnacalis]	1	395	46	5.17	3	1	1	High	1	1.21	1.104	0.772	0.605	0.854	0.772	0.827	0.902	1.139	1.035	0.869	0.9	0.805	0.946
TRINITY_DN19080_c0_g1_i4_orf1	synaptic vesicle 2-related protein-like isoform X1 [Ostrinia furnacalis]	1	511	55.8	7.08	3	2	1	High	1	1.052	1.052	0.836	0.872	0.817	0.824	0.905	0.881	0.891	0.763	0.895	0.686	0.579	0.595
TRINITY_DN5553_c0_g1_i4_orf1	uncharacterized protein LOC114353828 [Ostrinia furnacalis]	1	149	16.5	6.7	6	1	1	High	1	0.943	0.995	1.197	1.4	1.147	1.317	1.473	1.29	1.569	1.736	2.024	2.708	2.464	2.645
TRINITY_DN18404_c0_g1_i5_orf1	periodic tryptophan protein 1 homolog isoform X1 [Ostrinia furnacalis]	1	464	51.1	4.63	2	1	1	High	1	1.008	0.993	0.25	0.278	0.312	0.283	0.318	0.33	0.323	0.305	0.346	0.36	0.361	0.368
TRINITY_DN14429_c0_g1_i2_orf1	NADH dehydrogenase [ubiquinone] 1 beta subcomplex subunit 11, mitochonc	1	143	16.4	6.52	8	1	1	High	1	1.114	1.049	0.268	0.313	0.401	0.454	0.382	0.464	0.487	0.447	0.381	0.7	0.661	0.642
TRINITY_DN50820_c0_g1_i2_orf1	transmembrane protein 135-like isoform X2 [Ostrinia furnacalis]	1	398	45.2	9.63	2	1	1	High	1	1.027	0.964	0.527	0.641	0.584	0.468	0.683	0.525	0.481	0.466	0.838	0.973	0.661	0.753
TRINITY_DN2312_c0_g1_i4_orf1	endoplasmic reticulum-Golgi intermediate compartment protein 3 [Ostrinia fu	1	386	43	6.71	4	1	1	High	1	1.076	0.954	1.565	1.604	1.388	1.494	1.361	1.362	1.518	1.185	1.463	0.687	0.722	0.72
TRINITY_DN29288_c0_g1_i1_orf1	uncharacterized protein LOC114355446 [Ostrinia furnacalis]	1	274	29.9	9.57	6	1	1	High	1	0.991	1.179	1.36	1.583	1.261	1.105	0.924	1.208	1.021	1.042	1.267	1.182	1.082	1.15
TRINITY_DN1575_c0_g1_i7_orf1	adenine phosphoribosyltransferase [Ostrinia furnacalis]	1	183	20	7.77	7	1	1	High	1	1.008	1.016	1.441	1.352	1.432	1.344	1.337	1.282	1.084	1.143	1.263	1.174	1.081	1.087
TRINITY_DN86772_c0_g1_i3_orfp1	x-tox [Spodoptera exigua]	1	77	8.5	5.33	9	1	1	High	1	0.884	0.758	6.342	6.479	6.373	5.964	5.684	5.386	4.197	3.183	4.369	2.561	2.63	2.441
TRINITY_DN47260_c0_g1_i2_orf1	SET domain-containing protein SmydA-8-like [Ostrinia furnacalis]	1	546	61.9	7.44	3	1	1	High	1	0.72	0.845	1.194	1.317	1.078	1.339	1.273	1.872	1.257	1.657	1.653	1.471	1.297	1.824
TRINITY_DN2098_c0_g1_i1_orf1	dolichyl pyrophosphate Man9GlcNAc2 alpha-1,3-glucosyltransferase [Ostrinia	1	531	61.7	8.6	2	1	1	High	1	1.111	1.139	1.229	0.942	1.233	1.449	1.096	1.45	1.172	1.42	1.133	0.871	0.785	0.93
TRINITY_DN11069_c0_g1_i6_orf1	ganglioside-induced differentiation-associated protein 1 [Ostrinia furnacalis]	1	294	33.6	6.55	4	1	1	High	1	1.089	1.166	0.567	0.605	0.675	0.8	0.808	0.732	0.688	0.734	0.682	0.607	0.68	0.681
TRINITY_DN83621_c0_g1_i1_orf1	ataxin-2-like protein [Ostrinia furnacalis]	1	449	49.3	6.9	4	1	1	High	1	1.229	1.017	0.84	0.926	0.865	0.994	1.008	0.9	0.96	0.781	1.186	0.922	1.069	0.871
TRINITY_DN11076_c0_g2_i1_orf1	5'-AMP-activated protein kinase subunit beta-1 isoform X1 [Ostrinia furnacalis	1	281	31.5	5.67	5	1	1	High	1	0.918	0.853	1.115	0.893	0.985	1.342	1.158	1.169	1.098	0.902	1.218	1.035	0.966	1.201
TRINITY_DN11876_c0_g1_i2_orf1	protein TAPT1 homolog [Ostrinia furnacalis]	1	599	68.7	7.21	1	1	1	High	1	1.068	1.202	0.63	0.814	0.805	0.648	0.734	0.784	0.856	0.686	0.707	1.056	0.863	0.965
TRINITY_DN20238_c0_g1_i7_orf1	zinc finger FYVE domain-containing protein 26 [Ostrinia furnacalis]	1	793	88.9	8.27	2	1	1	High	1	1.554	1.813	1.494	1.471	1.294	1.046	0.764	0.855	1.025	0.741	0.999	1.072	1.345	1.017
TRINITY_DN99694_c0_g1_i1_orf1	hypothetical protein HF086_013792 [Spodoptera exigua]	1	77	8.8	5.19	10	1	1	High	1	0.895	1.045	1.296	1.264	1.527	1.38	1.303	1.458	1.19	2.046	1.243	1.052	1.112	1.171
TRINITY_DN40704_c0_g1_i2_orf1	COX assembly mitochondrial protein homolog [Ostrinia furnacalis]	1	117	13.6	8.65	9	1	1	High	1	0.961	0.949	0.362	0.416	0.429	0.408	0.433	0.361	0.351	0.357	0.325	0.577	0.532	0.515
TRINITY_DN4256_c0_g1_i1_orf1	chitinase-3-like protein 1 [Ostrinia furnacalis]	1	476	53.8	8.56	2	1	1	High	1	0.938	0.943	1.605	1.343	1.305	1.553	1.478	1.482	1.74	1.993	1.655	2.392	2.051	2.007
TRINITY_DN54336_c0_g1_i1_orf1	basement membrane-specific heparan sulfate proteoglycan core protein isofo	1	67	7.1	4.42	30	1	1	High	1	1.036	0.929	0.828	0.678	0.824	0.776	0.653	0.789	0.849	0.905	0.72	0.658	0.761	0.681
TRINITY_DN3469_c0_g1_i4_orf1	LOW QUALITY PROTEIN: cadherin-87A-like [Ostrinia furnacalis]	1	1831	199.5	4.68	1	1	1	High	1	0.872	0.798	0.954	1.159	0.873	0.722	0.778	0.717	0.697	1.117	0.682	0.906	0.87	0.912
TRINITY_DN171_c0_g1_i1_orf1	alkaline phosphatase, tissue-nonspecific isozyme-like isoform X1 [Ostrinia furn	1	558	61.1	6.33	2	1	1	High	1	0.953	0.961	1.051	0.916	1.142	1.23	1.64	1.472	1.125	1.721	1.057	0.825	0.822	0.891
TRINITY_DN29521_c0_g1_i1_orf1	density-regulated protein homolog [Ostrinia furnacalis]	2	177	20.1	5.3	8	2	2	High	1	0.926	0.968	0.798	0.778	0.836	0.731	0.699	0.659	0.509	0.501	0.627	0.597	0.638	0.58
TRINITY_DN8621_c0_g1_i5_orf1	aminopeptidase N-like isoform X2 [Ostrinia furnacalis]	1	596	69.5	8.47	2	1	1	High	1	0.926	0.977	0.439	0.409	0.456	0.424	0.445	0.395	0.421	0.493	0.46	0.282	0.358	0.272
TRINITY_DN106534_c0_g1_i1_orf1	nucleolar complex protein 2 homolog [Ostrinia furnacalis]	1	683	78.2	8.35	2	1	1	High	1	1.119	0.947	0.784	0.72	0.722	0.606	0.751	0.659	0.482	0.417	0.582	0.41	0.382	0.322
TRINITY_DN15222_c0_g1_i4_orf1	lysosomal alpha-mannosidase-like [Ostrinia furnacalis]	1	103	11.9	4.67	17	1	1	High	1	0.889	0.978	0.855	0.944	0.91	1.526	1.433	1.448	0.977	0.615	0.666	5.079	5.425	5.184
TRINITY_DN30169_c0_g1_i1_orfp1	TRINITY_DN30169_c0_g1_i1_m.11367 TRINITY_DN30169_c0_g1::TRINITY_DN3C	1	51	6.1	9.42	12	2	1	High	1	1.034	1.09	2.378	1.819	2.51	3.045	3.443	3.62	4.8	4.572	2.733	3.582	3.871	4.051
TRINITY_DN14336_c0_g3_i2_orf1	calbindin-32 isoform X1 [Ostrinia furnacalis]	1	316	36.7	4.65	5	1	1	High	1	0.977	1.068	0.556	0.517	0.506	0.547	0.464	0.458	0.546	0.48	0.609	0.47	0.497	0.48
TRINITY_DN32161_c0_g1_i1_orf1	uncharacterized protein LOC114352518 [Ostrinia furnacalis]	1	130	15.2	5.58	6	1	1	High	1	0.937	0.961	0.442	0.468	0.483	0.621	0.627	0.636	0.62	0.641	0.768	0.772	0.676	0.72
TRINITY_DN144286_c0_g1_i1_orf1	protein C12orf4 homolog [Aphidius gifuensis]	1	251	27.9	6.92	3	1	1	High	1	1.065	0.959	0.958	1.014	0.954	1.012	0.915	1.022	0.922	0.909	0.803	1.022	0.923	0.919
TRINITY_DN44857_c0_g1_i4_orf1	UPF0183 protein CG7083 [Ostrinia furnacalis]	1	429	48.4	8.4	2	1	1	High	1	1.16	1.366	1.714	1.443	1.555	1.659	2.001	1.486	1.425	1.54	1.449	1.661	1.624	1.568
TRINITY_DN5772_c0_g1_i6_orf1	uncharacterized protein LOC114364899 isoform X2 [Ostrinia furnacalis]	1	181	19.4	7.09	8	1	1	High	1	1.113	1.064	0.988	0.906	0.872	0.977	0.917	1.033	1.122	0.732	1.007	1.506	1.416	1.404
TRINITY_DN2541_c0_g1_i11_orf1	alanine--tRNA ligase, mitochondrial [Ostrinia furnacalis]	1	1003	112	7.34	1	1	1	High	1	1.324	0.979	0.707	0.754	1.128	0.777	0.894	0.961	0.791	0.863	0.859	0.681	0.909	0.813
TRINITY_DN1104_c0_g1_i1_orfp1	TRINITY_DN1104_c0_g1_i1_m.5521 TRINITY_DN1104_c0_g1::TRINITY_DN1104_	1	203	21.2	9.91	6	1	1	High	1	0.769	0.839	1.045	1.277	1.327	1.129	1.047	0.989	0.733	0.705	0.854	0.527	0.866	0.413
TRINITY_DN10287_c0_g1_i1_orf1	nibrin [Ostrinia furnacalis]	1	638	71.6	7.34	3	1	1	High	1	0.968	1.027	0.754	0.67	0.858	0.885	0.711	0.582	0.732	0.706	0.693	0.621	0.634	0.797
TRINITY_DN6638_c0_g1_i1_orf1	ubiquinone biosynthesis protein COQ4 homolog, mitochondrial [Ostrinia furna	1	247	28.6	9.1	4	1	1	High	1	1.142	1.025	0.934	0.966	1.048	0.705	0.574	0.73	0.733	0.968	0.842	0.729	0.812	0.793
TRINITY_DN10796_c0_g1_i2_orf1	SH3 domain-binding protein 5 homolog [Ostrinia furnacalis]	1	476	54.2	5.27	2	1	1	High	1	1.059	1.437	1.083	1.066	0.935	1.074	0.997	1.292	1.039	1.189	1.052	1.033	0.935	0.94
TRINITY_DN30224_c0_g1_i1_orf1	PREDICTED: serine--tRNA ligase, mitochondrial [Amyelois transitella]	1	464	53.2	9.23	2	1	1	High	1	1.088	1.022	0.693	0.739	0.682	0.784	0.729	0.807	0.575	0.584	0.74	0.692	0.643	0.595
TRINITY_DN24723_c2_g1_i1_orf1	hypothetical protein evm_001103 [Chilo suppressalis]	1	103	11.6	9.32	9	1	1	High	1	0.905	1.288	4.264	4.251	3.609	2.766	3.769	4.344	4.555	3.785	4.438	2.208	2.209	1.974

TRINITY_DN8726_c0_g2_i3_orf1	dnaJ homolog subfamily C member 25 homolog [Ostrinia furnacalis]	1	336	40.8	8.81	3	1	1	High	1	0.886	0.892	1.192	1.208	1.356	1.119	1.108	1.277	1.181	1.093	1.203	1.207	1.429	1.348
TRINITY_DN5121_c0_g1_i1_orf1	unnamed protein product [Parnassius apollo]	1	87	9.4	5.06	9	1	1	High	1	0.87	0.841	0.701	0.625	0.766	0.704	0.793	0.736	0.664	0.927	0.695	0.776	0.824	0.783
TRINITY_DN937_c0_g1_i2_orf1	protein brunelleschi [Ostrinia furnacalis]	1	1074	118.9	6.67	1	2	1	High	1	0.858	1.033	0.859	0.751	0.869	0.676	0.712	0.707	0.705	0.608	0.673	0.715	0.678	0.641
TRINITY_DN21126_c0_g1_i1_orf1	serine/threonine-protein kinase unc-51 isoform X5 [Ostrinia furnacalis]	1	356	38.8	9.52	3	1	1	High	1	1.014	1.071	0.701	0.94	0.692	0.685	0.813	0.846	0.632	0.719	0.845	0.885	0.957	0.838
TRINITY_DN12133_c0_g2_i1_orf1	O-acyltransferase like protein-like [Ostrinia furnacalis]	1	740	84.1	7.17	1	1	1	High	1	1.005	0.926	0.361	0.332	0.459	0.336	0.412	0.44	0.372	0.518	0.425	0.435	0.433	0.432
TRINITY_DN34509_c0_g1_i1_orf1	transcription initiation factor IIA subunit 2 [Aphidius gifuensis]	1	118	13.4	9.55	6	1	1	High	1	0.978	1	0.547	0.499	0.626	0.502	0.551	0.475	0.488	0.639	0.474	0.68	0.794	0.654
TRINITY_DN64403_c0_g2_i1_orf1	carboxylesterase [Ostrinia furnacalis]	1	95	10.6	5.1	14	1	1	High	1	0.973	0.992	0.173	0.158	0.21	0.268	0.344	0.28	0.167	0.206	0.196	0.184	0.115	0.183
TRINITY_DN5554_c0_g1_i2_orf1	double-stranded RNA-binding protein Staufen homolog 2 isoform X5 [Pectinc	1	549	56.8	9.91	2	2	1	High	1	0.97	1.015	0.4	0.402	0.365	0.432	0.442	0.433	0.417	0.433	0.444	0.547	0.535	0.498
TRINITY_DN8986_c0_g1_i1_orf1	HBS1-like protein [Ostrinia furnacalis]	1	678	74.8	5.78	1	1	1	High	1	0.948	0.993	1.16	1.544	1.3	1.135	1.212	1.243	1.016	1.259	1.47	1.273	1.232	1.329
TRINITY_DN61536_c0_g2_i1_orf1	cubilin homolog [Ostrinia furnacalis]	1	88	9.6	4.59	14	1	1	High	1	1.143	1.108	1.193	1.134	0.936	0.968	1.227	1.125	1.001	1.046	0.887	0.935	0.943	1.109
TRINITY_DN1239_c0_g1_i3_orf1	uncharacterized protein LOC114355269 [Ostrinia furnacalis]	1	575	63.4	8.69	2	1	1	High	1	0.968	1.101	0.431	0.506	0.497	0.433	0.472	0.57	0.36	0.362	0.357	0.411	0.392	0.404
TRINITY_DN90497_c0_g1_i1_orf1	midasin-like [Ostrinia furnacalis]	1	328	37	7.39	4	1	1	High	1	1.06	1.303	1.253	1.183	1.288	1.37	1.336	1.408	1.557	1.103	1.174	0.829	0.81	0.604
TRINITY_DN21331_c0_g1_i6_orf1	sodium-independent sulfate anion transporter-like [Ostrinia furnacalis]	1	635	67.9	8.44	2	1	1	High	1	0.985	1.048	1.617	1.577	1.14	1.491	1.427	1.267	1.478	2	1.335	1.182	1.044	1.237
TRINITY_DN104586_c0_g1_i1_orf1	Chlorophyll a-b binding protein 37, chloroplastic, partial [Trichinella patagonie	1	95	10.4	9.47	15	1	1	High	1	0.863	0.871	0.195	0.209	0.276	0.248	0.284	0.263	0.257	0.274	0.257	0.312	0.188	0.211
TRINITY_DN13626_c0_g2_i1_orf1	charged multivesicular body protein 2b [Ostrinia furnacalis]	1	210	23.6	5.87	4	1	1	High	1	1.01	0.967	1.063	1.237	1.039	0.778	0.966	0.983	0.991	0.822	0.989	1.261	1.225	1.22
TRINITY_DN12582_c0_g1_i5_orf1	uncharacterized protein LOC114355527 isoform X1 [Ostrinia furnacalis]	1	205	22.9	5.74	5	1	1	High	1	1.025	1.154	3.046	3.649	2.651	2.684	2.167	4.082	4.757	5.199	3.393	4.101	4.309	2.624
TRINITY_DN31310_c0_g1_i1_orf1	PREDICTED: multiple epidermal growth factor-like domains protein 10 isoform	1	86	9.2	7.06	10	1	1	High	1	0.918	1.188	1.495	1.601	1.577	1.622	1.572	1.516	1.643	1.549	1.639	1.262	1.921	1.916
TRINITY_DN121650_c0_g1_i1_orf1	carboxylesterase [Ostrinia furnacalis]	1	67	7.6	4.89	19	1	1	High	1	0.952	0.99	2.47	2.206	2.192	2.619	2.6	2.625	2.531	2.342	2.312	4.906	4.919	4.798
TRINITY_DN3706_c0_g1_i6_orf1	irregular chiasm C-roughest protein-like isoform X1 [Ostrinia furnacalis]	1	670	74	7.25	1	1	1	High	1	1.093	1.042	1.091	0.96	0.928	1.139	0.938	1.07	0.876	1.158	1.066	1.186	1.133	0.944
TRINITY_DN57092_c0_g1_i1_orf1	transcription initiation factor TFIID subunit 7 [Ostrinia furnacalis]	1	375	43.8	6.21	3	1	1	High	1	0.914	1.079	1.014	1.284	1.096	1.129	1.026	0.992	1.066	1.378	1.157	1.105	1.194	1.097
TRINITY_DN13375_c0_g1_i6_orf1	thioredoxin, mitochondrial isoform X2 [Ostrinia furnacalis]	1	148	16.4	7.34	5	1	1	High	1	0.913	0.942	0.517	0.551	0.597	0.673	0.745	0.698	0.657	0.417	0.525	1.024	0.97	0.935
TRINITY_DN51829_c0_g1_i1_orf1	FAS-associated factor 2 [Ostrinia furnacalis]	2	437	49.2	5.94	4	2	2	High	1	1.069	1.02	1.017	1.045	0.912	1.091	1.022	1.079	0.905	0.781	0.918	1.169	1.041	1.178
TRINITY_DN5696_c0_g1_i4_orf1	serine protease snake-like isoform X1 [Ostrinia furnacalis]	1	393	43.2	8.38	3	1	1	High	1	0.67	0.774	2.191	1.644	2.055	1.935	2.333	2.465	1.363	0.884	1.265	1.029	1.015	1.077
TRINITY_DN2004_c0_g1_i20_orf1	hypothetical protein evm_006436 [Chilo suppressalis]	1	295	32.7	5.15	4	1	1	High	1	0.86	0.984	0.904	1.073	0.854	0.715	0.753	0.826	0.633	0.851	0.591	1.197	1.063	1.384
TRINITY_DN110460_c0_g2_i1_orf1	Similar to chaf1a-b: Chromatin assembly factor 1 subunit A-B (Xenopus laevis)	1	253	29.2	8.54	3	1	1	High	1	1.039	0.999	0.631	0.636	0.664	0.747	0.649	0.599	0.681	0.58	0.793	0.323	0.335	0.366
TRINITY_DN4013_c0_g1_i4_orf1	uncharacterized protein LOC114353190 isoform X1 [Ostrinia furnacalis]	1	102	10.9	8.84	10	1	1	High	1	0.948	0.912	1.257	1.41	1.221	0.819	1.14	1.043	0.929	0.946	1.164	2.156	2.111	1.856
TRINITY_DN59885_c0_g1_i3_orf1	TGF-beta-activated kinase 1 and MAP3K7-binding protein 1-like [Ostrinia furr	1	454	51	5.88	3	2	1	High	1	1.065	0.959	3.617	4.083	3.708	5.379	5.49	5.137	5.411	4.712	6.231	5.116	5.157	5.414
TRINITY_DN5207_c0_g2_i3_orf1	mucin-like [Galleria mellonella]	1	127	12.8	7.87	10	1	1	High	1	0.855	0.947	1.032	1.135	1.081	1.156	1.167	1.295	1.271	1.117	1.388	1.152	1.256	1.036
TRINITY_DN7275_c0_g1_i14_orf1	uncharacterized protein LOC114353817 [Ostrinia furnacalis]	1	144	15.4	4.6	9	1	1	High	1	1.033	0.853	0.673	0.806	0.725	1.215	0.876	0.952	1.078	1.037	1.076	0.76	0.894	0.781
TRINITY_DN2499_c0_g1_i4_orf1	WD repeat-containing protein 92 isoform X1 [Ostrinia furnacalis]	1	357	40	7.83	2	1	1	High	1	0.949	0.814	1.294	1.188	1.049	0.76	0.717	0.787	0.804	0.968	1.073	0.875	1.002	1.113
TRINITY_DN60792_c0_g1_i2_orf1	ATP-binding cassette sub-family D member 2 [Ostrinia furnacalis]	1	170	18.5	7.69	6	1	1	High	1	1.056	1.186	1.445	1.803	1.861	1.289	1.424	1.244	1.23	1.167	1.227	0.866	0.977	1.108
TRINITY_DN11245_c0_g1_i2_orf1	ITG-like peptide [Ostrinia furnacalis]	1	213	23.4	6.4	6	1	1	High	1	0.885	0.906	1.05	1.176	1.026	1.192	1.301	1.17	1.021	1.203	1.569	0.823	0.826	0.749
TRINITY_DN12397_c0_g1_i1_orf1	39S ribosomal protein L27, mitochondrial [Ostrinia furnacalis]	1	138	16	9.95	9	1	1	High	1	0.917	1.002	0.806	0.692	0.734	0.819	1.005	0.899	0.901	1.168	0.987	0.75	0.772	0.835
TRINITY_DN7378_c0_g1_i5_orf1	uncharacterized protein LOC114355479 isoform X2 [Ostrinia furnacalis]	1	1421	158.4	5.22	1	1	1	High	1	1.06	1.026	0.943	1.116	1.174	1.059	1.328	1.338	1.196	1.167	1.305	1.215	1.413	1.513
TRINITY_DN21181_c0_g1_i6_orf1	unnamed protein product, partial [Brenthis ino]	1	517	58.3	7.99	3	1	1	High	1	1.111	1.179	0.613	0.481	0.403	0.469	0.572	0.507	0.544	0.523	0.416	0.474	0.474	0.509
TRINITY_DN72017_c0_g1_i1_orf1	probable peroxisomal acyl-coenzyme A oxidase 1 isoform X1 [Ostrinia furnaca	1	88	9.7	9.38	11	1	1	High	1	1.103	1.274	1.273	1.102	0.982	1.252	1.329	1.128	1.382	1.436	1.16	1.114	0.962	0.923
TRINITY_DN88539_c0_g2_i1_orf1	uncharacterized protein LOC114352312 isoform X1 [Ostrinia furnacalis]	1	871	96	7.12	2	1	1	High	1	0.957	0.964	0.663	0.807	0.724	0.587	0.537	0.605	0.533	0.662	0.552	0.66	0.7	0.665
TRINITY_DN11666_c0_g1_i6_orf1	P protein-like [Ostrinia furnacalis]	1	813	91.4	7.62	2	1	1	High	1	1.074	1.04	1.081	1.028	1.335	1.133	1.498	1.214	0.973	0.906	0.857	1.57	1.737	1.619
TRINITY_DN2342_c0_g1_i1_orf1	BRCA1-associated protein [Ostrinia furnacalis]	1	562	61.4	5.25	1	1	1	High	1	0.992	0.821	1.087	0.946	0.991	0.774	0.888	0.837	0.745	0.859	0.808	1.181	0.925	0.917
TRINITY_DN113626_c0_g1_i3_orfp	TRINITY_DN113626_c0_g1_i3_m.80721 TRINITY_DN113626_c0_g1::TRINITY_DN	1	117	11.6	9.63	9	1	1	High	1	0.955	1.085	0.282	0.542	0.49	0.478	0.815	0.873	0.392	0.297	0.303	0.391	0.36	0.232
TRINITY_DN14679_c0_g1_i1_orf1	hypothetical protein evm_003043 [Chilo suppressalis]	1	820	90	7.3	1	1	1	High	1	1.032	1.038	0.066	0.07	0.03	0.106	0.084	0.108	0.098	0.081	0.122	0.077	0.07	0.083
TRINITY_DN10742_c0_g1_i4_orf1	ethanolamine-phosphate cytidylyltransferase isoform X1 [Ostrinia furnacalis]	1	383	43.2	7.46	4	1	1	High	1	0.974	1.2	1.449	1.213	1.415	1.508	1.747	1.498	1.435	1.306	1.453	1.204	1.17	1.233
TRINITY_DN34087_c0_g1_i4_orf1	protein SMG8 [Ostrinia furnacalis]	1	383	41.7	6.87	2	1	1	High	1	1.16	0.89	1.237	1.084	1.423	1.233	1.276	1.263	1.083	1.524	1.719	1.375	1.229	1.456
TRINITY_DN6299_c0_g1_i1_orf1	death-inducer obliterator 1 isoform X2 [Ostrinia furnacalis]	1	1336	147.7	9.13	1	1	1	High	1	1.105	1.107	0.734	0.801	0.693	0.832	0.893	0.92	0.561	0.846	0.816	0.834	0.672	0.755
TRINITY_DN20322_c0_g1_i1_orf1	KIF1-binding protein-like [Ostrinia furnacalis]	1	596	68.5	5.22	2	1	1	High	1	0.857	0.951	0.838	1.149	0.959	1.145	1.26	0.87	0.804	0.907	1.186	1.289	1.108	1.505
TRINITY_DN22962_c0_g1_i1_orf1	lysosomal acid glucosylceramidase-like isoform X2 [Ostrinia furnacalis]	1	476	53.5	7.05	4	1	1	High	1	0.875	0.851	2.748	2.66	2.71	1.727	1.419	1.691	1.344	1.433	1.477	1.596	1.751	1.233
TRINITY_DN47609_c0_g1_i1_orfp1	TRINITY_DN47609_c0_g1_i1_m.57205 TRINITY_DN47609_c0_g1::TRINITY_DN47	1	67	6.9	9.96	27	1	1	High	1	1.167	1.263	1.671	1.439	1.801	2.048	1.554	1.462	2.043	2.003	2.09	1.661	1.718	1.631
TRINITY_DN12464_c0_g1_i3_orf1	PH and SEC7 domain-containing protein 1 [Trichoplusia ni]	1	949	104.7	7.78	1	1	1	High	1	1.029	1.116	1.05	1.057	0.935	0.802	1.049	1.148	1.078	0.953	1.226	1.555	1.806	1.743
TRINITY_DN8971_c1_g1_i4_orf1	synaptosomal-associated protein 25 isoform X1 [Bombyx mori]	1	212	23.6	4.72	6	1	1	High	1	1.655	0.912	0.554	0.541	0.536	0.459	0.776	0.485	0.32	0.351	0.401	0.641	0.721	0.771
TRINITY_DN42364_c0_g1_i4_orf1	brain tumor protein [Ostrinia furnacalis]	1	877	94.4	5.73	1	1	1	High	1	0.965	0.978	0.561	0.509	0.669	0.623	0.565	0.597	0.572	0.531	0.543	0.668	0.591	0.532
TRINITY_DN49508_c0_g2_i8_orf1	putative fatty acyl-CoA reductase CG5065 [Ostrinia furnacalis]	1	535	60.6	8.22	2	1	1	High	1	0.991	0.934	0.259	0.218	0.287	0.292	0.355	0.339	0.258	0.365	0.238	0.22	0.226	0.215
TRINITY_DN33705_c0_g1_i1_orf1	atlastin-like isoform X4 [Ostrinia furnacalis]	1	102	11.1	5.29	6	2	1	High	1	0.994	0.917	1.597	1.389	1.397	1.769	1.821	1.844	2.306	1.074	1.149	1.255	1.265	1.368
TRINITY_DN129259_c0_g2_i1_orf1	spectrin alpha chain-like isoform X3 [Helicoverpa armigera]	1	77	8.6	4.7	12	1	1	High	1	0.878	1.003	0.76	0.912	1.013	0.838	0.9	0.859	0.746	0.817	0.783	0.802	0.778	0.835
TRINITY_DN86309_c0_g1_i4_orf1	filamin-A isoform X2 [Ostrinia furnacalis]	1	136	14.2	4.34	6	1	1	High	1	1.001	1.133	1.03	0.908	1.029	1.023	1.043	1.043	1.126	0.953	0.983	1.683	1.616	1.364
TRINITY_DN3000_c0_g1_i6_orf1	fatty-acid amide hydrolase 2-A isoform X3 [Ostrinia furnacalis]	1	521	56.2	8.19	2	1	1	High	1	0.778	0.871	0.995	0.944	0.797	0.943	1.056	0.832	0.945	1.417	0.702	0.83	1.071	1.054
TRINITY_DN3738_c0_g1_i5_orf1	23 kDa integral membrane protein-like [Ostrinia furnacalis]	1	259	29	7.85	4	1	1	High	1	1.062	0.924	1.245	1.347	1.485	1.606	1.403	1.755	1.88	2.045	1.744	1.245	1.112	1.505
TRINITY_DN526_c0_g1_i1_orf1	secretory phospholipase A2 receptor-like [Ostrinia furnacalis]	1	256	29.1	5.4	4	1	1	High	1	1.013	1.056	1.455	1.229	1.381	0.841	0.771	0.809	1.111	1.05	1.033	1.112	1.132	1.224
TRINITY_DN4659_c0_g1_i2_orf1	uncharacterized protein LOC114351134 [Ostrinia furnacalis]	1	394	45	5.44	2	1	1	High	1	0.965	0.837	0.552	0.475	0.478	0.486	0.58	0.535	0.538	0.493	0.558	1.062	1.002	0.808
TRINITY_DN6642_c0_g1_i2_orf1	protein purity of essence [Ostrinia furnacalis]	1	311	34.3	6.06	5	1	1	High	1	0.975	0.823	1.294	1.334	1.363	1.73	2.509	1.8	1.507	1.464	1.339	0.894	1.021	1.006
TRINITY_DN13160_c0_g1_i1_orf1	serine/threonine-protein kinase 10-like, partial [Ostrinia furnacalis]	1	312	34.7	9.73	4	3	1	High	1	0.855	1.009	0.67	0.783	0.649	0.513	0.697	0.641	0.623	0.614	0.638	0.521	0.665	0.691
TRINITY_DN69557_c0_g1_i1_orf1	hypothetical protein G9C98_005708, partial [Cotesia typhae]	1	214	24.7	9.32	3	1	1	High	1	1.041	0.984	1.253	1.015	1.152	0.91	0.908	1.148	1.017	0.813	0.795	0.647	0.541	0.58
TRINITY_DN3105_c0_g1_i4_orf1	nose resistant to fluoxetine protein 6-like [Ostrinia furnacalis]	1	727	81.7	8.18	2	1	1	High	1	1.031	1.263	1.464	1.236	1.232	0.813	0.872	1.004	0.913	0.881	0.807	0.611	0.473	0.79
TRINITY_DN99900_c0_g1_i3_orf1	laminin subunit alpha-like isoform X1 [Ostrinia furnacalis]	1	105	11.3	7.99	8	1	1	High	1	1.102	1.385	1.603	1.869	1.335	1.303	0.988	1.504	1.471	0.91	1.019	0.929	1.08	1.233
TRINITY_DN22257_c0_g1_i7_orf1	spatacsin isoform X1 [Ostrinia furnacalis]	1	1956	224.3	6.44	1	1	1	High	1	0.771	0.704	1.231	1.43	1.131	1.436	1.601	1.474	1.639	1.821	1.426	1.072	1.141	1.499
TRINITY_DN38568_c0_g1_i1_orf1	unnamed protein product, partial [Diatraea saccharalis]	1	337	37.5	5.88	4	1	1	High	1	1.008	1.147	1.796	1.965	1.458	2.097	1.883	1.521	1.62	1.48	1.586	0.738	0.646	0.584
TRINITY_DN36281_c0_g1_i2_orf1	putative uncharacterized protein DDB_G0282499 isoform X1 [Ostrinia furnacali	1	689	81.1	8.94	2	1	1	High	1	1.245	0.957	1	1.105	1.331	1.138	1.263	1.179	0.993	1.119	1.231	2.481	2.531	1.734
TRINITY_DN21134_c0_g1_i6_orf1	signal recognition particle 14 kDa protein [Ostrinia furnacalis]	1	107	12.1	10.18	9	1	1	High	1	0.835	1.105	0.985	1.102	0.995	0.894	1.194	1.263	1.062	1.36	1.003	1.075	0.988	1.085
TRINITY_DN978_c9_g2_i1_orf1	hypothetical protein evm_000959 [Chilo suppressalis]	1	131	14.8	5.63	8	1	1	High	1	1.098	1.038	0.674	0.763	0.764	0.697	0.681	0.701	0.685	0.521	0.687	2.005	2.175	2.059
TRINITY_DN30097_c0_g1_i2_orf1	unnamed protein product [Chilo suppressalis]	1	576	65.3	7.09	1	1	1	High	1	1.185	1.347	0.75	0.564	0.604	0.753	0.754	0.698	0.623	0.611	0.527	0.88	0.752	0.795
TRINITY_DN54275_c0_g1_i4_orf1	PREDICTED: aryl-hydrocarbon-interacting protein-like 1 [Amyelois transitella]	1	324	37.6	7.18	3	1	1	High	1	0.872	0.913	1.063	1.153	1.432	1.031	1.195	1.141	1.129	1.594	1.193	1.194	1.117	1.238
TRINITY_DN73945_c0_g5_i3_orf1	cyclin-dependent kinase 12 isoform X1 [Diachasma alloeum]	1	799	88.9	9.13	1	1	1	High	1	1.07	1.06	0.665	0.637	0.764	0.81	0.714	0.863	0.651	0.513	0.502	0.66	0.633	0.651
TRINITY_DN19829_c0_g2_i1_orf1	28S ribosomal protein S35, mitochondrial [Ostrinia furnacalis]	1	158	18.6	5.12	6	1	1	High	1	0.975	0.951	0.477	0.428	0.573	0.419	0.435	0.401	0.527	0.486	0.424	0.495	0.494	0.461
TRINITY_DN12024_c0_g2_i2_orf1	pancreatic lipase-related protein 2 isoform X1 [Ostrinia furnacalis]	1	294	33.2	8.41	3	1	1	High	1	0.923	0.981	1.089	0.984	1.175	1.194	1.292	1.121	1.302	1.522	1.278	1.369	1.298	1.36
TRINITY_DN75188_c0_g1_i1_orf1	fatty acid-binding protein 1-like [Ostrinia furnacalis]	1	131	14.7	5.15	7	1	1	High	1	0.94	1.044	0.231	0.18	0.173	0.189	0.189	0.155	0.133	0.139	0.139	0.252	0.204	0.229
TRINITY_DN1459_c0_g1_i2_orf1	reticulon-1 isoform X1 [Ostrinia furnacalis]	1	133	13.9	5.2	7	2	1	High	1	1.074	0.994	1.187	1.238	1.283	0.927	1.073	0.983	1.057	0.916	1.172	0.89	0.879	0.858
TRINITY_DN5637_c0_g1_i3_orf1	U3 small nucleolar RNA-associated protein 6 homolog [Ostrinia furnacalis]	1	566	66.8	6.09	2	1	1	High	1	0.68	0.595	0.561	0.858	0.687	0.623	0.703	0.802	0.675	1.505	1.01	0.587	0.761	0.793
TRINITY_DN64181_c0_g1_i1_orf1	uncharacterized protein LOC114356431 isoform X2 [Ostrinia furnacalis]	1	71	7.9	7.05	18	1	1	High	1	1.297	1.269	5.928	4.924	4	3.462	1.759	2.889	3.356	2.457	3.683	10.081	9.715	7.934
TRINITY_DN384_c0_g1_i8_orf1	unnamed protein product [Chilo suppressalis]	1	1943	216.4	6.83	1	1	1	High	1	0.838	0.671	1.629	1.812	1.563	2.077	1.815	1.852	1.772	1.983	1.652	1.173	0.909	1.074
TRINITY_DN17825_c1_g1_i1_orf1	39S ribosomal protein L1, mitochondrial [Ostrinia furnacalis]	1	350	39.6	9.14	3	2	1	High	1	0.978	0.927	0.29	0.329	0.372	0.336	0.329	0.3	0.268	0.229	0.325	0.312	0.317	0.321
TRINITY_DN38307_c0_g1_i1_orfp1	TRINITY_DN38307_c0_g1_i1_m.10661 TRINITY_DN38307_c0_g1::TRINITY_DN38	1	65	7.9	7.24	17	1	1	High	1	0.741	1.262	4.581	3.035	3.407	7.437	5.538	5.048	4.837	3.613	4.328	13.389	14.227	15.776
TRINITY_DN45037_c0_g1_i1_orf1	trafficking protein particle complex subunit 1 [Ostrinia furnacalis]	1	145	16.8	8.88	9	1	1	High	1	0.726	0.937	2.196	1.734	2.159	2.124	2.268	3.039	4.667	4.32	4.151	2.609	2.909	3.34
TRINITY_DN6205_c0_g1_i8_orf1	phenoloxidase-activating factor 2-like [Ostrinia furnacalis]	1	240	26.2	8.51	5	1	1	High	1	0.864	1.102	1.393	1.455	1.296	0.807	0.98	0.87	0.821	1.072	0.787	1.144	1.285	1.257
TRINITY_DN4991_c0_g1_i1_orf1	unnamed protein product, partial [Iphiclides podalirius]	1	293	33.6	6.35	3	1	1	High	1	1.065	1.298	1.226	1.571	1.519	1.11	1.382	1.457	1.069	1.33	1.137	1.011	1.072	1.058
TRINITY_DN5124_c0_g1_i2_orf1	glucose dehydrogenase [FAD, quinone]-like [Ostrinia furnacalis]	1	585	65.5	7.2	2	1	1	High	1	1.199	1.024	1.903	1.932	1.253	1.523	1.618	1.426	1.717	3.839	2.122	2.082	1.504	1.89
TRINITY_DN1045_c0_g1_i6_orf1	ornithine decarboxylase 1-like isoform X1 [Ostrinia furnacalis]	1	476	52.1	7.62	2	1	1	High	1	1.118	1.109	0.649	0.75	0.719	0.745	0.89	0.691	0.561	0.78	0.713	0.989	0.96	1.027
TRINITY_DN34159_c0_g2_i1_orf1	guanine nucleotide exchange factor subunit Rich isoform X1 [Ostrinia furnacali	1	196	21.7	9.69	5	1	1	High	1	0.993	1.112	0.581	0.461	0.443	0.429	0.442	0.51	0.978	0.449	0.593	0.678	0.739	0.624
TRINITY_DN336_c0_g1_i6_orfp1	TRINITY_DN336_c0_g1_i6_m.64791 TRINITY_DN336_c0_g1::TRINITY_DN336_c0	1	60	6.3	5.11	12	1	1	High	1	1.017	1.032	0.095	0.06	0.168	0.105	0.098	0.108	0.188	0.159	0.195	0.045	0.061	0.057
TRINITY_DN6118_c0_g1_i13_orf1	trehalase-2 [Omphisa fuscidentalis]	1	666	76.2	6.02	2	1	1	High	1	0.941	1.063	1.012	0.765	0.92	0.993	0.934	1.062	1.037	1.517	0.94	0.762	0.79	0.972
TRINITY_DN49742_c0_g1_i4_orf1	neuroglobin-like [Ostrinia furnacalis]	1	190	21.5	5.6	11	1	1	High	1	1.287	1.129	0.526	0.542	0.685	0.539	0.498	0.563	0.524	0.367	0.55	0.554	0.606	0.477
TRINITY_DN1421_c0_g1_i2_orf1	uncharacterized protein C14orf119 isoform X1 [Ostrinia furnacalis]	1	176	19.7	4.22	6	1	1	High	1	0.804	0.909	0.927	0.809	0.855	0.982	1.072	0.723	0.701	1.154	1.08	0.721	0.836	1.199
TRINITY_DN124303_c0_g1_i4_orf1	dnaJ homolog dnj-5 [Trichoplusia ni]	1	97	11.2	6.54	15	1	1	High	1	1.066	1.114	1.31	1.278	0.938	1.064	0.993	1.742	1.408	1.767	0.844	1.073	0.655	1.133
TRINITY_DN22526_c0_g1_i3_orf1	alpha-1,3-mannosyl-glycoprotein 2-beta-N-acetylglucosaminyltransferase [C	1	436	50.2	7.72	2	1	1	High	1	0.978	0.896	0.939	0.807	0.912	1.024	1.059	0.934	0.751	0.935	0.639	0.94	0.879	1.107
TRINITY_DN9518_c0_g1_i6_orf1	ralA-binding protein 1 isoform X3 [Vanessa tameamea]	1	533	60.6	5.8	2	1	1	High	1	0.995	0.923	1.195	1.096	1.074	0.896	0.983	1.089	1.074	0.838	0.91	1.128	1.04	1.13
TRINITY_DN6808_c0_g1_i2_orf1	rap1 GTPase-GDP dissociation stimulator 1-B [Ostrinia furnacalis]	1	177	19.4	6.29	5	1	1	High	1	0.967	0.923	0.845	0.847	0.928	0.929	0.98	1.006	0.725	1.243	0.83	0.813	0.813	0.949

TRINITY_DN41664_c0_g1_i4_orf1	uncharacterized protein LOC114356631 [Ostrinia furnacalis]	1	117	14.5	8.98	9	1	1	High	1	1.065	1.042	1.041	0.993	0.987	0.827	0.775	0.736	0.743	0.738	0.713	0.941	0.932	0.953
TRINITY_DN15388_c0_g1_i5_orf1	RNA-binding protein 28-like isoform X1 [Ostrinia furnacalis]	1	754	85.7	9.26	1	1	1	High	1	1.027	1.004	0.505	0.486	0.626	0.637	0.553	0.591	0.588	0.516	0.437	0.566	0.587	0.472
TRINITY_DN15256_c0_g1_i8_orf1	pre-mRNA-splicing regulator female-lethal(2)D [Ostrinia furnacalis]	1	299	33.3	5.2	3	1	1	High	1	1.421	1.353	0.772	1.037	0.826	0.987	1.015	0.984	0.546	0.774	1.063	1.437	1.121	1.302
TRINITY_DN10479_c0_g1_i6_orf1	unnamed protein product [Chrysodeixis includens]	1	243	26.6	6.54	4	1	1	High	1	0.78	0.941	0.533	0.58	0.557	0.535	0.673	0.596	0.473	0.463	0.513	1.45	1.369	1.554
TRINITY_DN131371_c0_g1_i1_orf1	golgin subfamily B member 1-like [Ostrinia furnacalis]	1	106	11.9	5.29	8	1	1	High	1	1.032	0.959	0.784	0.893	0.554	0.568	0.75	0.745	0.501	0.369	0.452	0.596	0.612	0.757
TRINITY_DN3273_c0_g1_i4_orf1	FK506-binding protein-like [Galleria mellonella]	1	289	33.2	5.24	3	1	1	High	1	1.039	0.985	1.083	0.895	1.039	0.98	0.99	1.056	1.092	1.011	0.983	1.336	1.261	1.165
TRINITY_DN10629_c0_g1_i1_orf1	caspase-1-like [Ostrinia furnacalis]	1	285	32.9	6.86	3	1	1	High	1	0.854	0.911	0.62	0.815	0.678	0.745	0.86	0.745	0.624	0.616	0.728	0.548	0.353	0.521
TRINITY_DN31619_c0_g1_i2_orf1	endocuticle structural glycoprotein ABD-4-like [Ostrinia furnacalis]	1	137	14.7	5.03	7	1	1	High	1	1.01	0.949	0.757	0.759	0.701	0.539	0.702	0.714	0.7	0.534	0.587	2.45	2.714	2.14
TRINITY_DN28543_c0_g1_i2_orf1	uncharacterized protein KIAA2013 homolog [Ostrinia furnacalis]	1	625	71.1	8.31	1	1	1	High	1	0.922	1.004	0.827	0.836	0.863	0.925	0.86	0.897	0.775	0.774	0.937	0.831	0.947	0.946
TRINITY_DN10672_c0_g1_i3_orf1	neurofilament heavy polypeptide-like isoform X10 [Ostrinia furnacalis]	1	559	60	7.17	2	1	1	High	1	0.965	1.005	0.734	0.754	0.791	0.747	0.856	0.777	0.639	0.526	0.675	0.8	0.811	0.751
TRINITY_DN1650_c0_g1_i5_orf1	uncharacterized protein LOC114355357 [Ostrinia furnacalis]	1	177	19.4	8.16	4	1	1	High	1	1.027	1.065	2.444	1.567	2.161	2.861	2.194	3.162	2.291	1.706	1.16	1.545	1.61	1.537
TRINITY_DN12787_c1_g1_i1_orf1	unnamed protein product [Parnassius apollo]	1	101	11.8	7.01	9	1	1	High	1	1.077	1.345	0.835	0.953	0.807	0.735	0.884	0.825	0.821	0.802	0.845	0.886	1.011	0.797
TRINITY_DN141353_c0_g1_i1_orf1	uncharacterized protein LOC123263755 [Cotesia glomerata]	1	76	9.1	10.13	14	1	1	High	1	1.021	0.762	0.804	0.714	0.578	1.017	0.753	0.842	0.749	1.509	0.693	0.394	0.461	0.392
TRINITY_DN52_c0_g1_i4_orf1	unnamed protein product [Chilo suppressalis]	1	504	57	9.33	2	1	1	High	1	0.99	1.036	0.811	0.863	0.854	0.988	1.041	1.013	1.065	0.868	0.834	1.118	1.051	1.068
TRINITY_DN17003_c0_g1_i1_orf1	mucin-5AC [Ostrinia furnacalis]	1	657	72.5	9.16	2	1	1	High	1	0.974	1.092	0.802	0.945	0.475	0.765	0.583	0.621	0.751	0.563	0.631	1.567	1.51	1.077
TRINITY_DN9029_c0_g1_i4_orf1	venom protease-like [Ostrinia furnacalis]	1	83	9.1	5.31	14	1	1	High	1	0.985	0.97	0.954	1.007	1.01	0.885	0.994	0.791	0.784	1.029	0.977	0.711	0.747	0.822
TRINITY_DN9119_c0_g1_i3_orf1	actin-binding Rho-activating protein [Helicoverpa armigera]	1	192	21.7	7.3	4	1	1	High	1	0.962	0.868	0.421	0.317	0.366	0.345	0.353	0.388	0.387	0.422	0.433	0.404	0.455	0.385
TRINITY_DN18912_c1_g1_i1_orf1	engulfment and cell motility protein 1 [Ostrinia furnacalis]	1	742	84.3	5.99	1	1	1	High	1	1.087	1.033	0.732	0.724	0.702	0.639	0.816	0.8	0.576	0.701	0.791	0.865	0.869	1.009
TRINITY_DN6205_c0_g1_i1_orf1	phenoloxidase-activating factor 2-like [Ostrinia furnacalis]	1	274	29.9	7.62	4	1	1	High	1	1.102	1.211	1.812	1.757	1.887	1.409	1.414	1.609	2.073	2.238	2.106	2.141	2.271	2.043
TRINITY_DN28018_c0_g5_i1_orf1	microtubule-associated protein futsch-like isoform X6 [Ostrinia furnacalis]	1	429	48.9	4.75	2	1	1	High	1	0.92	0.899	0.91	0.925	0.951	1.081	1.06	1.019	0.854	1.026	1.07	0.992	0.956	0.965
TRINITY_DN81715_c0_g1_i1_orf1	gamma-interferon-inducible lysosomal thiol reductase-like [Ostrinia furnacalis	1	232	26.1	7.08	3	1	1	High	1	1.078	1.097	0.434	0.438	0.489	0.381	0.474	0.448	0.353	0.444	0.341	0.897	0.806	0.868
TRINITY_DN816_c0_g1_i3_orf1	calcium-binding mitochondrial carrier protein SCaMC-2 isoform X1 [Ostrinia fu	1	473	53	7.93	2	1	1	High	1	1.032	0.807	0.548	0.618	0.594	0.437	0.661	0.606	0.491	0.501	0.548	0.64	0.672	0.614
TRINITY_DN33272_c0_g1_i5_orf1	Low-density lipoprotein receptor-related protein 1 [Papilio xuthus]	1	86	9.2	4.67	13	1	1	High	1	0.892	0.977	3.081	3.877	3.779	3.437	3.412	3.496	2.337	2.648	3.892	4.788	5.18	4.989
TRINITY_DN5457_c0_g1_i4_orf1	unnamed protein product [Chrysodeixis includens]	1	184	20.1	5.15	4	1	1	High	1	1.039	0.949	0.678	0.685	0.656	0.563	0.611	0.574	0.549	0.577	0.52	0.778	0.87	0.861
TRINITY_DN7986_c1_g1_i4_orf1	neuropathy target esterase sws [Ostrinia furnacalis]	1	1278	142.9	8.31	1	1	1	High	1	1.078	0.996	0.899	0.927	0.988	0.647	0.791	1.14	0.818	0.705	1.035	0.884	0.663	0.732
TRINITY_DN11084_c1_g1_i2_orf1	uncharacterized protein LOC114362953 [Ostrinia furnacalis]	1	364	41.8	6.29	2	1	1	High	1	0.836	0.931	0.812	0.946	0.988	1.003	0.991	0.99	0.874	0.836	0.93	1.061	1.054	1.129
TRINITY_DN16226_c0_g1_i1_orf1	zinc finger CCCH domain-containing protein 14 isoform X1 [Ostrinia furnacalis	1	921	104	8.88	1	1	1	High	1	0.982	0.982	1.1	1.171	1.084	0.85	0.986	1.007	1.076	1.104	0.75	0.966	1.117	1.01
TRINITY_DN3760_c0_g1_i1_orf1	something about silencing protein 10 [Ostrinia furnacalis]	2	465	53.7	5.83	5	2	2	High	1	1.074	1.099	0.611	0.667	0.748	0.77	0.557	0.646	0.767	0.844	0.647	0.539	0.545	0.572
TRINITY_DN124711_c0_g1_i1_orf1	muskelin isoform X1 [Ostrinia furnacalis]	1	269	31.3	6.79	4	1	1	High	1	0.974	1.117	0.715	0.777	0.584	1.618	1.317	1.435	0.702	0.815	0.522	0.638	0.689	0.627
TRINITY_DN10057_c0_g2_i1_orf1	cell wall protein DAN4 [Ostrinia furnacalis]	1	1433	157.8	5.31	1	1	1	High	1	1.063	0.995	0.585	0.527	0.491	0.483	0.621	0.55	0.658	0.404	0.495	1.828	1.98	1.96
TRINITY_DN94755_c0_g1_i5_orfp1	TRINITY_DN94755_c0_g1_i5_m.62794 TRINITY_DN94755_c0_g1::TRINITY_DN94	1	69	8	9.35	16	1	1	High	1	1.022	0.909	0.06	0.082	0.131	0.092	0.098	0.089	0.089	0.078	0.09	0.073	0.098	0.089
TRINITY_DN4929_c0_g1_i1_orf1	unnamed protein product [Danaus chrysippus]	1	573	65	6.65	2	1	1	High	1	1.119	0.962	0.458	0.425	0.522	0.69	0.814	0.952	0.707	0.626	0.73	0.818	0.825	0.786
TRINITY_DN3962_c0_g1_i6_orf1	follicle-stimulating hormone receptor-like [Ostrinia furnacalis]	1	477	53.6	7.5	1	3	1	High	1	1.069	1.087	1.396	1.336	1.067	1.641	1.027	1.315	0.994	0.709	0.998	1.761	1.745	1.517
TRINITY_DN29144_c0_g3_i1_orf1	PREDICTED: ADP-ribosylation factor 6 [Papilio polytes]	1	175	20.1	8.97	9	1	1	High	1	0.812	0.927	1.093	0.987	1.127	1.447	2.241	1.488	0.572	0.527	0.603	1.16	1.191	1.18
TRINITY_DN29106_c1_g1_i5_orf1	uncharacterized protein LOC114365311 [Ostrinia furnacalis]	1	283	32.6	6.83	4	1	1	High	1	1.125	0.752	1.019	1.003	1.165	1.148	1.179	1.159	1.338	1.775	1.075	0.997	0.994	1.099
TRINITY_DN10871_c0_g2_i1_orf1	eukaryotic translation initiation factor 4 gamma 2 [Ostrinia furnacalis]	1	255	27.2	9.7	4	1	1	High	1	1.011	1.026	1.041	1.266	1.121	0.944	0.875	0.988	0.86	1.018	1.043	1.006	0.829	1.106
TRINITY_DN32362_c0_g1_i1_orf1	uncharacterized protein LOC114350902 [Ostrinia furnacalis]	1	443	48.5	9.44	4	1	1	High	1	0.9	0.979	0.893	0.983	0.941	0.837	0.956	0.845	0.735	0.785	0.977	1.103	0.959	1.137
TRINITY_DN67495_c0_g1_i1_orf1	hypothetical protein KGM_200102A, partial [Danaus plexippus plexippus]	1	76	8.4	7.53	16	1	1	High	1	0.983	0.981	0.693	0.814	0.713	0.707	0.801	0.865	0.864	0.937	0.992	0.618	0.768	0.623
TRINITY_DN9741_c0_g1_i3_orf1	metaxin-2 isoform X4 [Manduca sexta]	1	349	39.8	6.19	2	1	1	High	1	1.069	0.944	0.533	0.568	0.581	0.641	0.586	0.615	0.508	0.528	0.537	0.666	0.7	0.695
TRINITY_DN31943_c0_g1_i1_orf1	proteoglycan Cow [Ostrinia furnacalis]	1	371	41.1	7.02	2	1	1	High	1	1.035	1.127	0.913	0.991	0.808	0.876	1.299	0.941	0.934	1.13	1.208	1.6	1.68	1.758
TRINITY_DN4273_c1_g1_i5_orf1	tetraspanin-13 isoform X1 [Ostrinia furnacalis]	1	226	25.3	7.47	4	1	1	High	1	0.881	0.891	1.186	1.242	1.436	1.206	1.204	1.129	1.211	1.16	1.33	1.875	2.152	1.994
TRINITY_DN2475_c0_g1_i1_orf1	LOW QUALITY PROTEIN: uncharacterized protein LOC114363766 [Ostrinia furr	1	527	58.6	9.41	2	1	1	High	1	0.843	1.167	1.254	1.37	1.321	1.265	1.439	1.366	1.186	2.218	1.374	1.462	1.344	1.214
TRINITY_DN44285_c0_g1_i1_orf1	uncharacterized protein LOC114354962 [Ostrinia furnacalis]	1	35	3.8	4.01	31	1	1	High	1	1.137	1.099	0.911	0.953	0.943	1.099	1.173	1.12	1.297	1.388	1.189	1.056	1.123	1.092
TRINITY_DN54925_c0_g1_i1_orf1	unnamed protein product [Arctia plantaginis]	1	350	39.3	8.29	3	1	1	High	1	1.142	1.092	0.968	0.883	1.06	0.798	1.024	1.003	1.029	0.885	0.92	1.32	1.268	1.083
TRINITY_DN806_c0_g2_i1_orf1	uncharacterized protein LOC114355167 [Ostrinia furnacalis]	1	160	17.8	4.63	6	1	1	High	1	1.055	1.011	0.952	0.861	0.964	0.831	0.815	0.802	0.638	0.866	0.683	2.529	2.645	2.649
TRINITY_DN376_c1_g1_i1_orf1	matrix metalloproteinase-25-like [Ostrinia furnacalis]	1	512	59.2	5.5	2	1	1	High	1	1.134	1.205	1.763	1.786	1.73	1.967	2.127	1.815	2.119	2.215	2.27	2.483	2.726	2.684
TRINITY_DN42310_c0_g1_i1_orf1	uncharacterized protein LOC114349955 [Ostrinia furnacalis]	1	74	8.4	9.7	14	1	1	High	1	0.945	0.971	0.599	0.658	0.727	0.622	0.567	0.693	0.627	0.525	0.579	0.605	0.584	0.499
TRINITY_DN6396_c0_g1_i1_orf1	PR domain zinc finger protein 10-like [Ostrinia furnacalis]	1	699	79.4	8.63	1	1	1	High	1	1.078	1.102	0.734	0.757	0.787	0.569	0.597	0.666	0.514	0.574	0.447	0.608	0.66	0.701
TRINITY_DN64_c0_g1_i4_orf1	unnamed protein product [Chilo suppressalis]	1	1416	161.7	8.44	0	1	1	High	1	1.144	1.443	1.098	1.112	0.869	1.068	0.841	0.862	0.784	0.93	0.507	0.567	0.613	0.339
TRINITY_DN147475_c0_g1_i1_orf1	casein kinase II subunit beta, partial [Rhincodon typus]	1	158	18.3	4.56	5	1	1	High	1	0.95	0.98	0.629	0.693	0.664	0.504	0.628	0.582	0.454	0.476	0.631	0.934	0.975	0.989
TRINITY_DN49872_c0_g1_i2_orf1	probable cytosolic iron-sulfur protein assembly protein Ciao1 [Ostrinia furnaca	1	335	37.6	5.1	3	1	1	High	1	1.018	0.959	0.876	0.758	0.675	1.061	1.003	0.983	0.931	0.8	0.902	1.367	1.299	1.122
TRINITY_DN7533_c0_g1_i1_orf1	WD repeat-containing protein 89-like [Ostrinia furnacalis]	1	381	42.7	5.31	3	1	1	High	1	1.209	1.185	0.93	0.883	1.065	0.869	0.952	1.138	1.11	1.211	0.865	0.879	1.048	0.958
TRINITY_DN21285_c0_g1_i3_orf1	uncharacterized protein LOC114351683 isoform X7 [Ostrinia furnacalis]	1	180	20.3	9.1	6	1	1	High	1	0.974	0.888	1.467	1.462	1.412	1.284	1.386	1.409	1.522	1.078	1.286	1.036	1.084	1.037
TRINITY_DN20356_c0_g1_i5_orf1	uncharacterized protein LOC114362428 [Ostrinia furnacalis]	1	533	60	9.7	2	1	1	High	1	1.05	0.964	11.209	12.224	11.917	0.267	0.586	1.112	1	1.05	1.375	1.765	1.464	1.271
TRINITY_DN11735_c0_g1_i5_orf1	TBC1 domain family member 22B isoform X1 [Ostrinia furnacalis]	1	489	56.1	6.57	2	1	1	High	1	0.727	0.975	0.742	0.924	1.139	1.04	0.934	0.902	0.966	1.066	0.992	0.675	0.635	0.485
TRINITY_DN1181_c0_g1_i1_orf1	cytochrome c oxidase assembly factor 5 [Ostrinia furnacalis]	1	81	9.3	8.6	12	1	1	High	1	1.195	1.132	0.574	0.653	0.591	0.63	0.706	0.794	0.712	1.104	0.785	0.945	0.746	1.064
TRINITY_DN6221_c0_g1_i5_orf1	unnamed protein product [Diatraea saccharalis]	1	87	9.6	9.28	9	1	1	High	1	0.944	1.05	0.2	0.323	0.358	0.238	0.251	0.304	0.3	0.346	0.237	0.255	0.223	0.253
TRINITY_DN91877_c0_g1_i1_orf1	NADH dehydrogenase [ubiquinone] 1 alpha subcomplex assembly factor 2 [Os	1	150	17.3	9.33	6	1	1	High	1	0.964	0.953	0.975	0.969	0.923	0.484	0.549	0.471	1.04	0.888	0.533	0.671	0.694	0.69
TRINITY_DN11587_c0_g1_i7_orf1	elongation of very long chain fatty acids protein AAEL008004-like isoform X1 [	1	310	36.4	9.48	4	1	1	High	1	1.011	1.014	0.155	0.113	0.173	0.122	0.166	0.167	0.122	0.141	0.142	0.211	0.187	0.196
TRINITY_DN710_c0_g1_i11_orfp1	TRINITY_DN710_c0_g1_i11_m.67699 TRINITY_DN710_c0_g1::TRINITY_DN710_c	1	193	21.3	7.71	7	1	1	High	1	0.874	0.812	3.498	4.069	3.914	4.342	4.044	3.725	2.467	2.255	2.685	1.096	1.071	1.168
TRINITY_DN20244_c0_g1_i1_orfp1	uncharacterized protein LOC125235519 [Leguminivora glycinivorella]	1	117	12.3	7.47	7	1	1	High	1	1.216	1.016	0.691	0.433	0.519	0.525	0.565	0.605	0.711	0.61	0.748	1.93	2.581	2.157
TRINITY_DN14347_c0_g1_i1_orf1	putative nuclease HARBI1 [Ostrinia furnacalis]	1	495	55.8	8.48	2	1	1	High	1	0.824	0.964	0.928	1.075	0.852	1.094	0.796	0.735	0.805	0.549	0.532	0.96	0.869	0.971
TRINITY_DN86956_c0_g5_i1_orf1	PREDICTED: protein sly1 homolog [Microplitis demolitor]	1	629	71.3	6.6	1	1	1	High	1	1.074	1.194	0.381	0.351	0.393	0.396	0.308	0.328	0.393	0.275	0.332	0.561	0.424	0.411
TRINITY_DN4142_c0_g1_i5_orf1	GDP-fucose protein O-fucosyltransferase 1 [Ostrinia furnacalis]	1	378	43.5	8.6	2	1	1	High	1	1.304	1.087	1.173	1.263	1.537	1.319	1.538	1.283	1.009	1.092	0.874	1.155	1.099	1.056
TRINITY_DN102051_c0_g1_i1_orf1	spectrin repeat domain-containing protein [Phthorimaea operculella]	1	75	7.8	9.48	12	1	1	High	1	0.872	0.89	1.083	1.149	1.241	1.279	0.932	1.043	1.165	0.905	1.136	0.752	0.663	0.684
TRINITY_DN42738_c0_g1_i1_orf1	arf-GAP domain and FG repeat-containing protein 1 [Ostrinia furnacalis]	1	347	38.3	8.72	5	1	1	High	1	1.046	1.04	0.521	0.489	0.563	0.507	0.523	0.644	0.5	0.364	0.4	0.457	0.474	0.501
TRINITY_DN4535_c0_g1_i2_orf1	mitochondrial Rho GTPase [Pectinophora gossypiella]	1	629	71.5	6.4	2	1	1	High	1	0.863	0.789	0.786	0.6	0.761	0.806	1.03	0.874	0.734	1.004	0.668	0.764	0.809	0.897
TRINITY_DN48641_c0_g1_i4_orf1	RNA-binding protein 45-like [Galleria mellonella]	1	475	53.8	8.21	2	1	1	High	1	1.218	1.089	0.577	0.654	0.661	0.501	0.362	0.396	0.431	0.449	0.47	1.367	1.652	1.444
TRINITY_DN17693_c0_g1_i10_orf1	acetylcholinesterase-like [Ostrinia furnacalis]	1	656	74	6.4	2	1	1	High	1	1.017	1.016	1.137	1.238	1.047	1.322	1.021	0.893	2.561	2.088	2.534	1.363	1.463	1.31
TRINITY_DN4143_c0_g1_i1_orf1	zinc finger protein 530-like isoform X8 [Ostrinia furnacalis]	1	180	20.3	4.83	5	1	1	High	1	0.996	1.074	0.257	0.32	0.405	0.414	0.304	0.237	0.26	0.558	0.436	0.582	0.477	0.49
TRINITY_DN84357_c0_g1_i1_orf1	4-coumarate--CoA ligase 1-like [Ostrinia furnacalis]	1	531	58.8	7.71	2	1	1	High	1	0.963	0.983	0.732	0.708	0.786	0.294	0.428	0.396	0.553	0.547	0.595	0.291	0.351	0.305
TRINITY_DN957_c0_g1_i18_orf1	proton channel OtopLc-like isoform X6 [Ostrinia furnacalis]	1	896	100.2	8.92	1	1	1	High	1	0.995	1.031	0.693	0.762	0.59	0.963	1.193	1.125	1.042	0.917	0.9	1.051	1.011	0.935
TRINITY_DN4842_c0_g1_i5_orf1	cytochrome c oxidase assembly factor 4 homolog, mitochondrial isoform X1 [C	1	70	8.2	7.21	13	1	1	High	1	1	1.051	0.355	0.45	0.381	0.328	0.302	0.346	0.335	0.345	0.405	0.503	0.381	0.472
TRINITY_DN47609_c0_g1_i3_orfp1	TRINITY_DN47609_c0_g1_i3_m.57209 TRINITY_DN47609_c0_g1::TRINITY_DN47	1	99	10.1	6.57	11	1	1	High	1	1.083	1.158	1.203	0.945	0.917	1.305	1.091	0.976	1.797	5.591	1.605	1.147	1.221	1.247
TRINITY_DN43253_c0_g1_i5_orf1	glucose dehydrogenase [FAD, quinone]-like [Ostrinia furnacalis]	1	650	72.3	8.69	2	1	1	High	1	1.151	1.018	0.934	0.832	0.884	1.092	0.833	1.076	0.586	0.724	1.015	0.686	0.99	0.817
TRINITY_DN3638_c0_g1_i1_orf1	DNA replication licensing factor Mcm3 [Ostrinia furnacalis]	1	805	90.7	6.29	1	1	1	High	1	1.144	1.393	0.514	0.518	0.486	0.581	0.585	0.523	0.64	0.568	0.55	0.666	0.629	0.579
TRINITY_DN3918_c0_g1_i1_orf1	odorant binding protein 3 [Ostrinia furnacalis]	1	149	16.3	4.86	5	1	1	High	1	0.992	0.929	0.839	0.892	0.978	0.78	0.816	0.791	0.658	1.521	0.743	0.421	0.545	0.62
TRINITY_DN1274_c0_g1_i4_orf1	venom dipeptidyl peptidase 4-like [Ostrinia furnacalis]	1	743	83.9	5.85	1	1	1	High	1	1.006	0.905	0.669	0.826	0.658	0.79	0.861	0.97	0.984	0.846	0.831	1.76	1.555	1.378
TRINITY_DN82320_c0_g1_i2_orf1	glutathione S-transferase sigma3 [Glyphodes pyloalis]	1	64	7.2	9.28	13	1	1	High	1	0.867	1.054	0.129	0.095	0.17	0.194	0.243	0.189	0.242	0.27	0.324	0.225	0.177	0.212
TRINITY_DN8527_c0_g2_i1_orfp1	TRINITY_DN8527_c0_g2_i1_m.16937 TRINITY_DN8527_c0_g2::TRINITY_DN8527	1	53	6	4.64	21	1	1	High	1	1.069	1.069	1.738	1.662	1.375	1.866	1.852	1.718	1.842	1.661	1.719	0.894	0.874	1.119
TRINITY_DN22314_c0_g1_i2_orf1	phosphatidate cytidylyltransferase, photoreceptor-specific [Ostrinia furnacalis]	1	447	51.7	7.14	3	1	1	High	1	1.33	1.289	1.437	1.435	1.633	1.403	1.517	1.495	1.324	1.437	1.187	1.498	1.262	1.558
TRINITY_DN121156_c0_g2_i1_orf1	terminal nucleotidyltransferase 5C isoform X1 [Ostrinia furnacalis]	1	159	17.3	4.79	6	1	1	High	1	1.011	1.023	0.952	0.832	0.902	0.98	0.865	0.991	0.87	0.832	0.708	0.909	0.689	0.727
TRINITY_DN713_c0_g1_i4_orf1	periodic tryptophan protein 2 homolog isoform X1 [Ostrinia furnacalis]	1	918	102.3	6.35	1	1	1	High	1	1.003	1.018	0.168	0.182	0.325	0.229	0.177	0.273	0.165	0.16	0.23	0.254	0.27	0.212
TRINITY_DN8430_c0_g1_i1_orf1	transducin beta-like protein 3 [Ostrinia furnacalis]	1	792	88.8	6.74	1	1	1	High	1	1.202	1.102	0.818	0.865	0.977	0.764	0.923	0.893	1.012	1.055	0.95	0.876	0.698	0.916
TRINITY_DN6985_c0_g1_i5_orf1	LYR motif-containing protein 4B [Ostrinia furnacalis]	1	89	10.5	9.94	9	1	1	High	1	0.838	0.885	0.625	0.654	0.766	0.703	0.781	0.843	0.666	0.65	0.583	0.832	0.695	0.746
TRINITY_DN4116_c0_g1_i3_orf1	transmembrane protein 131 homolog [Ostrinia furnacalis]	1	621	66.6	9.03	1	1	1	High	1	0.926	0.931	0.692	0.819	0.64	0.514	0.604	0.578	0.475	0.485	0.429	0.813	0.781	0.681
TRINITY_DN9117_c0_g1_i1_orf1	spherulin-2A-like [Ostrinia furnacalis]	1	269	30	8.84	4	1	1	High	1	0.936	1.109	0.539	0.691	0.703	0.581	0.736	0.543	0.66	0.641	0.618	0.476	0.462	0.502
TRINITY_DN33272_c0_g1_i1_orf1	unnamed protein product, partial [Iphiclides podalirius]	1	445	47.8	5.33	4	1	1	High	1	0.927	0.936	1.619	1.497	1.436	1.801	1.921	1.632	1.947	1.983	1.997	2.752	2.392	3.048
TRINITY_DN32956_c0_g1_i4_orf1	inositol-trisphosphate 3-kinase A isoform X1 [Vanessa tameamea]	1	351	40.5	7.88	3	1	1	High	1	1.163	1.147	0.867	1.023	0.936	0.908	0.879	0.766	0.877	0.705	0.784	1.382	1.777	1.445
TRINITY_DN5211_c0_g1_i1_orf1	elongation of very long chain fatty acids protein AAEL008004-like [Ostrinia fur	1	269	32.2	9.39	4	1	1	High	1	1.052	1.089	0.663	0.678	0.71	0.561	0.8	0.595	0.675	0.565	0.603	1.515	1.308	1.495
TRINITY_DN21961_c0_g2_i5_orf1	hypothetical protein HW555_000844 [Spodoptera exigua]	1	197	22.5	6.54	4	1	1	High	1	1.028	0.971	0.908	0.89	1.012	0.785	1.02	1.181	0.824	0.759	0.812	0.807	0.814	1.046
TRINITY_DN53246_c0_g7_i1_orf1	E3 ubiquitin-protein ligase HUWE1 isoform X3 [Chelonus insularis]	1	715	81.4	5.94	1	1	1	High	1	0.966	1.053	0.741	0.53	0.682	0.923	0.815	1.011	0.961	0.828	0.535	0.769	0.81	0.708
TRINITY_DN5009_c0_g1_i2_orf1	GSCOCG00009487001-RA-CDS [Cotesia congregata]	1	193	22	11.4	4	2	1	High	1	1.061	0.988	0.655	0.665	0.816	0.607	0.561	0.569	0.516	0.355	0.497	0.526	0.563	0.506
TRINITY_DN5667_c0_g1_i4_orf1	spodomicin-like [Ostrinia furnacalis]	1	63	7.1	8.29	16	1	1	High	1	0.791	1.079	1.694	1.578	1.987	1.659	1.563	1.789	1.983	1.77	2.386	2.16	1.588	2.24

TRINITY_DN2815_c0_g1_i3_orf1	uncharacterized protein LOC114364075 [Ostrinia furnacalis]	1	313	35.8	8.88	3	1	1	High	1	0.964	0.838	0.155	0.149	0.241	0.188	0.244	0.206	0.181	0.174	0.185	0.203	0.24	0.172
TRINITY_DN19920_c1_g1_i2_orf1	probable ATP-dependent RNA helicase DDX10 [Ostrinia furnacalis]	1	815	92.9	7.78	2	1	1	High	1	0.998	1.041	0.656	0.717	0.652	0.606	0.694	0.626	0.598	0.779	0.735	0.611	0.559	0.701
TRINITY_DN94355_c0_g1_i2_orf1	uncharacterized protein LOC126369488 [Pectinophora gossypiella]	1	1589	179.7	8.48	0	1	1	High	1	1.019	0.942	0.15	0.17	0.202	0.053	0.073	0.076	0.073	0.079	0.072	0.097	0.076	0.098
TRINITY_DN3614_c0_g2_i1_orf1	PC4 and SFRS1-interacting protein isoform X4 [Galleria mellonella]	1	106	11.9	5.21	13	1	1	High	1	0.934	1.02	0.646	0.631	0.659	0.321	0.313	0.37	0.257	0.236	0.353	0.779	0.805	0.91
TRINITY_DN33485_c0_g1_i4_orf1	luciferin 4-monooxygenase-like [Ostrinia furnacalis]	1	543	59.5	6.76	3	1	1	High	1	1.046	1.223	0.783	0.868	1.059	0.713	0.735	0.723	0.769	0.926	0.804	0.918	1.015	1.123
TRINITY_DN8487_c0_g1_i4_orf1	RING finger protein 121 [Ostrinia furnacalis]	1	316	37.3	8.7	2	1	1	High	1	1.188	1.059	1.275	1.072	1.139	1.036	1.098	1.1	0.916	1.061	1.081	1.168	0.979	0.984
TRINITY_DN18009_c0_g1_i1_orf1	pre-mRNA-splicing factor ISY1 homolog [Ostrinia furnacalis]	1	265	30.7	5.31	5	1	1	High	1	1.049	1.132	1.118	1.083	1.212	1.102	0.922	0.925	0.921	0.752	0.906	0.621	0.595	0.542
TRINITY_DN962_c5_g1_i1_orf1	histone deacetylase 5 isoform X5 [Pectinophora gossypiella]	1	463	48.7	7.03	3	1	1	High	1	0.747	0.898	0.789	0.992	0.963	1.459	2.204	1.553	1.135	0.912	0.808	1.398	1.518	1.809
TRINITY_DN311_c0_g1_i4_orfp1	TRINITY_DN311_c0_g1_i4_m.65135 TRINITY_DN311_c0_g1::TRINITY_DN311_c0	1	125	13.8	5.33	6	1	1	High	1	0.966	0.979	0.09	0.074	0.172	0.235	0.226	0.216	0.264	0.188	0.266	0.205	0.174	0.18
TRINITY_DN1098_c1_g1_i4_orf1	lysozyme 10 [Ostrinia furnacalis]	1	182	21.1	9.2	5	1	1	High	1	1.037	1.271	1.59	1.684	1.346	2.426	1.874	2.062	1.922	2.066	1.741	2.404	1.988	2.192
TRINITY_DN24142_c0_g1_i1_orf1	arylalkylamine N-acetyltransferase [Chilo suppressalis]	1	260	29.3	6	3	1	1	High	1	1.012	1.016	0.547	0.64	0.597	0.546	0.625	0.572	0.464	0.444	0.386	0.524	0.505	0.338
TRINITY_DN15774_c0_g1_i3_orf1	uncharacterized protein LOC114366599 [Ostrinia furnacalis]	1	394	45.7	6.16	4	1	1	High	1	1.063	1.12	1.478	1.688	1.814	1.775	1.572	1.566	1.906	1.65	1.829	2.138	1.684	1.727
TRINITY_DN4955_c0_g1_i2_orf1	RNA pseudouridylate synthase domain-containing protein 1-like isoform X2 [(	1	272	30.7	7.62	5	1	1	High	1	0.933	1.046	1.285	1.385	1.253	1.546	1.521	1.453	1.296	1.838	1.086	0.988	0.998	1.226
TRINITY_DN82104_c0_g1_i5_orf1	uncharacterized protein LOC114349939 [Ostrinia furnacalis]	1	978	108	9.01	1	1	1	High	1	0.69	0.741	1.102	1.008	1.093	1.109	1.489	1.167	1.33	1.268	0.927	1.936	1.906	1.906
TRINITY_DN69691_c0_g2_i1_orf1	protein HGH1 homolog [Ostrinia furnacalis]	1	225	25.4	5.62	6	1	1	High	1	1.117	1.13	1.094	1.403	1.247	1.363	1.355	1.221	1.291	1.124	1.747	1.259	1.263	1.502
TRINITY_DN21125_c0_g1_i1_orf1	protein angel homolog 1 isoform X3 [Ostrinia furnacalis]	1	520	58.9	8.02	2	1	1	High	1	0.936	0.921	0.806	0.701	0.855	0.957	1.027	0.899	0.717	0.744	0.675	0.928	1.192	0.975
TRINITY_DN2649_c0_g1_i3_orf1	GPI inositol-deacylase isoform X1 [Ostrinia furnacalis]	1	1143	129.9	7.62	1	1	1	High	1	0.989	0.87	0.754	0.734	0.741	0.944	0.857	0.846	0.84	0.73	0.779	0.895	0.927	0.852
TRINITY_DN928_c0_g1_i3_orf1	fasciclin-2-like [Ostrinia furnacalis]	1	264	30	5.16	3	1	1	High	1	0.826	0.992	0.942	0.979	0.907	0.715	0.787	0.777	0.857	0.765	0.88	0.484	0.468	0.49
TRINITY_DN58872_c0_g1_i1_orfp1	cuticle protein 64-like [Pectinophora gossypiella]	1	87	8.7	9.03	9	1	1	High	1	0.835	0.716	0.744	0.848	0.67	0.761	1.05	0.858	0.856	1.168	0.809	1.671	2.021	1.769
TRINITY_DN6362_c0_g1_i4_orf1	sodium/hydrogen exchanger 7 isoform X4 [Galleria mellonella]	1	663	73.1	7.01	3	1	1	High	1	1.071	0.926	0.819	0.618	0.755	1.082	1.081	0.896	0.769	0.805	0.732	1.333	1.392	1.073
TRINITY_DN136467_c0_g1_i1_orf1	DNA-directed RNA polymerase II subunit RPB11 [Helicoverpa armigera]	1	117	13.5	5.83	7	1	1	High	1	0.924	1.042	1.135	1.019	1.156	0.971	1.105	1.003	0.778	0.796	1.316	1.111	0.998	1.162
TRINITY_DN4762_c0_g1_i2_orf1	ATPase family AAA domain-containing protein 1 isoform X2 [Ostrinia furnacali	1	383	43.3	6.81	2	1	1	High	1	1.128	0.949	0.196	0.244	0.314	0.34	0.286	0.188	0.191	0.2	0.223	0.524	0.475	0.488
TRINITY_DN110402_c0_g2_i1_orf1	apolipophorins-like [Ostrinia furnacalis]	1	104	12.3	7.99	8	1	1	High	1	0.938	0.974	0.998	1.077	1.285	1.179	1.42	1.226	1.295	1.566	1.414	1.943	1.936	2.013
TRINITY_DN10658_c0_g1_i1_orf1	DNA-directed RNA polymerase II subunit RPB9 [Ostrinia furnacalis]	1	126	14.8	7.3	7	1	1	High	1	0.895	0.853	0.449	0.396	0.619	0.599	0.706	0.614	0.483	0.616	0.596	0.505	0.824	0.758
TRINITY_DN1637_c0_g1_i5_orf1	ragulator complex protein LAMTOR3 homolog [Ostrinia furnacalis]	1	125	13.9	7.39	6	1	1	High	1	0.842	0.926	0.999	0.761	0.782	0.783	0.762	0.821	0.847	0.775	0.5	0.975	0.875	1.018
TRINITY_DN47723_c0_g1_i1_orf1	dnaJ homolog subfamily C member 21 [Ostrinia furnacalis]	1	607	69.6	6.13	1	1	1	High	1	1.3	1.024	0.507	0.62	0.562	0.518	0.527	0.556	0.628	0.506	0.589	0.871	0.999	0.92
TRINITY_DN6589_c0_g1_i2_orf1	hypothetical protein evm_004467 [Chilo suppressalis]	1	413	45.8	6.04	3	1	1	High	1	0.935	1.019	1.174	0.894	0.89	0.838	0.932	0.922	0.829	0.786	0.876	1.062	1.035	0.931
TRINITY_DN32700_c0_g1_i2_orf1	ribosomal protein S6 kinase 2 beta [Ostrinia furnacalis]	1	770	86.3	6.89	1	1	1	High	1	0.929	1.019	0.763	0.612	0.784	0.67	0.742	0.773	0.67	0.942	0.685	1.059	1.088	1.069
TRINITY_DN100_c0_g1_i9_orf1	uncharacterized protein LOC114353052 [Ostrinia furnacalis]	1	153	17.7	5.35	9	1	1	High	1	1.001	1.019	0.895	1.056	0.965	0.96	0.991	0.901	0.765	0.864	0.908	0.474	0.475	0.489
TRINITY_DN1199_c0_g1_i1_orf1	pupal cuticle protein 36a-like [Ostrinia furnacalis]	1	246	23.9	7.14	2	2	1	High	1	1.006	0.976	0.264	0.285	0.287	0.231	0.288	0.221	0.196	0.221	0.212	0.211	0.181	0.24
TRINITY_DN4465_c0_g1_i9_orf1	PREDICTED: nucleolar protein 6 [Amyelois transitella]	1	1120	127.2	7.05	1	1	1	High	1	1.016	1.043	0.788	1.007	0.998	0.96	0.91	0.748	0.858	0.799	0.953	0.824	0.788	0.993
TRINITY_DN1628_c0_g1_i1_orf1	uncharacterized protein LOC114363979 [Ostrinia furnacalis]	1	72	8.1	4.59	11	1	1	High	1	0.926	0.789	0.61	0.736	0.628	0.874	0.776	0.76	0.663	0.539	0.703	1.052	1.062	0.923
TRINITY_DN1066_c0_g1_i8_orf1	hypothetical protein evm_012420 [Chilo suppressalis]	1	448	47.8	8.53	2	1	1	High	1	0.89	1.096	1.293	1.132	1.078	0.822	0.5	0.837	0.731	0.63	0.682	1.542	1.51	1.211
TRINITY_DN16605_c0_g1_i3_orf1	unnamed protein product [Chrysodeixis includens]	1	544	60.5	7.56	1	1	1	High	1	0.929	1.044	0.799	0.803	0.836	1.047	1.05	0.768	0.914	0.847	0.87	1.378	1.211	1.272
TRINITY_DN30679_c0_g1_i5_orf1	replication factor C subunit 4 [Ostrinia furnacalis]	1	351	38.6	8.06	2	1	1	High	1	0.965	1.078	1.189	1.844	0.99	0.876	1.333	0.963	0.972	1.289	1.687	1.28	0.937	1.079
TRINITY_DN16482_c0_g1_i6_orf1	transmembrane protein 120 homolog isoform X2 [Ostrinia furnacalis]	1	403	47.7	9.72	2	1	1	High	1	0.912	1.001	0.498	0.538	0.496	0.47	0.439	0.467	0.445	0.349	0.457	0.517	0.548	0.48
TRINITY_DN1272_c1_g1_i4_orf1	E3 ubiquitin-protein transferase MAEA [Ostrinia furnacalis]	1	458	51.4	9.17	3	1	1	High	1	1.116	1.154	2.064	2.227	1.232	1.203	0.873	0.894	0.699	0.654	0.789	1.089	1.068	0.915
TRINITY_DN14904_c1_g2_i2_orf1	autophagy protein 12-like [Ostrinia furnacalis]	1	120	13.5	6.16	9	1	1	High	1	1.199	0.907	1.374	1.233	1.392	1.619	1.489	1.406	1.431	1.333	1.493	1.678	1.644	1.493
TRINITY_DN6071_c0_g1_i1_orf1	transcription initiation factor IIB isoform X1 [Manduca sexta]	1	315	34.3	8.27	4	1	1	High	1	0.875	0.868	0.862	0.874	0.856	0.629	0.753	0.736	0.728	0.663	0.82	0.882	0.855	0.911
TRINITY_DN14134_c0_g2_i3_orf1	anoctamin-8-like isoform X2 [Helicoverpa zea]	1	747	84.2	6.76	1	1	1	High	1	1.21	1.277	0.884	0.897	0.924	0.783	0.947	1.125	0.929	0.852	0.734	0.673	0.94	0.638
TRINITY_DN4589_c0_g2_i1_orf1	thymidylate kinase [Ostrinia furnacalis]	1	219	25	7.11	6	1	1	High	1	1.029	1.3	1.359	1.66	1.361	1.394	1.427	1.554	1.696	3.249	1.502	1.346	1.063	1.055
TRINITY_DN43576_c0_g1_i3_orf1	regulator of microtubule dynamics protein 1-like [Ostrinia furnacalis]	1	303	35.5	8.84	2	1	1	High	1	0.899	1.055	0.727	0.738	0.688	0.721	0.726	0.659	0.622	0.631	0.577	0.643	0.652	0.655
TRINITY_DN67193_c0_g1_i1_orf1	A-kinase anchor protein 14-like [Ostrinia furnacalis]	1	153	15.2	10.18	6	2	1	High	1	0.973	1.294	3.902	2.157	3.216	4.345	4.103	4.722	5.528	5.501	4.466	5.311	6.365	5.546
TRINITY_DN42854_c0_g3_i2_orf1	amyloid beta (A4) precursor-like protein 2, isoform CRA_b [Homo sapiens]	1	633	73.2	4.7	2	1	1	High	1	0.908	1.019	1.328	1.286	1.276	1.459	1.369	1.412	2.043	1.64	1.783	1.812	1.718	1.675
TRINITY_DN83295_c0_g1_i3_orf1	SSSX-APN4 [Ostrinia furnacalis]	1	315	35.8	5.95	3	1	1	High	1	0.915	0.914	0.462	0.486	0.491	0.517	0.44	0.42	0.461	0.496	0.517	0.356	0.395	0.305
TRINITY_DN2252_c0_g1_i4_orfp1	TRINITY_DN2252_c0_g1_i4_m.69997 TRINITY_DN2252_c0_g1::TRINITY_DN2252	1	167	19.1	5.39	5	1	1	High	1	1.028	1.105	0.608	0.8	0.407	0.715	0.638	0.409	0.44	0.54	0.516	0.938	0.885	0.769
TRINITY_DN23534_c0_g2_i2_orf1	armadillo repeat-containing protein 8-like [Maniola hyperantus]	1	424	46.1	6.77	2	1	1	High	1	1.008	0.854	0.675	0.723	0.671	0.774	0.801	0.807	0.954	0.869	0.764	1.01	0.771	0.917
TRINITY_DN51813_c0_g1_i1_orf1	uncharacterized protein LOC114350216 [Ostrinia furnacalis]	1	427	48.1	6.64	1	2	1	High	1	0.85	0.845	3.248	4.133	3.055	3.097	4.029	2.952	4.837	5.999	7.463	6.033	5.261	7.006
TRINITY_DN142652_c0_g1_i1_orf1	pre-mRNA-splicing factor RBM22 [Chelonus insularis]	1	403	45.3	8.54	2	1	1	High	1	1.17	1.312	0.509	0.668	0.536	0.43	0.607	0.436	0.336	0.289	0.364	0.634	0.56	0.639
TRINITY_DN5028_c0_g1_i11_orf1	NTF2-related export protein [Ostrinia furnacalis]	1	136	15.3	5.95	10	1	1	High	1	1.131	0.99	1.026	1.092	1.384	1.053	1.13	0.978	1.374	1.315	1.197	1.605	1.668	1.92
TRINITY_DN12951_c1_g2_i2_orf1	protein kinase C isoform X2 [Ostrinia furnacalis]	1	703	79.3	7.03	1	1	1	High	1	0.877	1.024	1.321	1.455	1.15	1.255	1.063	1.454	1.282	1.181	1.137	1.395	1.188	1.08
TRINITY_DN4741_c0_g1_i4_orf1	PREDICTED: 23 kDa integral membrane protein-like [Papilio xuthus]	1	247	26.8	5.95	4	1	1	High	1	1.086	1.022	0.921	1.11	1.288	0.622	0.731	1.008	0.624	0.659	0.633	0.844	0.939	0.923
TRINITY_DN109733_c0_g1_i1_orf1	uncharacterized protein LOC112452128 [Temnothorax curvispinosus]	1	332	37.9	8.79	2	2	1	High	1	1.148	0.947	0.102	0.332	0.252	0.309	0.327	0.333	0.24	0.222	0.238	0.161	0.34	0.301
TRINITY_DN9591_c0_g1_i1_orf1	probable 39S ribosomal protein L49, mitochondrial [Ostrinia furnacalis]	1	194	22.5	9.22	7	1	1	High	1	0.885	1.005	0.96	0.88	1.033	0.908	0.949	0.844	0.82	0.933	0.826	0.601	0.724	0.676
TRINITY_DN52296_c0_g1_i6_orf1	protein takeout-like [Ostrinia furnacalis]	1	174	19.5	8.59	3	1	1	High	1	0.948	0.88	0.431	0.529	0.518	0.311	0.415	0.339	0.344	0.265	0.312	0.437	0.376	0.39
TRINITY_DN320_c0_g1_i2_orf1	venom carboxylesterase-6-like [Ostrinia furnacalis]	1	623	69.7	5.92	4	1	1	High	1	0.829	0.969	1.169	0.843	0.852	0.877	1.069	1.121	1.002	1.663	0.901	0.788	0.615	0.937
TRINITY_DN22009_c0_g1_i1_orf1	armadillo repeat-containing protein 8-like [Ostrinia furnacalis]	1	178	19.2	5.6	7	1	1	High	1	1.02	1.139	1.034	1.078	1.084	1.245	1.281	1.079	1.038	0.89	0.997	1.099	1.162	1.041
TRINITY_DN8087_c0_g1_i9_orf1	cysteine-rich with EGF-like domain protein 2 isoform X1 [Ostrinia furnacalis]	1	385	42.5	5.76	2	1	1	High	1	0.887	0.876	0.196	0.233	0.302	0.237	0.233	0.253	0.303	0.365	0.288	0.401	0.443	0.365
TRINITY_DN58314_c0_g1_i1_orf1	alaserpin-like isoform X16 [Ostrinia furnacalis]	1	41	4.7	6.49	32	1	1	High	1	1.042	1.128	1.144	0.898	0.835	1.106	1.201	0.901	1.037	1.002	1.044	1.099	0.912	0.966
TRINITY_DN4636_c0_g3_i1_orf1	microtubule-associated serine/threonine-protein kinase 4 [Ostrinia furnacalis]	1	405	44.6	9.45	2	1	1	High	1	0.978	0.805	1.264	1.36	1.095	0.83	1.132	0.888	0.672	0.777	0.907	0.851	0.881	0.802
TRINITY_DN9274_c0_g1_i7_orf1	heme transporter hrg1-B-like [Ostrinia furnacalis]	1	151	17.4	8.6	6	2	1	High	1	0.957	0.882	0.975	0.996	1.057	0.847	0.926	0.89	1.029	0.978	0.972	0.929	1.105	0.869
TRINITY_DN2904_c0_g1_i4_orf1	ATP-dependent DNA/RNA helicase DHX36 isoform X1 [Ostrinia furnacalis]	1	1102	124.7	7.8	1	1	1	High	1	0.867	0.917	0.971	1.007	0.975	1.131	1.113	1.104	0.965	0.859	0.825	1.354	1.182	1.14
TRINITY_DN5910_c1_g1_i6_orf1	protein SAND [Ostrinia furnacalis]	1	558	62.8	6.32	1	1	1	High	1	1.053	0.846	0.787	1.042	0.934	0.995	1.021	1.466	1.171	0.897	1.102	1.449	1.268	1.209
TRINITY_DN15903_c0_g1_i2_orf1	proton-coupled amino acid transporter-like protein pathetic isoform X1 [Ostri	1	476	51.9	6.07	2	1	1	High	1	2.228	0.969	1.831	2.114	0.372	0.42	0.47	0.354	0.328	0.31	0.266	0.309	0.329	0.332
TRINITY_DN110523_c0_g2_i1_orf1	uncharacterized protein LOC107036393 [Diachasma alloeum]	1	449	49	7.03	3	1	1	High	1	1.101	0.969	1.197	1.245	1.422	0.752	0.876	0.757	0.929	1.052	0.883	1.996	2.028	1.787
TRINITY_DN19135_c0_g1_i1_orf1	ER membrane protein complex subunit 10 [Ostrinia furnacalis]	1	222	25.1	6.55	4	1	1	High	1	1.066	1.036	0.672	0.532	0.703	0.601	0.558	0.729	0.531	0.474	0.54	0.639	0.694	0.787
TRINITY_DN143895_c0_g1_i1_orf1	cathepsin L-like [Aphidius gifuensis]	1	342	38.3	6.46	2	1	1	High	1	0.834	1.013	5.558	4.445	4.143	6.675	5.86	6.115	9.535	8.381	9.108	7.773	7.336	7.346
TRINITY_DN2178_c0_g1_i1_orf1	carboxypeptidase B-like [Ostrinia furnacalis]	1	427	48.1	5.82	2	1	1	High	1	1.022	0.978	0.166	0.126	0.144	0.162	0.243	0.191	0.18	0.33	0.175	0.158	0.205	0.197
TRINITY_DN20717_c0_g1_i1_orf1	putative uncharacterized protein DDB_G0282133 isoform X1 [Ostrinia furnacali	1	46	5.2	5.19	17	1	1	High	1	1.1	1.082	2.395	2.763	2.82	1.822	1.709	2.054	1.79	1.424	1.893	4.271	4.554	4.785
TRINITY_DN60048_c0_g2_i1_orf1	facilitated trehalose transporter Tret1-like [Venturia canescens]	1	479	53.5	7.33	2	1	1	High	1	1.061	0.986	0.608	0.729	0.581	0.541	0.638	0.638	0.572	0.485	0.561	0.627	0.735	0.515
TRINITY_DN501_c1_g1_i1_orf1	sodium- and chloride-dependent GABA transporter ine isoform X1 [Ostrinia fu	1	725	80.8	7.4	1	1	1	High	1	1.025	0.961	0.409	0.391	0.474	0.434	0.395	0.393	0.337	0.322	0.388	0.372	0.432	0.414
TRINITY_DN1344_c0_g1_i1_orf1	ribosomal RNA small subunit methyltransferase NEP1 [Ostrinia furnacalis]	1	144	15.9	9.13	10	1	1	High	1	1.193	1.14	1.261	1.668	1.535	1.44	1.877	1.725	1.776	1.916	1.773	0.957	0.785	0.8
TRINITY_DN143833_c0_g1_i1_orf1	zinc finger protein-like 1 [Diachasma alloeum]	1	318	35.8	8.13	3	1	1	High	1	1.073	1.046	0.808	0.84	0.909	1.01	0.981	1.06	0.911	0.923	0.932	0.822	0.873	0.925
TRINITY_DN4246_c0_g2_i3_orf1	interferon-inducible double-stranded RNA-dependent protein kinase activato	1	91	9.8	9.83	13	1	1	High	1	0.831	0.564	0.668	0.858	1.027	1.175	1.077	1.333	0.954	1.149	0.791	0.953	0.924	0.653
TRINITY_DN43206_c0_g1_i6_orf1	unnamed protein product [Parnassius apollo]	1	567	63.1	6.67	2	1	1	High	1	1.134	1.319	1.281	1.35	1.431	1.213	1.109	1.239	1.327	1.21	1.032	1.243	1.27	1.306
TRINITY_DN1697_c0_g1_i1_orf1	mitogen-activated protein kinase-binding protein 1 [Ostrinia furnacalis]	1	1496	163.6	8.13	1	1	1	High	1	1.065	1.026	0.803	0.918	0.838	0.85	0.862	0.826	0.866	0.637	0.735	0.689	0.718	0.596
TRINITY_DN45836_c0_g1_i1_orf1	metallophosphoesterase 1 isoform X1 [Ostrinia furnacalis]	1	410	47.9	8.68	2	1	1	High	1	0.911	1.253	0.912	0.813	1.07	1.21	1.014	0.908	0.801	0.83	0.789	1.223	0.962	0.859
TRINITY_DN105749_c0_g1_i1_orf1	hypothetical protein evm_009828 [Chilo suppressalis]	1	123	14.2	8.68	6	1	1	High	1	1.271	1.237	1.131	1.465	1.507	1.676	1.986	1.694	1.397	1.805	1.157	1.526	1.381	1.699
TRINITY_DN27736_c0_g1_i4_orf1	nitric oxide synthase-interacting protein homolog [Ostrinia furnacalis]	1	290	32.4	8.95	5	2	1	High	1	1.049	1.187	1.099	1.075	0.923	0.878	1.118	1.013	0.931	1.025	1.006	1.125	1.123	1.031
TRINITY_DN59804_c0_g1_i1_orf1	DNA methyltransferase 1-associated protein 1 [Ostrinia furnacalis]	1	465	53.6	9.19	2	1	1	High	1	1.07	0.817	0.798	0.922	0.749	0.931	1.288	0.913	1.029	1.3	1.297	1.215	1.243	1.185
TRINITY_DN113353_c0_g1_i1_orf1	unnamed protein product [Parnassius apollo]	1	68	7.5	5.3	19	1	1	High	1	0.99	0.937	1.865	1.559	1.905	2.378	1.803	2.156	2.484	2.67	2.427	3.362	2.766	2.918
TRINITY_DN544_c0_g1_i4_orf1	E3 ubiquitin-protein ligase Siah1-like [Ostrinia furnacalis]	1	542	61.5	8.87	1	1	1	High	1	1.055	1.124	1.164	1.017	0.96	1.193	1.268	0.97	0.972	0.805	1.235	0.924	0.862	0.887
TRINITY_DN71917_c0_g3_i1_orf1	leucine-rich repeat and immunoglobulin-like domain-containing nogo recept	1	845	92.4	9.28	2	1	1	High	1	1.008	1.033	1.319	2.012	1.972	1.5	1.668	2.159	1.327	1.49	1.307	1.368	1.256	1.183
TRINITY_DN25997_c1_g1_i1_orf1	uncharacterized protein LOC114366342 [Ostrinia furnacalis]	1	464	51.9	8.76	2	1	1	High	1	1.112	0.966	0.641	0.739	0.69	0.692	0.503	0.569	0.664	0.622	0.68	0.722	0.796	0.736
TRINITY_DN13760_c1_g1_i1_orf1	pre-mRNA-processing factor 40 homolog A isoform X1 [Ostrinia furnacalis]	1	818	92.5	8.25	1	1	1	High	1	1.129	0.786	2.939	2.626	2.652	2.143	1.743	2.081	3.206	2.987	3.027	4.744	4.831	4.993
TRINITY_DN96557_c0_g1_i1_orf1	charged multivesicular body protein 4B [Phyllostomus discolor]	1	73	8	10.24	14	2	1	High	1	1.043	1.029	0.674	0.706	0.723	0.606	0.646	0.606	0.559	0.489	0.63	0.212	0.253	0.236
TRINITY_DN9400_c0_g1_i8_orf1	PREDICTED: monoacylglycerol lipase ABHD12-like [Amyelois transitella]	1	219	24.6	8.02	5	1	1	High	1	1.134	1.25	2.374	2.226	2.556	1.973	2.089	2.096	2.206	3.672	2.245	1.35	1.17	1.51
TRINITY_DN9420_c0_g1_i2_orf1	protein PFC0760c-like isoform X2 [Ostrinia furnacalis]	1	41	4.7	4.59	22	1	1	High	1	1.05	1.03	1.831	1.918	1.782	1.359	1.363	1.341	1.473	1.336	1.599	1.407	1.652	1.605
TRINITY_DN45227_c0_g1_i3_orf1	uncharacterized protein LOC114359191 [Ostrinia furnacalis]	1	104	11.2	8.46	8	1	1	High	1	1.134	1.067	0.425	0.486	0.502	0.377	0.466	0.39	0.389	0.338	0.444	0.529	0.467	0.412
TRINITY_DN1064_c0_g1_i3_orf1	uncharacterized protein LOC114356505 [Ostrinia furnacalis]	1	1718	199.5	6.04	1	1	1	High	1	0.977	0.995	1.041	1.106	1.008	1.016	1.016	0.977	0.934	1.023	1.014	1.024	0.949	1.053
TRINITY_DN11649_c0_g1_i4_orf1	ubiquitin carboxyl-terminal hydrolase 32-like, partial [Ostrinia furnacalis]	1	1032	114.7	7.56	1	1	1	High	1	0.997	1.047	0.97	0.955	1.013	1.506	1.64	1.674	1.287	1.2	1.245	0.872	0.892	0.826
TRINITY_DN6140_c0_g3_i3_orf1	CD63 antigen-like [Ostrinia furnacalis]	1	291	32.2	4.82	4	1	1	High	1	0.884	0.94	0.051	0.095	0.094	0.22	0.192	0.234	0.103	0.108	0.161	0.116	0.259	0.107
TRINITY_DN5126_c0_g2_i1_orf1	cytochrome P450 monooxygenase CYP4L47 [Ostrinia furnacalis]	1	115	13	9.42	7	1	1	High	1	0.958	0.991	0.548	0.567	0.644	2.237	1.957	2.236	0.604	0.576	0.599	0.588	0.455	0.428
TRINITY_DN22836_c0_g1_i5_orf1	AP-3 complex subunit beta-2 [Ostrinia furnacalis]	1	619	67.7	8.92	2	1	1	High	1	1.12	1.208	0.772	0.802	0.82	0.795	1.092	0.876	0.782	0.606	0.839	0.705	0.79	0.743

TRINITY_DN17651_c0_g1_i2_orf1	transmembrane protein 70 homolog, mitochondrial [Ostrinia furnacalis]	1	212	24.2	8.29	5	1	1	High	1	1.096	0.983	0.527	0.553	0.604	0.59	0.696	0.664	0.496	0.796	0.719	0.675	0.596	0.639
TRINITY_DN105359_c0_g2_i5_orf1	uncharacterized protein LOC114359499 [Ostrinia furnacalis]	1	353	39.7	5.17	4	1	1	High	1	1.091	0.966	0.734	0.771	1.175	0.592	0.689	0.675	0.465	0.309	0.55	1.168	1.124	1.004
TRINITY_DN40519_c0_g1_i4_orf1	tyrosine-protein phosphatase corkscrew [Ostrinia furnacalis]	1	636	73.3	7.27	1	1	1	High	1	0.877	0.972	0.777	0.872	0.88	0.88	0.885	0.711	0.817	1.036	1.071	0.999	0.939	0.872
TRINITY_DN15417_c0_g1_i6_orf1	copper chaperone for superoxide dismutase [Ostrinia furnacalis]	1	286	30.5	6.06	3	1	1	High	1	1.105	0.989	0.738	0.676	0.849	1.026	0.832	0.982	0.993	0.906	0.711	0.961	0.938	1.094
TRINITY_DN8674_c0_g2_i1_orf1	N(G),N(G)-dimethylarginine dimethylaminohydrolase 1 [Ostrinia furnacalis]	1	266	29.3	5.71	4	1	1	High	1	0.967	0.87	1.174	1.249	1.026	1.32	1.407	1.346	1.162	1.21	1.157	1.466	1.568	1.59
TRINITY_DN20960_c0_g1_i1_orf1	aldo-keto reductase AKR2E4-like [Ostrinia furnacalis]	1	327	37.3	6.54	3	1	1	High	1	1.115	1.227	1.111	1.166	1.124	1.273	1.222	1.206	1.042	1.011	0.864	1.025	0.957	0.886
TRINITY_DN36262_c0_g1_i1_orf1	trypsin, alkaline C-like [Maniola jurtina]	1	105	11.8	5.8	9	1	1	High	1	1.086	1.02	0.236	0.241	0.32	0.292	0.255	0.334	0.283	0.178	0.3	0.339	0.35	0.325
TRINITY_DN40911_c0_g1_i1_orf1	peroxisomal membrane protein PEX16 [Ostrinia furnacalis]	1	329	38.2	9.52	4	1	1	High	1	1.002	1.021	1.583	1.499	1.64	0.573	0.649	0.683	0.727	0.673	0.751	0.67	0.836	0.861
TRINITY_DN107035_c0_g1_i1_orf1	splicing factor 3A subunit 3 [Ostrinia furnacalis]	1	502	57.7	6.3	1	3	1	High	1	1.034	0.946	0.331	0.313	0.391	0.367	0.34	0.346	0.322	0.267	0.34	0.411	0.445	0.409
TRINITY_DN64196_c0_g1_i2_orf1	ceramide-1-phosphate transfer protein [Ostrinia furnacalis]	1	211	24.3	6.16	3	1	1	High	1	1.047	1.091	0.73	0.693	0.73	0.961	1.028	0.83	0.786	0.74	1.008	0.925	0.841	0.725
TRINITY_DN130051_c0_g1_i1_orf1	5-methyltetrahydropteroyltriglutamate--homocysteine S-methyltransferase-li	1	116	13	7.24	10	1	1	High	1	0.932	0.941	0.278	0.422	0.274	0.368	0.588	0.414	0.335	0.238	0.397	0.314	0.086	0.229
TRINITY_DN142657_c0_g1_i1_orf1	sorting and assembly machinery component 50 homolog [Diachasma alloeum]	1	486	53.8	8.32	2	1	1	High	1	1.007	1.006	3.523	3.288	3.432	4.866	4.449	5.363	8.167	6.551	6.011	4.316	4.485	3.487
TRINITY_DN2913_c0_g1_i5_orf1	aquaporin-11 isoform X1 [Spodoptera litura]	1	324	35.6	6.62	2	1	1	High	1	0.971	0.937	0.69	0.673	0.623	0.741	0.589	0.789	0.673	0.574	0.532	0.571	0.683	0.511
TRINITY_DN7477_c0_g1_i1_orf1	ethanolamine kinase [Ostrinia furnacalis]	1	354	41.2	5.12	3	1	1	High	1	0.928	0.955	1.362	1.357	1.129	0.99	1.327	1.295	1.092	1.006	0.875	1.141	0.852	1.032
TRINITY_DN3515_c0_g1_i3_orf1	SCY1-like protein 2 [Ostrinia furnacalis]	1	865	96.9	7.83	1	1	1	High	1	1.101	1.112	1.19	1.54	1.177	1.39	1.643	1.353	1.297	1.407	1.034	1.418	1.573	1.704
TRINITY_DN37658_c0_g1_i1_orf1	pre-mRNA-splicing factor ATP-dependent RNA helicase DHX16 [Ostrinia furna	1	886	102.3	7.03	1	1	1	High	1	1.021	0.845	0.761	0.786	0.901	0.938	0.907	0.867	1.01	0.891	0.876	0.838	0.749	0.892
TRINITY_DN467_c9_g1_i2_orf1	band 4.1-like protein 5 [Ostrinia furnacalis]	1	671	76.1	8.46	1	1	1	High	1	0.999	1.034	0.983	0.851	1.055	0.625	0.723	0.889	0.88	0.696	0.824	0.893	0.778	0.881
TRINITY_DN2061_c0_g1_i3_orf1	uncharacterized protein LOC114357318 isoform X1 [Ostrinia furnacalis]	1	2334	255.1	6.39	1	1	1	High	1	1.105	1.196	0.812	0.882	0.887	1.277	0.786	0.939	0.836	0.757	0.979	1.416	1.672	1.242
TRINITY_DN64472_c0_g2_i1_orf1	repressed by EFG1 protein 1-like isoform X3 [Ostrinia furnacalis]	1	190	21.3	8.07	3	1	1	High	1	0.787	0.989	0.389	0.583	0.507	0.579	0.617	0.499	0.397	0.372	0.42	0.588	0.372	0.259
TRINITY_DN6572_c0_g1_i2_orf1	zinc finger protein 330 homolog [Ostrinia furnacalis]	1	311	34.4	6.1	3	1	1	High	1	1.237	1.131	0.648	0.822	0.681	0.63	0.553	0.659	0.537	0.537	0.603	0.916	0.831	0.764
TRINITY_DN8139_c0_g1_i2_orf1	synaptic vesicle glycoprotein 2B-like [Galleria mellonella]	1	531	58.2	6.44	2	1	1	High	1	0.957	1.542	2.249	1.624	2.218	2.171	1.85	2.681	2.135	1.593	1.558	1.78	1.603	2.43
TRINITY_DN8352_c0_g1_i3_orf1	TRPL translocation defect protein 14 isoform X1 [Ostrinia furnacalis]	1	506	57.6	8.9	3	1	1	High	1	0.916	0.961	0.643	0.798	0.636	0.718	0.692	0.788	0.843	0.747	0.722	0.674	0.634	0.602
TRINITY_DN21559_c0_g1_i2_orf1	protein bicaudal D [Ostrinia furnacalis]	1	239	27.1	4.74	5	1	1	High	1	1.138	1.172	1.024	0.697	0.81	0.796	0.819	0.885	0.747	0.702	0.709	1.163	0.899	1.021
TRINITY_DN3504_c0_g1_i3_orfp2	TRINITY_DN3504_c0_g1_i3_m.43947 TRINITY_DN3504_c0_g1::TRINITY_DN3504	1	207	22.4	5.54	3	1	1	High	1	1.165	1.039	0.634	0.598	0.548	0.64	0.554	0.53	0.51	0.291	0.439	0.225	0.263	0.18
TRINITY_DN26186_c0_g1_i7_orf1	sodium- and chloride-dependent glycine transporter 1-like [Ostrinia furnacalis	1	642	71.2	5.22	2	1	1	High	1	0.978	1.072	0.239	0.235	0.302	0.349	0.337	0.549	0.303	0.288	0.29	0.43	0.449	0.452
TRINITY_DN56910_c0_g2_i1_orf1	mitochondrial ribonuclease P protein 1 homolog [Ostrinia furnacalis]	1	411	48.8	9.45	3	1	1	High	1	0.868	0.799	0.477	0.459	0.536	0.416	0.521	0.421	0.39	0.455	0.498	0.573	0.521	0.499
TRINITY_DN122786_c0_g2_i1_orf1	glucose dehydrogenase [FAD, quinone]-like [Ostrinia furnacalis]	1	130	13.3	4.42	8	1	1	High	1	0.931	0.975	0.559	0.466	0.806	0.591	0.494	0.526	0.54	0.616	0.501	0.589	0.536	0.575
TRINITY_DN3106_c0_g1_i6_orf1	major facilitator superfamily domain-containing protein 10 isoform X2 [Ostrini	1	479	51.8	9.57	2	1	1	High	1	1.193	0.842	1.176	0.738	1.217	1.061	0.831	1.07	1.007	0.862	0.804	0.838	1.058	0.78
TRINITY_DN74069_c0_g1_i1_orf1	unnamed protein product [Parnassius apollo]	1	95	10.1	7.46	9	1	1	High	1	1.144	1.383	0.834	1.061	0.807	1.013	0.929	1.142	1.054	0.833	0.903	1.713	1.565	1.438
TRINITY_DN298_c0_g1_i4_orf1	luc7-like protein 3 isoform X1 [Ostrinia furnacalis]	1	373	44.5	8.15	3	1	1	High	1	1.079	1.005	0.614	0.685	0.647	0.513	0.557	0.576	0.589	0.518	0.584	0.811	0.853	0.818
TRINITY_DN110132_c0_g1_i1_orf1	uncharacterized protein LOC114361588 isoform X14 [Ostrinia furnacalis]	1	75	8.1	9.44	15	3	1	High	1	1.073	1.032	0.865	0.993	0.983	1.008	0.937	1.007	0.813	0.708	0.952	0.779	0.726	0.79
TRINITY_DN52864_c0_g1_i1_orf1	odorant binding protein 18 [Conogethes pinicolalis]	1	184	20.9	7.25	5	1	1	High	1	0.868	0.858	1.013	0.845	1.028	1.178	1.092	0.994	1.127	1.336	1.18	1.319	1.662	1.558
TRINITY_DN12873_c0_g2_i1_orf1	proteoglycan 4-like [Ostrinia furnacalis]	1	223	25	4.42	4	1	1	High	1	1.154	1.205	1.785	1.641	1.602	1.912	1.561	1.743	1.927	1.981	1.444	1.263	1.142	1.445
TRINITY_DN1604_c0_g1_i4_orf1	ubiquitin-conjugating enzyme E2 S [Ostrinia furnacalis]	1	220	24.2	7.83	5	1	1	High	1	0.942	1.05	0.875	0.823	0.921	1.141	1.178	1.046	1.122	0.954	1.128	1.32	1.384	1.447
TRINITY_DN647_c4_g1_i1_orf1	coiled-coil domain-containing protein 51-like isoform X2 [Ostrinia furnacalis]	1	363	41.4	7.23	2	1	1	High	1	1.046	1.036	0.718	0.732	0.8	0.69	0.658	0.692	0.643	0.6	0.649	0.765	0.796	0.673
TRINITY_DN12951_c1_g1_i5_orf1	ADP-ribosylation factor-like protein 2 isoform X1 [Hyposmocoma kahamanoa]	1	184	20.9	7.99	7	1	1	High	1	1.23	1.14	0.91	1.139	1.055	0.946	1.18	1.027	0.895	0.947	0.859	1.116	1.185	1.159
TRINITY_DN108433_c0_g1_i1_orf1	alpha-2-macroglobulin receptor-associated protein [Diachasma alloeum]	1	380	43.9	7.58	2	1	1	High	1	0.839	0.83	1.404	1.427	1.464	1.375	1.54	1.57	1.481	1.747	1.612	1.819	1.792	1.717
TRINITY_DN4345_c0_g1_i9_orf1	uncharacterized protein LOC114357127 [Ostrinia furnacalis]	1	810	92.7	9.64	2	1	1	High	1	0.95	0.994	0.637	0.697	0.64	0.448	0.357	0.491	0.325	0.322	0.343	0.59	0.661	0.567
TRINITY_DN22577_c0_g1_i2_orf1	probable beta-hexosaminidase fdl isoform X1 [Ostrinia furnacalis]	1	574	63.8	6.81	2	1	1	High	1	0.919	0.986	0.852	1.064	0.877	0.864	1.01	0.903	1.01	0.748	0.885	1.202	1.167	1.129
TRINITY_DN145647_c0_g1_i1_orf1	PREDICTED: U6 snRNA-associated Sm-like protein LSm5 isoform X1 [Fopius ar	1	96	10.5	4.81	6	1	1	High	1	1.022	1.019	0.344	0.339	0.354	0.31	0.415	0.423	0.364	0.36	0.334	0.4	0.414	0.424
TRINITY_DN1786_c0_g1_i11_orf1	ATP-binding cassette sub-family A member 1-like [Ostrinia furnacalis]	1	1652	183.2	7.4	1	1	1	High	1	0.963	0.951	1.563	1.332	1.515	1.515	1.419	1.459	1.393	1.995	1.392	1.4	1.437	1.474
TRINITY_DN49956_c0_g1_i1_orf1	UPF0585 protein CG18661 [Ostrinia furnacalis]	1	228	25.7	5.05	6	1	1	High	1	0.751	0.981	1.453	1.402	1.529	1.609	1.449	1.426	1.532	2.191	1.28	1.419	1.2	1.452
TRINITY_DN5655_c0_g1_i2_orf1	uncharacterized protein LOC114359603 [Ostrinia furnacalis]	1	97	10.8	9.92	8	1	1	High	1	1.147	1.121	2.48	2.476	2.402	3.086	2.701	2.91	2.702	2.398	2.686	4.099	4.516	3.799
TRINITY_DN7735_c1_g1_i1_orf1	cuticular protein CPH [Spodoptera litura]	1	154	15.2	10.11	5	1	1	High	1	0.939	1.008	0.821	0.985	0.919	0.966	0.943	1.007	0.871	0.744	0.827	2.284	2.103	1.75
TRINITY_DN16385_c0_g1_i4_orf1	cytosolic Fe-S cluster assembly factor NUBP1 homolog [Ostrinia furnacalis]	1	264	27.6	5.4	3	1	1	High	1	1.07	1.143	0.79	0.882	0.946	0.907	0.824	0.998	0.845	0.728	0.668	0.934	0.771	0.913
TRINITY_DN30498_c0_g1_i3_orf1	lipase 3-like [Ostrinia furnacalis]	1	495	56.9	6.07	3	1	1	High	1	0.885	1.186	1.894	1.681	1.429	1.67	1.397	1.512	2.161	2.634	1.82	2.162	2.024	2.164
TRINITY_DN1416_c0_g2_i1_orf1	uncharacterized protein LOC114352565 [Ostrinia furnacalis]	1	204	23.7	8.69	3	1	1	High	1	0.937	1.159	0.665	0.728	0.772	1.057	1.03	1.058	0.828	0.639	0.644	0.618	0.793	0.673
TRINITY_DN13530_c0_g1_i1_orf1	unnamed protein product [Euphydryas editha]	1	571	63.4	9.11	2	1	1	High	1	1.077	1.084	0.517	0.511	0.567	0.476	0.573	0.546	0.506	0.452	0.415	0.529	0.523	0.467
TRINITY_DN3707_c0_g1_i1_orf1	protein FAM160B1-like isoform X1 [Ostrinia furnacalis]	1	817	93.1	5.6	1	1	1	High	1	1.069	1.037	3.047	2.999	2.736	3.251	2.966	3.598	3.998	3.395	4.01	4.549	4.098	4.157
TRINITY_DN30233_c0_g1_i2_orf1	39S ribosomal protein L10, mitochondrial [Ostrinia furnacalis]	1	246	27.9	9.79	2	2	1	High	1	0.931	0.941	0.364	0.409	0.399	0.382	0.477	0.431	0.284	0.317	0.353	0.441	0.419	0.434
TRINITY_DN31486_c0_g1_i1_orf1	transcription initiation factor TFIID subunit 3 [Ostrinia furnacalis]	1	1149	127.9	9.64	1	1	1	High	1	0.896	0.948	0.916	1.1	1.044	1.086	0.982	0.865	0.756	0.961	0.887	1.188	0.999	0.866
TRINITY_DN41602_c0_g3_i1_orf1	CCR4-NOT transcription complex subunit 3 [Cotesia glomerata]	1	774	87.1	6.73	1	1	1	High	1	1.097	1.049	0.653	0.673	0.732	0.581	0.611	0.648	0.636	0.989	0.611	0.793	0.742	0.725
TRINITY_DN33024_c0_g1_i1_orf1	hypothetical protein evm_000959 [Chilo suppressalis]	1	79	8.7	4.91	14	1	1	High	1	0.98	1.083	0.858	0.91	0.93	1.185	1.102	1.009	0.706	0.646	0.622	0.794	1.088	0.817
TRINITY_DN59965_c0_g4_i1_orf1	TKT protein, partial [Homo sapiens]	1	409	45.1	6.84	4	1	1	High	1	1.593	1.226	1.855	2.156	2.069	3.936	2.27	4.158	4.655	3.405	7.609	3.285	3.542	2.269
TRINITY_DN1633_c0_g1_i1_orf1	collagen alpha-2(I) chain-like isoform X1 [Ostrinia furnacalis]	1	445	46.3	8.62	2	1	1	High	1	0.926	0.844	0.491	0.508	0.517	0.511	0.631	0.495	0.683	0.419	0.501	0.512	0.474	0.643
TRINITY_DN23926_c0_g1_i4_orf1	programmed cell death protein 10 [Ostrinia furnacalis]	1	209	23.5	9.01	5	1	1	High	1	0.979	0.965	0.564	0.552	0.586	0.536	0.546	0.63	0.382	0.368	0.39	0.738	0.682	0.824
TRINITY_DN36899_c0_g1_i1_orf1	glucose dehydrogenase [FAD, quinone]-like [Ostrinia furnacalis]	1	623	70	7.08	2	1	1	High	1	1.044	1.133	1.579	1.61	1.545	1.588	1.514	1.406	1.285	1.097	1.468	2.492	2.367	2.136
TRINITY_DN110400_c0_g1_i1_orf1	DNA replication factor Cdt1 [Chelonus insularis]	1	725	82.5	9.66	1	1	1	High	1	1.084	1.166	0.874	1.425	1.41	2.162	0.683	0.493	0.52	1.181	0.464	0.406	0.552	0.65
TRINITY_DN9400_c0_g1_i1_orf1	lysophosphatidylserine lipase ABHD12 isoform X2 [Maniola hyperantus]	1	263	29.8	9.31	3	1	1	High	1	1.016	1.063	0.884	0.817	0.797	1.753	1.829	1.823	1.387	1.131	1.475	0.869	0.85	1.013
TRINITY_DN38106_c0_g1_i6_orf1	unnamed protein product [Pieris macdunnoughi]	1	431	47.5	7.33	2	1	1	High	1	1.069	1.339	1.772	2.195	2.002	1.319	1.255	1.196	1.015	1.165	0.996	1.736	1.865	1.628
TRINITY_DN2912_c0_g1_i1_orf1	uncharacterized protein LOC114352866 [Ostrinia furnacalis]	1	435	48.8	8.12	3	1	1	High	1	1.231	1.02	0.924	1.415	1.335	1.673	0.925	1.423	1.646	1.948	1.31	2.533	3.551	1.731
TRINITY_DN8747_c0_g1_i2_orf1	lipopolysaccharide-induced tumor necrosis factor-alpha factor homolog [Ostri	1	111	12.2	7.88	6	1	1	High	1	0.885	0.966	1.78	1.668	1.659	1.573	1.658	1.626	1.808	1.391	1.753	1.415	1.428	1.373
TRINITY_DN781_c0_g1_i7_orf1	uncharacterized protein LOC114356786 [Ostrinia furnacalis]	1	371	43.3	4.92	3	1	1	High	1	0.819	1.077	1.198	1.051	0.942	1.328	1.372	1.242	1.365	1.863	1.423	1.276	1.265	1.355
TRINITY_DN44089_c0_g5_i1_orf1	nuclear pore complex protein Nup160 homolog isoform X1 [Ostrinia furnacalis	1	796	85.7	6.61	1	1	1	High	1	0.941	1.202	1.06	0.933	1.145	1.014	0.918	0.975	0.762	0.983	0.732	0.852	0.917	0.852
TRINITY_DN2455_c0_g1_i12_orf1	LOW QUALITY PROTEIN: uncharacterized protein LOC114361878 [Ostrinia furr	1	1072	119.8	8.7	1	1	1	High	1	1.226	0.974	0.87	1.334	1.214	1.234	1.054	0.929	1.252	1.161	1.365	1.405	1.676	1.287
TRINITY_DN13711_c0_g1_i1_orf1	putative nuclease HARBI1 [Myzus persicae]	1	386	44.4	7.96	2	1	1	High	1	0.877	1.147	1.175	1.252	1.296	1.401	1.335	1.232	1.331	1.438	1.58	1.082	0.959	0.952
TRINITY_DN5818_c1_g1_i2_orf1	unnamed protein product [Chrysodeixis includens]	1	390	42.7	5.22	2	1	1	High	1	0.976	1.01	0.71	0.638	0.671	0.456	0.689	0.553	0.565	0.499	0.663	0.714	0.735	0.703
TRINITY_DN2532_c0_g3_i1_orf1	hypothetical protein O3G_MSEX013489 [Manduca sexta]	1	160	18.9	6.98	6	1	1	High	1	0.836	1	0.907	0.815	0.94	0.797	0.764	0.755	0.797	1.078	0.805	0.874	0.798	0.933
TRINITY_DN82628_c0_g1_i2_orf1	ORF type:internal len:148 hit:XP_028162129.1 TRINITY_DN82628_c0_g1_i2:3-44	1	148	16.4	5.1	9	1	1	High	1	0.881	0.985	1.17	1.205	1.111	1.055	1.064	1.098	0.876	0.897	1.122	0.467	0.439	0.57
TRINITY_DN46625_c0_g1_i1_orf1	ferritin subunit isoform X1 [Belonocnema kinseyi]	1	215	24.1	5.3	4	2	1	High	1	0.945	1.016	1.946	1.371	1.805	1.821	2.101	2.021	3.703	3.229	2.224	5.03	4.635	5.224
TRINITY_DN11868_c0_g1_i2_orf1	uncharacterized protein LOC114361308 [Ostrinia furnacalis]	1	514	57.3	6.77	1	1	1	High	1	0.898	1.044	1.842	1.864	1.804	1.499	1.418	1.586	1.266	1.046	1.355	1.861	1.997	1.78
TRINITY_DN6466_c0_g1_i5_orf1	mitochondrial coenzyme A transporter SLC25A42 [Ostrinia furnacalis]	1	293	32.1	10.18	3	1	1	High	1	0.944	1.036	0.888	0.865	0.822	1	1.013	0.868	0.847	1.172	1.143	0.962	0.975	1.02
TRINITY_DN2984_c0_g1_i3_orf1	connectin-like [Ostrinia furnacalis]	1	623	70.1	6.54	1	1	1	High	1	0.939	0.976	0.724	0.86	0.821	0.726	0.783	0.825	0.79	0.701	0.755	0.982	0.995	0.951
TRINITY_DN3045_c0_g1_i7_orf1	hypothetical protein evm_007836 [Chilo suppressalis]	1	3092	344.5	8.22	0	1	1	High	1	0.991	1.062	0.832	0.843	0.873	0.765	0.607	0.33	0.447	0.591	0.54	0.936	0.679	0.62
TRINITY_DN4635_c0_g1_i4_orf1	putative salivary secreted peptide [Operophtera brumata]	1	109	12.4	9.19	6	1	1	High	1	0.79	0.875	0.894	1.177	0.954	0.986	1.547	0.928	1.2	1.278	1.249	1.465	1.421	1.726
TRINITY_DN37696_c0_g1_i3_orf1	E3 ubiquitin-protein ligase TM129 [Ostrinia furnacalis]	1	365	40.8	7.64	2	1	1	High	1	0.754	0.804	0.657	0.739	0.709	0.635	0.637	0.674	0.788	0.827	0.671	0.686	0.695	0.76
TRINITY_DN23004_c0_g1_i1_orf1	uncharacterized protein LOC114365313 [Ostrinia furnacalis]	1	568	66.1	7.66	2	1	1	High	1	1.083	1.068	0.702	0.741	0.714	0.662	0.757	0.661	0.539	0.543	0.555	0.583	0.619	0.516
TRINITY_DN69691_c0_g1_i1_orf1	protein HGH1 homolog [Ostrinia furnacalis]	1	187	22	4.92	4	1	1	Medium	1	1.035	0.994	1.177	1.173	1.152	1.012	1.169	1.541	1.413	3.491	0.964	1.248	1.337	1.692
TRINITY_DN6027_c0_g1_i13_orf1	5-demethoxyubiquinone hydroxylase, mitochondrial [Ostrinia furnacalis]	1	131	14.7	10.87	5	1	1	Medium	1	0.824	0.947	0.678	0.767	0.746	0.648	0.65	0.778	0.849	0.677	0.783	0.548	0.529	0.552
TRINITY_DN11375_c0_g1_i6_orf1	uncharacterized protein LOC114363514 isoform X2 [Ostrinia furnacalis]	1	662	72.2	4.94	1	1	1	Medium	1	0.891	1.086	0.717	0.498	0.445	0.758	0.63	0.6	0.542	0.459	0.736	1.18	0.873	0.927
TRINITY_DN5444_c0_g1_i1_orfp1	TRINITY_DN5444_c0_g1_i1_m.14077 TRINITY_DN5444_c0_g1::TRINITY_DN5444	1	89	9.9	4.92	8	1	1	Medium	1	0.896	0.927	3.254	2.849	2.843	3.156	3.505	2.929	3.93	3.441	3.557	6.953	8.205	7.113
TRINITY_DN23354_c0_g1_i7_orf1	TBC1 domain family member 20 [Ostrinia furnacalis]	1	430	49.4	5.91	1	1	1	Medium	1	1.095	1.249	1.212	1.257	1.56	1.238	1.721	1.529	1.584	2.527	4.231	2.726	1.856	2.9
TRINITY_DN8480_c0_g1_i1_orf1	lysosomal Pro-X carboxypeptidase [Ostrinia furnacalis]	1	466	53.1	6.76	2	1	1	Medium	1	1.111	0.913	1.639	1.771	1.929	1.485	1.804	1.553	1.569	1.517	1.047	2.093	2.249	2.359
TRINITY_DN80547_c0_g1_i5_orf1	EKC/KEOPS complex subunit Tprkb-like [Ostrinia furnacalis]	1	200	23.1	7.71	4	1	1	Medium	1	0.983	0.893	1.034	0.987	1.073	1.136	1.235	1.097	1.028	0.964	1.08	0.764	0.942	1.009
TRINITY_DN14905_c0_g2_i2_orf1	hypothetical protein evm_002970 [Chilo suppressalis]	1	161	17.2	9.41	4	1	1	Medium	1	0.948	1.104	1.009	1.249	1.047	0.996	1.007	0.926	0.982	0.964	1.131	1.373	1.534	1.151
TRINITY_DN17247_c0_g1_i14_orf1	uncharacterized protein LOC114356308 [Ostrinia furnacalis]	1	213	24.8	7.74	5	1	1	Medium	1	1.206	1.005	2.854	3.582	3.895	2.988	3.727	3.365	2.518	2.443	2.148	2.953	2.271	2.463
TRINITY_DN6125_c0_g1_i2_orf1	uncharacterized protein LOC114350989 [Ostrinia furnacalis]	1	1687	188.5	6.13	0	1	1	Medium	1	1.049	1.115	0.943	0.769	0.772	0.76	0.629	0.663	0.653	0.674	0.98	0.78	0.856	0.819
TRINITY_DN6130_c0_g1_i6_orf1	tryptophan--tRNA ligase, mitochondrial [Ostrinia furnacalis]	1	392	43.4	8.92	2	1	1	Medium	1	1.148	1.154	0.732	0.881	1.056	0.826	0.957	0.992	0.832	0.823	0.921	1.189	1.043	1.231
TRINITY_DN162_c0_g1_i4_orf1	ABC transporter G family member 23 isoform X1 [Ostrinia furnacalis]	1	793	88.2	6.42	2	1	1	Medium	1	0.896	1.011	0.936	0.938	0.825	0.939	0.787	0.898	0.765	0.875	0.99	1.198	1.188	1.281
TRINITY_DN787_c0_g1_i7_orf1	YLP motif-containing protein 1-like isoform X1 [Ostrinia furnacalis]	1	1197	137.9	6.38	1	1	1	Medium	1	0.955	0.865	0.436	0.418	0.527	0.491	0.559	0.484	0.492	0.435	0.373	0.67	0.708	0.705
TRINITY_DN122170_c0_g1_i2_orfp	TRINITY_DN122170_c0_g1_i2_m.81408 TRINITY_DN122170_c0_g1::TRINITY_DN	1	86	9	7.12	12	1	1	Medium	1	1.13	1.191	0.098	0.281	0.202	0.164	0.212	0.184	0.135	0.155	0.208	0.184	0.167	0.161
TRINITY_DN2403_c0_g1_i3_orf1	FAD-dependent oxidoreductase domain-containing protein 1 [Ostrinia furnaca	1	471	53.1	6.38	2	1	1	Medium	1	0.937	0.913	0.768	0.762	0.732	0.676	0.747	0.653	0.73	0.765	0.517	0.976	0.943	0.839
TRINITY_DN90582_c0_g1_i4_orf1	thiolase 1 [Heliothis subflexa]	1	32	3.3	10.68	19	1	1	Medium	1	0.906	0.868	0.981	0.928	1.031	0.947	0.868	1.062	0.983	0.725	0.602	0.781	0.935	0.865
TRINITY_DN21856_c0_g1_i1_orf1	anti-lipopolysaccharide factor 3 [Eriocheir sinensis]	1	123	14	8.84	11	1	1	Medium	1	1.05	1.088	1.528	1.45	1.518	1.415	1.372	1.396	1.309	1.378	1.411	1.496	1.598	1.484

TRINITY_DN74116_c0_g1_i2_orf1	venom protease [Galleria mellonella]	1	50	5.8	8.31	30	1	1	Medium	1	0.677	0.788	0.929	0.941	0.944	0.861	0.847	0.994	0.896	1.43	0.967	0.834	0.626	0.803
TRINITY_DN25960_c0_g1_i1_orf1	protein mini spindles [Ostrinia furnacalis]	1	91	9.6	8.1	8	1	1	Medium	1	1.028	0.949	0.821	0.818	0.87	0.757	0.84	0.89	0.706	0.685	0.683	0.686	0.75	0.632
TRINITY_DN26650_c0_g1_i1_orfp1	TRINITY_DN26650_c0_g1_i1_m.72504 TRINITY_DN26650_c0_g1::TRINITY_DN26	1	71	8.2	9.64	13	1	1	Medium	1	0.91	1.135	0.76	0.646	0.728	0.542	0.633	0.719	0.649	0.667	0.593	0.58	0.604	0.71
TRINITY_DN11566_c2_g1_i2_orf1	transmembrane protein 18 isoform X1 [Ostrinia furnacalis]	1	147	17.4	7.99	5	1	1	Medium	1	0.911	0.684	0.771	0.747	0.829	0.853	0.777	0.93	0.753	0.765	0.69	0.883	0.856	0.915
TRINITY_DN45962_c1_g1_i2_orf1	protein shifted isoform X1 [Ostrinia furnacalis]	1	371	40.8	8.62	4	1	1	Medium	1	1.309	1.122	1.335	1.465	0.934	0.756	1.735	0.944	1.633	1.221	1.499	0.927	1.143	0.829
TRINITY_DN2367_c1_g1_i20_orf1	RNA exonuclease 4-like [Ostrinia furnacalis]	1	137	16	4.7	6	1	1	Medium	1	1.408	1.256	1.679	2.109	1.455	1.346	1.139	1.477	0.624	0.836	1.305	1.777	1.768	1.627
TRINITY_DN30150_c0_g1_i7_orf1	unnamed protein product [Chrysodeixis includens]	1	1239	140.7	6.02	1	1	1	Medium	1	0.81	1.035	0.742	0.715	0.638	0.677	0.66	0.715	0.853	0.855	0.621	0.944	0.978	0.75
TRINITY_DN670_c0_g1_i3_orf1	myrosinase 1-like [Ostrinia furnacalis]	1	504	57.5	5.62	2	1	1	Medium	1	1.124	1.097	0.845	0.791	0.784	0.776	0.808	0.943	0.884	0.664	1.036	0.97	1.011	1.027
TRINITY_DN8482_c0_g1_i4_orf1	BET1 homolog [Ostrinia furnacalis]	1	112	13.1	8.4	9	1	1	Medium	1	0.754	0.718	0.506	0.819	2.354	7.084	0.295	5.736	4.255	0.414	0.32	0.672	0.644	1.891
TRINITY_DN23582_c0_g1_i1_orf1	unnamed protein product [Diatraea saccharalis]	1	252	26.5	4.26	3	2	1	Medium	1	0.94	0.916	0.696	0.682	0.774	0.749	0.801	0.745	1.123	1.068	1.141	1.708	1.775	1.762
TRINITY_DN1897_c0_g2_i4_orf1	phenoloxidase-activating factor 2-like [Hyposmocoma kahamanoa]	1	129	13.9	4.49	7	1	1	Medium	1	1.013	0.896	1.306	1.482	1.397	3.95	3.015	2.691	1.575	1.123	1.685	2.559	2.893	2.219
TRINITY_DN16493_c0_g1_i2_orf1	zinc finger E-box-binding homeobox 2-like [Ostrinia furnacalis]	1	429	51.4	8.82	3	1	1	Medium	1	0.747	1.133	0.79	0.781	1.147	0.888	0.963	1.254	1.288	1.061	0.981	0.812	1.05	0.673
TRINITY_DN22430_c0_g3_i1_orf1	V-type proton ATPase 16 kDa proteolipid subunit c [Homo sapiens]	1	155	15.7	8.44	12	1	1	Medium	1	1	0.937	0.089	0.067	0.179	0.121	0.097	0.131	0.095	0.115	0.115	0.12	0.123	0.117
TRINITY_DN141738_c0_g1_i1_orf1	PREDICTED: fibroblast growth factor 1 [Microplitis demolitor]	1	82	9.5	9.61	10	1	1	Medium	1	0.903	0.947	0.586	0.612	0.663	0.625	0.599	0.652	0.602	0.612	0.583	0.628	0.595	0.568
TRINITY_DN29038_c0_g2_i1_orf1	PREDICTED: ATP synthase lipid-binding protein, mitochondrial [Fopius arisanus	1	138	14.4	10.01	5	2	1	Medium	1	0.984	1.021	0.587	0.523	0.612	0.559	0.588	0.547	0.49	0.531	0.468	0.538	0.56	0.554
TRINITY_DN20682_c0_g2_i1_orf1	glutathione S-transferase delta3 [Glyphodes pyloalis]	1	122	14.1	5.85	8	1	1	Medium	1	0.965	1.01	0.149	0.16	0.234	0.187	0.272	0.22	0.209	0.173	0.177	0.272	0.281	0.243
TRINITY_DN13177_c0_g1_i9_orf1	uncharacterized protein LOC114358817 [Ostrinia furnacalis]	1	1935	213.6	4.61	1	1	1	Medium	1	1.317	1.089	1.441	1.463	1.35	1.356	1.008	1.2	1.248	1.201	1.224	1.305	1.268	1.316
TRINITY_DN23798_c0_g1_i1_orf1	arginine-glutamic acid dipeptide repeats protein [Ostrinia furnacalis]	1	1683	184.7	7.42	1	1	1	Medium	1	0.938	0.907	0.734	0.727	0.636	0.846	0.77	0.753	0.851	0.91	1.015	0.848	1.06	0.855
TRINITY_DN10106_c0_g2_i1_orf1	arrestin domain-containing protein 2-like isoform X3 [Ostrinia furnacalis]	1	149	16.9	5.27	8	1	1	Medium	1	0.867	1.005	0.621	0.697	0.743	0.602	0.658	0.591	0.509	0.765	0.714	0.726	0.611	0.584
TRINITY_DN8738_c0_g1_i1_orf1	unnamed protein product [Plutella xylostella]	1	317	37.1	8.35	4	1	1	Medium	1	1.118	0.963	0.803	0.936	0.954	0.752	0.943	0.772	0.676	0.533	0.831	1.246	1.304	1.11
TRINITY_DN4294_c0_g1_i6_orf1	protein vav isoform X2 [Ostrinia furnacalis]	1	808	92.5	7.94	1	1	1	Medium	1	0.949	0.942	0.775	0.812	0.704	0.796	0.806	0.906	0.89	0.888	0.961	0.711	0.692	0.694
TRINITY_DN2002_c0_g1_i5_orfp1	TRINITY_DN2002_c0_g1_i5_m.4230 TRINITY_DN2002_c0_g1::TRINITY_DN2002_	1	258	29.4	5.86	5	1	1	Medium	1	0.98	0.809	1.616	1.371	1.519	1.307	1.37	1.648	0.9	1.095	1.044	3.941	4.432	4.623
TRINITY_DN29707_c0_g1_i2_orf1	2-(3-amino-3-carboxypropyl)histidine synthase subunit 2 [Ostrinia furnacalis]	1	458	52.8	5.52	2	1	1	Medium	1	1.188	1.189	0.984	0.824	0.721	0.858	0.72	0.809	0.727	0.906	0.654	0.829	0.752	0.717
TRINITY_DN46633_c0_g1_i4_orf1	uncharacterized protein LOC114365425 [Ostrinia furnacalis]	1	221	25.9	7.59	5	1	1	Medium	1	1.023	1.126	0.909	0.974	0.897	0.858	0.78	0.798	1.117	0.92	1.189	0.622	0.685	0.579
TRINITY_DN100885_c0_g2_i1_orfp	CCHC-type zinc finger, partial [Cricetulus griseus]	1	147	16.2	6.8	6	2	1	Medium	1	0.909	0.958	0.762	0.775	0.699	0.776	0.759	0.783	0.726	0.659	0.793	0.721	0.85	0.684
TRINITY_DN4546_c0_g1_i3_orf1	telomerase Cajal body protein 1 homolog [Ostrinia furnacalis]	1	470	53.1	5.16	2	1	1	Medium	1	1.031	1.012	0.714	0.705	0.936	0.653	0.644	0.578	0.753	0.679	0.735	0.934	0.961	0.695
TRINITY_DN77559_c0_g1_i1_orf1	uncharacterized protein LOC114359392 isoform X2 [Ostrinia furnacalis]	1	264	28.7	5.68	5	1	1	Medium	1	1.049	0.819	1.366	1.43	1.303	1.643	1.687	1.824	1.452	1.51	1.686	1.072	1.097	1.004
TRINITY_DN15916_c0_g1_i1_orf1	balbiani ring protein 3-like [Bicyclus anynana]	1	214	22.5	9.17	3	1	1	Medium	1	1.128	1.049	0.407	0.561	0.655	0.91	0.696	0.786	0.753	0.698	0.637	0.675	0.632	0.6
TRINITY_DN66561_c0_g1_i1_orf1	uncharacterized protein LOC114353797 isoform X2 [Ostrinia furnacalis]	1	312	35.2	9	3	1	1	Medium	1	1.506	1.667	1.914	0.781	1.503	1.073	1.157	1.226	3.277	2.368	1.581	1.218	1.453	1.161
TRINITY_DN140613_c0_g1_i1_orf1	hypothetical protein M0804_013066 [Polistes exclamans]	1	223	25	6.21	4	1	1	Medium	1	1.033	0.818	1.074	1.055	1.323	1.13	0.944	1.052	0.755	0.729	0.98	0.808	0.891	0.883
TRINITY_DN2673_c2_g1_i2_orf1	aminopeptidase N3c [Ostrinia nubilalis]	1	519	59.7	5.14	2	1	1	Medium	1	1.133	1.048	0.936	0.879	0.83	0.819	1.038	0.936	1.196	1.264	1.402	0.712	0.665	0.866
TRINITY_DN13732_c0_g2_i3_orf1	60S ribosomal protein L35 [Ostrinia furnacalis]	1	153	17.9	10.61	5	1	1	Medium	1	0.76	0.743	0.779	0.851	0.75	0.77	0.882	0.878	0.925	1.022	0.979	1.209	1	1.188
TRINITY_DN3550_c0_g1_i4_orf1	UDP-glycosyltransferase UGT42C7 [Ostrinia furnacalis]	1	517	58.6	8.53	2	1	1	Medium	1	1.133	1.061	1.068	0.91	0.848	1.015	1.04	1.176	1.017	1.04	1.092	1.013	0.816	1.082
TRINITY_DN5011_c0_g1_i1_orf1	probable G-protein coupled receptor 158 isoform X1 [Galleria mellonella]	1	163	17.6	9.85	6	1	1	Medium	1	1.02	1.046	0.899	0.922	0.967	1.006	0.986	1.011	0.827	0.832	1.008	0.815	0.797	0.821
TRINITY_DN9486_c1_g1_i7_orfp1	uncharacterized protein LOC114351033 isoform X1 [Ostrinia furnacalis]	1	67	7	6.52	18	1	1	Medium	1	0.926	1.084	0.98	0.96	1.135	0.984	1.145	0.902	0.695	0.9	0.843	0.982	1.25	1.294
TRINITY_DN48878_c0_g2_i1_orf1	codanin-1 [Ostrinia furnacalis]	1	729	83.1	8.69	1	3	1	Medium	1	1.291	1.256	2.045	2.133	1.859	2.742	2.591	2.724	3.927	2.951	2.617	2.689	2.852	2.625
TRINITY_DN10332_c0_g1_i2_orfp1	TRINITY_DN10332_c0_g1_i2_m.42894 TRINITY_DN10332_c0_g1::TRINITY_DN1C	1	76	9	9.41	8	1	1	Medium	1	0.932	0.922	0.465	0.416	0.459	0.381	0.302	0.355	0.284	0.257	0.294	0.464	0.451	0.426
TRINITY_DN2560_c0_g1_i1_orf1	hypothetical protein NE865_02894 [Phthorimaea operculella]	1	128	14.9	9.91	5	1	1	Medium	1	1.014	1.02	0.784	0.984	0.93	0.712	1.087	0.709	0.83	0.882	0.841	0.95	0.848	0.791
TRINITY_DN3092_c0_g1_i2_orf1	replication factor C subunit 1 isoform X1 [Ostrinia furnacalis]	1	1097	123.1	9.22	1	1	1	Medium	1	0.904	0.902	0.651	0.651	0.858	0.701	0.74	0.591	1.373	1.249	1.161	0.721	0.758	0.643
TRINITY_DN5902_c0_g1_i4_orf1	evolutionarily conserved signaling intermediate in Toll pathway, mitochondrial	1	370	42.2	5.9	7	1	1	Medium	1	0.876	0.955	0.966	0.921	1.181	1.137	1.122	1.452	0.947	1.709	0.69	1.099	0.925	0.916
TRINITY_DN7313_c1_g1_i2_orf1	unnamed protein product [Chilo suppressalis]	1	895	97.8	9.35	2	1	1	Medium	1	0.985	0.943	1.376	1.281	0.952	1.051	0.865	0.756	0.976	0.986	1.185	1.162	1.274	1.021
TRINITY_DN66596_c0_g1_i1_orf1	CCR4-NOT transcription complex subunit 10 [Ostrinia furnacalis]	1	570	64.3	8.24	1	1	1	Medium	1	0.887	0.976	0.614	0.58	0.64	0.64	0.672	0.571	0.532	0.521	0.593	0.664	0.589	0.578
TRINITY_DN69697_c0_g1_i1_orf1	PREDICTED: uncharacterized protein LOC103573287 [Microplitis demolitor]	1	1025	115.8	5.58	1	2	1	Medium	1	1.053	1.265	0.84	0.733	0.709	0.481	0.63	0.689	0.737	0.822	0.68	0.641	0.489	0.584
TRINITY_DN27903_c0_g1_i1_orf1	desaturase MPVE [Helicoverpa assulta]	1	364	42.3	8.97	2	1	1	Medium	1	0.98	1.115	0.884	0.887	0.849	1.213	1.233	1.204	1.041	1.046	0.795	0.979	0.716	0.766
TRINITY_DN3089_c0_g1_i1_orf1	anaphase-promoting complex subunit 5 isoform X2 [Ostrinia furnacalis]	1	265	29.6	7.8	3	1	1	Medium	1	1.043	0.714	1.146	0.812	0.724	0.737	1.404	1.103	0.821	0.88	0.878	0.666	1.007	1.134
TRINITY_DN140669_c0_g1_i1_orf1	S-methyl-5'-thioadenosine phosphorylase-like isoform X1 [Hyposmocoma kal	1	71	8.1	10.15	10	1	1	Medium	1	1.034	1.106	0.195	0.087	0.252	0.21	0.254	0.286	0.245	0.233	0.218	0.31	0.255	0.282
TRINITY_DN1954_c0_g1_i4_orf1	unnamed protein product [Diatraea saccharalis]	1	700	79.2	8.81	2	1	1	Medium	1	0.895	0.995	1.052	1.106	1.097	0.938	0.955	0.963	0.872	0.749	0.839	0.92	0.901	0.876
TRINITY_DN144190_c0_g1_i1_orf1	PREDICTED: uncharacterized protein LOC103572804 isoform X2 [Microplitis de	1	245	26.8	4.93	3	1	1	Medium	1	0.971	0.891	0.012	0.039	0.065	0.078	0.086	0.079	0.109	0.077	0.092	0.078	0.072	0.073
TRINITY_DN141396_c0_g1_i1_orf1	pre-rRNA 2'-O-ribose RNA methyltransferase FTSJ3 [Diachasma alloeum]	1	839	96.6	9	1	1	1	Medium	1	1.125	1.046	0.753	0.764	0.842	0.835	1.132	0.928	0.763	0.597	0.739	0.835	0.785	0.655
TRINITY_DN29_c0_g1_i4_orf1	sodium-dependent nutrient amino acid transporter 1-like [Ostrinia furnacalis]	1	568	63	6.83	1	1	1	Medium	1	1.081	1.048	0.625	0.777	0.565	0.74	0.759	0.718	0.882	0.991	1.353	0.386	0.503	0.447
TRINITY_DN1266_c2_g1_i1_orf1	serine/threonine-protein kinase RIO3 [Ostrinia furnacalis]	1	496	57.3	5.8	2	1	1	Medium	1	1.257	0.979	0.5	0.445	0.311	0.586	0.657	0.679	0.37	0.49	0.484	0.831	0.704	0.569
TRINITY_DN11392_c0_g1_i4_orf1	chondroitin sulfate synthase 1 isoform X1 [Ostrinia furnacalis]	1	714	82.1	6.48	1	1	1	Medium	1	1.337	1.332	0.683	0.599	0.664	0.768	0.858	0.542	0.693	0.743	0.638	0.73	0.879	0.711
TRINITY_DN17864_c0_g1_i1_orf1	PREDICTED: erlin-2-B [Microplitis demolitor]	1	329	37.3	6.47	2	1	1	Medium	1	0.954	0.851	0.281	0.305	0.371	0.3	0.344	0.33	0.274	0.375	0.247	0.269	0.309	0.355
TRINITY_DN12748_c2_g1_i1_orfp1	TRINITY_DN12748_c2_g1_i1_m.21305 TRINITY_DN12748_c2_g1::TRINITY_DN12	1	886	100	8.73	1	1	1	Medium	1	1.083	1.095	0.964	0.906	1.067	1.476	1.286	1.35	1.422	1.071	1.15	0.715	0.756	0.605
TRINITY_DN115658_c0_g1_i1_orf1	hypothetical protein B5X24_HaOG203018 [Helicoverpa armigera]	1	506	58.4	9.04	2	1	1	Medium	1	1.151	1.071	0.988	1.154	1.123	0.822	0.764	0.834	0.685	0.599	0.82	0.682	0.682	0.635
TRINITY_DN10886_c0_g2_i4_orf1	hypothetical protein evm_007910 [Chilo suppressalis]	1	1442	163.7	6.74	0	1	1	Medium	1	0.963	0.927	0.795	1.269	1.021	1.267	1.274	1.274	1.163	1.24	0.893	1.293	0.488	0.498
TRINITY_DN144850_c0_g1_i1_orf1	phospholipase DDHD2 isoform X1 [Diachasma alloeum]	1	1193	133.6	5.47	1	1	1	Medium	1	0.916	0.968	0.951	0.96	0.946	1.037	0.87	1.024	0.818	0.809	0.864	1.002	0.944	0.872
TRINITY_DN9711_c0_g1_i10_orf1	cAMP-specific 3',5'-cyclic phosphodiesterase isoform X2 [Ostrinia furnacalis]	1	873	96.8	5.68	1	1	1	Medium	1	1.03	1.057	0.896	0.874	0.81	0.773	0.705	0.866	0.667	0.78	0.821	0.657	0.824	0.765
TRINITY_DN86833_c0_g3_i1_orf1	PREDICTED: glycerol-3-phosphate acyltransferase 1, mitochondrial isoform X1	1	862	98.9	8.07	1	2	1	Medium	1	1.026	1.025	1.353	1.548	1.515	1.341	1.571	1.368	1.938	1.606	2	2.195	2.22	2.25
TRINITY_DN41334_c0_g1_i1_orf1	sarcosine dehydrogenase, mitochondrial [Chelonus insularis]	1	897	100.7	8	3	1	1	Medium	1	0.917	1.058	1.036	0.927	0.828	0.973	0.691	0.98	1.021	0.832	0.847	0.664	0.644	0.644
TRINITY_DN13216_c0_g1_i5_orf1	uncharacterized protein LOC114358344 isoform X1 [Ostrinia furnacalis]	1	1324	148.9	5.9	1	1	1	Medium	1	1.165	1.006	0.315	0.275	0.359	0.319	0.32	0.321	0.336	0.297	0.358	0.901	0.991	0.974
TRINITY_DN26243_c0_g1_i2_orf1	dynein heavy chain 6, axonemal [Ostrinia furnacalis]	1	4081	466.3	5.87	0	1	1	Medium	1	0.954	0.972	0.675	0.538	0.652	0.754	0.632	0.781	0.621	0.55	0.604	0.55	0.784	0.778
TRINITY_DN27745_c0_g1_i6_orf1	unnamed protein product [Diatraea saccharalis]	1	543	61.9	7.09	2	1	1	Medium	1	1.088	0.636	0.595	0.456	0.608	0.88	0.749	0.719	0.742	0.431	0.886	0.626	0.656	0.498
TRINITY_DN15753_c0_g1_i1_orf1	uncharacterized protein LOC114366450 [Ostrinia furnacalis]	1	1555	172.3	5.66	1	1	1	Medium	1	0.91	0.996	0.814	0.818	0.758	0.623	0.707	0.794	0.786	0.669	0.774	0.98	0.934	0.909
TRINITY_DN21226_c0_g1_i2_orf1	COMM domain-containing protein 4 isoform X2 [Ostrinia furnacalis]	1	102	11.2	5.27	9	1	1	Medium	1	0.909	0.955	0.842	0.908	0.657	0.853	0.871	0.893	0.975	0.842	0.968	0.707	1.123	1.156
TRINITY_DN2893_c0_g1_i4_orf1	X-ray repair cross-complementing protein 5-like [Ostrinia furnacalis]	1	709	80.3	5.36	2	1	1	Medium	1	0.782	0.785	0.809	0.964	0.937	0.857	1.023	0.937	0.988	0.971	1.033	0.915	0.889	0.936
TRINITY_DN9538_c1_g3_i1_orf1	cilia- and flagella-associated protein 410 isoform X2 [Aphidius gifuensis]	1	411	47.7	5.59	2	2	1	Medium	1	1.065	1.148	2.693	2.562	2.41	2.897	2.904	2.732	3.689	3.056	3.593	4.508	4.248	4.456
TRINITY_DN46090_c0_g3_i1_orf1	tyrosine-protein kinase-like otk, partial [Ostrinia furnacalis]	1	500	55	7.3	1	2	1	Medium	1	0.985	1.004	1.157	1.166	1.293	1.354	1.41	1.253	1.952	2.063	2.117	2.03	1.6	1.467
TRINITY_DN9243_c0_g1_i4_orf1	copper-transporting ATPase 1 [Ostrinia furnacalis]	1	908	97.6	7.12	1	1	1	Medium	1	0.961	0.826	0.912	1.013	1.057	0.715	1.004	0.885	0.783	0.533	0.806	1.152	1.358	1.065
TRINITY_DN146264_c0_g1_i1_orf1	PREDICTED: protein preli-like [Fopius arisanus]	1	238	27.2	9.19	3	1	1	Medium	1	0.868	0.962	0.665	0.677	0.705	0.53	0.639	0.66	0.541	0.515	0.533	0.569	0.689	0.598
TRINITY_DN65057_c0_g2_i1_orf1	carboxypeptidase B-like [Ostrinia furnacalis]	1	181	21.3	8.76	4	1	1	Medium	1	1.239	2.153	0.974	2.167	1.482	0.805	1.276	1.648	1.363	1.543	1.054	1.259	1.084	1.077
TRINITY_DN20767_c0_g2_i1_orf1	glycosylated lysosomal membrane protein B-like [Vanessa atalanta]	1	270	30	5.83	2	1	1	Medium	1	0.918	0.908	0.621	0.653	0.609	0.48	0.672	0.698	0.608	0.56	0.47	1.12	1.091	1.277
TRINITY_DN5775_c0_g1_i1_orf1	proteasome assembly chaperone 2 [Ostrinia furnacalis]	1	247	27.8	6.67	3	1	1	Medium	1	1.002	0.969	0.585	0.651	0.668	0.641	0.617	0.694	0.545	0.528	0.568	0.714	0.644	0.651
TRINITY_DN2374_c0_g2_i2_orf1	uncharacterized protein LOC114357127 [Ostrinia furnacalis]	1	243	28.2	10.9	2	1	1	Medium	1	0.843	1.001	1.297	1.267	1.25	1.398	1.449	1.2	1.393	1.366	1.115	1.373	1.227	1.228
TRINITY_DN30154_c0_g1_i1_orf1	serine/threonine-protein kinase SIK2 [Ostrinia furnacalis]	1	1220	136.1	8.35	1	1	1	Medium	1	0.818	0.95	1.435	1.169	1.623	1.519	1.256	1.458	1.412	1.083	0.941	12.645	12.793	12.382
TRINITY_DN74889_c0_g1_i1_orf1	probable 28S ribosomal protein S23, mitochondrial [Ostrinia furnacalis]	1	158	17.8	8.31	6	1	1	Medium	1	1.007	1.028	0.552	0.518	0.53	0.592	0.664	0.671	0.581	0.571	0.645	0.462	0.489	0.425
TRINITY_DN7841_c1_g1_i9_orf1	TBC1 domain family member 13 [Ostrinia furnacalis]	1	384	44.4	5.73	4	1	1	Medium	1	1.11	1.084	0.977	0.868	1.135	0.95	1.114	1.044	0.947	0.967	0.767	1.087	1.072	1.053
TRINITY_DN974_c0_g2_i1_orfp1	TRINITY_DN974_c0_g2_i1_m.54007 TRINITY_DN974_c0_g2::TRINITY_DN974_c0	1	102	11.6	8.63	11	1	1	Medium	1	0.964	0.832	0.667	1.036	0.745	0.861	1.296	1	0.964	1.02	1.05	0.841	0.752	0.803
TRINITY_DN140538_c0_g2_i1_orf1	peptidyl-prolyl cis-trans isomerase NIMA-interacting 1 [Urocitellus parryii]	1	90	10.2	10.08	8	1	1	Medium	1	1.166	1.009	0.807	0.895	0.873	0.753	0.747	0.745	0.654	0.506	0.606	0.707	0.872	0.744
TRINITY_DN6768_c0_g1_i1_orf1	solute carrier family 25 member 46 [Ostrinia furnacalis]	1	406	45.7	7.17	2	1	1	Medium	1	1.03	1.278	0.81	1.118	1.027	0.949	0.957	0.986	0.857	0.927	0.78	0.949	1.057	0.894
TRINITY_DN1308_c0_g1_i4_orf1	serine proteinase stubble-like [Ostrinia furnacalis]	1	257	28.8	5.6	3	1	1	Medium	1	1.169	0.792	0.9	1.048	0.931	1.24	1.405	1.183	1.248	1.226	1.123	3.128	2.748	2.914
TRINITY_DN21492_c0_g1_i1_orf1	isocitrate dehydrogenase [NAD] subunit gamma, mitochondrial [Chelonus insu	1	385	42.3	8.54	3	1	1	Medium	1	1.036	0.928	0.683	0.623	0.626	0.655	0.754	0.746	0.66	0.806	0.67	0.689	0.708	0.686
TRINITY_DN9309_c0_g1_i5_orf1	uncharacterized protein LOC114361160 [Ostrinia furnacalis]	1	1192	139.3	7.17	1	1	1	Medium	1	1.043	1.14	0.535	0.596	0.606	0.643	0.638	0.686	0.578	0.507	0.508	0.83	0.758	0.7
TRINITY_DN3934_c0_g1_i9_orf1	protein cereblon [Ostrinia furnacalis]	1	440	48.8	6.02	1	1	1	Medium	1	0.878	0.837	0.974	0.791	1.322	1.288	0.766	1.242	1.041	12.091	0.595	0.663	1.362	1.189
TRINITY_DN145666_c0_g1_i1_orf1	PREDICTED: 40S ribosomal protein S29 [Microplitis demolitor]	1	56	6.5	9.6	14	1	1	Medium	1	1.053	0.949	0.714	0.7	0.665	0.52	0.742	0.617	0.537	0.619	0.626	0.709	0.625	0.723
TRINITY_DN40440_c0_g1_i1_orf1	Na(+)/H(+) exchange regulatory cofactor NHE-RF1 [Helicoverpa zea]	1	59	6.4	10.17	10	1	1	Medium	1	1.038	1.025	1.069	1.092	1.174	0.827	0.988	0.838	0.906	0.748	0.784	0.987	1.017	0.979
TRINITY_DN271_c0_g2_i6_orf1	hypothetical protein NE865_03378 [Phthorimaea operculella]	1	595	67.5	7.34	1	1	1	Medium	1	1.031	1.059	0.679	0.666	0.738	0.57	0.545	0.536	0.568	0.615	0.451	0.537	0.552	0.546
TRINITY_DN252_c0_g1_i3_orf1	probable phospholipid-transporting ATPase IM [Ostrinia furnacalis]	1	1150	129.3	6.21	1	1	1	Medium	1	0.722	1.04	0.795	0.647	0.872	0.674	0.79	0.562	0.583	0.787	0.73	1.206	0.959	1.151
TRINITY_DN49204_c0_g1_i1_orf1	uncharacterized protein C05D11.1-like [Chelonus insularis]	1	1022	115	6	1	1	1	Medium	1	1.053	1.06	0.755	0.785	0.742	1.135	0.988	0.933	0.913	0.793	0.84	0.697	0.76	0.706
TRINITY_DN29217_c0_g1_i3_orf1	protein yippee-like 5 [Ostrinia furnacalis]	1	121	13.8	6.3	5	1	1	Medium	1	1.056	1.185	1.74	1.468	1.593	1.658	1.481	1.766	1.719	1.758	1.792	1.833	1.68	1.928
TRINITY_DN11736_c0_g1_i1_orf1	uncharacterized protein LOC114352195 [Ostrinia furnacalis]	1	414	46.9	5.82	1	1	1	Medium	1	1.133	0.95	1.502	1.191	1.281	1.359	1.209	1.377	1.33	1.305	1.194	0.981	1.127	1.111
TRINITY_DN2647_c0_g1_i3_orf1	DNA repair protein complementing XP-G cells homolog isoform X1 [Ostrinia fu	1	1167	132.1	5.2	1	1	1	Medium	1	1.019	0.973	0.86	0.923	0.939	1.414	1.304	1.336	1.471	1.143	1.379	0.696	0.626	0.609
TRINITY_DN880_c0_g1_i6_orf1	cuticle protein 19-like [Ostrinia furnacalis]	1	231	26.5	6.92	3	1	1	Medium	1	0.876	0.819	0.627	0.769	0.656	0.788	0.706	0.734	0.681	0.576	0.667	0.959	1.171	0.954
TRINITY_DN10539_c0_g1_i1_orf1	uncharacterized protein LOC114358962 isoform X1 [Ostrinia furnacalis]	1	350	40.4	4.94	2	1	1	Medium	1	0.912	1.309	1.589	1.741	1.792	1.96	1.978	1.926	3.597	2.562	1.735	2.032	2.331	1.505

TRINITY_DN24490_c0_g1_i6_orf1	E3 ubiquitin-protein ligase Hakai [Ostrinia furnacalis]	1	360	39.8	8.5	2	1	1	Medium	1	0.933	1.022	0.46	0.629	0.762	0.664	0.774	0.587	0.533	0.715	0.504	0.802	0.991	1.015
TRINITY_DN6462_c0_g1_i5_orf1	probable histone-lysine N-methyltransferase CG1716 [Ostrinia furnacalis]	1	1570	177	5.68	1	1	1	Medium	1	0.942	0.841	1.707	1.593	1.455	1.483	1.611	1.614	2.187	1.792	2.323	2.065	1.768	1.976
TRINITY_DN22441_c0_g1_i1_orf1	Gamma-aminobutyric acid receptor-associated protein, partial [Cotesia chilon	1	126	15	8.12	6	1	1	Medium	1	1.193	1.057	1.206	1.259	1.578	1.369	1.53	1.478	1.586	1.397	1.28	1.643	1.707	1.457
TRINITY_DN77642_c0_g1_i1_orf1	peritrophic membrane chitin binding protein [Loxostege sticticalis]	1	224	24.3	4.54	6	1	1	Medium	1	1.135	1.246	1.19	1.183	1.258	0.816	0.996	0.838	0.925	0.897	0.933	1.503	1.387	1.412
TRINITY_DN12503_c0_g2_i1_orf1	MMS19 nucleotide excision repair protein homolog [Ostrinia furnacalis]	1	922	104.3	5.57	1	1	1	Medium	1	1.028	1.033	0.746	0.887	0.872	0.842	0.915	0.876	0.931	0.855	0.799	0.972	0.907	0.894
TRINITY_DN71494_c0_g1_i2_orf1	hypothetical protein O3G_MSEX005876 [Manduca sexta]	1	275	31.8	9.61	3	1	1	Medium	1	1.104	1.085	0.664	0.71	0.63	0.715	0.659	0.996	0.75	0.629	0.663	0.671	0.949	0.778
TRINITY_DN5908_c0_g1_i2_orf1	ATP-binding cassette sub-family B member 10, mitochondrial-like [Ostrinia fu	1	704	77.6	9.16	1	1	1	Medium	1	0.984	0.925	0.624	0.72	0.741	0.655	0.724	0.712	0.698	0.792	0.862	0.763	0.729	0.717
TRINITY_DN195_c8_g1_i1_orf1	hypothetical protein evm_009768 [Chilo suppressalis]	1	205	21.6	9.85	4	1	1	Medium	1	0.969	0.998	1.532	2.064	1.719	0.505	0.409	0.446	0.397	0.462	0.426	0.654	0.6	0.776
TRINITY_DN143637_c0_g1_i1_orf1	PX domain-containing protein kinase-like protein isoform X1 [Chelonus insula	1	589	65.7	9.51	2	1	1	Medium	1	0.811	0.824	1.359	1.522	1.642	1.163	1.314	1.332	1.682	1.665	1.526	1.834	1.915	1.999
TRINITY_DN20966_c0_g1_i6_orf1	clavesin-1-like [Ostrinia furnacalis]	1	305	35	6.65	4	1	1	Medium	1	1	0.948	0.62	0.806	0.783	0.642	0.744	0.677	0.719	0.858	0.683	0.71	0.628	0.677
TRINITY_DN5107_c0_g1_i4_orf1	peptide methionine sulfoxide reductase [Ostrinia furnacalis]	1	232	26.1	7.52	4	1	1	Medium	1	1.046	0.976	0.184	0.264	0.337	0.203	0.242	0.286	0.226	0.227	0.285	0.27	0.199	0.23
TRINITY_DN4565_c0_g1_i3_orf1	acid phosphatase type 7 isoform X1 [Ostrinia furnacalis]	1	65	7.5	7.05	14	1	1	Medium	1	0.938	0.931	1.159	1.156	1.244	1.172	1.123	1.183	1.188	1.246	1.101	1.152	1.31	1.214
TRINITY_DN92232_c0_g1_i1_orf1	protein SDA1 homolog [Ostrinia furnacalis]	1	725	84.7	9	1	1	1	Medium	1	1.03	1.039	0.226	0.362	0.337	0.327	0.441	0.508	0.387	0.39	0.291	0.366	0.348	0.397
TRINITY_DN146138_c0_g1_i1_orf1	E3 ubiquitin-protein ligase Ubr3 [Chelonus insularis]	1	810	92.7	7.34	2	1	1	Medium	1	1.008	0.957	1.531	1.678	1.532	1.236	1.249	1.583	1.605	1.572	1.475	1.624	1.629	1.521
TRINITY_DN57105_c0_g1_i2_orf1	transmembrane protein 161B isoform X1 [Galleria mellonella]	1	487	55.3	7.72	2	1	1	Medium	1	0.937	0.819	0.634	0.49	0.656	0.492	0.453	0.604	0.6	0.508	0.524	0.865	0.73	0.815
TRINITY_DN17505_c0_g1_i15_orf1	unnamed protein product [Chilo suppressalis]	1	2636	297.6	9.44	0	1	1	Medium	1	1.025	0.989	1.239	1.386	1.134	2.787	2.429	2.063	0.987	0.969	1.248	1.166	1.289	1.242
TRINITY_DN25542_c0_g1_i1_orf1	tRNA-dihydrouridine(47) synthase [NAD(P)(+)]-like [Ostrinia furnacalis]	1	590	67.1	8.32	2	1	1	Medium	1	1.011	0.999	0.522	0.639	0.62	0.552	0.55	0.664	0.473	0.519	0.524	0.588	0.551	0.561
TRINITY_DN102712_c0_g1_i1_orf1	transmembrane protein 177 [Ostrinia furnacalis]	1	324	37.3	8.29	3	1	1	Medium	1	0.92	0.897	0.934	0.822	0.76	0.602	0.676	0.574	1.035	1.161	1.14	0.79	0.676	0.952
TRINITY_DN4923_c0_g1_i4_orf1	O-acyltransferase like protein-like [Ostrinia furnacalis]	1	612	70.2	9.44	1	1	1	Medium	1	0.933	1.026	0.271	0.421	0.303	0.381	0.453	0.389	0.351	0.342	0.356	0.31	0.366	0.339
TRINITY_DN44256_c0_g1_i1_orf1	essential MCU regulator, mitochondrial [Cotesia glomerata]	1	90	9.7	4.75	11	2	1	Medium	1	0.943	0.898	0.627	0.729	0.825	0.766	0.749	0.843	0.684	0.645	0.729	1.057	1.26	1.131
TRINITY_DN12113_c0_g1_i1_orf1	WD repeat-containing protein 5 [Helicoverpa armigera]	1	346	37.6	8.51	2	1	1	Medium	1	1.089	1.123	0.67	0.711	0.694	0.775	0.744	0.7	0.988	0.764	0.81	0.653	0.748	0.728
TRINITY_DN5334_c0_g1_i6_orf1	RING finger and CHY zinc finger domain-containing protein 1 [Ostrinia furnaca	1	327	36.4	6.6	2	1	1	Medium	1	0.953	1.013	1.02	1.336	1.417	1.018	1.09	1.221	1.032	0.822	1.062	1.013	1.006	0.998
TRINITY_DN84631_c0_g1_i1_orf1	PREDICTED: rap guanine nucleotide exchange factor 2-like isoform X9 [Microp	1	141	16.1	5.58	7	1	1	Medium	1	0.882	0.796	0.877	0.98	1.078	1.133	1.698	1.369	1.058	0.932	0.951	1.167	1.39	1.284
TRINITY_DN36883_c0_g1_i1_orf1	PREDICTED: importin subunit alpha-4 [Microplitis demolitor]	1	515	57.2	5.05	2	1	1	Medium	1	1.051	1.21	0.992	1.126	0.96	1.176	0.83	0.988	0.905	0.855	0.935	1.374	1.24	0.974
TRINITY_DN111488_c0_g1_i1_orf1	LOW QUALITY PROTEIN: formin-J-like [Chelonus insularis]	1	202	22.4	5.5	3	1	1	Medium	1	1.069	1.127	1.809	2.048	1.868	1.447	1.791	1.623	1.859	1.473	1.411	0.694	0.518	0.69
TRINITY_DN2471_c0_g1_i3_orf1	multidrug resistance protein homolog 49-like [Ostrinia furnacalis]	1	1308	141.6	7.61	1	1	1	Medium	1	1.092	1.008	0.613	0.817	1.011	0.824	1.81	0.756	0.787	1.057	0.694	0.657	0.68	0.742
TRINITY_DN58636_c0_g1_i1_orf1	uncharacterized protein LOC114363665 [Ostrinia furnacalis]	1	580	66.5	5.47	1	1	1	Medium	1	1.112	1.15	0.471	0.527	0.465	0.428	0.399	0.415	0.449	0.306	0.404	0.625	0.478	0.542
TRINITY_DN1198_c0_g1_i1_orf1	protein suppressor of sable isoform X1 [Ostrinia furnacalis]	1	1190	134.2	7.4	1	1	1	Medium	1	1.223	0.963	0.969	0.944	0.784	0.812	0.855	0.791	0.805	0.728	0.778	0.79	0.818	0.781
TRINITY_DN9356_c0_g1_i1_orf1	vacuolar protein sorting-associated protein 33B [Ostrinia furnacalis]	1	650	72	7.56	2	1	1	Medium	1	0.932	1.005	0.821	0.746	0.824	0.832	0.771	0.804	0.936	0.87	0.615	0.845	0.798	0.969
TRINITY_DN3628_c0_g1_i5_orf1	palmitoyltransferase Hip14 isoform X2 [Ostrinia furnacalis]	1	615	68.3	8.07	2	1	1	Medium	1	1.174	1.036	0.603	0.632	0.477	0.537	0.498	0.592	0.634	0.509	0.488	0.625	0.719	0.62
TRINITY_DN37393_c0_g1_i1_orf1	protein melted [Pectinophora gossypiella]	1	325	36.2	7.64	2	1	1	Medium	1	1.025	1.051	0.53	0.624	0.717	0.565	0.535	0.651	0.554	0.533	0.495	0.641	0.62	0.574
TRINITY_DN37821_c0_g1_i6_orf1	uncharacterized protein LOC114350690 [Ostrinia furnacalis]	1	210	23.9	5.03	3	1	1	Medium	1	0.877	0.962	1.394	1.308	1.406	1.363	1.287	1.412	1.196	1.164	1.009	1.343	1.389	1.353
TRINITY_DN97680_c0_g1_i1_orf1	39S ribosomal protein L52, mitochondrial [Ostrinia furnacalis]	1	127	14.8	9.79	20	1	1	Medium	1	0.939	0.84	0.437	0.285	0.401	0.339	0.199	0.256	0.394	0.363	0.256	0.361	0.424	0.384
TRINITY_DN7556_c0_g1_i1_orf1	venom carboxylesterase-6-like [Ostrinia furnacalis]	1	491	54.8	5.1	1	1	1	Medium	1	1.049	1.024	0.23	0.239	0.308	0.263	0.282	0.282	0.235	0.296	0.291	0.304	0.279	0.425
TRINITY_DN3964_c1_g1_i2_orf1	phosphoinositide 3-kinase regulatory subunit 4 isoform X1 [Ostrinia furnacalis]	1	1151	127.9	7.08	1	1	1	Medium	1	0.904	0.844	0.696	0.72	0.739	0.852	0.886	0.923	0.622	0.574	0.601	0.654	0.672	0.523
TRINITY_DN16354_c0_g1_i2_orf1	uncharacterized protein LOC114349750 isoform X1 [Ostrinia furnacalis]	1	471	53	6.6	4	1	1	Medium	1	1.089	1.135	0.729	0.646	0.7	0.884	0.957	0.853	0.699	0.791	0.746	0.7	0.59	0.527
TRINITY_DN2346_c0_g2_i1_orf1	uncharacterized protein LOC113514389 isoform X1 [Galleria mellonella]	1	242	26.1	8.43	3	1	1	Medium	1	0.938	0.908	1.105	1.285	1.402	1.012	1.026	1.259	1.064	0.846	0.875	0.894	0.862	1.318
TRINITY_DN9028_c0_g1_i5_orf1	decaprenyl-diphosphate synthase subunit 2-like [Ostrinia furnacalis]	1	426	46.4	6.68	2	1	1	Medium	1	0.918	0.994	0.809	1.006	0.787	0.784	1.052	0.981	0.891	0.735	0.71	1.546	1.867	1.63
TRINITY_DN44709_c0_g1_i1_orf1	D-beta-hydroxybutyrate dehydrogenase, mitochondrial, partial [Chelonus insu	1	341	39.4	7.21	2	1	1	Medium	1	1.093	0.984	1.291	1.293	1.148	1.44	1.438	1.258	1.643	1.487	1.895	1.768	1.797	1.873
TRINITY_DN70_c2_g1_i1_orf1	inositol-tetrakisphosphate 1-kinase-like [Ostrinia furnacalis]	1	368	41.3	6.42	5	1	1	Medium	1	0.979	0.876	0.574	0.567	0.523	0.522	0.62	0.547	0.682	1.069	0.544	0.566	0.604	0.697
TRINITY_DN2623_c1_g1_i3_orf1	COPII coat assembly protein sec16-like [Ostrinia furnacalis]	1	107	10.9	10.87	7	1	1	Medium	1	1.076	0.962	0.892	0.881	0.858	0.921	0.823	0.75	0.88	0.757	0.933	1.442	1.468	1.386
TRINITY_DN33089_c0_g1_i1_orf1	nucleoporin NDC1 [Ostrinia furnacalis]	1	562	64.7	8.56	2	1	1	Medium	1	0.93	0.81	0.639	0.676	0.751	0.914	0.882	0.925	0.605	0.598	0.96	1.3	1.2	1.472
TRINITY_DN19286_c0_g1_i1_orf1	signal recognition particle 9 kDa protein [Ostrinia furnacalis]	1	78	9.4	8.84	18	1	1	Medium	1	0.953	1.128	0.572	0.65	0.473	0.501	0.667	0.315	0.542	0.582	0.574	0.666	0.478	0.531
TRINITY_DN4016_c0_g1_i1_orf1	60S acidic ribosomal protein P0 [Homo sapiens]	1	317	34.3	5.97	4	1	1	Medium	1	0.886	1.005	0.77	0.668	0.679	0.744	0.679	0.677	0.678	0.572	0.648	0.34	0.367	0.376
TRINITY_DN3482_c0_g2_i1_orf1	transcription elongation factor B polypeptide 3-like isoform X2 [Ostrinia furnac	1	802	89.7	9.26	1	1	1	Medium	1	1.092	1.078	0.938	1.222	0.905	0.705	0.875	1.018	0.842	0.651	0.744	1.087	1.134	1.045
TRINITY_DN56164_c0_g1_i1_orf1	hypothetical protein evm_010164 [Chilo suppressalis]	1	286	32.8	9.42	2	1	1	Medium	1	1.128	1.041	0.871	0.844	0.901	1.053	1.457	1.502	1.095	1.021	2.034	1.793	1.589	1.357
TRINITY_DN48097_c0_g1_i1_orf1	unnamed protein product [Homo sapiens]	1	608	70.4	6.86	1	1	1	Medium	1	0.861	1.047	1.069	0.62	1.046	1.099	1.029	1.149	1.164	0.844	0.656	0.419	0.443	0.468
TRINITY_DN23266_c0_g2_i1_orf1	medium-chain acyl-CoA ligase ACSF2, mitochondrial [Chelonus insularis]	1	580	65	8.13	1	2	1	Medium	1	1.059	1.053	0.283	0.344	0.374	0.294	0.3	0.292	0.296	0.267	0.314	0.31	0.331	0.326
TRINITY_DN110534_c0_g1_i3_orf1	unnamed protein product [Euphydryas editha]	1	1088	124.4	8.57	1	1	1	Medium	1	1.119	1.013	0.806	0.724	0.896	0.651	0.658	0.682	0.88	0.783	0.63	1.601	1.442	1.194
TRINITY_DN61042_c0_g2_i2_orfp1	TRINITY_DN61042_c0_g2_i2_m.5292 TRINITY_DN61042_c0_g2::TRINITY_DN61(	1	62	7.1	7.77	10	1	1	Medium	1	1.457	1.411	3.154	2.945	3.082	1.757	1.609	2.054	2.818	2.463	2.71	1.764	1.61	1.623
TRINITY_DN2243_c0_g1_i4_orf1	WD repeat-containing protein 75 [Ostrinia furnacalis]	1	893	99.1	6.57	1	1	1	Medium	1	1.073	1.031	0.333	0.306	0.327	0.287	0.367	0.305	0.267	0.268	0.284	0.235	0.268	0.293
TRINITY_DN10070_c0_g1_i1_orf1	40S ribosomal protein SA isoform 1 [Homo sapiens]	1	295	32.8	4.87	5	1	1	Medium	1	1.008	1.018	1.434	1.459	1.219	1.526	1.459	1.576	1.534	1.395	2.362	0.811	0.955	0.934
TRINITY_DN12323_c0_g2_i2_orf1	polyribonucleotide nucleotidyltransferase 1, mitochondrial [Ostrinia furnacalis]	1	755	82.8	8	1	1	1	Medium	1	1.11	0.982	0.477	0.456	0.537	0.555	0.579	0.585	0.433	0.409	0.499	0.476	0.566	0.457
TRINITY_DN21787_c0_g1_i1_orf1	thioredoxin-like protein 4A [Bombyx mori]	1	142	16.8	5.85	8	1	1	Medium	1	0.693	0.845	1.146	1.056	1.056	0.806	1.163	0.976	1.082	1.524	1.011	1.306	0.941	1.334
TRINITY_DN7102_c0_g1_i5_orf1	protein wings apart-like [Ostrinia furnacalis]	1	1078	119.6	7.53	1	1	1	Medium	1	0.736	0.779	1.148	1.293	2.183	1.946	2.224	1.954	2.236	1.864	2.263	2.361	2.714	2.47
TRINITY_DN47731_c0_g1_i2_orf1	nucleolar GTP-binding protein 2 [Ostrinia furnacalis]	1	269	30.3	5.62	4	1	1	Medium	1	1.193	0.91	0.092	0.18	0.164	0.201	0.129	0.149	0.162	0.102	0.255	0.419	0.412	0.423
TRINITY_DN101922_c0_g1_i1_orf1	PREDICTED: uncharacterized protein LOC103569676 [Microplitis demolitor]	1	290	33.4	10.4	4	1	1	Medium	1	0.895	0.871	0.638	0.532	0.602	0.652	0.743	0.652	0.638	0.758	0.805	0.672	0.711	0.637
TRINITY_DN46216_c0_g3_i1_orf1	unnamed protein product, partial [Brenthis ino]	1	85	9.6	4.41	11	1	1	Medium	1	0.908	0.912	1.188	1.245	1.144	1.376	1.353	1.435	0.721	0.624	0.729	0.22	0.169	0.227
TRINITY_DN52861_c0_g1_i1_orf1	protein MAK16 homolog A [Ostrinia furnacalis]	1	300	35.4	5.86	2	1	1	Medium	1	1.034	1.016	0.404	0.376	0.477	0.326	0.387	0.436	0.388	0.322	0.364	0.481	0.453	0.325
TRINITY_DN4460_c0_g1_i1_orf1	uncharacterized protein LOC114364170 [Ostrinia furnacalis]	1	381	40.4	5.76	2	1	1	Medium	1	1.187	1.042	1.212	1.108	1.148	1.139	1.149	2.155	0.878	0.966	1.076	1.207	1.013	0.941
TRINITY_DN5531_c7_g1_i2_orf1	LOW QUALITY PROTEIN: tyrosine-protein phosphatase CDC14 homolog [Ostri	1	699	78.7	9.55	3	1	1	Medium	1	0.996	1.114	1.545	1.247	1.545	1.549	1.208	2.167	1.648	3.688	1.293	1.303	1.247	1.413
TRINITY_DN101_c0_g1_i4_orf1	disco-interacting protein 2 [Melitaea cinxia]	1	1598	174.3	7.75	1	1	1	Medium	1	1.041	1.017	1.174	1.172	1.1	1.322	1.299	1.381	1.799	1.82	1.914	1.347	1.176	1.421
TRINITY_DN23734_c0_g1_i1_orf1	histone-lysine N-methyltransferase SMYD3 [Ostrinia furnacalis]	1	492	56.4	7.06	1	1	1	Medium	1	0.778	0.948	0.493	0.364	0.328	0.261	0.533	0.419	0.328	0.195	0.247	0.359	0.475	0.457
TRINITY_DN16174_c0_g1_i2_orf1	LOW QUALITY PROTEIN: ATP-dependent RNA helicase SUV3 homolog, mitoch	1	406	45.5	6.79	1	1	1	Medium	1	0.927	0.943	0.531	0.642	0.677	0.585	0.546	0.62	0.582	0.468	0.465	0.668	0.586	0.577
TRINITY_DN3014_c0_g1_i4_orf1	putative inorganic phosphate cotransporter isoform X1 [Ostrinia furnacalis]	1	520	56.6	8.7	1	1	1	Medium	1	0.91	1.024	0.378	0.373	0.427	0.39	0.351	0.582	0.385	0.342	0.418	0.28	0.249	0.245
TRINITY_DN13999_c0_g1_i4_orf1	LOW QUALITY PROTEIN: succinate--hydroxymethylglutarate CoA-transferase-	1	417	46	8.63	3	1	1	Medium	1	0.866	0.91	0.685	0.588	0.765	0.653	0.773	0.678	0.746	0.978	0.969	0.848	0.887	0.881
TRINITY_DN5991_c0_g1_i6_orf1	uncharacterized protein LOC114357071 [Ostrinia furnacalis]	1	514	56.7	9.32	2	1	1	Medium	1	1.168	1.278	0.74	0.813	0.969	1.228	1.195	1.195	0.556	0.618	0.693	0.612	0.763	0.717
TRINITY_DN5210_c0_g1_i3_orf1	uncharacterized protein LOC114358376 isoform X2 [Ostrinia furnacalis]	1	1038	118.7	6.34	1	1	1	Medium	1	1.037	1.095	0.849	0.832	0.72	0.919	1.072	0.945	0.755	0.652	0.726	0.637	0.846	0.738
TRINITY_DN19160_c0_g1_i1_orf1	alkyldihydroxyacetonephosphate synthase [Ostrinia furnacalis]	1	623	69.6	7.9	1	1	1	Medium	1	1.074	1.144	0.132	0.197	0.243	0.147	0.186	0.148	0.157	0.161	0.219	0.811	0.88	0.819
TRINITY_DN26879_c0_g1_i1_orf1	hypothetical protein O3G_MSEX014401 [Manduca sexta]	1	840	97.8	8.94	1	1	1	Medium	1	1.045	0.89	2.279	2.34	2.183	1.789	1.214	3.349	1.975	1.324	2.063	1.408	0.879	0.926
TRINITY_DN74037_c0_g5_i1_orf1	hypothetical protein JYU34_010754 [Plutella xylostella]	1	229	26.9	9.76	3	1	1	Medium	1	1.738	1.31	1.727	1.733	1.99	2.596	1.855	2.254	2.166	1.633	1.953	1.537	1.601	1.367
TRINITY_DN123139_c0_g1_i1_orfp	TRINITY_DN123139_c0_g1_i1_m.79879 TRINITY_DN123139_c0_g1::TRINITY_DN	1	67	7.6	8.18	9	1	1	Medium	1	0.978	1.072	0.612	0.627	0.588	0.521	0.531	0.49	0.387	0.47	0.465	0.516	0.527	0.573
TRINITY_DN5459_c0_g1_i1_orf1	protein takeout-like isoform X2 [Ostrinia furnacalis]	1	196	21.2	8.62	4	1	1	Medium	1	1.035	0.974	0.233	0.192	0.268	0.269	0.169	0.235	0.265	0.183	0.27	0.755	0.749	0.649
TRINITY_DN10220_c1_g1_i7_orf1	uncharacterized protein LOC124645895 isoform X2 [Helicoverpa zea]	1	462	52.5	7.2	2	1	1	Medium	1	0.99	0.983	2.235	1.818	2.155	2.059	1.997	2.22	2.012	1.7	1.317	2.419	2.427	2.507
TRINITY_DN11856_c0_g1_i4_orf1	cartilage-associated protein-like [Ostrinia furnacalis]	1	367	42.8	5.16	2	1	1	Medium	1	1.077	1.064	0.754	0.82	0.567	0.852	0.823	0.704	0.827	1.129	1.333	1.046	1.143	1.199
TRINITY_DN3463_c0_g1_i2_orf1	EH domain-binding protein 1-like protein 1 [Ostrinia furnacalis]	1	1043	117.6	5.96	1	1	1	Medium	1	0.94	0.605	0.826	0.858	1.217	1.157	0.818	0.805	0.785	0.834	0.84	0.638	0.963	1.038
TRINITY_DN42185_c0_g1_i7_orf1	ADP-ribosylation factor-like protein 13B isoform X2 [Ostrinia furnacalis]	1	494	56.2	9.17	1	1	1	Medium	1	1.063	1.2	1.249	1.334	1.341	1.214	1.218	1.16	0.911	0.814	1.109	1.031	1.058	1.016
TRINITY_DN20442_c0_g2_i1_orf1	hypothetical protein evm_008218 [Chilo suppressalis]	1	122	13.7	11.56	7	2	1	Medium	1	0.827	1.046	1.496	1.389	1.602	5.673	5.108	5.481	3.591	3.004	2.972	5.788	6.782	5.391
TRINITY_DN804_c0_g1_i7_orf1	hypothetical protein HF086_004695 [Spodoptera exigua]	1	113	12.3	8.53	5	1	1	Medium	1	1.167	1.089	1.891	1.958	1.947	1.65	1.376	1.685	1.133	1.109	1.174	0.77	0.836	0.839
TRINITY_DN24410_c0_g2_i1_orf1	spermine oxidase-like isoform X3 [Venturia canescens]	1	481	55.5	6.3	1	1	1	Medium	1	1.061	0.871	1.201	1.409	1.455	1.381	1.279	1.072	0.99	2.032	1.093	0.866	0.821	1.055
TRINITY_DN99673_c0_g1_i1_orf1	PREDICTED: pistil-specific extensin-like protein isoform X2 [Microplitis demolit	1	246	27.3	7.12	4	1	1	Medium	1	0.584	0.674	0.581	0.797	0.938	0.954	2.075	1.359	0.875	1.001	0.76	0.315	0.408	0.414
TRINITY_DN4782_c0_g1_i1_orf1	patched domain-containing protein 3-like [Ostrinia furnacalis]	1	948	107.4	5.78	1	1	1	Medium	1	0.923	0.989	0.965	0.882	1.132	0.907	0.682	0.939	0.793	0.689	0.765	1.055	1.215	1.229
TRINITY_DN69049_c0_g2_i1_orf1	membrane alanyl aminopeptidase-like [Ostrinia furnacalis]	1	277	30.8	4.87	5	1	1	Medium	1	0.946	1.056	0.554	0.631	0.623	0.38	0.537	0.446	0.612	0.699	0.683	0.681	0.701	0.692
TRINITY_DN64616_c0_g1_i1_orf1	uncharacterized protein LOC114366101 [Ostrinia furnacalis]	1	174	18.6	5.45	9	1	1	Medium	1	1.085	1.107	1.069	0.989	0.978	1.294	1.532	1.153	1.618	1.086	1.443	2.241	2.163	2.128
TRINITY_DN15040_c0_g4_i1_orf1	hypothetical protein O3G_MSEX013320 [Manduca sexta]	1	284	32	9.95	4	1	1	Medium	1	1.171	1.268	1.453	1.181	1.309	1.319	1.71	1.6	1.253	1.463	1.434	1.05	1.14	1.188
TRINITY_DN147596_c0_g1_i1_orf1	activator of basal transcription 1 [Diachasma alloeum]	1	212	25.4	10.05	3	1	1	Medium	1	0.973	0.974	0.499	0.517	0.723	0.671	0.685	0.648	0.556	0.51	0.366	0.474	0.517	0.485
TRINITY_DN25733_c0_g1_i3_orf1	polyphosphoinositide phosphatase [Ostrinia furnacalis]	1	906	103	8.62	1	2	1	Medium	1	0.997	0.972	1.284	1.061	1.118	1.321	1.166	1.351	1.26	1.082	1.132	1.129	1.185	1.145
TRINITY_DN42275_c0_g1_i1_orfp1	TRINITY_DN42275_c0_g1_i1_m.44265 TRINITY_DN42275_c0_g1::TRINITY_DN42	1	75	7.9	6.98	16	1	1	Medium	1	1.108	0.721	1.774	1.983	1.908	1.724	1.714	1.731	3.073	2.476	3.874	2.803	3.025	2.006
TRINITY_DN6639_c0_g1_i4_orf1	nuclear hormone receptor FTZ-F1 beta-like [Ostrinia furnacalis]	1	761	83.5	7.18	2	1	1	Medium	1	1.145	0.974	1.285	1.206	1.045	1.027	1.022	1.036	1.053	0.702	0.909	1.11	1.111	0.864
TRINITY_DN12666_c0_g1_i2_orf1	sodium- and chloride-dependent GABA transporter 1 [Ostrinia furnacalis]	1	605	68.6	7.74	2	1	1	Medium	1	1.134	1.195	1.575	1.464	1.514	1.437	1.421	1.352	1.612	1.736	1.567	1.188	1.232	1.08
TRINITY_DN7670_c0_g1_i1_orf1	striatin-interacting protein 1 [Ostrinia furnacalis]	1	820	94.8	5.81	1	1	1	Medium	1	1.068	0.969	0.615	0.58	0.787	0.636	0.689	0.763	0.62	0.707	0.677	0.963	0.985	1.005
TRINITY_DN8384_c0_g1_i1_orf1	hypothetical protein HN011_011041 [Eciton burchellii]	1	80	9.1	12.16	16	1	1	Medium	1	0.68	7.953	0.688	0.952	1.08	1.976	0.761	3.326	8.808	2.426	0.121	10.198	1.599	0.736
TRINITY_DN7213_c0_g1_i2_orf1	probable ATP-dependent RNA helicase CG8611 [Ostrinia furnacalis]	1	948	106.1	9.23	1	1	1	Medium	1	0.928	0.959	0.36	0.418	0.386	0.34	0.344	0.315	0.297	0.311	0.342	0.402	0.398	0.346
TRINITY_DN9354_c0_g1_i7_orf1	hypothetical protein evm_012205 [Chilo suppressalis]	1	293	31.1	6.64	3	1	1	Medium	1	0.98	0.864	0.897	0.652	0.792	0.616	0.68	0.628	0.712	0.812	0.799	1.235	1.159	1.146

TRINITY_DN4612_c0_g1_i1_orf1	uncharacterized protein LOC114362092 [Ostrinia furnacalis]	1	610	70.8	7.11	1	1	1	Medium	1	1.006	0.89	0.102	0.095	0.178	0.195	0.191	0.187	0.222	0.185	0.204	0.141	0.11	0.126
TRINITY_DN47677_c0_g1_i1_orf1	unnamed protein product [Euphydryas editha]	1	309	36.1	8.88	3	1	1	Medium	1	0.864	1.069	1.074	1.145	1.377	1.357	1.69	1.142	1.226	0.88	1.158	1.375	1.705	1.38
TRINITY_DN1504_c0_g1_i1_orf1	uncharacterized protein LOC114352862 [Ostrinia furnacalis]	1	742	83.4	7.39	1	1	1	Medium	1	1.136	0.914	0.658	0.749	0.575	0.521	0.576	0.57	0.362	0.387	0.532	1.132	1.104	1.152
TRINITY_DN10877_c0_g1_i1_orf1	spodomicin-like [Ostrinia furnacalis]	1	64	6.9	6.73	19	1	1	Medium	1	1.278	0.754	19.726	22.526	14.44	3.708	3.645	4.779	8.217	7.054	10.829	3.104	2.969	3.322
TRINITY_DN8964_c0_g1_i4_orf1	hypothetical protein evm_010115 [Chilo suppressalis]	1	410	45.3	9.13	5	2	1	Medium	1	1.119	1.028	1.011	0.996	1.09	1.036	0.943	0.941	0.74	0.684	0.783	0.603	0.435	0.555
TRINITY_DN144258_c0_g1_i1_orf1	PREDICTED: enhancer of rudimentary homolog [Microplitis demolitor]	1	103	12.1	6.29	10	1	1	Medium	1	0.759	0.957	0.629	0.71	0.546	0.389	0.455	0.488	0.414	0.271	0.462	0.955	0.8	0.887
TRINITY_DN146493_c0_g1_i1_orf1	anaphase-promoting complex subunit 1 [Chelonus insularis]	1	179	21	6.34	4	1	1	Medium	1	1.07	0.957	0.892	0.903	0.866	0.688	0.717	0.894	0.608	0.599	0.862	1.069	0.936	0.922
TRINITY_DN12317_c0_g1_i1_orf1	vacuolar protein sorting-associated protein 41 homolog [Ostrinia furnacalis]	1	851	97.7	5.88	1	1	1	Medium	1	1.002	1.071	1.291	1.225	1.236	1.135	1.353	1.143	1.247	1.039	1.075	1.483	1.188	1.32
TRINITY_DN12686_c0_g1_i4_orf1	maltase A1-like [Ostrinia furnacalis]	1	620	69.6	5.49	1	1	1	Medium	1	0.939	1.077	1.704	1.667	1.32	1.344	1.198	0.986	1.284	1.735	1.342	1.307	1.184	1.434
TRINITY_DN9998_c0_g1_i2_orf1	GSCOCG00008521001-RA-CDS [Cotesia congregata]	1	323	34.8	8.53	2	1	1	Medium	1	1.052	1.213	1.251	1.2	1.206	1.259	1.088	1.141	1.157	0.995	1.192	1.031	1.053	0.907
TRINITY_DN51934_c0_g2_i1_orf1	SCAN domain-containing protein 3-like [Pieris napi]	1	159	17.4	8.85	4	1	1	Medium	1	0.995	1.044	0.501	0.576	0.591	0.634	0.644	0.608	0.482	0.426	0.548	0.42	0.421	0.379
TRINITY_DN95544_c0_g2_i1_orf1	tubulin-specific chaperone D [Chelonus insularis]	1	1153	131	6.67	2	1	1	Medium	1	0.923	0.85	0.977	1.106	1.205	1.23	1.624	0.926	1.215	2.134	1.247	0.68	0.734	1.006
TRINITY_DN28376_c0_g1_i15_orfp	TRINITY_DN28376_c0_g1_i15_m.40022 TRINITY_DN28376_c0_g1::TRINITY_DN2	1	272	31.3	11.18	2	1	1	Medium	1	0.855	0.998	0.853	0.89	0.706	0.884	0.913	0.806	0.592	0.611	0.748	0.857	0.708	0.711
TRINITY_DN3196_c0_g1_i1_orf1	organic cation transporter-like protein [Ostrinia furnacalis]	1	486	54	8.19	5	1	1	Medium	1	0.938	0.976	1.561	1.487	1.572	2.027	1.837	1.873	1.6	1.802	1.583	1.353	1.223	1.349
TRINITY_DN8008_c0_g1_i6_orf1	uncharacterized protein LOC114357965 isoform X1 [Ostrinia furnacalis]	1	4508	506.7	7.62	0	1	1	Medium	1	1.141	1.082	3.195	2.991	2.981	3.789	3.691	3.555	4.918	4.642	4.52	1.688	1.394	1.501
TRINITY_DN1013_c0_g1_i3_orf1	TELO2-interacting protein 1 homolog isoform X2 [Ostrinia furnacalis]	1	1110	124.9	6.24	1	1	1	Medium	1	1.141	1.003	0.811	1.049	0.908	0.24	0.183	0.315	0.369	0.307	0.427	0.702	0.858	0.862
TRINITY_DN2425_c0_g1_i1_orf1	thyroid receptor-interacting protein 11 [Ostrinia furnacalis]	1	2308	262.5	7.96	1	1	1	Medium	1	1.092	1.079	0.418	0.582	0.453	0.611	0.423	0.377	0.196	0.247	0.26	0.307	0.271	0.331
TRINITY_DN6841_c0_g2_i1_orf1	uncharacterized protein LOC114364302, partial [Ostrinia furnacalis]	1	126	13.6	4.87	10	1	1	Medium	1	0.778	0.769	0.947	0.904	0.826	0.975	0.728	0.815	0.878	0.962	0.709	0.872	0.902	0.839
TRINITY_DN78546_c0_g5_i1_orf1	kinesin-like protein KIF13A isoform X9 [Cephus cinctus]	1	300	34.4	6.65	2	1	1	Medium	1	0.688	0.775	0.403	0.607	0.48	0.482	0.39	0.386	0.344	0.321	0.223	0.364	0.562	0.431
TRINITY_DN87170_c0_g1_i3_orf1	uncharacterized protein LOC114360175 [Ostrinia furnacalis]	1	264	29.2	5.57	2	1	1	Medium	1	1.011	0.946	0.783	0.783	0.773	0.623	0.596	0.614	0.663	0.62	0.685	0.999	0.996	0.986
TRINITY_DN129835_c0_g1_i2_orf1	alpha-tocopherol transfer protein-like [Chelonus insularis]	1	305	35.4	8.34	2	1	1	Medium	1	0.927	0.703	0.881	0.87	1.043	0.533	0.451	0.401	0.677	0.569	0.372	8.168	8.09	8.084
TRINITY_DN5770_c0_g1_i4_orf1	rotatin-like [Ostrinia furnacalis]	1	1018	111.2	7.9	1	1	1	Medium	1	1.043	1.217	2.607	2.681	2.344	0.677	0.867	0.682	3.758	0.814	0.683	1.134	1.035	0.87
TRINITY_DN14219_c0_g1_i7_orf1	nuclear pore complex protein Nup133-like [Ostrinia furnacalis]	1	395	44	5.14	3	1	1	Medium	1	0.985	1.01	0.908	0.928	1.08	0.971	1.018	0.946	1.095	0.828	0.594	1.098	1.187	1.089
TRINITY_DN5697_c0_g1_i1_orf1	GPI ethanolamine phosphate transferase 2-like [Ostrinia furnacalis]	1	798	89.9	6.9	1	1	1	Medium	1	1.037	0.98	0.533	0.64	0.599	0.414	0.507	0.462	0.422	0.447	0.519	0.745	0.74	0.701
TRINITY_DN53462_c0_g1_i1_orf1	uncharacterized protein LOC118072968 isoform X1 [Chelonus insularis]	1	612	69.1	7.42	1	1	1	Medium	1	1.017	1.028	0.75	0.796	0.845	0.36	0.533	0.508	0.396	0.309	0.382	0.361	0.268	0.26
TRINITY_DN38498_c0_g3_i1_orf1	unnamed protein product [Parnassius apollo]	1	315	36.4	9.1	3	1	1	Medium	1	0.693	1.061	0.554	0.453	0.298	0.536	0.364	0.519	0.398	0.169	0.16	0.164	0.154	0.155
TRINITY_DN15338_c0_g1_i7_orf1	methyltransferase-like protein 17, mitochondrial [Ostrinia furnacalis]	1	439	50.6	9.09	2	1	1	Medium	1	0.722	0.713	0.302	0.411	0.317	0.298	0.364	0.458	0.303	1.452	0.389	0.432	0.407	0.478
TRINITY_DN39837_c0_g1_i1_orf1	unnamed protein product [Plutella xylostella]	1	597	67.1	8.81	5	1	1	Medium	1	0.908	1.036	1.45	1.712	1.659	1.328	1.828	1.304	1.328	1.522	2.006	1.044	0.836	1.012
TRINITY_DN8603_c0_g1_i1_orf1	adenosine kinase 2 isoform X2 [Cotesia glomerata]	1	345	38.5	6.19	7	1	1	Medium	1	0.733	0.774	0.422	0.27	0.42	0.507	0.635	0.721	0.615	0.601	0.411	0.312	0.261	0.488
TRINITY_DN82311_c0_g1_i1_orf1	pleckstrin homology-like domain family B member 1 isoform X2 [Ostrinia furna	1	69	7.8	9.52	20	1	1	Medium	1	0.544	1.361	0.64	0.866	0.632	0.447	0.616	0.642	0.565	0.456	0.396	0.891	0.891	0.993
TRINITY_DN76307_c0_g1_i1_orf1	PREDICTED: quinone oxidoreductase-like protein 2 homolog [Microplitis demo	1	269	29.6	5.91	4	1	1	Medium	1	1.059	0.98	1.072	1.207	1.072	1.652	1.595	1.459	1.495	1.256	1.385	1.135	1.214	0.998
TRINITY_DN79803_c0_g1_i7_orf1	dnaJ homolog subfamily C member 22 [Ostrinia furnacalis]	1	373	44.1	9.09	2	1	1	Medium	1	0.966	0.805	0.677	0.617	0.718	0.705	0.706	0.835	0.6	0.543	0.52	1.109	1.06	0.86
TRINITY_DN56270_c0_g1_i1_orf1	PREDICTED: putative elongator complex protein 1 [Microplitis demolitor]	1	1013	116.8	6.81	1	1	1	Medium	1	1.093	0.988	1.068	0.805	0.831	0.814	0.254	0.542	0.922	0.745	0.826	0.526	0.586	0.478
TRINITY_DN106856_c0_g1_i1_orf1	serine/threonine-protein kinase RIO1 [Cephus cinctus]	1	540	62.9	6.52	2	1	1	Medium	1	0.934	1.065	1.072	1.117	0.935	1.177	0.874	1.217	1.284	1.032	1.226	0.901	1.023	0.975
TRINITY_DN81258_c0_g1_i2_orf1	jg27820 [Pararge aegeria aegeria]	1	1261	145.6	8.68	0	1	1	Medium	1	1.009	1.011	0.961	0.876	0.91	1.13	0.996	1.141	1.023	0.784	1.058	0.634	0.703	0.475
TRINITY_DN4567_c0_g3_i1_orf1	hypothetical protein JYU34_019391 [Plutella xylostella]	1	874	100.3	9.07	1	1	1	Medium	1	0.839	0.772	1.253	1.223	1.124	0.929	1.18	1.094	1.254	1.18	0.946	1.128	0.956	0.783
TRINITY_DN34115_c0_g1_i1_orf1	transcription factor 23 [Ostrinia furnacalis]	1	120	13.9	4.84	5	1	1	Medium	1	0.943	0.919	1.329	1.392	1.238	1.189	1.242	1.459	1.512	1.562	1.262	1.04	1.25	1.487
TRINITY_DN3275_c0_g2_i3_orf1	hypothetical protein B5X24_HaOG216046 [Helicoverpa armigera]	1	25	2.8	4.75	36	1	1	Medium	1	0.513	0.316	5.49	5.69	6.163	4.458	4.325	3.132	2.48	1.718	3.33	1.661	1.78	2.372
TRINITY_DN47151_c0_g1_i1_orf1	unnamed protein product [Danaus chrysippus]	1	142	15.8	9.41	6	1	1	Medium	1	1.128	1.186	1.655	1.449	1.558	1.675	1.419	1.793	1.233	1.11	1.095	0.893	0.847	1.02
TRINITY_DN76377_c0_g1_i1_orf1	uncharacterized protein LOC111357764, partial [Spodoptera litura]	1	237	27.4	8.27	4	1	1	Medium	1	1.032	0.997	0.367	0.442	0.371	0.505	0.494	0.487	0.292	0.271	0.326	0.958	0.991	0.957
TRINITY_DN22513_c0_g1_i4_orf1	DNA-directed RNA polymerase II subunit RPB1 [Ostrinia furnacalis]	1	365	40.9	7.46	5	1	1	Medium	1	1.051	0.864	1.829	2.232	2.199	2.124	2.254	1.907	1.735	1.792	2.226	0.784	0.866	0.717
TRINITY_DN87603_c0_g2_i1_orf1	40S ribosomal protein S3-3, partial [Trichinella patagoniensis]	1	70	7.9	9.8	13	1	1	Medium	1	1.271	0.964	0.457	0.593	0.594	0.819	0.964	0.882	0.521	0.564	0.824	0.869	1.013	1.059
TRINITY_DN60358_c0_g1_i3_orf1	transcription termination factor 5, mitochondrial-like [Ostrinia furnacalis]	1	535	62.6	9.17	1	1	1	Medium	1	0.89	1.047	1.409	1.282	1.5	0.924	1.365	1.304	1.572	1.439	1.345	1.28	1.843	1.119
TRINITY_DN17791_c0_g1_i1_orf1	hypothetical protein EVAR_21272_1 [Eumeta japonica]	1	59	6.8	6.51	22	1	1	Medium	1	0.843	1.032	1.045	0.87	0.885	1.063	0.969	0.996	0.996	0.994	0.979	1.08	1.141	1.436
TRINITY_DN147691_c0_g1_i1_orf1	WD repeat-containing protein 46 [Orussus abietinus]	1	514	57.6	9.64	3	1	1	Medium	1	0.958	0.924	1.239	1.151	1.226	1.312	1.527	1.258	1.178	1.134	1.326	0.885	0.93	0.81
TRINITY_DN3862_c0_g1_i7_orf1	venom acid phosphatase Acph-1-like [Ostrinia furnacalis]	1	388	43.7	5.35	2	1	1	Medium	1	1.023	1.004	0.127	0.136	0.19	0.164	0.154	0.153	0.133	0.101	0.13	0.143	0.169	0.135
TRINITY_DN144342_c0_g1_i1_orfp	TRINITY_DN144342_c0_g1_i1_m.83164 TRINITY_DN144342_c0_g1::TRINITY_DN	1	112	13.1	9.11	6	1	1	Medium	1	1.021	0.974	0.83	0.766	0.908	1.069	1.131	1.375	1.058	0.701	0.586	0.751	0.715	0.787
TRINITY_DN111621_c0_g3_i1_orf1	Serine proteinase stubble [Eumeta japonica]	1	169	18.5	7.39	4	1	1	Medium	1	1.058	1.268	1.015	0.839	0.842	0.98	0.947	1.006	1.11	0.974	1.456	2.121	2.121	2.177
TRINITY_DN7900_c0_g1_i4_orf1	uncharacterized protein LOC114366119 [Ostrinia furnacalis]	1	123	13.5	8.88	9	1	1	Medium	1	0.92	0.703	3.987	3.323	3.48	4.16	3.385	4.725	5.919	5.255	4.201	4.937	4.389	5.623
TRINITY_DN62141_c0_g1_i5_orf1	USP6 N-terminal-like protein isoform X1 [Ostrinia furnacalis]	1	610	70	7.64	1	1	1	Medium	1	1.175	1.159	1.033	1.072	1.021	1.047	1.02	1.03	1.025	0.893	1.046	0.804	1.087	0.876
TRINITY_DN4123_c0_g1_i1_orf1	uncharacterized protein LOC114355030 [Ostrinia furnacalis]	1	139	15.8	5.31	4	1	1	Medium	1	0.748	0.871	1.457	2.048	1.926	1.997	1.627	2.094	1.247	1.155	1.314	0.804	0.748	0.993
TRINITY_DN48694_c0_g1_i1_orfp1	TRINITY_DN48694_c0_g1_i1_m.75338 TRINITY_DN48694_c0_g1::TRINITY_DN48	1	83	9.1	4.25	13	1	1	Medium	1	0.815	0.867	3.795	4.173	4.067	0.258	0.155	0.496	3.025	1.069	1.704	3.523	3.429	3.237
TRINITY_DN15988_c0_g1_i1_orf1	uncharacterized protein LOC114351163 [Ostrinia furnacalis]	1	910	98.4	8.34	1	1	1	Medium	1	1.085	1.127	1.071	0.786	1.016	0.735	0.992	1.216	0.76	0.876	0.729	0.758	0.993	0.77
TRINITY_DN89613_c0_g1_i13_orf1	PREDICTED: uncharacterized protein LOC106137743 [Amyelois transitella]	1	1555	176.4	8.34	0	1	1	Medium	1	0.937	0.984	0.849	0.852	0.75	0.773	0.852	0.764	0.594	0.548	0.696	0.568	0.498	0.493
TRINITY_DN830_c0_g1_i4_orf1	eIF-2-alpha kinase GCN2 [Ostrinia furnacalis]	1	1382	155.6	6.73	1	1	1	Medium	1	1.041	0.781	1.187	1.083	1.207	1.109	1.281	1.38	1.31	1.325	0.951	1.09	0.983	1.17
TRINITY_DN6025_c0_g2_i1_orfp1	TRINITY_DN6025_c0_g2_i1_m.7749 TRINITY_DN6025_c0_g2::TRINITY_DN6025_	1	105	11.6	7.55	10	1	1	Medium	1	0.611	0.975	4.397	4.786	4.733	5.022	3.999	3.444	3.441	3.037	4.525	2.373	2.661	1.646
TRINITY_DN6710_c0_g1_i6_orf1	multiple C2 and transmembrane domain-containing protein-like [Ostrinia furn	1	614	69.9	9.07	1	1	1	Medium	1	0.997	1.09	0.364	0.65	0.406	0.254	0.382	0.286	0.332	0.347	0.304	0.666	0.953	0.801
TRINITY_DN58013_c0_g1_i6_orf1	uncharacterized protein LOC114355413 isoform X1 [Ostrinia furnacalis]	1	655	71.1	9.36	2	1	1	Medium	1	1.134	1.109	1.206	1.266	1.11	1.187	1.448	1.502	1.31	1.056	1.306	1.256	1.124	1.143
TRINITY_DN40434_c0_g1_i2_orf1	deoxyribodipyrimidine photo-lyase [Ostrinia furnacalis]	1	523	60.4	8.72	1	1	1	Medium	1	0.963	0.961	1.488	1.998	1.874	1.251	1.395	1.374	1.129	0.883	1.025	0.885	0.825	0.962
TRINITY_DN434_c0_g1_i4_orf1	uncharacterized protein LOC126367148 [Pectinophora gossypiella]	1	714	79.9	8.68	2	1	1	Medium	1	0.884	1.002	0.585	0.596	0.8	0.877	0.532	0.87	0.583	0.595	0.752	0.411	0.435	0.523
TRINITY_DN14269_c0_g1_i5_orf1	probable DNA mismatch repair protein Msh6 isoform X1 [Ostrinia furnacalis]	1	619	69.9	7.94	2	1	1	Medium	1	0.996	1.17	1.258	1.284	1.354	1.338	1.193	1.259	1.164	1.291	1.105	1.037	1.348	1.245
TRINITY_DN6602_c0_g1_i4_orf1	PREDICTED: E3 ubiquitin-protein ligase RNF181-like [Amyelois transitella]	1	147	17.2	5.88	5	1	1	Medium	1	0.966	0.879	0.943	0.77	0.838	0.822	0.715	0.73	0.64	0.51	0.645	0.897	0.942	0.915
TRINITY_DN23962_c0_g1_i3_orf1	uncharacterized protein LOC124629606 [Helicoverpa zea]	1	1790	200.9	8.43	1	1	1	Medium	1	1.083	1.117	0.966	0.769	0.893	0.967	0.928	1.054	0.739	0.901	0.883	0.98	0.813	0.776
TRINITY_DN5670_c0_g1_i2_orf1	DNA polymerase alpha subunit B [Ostrinia furnacalis]	1	581	63.6	6.25	1	1	1	Medium	1	1.26	1.135	1.018	0.986	0.953	1.021	1.171	0.91	0.655	0.665	0.701	0.854	0.923	0.892
TRINITY_DN135846_c0_g1_i1_orf1	zinc finger protein 253-like [Ostrinia furnacalis]	1	543	63.2	8.81	2	1	1	Medium	1	1.141	1.492	0.999	0.916	0.906	1.119	1.19	1.304	1.035	1.523	0.838	1.008	1.201	1.26
TRINITY_DN33346_c0_g1_i1_orf1	PREDICTED: U6 snRNA-associated Sm-like protein LSm3 [Papilio xuthus]	1	102	11.7	4.88	14	1	1	Medium	1	0.952	0.857	2.711	2.568	2.53	3.089	3.232	3.138	2.772	2.717	3.171	2.158	2.301	2.147
TRINITY_DN61335_c0_g2_i1_orf1	uncharacterized protein LOC114352149 [Ostrinia furnacalis]	1	568	65.6	7.11	1	1	1	Medium	1	0.963	1.11	1.637	1.424	1.465	1.382	1.306	1.322	1.329	1.197	1.425	0.93	1.135	0.968
TRINITY_DN16127_c0_g1_i2_orf1	transcription intermediary factor 1-alpha [Ostrinia furnacalis]	1	375	41.2	7.4	3	1	1	Medium	1	0.679	0.681	1.159	0.997	0.904	0.874	1.19	1.338	1.094	1.315	1.173	0.866	1.109	1.077
TRINITY_DN58531_c0_g1_i1_orf1	uncharacterized protein LOC114357371 [Ostrinia furnacalis]	1	266	29.7	9.25	3	1	1	Medium	1	1.157	1.214	0.334	0.299	0.461	0.21	0.362	0.457	0.202	0.273	0.358	0.817	0.658	0.851
TRINITY_DN4694_c0_g1_i6_orf1	uncharacterized protein LOC114362122 [Ostrinia furnacalis]	1	205	23.9	8.31	4	1	1	Medium	1	0.884	0.916	1.242	0.959	1.399	1.285	1.245	1.333	1.244	1.187	1.161	1.118	1.123	1.339
TRINITY_DN1791_c0_g1_i3_orf1	succinate dehydrogenase assembly factor 2-B, mitochondrial-like [Ostrinia fur	1	142	16.8	9.33	4	1	1	Medium	1	1.094	1.073	0.764	0.686	0.708	0.662	0.627	0.637	0.516	0.382	0.547	0.484	0.523	0.519
TRINITY_DN40191_c2_g1_i1_orf1	stress-activated map kinase-interacting protein 1 [Ostrinia furnacalis]	1	527	60.4	6.89	1	1	1	Medium	1	0.942	1.24	2.449	1.631	0.987	2.125	2.078	1.33	2.773	2.428	2.091	3.439	3.072	2.693
TRINITY_DN4323_c0_g1_i1_orf1	uncharacterized protein LOC114364842 [Ostrinia furnacalis]	1	2276	247.4	6.61	0	1	1	Medium	1	1.105	1.177	0.546	0.809	1.206	0.664	1.132	0.77	0.951	0.714	0.715	0.563	0.686	0.735
TRINITY_DN3618_c0_g1_i4_orf1	WD repeat-containing protein 74-like isoform X1 [Ostrinia furnacalis]	1	424	47.6	8.47	4	1	1	Medium	1	0.853	1.01	0.495	0.468	0.571	0.832	0.637	0.947	0.823	0.567	0.63	0.785	0.889	0.689
TRINITY_DN8290_c0_g1_i3_orf1	zinc finger CCHC domain-containing protein 8 homolog [Ostrinia furnacalis]	1	640	70.8	4.61	2	1	1	Medium	1	1.054	1.124	0.737	0.705	0.83	0.69	0.712	0.603	0.72	0.42	0.636	0.871	0.819	0.859
TRINITY_DN83327_c0_g1_i1_orf1	trypsin-like serine proteinase T22 [Ostrinia nubilalis]	1	263	28.4	8.9	4	1	1	Medium	1	1.16	1.195	0.264	0.425	0.404	0.278	0.332	0.36	0.387	0.343	0.514	0.359	0.303	0.291
TRINITY_DN130159_c0_g2_i1_orf1	lachesin-like [Chelonus insularis]	1	129	14.4	6.79	12	1	1	Medium	1	1.069	0.992	0.838	0.807	0.851	0.781	0.771	0.744	0.726	0.677	0.688	0.502	0.619	0.628
TRINITY_DN6392_c0_g1_i9_orf1	putative mediator of RNA polymerase II transcription subunit 29 isoform X4 [O	1	185	19.9	9.06	5	1	1	Medium	1	0.943	0.882	1.614	2.034	1.436	1.286	1.45	1.096	1.274	1.347	1.726	1.155	0.917	1.064
TRINITY_DN8853_c0_g1_i4_orf1	uncharacterized protein LOC114351488 isoform X1 [Ostrinia furnacalis]	1	115	12.9	9.09	9	1	1	Medium	1	1.037	0.87	1.805	1.708	1.781	3.279	2.784	3.261	2.041	1.29	1.571	0.847	0.932	0.966
TRINITY_DN14409_c0_g1_i1_orf1	endoplasmic reticulum mannosyl-oligosaccharide 1,2-alpha-mannosidase [Os	1	578	65.8	6.46	1	1	1	Medium	1	1.025	1.1	1.295	1.231	1.397	1.196	1.203	1.009	0.95	0.87	1.014	0.951	1.065	0.969
TRINITY_DN12227_c0_g2_i3_orf1	exonuclease 3'-5' domain-containing protein 2 [Ostrinia furnacalis]	1	573	66.2	8.53	2	1	1	Medium	1	0.862	0.956	0.743	0.849	0.884	0.634	0.605	0.649	0.499	0.353	0.601	0.708	0.881	0.915
TRINITY_DN144807_c0_g1_i1_orf1	hypothetical protein G9C98_004245 [Cotesia typhae]	1	684	75.4	4.81	1	1	1	Medium	1	0.954	0.99	0.189	0.209	0.256	0.131	0.294	0.192	0.164	0.218	0.209	0.182	0.168	0.181
TRINITY_DN7407_c0_g1_i9_orf1	sec1 family domain-containing protein 2-like [Ostrinia furnacalis]	1	652	72.2	6.74	1	1	1	Medium	1	0.878	0.974	0.702	0.726	0.62	0.56	0.777	0.609	0.646	0.582	0.709	0.624	0.561	0.667
TRINITY_DN83542_c0_g1_i1_orf1	PREDICTED: WASH complex subunit strumpellin [Microplitis demolitor]	1	155	17.9	6.09	8	1	1	Medium	1	0.981	0.988	0.615	0.743	0.613	0.672	0.64	0.694	0.643	0.857	0.677	0.61	0.455	0.513
TRINITY_DN5935_c0_g1_i2_orf1	glucose dehydrogenase [FAD, quinone]-like [Ostrinia furnacalis]	1	596	66.1	5.34	3	1	1	Medium	1	0.698	0.612	1.011	0.394	0.865	0.898	0.908	0.604	1.163	1.397	0.695	0.908	1.093	0.741
TRINITY_DN31216_c0_g1_i2_orf1	uncharacterized protein LOC114361092 [Ostrinia furnacalis]	1	263	28.4	8.72	3	1	1	Medium	1	0.883	0.985	0.76	0.712	0.825	0.748	0.723	0.756	0.688	0.557	0.659	0.792	0.689	0.802
TRINITY_DN99_c0_g1_i3_orf1	uncharacterized protein LOC126375979 [Pectinophora gossypiella]	1	1186	140.2	8.95	1	1	1	Medium	1	0.838	0.832	0.34	0.312	0.445	0.494	0.392	0.368	0.565	0.675	0.8	0.347	0.338	0.341
TRINITY_DN30177_c0_g2_i1_orf1	uncharacterized protein LOC114365032 [Ostrinia furnacalis]	1	568	63	5.16	2	1	1	Medium	1	1.008	0.924	1.428	1.662	1.461	5.423	5.596	5.106	1.648	1.985	1.869	1.857	1.873	1.769
TRINITY_DN3212_c0_g1_i7_orfp1	TRINITY_DN3212_c0_g1_i7_m.38385 TRINITY_DN3212_c0_g1::TRINITY_DN3212	1	120	13.8	10.24	9	1	1	Medium	1	0.877	0.953	1.265	1.327	1.662	1.15	2.275	1.909	2.62	2.138	1.468	1.704	1.68	2.069
TRINITY_DN3082_c1_g1_i7_orf1	ribosomal RNA processing protein 1 homolog [Ostrinia furnacalis]	1	739	84.5	9.66	1	1	1	Medium	1	1.138	1.135	0.219	0.254	0.233	0.251	0.248	0.334	0.232	0.241	0.294	0.322	0.315	0.317
TRINITY_DN640_c0_g1_i6_orf1	pancreatic triacylglycerol lipase-like [Ostrinia furnacalis]	1	238	26.5	6.81	5	1	1	Medium	1	1.095	1.053	0.977	1.068	1.439	1.097	1.106	1.509	1.26	2.01	0.844	1.244	1.145	1.307
TRINITY_DN107962_c0_g1_i1_orf1	unnamed protein product [Euphydryas editha]	1	89	10.3	5.82	8	1	1	Medium	1	0.975	0.97	0.825	0.918	0.999	0.96	1.123	0.939	0.681	0.814	0.827	0.761	0.537	0.489
TRINITY_DN45963_c0_g1_i1_orf1	UPF0505 protein C16orf62 homolog [Ostrinia furnacalis]	1	84	9.5	8.05	8	1	1	Medium	1	1.134	0.945	1.153	0.979	0.726	0.861	1.114	1.295	1.106	1.1	0.651	1.107	1.035	1.333
TRINITY_DN6711_c0_g1_i1_orf1	mitochondrial uncoupling protein 4 [Ostrinia furnacalis]	1	316	34.7	9.6	3	1	1	Medium	1	1.026	0.978	0.552	0.562	0.568	0.547	0.571	0.536	0.594	0.552	0.504	0.5	0.588	0.6

TRINITY_DN8089_c0_g1_i3_orf1	transmembrane protein 230 [Ostrinia furnacalis]	1	135	15.6	7.56	4	1	1	Medium	1	0.778	0.825	0.692	1.053	1.136	0.865	0.997	0.747	0.888	0.496	0.867	1.003	0.794	0.99
TRINITY_DN50074_c0_g1_i1_orf1	uncharacterized protein LOC114364628 [Ostrinia furnacalis]	1	228	25.7	6.79	5	1	1	Medium	1	0.839	0.934	1.076	1.177	1.077	1.118	1.182	1.089	1.213	1.491	1.372	0.786	0.675	0.662
TRINITY_DN19328_c0_g1_i1_orf1	hypothetical protein evm_002753 [Chilo suppressalis]	1	299	34.3	5.45	7	1	1	Medium	1	0.872	0.939	1.158	1.308	1.212	1.452	1.604	1.282	1.01	1.142	1.239	1.713	1.696	1.61
TRINITY_DN80134_c0_g1_i1_orf1	gephyrin isoform X2 [Ostrinia furnacalis]	1	54	6	5.78	31	1	1	Medium	1	1.064	1.241	0.936	0.942	1.151	1.007	1.133	1.215	1.192	1.372	1.293	1.237	1.172	1.182
TRINITY_DN8386_c0_g1_i6_orf1	F-box/WD repeat-containing protein 9-like [Ostrinia furnacalis]	1	437	49.3	5.45	2	1	1	Medium	1	0.826	1.03	1.603	1.27	1.372	1.313	0.9	1.471	1.32	1.587	1.618	1.62	1.608	1.322
TRINITY_DN46778_c0_g1_i2_orf1	Deoxycytidylate deaminase [Papilio xuthus]	1	189	21.4	7.12	5	1	1	Medium	1	1.083	1.036	0.345	0.354	0.389	0.385	0.438	0.418	0.468	0.414	0.449	0.602	0.62	0.576
TRINITY_DN41296_c0_g1_i1_orf1	exocyst complex component 5 [Ostrinia furnacalis]	1	724	81.1	6.23	1	1	1	Medium	1	0.913	1.004	1.282	1.145	1.164	1.549	1.459	1.422	1.5	1.491	1.419	1.269	1.353	1.355
TRINITY_DN89083_c0_g1_i1_orf1	lysine-specific demethylase 4A isoform X2 [Diachasma alloeum]	1	108	12.5	7.44	6	1	1	Medium	1	0.792	0.783	1.39	1.529	1.422	2.051	1.777	1.803	1.697	1.932	2.254	1.958	1.92	2.087
TRINITY_DN38371_c0_g1_i7_orf1	protein smoothened isoform X2 [Ostrinia furnacalis]	1	750	84.4	8.84	1	1	1	Medium	1	0.89	0.859	1.267	1.148	1.077	1.009	1.239	1.05	1.116	0.998	1.052	1.168	1.394	1.359
TRINITY_DN145448_c0_g1_i1_orf1	GSCOCG00008786001-RA-CDS [Cotesia congregata]	1	149	16.5	5.4	4	1	1	Medium	1	1.006	0.962	0.904	0.839	0.869	0.823	0.859	0.791	0.879	0.758	0.936	0.965	1.143	0.983
TRINITY_DN9790_c0_g1_i4_orf1	protein IWS1 homolog [Ostrinia furnacalis]	1	105	12.2	10.84	9	1	1	Medium	1	0.848	1.001	0.451	0.72	0.554	0.488	0.526	0.584	0.219	0.494	0.428	0.562	0.695	0.586
