# Supplementary material for: Comparative Proteomics and Metabonomics Analysis of Different Diapause Stages Revealed a New Regulation Mechanism of Diapause in Loxostege sticticalis (Lepidoptera: Pyralidae)
Source: Molecules. 2024 Jul 25;29(15):3472. doi: 10.3390/molecules29153472 (PMC11314584; doi:10.3390/molecules29153472)
Supplement: Supplementary file 1 [file molecules-29-03472-s001.zip › analysis process/proteomic/Protein Network Node Attribute Table.pdf]

Node1	Node2	node1_accession_id	node2_accession_id	neighborhood_on_chromosome	gene_fusion	phylogenetic_cooccurrenc	homology	coexpression	experimentally_determined_interaction	database_annotated	automated_textmining	combined_score
A0A3S2TG98	A0A3S2L860	TRINITY_DN144956_c0_g1_i1_orf1	TRINITY_DN58207_c0_g1_i1_orf1	0.081	0	0	0	0.99	0.999	0	0.693	0.999
A0A3S2LGN6	A0A3S2L860	TRINITY_DN82324_c0_g1_i4_orf1	TRINITY_DN58207_c0_g1_i1_orf1	0.061	0	0	0	0.979	0.999	0.581	0.507	0.999
A0A437BHG9	A0A3S2L860	TRINITY_DN97589_c0_g1_i3_orf1	TRINITY_DN58207_c0_g1_i1_orf1	0	0	0	0	0.911	0.999	0.71	0.243	0.999
A0A3S2NP74	A0A3S2L149	TRINITY_DN1366_c0_g1_i5_orf1	TRINITY_DN14436_c0_g1_i7_orf1	0	0	0	0	0.544	0.975	0.997	0.873	0.999
A0A437BDZ4	A0A3S2L860	TRINITY_DN7991_c0_g1_i9_orf1	TRINITY_DN58207_c0_g1_i1_orf1	0.061	0	0	0	0.977	0.999	0.944	0.765	0.999
A0A3S2LAA0	A0A3S2L860	TRINITY_DN28039_c0_g1_i1_orf1	TRINITY_DN58207_c0_g1_i1_orf1	0	0	0	0	0.972	0.999	0.617	0.381	0.999
A0A437BJ32	A0A3S2LB03	TRINITY_DN15234_c0_g1_i3_orf1	TRINITY_DN3393_c0_g2_i1_orf1	0	0	0	0	0.996	0.999	0.535	0.381	0.999
A0A3S2NYT8	A0A3S2L149	TRINITY_DN10458_c0_g1_i1_orf1	TRINITY_DN14436_c0_g1_i7_orf1	0	0	0	0	0.418	0.813	0.934	0.859	0.999
A0A3S2LC29	A0A3S2L860	TRINITY_DN1509_c0_g1_i1_orf1	TRINITY_DN58207_c0_g1_i1_orf1	0.081	0	0	0	0.972	0.91	0	0.622	0.999
A0A3S2M6Z7	A0A3S2L149	TRINITY_DN22430_c0_g3_i1_orf1	TRINITY_DN14436_c0_g1_i7_orf1	0	0	0	0	0.415	0.946	0.931	0.787	0.999
A0A3S2P8Y5	A0A3S2LB03	TRINITY_DN137_c0_g1_i1_orf1	TRINITY_DN3393_c0_g2_i1_orf1	0	0	0	0	0.999	0.999	0.581	0.765	0.999
A0A3S2NAD9	A0A3S2L149	TRINITY_DN1044_c0_g1_i2_orf1	TRINITY_DN14436_c0_g1_i7_orf1	0	0	0	0	0.575	0.976	0.997	0.864	0.999
A0A3S2N9K5	A0A3S2LD85	TRINITY_DN10637_c0_g1_i4_orf1	TRINITY_DN17351_c0_g1_i3_orf1	0.112	0	0	0	0.425	0.968	0.935	0.621	0.999
A0A3S2LA54	A0A3S2L149	TRINITY_DN15624_c0_g1_i1_orf1	TRINITY_DN14436_c0_g1_i7_orf1	0	0	0	0	0.375	0.855	0.933	0.818	0.999
A0A3S2TJI5	A0A3S2L2W8	TRINITY_DN16749_c0_g1_i1_orf1	TRINITY_DN103457_c0_g1_i1_orf1	0	0	0	0	0.366	0.933	0.968	0.681	0.999
A0A3S2LEV6	A0A3S2L860	TRINITY_DN87603_c0_g2_i1_orf1	TRINITY_DN58207_c0_g1_i1_orf1	0.081	0	0	0	0.995	0.999	0.581	0.52	0.999
A0A437BJ32	A0A3S2L860	TRINITY_DN15234_c0_g1_i3_orf1	TRINITY_DN58207_c0_g1_i1_orf1	0	0	0	0	0.987	0.999	0.951	0.658	0.999
A0A3S2NS36	A0A3S2L860	TRINITY_DN3733_c0_g1_i1_orf1	TRINITY_DN58207_c0_g1_i1_orf1	0	0	0	0	0.894	0.999	0.94	0.567	0.999
A0A3S2M865	A0A3S2L860	TRINITY_DN11297_c0_g1_i1_orf1	TRINITY_DN58207_c0_g1_i1_orf1	0.054	0	0	0	0.858	0.999	0.815	0.324	0.999
A0A3S2LC29	A0A3S2LB03	TRINITY_DN1509_c0_g1_i1_orf1	TRINITY_DN3393_c0_g2_i1_orf1	0	0	0	0	0.967	0.91	0.951	0.595	0.999
A0A3S2LEV6	A0A3S2LB28	TRINITY_DN87603_c0_g2_i1_orf1	TRINITY_DN17446_c0_g1_i1_orf1	0	0	0	0	0.964	0.674	0.927	0.477	0.999
A0A3S2LGY4	A0A3S2LEB1	TRINITY_DN4779_c0_g1_i5_orf1	TRINITY_DN7464_c1_g1_i1_orf1	0	0	0	0.397	0.999	0.948	0.745	0.774	0.999
A0A3S2LGN6	A0A3S2LR49	TRINITY_DN82324_c0_g1_i4_orf1	TRINITY_DN102260_c0_g1_i1_orf1	0.075	0	0	0	0.988	0.999	0.784	0.177	0.999
A0A3S2TG98	A0A3S2LR49	TRINITY_DN144956_c0_g1_i1_orf1	TRINITY_DN102260_c0_g1_i1_orf1	0.112	0	0	0	0.99	0.999	0	0.672	0.999
A0A437B0D7	A0A3S2LT35	TRINITY_DN8949_c0_g1_i2_orf1	TRINITY_DN3985_c0_g2_i1_orf1	0.112	0	0	0	0.337	0.999	0.815	0.312	0.999
A0A437BQJ6	A0A3S2LT35	TRINITY_DN146718_c0_g1_i1_orf1	TRINITY_DN3985_c0_g2_i1_orf1	0	0	0	0	0.971	0.999	0.551	0.525	0.999
A0A3S2N9A9	A0A3S2L2W8	TRINITY_DN21792_c0_g1_i1_orf1	TRINITY_DN103457_c0_g1_i1_orf1	0	0	0	0	0.368	0.925	0.968	0.207	0.999
A0A3S2LR49	A0A3S2LB03	TRINITY_DN102260_c0_g1_i1_orf1	TRINITY_DN3393_c0_g2_i1_orf1	0	0	0.216	0	0.992	0.999	0.581	0.615	0.999
A0A437BML7	A0A3S2LB03	TRINITY_DN71840_c0_g1_i1_orf1	TRINITY_DN3393_c0_g2_i1_orf1	0	0	0	0	0.995	0.999	0.551	0.813	0.999
A0A3S2M865	A0A3S2LB03	TRINITY_DN11297_c0_g1_i1_orf1	TRINITY_DN3393_c0_g2_i1_orf1	0	0	0	0	0.996	0.999	0.808	0.375	0.999
A0A3S2LGN6	A0A3S2LB03	TRINITY_DN82324_c0_g1_i4_orf1	TRINITY_DN3393_c0_g2_i1_orf1	0	0	0	0	0.991	0.999	0.968	0.626	0.999
A0A3S2LC29	A0A3S2LGN6	TRINITY_DN1509_c0_g1_i1_orf1	TRINITY_DN82324_c0_g1_i4_orf1	0.112	0	0	0	0.973	0.91	0.951	0.439	0.999
A0A437BJ80	A0A3S2LM80	TRINITY_DN8291_c0_g1_i3_orf1	TRINITY_DN6985_c0_g1_i5_orf1	0	0	0	0	0.232	0.994	0.936	0.952	0.999
A0A3S2LNA9	A0A3S2LNZ7	TRINITY_DN3749_c0_g1_i1_orf1	TRINITY_DN3134_c0_g1_i1_orf1	0	0	0	0	0.931	0.999	0.965	0.721	0.999
A0A437BIM5	A0A3S2LT35	TRINITY_DN9101_c0_g2_i1_orf1	TRINITY_DN3985_c0_g2_i1_orf1	0.059	0	0	0	0.946	0.999	0.707	0.38	0.999
A0A437AWA0	A0A3S2LT35	TRINITY_DN135_c0_g1_i1_orf1	TRINITY_DN3985_c0_g2_i1_orf1	0.461	0	0	0	0.909	0.999	0.745	0.814	0.999
A0A3S2LVN6	A0A3S2LT35	TRINITY_DN7464_c0_g1_i14_orf1	TRINITY_DN3985_c0_g2_i1_orf1	0.461	0	0.223	0	0.971	0.999	0.951	0.637	0.999
A0A3S2LVN6	A0A3S2L860	TRINITY_DN7464_c0_g1_i14_orf1	TRINITY_DN58207_c0_g1_i1_orf1	0.081	0	0	0	0.991	0.999	0.951	0.795	0.999
A0A3S2NXV9	A0A3S2LB03	TRINITY_DN18869_c0_g1_i1_orf1	TRINITY_DN3393_c0_g2_i1_orf1	0	0	0	0	0.992	0.999	0.968	0.623	0.999
A0A437BDC5	A0A3S2LB03	TRINITY_DN7241_c0_g2_i2_orf1	TRINITY_DN3393_c0_g2_i1_orf1	0	0	0	0	0.905	0.91	0.951	0.862	0.999
A0A3S2TPR7	A0A3S2LB28	TRINITY_DN32822_c0_g1_i1_orf1	TRINITY_DN17446_c0_g1_i1_orf1	0	0	0	0	0.095	0.773	0.995	0.519	0.999
A0A3S2TG98	A0A3S2LC29	TRINITY_DN144956_c0_g1_i1_orf1	TRINITY_DN1509_c0_g1_i1_orf1	0.461	0	0	0	0.966	0.91	0.951	0.702	0.999
A0A437BLW6	A0A3S2LD85	TRINITY_DN25975_c0_g3_i2_orf1	TRINITY_DN17351_c0_g1_i3_orf1	0.081	0	0	0	0.549	0.913	0.997	0.605	0.999
A0A437BNN5	A0A3S2LEB1	TRINITY_DN20776_c0_g1_i3_orf1	TRINITY_DN7464_c1_g1_i1_orf1	0	0	0	0.433	0.999	0.948	0.745	0.728	0.999
A0A437BRP2	A0A3S2L860	TRINITY_DN38075_c0_g1_i1_orf1	TRINITY_DN58207_c0_g1_i1_orf1	0.061	0	0	0	0.935	0.999	0.815	0.822	0.999
A0A3S2NUQ5	A0A3S2LIH0	TRINITY_DN36648_c0_g1_i1_orf1	TRINITY_DN5811_c0_g1_i4_orf1	0	0	0	0	0.132	0.202	0.998	0.208	0.999
A0A437AWA0	A0A3S2L860	TRINITY_DN135_c0_g1_i1_orf1	TRINITY_DN58207_c0_g1_i1_orf1	0.081	0	0	0	0.99	0.999	0.951	0.352	0.999
A0A3S2TSS2	A0A3S2LNB4	TRINITY_DN154_c0_g1_i4_orf1	TRINITY_DN9002_c0_g1_i1_orf1	0	0	0	0	0.602	0.89	0.931	0.637	0.999
A0A3S2LA54	A0A3S2LD85	TRINITY_DN15624_c0_g1_i1_orf1	TRINITY_DN17351_c0_g1_i3_orf1	0	0	0	0	0.59	0.93	0.934	0.546	0.999
A0A3S2NS36	A0A3S2LT35	TRINITY_DN3733_c0_g1_i1_orf1	TRINITY_DN3985_c0_g2_i1_orf1	0	0	0	0	0.74	0.999	0.94	0.603	0.999
A0A3S2TG98	A0A3S2LGN6	TRINITY_DN144956_c0_g1_i1_orf1	TRINITY_DN82324_c0_g1_i4_orf1	0.112	0	0	0	0.995	0.999	0.968	0.404	0.999
A0A437BPJ9	A0A3S2LHU2	TRINITY_DN5756_c0_g1_i4_orf1	TRINITY_DN620_c0_g1_i4_orf1	0.086	0	0	0	0.562	0.834	0.894	0.843	0.999
A0A3S2LEV6	A0A3S2LR49	TRINITY_DN87603_c0_g2_i1_orf1	TRINITY_DN102260_c0_g1_i1_orf1	0.112	0	0	0	0.993	0.999	0.581	0.731	0.999
A0A3S2LC29	A0A3S2LR49	TRINITY_DN1509_c0_g1_i1_orf1	TRINITY_DN102260_c0_g1_i1_orf1	0.112	0	0	0	0.968	0.91	0.499	0.471	0.999
A0A3S2LC29	A0A3S2LT35	TRINITY_DN1509_c0_g1_i1_orf1	TRINITY_DN3985_c0_g2_i1_orf1	0.461	0	0	0	0.96	0.91	0.515	0.574	0.999
A0A437BML7	A0A3S2LT35	TRINITY_DN71840_c0_g1_i1_orf1	TRINITY_DN3985_c0_g2_i1_orf1	0.461	0	0	0	0.97	0.999	0.951	0.843	0.999
A0A3S2LAA0	A0A3S2LT35	TRINITY_DN28039_c0_g1_i1_orf1	TRINITY_DN3985_c0_g2_i1_orf1	0.061	0	0	0	0.967	0.999	0	0.437	0.999
A0A3S2LNS6	A0A3S2LUY1	TRINITY_DN24024_c0_g1_i1_orf1	TRINITY_DN332_c0_g1_i6_orf1	0	0	0	0	0.483	0.948	0.997	0.786	0.999
A0A437B0D7	A0A3S2LB03	TRINITY_DN8949_c0_g1_i2_orf1	TRINITY_DN3393_c0_g2_i1_orf1	0	0	0	0	0.991	0.999	0.551	0.411	0.999
A0A437BDZ4	A0A3S2LB03	TRINITY_DN7991_c0_g1_i9_orf1	TRINITY_DN3393_c0_g2_i1_orf1	0	0	0	0	0.971	0.999	0.551	0.725	0.999
A0A3S2NHB4	A0A3S2LB03	TRINITY_DN50724_c0_g2_i1_orf1	TRINITY_DN3393_c0_g2_i1_orf1	0	0	0	0	0.996	0.999	0.968	0.812	0.999
A0A3S2TBV5	A0A3S2LB28	TRINITY_DN119797_c0_g1_i1_orf1	TRINITY_DN17446_c0_g1_i1_orf1	0	0	0	0	0.874	0.795	0.928	0.517	0.999
A0A437BW92	A0A3S2LB28	TRINITY_DN17049_c0_g1_i6_orf1	TRINITY_DN17446_c0_g1_i1_orf1	0	0	0	0	0.985	0.991	0.815	0.952	0.999
A0A3S2NC30	A0A3S2LB28	TRINITY_DN14498_c0_g1_i1_orf1	TRINITY_DN17446_c0_g1_i1_orf1	0	0	0	0	0.608	0.672	0.995	0.542	0.999
A0A437B6J8	A0A3S2LEB1	TRINITY_DN5262_c0_g1_i7_orf1	TRINITY_DN7464_c1_g1_i1_orf1	0	0	0	0.379	0.994	0.964	0.951	0.894	0.999
A0A3S2NXV9	A0A3S2L860	TRINITY_DN18869_c0_g1_i1_orf1	TRINITY_DN58207_c0_g1_i1_orf1	0	0	0	0	0.997	0.999	0.581	0.565	0.999
A0A3S2LHJ5	A0A3S2LD85	TRINITY_DN4434_c0_g1_i7_orf1	TRINITY_DN17351_c0_g1_i3_orf1	0.461	0	0	0	0.394	0.973	0.995	0.661	0.999
A0A437AXU0	A0A3S2LEE0	TRINITY_DN22815_c0_g1_i2_orf1	TRINITY_DN9558_c0_g1_i2_orf1	0	0	0	0	0.975	0.948	0.938	0.465	0.999
A0A3S2M865	A0A3S2LT35	TRINITY_DN11297_c0_g1_i1_orf1	TRINITY_DN3985_c0_g2_i1_orf1	0.081	0	0	0	0.971	0.999	0.951	0.531	0.999
A0A3S2L860	A0A3S2LT35	TRINITY_DN58207_c0_g1_i1_orf1	TRINITY_DN3985_c0_g2_i1_orf1	0.081	0	0	0	0.968	0.999	0.951	0.784	0.999
A0A3S2LT68	A0A3S2LT35	TRINITY_DN7583_c0_g1_i1_orf1	TRINITY_DN3985_c0_g2_i1_orf1	0.129	0	0	0	0.578	0.993	0.606	0.817	0.999
A0A437AWG4	A0A3S2LT35	TRINITY_DN19186_c0_g1_i1_orf1	TRINITY_DN3985_c0_g2_i1_orf1	0.089	0	0	0	0.506	0.993	0.606	0.857	0.999
A0A3S2LJ09	A0A3S2L1J1	TRINITY_DN80560_c0_g1_i1_orf1	TRINITY_DN67716_c0_g1_i1_orf1	0.079	0	0	0	0.355	0	0.995	0.879	0.999
A0A3S2NYT8	A0A3S2L1J1	TRINITY_DN10458_c0_g1_i1_orf1	TRINITY_DN67716_c0_g1_i1_orf1	0.052	0	0	0	0.321	0	0.995	0.747	0.999
A0A3S2NHB4	A0A3S2L860	TRINITY_DN50724_c0_g2_i1_orf1	TRINITY_DN58207_c0_g1_i1_orf1	0	0	0	0	0.981	0.999	0.581	0.695	0.999
A0A3S2P8Y5	A0A3S2L860	TRINITY_DN137_c0_g1_i1_orf1	TRINITY_DN58207_c0_g1_i1_orf1	0.061	0	0	0	0.999	0.999	0.951	0.853	0.999
A0A437ATU4	A0A3S2LB03	TRINITY_DN33926_c0_g1_i1_orf1	TRINITY_DN3393_c0_g2_i1_orf1	0	0	0	0	0.94	0.999	0.894	0.584	0.999
A0A3S2NJI1	A0A3S2LB28	TRINITY_DN3878_c0_g1_i4_orf1	TRINITY_DN17446_c0_g1_i1_orf1	0	0	0	0	0.364	0.867	0.976	0.79	0.999
A0A3S2LC29	A0A3S2LEV6	TRINITY_DN1509_c0_g1_i1_orf1	TRINITY_DN87603_c0_g2_i1_orf1	0.461	0	0.205	0	0.967	0.91	0.951	0.817	0.999
A0A437BRP2	A0A3S2LT35	TRINITY_DN38075_c0_g1_i1_orf1	TRINITY_DN3985_c0_g2_i1_orf1	0.112	0	0	0	0.554	0.999	0.815	0.738	0.999
A0A3S2LEV6	A0A3S2LT35	TRINITY_DN87603_c0_g2_i1_orf1	TRINITY_DN3985_c0_g2_i1_orf1	0.461	0	0	0	0.968	0.999	0.535	0.869	0.999

A0A3S2LT68	A0A3S2LUB1	TRINITY_DN7583_c0_g1_i1_orf1	TRINITY_DN16939_c0_g1_i4_orf1	0	0	0	0	0.395	0.523	0.951	0.93	0.999
A0A437BHG9	A0A3S2LB03	TRINITY_DN97589_c0_g1_i3_orf1	TRINITY_DN3393_c0_g2_i1_orf1	0	0	0	0	0.972	0.999	0	0.208	0.999
A0A3S2L860	A0A3S2LB03	TRINITY_DN58207_c0_g1_i1_orf1	TRINITY_DN3393_c0_g2_i1_orf1	0	0	0	0	0.997	0.999	0.581	0.437	0.999
A0A437BW52	A0A3S2LBF8	TRINITY_DN466_c0_g1_i5_orf1	TRINITY_DN26251_c0_g1_i1_orf1	0	0	0.349	0.249	0.271	0.813	0.976	0.611	0.999
A0A3S2N6A5	A0A3S2LD17	TRINITY_DN6871_c0_g1_i3_orf1	TRINITY_DN5841_c0_g1_i2_orf1	0	0	0	0	0.143	0	0.998	0.752	0.999
A0A3S2NP74	A0A3S2LD85	TRINITY_DN1366_c0_g1_i5_orf1	TRINITY_DN17351_c0_g1_i3_orf1	0.461	0	0	0	0.498	0.966	0.997	0.591	0.999
A0A3S2TG98	A0A3S2LEV6	TRINITY_DN144956_c0_g1_i1_orf1	TRINITY_DN87603_c0_g2_i1_orf1	0.461	0	0	0	0.846	0.999	0.712	0.126	0.999
A0A3S2LAA0	A0A3S2LGN6	TRINITY_DN28039_c0_g1_i1_orf1	TRINITY_DN82324_c0_g1_i4_orf1	0.05	0	0	0	0.891	0.999	0	0.426	0.999
A0A437BUZ1	A0A3S2LHU2	TRINITY_DN13368_c0_g1_i1_orf1	TRINITY_DN620_c0_g1_i4_orf1	0.064	0	0	0	0.648	0.792	0.963	0.931	0.999
A0A437BBQ1	A0A3S2LQK2	TRINITY_DN17215_c0_g1_i4_orf1	TRINITY_DN19829_c0_g1_i1_orf1	0	0	0	0	0.442	0.943	0.968	0	0.999
A0A437BJ32	A0A3S2LT35	TRINITY_DN15234_c0_g1_i3_orf1	TRINITY_DN3985_c0_g2_i1_orf1	0	0	0	0	0.968	0.999	0.951	0.621	0.999
A0A3S2LUB1	A0A3S2LT35	TRINITY_DN16939_c0_g1_i4_orf1	TRINITY_DN3985_c0_g2_i1_orf1	0.461	0	0	0	0.182	0.993	0.606	0.127	0.999
A0A437BML7	A0A3S2L860	TRINITY_DN71840_c0_g1_i1_orf1	TRINITY_DN58207_c0_g1_i1_orf1	0.081	0	0	0	0.99	0.999	0.951	0.87	0.999
A0A3S2TG98	A0A3S2LB03	TRINITY_DN144956_c0_g1_i1_orf1	TRINITY_DN3393_c0_g2_i1_orf1	0	0	0	0	0.997	0.999	0.968	0.841	0.999
A0A437B289	A0A3S2LD85	TRINITY_DN9715_c0_g1_i1_orf1	TRINITY_DN17351_c0_g1_i3_orf1	0.461	0	0	0	0.507	0.987	0.895	0.798	0.999
A0A3S2LAA0	A0A3S2LEV6	TRINITY_DN28039_c0_g1_i1_orf1	TRINITY_DN87603_c0_g2_i1_orf1	0.061	0	0	0	0.989	0.999	0	0.581	0.999
A0A3S2LEV6	A0A3S2LGN6	TRINITY_DN87603_c0_g2_i1_orf1	TRINITY_DN82324_c0_g1_i4_orf1	0.112	0	0	0	0.992	0.999	0.968	0.848	0.999
A0A3S2LJ09	A0A3S2LRY7	TRINITY_DN80560_c0_g1_i1_orf1	TRINITY_DN86090_c0_g1_i1_orf1	0	0	0	0	0.973	0.999	0.95	0.513	0.999
A0A3S2LGN6	A0A3S2LT35	TRINITY_DN82324_c0_g1_i4_orf1	TRINITY_DN3985_c0_g2_i1_orf1	0.112	0	0	0	0.968	0.999	0.523	0.734	0.999
A0A3S2LR49	A0A3S2L860	TRINITY_DN102260_c0_g1_i1_orf1	TRINITY_DN58207_c0_g1_i1_orf1	0	0	0	0	0.94	0.999	0.815	0.478	0.999
A0A3S2NL72	A0A3S2LB28	TRINITY_DN2716_c0_g2_i1_orf1	TRINITY_DN17446_c0_g1_i1_orf1	0	0	0	0	0.284	0.944	0.976	0.344	0.999
A0A437B2R6	A0A3S2LGZ7	TRINITY_DN8430_c0_g1_i1_orf1	TRINITY_DN15965_c0_g1_i1_orf1	0	0	0.418	0.17	0.956	0.88	0.927	0.386	0.999
A0A3S2P9R9	A0A3S2LNB4	TRINITY_DN2356_c2_g1_i6_orf1	TRINITY_DN9002_c0_g1_i1_orf1	0	0	0	0	0.368	0.937	0.927	0.774	0.999
A0A437BG93	A0A3S2LQ04	TRINITY_DN44219_c0_g1_i1_orf1	TRINITY_DN22871_c0_g2_i1_orf1	0	0	0	0	0.41	0.964	0.959	0.782	0.999
A0A3S2LAA0	A0A3S2LR49	TRINITY_DN28039_c0_g1_i1_orf1	TRINITY_DN102260_c0_g1_i1_orf1	0.05	0	0	0	0.974	0.999	0	0.557	0.999
A0A3S2TG98	A0A3S2LT35	TRINITY_DN144956_c0_g1_i1_orf1	TRINITY_DN3985_c0_g2_i1_orf1	0.461	0	0	0	0.93	0.999	0	0.332	0.999
A0A437BHG9	A0A3S2LT35	TRINITY_DN97589_c0_g1_i3_orf1	TRINITY_DN3985_c0_g2_i1_orf1	0.063	0	0	0	0.955	0.999	0.71	0.853	0.999
A0A3S2NMT3	A0A3S2LUY1	TRINITY_DN16539_c0_g1_i7_orf1	TRINITY_DN332_c0_g1_i6_orf1	0	0	0	0	0.611	0.948	0.997	0.715	0.999
A0A437BCR1	A0A3S2LVB0	TRINITY_DN19115_c0_g1_i1_orf1	TRINITY_DN26649_c0_g1_i2_orf1	0	0	0	0	0.759	0.999	0.745	0.337	0.999
A0A437BQJ6	A0A3S2L860	TRINITY_DN146718_c0_g1_i1_orf1	TRINITY_DN58207_c0_g1_i1_orf1	0	0	0	0	0.98	0.999	0	0.318	0.999
A0A3S2LAA0	A0A3S2LB03	TRINITY_DN28039_c0_g1_i1_orf1	TRINITY_DN3393_c0_g2_i1_orf1	0	0	0	0	0.978	0.999	0	0.272	0.999
A0A3S2TDB1	A0A3S2LB28	TRINITY_DN2084_c0_g1_i1_orf1	TRINITY_DN17446_c0_g1_i1_orf1	0	0	0	0	0.594	0.762	0.927	0.819	0.999
A0A3S2NV61	A0A3S2LB28	TRINITY_DN121893_c0_g1_i1_orf1	TRINITY_DN17446_c0_g1_i1_orf1	0	0	0	0	0.943	0.809	0.928	0.282	0.999
A0A3S2NN57	A0A3S2LBV7	TRINITY_DN13055_c0_g1_i5_orf1	TRINITY_DN14487_c0_g1_i4_orf1	0	0	0	0	0.801	0.974	0.862	0.412	0.999
A0A437BDZ4	A0A3S2LT35	TRINITY_DN7991_c0_g1_i9_orf1	TRINITY_DN3985_c0_g2_i1_orf1	0.112	0	0	0	0.959	0.999	0.745	0.481	0.999
A0A437ATU4	A0A3S2LT35	TRINITY_DN33926_c0_g1_i1_orf1	TRINITY_DN3985_c0_g2_i1_orf1	0.112	0	0	0	0.967	0.999	0.535	0.619	0.999
A0A3S2LC29	A0A3S2LUB1	TRINITY_DN1509_c0_g1_i1_orf1	TRINITY_DN16939_c0_g1_i4_orf1	0.461	0	0	0	0.821	0.867	0	0.929	0.999
A0A3S2LRY7	A0A3S2LUU7	TRINITY_DN86090_c0_g1_i1_orf1	TRINITY_DN83005_c0_g1_i1_orf1	0	0	0	0	0.991	0.999	0.95	0.613	0.999
A0A3S2NPG0	A0A3S2LUY1	TRINITY_DN10058_c0_g1_i1_orf1	TRINITY_DN332_c0_g1_i6_orf1	0	0	0	0	0.373	0.774	0.997	0.785	0.999
A0A437BIM5	A0A3S2LB03	TRINITY_DN9101_c0_g2_i1_orf1	TRINITY_DN3393_c0_g2_i1_orf1	0	0	0	0	0.992	0.999	0.551	0.866	0.999
A0A3S2P8Y5	A0A3S2LT35	TRINITY_DN137_c0_g1_i1_orf1	TRINITY_DN3985_c0_g2_i1_orf1	0.112	0	0	0	0.964	0.999	0.951	0.817	0.999
A0A3S2NHB4	A0A3S2LT35	TRINITY_DN50724_c0_g2_i1_orf1	TRINITY_DN3985_c0_g2_i1_orf1	0.064	0	0	0	0.973	0.999	0	0.936	0.999
A0A3S2LJ09	A0A3S2LUU7	TRINITY_DN80560_c0_g1_i1_orf1	TRINITY_DN83005_c0_g1_i1_orf1	0.461	0	0	0	0.771	0.999	0.796	0.511	0.999
A0A3S2M6Z7	A0A3S2LD85	TRINITY_DN22430_c0_g3_i1_orf1	TRINITY_DN17351_c0_g1_i3_orf1	0.081	0	0	0	0.262	0.852	0.938	0.811	0.999
A0A437AS99	A0A3S2LQK2	TRINITY_DN754_c1_g1_i6_orf1	TRINITY_DN19829_c0_g1_i1_orf1	0	0	0	0	0.519	0.937	0.968	0	0.999
A0A3S2LAW0	A0A3S2LSD2	TRINITY_DN2082_c0_g1_i2_orf1	TRINITY_DN401_c0_g1_i15_orf1	0	0	0	0	0.138	0	0.997	0.531	0.999
A0A3S2NXV9	A0A3S2LT35	TRINITY_DN18869_c0_g1_i1_orf1	TRINITY_DN3985_c0_g2_i1_orf1	0.089	0	0	0	0.97	0.999	0.551	0.419	0.999
A0A3S2LD85	A0A3S2L149	TRINITY_DN17351_c0_g1_i3_orf1	TRINITY_DN14436_c0_g1_i7_orf1	0	0	0	0	0.629	0.983	0.997	0.77	0.999
A0A437ATU4	A0A3S2L860	TRINITY_DN33926_c0_g1_i1_orf1	TRINITY_DN58207_c0_g1_i1_orf1	0.061	0	0	0	0.98	0.999	0	0.311	0.999
A0A3S2TG98	A0A3S2LAA0	TRINITY_DN144956_c0_g1_i1_orf1	TRINITY_DN28039_c0_g1_i1_orf1	0.061	0	0	0	0.736	0.999	0	0.129	0.999
A0A437AWA0	A0A3S2LB03	TRINITY_DN135_c0_g1_i1_orf1	TRINITY_DN3393_c0_g2_i1_orf1	0	0	0	0	0.997	0.999	0.587	0.567	0.999
A0A3S2LEV6	A0A3S2LB03	TRINITY_DN87603_c0_g2_i1_orf1	TRINITY_DN3393_c0_g2_i1_orf1	0	0	0	0	0.997	0.999	0.968	0.868	0.999
A0A3S2LR49	A0A3S2LT35	TRINITY_DN102260_c0_g1_i1_orf1	TRINITY_DN3985_c0_g2_i1_orf1	0.112	0	0	0	0.971	0.999	0.951	0.386	0.999
A0A3S2LB03	A0A3S2LT35	TRINITY_DN3393_c0_g2_i1_orf1	TRINITY_DN3985_c0_g2_i1_orf1	0	0	0	0	0.97	0.999	0.551	0.81	0.999
A0A437BLW6	A0A3S2L149	TRINITY_DN25975_c0_g3_i2_orf1	TRINITY_DN14436_c0_g1_i7_orf1	0	0	0	0	0.45	0.917	0.997	0.601	0.999
A0A3S2NS36	A0A3S2LB03	TRINITY_DN3733_c0_g1_i1_orf1	TRINITY_DN3393_c0_g2_i1_orf1	0	0	0	0	0.915	0.999	0.581	0.403	0.999
A0A437BRP2	A0A3S2LB03	TRINITY_DN38075_c0_g1_i1_orf1	TRINITY_DN3393_c0_g2_i1_orf1	0	0	0	0	0.996	0.999	0.803	0.906	0.999
A0A3S2L174	A0A3S2LDJ5	TRINITY_DN45449_c0_g1_i1_orf1	TRINITY_DN3649_c0_g1_i6_orf1	0	0	0	0	0.146	0.999	0.712	0.79	0.999
A0A437BUX5	A0A3S2LEB1	TRINITY_DN1725_c0_g1_i7_orf1	TRINITY_DN7464_c1_g1_i1_orf1	0	0	0.383	0.403	0.999	0.948	0.745	0.738	0.999
A0A437BRA4	A0A3S2LEE0	TRINITY_DN20279_c0_g1_i1_orf1	TRINITY_DN9558_c0_g1_i2_orf1	0	0	0	0	0.959	0.948	0.95	0.619	0.999
A0A437AZ46	A0A3S2LHU2	TRINITY_DN5857_c0_g1_i13_orf1	TRINITY_DN620_c0_g1_i4_orf1	0	0	0	0	0.303	0.945	0.755	0.864	0.999
A0A3S2LEV6	A0A3S2LUB1	TRINITY_DN87603_c0_g2_i1_orf1	TRINITY_DN16939_c0_g1_i4_orf1	0.461	0	0	0	0.849	0.945	0	0.862	0.999
A0A437B0D7	A0A3S2L860	TRINITY_DN8949_c0_g1_i2_orf1	TRINITY_DN58207_c0_g1_i1_orf1	0.061	0	0	0	0.853	0.999	0.815	0.533	0.999
A0A437BIM5	A0A3S2L860	TRINITY_DN9101_c0_g2_i1_orf1	TRINITY_DN58207_c0_g1_i1_orf1	0	0	0	0	0.996	0.999	0.943	0.757	0.999
A0A437BQJ6	A0A3S2LB03	TRINITY_DN146718_c0_g1_i1_orf1	TRINITY_DN3393_c0_g2_i1_orf1	0	0	0.228	0	0.981	0.999	0.968	0.783	0.999
A0A3S2LVN6	A0A3S2LB03	TRINITY_DN7464_c0_g1_i14_orf1	TRINITY_DN3393_c0_g2_i1_orf1	0	0	0	0	0.997	0.999	0.581	0.663	0.999
A0A3S2NAD9	A0A3S2LD85	TRINITY_DN1044_c0_g1_i2_orf1	TRINITY_DN17351_c0_g1_i3_orf1	0	0	0	0	0.605	0.968	0.997	0.815	0.999
A0A3S2TJI5	A0A3S2LQK2	TRINITY_DN16749_c0_g1_i1_orf1	TRINITY_DN19829_c0_g1_i1_orf1	0	0	0	0	0.302	0.904	0.894	0.878	0.999
A0A437BV73	A0A3S2LSK5	TRINITY_DN31663_c0_g1_i2_orf1	TRINITY_DN4380_c0_g1_i9_orf1	0	0	0	0	0.186	0.876	0.995	0	0.999
