# Supplementary material for: Comparative Proteomics and Metabonomics Analysis of Different Diapause Stages Revealed a New Regulation Mechanism of Diapause in Loxostege sticticalis (Lepidoptera: Pyralidae)
Source: Molecules. 2024 Jul 25;29(15):3472. doi: 10.3390/molecules29153472 (PMC11314584; doi:10.3390/molecules29153472)
Supplement: Supplementary file 1 [file molecules-29-03472-s001.zip › analysis process/proteomic/sample information.pdf]

Sample name	Analysis n	Group name
C11_CK1	ND1	CK
C12_CK2	ND2	CK
C13_CK3	ND3	CK
C21_ZYQ1	PreD1	ZYQ
C22_ZYQ2	PreD2	ZYQ
C23_ZYQ3	PreD3	ZYQ
C31_ZY1	D1	ZY
C32_ZY2	D2	ZY
C33_ZY3	D3	ZY
C41_LCL1	CT1	LCL
C42_LCL2	CT2	LCL
C43_LCL3	CT3	LCL
C51_JCZY1	RD1	JCZY
C52_JCZY2	RD2	JCZY
C53_JCZY3	RD3	JCZY
