# Supplementary material for: Comparative Proteomics and Metabonomics Analysis of Different Diapause Stages Revealed a New Regulation Mechanism of Diapause in Loxostege sticticalis (Lepidoptera: Pyralidae)
Source: Molecules. 2024 Jul 25;29(15):3472. doi: 10.3390/molecules29153472 (PMC11314584; doi:10.3390/molecules29153472)
Supplement: Supplementary file 1 [file molecules-29-03472-s001.zip › analysis process/proteomic/WGCNA/Module Component Details Table.pdf]

protein id	module	kME
TRINITY_DN280_c0_g1_i8_orf1	blue	0.93892
TRINITY_DN20776_c0_g1_i3_orf1	turquoise	0.99613
TRINITY_DN5019_c0_g1_i2_orf1	turquoise	0.99686
TRINITY_DN146126_c0_g1_i1_orf1	turquoise	0.69698
TRINITY_DN125565_c1_g1_i1_orf1	green	0.74148
TRINITY_DN1856_c0_g1_i3_orf1	turquoise	0.79882
TRINITY_DN4360_c0_g1_i4_orf1	brown	0.40461
TRINITY_DN10479_c0_g1_i6_orf1	red	0.95606
TRINITY_DN142442_c0_g1_i1_orf1	green	0.77696
TRINITY_DN9280_c0_g1_i1_orf1	turquoise	0.53979
TRINITY_DN3194_c0_g1_i6_orf1	turquoise	0.99199
TRINITY_DN11492_c0_g1_i8_orf1	green	0.74965
TRINITY_DN2407_c0_g1_i2_orf1	brown	0.62676
TRINITY_DN12367_c0_g1_i8_orf1	green	0.78058
TRINITY_DN19980_c0_g1_i4_orf1	black	0.94726
TRINITY_DN2172_c0_g2_i8_orf1	red	0.92514
TRINITY_DN8019_c0_g1_i4_orf1	turquoise	0.99181
TRINITY_DN23640_c0_g1_i5_orf1	green	0.90538
TRINITY_DN1274_c0_g1_i4_orf1	blue	0.84496
TRINITY_DN2054_c0_g1_i1_orf1	red	0.94082
TRINITY_DN14755_c0_g1_i4_orf1	yellow	0.54053
TRINITY_DN1884_c0_g2_i2_orf1	turquoise	0.8524
TRINITY_DN4741_c0_g1_i4_orf1	turquoise	0.45682
TRINITY_DN5829_c0_g1_i1_orf1	blue	0.9688
TRINITY_DN5262_c0_g1_i7_orf1	turquoise	0.97382
TRINITY_DN3673_c0_g1_i10_orf1	turquoise	0.887
TRINITY_DN2076_c0_g2_i1_orf1	brown	0.83974
TRINITY_DN3913_c0_g1_i6_orf1	yellow	0.96828
TRINITY_DN7414_c0_g1_i1_orf1	turquoise	0.8323
TRINITY_DN2695_c0_g1_i8_orfp1	turquoise	0.5257
TRINITY_DN14826_c0_g1_i1_orf1	turquoise	0.98766
TRINITY_DN6063_c1_g2_i1_orf1	turquoise	0.93502
TRINITY_DN3732_c1_g1_i5_orf1	yellow	0.69541
TRINITY_DN31163_c1_g1_i4_orf1	brown	0.76767
TRINITY_DN31611_c0_g1_i2_orf1	green	0.9349
TRINITY_DN11050_c0_g1_i8_orf1	turquoise	0.75664
TRINITY_DN59335_c0_g1_i2_orf1	turquoise	0.94575
TRINITY_DN46140_c0_g1_i1_orf1	turquoise	0.72227
TRINITY_DN14935_c0_g1_i1_orf1	turquoise	0.57261
TRINITY_DN11013_c0_g1_i3_orf1	turquoise	0.97385
TRINITY_DN18404_c0_g1_i5_orf1	turquoise	0.98441
TRINITY_DN10415_c0_g1_i5_orf1	red	0.67298
TRINITY_DN50787_c0_g2_i2_orf1	turquoise	0.97264
TRINITY_DN448_c0_g1_i20_orf1	brown	0.95897
TRINITY_DN11666_c0_g1_i6_orf1	blue	0.79378
TRINITY_DN2497_c0_g1_i2_orf1	turquoise	0.87899
TRINITY_DN19639_c0_g2_i1_orf1	blue	0.86531
TRINITY_DN125521_c0_g2_i1_orf1	brown	0.88713
TRINITY_DN51776_c0_g2_i1_orf1	yellow	0.64208
TRINITY_DN11060_c0_g1_i6_orf1	black	0.97547
TRINITY_DN7920_c0_g1_i2_orf1	turquoise	0.95303
TRINITY_DN1108_c1_g2_i1_orfp1	brown	0.82534
TRINITY_DN21492_c0_g1_i1_orf1	turquoise	0.89412
TRINITY_DN2097_c1_g2_i2_orf1	blue	0.83736
TRINITY_DN16316_c0_g1_i7_orf1	black	0.63196
TRINITY_DN9615_c0_g1_i1_orf1	green	0.83845
TRINITY_DN9311_c0_g1_i1_orf1	blue	0.93855

TRINITY_DN124711_c0_g1_i1_orf1	grey	-0.13227
TRINITY_DN2345_c0_g1_i4_orf1	red	0.89352
TRINITY_DN110231_c0_g1_i1_orf1	blue	0.94954
TRINITY_DN3715_c0_g1_i2_orf1	blue	0.92229
TRINITY_DN4748_c0_g1_i5_orf1	blue	0.89682
TRINITY_DN146138_c0_g1_i1_orf1	green	0.8203
TRINITY_DN6396_c0_g1_i1_orf1	turquoise	0.90375
TRINITY_DN7102_c0_g1_i5_orf1	green	0.87596
TRINITY_DN33883_c0_g1_i1_orf1	turquoise	0.95772
TRINITY_DN15591_c0_g1_i3_orf1	red	0.80694
TRINITY_DN1313_c0_g1_i2_orf1	red	0.9325
TRINITY_DN4014_c0_g1_i1_orf1	turquoise	0.51821
TRINITY_DN79734_c0_g2_i3_orf1	turquoise	0.94595
TRINITY_DN10304_c0_g2_i1_orf1	blue	0.96217
TRINITY_DN36061_c0_g4_i2_orf1	red	0.97214
TRINITY_DN48250_c0_g1_i1_orf1	green	0.95471
TRINITY_DN11172_c1_g1_i1_orf1	green	0.54675
TRINITY_DN39509_c0_g1_i1_orf1	grey	0.81992
TRINITY_DN6199_c2_g1_i3_orf1	blue	0.69515
TRINITY_DN69535_c0_g1_i2_orf1	turquoise	0.83259
TRINITY_DN446_c0_g1_i20_orf1	turquoise	0.73719
TRINITY_DN4343_c0_g1_i2_orf1	brown	0.91893
TRINITY_DN3753_c0_g1_i7_orf1	turquoise	0.93624
TRINITY_DN4695_c0_g1_i4_orf1	brown	0.87528
TRINITY_DN18222_c0_g1_i5_orf1	brown	0.68415
TRINITY_DN146493_c0_g1_i1_orf1	turquoise	0.55327
TRINITY_DN25916_c0_g1_i1_orf1	turquoise	0.708
TRINITY_DN252_c0_g1_i3_orf1	red	0.79618
TRINITY_DN85290_c0_g2_i1_orf1	brown	0.615
TRINITY_DN3760_c0_g1_i1_orf1	turquoise	0.81203
TRINITY_DN5046_c0_g3_i1_orf1	red	0.86356
TRINITY_DN9536_c0_g1_i4_orf1	red	0.86094
TRINITY_DN9044_c0_g1_i1_orf1	green	0.78173
TRINITY_DN14063_c0_g1_i7_orf1	turquoise	0.96626
TRINITY_DN3022_c0_g1_i1_orf1	brown	0.75228
TRINITY_DN51424_c0_g2_i1_orf1	brown	0.86418
TRINITY_DN19990_c0_g1_i1_orf1	blue	0.98166
TRINITY_DN1578_c0_g3_i1_orf1	turquoise	0.95572
TRINITY_DN27751_c0_g2_i1_orf1	turquoise	0.97585
TRINITY_DN10066_c0_g2_i2_orf1	turquoise	0.80021
TRINITY_DN30713_c0_g1_i3_orf1	brown	0.48454
TRINITY_DN307_c1_g1_i1_orf1	green	0.74865
TRINITY_DN135780_c0_g1_i1_orf1	brown	0.94228
TRINITY_DN9998_c0_g1_i2_orf1	yellow	0.73982
TRINITY_DN11616_c0_g1_i3_orf1	turquoise	0.86384
TRINITY_DN3667_c0_g1_i4_orf1	red	0.49272
TRINITY_DN16931_c0_g1_i1_orf1	turquoise	0.97076
TRINITY_DN122423_c0_g1_i1_orf1	red	0.71461
TRINITY_DN10900_c0_g1_i7_orf1	brown	0.51372
TRINITY_DN6044_c0_g1_i4_orf1	turquoise	0.99062
TRINITY_DN29369_c0_g1_i1_orf1	black	0.79645
TRINITY_DN32583_c0_g1_i4_orf1	grey	0.81328
TRINITY_DN14274_c0_g1_i3_orf1	blue	0.78025
TRINITY_DN9248_c0_g1_i10_orf1	turquoise	0.83146
TRINITY_DN44083_c0_g1_i2_orf1	brown	0.96773
TRINITY_DN12767_c0_g1_i2_orf1	turquoise	0.73506
TRINITY_DN1212_c0_g1_i8_orf1	yellow	0.95938
TRINITY_DN717_c0_g1_i2_orfp1	turquoise	0.98416

TRINITY_DN6247_c0_g1_i2_orf1	turquoise	0.91339
TRINITY_DN22272_c0_g1_i1_orf1	green	0.36279
TRINITY_DN18323_c0_g1_i5_orf1	green	0.9119
TRINITY_DN105574_c0_g1_i1_orf1	black	0.91154
TRINITY_DN38180_c0_g1_i3_orf1	brown	0.86434
TRINITY_DN2962_c0_g1_i7_orf1	yellow	0.3604
TRINITY_DN20294_c0_g2_i1_orf1	turquoise	0.99402
TRINITY_DN578_c0_g1_i5_orf1	green	0.93989
TRINITY_DN71465_c0_g1_i1_orf1	turquoise	0.97604
TRINITY_DN2514_c1_g1_i13_orf1	brown	0.89793
TRINITY_DN17189_c0_g1_i2_orf1	turquoise	0.74082
TRINITY_DN21533_c0_g1_i6_orf1	black	0.84033
TRINITY_DN33146_c0_g1_i1_orf1	turquoise	0.43783
TRINITY_DN3027_c0_g1_i4_orf1	turquoise	0.98064
TRINITY_DN12464_c0_g1_i3_orf1	blue	0.89525
TRINITY_DN12250_c0_g1_i4_orf1	turquoise	0.8415
TRINITY_DN12584_c0_g1_i1_orf1	turquoise	0.94174
TRINITY_DN122393_c0_g1_i1_orf1	yellow	0.93327
TRINITY_DN59429_c0_g1_i6_orf1	brown	0.93339
TRINITY_DN13799_c0_g1_i1_orf1	green	0.94617
TRINITY_DN1144_c0_g1_i10_orf1	green	0.95316
TRINITY_DN787_c0_g1_i7_orf1	turquoise	0.86709
TRINITY_DN30300_c0_g2_i1_orf1	green	0.81074
TRINITY_DN4429_c0_g1_i5_orf1	turquoise	0.88938
TRINITY_DN3037_c0_g1_i1_orf1	pink	0.63025
TRINITY_DN4501_c0_g2_i1_orf1	yellow	0.72541
TRINITY_DN21341_c0_g1_i1_orf1	green	0.48673
TRINITY_DN10662_c0_g1_i4_orf1	turquoise	0.94477
TRINITY_DN40911_c0_g1_i1_orf1	yellow	0.43294
TRINITY_DN51441_c0_g1_i5_orf1	red	0.90555
TRINITY_DN3835_c0_g1_i3_orf1	turquoise	0.9221
TRINITY_DN8076_c0_g1_i6_orf1	turquoise	0.68487
TRINITY_DN3406_c0_g1_i17_orf1	brown	0.90314
TRINITY_DN13312_c0_g2_i1_orf1	yellow	0.75671
TRINITY_DN10766_c0_g1_i1_orf1	blue	0.97001
TRINITY_DN82320_c0_g1_i2_orf1	turquoise	0.94879
TRINITY_DN26251_c0_g1_i1_orf1	turquoise	0.86477
TRINITY_DN1750_c1_g1_i5_orf1	brown	0.95343
TRINITY_DN22242_c0_g2_i1_orf1	brown	0.85113
TRINITY_DN4183_c0_g1_i8_orf1	turquoise	0.97048
TRINITY_DN558_c0_g1_i4_orf1	pink	0.85033
TRINITY_DN140669_c0_g1_i1_orf1	turquoise	0.97573
TRINITY_DN1993_c0_g1_i1_orf1	yellow	0.9011
TRINITY_DN69_c0_g1_i1_orf1	brown	0.97396
TRINITY_DN2186_c0_g1_i17_orf1	yellow	0.85126
TRINITY_DN53427_c0_g1_i2_orf1	blue	0.8788
TRINITY_DN5182_c0_g1_i5_orf1	red	0.90603
TRINITY_DN12242_c0_g1_i5_orf1	turquoise	0.87075
TRINITY_DN11657_c0_g1_i2_orf1	turquoise	0.79868
TRINITY_DN9458_c0_g1_i4_orf1	brown	0.27559
TRINITY_DN7670_c0_g1_i1_orf1	red	0.87827
TRINITY_DN8953_c0_g1_i4_orf1	brown	0.89243
TRINITY_DN13944_c0_g1_i1_orf1	turquoise	0.91584
TRINITY_DN4016_c0_g1_i1_orf1	turquoise	0.66565
TRINITY_DN8352_c0_g1_i3_orf1	turquoise	0.77849
TRINITY_DN21357_c0_g1_i5_orf1	turquoise	0.94908
TRINITY_DN1954_c0_g1_i4_orf1	yellow	0.53067
TRINITY_DN10629_c0_g1_i1_orf1	turquoise	0.60959

TRINITY_DN8087_c0_g1_i9_orf1	turquoise	0.94457
TRINITY_DN98242_c0_g1_i1_orf1	red	0.925
TRINITY_DN2489_c0_g1_i1_orf1	blue	0.99386
TRINITY_DN48638_c0_g1_i5_orf1	turquoise	0.85458
TRINITY_DN2146_c0_g1_i1_orf1	turquoise	0.67684
TRINITY_DN13411_c0_g1_i4_orf1	black	0.9317
TRINITY_DN32769_c1_g1_i5_orf1	turquoise	0.9458
TRINITY_DN959_c0_g1_i7_orf1	turquoise	0.9154
TRINITY_DN4135_c0_g1_i5_orf1	turquoise	0.96844
TRINITY_DN2535_c0_g1_i4_orf1	turquoise	0.85013
TRINITY_DN18388_c0_g1_i6_orf1	green	0.91757
TRINITY_DN37599_c0_g1_i1_orf1	turquoise	0.87821
TRINITY_DN2700_c2_g1_i1_orf1	brown	0.61585
TRINITY_DN13098_c2_g1_i2_orf1	green	0.9596
TRINITY_DN7991_c0_g1_i9_orf1	turquoise	0.94807
TRINITY_DN2876_c0_g1_i3_orf1	turquoise	0.81671
TRINITY_DN825_c23_g1_i5_orf1	yellow	0.54826
TRINITY_DN14856_c0_g1_i1_orf1	blue	0.95879
TRINITY_DN27045_c0_g1_i1_orf1	yellow	0.72968
TRINITY_DN36899_c0_g1_i1_orf1	blue	0.90992
TRINITY_DN5569_c0_g1_i1_orf1	blue	0.59311
TRINITY_DN4725_c0_g1_i4_orf1	turquoise	0.96581
TRINITY_DN14009_c0_g1_i1_orf1	blue	0.91993
TRINITY_DN57998_c1_g1_i1_orf1	blue	0.97396
TRINITY_DN8651_c0_g1_i16_orf1	brown	0.83252
TRINITY_DN21367_c0_g1_i1_orf1	brown	0.66339
TRINITY_DN47609_c0_g1_i1_orfp1	green	0.8522
TRINITY_DN1173_c1_g1_i9_orf1	turquoise	0.94678
TRINITY_DN11259_c0_g1_i1_orf1	turquoise	0.97848
TRINITY_DN13598_c1_g1_i1_orf1	turquoise	0.83933
TRINITY_DN18681_c0_g1_i7_orf1	turquoise	0.80395
TRINITY_DN19810_c1_g1_i7_orf1	red	0.9132
TRINITY_DN2035_c0_g1_i1_orf1	black	0.93915
TRINITY_DN49143_c0_g1_i1_orf1	blue	0.97673
TRINITY_DN10234_c0_g1_i1_orf1	turquoise	0.52063
TRINITY_DN1337_c0_g2_i1_orf1	turquoise	0.89476
TRINITY_DN5556_c0_g1_i3_orf1	green	0.95757
TRINITY_DN79210_c0_g1_i1_orf1	turquoise	0.98419
TRINITY_DN37821_c0_g1_i6_orf1	black	0.90517
TRINITY_DN50517_c0_g1_i3_orf1	blue	0.77072
TRINITY_DN26789_c0_g1_i2_orf1	green	0.77829
TRINITY_DN51239_c0_g1_i5_orf1	turquoise	0.82384
TRINITY_DN35662_c0_g1_i5_orf1	turquoise	0.98942
TRINITY_DN141353_c0_g1_i1_orf1	grey	0.61855
TRINITY_DN25901_c0_g1_i2_orf1	turquoise	0.82576
TRINITY_DN28366_c0_g1_i1_orf1	turquoise	0.42471
TRINITY_DN20185_c0_g1_i6_orf1	red	0.88719
TRINITY_DN2184_c0_g1_i4_orf1	turquoise	0.91654
TRINITY_DN5740_c0_g1_i4_orf1	turquoise	0.98714
TRINITY_DN41997_c0_g1_i2_orf1	turquoise	0.90457
TRINITY_DN13511_c0_g1_i4_orf1	black	0.73994
TRINITY_DN1860_c0_g1_i1_orf1	grey	-0.38337
TRINITY_DN33038_c0_g1_i1_orf1	turquoise	0.88681
TRINITY_DN2942_c0_g1_i6_orf1	red	0.86936
TRINITY_DN1455_c0_g1_i4_orf1	brown	0.91679
TRINITY_DN29229_c0_g1_i4_orf1	turquoise	0.7805
TRINITY_DN91989_c0_g1_i1_orf1	turquoise	0.97369
TRINITY_DN4108_c0_g1_i6_orf1	black	0.91411

TRINITY_DN33272_c0_g1_i5_orf1	black	0.95367
TRINITY_DN40823_c0_g1_i1_orf1	turquoise	0.88348
TRINITY_DN1422_c0_g1_i4_orf1	turquoise	0.98717
TRINITY_DN19160_c0_g1_i1_orf1	red	0.89978
TRINITY_DN288_c0_g1_i9_orf1	red	0.90533
TRINITY_DN8854_c0_g1_i2_orf1	brown	0.86579
TRINITY_DN2227_c0_g1_i5_orf1	blue	0.83197
TRINITY_DN1986_c0_g1_i1_orf1	blue	0.9638
TRINITY_DN46090_c0_g2_i1_orf1	red	0.87256
TRINITY_DN11044_c0_g1_i4_orf1	black	0.78891
TRINITY_DN53294_c0_g1_i1_orf1	green	0.79627
TRINITY_DN886_c0_g2_i4_orf1	turquoise	0.95713
TRINITY_DN1013_c0_g1_i3_orf1	turquoise	0.59427
TRINITY_DN17828_c0_g1_i1_orf1	turquoise	0.49432
TRINITY_DN1285_c0_g1_i6_orf1	turquoise	0.99586
TRINITY_DN17615_c0_g1_i3_orf1	brown	0.95245
TRINITY_DN4695_c0_g1_i3_orf1	brown	0.6836
TRINITY_DN5169_c0_g1_i5_orf1	turquoise	0.93073
TRINITY_DN2407_c0_g1_i6_orf1	blue	0.84303
TRINITY_DN13419_c0_g1_i5_orf1	blue	0.69668
TRINITY_DN9316_c1_g1_i1_orf1	turquoise	0.97059
TRINITY_DN8584_c0_g1_i6_orf1	turquoise	0.82053
TRINITY_DN2374_c0_g2_i2_orf1	green	0.80568
TRINITY_DN1491_c0_g1_i8_orf1	blue	0.98688
TRINITY_DN1080_c0_g1_i1_orf1	black	0.94441
TRINITY_DN6161_c0_g1_i1_orf1	blue	0.68616
TRINITY_DN9510_c0_g2_i1_orf1	turquoise	0.4825
TRINITY_DN6653_c0_g1_i1_orf1	yellow	0.74305
TRINITY_DN17406_c0_g1_i1_orf1	blue	0.71262
TRINITY_DN1254_c0_g1_i1_orf1	blue	0.66861
TRINITY_DN1498_c0_g1_i2_orf1	turquoise	0.88129
TRINITY_DN971_c0_g1_i10_orfp1	brown	0.95599
TRINITY_DN1073_c0_g1_i3_orf1	yellow	0.83074
TRINITY_DN1772_c1_g3_i1_orf1	turquoise	0.87853
TRINITY_DN21743_c0_g1_i2_orf1	pink	0.66922
TRINITY_DN11274_c0_g1_i4_orf1	green	0.83625
TRINITY_DN2532_c0_g3_i1_orf1	turquoise	0.36552
TRINITY_DN891_c5_g1_i5_orf1	red	0.64821
TRINITY_DN9420_c0_g1_i2_orf1	black	0.87723
TRINITY_DN69049_c0_g2_i1_orf1	turquoise	0.82197
TRINITY_DN8701_c0_g1_i3_orf1	red	0.87317
TRINITY_DN46132_c0_g2_i2_orf1	yellow	0.80227
TRINITY_DN1901_c0_g1_i6_orf1	turquoise	0.98833
TRINITY_DN13783_c0_g4_i2_orf1	turquoise	0.9937
TRINITY_DN280_c0_g1_i12_orf1	turquoise	0.86413
TRINITY_DN53866_c0_g1_i1_orf1	blue	0.97179
TRINITY_DN7867_c0_g1_i1_orf1	green	0.94676
TRINITY_DN14922_c0_g3_i2_orf1	yellow	0.93566
TRINITY_DN83622_c0_g1_i2_orf1	brown	0.69057
TRINITY_DN21150_c0_g1_i4_orf1	turquoise	0.95026
TRINITY_DN9542_c0_g1_i4_orf1	green	0.93475
TRINITY_DN590_c0_g1_i4_orf1	blue	0.81657
TRINITY_DN98723_c1_g1_i1_orf1	turquoise	0.86925
TRINITY_DN131924_c0_g1_i2_orf1	brown	0.37659
TRINITY_DN5243_c1_g1_i1_orf1	turquoise	0.60222
TRINITY_DN19286_c0_g1_i1_orf1	turquoise	0.8663
TRINITY_DN125150_c0_g1_i1_orf1	black	0.91571
TRINITY_DN1592_c0_g1_i1_orf1	brown	0.90561

TRINITY_DN1084_c0_g1_i2_orf1	brown	0.90177
TRINITY_DN97097_c0_g1_i4_orf1	blue	0.92373
TRINITY_DN16749_c0_g1_i1_orf1	turquoise	0.9695
TRINITY_DN20682_c0_g1_i2_orf1	turquoise	0.90757
TRINITY_DN4681_c0_g2_i2_orf1	turquoise	0.65473
TRINITY_DN6510_c1_g1_i1_orf1	blue	0.78636
TRINITY_DN38435_c0_g1_i1_orf1	turquoise	0.65771
TRINITY_DN60949_c0_g1_i4_orf1	black	0.69447
TRINITY_DN13923_c0_g2_i1_orf1	turquoise	0.57116
TRINITY_DN5813_c0_g1_i9_orf1	brown	0.87589
TRINITY_DN9593_c0_g1_i2_orf1	green	0.94268
TRINITY_DN1865_c1_g1_i3_orf1	pink	0.70128
TRINITY_DN7580_c0_g1_i1_orf1	yellow	0.77846
TRINITY_DN42333_c0_g1_i5_orf1	blue	0.81249
TRINITY_DN181_c0_g1_i3_orf1	turquoise	0.82027
TRINITY_DN2403_c0_g1_i3_orf1	turquoise	0.61943
TRINITY_DN34426_c0_g1_i1_orf1	blue	0.6303
TRINITY_DN4686_c0_g2_i1_orf1	red	0.92579
TRINITY_DN37141_c0_g1_i2_orf1	turquoise	0.80454
TRINITY_DN5893_c0_g1_i7_orf1	blue	0.92345
TRINITY_DN3732_c0_g1_i2_orf1	turquoise	0.44148
TRINITY_DN4235_c0_g1_i2_orf1	green	0.94725
TRINITY_DN817_c0_g1_i3_orf1	turquoise	0.83811
TRINITY_DN8676_c0_g1_i1_orf1	turquoise	0.98295
TRINITY_DN15458_c0_g1_i3_orf1	turquoise	0.71608
TRINITY_DN54477_c0_g1_i1_orf1	turquoise	0.56954
TRINITY_DN45598_c0_g1_i2_orf1	turquoise	0.98138
TRINITY_DN18937_c0_g1_i1_orf1	turquoise	0.55734
TRINITY_DN28922_c0_g1_i2_orf1	blue	0.97927
TRINITY_DN2442_c0_g1_i2_orf1	blue	0.96476
TRINITY_DN58261_c0_g1_i2_orf1	yellow	0.84764
TRINITY_DN110519_c0_g1_i1_orf1	blue	0.93986
TRINITY_DN129863_c0_g1_i1_orf1	black	0.89611
TRINITY_DN4341_c0_g1_i4_orf1	brown	0.81022
TRINITY_DN4143_c0_g1_i1_orf1	turquoise	0.89196
TRINITY_DN4636_c0_g3_i1_orf1	yellow	0.56303
TRINITY_DN3105_c0_g1_i4_orf1	yellow	0.76779
TRINITY_DN2953_c1_g1_i10_orf1	blue	0.98007
TRINITY_DN11826_c0_g1_i4_orf1	green	0.97047
TRINITY_DN122786_c0_g2_i1_orf1	turquoise	0.90051
TRINITY_DN14754_c0_g1_i6_orf1	yellow	0.81242
TRINITY_DN15865_c0_g1_i1_orf1	red	0.90218
TRINITY_DN7622_c0_g2_i2_orf1	green	0.63197
TRINITY_DN2954_c0_g1_i1_orf1	turquoise	0.91413
TRINITY_DN23740_c1_g1_i1_orf1	turquoise	0.67102
TRINITY_DN54711_c0_g1_i1_orf1	turquoise	0.8915
TRINITY_DN108818_c0_g1_i5_orf1	turquoise	0.84
TRINITY_DN21782_c0_g1_i8_orf1	turquoise	0.92085
TRINITY_DN1710_c0_g2_i2_orf1	grey	-0.75315
TRINITY_DN22944_c0_g3_i1_orf1	green	0.77774
TRINITY_DN8046_c0_g1_i5_orf1	turquoise	0.41989
TRINITY_DN4133_c0_g1_i2_orfp2	yellow	0.80362
TRINITY_DN74889_c0_g1_i1_orf1	turquoise	0.89433
TRINITY_DN9916_c0_g1_i1_orf1	red	0.95893
TRINITY_DN20960_c0_g1_i1_orf1	yellow	0.61548
TRINITY_DN4125_c0_g1_i14_orf1	blue	0.88061
TRINITY_DN50571_c1_g1_i1_orf1	turquoise	0.8696
TRINITY_DN2178_c0_g1_i1_orf1	turquoise	0.96743

TRINITY_DN141462_c0_g1_i1_orf1	turquoise	0.99258
TRINITY_DN5439_c0_g1_i2_orf1	brown	0.95102
TRINITY_DN5503_c0_g1_i5_orf1	blue	0.62124
TRINITY_DN1465_c2_g1_i2_orf1	blue	0.97017
TRINITY_DN10364_c0_g1_i5_orf1	blue	0.97825
TRINITY_DN28152_c0_g1_i1_orf1	turquoise	0.98238
TRINITY_DN1116_c0_g1_i6_orf1	brown	0.54709
TRINITY_DN6436_c0_g1_i1_orf1	red	0.95114
TRINITY_DN1066_c0_g1_i4_orf1	turquoise	0.99212
TRINITY_DN7803_c0_g1_i2_orf1	red	0.89304
TRINITY_DN3985_c0_g2_i1_orf1	turquoise	0.90388
TRINITY_DN38371_c0_g1_i7_orf1	blue	0.73611
TRINITY_DN646_c0_g1_i5_orf1	turquoise	0.9732
TRINITY_DN2544_c1_g1_i2_orf1	yellow	0.77406
TRINITY_DN14347_c0_g1_i1_orf1	grey	-0.74211
TRINITY_DN16482_c0_g1_i6_orf1	turquoise	0.97765
TRINITY_DN830_c0_g1_i4_orf1	brown	0.66634
TRINITY_DN43637_c0_g1_i1_orf1	turquoise	0.47927
TRINITY_DN13384_c0_g1_i1_orf1	turquoise	0.5844
TRINITY_DN14389_c0_g1_i4_orf1	blue	0.88967
TRINITY_DN1444_c1_g1_i5_orf1	red	0.95723
TRINITY_DN5495_c0_g1_i5_orf1	blue	0.86408
TRINITY_DN3952_c0_g1_i3_orf1	blue	0.90728
TRINITY_DN11069_c0_g1_i6_orf1	turquoise	0.88747
TRINITY_DN14301_c0_g2_i1_orf1	turquoise	0.96044
TRINITY_DN64627_c0_g1_i1_orf1	turquoise	0.73402
TRINITY_DN184_c0_g1_i10_orf1	black	0.86893
TRINITY_DN18860_c0_g1_i1_orf1	turquoise	0.98105
TRINITY_DN15685_c0_g1_i5_orf1	yellow	0.82268
TRINITY_DN1245_c0_g1_i4_orf1	brown	0.78281
TRINITY_DN338_c1_g1_i9_orf1	green	0.94541
TRINITY_DN60358_c0_g1_i3_orf1	green	0.61832
TRINITY_DN2044_c0_g1_i5_orfp1	brown	0.94136
TRINITY_DN1098_c1_g1_i4_orf1	green	0.86835
TRINITY_DN4767_c0_g1_i6_orf1	green	0.98441
TRINITY_DN33272_c0_g1_i1_orf1	blue	0.84833
TRINITY_DN13287_c0_g1_i5_orf1	turquoise	0.60525
TRINITY_DN120089_c0_g1_i1_orf1	yellow	0.84659
TRINITY_DN6140_c0_g3_i3_orf1	turquoise	0.96846
TRINITY_DN12526_c0_g1_i5_orf1	blue	0.77121
TRINITY_DN108051_c0_g1_i2_orf1	turquoise	0.98744
TRINITY_DN45633_c0_g1_i1_orf1	turquoise	0.80196
TRINITY_DN1820_c0_g1_i6_orf1	turquoise	0.98078
TRINITY_DN2331_c0_g1_i1_orf1	turquoise	0.37049
TRINITY_DN82810_c0_g1_i1_orf1	turquoise	0.81992
TRINITY_DN79673_c0_g1_i1_orf1	turquoise	0.68421
TRINITY_DN94355_c0_g1_i2_orf1	turquoise	0.98971
TRINITY_DN957_c0_g1_i18_orf1	grey	0.20556
TRINITY_DN7405_c0_g1_i3_orf1	turquoise	0.91755
TRINITY_DN8659_c0_g2_i1_orf1	blue	0.86682
TRINITY_DN3584_c0_g1_i3_orf1	turquoise	0.74598
TRINITY_DN5593_c0_g1_i1_orf1	turquoise	0.83677
TRINITY_DN6482_c0_g1_i1_orf1	yellow	0.86986
TRINITY_DN5233_c0_g1_i1_orf1	turquoise	0.80596
TRINITY_DN298_c0_g1_i4_orf1	turquoise	0.84339
TRINITY_DN7603_c0_g1_i5_orf1	red	0.96761
TRINITY_DN9759_c0_g1_i1_orf1	turquoise	0.82717
TRINITY_DN1423_c0_g1_i4_orf1	blue	0.90043

TRINITY_DN429_c0_g1_i12_orf1	red	0.84384
TRINITY_DN29018_c0_g1_i4_orf1	turquoise	0.97347
TRINITY_DN10441_c0_g1_i3_orf1	yellow	0.89116
TRINITY_DN3664_c0_g1_i8_orf1	turquoise	0.87864
TRINITY_DN135781_c0_g1_i1_orf1	brown	0.86675
TRINITY_DN2614_c0_g2_i3_orf1	green	0.85563
TRINITY_DN25582_c0_g1_i3_orf1	brown	0.92624
TRINITY_DN277_c1_g1_i1_orf1	red	0.90353
TRINITY_DN791_c0_g1_i2_orf1	turquoise	0.98813
TRINITY_DN9715_c0_g1_i1_orf1	turquoise	0.99117
TRINITY_DN31286_c0_g1_i6_orfp1	brown	0.95877
TRINITY_DN64141_c0_g1_i4_orf1	green	0.91698
TRINITY_DN9316_c0_g3_i1_orf1	brown	0.62378
TRINITY_DN100_c0_g1_i13_orf1	yellow	0.8026
TRINITY_DN7228_c0_g1_i6_orf1	turquoise	0.90405
TRINITY_DN14443_c0_g1_i1_orf1	red	0.98862
TRINITY_DN19034_c0_g1_i1_orf1	blue	0.72934
TRINITY_DN133228_c0_g1_i3_orf1	turquoise	0.7343
TRINITY_DN44557_c0_g1_i4_orf1	turquoise	0.90217
TRINITY_DN108_c0_g1_i1_orf1	green	0.78053
TRINITY_DN492_c0_g1_i4_orf1	turquoise	0.94877
TRINITY_DN5675_c0_g1_i6_orf1	blue	0.82713
TRINITY_DN154_c0_g1_i4_orf1	turquoise	0.9197
TRINITY_DN3312_c0_g1_i10_orf1	turquoise	0.96258
TRINITY_DN9591_c0_g1_i1_orf1	yellow	0.78438
TRINITY_DN11566_c2_g1_i2_orf1	red	0.48794
TRINITY_DN9435_c0_g1_i7_orf1	brown	0.82531
TRINITY_DN4390_c0_g1_i4_orf1	grey	0.76292
TRINITY_DN2186_c0_g1_i13_orf1	yellow	0.83058
TRINITY_DN19000_c0_g1_i4_orf1	turquoise	0.99464
TRINITY_DN13615_c0_g1_i3_orf1	green	0.85706
TRINITY_DN1407_c0_g1_i5_orf1	blue	0.95358
TRINITY_DN16451_c0_g1_i7_orf1	pink	0.89828
TRINITY_DN22175_c0_g1_i1_orf1	brown	0.78928
TRINITY_DN1528_c0_g1_i4_orf1	red	0.77435
TRINITY_DN2146_c0_g2_i1_orf1	green	0.86291
TRINITY_DN48410_c0_g1_i1_orf1	turquoise	0.98766
TRINITY_DN3283_c0_g2_i1_orf1	turquoise	0.74094
TRINITY_DN107035_c0_g1_i1_orf1	turquoise	0.98733
TRINITY_DN3649_c0_g1_i6_orf1	turquoise	0.68565
TRINITY_DN4908_c1_g1_i5_orf1	red	0.94924
TRINITY_DN12397_c0_g1_i1_orf1	grey	0.83441
TRINITY_DN27984_c0_g2_i1_orf1	blue	0.60564
TRINITY_DN3860_c0_g1_i5_orf1	turquoise	0.77682
TRINITY_DN44094_c0_g1_i1_orf1	blue	0.83639
TRINITY_DN101991_c0_g1_i5_orf1	yellow	0.91718
TRINITY_DN54543_c0_g5_i2_orf1	turquoise	0.98033
TRINITY_DN13371_c0_g1_i4_orf1	turquoise	0.87655
TRINITY_DN23175_c0_g1_i6_orf1	turquoise	0.99075
TRINITY_DN9119_c0_g1_i3_orf1	turquoise	0.96529
TRINITY_DN31751_c0_g1_i5_orf1	turquoise	0.9289
TRINITY_DN5300_c0_g1_i2_orf1	brown	0.9002
TRINITY_DN54336_c0_g1_i1_orf1	turquoise	0.72354
TRINITY_DN16840_c1_g1_i1_orf1	black	0.80557
TRINITY_DN18794_c0_g1_i5_orf1	turquoise	0.8543
TRINITY_DN18159_c0_g1_i6_orf1	turquoise	0.75229
TRINITY_DN31598_c0_g1_i1_orf1	blue	0.36687
TRINITY_DN27035_c0_g1_i1_orf1	green	0.95677

TRINITY_DN76633_c0_g1_i1_orfp1	brown	0.74808
TRINITY_DN6016_c0_g1_i8_orf1	turquoise	0.57841
TRINITY_DN85319_c0_g1_i1_orf1	turquoise	0.99779
TRINITY_DN14262_c0_g1_i5_orf1	brown	0.87394
TRINITY_DN39813_c0_g1_i1_orf1	turquoise	0.46673
TRINITY_DN45530_c0_g1_i1_orf1	green	0.97334
TRINITY_DN25733_c0_g1_i3_orf1	brown	0.69285
TRINITY_DN34455_c0_g1_i1_orf1	yellow	0.867
TRINITY_DN1322_c0_g1_i4_orf1	red	0.41563
TRINITY_DN37218_c0_g1_i12_orf1	turquoise	0.91319
TRINITY_DN53167_c0_g1_i2_orf1	turquoise	0.96971
TRINITY_DN30476_c0_g1_i1_orf1	yellow	0.79526
TRINITY_DN1079_c0_g1_i4_orf1	turquoise	0.98815
TRINITY_DN29698_c0_g1_i3_orf1	brown	0.9313
TRINITY_DN15578_c0_g2_i1_orfp1	black	0.92349
TRINITY_DN30233_c0_g1_i2_orf1	turquoise	0.98555
TRINITY_DN8651_c0_g1_i18_orf1	yellow	0.97786
TRINITY_DN13500_c0_g1_i1_orf1	brown	0.8492
TRINITY_DN99694_c0_g1_i1_orf1	brown	0.71854
TRINITY_DN43412_c0_g1_i2_orf1	turquoise	0.85222
TRINITY_DN13160_c0_g1_i1_orf1	turquoise	0.8613
TRINITY_DN4793_c0_g1_i7_orf1	yellow	0.88777
TRINITY_DN27500_c0_g1_i4_orf1	yellow	0.91758
TRINITY_DN3343_c0_g2_i1_orf1	turquoise	0.87714
TRINITY_DN21181_c0_g1_i6_orf1	turquoise	0.95241
TRINITY_DN7291_c0_g1_i3_orf1	blue	0.85254
TRINITY_DN41697_c0_g1_i1_orf1	turquoise	0.73469
TRINITY_DN4194_c0_g1_i1_orf1	red	0.85108
TRINITY_DN47257_c0_g1_i4_orf1	yellow	0.71355
TRINITY_DN4381_c0_g2_i1_orf1	turquoise	0.93329
TRINITY_DN868_c0_g1_i4_orf1	turquoise	0.90673
TRINITY_DN23164_c0_g1_i4_orf1	yellow	0.68965
TRINITY_DN6992_c0_g1_i6_orf1	turquoise	0.36953
TRINITY_DN27488_c0_g1_i9_orf1	grey	0.08576
TRINITY_DN1309_c0_g2_i1_orf1	turquoise	0.50215
TRINITY_DN43328_c0_g1_i1_orf1	red	0.94164
TRINITY_DN135679_c0_g1_i1_orfp1	turquoise	0.54193
TRINITY_DN4820_c0_g1_i1_orf1	turquoise	0.95235
TRINITY_DN2823_c0_g1_i6_orf1	brown	0.62359
TRINITY_DN28661_c0_g1_i1_orf1	black	0.97007
TRINITY_DN11467_c0_g1_i5_orf1	green	0.91974
TRINITY_DN4279_c0_g1_i4_orf1	grey	0.47419
TRINITY_DN12009_c0_g1_i1_orf1	black	0.86769
TRINITY_DN3045_c0_g1_i7_orf1	turquoise	0.66574
TRINITY_DN31609_c0_g1_i3_orf1	brown	0.77948
TRINITY_DN96557_c0_g1_i1_orf1	yellow	0.66573
TRINITY_DN37856_c0_g1_i5_orf1	turquoise	0.87946
TRINITY_DN29555_c0_g1_i8_orf1	black	0.92896
TRINITY_DN5274_c0_g2_i2_orf1	brown	0.94631
TRINITY_DN932_c0_g1_i4_orf1	turquoise	0.97329
TRINITY_DN17071_c0_g1_i6_orf1	turquoise	0.57291
TRINITY_DN3245_c2_g1_i4_orf1	turquoise	0.85653
TRINITY_DN972_c0_g2_i1_orf1	turquoise	0.82151
TRINITY_DN3836_c0_g1_i4_orf1	turquoise	0.91872
TRINITY_DN825_c8_g1_i5_orf1	turquoise	0.99112
TRINITY_DN32359_c0_g2_i1_orf1	red	0.83608
TRINITY_DN4898_c0_g1_i7_orf1	red	0.92858
TRINITY_DN4199_c0_g1_i1_orf1	turquoise	0.95298

TRINITY_DN14464_c0_g1_i1_orf1	turquoise	0.9334
TRINITY_DN2274_c0_g1_i6_orf1	turquoise	0.97617
TRINITY_DN61042_c0_g2_i2_orfp1	brown	0.74469
TRINITY_DN280_c4_g1_i5_orf1	pink	0.81813
TRINITY_DN21559_c0_g1_i2_orf1	turquoise	0.64711
TRINITY_DN4979_c0_g2_i9_orf1	blue	0.73553
TRINITY_DN15967_c0_g1_i4_orf1	blue	0.8016
TRINITY_DN146718_c0_g1_i1_orf1	turquoise	0.91503
TRINITY_DN6262_c0_g1_i2_orf1	turquoise	0.61946
TRINITY_DN5266_c0_g1_i1_orf1	brown	0.99239
TRINITY_DN2749_c0_g2_i3_orf1	turquoise	0.6077
TRINITY_DN2971_c0_g1_i1_orf1	blue	0.81365
TRINITY_DN6275_c0_g1_i3_orf1	black	0.90042
TRINITY_DN428_c0_g1_i8_orf1	green	0.9556
TRINITY_DN8716_c0_g1_i3_orf1	brown	0.69457
TRINITY_DN801_c0_g1_i2_orf1	blue	0.85303
TRINITY_DN36699_c0_g1_i3_orf1	turquoise	0.73782
TRINITY_DN1329_c0_g1_i5_orf1	yellow	0.90349
TRINITY_DN10174_c0_g1_i4_orf1	red	0.78753
TRINITY_DN2577_c0_g1_i1_orf1	turquoise	0.84782
TRINITY_DN73923_c0_g1_i1_orf1	red	0.97144
TRINITY_DN13686_c0_g2_i1_orf1	blue	0.98386
TRINITY_DN9872_c0_g1_i2_orf1	red	0.88256
TRINITY_DN52_c0_g1_i4_orf1	red	0.59548
TRINITY_DN842_c0_g1_i9_orf1	yellow	0.71909
TRINITY_DN37830_c0_g1_i1_orf1	turquoise	0.94881
TRINITY_DN1294_c0_g1_i3_orf1	turquoise	0.99645
TRINITY_DN38540_c0_g1_i1_orf1	turquoise	0.9198
TRINITY_DN2735_c0_g1_i4_orf1	yellow	0.68212
TRINITY_DN38720_c0_g1_i3_orf1	turquoise	0.57616
TRINITY_DN5275_c0_g1_i1_orf1	turquoise	0.88544
TRINITY_DN493_c0_g1_i4_orf1	turquoise	0.87654
TRINITY_DN5453_c0_g1_i2_orf1	brown	0.70857
TRINITY_DN2894_c0_g1_i2_orf1	turquoise	0.99346
TRINITY_DN2043_c0_g1_i3_orf1	blue	0.92667
TRINITY_DN1272_c1_g1_i4_orf1	yellow	0.40383
TRINITY_DN8964_c0_g1_i4_orf1	yellow	0.83554
TRINITY_DN6642_c0_g2_i1_orf1	turquoise	0.77586
TRINITY_DN29604_c0_g2_i2_orf1	blue	0.99568
TRINITY_DN33953_c0_g1_i4_orf1	brown	0.79898
TRINITY_DN843_c0_g1_i5_orf1	blue	0.7833
TRINITY_DN542_c0_g1_i4_orf1	turquoise	0.97792
TRINITY_DN17312_c0_g1_i1_orf1	turquoise	0.90427
TRINITY_DN1091_c0_g1_i1_orf1	blue	0.81496
TRINITY_DN27398_c0_g1_i3_orf1	turquoise	0.47061
TRINITY_DN18164_c0_g1_i7_orf1	red	0.90335
TRINITY_DN115_c0_g1_i6_orf1	turquoise	0.94288
TRINITY_DN18782_c0_g1_i4_orf1	brown	0.73281
TRINITY_DN129226_c0_g4_i1_orf1	turquoise	0.62313
TRINITY_DN11986_c0_g1_i1_orf1	yellow	0.68753
TRINITY_DN20369_c0_g1_i2_orf1	turquoise	0.98322
TRINITY_DN6147_c0_g1_i2_orf1	turquoise	0.95949
TRINITY_DN30154_c0_g1_i1_orf1	blue	0.98249
TRINITY_DN28311_c0_g1_i2_orf1	yellow	0.68837
TRINITY_DN6153_c0_g1_i6_orf1	turquoise	0.5119
TRINITY_DN1012_c0_g1_i2_orf1	black	0.85544
TRINITY_DN778_c0_g1_i1_orf1	blue	0.92583
TRINITY_DN6680_c0_g1_i1_orf1	green	0.7633

TRINITY_DN198_c0_g1_i2_orf1	red	0.65113
TRINITY_DN38498_c0_g3_i1_orf1	turquoise	0.79852
TRINITY_DN42759_c0_g2_i1_orf1	turquoise	0.97129
TRINITY_DN962_c5_g1_i1_orf1	blue	0.54905
TRINITY_DN27745_c0_g1_i6_orf1	turquoise	0.50348
TRINITY_DN37366_c0_g1_i7_orf1	turquoise	0.80694
TRINITY_DN6656_c0_g1_i1_orf1	red	0.90298
TRINITY_DN7047_c0_g1_i1_orf1	turquoise	0.99043
TRINITY_DN619_c0_g1_i1_orf1	blue	0.92359
TRINITY_DN98814_c0_g1_i2_orf1	red	0.88251
TRINITY_DN21533_c0_g1_i7_orf1	black	0.84842
TRINITY_DN5406_c0_g2_i1_orf1	black	0.9648
TRINITY_DN7674_c0_g1_i2_orf1	turquoise	0.71947
TRINITY_DN268_c1_g1_i7_orf1	blue	0.98837
TRINITY_DN213_c0_g1_i5_orf1	black	0.70688
TRINITY_DN32687_c0_g1_i2_orf1	turquoise	0.45881
TRINITY_DN231_c1_g2_i1_orf1	grey	0.18987
TRINITY_DN8306_c0_g1_i4_orf1	blue	0.84848
TRINITY_DN6580_c0_g1_i4_orf1	brown	0.90648
TRINITY_DN14460_c0_g1_i6_orf1	green	0.92828
TRINITY_DN11383_c0_g2_i4_orf1	brown	0.95594
TRINITY_DN11245_c0_g1_i2_orf1	yellow	0.65026
TRINITY_DN23167_c0_g1_i4_orf1	turquoise	0.81619
TRINITY_DN2043_c0_g1_i11_orf1	blue	0.85707
TRINITY_DN14524_c0_g1_i1_orf1	green	0.51348
TRINITY_DN16123_c0_g1_i1_orf1	grey	0.73002
TRINITY_DN9464_c0_g1_i1_orf1	turquoise	0.99235
TRINITY_DN1641_c0_g1_i6_orf1	turquoise	0.7521
TRINITY_DN65518_c0_g1_i1_orf1	brown	0.95757
TRINITY_DN1425_c0_g1_i4_orf1	red	0.91743
TRINITY_DN22941_c0_g1_i1_orf1	turquoise	0.86205
TRINITY_DN4836_c0_g1_i4_orf1	turquoise	0.84468
TRINITY_DN29956_c1_g1_i1_orf1	green	0.74456
TRINITY_DN1665_c1_g1_i2_orf1	turquoise	0.86754
TRINITY_DN5538_c0_g1_i1_orf1	green	0.79084
TRINITY_DN29017_c0_g1_i4_orf1	green	0.90574
TRINITY_DN10379_c0_g1_i3_orf1	turquoise	0.90808
TRINITY_DN13174_c0_g1_i4_orf1	turquoise	0.96436
TRINITY_DN9575_c0_g1_i1_orf1	turquoise	0.75834
TRINITY_DN220_c0_g1_i3_orf1	turquoise	0.84772
TRINITY_DN747_c0_g1_i1_orf1	turquoise	0.74813
TRINITY_DN33249_c0_g1_i1_orf1	turquoise	0.97094
TRINITY_DN6693_c0_g1_i1_orf1	turquoise	0.99087
TRINITY_DN4565_c0_g2_i1_orf1	black	0.91607
TRINITY_DN4245_c0_g2_i1_orf1	brown	0.96634
TRINITY_DN383_c0_g1_i1_orf1	turquoise	0.6048
TRINITY_DN21451_c0_g1_i3_orf1	turquoise	0.78155
TRINITY_DN11133_c0_g1_i5_orf1	brown	0.55477
TRINITY_DN19651_c0_g1_i1_orf1	red	0.86179
TRINITY_DN31967_c0_g1_i5_orf1	red	0.86011
TRINITY_DN2497_c0_g1_i1_orf1	turquoise	0.61208
TRINITY_DN100885_c0_g2_i1_orfp1	turquoise	0.85525
TRINITY_DN7267_c1_g1_i4_orf1	turquoise	0.87858
TRINITY_DN125427_c0_g1_i1_orf1	blue	0.96323
TRINITY_DN18196_c0_g1_i4_orf1	brown	0.8972
TRINITY_DN1012_c0_g2_i1_orf1	brown	0.70128
TRINITY_DN44877_c0_g1_i2_orf1	turquoise	0.75313
TRINITY_DN9544_c0_g1_i1_orf1	black	0.88236

TRINITY_DN7464_c0_g1_i14_orf1	turquoise	0.913
TRINITY_DN8754_c0_g1_i2_orf1	turquoise	0.98375
TRINITY_DN29448_c0_g1_i1_orf1	turquoise	0.95649
TRINITY_DN4076_c1_g2_i2_orf1	green	0.96184
TRINITY_DN4635_c0_g1_i4_orf1	blue	0.76074
TRINITY_DN10106_c0_g2_i1_orf1	turquoise	0.84332
TRINITY_DN3975_c0_g1_i7_orf1	brown	0.54783
TRINITY_DN110402_c0_g2_i1_orf1	blue	0.91068
TRINITY_DN1266_c6_g1_i1_orf1	turquoise	0.85964
TRINITY_DN63152_c0_g1_i7_orf1	brown	0.90792
TRINITY_DN21331_c0_g1_i6_orf1	brown	0.74883
TRINITY_DN86127_c1_g1_i2_orfp1	pink	0.9188
TRINITY_DN15545_c0_g1_i1_orf1	yellow	0.83373
TRINITY_DN858_c0_g1_i3_orf1	black	0.86721
TRINITY_DN4869_c0_g1_i10_orf1	turquoise	0.99062
TRINITY_DN1947_c0_g1_i6_orf1	turquoise	0.95709
TRINITY_DN8780_c0_g1_i3_orf1	green	0.92672
TRINITY_DN33_c0_g1_i8_orf1	turquoise	0.59662
TRINITY_DN54554_c0_g1_i1_orf1	green	0.71727
TRINITY_DN3953_c0_g1_i2_orf1	red	0.8728
TRINITY_DN41506_c0_g1_i4_orf1	turquoise	0.39385
TRINITY_DN23824_c0_g1_i1_orf1	turquoise	0.9459
TRINITY_DN14398_c0_g1_i4_orf1	yellow	0.9058
TRINITY_DN23746_c0_g1_i2_orf1	green	0.86914
TRINITY_DN391_c0_g1_i4_orf1	blue	0.9537
TRINITY_DN31645_c0_g1_i3_orf1	yellow	0.97701
TRINITY_DN3618_c0_g1_i4_orf1	turquoise	0.58372
TRINITY_DN26663_c0_g1_i4_orf1	yellow	0.81444
TRINITY_DN31118_c0_g2_i1_orf1	yellow	0.91009
TRINITY_DN2688_c0_g2_i1_orf1	yellow	0.90497
TRINITY_DN8971_c1_g1_i4_orf1	turquoise	0.82507
TRINITY_DN3838_c0_g1_i8_orf1	red	0.85959
TRINITY_DN14391_c1_g1_i2_orf1	blue	0.55754
TRINITY_DN12769_c0_g1_i5_orf1	red	0.84505
TRINITY_DN28428_c0_g1_i2_orf1	red	0.95122
TRINITY_DN12690_c0_g1_i1_orf1	yellow	0.93786
TRINITY_DN27111_c0_g1_i1_orf1	blue	0.54285
TRINITY_DN7539_c0_g1_i2_orf1	blue	0.98413
TRINITY_DN106479_c1_g1_i1_orf1	black	0.79988
TRINITY_DN5149_c0_g1_i1_orf1	yellow	0.9112
TRINITY_DN4592_c0_g1_i1_orf1	blue	0.97559
TRINITY_DN10403_c0_g1_i3_orf1	blue	0.69261
TRINITY_DN12777_c0_g1_i5_orf1	red	0.8103
TRINITY_DN45949_c0_g1_i1_orf1	yellow	0.90611
TRINITY_DN7808_c0_g1_i1_orf1	turquoise	0.83186
TRINITY_DN52864_c0_g1_i1_orf1	blue	0.83499
TRINITY_DN28938_c0_g1_i1_orf1	turquoise	0.69483
TRINITY_DN32161_c0_g1_i1_orf1	turquoise	0.77223
TRINITY_DN334_c0_g1_i2_orf1	turquoise	0.98451
TRINITY_DN122867_c1_g1_i1_orf1	red	0.85619
TRINITY_DN1108_c3_g1_i1_orfp1	brown	0.80174
TRINITY_DN38075_c0_g1_i1_orf1	turquoise	0.97159
TRINITY_DN23740_c0_g1_i3_orf1	blue	0.80024
TRINITY_DN41922_c0_g3_i1_orf1	turquoise	0.95581
TRINITY_DN8155_c0_g1_i1_orf1	yellow	0.63544
TRINITY_DN4977_c0_g1_i2_orf1	turquoise	0.98304
TRINITY_DN6108_c0_g1_i5_orf1	green	0.89072
TRINITY_DN33183_c0_g1_i4_orf1	turquoise	0.962

TRINITY_DN4731_c0_g2_i1_orf1	turquoise	0.80662
TRINITY_DN277_c0_g1_i5_orf1	turquoise	0.60032
TRINITY_DN10332_c0_g1_i2_orfp1	turquoise	0.9676
TRINITY_DN214_c0_g1_i4_orf1	yellow	0.80248
TRINITY_DN19110_c0_g1_i2_orf1	blue	0.88788
TRINITY_DN1408_c0_g1_i10_orf1	green	0.83213
TRINITY_DN48610_c0_g1_i2_orf1	yellow	0.98069
TRINITY_DN2258_c0_g2_i1_orf1	turquoise	0.95487
TRINITY_DN30224_c0_g1_i1_orf1	turquoise	0.89286
TRINITY_DN24699_c0_g1_i3_orf1	turquoise	0.82669
TRINITY_DN501_c0_g1_i5_orf1	blue	0.95932
TRINITY_DN9871_c0_g1_i11_orf1	yellow	0.97082
TRINITY_DN1604_c0_g1_i4_orf1	blue	0.81662
TRINITY_DN35725_c0_g1_i1_orf1	turquoise	0.96421
TRINITY_DN37699_c0_g1_i3_orfp1	turquoise	0.94072
TRINITY_DN20582_c0_g1_i1_orf1	turquoise	0.9918
TRINITY_DN7512_c0_g1_i1_orf1	turquoise	0.95043
TRINITY_DN21719_c0_g1_i2_orf1	blue	0.96643
TRINITY_DN49147_c0_g2_i1_orf1	blue	0.9682
TRINITY_DN96170_c0_g1_i1_orf1	turquoise	0.97449
TRINITY_DN12757_c0_g1_i1_orf1	red	0.87277
TRINITY_DN2521_c1_g1_i2_orf1	brown	0.48046
TRINITY_DN63561_c1_g1_i2_orf1	turquoise	0.87989
TRINITY_DN41_c0_g1_i5_orf1	blue	0.94245
TRINITY_DN29288_c0_g1_i1_orf1	yellow	0.34147
TRINITY_DN14209_c0_g1_i1_orf1	turquoise	0.50364
TRINITY_DN127151_c0_g1_i1_orf1	turquoise	0.93903
TRINITY_DN937_c0_g1_i2_orf1	turquoise	0.81508
TRINITY_DN43228_c0_g1_i1_orf1	grey	-0.19322
TRINITY_DN28654_c0_g1_i2_orf1	green	0.76166
TRINITY_DN3227_c0_g1_i5_orf1	blue	0.49171
TRINITY_DN38644_c0_g1_i1_orf1	red	0.85987
TRINITY_DN3474_c1_g2_i7_orf1	turquoise	0.92484
TRINITY_DN95530_c0_g1_i1_orf1	brown	0.54554
TRINITY_DN1659_c0_g1_i3_orf1	green	0.86235
TRINITY_DN19493_c0_g1_i5_orf1	red	0.91282
TRINITY_DN11069_c0_g2_i1_orf1	turquoise	0.99352
TRINITY_DN47784_c0_g2_i1_orfp1	brown	0.83745
TRINITY_DN8367_c0_g2_i2_orf1	black	0.89914
TRINITY_DN18279_c0_g1_i1_orf1	brown	0.72314
TRINITY_DN62707_c0_g1_i1_orf1	brown	0.78774
TRINITY_DN10747_c0_g1_i5_orf1	blue	0.9858
TRINITY_DN19995_c0_g1_i2_orf1	turquoise	0.44573
TRINITY_DN2591_c0_g1_i4_orf1	red	0.91685
TRINITY_DN5105_c0_g1_i10_orf1	turquoise	0.60298
TRINITY_DN5834_c0_g1_i2_orf1	turquoise	0.72278
TRINITY_DN6423_c0_g1_i6_orf1	blue	0.87083
TRINITY_DN4439_c0_g1_i2_orf1	red	0.91289
TRINITY_DN3893_c0_g2_i3_orf1	turquoise	0.8015
TRINITY_DN4247_c0_g1_i4_orf1	grey	0.23428
TRINITY_DN2070_c1_g1_i1_orf1	turquoise	0.97435
TRINITY_DN139326_c0_g1_i1_orf1	turquoise	0.70665
TRINITY_DN4567_c0_g3_i1_orf1	brown	0.66808
TRINITY_DN5132_c0_g1_i4_orf1	green	0.93216
TRINITY_DN18300_c0_g1_i17_orf1	turquoise	0.94061
TRINITY_DN24317_c0_g1_i7_orf1	turquoise	0.98276
TRINITY_DN374_c0_g1_i4_orf1	turquoise	0.83122
TRINITY_DN6308_c0_g1_i6_orf1	turquoise	0.98456

TRINITY_DN82628_c0_g1_i2_orf1	yellow	0.9626
TRINITY_DN931_c0_g1_i4_orf1	red	0.70225
TRINITY_DN2675_c0_g1_i1_orf1	grey	0.17125
TRINITY_DN57636_c0_g1_i4_orf1	yellow	0.79667
TRINITY_DN9931_c0_g1_i1_orf1	turquoise	0.93986
TRINITY_DN81181_c0_g1_i6_orfp1	yellow	0.52724
TRINITY_DN22513_c0_g1_i4_orf1	yellow	0.83188
TRINITY_DN34816_c0_g1_i4_orf1	green	0.72058
TRINITY_DN7919_c0_g1_i4_orf1	turquoise	0.5024
TRINITY_DN4141_c0_g1_i9_orf1	turquoise	0.9825
TRINITY_DN82008_c0_g1_i1_orf1	turquoise	0.98069
TRINITY_DN6696_c0_g1_i4_orf1	turquoise	0.8137
TRINITY_DN6325_c0_g1_i8_orf1	yellow	0.83697
TRINITY_DN22046_c1_g1_i5_orf1	brown	0.82699
TRINITY_DN3407_c0_g1_i9_orf1	blue	0.63025
TRINITY_DN43420_c0_g2_i1_orf1	turquoise	0.99386
TRINITY_DN16400_c0_g2_i1_orf1	yellow	0.91391
TRINITY_DN67026_c0_g1_i6_orf1	green	0.58137
TRINITY_DN210_c0_g1_i9_orf1	red	0.64281
TRINITY_DN3159_c0_g1_i4_orf1	turquoise	0.98995
TRINITY_DN23926_c0_g1_i4_orf1	turquoise	0.83611
TRINITY_DN13216_c0_g1_i5_orf1	red	0.91777
TRINITY_DN15339_c0_g1_i6_orf1	turquoise	0.84388
TRINITY_DN98538_c0_g1_i1_orf1	turquoise	0.98028
TRINITY_DN41645_c0_g1_i1_orf1	yellow	0.66472
TRINITY_DN17574_c0_g1_i2_orf1	green	0.90282
TRINITY_DN34159_c0_g2_i1_orf1	turquoise	0.75046
TRINITY_DN10371_c0_g1_i2_orf1	black	0.71209
TRINITY_DN56270_c0_g1_i1_orf1	turquoise	0.48177
TRINITY_DN3835_c0_g1_i4_orf1	turquoise	0.71677
TRINITY_DN67193_c0_g1_i1_orf1	green	0.95631
TRINITY_DN1475_c0_g1_i6_orf1	yellow	0.76419
TRINITY_DN33967_c0_g1_i1_orf1	turquoise	0.87574
TRINITY_DN125_c0_g1_i2_orf1	green	0.82367
TRINITY_DN16939_c0_g1_i4_orf1	turquoise	0.99403
TRINITY_DN81084_c0_g3_i1_orf1	turquoise	0.61334
TRINITY_DN95850_c0_g4_i3_orf1	brown	0.80851
TRINITY_DN97042_c0_g1_i6_orf1	blue	0.81846
TRINITY_DN27456_c0_g2_i1_orf1	blue	0.75674
TRINITY_DN413_c0_g1_i11_orf1	turquoise	0.95398
TRINITY_DN1363_c0_g1_i11_orf1	brown	0.968
TRINITY_DN1622_c0_g1_i6_orf1	brown	0.84296
TRINITY_DN14477_c0_g1_i12_orf1	red	0.84222
TRINITY_DN20130_c0_g1_i1_orf1	turquoise	0.94323
TRINITY_DN1427_c0_g1_i9_orf1	turquoise	0.8905
TRINITY_DN5432_c1_g1_i3_orf1	yellow	0.69345
TRINITY_DN74086_c0_g1_i1_orf1	blue	0.96419
TRINITY_DN2973_c1_g1_i9_orf1	turquoise	0.957
TRINITY_DN11204_c0_g1_i3_orf1	blue	0.6697
TRINITY_DN73224_c0_g4_i2_orf1	red	0.95193
TRINITY_DN77005_c0_g3_i1_orf1	red	0.80929
TRINITY_DN24325_c0_g1_i12_orf1	turquoise	0.91677
TRINITY_DN7213_c0_g1_i2_orf1	turquoise	0.99191
TRINITY_DN7966_c0_g1_i4_orf1	turquoise	0.99469
TRINITY_DN51813_c0_g1_i1_orf1	green	0.937
TRINITY_DN8079_c0_g1_i2_orf1	turquoise	0.62306
TRINITY_DN5298_c0_g1_i3_orf1	turquoise	0.54552
TRINITY_DN8652_c0_g1_i8_orf1	black	0.84334

TRINITY_DN701_c0_g1_i1_orf1	brown	0.93367
TRINITY_DN7037_c0_g1_i4_orf1	turquoise	0.81366
TRINITY_DN44777_c0_g1_i2_orf1	blue	0.64474
TRINITY_DN2193_c0_g1_i7_orf1	yellow	0.93746
TRINITY_DN97138_c0_g1_i2_orf1	blue	0.98725
TRINITY_DN24163_c0_g1_i1_orf1	turquoise	0.94713
TRINITY_DN7335_c0_g1_i1_orf1	turquoise	0.8125
TRINITY_DN6310_c0_g2_i10_orf1	green	0.81219
TRINITY_DN2977_c0_g1_i3_orf1	turquoise	0.81986
TRINITY_DN9765_c0_g1_i6_orf1	red	0.86523
TRINITY_DN117_c0_g1_i4_orf1	turquoise	0.9873
TRINITY_DN5508_c0_g1_i1_orf1	blue	0.80965
TRINITY_DN25975_c0_g3_i2_orf1	turquoise	0.98451
TRINITY_DN6330_c0_g1_i1_orfp1	brown	0.97432
TRINITY_DN21435_c0_g1_i2_orf1	brown	0.88011
TRINITY_DN4820_c0_g2_i2_orf1	blue	0.79615
TRINITY_DN76216_c0_g2_i3_orf1	blue	0.83765
TRINITY_DN14904_c1_g2_i2_orf1	black	0.77879
TRINITY_DN6711_c0_g1_i1_orf1	turquoise	0.97943
TRINITY_DN1172_c0_g1_i1_orf1	brown	0.94476
TRINITY_DN3434_c0_g1_i1_orf1	turquoise	0.8936
TRINITY_DN3321_c0_g1_i3_orf1	turquoise	0.90106
TRINITY_DN3010_c0_g1_i4_orf1	yellow	0.91571
TRINITY_DN9560_c0_g1_i5_orf1	yellow	0.9445
TRINITY_DN40945_c0_g1_i1_orf1	black	0.69469
TRINITY_DN20966_c0_g1_i6_orf1	turquoise	0.83002
TRINITY_DN14597_c0_g1_i5_orf1	turquoise	0.80219
TRINITY_DN2611_c0_g1_i10_orf1	turquoise	0.68438
TRINITY_DN40015_c0_g1_i2_orf1	turquoise	0.92983
TRINITY_DN5028_c0_g1_i11_orf1	blue	0.86149
TRINITY_DN12671_c0_g1_i4_orf1	blue	0.99238
TRINITY_DN364_c2_g1_i2_orf1	black	0.89736
TRINITY_DN27491_c0_g1_i1_orf1	yellow	0.90147
TRINITY_DN4309_c0_g1_i1_orf1	turquoise	0.946
TRINITY_DN5009_c0_g1_i2_orf1	turquoise	0.88924
TRINITY_DN3058_c0_g1_i1_orf1	yellow	0.57586
TRINITY_DN2874_c0_g1_i4_orf1	turquoise	0.61544
TRINITY_DN5080_c0_g1_i5_orf1	green	0.95749
TRINITY_DN34726_c0_g2_i1_orf1	turquoise	0.90724
TRINITY_DN13651_c0_g1_i2_orf1	turquoise	0.99343
TRINITY_DN2425_c0_g1_i3_orf1	blue	0.87729
TRINITY_DN3840_c0_g1_i1_orf1	turquoise	0.68128
TRINITY_DN57462_c0_g1_i1_orf1	turquoise	0.80002
TRINITY_DN51480_c0_g1_i1_orf1	brown	0.90985
TRINITY_DN57202_c0_g1_i1_orf1	turquoise	0.98541
TRINITY_DN19122_c0_g1_i7_orf1	turquoise	0.95833
TRINITY_DN35865_c0_g1_i1_orf1	red	0.57815
TRINITY_DN1666_c0_g1_i2_orf1	turquoise	0.83839
TRINITY_DN11188_c0_g1_i2_orf1	turquoise	0.57742
TRINITY_DN4731_c0_g1_i1_orf1	turquoise	0.98585
TRINITY_DN2499_c0_g1_i4_orf1	black	0.27605
TRINITY_DN21609_c0_g1_i1_orf1	brown	0.78533
TRINITY_DN16824_c0_g1_i7_orf1	brown	0.83861
TRINITY_DN18620_c0_g1_i5_orf1	blue	0.79538
TRINITY_DN334_c0_g1_i3_orf1	turquoise	0.91494
TRINITY_DN5382_c0_g2_i1_orf1	turquoise	0.70275
TRINITY_DN3492_c0_g1_i1_orf1	yellow	0.91734
TRINITY_DN1393_c0_g1_i2_orf1	blue	0.8221

TRINITY_DN6916_c0_g1_i4_orf1	turquoise	0.77259
TRINITY_DN84669_c0_g1_i1_orf1	turquoise	0.40343
TRINITY_DN19303_c0_g1_i5_orf1	red	0.72429
TRINITY_DN47914_c0_g2_i1_orf1	red	0.95228
TRINITY_DN19155_c0_g1_i1_orf1	turquoise	0.83356
TRINITY_DN1406_c0_g2_i2_orf1	yellow	0.66489
TRINITY_DN22572_c0_g1_i1_orf1	turquoise	0.86718
TRINITY_DN41952_c0_g1_i4_orf1	yellow	0.78492
TRINITY_DN17651_c0_g1_i2_orf1	turquoise	0.86652
TRINITY_DN10131_c0_g1_i7_orf1	red	0.95965
TRINITY_DN12024_c0_g2_i2_orf1	green	0.86714
TRINITY_DN40197_c0_g1_i1_orf1	turquoise	0.84351
TRINITY_DN4886_c0_g1_i6_orf1	blue	0.9354
TRINITY_DN279_c0_g1_i10_orf1	red	0.97664
TRINITY_DN34534_c0_g2_i1_orf1	blue	0.66412
TRINITY_DN140613_c0_g1_i1_orf1	yellow	0.59569
TRINITY_DN11172_c0_g1_i4_orf1	yellow	0.57607
TRINITY_DN445_c0_g1_i2_orf1	blue	0.85415
TRINITY_DN1914_c0_g1_i4_orf1	pink	0.88495
TRINITY_DN2432_c0_g1_i1_orf1	blue	0.65372
TRINITY_DN4612_c0_g1_i1_orf1	turquoise	0.96889
TRINITY_DN6933_c1_g1_i1_orf1	turquoise	0.98134
TRINITY_DN7112_c0_g1_i1_orf1	turquoise	0.81943
TRINITY_DN14572_c0_g1_i1_orf1	red	0.87775
TRINITY_DN4356_c0_g1_i6_orf1	turquoise	0.67491
TRINITY_DN47114_c0_g1_i5_orf1	turquoise	0.9972
TRINITY_DN47677_c0_g1_i1_orf1	blue	0.61562
TRINITY_DN27021_c0_g1_i1_orf1	brown	0.56498
TRINITY_DN76307_c0_g1_i1_orf1	brown	0.71226
TRINITY_DN4070_c0_g1_i4_orf1	red	0.99044
TRINITY_DN97680_c0_g1_i1_orf1	turquoise	0.93864
TRINITY_DN25997_c1_g2_i4_orf1	turquoise	0.72532
TRINITY_DN10396_c0_g1_i1_orf1	green	0.83667
TRINITY_DN23167_c0_g2_i1_orf1	blue	0.89932
TRINITY_DN27721_c1_g1_i2_orf1	turquoise	0.97372
TRINITY_DN517_c0_g1_i5_orf1	turquoise	0.97973
TRINITY_DN8095_c0_g1_i3_orf1	yellow	0.78161
TRINITY_DN11894_c1_g1_i5_orf1	turquoise	0.94525
TRINITY_DN2967_c0_g1_i4_orf1	turquoise	0.91144
TRINITY_DN31390_c0_g1_i2_orf1	yellow	0.73711
TRINITY_DN57765_c0_g1_i1_orf1	turquoise	0.83063
TRINITY_DN15411_c0_g1_i4_orf1	blue	0.92405
TRINITY_DN64788_c0_g1_i1_orf1	brown	0.54048
TRINITY_DN364_c0_g1_i2_orf1	black	0.96803
TRINITY_DN12555_c0_g1_i1_orf1	blue	0.83934
TRINITY_DN5954_c0_g1_i2_orf1	blue	0.87395
TRINITY_DN2252_c0_g1_i4_orfp1	turquoise	0.71561
TRINITY_DN2170_c0_g2_i1_orf1	green	0.89091
TRINITY_DN110523_c0_g2_i1_orf1	blue	0.82592
TRINITY_DN33_c0_g1_i14_orf1	brown	0.93404
TRINITY_DN42964_c0_g1_i1_orf1	blue	0.78027
TRINITY_DN24266_c0_g2_i2_orf1	red	0.86401
TRINITY_DN7241_c0_g2_i2_orf1	turquoise	0.91033
TRINITY_DN610_c0_g1_i1_orf1	brown	0.88222
TRINITY_DN18128_c0_g1_i4_orf1	green	0.98417
TRINITY_DN29009_c0_g2_i2_orf1	brown	0.90042
TRINITY_DN276_c0_g1_i1_orf1	green	0.86691
TRINITY_DN848_c0_g1_i1_orf1	turquoise	0.90885

TRINITY_DN8407_c0_g1_i2_orf1	green	0.80818
TRINITY_DN5910_c1_g1_i6_orf1	blue	0.62502
TRINITY_DN2571_c0_g2_i1_orf1	turquoise	0.95515
TRINITY_DN100327_c0_g1_i1_orf1	green	0.90777
TRINITY_DN1593_c0_g1_i1_orf1	brown	0.88693
TRINITY_DN26649_c0_g1_i2_orf1	turquoise	0.98438
TRINITY_DN83327_c0_g1_i1_orf1	turquoise	0.95007
TRINITY_DN2668_c0_g1_i6_orf1	turquoise	0.84492
TRINITY_DN2958_c0_g1_i2_orf1	yellow	0.83116
TRINITY_DN69049_c0_g1_i2_orf1	turquoise	0.99447
TRINITY_DN14601_c0_g1_i2_orf1	turquoise	0.94412
TRINITY_DN9770_c0_g1_i1_orf1	turquoise	0.97313
TRINITY_DN45477_c0_g1_i1_orf1	turquoise	0.94708
TRINITY_DN4237_c1_g1_i5_orf1	turquoise	0.97747
TRINITY_DN37538_c0_g4_i1_orf1	turquoise	0.96116
TRINITY_DN6671_c0_g1_i6_orf1	turquoise	0.95867
TRINITY_DN3784_c0_g1_i1_orf1	turquoise	0.98838
TRINITY_DN993_c0_g1_i7_orf1	blue	0.82139
TRINITY_DN1044_c0_g1_i2_orf1	turquoise	0.99039
TRINITY_DN2478_c0_g1_i1_orf1	turquoise	0.76885
TRINITY_DN26010_c0_g1_i2_orf1	turquoise	0.98751
TRINITY_DN8030_c0_g1_i2_orf1	brown	0.93303
TRINITY_DN104297_c0_g1_i1_orf1	green	0.61115
TRINITY_DN70485_c0_g1_i2_orf1	blue	0.85698
TRINITY_DN102051_c0_g1_i1_orf1	yellow	0.87085
TRINITY_DN6203_c0_g1_i1_orfp1	green	0.38116
TRINITY_DN24723_c2_g1_i1_orf1	brown	0.95016
TRINITY_DN22664_c0_g1_i1_orf1	turquoise	0.91488
TRINITY_DN42364_c0_g1_i4_orf1	turquoise	0.96519
TRINITY_DN9555_c0_g1_i1_orf1	turquoise	0.85276
TRINITY_DN3464_c0_g1_i1_orf1	blue	0.865
TRINITY_DN3887_c0_g1_i1_orf1	blue	0.87848
TRINITY_DN21218_c0_g2_i3_orf1	turquoise	0.88171
TRINITY_DN3918_c0_g1_i1_orf1	grey	0.5374
TRINITY_DN3014_c0_g1_i4_orf1	turquoise	0.92011
TRINITY_DN11569_c0_g1_i1_orf1	green	0.59512
TRINITY_DN18273_c0_g1_i4_orf1	green	0.88645
TRINITY_DN12514_c0_g2_i1_orf1	green	0.89376
TRINITY_DN9101_c0_g2_i1_orf1	turquoise	0.95235
TRINITY_DN13221_c0_g1_i3_orf1	blue	0.81887
TRINITY_DN61135_c0_g1_i1_orf1	green	0.864
TRINITY_DN6365_c0_g1_i4_orf1	turquoise	0.67507
TRINITY_DN3488_c0_g1_i2_orf1	turquoise	0.90489
TRINITY_DN24528_c0_g1_i1_orf1	brown	0.5635
TRINITY_DN20499_c0_g3_i1_orf1	turquoise	0.96245
TRINITY_DN1965_c0_g1_i7_orf1	turquoise	0.97715
TRINITY_DN19659_c1_g1_i1_orf1	yellow	0.68199
TRINITY_DN37538_c0_g3_i1_orf1	turquoise	0.94229
TRINITY_DN1402_c1_g1_i6_orf1	grey	0.67905
TRINITY_DN10195_c0_g1_i8_orf1	green	0.88293
TRINITY_DN3231_c0_g1_i12_orf1	yellow	0.91671
TRINITY_DN15753_c0_g1_i1_orf1	red	0.84442
TRINITY_DN6221_c0_g1_i5_orf1	turquoise	0.97314
TRINITY_DN9019_c0_g1_i5_orf1	green	0.69649
TRINITY_DN350_c0_g1_i10_orf1	yellow	0.83066
TRINITY_DN2265_c0_g1_i5_orf1	turquoise	0.78322
TRINITY_DN1532_c0_g1_i6_orf1	green	0.83649
TRINITY_DN89083_c0_g1_i1_orf1	green	0.9447

TRINITY_DN5349_c0_g1_i1_orf1	turquoise	0.97509
TRINITY_DN892_c7_g1_i2_orf1	brown	0.76653
TRINITY_DN24789_c0_g1_i9_orfp1	yellow	0.77312
TRINITY_DN31225_c0_g1_i1_orf1	yellow	0.87853
TRINITY_DN7226_c0_g1_i5_orf1	brown	0.95644
TRINITY_DN12003_c0_g2_i1_orf1	brown	0.86343
TRINITY_DN2848_c0_g1_i2_orf1	green	0.97699
TRINITY_DN143497_c0_g1_i1_orf1	blue	0.93837
TRINITY_DN11231_c1_g1_i1_orfp1	brown	0.92726
TRINITY_DN17208_c0_g1_i2_orf1	turquoise	0.8622
TRINITY_DN1175_c1_g1_i2_orf1	brown	0.9144
TRINITY_DN11464_c0_g1_i3_orf1	red	0.91039
TRINITY_DN2606_c0_g1_i5_orf1	green	0.9133
TRINITY_DN130778_c0_g1_i1_orf1	turquoise	0.54253
TRINITY_DN437_c0_g1_i1_orf1	turquoise	0.6736
TRINITY_DN57749_c0_g1_i4_orf1	yellow	0.36031
TRINITY_DN64126_c0_g1_i1_orf1	brown	0.92755
TRINITY_DN96170_c0_g2_i1_orf1	turquoise	0.98521
TRINITY_DN39673_c0_g1_i1_orf1	turquoise	0.96354
TRINITY_DN6908_c0_g1_i1_orf1	green	0.79597
TRINITY_DN6588_c0_g1_i4_orf1	turquoise	0.52997
TRINITY_DN41842_c0_g1_i2_orf1	turquoise	0.99044
TRINITY_DN17935_c0_g1_i1_orf1	red	0.86375
TRINITY_DN1054_c0_g1_i8_orf1	black	0.91134
TRINITY_DN4659_c0_g1_i2_orf1	red	0.91405
TRINITY_DN8833_c0_g1_i1_orf1	turquoise	0.98806
TRINITY_DN1104_c0_g1_i1_orfp1	yellow	0.82771
TRINITY_DN45220_c0_g1_i1_orf1	blue	0.83793
TRINITY_DN12534_c0_g1_i4_orf1	brown	0.98594
TRINITY_DN23941_c0_g1_i5_orf1	yellow	0.93938
TRINITY_DN3797_c0_g2_i3_orf1	turquoise	0.39923
TRINITY_DN4385_c0_g2_i1_orf1	turquoise	0.75584
TRINITY_DN1447_c0_g1_i5_orf1	turquoise	0.8977
TRINITY_DN3588_c0_g1_i4_orf1	yellow	0.28403
TRINITY_DN1569_c0_g1_i6_orf1	green	0.7195
TRINITY_DN51995_c0_g3_i1_orf1	brown	0.64365
TRINITY_DN37393_c0_g1_i1_orf1	turquoise	0.96605
TRINITY_DN4476_c0_g1_i5_orf1	turquoise	0.97476
TRINITY_DN13660_c0_g1_i1_orf1	brown	0.94937
TRINITY_DN913_c0_g1_i6_orf1	green	0.93124
TRINITY_DN10398_c0_g1_i12_orf1	pink	0.64021
TRINITY_DN72017_c0_g1_i1_orf1	brown	0.41678
TRINITY_DN49508_c0_g2_i8_orf1	turquoise	0.96583
TRINITY_DN32601_c0_g1_i2_orf1	red	0.9709
TRINITY_DN21715_c0_g1_i1_orf1	brown	0.8553
TRINITY_DN2927_c0_g1_i6_orf1	turquoise	0.98611
TRINITY_DN8958_c0_g1_i1_orf1	turquoise	0.77673
TRINITY_DN71863_c0_g1_i2_orf1	green	0.99127
TRINITY_DN2471_c0_g1_i3_orf1	grey	0.19179
TRINITY_DN4125_c1_g1_i5_orf1	turquoise	0.97123
TRINITY_DN928_c0_g1_i3_orf1	yellow	0.82664
TRINITY_DN9794_c0_g2_i8_orf1	blue	0.74523
TRINITY_DN136031_c0_g1_i7_orf1	yellow	0.75374
TRINITY_DN10581_c0_g1_i5_orf1	brown	0.89311
TRINITY_DN51342_c0_g1_i7_orf1	brown	0.31617
TRINITY_DN315_c0_g1_i1_orf1	brown	0.69009
TRINITY_DN38106_c0_g1_i6_orf1	black	0.69679
TRINITY_DN2438_c0_g1_i1_orf1	blue	0.77108

TRINITY_DN5525_c0_g1_i4_orf1	turquoise	0.81398
TRINITY_DN1063_c0_g1_i16_orf1	green	0.8281
TRINITY_DN3366_c0_g1_i6_orf1	turquoise	0.98107
TRINITY_DN20878_c0_g4_i2_orf1	turquoise	0.67611
TRINITY_DN640_c0_g1_i2_orf1	turquoise	0.67346
TRINITY_DN23502_c0_g1_i1_orf1	turquoise	0.78981
TRINITY_DN28626_c0_g1_i5_orf1	grey	0.30725
TRINITY_DN46124_c0_g1_i1_orf1	turquoise	0.56268
TRINITY_DN51523_c0_g1_i4_orf1	green	0.58669
TRINITY_DN3924_c0_g1_i5_orf1	turquoise	0.89715
TRINITY_DN78873_c0_g1_i4_orf1	turquoise	0.89947
TRINITY_DN4688_c0_g1_i2_orf1	blue	0.98771
TRINITY_DN7828_c0_g1_i2_orf1	red	0.93844
TRINITY_DN6901_c0_g1_i4_orf1	turquoise	0.62028
TRINITY_DN10512_c0_g1_i1_orf1	yellow	0.93136
TRINITY_DN14372_c0_g2_i1_orf1	turquoise	0.96923
TRINITY_DN3504_c0_g1_i4_orfp1	turquoise	0.90969
TRINITY_DN3428_c0_g1_i1_orf1	turquoise	0.98404
TRINITY_DN2922_c0_g1_i1_orf1	yellow	0.92232
TRINITY_DN7938_c0_g1_i3_orf1	black	0.91255
TRINITY_DN3702_c0_g1_i1_orf1	turquoise	0.73205
TRINITY_DN17215_c0_g1_i4_orf1	turquoise	0.93931
TRINITY_DN17045_c0_g2_i3_orf1	turquoise	0.95088
TRINITY_DN49942_c0_g1_i2_orf1	turquoise	0.5734
TRINITY_DN40126_c0_g2_i1_orf1	brown	0.95792
TRINITY_DN17061_c0_g1_i1_orf1	yellow	0.91715
TRINITY_DN6994_c0_g1_i4_orf1	turquoise	0.795
TRINITY_DN8783_c0_g1_i9_orf1	pink	0.80401
TRINITY_DN1181_c0_g1_i1_orf1	turquoise	0.61543
TRINITY_DN667_c0_g1_i13_orf1	turquoise	0.83782
TRINITY_DN3638_c0_g1_i1_orf1	turquoise	0.93597
TRINITY_DN42542_c0_g1_i1_orf1	turquoise	0.8169
TRINITY_DN22_c0_g1_i3_orf1	grey	-0.18181
TRINITY_DN19043_c0_g3_i2_orf1	blue	0.85541
TRINITY_DN3039_c0_g2_i1_orf1	green	0.87308
TRINITY_DN1968_c0_g1_i3_orf1	yellow	0.45373
TRINITY_DN80560_c0_g1_i1_orf1	turquoise	0.97831
TRINITY_DN48536_c0_g1_i3_orf1	turquoise	0.92748
TRINITY_DN10138_c0_g1_i1_orf1	green	0.95847
TRINITY_DN344_c0_g1_i1_orf1	turquoise	0.97158
TRINITY_DN928_c0_g2_i1_orf1	brown	0.75432
TRINITY_DN23432_c0_g1_i1_orf1	turquoise	0.93214
TRINITY_DN2813_c0_g1_i3_orf1	turquoise	0.30785
TRINITY_DN135846_c0_g1_i1_orf1	grey	0.2808
TRINITY_DN103118_c0_g1_i4_orf1	turquoise	0.49064
TRINITY_DN25136_c0_g1_i1_orf1	turquoise	0.76847
TRINITY_DN23801_c0_g1_i2_orf1	turquoise	0.89622
TRINITY_DN52553_c0_g2_i1_orf1	blue	0.96324
TRINITY_DN28729_c0_g1_i9_orf1	green	0.87357
TRINITY_DN42903_c0_g1_i4_orf1	turquoise	0.84261
TRINITY_DN17172_c0_g1_i5_orf1	brown	0.95281
TRINITY_DN9109_c0_g1_i1_orf1	blue	0.80822
TRINITY_DN2400_c0_g1_i1_orf1	blue	0.98113
TRINITY_DN6231_c0_g1_i6_orf1	turquoise	0.97463
TRINITY_DN61112_c0_g1_i4_orfp1	turquoise	0.98932
TRINITY_DN6668_c0_g1_i4_orf1	turquoise	0.82009
TRINITY_DN1348_c0_g1_i1_orf1	turquoise	0.99787
TRINITY_DN82104_c0_g1_i5_orf1	blue	0.90062

TRINITY_DN1199_c0_g1_i1_orf1	turquoise	0.99027
TRINITY_DN4384_c0_g1_i5_orf1	blue	0.98958
TRINITY_DN3593_c0_g1_i3_orfp1	brown	0.58163
TRINITY_DN6415_c0_g2_i1_orf1	brown	0.94567
TRINITY_DN1697_c0_g1_i1_orf1	turquoise	0.73627
TRINITY_DN14046_c0_g1_i1_orf1	red	0.91151
TRINITY_DN41259_c0_g1_i6_orf1	red	0.97942
TRINITY_DN84357_c0_g1_i1_orf1	turquoise	0.72766
TRINITY_DN31_c0_g1_i3_orfp1	blue	0.96088
TRINITY_DN80328_c0_g1_i5_orf1	green	0.99609
TRINITY_DN3418_c0_g1_i3_orf1	green	0.92208
TRINITY_DN50237_c0_g1_i8_orf1	green	0.91312
TRINITY_DN11746_c0_g2_i1_orf1	turquoise	0.78889
TRINITY_DN28638_c0_g1_i1_orf1	turquoise	0.95307
TRINITY_DN18918_c0_g1_i2_orf1	turquoise	0.58628
TRINITY_DN9790_c0_g1_i4_orf1	turquoise	0.83101
TRINITY_DN63568_c0_g1_i1_orf1	blue	0.88151
TRINITY_DN53115_c0_g1_i1_orf1	turquoise	0.85767
TRINITY_DN1585_c0_g1_i1_orf1	yellow	0.70141
TRINITY_DN96739_c0_g1_i1_orf1	black	0.90469
TRINITY_DN19669_c0_g1_i1_orf1	black	0.5553
TRINITY_DN83150_c0_g1_i1_orf1	green	0.48618
TRINITY_DN2311_c0_g3_i1_orf1	blue	0.9588
TRINITY_DN2748_c0_g1_i6_orf1	turquoise	0.9124
TRINITY_DN2908_c0_g1_i1_orf1	blue	0.94863
TRINITY_DN12671_c0_g1_i6_orf1	blue	0.98574
TRINITY_DN143637_c0_g1_i1_orf1	green	0.82939
TRINITY_DN15244_c0_g1_i5_orf1	turquoise	0.91925
TRINITY_DN1081_c0_g1_i7_orf1	turquoise	0.95406
TRINITY_DN15882_c0_g1_i1_orf1	turquoise	0.9867
TRINITY_DN5956_c1_g1_i5_orf1	turquoise	0.98365
TRINITY_DN1369_c0_g2_i3_orf1	turquoise	0.71227
TRINITY_DN9146_c0_g1_i1_orf1	red	0.90652
TRINITY_DN8944_c0_g1_i1_orf1	turquoise	0.8315
TRINITY_DN21623_c0_g2_i1_orf1	red	0.7292
TRINITY_DN3190_c0_g1_i1_orf1	blue	0.96884
TRINITY_DN3551_c0_g1_i4_orf1	turquoise	0.96343
TRINITY_DN48713_c0_g1_i1_orf1	turquoise	0.62571
TRINITY_DN19923_c0_g1_i1_orf1	blue	0.90458
TRINITY_DN969_c0_g1_i3_orf1	turquoise	0.87195
TRINITY_DN3651_c0_g1_i5_orf1	turquoise	0.49147
TRINITY_DN4501_c0_g1_i3_orf1	turquoise	0.71451
TRINITY_DN48023_c1_g1_i1_orf1	blue	0.89451
TRINITY_DN14094_c0_g1_i1_orfp1	turquoise	0.36536
TRINITY_DN9874_c0_g1_i7_orf1	turquoise	0.95531
TRINITY_DN37336_c1_g1_i1_orf1	turquoise	0.6469
TRINITY_DN3520_c0_g1_i4_orf1	turquoise	0.57768
TRINITY_DN34153_c0_g2_i2_orf1	brown	0.79646
TRINITY_DN27332_c0_g2_i1_orf1	yellow	0.76337
TRINITY_DN24410_c0_g2_i1_orf1	brown	0.57943
TRINITY_DN85490_c0_g2_i1_orf1	turquoise	0.78311
TRINITY_DN211_c1_g1_i10_orf1	turquoise	0.91202
TRINITY_DN24631_c0_g2_i1_orf1	blue	0.90383
TRINITY_DN5562_c1_g2_i1_orf1	turquoise	0.88703
TRINITY_DN82426_c0_g1_i6_orfp1	blue	0.97095
TRINITY_DN9637_c0_g1_i14_orf1	red	0.88445
TRINITY_DN41179_c0_g1_i1_orf1	turquoise	0.9359
TRINITY_DN8044_c0_g1_i2_orf1	red	0.62679

TRINITY_DN1710_c0_g1_i1_orf1	green	0.94122
TRINITY_DN1173_c0_g1_i11_orf1	yellow	0.86251
TRINITY_DN62_c0_g1_i7_orf1	yellow	0.85005
TRINITY_DN25210_c0_g1_i1_orf1	turquoise	0.94614
TRINITY_DN9498_c0_g1_i3_orf1	turquoise	0.98805
TRINITY_DN1405_c0_g1_i1_orf1	red	0.89021
TRINITY_DN618_c0_g1_i3_orf1	brown	0.975
TRINITY_DN41546_c0_g1_i15_orf1	turquoise	0.87654
TRINITY_DN20984_c0_g1_i4_orf1	turquoise	0.9238
TRINITY_DN7170_c0_g1_i11_orf1	black	0.74796
TRINITY_DN45000_c0_g1_i5_orf1	turquoise	0.93089
TRINITY_DN4002_c0_g1_i1_orf1	turquoise	0.77591
TRINITY_DN44110_c0_g1_i4_orf1	brown	0.67391
TRINITY_DN7247_c0_g1_i6_orf1	grey	-0.42143
TRINITY_DN13067_c0_g1_i6_orf1	blue	0.90218
TRINITY_DN5200_c0_g1_i2_orf1	red	0.82823
TRINITY_DN69713_c0_g1_i1_orf1	turquoise	0.97239
TRINITY_DN22156_c0_g1_i1_orf1	turquoise	0.69572
TRINITY_DN14670_c0_g1_i1_orf1	brown	0.91875
TRINITY_DN5107_c0_g1_i4_orf1	turquoise	0.9844
TRINITY_DN15961_c0_g1_i1_orf1	green	0.88689
TRINITY_DN19917_c0_g1_i1_orf1	turquoise	0.99361
TRINITY_DN227_c0_g1_i1_orf1	turquoise	0.79845
TRINITY_DN26961_c0_g1_i1_orf1	yellow	0.87448
TRINITY_DN2343_c1_g1_i8_orf1	turquoise	0.98379
TRINITY_DN16905_c0_g1_i1_orf1	black	0.78925
TRINITY_DN4301_c2_g2_i4_orf1	turquoise	0.86124
TRINITY_DN17049_c0_g1_i6_orf1	turquoise	0.99259
TRINITY_DN18482_c0_g1_i3_orf1	brown	0.92764
TRINITY_DN15858_c0_g1_i1_orf1	brown	0.96528
TRINITY_DN22515_c0_g1_i10_orf1	green	0.92001
TRINITY_DN838_c0_g1_i18_orf1	yellow	0.73562
TRINITY_DN112120_c0_g1_i1_orf1	turquoise	0.9421
TRINITY_DN14587_c0_g1_i7_orf1	green	0.88886
TRINITY_DN8555_c0_g1_i1_orf1	green	0.85008
TRINITY_DN40704_c0_g1_i2_orf1	turquoise	0.96119
TRINITY_DN3747_c1_g2_i1_orf1	turquoise	0.44119
TRINITY_DN10070_c0_g1_i1_orf1	brown	0.74493
TRINITY_DN5312_c4_g1_i2_orf1	turquoise	0.89973
TRINITY_DN2844_c0_g1_i2_orf1	brown	0.83923
TRINITY_DN12367_c0_g1_i4_orf1	turquoise	0.34624
TRINITY_DN6545_c0_g1_i6_orf1	turquoise	0.82788
TRINITY_DN48096_c0_g2_i2_orf1	turquoise	0.83246
TRINITY_DN19830_c0_g1_i1_orf1	turquoise	0.71983
TRINITY_DN23444_c0_g1_i10_orf1	turquoise	0.88505
TRINITY_DN30070_c0_g1_i6_orf1	turquoise	0.9178
TRINITY_DN4710_c0_g1_i1_orf1	turquoise	0.95878
TRINITY_DN13139_c0_g1_i1_orf1	turquoise	0.76898
TRINITY_DN105157_c0_g1_i1_orf1	yellow	0.71665
TRINITY_DN138481_c0_g1_i2_orf1	blue	0.96789
TRINITY_DN15865_c0_g2_i2_orf1	blue	0.92972
TRINITY_DN6205_c0_g1_i8_orf1	black	0.47923
TRINITY_DN3260_c0_g1_i6_orf1	turquoise	0.72079
TRINITY_DN103511_c0_g1_i4_orf1	green	0.8062
TRINITY_DN15247_c0_g1_i2_orf1	black	0.95523
TRINITY_DN2326_c0_g1_i1_orf1	black	0.77639
TRINITY_DN1470_c0_g1_i8_orf1	brown	0.88787
TRINITY_DN135449_c0_g1_i5_orf1	green	0.96396

TRINITY_DN9569_c1_g1_i7_orf1	yellow	0.76044
TRINITY_DN4245_c0_g1_i5_orf1	brown	0.97414
TRINITY_DN38230_c0_g1_i4_orf1	yellow	0.84908
TRINITY_DN9926_c1_g1_i1_orf1	black	0.70601
TRINITY_DN73230_c0_g1_i1_orf1	turquoise	0.48683
TRINITY_DN18650_c0_g1_i1_orf1	blue	0.85529
TRINITY_DN10399_c0_g1_i2_orf1	turquoise	0.97549
TRINITY_DN28711_c1_g1_i1_orf1	blue	0.86612
TRINITY_DN240_c0_g1_i4_orf1	turquoise	0.61659
TRINITY_DN7574_c0_g1_i10_orf1	turquoise	0.93673
TRINITY_DN18216_c0_g1_i4_orf1	yellow	0.48544
TRINITY_DN1533_c0_g2_i1_orf1	blue	0.96133
TRINITY_DN3482_c0_g2_i1_orf1	red	0.70555
TRINITY_DN20957_c0_g1_i1_orf1	turquoise	0.68039
TRINITY_DN12113_c0_g1_i1_orf1	turquoise	0.79424
TRINITY_DN39837_c0_g1_i1_orf1	brown	0.81003
TRINITY_DN117362_c0_g1_i5_orf1	turquoise	0.77065
TRINITY_DN2069_c1_g1_i8_orf1	blue	0.94275
TRINITY_DN32586_c0_g2_i1_orf1	yellow	0.85713
TRINITY_DN20067_c0_g1_i6_orf1	red	0.92616
TRINITY_DN5568_c0_g2_i2_orf1	red	0.91173
TRINITY_DN1153_c1_g1_i1_orf1	turquoise	0.58134
TRINITY_DN101922_c0_g1_i1_orf1	turquoise	0.77483
TRINITY_DN46173_c0_g3_i1_orf1	yellow	0.98493
TRINITY_DN90321_c0_g2_i1_orf1	turquoise	0.79092
TRINITY_DN3503_c0_g1_i1_orfp1	turquoise	0.96549
TRINITY_DN29633_c0_g1_i8_orf1	red	0.79308
TRINITY_DN3166_c1_g1_i6_orf1	brown	0.94938
TRINITY_DN7123_c0_g1_i1_orf1	blue	0.95252
TRINITY_DN10229_c0_g1_i6_orf1	black	0.94308
TRINITY_DN14673_c0_g1_i3_orf1	brown	0.6719
TRINITY_DN100_c0_g1_i9_orf1	yellow	0.88093
TRINITY_DN9554_c0_g1_i1_orf1	grey	-0.11927
TRINITY_DN33418_c0_g1_i1_orf1	turquoise	0.39311
TRINITY_DN146758_c0_g1_i1_orf1	turquoise	0.84638
TRINITY_DN52861_c0_g1_i1_orf1	turquoise	0.98282
TRINITY_DN26650_c0_g1_i1_orfp1	turquoise	0.89574
TRINITY_DN2232_c1_g1_i3_orf1	turquoise	0.66944
TRINITY_DN14217_c0_g1_i1_orf1	red	0.64239
TRINITY_DN14774_c0_g1_i4_orf1	blue	0.84734
TRINITY_DN2861_c0_g2_i1_orf1	green	0.91125
TRINITY_DN70382_c0_g1_i10_orf1	turquoise	0.72919
TRINITY_DN5337_c0_g1_i6_orf1	brown	0.8769
TRINITY_DN25681_c0_g1_i5_orf1	turquoise	0.70802
TRINITY_DN535_c3_g2_i1_orf1	turquoise	0.88893
TRINITY_DN1617_c0_g1_i5_orf1	turquoise	0.96688
TRINITY_DN9492_c0_g1_i7_orf1	brown	0.90538
TRINITY_DN23264_c0_g1_i1_orf1	turquoise	0.88162
TRINITY_DN123184_c0_g1_i1_orf1	red	0.92761
TRINITY_DN841_c0_g1_i4_orf1	yellow	0.58641
TRINITY_DN21943_c1_g1_i1_orf1	yellow	0.67709
TRINITY_DN7908_c0_g1_i5_orf1	turquoise	0.7489
TRINITY_DN3276_c0_g1_i4_orf1	black	0.7574
TRINITY_DN9044_c0_g1_i2_orf1	black	0.81987
TRINITY_DN30932_c0_g1_i2_orf1	turquoise	0.95974
TRINITY_DN171_c0_g1_i1_orf1	brown	0.5951
TRINITY_DN2343_c1_g1_i2_orf1	black	0.7179
TRINITY_DN5442_c0_g1_i4_orf1	red	0.85995

TRINITY_DN51766_c0_g1_i2_orf1	turquoise	0.98516
TRINITY_DN2438_c0_g1_i4_orf1	red	0.81911
TRINITY_DN896_c0_g1_i2_orf1	black	0.85369
TRINITY_DN1506_c0_g1_i6_orfp1	yellow	0.7586
TRINITY_DN26149_c0_g1_i5_orf1	blue	0.96704
TRINITY_DN22674_c0_g1_i2_orf1	green	0.83948
TRINITY_DN37654_c0_g1_i5_orf1	green	0.85291
TRINITY_DN8908_c0_g1_i1_orf1	turquoise	0.8428
TRINITY_DN14937_c0_g1_i7_orf1	turquoise	0.96008
TRINITY_DN1775_c0_g1_i3_orf1	turquoise	0.92324
TRINITY_DN6914_c0_g1_i2_orf1	turquoise	0.55547
TRINITY_DN34509_c0_g1_i1_orf1	turquoise	0.88766
TRINITY_DN45962_c1_g1_i2_orf1	grey	0.38423
TRINITY_DN2464_c0_g1_i12_orf1	brown	0.94241
TRINITY_DN7633_c0_g1_i1_orf1	green	0.91253
TRINITY_DN14843_c0_g1_i1_orf1	turquoise	0.65208
TRINITY_DN1123_c2_g1_i3_orf1	yellow	0.96091
TRINITY_DN5603_c0_g1_i1_orf1	black	0.81433
TRINITY_DN1436_c0_g1_i5_orf1	red	0.59303
TRINITY_DN4069_c0_g1_i5_orf1	brown	0.92894
TRINITY_DN143895_c0_g1_i1_orf1	green	0.98868
TRINITY_DN46367_c0_g1_i2_orf1	turquoise	0.97321
TRINITY_DN7024_c0_g1_i1_orf1	turquoise	0.98608
TRINITY_DN4013_c0_g1_i4_orf1	blue	0.91244
TRINITY_DN10824_c0_g1_i3_orf1	blue	0.9754
TRINITY_DN1982_c0_g1_i17_orf1	yellow	0.88605
TRINITY_DN198_c2_g1_i2_orf1	blue	0.83542
TRINITY_DN124171_c0_g1_i4_orf1	yellow	0.83175
TRINITY_DN1273_c0_g1_i4_orf1	turquoise	0.66625
TRINITY_DN18230_c1_g2_i1_orf1	green	0.95806
TRINITY_DN18728_c0_g1_i2_orf1	turquoise	0.98648
TRINITY_DN3015_c0_g1_i7_orf1	brown	0.96006
TRINITY_DN1921_c1_g1_i5_orf1	red	0.92062
TRINITY_DN34745_c0_g2_i1_orf1	red	0.93112
TRINITY_DN5765_c0_g2_i3_orf1	turquoise	0.84367
TRINITY_DN43391_c0_g1_i5_orf1	turquoise	0.97962
TRINITY_DN30012_c1_g1_i1_orf1	brown	0.94743
TRINITY_DN726_c0_g1_i8_orf1	brown	0.86677
TRINITY_DN1436_c0_g1_i3_orf1	blue	0.79265
TRINITY_DN3235_c0_g1_i1_orf1	turquoise	0.79132
TRINITY_DN364_c5_g1_i3_orf1	black	0.97016
TRINITY_DN2243_c0_g1_i4_orf1	turquoise	0.98809
TRINITY_DN20321_c0_g1_i5_orf1	turquoise	0.83082
TRINITY_DN4425_c0_g1_i4_orf1	yellow	0.66951
TRINITY_DN128231_c0_g1_i5_orf1	green	0.46481
TRINITY_DN27704_c0_g1_i1_orf1	turquoise	0.98024
TRINITY_DN3906_c0_g1_i5_orf1	yellow	0.9313
TRINITY_DN5022_c0_g1_i4_orf1	black	0.95945
TRINITY_DN2430_c0_g1_i1_orf1	turquoise	0.93008
TRINITY_DN44857_c0_g1_i4_orf1	black	0.74926
TRINITY_DN8625_c0_g1_i1_orf1	brown	0.72253
TRINITY_DN359_c0_g1_i5_orf1	black	0.59031
TRINITY_DN7583_c0_g1_i1_orf1	turquoise	0.92525
TRINITY_DN14239_c0_g1_i5_orf1	brown	0.68558
TRINITY_DN1607_c0_g1_i16_orf1	yellow	0.87533
TRINITY_DN30185_c0_g1_i3_orf1	green	0.3225
TRINITY_DN23586_c0_g1_i3_orf1	turquoise	0.96661
TRINITY_DN5475_c0_g1_i3_orf1	red	0.76839

TRINITY_DN45037_c0_g1_i1_orf1	green	0.94812
TRINITY_DN24218_c0_g1_i1_orf1	red	0.84613
TRINITY_DN5578_c0_g1_i10_orf1	turquoise	0.96195
TRINITY_DN5696_c0_g1_i4_orf1	brown	0.79686
TRINITY_DN42759_c0_g3_i1_orf1	turquoise	0.97534
TRINITY_DN3469_c0_g1_i4_orf1	grey	-0.00042
TRINITY_DN1786_c0_g1_i11_orf1	green	0.85826
TRINITY_DN26089_c0_g1_i1_orf1	blue	0.53211
TRINITY_DN106534_c0_g1_i1_orf1	turquoise	0.75755
TRINITY_DN3073_c0_g1_i7_orf1	blue	0.97176
TRINITY_DN2286_c2_g1_i1_orf1	green	0.83676
TRINITY_DN7735_c1_g1_i1_orf1	blue	0.92153
TRINITY_DN5444_c0_g2_i1_orf1	blue	0.98677
TRINITY_DN880_c0_g1_i6_orf1	red	0.89765
TRINITY_DN19116_c0_g1_i3_orf1	brown	0.91719
TRINITY_DN95414_c0_g1_i1_orf1	turquoise	0.84362
TRINITY_DN642_c0_g1_i6_orf1	turquoise	0.92255
TRINITY_DN3472_c0_g1_i6_orf1	grey	0.75993
TRINITY_DN2691_c0_g1_i1_orf1	turquoise	0.96003
TRINITY_DN1718_c6_g1_i4_orf1	yellow	0.85346
TRINITY_DN1206_c0_g1_i6_orf1	brown	0.97034
TRINITY_DN37055_c0_g1_i1_orf1	brown	0.74499
TRINITY_DN114344_c0_g1_i4_orf1	yellow	0.9496
TRINITY_DN6358_c0_g1_i5_orf1	yellow	0.5804
TRINITY_DN1262_c0_g1_i2_orf1	yellow	0.89122
TRINITY_DN119893_c0_g2_i3_orf1	yellow	0.6867
TRINITY_DN43881_c0_g1_i2_orf1	yellow	0.76925
TRINITY_DN18502_c0_g1_i1_orf1	red	0.90996
TRINITY_DN18922_c0_g1_i1_orf1	red	0.76699
TRINITY_DN34040_c0_g2_i1_orf1	turquoise	0.90543
TRINITY_DN95665_c0_g1_i1_orf1	turquoise	0.96304
TRINITY_DN21852_c0_g1_i1_orf1	turquoise	0.62369
TRINITY_DN147458_c0_g1_i1_orf1	red	0.93908
TRINITY_DN501_c1_g1_i1_orf1	turquoise	0.99085
TRINITY_DN83005_c0_g1_i1_orf1	turquoise	0.98355
TRINITY_DN8692_c0_g1_i2_orf1	blue	0.95928
TRINITY_DN72999_c0_g1_i1_orf1	red	0.93998
TRINITY_DN1103_c0_g1_i19_orf1	yellow	0.79512
TRINITY_DN1416_c0_g2_i1_orf1	turquoise	0.55073
TRINITY_DN146006_c0_g1_i1_orf1	turquoise	0.59395
TRINITY_DN206_c0_g1_i8_orf1	blue	0.92251
TRINITY_DN21251_c1_g1_i1_orf1	turquoise	0.93389
TRINITY_DN895_c0_g2_i1_orf1	blue	0.87803
TRINITY_DN8703_c0_g1_i2_orf1	green	0.92642
TRINITY_DN19098_c0_g1_i4_orf1	brown	0.82534
TRINITY_DN22604_c0_g1_i3_orf1	green	0.74578
TRINITY_DN25534_c0_g1_i1_orf1	blue	0.94149
TRINITY_DN1768_c0_g1_i2_orf1	blue	0.8106
TRINITY_DN32538_c0_g1_i2_orf1	turquoise	0.93269
TRINITY_DN30178_c0_g1_i3_orf1	red	0.879
TRINITY_DN15373_c0_g1_i2_orf1	yellow	0.80615
TRINITY_DN33452_c0_g1_i3_orf1	turquoise	0.5358
TRINITY_DN127056_c0_g1_i1_orf1	turquoise	0.53083
TRINITY_DN24873_c0_g1_i4_orf1	turquoise	0.98372
TRINITY_DN2402_c1_g1_i8_orf1	turquoise	0.41981
TRINITY_DN1305_c0_g1_i6_orf1	brown	0.67499
TRINITY_DN2168_c0_g1_i2_orf1	turquoise	0.94804
TRINITY_DN64472_c0_g2_i1_orf1	turquoise	0.85469

TRINITY_DN7556_c0_g1_i1_orf1	turquoise	0.98157
TRINITY_DN36281_c0_g1_i2_orf1	blue	0.91673
TRINITY_DN19262_c0_g1_i1_orf1	turquoise	0.84889
TRINITY_DN14865_c0_g1_i2_orf1	red	0.82234
TRINITY_DN2881_c0_g1_i7_orf1	grey	-0.5841
TRINITY_DN43076_c0_g1_i6_orf1	red	0.88483
TRINITY_DN8366_c0_g1_i4_orf1	turquoise	0.95565
TRINITY_DN34830_c0_g1_i1_orf1	blue	0.87501
TRINITY_DN8252_c0_g1_i6_orf1	brown	0.69916
TRINITY_DN14987_c0_g1_i3_orf1	red	0.94956
TRINITY_DN29440_c1_g1_i4_orf1	turquoise	0.92028
TRINITY_DN23962_c0_g1_i3_orf1	turquoise	0.63643
TRINITY_DN12771_c0_g1_i1_orf1	red	0.81688
TRINITY_DN54586_c1_g1_i1_orf1	red	0.81542
TRINITY_DN3597_c0_g1_i10_orf1	turquoise	0.65704
TRINITY_DN12317_c0_g1_i1_orf1	black	0.76831
TRINITY_DN1459_c0_g1_i2_orf1	yellow	0.7904
TRINITY_DN44792_c0_g1_i1_orf1	red	0.70058
TRINITY_DN2803_c4_g1_i1_orf1	blue	0.79489
TRINITY_DN3219_c0_g1_i6_orf1	brown	0.39969
TRINITY_DN38341_c0_g2_i2_orf1	turquoise	0.94052
TRINITY_DN2710_c0_g1_i4_orf1	brown	0.80764
TRINITY_DN76529_c0_g1_i1_orfp1	yellow	0.88788
TRINITY_DN117042_c0_g1_i2_orf1	green	0.85026
TRINITY_DN3209_c0_g2_i6_orf1	blue	0.31557
TRINITY_DN2238_c0_g2_i1_orf1	turquoise	0.95262
TRINITY_DN40191_c2_g1_i1_orf1	blue	0.73116
TRINITY_DN86621_c0_g1_i2_orf1	blue	0.81823
TRINITY_DN89829_c0_g1_i1_orf1	red	0.89771
TRINITY_DN36324_c0_g1_i12_orf1	blue	0.64597
TRINITY_DN10636_c0_g1_i1_orf1	turquoise	0.91159
TRINITY_DN79083_c0_g1_i2_orf1	turquoise	0.40112
TRINITY_DN10455_c0_g2_i1_orf1	brown	0.77604
TRINITY_DN58013_c0_g1_i6_orf1	brown	0.5945
TRINITY_DN2673_c2_g1_i2_orf1	pink	0.53657
TRINITY_DN1384_c0_g1_i5_orf1	blue	0.82772
TRINITY_DN17838_c0_g1_i4_orf1	green	0.90358
TRINITY_DN27960_c0_g1_i1_orf1	turquoise	0.95674
TRINITY_DN2515_c0_g1_i6_orf1	blue	0.98269
TRINITY_DN2450_c0_g1_i6_orf1	red	0.9684
TRINITY_DN49527_c0_g1_i1_orf1	turquoise	0.96701
TRINITY_DN486_c0_g1_i5_orf1	red	0.90559
TRINITY_DN65299_c0_g4_i1_orf1	black	0.95396
TRINITY_DN42506_c0_g1_i1_orf1	turquoise	0.95556
TRINITY_DN4711_c0_g1_i2_orf1	blue	0.76884
TRINITY_DN87603_c0_g2_i1_orf1	turquoise	0.56801
TRINITY_DN1287_c0_g1_i5_orf1	green	0.97577
TRINITY_DN2647_c0_g1_i3_orf1	pink	0.90467
TRINITY_DN6684_c0_g1_i4_orf1	grey	-0.755
TRINITY_DN38568_c0_g1_i1_orf1	yellow	0.89534
TRINITY_DN1477_c0_g1_i5_orf1	turquoise	0.98393
TRINITY_DN6248_c0_g1_i1_orf1	turquoise	0.93225
TRINITY_DN357_c0_g1_i8_orf1	turquoise	0.79047
TRINITY_DN76815_c0_g1_i3_orf1	yellow	0.69553
TRINITY_DN116467_c0_g1_i1_orf1	blue	0.82237
TRINITY_DN380_c0_g2_i2_orf1	brown	0.9669
TRINITY_DN37538_c0_g1_i1_orf1	turquoise	0.9868
TRINITY_DN4323_c0_g1_i1_orf1	turquoise	0.56725

TRINITY_DN3255_c0_g1_i1_orf1	blue	0.90367
TRINITY_DN29156_c0_g1_i1_orf1	turquoise	0.96294
TRINITY_DN16258_c0_g1_i2_orf1	turquoise	0.91867
TRINITY_DN20767_c0_g2_i1_orf1	red	0.95438
TRINITY_DN1264_c0_g1_i2_orf1	pink	0.93741
TRINITY_DN77005_c0_g2_i1_orf1	blue	0.6006
TRINITY_DN81488_c0_g1_i1_orf1	blue	0.82656
TRINITY_DN581_c3_g2_i1_orf1	brown	0.86921
TRINITY_DN5031_c0_g1_i1_orf1	turquoise	0.91539
TRINITY_DN1952_c0_g1_i2_orf1	turquoise	0.78267
TRINITY_DN461_c0_g1_i5_orf1	turquoise	0.71282
TRINITY_DN1814_c0_g2_i4_orfp1	yellow	0.92033
TRINITY_DN24970_c0_g1_i4_orf1	brown	0.98506
TRINITY_DN20676_c0_g1_i6_orf1	green	0.87522
TRINITY_DN42753_c0_g1_i2_orf1	blue	0.77888
TRINITY_DN36928_c0_g1_i2_orf1	turquoise	0.80162
TRINITY_DN9980_c0_g1_i1_orf1	red	0.73073
TRINITY_DN13157_c0_g1_i1_orf1	yellow	0.53245
TRINITY_DN4159_c1_g1_i1_orf1	red	0.88172
TRINITY_DN384_c0_g1_i8_orf1	brown	0.94933
TRINITY_DN48097_c0_g1_i1_orf1	yellow	0.73497
TRINITY_DN15682_c0_g1_i4_orf1	brown	0.60179
TRINITY_DN72369_c0_g1_i1_orf1	green	0.90731
TRINITY_DN95971_c0_g5_i1_orf1	yellow	0.82896
TRINITY_DN146957_c0_g1_i1_orf1	black	0.84087
TRINITY_DN11693_c0_g1_i6_orf1	black	0.82033
TRINITY_DN4132_c0_g1_i14_orf1	turquoise	0.76807
TRINITY_DN142588_c0_g1_i1_orf1	red	0.9142
TRINITY_DN17329_c0_g2_i3_orf1	blue	0.79923
TRINITY_DN9325_c0_g1_i1_orf1	green	0.86766
TRINITY_DN90289_c0_g1_i5_orf1	turquoise	0.94773
TRINITY_DN272_c0_g1_i1_orf1	green	0.78984
TRINITY_DN20558_c0_g1_i2_orf1	brown	0.82342
TRINITY_DN13496_c0_g1_i7_orf1	turquoise	0.96022
TRINITY_DN9383_c0_g1_i3_orf1	blue	0.77162
TRINITY_DN111621_c0_g3_i1_orf1	blue	0.89136
TRINITY_DN2918_c0_g1_i1_orf1	turquoise	0.96042
TRINITY_DN695_c0_g1_i12_orf1	yellow	0.9279
TRINITY_DN10994_c0_g1_i4_orf1	brown	0.96999
TRINITY_DN16972_c0_g1_i1_orf1	blue	0.46908
TRINITY_DN76283_c0_g6_i1_orf1	turquoise	0.9889
TRINITY_DN2475_c0_g2_i1_orf1	turquoise	0.55932
TRINITY_DN3766_c0_g1_i10_orf1	brown	0.8368
TRINITY_DN2896_c0_g1_i2_orf1	blue	0.96594
TRINITY_DN26301_c0_g1_i1_orf1	blue	0.99574
TRINITY_DN11514_c0_g1_i1_orf1	black	0.97002
TRINITY_DN4959_c0_g1_i1_orf1	turquoise	0.98005
TRINITY_DN79868_c0_g1_i1_orf1	turquoise	0.80239
TRINITY_DN17913_c0_g1_i8_orf1	green	0.86952
TRINITY_DN14168_c0_g1_i1_orf1	turquoise	0.95919
TRINITY_DN2264_c0_g1_i1_orf1	green	0.89196
TRINITY_DN1960_c5_g1_i3_orf1	brown	0.9533
TRINITY_DN2835_c0_g1_i6_orf1	blue	0.82667
TRINITY_DN49265_c0_g3_i2_orf1	turquoise	0.98257
TRINITY_DN11637_c0_g1_i1_orf1	turquoise	0.56299
TRINITY_DN1760_c0_g1_i4_orf1	turquoise	0.48478
TRINITY_DN81248_c0_g1_i1_orf1	turquoise	0.83771
TRINITY_DN32_c0_g1_i4_orf1	pink	0.96661

TRINITY_DN72934_c0_g1_i1_orf1	turquoise	0.96406
TRINITY_DN48410_c0_g2_i1_orf1	turquoise	0.98536
TRINITY_DN4891_c0_g1_i4_orf1	turquoise	0.64718
TRINITY_DN15845_c0_g1_i1_orf1	turquoise	0.82181
TRINITY_DN644_c0_g1_i1_orf1	blue	0.93218
TRINITY_DN136028_c0_g2_i1_orf1	turquoise	0.94269
TRINITY_DN139212_c0_g1_i4_orf1	turquoise	0.87518
TRINITY_DN31253_c0_g1_i2_orf1	turquoise	0.92676
TRINITY_DN125140_c0_g1_i1_orf1	black	0.86759
TRINITY_DN4859_c0_g1_i5_orf1	turquoise	0.7723
TRINITY_DN77572_c0_g1_i1_orf1	turquoise	0.55395
TRINITY_DN147475_c0_g1_i1_orf1	red	0.93715
TRINITY_DN135188_c0_g1_i2_orf1	brown	0.96434
TRINITY_DN23364_c0_g1_i1_orf1	blue	0.9298
TRINITY_DN3029_c4_g1_i1_orf1	turquoise	0.76825
TRINITY_DN1509_c0_g1_i1_orf1	turquoise	0.94727
TRINITY_DN57918_c0_g1_i1_orf1	turquoise	0.72961
TRINITY_DN747_c0_g1_i4_orf1	turquoise	0.97923
TRINITY_DN2141_c0_g1_i1_orf1	black	0.85023
TRINITY_DN21380_c0_g1_i1_orf1	blue	0.57164
TRINITY_DN26254_c0_g1_i1_orf1	yellow	0.96998
TRINITY_DN38482_c0_g1_i4_orf1	pink	0.59324
TRINITY_DN7688_c0_g1_i10_orf1	turquoise	0.98234
TRINITY_DN6380_c0_g1_i1_orf1	turquoise	0.73595
TRINITY_DN33728_c0_g2_i1_orf1	blue	0.90874
TRINITY_DN25960_c0_g1_i1_orf1	turquoise	0.79493
TRINITY_DN5092_c0_g1_i2_orf1	turquoise	0.97264
TRINITY_DN350_c0_g1_i4_orf1	brown	0.965
TRINITY_DN5207_c0_g2_i3_orf1	green	0.78129
TRINITY_DN3301_c0_g1_i2_orf1	green	0.97546
TRINITY_DN8430_c0_g1_i1_orf1	turquoise	0.59521
TRINITY_DN43505_c0_g1_i1_orf1	green	0.94244
TRINITY_DN496_c0_g1_i7_orf1	turquoise	0.63693
TRINITY_DN12579_c0_g1_i1_orf1	turquoise	0.95146
TRINITY_DN52761_c0_g1_i2_orf1	brown	0.80294
TRINITY_DN1493_c0_g1_i5_orf1	brown	0.5529
TRINITY_DN17394_c0_g1_i1_orf1	turquoise	0.90229
TRINITY_DN12387_c1_g2_i1_orf1	blue	0.97068
TRINITY_DN15400_c0_g1_i1_orf1	black	0.88233
TRINITY_DN10455_c0_g1_i2_orf1	turquoise	0.88642
TRINITY_DN19748_c0_g1_i4_orf1	black	0.90806
TRINITY_DN344_c1_g1_i1_orf1	turquoise	0.7532
TRINITY_DN471_c0_g1_i6_orf1	red	0.89098
TRINITY_DN7735_c0_g1_i4_orf1	yellow	0.90821
TRINITY_DN2956_c0_g1_i6_orf1	yellow	0.89571
TRINITY_DN22842_c0_g1_i4_orf1	turquoise	0.98046
TRINITY_DN3433_c2_g1_i2_orf1	brown	0.83308
TRINITY_DN35245_c0_g1_i1_orf1	turquoise	0.87909
TRINITY_DN9920_c0_g1_i1_orf1	blue	0.94794
TRINITY_DN1216_c0_g1_i4_orf1	brown	0.93369
TRINITY_DN20_c0_g1_i1_orf1	turquoise	0.32288
TRINITY_DN35351_c0_g1_i3_orf1	blue	0.97308
TRINITY_DN2638_c0_g1_i7_orf1	red	0.95396
TRINITY_DN2848_c0_g1_i1_orf1	brown	0.44345
TRINITY_DN12582_c0_g1_i5_orf1	green	0.8988
TRINITY_DN11596_c0_g1_i1_orf1	grey	0.65047
TRINITY_DN2302_c0_g1_i1_orf1	yellow	0.74646
TRINITY_DN1612_c0_g1_i3_orf1	green	0.91674

TRINITY_DN1306_c0_g1_i8_orf1	yellow	0.88293
TRINITY_DN34056_c0_g1_i4_orf1	grey	0.78749
TRINITY_DN6202_c0_g1_i2_orf1	red	0.84631
TRINITY_DN23416_c1_g1_i2_orf1	blue	0.69085
TRINITY_DN20717_c0_g1_i1_orf1	blue	0.89157
TRINITY_DN10774_c0_g2_i3_orf1	brown	0.89815
TRINITY_DN54925_c0_g1_i1_orf1	red	0.79173
TRINITY_DN16900_c0_g2_i1_orf1	turquoise	0.95766
TRINITY_DN23946_c0_g1_i1_orf1	red	0.91671
TRINITY_DN21909_c0_g1_i1_orf1	turquoise	0.99536
TRINITY_DN2780_c0_g1_i5_orf1	turquoise	0.97102
TRINITY_DN19746_c0_g1_i5_orf1	blue	0.83682
TRINITY_DN19250_c0_g2_i2_orf1	turquoise	0.75751
TRINITY_DN20238_c0_g1_i7_orf1	turquoise	0.4908
TRINITY_DN1047_c0_g1_i6_orf1	brown	0.98235
TRINITY_DN338_c2_g1_i2_orf1	turquoise	0.97145
TRINITY_DN1091_c0_g2_i10_orf1	blue	0.76323
TRINITY_DN295_c5_g1_i2_orf1	brown	0.91394
TRINITY_DN12193_c0_g1_i6_orf1	green	0.82505
TRINITY_DN53807_c0_g2_i1_orf1	green	0.96729
TRINITY_DN1515_c0_g1_i2_orf1	turquoise	0.74883
TRINITY_DN37165_c0_g1_i4_orf1	turquoise	0.97688
TRINITY_DN18148_c0_g2_i1_orf1	green	0.82036
TRINITY_DN7909_c0_g2_i1_orf1	turquoise	0.67192
TRINITY_DN20346_c0_g1_i1_orf1	turquoise	0.98725
TRINITY_DN662_c0_g1_i1_orf1	turquoise	0.68874
TRINITY_DN1149_c0_g1_i4_orf1	brown	0.90111
TRINITY_DN416_c0_g1_i1_orf1	yellow	0.95005
TRINITY_DN3614_c0_g2_i1_orf1	red	0.85347
TRINITY_DN15382_c0_g1_i3_orf1	black	0.82003
TRINITY_DN4814_c0_g1_i6_orf1	turquoise	0.81978
TRINITY_DN27723_c0_g1_i2_orf1	turquoise	0.79885
TRINITY_DN3454_c0_g1_i1_orf1	turquoise	0.8863
TRINITY_DN10057_c0_g2_i1_orf1	red	0.94776
TRINITY_DN25856_c0_g1_i1_orf1	turquoise	0.98625
TRINITY_DN21170_c0_g1_i5_orf1	yellow	0.77992
TRINITY_DN82_c0_g1_i1_orf1	black	0.54406
TRINITY_DN2593_c0_g3_i1_orf1	turquoise	0.952
TRINITY_DN6235_c0_g1_i5_orf1	turquoise	0.9779
TRINITY_DN11620_c0_g1_i2_orf1	blue	0.52354
TRINITY_DN34751_c0_g1_i1_orf1	blue	0.86028
TRINITY_DN2593_c0_g1_i1_orf1	turquoise	0.93151
TRINITY_DN3092_c0_g1_i2_orf1	grey	0.87549
TRINITY_DN51836_c0_g3_i1_orf1	blue	0.47597
TRINITY_DN2215_c0_g2_i1_orf1	yellow	0.97802
TRINITY_DN2772_c0_g1_i3_orf1	brown	0.84241
TRINITY_DN8685_c0_g1_i5_orf1	brown	0.86889
TRINITY_DN8343_c0_g1_i2_orf1	turquoise	0.95584
TRINITY_DN26209_c0_g1_i6_orf1	green	0.90483
TRINITY_DN628_c0_g1_i1_orf1	brown	0.92279
TRINITY_DN100821_c0_g1_i1_orf1	turquoise	0.97455
TRINITY_DN2615_c0_g1_i1_orf1	turquoise	0.59089
TRINITY_DN18396_c0_g1_i1_orf1	turquoise	0.79352
TRINITY_DN93566_c0_g2_i1_orf1	turquoise	0.90589
TRINITY_DN5174_c0_g3_i1_orf1	brown	0.69533
TRINITY_DN105749_c0_g1_i1_orf1	green	0.58581
TRINITY_DN43942_c0_g1_i1_orf1	turquoise	0.91654
TRINITY_DN5998_c0_g2_i1_orf1	yellow	0.70983

TRINITY_DN1789_c0_g1_i5_orf1	brown	0.67072
TRINITY_DN4068_c1_g2_i1_orf1	yellow	0.85093
TRINITY_DN7064_c0_g1_i20_orfp1	brown	0.65439
TRINITY_DN16965_c0_g2_i1_orf1	turquoise	0.87946
TRINITY_DN97472_c0_g1_i5_orf1	yellow	0.85353
TRINITY_DN8700_c9_g1_i1_orf1	blue	0.90898
TRINITY_DN4916_c0_g2_i1_orf1	brown	0.65247
TRINITY_DN5531_c0_g3_i3_orf1	yellow	0.82377
TRINITY_DN41_c0_g1_i3_orf1	black	0.95269
TRINITY_DN135679_c0_g1_i2_orfp1	brown	0.85625
TRINITY_DN6602_c0_g1_i4_orf1	turquoise	0.55303
TRINITY_DN13259_c0_g1_i2_orf1	turquoise	0.91507
TRINITY_DN4145_c0_g1_i1_orf1	turquoise	0.7325
TRINITY_DN51830_c0_g1_i4_orf1	yellow	0.63067
TRINITY_DN41573_c0_g1_i1_orf1	yellow	0.6918
TRINITY_DN1633_c0_g1_i1_orf1	turquoise	0.88964
TRINITY_DN42824_c0_g1_i5_orf1	turquoise	0.90995
TRINITY_DN5408_c0_g1_i5_orf1	blue	0.88073
TRINITY_DN108819_c0_g1_i1_orf1	turquoise	0.98774
TRINITY_DN3430_c0_g1_i1_orf1	red	0.86544
TRINITY_DN15762_c0_g1_i2_orf1	turquoise	0.70222
TRINITY_DN4152_c0_g1_i1_orf1	turquoise	0.77378
TRINITY_DN31377_c0_g2_i1_orf1	blue	0.47361
TRINITY_DN867_c0_g1_i1_orf1	blue	0.88287
TRINITY_DN3300_c0_g2_i1_orf1	black	0.91567
TRINITY_DN1824_c0_g2_i2_orf1	turquoise	0.90879
TRINITY_DN511_c0_g2_i1_orf1	brown	0.9774
TRINITY_DN28729_c0_g1_i7_orf1	blue	0.55174
TRINITY_DN9492_c1_g1_i1_orf1	brown	0.92621
TRINITY_DN3627_c0_g1_i7_orf1	brown	0.40335
TRINITY_DN88640_c0_g1_i1_orf1	turquoise	0.89807
TRINITY_DN4151_c1_g1_i4_orf1	turquoise	0.78089
TRINITY_DN4463_c0_g1_i2_orf1	turquoise	0.65489
TRINITY_DN1732_c0_g1_i17_orf1	green	0.65639
TRINITY_DN970_c0_g1_i4_orf1	brown	0.96988
TRINITY_DN14743_c0_g1_i4_orf1	turquoise	0.58118
TRINITY_DN1445_c0_g2_i4_orf1	turquoise	0.81723
TRINITY_DN10658_c0_g1_i1_orf1	turquoise	0.74002
TRINITY_DN141352_c0_g1_i1_orf1	turquoise	0.94786
TRINITY_DN10831_c1_g1_i1_orf1	turquoise	0.89444
TRINITY_DN3332_c0_g1_i11_orf1	turquoise	0.96523
TRINITY_DN136358_c0_g1_i1_orf1	green	0.90058
TRINITY_DN5004_c0_g1_i2_orf1	red	0.81157
TRINITY_DN54387_c0_g1_i1_orf1	blue	0.55643
TRINITY_DN17446_c0_g1_i1_orf1	turquoise	0.95081
TRINITY_DN3598_c0_g1_i1_orf1	turquoise	0.97716
TRINITY_DN104586_c0_g1_i1_orf1	turquoise	0.9752
TRINITY_DN1318_c0_g1_i5_orf1	turquoise	0.98363
TRINITY_DN46372_c0_g1_i1_orf1	yellow	0.95922
TRINITY_DN21000_c0_g1_i1_orf1	turquoise	0.62331
TRINITY_DN10619_c0_g5_i7_orf1	red	0.77177
TRINITY_DN2584_c0_g1_i7_orf1	blue	0.93779
TRINITY_DN3433_c0_g1_i15_orf1	brown	0.95563
TRINITY_DN2049_c1_g1_i3_orf1	green	0.86487
TRINITY_DN98147_c0_g2_i1_orf1	blue	0.96122
TRINITY_DN2749_c0_g1_i4_orf1	turquoise	0.63146
TRINITY_DN38835_c0_g2_i1_orf1	green	0.56734
TRINITY_DN1421_c0_g1_i1_orf1	black	0.93539

TRINITY_DN3821_c1_g1_i7_orf1	brown	0.95189
TRINITY_DN10220_c1_g1_i7_orf1	black	0.92469
TRINITY_DN12823_c0_g1_i1_orf1	turquoise	0.63336
TRINITY_DN2343_c1_g1_i12_orf1	yellow	0.60721
TRINITY_DN1354_c0_g1_i6_orf1	turquoise	0.92333
TRINITY_DN49409_c0_g1_i2_orf1	turquoise	0.99265
TRINITY_DN20339_c0_g1_i3_orf1	red	0.86386
TRINITY_DN130_c0_g1_i7_orf1	turquoise	0.89496
TRINITY_DN3298_c0_g2_i4_orf1	yellow	0.74955
TRINITY_DN42082_c0_g2_i2_orfp1	turquoise	0.94457
TRINITY_DN1029_c0_g1_i1_orfp1	yellow	0.93213
TRINITY_DN129835_c0_g1_i2_orf1	blue	0.93272
TRINITY_DN24476_c0_g1_i1_orf1	turquoise	0.97797
TRINITY_DN2953_c1_g1_i11_orf1	turquoise	0.85581
TRINITY_DN19043_c0_g2_i1_orf1	blue	0.98774
TRINITY_DN8406_c0_g1_i4_orf1	turquoise	0.49319
TRINITY_DN32306_c0_g1_i3_orf1	turquoise	0.9081
TRINITY_DN38471_c0_g2_i1_orf1	grey	0.7106
TRINITY_DN147517_c0_g1_i1_orf1	turquoise	0.9611
TRINITY_DN66442_c0_g2_i3_orf1	turquoise	0.33571
TRINITY_DN3111_c0_g1_i5_orf1	turquoise	0.88662
TRINITY_DN48237_c0_g1_i5_orf1	turquoise	0.98759
TRINITY_DN106156_c1_g1_i1_orf1	green	0.98063
TRINITY_DN71494_c0_g1_i2_orf1	turquoise	0.78367
TRINITY_DN7992_c0_g1_i4_orf1	black	0.89033
TRINITY_DN327_c1_g1_i4_orf1	turquoise	0.99886
TRINITY_DN4708_c0_g1_i5_orf1	blue	0.48776
TRINITY_DN40281_c0_g2_i1_orf1	turquoise	0.89234
TRINITY_DN35277_c0_g1_i1_orf1	turquoise	0.94753
TRINITY_DN9740_c0_g1_i4_orf1	black	0.83758
TRINITY_DN4762_c0_g1_i2_orf1	turquoise	0.93019
TRINITY_DN4572_c0_g3_i1_orf1	blue	0.95847
TRINITY_DN799_c0_g1_i7_orf1	turquoise	0.93232
TRINITY_DN13330_c0_g1_i4_orf1	blue	0.66924
TRINITY_DN41736_c0_g2_i1_orf1	red	0.86748
TRINITY_DN5122_c0_g1_i3_orf1	green	0.89504
TRINITY_DN124300_c0_g1_i2_orf1	turquoise	0.94618
TRINITY_DN116951_c0_g3_i2_orf1	yellow	0.74479
TRINITY_DN25686_c0_g1_i4_orf1	red	0.85816
TRINITY_DN1260_c0_g2_i1_orf1	blue	0.50362
TRINITY_DN16385_c0_g1_i4_orf1	turquoise	0.74372
TRINITY_DN2701_c1_g1_i6_orf1	blue	0.78745
TRINITY_DN86956_c0_g5_i1_orf1	turquoise	0.96992
TRINITY_DN43355_c0_g1_i1_orf1	turquoise	0.83809
TRINITY_DN53847_c0_g1_i7_orf1	red	0.62858
TRINITY_DN5133_c0_g1_i7_orf1	turquoise	0.92155
TRINITY_DN111110_c0_g1_i1_orf1	yellow	0.84635
TRINITY_DN43611_c0_g1_i1_orf1	turquoise	0.99306
TRINITY_DN670_c0_g1_i3_orf1	turquoise	0.59585
TRINITY_DN6015_c1_g1_i3_orf1	green	0.93447
TRINITY_DN23474_c1_g1_i1_orf1	yellow	0.805
TRINITY_DN28299_c0_g1_i1_orf1	brown	0.92115
TRINITY_DN21539_c0_g1_i1_orf1	turquoise	0.88223
TRINITY_DN1391_c1_g2_i4_orf1	turquoise	0.8232
TRINITY_DN442_c0_g1_i10_orf1	turquoise	0.5244
TRINITY_DN42177_c0_g1_i4_orf1	yellow	0.71047
TRINITY_DN2722_c0_g1_i1_orf1	yellow	0.92124
TRINITY_DN16408_c0_g1_i1_orf1	turquoise	0.89669

TRINITY_DN18696_c0_g1_i1_orf1	turquoise	0.90197
TRINITY_DN5585_c0_g1_i4_orf1	brown	0.61459
TRINITY_DN1191_c0_g1_i4_orf1	turquoise	0.99128
TRINITY_DN710_c0_g1_i11_orfp1	brown	0.93769
TRINITY_DN38301_c0_g1_i2_orf1	turquoise	0.97299
TRINITY_DN13303_c0_g1_i6_orf1	black	0.93775
TRINITY_DN70236_c0_g1_i1_orf1	turquoise	0.30211
TRINITY_DN879_c0_g1_i2_orf1	turquoise	0.57079
TRINITY_DN20680_c0_g1_i5_orf1	blue	0.86334
TRINITY_DN6299_c0_g1_i1_orf1	turquoise	0.76928
TRINITY_DN3862_c0_g1_i7_orf1	turquoise	0.99593
TRINITY_DN712_c0_g2_i1_orf1	blue	0.98095
TRINITY_DN38835_c0_g3_i1_orf1	turquoise	0.64037
TRINITY_DN44557_c0_g2_i1_orf1	turquoise	0.94752
TRINITY_DN16516_c0_g1_i1_orf1	blue	0.73696
TRINITY_DN245_c0_g1_i4_orf1	brown	0.83727
TRINITY_DN6071_c0_g1_i1_orf1	turquoise	0.52921
TRINITY_DN8595_c0_g1_i3_orf1	brown	0.99112
TRINITY_DN62557_c0_g1_i1_orf1	brown	0.75006
TRINITY_DN6595_c1_g1_i6_orf1	black	0.64138
TRINITY_DN54275_c0_g1_i4_orf1	green	0.69468
TRINITY_DN25492_c0_g1_i1_orf1	yellow	0.71198
TRINITY_DN892_c0_g1_i9_orf1	red	0.82692
TRINITY_DN53238_c1_g1_i5_orf1	yellow	0.71695
TRINITY_DN10877_c0_g1_i1_orf1	brown	0.78145
TRINITY_DN10646_c0_g1_i2_orf1	blue	0.85125
TRINITY_DN1772_c1_g2_i1_orf1	brown	0.95266
TRINITY_DN13648_c0_g1_i6_orf1	blue	0.94501
TRINITY_DN4567_c0_g1_i5_orf1	turquoise	0.45533
TRINITY_DN30306_c0_g2_i1_orf1	brown	0.95211
TRINITY_DN8724_c0_g1_i5_orf1	turquoise	0.60907
TRINITY_DN32022_c0_g1_i1_orf1	turquoise	0.90626
TRINITY_DN59291_c0_g1_i1_orf1	red	0.92676
TRINITY_DN9406_c0_g1_i5_orf1	brown	0.53312
TRINITY_DN4401_c0_g2_i1_orf1	red	0.63393
TRINITY_DN10183_c0_g2_i3_orf1	red	0.73622
TRINITY_DN42171_c0_g1_i1_orf1	red	0.92733
TRINITY_DN40_c0_g1_i3_orf1	turquoise	0.98631
TRINITY_DN2642_c0_g1_i5_orf1	turquoise	0.83817
TRINITY_DN2483_c0_g1_i1_orf1	green	0.86316
TRINITY_DN2890_c0_g1_i2_orf1	yellow	0.8681
TRINITY_DN1935_c0_g1_i1_orf1	blue	0.94373
TRINITY_DN21285_c0_g1_i3_orf1	brown	0.87617
TRINITY_DN9_c0_g1_i7_orf1	brown	0.55304
TRINITY_DN84631_c0_g1_i1_orf1	black	0.4983
TRINITY_DN3949_c0_g1_i1_orf1	blue	0.84411
TRINITY_DN3063_c0_g1_i5_orf1	turquoise	0.94672
TRINITY_DN27_c0_g1_i1_orf1	turquoise	0.92826
TRINITY_DN23570_c0_g1_i2_orf1	turquoise	0.99069
TRINITY_DN19727_c0_g1_i7_orf1	turquoise	0.93598
TRINITY_DN21035_c0_g1_i14_orf1	turquoise	0.6579
TRINITY_DN28503_c0_g1_i6_orf1	yellow	0.88471
TRINITY_DN6406_c0_g1_i1_orf1	turquoise	0.8885
TRINITY_DN569_c0_g3_i12_orf1	blue	0.84911
TRINITY_DN20279_c0_g1_i1_orf1	turquoise	0.9683
TRINITY_DN132_c0_g2_i2_orf1	black	0.82615
TRINITY_DN213_c0_g1_i3_orf1	green	0.77889
TRINITY_DN2887_c0_g1_i1_orf1	yellow	0.85863

TRINITY_DN7247_c0_g1_i7_orf1	red	0.8591
TRINITY_DN2818_c0_g1_i2_orf1	black	0.94776
TRINITY_DN46_c0_g1_i2_orf1	turquoise	0.76324
TRINITY_DN12700_c0_g1_i7_orf1	grey	0.07338
TRINITY_DN51050_c0_g1_i3_orf1	turquoise	0.83664
TRINITY_DN294_c0_g1_i2_orf1	turquoise	0.69834
TRINITY_DN23732_c0_g1_i1_orf1	blue	0.87817
TRINITY_DN4816_c0_g2_i3_orf1	brown	0.962
TRINITY_DN21126_c0_g1_i1_orf1	turquoise	0.72121
TRINITY_DN22430_c0_g3_i1_orf1	turquoise	0.99132
TRINITY_DN12286_c1_g1_i2_orf1	blue	0.9087
TRINITY_DN12372_c0_g1_i4_orf1	turquoise	0.6929
TRINITY_DN2133_c0_g2_i1_orf1	brown	0.84019
TRINITY_DN110534_c0_g1_i3_orf1	red	0.90238
TRINITY_DN5757_c0_g1_i1_orf1	blue	0.94996
TRINITY_DN108122_c0_g1_i9_orf1	turquoise	0.92596
TRINITY_DN8012_c0_g1_i3_orf1	turquoise	0.87361
TRINITY_DN4451_c0_g1_i1_orf1	turquoise	0.89031
TRINITY_DN537_c0_g1_i1_orf1	blue	0.85873
TRINITY_DN1465_c0_g2_i1_orf1	turquoise	0.96571
TRINITY_DN22242_c0_g1_i1_orf1	brown	0.83024
TRINITY_DN8694_c1_g1_i4_orf1	black	0.80205
TRINITY_DN6710_c0_g1_i6_orf1	turquoise	0.75802
TRINITY_DN10030_c0_g1_i2_orf1	turquoise	0.96873
TRINITY_DN325_c0_g1_i15_orf1	black	0.73361
TRINITY_DN391_c1_g2_i1_orf1	turquoise	0.9793
TRINITY_DN5218_c0_g1_i4_orf1	turquoise	0.83692
TRINITY_DN5692_c0_g1_i4_orf1	yellow	0.60613
TRINITY_DN50875_c0_g1_i3_orf1	red	0.90597
TRINITY_DN56308_c0_g1_i2_orf1	green	0.99638
TRINITY_DN80424_c0_g1_i1_orf1	black	0.53642
TRINITY_DN45953_c0_g1_i1_orf1	turquoise	0.35532
TRINITY_DN54612_c0_g1_i3_orf1	brown	0.69572
TRINITY_DN2745_c0_g1_i4_orf1	brown	0.84815
TRINITY_DN4204_c0_g1_i1_orf1	brown	0.65304
TRINITY_DN19628_c1_g1_i1_orf1	turquoise	0.97879
TRINITY_DN6589_c0_g1_i2_orf1	red	0.57025
TRINITY_DN47731_c0_g1_i2_orf1	turquoise	0.92673
TRINITY_DN121_c0_g1_i9_orf1	turquoise	0.99063
TRINITY_DN14831_c0_g1_i9_orf1	black	0.8471
TRINITY_DN63389_c0_g1_i4_orf1	green	0.75919
TRINITY_DN64171_c0_g1_i1_orf1	turquoise	0.39089
TRINITY_DN3411_c0_g2_i1_orf1	turquoise	0.73594
TRINITY_DN135077_c0_g1_i1_orf1	yellow	0.83412
TRINITY_DN32687_c0_g1_i1_orf1	turquoise	0.3506
TRINITY_DN11655_c0_g1_i1_orf1	turquoise	0.96544
TRINITY_DN26355_c0_g1_i4_orf1	turquoise	0.86059
TRINITY_DN16122_c0_g1_i4_orf1	turquoise	0.93868
TRINITY_DN4262_c0_g1_i16_orf1	red	0.87079
TRINITY_DN11159_c0_g1_i5_orf1	blue	0.56496
TRINITY_DN9465_c0_g1_i4_orf1	blue	0.55754
TRINITY_DN44256_c0_g1_i1_orf1	red	0.92743
TRINITY_DN2563_c0_g1_i4_orf1	turquoise	0.82608
TRINITY_DN5873_c0_g4_i1_orf1	turquoise	0.86143
TRINITY_DN38274_c0_g1_i1_orf1	blue	0.96636
TRINITY_DN3791_c0_g1_i2_orf1	turquoise	0.94441
TRINITY_DN816_c0_g1_i3_orf1	turquoise	0.90291
TRINITY_DN9412_c0_g1_i1_orf1	blue	0.87429

TRINITY_DN1833_c0_g1_i5_orf1	blue	0.52655
TRINITY_DN18539_c0_g1_i1_orf1	blue	0.96977
TRINITY_DN12932_c0_g1_i1_orf1	brown	0.73842
TRINITY_DN343_c0_g1_i5_orf1	turquoise	0.45309
TRINITY_DN17003_c0_g1_i1_orf1	red	0.87612
TRINITY_DN8659_c0_g1_i1_orf1	red	0.98864
TRINITY_DN7183_c0_g1_i2_orf1	blue	0.82709
TRINITY_DN101682_c0_g1_i1_orf1	turquoise	0.84775
TRINITY_DN15904_c0_g1_i1_orf1	turquoise	0.67933
TRINITY_DN2885_c1_g1_i2_orf1	red	0.87217
TRINITY_DN108573_c0_g1_i1_orf1	turquoise	0.98202
TRINITY_DN78492_c0_g1_i1_orf1	turquoise	0.73922
TRINITY_DN41761_c0_g1_i4_orf1	blue	0.96265
TRINITY_DN1466_c0_g1_i4_orf1	green	0.75051
TRINITY_DN4795_c0_g1_i2_orf1	turquoise	0.76669
TRINITY_DN36817_c0_g1_i1_orf1	turquoise	0.62752
TRINITY_DN85412_c0_g1_i1_orf1	blue	0.85734
TRINITY_DN63914_c0_g1_i1_orf1	turquoise	0.97973
TRINITY_DN17655_c0_g1_i1_orf1	red	0.88495
TRINITY_DN6621_c0_g1_i1_orf1	yellow	0.98007
TRINITY_DN16084_c0_g1_i4_orf1	turquoise	0.53743
TRINITY_DN27114_c0_g1_i1_orf1	blue	0.79952
TRINITY_DN41108_c0_g1_i1_orf1	turquoise	0.61256
TRINITY_DN311_c0_g1_i4_orfp1	turquoise	0.95895
TRINITY_DN3647_c2_g1_i3_orf1	turquoise	0.83278
TRINITY_DN32479_c0_g1_i8_orf1	turquoise	0.91782
TRINITY_DN3177_c0_g1_i1_orf1	blue	0.86137
TRINITY_DN4757_c0_g1_i3_orf1	red	0.94494
TRINITY_DN5296_c0_g2_i1_orf1	red	0.6244
TRINITY_DN21494_c0_g1_i2_orf1	turquoise	0.97745
TRINITY_DN23069_c0_g2_i3_orf1	brown	0.8952
TRINITY_DN5080_c0_g1_i1_orf1	green	0.97589
TRINITY_DN16125_c0_g1_i3_orf1	yellow	0.72711
TRINITY_DN6185_c0_g1_i12_orf1	turquoise	0.73157
TRINITY_DN2110_c0_g1_i3_orf1	green	0.94925
TRINITY_DN3978_c0_g2_i1_orf1	blue	0.85218
TRINITY_DN9718_c0_g1_i7_orf1	green	0.92391
TRINITY_DN32420_c0_g1_i2_orf1	turquoise	0.61313
TRINITY_DN10785_c0_g1_i4_orf1	turquoise	0.87144
TRINITY_DN2224_c0_g1_i1_orf1	turquoise	0.72316
TRINITY_DN1691_c0_g1_i3_orf1	turquoise	0.53691
TRINITY_DN13093_c0_g1_i2_orf1	turquoise	0.78299
TRINITY_DN48851_c0_g1_i2_orf1	turquoise	0.67373
TRINITY_DN123139_c0_g1_i1_orfp1	turquoise	0.96359
TRINITY_DN9135_c0_g1_i4_orf1	yellow	0.68006
TRINITY_DN10871_c0_g2_i1_orf1	grey	-0.19838
TRINITY_DN23183_c0_g1_i2_orf1	black	0.82101
TRINITY_DN4866_c0_g1_i2_orf1	yellow	0.73477
TRINITY_DN33_c0_g1_i1_orf1	turquoise	0.92567
TRINITY_DN53684_c0_g1_i1_orf1	turquoise	0.98052
TRINITY_DN841_c0_g1_i8_orf1	turquoise	0.47113
TRINITY_DN7275_c0_g1_i14_orf1	grey	0.56398
TRINITY_DN52859_c0_g1_i4_orf1	grey	0.71289
TRINITY_DN18563_c2_g1_i1_orf1	blue	0.81914
TRINITY_DN661_c0_g2_i2_orf1	blue	0.96546
TRINITY_DN11327_c0_g1_i1_orf1	brown	0.66077
TRINITY_DN1978_c0_g1_i4_orf1	black	0.55665
TRINITY_DN26985_c0_g1_i5_orf1	blue	0.68352

TRINITY_DN8702_c0_g1_i1_orf1	brown	0.98701
TRINITY_DN1459_c1_g1_i1_orf1	turquoise	0.85285
TRINITY_DN15916_c0_g1_i1_orf1	turquoise	0.77438
TRINITY_DN45271_c0_g1_i1_orf1	blue	0.8451
TRINITY_DN9079_c1_g1_i1_orf1	brown	0.47337
TRINITY_DN28875_c0_g1_i1_orf1	brown	0.76614
TRINITY_DN667_c0_g1_i5_orf1	red	0.78919
TRINITY_DN75086_c0_g1_i5_orf1	black	0.94633
TRINITY_DN8136_c0_g1_i1_orf1	grey	-0.33423
TRINITY_DN37532_c0_g1_i1_orf1	red	0.90379
TRINITY_DN22018_c0_g1_i3_orf1	blue	0.75675
TRINITY_DN2457_c0_g1_i8_orf1	brown	0.86654
TRINITY_DN27885_c0_g1_i3_orf1	turquoise	0.76539
TRINITY_DN11639_c0_g1_i1_orf1	turquoise	0.98154
TRINITY_DN53760_c0_g1_i1_orf1	turquoise	0.31468
TRINITY_DN42854_c0_g3_i2_orf1	green	0.91863
TRINITY_DN5488_c0_g1_i5_orf1	green	0.91653
TRINITY_DN29934_c0_g1_i6_orf1	turquoise	0.88195
TRINITY_DN72541_c0_g1_i2_orf1	turquoise	0.71814
TRINITY_DN164_c0_g1_i11_orf1	turquoise	0.9357
TRINITY_DN1505_c0_g1_i1_orf1	turquoise	0.9773
TRINITY_DN1005_c0_g2_i1_orf1	red	0.48168
TRINITY_DN3299_c0_g1_i2_orf1	turquoise	0.91609
TRINITY_DN36199_c0_g1_i1_orf1	turquoise	0.99093
TRINITY_DN5907_c0_g1_i4_orf1	blue	0.98897
TRINITY_DN14705_c0_g2_i1_orf1	turquoise	0.77871
TRINITY_DN12106_c0_g1_i4_orf1	black	0.93267
TRINITY_DN22375_c0_g1_i4_orf1	green	0.85513
TRINITY_DN14183_c0_g1_i3_orf1	turquoise	0.85046
TRINITY_DN1628_c0_g1_i1_orf1	red	0.8375
TRINITY_DN244_c1_g1_i5_orf1	green	0.91237
TRINITY_DN45948_c1_g1_i1_orf1	black	0.96831
TRINITY_DN4025_c0_g1_i1_orf1	turquoise	0.96968
TRINITY_DN79657_c0_g1_i1_orf1	black	0.91706
TRINITY_DN6162_c1_g1_i1_orf1	black	0.95132
TRINITY_DN7336_c0_g1_i13_orf1	turquoise	0.95958
TRINITY_DN2062_c0_g1_i9_orf1	turquoise	0.96356
TRINITY_DN21341_c0_g1_i4_orf1	turquoise	0.97298
TRINITY_DN11868_c0_g1_i2_orf1	black	0.94021
TRINITY_DN32700_c0_g1_i2_orf1	red	0.83142
TRINITY_DN13094_c0_g1_i1_orf1	turquoise	0.64017
TRINITY_DN9000_c0_g2_i1_orf1	red	0.92833
TRINITY_DN15154_c0_g1_i5_orf1	turquoise	0.66934
TRINITY_DN38424_c0_g1_i1_orf1	turquoise	0.59139
TRINITY_DN17896_c0_g1_i1_orf1	yellow	0.83701
TRINITY_DN28039_c0_g1_i1_orf1	turquoise	0.40079
TRINITY_DN31851_c0_g1_i2_orf1	turquoise	0.74754
TRINITY_DN17368_c0_g1_i6_orf1	green	0.76835
TRINITY_DN38392_c0_g1_i1_orf1	green	0.49407
TRINITY_DN655_c0_g1_i3_orf1	turquoise	0.93239
TRINITY_DN2826_c0_g1_i7_orf1	turquoise	0.80064
TRINITY_DN5664_c0_g1_i1_orf1	blue	0.93166
TRINITY_DN4532_c0_g1_i1_orf1	turquoise	0.97086
TRINITY_DN140_c1_g1_i2_orf1	blue	0.86402
TRINITY_DN10889_c0_g1_i8_orf1	yellow	0.89821
TRINITY_DN19413_c0_g1_i2_orf1	turquoise	0.91998
TRINITY_DN8116_c0_g1_i1_orf1	turquoise	0.99231
TRINITY_DN12336_c0_g1_i1_orfp1	yellow	0.8426

TRINITY_DN90327_c0_g1_i1_orf1	blue	0.75309
TRINITY_DN26411_c0_g1_i2_orfp1	turquoise	0.87338
TRINITY_DN8290_c0_g1_i3_orf1	turquoise	0.79272
TRINITY_DN100208_c0_g1_i1_orf1	blue	0.56865
TRINITY_DN1557_c0_g1_i9_orf1	turquoise	0.96854
TRINITY_DN145448_c0_g1_i1_orf1	red	0.82085
TRINITY_DN4802_c0_g1_i4_orf1	brown	0.87409
TRINITY_DN15388_c0_g1_i5_orf1	turquoise	0.95404
TRINITY_DN10403_c0_g1_i1_orf1	brown	0.88841
TRINITY_DN4068_c0_g2_i4_orf1	brown	0.84214
TRINITY_DN19814_c0_g1_i4_orf1	turquoise	0.87245
TRINITY_DN21984_c0_g1_i6_orf1	turquoise	0.91702
TRINITY_DN9198_c0_g1_i4_orf1	turquoise	0.42396
TRINITY_DN6572_c0_g1_i2_orf1	turquoise	0.85703
TRINITY_DN1656_c2_g1_i5_orf1	turquoise	0.75293
TRINITY_DN5064_c0_g1_i4_orf1	blue	0.83231
TRINITY_DN804_c0_g1_i7_orf1	yellow	0.91245
TRINITY_DN235_c0_g1_i2_orf1	yellow	0.85602
TRINITY_DN2709_c0_g1_i4_orf1	turquoise	0.84635
TRINITY_DN291_c0_g1_i2_orf1	blue	0.85871
TRINITY_DN31119_c0_g1_i1_orf1	turquoise	0.84498
TRINITY_DN257_c0_g1_i7_orf1	turquoise	0.82467
TRINITY_DN14313_c0_g1_i1_orf1	turquoise	0.98626
TRINITY_DN13999_c0_g1_i4_orf1	turquoise	0.40143
TRINITY_DN2904_c0_g1_i4_orf1	blue	0.73447
TRINITY_DN11649_c0_g1_i4_orf1	pink	0.92865
TRINITY_DN42646_c0_g2_i1_orf1	turquoise	0.93678
TRINITY_DN130069_c0_g6_i1_orf1	blue	0.67282
TRINITY_DN1875_c0_g1_i1_orf1	turquoise	0.81413
TRINITY_DN1166_c0_g3_i4_orf1	green	0.74043
TRINITY_DN15965_c0_g1_i1_orf1	turquoise	0.97497
TRINITY_DN19942_c0_g1_i2_orf1	turquoise	0.92492
TRINITY_DN4469_c0_g1_i2_orf1	turquoise	0.92662
TRINITY_DN9991_c0_g1_i4_orf1	brown	0.78861
TRINITY_DN3483_c0_g1_i5_orf1	green	0.93174
TRINITY_DN5433_c0_g1_i5_orf1	black	0.76956
TRINITY_DN5458_c1_g1_i9_orf1	black	0.87556
TRINITY_DN5121_c0_g1_i1_orf1	turquoise	0.62327
TRINITY_DN51934_c0_g2_i1_orf1	turquoise	0.90376
TRINITY_DN81803_c0_g2_i1_orf1	turquoise	0.98874
TRINITY_DN11396_c0_g1_i1_orf1	turquoise	0.94198
TRINITY_DN69691_c0_g2_i1_orf1	green	0.57135
TRINITY_DN27264_c0_g1_i1_orf1	blue	0.92509
TRINITY_DN2997_c0_g1_i6_orf1	turquoise	0.86437
TRINITY_DN5055_c0_g1_i12_orf1	black	0.63506
TRINITY_DN11263_c0_g1_i5_orf1	brown	0.71099
TRINITY_DN49038_c0_g4_i1_orf1	brown	0.98273
TRINITY_DN3457_c0_g1_i4_orf1	turquoise	0.78793
TRINITY_DN12806_c0_g2_i1_orf1	turquoise	0.81393
TRINITY_DN43841_c0_g1_i1_orf1	turquoise	0.73894
TRINITY_DN7316_c0_g2_i1_orf1	blue	0.54147
TRINITY_DN8116_c0_g1_i2_orf1	turquoise	0.99072
TRINITY_DN7388_c0_g1_i7_orf1	black	0.68049
TRINITY_DN1860_c0_g1_i2_orf1	turquoise	0.672
TRINITY_DN8926_c0_g1_i4_orf1	black	0.74906
TRINITY_DN38211_c0_g1_i1_orf1	red	0.863
TRINITY_DN3322_c0_g1_i2_orf1	turquoise	0.42566
TRINITY_DN14035_c0_g1_i1_orf1	turquoise	0.96476

TRINITY_DN4770_c0_g1_i4_orf1	turquoise	0.85183
TRINITY_DN2802_c1_g1_i1_orf1	red	0.88895
TRINITY_DN24350_c0_g1_i1_orf1	red	0.6725
TRINITY_DN10644_c0_g1_i2_orf1	turquoise	0.92795
TRINITY_DN42719_c0_g1_i1_orf1	blue	0.9586
TRINITY_DN1572_c0_g1_i6_orf1	turquoise	0.98877
TRINITY_DN905_c0_g1_i4_orf1	turquoise	0.98006
TRINITY_DN11121_c0_g1_i5_orf1	turquoise	0.93759
TRINITY_DN9486_c1_g1_i7_orfp1	red	0.60068
TRINITY_DN44070_c0_g2_i2_orf1	red	0.58784
TRINITY_DN2101_c0_g1_i6_orf1	blue	0.93998
TRINITY_DN23429_c0_g2_i1_orf1	yellow	0.96066
TRINITY_DN25976_c0_g1_i4_orf1	green	0.83673
TRINITY_DN8107_c0_g1_i1_orf1	turquoise	0.34048
TRINITY_DN31417_c0_g1_i3_orf1	blue	0.98967
TRINITY_DN8771_c0_g2_i1_orf1	grey	0.86612
TRINITY_DN20749_c0_g1_i3_orf1	turquoise	0.97479
TRINITY_DN32780_c0_g1_i2_orf1	red	0.5115
TRINITY_DN13435_c0_g1_i1_orf1	blue	0.38235
TRINITY_DN5811_c0_g1_i4_orf1	turquoise	0.42313
TRINITY_DN8024_c0_g1_i6_orf1	turquoise	0.52077
TRINITY_DN7590_c0_g1_i4_orf1	green	0.8493
TRINITY_DN467_c0_g3_i1_orf1	blue	0.89825
TRINITY_DN21971_c0_g1_i4_orf1	turquoise	0.87228
TRINITY_DN36496_c0_g1_i1_orf1	turquoise	0.55625
TRINITY_DN107617_c3_g1_i1_orf1	turquoise	0.96252
TRINITY_DN56110_c0_g1_i1_orf1	turquoise	0.99044
TRINITY_DN6205_c0_g1_i1_orf1	green	0.84866
TRINITY_DN3401_c0_g1_i1_orf1	turquoise	0.96279
TRINITY_DN1334_c0_g1_i2_orf1	turquoise	0.91052
TRINITY_DN5578_c0_g1_i4_orf1	turquoise	0.92906
TRINITY_DN40562_c0_g2_i1_orf1	turquoise	0.8767
TRINITY_DN4785_c0_g2_i1_orf1	blue	0.83185
TRINITY_DN2031_c11_g1_i2_orfp1	turquoise	0.43113
TRINITY_DN4272_c0_g1_i1_orf1	turquoise	0.92672
TRINITY_DN11612_c0_g3_i1_orf1	turquoise	0.91998
TRINITY_DN46633_c0_g1_i4_orf1	turquoise	0.34793
TRINITY_DN36262_c0_g1_i1_orf1	turquoise	0.98584
TRINITY_DN6556_c0_g1_i7_orf1	turquoise	0.92847
TRINITY_DN7064_c0_g1_i6_orf1	turquoise	0.74343
TRINITY_DN23838_c0_g1_i4_orf1	grey	0.17172
TRINITY_DN3257_c0_g1_i4_orf1	brown	0.94917
TRINITY_DN2089_c0_g1_i5_orf1	turquoise	0.92097
TRINITY_DN44285_c0_g1_i1_orf1	green	0.43305
TRINITY_DN2930_c0_g1_i8_orf1	turquoise	0.83622
TRINITY_DN207_c0_g2_i3_orf1	turquoise	0.96459
TRINITY_DN3532_c0_g1_i12_orf1	yellow	0.82276
TRINITY_DN21570_c0_g1_i1_orf1	turquoise	0.40887
TRINITY_DN18036_c0_g1_i7_orf1	turquoise	0.99385
TRINITY_DN145227_c0_g1_i1_orf1	turquoise	0.67215
TRINITY_DN4631_c0_g1_i7_orf1	turquoise	0.96992
TRINITY_DN2716_c0_g2_i1_orf1	turquoise	0.94165
TRINITY_DN54205_c0_g1_i1_orf1	brown	0.96304
TRINITY_DN3244_c0_g1_i4_orf1	turquoise	0.791
TRINITY_DN1494_c0_g2_i1_orf1	turquoise	0.95477
TRINITY_DN56250_c0_g1_i7_orf1	turquoise	0.58242
TRINITY_DN5421_c0_g1_i1_orf1	green	0.91855
TRINITY_DN2876_c0_g1_i5_orf1	turquoise	0.80617

TRINITY_DN10071_c0_g1_i2_orf1	turquoise	0.62945
TRINITY_DN4822_c0_g1_i9_orf1	yellow	0.60343
TRINITY_DN33763_c0_g1_i1_orf1	brown	0.72815
TRINITY_DN7647_c0_g1_i4_orf1	turquoise	0.86629
TRINITY_DN87170_c0_g1_i3_orf1	red	0.92448
TRINITY_DN271_c0_g2_i6_orf1	turquoise	0.93085
TRINITY_DN44491_c0_g1_i12_orf1	brown	0.8374
TRINITY_DN4434_c0_g1_i7_orf1	turquoise	0.98436
TRINITY_DN5976_c0_g1_i1_orf1	turquoise	0.95677
TRINITY_DN9090_c0_g1_i9_orf1	green	0.93941
TRINITY_DN33408_c0_g1_i1_orf1	grey	0.58695
TRINITY_DN104_c0_g1_i4_orf1	blue	0.91546
TRINITY_DN131471_c0_g1_i1_orf1	green	0.87582
TRINITY_DN4679_c0_g2_i13_orf1	black	0.79642
TRINITY_DN1652_c0_g1_i12_orf1	turquoise	0.78439
TRINITY_DN105901_c0_g1_i2_orfp1	turquoise	0.92792
TRINITY_DN62184_c1_g1_i1_orf1	green	0.81036
TRINITY_DN131264_c0_g1_i2_orf1	blue	0.75491
TRINITY_DN15222_c0_g1_i4_orf1	blue	0.94818
TRINITY_DN31433_c0_g1_i1_orf1	turquoise	0.95976
TRINITY_DN7778_c0_g1_i1_orf1	yellow	0.89925
TRINITY_DN25345_c0_g1_i1_orf1	blue	0.88803
TRINITY_DN740_c0_g1_i1_orf1	turquoise	0.98906
TRINITY_DN4189_c0_g1_i4_orf1	green	0.57481
TRINITY_DN10118_c0_g1_i4_orf1	yellow	0.87353
TRINITY_DN42373_c0_g4_i1_orf1	turquoise	0.7879
TRINITY_DN85004_c0_g1_i1_orf1	yellow	0.89647
TRINITY_DN80134_c0_g1_i1_orf1	blue	0.27685
TRINITY_DN195_c4_g1_i1_orf1	turquoise	0.54835
TRINITY_DN57856_c0_g2_i1_orf1	yellow	0.89944
TRINITY_DN5653_c0_g1_i4_orf1	turquoise	0.8443
TRINITY_DN14269_c0_g1_i5_orf1	brown	0.6032
TRINITY_DN7770_c0_g1_i4_orf1	turquoise	0.95325
TRINITY_DN2167_c0_g1_i6_orf1	turquoise	0.95801
TRINITY_DN2778_c0_g1_i5_orf1	turquoise	0.94203
TRINITY_DN30273_c1_g1_i1_orf1	red	0.90234
TRINITY_DN141381_c0_g1_i1_orf1	turquoise	0.51301
TRINITY_DN106730_c0_g1_i1_orf1	turquoise	0.95985
TRINITY_DN5129_c0_g3_i3_orf1	turquoise	0.9689
TRINITY_DN33452_c0_g1_i1_orf1	turquoise	0.78983
TRINITY_DN2796_c0_g1_i28_orf1	black	0.88996
TRINITY_DN2618_c0_g1_i3_orf1	brown	0.91942
TRINITY_DN4410_c0_g1_i1_orf1	blue	0.95012
TRINITY_DN98995_c0_g1_i2_orf1	yellow	0.90363
TRINITY_DN8258_c0_g1_i5_orf1	brown	0.9472
TRINITY_DN69871_c0_g1_i1_orf1	yellow	0.82341
TRINITY_DN81031_c0_g1_i1_orf1	green	0.98441
TRINITY_DN14409_c0_g1_i1_orf1	yellow	0.63926
TRINITY_DN51498_c0_g1_i1_orf1	brown	0.41851
TRINITY_DN5149_c0_g1_i14_orfp1	yellow	0.6234
TRINITY_DN76333_c0_g1_i2_orf1	yellow	0.93103
TRINITY_DN23183_c1_g1_i2_orf1	blue	0.83448
TRINITY_DN10871_c0_g1_i3_orf1	turquoise	0.70321
TRINITY_DN650_c0_g1_i3_orf1	red	0.90617
TRINITY_DN2416_c0_g1_i5_orf1	blue	0.9004
TRINITY_DN607_c0_g1_i16_orf1	turquoise	0.88774
TRINITY_DN3393_c0_g2_i1_orf1	turquoise	0.96368
TRINITY_DN755_c0_g1_i3_orf1	turquoise	0.97898

TRINITY_DN7040_c0_g1_i4_orf1	green	0.95222
TRINITY_DN83948_c0_g1_i3_orf1	turquoise	0.88668
TRINITY_DN16539_c0_g1_i7_orf1	turquoise	0.73448
TRINITY_DN86090_c0_g1_i1_orf1	turquoise	0.97627
TRINITY_DN2304_c0_g1_i4_orf1	turquoise	0.88696
TRINITY_DN5670_c0_g1_i2_orf1	turquoise	0.60413
TRINITY_DN11448_c0_g1_i15_orf1	yellow	0.95823
TRINITY_DN5925_c0_g1_i5_orf1	turquoise	0.98169
TRINITY_DN14185_c0_g1_i1_orf1	brown	0.91731
TRINITY_DN60787_c0_g1_i5_orf1	brown	0.94924
TRINITY_DN8046_c0_g1_i4_orf1	yellow	0.20198
TRINITY_DN5933_c0_g1_i1_orf1	yellow	0.77019
TRINITY_DN11799_c0_g1_i4_orf1	turquoise	0.97136
TRINITY_DN33885_c0_g1_i1_orf1	brown	0.8284
TRINITY_DN14855_c0_g1_i1_orf1	turquoise	0.70288
TRINITY_DN12101_c0_g1_i2_orf1	turquoise	0.87548
TRINITY_DN11409_c0_g1_i4_orf1	turquoise	0.97156
TRINITY_DN59829_c0_g1_i1_orf1	red	0.98582
TRINITY_DN66596_c0_g1_i1_orf1	turquoise	0.95954
TRINITY_DN95850_c0_g1_i1_orf1	black	0.73167
TRINITY_DN15376_c0_g1_i1_orf1	turquoise	0.96077
TRINITY_DN5628_c0_g1_i5_orf1	yellow	0.93316
TRINITY_DN91877_c0_g1_i1_orf1	turquoise	0.44056
TRINITY_DN12545_c0_g1_i7_orf1	brown	0.86236
TRINITY_DN39725_c0_g1_i4_orf1	blue	0.32314
TRINITY_DN657_c0_g1_i2_orf1	turquoise	0.98895
TRINITY_DN11680_c0_g1_i1_orf1	brown	0.91352
TRINITY_DN44073_c0_g1_i3_orf1	blue	0.83012
TRINITY_DN18222_c0_g1_i4_orf1	green	0.54715
TRINITY_DN2815_c0_g1_i3_orf1	turquoise	0.98791
TRINITY_DN17031_c0_g1_i1_orf1	brown	0.93532
TRINITY_DN745_c7_g1_i1_orf1	green	0.97697
TRINITY_DN27833_c0_g2_i1_orf1	brown	0.7229
TRINITY_DN17255_c0_g1_i9_orf1	grey	0.39273
TRINITY_DN6685_c0_g1_i8_orf1	turquoise	0.97027
TRINITY_DN321_c0_g1_i1_orf1	blue	0.95914
TRINITY_DN9242_c0_g1_i1_orf1	yellow	0.59722
TRINITY_DN57496_c0_g1_i1_orf1	turquoise	0.83876
TRINITY_DN22871_c0_g2_i1_orf1	turquoise	0.81001
TRINITY_DN2202_c0_g1_i9_orf1	green	0.91462
TRINITY_DN2181_c1_g1_i8_orf1	turquoise	0.68772
TRINITY_DN14134_c0_g2_i3_orf1	turquoise	0.67223
TRINITY_DN3738_c0_g1_i5_orf1	green	0.80252
TRINITY_DN30208_c0_g1_i3_orf1	yellow	0.99366
TRINITY_DN766_c0_g1_i1_orf1	turquoise	0.72321
TRINITY_DN20322_c0_g1_i1_orf1	blue	0.64921
TRINITY_DN2676_c0_g1_i2_orf1	blue	0.76288
TRINITY_DN17376_c0_g1_i2_orf1	turquoise	0.95928
TRINITY_DN25373_c0_g1_i1_orf1	turquoise	0.94013
TRINITY_DN1255_c0_g1_i17_orf1	brown	0.93788
TRINITY_DN23266_c0_g2_i1_orf1	turquoise	0.99399
TRINITY_DN31431_c0_g1_i1_orf1	turquoise	0.61133
TRINITY_DN3158_c0_g1_i5_orf1	black	0.92542
TRINITY_DN24310_c0_g1_i2_orf1	green	0.88687
TRINITY_DN77559_c0_g1_i1_orf1	brown	0.9075
TRINITY_DN48838_c0_g1_i6_orf1	turquoise	0.88188
TRINITY_DN15667_c0_g1_i2_orf1	turquoise	0.97428
TRINITY_DN4813_c0_g1_i5_orf1	black	0.59471

TRINITY_DN9156_c0_g1_i1_orf1	turquoise	0.93119
TRINITY_DN4596_c0_g1_i14_orf1	black	0.69672
TRINITY_DN8674_c0_g2_i1_orf1	blue	0.82137
TRINITY_DN109931_c0_g1_i1_orf1	yellow	0.972
TRINITY_DN58636_c0_g1_i1_orf1	turquoise	0.96247
TRINITY_DN1024_c0_g4_i1_orf1	brown	0.97311
TRINITY_DN2784_c0_g1_i3_orf1	green	0.87993
TRINITY_DN18374_c0_g1_i1_orf1	brown	0.87075
TRINITY_DN15136_c0_g1_i2_orf1	yellow	0.95223
TRINITY_DN1230_c1_g1_i5_orf1	brown	0.95636
TRINITY_DN46715_c0_g1_i1_orf1	pink	0.81289
TRINITY_DN146264_c0_g1_i1_orf1	turquoise	0.92057
TRINITY_DN10502_c0_g1_i4_orf1	turquoise	0.85395
TRINITY_DN5149_c0_g1_i12_orfp1	yellow	0.84482
TRINITY_DN6243_c0_g1_i5_orf1	black	0.96163
TRINITY_DN2097_c1_g1_i1_orf1	blue	0.81837
TRINITY_DN89613_c0_g1_i13_orf1	turquoise	0.67556
TRINITY_DN4950_c0_g1_i2_orf1	turquoise	0.84047
TRINITY_DN1749_c0_g2_i2_orf1	blue	0.95405
TRINITY_DN6974_c0_g2_i1_orf1	blue	0.79939
TRINITY_DN109943_c0_g1_i1_orf1	yellow	0.94603
TRINITY_DN91198_c0_g2_i1_orf1	turquoise	0.63555
TRINITY_DN9717_c0_g2_i1_orf1	green	0.8871
TRINITY_DN144956_c0_g1_i1_orf1	turquoise	0.94065
TRINITY_DN5462_c0_g2_i1_orf1	black	0.52057
TRINITY_DN3082_c1_g1_i7_orf1	turquoise	0.98571
TRINITY_DN10090_c0_g1_i1_orf1	green	0.88518
TRINITY_DN72056_c0_g1_i1_orf1	turquoise	0.97252
TRINITY_DN18031_c0_g1_i1_orf1	brown	0.5502
TRINITY_DN3712_c0_g1_i1_orf1	black	0.88907
TRINITY_DN825_c0_g1_i18_orfp1	yellow	0.89033
TRINITY_DN19687_c0_g1_i1_orf1	turquoise	0.76426
TRINITY_DN47_c0_g1_i2_orf1	turquoise	0.93355
TRINITY_DN48602_c0_g1_i6_orf1	black	0.70813
TRINITY_DN97589_c0_g1_i3_orf1	turquoise	0.87209
TRINITY_DN2821_c0_g1_i1_orf1	pink	0.76819
TRINITY_DN2205_c0_g1_i3_orf1	green	0.91723
TRINITY_DN26429_c0_g1_i4_orf1	turquoise	0.90551
TRINITY_DN11125_c0_g1_i1_orf1	turquoise	0.47162
TRINITY_DN144258_c0_g1_i1_orf1	red	0.89067
TRINITY_DN2529_c0_g1_i3_orf1	brown	0.86374
TRINITY_DN116972_c0_g1_i1_orf1	yellow	0.82448
TRINITY_DN9207_c0_g1_i1_orf1	turquoise	0.77199
TRINITY_DN28622_c0_g1_i1_orf1	red	0.94589
TRINITY_DN6933_c0_g1_i2_orf1	turquoise	0.98824
TRINITY_DN699_c0_g1_i5_orf1	brown	0.69796
TRINITY_DN12133_c0_g2_i1_orf1	turquoise	0.96103
TRINITY_DN5507_c0_g1_i1_orf1	pink	0.83889
TRINITY_DN2593_c0_g2_i1_orf1	turquoise	0.89088
TRINITY_DN29541_c0_g1_i1_orf1	green	0.63316
TRINITY_DN49786_c0_g1_i1_orf1	turquoise	0.33333
TRINITY_DN136906_c0_g1_i1_orf1	turquoise	0.74995
TRINITY_DN3324_c0_g1_i3_orf1	yellow	0.26652
TRINITY_DN117844_c0_g1_i1_orf1	brown	0.95714
TRINITY_DN1888_c0_g2_i1_orf1	turquoise	0.83423
TRINITY_DN1956_c1_g1_i5_orf1	turquoise	0.98508
TRINITY_DN26994_c1_g1_i6_orf1	green	0.54137
TRINITY_DN1196_c0_g1_i4_orf1	blue	0.85167

TRINITY_DN15988_c0_g1_i1_orf1	turquoise	0.49888
TRINITY_DN12666_c0_g1_i2_orf1	brown	0.84191
TRINITY_DN16343_c0_g1_i6_orf1	turquoise	0.98117
TRINITY_DN5852_c0_g1_i6_orf1	brown	0.43788
TRINITY_DN1753_c1_g1_i8_orf1	turquoise	0.59383
TRINITY_DN19058_c1_g1_i1_orf1	blue	0.97287
TRINITY_DN106038_c0_g1_i1_orf1	turquoise	0.92652
TRINITY_DN10796_c0_g2_i1_orf1	turquoise	0.97823
TRINITY_DN18756_c0_g1_i6_orf1	blue	0.70121
TRINITY_DN31520_c1_g1_i1_orf1	turquoise	0.76112
TRINITY_DN5281_c0_g2_i3_orf1	turquoise	0.56748
TRINITY_DN2783_c1_g1_i2_orf1	turquoise	0.91026
TRINITY_DN146544_c0_g1_i1_orf1	turquoise	0.97114
TRINITY_DN2706_c0_g1_i3_orf1	brown	0.94261
TRINITY_DN779_c0_g1_i12_orf1	yellow	0.89999
TRINITY_DN10297_c0_g1_i1_orf1	turquoise	0.60304
TRINITY_DN74069_c0_g1_i1_orf1	red	0.83856
TRINITY_DN112706_c0_g1_i2_orf1	turquoise	0.4265
TRINITY_DN16605_c0_g1_i3_orf1	red	0.77667
TRINITY_DN16487_c0_g1_i1_orf1	turquoise	0.82801
TRINITY_DN46778_c0_g1_i2_orf1	turquoise	0.92897
TRINITY_DN31118_c0_g1_i1_orf1	yellow	0.93845
TRINITY_DN16145_c0_g1_i12_orf1	blue	0.76442
TRINITY_DN17845_c0_g1_i3_orf1	brown	0.82973
TRINITY_DN1738_c0_g1_i5_orf1	green	0.85058
TRINITY_DN10336_c0_g1_i9_orf1	blue	0.85526
TRINITY_DN12320_c0_g1_i1_orf1	turquoise	0.82156
TRINITY_DN21218_c0_g1_i4_orf1	turquoise	0.77195
TRINITY_DN57348_c0_g1_i4_orf1	blue	0.86271
TRINITY_DN2299_c0_g1_i3_orf1	yellow	0.76694
TRINITY_DN5235_c0_g1_i7_orf1	red	0.93427
TRINITY_DN124654_c0_g1_i1_orf1	green	0.90603
TRINITY_DN10287_c0_g1_i1_orf1	turquoise	0.78863
TRINITY_DN14730_c0_g1_i7_orf1	turquoise	0.97115
TRINITY_DN48983_c0_g1_i2_orf1	turquoise	0.68413
TRINITY_DN9002_c0_g1_i1_orf1	turquoise	0.95935
TRINITY_DN6239_c0_g1_i1_orf1	red	0.89124
TRINITY_DN1173_c0_g1_i12_orf1	turquoise	0.76457
TRINITY_DN15291_c0_g1_i11_orf1	blue	0.7568
TRINITY_DN8173_c0_g1_i3_orf1	turquoise	0.94656
TRINITY_DN64719_c0_g1_i2_orfp1	yellow	0.85747
TRINITY_DN1038_c1_g1_i3_orf1	yellow	0.83738
TRINITY_DN3209_c0_g1_i1_orf1	red	0.49775
TRINITY_DN4036_c0_g2_i1_orf1	turquoise	0.92852
TRINITY_DN536_c0_g1_i7_orf1	turquoise	0.95935
TRINITY_DN129207_c0_g1_i1_orf1	turquoise	0.90289
TRINITY_DN754_c1_g1_i8_orf1	turquoise	0.93932
TRINITY_DN11698_c0_g1_i1_orf1	brown	0.965
TRINITY_DN40211_c0_g1_i1_orf1	turquoise	0.76694
TRINITY_DN1575_c0_g1_i7_orf1	brown	0.824
TRINITY_DN11388_c0_g1_i4_orf1	yellow	0.79296
TRINITY_DN29120_c0_g1_i6_orf1	turquoise	0.97729
TRINITY_DN4817_c0_g1_i4_orf1	black	0.88592
TRINITY_DN33248_c0_g1_i1_orf1	turquoise	0.9804
TRINITY_DN79803_c0_g1_i7_orf1	red	0.88736
TRINITY_DN4747_c0_g1_i4_orf1	turquoise	0.93237
TRINITY_DN5666_c0_g1_i2_orf1	red	0.72161
TRINITY_DN3616_c0_g2_i2_orf1	brown	0.90773

TRINITY_DN2455_c0_g1_i12_orf1	blue	0.63922
TRINITY_DN5081_c0_g1_i5_orf1	brown	0.93331
TRINITY_DN11823_c1_g1_i2_orf1	yellow	0.72687
TRINITY_DN1404_c0_g1_i6_orf1	yellow	0.88157
TRINITY_DN8133_c0_g1_i4_orf1	red	0.79419
TRINITY_DN34432_c0_g1_i1_orf1	turquoise	0.91776
TRINITY_DN44517_c0_g1_i4_orf1	brown	0.94185
TRINITY_DN2825_c0_g1_i3_orf1	turquoise	0.9782
TRINITY_DN15624_c0_g1_i1_orf1	turquoise	0.94738
TRINITY_DN400_c0_g1_i1_orf1	brown	0.91782
TRINITY_DN11297_c0_g1_i1_orf1	turquoise	0.91726
TRINITY_DN467_c9_g1_i2_orf1	turquoise	0.57647
TRINITY_DN43656_c0_g1_i1_orf1	turquoise	0.94463
TRINITY_DN4991_c0_g1_i1_orf1	yellow	0.67083
TRINITY_DN12858_c0_g1_i5_orf1	red	0.93291
TRINITY_DN36581_c0_g1_i5_orf1	yellow	0.6665
TRINITY_DN276_c0_g1_i2_orf1	blue	0.89522
TRINITY_DN50676_c0_g1_i1_orf1	turquoise	0.67575
TRINITY_DN34087_c0_g1_i4_orf1	green	0.67973
TRINITY_DN5768_c0_g1_i2_orf1	yellow	0.83459
TRINITY_DN1848_c0_g1_i2_orf1	green	0.72565
TRINITY_DN29414_c1_g2_i1_orf1	green	0.95395
TRINITY_DN18538_c0_g3_i1_orf1	green	0.83824
TRINITY_DN4835_c0_g1_i2_orf1	red	0.94742
TRINITY_DN69307_c0_g1_i6_orf1	brown	0.8648
TRINITY_DN9437_c0_g1_i1_orf1	turquoise	0.85057
TRINITY_DN23798_c0_g1_i1_orf1	turquoise	0.34605
TRINITY_DN11448_c0_g1_i11_orf1	yellow	0.9021
TRINITY_DN166_c0_g1_i4_orf1	blue	0.82029
TRINITY_DN3275_c0_g1_i4_orf1	green	0.62344
TRINITY_DN50820_c0_g1_i2_orf1	turquoise	0.73082
TRINITY_DN1198_c0_g1_i1_orf1	turquoise	0.78377
TRINITY_DN5497_c0_g1_i6_orf1	brown	0.86575
TRINITY_DN8598_c0_g1_i2_orf1	turquoise	0.95215
TRINITY_DN23783_c0_g2_i1_orf1	turquoise	0.84686
TRINITY_DN14475_c0_g1_i1_orf1	turquoise	0.83757
TRINITY_DN13602_c0_g1_i4_orf1	brown	0.7522
TRINITY_DN94337_c0_g1_i1_orf1	turquoise	0.90744
TRINITY_DN636_c1_g1_i9_orf1	green	0.92364
TRINITY_DN4497_c2_g1_i3_orf1	brown	0.96756
TRINITY_DN5099_c0_g1_i3_orf1	brown	0.96039
TRINITY_DN31348_c0_g1_i1_orf1	brown	0.91792
TRINITY_DN13626_c0_g2_i1_orf1	blue	0.66202
TRINITY_DN3521_c0_g2_i1_orf1	blue	0.76201
TRINITY_DN50743_c0_g1_i1_orf1	brown	0.71096
TRINITY_DN52244_c1_g1_i1_orf1	black	0.25361
TRINITY_DN35582_c0_g1_i1_orf1	yellow	0.77024
TRINITY_DN8979_c0_g1_i5_orf1	black	0.90189
TRINITY_DN37538_c0_g2_i1_orf1	turquoise	0.9837
TRINITY_DN2968_c0_g1_i3_orf1	blue	0.81724
TRINITY_DN31342_c2_g2_i1_orf1	black	0.65195
TRINITY_DN69691_c0_g1_i1_orf1	green	0.51657
TRINITY_DN802_c0_g1_i2_orf1	red	0.89005
TRINITY_DN34166_c0_g1_i1_orf1	black	0.8918
TRINITY_DN6813_c1_g1_i1_orf1	brown	0.87327
TRINITY_DN3017_c0_g1_i6_orf1	blue	0.74225
TRINITY_DN43667_c0_g1_i1_orf1	green	0.83712
TRINITY_DN4724_c0_g1_i4_orf1	yellow	0.84803

TRINITY_DN42159_c0_g1_i6_orf1	green	0.7049
TRINITY_DN95713_c0_g1_i1_orf1	turquoise	0.83717
TRINITY_DN1103_c0_g1_i15_orf1	yellow	0.7128
TRINITY_DN12951_c1_g2_i2_orf1	black	0.69164
TRINITY_DN125441_c0_g1_i5_orf1	black	0.95974
TRINITY_DN54134_c0_g1_i1_orf1	turquoise	0.85964
TRINITY_DN2356_c2_g1_i6_orf1	turquoise	0.8227
TRINITY_DN754_c1_g1_i6_orf1	turquoise	0.9741
TRINITY_DN145666_c0_g1_i1_orf1	turquoise	0.91498
TRINITY_DN59804_c0_g1_i1_orf1	blue	0.49109
TRINITY_DN7075_c0_g2_i1_orf1	brown	0.90039
TRINITY_DN30510_c0_g1_i6_orf1	brown	0.92007
TRINITY_DN22654_c0_g2_i4_orf1	turquoise	0.87896
TRINITY_DN47151_c0_g1_i1_orf1	yellow	0.83943
TRINITY_DN14684_c0_g2_i1_orf1	blue	0.93526
TRINITY_DN67649_c0_g1_i1_orf1	red	0.96621
TRINITY_DN95_c0_g1_i5_orf1	blue	0.58365
TRINITY_DN13186_c0_g1_i1_orf1	turquoise	0.88175
TRINITY_DN27247_c0_g2_i1_orfp1	brown	0.65699
TRINITY_DN38225_c0_g2_i1_orf1	brown	0.89086
TRINITY_DN14944_c0_g1_i7_orf1	red	0.78895
TRINITY_DN2475_c0_g1_i1_orf1	green	0.69812
TRINITY_DN11215_c0_g1_i1_orf1	turquoise	0.94824
TRINITY_DN33967_c2_g2_i1_orf1	red	0.77395
TRINITY_DN9965_c0_g1_i1_orf1	turquoise	0.89929
TRINITY_DN5074_c0_g1_i7_orf1	blue	0.91728
TRINITY_DN22577_c0_g1_i2_orf1	red	0.75138
TRINITY_DN9853_c0_g3_i1_orf1	turquoise	0.98639
TRINITY_DN9938_c0_g2_i1_orf1	turquoise	0.85422
TRINITY_DN2999_c1_g2_i1_orf1	turquoise	0.82216
TRINITY_DN15448_c0_g1_i1_orf1	turquoise	0.95688
TRINITY_DN14874_c0_g1_i6_orf1	brown	0.58278
TRINITY_DN88539_c0_g2_i1_orf1	turquoise	0.89115
TRINITY_DN5190_c0_g3_i1_orf1	black	0.89766
TRINITY_DN107962_c0_g1_i1_orf1	yellow	0.73843
TRINITY_DN16390_c0_g1_i4_orf1	blue	0.78576
TRINITY_DN855_c0_g1_i5_orf1	black	0.79163
TRINITY_DN37923_c0_g1_i1_orf1	red	0.91267
TRINITY_DN11322_c0_g1_i2_orf1	turquoise	0.85421
TRINITY_DN64616_c0_g1_i1_orf1	blue	0.90654
TRINITY_DN4255_c0_g1_i11_orf1	black	0.95579
TRINITY_DN32532_c0_g1_i1_orf1	green	0.89485
TRINITY_DN7134_c0_g1_i1_orf1	turquoise	0.85497
TRINITY_DN1073_c0_g1_i4_orf1	turquoise	0.98807
TRINITY_DN5234_c0_g1_i2_orf1	turquoise	0.87559
TRINITY_DN49872_c0_g1_i2_orf1	red	0.70656
TRINITY_DN1756_c0_g1_i3_orf1	brown	0.91038
TRINITY_DN9028_c0_g1_i5_orf1	blue	0.87221
TRINITY_DN91533_c0_g1_i1_orf1	blue	0.97302
TRINITY_DN101358_c0_g2_i1_orf1	turquoise	0.78532
TRINITY_DN1109_c0_g1_i6_orf1	turquoise	0.8775
TRINITY_DN7022_c0_g1_i7_orf1	yellow	0.71562
TRINITY_DN36476_c1_g1_i1_orfp1	brown	0.97325
TRINITY_DN6871_c0_g1_i3_orf1	turquoise	0.59927
TRINITY_DN566_c0_g1_i13_orf1	brown	0.9359
TRINITY_DN32509_c0_g1_i3_orf1	turquoise	0.95561
TRINITY_DN39933_c0_g1_i2_orf1	brown	0.73375
TRINITY_DN2175_c0_g1_i4_orf1	yellow	0.82557

TRINITY_DN7836_c0_g1_i2_orf1	turquoise	0.87744
TRINITY_DN12323_c0_g2_i2_orf1	turquoise	0.96657
TRINITY_DN399_c3_g2_i6_orf1	green	0.96956
TRINITY_DN4443_c0_g1_i4_orf1	black	0.95072
TRINITY_DN6244_c0_g1_i4_orf1	blue	0.76422
TRINITY_DN14298_c0_g3_i1_orf1	red	0.94584
TRINITY_DN144_c0_g1_i4_orf1	blue	0.92553
TRINITY_DN5001_c0_g1_i4_orf1	brown	0.81943
TRINITY_DN7131_c0_g1_i2_orf1	yellow	0.74015
TRINITY_DN45924_c0_g1_i14_orf1	turquoise	0.99058
TRINITY_DN17559_c0_g1_i4_orf1	turquoise	0.98227
TRINITY_DN38685_c0_g1_i4_orf1	brown	0.90806
TRINITY_DN7152_c0_g1_i1_orf1	brown	0.95084
TRINITY_DN45449_c0_g1_i1_orf1	turquoise	0.79896
TRINITY_DN34465_c0_g1_i1_orf1	green	0.21339
TRINITY_DN747_c0_g2_i1_orf1	turquoise	0.65919
TRINITY_DN4182_c0_g1_i6_orf1	turquoise	0.50622
TRINITY_DN6308_c0_g1_i3_orf1	turquoise	0.86388
TRINITY_DN2508_c0_g1_i2_orf1	black	0.84288
TRINITY_DN18593_c0_g1_i1_orf1	turquoise	0.79359
TRINITY_DN101658_c0_g1_i1_orf1	brown	0.79487
TRINITY_DN44658_c0_g1_i2_orf1	red	0.90178
TRINITY_DN585_c0_g1_i5_orf1	blue	0.96079
TRINITY_DN16091_c0_g1_i1_orfp1	green	0.74514
TRINITY_DN18558_c0_g1_i7_orf1	turquoise	0.80043
TRINITY_DN29291_c0_g1_i1_orf1	turquoise	0.92452
TRINITY_DN3515_c0_g1_i3_orf1	blue	0.64108
TRINITY_DN67243_c0_g1_i1_orf1	brown	0.90244
TRINITY_DN4929_c1_g2_i5_orf1	turquoise	0.88032
TRINITY_DN98313_c0_g1_i1_orf1	green	0.90499
TRINITY_DN42856_c0_g1_i1_orf1	black	0.94347
TRINITY_DN16354_c0_g1_i2_orf1	turquoise	0.73952
TRINITY_DN22422_c0_g1_i4_orf1	turquoise	0.51333
TRINITY_DN4294_c0_g1_i6_orf1	turquoise	0.53786
TRINITY_DN8406_c0_g1_i2_orf1	yellow	0.83698
TRINITY_DN40434_c0_g1_i2_orf1	yellow	0.79906
TRINITY_DN12227_c0_g2_i3_orf1	turquoise	0.6058
TRINITY_DN5597_c0_g1_i2_orf1	turquoise	0.88376
TRINITY_DN53810_c0_g1_i1_orf1	turquoise	0.8936
TRINITY_DN48765_c0_g1_i7_orf1	blue	0.64294
TRINITY_DN43942_c0_g2_i1_orf1	turquoise	0.96919
TRINITY_DN38307_c0_g1_i1_orfp1	blue	0.88
TRINITY_DN4742_c0_g1_i1_orf1	grey	-0.67547
TRINITY_DN61674_c0_g1_i2_orf1	turquoise	0.9895
TRINITY_DN40176_c0_g1_i1_orf1	red	0.43994
TRINITY_DN14677_c0_g2_i3_orf1	red	0.82523
TRINITY_DN63943_c0_g1_i5_orf1	blue	0.62976
TRINITY_DN5867_c0_g1_i1_orf1	turquoise	0.98592
TRINITY_DN9820_c0_g1_i1_orf1	yellow	0.96603
TRINITY_DN86844_c0_g2_i1_orf1	turquoise	0.95708
TRINITY_DN14498_c0_g1_i1_orf1	turquoise	0.89543
TRINITY_DN62192_c0_g1_i2_orf1	green	0.73304
TRINITY_DN62_c0_g1_i18_orf1	turquoise	0.86542
TRINITY_DN10722_c0_g3_i1_orf1	turquoise	0.78271
TRINITY_DN3431_c0_g1_i1_orf1	green	0.75083
TRINITY_DN1170_c0_g1_i8_orf1	turquoise	0.7184
TRINITY_DN19244_c0_g1_i7_orf1	turquoise	0.95696
TRINITY_DN295_c2_g1_i2_orf1	green	0.93585

TRINITY_DN6074_c0_g1_i1_orf1	turquoise	0.98705
TRINITY_DN41086_c0_g1_i4_orf1	turquoise	0.98888
TRINITY_DN21215_c0_g1_i7_orf1	turquoise	0.59323
TRINITY_DN1034_c0_g2_i1_orf1	brown	0.84799
TRINITY_DN143509_c0_g1_i1_orf1	yellow	0.84387
TRINITY_DN82017_c0_g1_i5_orf1	turquoise	0.37168
TRINITY_DN1351_c0_g1_i1_orf1	turquoise	0.7868
TRINITY_DN86772_c0_g1_i3_orfp1	brown	0.95567
TRINITY_DN3066_c0_g1_i5_orf1	red	0.48333
TRINITY_DN1497_c0_g2_i6_orf1	black	0.89341
TRINITY_DN3196_c0_g1_i1_orf1	brown	0.94238
TRINITY_DN2346_c0_g2_i1_orf1	brown	0.35073
TRINITY_DN15755_c0_g1_i1_orf1	brown	0.85255
TRINITY_DN41708_c0_g1_i1_orf1	pink	0.87657
TRINITY_DN214_c0_g1_i3_orf1	brown	0.83513
TRINITY_DN18338_c0_g1_i6_orf1	blue	0.8962
TRINITY_DN14953_c0_g1_i5_orf1	turquoise	0.96004
TRINITY_DN875_c0_g1_i3_orf1	blue	0.93125
TRINITY_DN84938_c0_g1_i4_orf1	brown	0.70987
TRINITY_DN2267_c0_g1_i1_orf1	turquoise	0.98714
TRINITY_DN9072_c0_g1_i1_orf1	red	0.74873
TRINITY_DN3800_c0_g1_i7_orf1	yellow	0.97455
TRINITY_DN6535_c0_g1_i3_orf1	turquoise	0.94283
TRINITY_DN940_c0_g1_i4_orf1	turquoise	0.59891
TRINITY_DN33008_c0_g1_i1_orf1	red	0.79881
TRINITY_DN146217_c0_g1_i1_orf1	yellow	0.88388
TRINITY_DN42738_c0_g1_i1_orf1	turquoise	0.96379
TRINITY_DN2058_c0_g1_i2_orf1	blue	0.88913
TRINITY_DN3529_c0_g1_i7_orf1	turquoise	0.49155
TRINITY_DN5564_c0_g1_i5_orf1	brown	0.45925
TRINITY_DN4533_c0_g1_i1_orf1	red	0.93942
TRINITY_DN7565_c0_g1_i3_orf1	blue	0.90557
TRINITY_DN24751_c0_g1_i1_orf1	turquoise	0.99051
TRINITY_DN1091_c0_g3_i1_orf1	brown	0.78379
TRINITY_DN6059_c0_g1_i1_orf1	turquoise	0.97821
TRINITY_DN3325_c0_g1_i1_orf1	black	0.81226
TRINITY_DN20614_c0_g1_i1_orf1	blue	0.64538
TRINITY_DN107261_c0_g1_i1_orf1	turquoise	0.94319
TRINITY_DN4233_c0_g2_i2_orf1	red	0.87418
TRINITY_DN6988_c0_g1_i3_orf1	green	0.96315
TRINITY_DN16174_c0_g1_i2_orf1	turquoise	0.94477
TRINITY_DN2808_c0_g1_i8_orf1	red	0.88922
TRINITY_DN629_c0_g1_i6_orf1	red	0.76477
TRINITY_DN1161_c0_g1_i2_orf1	brown	0.9098
TRINITY_DN13347_c0_g1_i1_orf1	turquoise	0.94521
TRINITY_DN3970_c0_g1_i1_orf1	turquoise	0.76611
TRINITY_DN32448_c0_g1_i1_orf1	green	0.87731
TRINITY_DN13375_c0_g1_i6_orf1	red	0.89237
TRINITY_DN24323_c0_g1_i3_orf1	turquoise	0.59369
TRINITY_DN4589_c0_g1_i1_orf1	turquoise	0.97374
TRINITY_DN37585_c0_g1_i1_orf1	blue	0.92155
TRINITY_DN5818_c1_g1_i2_orf1	turquoise	0.87906
TRINITY_DN30704_c0_g1_i1_orf1	turquoise	0.95254
TRINITY_DN13856_c0_g1_i1_orf1	blue	0.81841
TRINITY_DN52649_c0_g1_i6_orf1	brown	0.85027
TRINITY_DN8226_c0_g1_i1_orf1	yellow	0.94357
TRINITY_DN7161_c0_g1_i7_orf1	turquoise	0.96345
TRINITY_DN2623_c0_g1_i3_orf1	turquoise	0.87276

TRINITY_DN482_c0_g1_i1_orf1	red	0.89243
TRINITY_DN131662_c0_g1_i4_orf1	turquoise	0.84965
TRINITY_DN35763_c0_g1_i2_orf1	brown	0.90017
TRINITY_DN4189_c0_g2_i1_orf1	blue	0.95139
TRINITY_DN4952_c0_g1_i1_orf1	yellow	0.70498
TRINITY_DN1914_c0_g1_i6_orf1	turquoise	0.98826
TRINITY_DN2271_c0_g1_i12_orf1	green	0.87511
TRINITY_DN29038_c0_g2_i1_orf1	turquoise	0.98818
TRINITY_DN2177_c0_g1_i1_orf1	turquoise	0.8236
TRINITY_DN44335_c0_g1_i7_orf1	turquoise	0.80399
TRINITY_DN18839_c0_g1_i4_orf1	turquoise	0.3936
TRINITY_DN115658_c0_g1_i1_orf1	turquoise	0.57272
TRINITY_DN5852_c0_g1_i13_orf1	yellow	0.68026
TRINITY_DN1673_c0_g1_i2_orf1	turquoise	0.90731
TRINITY_DN16894_c0_g1_i5_orf1	turquoise	0.86101
TRINITY_DN13114_c0_g1_i1_orf1	turquoise	0.94613
TRINITY_DN12392_c0_g1_i3_orf1	brown	0.64106
TRINITY_DN11375_c0_g1_i4_orf1	red	0.64993
TRINITY_DN17738_c0_g1_i2_orf1	turquoise	0.99728
TRINITY_DN19829_c0_g2_i1_orf1	turquoise	0.96698
TRINITY_DN2378_c0_g1_i5_orf1	brown	0.97748
TRINITY_DN30498_c0_g1_i3_orf1	green	0.87688
TRINITY_DN57900_c0_g1_i2_orf1	yellow	0.7705
TRINITY_DN141738_c0_g1_i1_orf1	turquoise	0.976
TRINITY_DN87648_c0_g1_i1_orfp1	yellow	0.78305
TRINITY_DN16978_c0_g1_i1_orf1	pink	0.49079
TRINITY_DN48641_c0_g1_i4_orf1	red	0.97135
TRINITY_DN8724_c0_g1_i2_orf1	black	0.93451
TRINITY_DN8473_c0_g1_i5_orf1	green	0.92589
TRINITY_DN15607_c0_g1_i6_orf1	turquoise	0.98572
TRINITY_DN57105_c0_g1_i2_orf1	turquoise	0.73435
TRINITY_DN512_c0_g1_i10_orf1	brown	0.92207
TRINITY_DN1133_c0_g1_i6_orf1	red	0.86868
TRINITY_DN105359_c0_g2_i5_orf1	red	0.83284
TRINITY_DN36928_c0_g1_i5_orf1	turquoise	0.43558
TRINITY_DN11375_c0_g1_i6_orf1	red	0.80978
TRINITY_DN5595_c0_g1_i1_orf1	blue	0.95431
TRINITY_DN9085_c0_g1_i1_orf1	turquoise	0.95982
TRINITY_DN8640_c0_g1_i4_orf1	turquoise	0.81857
TRINITY_DN4144_c0_g1_i7_orf1	red	0.86318
TRINITY_DN12024_c0_g1_i4_orf1	green	0.87327
TRINITY_DN37986_c0_g1_i2_orf1	turquoise	0.76865
TRINITY_DN3814_c1_g1_i1_orf1	turquoise	0.96149
TRINITY_DN31815_c0_g1_i4_orf1	turquoise	0.91046
TRINITY_DN36632_c0_g1_i1_orf1	brown	0.77777
TRINITY_DN40440_c0_g1_i1_orf1	turquoise	0.33694
TRINITY_DN34727_c0_g1_i3_orf1	blue	0.91937
TRINITY_DN42185_c0_g1_i7_orf1	yellow	0.58958
TRINITY_DN376_c1_g1_i1_orf1	green	0.9142
TRINITY_DN6309_c0_g1_i7_orf1	brown	0.77392
TRINITY_DN52788_c0_g1_i1_orf1	blue	0.96278
TRINITY_DN10222_c0_g1_i2_orf1	brown	0.92576
TRINITY_DN9062_c0_g2_i3_orf1	red	0.94118
TRINITY_DN4767_c0_g1_i4_orf1	green	0.95044
TRINITY_DN61744_c0_g1_i1_orf1	turquoise	0.67341
TRINITY_DN10650_c0_g1_i1_orf1	brown	0.60378
TRINITY_DN7573_c0_g2_i1_orf1	turquoise	0.96954
TRINITY_DN11886_c0_g1_i1_orf1	turquoise	0.92047

TRINITY_DN19115_c0_g1_i1_orf1	turquoise	0.89435
TRINITY_DN103107_c0_g1_i2_orf1	yellow	0.50341
TRINITY_DN4246_c0_g2_i3_orf1	brown	0.46782
TRINITY_DN22962_c0_g1_i1_orf1	brown	0.67668
TRINITY_DN15417_c0_g1_i6_orf1	turquoise	0.43426
TRINITY_DN974_c0_g2_i1_orfp1	pink	0.69218
TRINITY_DN609_c0_g1_i1_orf1	yellow	0.78821
TRINITY_DN1630_c0_g1_i6_orf1	blue	0.93638
TRINITY_DN64222_c0_g1_i1_orf1	blue	0.9012
TRINITY_DN36434_c0_g2_i3_orf1	brown	0.93489
TRINITY_DN19080_c0_g1_i4_orf1	turquoise	0.62633
TRINITY_DN8915_c0_g1_i3_orf1	yellow	0.93021
TRINITY_DN9117_c0_g1_i1_orf1	turquoise	0.85283
TRINITY_DN114982_c0_g1_i1_orf1	blue	0.83072
TRINITY_DN2983_c0_g1_i6_orf1	brown	0.86564
TRINITY_DN6660_c0_g1_i5_orf1	turquoise	0.48164
TRINITY_DN10373_c0_g1_i1_orf1	black	0.93005
TRINITY_DN35800_c0_g1_i6_orf1	brown	0.8293
TRINITY_DN20658_c0_g1_i1_orf1	brown	0.97654
TRINITY_DN15362_c0_g1_i1_orf1	turquoise	0.77187
TRINITY_DN18909_c0_g1_i8_orf1	turquoise	0.95993
TRINITY_DN1470_c0_g1_i2_orf1	yellow	0.94342
TRINITY_DN2140_c0_g1_i1_orf1	blue	0.97947
TRINITY_DN19135_c0_g1_i1_orf1	turquoise	0.90704
TRINITY_DN59852_c0_g1_i1_orf1	brown	0.86445
TRINITY_DN542_c0_g2_i1_orf1	turquoise	0.98922
TRINITY_DN4276_c0_g1_i11_orf1	black	0.80405
TRINITY_DN46090_c0_g3_i1_orf1	green	0.83546
TRINITY_DN22597_c0_g1_i4_orf1	yellow	0.89028
TRINITY_DN16673_c0_g1_i1_orf1	turquoise	0.71543
TRINITY_DN2004_c0_g1_i20_orf1	red	0.79612
TRINITY_DN48497_c0_g1_i1_orf1	green	0.10497
TRINITY_DN20215_c0_g2_i1_orf1	red	0.87257
TRINITY_DN3929_c0_g3_i3_orf1	turquoise	0.93228
TRINITY_DN3975_c0_g1_i10_orf1	green	0.68342
TRINITY_DN3241_c0_g1_i1_orf1	green	0.56099
TRINITY_DN5628_c0_g1_i3_orf1	yellow	0.85162
TRINITY_DN35051_c0_g1_i1_orf1	turquoise	0.99083
TRINITY_DN14154_c0_g1_i1_orf1	blue	0.65432
TRINITY_DN18172_c0_g1_i6_orf1	turquoise	0.9867
TRINITY_DN5678_c0_g2_i3_orf1	turquoise	0.97636
TRINITY_DN4025_c0_g1_i13_orf1	turquoise	0.87784
TRINITY_DN10360_c0_g1_i16_orf1	brown	0.78881
TRINITY_DN64_c0_g1_i4_orf1	turquoise	0.53246
TRINITY_DN15420_c0_g3_i2_orf1	blue	0.9613
TRINITY_DN162_c0_g1_i4_orf1	red	0.80445
TRINITY_DN14328_c0_g1_i12_orf1	brown	0.94718
TRINITY_DN146236_c0_g1_i1_orf1	yellow	0.76806
TRINITY_DN42719_c0_g2_i1_orf1	green	0.90942
TRINITY_DN38562_c0_g1_i3_orf1	turquoise	0.68043
TRINITY_DN659_c0_g2_i1_orf1	turquoise	0.64355
TRINITY_DN31399_c0_g1_i3_orf1	turquoise	0.89518
TRINITY_DN64446_c0_g1_i1_orf1	turquoise	0.99679
TRINITY_DN16147_c0_g1_i4_orf1	brown	0.79515
TRINITY_DN7960_c0_g1_i2_orf1	brown	0.9372
TRINITY_DN42337_c0_g1_i5_orf1	blue	0.94309
TRINITY_DN3539_c0_g1_i7_orf1	turquoise	0.89755
TRINITY_DN5704_c0_g1_i6_orf1	turquoise	0.97492

TRINITY_DN4004_c0_g1_i1_orf1	brown	0.69859
TRINITY_DN97883_c0_g1_i2_orf1	black	0.83792
TRINITY_DN3675_c0_g1_i1_orf1	blue	0.88791
TRINITY_DN8458_c0_g2_i1_orf1	blue	0.68269
TRINITY_DN120144_c0_g1_i1_orf1	red	0.84508
TRINITY_DN69697_c0_g1_i1_orf1	turquoise	0.79985
TRINITY_DN267_c0_g1_i1_orf1	blue	0.93786
TRINITY_DN94248_c0_g2_i3_orf1	turquoise	0.9719
TRINITY_DN9455_c0_g1_i6_orf1	red	0.8928
TRINITY_DN10385_c0_g1_i5_orf1	red	0.92124
TRINITY_DN12787_c1_g1_i1_orf1	turquoise	0.79887
TRINITY_DN4944_c0_g1_i2_orf1	turquoise	0.99273
TRINITY_DN12387_c0_g1_i1_orf1	blue	0.97033
TRINITY_DN1194_c0_g1_i5_orf1	blue	0.78994
TRINITY_DN1816_c0_g1_i5_orf1	turquoise	0.75965
TRINITY_DN2880_c0_g1_i2_orf1	black	0.98176
TRINITY_DN11584_c0_g1_i2_orf1	turquoise	0.93393
TRINITY_DN1123_c2_g1_i5_orf1	yellow	0.74784
TRINITY_DN79000_c1_g1_i1_orf1	turquoise	0.9409
TRINITY_DN8258_c0_g1_i3_orf1	brown	0.93927
TRINITY_DN12973_c0_g1_i1_orf1	turquoise	0.97329
TRINITY_DN668_c0_g1_i4_orf1	blue	0.92225
TRINITY_DN10530_c0_g1_i1_orf1	turquoise	0.9929
TRINITY_DN48020_c0_g1_i1_orf1	turquoise	0.97185
TRINITY_DN11124_c0_g1_i4_orf1	turquoise	0.96589
TRINITY_DN2107_c0_g2_i3_orf1	blue	0.93769
TRINITY_DN2062_c0_g1_i11_orf1	turquoise	0.97086
TRINITY_DN3335_c0_g1_i1_orf1	red	0.67878
TRINITY_DN29563_c0_g1_i5_orf1	red	0.88987
TRINITY_DN54366_c0_g1_i1_orf1	blue	0.89458
TRINITY_DN1741_c0_g1_i5_orf1	brown	0.8808
TRINITY_DN6313_c0_g1_i4_orf1	turquoise	0.92649
TRINITY_DN2695_c0_g1_i14_orfp1	turquoise	0.89687
TRINITY_DN71840_c0_g1_i1_orf1	turquoise	0.96943
TRINITY_DN13711_c0_g1_i1_orf1	brown	0.79359
TRINITY_DN138481_c0_g1_i5_orf1	blue	0.99149
TRINITY_DN14298_c0_g1_i1_orf1	red	0.97429
TRINITY_DN3707_c0_g1_i1_orf1	green	0.95704
TRINITY_DN3878_c0_g1_i4_orf1	turquoise	0.98598
TRINITY_DN218_c0_g1_i1_orf1	blue	0.93721
TRINITY_DN16128_c0_g1_i5_orf1	turquoise	0.88325
TRINITY_DN975_c0_g1_i1_orf1	brown	0.76554
TRINITY_DN1280_c0_g1_i1_orf1	red	0.96705
TRINITY_DN34406_c0_g2_i9_orfp1	black	0.86202
TRINITY_DN16830_c0_g1_i5_orf1	turquoise	0.99024
TRINITY_DN460_c0_g1_i3_orf1	blue	0.54388
TRINITY_DN13119_c0_g1_i4_orf1	yellow	0.97251
TRINITY_DN2201_c0_g1_i1_orf1	red	0.67537
TRINITY_DN29873_c0_g1_i1_orf1	brown	0.90533
TRINITY_DN24281_c0_g1_i1_orf1	turquoise	0.95881
TRINITY_DN5459_c0_g1_i1_orf1	turquoise	0.80047
TRINITY_DN2255_c0_g1_i1_orf1	brown	0.92533
TRINITY_DN15338_c0_g1_i7_orf1	turquoise	0.46387
TRINITY_DN41129_c0_g1_i1_orf1	green	0.32862
TRINITY_DN5908_c0_g1_i2_orf1	turquoise	0.83468
TRINITY_DN3831_c0_g1_i7_orf1	brown	0.9356
TRINITY_DN1661_c0_g1_i1_orf1	red	0.94224
TRINITY_DN8405_c0_g1_i4_orf1	red	0.71981

TRINITY_DN21596_c0_g1_i1_orf1	turquoise	0.61678
TRINITY_DN58413_c0_g1_i4_orf1	turquoise	0.97765
TRINITY_DN15175_c0_g1_i1_orf1	blue	0.98452
TRINITY_DN2570_c0_g1_i1_orf1	brown	0.84492
TRINITY_DN50593_c0_g1_i1_orf1	yellow	0.75998
TRINITY_DN67716_c0_g1_i1_orf1	turquoise	0.91261
TRINITY_DN10290_c0_g1_i7_orf1	blue	0.8335
TRINITY_DN75746_c0_g1_i1_orfp1	turquoise	0.98387
TRINITY_DN2270_c0_g2_i1_orf1	turquoise	0.98354
TRINITY_DN99063_c0_g1_i1_orf1	yellow	0.86855
TRINITY_DN11798_c0_g2_i1_orf1	brown	0.84201
TRINITY_DN18912_c1_g1_i1_orf1	turquoise	0.75213
TRINITY_DN184_c0_g1_i1_orf1	brown	0.40908
TRINITY_DN48619_c0_g1_i1_orf1	turquoise	0.90239
TRINITY_DN805_c0_g1_i5_orf1	green	0.94249
TRINITY_DN125967_c0_g1_i1_orf1	blue	0.75303
TRINITY_DN21555_c0_g1_i4_orf1	blue	0.99374
TRINITY_DN143496_c0_g1_i1_orf1	turquoise	0.65129
TRINITY_DN7391_c0_g1_i2_orf1	turquoise	0.50683
TRINITY_DN760_c1_g2_i6_orf1	turquoise	0.95803
TRINITY_DN2894_c0_g3_i1_orf1	turquoise	0.99065
TRINITY_DN346_c0_g1_i7_orf1	brown	0.83984
TRINITY_DN7626_c0_g1_i1_orf1	turquoise	0.99277
TRINITY_DN11076_c0_g2_i1_orf1	green	0.48736
TRINITY_DN3964_c1_g1_i2_orf1	turquoise	0.62415
TRINITY_DN26824_c0_g1_i1_orf1	turquoise	0.81276
TRINITY_DN86355_c0_g1_i1_orf1	turquoise	0.49961
TRINITY_DN125071_c0_g1_i1_orf1	turquoise	0.57831
TRINITY_DN4779_c0_g1_i5_orf1	turquoise	0.9799
TRINITY_DN1803_c0_g1_i3_orf1	turquoise	0.93364
TRINITY_DN8480_c0_g1_i1_orf1	blue	0.77487
TRINITY_DN3019_c0_g1_i1_orf1	pink	0.89612
TRINITY_DN9356_c0_g1_i1_orf1	turquoise	0.62166
TRINITY_DN27087_c0_g1_i1_orf1	turquoise	0.97602
TRINITY_DN63030_c0_g1_i5_orf1	yellow	0.83336
TRINITY_DN834_c0_g1_i1_orf1	turquoise	0.62205
TRINITY_DN41311_c0_g2_i3_orf1	turquoise	0.87667
TRINITY_DN23774_c0_g1_i1_orf1	turquoise	0.7038
TRINITY_DN10476_c0_g1_i1_orf1	turquoise	0.99274
TRINITY_DN11194_c0_g1_i4_orf1	turquoise	0.87323
TRINITY_DN2109_c0_g1_i4_orf1	blue	0.85663
TRINITY_DN15959_c0_g1_i1_orf1	turquoise	0.98888
TRINITY_DN27968_c0_g2_i2_orf1	blue	0.96436
TRINITY_DN391_c5_g1_i1_orf1	turquoise	0.99298
TRINITY_DN11178_c0_g1_i1_orf1	turquoise	0.7658
TRINITY_DN4524_c0_g1_i2_orf1	blue	0.77938
TRINITY_DN1757_c0_g1_i4_orf1	turquoise	0.85341
TRINITY_DN2542_c0_g2_i1_orf1	turquoise	0.68026
TRINITY_DN44407_c0_g4_i2_orf1	turquoise	0.6468
TRINITY_DN120979_c0_g1_i1_orf1	turquoise	0.94802
TRINITY_DN26993_c1_g1_i8_orf1	yellow	0.83795
TRINITY_DN49956_c0_g1_i1_orf1	green	0.80676
TRINITY_DN434_c0_g1_i4_orf1	turquoise	0.65214
TRINITY_DN34134_c0_g2_i1_orf1	brown	0.92005
TRINITY_DN104139_c0_g1_i1_orf1	turquoise	0.72851
TRINITY_DN8714_c0_g1_i6_orf1	black	0.78752
TRINITY_DN8553_c0_g1_i4_orf1	turquoise	0.63484
TRINITY_DN7277_c0_g1_i1_orf1	red	0.64246

TRINITY_DN44119_c0_g1_i1_orf1	turquoise	0.92659
TRINITY_DN46216_c0_g3_i1_orf1	yellow	0.93723
TRINITY_DN3861_c0_g3_i2_orf1	green	0.89964
TRINITY_DN24689_c0_g1_i1_orf1	blue	0.51252
TRINITY_DN4228_c0_g1_i5_orf1	blue	0.8439
TRINITY_DN2472_c0_g1_i6_orf1	green	0.94108
TRINITY_DN9239_c0_g2_i2_orf1	blue	0.84286
TRINITY_DN3433_c0_g1_i6_orf1	brown	0.75464
TRINITY_DN76377_c0_g1_i1_orf1	red	0.9387
TRINITY_DN170_c1_g1_i5_orf1	turquoise	0.89823
TRINITY_DN259_c0_g1_i8_orf1	turquoise	0.93119
TRINITY_DN3896_c0_g1_i1_orf1	blue	0.90155
TRINITY_DN585_c0_g1_i12_orf1	black	0.91569
TRINITY_DN23020_c0_g1_i1_orf1	blue	0.56596
TRINITY_DN15774_c0_g1_i3_orf1	green	0.8813
TRINITY_DN10742_c0_g1_i4_orf1	brown	0.85342
TRINITY_DN33837_c0_g1_i6_orf1	red	0.83573
TRINITY_DN7740_c0_g1_i2_orf1	brown	0.98363
TRINITY_DN2953_c1_g1_i2_orf1	turquoise	0.9476
TRINITY_DN1534_c0_g1_i3_orf1	brown	0.63176
TRINITY_DN81312_c0_g1_i1_orf1	turquoise	0.83666
TRINITY_DN31058_c0_g1_i6_orf1	turquoise	0.86215
TRINITY_DN13385_c0_g1_i4_orf1	yellow	0.81265
TRINITY_DN13718_c0_g1_i4_orf1	brown	0.7557
TRINITY_DN5354_c0_g1_i4_orf1	turquoise	0.68232
TRINITY_DN8158_c0_g1_i2_orf1	green	0.83897
TRINITY_DN935_c0_g1_i3_orf1	pink	0.90624
TRINITY_DN51252_c0_g2_i1_orf1	turquoise	0.89187
TRINITY_DN3355_c0_g2_i4_orf1	turquoise	0.90402
TRINITY_DN4065_c0_g1_i5_orf1	black	0.86929
TRINITY_DN4929_c0_g1_i1_orf1	turquoise	0.65046
TRINITY_DN145647_c0_g1_i1_orf1	turquoise	0.98829
TRINITY_DN394_c0_g1_i4_orf1	brown	0.87373
TRINITY_DN9324_c1_g2_i2_orf1	red	0.80236
TRINITY_DN29879_c0_g1_i3_orf1	blue	0.85235
TRINITY_DN20682_c0_g2_i1_orf1	turquoise	0.98992
TRINITY_DN8691_c0_g1_i3_orf1	turquoise	0.93143
TRINITY_DN40_c0_g2_i1_orf1	turquoise	0.97927
TRINITY_DN121802_c0_g1_i6_orfp1	brown	0.68198
TRINITY_DN48973_c0_g1_i5_orf1	turquoise	0.68141
TRINITY_DN53311_c0_g2_i1_orf1	red	0.92502
TRINITY_DN9340_c0_g1_i4_orf1	brown	0.92812
TRINITY_DN4937_c0_g1_i2_orf1	red	0.76283
TRINITY_DN2348_c0_g1_i1_orfp1	blue	0.92444
TRINITY_DN3616_c0_g2_i1_orf1	blue	0.9852
TRINITY_DN115498_c0_g1_i1_orf1	turquoise	0.92235
TRINITY_DN1641_c0_g1_i8_orf1	turquoise	0.74259
TRINITY_DN122170_c0_g1_i2_orfp1	turquoise	0.98483
TRINITY_DN4256_c0_g1_i1_orf1	blue	0.79504
TRINITY_DN121047_c0_g1_i3_orf1	turquoise	0.79181
TRINITY_DN20007_c0_g1_i1_orf1	turquoise	0.90558
TRINITY_DN31047_c0_g1_i4_orf1	turquoise	0.98091
TRINITY_DN6381_c0_g1_i2_orf1	turquoise	0.71837
TRINITY_DN2084_c0_g1_i1_orf1	turquoise	0.96667
TRINITY_DN32956_c0_g1_i4_orf1	red	0.91836
TRINITY_DN12969_c0_g1_i3_orf1	brown	0.65432
TRINITY_DN2374_c0_g1_i1_orf1	turquoise	0.85246
TRINITY_DN1552_c0_g1_i3_orf1	red	0.93658

TRINITY_DN138086_c0_g1_i1_orf1	brown	0.88918
TRINITY_DN121156_c0_g2_i1_orf1	turquoise	0.58085
TRINITY_DN5756_c0_g1_i4_orf1	turquoise	0.94424
TRINITY_DN12748_c2_g1_i1_orfp1	pink	0.89331
TRINITY_DN9711_c0_g1_i10_orf1	turquoise	0.80476
TRINITY_DN397_c0_g1_i1_orf1	red	0.96089
TRINITY_DN3223_c0_g1_i4_orf1	blue	0.73533
TRINITY_DN1005_c0_g1_i5_orf1	blue	0.83233
TRINITY_DN147427_c0_g1_i1_orf1	turquoise	0.79629
TRINITY_DN3057_c0_g2_i1_orf1	turquoise	0.83456
TRINITY_DN14701_c0_g1_i2_orf1	turquoise	0.80277
TRINITY_DN13285_c0_g1_i9_orf1	black	0.94279
TRINITY_DN17212_c0_g1_i6_orf1	turquoise	0.87468
TRINITY_DN1491_c0_g1_i4_orf1	blue	0.77226
TRINITY_DN101_c0_g1_i4_orf1	green	0.80226
TRINITY_DN1870_c0_g1_i6_orf1	green	0.84045
TRINITY_DN17417_c0_g1_i11_orf1	turquoise	0.91153
TRINITY_DN11942_c0_g1_i1_orf1	red	0.30882
TRINITY_DN5012_c0_g1_i6_orf1	turquoise	0.85325
TRINITY_DN110132_c0_g1_i1_orf1	turquoise	0.54029
TRINITY_DN1034_c0_g1_i4_orf1	brown	0.8646
TRINITY_DN90497_c0_g1_i1_orf1	yellow	0.8185
TRINITY_DN41952_c0_g1_i1_orf1	brown	0.87496
TRINITY_DN6876_c0_g2_i1_orf1	turquoise	0.89822
TRINITY_DN39266_c0_g1_i1_orf1	green	0.9538
TRINITY_DN18391_c0_g2_i8_orf1	red	0.82022
TRINITY_DN4273_c1_g1_i5_orf1	blue	0.94853
TRINITY_DN11876_c0_g1_i2_orf1	turquoise	0.73296
TRINITY_DN2655_c0_g2_i1_orf1	green	0.43271
TRINITY_DN10430_c0_g1_i4_orf1	brown	0.77809
TRINITY_DN9062_c0_g2_i1_orf1	red	0.68036
TRINITY_DN12_c0_g1_i5_orf1	turquoise	0.54072
TRINITY_DN12686_c0_g1_i4_orf1	black	0.57937
TRINITY_DN117707_c0_g1_i3_orf1	turquoise	0.87021
TRINITY_DN5070_c0_g1_i1_orf1	blue	0.78684
TRINITY_DN1375_c0_g1_i5_orf1	turquoise	0.92794
TRINITY_DN23534_c0_g2_i2_orf1	turquoise	0.47779
TRINITY_DN113626_c0_g1_i3_orfp1	turquoise	0.78068
TRINITY_DN101_c0_g2_i2_orf1	blue	0.74273
TRINITY_DN12301_c0_g1_i1_orf1	green	0.94222
TRINITY_DN1083_c0_g1_i4_orf1	pink	0.69289
TRINITY_DN13055_c0_g1_i5_orf1	turquoise	0.88656
TRINITY_DN2897_c0_g2_i1_orf1	blue	0.7691
TRINITY_DN906_c0_g1_i4_orf1	blue	0.98985
TRINITY_DN13898_c0_g1_i2_orf1	blue	0.74854
TRINITY_DN11015_c0_g1_i8_orf1	pink	0.83616
TRINITY_DN22044_c0_g2_i1_orf1	yellow	0.88308
TRINITY_DN60048_c0_g2_i1_orf1	turquoise	0.93113
TRINITY_DN52893_c0_g1_i1_orf1	turquoise	0.97615
TRINITY_DN5363_c0_g1_i1_orf1	turquoise	0.31303
TRINITY_DN72707_c0_g1_i1_orf1	turquoise	0.98057
TRINITY_DN28989_c0_g1_i7_orf1	blue	0.72529
TRINITY_DN56155_c0_g1_i1_orf1	blue	0.96656
TRINITY_DN6426_c0_g1_i2_orf1	green	0.83402
TRINITY_DN3773_c0_g1_i4_orf1	turquoise	0.94641
TRINITY_DN43792_c0_g1_i1_orf1	turquoise	0.95117
TRINITY_DN51197_c0_g1_i3_orf1	black	0.56189
TRINITY_DN18592_c0_g1_i4_orf1	blue	0.59796

TRINITY_DN25987_c0_g1_i5_orf1	blue	0.88141
TRINITY_DN11970_c0_g1_i4_orf1	blue	0.96596
TRINITY_DN22956_c0_g1_i1_orf1	turquoise	0.76309
TRINITY_DN31980_c0_g1_i1_orf1	blue	0.62537
TRINITY_DN5170_c0_g1_i5_orf1	black	0.78168
TRINITY_DN1215_c0_g1_i2_orf1	yellow	0.75047
TRINITY_DN2684_c0_g2_i3_orf1	blue	0.95702
TRINITY_DN24132_c0_g1_i2_orf1	blue	0.7084
TRINITY_DN20344_c0_g1_i5_orf1	brown	0.91406
TRINITY_DN52761_c0_g2_i1_orf1	brown	0.76824
TRINITY_DN12222_c0_g1_i1_orf1	red	0.84433
TRINITY_DN55160_c0_g2_i1_orf1	turquoise	0.94956
TRINITY_DN4257_c0_g1_i2_orf1	turquoise	0.33191
TRINITY_DN1097_c0_g1_i1_orf1	turquoise	0.70548
TRINITY_DN23582_c0_g1_i1_orf1	blue	0.85711
TRINITY_DN44219_c0_g1_i1_orf1	turquoise	0.94438
TRINITY_DN714_c0_g1_i3_orf1	blue	0.97614
TRINITY_DN3534_c0_g1_i2_orf1	turquoise	0.97975
TRINITY_DN86833_c0_g3_i1_orf1	blue	0.82541
TRINITY_DN452_c1_g1_i3_orf1	turquoise	0.93468
TRINITY_DN2668_c0_g1_i7_orf1	turquoise	0.95131
TRINITY_DN39532_c0_g1_i1_orf1	turquoise	0.8999
TRINITY_DN8646_c0_g1_i2_orf1	turquoise	0.3763
TRINITY_DN24024_c0_g1_i1_orf1	turquoise	0.67355
TRINITY_DN20347_c0_g1_i6_orf1	brown	0.80612
TRINITY_DN35991_c0_g1_i2_orf1	brown	0.71306
TRINITY_DN29_c0_g1_i4_orf1	turquoise	0.3985
TRINITY_DN8037_c0_g2_i1_orf1	black	0.91549
TRINITY_DN2314_c0_g1_i7_orf1	green	0.9361
TRINITY_DN5770_c0_g1_i4_orf1	yellow	0.35841
TRINITY_DN53233_c0_g1_i1_orf1	blue	0.61045
TRINITY_DN1771_c0_g2_i1_orf1	turquoise	0.78642
TRINITY_DN103457_c0_g1_i1_orf1	turquoise	0.95165
TRINITY_DN64769_c0_g1_i3_orf1	red	0.96492
TRINITY_DN9538_c1_g3_i1_orf1	green	0.92868
TRINITY_DN8139_c0_g1_i2_orf1	black	0.70947
TRINITY_DN1252_c0_g1_i3_orf1	blue	0.91063
TRINITY_DN4538_c0_g1_i4_orf1	turquoise	0.89236
TRINITY_DN94475_c0_g1_i1_orf1	turquoise	0.46826
TRINITY_DN40281_c0_g1_i1_orf1	brown	0.70448
TRINITY_DN29217_c0_g1_i3_orf1	green	0.92371
TRINITY_DN25870_c0_g2_i6_orf1	red	0.90079
TRINITY_DN2559_c0_g1_i4_orf1	yellow	0.93878
TRINITY_DN98016_c0_g1_i1_orf1	blue	0.91769
TRINITY_DN142652_c0_g1_i1_orf1	turquoise	0.92058
TRINITY_DN1597_c0_g1_i5_orfp1	green	0.89665
TRINITY_DN13018_c0_g1_i1_orf1	turquoise	0.67156
TRINITY_DN5118_c0_g1_i1_orf1	red	0.83469
TRINITY_DN2038_c0_g1_i2_orf1	turquoise	0.77361
TRINITY_DN20009_c0_g1_i1_orf1	green	0.78897
TRINITY_DN12826_c0_g1_i1_orf1	turquoise	0.90082
TRINITY_DN20323_c0_g1_i1_orf1	turquoise	0.63086
TRINITY_DN36648_c0_g1_i1_orf1	turquoise	0.97535
TRINITY_DN11566_c0_g1_i6_orf1	red	0.63139
TRINITY_DN19361_c0_g1_i7_orf1	turquoise	0.97913
TRINITY_DN8569_c1_g2_i7_orf1	blue	0.88506
TRINITY_DN36701_c0_g1_i4_orf1	turquoise	0.97006
TRINITY_DN87803_c0_g1_i2_orf1	blue	0.95918

TRINITY_DN12432_c0_g1_i2_orf1	black	0.47276
TRINITY_DN1252_c0_g1_i1_orf1	brown	0.6945
TRINITY_DN22983_c0_g1_i2_orfp1	turquoise	0.99755
TRINITY_DN47723_c0_g1_i1_orf1	turquoise	0.75968
TRINITY_DN9558_c0_g1_i2_orf1	turquoise	0.86435
TRINITY_DN1048_c0_g1_i6_orf1	green	0.90116
TRINITY_DN60792_c0_g1_i2_orf1	yellow	0.80929
TRINITY_DN50471_c0_g1_i4_orf1	blue	0.93748
TRINITY_DN11084_c1_g1_i2_orf1	blue	0.68252
TRINITY_DN8369_c0_g1_i1_orf1	turquoise	0.9762
TRINITY_DN35162_c0_g1_i4_orf1	yellow	0.40503
TRINITY_DN113327_c0_g1_i2_orf1	green	0.52429
TRINITY_DN144807_c0_g1_i1_orf1	turquoise	0.98443
TRINITY_DN14934_c0_g1_i17_orf1	turquoise	0.92004
TRINITY_DN6025_c0_g2_i1_orfp1	brown	0.94362
TRINITY_DN12256_c0_g1_i1_orf1	black	0.98123
TRINITY_DN14487_c0_g1_i4_orf1	turquoise	0.90605
TRINITY_DN30638_c0_g1_i1_orf1	turquoise	0.9887
TRINITY_DN27321_c0_g1_i1_orf1	red	0.95311
TRINITY_DN1249_c0_g1_i6_orf1	turquoise	0.86305
TRINITY_DN33485_c0_g1_i4_orf1	turquoise	0.59648
TRINITY_DN109540_c0_g1_i3_orf1	turquoise	0.98365
TRINITY_DN6317_c1_g2_i3_orf1	turquoise	0.87377
TRINITY_DN8245_c0_g1_i4_orf1	green	0.8804
TRINITY_DN6087_c0_g1_i7_orf1	turquoise	0.981
TRINITY_DN2876_c0_g1_i1_orf1	brown	0.82947
TRINITY_DN25234_c0_g1_i1_orf1	black	0.84991
TRINITY_DN22747_c0_g1_i5_orf1	turquoise	0.79013
TRINITY_DN3984_c0_g1_i4_orf1	turquoise	0.5801
TRINITY_DN3562_c0_g1_i4_orf1	turquoise	0.86024
TRINITY_DN9282_c0_g1_i2_orf1	blue	0.9229
TRINITY_DN92232_c0_g1_i1_orf1	turquoise	0.95952
TRINITY_DN7106_c0_g1_i5_orf1	brown	0.67737
TRINITY_DN129_c0_g1_i6_orf1	turquoise	0.46704
TRINITY_DN4456_c0_g1_i1_orf1	blue	0.62279
TRINITY_DN28981_c0_g1_i1_orf1	turquoise	0.96959
TRINITY_DN29579_c0_g1_i1_orf1	blue	0.7211
TRINITY_DN146119_c0_g1_i1_orf1	red	0.84546
TRINITY_DN2986_c1_g1_i1_orf1	turquoise	0.93387
TRINITY_DN698_c0_g1_i5_orf1	turquoise	0.97874
TRINITY_DN42205_c0_g1_i4_orf1	red	0.8527
TRINITY_DN56121_c0_g1_i4_orf1	turquoise	0.85876
TRINITY_DN12997_c0_g2_i1_orf1	yellow	0.76338
TRINITY_DN5124_c0_g1_i2_orf1	green	0.72283
TRINITY_DN24391_c1_g1_i1_orf1	red	0.78331
TRINITY_DN25273_c0_g1_i1_orf1	blue	0.99814
TRINITY_DN2019_c0_g1_i4_orf1	turquoise	0.83248
TRINITY_DN9302_c0_g1_i1_orf1	blue	0.91698
TRINITY_DN24142_c0_g1_i1_orf1	turquoise	0.92378
TRINITY_DN5467_c0_g1_i5_orf1	blue	0.94696
TRINITY_DN26853_c0_g1_i1_orf1	turquoise	0.62139
TRINITY_DN80245_c0_g1_i1_orf1	turquoise	0.7503
TRINITY_DN12134_c0_g1_i4_orf1	red	0.98331
TRINITY_DN3119_c0_g1_i7_orf1	blue	0.81024
TRINITY_DN1045_c0_g1_i6_orf1	turquoise	0.69486
TRINITY_DN5848_c0_g1_i6_orf1	blue	0.75531
TRINITY_DN50725_c0_g1_i6_orf1	yellow	0.53608
TRINITY_DN2798_c0_g1_i5_orf1	green	0.93235

TRINITY_DN9286_c0_g1_i2_orf1	yellow	0.84371
TRINITY_DN98091_c0_g1_i3_orf1	turquoise	0.97673
TRINITY_DN9457_c0_g1_i9_orf1	black	0.90356
TRINITY_DN19807_c0_g1_i1_orf1	brown	0.88974
TRINITY_DN44633_c0_g1_i4_orf1	blue	0.66072
TRINITY_DN1831_c0_g1_i3_orf1	red	0.88951
TRINITY_DN84322_c0_g2_i1_orf1	turquoise	0.96335
TRINITY_DN8824_c0_g2_i1_orf1	turquoise	0.92681
TRINITY_DN120439_c1_g1_i1_orf1	yellow	0.7429
TRINITY_DN12920_c0_g3_i1_orf1	yellow	0.52521
TRINITY_DN38783_c0_g1_i1_orf1	brown	0.84413
TRINITY_DN1902_c0_g1_i4_orf1	blue	0.96988
TRINITY_DN13368_c0_g1_i1_orf1	turquoise	0.98031
TRINITY_DN22009_c0_g1_i1_orf1	grey	-0.63177
TRINITY_DN18118_c0_g2_i10_orf1	blue	0.63074
TRINITY_DN56690_c0_g1_i4_orf1	blue	0.72321
TRINITY_DN4394_c0_g2_i1_orf1	green	0.86375
TRINITY_DN22951_c0_g1_i1_orf1	turquoise	0.70693
TRINITY_DN938_c0_g1_i7_orf1	black	0.81512
TRINITY_DN2394_c0_g1_i4_orf1	turquoise	0.98765
TRINITY_DN371_c0_g1_i6_orf1	turquoise	0.83496
TRINITY_DN3909_c0_g2_i2_orf1	turquoise	0.89932
TRINITY_DN3856_c0_g1_i7_orf1	turquoise	0.87576
TRINITY_DN4156_c0_g1_i2_orf1	red	0.97353
TRINITY_DN2254_c0_g1_i4_orf1	yellow	0.82564
TRINITY_DN109733_c0_g1_i1_orf1	turquoise	0.95863
TRINITY_DN26337_c0_g1_i3_orf1	blue	0.86077
TRINITY_DN3113_c1_g2_i1_orf1	turquoise	0.87941
TRINITY_DN64892_c0_g1_i1_orf1	green	0.91795
TRINITY_DN248_c0_g1_i1_orf1	yellow	0.92404
TRINITY_DN5512_c0_g1_i8_orf1	turquoise	0.96938
TRINITY_DN1099_c1_g1_i2_orf1	red	0.61956
TRINITY_DN26130_c0_g1_i1_orf1	turquoise	0.95677
TRINITY_DN21567_c0_g1_i7_orf1	brown	0.35618
TRINITY_DN9741_c0_g1_i3_orf1	turquoise	0.94716
TRINITY_DN21559_c0_g2_i1_orf1	red	0.92453
TRINITY_DN4920_c0_g1_i5_orf1	yellow	0.77589
TRINITY_DN1853_c0_g1_i3_orf1	red	0.89479
TRINITY_DN7407_c0_g1_i9_orf1	turquoise	0.86711
TRINITY_DN6169_c0_g1_i15_orf1	turquoise	0.77248
TRINITY_DN4565_c0_g1_i3_orf1	green	0.83003
TRINITY_DN40028_c0_g1_i1_orf1	turquoise	0.86901
TRINITY_DN4923_c0_g1_i4_orf1	turquoise	0.96975
TRINITY_DN1492_c0_g1_i4_orf1	red	0.6038
TRINITY_DN2098_c0_g1_i1_orf1	yellow	0.61684
TRINITY_DN1628_c0_g2_i3_orf1	green	0.4724
TRINITY_DN8747_c0_g1_i2_orf1	brown	0.91312
TRINITY_DN6698_c0_g2_i1_orf1	yellow	0.76161
TRINITY_DN14996_c0_g1_i2_orf1	turquoise	0.92005
TRINITY_DN661_c0_g3_i5_orf1	blue	0.94578
TRINITY_DN14235_c0_g1_i1_orf1	turquoise	0.97587
TRINITY_DN821_c0_g1_i8_orf1	turquoise	0.90286
TRINITY_DN987_c0_g1_i11_orf1	turquoise	0.50302
TRINITY_DN4380_c0_g1_i9_orf1	turquoise	0.90642
TRINITY_DN96884_c0_g1_i1_orf1	turquoise	0.66412
TRINITY_DN39404_c0_g1_i7_orf1	blue	0.80421
TRINITY_DN10266_c0_g1_i5_orf1	turquoise	0.79374
TRINITY_DN12865_c0_g1_i1_orf1	blue	0.89816

TRINITY_DN137_c0_g1_i1_orf1	turquoise	0.93284
TRINITY_DN863_c0_g1_i6_orf1	turquoise	0.72489
TRINITY_DN2170_c4_g1_i2_orf1	green	0.91002
TRINITY_DN35002_c0_g2_i2_orf1	yellow	0.42026
TRINITY_DN3732_c0_g1_i6_orf1	yellow	0.73675
TRINITY_DN18027_c0_g2_i1_orf1	blue	0.90706
TRINITY_DN11928_c0_g1_i3_orf1	turquoise	0.95328
TRINITY_DN1073_c0_g1_i1_orf1	turquoise	0.82721
TRINITY_DN35669_c0_g1_i1_orf1	turquoise	0.8937
TRINITY_DN105055_c0_g1_i1_orfp1	turquoise	0.90954
TRINITY_DN934_c2_g1_i7_orf1	red	0.92254
TRINITY_DN47842_c0_g1_i1_orf1	blue	0.65102
TRINITY_DN6503_c0_g1_i8_orf1	turquoise	0.88122
TRINITY_DN38431_c0_g1_i1_orf1	blue	0.97329
TRINITY_DN1344_c0_g1_i1_orf1	brown	0.75569
TRINITY_DN4134_c2_g1_i2_orf1	blue	0.92644
TRINITY_DN63536_c0_g1_i1_orf1	brown	0.83093
TRINITY_DN68770_c0_g1_i1_orf1	brown	0.65778
TRINITY_DN22928_c0_g1_i6_orf1	black	0.86393
TRINITY_DN27771_c0_g2_i1_orf1	brown	0.87495
TRINITY_DN1038_c0_g1_i4_orf1	blue	0.69678
TRINITY_DN700_c0_g1_i3_orf1	turquoise	0.98217
TRINITY_DN4320_c0_g1_i1_orf1	blue	0.9262
TRINITY_DN65604_c0_g1_i2_orf1	turquoise	0.94147
TRINITY_DN4752_c0_g1_i3_orf1	turquoise	0.95309
TRINITY_DN17657_c0_g1_i1_orf1	yellow	0.7732
TRINITY_DN5753_c0_g1_i10_orf1	turquoise	0.73946
TRINITY_DN5422_c0_g1_i1_orf1	blue	0.50544
TRINITY_DN4822_c0_g1_i6_orf1	green	0.72649
TRINITY_DN50724_c0_g2_i1_orf1	turquoise	0.9647
TRINITY_DN22053_c0_g1_i13_orf1	blue	0.86865
TRINITY_DN91_c0_g1_i9_orf1	turquoise	0.89744
TRINITY_DN95056_c0_g2_i2_orf1	turquoise	0.33021
TRINITY_DN2350_c0_g1_i6_orf1	yellow	0.78415
TRINITY_DN3582_c0_g1_i2_orf1	turquoise	0.87988
TRINITY_DN21722_c0_g1_i3_orf1	turquoise	0.98992
TRINITY_DN1772_c7_g1_i7_orf1	turquoise	0.9337
TRINITY_DN5667_c0_g1_i4_orf1	green	0.8756
TRINITY_DN2395_c0_g1_i7_orf1	green	0.85366
TRINITY_DN47123_c0_g1_i1_orf1	turquoise	0.96933
TRINITY_DN11587_c0_g1_i7_orf1	turquoise	0.99488
TRINITY_DN4008_c0_g1_i7_orf1	turquoise	0.90709
TRINITY_DN4905_c0_g1_i6_orf1	turquoise	0.93414
TRINITY_DN131603_c0_g1_i4_orfp1	brown	0.49987
TRINITY_DN28509_c0_g1_i1_orf1	turquoise	0.89556
TRINITY_DN9592_c0_g1_i2_orf1	red	0.69955
TRINITY_DN57904_c0_g2_i1_orf1	red	0.98049
TRINITY_DN286_c0_g1_i2_orf1	red	0.93265
TRINITY_DN24164_c0_g1_i1_orf1	turquoise	0.86409
TRINITY_DN40508_c0_g1_i1_orf1	turquoise	0.86969
TRINITY_DN14301_c0_g1_i1_orf1	turquoise	0.83922
TRINITY_DN38412_c0_g1_i1_orf1	blue	0.84224
TRINITY_DN66302_c0_g1_i1_orf1	turquoise	0.97549
TRINITY_DN27276_c0_g1_i5_orf1	turquoise	0.52524
TRINITY_DN7976_c0_g1_i4_orf1	yellow	0.64845
TRINITY_DN1177_c0_g1_i4_orf1	yellow	0.94455
TRINITY_DN7655_c0_g1_i3_orf1	turquoise	0.96059
TRINITY_DN11448_c0_g1_i4_orf1	turquoise	0.6918

TRINITY_DN2919_c0_g1_i5_orf1	red	0.84002
TRINITY_DN28660_c0_g1_i4_orf1	turquoise	0.36597
TRINITY_DN2433_c0_g1_i3_orf1	yellow	0.68518
TRINITY_DN53246_c0_g7_i1_orf1	turquoise	0.5497
TRINITY_DN3450_c0_g1_i3_orf1	turquoise	0.84117
TRINITY_DN2461_c0_g1_i5_orf1	brown	0.97906
TRINITY_DN2827_c3_g1_i3_orf1	turquoise	0.313
TRINITY_DN9694_c0_g1_i1_orf1	yellow	0.84041
TRINITY_DN9468_c1_g1_i4_orf1	yellow	0.93944
TRINITY_DN6497_c0_g1_i1_orf1	green	0.96014
TRINITY_DN120_c0_g1_i2_orf1	yellow	0.93344
TRINITY_DN679_c0_g1_i2_orf1	turquoise	0.99259
TRINITY_DN5417_c0_g1_i1_orf1	turquoise	0.99464
TRINITY_DN230_c2_g1_i5_orf1	yellow	0.76481
TRINITY_DN10257_c0_g1_i2_orf1	turquoise	0.88913
TRINITY_DN147596_c0_g1_i1_orf1	turquoise	0.88012
TRINITY_DN19293_c0_g1_i4_orf1	pink	0.77768
TRINITY_DN15930_c0_g1_i5_orf1	blue	0.53594
TRINITY_DN19998_c0_g1_i1_orf1	blue	0.93502
TRINITY_DN56459_c0_g1_i2_orf1	brown	0.98988
TRINITY_DN1074_c0_g1_i7_orf1	red	0.98045
TRINITY_DN27300_c0_g1_i1_orfp1	yellow	0.73642
TRINITY_DN1175_c1_g1_i1_orf1	black	0.96108
TRINITY_DN2813_c0_g1_i7_orf1	black	0.98488
TRINITY_DN1292_c0_g1_i3_orf1	pink	0.93669
TRINITY_DN17864_c0_g1_i1_orf1	turquoise	0.97928
TRINITY_DN71832_c0_g1_i1_orf1	blue	0.69921
TRINITY_DN4451_c0_g2_i4_orf1	turquoise	0.81597
TRINITY_DN57798_c0_g1_i1_orf1	turquoise	0.91145
TRINITY_DN52316_c0_g1_i1_orf1	green	0.97628
TRINITY_DN467_c3_g1_i5_orf1	green	0.92358
TRINITY_DN60821_c0_g1_i1_orf1	turquoise	0.66947
TRINITY_DN66453_c0_g1_i4_orfp1	brown	0.88716
TRINITY_DN14507_c0_g1_i5_orf1	red	0.85787
TRINITY_DN7549_c0_g1_i1_orf1	blue	0.93971
TRINITY_DN52296_c0_g1_i6_orf1	turquoise	0.95869
TRINITY_DN483_c0_g1_i6_orf1	brown	0.48636
TRINITY_DN29144_c0_g3_i1_orf1	black	0.352
TRINITY_DN172_c8_g2_i1_orf1	red	0.79825
TRINITY_DN117_c0_g1_i6_orf1	turquoise	0.98209
TRINITY_DN19079_c0_g1_i5_orf1	turquoise	0.85863
TRINITY_DN14112_c0_g1_i3_orf1	brown	0.91891
TRINITY_DN13576_c0_g1_i1_orf1	blue	0.95892
TRINITY_DN7560_c0_g1_i4_orf1	red	0.87343
TRINITY_DN11457_c0_g1_i5_orf1	turquoise	0.94638
TRINITY_DN6004_c0_g1_i1_orf1	blue	0.996
TRINITY_DN141_c0_g1_i1_orf1	turquoise	0.89787
TRINITY_DN5682_c0_g1_i6_orf1	brown	0.83837
TRINITY_DN20560_c0_g1_i6_orf1	black	0.96433
TRINITY_DN2401_c0_g2_i1_orf1	yellow	0.71911
TRINITY_DN1703_c0_g1_i6_orf1	blue	0.96976
TRINITY_DN4064_c0_g2_i1_orf1	brown	0.61666
TRINITY_DN61536_c0_g3_i1_orf1	yellow	0.42651
TRINITY_DN3292_c2_g2_i1_orf1	blue	0.83763
TRINITY_DN1443_c0_g1_i4_orf1	turquoise	0.96034
TRINITY_DN741_c0_g1_i10_orf1	brown	0.85801
TRINITY_DN21856_c0_g1_i1_orf1	black	0.96667
TRINITY_DN18136_c0_g1_i1_orf1	black	0.68867

TRINITY_DN4689_c0_g1_i5_orf1	blue	0.90686
TRINITY_DN10110_c1_g2_i1_orf1	red	0.74649
TRINITY_DN862_c0_g1_i4_orf1	blue	0.83624
TRINITY_DN107840_c1_g1_i1_orf1	turquoise	0.95044
TRINITY_DN23398_c0_g1_i1_orf1	green	0.94022
TRINITY_DN556_c0_g1_i4_orf1	turquoise	0.47061
TRINITY_DN920_c0_g1_i4_orf1	yellow	0.76736
TRINITY_DN12495_c0_g1_i2_orf1	turquoise	0.99184
TRINITY_DN6535_c0_g2_i1_orf1	turquoise	0.98491
TRINITY_DN31232_c1_g1_i9_orf1	turquoise	0.90378
TRINITY_DN33705_c0_g1_i1_orf1	brown	0.70859
TRINITY_DN976_c0_g1_i5_orf1	green	0.64929
TRINITY_DN4408_c6_g1_i1_orf1	yellow	0.83657
TRINITY_DN24917_c0_g2_i1_orf1	turquoise	0.78246
TRINITY_DN2170_c0_g1_i2_orf1	blue	0.67771
TRINITY_DN2847_c0_g1_i20_orf1	brown	0.86157
TRINITY_DN64297_c0_g1_i1_orf1	blue	0.80867
TRINITY_DN65247_c1_g1_i1_orf1	green	0.81518
TRINITY_DN8940_c0_g1_i4_orf1	turquoise	0.75975
TRINITY_DN59965_c0_g4_i1_orf1	green	0.79675
TRINITY_DN478_c0_g1_i16_orf1	turquoise	0.92893
TRINITY_DN21961_c0_g2_i5_orf1	turquoise	0.38757
TRINITY_DN51658_c0_g1_i1_orf1	grey	-0.61265
TRINITY_DN1039_c0_g1_i5_orf1	pink	0.64799
TRINITY_DN6418_c0_g1_i28_orf1	turquoise	0.97325
TRINITY_DN4248_c0_g1_i4_orf1	brown	0.40813
TRINITY_DN57_c0_g2_i3_orf1	turquoise	0.57497
TRINITY_DN8008_c0_g1_i6_orf1	brown	0.93367
TRINITY_DN28501_c0_g1_i2_orfp1	yellow	0.84629
TRINITY_DN17505_c0_g1_i15_orf1	brown	0.53023
TRINITY_DN30673_c0_g1_i5_orf1	red	0.63301
TRINITY_DN55147_c0_g1_i1_orfp1	yellow	0.83142
TRINITY_DN19460_c0_g1_i1_orf1	yellow	0.94249
TRINITY_DN146181_c0_g1_i1_orf1	brown	0.84581
TRINITY_DN3665_c0_g1_i2_orf1	turquoise	0.98455
TRINITY_DN14018_c0_g1_i4_orf1	turquoise	0.93451
TRINITY_DN12673_c3_g1_i2_orf1	blue	0.93401
TRINITY_DN350_c0_g1_i5_orf1	brown	0.92731
TRINITY_DN11856_c0_g1_i4_orf1	red	0.41164
TRINITY_DN3504_c0_g1_i3_orfp2	turquoise	0.80388
TRINITY_DN2718_c0_g1_i6_orf1	red	0.89552
TRINITY_DN3619_c0_g2_i1_orf1	turquoise	0.90366
TRINITY_DN7682_c0_g1_i2_orf1	blue	0.83632
TRINITY_DN1763_c0_g3_i2_orf1	turquoise	0.67424
TRINITY_DN8682_c0_g1_i4_orf1	blue	0.78378
TRINITY_DN8644_c0_g1_i3_orf1	brown	0.42882
TRINITY_DN62729_c0_g1_i13_orf1	blue	0.57503
TRINITY_DN110460_c0_g2_i1_orf1	turquoise	0.694
TRINITY_DN19261_c0_g1_i3_orf1	brown	0.86615
TRINITY_DN15737_c0_g1_i7_orf1	turquoise	0.88081
TRINITY_DN38366_c0_g1_i4_orfp1	yellow	0.85219
TRINITY_DN10630_c0_g1_i2_orf1	blue	0.73874
TRINITY_DN1391_c0_g1_i29_orfp1	brown	0.93416
TRINITY_DN5767_c0_g1_i4_orf1	turquoise	0.9349
TRINITY_DN5460_c0_g1_i5_orf1	red	0.50345
TRINITY_DN3647_c1_g1_i5_orf1	black	0.95838
TRINITY_DN61048_c0_g1_i2_orf1	black	0.9049
TRINITY_DN34689_c0_g1_i4_orf1	turquoise	0.8534

TRINITY_DN1424_c0_g1_i5_orf1	brown	0.89219
TRINITY_DN4053_c0_g1_i5_orf1	yellow	0.84274
TRINITY_DN82311_c0_g1_i1_orf1	turquoise	0.49997
TRINITY_DN381_c0_g1_i1_orf1	blue	0.96337
TRINITY_DN389_c0_g1_i2_orf1	red	0.88969
TRINITY_DN1989_c0_g1_i1_orf1	pink	0.7213
TRINITY_DN7040_c0_g2_i1_orf1	green	0.98143
TRINITY_DN4300_c0_g1_i5_orf1	turquoise	0.90278
TRINITY_DN7754_c0_g1_i2_orf1	black	0.94596
TRINITY_DN3175_c0_g1_i7_orf1	brown	0.97486
TRINITY_DN8660_c0_g1_i1_orf1	blue	0.61189
TRINITY_DN24322_c0_g1_i4_orf1	yellow	0.95004
TRINITY_DN21930_c0_g1_i1_orf1	blue	0.91433
TRINITY_DN20527_c0_g1_i1_orf1	turquoise	0.54426
TRINITY_DN2083_c0_g1_i4_orf1	turquoise	0.95999
TRINITY_DN1308_c0_g1_i4_orf1	blue	0.96589
TRINITY_DN48413_c1_g1_i2_orf1	turquoise	0.80379
TRINITY_DN14286_c0_g1_i5_orf1	turquoise	0.91684
TRINITY_DN26790_c0_g1_i3_orf1	turquoise	0.64235
TRINITY_DN2624_c0_g1_i6_orf1	brown	0.93753
TRINITY_DN14611_c0_g1_i5_orf1	brown	0.89852
TRINITY_DN104663_c1_g1_i2_orf1	green	0.95699
TRINITY_DN5510_c0_g1_i9_orf1	yellow	0.93866
TRINITY_DN2802_c0_g1_i1_orf1	turquoise	0.81912
TRINITY_DN768_c0_g1_i7_orf1	green	0.92606
TRINITY_DN8654_c0_g1_i1_orf1	green	0.64763
TRINITY_DN68725_c0_g1_i1_orf1	turquoise	0.78833
TRINITY_DN4081_c0_g1_i1_orf1	turquoise	0.3117
TRINITY_DN29743_c0_g1_i9_orf1	red	0.89982
TRINITY_DN114960_c0_g1_i4_orf1	blue	0.93312
TRINITY_DN3478_c0_g1_i10_orf1	turquoise	0.43949
TRINITY_DN59042_c1_g1_i1_orf1	turquoise	0.94135
TRINITY_DN1084_c0_g2_i2_orf1	yellow	0.73958
TRINITY_DN1381_c0_g1_i5_orf1	yellow	0.80728
TRINITY_DN11973_c0_g1_i1_orf1	turquoise	0.93219
TRINITY_DN8083_c0_g1_i1_orf1	blue	0.79066
TRINITY_DN5648_c0_g1_i5_orf1	turquoise	0.95487
TRINITY_DN19829_c0_g1_i1_orf1	turquoise	0.92226
TRINITY_DN4314_c0_g1_i9_orf1	brown	0.9109
TRINITY_DN230_c1_g1_i3_orf1	brown	0.66684
TRINITY_DN13177_c0_g1_i9_orf1	black	0.56528
TRINITY_DN4040_c0_g1_i10_orf1	turquoise	0.97518
TRINITY_DN129808_c0_g1_i1_orf1	turquoise	0.76753
TRINITY_DN13760_c1_g1_i1_orf1	blue	0.82363
TRINITY_DN98692_c0_g3_i1_orf1	black	0.97833
TRINITY_DN36718_c0_g1_i1_orf1	turquoise	0.9735
TRINITY_DN1227_c0_g1_i1_orf1	turquoise	0.88783
TRINITY_DN334_c0_g1_i4_orf1	turquoise	0.9841
TRINITY_DN17409_c0_g1_i5_orf1	grey	-0.36814
TRINITY_DN8853_c0_g1_i4_orf1	brown	0.80438
TRINITY_DN66287_c0_g1_i1_orfp1	yellow	0.60072
TRINITY_DN1814_c0_g1_i11_orf1	yellow	0.88114
TRINITY_DN713_c0_g1_i4_orf1	turquoise	0.98888
TRINITY_DN639_c0_g1_i10_orf1	turquoise	0.68685
TRINITY_DN23204_c0_g1_i1_orf1	turquoise	0.69869
TRINITY_DN12885_c0_g1_i1_orf1	green	0.82118
TRINITY_DN5086_c0_g1_i1_orf1	turquoise	0.87665
TRINITY_DN8637_c0_g1_i1_orf1	green	0.94784

TRINITY_DN14019_c0_g1_i5_orf1	red	0.76639
TRINITY_DN16127_c0_g1_i2_orf1	green	0.69893
TRINITY_DN647_c4_g1_i1_orf1	turquoise	0.9448
TRINITY_DN6027_c0_g1_i13_orf1	turquoise	0.6477
TRINITY_DN870_c0_g1_i3_orf1	yellow	0.93693
TRINITY_DN32487_c0_g1_i1_orf1	turquoise	0.98912
TRINITY_DN4085_c0_g1_i1_orf1	red	0.78659
TRINITY_DN69707_c0_g1_i1_orf1	yellow	0.92938
TRINITY_DN39975_c0_g1_i4_orf1	red	0.80812
TRINITY_DN27300_c0_g1_i7_orfp1	brown	0.79528
TRINITY_DN1362_c0_g1_i4_orf1	blue	0.87078
TRINITY_DN6785_c0_g1_i1_orf1	turquoise	0.71143
TRINITY_DN13941_c0_g1_i6_orf1	turquoise	0.69021
TRINITY_DN5210_c0_g1_i3_orf1	turquoise	0.65617
TRINITY_DN94625_c0_g1_i1_orf1	turquoise	0.98727
TRINITY_DN3826_c0_g1_i1_orf1	brown	0.78338
TRINITY_DN27723_c0_g1_i1_orf1	red	0.9477
TRINITY_DN8922_c0_g1_i3_orf1	green	0.84122
TRINITY_DN50517_c0_g1_i1_orf1	black	0.95412
TRINITY_DN7776_c0_g1_i9_orf1	black	0.9561
TRINITY_DN3355_c0_g1_i1_orf1	turquoise	0.79603
TRINITY_DN1694_c0_g1_i1_orf1	green	0.87761
TRINITY_DN8726_c0_g2_i3_orf1	black	0.87912
TRINITY_DN26569_c0_g1_i4_orf1	green	0.53063
TRINITY_DN15513_c0_g1_i6_orf1	turquoise	0.83605
TRINITY_DN57536_c0_g1_i14_orf1	black	0.76198
TRINITY_DN1926_c0_g1_i5_orf1	brown	0.92646
TRINITY_DN16187_c0_g1_i1_orf1	turquoise	0.86128
TRINITY_DN33801_c0_g1_i1_orf1	turquoise	0.9498
TRINITY_DN1226_c0_g1_i11_orfp1	brown	0.91415
TRINITY_DN41280_c0_g1_i2_orf1	pink	0.88842
TRINITY_DN2594_c0_g2_i4_orf1	turquoise	0.98782
TRINITY_DN1268_c0_g1_i1_orf1	turquoise	0.89766
TRINITY_DN9647_c0_g1_i1_orf1	turquoise	0.95526
TRINITY_DN2879_c0_g1_i4_orf1	red	0.90104
TRINITY_DN3377_c0_g1_i1_orf1	brown	0.7383
TRINITY_DN4694_c0_g2_i1_orf1	blue	0.87017
TRINITY_DN2196_c0_g1_i2_orf1	turquoise	0.82056
TRINITY_DN1897_c0_g2_i4_orf1	black	0.61059
TRINITY_DN51776_c0_g1_i1_orf1	brown	0.87308
TRINITY_DN1355_c0_g1_i7_orf1	green	0.9744
TRINITY_DN26879_c0_g1_i1_orf1	brown	0.75851
TRINITY_DN4276_c0_g1_i6_orf1	blue	0.81816
TRINITY_DN22836_c0_g1_i5_orf1	turquoise	0.76502
TRINITY_DN2627_c0_g1_i2_orf1	turquoise	0.97314
TRINITY_DN51045_c0_g1_i1_orf1	turquoise	0.98704
TRINITY_DN30950_c0_g1_i13_orf1	turquoise	0.87445
TRINITY_DN2807_c0_g1_i4_orf1	turquoise	0.97577
TRINITY_DN313_c0_g1_i5_orf1	turquoise	0.89418
TRINITY_DN52944_c0_g1_i1_orf1	blue	0.85353
TRINITY_DN14920_c0_g1_i1_orf1	turquoise	0.92243
TRINITY_DN20_c0_g1_i11_orf1	turquoise	0.96073
TRINITY_DN1481_c0_g1_i4_orf1	blue	0.80766
TRINITY_DN960_c1_g1_i6_orf1	black	0.96301
TRINITY_DN52887_c0_g1_i1_orf1	grey	0.7408
TRINITY_DN36856_c0_g1_i1_orf1	blue	0.89698
TRINITY_DN8076_c0_g1_i5_orf1	turquoise	0.82795
TRINITY_DN27771_c0_g1_i1_orf1	turquoise	0.75552

TRINITY_DN2473_c0_g1_i2_orf1	turquoise	0.5397
TRINITY_DN21872_c0_g1_i2_orf1	turquoise	0.72447
TRINITY_DN130159_c0_g2_i1_orf1	turquoise	0.77226
TRINITY_DN72816_c0_g1_i2_orf1	red	0.97662
TRINITY_DN43293_c0_g1_i2_orf1	turquoise	0.733
TRINITY_DN3747_c1_g1_i3_orf1	turquoise	0.93055
TRINITY_DN6058_c0_g1_i3_orf1	brown	0.94941
TRINITY_DN10672_c0_g1_i3_orf1	turquoise	0.82979
TRINITY_DN5919_c0_g1_i4_orf1	turquoise	0.9674
TRINITY_DN1543_c0_g2_i2_orf1	brown	0.63732
TRINITY_DN19521_c0_g1_i1_orf1	brown	0.82658
TRINITY_DN1450_c0_g2_i1_orf1	brown	0.95248
TRINITY_DN92153_c0_g2_i2_orf1	turquoise	0.67116
TRINITY_DN11735_c0_g1_i5_orf1	yellow	0.70915
TRINITY_DN883_c0_g1_i8_orf1	turquoise	0.88711
TRINITY_DN8603_c0_g1_i1_orf1	turquoise	0.66174
TRINITY_DN31619_c0_g1_i2_orf1	blue	0.87483
TRINITY_DN24490_c0_g1_i6_orf1	turquoise	0.62869
TRINITY_DN11817_c0_g1_i4_orf1	brown	0.92102
TRINITY_DN1132_c0_g1_i5_orf1	turquoise	0.97349
TRINITY_DN2187_c0_g1_i1_orf1	brown	0.96579
TRINITY_DN8258_c0_g1_i6_orf1	yellow	0.88091
TRINITY_DN7688_c0_g1_i2_orf1	turquoise	0.98044
TRINITY_DN73945_c0_g5_i3_orf1	turquoise	0.86238
TRINITY_DN15870_c0_g1_i3_orf1	turquoise	0.98719
TRINITY_DN96566_c0_g1_i1_orf1	turquoise	0.93702
TRINITY_DN10639_c0_g1_i6_orf1	turquoise	0.74277
TRINITY_DN61777_c0_g1_i4_orf1	green	0.94372
TRINITY_DN20499_c0_g1_i1_orf1	turquoise	0.97269
TRINITY_DN18291_c0_g1_i1_orf1	green	0.73922
TRINITY_DN2290_c0_g1_i2_orfp1	blue	0.88828
TRINITY_DN52395_c0_g2_i2_orf1	yellow	0.95266
TRINITY_DN3179_c0_g1_i1_orf1	turquoise	0.81283
TRINITY_DN5208_c0_g1_i7_orf1	blue	0.5727
TRINITY_DN25997_c1_g1_i1_orf1	turquoise	0.87362
TRINITY_DN547_c0_g1_i1_orf1	turquoise	0.91161
TRINITY_DN70_c2_g1_i1_orf1	turquoise	0.66136
TRINITY_DN21531_c0_g1_i1_orf1	turquoise	0.9111
TRINITY_DN582_c0_g1_i5_orf1	brown	0.85111
TRINITY_DN2840_c0_g1_i5_orf1	red	0.96336
TRINITY_DN69557_c0_g1_i1_orf1	yellow	0.87447
TRINITY_DN5420_c0_g1_i2_orf1	blue	0.88268
TRINITY_DN4270_c0_g1_i1_orf1	blue	0.67586
TRINITY_DN14952_c0_g3_i1_orf1	black	0.57527
TRINITY_DN22875_c0_g1_i6_orf1	brown	0.92429
TRINITY_DN8766_c0_g1_i1_orf1	black	0.92378
TRINITY_DN16077_c0_g1_i13_orf1	turquoise	0.9278
TRINITY_DN116874_c0_g1_i1_orfp1	blue	0.50821
TRINITY_DN4798_c0_g1_i3_orf1	red	0.68041
TRINITY_DN18035_c0_g1_i7_orf1	turquoise	0.80358
TRINITY_DN53136_c0_g1_i1_orf1	turquoise	0.96896
TRINITY_DN3513_c0_g1_i5_orf1	green	0.89252
TRINITY_DN16886_c0_g1_i4_orf1	turquoise	0.96904
TRINITY_DN10231_c0_g2_i1_orf1	blue	0.7369
TRINITY_DN14298_c0_g1_i3_orf1	blue	0.64943
TRINITY_DN82801_c0_g1_i1_orf1	turquoise	0.81232
TRINITY_DN88876_c0_g1_i1_orf1	blue	0.76682
TRINITY_DN2064_c1_g1_i1_orf1	turquoise	0.92863

TRINITY_DN4116_c0_g1_i3_orf1	turquoise	0.78281
TRINITY_DN8543_c0_g1_i1_orf1	turquoise	0.9772
TRINITY_DN58261_c0_g1_i1_orf1	turquoise	0.84927
TRINITY_DN3733_c0_g1_i1_orf1	turquoise	0.97065
TRINITY_DN9132_c0_g1_i5_orf1	brown	0.89443
TRINITY_DN56993_c0_g1_i4_orf1	red	0.85405
TRINITY_DN6870_c0_g1_i5_orf1	blue	0.7209
TRINITY_DN726_c0_g1_i2_orf1	brown	0.75348
TRINITY_DN3749_c0_g1_i1_orf1	turquoise	0.98746
TRINITY_DN11670_c0_g1_i1_orf1	blue	0.97236
TRINITY_DN12476_c0_g1_i4_orf1	turquoise	0.84572
TRINITY_DN92_c1_g2_i1_orf1	black	0.93594
TRINITY_DN4242_c0_g1_i6_orf1	brown	0.72916
TRINITY_DN7341_c0_g1_i8_orf1	turquoise	0.60054
TRINITY_DN2793_c0_g2_i1_orf1	green	0.84443
TRINITY_DN28802_c0_g1_i1_orf1	black	0.83719
TRINITY_DN6771_c0_g2_i1_orf1	green	0.81935
TRINITY_DN2172_c0_g2_i5_orf1	turquoise	0.87158
TRINITY_DN14365_c0_g1_i2_orf1	turquoise	0.5947
TRINITY_DN25783_c0_g1_i2_orf1	turquoise	0.83672
TRINITY_DN26488_c0_g1_i6_orf1	black	0.74914
TRINITY_DN3029_c1_g2_i1_orf1	brown	0.8092
TRINITY_DN1575_c0_g1_i10_orf1	black	0.66553
TRINITY_DN4464_c0_g2_i1_orf1	black	0.94212
TRINITY_DN28577_c0_g1_i6_orf1	turquoise	0.99477
TRINITY_DN9836_c0_g1_i2_orf1	green	0.5271
TRINITY_DN23343_c0_g1_i9_orf1	turquoise	0.9729
TRINITY_DN2338_c0_g2_i1_orf1	green	0.61566
TRINITY_DN14967_c0_g2_i1_orf1	turquoise	0.9771
TRINITY_DN16234_c0_g2_i3_orf1	green	0.7897
TRINITY_DN500_c0_g1_i1_orf1	turquoise	0.82859
TRINITY_DN3628_c0_g1_i5_orf1	turquoise	0.9322
TRINITY_DN19821_c0_g2_i4_orf1	green	0.81121
TRINITY_DN5982_c0_g1_i3_orf1	turquoise	0.89352
TRINITY_DN2103_c0_g1_i1_orf1	turquoise	0.98878
TRINITY_DN53167_c0_g1_i3_orf1	turquoise	0.98564
TRINITY_DN84_c0_g1_i4_orf1	turquoise	0.86311
TRINITY_DN361_c0_g1_i5_orf1	blue	0.9128
TRINITY_DN27848_c0_g1_i2_orf1	turquoise	0.9823
TRINITY_DN696_c1_g1_i10_orf1	blue	0.97868
TRINITY_DN107288_c0_g1_i2_orf1	turquoise	0.80948
TRINITY_DN79319_c0_g1_i8_orfp1	turquoise	0.97359
TRINITY_DN6205_c0_g1_i4_orfp1	blue	0.65609
TRINITY_DN99_c0_g1_i3_orf1	turquoise	0.69873
TRINITY_DN7854_c0_g1_i4_orf1	blue	0.95164
TRINITY_DN61335_c0_g2_i1_orf1	brown	0.82187
TRINITY_DN1249_c0_g1_i10_orf1	turquoise	0.95428
TRINITY_DN36682_c0_g1_i1_orf1	turquoise	0.89133
TRINITY_DN7556_c0_g1_i3_orf1	green	0.6576
TRINITY_DN43431_c0_g1_i1_orf1	black	0.93698
TRINITY_DN87522_c0_g2_i1_orf1	blue	0.44383
TRINITY_DN5748_c0_g1_i6_orf1	brown	0.88977
TRINITY_DN6415_c0_g1_i1_orf1	yellow	0.72845
TRINITY_DN6642_c0_g1_i2_orf1	brown	0.81031
TRINITY_DN18909_c0_g1_i6_orf1	turquoise	0.69393
TRINITY_DN23042_c0_g1_i1_orf1	blue	0.7661
TRINITY_DN3889_c0_g1_i7_orfp1	turquoise	0.95351
TRINITY_DN4394_c0_g1_i4_orf1	brown	0.89777

TRINITY_DN53400_c0_g1_i1_orf1	turquoise	0.81265
TRINITY_DN2224_c0_g2_i1_orf1	turquoise	0.7458
TRINITY_DN1154_c0_g1_i1_orf1	turquoise	0.93587
TRINITY_DN4707_c0_g1_i1_orf1	turquoise	0.93653
TRINITY_DN19328_c0_g1_i1_orf1	blue	0.80927
TRINITY_DN2830_c0_g1_i9_orf1	turquoise	0.65722
TRINITY_DN32681_c0_g1_i3_orf1	turquoise	0.93937
TRINITY_DN85161_c0_g1_i2_orf1	yellow	0.62666
TRINITY_DN130575_c0_g1_i1_orfp1	brown	0.97478
TRINITY_DN11159_c0_g2_i1_orf1	turquoise	0.83348
TRINITY_DN139537_c0_g1_i1_orf1	blue	0.86875
TRINITY_DN27994_c0_g1_i1_orf1	turquoise	0.91716
TRINITY_DN69170_c0_g2_i1_orf1	turquoise	0.88448
TRINITY_DN7839_c0_g1_i4_orf1	turquoise	0.93162
TRINITY_DN47260_c0_g1_i2_orf1	green	0.83788
TRINITY_DN2663_c0_g1_i12_orf1	green	0.70682
TRINITY_DN7329_c0_g1_i6_orf1	turquoise	0.8297
TRINITY_DN35809_c0_g1_i1_orf1	brown	0.95488
TRINITY_DN140_c0_g1_i5_orf1	yellow	0.74155
TRINITY_DN50517_c0_g1_i5_orf1	red	0.88017
TRINITY_DN5457_c0_g1_i4_orf1	turquoise	0.81838
TRINITY_DN19092_c2_g1_i1_orf1	brown	0.79567
TRINITY_DN2749_c4_g1_i2_orf1	pink	0.70216
TRINITY_DN27725_c0_g1_i2_orf1	red	0.77358
TRINITY_DN141396_c0_g1_i1_orf1	turquoise	0.68139
TRINITY_DN19260_c0_g1_i5_orf1	red	0.76888
TRINITY_DN2743_c0_g1_i5_orf1	brown	0.66512
TRINITY_DN1366_c0_g1_i5_orf1	turquoise	0.98896
TRINITY_DN6143_c0_g2_i1_orf1	turquoise	0.90671
TRINITY_DN146841_c0_g1_i1_orf1	yellow	0.83968
TRINITY_DN554_c0_g1_i1_orf1	brown	0.8515
TRINITY_DN1427_c0_g1_i7_orf1	turquoise	0.90622
TRINITY_DN27852_c0_g1_i1_orf1	turquoise	0.89971
TRINITY_DN51938_c0_g3_i1_orf1	grey	-0.16234
TRINITY_DN4280_c0_g1_i8_orf1	turquoise	0.59228
TRINITY_DN376_c0_g1_i1_orf1	turquoise	0.7567
TRINITY_DN2490_c0_g2_i1_orfp1	turquoise	0.99162
TRINITY_DN10933_c0_g2_i1_orf1	green	0.86127
TRINITY_DN22257_c0_g1_i7_orf1	green	0.845
TRINITY_DN21619_c0_g1_i1_orf1	turquoise	0.94892
TRINITY_DN54269_c0_g1_i3_orf1	blue	0.90818
TRINITY_DN35757_c0_g1_i1_orf1	green	0.56514
TRINITY_DN7325_c0_g1_i1_orf1	turquoise	0.70373
TRINITY_DN19951_c0_g1_i5_orf1	blue	0.93978
TRINITY_DN143603_c0_g1_i1_orf1	turquoise	0.96813
TRINITY_DN20710_c0_g2_i2_orf1	brown	0.54417
TRINITY_DN1123_c2_g1_i4_orf1	yellow	0.90278
TRINITY_DN130051_c0_g1_i1_orf1	turquoise	0.8756
TRINITY_DN4571_c0_g1_i4_orf1	red	0.84693
TRINITY_DN7212_c0_g1_i4_orf1	green	0.88341
TRINITY_DN143532_c0_g1_i1_orf1	brown	0.5761
TRINITY_DN5176_c0_g1_i2_orf1	yellow	0.73202
TRINITY_DN8390_c0_g1_i2_orf1	blue	0.96308
TRINITY_DN436_c0_g2_i5_orfp1	pink	0.8381
TRINITY_DN34479_c0_g1_i2_orf1	turquoise	0.63526
TRINITY_DN14944_c0_g1_i9_orf1	blue	0.84937
TRINITY_DN102712_c0_g1_i1_orf1	grey	0.74647
TRINITY_DN12594_c0_g1_i1_orf1	green	0.39977

TRINITY_DN2566_c0_g1_i5_orf1	blue	0.84009
TRINITY_DN2984_c0_g1_i3_orf1	red	0.92097
TRINITY_DN21533_c0_g1_i4_orf1	black	0.9329
TRINITY_DN97378_c0_g1_i2_orf1	green	0.94758
TRINITY_DN12683_c0_g1_i3_orf1	turquoise	0.96738
TRINITY_DN8394_c1_g1_i9_orf1	turquoise	0.57742
TRINITY_DN1982_c0_g1_i24_orf1	yellow	0.90577
TRINITY_DN7794_c0_g1_i1_orf1	pink	0.86363
TRINITY_DN36045_c0_g1_i2_orf1	turquoise	0.89441
TRINITY_DN4021_c0_g1_i1_orf1	blue	0.95486
TRINITY_DN99020_c0_g1_i1_orf1	blue	0.87911
TRINITY_DN2456_c0_g1_i2_orf1	turquoise	0.95601
TRINITY_DN38693_c0_g1_i4_orf1	turquoise	0.90806
TRINITY_DN3062_c0_g1_i1_orf1	turquoise	0.99228
TRINITY_DN56795_c1_g1_i1_orf1	turquoise	0.34885
TRINITY_DN50151_c0_g1_i1_orf1	turquoise	0.99266
TRINITY_DN9_c0_g1_i11_orf1	blue	0.91741
TRINITY_DN50225_c0_g1_i1_orf1	red	0.8628
TRINITY_DN14501_c0_g1_i1_orf1	turquoise	0.87961
TRINITY_DN17133_c0_g1_i1_orf1	blue	0.80477
TRINITY_DN75188_c0_g1_i1_orf1	turquoise	0.99393
TRINITY_DN1601_c0_g1_i4_orf1	red	0.90733
TRINITY_DN2338_c0_g2_i2_orf1	turquoise	0.6246
TRINITY_DN59028_c0_g1_i1_orf1	green	0.92082
TRINITY_DN4142_c0_g1_i5_orf1	brown	0.31027
TRINITY_DN129259_c0_g2_i1_orf1	turquoise	0.62136
TRINITY_DN29026_c0_g1_i4_orf1	brown	0.89188
TRINITY_DN85476_c0_g1_i1_orf1	yellow	0.8534
TRINITY_DN4561_c0_g1_i3_orf1	red	0.89892
TRINITY_DN2516_c0_g2_i10_orf1	brown	0.88912
TRINITY_DN108354_c0_g1_i1_orf1	turquoise	0.9506
TRINITY_DN4321_c0_g1_i1_orf1	pink	0.81134
TRINITY_DN23194_c0_g1_i4_orf1	blue	0.7512
TRINITY_DN12013_c0_g1_i6_orf1	yellow	0.69948
TRINITY_DN71698_c0_g1_i1_orfp1	brown	0.94595
TRINITY_DN1233_c0_g2_i1_orf1	red	0.94916
TRINITY_DN18869_c0_g1_i1_orf1	turquoise	0.9097
TRINITY_DN111488_c0_g1_i1_orf1	yellow	0.92465
TRINITY_DN3263_c0_g1_i2_orf1	brown	0.85559
TRINITY_DN41602_c0_g3_i1_orf1	turquoise	0.80147
TRINITY_DN10548_c0_g2_i1_orf1	turquoise	0.85382
TRINITY_DN8432_c0_g2_i1_orf1	turquoise	0.60472
TRINITY_DN13997_c0_g1_i5_orf1	green	0.94446
TRINITY_DN1030_c0_g1_i6_orf1	green	0.90955
TRINITY_DN8812_c0_g1_i1_orf1	black	0.78962
TRINITY_DN6855_c1_g1_i3_orf1	turquoise	0.7363
TRINITY_DN6994_c0_g1_i3_orf1	yellow	0.72547
TRINITY_DN1066_c0_g1_i8_orf1	red	0.74264
TRINITY_DN4861_c0_g1_i7_orf1	turquoise	0.95161
TRINITY_DN2352_c0_g1_i15_orf1	blue	0.86644
TRINITY_DN43369_c0_g2_i1_orf1	turquoise	0.73706
TRINITY_DN1889_c0_g1_i1_orf1	yellow	0.93969
TRINITY_DN140423_c0_g1_i2_orf1	yellow	0.61783
TRINITY_DN40650_c0_g1_i1_orf1	turquoise	0.92401
TRINITY_DN121650_c0_g1_i1_orf1	blue	0.89963
TRINITY_DN348_c0_g2_i3_orf1	turquoise	0.99102
TRINITY_DN1103_c0_g1_i12_orf1	green	0.84311
TRINITY_DN4550_c1_g1_i5_orfp2	turquoise	0.97408

TRINITY_DN51968_c0_g1_i1_orf1	turquoise	0.8318
TRINITY_DN6881_c0_g1_i1_orf1	yellow	0.83368
TRINITY_DN2579_c0_g1_i7_orf1	turquoise	0.98946
TRINITY_DN3056_c0_g1_i1_orf1	blue	0.9593
TRINITY_DN13973_c0_g1_i6_orf1	blue	0.83368
TRINITY_DN69334_c0_g1_i1_orf1	turquoise	0.47433
TRINITY_DN47591_c1_g1_i1_orf1	turquoise	0.87372
TRINITY_DN753_c0_g1_i4_orf1	turquoise	0.99241
TRINITY_DN27592_c0_g1_i1_orf1	turquoise	0.7406
TRINITY_DN5560_c0_g1_i5_orf1	green	0.62282
TRINITY_DN10701_c0_g2_i2_orf1	turquoise	0.89841
TRINITY_DN10521_c0_g1_i7_orf1	turquoise	0.9644
TRINITY_DN21420_c0_g1_i2_orf1	brown	0.89094
TRINITY_DN89483_c0_g1_i1_orf1	brown	0.85047
TRINITY_DN6985_c0_g1_i5_orf1	turquoise	0.75279
TRINITY_DN4895_c0_g1_i2_orf1	turquoise	0.97516
TRINITY_DN10680_c0_g1_i5_orf1	blue	0.85147
TRINITY_DN18569_c0_g2_i1_orf1	turquoise	0.9784
TRINITY_DN1791_c0_g1_i3_orf1	turquoise	0.88565
TRINITY_DN12576_c0_g1_i2_orf1	red	0.88298
TRINITY_DN76283_c0_g2_i1_orf1	turquoise	0.94695
TRINITY_DN3028_c0_g1_i1_orf1	turquoise	0.96444
TRINITY_DN20244_c0_g1_i1_orfp1	red	0.89387
TRINITY_DN452_c9_g1_i1_orf1	brown	0.89358
TRINITY_DN19866_c0_g1_i4_orf1	brown	0.28858
TRINITY_DN3832_c0_g1_i1_orf1	turquoise	0.87153
TRINITY_DN7776_c0_g1_i1_orf1	green	0.86883
TRINITY_DN10886_c0_g2_i4_orf1	yellow	0.52107
TRINITY_DN10095_c0_g1_i5_orf1	blue	0.80635
TRINITY_DN1352_c0_g1_i5_orf1	brown	0.93801
TRINITY_DN19377_c0_g1_i4_orf1	brown	0.84712
TRINITY_DN34676_c1_g1_i3_orf1	brown	0.56352
TRINITY_DN25360_c0_g1_i2_orf1	blue	0.66519
TRINITY_DN49204_c0_g1_i1_orf1	turquoise	0.54444
TRINITY_DN32896_c0_g3_i1_orf1	turquoise	0.73342
TRINITY_DN53358_c0_g1_i3_orf1	green	0.81487
TRINITY_DN756_c0_g1_i11_orf1	turquoise	0.84943
TRINITY_DN21123_c0_g1_i1_orf1	turquoise	0.9404
TRINITY_DN15900_c0_g1_i6_orf1	turquoise	0.97585
TRINITY_DN47219_c0_g1_i3_orf1	red	0.84804
TRINITY_DN11736_c0_g1_i1_orf1	brown	0.80937
TRINITY_DN9354_c0_g1_i7_orf1	red	0.86163
TRINITY_DN45859_c0_g1_i1_orf1	turquoise	0.74657
TRINITY_DN37729_c0_g1_i8_orf1	turquoise	0.74053
TRINITY_DN1767_c0_g2_i15_orf1	blue	0.88091
TRINITY_DN42705_c0_g1_i3_orf1	turquoise	0.78215
TRINITY_DN874_c2_g1_i1_orf1	brown	0.93416
TRINITY_DN13972_c0_g1_i5_orf1	turquoise	0.8882
TRINITY_DN1540_c0_g1_i14_orf1	brown	0.9013
TRINITY_DN42120_c0_g1_i2_orf1	yellow	0.76169
TRINITY_DN18773_c0_g1_i3_orf1	turquoise	0.94446
TRINITY_DN7534_c0_g1_i15_orf1	black	0.98254
TRINITY_DN3637_c0_g1_i2_orf1	blue	0.79166
TRINITY_DN8738_c0_g1_i1_orf1	red	0.88963
TRINITY_DN1732_c0_g1_i15_orf1	green	0.86657
TRINITY_DN7787_c0_g1_i1_orf1	turquoise	0.95838
TRINITY_DN58751_c0_g1_i2_orf1	brown	0.90029
TRINITY_DN7473_c0_g1_i1_orf1	red	0.67963

TRINITY_DN1814_c0_g2_i1_orf1	yellow	0.97548
TRINITY_DN20356_c0_g1_i5_orf1	yellow	0.42997
TRINITY_DN48548_c0_g1_i1_orf1	green	0.92749
TRINITY_DN6567_c0_g1_i5_orf1	turquoise	0.68134
TRINITY_DN11808_c0_g1_i8_orf1	brown	0.31358
TRINITY_DN5891_c0_g2_i4_orf1	turquoise	0.98201
TRINITY_DN7964_c0_g1_i6_orfp1	turquoise	0.95815
TRINITY_DN7488_c0_g1_i1_orf1	turquoise	0.479
TRINITY_DN4621_c0_g1_i4_orf1	brown	0.95375
TRINITY_DN5162_c0_g1_i3_orf1	red	0.81426
TRINITY_DN126648_c0_g1_i1_orf1	turquoise	0.79536
TRINITY_DN4304_c0_g1_i3_orf1	brown	0.89946
TRINITY_DN114890_c0_g1_i4_orf1	brown	0.86827
TRINITY_DN113353_c0_g1_i1_orf1	green	0.92254
TRINITY_DN66671_c0_g1_i1_orf1	green	0.90083
TRINITY_DN19251_c0_g1_i8_orf1	yellow	0.93326
TRINITY_DN3439_c0_g2_i2_orf1	brown	0.96352
TRINITY_DN15000_c0_g1_i4_orf1	pink	0.8733
TRINITY_DN8980_c0_g1_i2_orf1	turquoise	0.90253
TRINITY_DN3254_c0_g1_i1_orf1	turquoise	0.73512
TRINITY_DN1749_c0_g1_i1_orf1	blue	0.96496
TRINITY_DN65988_c0_g1_i4_orf1	turquoise	0.94129
TRINITY_DN518_c0_g1_i1_orf1	red	0.9213
TRINITY_DN12293_c0_g1_i1_orf1	turquoise	0.62689
TRINITY_DN1827_c0_g1_i4_orf1	brown	0.90808
TRINITY_DN6136_c0_g1_i1_orf1	green	0.8119
TRINITY_DN2342_c0_g1_i1_orf1	red	0.51605
TRINITY_DN565_c0_g2_i1_orf1	pink	0.91179
TRINITY_DN14905_c0_g2_i2_orf1	blue	0.76663
TRINITY_DN3126_c0_g1_i4_orf1	yellow	0.39704
TRINITY_DN2688_c0_g1_i3_orf1	brown	0.78763
TRINITY_DN6424_c0_g1_i2_orf1	green	0.6529
TRINITY_DN12873_c0_g2_i1_orf1	brown	0.87089
TRINITY_DN7291_c0_g1_i5_orf1	grey	-0.52857
TRINITY_DN4744_c0_g1_i7_orf1	yellow	0.92296
TRINITY_DN18804_c0_g1_i5_orf1	blue	0.95619
TRINITY_DN64196_c0_g1_i2_orf1	turquoise	0.60847
TRINITY_DN811_c0_g1_i15_orf1	black	0.82938
TRINITY_DN4010_c0_g2_i1_orf1	yellow	0.98122
TRINITY_DN17772_c0_g2_i3_orf1	blue	0.59511
TRINITY_DN55148_c0_g1_i1_orf1	brown	0.80447
TRINITY_DN1383_c0_g1_i2_orf1	turquoise	0.77382
TRINITY_DN16349_c0_g1_i10_orf1	turquoise	0.84833
TRINITY_DN1134_c0_g1_i4_orf1	yellow	0.92023
TRINITY_DN1232_c0_g1_i1_orf1	yellow	0.79864
TRINITY_DN111_c0_g2_i2_orf1	turquoise	0.33866
TRINITY_DN33365_c0_g1_i1_orf1	blue	0.97586
TRINITY_DN34423_c0_g1_i2_orf1	brown	0.90243
TRINITY_DN10769_c0_g1_i1_orf1	blue	0.77521
TRINITY_DN15812_c0_g1_i2_orf1	blue	0.84643
TRINITY_DN8511_c0_g1_i1_orf1	turquoise	0.97806
TRINITY_DN1103_c0_g1_i18_orf1	blue	0.71933
TRINITY_DN5126_c0_g1_i3_orf1	brown	0.749
TRINITY_DN20793_c0_g2_i1_orf1	blue	0.91847
TRINITY_DN140_c0_g1_i1_orf1	brown	0.90981
TRINITY_DN741_c0_g1_i1_orf1	brown	0.59838
TRINITY_DN12497_c0_g1_i1_orf1	turquoise	0.99475
TRINITY_DN1639_c0_g2_i2_orf1	turquoise	0.82541

TRINITY_DN1785_c0_g1_i5_orf1	green	0.24167
TRINITY_DN6391_c0_g1_i1_orf1	brown	0.79778
TRINITY_DN71917_c0_g3_i1_orf1	brown	0.78355
TRINITY_DN2002_c0_g1_i5_orfp1	blue	0.96115
TRINITY_DN3991_c0_g1_i6_orf1	brown	0.91219
TRINITY_DN2794_c1_g1_i8_orf1	blue	0.92123
TRINITY_DN364_c1_g1_i2_orf1	black	0.96732
TRINITY_DN19411_c0_g1_i1_orf1	turquoise	0.42281
TRINITY_DN84478_c0_g1_i8_orf1	green	0.93314
TRINITY_DN2836_c0_g1_i4_orf1	black	0.8468
TRINITY_DN2114_c0_g1_i5_orf1	turquoise	0.9809
TRINITY_DN235_c0_g3_i1_orf1	red	0.88502
TRINITY_DN6991_c0_g1_i24_orf1	yellow	0.85404
TRINITY_DN2596_c0_g1_i6_orf1	brown	0.73318
TRINITY_DN2975_c0_g1_i4_orf1	turquoise	0.95896
TRINITY_DN51829_c0_g1_i1_orf1	red	0.64819
TRINITY_DN9562_c0_g1_i3_orf1	green	0.79802
TRINITY_DN8536_c0_g1_i2_orf1	turquoise	0.84291
TRINITY_DN5383_c0_g1_i4_orf1	black	0.97263
TRINITY_DN34786_c0_g1_i1_orf1	blue	0.9231
TRINITY_DN5630_c4_g1_i2_orf1	turquoise	0.91333
TRINITY_DN25896_c0_g1_i6_orf1	turquoise	0.93671
TRINITY_DN15202_c0_g1_i6_orf1	brown	0.96992
TRINITY_DN11402_c0_g1_i1_orf1	turquoise	0.79801
TRINITY_DN3971_c0_g1_i1_orf1	turquoise	0.82478
TRINITY_DN12503_c0_g2_i1_orf1	turquoise	0.75431
TRINITY_DN2627_c0_g2_i1_orf1	blue	0.70825
TRINITY_DN8596_c0_g1_i2_orf1	red	0.82266
TRINITY_DN843_c0_g1_i2_orf1	blue	0.83521
TRINITY_DN5256_c0_g1_i1_orf1	blue	0.54743
TRINITY_DN356_c2_g1_i3_orf1	brown	0.86761
TRINITY_DN6612_c0_g1_i4_orf1	yellow	0.96651
TRINITY_DN18592_c0_g2_i1_orf1	green	0.78418
TRINITY_DN10558_c0_g1_i4_orf1	red	0.79098
TRINITY_DN4766_c0_g1_i4_orf1	red	0.80987
TRINITY_DN582_c0_g1_i2_orf1	black	0.6402
TRINITY_DN4255_c0_g1_i10_orf1	brown	0.91961
TRINITY_DN13887_c0_g1_i5_orf1	blue	0.98835
TRINITY_DN3755_c0_g1_i3_orf1	turquoise	0.64317
TRINITY_DN783_c0_g1_i7_orf1	red	0.96647
TRINITY_DN17271_c0_g1_i1_orf1	red	0.949
TRINITY_DN2182_c0_g1_i4_orf1	brown	0.88716
TRINITY_DN4514_c0_g1_i1_orf1	turquoise	0.79143
TRINITY_DN20710_c0_g1_i2_orf1	blue	0.92927
TRINITY_DN18009_c0_g1_i1_orf1	yellow	0.88949
TRINITY_DN23354_c0_g1_i7_orf1	blue	0.53752
TRINITY_DN4026_c0_g1_i4_orf1	turquoise	0.81225
TRINITY_DN21278_c0_g2_i2_orf1	brown	0.94602
TRINITY_DN19537_c0_g1_i1_orf1	blue	0.8638
TRINITY_DN4998_c0_g1_i21_orf1	brown	0.92588
TRINITY_DN23790_c0_g1_i1_orf1	turquoise	0.70131
TRINITY_DN1664_c0_g1_i4_orf1	black	0.96102
TRINITY_DN8406_c0_g1_i3_orfp1	turquoise	0.5285
TRINITY_DN24399_c0_g1_i1_orf1	turquoise	0.95888
TRINITY_DN920_c0_g1_i6_orf1	pink	0.92505
TRINITY_DN948_c0_g1_i1_orf1	turquoise	0.9965
TRINITY_DN3805_c0_g1_i2_orf1	turquoise	0.64158
TRINITY_DN42461_c0_g1_i4_orf1	yellow	0.89033

TRINITY_DN7073_c0_g1_i1_orf1	turquoise	0.98113
TRINITY_DN47575_c0_g1_i1_orf1	turquoise	0.83508
TRINITY_DN19092_c0_g1_i2_orf1	turquoise	0.97443
TRINITY_DN47666_c0_g1_i4_orf1	turquoise	0.73227
TRINITY_DN2024_c0_g1_i12_orfp1	blue	0.8256
TRINITY_DN1328_c0_g1_i6_orf1	brown	0.88124
TRINITY_DN19920_c1_g1_i2_orf1	turquoise	0.89088
TRINITY_DN22678_c0_g1_i4_orf1	turquoise	0.95923
TRINITY_DN810_c0_g1_i4_orf1	green	0.72224
TRINITY_DN74020_c0_g1_i2_orf1	green	0.69079
TRINITY_DN2894_c0_g2_i3_orf1	red	0.88981
TRINITY_DN18863_c0_g1_i3_orf1	turquoise	0.87526
TRINITY_DN10079_c0_g1_i1_orf1	brown	0.66761
TRINITY_DN8838_c0_g1_i1_orf1	turquoise	0.88206
TRINITY_DN14073_c0_g1_i1_orf1	turquoise	0.91364
TRINITY_DN4794_c1_g1_i9_orf1	green	0.69273
TRINITY_DN15327_c2_g1_i2_orf1	yellow	0.77686
TRINITY_DN50085_c0_g1_i1_orf1	turquoise	0.96939
TRINITY_DN124950_c0_g2_i1_orf1	turquoise	0.81873
TRINITY_DN3609_c0_g1_i6_orf1	black	0.91091
TRINITY_DN9506_c0_g1_i2_orf1	turquoise	0.51343
TRINITY_DN9243_c0_g1_i4_orf1	red	0.71984
TRINITY_DN4938_c0_g1_i13_orf1	turquoise	0.92761
TRINITY_DN17825_c1_g1_i1_orf1	turquoise	0.99243
TRINITY_DN13236_c0_g1_i4_orf1	yellow	0.86413
TRINITY_DN71308_c0_g1_i4_orf1	black	0.83217
TRINITY_DN27033_c1_g1_i3_orfp1	green	0.52876
TRINITY_DN14458_c0_g1_i2_orf1	blue	0.96284
TRINITY_DN83295_c0_g1_i3_orf1	turquoise	0.91868
TRINITY_DN512_c1_g1_i4_orf1	green	0.83015
TRINITY_DN103475_c0_g1_i4_orf1	brown	0.96538
TRINITY_DN1781_c0_g1_i8_orf1	red	0.82845
TRINITY_DN14904_c0_g1_i1_orf1	brown	0.721
TRINITY_DN74037_c0_g5_i1_orf1	brown	0.75493
TRINITY_DN605_c0_g1_i4_orf1	turquoise	0.92952
TRINITY_DN4842_c0_g1_i5_orf1	turquoise	0.97218
TRINITY_DN7776_c0_g1_i5_orf1	black	0.8942
TRINITY_DN133474_c0_g2_i2_orf1	brown	0.47222
TRINITY_DN6084_c0_g1_i4_orf1	turquoise	0.74771
TRINITY_DN4424_c0_g1_i1_orf1	yellow	0.72555
TRINITY_DN649_c1_g1_i13_orf1	turquoise	0.90509
TRINITY_DN120593_c0_g1_i1_orf1	red	0.97865
TRINITY_DN2320_c0_g1_i4_orf1	turquoise	0.677
TRINITY_DN8771_c0_g1_i5_orf1	black	0.96181
TRINITY_DN113272_c0_g1_i1_orf1	blue	0.88785
TRINITY_DN5177_c0_g1_i2_orf1	green	0.858
TRINITY_DN26375_c0_g1_i1_orf1	turquoise	0.97767
TRINITY_DN886_c0_g1_i1_orf1	turquoise	0.954
TRINITY_DN22441_c0_g1_i1_orf1	green	0.75643
TRINITY_DN1068_c0_g1_i3_orf1	yellow	0.77045
TRINITY_DN812_c2_g1_i1_orf1	brown	0.98253
TRINITY_DN1480_c0_g1_i5_orf1	blue	0.85343
TRINITY_DN703_c13_g1_i1_orf1	green	0.96383
TRINITY_DN9400_c0_g1_i1_orf1	pink	0.87996
TRINITY_DN81791_c0_g2_i2_orf1	red	0.59834
TRINITY_DN1504_c0_g1_i1_orf1	red	0.95711
TRINITY_DN1955_c0_g1_i5_orf1	grey	0.53031
TRINITY_DN4497_c0_g1_i4_orf1	turquoise	0.91585

TRINITY_DN7868_c0_g1_i8_orf1	turquoise	0.89664
TRINITY_DN77318_c0_g2_i1_orf1	brown	0.63382
TRINITY_DN73900_c0_g1_i1_orf1	brown	0.9277
TRINITY_DN33867_c0_g1_i8_orf1	green	0.61457
TRINITY_DN2758_c0_g1_i7_orf1	black	0.72568
TRINITY_DN6122_c0_g1_i6_orf1	green	0.97256
TRINITY_DN79804_c0_g1_i1_orf1	turquoise	0.94858
TRINITY_DN2770_c0_g2_i4_orf1	yellow	0.49176
TRINITY_DN86580_c0_g1_i1_orf1	turquoise	0.65477
TRINITY_DN3476_c0_g1_i5_orf1	turquoise	0.97315
TRINITY_DN1554_c0_g1_i9_orf1	red	0.87015
TRINITY_DN9234_c0_g1_i5_orf1	turquoise	0.76112
TRINITY_DN45227_c0_g1_i3_orf1	turquoise	0.98197
TRINITY_DN12960_c0_g1_i1_orf1	brown	0.73155
TRINITY_DN5581_c0_g1_i1_orf1	blue	0.97907
TRINITY_DN36788_c0_g1_i2_orf1	turquoise	0.9303
TRINITY_DN8783_c0_g1_i5_orf1	brown	0.57152
TRINITY_DN34115_c0_g1_i1_orf1	green	0.78446
TRINITY_DN8367_c0_g1_i1_orf1	turquoise	0.96441
TRINITY_DN26408_c0_g1_i7_orf1	turquoise	0.98693
TRINITY_DN4345_c0_g1_i9_orf1	turquoise	0.85537
TRINITY_DN28806_c0_g1_i1_orf1	turquoise	0.94289
TRINITY_DN8440_c0_g1_i9_orf1	brown	0.90941
TRINITY_DN3959_c1_g2_i1_orf1	turquoise	0.98935
TRINITY_DN2338_c3_g2_i3_orf1	pink	0.86879
TRINITY_DN77830_c0_g2_i2_orf1	yellow	0.74157
TRINITY_DN1368_c0_g1_i6_orf1	turquoise	0.90068
TRINITY_DN7749_c1_g1_i2_orf1	blue	0.63504
TRINITY_DN14107_c0_g1_i4_orf1	turquoise	0.93845
TRINITY_DN2277_c0_g1_i11_orf1	blue	0.80635
TRINITY_DN7246_c0_g1_i7_orf1	blue	0.91367
TRINITY_DN4449_c0_g2_i1_orf1	green	0.92798
TRINITY_DN27300_c0_g1_i6_orfp1	yellow	0.70986
TRINITY_DN5562_c0_g1_i3_orf1	turquoise	0.84989
TRINITY_DN5008_c0_g1_i1_orf1	turquoise	0.60466
TRINITY_DN50074_c0_g1_i1_orf1	yellow	0.6904
TRINITY_DN77425_c0_g1_i2_orf1	blue	0.98643
TRINITY_DN40126_c0_g1_i1_orf1	brown	0.93941
TRINITY_DN42275_c0_g1_i1_orfp1	green	0.8542
TRINITY_DN63492_c0_g1_i1_orf1	grey	-0.63621
TRINITY_DN41848_c0_g1_i4_orf1	brown	0.85749
TRINITY_DN37699_c0_g1_i4_orfp1	turquoise	0.89838
TRINITY_DN8310_c0_g2_i1_orf1	yellow	0.77845
TRINITY_DN12014_c0_g1_i2_orf1	black	0.89
TRINITY_DN6103_c0_g1_i6_orf1	turquoise	0.87132
TRINITY_DN3869_c0_g1_i4_orf1	turquoise	0.45292
TRINITY_DN35301_c0_g1_i3_orf1	turquoise	0.96842
TRINITY_DN36592_c0_g1_i1_orf1	turquoise	0.88139
TRINITY_DN348_c0_g2_i1_orf1	turquoise	0.7375
TRINITY_DN1507_c0_g1_i5_orf1	black	0.94998
TRINITY_DN3759_c0_g1_i1_orf1	turquoise	0.82522
TRINITY_DN107_c0_g1_i1_orf1	turquoise	0.83743
TRINITY_DN2907_c0_g2_i4_orf1	turquoise	0.67337
TRINITY_DN5563_c1_g2_i2_orf1	yellow	0.77665
TRINITY_DN6470_c0_g3_i2_orf1	blue	0.96347
TRINITY_DN33619_c0_g1_i1_orf1	turquoise	0.97572
TRINITY_DN5661_c0_g1_i5_orf1	yellow	0.91409
TRINITY_DN1957_c0_g1_i4_orf1	green	0.97515

TRINITY_DN29100_c0_g1_i2_orf1	yellow	0.87196
TRINITY_DN5405_c1_g1_i13_orf1	yellow	0.85663
TRINITY_DN1293_c0_g1_i4_orf1	blue	0.88394
TRINITY_DN29034_c0_g1_i1_orf1	turquoise	0.81591
TRINITY_DN21792_c0_g1_i1_orf1	turquoise	0.96772
TRINITY_DN2200_c0_g1_i4_orf1	yellow	0.74571
TRINITY_DN13233_c0_g1_i3_orf1	turquoise	0.88097
TRINITY_DN96080_c0_g2_i1_orf1	turquoise	0.96778
TRINITY_DN443_c0_g1_i2_orf1	black	0.82839
TRINITY_DN5111_c0_g1_i2_orf1	turquoise	0.85198
TRINITY_DN7064_c0_g1_i19_orf1	brown	0.9277
TRINITY_DN9979_c0_g1_i1_orf1	yellow	0.56942
TRINITY_DN2318_c1_g1_i1_orf1	yellow	0.71809
TRINITY_DN143792_c0_g1_i1_orf1	green	0.72945
TRINITY_DN4062_c0_g2_i1_orf1	brown	0.88393
TRINITY_DN7226_c0_g1_i2_orf1	brown	0.92278
TRINITY_DN3374_c0_g1_i7_orf1	yellow	0.81382
TRINITY_DN2265_c0_g2_i1_orf1	turquoise	0.9539
TRINITY_DN32997_c0_g1_i8_orf1	turquoise	0.9753
TRINITY_DN364_c0_g2_i1_orf1	black	0.9307
TRINITY_DN23564_c0_g1_i7_orf1	brown	0.90373
TRINITY_DN2813_c0_g1_i10_orf1	black	0.95983
TRINITY_DN5472_c0_g1_i1_orf1	turquoise	0.66434
TRINITY_DN36893_c0_g1_i1_orf1	turquoise	0.83456
TRINITY_DN6189_c0_g1_i1_orf1	turquoise	0.77914
TRINITY_DN24539_c0_g1_i4_orf1	red	0.89567
TRINITY_DN7128_c0_g1_i7_orf1	blue	0.8626
TRINITY_DN33995_c0_g1_i5_orf1	yellow	0.78767
TRINITY_DN441_c0_g2_i1_orf1	turquoise	0.81522
TRINITY_DN15157_c0_g1_i1_orf1	brown	0.85333
TRINITY_DN225_c0_g1_i6_orf1	green	0.5828
TRINITY_DN8949_c0_g1_i2_orf1	turquoise	0.94938
TRINITY_DN4207_c0_g1_i1_orf1	turquoise	0.72701
TRINITY_DN2803_c2_g1_i8_orf1	turquoise	0.94037
TRINITY_DN2574_c0_g1_i5_orf1	turquoise	0.97349
TRINITY_DN8473_c0_g1_i6_orf1	brown	0.85227
TRINITY_DN5161_c0_g1_i5_orf1	turquoise	0.63465
TRINITY_DN9661_c0_g1_i1_orf1	grey	-0.30408
TRINITY_DN295_c3_g1_i1_orfp1	brown	0.96119
TRINITY_DN48694_c0_g1_i1_orfp1	blue	0.46675
TRINITY_DN1407_c0_g1_i12_orf1	blue	0.84206
TRINITY_DN9517_c0_g1_i7_orf1	red	0.70349
TRINITY_DN4056_c0_g1_i8_orf1	turquoise	0.82095
TRINITY_DN1672_c0_g1_i6_orf1	black	0.87814
TRINITY_DN70_c6_g1_i1_orf1	green	0.91921
TRINITY_DN3588_c0_g1_i1_orf1	turquoise	0.90025
TRINITY_DN29351_c0_g1_i1_orfp1	green	0.80491
TRINITY_DN3916_c0_g1_i6_orf1	yellow	0.61589
TRINITY_DN1333_c0_g1_i6_orf1	yellow	0.82685
TRINITY_DN25542_c0_g1_i1_orf1	turquoise	0.97461
TRINITY_DN2072_c0_g1_i1_orf1	yellow	0.70474
TRINITY_DN1125_c0_g1_i4_orf1	yellow	0.74118
TRINITY_DN23004_c0_g1_i1_orf1	turquoise	0.91845
TRINITY_DN552_c0_g1_i3_orf1	red	0.83337
TRINITY_DN323_c0_g2_i5_orf1	grey	0.10008
TRINITY_DN18338_c0_g1_i7_orf1	turquoise	0.721
TRINITY_DN1866_c0_g1_i4_orf1	turquoise	0.84248
TRINITY_DN96_c0_g1_i1_orf1	turquoise	0.9791

TRINITY_DN60946_c0_g2_i3_orf1	black	0.94168
TRINITY_DN620_c0_g1_i4_orf1	turquoise	0.98501
TRINITY_DN261_c0_g1_i5_orfp1	blue	0.88631
TRINITY_DN1124_c0_g1_i7_orf1	blue	0.94921
TRINITY_DN48970_c0_g1_i1_orf1	red	0.89252
TRINITY_DN6669_c0_g1_i3_orf1	black	0.78515
TRINITY_DN21125_c0_g1_i1_orf1	red	0.68097
TRINITY_DN11612_c0_g2_i1_orf1	yellow	0.32719
TRINITY_DN72285_c1_g1_i1_orf1	turquoise	0.53546
TRINITY_DN1277_c4_g1_i5_orf1	turquoise	0.99088
TRINITY_DN101325_c0_g1_i4_orf1	turquoise	0.88311
TRINITY_DN10694_c1_g2_i1_orf1	red	0.82483
TRINITY_DN2338_c0_g1_i5_orf1	brown	0.90596
TRINITY_DN206_c0_g1_i11_orf1	blue	0.97869
TRINITY_DN68401_c1_g1_i1_orf1	yellow	0.79318
TRINITY_DN3053_c0_g1_i2_orf1	turquoise	0.98643
TRINITY_DN8717_c0_g1_i5_orf1	turquoise	0.85709
TRINITY_DN57150_c0_g2_i1_orf1	turquoise	0.75671
TRINITY_DN30177_c0_g2_i1_orf1	brown	0.63788
TRINITY_DN27641_c0_g1_i1_orf1	turquoise	0.98792
TRINITY_DN4622_c0_g1_i1_orf1	turquoise	0.84349
TRINITY_DN11820_c0_g1_i1_orf1	red	0.85861
TRINITY_DN24121_c1_g1_i6_orf1	yellow	0.8468
TRINITY_DN22797_c0_g1_i5_orf1	yellow	0.87114
TRINITY_DN12331_c0_g1_i5_orf1	blue	0.95511
TRINITY_DN664_c0_g1_i18_orf1	green	0.93081
TRINITY_DN4125_c0_g1_i6_orf1	black	0.67902
TRINITY_DN54524_c0_g1_i6_orf1	blue	0.952
TRINITY_DN119265_c0_g2_i1_orf1	black	0.88064
TRINITY_DN3545_c0_g1_i6_orf1	blue	0.80971
TRINITY_DN1826_c0_g2_i4_orf1	turquoise	0.88119
TRINITY_DN84883_c0_g1_i1_orf1	red	0.77755
TRINITY_DN18230_c1_g1_i1_orf1	black	0.91657
TRINITY_DN129226_c0_g1_i2_orf1	red	0.87252
TRINITY_DN1455_c0_g1_i8_orf1	yellow	0.97646
TRINITY_DN2065_c1_g2_i1_orf1	turquoise	0.88279
TRINITY_DN9608_c0_g1_i3_orf1	brown	0.89647
TRINITY_DN9724_c0_g1_i4_orf1	turquoise	0.6365
TRINITY_DN827_c1_g1_i1_orf1	black	0.87898
TRINITY_DN625_c2_g2_i2_orf1	green	0.96707
TRINITY_DN11117_c0_g1_i1_orf1	turquoise	0.96684
TRINITY_DN57074_c0_g2_i1_orf1	turquoise	0.96598
TRINITY_DN15811_c0_g1_i7_orf1	turquoise	0.74092
TRINITY_DN13350_c0_g1_i4_orf1	yellow	0.87938
TRINITY_DN4540_c0_g1_i9_orf1	red	0.76091
TRINITY_DN2650_c0_g1_i1_orf1	brown	0.8133
TRINITY_DN28711_c0_g1_i1_orf1	blue	0.82669
TRINITY_DN36987_c0_g1_i1_orf1	red	0.70204
TRINITY_DN1196_c0_g1_i5_orf1	blue	0.93235
TRINITY_DN56164_c0_g1_i1_orf1	blue	0.55468
TRINITY_DN20763_c0_g1_i2_orf1	turquoise	0.57884
TRINITY_DN3929_c0_g1_i1_orf1	turquoise	0.95071
TRINITY_DN110376_c0_g1_i1_orf1	turquoise	0.64718
TRINITY_DN119291_c0_g1_i1_orf1	green	0.4806
TRINITY_DN1231_c0_g1_i4_orf1	red	0.9469
TRINITY_DN2738_c1_g1_i3_orf1	turquoise	0.9097
TRINITY_DN4217_c0_g1_i2_orf1	blue	0.95579
TRINITY_DN12003_c0_g1_i1_orf1	brown	0.56919

TRINITY_DN2682_c0_g1_i4_orf1	turquoise	0.88161
TRINITY_DN3499_c0_g1_i8_orf1	red	0.86698
TRINITY_DN2026_c0_g1_i4_orf1	turquoise	0.89365
TRINITY_DN15903_c0_g1_i2_orf1	turquoise	0.51917
TRINITY_DN140538_c0_g2_i1_orf1	turquoise	0.80387
TRINITY_DN64181_c0_g1_i1_orf1	blue	0.83956
TRINITY_DN4439_c0_g2_i1_orf1	red	0.98065
TRINITY_DN28221_c0_g2_i1_orf1	green	0.95857
TRINITY_DN7957_c0_g1_i5_orf1	green	0.97238
TRINITY_DN2365_c0_g1_i6_orf1	turquoise	0.9166
TRINITY_DN5828_c0_g1_i5_orf1	turquoise	0.58143
TRINITY_DN703_c0_g1_i2_orf1	green	0.98619
TRINITY_DN29521_c0_g1_i1_orf1	turquoise	0.80587
TRINITY_DN48846_c0_g1_i1_orf1	brown	0.89956
TRINITY_DN2704_c0_g1_i5_orf1	turquoise	0.9689
TRINITY_DN61711_c0_g1_i1_orf1	turquoise	0.85974
TRINITY_DN39490_c0_g1_i1_orf1	turquoise	0.84536
TRINITY_DN95558_c0_g3_i1_orf1	black	0.91927
TRINITY_DN46625_c0_g1_i1_orf1	blue	0.87171
TRINITY_DN4810_c0_g1_i3_orf1	turquoise	0.95865
TRINITY_DN53281_c0_g1_i11_orf1	brown	0.68934
TRINITY_DN17437_c0_g1_i1_orf1	blue	0.90039
TRINITY_DN7986_c1_g1_i4_orf1	turquoise	0.46602
TRINITY_DN17326_c0_g1_i8_orf1	brown	0.98079
TRINITY_DN56708_c0_g3_i1_orfp1	yellow	0.87509
TRINITY_DN466_c0_g1_i5_orf1	turquoise	0.63186
TRINITY_DN99673_c0_g1_i1_orf1	pink	0.83998
TRINITY_DN15370_c0_g1_i4_orf1	turquoise	0.96339
TRINITY_DN2367_c1_g1_i20_orf1	blue	0.42723
TRINITY_DN67231_c0_g1_i1_orf1	yellow	0.9274
TRINITY_DN31314_c0_g1_i4_orf1	blue	0.96974
TRINITY_DN69236_c0_g1_i1_orf1	turquoise	0.82673
TRINITY_DN12087_c0_g1_i2_orf1	turquoise	0.65787
TRINITY_DN3273_c0_g1_i4_orf1	blue	0.83986
TRINITY_DN2694_c0_g1_i3_orf1	yellow	0.60161
TRINITY_DN919_c0_g1_i7_orf1	black	0.61555
TRINITY_DN12767_c0_g1_i1_orf1	turquoise	0.88695
TRINITY_DN172_c1_g1_i3_orf1	turquoise	0.68425
TRINITY_DN631_c0_g1_i6_orf1	turquoise	0.97981
TRINITY_DN63533_c0_g1_i2_orf1	yellow	0.74084
TRINITY_DN56430_c0_g1_i1_orf1	blue	0.82919
TRINITY_DN9904_c0_g1_i1_orf1	brown	0.46098
TRINITY_DN17351_c0_g1_i3_orf1	turquoise	0.98398
TRINITY_DN5346_c0_g1_i5_orf1	turquoise	0.55379
TRINITY_DN33893_c0_g1_i1_orf1	red	0.91682
TRINITY_DN2338_c0_g1_i3_orf1	grey	0.81913
TRINITY_DN5444_c0_g1_i1_orfp1	blue	0.87516
TRINITY_DN49530_c0_g1_i1_orf1	brown	0.93485
TRINITY_DN2924_c0_g1_i2_orf1	turquoise	0.72693
TRINITY_DN5721_c0_g1_i5_orf1	turquoise	0.86639
TRINITY_DN49872_c0_g2_i1_orf1	turquoise	0.81555
TRINITY_DN22589_c0_g1_i6_orfp1	brown	0.59765
TRINITY_DN46202_c0_g1_i1_orf1	blue	0.57675
TRINITY_DN4324_c0_g1_i1_orf1	blue	0.97921
TRINITY_DN31584_c0_g2_i2_orf1	grey	0.66316
TRINITY_DN1110_c1_g1_i9_orf1	blue	0.94943
TRINITY_DN56998_c0_g1_i2_orf1	yellow	0.49766
TRINITY_DN55160_c0_g1_i1_orf1	turquoise	0.89076

TRINITY_DN6262_c0_g2_i1_orf1	turquoise	0.67862
TRINITY_DN14336_c0_g3_i2_orf1	turquoise	0.95214
TRINITY_DN20442_c0_g2_i1_orf1	blue	0.70253
TRINITY_DN23360_c0_g1_i3_orf1	turquoise	0.94494
TRINITY_DN31503_c0_g1_i4_orf1	turquoise	0.86254
TRINITY_DN34703_c0_g1_i4_orf1	red	0.84736
TRINITY_DN31216_c0_g1_i2_orf1	turquoise	0.84121
TRINITY_DN157_c0_g1_i4_orf1	turquoise	0.90457
TRINITY_DN745_c5_g1_i2_orf1	brown	0.92291
TRINITY_DN33926_c0_g1_i1_orf1	turquoise	0.85614
TRINITY_DN33024_c0_g1_i1_orf1	turquoise	0.39483
TRINITY_DN4900_c0_g1_i6_orf1	green	0.83932
TRINITY_DN51429_c1_g1_i1_orf1	green	0.81856
TRINITY_DN4558_c0_g2_i1_orf1	green	0.7092
TRINITY_DN3131_c0_g1_i5_orf1	blue	0.56884
TRINITY_DN30097_c0_g1_i2_orf1	turquoise	0.87851
TRINITY_DN2913_c0_g1_i5_orf1	turquoise	0.86052
TRINITY_DN28018_c0_g6_i1_orf1	blue	0.92753
TRINITY_DN1008_c0_g1_i2_orf1	brown	0.92713
TRINITY_DN49742_c0_g1_i4_orf1	turquoise	0.95193
TRINITY_DN64403_c0_g2_i1_orf1	turquoise	0.97107
TRINITY_DN39170_c0_g1_i4_orf1	turquoise	0.59478
TRINITY_DN1725_c0_g1_i7_orf1	turquoise	0.95443
TRINITY_DN2441_c0_g1_i1_orf1	turquoise	0.81742
TRINITY_DN2769_c0_g1_i1_orf1	turquoise	0.94387
TRINITY_DN1237_c0_g1_i4_orf1	yellow	0.59276
TRINITY_DN123396_c0_g1_i1_orf1	turquoise	0.99194
TRINITY_DN36144_c0_g1_i3_orf1	green	0.90751
TRINITY_DN6532_c2_g1_i1_orf1	green	0.91607
TRINITY_DN2266_c0_g1_i6_orf1	turquoise	0.85104
TRINITY_DN13395_c0_g1_i1_orf1	red	0.95375
TRINITY_DN1616_c0_g1_i3_orf1	red	0.90075
TRINITY_DN34015_c0_g1_i7_orf1	turquoise	0.71606
TRINITY_DN33009_c0_g1_i2_orfp1	turquoise	0.35526
TRINITY_DN15318_c0_g1_i1_orf1	yellow	0.7057
TRINITY_DN101995_c0_g1_i1_orf1	yellow	0.93982
TRINITY_DN48554_c0_g1_i1_orf1	turquoise	0.97657
TRINITY_DN3801_c0_g1_i9_orf1	turquoise	0.82695
TRINITY_DN11772_c0_g1_i1_orf1	blue	0.94216
TRINITY_DN688_c0_g1_i8_orf1	blue	0.96502
TRINITY_DN31310_c0_g1_i1_orf1	green	0.80816
TRINITY_DN1267_c0_g2_i10_orf1	yellow	0.86808
TRINITY_DN8261_c0_g1_i1_orf1	turquoise	0.96978
TRINITY_DN147691_c0_g1_i1_orf1	yellow	0.765
TRINITY_DN30509_c0_g1_i9_orf1	yellow	0.5748
TRINITY_DN10539_c0_g1_i1_orf1	green	0.81671
TRINITY_DN4911_c0_g1_i6_orf1	green	0.66298
TRINITY_DN2100_c0_g1_i2_orf1	red	0.82022
TRINITY_DN3833_c0_g1_i4_orf1	blue	0.96939
TRINITY_DN13088_c0_g1_i5_orf1	blue	0.96877
TRINITY_DN3847_c1_g1_i1_orf1	turquoise	0.97438
TRINITY_DN3459_c0_g1_i4_orf1	blue	0.84507
TRINITY_DN49221_c0_g1_i1_orf1	turquoise	0.34851
TRINITY_DN86149_c0_g1_i1_orf1	turquoise	0.97188
TRINITY_DN1520_c0_g1_i9_orf1	red	0.6336
TRINITY_DN17326_c0_g1_i5_orf1	green	0.86558
TRINITY_DN60903_c0_g1_i1_orf1	turquoise	0.35244
TRINITY_DN18624_c0_g1_i5_orf1	blue	0.97865

TRINITY_DN2468_c0_g1_i7_orf1	yellow	0.81741
TRINITY_DN7942_c0_g1_i1_orf1	turquoise	0.92163
TRINITY_DN5513_c0_g1_i1_orf1	yellow	0.83274
TRINITY_DN116_c1_g1_i8_orf1	yellow	0.96237
TRINITY_DN4944_c0_g1_i5_orf1	turquoise	0.95249
TRINITY_DN22815_c0_g1_i2_orf1	turquoise	0.97922
TRINITY_DN12064_c0_g2_i1_orf1	turquoise	0.42987
TRINITY_DN16011_c0_g1_i3_orf1	red	0.91168
TRINITY_DN34423_c0_g1_i3_orf1	brown	0.92263
TRINITY_DN5772_c0_g1_i6_orf1	red	0.80625
TRINITY_DN21214_c0_g2_i1_orf1	turquoise	0.96383
TRINITY_DN30131_c0_g1_i1_orf1	turquoise	0.93585
TRINITY_DN11153_c0_g1_i1_orf1	turquoise	0.99521
TRINITY_DN17423_c0_g1_i2_orf1	brown	0.59743
TRINITY_DN18218_c0_g1_i7_orf1	blue	0.8265
TRINITY_DN3176_c0_g1_i2_orf1	turquoise	0.80538
TRINITY_DN36538_c0_g1_i2_orf1	turquoise	0.98716
TRINITY_DN4676_c0_g1_i16_orf1	blue	0.96159
TRINITY_DN2117_c0_g1_i1_orf1	turquoise	0.93903
TRINITY_DN14734_c0_g1_i2_orf1	turquoise	0.41198
TRINITY_DN10429_c0_g1_i2_orf1	turquoise	0.98599
TRINITY_DN8621_c0_g1_i5_orf1	turquoise	0.9262
TRINITY_DN7739_c0_g1_i2_orf1	black	0.93878
TRINITY_DN18249_c0_g1_i1_orf1	turquoise	0.78749
TRINITY_DN70409_c0_g1_i3_orf1	turquoise	0.62662
TRINITY_DN19186_c0_g1_i1_orf1	turquoise	0.97857
TRINITY_DN840_c5_g1_i11_orf1	grey	0.76533
TRINITY_DN14030_c0_g1_i1_orf1	turquoise	0.69962
TRINITY_DN5310_c2_g1_i2_orf1	brown	0.78752
TRINITY_DN2040_c0_g1_i6_orf1	brown	0.98023
TRINITY_DN38667_c0_g1_i9_orf1	turquoise	0.72669
TRINITY_DN107708_c0_g1_i1_orf1	yellow	0.77071
TRINITY_DN64810_c0_g1_i1_orf1	turquoise	0.9641
TRINITY_DN5215_c0_g1_i1_orf1	yellow	0.61393
TRINITY_DN7861_c0_g1_i5_orf1	pink	0.96884
TRINITY_DN58531_c0_g1_i1_orf1	turquoise	0.82122
TRINITY_DN24_c0_g1_i1_orf1	brown	0.86538
TRINITY_DN56379_c0_g1_i1_orf1	turquoise	0.7947
TRINITY_DN52768_c0_g1_i1_orf1	turquoise	0.98631
TRINITY_DN8561_c0_g4_i1_orf1	turquoise	0.84677
TRINITY_DN21719_c0_g2_i4_orf1	blue	0.94825
TRINITY_DN59388_c0_g1_i1_orf1	blue	0.8858
TRINITY_DN1576_c0_g1_i4_orf1	green	0.59331
TRINITY_DN82324_c0_g1_i4_orf1	turquoise	0.93719
TRINITY_DN338_c0_g1_i1_orf1	blue	0.97131
TRINITY_DN19702_c0_g1_i4_orf1	red	0.68728
TRINITY_DN147676_c0_g1_i1_orf1	turquoise	0.79833
TRINITY_DN972_c0_g1_i6_orf1	turquoise	0.73887
TRINITY_DN1650_c0_g1_i5_orf1	brown	0.77549
TRINITY_DN2396_c0_g1_i9_orfp1	brown	0.94321
TRINITY_DN2600_c0_g1_i7_orf1	red	0.97349
TRINITY_DN63719_c0_g1_i5_orf1	turquoise	0.9596
TRINITY_DN6392_c0_g1_i9_orf1	brown	0.74949
TRINITY_DN13718_c0_g1_i7_orf1	turquoise	0.71134
TRINITY_DN14436_c0_g1_i7_orf1	turquoise	0.99072
TRINITY_DN73_c0_g1_i6_orf1	brown	0.92443
TRINITY_DN574_c0_g1_i4_orf1	brown	0.95997
TRINITY_DN10484_c0_g1_i8_orf1	brown	0.81849

TRINITY_DN86309_c0_g1_i4_orf1	blue	0.9014
TRINITY_DN12964_c0_g1_i1_orf1	turquoise	0.95373
TRINITY_DN1370_c0_g1_i2_orf1	green	0.933
TRINITY_DN12424_c0_g1_i2_orf1	brown	0.90002
TRINITY_DN130075_c1_g2_i1_orf1	turquoise	0.89432
TRINITY_DN44288_c0_g1_i2_orf1	turquoise	0.97927
TRINITY_DN114834_c0_g1_i1_orf1	blue	0.94085
TRINITY_DN17726_c0_g1_i1_orf1	turquoise	0.81629
TRINITY_DN17299_c0_g1_i4_orf1	turquoise	0.9685
TRINITY_DN11552_c0_g1_i4_orf1	turquoise	0.84315
TRINITY_DN126127_c0_g1_i1_orf1	green	0.88461
TRINITY_DN62_c1_g1_i3_orf1	brown	0.67328
TRINITY_DN54410_c0_g2_i1_orf1	brown	0.39683
TRINITY_DN114198_c0_g1_i1_orf1	turquoise	0.78176
TRINITY_DN11108_c0_g1_i4_orf1	brown	0.8703
TRINITY_DN31327_c0_g2_i1_orf1	turquoise	0.97452
TRINITY_DN14250_c0_g1_i1_orf1	blue	0.65409
TRINITY_DN15380_c0_g1_i1_orf1	turquoise	0.99118
TRINITY_DN13530_c0_g1_i1_orf1	turquoise	0.98677
TRINITY_DN18568_c0_g1_i2_orfp1	brown	0.64114
TRINITY_DN4955_c0_g1_i2_orf1	brown	0.76999
TRINITY_DN37585_c0_g2_i1_orf1	blue	0.8893
TRINITY_DN52553_c0_g1_i1_orf1	blue	0.97321
TRINITY_DN26168_c0_g1_i1_orf1	turquoise	0.84659
TRINITY_DN65974_c0_g1_i2_orf1	brown	0.50899
TRINITY_DN30_c0_g1_i6_orf1	turquoise	0.93872
TRINITY_DN66822_c0_g1_i1_orf1	turquoise	0.98407
TRINITY_DN14242_c0_g1_i2_orfp1	brown	0.85026
TRINITY_DN12951_c1_g1_i5_orf1	red	0.6778
TRINITY_DN78546_c0_g5_i1_orf1	turquoise	0.85139
TRINITY_DN4213_c0_g1_i4_orf1	turquoise	0.89644
TRINITY_DN5559_c0_g1_i1_orf1	turquoise	0.94785
TRINITY_DN18933_c0_g1_i3_orf1	red	0.93611
TRINITY_DN4790_c0_g1_i6_orf1	black	0.94586
TRINITY_DN2283_c0_g2_i1_orf1	turquoise	0.96007
TRINITY_DN7613_c1_g2_i1_orf1	turquoise	0.91763
TRINITY_DN94755_c0_g1_i5_orfp1	turquoise	0.99178
TRINITY_DN26439_c0_g1_i2_orf1	brown	0.92517
TRINITY_DN4080_c0_g1_i8_orf1	brown	0.82068
TRINITY_DN12820_c0_g1_i1_orf1	red	0.85605
TRINITY_DN1494_c0_g1_i3_orf1	turquoise	0.98141
TRINITY_DN41166_c0_g1_i1_orf1	yellow	0.66626
TRINITY_DN19885_c0_g1_i1_orf1	blue	0.7563
TRINITY_DN25423_c0_g1_i1_orf1	brown	0.68526
TRINITY_DN5841_c0_g1_i2_orf1	turquoise	0.79766
TRINITY_DN3343_c0_g1_i4_orf1	turquoise	0.98997
TRINITY_DN334_c0_g1_i1_orf1	turquoise	0.98768
TRINITY_DN16924_c0_g1_i1_orf1	blue	0.69153
TRINITY_DN11665_c0_g1_i4_orf1	blue	0.8877
TRINITY_DN27958_c0_g1_i1_orf1	turquoise	0.52083
TRINITY_DN117_c0_g1_i5_orf1	turquoise	0.9836
TRINITY_DN2822_c0_g1_i4_orf1	yellow	0.71931
TRINITY_DN26013_c0_g1_i1_orf1	black	0.91656
TRINITY_DN25779_c0_g1_i6_orf1	turquoise	0.85719
TRINITY_DN4782_c0_g1_i1_orf1	red	0.76872
TRINITY_DN3600_c0_g1_i1_orf1	blue	0.80783
TRINITY_DN2859_c0_g1_i7_orf1	turquoise	0.94861
TRINITY_DN99900_c0_g1_i3_orf1	yellow	0.54387

TRINITY_DN21981_c0_g1_i8_orf1	turquoise	0.95575
TRINITY_DN7964_c0_g1_i1_orfp1	turquoise	0.96538
TRINITY_DN2941_c0_g1_i1_orf1	brown	0.5892
TRINITY_DN452_c0_g1_i4_orf1	brown	0.76447
TRINITY_DN41321_c1_g1_i3_orf1	blue	0.89866
TRINITY_DN12661_c0_g1_i3_orf1	black	0.91338
TRINITY_DN1563_c0_g1_i4_orf1	blue	0.97679
TRINITY_DN5840_c0_g1_i6_orf1	red	0.93303
TRINITY_DN2425_c0_g1_i1_orf1	turquoise	0.91571
TRINITY_DN4628_c0_g1_i1_orf1	brown	0.464
TRINITY_DN77642_c0_g1_i1_orf1	red	0.79218
TRINITY_DN2257_c0_g1_i4_orf1	turquoise	0.70961
TRINITY_DN1445_c0_g1_i1_orf1	turquoise	0.9878
TRINITY_DN17247_c0_g1_i14_orf1	brown	0.84531
TRINITY_DN2171_c0_g1_i1_orf1	black	0.93793
TRINITY_DN5238_c0_g1_i2_orf1	turquoise	0.95117
TRINITY_DN133760_c0_g1_i1_orf1	turquoise	0.85527
TRINITY_DN40439_c0_g1_i5_orf1	green	0.939
TRINITY_DN21609_c0_g2_i1_orf1	turquoise	0.95026
TRINITY_DN468_c0_g1_i3_orf1	turquoise	0.97797
TRINITY_DN6098_c1_g1_i5_orf1	brown	0.96654
TRINITY_DN1310_c0_g1_i4_orf1	turquoise	0.98488
TRINITY_DN3472_c1_g1_i4_orf1	red	0.91423
TRINITY_DN351_c14_g1_i2_orf1	turquoise	0.64918
TRINITY_DN11962_c0_g1_i2_orf1	yellow	0.58928
TRINITY_DN41296_c0_g1_i1_orf1	green	0.91796
TRINITY_DN535_c1_g1_i2_orf1	red	0.93995
TRINITY_DN2852_c0_g1_i9_orf1	turquoise	0.99069
TRINITY_DN2047_c0_g1_i1_orf1	turquoise	0.97768
TRINITY_DN1469_c0_g1_i1_orf1	turquoise	0.6763
TRINITY_DN19584_c0_g1_i2_orf1	turquoise	0.54009
TRINITY_DN27556_c0_g1_i1_orf1	turquoise	0.92893
TRINITY_DN4012_c0_g4_i2_orf1	turquoise	0.68196
TRINITY_DN3109_c0_g1_i5_orf1	black	0.94491
TRINITY_DN7618_c0_g1_i4_orf1	blue	0.86458
TRINITY_DN23444_c0_g1_i11_orf1	turquoise	0.42273
TRINITY_DN1675_c0_g1_i1_orf1	blue	0.70344
TRINITY_DN26186_c0_g1_i7_orf1	turquoise	0.94507
TRINITY_DN42310_c0_g1_i1_orf1	turquoise	0.9302
TRINITY_DN2207_c0_g1_i6_orf1	brown	0.84859
TRINITY_DN82944_c0_g1_i4_orf1	brown	0.96973
TRINITY_DN549_c0_g1_i14_orf1	yellow	0.94418
TRINITY_DN13515_c0_g1_i1_orf1	green	0.85341
TRINITY_DN17137_c0_g1_i2_orf1	yellow	0.954
TRINITY_DN3456_c0_g2_i1_orf1	turquoise	0.4574
TRINITY_DN42337_c0_g1_i6_orf1	red	0.92025
TRINITY_DN2251_c0_g1_i4_orf1	brown	0.84751
TRINITY_DN30169_c0_g1_i1_orfp1	green	0.95368
TRINITY_DN6483_c0_g1_i6_orf1	brown	0.98835
TRINITY_DN336_c0_g1_i6_orfp1	turquoise	0.96407
TRINITY_DN24469_c0_g2_i2_orf1	red	0.96347
TRINITY_DN13563_c0_g1_i1_orf1	yellow	0.74388
TRINITY_DN5775_c0_g1_i1_orf1	turquoise	0.96479
TRINITY_DN3949_c1_g1_i1_orf1	turquoise	0.81567
TRINITY_DN3310_c0_g1_i1_orf1	blue	0.93295
TRINITY_DN44261_c0_g1_i1_orf1	turquoise	0.65759
TRINITY_DN2312_c0_g1_i4_orf1	yellow	0.91826
TRINITY_DN11392_c0_g1_i4_orf1	turquoise	0.87046

TRINITY_DN2207_c0_g1_i4_orf1	yellow	0.76522
TRINITY_DN1391_c1_g2_i2_orf1	yellow	0.9829
TRINITY_DN26805_c0_g2_i3_orf1	green	0.90995
TRINITY_DN18242_c0_g1_i3_orf1	turquoise	0.90465
TRINITY_DN1707_c0_g1_i1_orf1	turquoise	0.9563
TRINITY_DN6586_c0_g1_i1_orf1	red	0.95704
TRINITY_DN1316_c0_g1_i1_orf1	turquoise	0.96168
TRINITY_DN13901_c0_g1_i4_orf1	black	0.84989
TRINITY_DN246_c1_g1_i5_orf1	blue	0.93782
TRINITY_DN2649_c0_g1_i3_orf1	turquoise	0.59829
TRINITY_DN5655_c0_g1_i2_orf1	green	0.8518
TRINITY_DN1637_c0_g1_i5_orf1	red	0.62378
TRINITY_DN66040_c0_g1_i2_orf1	blue	0.90553
TRINITY_DN695_c0_g1_i5_orf1	brown	0.82584
TRINITY_DN3275_c0_g2_i3_orf1	brown	0.86634
TRINITY_DN113778_c0_g2_i1_orf1	red	0.8885
TRINITY_DN12474_c0_g1_i6_orf1	turquoise	0.99105
TRINITY_DN15040_c0_g4_i1_orf1	brown	0.70254
TRINITY_DN58207_c0_g1_i1_orf1	turquoise	0.94495
TRINITY_DN80547_c0_g1_i5_orf1	yellow	0.60772
TRINITY_DN12586_c0_g1_i4_orf1	brown	0.86418
TRINITY_DN6908_c0_g1_i3_orf1	brown	0.71988
TRINITY_DN110400_c0_g1_i1_orf1	grey	-0.11296
TRINITY_DN28741_c0_g1_i3_orf1	brown	0.38173
TRINITY_DN2873_c0_g1_i7_orf1	yellow	0.47921
TRINITY_DN5562_c1_g1_i3_orf1	turquoise	0.97746
TRINITY_DN7579_c1_g3_i1_orf1	green	0.92216
TRINITY_DN7686_c0_g1_i4_orf1	turquoise	0.72847
TRINITY_DN467_c4_g1_i2_orf1	red	0.829
TRINITY_DN2812_c0_g1_i5_orf1	turquoise	0.66704
TRINITY_DN15160_c0_g1_i1_orf1	turquoise	0.67746
TRINITY_DN4289_c0_g1_i5_orf1	brown	0.74179
TRINITY_DN8527_c0_g2_i1_orfp1	brown	0.8818
TRINITY_DN6587_c0_g1_i3_orf1	brown	0.89163
TRINITY_DN67623_c0_g1_i1_orf1	turquoise	0.99208
TRINITY_DN128_c0_g1_i5_orf1	yellow	0.75005
TRINITY_DN5198_c0_g1_i5_orfp1	brown	0.88741
TRINITY_DN3859_c0_g1_i5_orf1	brown	0.87641
TRINITY_DN1239_c0_g1_i3_orf1	turquoise	0.97293
TRINITY_DN36883_c0_g1_i1_orf1	red	0.64613
TRINITY_DN15114_c0_g2_i1_orf1	blue	0.78005
TRINITY_DN10745_c0_g1_i14_orf1	red	0.94081
TRINITY_DN34413_c0_g1_i1_orf1	turquoise	0.5786
TRINITY_DN49936_c0_g2_i1_orf1	turquoise	0.93116
TRINITY_DN7868_c0_g1_i2_orf1	green	0.90437
TRINITY_DN22443_c0_g2_i3_orf1	blue	0.94275
TRINITY_DN105506_c0_g1_i8_orf1	yellow	0.9019
TRINITY_DN248_c0_g1_i12_orf1	yellow	0.83455
TRINITY_DN2943_c2_g2_i1_orf1	black	0.91029
TRINITY_DN139438_c0_g1_i1_orf1	turquoise	0.90182
TRINITY_DN3822_c0_g1_i7_orf1	green	0.95303
TRINITY_DN6362_c0_g1_i4_orf1	red	0.72882
TRINITY_DN987_c0_g1_i3_orf1	green	0.76007
TRINITY_DN56877_c0_g1_i4_orf1	grey	0.19484
TRINITY_DN33346_c0_g1_i1_orf1	brown	0.93614
TRINITY_DN32822_c0_g1_i1_orf1	turquoise	0.97077
TRINITY_DN1380_c0_g1_i5_orf1	yellow	0.41323
TRINITY_DN2170_c1_g1_i3_orf1	blue	0.92466

TRINITY_DN806_c0_g2_i1_orf1	blue	0.90789
TRINITY_DN3127_c0_g1_i9_orf1	turquoise	0.9478
TRINITY_DN16868_c0_g2_i1_orf1	green	0.79687
TRINITY_DN5437_c0_g1_i1_orf1	yellow	0.85602
TRINITY_DN121893_c0_g1_i1_orf1	turquoise	0.82165
TRINITY_DN1304_c0_g1_i6_orf1	black	0.92009
TRINITY_DN1353_c0_g1_i1_orf1	turquoise	0.9489
TRINITY_DN1023_c1_g1_i1_orf1	green	0.8867
TRINITY_DN7477_c0_g1_i1_orf1	brown	0.54056
TRINITY_DN131371_c0_g1_i1_orf1	turquoise	0.75822
TRINITY_DN3229_c0_g1_i1_orf1	turquoise	0.9471
TRINITY_DN2344_c1_g1_i4_orf1	turquoise	0.50924
TRINITY_DN4041_c0_g1_i6_orf1	blue	0.89118
TRINITY_DN1790_c1_g1_i3_orf1	turquoise	0.93577
TRINITY_DN3005_c0_g1_i7_orf1	blue	0.98162
TRINITY_DN29034_c0_g1_i2_orf1	turquoise	0.98368
TRINITY_DN29402_c0_g1_i1_orf1	blue	0.72158
TRINITY_DN6747_c0_g1_i7_orf1	turquoise	0.99224
TRINITY_DN5554_c0_g1_i2_orf1	turquoise	0.96858
TRINITY_DN781_c0_g1_i7_orf1	green	0.80783
TRINITY_DN14306_c0_g1_i1_orf1	turquoise	0.59923
TRINITY_DN132857_c0_g1_i1_orf1	blue	0.84783
TRINITY_DN81258_c0_g1_i2_orf1	yellow	0.76065
TRINITY_DN76036_c0_g1_i1_orf1	turquoise	0.96662
TRINITY_DN5153_c1_g1_i1_orf1	brown	0.93309
TRINITY_DN2914_c0_g1_i1_orf1	turquoise	0.86403
TRINITY_DN5211_c0_g1_i1_orf1	red	0.96434
TRINITY_DN122423_c0_g5_i1_orf1	turquoise	0.81435
TRINITY_DN779_c0_g1_i3_orf1	yellow	0.93099
TRINITY_DN5991_c0_g1_i6_orf1	turquoise	0.53387
TRINITY_DN49047_c0_g1_i2_orf1	turquoise	0.5934
TRINITY_DN59422_c0_g1_i2_orf1	yellow	0.94268
TRINITY_DN51737_c0_g1_i3_orf1	red	0.88074
TRINITY_DN14429_c0_g1_i2_orf1	turquoise	0.87183
TRINITY_DN41664_c0_g1_i4_orf1	turquoise	0.6067
TRINITY_DN23714_c0_g1_i4_orf1	turquoise	0.68849
TRINITY_DN3251_c0_g1_i6_orf1	green	0.87357
TRINITY_DN111985_c0_g1_i1_orf1	green	0.62639
TRINITY_DN13118_c0_g1_i6_orf1	red	0.92729
TRINITY_DN21743_c0_g1_i1_orf1	green	0.89252
TRINITY_DN8241_c0_g1_i3_orf1	yellow	0.6553
TRINITY_DN25341_c0_g1_i1_orf1	turquoise	0.94026
TRINITY_DN36460_c0_g1_i2_orf1	brown	0.90595
TRINITY_DN1423_c0_g1_i8_orf1	blue	0.90819
TRINITY_DN1720_c0_g1_i3_orf1	green	0.9207
TRINITY_DN2927_c0_g1_i2_orf1	turquoise	0.96856
TRINITY_DN139335_c0_g2_i1_orf1	blue	0.72035
TRINITY_DN8986_c0_g1_i1_orf1	black	0.72373
TRINITY_DN51568_c0_g1_i1_orf1	turquoise	0.96296
TRINITY_DN20118_c0_g1_i4_orfp1	turquoise	0.67719
TRINITY_DN26688_c0_g1_i2_orf1	turquoise	0.99456
TRINITY_DN6472_c0_g1_i5_orf1	black	0.8445
TRINITY_DN4694_c0_g1_i6_orf1	green	0.69806
TRINITY_DN39200_c0_g1_i5_orf1	turquoise	0.99198
TRINITY_DN5300_c0_g1_i3_orf1	yellow	0.84617
TRINITY_DN8245_c0_g1_i3_orf1	green	0.9492
TRINITY_DN10652_c0_g1_i4_orf1	yellow	0.66778
TRINITY_DN74116_c0_g1_i2_orf1	brown	0.46353

TRINITY_DN1540_c0_g1_i7_orf1	green	0.97499
TRINITY_DN71610_c0_g1_i1_orf1	blue	0.74601
TRINITY_DN23734_c0_g1_i1_orf1	turquoise	0.91108
TRINITY_DN23978_c0_g1_i2_orf1	blue	0.59713
TRINITY_DN661_c0_g1_i1_orf1	blue	0.96054
TRINITY_DN56910_c0_g2_i1_orf1	turquoise	0.94711
TRINITY_DN4281_c0_g1_i1_orf1	turquoise	0.85512
TRINITY_DN4403_c0_g1_i3_orf1	turquoise	0.71551
TRINITY_DN1752_c0_g1_i18_orf1	turquoise	0.77522
TRINITY_DN35099_c0_g1_i1_orf1	red	0.79223
TRINITY_DN7493_c0_g1_i1_orf1	green	0.68721
TRINITY_DN1718_c1_g1_i5_orf1	yellow	0.92679
TRINITY_DN31118_c1_g1_i1_orf1	yellow	0.86603
TRINITY_DN4494_c0_g1_i1_orf1	red	0.94426
TRINITY_DN12065_c0_g1_i4_orf1	blue	0.846
TRINITY_DN1114_c0_g1_i4_orf1	brown	0.62698
TRINITY_DN143852_c0_g1_i1_orf1	turquoise	0.73229
TRINITY_DN5880_c0_g2_i2_orf1	brown	0.84128
TRINITY_DN12873_c0_g1_i3_orf1	brown	0.86531
TRINITY_DN48590_c0_g1_i1_orf1	red	0.91575
TRINITY_DN120500_c0_g1_i1_orf1	pink	0.84862
TRINITY_DN6291_c0_g1_i4_orf1	pink	0.85837
TRINITY_DN9094_c0_g1_i1_orf1	turquoise	0.88255
TRINITY_DN8729_c0_g1_i7_orf1	turquoise	0.89641
TRINITY_DN5857_c0_g1_i13_orf1	turquoise	0.95421
TRINITY_DN2300_c0_g1_i1_orf1	turquoise	0.95419
TRINITY_DN74654_c0_g1_i4_orf1	green	0.9234
TRINITY_DN1326_c0_g1_i2_orf1	blue	0.94719
TRINITY_DN1004_c0_g2_i1_orf1	green	0.87618
TRINITY_DN7251_c0_g1_i3_orf1	turquoise	0.8338
TRINITY_DN670_c0_g1_i15_orf1	turquoise	0.46622
TRINITY_DN1716_c0_g1_i14_orf1	red	0.85909
TRINITY_DN11370_c0_g1_i6_orf1	black	0.92605
TRINITY_DN102260_c0_g1_i1_orf1	turquoise	0.59529
TRINITY_DN625_c9_g1_i7_orf1	brown	0.93399
TRINITY_DN10264_c1_g1_i5_orf1	green	0.91042
TRINITY_DN57111_c0_g1_i1_orf1	yellow	0.90583
TRINITY_DN2795_c0_g1_i1_orf1	turquoise	0.65431
TRINITY_DN8224_c0_g1_i7_orf1	brown	0.93117
TRINITY_DN6423_c0_g1_i5_orf1	blue	0.94651
TRINITY_DN925_c0_g1_i5_orf1	grey	0.24231
TRINITY_DN3132_c0_g1_i10_orf1	turquoise	0.75583
TRINITY_DN36494_c0_g1_i1_orf1	turquoise	0.98563
TRINITY_DN61222_c0_g1_i1_orf1	turquoise	0.97255
TRINITY_DN23616_c0_g1_i4_orf1	turquoise	0.68052
TRINITY_DN4550_c1_g1_i19_orf1	blue	0.57601
TRINITY_DN31001_c0_g1_i1_orf1	yellow	0.85824
TRINITY_DN1722_c0_g1_i2_orf1	blue	0.83117
TRINITY_DN47389_c0_g1_i2_orf1	turquoise	0.95572
TRINITY_DN2946_c0_g1_i1_orf1	blue	0.95535
TRINITY_DN829_c0_g1_i8_orf1	yellow	0.78066
TRINITY_DN38506_c0_g1_i4_orf1	brown	0.9698
TRINITY_DN80660_c0_g1_i1_orf1	brown	0.79472
TRINITY_DN1014_c0_g2_i8_orf1	green	0.8538
TRINITY_DN108200_c0_g1_i1_orf1	turquoise	0.96152
TRINITY_DN9003_c0_g1_i20_orf1	turquoise	0.98229
TRINITY_DN5553_c0_g1_i4_orf1	blue	0.914
TRINITY_DN143833_c0_g1_i1_orf1	turquoise	0.57494

TRINITY_DN10940_c0_g1_i10_orfp1	turquoise	0.9652
TRINITY_DN472_c1_g1_i3_orf1	black	0.85144
TRINITY_DN26293_c0_g1_i4_orf1	red	0.85186
TRINITY_DN62091_c0_g1_i1_orf1	brown	0.91767
TRINITY_DN19662_c0_g2_i1_orf1	green	0.9827
TRINITY_DN3411_c0_g1_i2_orf1	turquoise	0.92321
TRINITY_DN60680_c0_g1_i2_orf1	turquoise	0.97716
TRINITY_DN1180_c0_g1_i4_orf1	yellow	0.96964
TRINITY_DN11376_c0_g2_i1_orf1	turquoise	0.9826
TRINITY_DN81926_c0_g1_i1_orf1	turquoise	0.92988
TRINITY_DN1882_c0_g1_i4_orf1	turquoise	0.69857
TRINITY_DN9164_c0_g1_i3_orf1	turquoise	0.93457
TRINITY_DN15597_c0_g1_i1_orf1	black	0.88499
TRINITY_DN5686_c0_g1_i4_orf1	turquoise	0.97721
TRINITY_DN1567_c0_g1_i15_orf1	turquoise	0.91466
TRINITY_DN48460_c0_g1_i1_orf1	red	0.84607
TRINITY_DN16643_c0_g2_i4_orf1	blue	0.95867
TRINITY_DN2102_c0_g1_i11_orf1	blue	0.8493
TRINITY_DN6312_c0_g1_i1_orf1	red	0.88776
TRINITY_DN749_c0_g1_i1_orf1	red	0.89021
TRINITY_DN5531_c7_g1_i2_orf1	green	0.5826
TRINITY_DN18918_c0_g1_i3_orf1	turquoise	0.73459
TRINITY_DN4121_c0_g1_i1_orf1	turquoise	0.95955
TRINITY_DN6125_c0_g1_i2_orf1	turquoise	0.73179
TRINITY_DN18329_c0_g1_i2_orf1	turquoise	0.72812
TRINITY_DN2652_c0_g2_i1_orf1	blue	0.99468
TRINITY_DN11347_c0_g1_i1_orf1	turquoise	0.97403
TRINITY_DN5126_c0_g2_i1_orf1	pink	0.80412
TRINITY_DN9376_c1_g1_i3_orf1	brown	0.85755
TRINITY_DN45097_c0_g1_i5_orf1	black	0.9524
TRINITY_DN1173_c1_g1_i10_orf1	yellow	0.79353
TRINITY_DN3307_c1_g1_i2_orf1	turquoise	0.35993
TRINITY_DN6698_c0_g2_i2_orf1	brown	0.84775
TRINITY_DN121439_c0_g1_i1_orf1	blue	0.65249
TRINITY_DN332_c0_g1_i6_orf1	turquoise	0.89296
TRINITY_DN1293_c1_g1_i4_orf1	black	0.54308
TRINITY_DN757_c3_g1_i2_orf1	brown	0.98681
TRINITY_DN3212_c0_g1_i7_orfp1	green	0.81864
TRINITY_DN2012_c0_g1_i3_orf1	blue	0.93264
TRINITY_DN41334_c0_g1_i1_orf1	turquoise	0.36851
TRINITY_DN10458_c0_g1_i1_orf1	turquoise	0.92756
TRINITY_DN57137_c0_g1_i1_orfp1	brown	0.97124
TRINITY_DN32514_c0_g2_i1_orf1	blue	0.96738
TRINITY_DN4123_c0_g1_i1_orf1	brown	0.82273
TRINITY_DN33178_c0_g1_i1_orf1	turquoise	0.92175
TRINITY_DN10792_c0_g2_i5_orf1	turquoise	0.98607
TRINITY_DN22213_c0_g1_i3_orf1	red	0.63741
TRINITY_DN17995_c0_g4_i1_orf1	blue	0.76223
TRINITY_DN17907_c0_g1_i13_orf1	brown	0.60475
TRINITY_DN8143_c0_g1_i6_orf1	black	0.83759
TRINITY_DN29229_c0_g1_i5_orfp1	turquoise	0.91001
TRINITY_DN146364_c0_g1_i1_orf1	turquoise	0.58487
TRINITY_DN1386_c0_g1_i6_orf1	turquoise	0.94732
TRINITY_DN1783_c0_g1_i2_orf1	green	0.7926
TRINITY_DN17861_c0_g1_i5_orf1	turquoise	0.82179
TRINITY_DN13732_c0_g2_i3_orf1	blue	0.75164
TRINITY_DN26195_c0_g1_i6_orf1	yellow	0.56927
TRINITY_DN25285_c0_g1_i1_orf1	turquoise	0.59492

TRINITY_DN9410_c0_g1_i4_orf1	turquoise	0.86989
TRINITY_DN17905_c0_g3_i1_orf1	turquoise	0.96237
TRINITY_DN16816_c0_g1_i1_orf1	turquoise	0.95831
TRINITY_DN9059_c0_g1_i1_orf1	turquoise	0.94674
TRINITY_DN58872_c0_g1_i1_orfp1	blue	0.91245
TRINITY_DN12775_c0_g1_i10_orfp1	brown	0.92647
TRINITY_DN2160_c0_g1_i13_orf1	turquoise	0.65594
TRINITY_DN29190_c0_g1_i4_orf1	brown	0.29411
TRINITY_DN96801_c0_g1_i1_orf1	brown	0.52002
TRINITY_DN35633_c0_g2_i1_orf1	red	0.95085
TRINITY_DN3687_c0_g1_i1_orf1	yellow	0.4071
TRINITY_DN35635_c0_g1_i1_orf1	turquoise	0.96983
TRINITY_DN268_c3_g1_i2_orf1	brown	0.90235
TRINITY_DN7785_c0_g1_i1_orf1	blue	0.91029
TRINITY_DN33488_c0_g1_i2_orf1	brown	0.96814
TRINITY_DN14721_c0_g1_i2_orf1	red	0.91306
TRINITY_DN8291_c0_g1_i3_orf1	turquoise	0.88546
TRINITY_DN13063_c0_g1_i1_orf1	blue	0.78283
TRINITY_DN628_c0_g1_i7_orf1	turquoise	0.95793
TRINITY_DN5962_c0_g1_i1_orf1	turquoise	0.39523
TRINITY_DN72859_c0_g1_i1_orf1	turquoise	0.76812
TRINITY_DN6638_c0_g1_i1_orf1	turquoise	0.59428
TRINITY_DN2673_c0_g3_i1_orf1	turquoise	0.89944
TRINITY_DN15234_c0_g1_i3_orf1	turquoise	0.8364
TRINITY_DN109144_c0_g1_i5_orf1	turquoise	0.91741
TRINITY_DN61_c0_g2_i3_orf1	turquoise	0.82348
TRINITY_DN36612_c0_g1_i1_orf1	turquoise	0.56165
TRINITY_DN3471_c0_g1_i1_orf1	turquoise	0.92638
TRINITY_DN57454_c0_g1_i4_orf1	turquoise	0.91825
TRINITY_DN7900_c0_g1_i4_orf1	green	0.96653
TRINITY_DN3511_c0_g2_i1_orf1	turquoise	0.76591
TRINITY_DN6967_c0_g1_i3_orf1	blue	0.72921
TRINITY_DN2505_c0_g1_i1_orf1	turquoise	0.94333
TRINITY_DN37307_c0_g1_i4_orf1	brown	0.84081
TRINITY_DN106_c0_g1_i3_orf1	blue	0.45311
TRINITY_DN9079_c0_g1_i5_orf1	brown	0.96435
TRINITY_DN47605_c0_g2_i1_orf1	turquoise	0.9111
TRINITY_DN10058_c0_g1_i1_orf1	turquoise	0.81711
TRINITY_DN15478_c0_g1_i1_orf1	green	0.82311
TRINITY_DN15896_c0_g1_i4_orf1	turquoise	0.98071
TRINITY_DN24971_c0_g1_i3_orf1	blue	0.99134
TRINITY_DN34536_c0_g1_i6_orf1	turquoise	0.99574
TRINITY_DN699_c0_g2_i1_orf1	blue	0.86569
TRINITY_DN7233_c0_g2_i1_orf1	yellow	0.72405
TRINITY_DN2745_c0_g1_i2_orf1	red	0.7679
TRINITY_DN30037_c0_g1_i5_orf1	pink	0.77685
TRINITY_DN1197_c0_g1_i6_orf1	yellow	0.73244
TRINITY_DN35377_c0_g1_i3_orf1	turquoise	0.71818
TRINITY_DN659_c0_g1_i3_orf1	turquoise	0.95855
TRINITY_DN8454_c0_g1_i4_orf1	yellow	0.84293
TRINITY_DN64510_c0_g1_i1_orf1	turquoise	0.99042
TRINITY_DN7711_c1_g1_i3_orf1	blue	0.90097
TRINITY_DN5029_c0_g1_i1_orf1	green	0.96608
TRINITY_DN58125_c0_g1_i1_orf1	blue	0.97662
TRINITY_DN48878_c0_g2_i1_orf1	green	0.94989
TRINITY_DN21545_c0_g1_i2_orf1	brown	0.91832
TRINITY_DN195_c0_g3_i6_orf1	black	0.85512
TRINITY_DN2442_c0_g1_i6_orf1	brown	0.88786

TRINITY_DN5558_c0_g1_i4_orf1	brown	0.75131
TRINITY_DN9862_c0_g2_i1_orf1	turquoise	0.91817
TRINITY_DN35147_c0_g1_i1_orf1	green	0.60669
TRINITY_DN57998_c1_g3_i1_orf1	blue	0.99325
TRINITY_DN106476_c0_g1_i3_orf1	turquoise	0.98453
TRINITY_DN32362_c0_g1_i1_orf1	red	0.72019
TRINITY_DN5697_c0_g1_i1_orf1	turquoise	0.87423
TRINITY_DN394_c0_g1_i2_orf1	brown	0.89778
TRINITY_DN472_c0_g1_i6_orf1	green	0.82261
TRINITY_DN146524_c0_g1_i1_orf1	blue	0.95362
TRINITY_DN6014_c1_g1_i2_orf1	blue	0.92748
TRINITY_DN2049_c1_g1_i2_orf1	brown	0.44108
TRINITY_DN2623_c1_g1_i3_orf1	red	0.89211
TRINITY_DN955_c0_g1_i2_orf1	black	0.8575
TRINITY_DN5087_c0_g1_i6_orf1	turquoise	0.9815
TRINITY_DN14565_c0_g1_i11_orf1	turquoise	0.95811
TRINITY_DN67495_c0_g1_i1_orf1	turquoise	0.54245
TRINITY_DN10007_c0_g1_i1_orf1	turquoise	0.8408
TRINITY_DN3087_c0_g1_i1_orf1	brown	0.63733
TRINITY_DN4956_c0_g1_i6_orf1	turquoise	0.97903
TRINITY_DN6822_c0_g2_i4_orf1	black	0.95665
TRINITY_DN3461_c0_g1_i1_orf1	turquoise	0.87375
TRINITY_DN978_c9_g2_i1_orf1	red	0.93542
TRINITY_DN104596_c0_g1_i1_orf1	green	0.97494
TRINITY_DN135_c0_g1_i1_orf1	turquoise	0.83077
TRINITY_DN4051_c0_g1_i1_orf1	turquoise	0.30158
TRINITY_DN10637_c0_g1_i4_orf1	turquoise	0.98773
TRINITY_DN30663_c0_g1_i1_orf1	turquoise	0.97015
TRINITY_DN2912_c0_g1_i1_orf1	blue	0.78449
TRINITY_DN20426_c0_g2_i1_orf1	turquoise	0.4479
TRINITY_DN11065_c0_g2_i1_orf1	turquoise	0.87106
TRINITY_DN15836_c0_g1_i1_orf1	turquoise	0.72622
TRINITY_DN8621_c0_g1_i4_orf1	yellow	0.68865
TRINITY_DN115082_c0_g1_i5_orf1	turquoise	0.50808
TRINITY_DN130439_c0_g1_i1_orf1	black	0.79153
TRINITY_DN93764_c0_g1_i1_orf1	brown	0.76695
TRINITY_DN526_c0_g1_i1_orf1	black	0.32951
TRINITY_DN7062_c0_g1_i1_orf1	black	0.87633
TRINITY_DN1298_c0_g1_i3_orf1	turquoise	0.81146
TRINITY_DN37418_c0_g1_i4_orf1	brown	0.4605
TRINITY_DN47591_c0_g1_i2_orf1	turquoise	0.40916
TRINITY_DN2855_c0_g1_i6_orf1	black	0.79618
TRINITY_DN7570_c0_g1_i18_orf1	yellow	0.44596
TRINITY_DN13322_c0_g1_i6_orf1	brown	0.96189
TRINITY_DN16933_c0_g1_i10_orf1	turquoise	0.9115
TRINITY_DN616_c1_g1_i6_orf1	brown	0.6484
TRINITY_DN34347_c0_g1_i1_orf1	turquoise	0.63079
TRINITY_DN17693_c0_g1_i10_orf1	green	0.61384
TRINITY_DN11825_c0_g1_i4_orf1	turquoise	0.98818
TRINITY_DN98334_c0_g1_i1_orf1	blue	0.96484
TRINITY_DN119797_c0_g1_i1_orf1	turquoise	0.78951
TRINITY_DN1437_c0_g1_i6_orf1	turquoise	0.85262
TRINITY_DN3698_c0_g1_i4_orf1	turquoise	0.38479
TRINITY_DN2392_c0_g2_i1_orf1	brown	0.86428
TRINITY_DN2936_c0_g1_i1_orf1	red	0.98419
TRINITY_DN1265_c0_g1_i4_orf1	pink	0.8929
TRINITY_DN5244_c0_g1_i1_orf1	turquoise	0.93798
TRINITY_DN15256_c0_g1_i8_orf1	red	0.74707

TRINITY_DN1355_c0_g1_i5_orf1	yellow	0.96582
TRINITY_DN648_c0_g1_i5_orf1	black	0.67562
TRINITY_DN1503_c0_g1_i6_orf1	brown	0.89639
TRINITY_DN285_c0_g1_i4_orf1	brown	0.96561
TRINITY_DN705_c0_g1_i1_orf1	turquoise	0.77829
TRINITY_DN8386_c0_g1_i6_orf1	green	0.71218
TRINITY_DN3370_c0_g1_i5_orf1	turquoise	0.83149
TRINITY_DN825_c2_g1_i5_orf1	turquoise	0.95085
TRINITY_DN9239_c0_g1_i1_orf1	blue	0.8684
TRINITY_DN143_c0_g3_i1_orf1	turquoise	0.91441
TRINITY_DN40586_c0_g1_i4_orf1	turquoise	0.84022
TRINITY_DN1880_c0_g1_i4_orf1	brown	0.96614
TRINITY_DN2173_c0_g1_i1_orf1	turquoise	0.91149
TRINITY_DN14220_c0_g1_i1_orf1	turquoise	0.81557
TRINITY_DN144342_c0_g1_i1_orfp1	grey	-0.22427
TRINITY_DN5952_c0_g1_i6_orf1	red	0.91327
TRINITY_DN46409_c0_g1_i1_orf1	turquoise	0.95787
TRINITY_DN2947_c0_g1_i4_orf1	brown	0.88647
TRINITY_DN251_c0_g1_i2_orf1	green	0.74291
TRINITY_DN10716_c1_g1_i1_orf1	turquoise	0.91392
TRINITY_DN6439_c0_g1_i1_orf1	turquoise	0.82698
TRINITY_DN144190_c0_g1_i1_orf1	turquoise	0.98399
TRINITY_DN23089_c0_g1_i1_orf1	turquoise	0.68487
TRINITY_DN54150_c0_g1_i1_orf1	turquoise	0.93582
TRINITY_DN9029_c0_g1_i4_orf1	yellow	0.64292
TRINITY_DN24318_c0_g1_i1_orf1	turquoise	0.86493
TRINITY_DN4954_c0_g1_i5_orf1	black	0.85865
TRINITY_DN27110_c0_g1_i4_orf1	red	0.29095
TRINITY_DN46372_c0_g2_i1_orf1	yellow	0.73325
TRINITY_DN65681_c0_g1_i1_orf1	blue	0.91668
TRINITY_DN4808_c0_g1_i3_orf1	red	0.96183
TRINITY_DN798_c1_g1_i3_orf1	green	0.89685
TRINITY_DN1706_c0_g1_i7_orf1	turquoise	0.92718
TRINITY_DN115210_c0_g4_i1_orf1	blue	0.46699
TRINITY_DN1868_c0_g1_i1_orf1	red	0.91797
TRINITY_DN3110_c0_g1_i4_orf1	brown	0.46797
TRINITY_DN83542_c0_g1_i1_orf1	turquoise	0.77869
TRINITY_DN26963_c0_g1_i1_orf1	turquoise	0.98579
TRINITY_DN80328_c0_g1_i9_orf1	green	0.99205
TRINITY_DN108433_c0_g1_i1_orf1	green	0.93537
TRINITY_DN4367_c0_g1_i1_orf1	blue	0.96778
TRINITY_DN25251_c0_g2_i1_orf1	red	0.51447
TRINITY_DN78686_c0_g1_i1_orf1	red	0.92766
TRINITY_DN2719_c1_g1_i6_orf1	yellow	0.87084
TRINITY_DN19187_c0_g1_i1_orf1	turquoise	0.9489
TRINITY_DN7122_c0_g1_i1_orf1	turquoise	0.66292
TRINITY_DN45400_c0_g1_i1_orf1	red	0.51753
TRINITY_DN1895_c0_g1_i2_orf1	yellow	0.95985
TRINITY_DN4602_c0_g1_i4_orf1	green	0.89861
TRINITY_DN1330_c0_g1_i1_orf1	turquoise	0.96798
TRINITY_DN592_c0_g1_i6_orf1	yellow	0.96543
TRINITY_DN1201_c0_g1_i4_orf1	brown	0.9201
TRINITY_DN24693_c1_g1_i1_orf1	turquoise	0.93591
TRINITY_DN1841_c0_g1_i2_orf1	green	0.82149
TRINITY_DN43350_c0_g3_i1_orf1	brown	0.95805
TRINITY_DN2993_c0_g1_i4_orf1	turquoise	0.99518
TRINITY_DN1211_c0_g1_i10_orf1	yellow	0.95052
TRINITY_DN30150_c0_g1_i7_orf1	turquoise	0.55037

TRINITY_DN29707_c0_g1_i2_orf1	turquoise	0.81238
TRINITY_DN6351_c0_g1_i4_orf1	turquoise	0.92247
TRINITY_DN24668_c0_g1_i8_orf1	black	0.79965
TRINITY_DN81715_c0_g1_i1_orf1	turquoise	0.79024
TRINITY_DN26882_c0_g1_i1_orf1	blue	0.96972
TRINITY_DN2921_c1_g1_i4_orf1	black	0.8584
TRINITY_DN36230_c0_g1_i1_orf1	brown	0.40441
TRINITY_DN33867_c0_g1_i9_orf1	turquoise	0.85701
TRINITY_DN59885_c0_g1_i3_orf1	green	0.96576
TRINITY_DN578_c0_g1_i3_orf1	blue	0.39032
TRINITY_DN3135_c0_g1_i6_orf1	turquoise	0.83151
TRINITY_DN185_c0_g1_i6_orf1	red	0.86002
TRINITY_DN2621_c0_g1_i1_orf1	yellow	0.71774
TRINITY_DN20796_c0_g1_i4_orf1	blue	0.95162
TRINITY_DN12527_c0_g1_i4_orf1	turquoise	0.78012
TRINITY_DN34821_c0_g1_i4_orf1	red	0.89786
TRINITY_DN2406_c0_g1_i6_orf1	brown	0.55151
TRINITY_DN1999_c0_g1_i9_orf1	turquoise	0.73853
TRINITY_DN2061_c0_g1_i3_orf1	red	0.79291
TRINITY_DN27903_c0_g1_i1_orf1	pink	0.83893
TRINITY_DN4572_c0_g1_i2_orf1	blue	0.9572
TRINITY_DN19939_c0_g1_i4_orf1	turquoise	0.56664
TRINITY_DN104597_c0_g1_i2_orf1	pink	0.60382
TRINITY_DN142485_c0_g1_i1_orf1	turquoise	0.90535
TRINITY_DN6325_c0_g1_i9_orf1	brown	0.89369
TRINITY_DN29009_c0_g2_i3_orf1	pink	0.71995
TRINITY_DN1266_c2_g1_i1_orf1	turquoise	0.82389
TRINITY_DN14532_c0_g1_i1_orf1	blue	0.89361
TRINITY_DN142657_c0_g1_i1_orf1	green	0.96385
TRINITY_DN7289_c0_g1_i1_orf1	turquoise	0.87542
TRINITY_DN19690_c0_g1_i1_orf1	green	0.88583
TRINITY_DN31663_c0_g1_i2_orf1	turquoise	0.953
TRINITY_DN28592_c0_g1_i2_orf1	turquoise	0.99145
TRINITY_DN640_c0_g1_i5_orf1	turquoise	0.74586
TRINITY_DN3486_c0_g1_i5_orf1	yellow	0.77212
TRINITY_DN49785_c1_g1_i3_orf1	blue	0.97489
TRINITY_DN971_c0_g1_i5_orfp1	brown	0.95454
TRINITY_DN33089_c0_g1_i1_orf1	red	0.77702
TRINITY_DN42269_c2_g1_i1_orf1	turquoise	0.9042
TRINITY_DN2967_c0_g1_i7_orf1	turquoise	0.62405
TRINITY_DN29969_c0_g1_i5_orf1	yellow	0.75175
TRINITY_DN549_c0_g1_i7_orf1	yellow	0.97185
TRINITY_DN237_c1_g1_i1_orf1	turquoise	0.84344
TRINITY_DN3332_c0_g1_i2_orf1	turquoise	0.98625
TRINITY_DN43576_c0_g1_i3_orf1	turquoise	0.93519
TRINITY_DN34857_c0_g1_i6_orf1	turquoise	0.50099
TRINITY_DN3008_c0_g1_i12_orf1	turquoise	0.90763
TRINITY_DN1209_c0_g1_i9_orf1	brown	0.86441
TRINITY_DN8985_c0_g1_i4_orf1	green	0.94388
TRINITY_DN31637_c0_g1_i3_orf1	turquoise	0.94935
TRINITY_DN64759_c0_g1_i1_orf1	turquoise	0.62481
TRINITY_DN2474_c0_g1_i5_orf1	red	0.8341
TRINITY_DN9475_c0_g1_i6_orf1	blue	0.91433
TRINITY_DN3383_c0_g1_i5_orf1	blue	0.55609
TRINITY_DN1759_c0_g1_i4_orf1	black	0.8958
TRINITY_DN20658_c0_g2_i3_orf1	brown	0.93704
TRINITY_DN38650_c0_g1_i2_orf1	turquoise	0.91941
TRINITY_DN15046_c0_g1_i8_orf1	turquoise	0.95037

TRINITY_DN83374_c0_g1_i1_orf1	blue	0.92829
TRINITY_DN5748_c0_g1_i5_orf1	brown	0.92531
TRINITY_DN132043_c0_g1_i1_orf1	turquoise	0.87972
TRINITY_DN81719_c0_g1_i1_orf1	turquoise	0.92305
TRINITY_DN6563_c0_g1_i1_orf1	turquoise	0.93516
TRINITY_DN3962_c0_g1_i6_orf1	blue	0.6788
TRINITY_DN129869_c0_g4_i1_orf1	yellow	0.66862
TRINITY_DN9732_c0_g1_i7_orf1	blue	0.94412
TRINITY_DN3758_c0_g1_i2_orf1	brown	0.91006
TRINITY_DN74538_c0_g1_i1_orf1	blue	0.96993
TRINITY_DN103_c0_g1_i1_orf1	green	0.68831
TRINITY_DN109503_c0_g1_i4_orf1	brown	0.27612
TRINITY_DN40345_c0_g1_i6_orf1	turquoise	0.94491
TRINITY_DN2082_c0_g1_i2_orf1	turquoise	0.98361
TRINITY_DN61536_c0_g7_i1_orf1	blue	0.69331
TRINITY_DN661_c1_g2_i1_orf1	blue	0.89559
TRINITY_DN4301_c0_g1_i5_orf1	turquoise	0.76424
TRINITY_DN13167_c0_g1_i1_orf1	blue	0.82152
TRINITY_DN26503_c0_g1_i1_orf1	turquoise	0.94783
TRINITY_DN311_c0_g1_i8_orfp1	brown	0.44926
TRINITY_DN19662_c4_g1_i1_orf1	green	0.98414
TRINITY_DN140212_c0_g1_i1_orf1	green	0.80497
TRINITY_DN6163_c0_g1_i4_orf1	red	0.87031
TRINITY_DN30027_c0_g1_i1_orf1	turquoise	0.88964
TRINITY_DN68397_c0_g1_i2_orf1	blue	0.95758
TRINITY_DN7451_c0_g1_i10_orf1	red	0.83359
TRINITY_DN2630_c0_g3_i3_orf1	turquoise	0.85316
TRINITY_DN6130_c0_g1_i6_orf1	red	0.80324
TRINITY_DN9733_c0_g1_i2_orf1	green	0.841
TRINITY_DN14679_c0_g1_i1_orf1	turquoise	0.98393
TRINITY_DN5112_c0_g1_i1_orf1	turquoise	0.9377
TRINITY_DN15736_c0_g1_i2_orf1	red	0.62301
TRINITY_DN12608_c0_g1_i1_orf1	turquoise	0.80901
TRINITY_DN5191_c0_g2_i1_orf1	brown	0.75688
TRINITY_DN12442_c0_g1_i4_orf1	turquoise	0.87938
TRINITY_DN19731_c0_g1_i1_orf1	brown	0.93955
TRINITY_DN104507_c0_g1_i2_orf1	red	0.85729
TRINITY_DN3976_c0_g1_i6_orf1	turquoise	0.94728
TRINITY_DN21124_c0_g1_i4_orf1	red	0.96592
TRINITY_DN2782_c0_g1_i7_orf1	red	0.60248
TRINITY_DN72_c0_g1_i16_orf1	blue	0.85503
TRINITY_DN31943_c0_g1_i1_orf1	blue	0.86066
TRINITY_DN23229_c0_g1_i2_orf1	blue	0.88924
TRINITY_DN7294_c0_g2_i4_orf1	turquoise	0.9171
TRINITY_DN110888_c0_g1_i2_orf1	turquoise	0.8462
TRINITY_DN12231_c0_g1_i1_orf1	green	0.90496
TRINITY_DN2120_c0_g1_i2_orf1	turquoise	0.73732
TRINITY_DN27979_c0_g1_i2_orf1	turquoise	0.99356
TRINITY_DN53462_c0_g1_i1_orf1	turquoise	0.76127
TRINITY_DN195_c8_g1_i1_orf1	yellow	0.38091
TRINITY_DN55154_c0_g2_i1_orf1	brown	0.52413
TRINITY_DN16899_c0_g2_i1_orf1	turquoise	0.51628
TRINITY_DN1540_c0_g1_i9_orf1	brown	0.70483
TRINITY_DN45446_c0_g1_i2_orf1	turquoise	0.93018
TRINITY_DN11948_c0_g1_i8_orf1	brown	0.96544
TRINITY_DN701_c1_g1_i4_orf1	brown	0.44961
TRINITY_DN1651_c0_g2_i1_orf1	green	0.88425
TRINITY_DN46022_c0_g1_i1_orf1	turquoise	0.86155

TRINITY_DN14922_c0_g1_i4_orf1	yellow	0.62008
TRINITY_DN71699_c0_g1_i1_orf1	blue	0.84555
TRINITY_DN21506_c0_g1_i4_orf1	turquoise	0.96892
TRINITY_DN15265_c0_g1_i1_orf1	turquoise	0.52906
TRINITY_DN46173_c0_g3_i2_orf1	turquoise	0.98164
TRINITY_DN28759_c0_g1_i1_orf1	red	0.94858
TRINITY_DN31676_c0_g1_i4_orf1	green	0.9255
TRINITY_DN5914_c1_g1_i9_orf1	brown	0.97888
TRINITY_DN122321_c0_g1_i1_orf1	brown	0.89957
TRINITY_DN7516_c0_g2_i1_orf1	red	0.79947
TRINITY_DN1571_c0_g1_i9_orf1	turquoise	0.5001
TRINITY_DN2783_c0_g1_i22_orf1	turquoise	0.89608
TRINITY_DN7434_c0_g1_i1_orf1	turquoise	0.48269
TRINITY_DN33430_c0_g1_i5_orf1	blue	0.5516
TRINITY_DN64772_c0_g1_i1_orf1	green	0.97571
TRINITY_DN31303_c0_g1_i4_orf1	turquoise	0.94489
TRINITY_DN15706_c0_g2_i5_orf1	turquoise	0.922
TRINITY_DN3055_c0_g1_i9_orf1	yellow	0.70714
TRINITY_DN7378_c0_g1_i5_orf1	blue	0.68403
TRINITY_DN8846_c0_g1_i1_orf1	turquoise	0.32878
TRINITY_DN6241_c0_g1_i1_orf1	turquoise	0.41551
TRINITY_DN556_c0_g2_i1_orf1	turquoise	0.51845
TRINITY_DN4502_c0_g1_i3_orf1	brown	0.96501
TRINITY_DN335_c1_g1_i5_orf1	brown	0.93181
TRINITY_DN4030_c0_g2_i1_orf1	turquoise	0.95473
TRINITY_DN479_c6_g1_i2_orf1	blue	0.90559
TRINITY_DN2013_c0_g1_i15_orf1	green	0.80786
TRINITY_DN2040_c0_g1_i15_orfp1	yellow	0.90523
TRINITY_DN26243_c0_g1_i2_orf1	turquoise	0.83727
TRINITY_DN1093_c0_g1_i6_orf1	green	0.96417
TRINITY_DN17003_c1_g1_i1_orf1	blue	0.94473
TRINITY_DN12401_c0_g2_i4_orf1	turquoise	0.70795
TRINITY_DN8641_c0_g1_i1_orf1	blue	0.97041
TRINITY_DN9100_c0_g1_i5_orf1	red	0.92005
TRINITY_DN4732_c0_g1_i2_orf1	red	0.54667
TRINITY_DN17759_c0_g1_i5_orf1	brown	0.91798
TRINITY_DN10520_c0_g1_i2_orf1	turquoise	0.9813
TRINITY_DN3769_c0_g1_i1_orf1	yellow	0.87283
TRINITY_DN152_c0_g1_i4_orf1	turquoise	0.69214
TRINITY_DN11981_c0_g1_i7_orf1	brown	0.92146
TRINITY_DN1326_c0_g1_i1_orf1	blue	0.93018
TRINITY_DN40669_c0_g2_i1_orf1	blue	0.9663
TRINITY_DN26947_c0_g1_i1_orf1	turquoise	0.48485
TRINITY_DN31585_c0_g1_i1_orf1	turquoise	0.88022
TRINITY_DN24043_c0_g1_i1_orf1	turquoise	0.97828
TRINITY_DN63662_c0_g4_i1_orf1	turquoise	0.98871
TRINITY_DN418_c1_g1_i3_orf1	green	0.86981
TRINITY_DN22824_c0_g1_i4_orf1	yellow	0.61071
TRINITY_DN5011_c0_g1_i1_orf1	turquoise	0.51774
TRINITY_DN782_c0_g1_i5_orf1	red	0.72046
TRINITY_DN1093_c0_g1_i4_orf1	green	0.95821
TRINITY_DN36006_c0_g1_i5_orf1	turquoise	0.68448
TRINITY_DN77480_c0_g1_i2_orf1	yellow	0.8868
TRINITY_DN34399_c0_g1_i1_orf1	turquoise	0.74996
TRINITY_DN1354_c5_g1_i1_orf1	blue	0.68962
TRINITY_DN5829_c0_g2_i1_orf1	red	0.88316
TRINITY_DN6307_c0_g1_i5_orf1	turquoise	0.93114
TRINITY_DN9400_c0_g1_i8_orf1	brown	0.84897

TRINITY_DN401_c0_g1_i15_orf1	turquoise	0.95675
TRINITY_DN2596_c0_g1_i2_orf1	turquoise	0.42375
TRINITY_DN15858_c0_g1_i2_orf1	brown	0.93698
TRINITY_DN1400_c0_g1_i21_orf1	turquoise	0.8054
TRINITY_DN4589_c0_g2_i1_orf1	brown	0.55472
TRINITY_DN4546_c0_g1_i3_orf1	turquoise	0.71505
TRINITY_DN3134_c0_g1_i1_orf1	turquoise	0.98941
TRINITY_DN2323_c0_g1_i4_orf1	blue	0.94252
TRINITY_DN430_c0_g1_i5_orf1	turquoise	0.98161
TRINITY_DN3616_c0_g1_i4_orf1	blue	0.83433
TRINITY_DN8580_c0_g1_i12_orf1	green	0.86045
TRINITY_DN28376_c0_g1_i15_orfp1	turquoise	0.61296
TRINITY_DN44709_c0_g1_i1_orf1	blue	0.74833
TRINITY_DN9309_c0_g1_i5_orf1	turquoise	0.90542
TRINITY_DN7464_c1_g1_i1_orf1	turquoise	0.95223
TRINITY_DN1285_c0_g2_i1_orf1	turquoise	0.99067
TRINITY_DN7630_c0_g2_i1_orf1	brown	0.9871
TRINITY_DN4944_c1_g1_i4_orf1	turquoise	0.97102
TRINITY_DN47930_c0_g1_i4_orf1	yellow	0.89502
TRINITY_DN3292_c2_g1_i4_orf1	turquoise	0.96677
TRINITY_DN3332_c0_g1_i9_orf1	yellow	0.79029
TRINITY_DN20133_c0_g1_i1_orf1	brown	0.58811
TRINITY_DN12508_c0_g1_i1_orf1	turquoise	0.61556
TRINITY_DN942_c0_g1_i1_orf1	red	0.93788
TRINITY_DN6462_c0_g1_i5_orf1	green	0.92576
TRINITY_DN91946_c0_g1_i1_orf1	brown	0.93105
