# Supplementary material for: Comparative Proteomics and Metabonomics Analysis of Different Diapause Stages Revealed a New Regulation Mechanism of Diapause in Loxostege sticticalis (Lepidoptera: Pyralidae)
Source: Molecules. 2024 Jul 25;29(15):3472. doi: 10.3390/molecules29153472 (PMC11314584; doi:10.3390/molecules29153472)
Supplement: Supplementary file 1 [file molecules-29-03472-s001.zip › analysis process/proteomic/WGCNA/Table of correlation coefficients between modules and phenotypes.pdf]

	JH	Fat acid	Trehalose
MEgreen	-0.694 (0.0041)	-0.347 (0.205)	0 (1)
MEturquo	0.694 (0.0041)	0 (1)	-0.231 (0.407)
MEblue	-0.694 (0.0041)	-0.309 (0.262)	0.154 (0.584)
MEyellow	-0.231 (0.407)	0.694 (0.0041)	0.347 (0.205)
MEbrown	-0.694 (0.0041)	0.347 (0.205)	0.694 (0.0041)
MEblack	-0.694 (0.0041)	0.386 (0.155)	0 (1)
MEgrey	0.347 (0.205)	-0.656 (0.00792)	-0.0772 (0.784)
MEred	0.347 (0.205)	0 (1)	-0.347 (0.205)
MEpink	0 (1)	-0.347 (0.205)	0.694 (0.0041)
