# Supplementary figures and images for: Comparative Proteomics and Metabonomics Analysis of Different Diapause Stages Revealed a New Regulation Mechanism of Diapause in Loxostege sticticalis (Lepidoptera: Pyralidae)
Source: Molecules. 2024 Jul 25;29(15):3472. doi: 10.3390/molecules29153472 (PMC11314584; doi:10.3390/molecules29153472)

# Protein molecular weight distribution

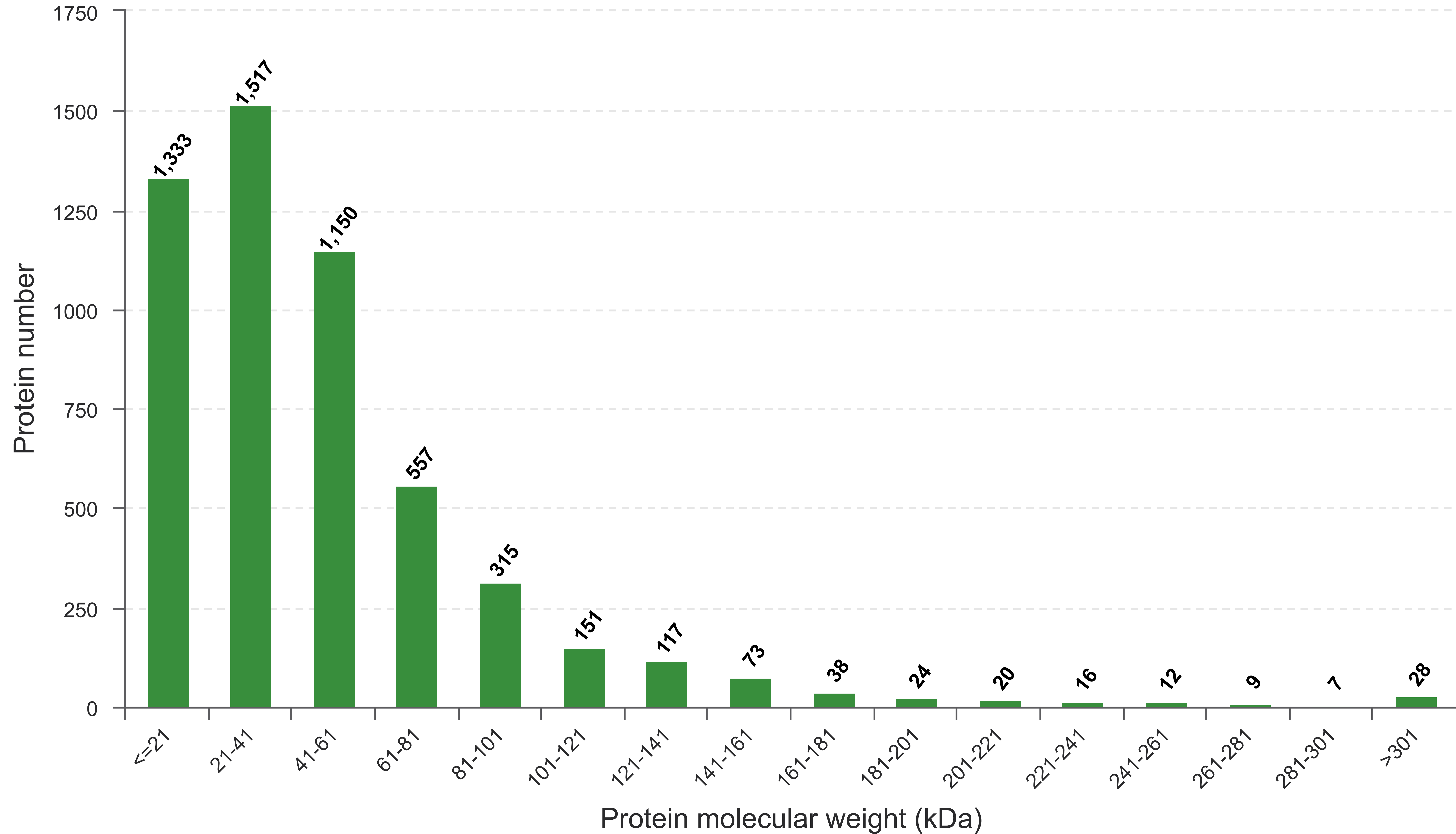

Supplement: Supplementary file 1 [file molecules-29-03472-s001.zip › analysis process/Supplement file S1-S6/Figure S1. protein-molecular-weight.pdf]

Peptide length distribution

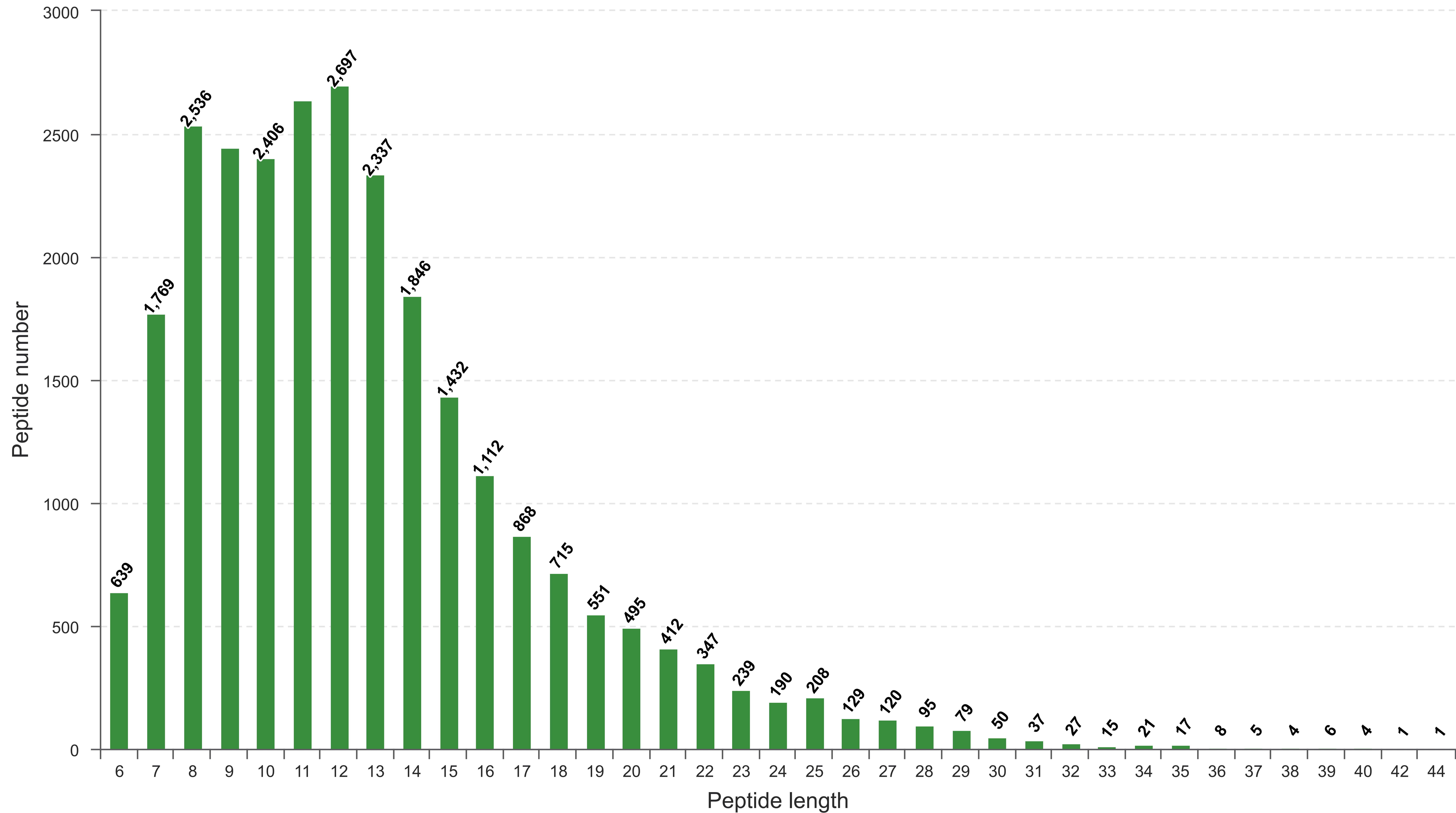

Supplement: Supplementary file 1 [file molecules-29-03472-s001.zip › analysis process/Supplement file S1-S6/Figure S2. peptide-length-distribut.pdf]
