# Supplementary material for: Comparative Proteomics and Metabonomics Analysis of Different Diapause Stages Revealed a New Regulation Mechanism of Diapause in Loxostege sticticalis (Lepidoptera: Pyralidae)
Source: Molecules. 2024 Jul 25;29(15):3472. doi: 10.3390/molecules29153472 (PMC11314584; doi:10.3390/molecules29153472)
Supplement: Supplementary file 1 [file neg.pdf › analysis process/metabolic/Data preprocessing/neg.pdf]

ID	Metabolite	Metab ID	Library ID	level	KEGG Compound ID	M/Z	Retention time	Mode	Adducts	Formula
neg_1006	(2R,3S)-2-methylisocitrate	metab_6233	HMDB0303970	B(ii)	-	237.9888	0.5753	neg	M+Cl	C7H7O7-3
neg_1013	Sarcosine	metab_6241	PW_C000185;HMDB0000271;MJDBOTE0000363	B(ii)	C00213	88.0395	0.5828	neg	M-H	C3H7NO2
neg_1015	Miglitol	metab_6243	HMDB0014634;MJDBOTE0000907	B(ii)	C07708	242.0796	0.5828	neg	M+Cl	C8H17NO5
neg_1018	Trehalose 6-Phosphate	metab_6246	HMDB0001124	B(i)	C00689	421.076	0.5828	neg	M-H	C12H23O14P
neg_1025	Bialaphos	metab_6253	HMDB0249177	B(ii)	C06457	344.1024	0.5986	neg	M+Na-2H	C11H22N3O6P
neg_1028	Allantoic Acid	metab_6256	HMDB0001209	B(i)	C00499	175.0467	0.6065	neg	M-H	C4H8N4O4
neg_1031	Threonic Acid	metab_6260	HMDB0000943	B(i)	C01620	135.0292	0.6145	neg	M-H	C4H8O5
neg_1034	2-Hydroxyethanesulfonate	metab_6263	HMDB0003903	B(ii)	C05123	124.9908	0.6223	neg	M-H	C2H6O4S
neg_1043	Mesylate	metab_6273	HMDB0240280	B(ii)	C11145	94.98	0.6382	neg	M-H	CH4O3S
neg_1052	Fructose lactate	metab_6281	HMDB0252491	B(ii)	-	267.0725	0.6461	neg	M-H	C9H16O9
neg_1056	Glyceric Acid	metab_6284	PW_C000090;HMDB0000139	B(i)	C00258	105.0186	0.6541	neg	M-H	C3H6O4
neg_1058	Cytidine 3'-Phosphate	metab_6286	HMDB0240312	B(i)	C05822	322.0449	0.6541	neg	M-H	C9H14N3O8P
neg_1059	Molinate	metab_6287	HMDB0254835	B(ii)	C11086	351.0701	0.6541	neg	M+K-2H	C13H14N8O2
neg_1070	Pentose	metab_6299	HMDB0250736;HMDB0003402;MJDBOTE0001676	B(i)	C08348	149.0449	0.67	neg	M-H	C5H10O5
neg_1075	8-oxo-dGDP	metab_6303	HMDB0059648	B(ii)	C20176	424.0074	0.67	neg	M-H2O-H	C10H15N5O11P2
neg_1079	Imidazole-4-acetaldehyde	metab_6306	HMDB0003905;PW_C001992	B(ii)	C05130	265.0944	0.678	neg	2M+FA-H	C5H6N2O
neg_1081	Adenosine diphosphate ribose	metab_6308	PW_C000914;HMDB0001178	B(ii)	C00301	540.0551	0.678	neg	M-H2O-H	C15H23N5O14P2
neg_1084	10-Deacetyltaxol	metab_6311	HMDB0244265	B(ii)	-	846.2916	0.678	neg	M+Cl	C45H49NO13
neg_1087	Hydroxyethylflurazepam	metab_6314	HMDB0253267	B(ii)	-	440.0968	0.6858	neg	M+K-2H	C21H23ClFN3O2
neg_1105	Acetyl adenylate	metab_6331	PW_C002987;HMDB0006880	B(ii)	C05993	426.0231	0.7256	neg	M+K-2H	C12H16N5O8P
neg_1106	Alanylhydroxyproline	metab_6332	HMDB0028688	B(ii)	-	247.0936	0.7336	neg	M+FA-H	C8H14N2O4
neg_1108	Malvidin 3-glucoside	metab_6333	HMDB0030777	B(ii)	C12140	528.0997	0.7336	neg	M+Cl	C23H25O12+
neg_1110	Cyclo(Ac-cys-asn-dmt-amf-gly-asp-cys)	metab_6336	HMDB0244566	B(ii)	-	850.2281	0.7336	neg	M-H2O-H	C34H47N9O12S3
neg_1115	Hippuryl-L-lysine	metab_6340	HMDB0253017	B(ii)	-	342.1199	0.7416	neg	M+Cl	C15H21N3O4
neg_1118	Stachyoside A	metab_6342	HMDB0039092	B(ii)	-	525.1867	0.7416	neg	M-H	C21H34O15
neg_1122	Hispidin	metab_6346	HMDB0253181	B(ii)	-	291.0533	0.7495	neg	M+FA-H	C13H10O5
neg_1127	Gamma-Glutamylalanine	metab_6351	HMDB0036301;HMDB0006248	B(ii)	C03740	217.0827	0.7655	neg	M-H	C8H14N2O5
neg_1129	5-Aminovaleric Acid	metab_6353	HMDB0003355	B(i)	C00431	116.0711	0.7734	neg	M-H	C5H11NO2
neg_1130	Succinic anhydride	metab_6355	HMDB0032523	B(ii)	C19524	145.0137	0.7734	neg	M+FA-H	C4H4O3
neg_1136	Sinapoyl-(S)-malate	metab_6359	HMDB0304485	B(ii)	C02887	337.0587	0.7814	neg	M-H	C15H14O9-2
neg_1149	Exherin	metab_6369	HMDB0252147	B(ii)	-	591.1797	0.8213	neg	M+Na-2H	C22H34N8O6S2
neg_1163	[(2S,3R,4R,5R)-5-(6-Aminopurin-9-yl)-3,4-	metab_6382	HMDB0247869	B(ii)	-	408.0123	0.8695	neg	M-H2O-H	C10H15N5O10P2
neg_1169	Alpha-Ketoglutarate cyanohydrin	metab_6386	HMDB0248224	B(ii)	-	166.9958	0.8855	neg	M+Na-2H	C5H6O5
neg_1174	Glaucarubol 15-O-beta-D-glucopyranoside	metab_6390	HMDB0035909	B(ii)	-	593.1951	0.8935	neg	M+Cl	C26H38O13
neg_1176	(S)-Isowillardiine	metab_6392	HMDB0030376	B(ii)	C08289	244.0573	0.9013	neg	M+FA-H	C7H9N3O4
neg_1199	Amylopectin	metab_6414	HMDB0003255	B(ii)	C00317	827.2709	0.9567	neg	M-H	C30H52O26
neg_1204	Guanine	metab_6421	PW_C000086;HMDB0000132;MJDBOTE0000457	B(i)	C00242	150.0415	0.9807	neg	M-H	C5H5N5O
neg_1206	L-Dopa	metab_6423	HMDB0000181;PW_C000117	B(ii)	C00355	196.061	0.9887	neg	M-H	C9H11NO4
neg_1208	Uridine Diphosphate Glucuronic Acid	metab_6425	HMDB0000935;PW_C000746	B(i)	C00167	579.0287	0.9887	neg	M-H	C15H22N2O18P2
neg_1211	4-Methylbenzoic Acid	metab_6428	HMDB0029635	B(i)	C01454	135.0445	0.9966	neg	M-H	C8H8O2
neg_1212	(2s,3r,4s,5r)-3,4,5-Trihydroxy-6-oxopiperid	metab_6429	HMDB0242545	B(ii)	-	236.0425	0.9966	neg	M+FA-H	C6H9NO6
neg_1218	Acipimox	metab_6435	HMDB0247941	B(ii)	-	153.0299	1.0124	neg	M-H	C6H6N2O3
neg_1219	3'-Adenylic Acid	metab_6436	HMDB0003540;HMDB0258140	B(i)	C01367	346.0563	1.0124	neg	M-H	C10H14N5O7P
neg_1223	Methionyl-Methionine	metab_6441	HMDB0028979	B(ii)	-	315.0587	1.0283	neg	M+Cl	C10H20N2O3S2
neg_1226	N-acetylaspartate	metab_6444	HMDB0000812	B(i)	C01042	174.0403	1.0443	neg	M-H	C6H9NO5
neg_1227	N-(2,6-Difluorophenyl)-2-oxoglycyl-N-((1S	metab_6445	HMDB0259684	B(ii)	-	530.0802	1.0443	neg	M-H2O-H	C22H17F6N3O7
neg_1231	2-Furoic Acid	metab_6449	HMDB0000617;MJDBOTE0000456	B(i)	C01546	111.0082	1.0602	neg	M-H	C5H4O3
neg_1235	Bis(carboxyethyl)carboxyfluorescein acetoxy	metab_6453	HMDB0244423;HMDB0249256	B(ii)	-	627.088	1.0679	neg	M+Cl	C30H24O13
neg_1238	Hibifolin	metab_6455	HMDB0253170	B(ii)	-	539.0682	1.0757	neg	M+FA-H	C21H18O14
neg_1241	GDP-L-fucose	metab_6459	HMDB0001095;PW_C000861	B(i)	C00325	588.0765	1.1076	neg	M-H	C16H25N5O15P2
neg_1244	2-carboxy-L-xylonolactone	metab_6461	HMDB0304063	B(ii)	-	381.0306	1.1234	neg	2M-H	C6H7O7-
neg_1245	L-Glutamic acid 5-phosphate	metab_6462	HMDB0001228	B(ii)	C03287	226.0119	1.1314	neg	M-H	C5H10NO7P
neg_1247	D-Ribulose 5-Phosphate	metab_6464	PW_C000479;HMDB0000618	B(i)	C00199	211.001	1.1393	neg	M-H2O-H	C5H11O8P
neg_1257	2'-Fluoro-2',3'-dideoxyinosine	metab_6474	HMDB0245537	B(ii)	-	275.0585	1.1792	neg	M+Na-2H	C10H11FN4O3
neg_1258	(2,6-Dioxo-3H-purin-9-yl) pyridine-3-carb	metab_6475	HMDB0242495	B(ii)	-	318.0459	1.1792	neg	M+FA-H	C11H7N5O4
neg_1259	Columbianadin	metab_6476	HMDB0250407	B(ii)	-	365.0801	1.1792	neg	M+K-2H	C19H20O5
neg_1263	2'-Deoxyadenosine 5'-phosphate	metab_6481	HMDB0000905	B(i)	C00360	330.0614	1.1872	neg	M-H	C10H14N5O6P
neg_1269	Homocarnosine	metab_6486	HMDB0000745	B(ii)	C00884	285.1208	1.2031	neg	M+FA-H	C10H16N4O3
neg_1275	1-O-Sinapoyl-beta-D-glucose	metab_6492	HMDB0302379	B(ii)	C01175	423.0669	1.2111	neg	M+K-2H	C17H22O10
neg_1276	Fructosamine	metab_6493	HMDB0002030	B(ii)	-	160.061	1.219	neg	M-H2O-H	C6H13NO5
neg_1278	Imidazoleacetic acid riboside	metab_6495	HMDB0002331	B(ii)	C05131	279.0601	1.219	neg	M+Na-2H	C10H14N2O6
neg_1282	Deoxyribose	metab_6500	HMDB0245099;HMDB0003224	B(i)	C01801	133.05	1.227	neg	M-H	C5H10O4
neg_1293	N-Acetyl-L-methionine	metab_6511	HMDB0011745	B(ii)	C02712	236.0599	1.243	neg	M+FA-H	C7H13NO3S
neg_1296	(S)-a-Amino-2,5-dihydro-5-oxo-4-isoxazo	metab_6514	HMDB0029404	B(ii)	-	333.0945	1.251	neg	M-H	C12H18N2O9
neg_1298	8-Hydroxyguanosine	metab_6516	HMDB0002044	B(ii)	-	280.0691	1.259	neg	M-H2O-H	C10H13N5O6
neg_13	L-Glutamine	metab_6518	HMDB0000641;PW_C000500;HMDB0003423;MJDBOT	B(i)	C00064	145.0614	0.5597	neg	M-H, M+K-2H,	C5H10N2O3
neg_1306	Lipoamide	metab_6526	HMDB0304823;HMDB0000962;LMFA08010006;PW_C	B(ii)	C00248	469.1352	1.291	neg	2M+Hac-H	C8H15NOS2
neg_1309	Gamma-Glutamyl-2-aminobutyrate	metab_6529	HMDB0242176;HMDB0240645	B(ii)	-	266.0674	1.315	neg	M+Cl	C9H15N2O5-
neg_1315	1,2-Dehydrosalsolinol	metab_6533	HMDB0012490	B(ii)	-	222.0771	1.331	neg	M+FA-H	C10H11NO2
neg_1319	2-Aminomuconic acid	metab_6537	HMDB0001241	B(ii)	C02220	359.0739	1.347	neg	2M+FA-H	C6H7NO4
neg_1325	Fenoxaprop	metab_6543	HMDB0252210	B(ii)	-	354.0135	1.371	neg	M+Na-2H	C16H12ClNO5
neg_1330	Tetrahydrodipicolinate	metab_6549	HMDB0012289	B(ii)	C03972	152.0346	1.395	neg	M-H2O-H	C7H9NO4
neg_1331	2-(3,4-Dihydroxybenzoyloxy)-4,6-dihydrox	metab_6550	HMDB0059651	B(ii)	C04524	342.988	1.395	neg	M+K-2H	C14H10O8
neg_1332	(1Ar,2Z,4E,14R,15aR)-8-chloro-9,11-dihyd	metab_6551	HMDB0242304	B(ii)	-	385.0447	1.395	neg	M+Na-2H	C18H17ClO6
neg_1338	Imidazolelactic acid	metab_6556	HMDB0002320	B(ii)	C05132	311.1001	1.427	neg	2M-H	C6H8N2O3
neg_134	Leonuriside A	metab_6558	HMDB0031721	B(ii)	-	369.0574	0.504	neg	M+Na-2H, M+	C14H20O9
neg_1348	Oxytetracycline	metab_6565	HMDB0014733;LMPK07000005;PW_C009084;MJDBO	B(ii)	C06624	495.1189	1.443	neg	M+Cl	C22H24N2O9
neg_1358	Tazobactam	metab_6575	HMDB0015544	B(ii)	C07771	281.0363	1.5151	neg	M-H2O-H	C10H12N4O5S
neg_1360	6-Hydroxy-R-acenocoumarol	metab_6578	HMDB0060882	B(ii)	-	414.0853	1.5231	neg	M+FA-H	C19H15NO7
neg_1377	Salicin	metab_6595	HMDB0003546	B(ii)	C01451	331.1041	1.6062	neg	M+FA-H	C13H18O7
neg_1378	Nicotinamide riboside	metab_6596	PW_C000681;HMDB0000855	B(ii)	C03150	236.0791	1.6123	neg	M-H2O-H	C11H15N2O5+
neg_1381	Benzoyl glucuronide (Benzoic acid)	metab_6600	HMDB0010324	B(ii)	-	335.0181	1.6279	neg	M+K-2H	C13H14O8
neg_1387	Nitrofurazone	metab_6606	HMDB0014480	B(ii)	C08042	179.0206	1.669	neg	M-H2O-H	C6H6N4O4
neg_1389	Trans-4-Carboxymethylenebut-2-en-4-olic	metab_6608	HMDB0060518	B(ii)	C12838	279.0163	1.669	neg	2M-H	C6H4O4
neg_1395	2,5-Dioxopyrrolidin-1-yl 2-(acetylthio)aceta	metab_6615	HMDB0247382	B(ii)	-	276.0179	1.6791	neg	M+FA-H	C8H9NO5S
neg_1400	O-Succinyl-L-homoserine	metab_6622	HMDB0255868	B(ii)	C01118	218.0669	1.6923	neg	M-H	C8H13NO6
neg_1401	4-Hydroxy-8-methoxy-2H-furo[2,3-h]-1-l	metab_6623	HMDB0032659	B(ii)	-	268.9875	1.6969	neg	M+K-2H	C12H8O5
neg_1413	Isoxanthopterin	metab_6636	HMDB0000704	B(i)	C03975	178.0366	1.7431	neg	M-H	C6H5N5O2
neg_1434	5-(2'-Carboxyethyl)-4,6-Dihydroxypicolinat	metab_6658	HMDB0006794	B(ii)	C05655	272.0404	1.824	neg	M+FA-H	C9H9NO6
neg_1440	6-Hydroxy-1H-indole-3-acetamide	metab_6664	HMDB0031173	B(ii)	-	235.0725	1.8356	neg	M+FA-H	C10H10N2O2
neg_1446	Tiglic acid	metab_6670	LMFA01020030;HMDB0001470	B(i)	C08279	99.0444	1.8436	neg	M-H	C5H8O2
neg_1447	Adipic Acid	metab_6671	LMFA01170048;HMDB0000448	B(i)	C06104	145.0501	1.8436	neg	M-H	C6H10O4
neg_1458	Naphthionic acid	metab_6683	HMDB0255449	B(ii)	-	668.0782	1.8522	neg	3M-H	C10H9NO3S
neg_1471	1,3,5-Triazine-2,4(1H,3H)-dione	metab_6697	HMDB0244186	B(ii)	-	112.0146	1.9613	neg	M-H	C3H3N3O2
neg_1476	4-Hydroxy-6-methyl-3-(1H-pyrazol-3-yl)-	metab_6702	HMDB0246447	B(ii)	-	213.0263	1.9613	neg	M+Na-2H	C9H8N2O3
neg_1477	Gamma-Glutaminyl-4-hydroxybenzene	metab_6703	HMDB0029451	B(ii)	-	219.0773	1.9613	neg	M-H2O-H	C11H14N2O4
neg_1478	(S,S)-Nt-Histidinylalanine	metab_6704	HMDB0040248	B(ii)	-	223.0833	1.9613	neg	M-H2O-H	C9H14N4O4
neg_1498	6-[3-(2-carboxyeth-1-en-1-yl)phenoxy]-3	metab_6726	HMDB0127983	B(ii)	-	361.0561	1.9693	neg	M+Na-2H	C15H16O9
neg_15	Glutamylhistidine	metab_6728	HMDB0028821	B(ii)	-	283.105	0.5753	neg	M-H, M+FA-H,	C11H16N4O5
neg_1528	Trioxsalen	metab_6760	HMDB0015575	B(ii)	C09314	263.0501	2.0093	neg	M+Cl	C14H12O3
neg_1530	Furylacryloylalanyllysine	metab_6763	HMDB0247373	B(ii)	-	372.1306	2.018	neg	M+Cl	C16H23N3O5
neg_154	S-Nitrosoglutathione	metab_6773	HMDB0257412;HMDB0004645	B(ii)	-	317.0551	0.5906	neg	M-H2O-H, 2M	C10H16N4O7S
neg_1542	Glycyl-L-Tyrosine	metab_6776	HMDB0028853	B(i)	-	237.0881	2.0542	neg	M-H	C11H14N2O4
neg_1546	L-Phenylalanine	metab_6780	HMDB0250791;PW_C000104;HMDB0000159;MJDBA	B(ii)	C00079	210.0769	2.0779	neg	M+FA-H	C9H11NO2
neg_155	D-Sorbitol	metab_6784	HMDB0000247;MJDBOTE0001050	B(i)	C00794	181.0712	0.5986	neg	M-H, M+FA-H	C6H14O6
neg_1551	Gentianine	metab_6786	HMDB0303030	B(ii)	C06525	220.0615	2.1104	neg	M+FA-H	C10H9NO2
neg_1556	Dehydrotremetone	metab_6791	HMDB0250959	B(ii)	C08741	235.0547	2.1467	neg	M+Cl	C13H12O2
neg_1577	4-Fluorobenzoic acid	metab_6812	HMDB0246425	B(ii)	C02371	279.0449	2.227	neg	2M-H	C7H5FO2
neg_1578	Sec-o-Glucosylhamaudol	metab_6813	HMDB0258199	B(ii)	C17484	437.1449	2.227	neg	M-H	C21H26O10
neg_1586	Sabiporide	metab_6822	HMDB0257436	B(ii)	-	453.1504	2.2819	neg	M+FA-H	C18H19F3N6O2
neg_1589	Blepharin	metab_6825	HMDB0029344	B(ii)	-	326.0887	2.3041	neg	M-H	C14H17NO8
neg_1601	Histidylvaline	metab_6839	HMDB0028898	B(ii)	-	299.1363	2.3457	neg	M+FA-H	C11H18N4O3
neg_161	Gardenin B	metab_6848	LMPK12111451;HMDB0302154	B(ii)	C15109	379.083	0.6223	neg	M-H, M+Na-2	C19H18O7
neg_163	Lactulose	metab_6870	HMDB0000740;MJDBOTE0001392	B(ii)	C07064	341.1091	0.6303	neg	M-H, 2M-H	C12H22O11
neg_1652	Imidazoleacetic acid ribotide	metab_6895	HMDB0006032	B(ii)	C04437	337.045	2.4409	neg	M-H	C10H15N2O9P
neg_1657	Iodinated glycerol	metab_6899	HMDB0253512	B(ii)	-	302.9718	2.4489	neg	M+FA-H	C6H11IO3
neg_1659	3-Hydroxychlorpropamide	metab_6901	HMDB0014025	B(ii)	-	629.0555	2.4489	neg	2M+FA-H	C10H13ClN2O4S
neg_170	Quinic Acid	metab_6943	HMDB0042004;HMDB0003072;MJDBOTE0000415	B(i)	C00296	191.0558	0.6541	neg	M-H, 2M+Hac-	C7H12O6
neg_1713	Nalidixic Acid	metab_6958	HMDB0014917	B(ii)	C05079	277.0833	2.5593	neg	M+FA-H	C12H12N2O3
neg_173	Lichenin	metab_6976	HMDB0302349	B(ii)	C00478	383.1199	0.6621	neg	M+Cl, 2M+Hac	C6H10O5
neg_1743	1,7-Dimethylguanosine	metab_6991	HMDB0001961	B(ii)	-	356.1217	2.6063	neg	M+FA-H	C12H17N5O5
neg_1777	Dihydrolipoate	metab_7021	PW_C007757;HMDB0012210	B(ii)	C02147	461.1174	2.6619	neg	2M+FA-H	C8H16O2S2
neg_1787	5-Hydroxypentanoic acid	metab_7032	HMDB0061927	B(ii)	C02804	235.1187	2.6774	neg	2M-H	C5H10O3
neg_1789	4-(2,6-Dioxo-1-propyl-3,7-dihydropurin-8	metab_7034	HMDB0256891	B(ii)	-	395.0655	2.6774	neg	M+FA-H	C14H14N4O5S
neg_1791	Fructosylvaline	metab_7037	HMDB0252498	B(ii)	-	260.1142	2.6854	neg	M-H2O-H	C11H21NO7
neg_1792	N-Formyl-L-aspartate	metab_7038	HMDB0060495	B(ii)	C01044	321.0556	2.6854	neg	2M-H	C5H7NO5
neg_1797	(1E)-1-Phenyltriaz-1-ene	metab_7042	HMDB0242314	B(ii)	-	166.0618	2.6934	neg	M+FA-H	C6H7N3
neg_1799	Porphobilinogen	metab_7044	HMDB0000245;PW_C000166	B(ii)	C00931	225.0879	2.6934	neg	M-H	C10H14N2O4
neg_18	Maltotetraose	metab_7045	HMDB0001296;MJDBOTE0000277	B(i)	C02052	701.1937	0.6541	neg	M+Cl, M+FA-H	C24H42O21
neg_180	3-Keto-b-D-galactose	metab_7046	HMDB0001385	B(ii)	C05394	177.0399	0.6858	neg	M-H2O-H, M-I	C6H10O6
neg_1800	Histidylproline	metab_7047	HMDB0304781;HMDB0028893	B(ii)	-	297.1207	2.6934	neg	M+FA-H	C11H16N4O3
neg_1804	Gamma-L-Glutamyl-S-(2-carboxy-1-prop	metab_7050	HMDB0029394	B(ii)	-	392.1139	2.6934	neg	M-H	C14H23N3O8S
neg_1808	Arbutin	metab_7054	HMDB0029943;MJDBOTE0001645	B(ii)	C06186	317.0895	2.7013	neg	M+FA-H	C12H16O7
neg_1809	Citalopram aldehyde	metab_7055	PW_C040544;HMDB0060462	B(ii)	C16612	340.0976	2.7013	neg	M+FA-H	C18H14FNO2
neg_1817	Hydroxyprolyl-Lysine	metab_7064	HMDB0028868	B(ii)	-	258.146	2.7173	neg	M-H	C11H21N3O4
neg_1824	Antiarrhythmic peptide	metab_7072	HMDB0248473	B(ii)	-	505.1841	2.7233	neg	M+Cl	C19H30N6O8
neg_1827	4-Hydroxy-5-phenyltetrahydro-1,3-oxazin	metab_7075	PW_C040532;HMDB0060389	B(ii)	C16595	238.0721	2.7299	neg	M+FA-H	C10H11NO3
neg_1829	N(2)-phenylacetyl-L-glutaminate	metab_7076	HMDB0062645	B(ii)	C05597	309.1098	2.7299	neg	M+FA-H	C13H16N2O4
neg_183	(2R,3S)-3-Hydroxy-1,1-dimethylpyrrolidin-	metab_7077	HMDB0242121	B(ii)	-	158.0817	0.6938	neg	M-H2O-H, M-I	C7H13NO3
neg_1830	Benzo[d]isoxazol-3-ol	metab_7078	HMDB0249006	B(ii)	-	404.0853	2.7299	neg	3M-H	C7H5NO2
neg_1833	Tetraazacyclododecanetetraacetic acid	metab_7081	HMDB0257787	B(ii)	-	385.1733	2.7374	neg	M-H2O-H	C16H28N4O8
neg_184	2-Keto-glutaramic acid	metab_7088	HMDB0001552	B(ii)	C00940	144.0296	0.6858	neg	M-H2O-H, M-I	C5H7NO4
neg_1847	Threonylhydroxyproline	metab_7094	HMDB0029062	B(ii)	-	213.0878	2.7655	neg	M-H2O-H	C9H16N2O5
neg_1852	Vinclozolin M2	metab_7099	HMDB0259812	B(ii)	C14207	304.0135	2.7705	neg	M+FA-H	C11H11Cl2NO2
neg_1854	N-(4-(2,5-Dihydro-5-oxo-3-furanyl)phenv	metab_7101	HMDB0253559	B(ii)	-	502.1336	2.7746	neg	M+FA-H	C23H23NO9
neg_1856	Pantothenic Acid	metab_7103	HMDB0250782;HMDB0000210;PW_C000136;MJDBOT	B(i)	C00864	218.1033	2.7825	neg	M-H	C9H17NO5
neg_1857	Cis-Zeatin O-glucoside	metab_7104	HMDB0012203	B(ii)	C15546	380.1571	2.7825	neg	M-H	C16H23N5O6
neg_1858	Minocycline	metab_7105	HMDB0015152;PW_C009450;LMPK07000002	B(ii)	C07225	494.1335	2.7825	neg	M+K-2H	C23H27N3O7
neg_1859	Butyric Acid	metab_7106	HMDB0000039;LMFA01010004;PW_C000028	B(i)	C00246	87.0443	2.7895	neg	M-H	C4H8O2
neg_1867	5-Hydroxytryptophol sulfate	metab_7115	HMDB0304913	B(ii)	-	301.0263	2.8035	neg	M+FA-H	C10H10NO5S-
neg_1868	Adenine	metab_7116	PW_C000024;HMDB0000034;MJDBOTE0000759	B(i)	C00147	134.0466	2.8105	neg	M-H	C5H5N5
neg_1870	Succinyladenosine	metab_7119	HMDB0000912	B(ii)	-	382.1011	2.8105	neg	M-H	C14H17N5O8
neg_1876	Dethiobiotin	metab_7125	HMDB0003581	B(ii)	C01909	259.1301	2.8235	neg	M+FA-H	C10H18N2O3
neg_1879	1-Acetyl-2-phenylhydrazine	metab_7128	HMDB0243811	B(ii)	-	149.0714	2.8291	neg	M-H	C8H10N2O
neg_1887	N-[(1-Chloro-4-hydroxyisoquinolin-3-YL)c	metab_7137	HMDB0252244	B(ii)	-	301.0003	2.846	neg	M+Na-2H	C12H9ClN2O4
neg_1888	Sweroside	metab_7138	HMDB0258643;MJDBOTE0001224	B(ii)	C17071	395.0757	2.846	neg	M+K-2H	C16H22O9
neg_1892	3-Amino-2,3-dihydrobenzoic acid	metab_7143	HMDB0251519	B(ii)	C12110	184.0611	2.862	neg	M+FA-H	C7H9NO2
neg_1896	Pirprofen	metab_7147	HMDB0256614	B(ii)	-	286.0393	2.87	neg	M+Cl	C13H14ClNO2
neg_19	Glu-Gln	metab_7151	HMDB0028817	B(i)	-	274.1047	0.6382	neg	M-H, M+Cl, M-	C10H17N3O6
neg_1905	(3S,5R,6R,7E)-3,5,6-Trihydroxy-7-megastig	metab_7157	HMDB0038736	B(ii)	-	529.3005	2.8816	neg	2M+FA-H	C13H22O4
neg_1906	Salsolinol	metab_7158	HMDB0005199;HMDB0042012	B(ii)	C09642	224.0927	2.8895	neg	M+FA-H	C10H13NO2
neg_1923	Casuarine 6-alpha-D-glucoside	metab_7176	HMDB0031999	B(ii)	-	388.1233	2.918	neg	M+Na-2H	C14H25NO10
neg_1929	5-(5-(2,6-Dichloro-4-(4,5-Dihydro-2-Oxa:	metab_7182	HMDB0259896	B(ii)	C06493	419.0302	2.9245	neg	M+K-2H	C18H20Cl2N2O3
neg_1950	Glutaminylphenylalanine	metab_7204	HMDB0028804	B(ii)	-	292.1305	2.9806	neg	M-H	C14H19N3O4
neg_1954	4-hydroxy-3-nitrophenylacetate	metab_7208	HMDB0062403	B(ii)	-	178.0141	2.9886	neg	M-H2O-H	C8H7NO5
neg_1959	L-cis-Cyclo(aspartylphenylalanyl)	metab_7213	HMDB0031360	B(ii)	-	297.0652	2.9966	neg	M+Cl	C13H14N2O4
neg_1964	2-(2-Hydroxyethyl)phenyl hydrogen sulfate	metab_7218	HMDB0304908	B(ii)	-	217.0174	3.0046	neg	M-H	C8H10O5S
neg_1967	Noradrenochrome	metab_7221	HMDB0013030	B(ii)	-	494.125	3.0046	neg	3M-H	C8H7NO3
neg_1968	3-Hydroxybenzyl Alcohol	metab_7222	HMDB0038331;HMDB0059712	B(i)	C03351	123.0446	3.0125	neg	M-H	C7H8O2
neg_1975	Delpazolid	metab_7230	HMDB0250974	B(ii)	-	345.0766	3.0205	neg	M+K-2H	C14H17FN4O3
neg_1979	L-Aspartate-semialdehyde	metab_7234	HMDB0012249	B(ii)	C00441	279.085	3.0285	neg	2M+FA-H	C4H7NO3
neg_1996	Sparstolonin B	metab_7250	HMDB0258408	B(ii)	-	303.0082	3.0766	neg	M+Cl	C15H8O5
neg_2	Gamma-Glutamylvaline	metab_7254	HMDB0011172	B(ii)	-	245.1144	2.4329	neg	M+Na-2H, M+	C10H18N2O5
neg_2003	Benzoquinoneacetic acid	metab_7260	HMDB0002334	B(ii)	-	165.0188	3.0846	neg	M-H	C8H6O4
neg_2004	Icofungipen	metab_7261	HMDB0253367	B(ii)	-	186.0768	3.0846	neg	M+FA-H	C7H11NO2

ID	Fragmentation Score	Theoretical Fragmentation Score	Mass Error	CAS ID	RSD	ND1	ND2	ND3	ND4	ND5	ND6	PreD1	PreD2	PreD3	PreD4	PreD5	PreD6	D1	D2	D3	D4	D5	D6	CT1	CT2	CT3	CT4	CT5	CT6	RD1	RD2	RD3	RD4	RD5	RD6	QC01	QC02	QC03
neg_1006	0	32.1	0.85426863	-	0.010415544	2.982	2.952	2.952	3.271	2.951	3.032	5.207	5.248	5.278	5.26	5.286	5.22	5.358	5.365	5.395	5.376	5.386	5.366	5.454	5.427	5.442	5.411	5.429	5.404	5.827	5.844	5.801	5.816	5.815	5.822	5.331	5.323	5.324
neg_1013	0	35.1	-9.6456056	107-97-1	0.003323848	5.799	5.773	5.773	5.754	5.763	5.766	5.36	5.398	5.403	5.42	5.414	5.42	5.503	5.527	5.529	5.549	5.553	5.563	5.524	5.5	5.505	5.527	5.505	5.512	5.185	5.181	5.17	5.154	5.161	5.139	5.643	5.645	5.646
neg_1015	0	35.2	-2.0614032	72432-03-2	0.005396489	5.158	5.168	5.134	5.157	5.14	5.212	3.642	3.984	3.634	3.934	3.922	4.229	4.063	3.787	3.683	3.937	3.686	3.742	3.954	3.672	3.967	3.673	3.67	3.672	5.621	5.619	5.619	5.592	5.605	5.585	5.2	5.196	5.197
neg_1018	79.5	0	1.81356156	4484-88-2	0.014972769	3.42	3.906	3.714	4.141	3.906	3.386	6.105	6.124	6.15	6.151	6.169	6.161	6.485	6.533	6.532	6.515	6.528	6.518	6.455	6.474	6.471	6.473	6.47	6.456	5.673	5.584	5.533	5.654	5.614	5.64	6.015	6.015	6.026
neg_1025	0	52.4	9.74376954	35597-43-4	0.011399558	4.624	4.569	4.61	4.657	4.63	4.709	5.742	5.828	5.82	5.833	5.845	5.82	5.814	5.837	5.845	5.841	5.849	5.779	5.83	5.813	5.838	5.849	5.799	5.84	5.368	5.378	5.333	5.332	5.282	5.366	5.528	5.537	5.528
neg_1028	86.5	0	-3.3597724	99-16-1	0.005498016	6.346	6.323	6.322	6.318	6.303	6.312	4.893	4.839	4.833	4.905	4.906	4.918	5.168	5.173	5.206	5.187	5.181	5.222	5.372	5.379	5.352	5.339	5.359	5.406	5.524	5.523	5.493	5.489	5.509	5.489	6.028	6.031	6.032
neg_1031	35.8	0	-4.7963781	7306-96-9	0.017584558	5.37	5.333	5.329	5.322	5.331	5.324	4.362	4.303	4.363	4.097	4.495	4.166	4.484	4.487	4.522	4.538	4.606	4.485	4.445	4.398	4.48	4.561	4.459	4.465	5.091	5.095	5.083	5.04	5.057	5.059	5.095	5.082	5.096
neg_1034	0	61	-4.8625737	107-36-8	0.00618818	3.181	3.151	3.151	3.158	3.15	3.146	3.064	3.111	3.123	3.122	3.13	3.136	3.65	3.87	3.869	3.866	3.265	3.879	3.445	3.747	3.646	3.971	3.937	3.697	5.958	5.956	5.962	5.965	5.965	5.969	5.015	5.009	5.011
neg_1043	0	64.5	-8.2655393	75-75-2	0.028027607	3.9	3.87	3.87	3.877	3.869	3.866	4.158	4.106	4.16	4.195	4.191	4.11	4.039	3.996	3.987	4.209	4.023	4.1	4.318	3.97	4.267	4.295	4.238	4.153	4.921	4.94	4.938	4.914	4.929	4.937	4.081	4.059	4.06
neg_1052	0	32.1	1.09999948	-	0.000702984	5.528	5.521	5.492	5.488	5.478	5.515	6.074	6.088	6.099	6.111	6.115	6.122	6.067	6.102	6.106	6.092	6.109	6.092	6.115	6.121	6.092	6.096	6.098	6.1	5.55	5.581	5.531	5.566	5.552	5.577	5.801	5.801	5.801
neg_1056	93.6	0	-6.6905638	473-81-4	0.008314233	6.14	6.114	6.126	6.119	6.105	6.112	5.061	5.053	5.094	5.089	5.094	5.09	5.031	5.044	5.057	5.047	5.037	5.052	5.216	5.239	5.237	5.239	5.219	5.24	4.635	4.634	4.612	4.649	4.633	4.665	5.775	5.775	5.781
neg_1058	82.3	0	0.94274103	84-52-6	0.023198496	6.009	5.976	5.946	5.937	5.931	5.99	4.485	4.455	4.543	4.524	4.418	4.532	4.562	4.687	4.521	4.656	4.683	4.743	4.717	4.801	4.772	4.728	4.529	4.799	5.552	5.567	5.508	5.493	5.506	5.509	5.447	5.465	5.449
neg_1059	0	35.7	-7.8005355	2212-67-1	0.022340445	4.953	4.876	4.929	5.012	4.983	4.909	5.386	5.409	5.448	5.499	5.414	5.435	5.373	5.339	5.322	5.361	5.332	5.355	5.526	5.48	5.538	5.547	5.511	5.483	5.459	5.536	5.466	5.412	5.459	5.468	5.28	5.296	5.278
neg_1070	70.9	0	-4.2035186	9000-69-5	0.009143559	5.964	5.939	5.943	5.94	5.93	5.936	5.527	5.569	5.557	5.562	5.566	5.577	5.851	5.879	5.881	5.884	5.881	5.881	5.849	5.856	5.855	5.855	5.854	5.857	5.536	5.521	5.525	5.539	5.555	5.529	5.846	5.845	5.853
neg_1075	0	38.7	1.97225272	-	0.077476363	5.503	5.481	5.454	5.406	5.39	5.457	4.342	4.389	4.401	4.4	4.408	4.414	4.505	4.547	4.54	4.54	4.543	4.544	4.527	4.529	4.526	4.529	4.527	4.529	4.431	4.429	4.426	4.426	4.424	4.419	4.513	4.451	4.508
neg_1079	0	30.1	0.79004229	645-14-7	0.02197597	3.64	3.61	3.609	3.768	3.608	3.841	5.104	5.057	5.067	5.077	5.056	5.059	5.389	5.416	5.423	5.436	5.43	5.447	5.257	5.26	5.237	5.287	5.242	5.245	4.831	4.765	4.799	4.761	4.785	4.795	4.537	4.521	4.538
neg_1081	0	33.9	2.26563543	20762-30-5	0.008291343	5.822	5.809	5.816	5.815	5.774	5.83	6.233	6.232	6.236	6.268	6.261	6.26	6.485	6.512	6.485	6.504	6.514	6.508	6.483	6.488	6.469	6.454	6.487	6.476	5.998	5.987	5.933	5.959	5.991	5.992	6.232	6.24	6.236
neg_1084	0	36.2	2.28199241	-	0.119981852	4.385	4.355	4.355	4.363	4.354	4.351	6.331	6.326	6.344	6.387	6.364	6.368	6.165	6.212	6.194	6.225	6.233	6.199	6.234	6.24	6.226	6.185	6.196	6.266	4.358	4.973	4.955	4.939	4.957	5.03	5.542	5.542	5.448
neg_1087	0	48.3	4.7033362	-	0.00980537	3.771	3.741	4.162	4.192	4.153	3.834	6.505	6.51	6.525	6.523	6.528	6.55	6.299	6.33	6.319	6.325	6.326	6.33	6.437	6.449	6.433	6.446	6.427	6.443	6.744	6.738	6.748	6.763	6.759	6.769	6.41	6.41	6.417
neg_1105	0	86.6	2.1139996	13015-87-7	0.008242845	5.958	5.945	5.953	5.949	5.919	6.003	6.019	6.029	6.033	6.053	6.049	6.05	6.193	6.225	6.194	6.209	6.21	6.215	6.028	6.002	6.022	6.006	6.028	5.992	5.689	5.7	5.674	5.694	5.672	5.652	6.021	6.027	6.021
neg_1106	0	36	0.17053994	76400-25-4	0.05996955	5.364	5.357	5.33	5.35	5.367	5.337	4.549	4.752	4.723	4.63	4.676	4.645	4.479	4.335	4.46	4.345	4.349	4.508	4.641	4.596	4.614	4.608	4.59	4.575	5.182	5.224	5.212	5.218	5.211	5.215	5.148	5.117	5.169
neg_1108	0	34.2	-8.8107869	7228-78-6	0.006881798	4.436	5.068	5.01	5.101	5.152	5.147	5.897	5.947	5.917	5.971	5.91	5.92	5.556	5.514	5.49	5.56	5.525	5.517	5.651	5.617	5.582	5.617	5.646	5.591	5.528	5.482	5.477	5.471	5.533	5.487	5.617	5.616	5.622
neg_1110	0	55.8	-5.3592306	-	0.016031806	5.142	5.112	5.112	5.12	5.111	5.108	6.355	6.351	6.354	6.414	6.357	6.365	6.469	6.515	6.449	6.533	6.477	6.486	6.463	6.436	6.456	6.471	6.445	6.432	5.275	5.264	5.503	5.23	5.108	5.301	6.098	6.109	6.111
neg_1115	0	80.8	-8.6865344	-	0.007776866	6.519	6.489	6.489	6.496	6.488	6.485	6.769	6.781	6.797	6.801	6.8	6.799	6.623	6.646	6.652	6.652	6.658	6.656	6.683	6.681	6.673	6.679	6.673	6.673	6.914	6.911	6.904	6.897	6.905	6.916	6.512	6.517	6.518
neg_1118	0	64.4	7.9298001	131862-10-7	0.043445834	4.932	4.902	4.902	4.909	4.901	4.898	5.437	5.511	5.486	5.48	5.525	5.495	5.275	5.258	5.34	5.342	5.343	5.375	5.405	5.391	5.361	5.393	5.426	5.425	5.699	5.713	5.698	5.7	5.72	5.759	4.957	4.924	4.957
neg_1122	0	38	9.15518879	-	0.054305396	5.185	5.191	5.275	5.308	5.26	5.223	3.521	3.569	3.581	3.579	3.587	3.593	3.685	3.726	3.719	3.72	3.722	3.723	3.706	3.708	3.706	3.709	3.706	3.708	3.611	3.608	3.605	3.605	3.603	3.598	4.534	4.575	4.577
neg_1127	0	46.7	-1.2669851	5875-41-2	0.012753973	4.662	4.643	4.644	4.595	4.686	4.615	5.247	5.232	5.268	5.275	5.27	5.259	5.426	5.46	5.467	5.453	5.476	5.49	5.317	5.273	5.334	5.332	5.308	5.276	5.034	4.995	4.953	5.014	5.034	4.986	5.097	5.088	5.098
neg_1129	91.3	0	-5.2509578	660-88-8	0.006175016	5.868	5.861	5.862	5.858	5.853	5.858	5.963	5.989	5.998	6.003	6.011	6	6.116	6.156	6.165	6.159	6.168	6.161	6.09	6.087	6.089	6.087	6.078	6.083	5.75	5.741	5.715	5.733	5.725	5.711	5.97	5.975	5.97
neg_1130	0	40.4	-5.4081453	108-30-5	0.001019148	6.854	6.877	6.885	6.882	6.88	6.885	7.274	7.301	7.305	7.309	7.306	7.309	7.124	7.157	7.17	7.165	7.167	7.165	7.145	7.14	7.121	7.118	7.135	7.132	5.76	5.769	5.724	5.822	5.819	5.792	7.004	7.004	7.003
neg_1136	0	37.3	6.61638992	-	0.010837116	6.172	6.177	6.163	6.15	6.154	6.152	4.051	4.182	3.572	3.815	4.37	3.754	3.695	3.38	3.681	0.9067	2.056	1.945	3.999	3.608	3.987	1.781	3.617	3.524	5.184	5.202	5.13	5.212	5.178	5.155	5.712	5.711	5.703
neg_1149	0	61.3	1.30052314	-	0.014359133	5.761	5.731	5.731	5.738	5.73	5.727	6.545	6.57	6.587	6.582	6.582	6.599	6.639	6.672	6.679	6.667	6.668	6.662	6.593	6.573	6.552	6.566	6.592	6.582	5.734	5.731	5.728	5.728	5.726	5.721	5.766	5.765	5.755
neg_1163	0	35.4	1.81465613	-	0.149040406	4.973	4.872	4.857	4.869	4.831	4.936	2.539	2.586	2.598	2.597	2.604	2.611	2.702	2.743	2.737	2.737	2.739	2.741	2.724	2.725	2.723	2.726	2.724	2.725	2.628	2.626	3.077	3.25	2.621	2.616	4.191	4.191	4.299
neg_1169	0	40.5	-2.8986279	-	0.007366229	4.904	4.923	4.926	4.92	4.942	4.959	4.488	4.564	4.638	4.587	4.588	4.582	4.157	4.265	4.365	4.15	4.454	4.247	4.385	4.375	4.443	4.401	4.418	4.413	4.041	4.038	4.035	4.035	4.034	4.028	4.693	4.688	4.693
neg_1174	0	39.8	-9.9109601	89202-77-7	0.019302769	4.791	4.761	4.761	4.768	4.76	4.757	5.457	5.527	5.538	5.548	5.575	5.561	6.09	6.118	6.113	6.138	6.093	6.138	6.109	6.082	6.095	6.116	6.119	6.118	4.899	4.768	4.793	4.758	4.844	4.78	5.515	5.515	5.529
neg_1176	0	43.2	-0.9358724	21381-33-9	0.033553459	3.692	3.848	3.789	3.647	3.565	3.694	3.752	3.675	3.866	3.971	3.936	3.783	4.323	4.438	4.367	4.391	4.306	4.348	4.678	4.506	4.553	4.571	4.491	4.487	4.586	4.364	4.624	4.544	4.508	4.594	5.272	5.297	5.272
neg_1199	0	63.8	4.21466432	9037-22-3	0.005641285	3.658	3.678	4.421	3.636	4.086	4.077	6.035	6.067	6.02	6.033	6.076	6.123	6.203	6.282	6.287	6.252	6.22	6.265	6.272	6.215	6.249	6.221	6.249	6.229	6.186	6.207	6.201	6.195	6.191	6.175	6.012	6.016	6.017
neg_1204	96.1	0	-3.9790084	73-40-5	0.025210062	5.399	5.401	5.366	5.351	5.364	5.383	4.441	4.489	4.501	4.499	4.507	4.513	4.605	4.647	4.64	4.64	4.642	4.644	4.627	4.628	4.626	4.629	4.627	4.628	4.531	4.529	4.525	4.525	4.524	4.518	4.551	4.57	4.571
neg_1206	0	66.7	-2.6336976	59-92-7	0.00554294	0.6196	0.597	0.5969	0.6024	0.5962	0.5937	5.488	5.493	5.465	5.453	5.42	5.478	5.314	5.335	5.381	5.415	5.421	5.374	5.227	5.309	5.122	5.139	5.242	5.214	6.308	6.31	6.293	6.294	6.31	6.307	5.568	5.566	5.571
neg_1208	80.6	0	2.94881448	2616-64-0	0.024832831	5	4.926	4.859	5.051	4.727	4.819	4.332	4.878	4.439	4.559	4.669	4.536	4.007	4.455	4.041	4.198	4.044	4.199	4.795	5.088	4.757	4.767	4.894	4.853	5.332	5.316	5.158	5.339	5.318	5.173	5.083	5.082	5.063
neg_1211	60.7	0	-4.6008199	99-94-5	0.053425079	2.521	2.491	2.491	2.498	2.565	2.486	3.146	3.046	2.463	2.462	3.077	3.01	2.693	2.609	2.602	2.628	2.604	2.606	2.589	3.194	2.588	3.022	2.589	2.591	4.931	4.918	4.932	4.892	4.927	4.971	3.625	3.623	3.583
neg_1212	0	40.8	7.03470587	-	0.000772636	5.613	5.926	5.986	5.995	6.032	6.027	6.117	6.162	6.18	6.178	6.207	6.182	5.658	5.699	5.717	5.688	5.7	5.697	5.91	5.915	5.904	5.905	5.919	5.925	5.681	5.7	5.708	5.68	5.698	5.694	6.018	6.019	6.019
neg_1218	0	36.7	-4.0026049	-	0.017240928	4.738	4.71	4.622	4.711	4.637	4.661	2.881	2.928	2.94	2.938	3.202	2.952	3.044	3.085	3.079	3.079	3.081	3.083	3.066	3.067	3.065	3.068	3.066	3.067	2.97	2.968	2.991	3.003	3.395	3.32	4.054	4.066	4.067
neg_1219	84.1	0	1.44382011	84-21-9	0.007700075	7.343	7.322	7.302	7.305	7.304	7.315	6.873	6.908	6.916	6.924	6.948	6.931	6.972	7.006	7.022	7.001	7.009	7.021	6.75	6.746	6.75	6.747	6.745	6.757	6.548	6.551	6.56	6.553	6.56	6.549	7.042	7.041	7.047
neg_1223	0	41.9	-7.8423686	-	0.01300821	1.868	3.339	4.132	4.087	4.188	4.331	6.34	6.367	6.386	6.386	6.395	6.389	6.521	6.55	6.574	6.56	6.569	6.567	6.7	6.689	6.686	6.686	6.695	6.687	6.634	6.632	6.622	6.626	6.623	6.621	5.633	5.622	5.623
neg_1226	71.1	0	-3.1175209	997-55-7	0.011507915	5.732	5.704	5.693	5.696	5.692	5.694	3.901	3.753	3.902	3.799	3.71	3.889	3.58	3.296	3.361	3.698	3.661	3.452	3.882	3.908	3.843	3.812	3.773	3.712	5.571	5.561	5.552	5.554	5.561	5.557	5.325	5.316	5.325
neg_1227	0	63.7	1.77557095	-	0.013372509	2.143	4.345	4.75	4.74	4.923	4.98	5.787	5.828	5.832	5.839	5.879	5.87	5.833	5.851	5.898	5.882	5.869	5.87	5.821	5.837	5.84	5.808	5.89	5.875	6.173	6.171	6.156	6.15	6.166	6.168	5.129	5.118	5.119
neg_1231	91.1	0	-5.4383043	88-14-2	0.004577878	5.349	5.305	5.313	5.313	5.307	5.307	6.662	6.68	6.699	6.702	6.7	6.703	6.686	6.708	6.725	6.723	6.725	6.727	6.574	6.573	6.577	6.566	6.568	6.574	5.167	5.153	5.154	5.168	5.156	5.133	6.453	6.453	6.456
neg_1235	0	38.8	-5.2455013	-	0.017019975	6.107	6.092	6.069	6.054	6.088	6.094	5.362	5.41	5.422	5.42	5.428	5.434	5.526	5.567	5.56	5.561	5.563	5.564	5.547	5.549	5.547	5.55	5.547	5.549	5.452	5.449	5.446	5.446	5.444	5.439	5.485	5.471	5.473
neg_1238	0	37	0.77560352	-	0.017254164	3.445	3.415	3.415	3.423	3.414	3.411	5.969	5.98	6.036	6.032	6.024	6.048	6.062	6.075	6.051	6.093	6.092	6.093	6.048	6.015	6.037	6.04	6.015	6.036	3.418	3.415	3.412	3.412	3.621	3.405	6.034	6.046	6.047
neg_1241	82.1	0	2.67074044	15839-70-0	0.033985697	5.425	5.281	5.368	5.441	5.461	5.419	5.388	5.463	5.464	5.448	5.545	5.467	4.582	4.632	4.997	4.59	4.621	4.831	5.341	5.325	5.299	5.245	5.292	5.109	5.601	5.592	5.667	5.617	5.618	5.678	5.417	5.417	5.442
neg_1244	0	56.3	-1.2605011	-	0.030723926	4.729	4.699	4.699	4.707	4.698	4.695	6.654	6.683	6.696	6.696	6.703	6.7	6.688	6.705	6.717	6.71	6.732	6.716	6.672	6.676	6.673	6.656	6.673	6.678	4.86	4.796	4.754	4.83	4.695	4.723	5.497	5.497	5.474
neg_1245	0	33	-1.1790737	13254-53-0	0.028989974	4.815	4.728	4.745	4.748	4.765	4.718	2.807	2.855	2.867	2.865	2.873	3.021	2.971	3.012	3.257	3.006	3.124	3.009	2.992	2.994	2.992	2.995	2.992	2.994	3.38	3.273	2.962	2.891	2.889	3.142	3.654	3.632	3.654
neg_1247	54.6	0	-1.4311277	551-85-9;415	0.010385926	5.967	5.918	5.916	5.921	5.91	5.909	5.223	5.28	5.27	5.274	5.3	5.275	5.203	5.206	5.189	5.216	5.222	5.215	5.227	5.216	5.14	5.195	5.226	5.231	5.677	5.676	5.662	5.672	5.662	5.661	5.696	5.696	5.704
neg_1257	0	34.9	9.19917304	-	0.051323518	4.892	4.862	4.862	4.87	4.861	4.858	4.843	4.899	4.876	4.946	4.95	4.914	5.097	5.138	5.169	5.189	5.204	5.171	5.098	5.041	5.128	5.037	5.029	5.031	4.865	4.863	4.859	4.859	4.858	4.852	4.886	4.885	4.923
neg_1258	0	39	-7.7839134	-	0.058263884	3.377	3.371	3.899	4.021	4.021	4.134	4.691	4.682	4.669	4.619	4.456	4.797	4.889	4.792	4.815	4.842	4.776	4.816	4.754	4.799	4.846	4.786	4.845	4.854	5.192	5.18	5.106	5.141	5.171	5.159	3.37	3.414	3.371
neg_1259	0	38.3	1.31240855	-	0.114767799	3.624	3.594	3.594	3.601	3.593	3.59	3.507	3.554	3.566	3.565	3.573	3.579	4.202	3.999	3.705	3.792	3.821	3.764	3.692	3.722	3.691	4.085	3.851	4.042	5.02	5.023	4.951	5.033	5.019	5.06	3.699	3.616	3.711
neg_1263	96.5	0	1.39905839	653-63-4	0.002111999	6.05	6.017	6.006	6	6.005	6.018	4.43	4.439	4.427	4.098	4.177	4.37	4.713	4.809	4.697	4.701	4.699	4.616	4.721	4.717	4.632	4.61	4.635	4.694	5.253	5.261	5.269	5.245	5.242	5.272	5.516	5.516	5.515
neg_1269	0	51.3	1.38953642	3650-73-5	0.005806493	5.642	5.482	5.463	5.482	5.464	5.473	5.262	5.309	5.321	5.32	5.328	5.334	5.426	5.467	5.46	5.46	5.463	5.464	5.447	5.449	5.446	5.45	5.447	5.449	5.351	5.349	5.346	5.346	5.344	5.339	5.372	5.375	5.377
neg_1275	0	34.6	-7.8475507	-	0.03265171	3.738	3.194	3.486	3.701	3.193	3.523	4.698	4.716	4.689	4.709	4.68	4.747	5.245	5.292	5.334	5.3	5.298	5.311	5.051	5.062	5.046	5.037	4.986	5.091	3.197	3.194	3.191	3.191	3.189	3.184	4.756	4.755	4.779
neg_1276	0	37.6	-3.1333729	4429-4-3	0.005460681	5.354	5.321	5.339	5.323	5.311	5.314	4.288	4.277	4.307	4.205	4.326	4.3	4.346	4.377	4.426	4.459	4.408	4.473	4.402	4.411	4.436	4.407	4.463	4.474	4.852	4.813	4.809	4.811	4.845	4.836	4.973	4.977	4.978
neg_1278	0	38.2	1.02057816	29605-99-0	0.014785047	5.722	5.686	5.666	5.679	5.662	5.691	4.858	4.873	4.894	4.918	4.918	4.912	5.14	5.176	5.199	5.175	5.156	5.147	4.997	4.983	4.95	4.93	4.975	4.968	4.733	4.772	4.738	4.669	4.752	4.713	5.204	5.215	5.216
neg_1282	72.4	0	-4.7232181	533-67-5	0.010998094	4.778	4.73	4.719	4.717	4.695	4.724	5.176	5.239	5.231	5.236	5.247	5.227	5.375	5.405	5.423	5.425	5.42	5.435	5.44	5.441	5.439	5.446	5.433	5.442	5	4.98	4.968	4.943	4.966	4.991	5.218	5.225	5.226
neg_1293	0	35.7	0.56608038	65-82-7	0.005780143	3.701	3.451	3.732	3.69	3.948	3.625	5.228	5.302	5.286	5.29	5.297	5.277	5.729	5.774	5.782	5.767	5.782	5.771	5.713	5.706	5.712	5.706	5.703	5.71	5.425	5.398	5.433	5.434	5.429	5.41	5.459	5.464	5.46
neg_1296	0	33.2	1.72556812	29790-46-3	0.015115896	5.213	5.108	5.054	5.043	4.99	4.978	5.421	5.451	5.476	5.508	5.481	5.509	5.85	5.871	5.918	5.926	5.931	5.925	5.872	5.87	5.862	5.876	5.87	5.882	5.65	5.63	5.609	5.61	5.614	5.616	5.729	5.716	5.724
neg_1298	0	38.1	1.29563088	3868-31-3	0.042485473	4.914	4.949	4.794	4.942	4.998	4.928	4.051	3.985	3.893	3.789	4.022	3.828	3.268	3.176	3.179	3.243	3.273	3.173	3.625	3.792	3.202	3.375	3.827	3.853	4.055	3.834	4.172	4.058	4.158	4.13	4.565	4.532	4.564
neg_13	65.1	0	-3.4786974	5959-95-5;56	0.008467837	6.759	6.731	6.719	6.726	6.723	6.723	7.039	7.063	7.076	7.084	7.093	7.083	7.164	7.192	7.2	7.191	7.201	7.195	6.968	6.95	6.96	6.964	6.959	6.958	6.485	6.484	6.469	6.477	6.48	6.484	6.915	6.914	6.921
neg_1306	0	51.4	5.78779249	940-69-2	0.002498193	4.934	4.917	4.952	4.985	4.968	4.954	3.274	3.191	3.642	3.495	3.431	3.92	3.966	4.31	4.052	4.266	4.226	4.101	4.19	4.394	3.998	4.124	3.955	4.227	5.74	5.691	5.731	5.737	5.743	5.741	4.739	4.738	4.737
neg_1309	0	32.3	-0.2396181	16869-42-4	0.006893694	3.691	3.803	3.874	3.732	3.783	3.638	6.132	6.16	6.155	6.147	6.152	6.179	5.937	5.971	6.003	5.982	5.984	5.956	5.855	5.883	5.813	5.805	5.861	5.84	5.739	5.728	5.723	5.733	5.727	5.73	5.854	5.86	5.855
neg_1315	0	69.4	-0.718957	4602-81-7	0.009846119	3.886	3.857	3.842	3.837	3.651	3.711	5.517	5.563	5.549	5.53	5.543	5.557	5.31	5.346	5.363	5.376	5.375	5.36	5.262	5.293	5.225	5.25	5.28	5.254	5.187	5.175	5.181	5.174	5.196	5.168	5.249	5.248	5.241
neg_1319	0	44.7	2.24920461	4548-99-6	0.001447935	6.81	6.786	6.79	6.792	6.781	6.791	5.812	5.834	5.851	5.847	5.851	5.85	5.775	5.796	5.815	5.809	5.814	5.811	5.538	5.542	5.502	5.507	5.518	5.548	4.389	4.267	4.363	4.528	4.328	4.243	6.277	6.277	6.276
neg_1325	0	32.4	-4.6108992	-	0.002226805	2.78	2.75	2.75	2.757	2.749	3.352	5.001	4.978	5.058	5.029	5.074	5.014	4.779	4.835	4.843	4.775	4.753	4.823	4.825	4.787	4.815	4.868	4.785	4.865	5.551	5.548	5.519	5.527	5.539	5.535	5.158	5.158	5.16
neg_1330	0	33.5	-4.3445036	4226-22-6	0.010971791	5.157	5.174	5.11	5.15	5.105	5.137	4.958	4.987	4.989	4.969	4.985	5.002	5.051	5.024	5.092	5.085	5.075	5.062	5.008	4.99	4.981	4.995	5	5.011	4.989	4.98	4.992	4.995	4.992	5.006	5.01	5.01	5.018
neg_1331	0	49.2	5.86967786	-	0.010846583	6.13	6.137	6.148	6.157	6.154	6.159	5.571	5.583	5.598	5.592	5.585	5.626	5.554	5.568	5.561	5.567	5.565	5.555	5.51	5.512	5.508	5.514	5.489	5.542	5.718	5.733	5.717	5.737	5.728	5.721	5.735	5.742	5.743
neg_1332	0	61.7	-3.6942523	-	0.013756587	5.561	5.545	5.546	5.594	5.571	5.572	5.091	5.161	5.09	5.14	5.116	5.109	4.752	4.754	4.813	4.794	4.845	4.845	4.965	5.012	4.975	4.933	4.967	4.989	3.355	3.597	3.737	3.703	3.643	3.934	5.161	5.16	5.15
neg_1338	0	63.6	1.10354179	876-19-7	0.184443656	5.507	5.442	5.408	5.403	5.37	5.379	1.143	1.187	1.199	1.197	1.205	1.21	1.297	1.336	1.33	1.482	1.332	2.722	1.318	1.319	1.317	1.32	1.318	1.319	3.656	3.893	3.739	3.865	3.98	3.965	3.603	3.754	3.748
neg_134	0	52.5	-5.8211265	121748-12-7	0.041113973	3.977	4.209	4.388	4.177	4.324	4.435	5.252	5.304	5.315	5.336	5.325	5.314	5.33	5.358	5.4	5.397	5.39	5.411	5.426	5.447	5.429	5.442	5.423	5.414	5.618	5.675	5.702	5.608	5.608	5.631	4.989	5.02	4.989
neg_1348	0	52.6	2.85617943	79-57-2	0.025412653	5.758	5.708	5.655	5.684	5.673	5.68	4.844	4.909	4.979	4.91	4.952	4.902	4.77	4.764	4.723	4.75	4.812	4.852	4.417	4.485	4.659	4.502	4.521	4.568	5.138	5.114	5.086	5.132	5.181	5.146	5.23	5.23	5.211
neg_1358	0	41.7	4.39511074	89786-04-9	0.015849872	5.993	5.965	5.944	5.963	5.941	5.95	5.758	5.781	5.778	5.772	5.8	5.806	5.846	5.857	5.871	5.869	5.901	5.879	5.792	5.782	5.756	5.785	5.778	5.775	5.753	5.734	5.728	5.739	5.748	5.736	5.826	5.815	5.827
neg_1360	0	65.5	6.16891434	-	0.019308927	5.056	5.029	5.082	5.084	5.024	5.103	5.799	5.804	5.82	5.812	5.836	5.826	5.859	5.88	5.877	5.871	5.891	5.872	5.701	5.685	5.674	5.686	5.676	5.71	6.351	6.347	6.342	6.335	6.366	6.342	5.959	5.964	5.947
neg_1377	0	42.8	2.27886753	138-52-3	0.00598511	0.1526	0.144	0.1439	0.146	0.1437	0.1427	5.376	5.389	5.431	5.416	5.413	5.411	6.293	6.299	6.318	6.309	6.325	6.323	6.262	6.268	6.249	6.263	6.267	6.264	5.635	5.626	5.604	5.608	5.641	5.624	5.684	5.679	5.679
neg_1378	0	52.9	-4.4540673	1341-23-7	0.007080667	4.959	4.928	4.915	4.916	4.892	4.931	5.069	5.078	5.082	5.114	5.116	5.11	5.158	5.148	5.162	5.172	5.18	5.18	5.19	5.189	5.187	5.211	5.207	5.194	5.346	5.345	5.343	5.34	5.357	5.336	5.12	5.12	5.125
neg_1381	0	38.6	2.03691476	19237-53-7	0.020830008	2.679	2.649	2.649	2.657	2.648	2.645	5.725	5.732	5.762	5.761	5.727	5.741	5.349	5.359	5.361	5.382	5.397	5.381	5.428	5.449	5.385	5.405	5.419	5.42	4.244	4.118	4.161	4.381	4.41	4.29	4.873	4.857	4.857
neg_1387	0	38	-2.2692879	59-87-0	0.014041584	5.458	5.56	5.557	5.565	5.554	5.58	6.125	6.137	6.173	6.173	6.168	6.16	5.733	5.736	5.738	5.745	5.756	5.756	5.855	5.859	5.84	5.86	5.853	5.854	5.084	5.076	5.057	5.051	5.068	5.067	5.718	5.707	5.708
neg_1389	0	45.6	6.08309784	-	0.007776523	6.494	6.489	6.489	6.491	6.485	6.498	6.695	6.692	6.725	6.724	6.713	6.714	6.819	6.835	6.824	6.838	6.845	6.827	6.808	6.817	6.796	6.818	6.806	6.831	6.531	6.535	6.522	6.519	6.539	6.519	6.604	6.608	6.602
neg_1395	0	51.8	-1.8606947	-	0.018716576	4.837	4.708	4.577	4.614	4.682	4.65	4.766	4.748	4.814	4.643	4.773	4.738	4.847	4.887	4.819	4.981	4.988	4.8	4.907	4.746	4.869	4.928	4.902	4.727	4.597	4.677	4.597	4.612	4.637	4.555	4.765	4.751	4.752
neg_1400	0	48.6	-0.6451964	1492-23-5	0.014714673	4.166	4.08	3.91	3.934	3.883	3.997	4.914	4.908	4.956	4.942	4.992	4.955	5.285	5.311	5.314	5.361	5.338	5.318	5.254	5.248	5.225	5.294	5.321	5.256	4.742	4.801	4.754	4.778	4.799	4.741	4.991	4.979	4.98
neg_1401	0	57.9	7.21969772	196503-96-5	0.003723238	5.174	5.16	5.182	5.177	5.178	5.181	5.529	5.523	5.572	5.566	5.564	5.551	5.712	5.739	5.722	5.733	5.754	5.725	5.69	5.721	5.69	5.711	5.7	5.718	5.34	5.343	5.324	5.318	5.34	5.31	5.352	5.355	5.354
neg_1413	83.8	0	-2.251072	529-69-1	0.008942774	6.049	6.086	6.092	6.086	6.081	6.098	6.355	6.366	6.405	6.409	6.4	6.385	6.43	6.445	6.433	6.439	6.453	6.455	6.378	6.389	6.369	6.39	6.385	6.373	6.091	6.083	6.085	6.078	6.081	6.076	6.272	6.264	6.266
neg_1434	0	71.6	-3.4710686	-	0.003539454	4.996	4.965	4.965	4.931	4.888	4.984	2.506	2.553	2.565	2.564	2.572	2.578	2.669	2.711	2.704	2.704	2.707	2.708	2.691	2.693	2.69	2.693	2.691	2.693	2.596	2.593	2.59	2.59	2.942	3.121	4.778	4.777	4.775
neg_1440	0	44.8	0.1084985	192184-73-9	0.00614239	4.843	4.735	4.704	4.656	4.643	4.66	4.17	4.269	4.248	4.401	4.3	4.326	4.785	4.856	4.849	4.784	4.824	4.853	4.718	4.802	4.803	4.772	4.746	4.76	5.275	5.271	5.304	5.283	5.254	5.284	4.919	4.917	4.922
neg_1446	[illegible]	[illegible]	[illegible]	[illegible]	[illegible]	[illegible]	[illegible]	[illegible]	[illegible]	[illegible]	[illegible]	[illegible]	[illegible]	[illegible]	[illegible]	[illegible]	[illegible]	[illegible]	[illegible]	[illegible]	[illegible]	[illegible]	[illegible]	[illegible]	[illegible]	[illegible]	[illegible]	[illegible]	[illegible]	[illegible]	[illegible]	[illegible]	[illegible]	[illegible]	[illegible]	[illegible]	[illegible]	[illegible]
neg_1447	[illegible]	[illegible]	[illegible]	[illegible]	[illegible]	[illegible]	[illegible]	[illegible]	[illegible]	[illegible]	[illegible]	[illegible]	[illegible]	[illegible]	[illegible]	[illegible]	[illegible]	[illegible]	[illegible]	[illegible]	[illegible]	[illegible]	[illegible]	[illegible]	[illegible]	[illegible]	[illegible]	[illegible]	[illegible]	[illegible]	[illegible]	[illegible]	[illegible]	[illegible]	[illegible]	[illegible]	[illegible]	[illegible]
neg_1458	[illegible]	[illegible]	[illegible]	[illegible]	[illegible]	[illegible]	[illegible]	[illegible]	[illegible]	[illegible]	[illegible]	[illegible]	[illegible]	[illegible]	[illegible]	[illegible]	[illegible]	[illegible]	[illegible]	[illegible]	[illegible]	[illegible]	[illegible]	[illegible]	[illegible]	[illegible]	[illegible]	[illegible]	[illegible]	[illegible]	[illegible]	[illegible]	[illegible]	[illegible]	[illegible]	[illegible]	[illegible]	[illegible]
neg_1471	[illegible]	[illegible]	[illegible]	[illegible]	[illegible]	[illegible]	[illegible]	[illegible]	[illegible]	[illegible]	[illegible]	[illegible]	[illegible]	[illegible]	[illegible]	[illegible]	[illegible]	[illegible]	[illegible]	[illegible]	[illegible]	[illegible]	[illegible]	[illegible]	[illegible]	[illegible]	[illegible]	[illegible]	[illegible]	[illegible]	[illegible]	[illegible]	[illegible]	[illegible]	[illegible]	[illegible]	[illegible]	[illegible]
neg_1476	[illegible]	[illegible]	[illegible]	[illegible]	[illegible]	[illegible]	[illegible]	[illegible]	[illegible]	[illegible]	[illegible]	[illegible]	[illegible]	[illegible]	[illegible]	[illegible]	[illegible]	[illegible]	[illegible]	[illegible]	[illegible]	[illegible]	[illegible]	[illegible]	[illegible]	[illegible]	[illegible]	[illegible]	[illegible]	[illegible]	[illegible]	[illegible]	[illegible]	[illegible]	[illegible]	[illegible]	[illegible]	[illegible]
neg_1477	[illegible]	[illegible]	[illegible]	[illegible]	[illegible]	[illegible]	[illegible]	[illegible]	[illegible]	[illegible]	[illegible]	[illegible]	[illegible]	[illegible]	[illegible]	[illegible]	[illegible]	[illegible]	[illegible]	[illegible]	[illegible]	[illegible]	[illegible]	[illegible]	[illegible]	[illegible]	[illegible]	[illegible]	[illegible]	[illegible]	[illegible]	[illegible]	[illegible]	[illegible]	[illegible]	[illegible]	[illegible]	[illegible]
neg_1478	[illegible]	[illegible]	[illegible]	[illegible]	[illegible]	[illegible]	[illegible]	[illegible]	[illegible]	[illegible]	[illegible]	[illegible]	[illegible]	[illegible]	[illegible]	[illegible]	[illegible]	[illegible]	[illegible]	[illegible]	[illegible]	[illegible]	[illegible]	[illegible]	[illegible]	[illegible]	[illegible]	[illegible]	[illegible]	[illegible]	[illegible]	[illegible]	[illegible]	[illegible]	[illegible]	[illegible]	[illegible]	[illegible]
neg_1498	[illegible]	[illegible]	[illegible]	[illegible]	[illegible]	[illegible]	[illegible]	[illegible]	[illegible]	[illegible]	[illegible]	[illegible]	[illegible]	[illegible]	[illegible]	[illegible]	[illegible]	[illegible]	[illegible]	[illegible]	[illegible]	[illegible]	[illegible]	[illegible]	[illegible]	[illegible]	[illegible]	[illegible]	[illegible]	[illegible]	[illegible]	[illegible]	[illegible]	[illegible]	[illegible]	[illegible]	[illegible]	[illegible]
neg_15	[illegible]	[illegible]	[illegible]	[illegible]	[illegible]	[illegible]	[illegible]	[illegible]	[illegible]	[illegible]	[illegible]	[illegible]	[illegible]	[illegible]	[illegible]	[illegible]	[illegible]	[illegible]	[illegible]	[illegible]	[illegible]	[illegible]	[illegible]	[illegible]	[illegible]	[illegible]	[illegible]	[illegible]	[illegible]	[illegible]	[illegible]	[illegible]	[illegible]	[illegible]	[illegible]	[illegible]	[illegible]	[illegible]
neg_1528	[illegible]	[illegible]	[illegible]	[illegible]	[illegible]	[illegible]	[illegible]	[illegible]	[illegible]	[illegible]	[illegible]	[illegible]	[illegible]	[illegible]	[illegible]	[illegible]	[illegible]	[illegible]	[illegible]	[illegible]	[illegible]	[illegible]	[illegible]	[illegible]	[illegible]	[illegible]	[illegible]	[illegible]	[illegible]	[illegible]	[illegible]	[illegible]	[illegible]	[illegible]	[illegible]	[illegible]	[illegible]	[illegible]
neg_1530	[illegible]	[illegible]	[illegible]	[illegible]	[illegible]	[illegible]	[illegible]	[illegible]	[illegible]	[illegible]	[illegible]	[illegible]	[illegible]	[illegible]	[illegible]	[illegible]	[illegible]	[illegible]	[illegible]	[illegible]	[illegible]	[illegible]	[illegible]	[illegible]	[illegible]	[illegible]	[illegible]	[illegible]	[illegible]	[illegible]	[illegible]	[illegible]	[illegible]	[illegible]	[illegible]	[illegible]	[illegible]	[illegible]
neg_154	[illegible]	[illegible]	[illegible]	[illegible]	[illegible]	[illegible]	[illegible]	[illegible]	[illegible]	[illegible]	[illegible]	[illegible]	[illegible]	[illegible]	[illegible]	[illegible]	[illegible]	[illegible]	[illegible]	[illegible]	[illegible]	[illegible]	[illegible]	[illegible]	[illegible]	[illegible]	[illegible]	[illegible]	[illegible]	[illegible]	[illegible]	[illegible]	[illegible]	[illegible]	[illegible]	[illegible]	[illegible]	[illegible]
neg_1542	[illegible]	[illegible]	[illegible]	[illegible]	[illegible]	[illegible]	[illegible]	[illegible]	[illegible]	[illegible]	[illegible]	[illegible]	[illegible]	[illegible]	[illegible]	[illegible]	[illegible]	[illegible]	[illegible]	[illegible]	[illegible]	[illegible]	[illegible]	[illegible]	[illegible]	[illegible]	[illegible]	[illegible]	[illegible]	[illegible]	[illegible]	[illegible]	[illegible]	[illegible]	[illegible]	[illegible]	[illegible]	[illegible]
neg_1546	[illegible]	[illegible]	[illegible]	[illegible]	[illegible]	[illegible]	[illegible]	[illegible]	[illegible]	[illegible]	[illegible]	[illegible]	[illegible]	[illegible]	[illegible]	[illegible]	[illegible]	[illegible]	[illegible]	[illegible]	[illegible]	[illegible]	[illegible]	[illegible]	[illegible]	[illegible]	[illegible]	[illegible]	[illegible]	[illegible]	[illegible]	[illegible]	[illegible]	[illegible]	[illegible]	[illegible]	[illegible]	[illegible]
neg_155	[illegible]	[illegible]	[illegible]	[illegible]	[illegible]	[illegible]	[illegible]	[illegible]	[illegible]	[illegible]	[illegible]	[illegible]	[illegible]	[illegible]	[illegible]	[illegible]	[illegible]	[illegible]	[illegible]	[illegible]	[illegible]	[illegible]	[illegible]	[illegible]	[illegible]	[illegible]	[illegible]	[illegible]	[illegible]	[illegible]	[illegible]	[illegible]	[illegible]	[illegible]	[illegible]	[illegible]	[illegible]	[illegible]
neg_1551	[illegible]	[illegible]	[illegible]	[illegible]	[illegible]	[illegible]	[illegible]	[illegible]	[illegible]	[illegible]	[illegible]	[illegible]	[illegible]	[illegible]	[illegible]	[illegible]	[illegible]	[illegible]	[illegible]	[illegible]	[illegible]	[illegible]	[illegible]	[illegible]	[illegible]	[illegible]	[illegible]	[illegible]	[illegible]	[illegible]	[illegible]	[illegible]	[illegible]	[illegible]	[illegible]	[illegible]	[illegible]	[illegible]
neg_1556	[illegible]	[illegible]	[illegible]	[illegible]	[illegible]	[illegible]	[illegible]	[illegible]	[illegible]	[illegible]	[illegible]	[illegible]	[illegible]	[illegible]	[illegible]	[illegible]	[illegible]	[illegible]	[illegible]	[illegible]	[illegible]	[illegible]	[illegible]	[illegible]	[illegible]	[illegible]	[illegible]	[illegible]	[illegible]	[illegible]	[illegible]	[illegible]	[illegible]	[illegible]	[illegible]	[illegible]	[illegible]	[illegible]
neg_1577	[illegible]	[illegible]	[illegible]	[illegible]	[illegible]	[illegible]	[illegible]	[illegible]	[illegible]	[illegible]	[illegible]	[illegible]	[illegible]	[illegible]	[illegible]	[illegible]	[illegible]	[illegible]	[illegible]	[illegible]	[illegible]	[illegible]	[illegible]	[illegible]	[illegible]	[illegible]	[illegible]	[illegible]	[illegible]	[illegible]	[illegible]	[illegible]	[illegible]	[illegible]	[illegible]	[illegible]	[illegible]	[illegible]
neg_1578	[illegible]	[illegible]	[illegible]	[illegible]	[illegible]	[illegible]	[illegible]	[illegible]	[illegible]	[illegible]	[illegible]	[illegible]	[illegible]	[illegible]	[illegible]	[illegible]	[illegible]	[illegible]	[illegible]	[illegible]	[illegible]	[illegible]	[illegible]	[illegible]	[illegible]	[illegible]	[illegible]	[illegible]	[illegible]	[illegible]	[illegible]	[illegible]	[illegible]	[illegible]	[illegible]	[illegible]	[illegible]	[illegible]
neg_1586	[illegible]	[illegible]	[illegible]	[illegible]	[illegible]	[illegible]	[illegible]	[illegible]	[illegible]	[illegible]	[illegible]	[illegible]	[illegible]	[illegible]	[illegible]	[illegible]	[illegible]	[illegible]	[illegible]	[illegible]	[illegible]	[illegible]	[illegible]	[illegible]	[illegible]	[illegible]	[illegible]	[illegible]	[illegible]	[illegible]	[illegible]	[illegible]	[illegible]	[illegible]	[illegible]	[illegible]	[illegible]	[illegible]
neg_1589	[illegible]	[illegible]	[illegible]	[illegible]	[illegible]	[illegible]	[illegible]	[illegible]	[illegible]	[illegible]	[illegible]	[illegible]	[illegible]	[illegible]	[illegible]	[illegible]	[illegible]	[illegible]	[illegible]	[illegible]	[illegible]	[illegible]	[illegible]	[illegible]	[illegible]	[illegible]	[illegible]	[illegible]	[illegible]	[illegible]	[illegible]	[illegible]	[illegible]	[illegible]	[illegible]	[illegible]	[illegible]	[illegible]
neg_1601	[illegible]	[illegible]	[illegible]	[illegible]	[illegible]	[illegible]	[illegible]	[illegible]	[illegible]	[illegible]	[illegible]	[illegible]	[illegible]	[illegible]	[illegible]	[illegible]	[illegible]	[illegible]	[illegible]	[illegible]	[illegible]	[illegible]	[illegible]	[illegible]	[illegible]	[illegible]	[illegible]	[illegible]	[illegible]	[illegible]	[illegible]	[illegible]	[illegible]	[illegible]	[illegible]	[illegible]	[illegible]	[illegible]
neg_161	[illegible]	[illegible]	[illegible]	[illegible]	[illegible]	[illegible]	[illegible]	[illegible]	[illegible]	[illegible]	[illegible]	[illegible]	[illegible]	[illegible]	[illegible]	[illegible]	[illegible]	[illegible]	[illegible]	[illegible]	[illegible]	[illegible]	[illegible]	[illegible]	[illegible]	[illegible]	[illegible]	[illegible]	[illegible]	[illegible]	[illegible]	[illegible]	[illegible]	[illegible]	[illegible]	[illegible]	[illegible]	[illegible]
neg_163	[illegible]	[illegible]	[illegible]	[illegible]	[illegible]	[illegible]	[illegible]	[illegible]	[illegible]	[illegible]	[illegible]	[illegible]	[illegible]	[illegible]	[illegible]	[illegible]	[illegible]	[illegible]	[illegible]	[illegible]	[illegible]	[illegible]	[illegible]	[illegible]	[illegible]	[illegible]	[illegible]	[illegible]	[illegible]	[illegible]	[illegible]	[illegible]	[illegible]	[illegible]	[illegible]	[illegible]	[illegible]	[illegible]
neg_1652	[illegible]	[illegible]	[illegible]	[illegible]	[illegible]	[illegible]	[illegible]	[illegible]	[illegible]	[illegible]	[illegible]	[illegible]	[illegible]	[illegible]	[illegible]	[illegible]	[illegible]	[illegible]	[illegible]	[illegible]	[illegible]	[illegible]	[illegible]	[illegible]	[illegible]	[illegible]	[illegible]	[illegible]	[illegible]	[illegible]	[illegible]	[illegible]	[illegible]	[illegible]	[illegible]	[illegible]	[illegible]	[illegible]
neg_1657	[illegible]	[illegible]	[illegible]	[illegible]	[illegible]	[illegible]	[illegible]	[illegible]	[illegible]	[illegible]	[illegible]	[illegible]	[illegible]	[illegible]	[illegible]	[illegible]	[illegible]	[illegible]	[illegible]	[illegible]	[illegible]	[illegible]	[illegible]	[illegible]	[illegible]	[illegible]	[illegible]	[illegible]	[illegible]	[illegible]	[illegible]	[illegible]	[illegible]	[illegible]	[illegible]	[illegible]	[illegible]	[illegible]
neg_1659	[illegible]	[illegible]	[illegible]	[illegible]	[illegible]	[illegible]	[illegible]	[illegible]	[illegible]	[illegible]	[illegible]	[illegible]	[illegible]	[illegible]	[illegible]	[illegible]	[illegible]	[illegible]	[illegible]	[illegible]	[illegible]	[illegible]	[illegible]	[illegible]	[illegible]	[illegible]	[illegible]	[illegible]	[illegible]	[illegible]	[illegible]	[illegible]	[illegible]	[illegible]	[illegible]	[illegible]	[illegible]	[illegible]
neg_170	[illegible]	[illegible]	[illegible]	[illegible]	[illegible]	[illegible]	[illegible]	[illegible]	[illegible]	[illegible]	[illegible]	[illegible]	[illegible]	[illegible]	[illegible]	[illegible]	[illegible]	[illegible]	[illegible]	[illegible]	[illegible]	[illegible]	[illegible]	[illegible]	[illegible]	[illegible]	[illegible]	[illegible]	[illegible]	[illegible]	[illegible]	[illegible]	[illegible]	[illegible]	[illegible]	[illegible]	[illegible]	[illegible]
neg_1713	[illegible]	[illegible]	[illegible]	[illegible]	[illegible]	[illegible]	[illegible]	[illegible]	[illegible]	[illegible]	[illegible]	[illegible]	[illegible]	[illegible]	[illegible]	[illegible]	[illegible]	[illegible]	[illegible]	[illegible]	[illegible]	[illegible]	[illegible]	[illegible]	[illegible]	[illegible]	[illegible]	[illegible]	[illegible]	[illegible]	[illegible]	[illegible]	[illegible]	[illegible]	[illegible]	[illegible]	[illegible]	[illegible]
neg_173	[illegible]	[illegible]	[illegible]	[illegible]	[illegible]	[illegible]	[illegible]	[illegible]	[illegible]	[illegible]	[illegible]	[illegible]	[illegible]	[illegible]	[illegible]	[illegible]	[illegible]	[illegible]	[illegible]	[illegible]	[illegible]	[illegible]	[illegible]	[illegible]	[illegible]	[illegible]	[illegible]	[illegible]	[illegible]	[illegible]	[illegible]	[illegible]	[illegible]	[illegible]	[illegible]	[illegible]	[illegible]	[illegible]
neg_1743	[illegible]	[illegible]	[illegible]	[illegible]	[illegible]	[illegible]	[illegible]	[illegible]	[illegible]	[illegible]	[illegible]	[illegible]	[illegible]	[illegible]	[illegible]	[illegible]	[illegible]	[illegible]	[illegible]	[illegible]	[illegible]	[illegible]	[illegible]	[illegible]	[illegible]	[illegible]	[illegible]	[illegible]	[illegible]	[illegible]	[illegible]	[illegible]	[illegible]	[illegible]	[illegible]	[illegible]	[illegible]	[illegible]
neg_1777	[illegible]	[illegible]	[illegible]	[illegible]	[illegible]	[illegible]	[illegible]	[illegible]	[illegible]	[illegible]	[illegible]	[illegible]	[illegible]	[illegible]	[illegible]	[illegible]	[illegible]	[illegible]	[illegible]	[illegible]	[illegible]	[illegible]	[illegible]	[illegible]	[illegible]	[illegible]	[illegible]	[illegible]	[illegible]	[illegible]	[illegible]	[illegible]	[illegible]	[illegible]	[illegible]	[illegible]	[illegible]	[illegible]
neg_1787	[illegible]	[illegible]	[illegible]	[illegible]	[illegible]	[illegible]	[illegible]	[illegible]	[illegible]	[illegible]	[illegible]	[illegible]	[illegible]	[illegible]	[illegible]	[illegible]	[illegible]	[illegible]	[illegible]	[illegible]	[illegible]	[illegible]	[illegible]	[illegible]	[illegible]	[illegible]	[illegible]	[illegible]	[illegible]	[illegible]	[illegible]	[illegible]	[illegible]	[illegible]	[illegible]	[illegible]	[illegible]	[illegible]
neg_1789	[illegible]	[illegible]	[illegible]	[illegible]	[illegible]	[illegible]	[illegible]	[illegible]	[illegible]	[illegible]	[illegible]	[illegible]	[illegible]	[illegible]	[illegible]	[illegible]	[illegible]	[illegible]	[illegible]	[illegible]	[illegible]	[illegible]	[illegible]	[illegible]	[illegible]	[illegible]	[illegible]	[illegible]	[illegible]	[illegible]	[illegible]	[illegible]	[illegible]	[illegible]	[illegible]	[illegible]	[illegible]	[illegible]
neg_1791	[illegible]	[illegible]	[illegible]	[illegible]	[illegible]	[illegible]	[illegible]	[illegible]	[illegible]	[illegible]	[illegible]	[illegible]	[illegible]	[illegible]	[illegible]	[illegible]	[illegible]	[illegible]	[illegible]	[illegible]	[illegible]	[illegible]	[illegible]	[illegible]	[illegible]	[illegible]	[illegible]	[illegible]	[illegible]	[illegible]	[illegible]	[illegible]	[illegible]	[illegible]	[illegible]	[illegible]	[illegible]	[illegible]
neg_1792	[illegible]	[illegible]	[illegible]	[illegible]	[illegible]	[illegible]	[illegible]	[illegible]	[illegible]	[illegible]	[illegible]	[illegible]	[illegible]	[illegible]	[illegible]	[illegible]	[illegible]	[illegible]	[illegible]	[illegible]	[illegible]	[illegible]	[illegible]	[illegible]	[illegible]	[illegible]	[illegible]	[illegible]	[illegible]	[illegible]	[illegible]	[illegible]	[illegible]	[illegible]	[illegible]	[illegible]	[illegible]	[illegible]
neg_1797	[illegible]	[illegible]	[illegible]	[illegible]	[illegible]	[illegible]	[illegible]	[illegible]	[illegible]	[illegible]	[illegible]	[illegible]	[illegible]	[illegible]	[illegible]	[illegible]	[illegible]	[illegible]	[illegible]	[illegible]	[illegible]	[illegible]	[illegible]	[illegible]	[illegible]	[illegible]	[illegible]	[illegible]	[illegible]	[illegible]	[illegible]	[illegible]	[illegible]	[illegible]	[illegible]	[illegible]	[illegible]	[illegible]
neg_1799	[illegible]	[illegible]	[illegible]	[illegible]	[illegible]	[illegible]	[illegible]	[illegible]	[illegible]	[illegible]	[illegible]	[illegible]	[illegible]	[illegible]	[illegible]	[illegible]	[illegible]	[illegible]	[illegible]	[illegible]	[illegible]	[illegible]	[illegible]	[illegible]	[illegible]	[illegible]	[illegible]	[illegible]	[illegible]	[illegible]	[illegible]	[illegible]	[illegible]	[illegible]	[illegible]	[illegible]	[illegible]	[illegible]
neg_18	[illegible]	[illegible]	[illegible]	[illegible]	[illegible]	[illegible]	[illegible]	[illegible]	[illegible]	[illegible]	[illegible]	[illegible]	[illegible]	[illegible]	[illegible]	[illegible]	[illegible]	[illegible]	[illegible]	[illegible]	[illegible]	[illegible]	[illegible]	[illegible]	[illegible]	[illegible]	[illegible]	[illegible]	[illegible]	[illegible]	[illegible]	[illegible]	[illegible]	[illegible]	[illegible]	[illegible]	[illegible]	[illegible]
neg_180	[illegible]	[illegible]	[illegible]	[illegible]	[illegible]	[illegible]	[illegible]	[illegible]	[illegible]	[illegible]	[illegible]	[illegible]	[illegible]	[illegible]	[illegible]	[illegible]	[illegible]	[illegible]	[illegible]	[illegible]	[illegible]	[illegible]	[illegible]	[illegible]	[illegible]	[illegible]	[illegible]	[illegible]	[illegible]	[illegible]	[illegible]	[illegible]	[illegible]	[illegible]	[illegible]	[illegible]	[illegible]	[illegible]
neg_1800	[illegible]	[illegible]	[illegible]	[illegible]	[illegible]	[illegible]	[illegible]	[illegible]	[illegible]	[illegible]	[illegible]	[illegible]	[illegible]	[illegible]	[illegible]	[illegible]	[illegible]	[illegible]	[illegible]	[illegible]	[illegible]	[illegible]	[illegible]	[illegible]	[illegible]	[illegible]	[illegible]	[illegible]	[illegible]	[illegible]	[illegible]	[illegible]	[illegible]	[illegible]	[illegible]	[illegible]	[illegible]	[illegible]
neg_1804	[illegible]	[illegible]	[illegible]	[illegible]	[illegible]	[illegible]	[illegible]	[illegible]	[illegible]	[illegible]	[illegible]	[illegible]	[illegible]	[illegible]	[illegible]	[illegible]	[illegible]	[illegible]	[illegible]	[illegible]	[illegible]	[illegible]	[illegible]	[illegible]	[illegible]	[illegible]	[illegible]	[illegible]	[illegible]	[illegible]	[illegible]	[illegible]	[illegible]	[illegible]	[illegible]	[illegible]	[illegible]	[illegible]
neg_1808	[illegible]	[illegible]	[illegible]	[illegible]	[illegible]	[illegible]	[illegible]	[illegible]	[illegible]	[illegible]	[illegible]	[illegible]	[illegible]	[illegible]	[illegible]	[illegible]	[illegible]	[illegible]	[illegible]	[illegible]	[illegible]	[illegible]	[illegible]	[illegible]	[illegible]	[illegible]	[illegible]	[illegible]	[illegible]	[illegible]	[illegible]	[illegible]	[illegible]	[illegible]	[illegible]	[illegible]	[illegible]	[illegible]
neg_1809	[illegible]	[illegible]	[illegible]	[illegible]	[illegible]	[illegible]	[illegible]	[illegible]	[illegible]	[illegible]	[illegible]	[illegible]	[illegible]	[illegible]	[illegible]	[illegible]	[illegible]	[illegible]	[illegible]	[illegible]	[illegible]	[illegible]	[illegible]	[illegible]	[illegible]	[illegible]	[illegible]	[illegible]	[illegible]	[illegible]	[illegible]	[illegible]	[illegible]	[illegible]	[illegible]	[illegible]	[illegible]	[illegible]
neg_1817	[illegible]	[illegible]	[illegible]	[illegible]	[illegible]	[illegible]	[illegible]	[illegible]	[illegible]	[illegible]	[illegible]	[illegible]	[illegible]	[illegible]	[illegible]	[illegible]	[illegible]	[illegible]	[illegible]	[illegible]	[illegible]	[illegible]	[illegible]	[illegible]	[illegible]	[illegible]	[illegible]	[illegible]	[illegible]	[illegible]	[illegible]	[illegible]	[illegible]	[illegible]	[illegible]	[illegible]	[illegible]	[illegible]
neg_1824	[illegible]	[illegible]	[illegible]	[illegible]	[illegible]	[illegible]	[illegible]	[illegible]	[illegible]	[illegible]	[illegible]	[illegible]	[illegible]	[illegible]	[illegible]	[illegible]	[illegible]	[illegible]	[illegible]	[illegible]	[illegible]	[illegible]	[illegible]	[illegible]	[illegible]	[illegible]	[illegible]	[illegible]	[illegible]	[illegible]	[illegible]	[illegible]	[illegible]	[illegible]	[illegible]	[illegible]	[illegible]	[illegible]
neg_1827	[illegible]	[illegible]	[illegible]	[illegible]	[illegible]	[illegible]	[illegible]	[illegible]	[illegible]	[illegible]	[illegible]	[illegible]	[illegible]	[illegible]	[illegible]	[illegible]	[illegible]	[illegible]	[illegible]	[illegible]	[illegible]	[illegible]	[illegible]	[illegible]	[illegible]	[illegible]	[illegible]	[illegible]	[illegible]	[illegible]	[illegible]	[illegible]	[illegible]	[illegible]	[illegible]	[illegible]	[illegible]	[illegible]
neg_1829	[illegible]	[illegible]	[illegible]	[illegible]	[illegible]	[illegible]	[illegible]	[illegible]	[illegible]	[illegible]	[illegible]	[illegible]	[illegible]	[illegible]	[illegible]	[illegible]	[illegible]	[illegible]	[illegible]	[illegible]	[illegible]	[illegible]	[illegible]	[illegible]	[illegible]	[illegible]	[illegible]	[illegible]	[illegible]	[illegible]	[illegible]	[illegible]	[illegible]	[illegible]	[illegible]	[illegible]	[illegible]	[illegible]
neg_183	[illegible]	[illegible]	[illegible]	[illegible]	[illegible]	[illegible]	[illegible]	[illegible]	[illegible]	[illegible]	[illegible]	[illegible]	[illegible]	[illegible]	[illegible]	[illegible]	[illegible]	[illegible]	[illegible]	[illegible]	[illegible]	[illegible]	[illegible]	[illegible]	[illegible]	[illegible]	[illegible]	[illegible]	[illegible]	[illegible]	[illegible]	[illegible]	[illegible]	[illegible]	[illegible]	[illegible]	[illegible]	[illegible]
neg_1830	[illegible]	[illegible]	[illegible]	[illegible]	[illegible]	[illegible]	[illegible]	[illegible]	[illegible]	[illegible]	[illegible]	[illegible]	[illegible]	[illegible]	[illegible]	[illegible]	[illegible]	[illegible]	[illegible]	[illegible]	[illegible]	[illegible]	[illegible]	[illegible]	[illegible]	[illegible]	[illegible]	[illegible]	[illegible]	[illegible]	[illegible]	[illegible]	[illegible]	[illegible]	[illegible]	[illegible]	[illegible]	[illegible]
neg_1833	[illegible]	[illegible]	[illegible]	[illegible]	[illegible]	[illegible]	[illegible]	[illegible]	[illegible]	[illegible]	[illegible]	[illegible]	[illegible]	[illegible]	[illegible]	[illegible]	[illegible]	[illegible]	[illegible]	[illegible]	[illegible]	[illegible]	[illegible]	[illegible]	[illegible]	[illegible]	[illegible]	[illegible]	[illegible]	[illegible]	[illegible]	[illegible]	[illegible]	[illegible]	[illegible]	[illegible]	[illegible]	[illegible]
neg_184	[illegible]	[illegible]	[illegible]	[illegible]	[illegible]	[illegible]	[illegible]	[illegible]	[illegible]	[illegible]	[illegible]	[illegible]	[illegible]	[illegible]	[illegible]	[illegible]	[illegible]	[illegible]	[illegible]	[illegible]	[illegible]	[illegible]	[illegible]	[illegible]	[illegible]	[illegible]	[illegible]	[illegible]	[illegible]	[illegible]	[illegible]	[illegible]	[illegible]	[illegible]	[illegible]	[illegible]	[illegible]	[illegible]
neg_1847	[illegible]	[illegible]	[illegible]	[illegible]	[illegible]	[illegible]	[illegible]	[illegible]	[illegible]	[illegible]	[illegible]	[illegible]	[illegible]	[illegible]	[illegible]	[illegible]	[illegible]	[illegible]	[illegible]	[illegible]	[illegible]	[illegible]	[illegible]	[illegible]	[illegible]	[illegible]	[illegible]	[illegible]	[illegible]	[illegible]	[illegible]	[illegible]	[illegible]	[illegible]	[illegible]	[illegible]	[illegible]	[illegible]
neg_1852	[illegible]	[illegible]	[illegible]	[illegible]	[illegible]	[illegible]	[illegible]	[illegible]	[illegible]	[illegible]	[illegible]	[illegible]	[illegible]	[illegible]	[illegible]	[illegible]	[illegible]	[illegible]	[illegible]	[illegible]	[illegible]	[illegible]	[illegible]	[illegible]	[illegible]	[illegible]	[illegible]	[illegible]	[illegible]	[illegible]	[illegible]	[illegible]	[illegible]	[illegible]	[illegible]	[illegible]	[illegible]	[illegible]
neg_1854	[illegible]	[illegible]	[illegible]	[illegible]	[illegible]	[illegible]	[illegible]	[illegible]	[illegible]	[illegible]	[illegible]	[illegible]	[illegible]	[illegible]	[illegible]	[illegible]	[illegible]	[illegible]	[illegible]	[illegible]	[illegible]	[illegible]	[illegible]	[illegible]	[illegible]	[illegible]	[illegible]	[illegible]	[illegible]	[illegible]	[illegible]	[illegible]	[illegible]	[illegible]	[illegible]	[illegible]	[illegible]	[illegible]
neg_1856	[illegible]	[illegible]	[illegible]	[illegible]	[illegible]	[illegible]	[illegible]	[illegible]	[illegible]	[illegible]	[illegible]	[illegible]	[illegible]	[illegible]	[illegible]	[illegible]	[illegible]	[illegible]	[illegible]	[illegible]	[illegible]	[illegible]	[illegible]	[illegible]	[illegible]	[illegible]	[illegible]	[illegible]	[illegible]	[illegible]	[illegible]	[illegible]	[illegible]	[illegible]	[illegible]	[illegible]	[illegible]	[illegible]
neg_1857	[illegible]	[illegible]	[illegible]	[illegible]	[illegible]	[illegible]	[illegible]	[illegible]	[illegible]	[illegible]	[illegible]	[illegible]	[illegible]	[illegible]	[illegible]	[illegible]	[illegible]	[illegible]	[illegible]	[illegible]	[illegible]	[illegible]	[illegible]	[illegible]	[illegible]	[illegible]	[illegible]	[illegible]	[illegible]	[illegible]	[illegible]	[illegible]	[illegible]	[illegible]	[illegible]	[illegible]	[illegible]	[illegible]
neg_1858	[illegible]	[illegible]	[illegible]	[illegible]	[illegible]	[illegible]	[illegible]	[illegible]	[illegible]	[illegible]	[illegible]	[illegible]	[illegible]	[illegible]	[illegible]	[illegible]	[illegible]	[illegible]	[illegible]	[illegible]	[illegible]	[illegible]	[illegible]	[illegible]	[illegible]	[illegible]	[illegible]	[illegible]	[illegible]	[illegible]	[illegible]	[illegible]	[illegible]	[illegible]	[illegible]	[illegible]	[illegible]	[illegible]
neg_1859	[illegible]	[illegible]	[illegible]	[illegible]	[illegible]	[illegible]	[illegible]	[illegible]	[illegible]	[illegible]	[illegible]	[illegible]	[illegible]	[illegible]	[illegible]	[illegible]	[illegible]	[illegible]	[illegible]	[illegible]	[illegible]	[illegible]	[illegible]	[illegible]	[illegible]	[illegible]	[illegible]	[illegible]	[illegible]	[illegible]	[illegible]	[illegible]	[illegible]	[illegible]	[illegible]	[illegible]	[illegible]	[illegible]
neg_1867	[illegible]	[illegible]	[illegible]	[illegible]	[illegible]	[illegible]	[illegible]	[illegible]	[illegible]	[illegible]	[illegible]	[illegible]	[illegible]	[illegible]	[illegible]	[illegible]	[illegible]	[illegible]	[illegible]	[illegible]	[illegible]	[illegible]	[illegible]	[illegible]	[illegible]	[illegible]	[illegible]	[illegible]	[illegible]	[illegible]	[illegible]	[illegible]	[illegible]	[illegible]	[illegible]	[illegible]	[illegible]	[illegible]
neg_1868	[illegible]	[illegible]	[illegible]	[illegible]	[illegible]	[illegible]	[illegible]	[illegible]	[illegible]	[illegible]	[illegible]	[illegible]	[illegible]	[illegible]	[illegible]	[illegible]	[illegible]	[illegible]	[illegible]	[illegible]	[illegible]	[illegible]	[illegible]	[illegible]	[illegible]	[illegible]	[illegible]	[illegible]	[illegible]	[illegible]	[illegible]	[illegible]	[illegible]	[illegible]	[illegible]	[illegible]	[illegible]	[illegible]
neg_1870	[illegible]	[illegible]	[illegible]	[illegible]	[illegible]	[illegible]	[illegible]	[illegible]	[illegible]	[illegible]	[illegible]	[illegible]	[illegible]	[illegible]	[illegible]	[illegible]	[illegible]	[illegible]	[illegible]	[illegible]	[illegible]	[illegible]	[illegible]	[illegible]	[illegible]	[illegible]	[illegible]	[illegible]	[illegible]	[illegible]	[illegible]	[illegible]	[illegible]	[illegible]	[illegible]	[illegible]	[illegible]	[illegible]
neg_1876	[illegible]	[illegible]	[illegible]	[illegible]	[illegible]	[illegible]	[illegible]	[illegible]	[illegible]	[illegible]	[illegible]	[illegible]	[illegible]	[illegible]	[illegible]	[illegible]	[illegible]	[illegible]	[illegible]	[illegible]	[illegible]	[illegible]	[illegible]	[illegible]	[illegible]	[illegible]	[illegible]	[illegible]	[illegible]	[illegible]	[illegible]	[illegible]	[illegible]	[illegible]	[illegible]	[illegible]	[illegible]	[illegible]
neg_1879	[illegible]	[illegible]	[illegible]	[illegible]	[illegible]	[illegible]	[illegible]	[illegible]	[illegible]	[illegible]	[illegible]	[illegible]	[illegible]	[illegible]	[illegible]	[illegible]	[illegible]	[illegible]	[illegible]	[illegible]	[illegible]	[illegible]	[illegible]	[illegible]	[illegible]	[illegible]	[illegible]	[illegible]	[illegible]	[illegible]	[illegible]	[illegible]	[illegible]	[illegible]	[illegible]	[illegible]	[illegible]	[illegible]
neg_1887	[illegible]	[illegible]	[illegible]	[illegible]	[illegible]	[illegible]	[illegible]	[illegible]	[illegible]	[illegible]	[illegible]	[illegible]	[illegible]	[illegible]	[illegible]	[illegible]	[illegible]	[illegible]	[illegible]	[illegible]	[illegible]	[illegible]	[illegible]	[illegible]	[illegible]	[illegible]	[illegible]	[illegible]	[illegible]	[illegible]	[illegible]	[illegible]	[illegible]	[illegible]	[illegible]	[illegible]	[illegible]	[illegible]
neg_1888	[illegible]	[illegible]	[illegible]	[illegible]	[illegible]	[illegible]	[illegible]	[illegible]	[illegible]	[illegible]	[illegible]	[illegible]	[illegible]	[illegible]	[illegible]	[illegible]	[illegible]	[illegible]	[illegible]	[illegible]	[illegible]	[illegible]	[illegible]	[illegible]	[illegible]	[illegible]	[illegible]	[illegible]	[illegible]	[illegible]	[illegible]	[illegible]	[illegible]	[illegible]	[illegible]	[illegible]	[illegible]	[illegible]
neg_1892	[illegible]	[illegible]	[illegible]	[illegible]	[illegible]	[illegible]	[illegible]	[illegible]	[illegible]	[illegible]	[illegible]	[illegible]	[illegible]	[illegible]	[illegible]	[illegible]	[illegible]	[illegible]	[illegible]	[illegible]	[illegible]	[illegible]	[illegible]	[illegible]	[illegible]	[illegible]	[illegible]	[illegible]	[illegible]	[illegible]	[illegible]	[illegible]	[illegible]	[illegible]	[illegible]	[illegible]	[illegible]	[illegible]
neg_1896	[illegible]	[illegible]	[illegible]	[illegible]	[illegible]	[illegible]	[illegible]	[illegible]	[illegible]	[illegible]	[illegible]	[illegible]	[illegible]	[illegible]	[illegible]	[illegible]	[illegible]	[illegible]	[illegible]	[illegible]	[illegible]	[illegible]	[illegible]	[illegible]	[illegible]	[illegible]	[illegible]	[illegible]	[illegible]	[illegible]	[illegible]	[illegible]	[illegible]	[illegible]	[illegible]	[illegible]	[illegible]	[illegible]
neg_19	[illegible]	[illegible]	[illegible]	[illegible]	[illegible]	[illegible]	[illegible]	[illegible]	[illegible]	[illegible]	[illegible]	[illegible]	[illegible]	[illegible]	[illegible]	[illegible]	[illegible]	[illegible]	[illegible]	[illegible]	[illegible]	[illegible]	[illegible]	[illegible]	[illegible]	[illegible]	[illegible]	[illegible]	[illegible]	[illegible]	[illegible]	[illegible]	[illegible]	[illegible]	[illegible]	[illegible]	[illegible]	[illegible]
neg_1905	[illegible]	[illegible]	[illegible]	[illegible]	[illegible]	[illegible]	[illegible]	[illegible]	[illegible]	[illegible]	[illegible]	[illegible]	[illegible]	[illegible]	[illegible]	[illegible]	[illegible]	[illegible]	[illegible]	[illegible]	[illegible]	[illegible]	[illegible]	[illegible]	[illegible]	[illegible]	[illegible]	[illegible]	[illegible]	[illegible]	[illegible]	[illegible]	[illegible]	[illegible]	[illegible]	[illegible]	[illegible]	[illegible]
neg_1906	[illegible]	[illegible]	[illegible]	[illegible]	[illegible]	[illegible]	[illegible]	[illegible]	[illegible]	[illegible]	[illegible]	[illegible]	[illegible]	[illegible]	[illegible]	[illegible]	[illegible]	[illegible]	[illegible]	[illegible]	[illegible]	[illegible]	[illegible]	[illegible]	[illegible]	[illegible]	[illegible]	[illegible]	[illegible]	[illegible]	[illegible]	[illegible]	[illegible]	[illegible]	[illegible]	[illegible]	[illegible]	[illegible]
neg_1923	[illegible]	[illegible]	[illegible]	[illegible]	[illegible]	[illegible]	[illegible]	[illegible]	[illegible]	[illegible]	[illegible]	[illegible]	[illegible]	[illegible]	[illegible]	[illegible]	[illegible]	[illegible]	[illegible]	[illegible]	[illegible]	[illegible]	[illegible]	[illegible]	[illegible]	[illegible]	[illegible]	[illegible]	[illegible]	[illegible]	[illegible]	[illegible]	[illegible]	[illegible]	[illegible]	[illegible]	[illegible]	[illegible]
neg_1929	[illegible]	[illegible]	[illegible]	[illegible]	[illegible]	[illegible]	[illegible]	[illegible]	[illegible]	[illegible]	[illegible]	[illegible]	[illegible]	[illegible]	[illegible]	[illegible]	[illegible]	[illegible]	[illegible]	[illegible]	[illegible]	[illegible]	[illegible]	[illegible]	[illegible]	[illegible]	[illegible]	[illegible]	[illegible]	[illegible]	[illegible]	[illegible]	[illegible]	[illegible]	[illegible]	[illegible]	[illegible]	[illegible]
neg_1950	[illegible]	[illegible]	[illegible]	[illegible]	[illegible]	[illegible]	[illegible]	[illegible]	[illegible]	[illegible]	[illegible]	[illegible]	[illegible]	[illegible]	[illegible]	[illegible]	[illegible]	[illegible]	[illegible]	[illegible]	[illegible]	[illegible]	[illegible]	[illegible]	[illegible]	[illegible]	[illegible]	[illegible]	[illegible]	[illegible]	[illegible]	[illegible]	[illegible]	[illegible]	[illegible]	[illegible]	[illegible]	[illegible]
neg_1954	[illegible]	[illegible]	[illegible]	[illegible]	[illegible]	[illegible]	[illegible]	[illegible]	[illegible]	[illegible]	[illegible]	[illegible]	[illegible]	[illegible]	[illegible]	[illegible]	[illegible]	[illegible]	[illegible]	[illegible]	[illegible]	[illegible]	[illegible]	[illegible]	[illegible]	[illegible]	[illegible]	[illegible]	[illegible]	[illegible]	[illegible]	[illegible]	[illegible]	[illegible]	[illegible]	[illegible]	[illegible]	[illegible]
neg_1959	[illegible]	[illegible]	[illegible]	[illegible]	[illegible]	[illegible]	[illegible]	[illegible]	[illegible]	[illegible]	[illegible]	[illegible]	[illegible]	[illegible]	[illegible]	[illegible]	[illegible]	[illegible]	[illegible]	[illegible]	[illegible]	[illegible]	[illegible]	[illegible]	[illegible]	[illegible]	[illegible]	[illegible]	[illegible]	[illegible]	[illegible]	[illegible]	[illegible]	[illegible]	[illegible]	[illegible]	[illegible]	[illegible]
neg_1964	[illegible]	[illegible]	[illegible]	[illegible]	[illegible]	[illegible]	[illegible]	[illegible]	[illegible]	[illegible]	[illegible]	[illegible]	[illegible]	[illegible]	[illegible]	[illegible]	[illegible]	[illegible]	[illegible]	[illegible]	[illegible]	[illegible]	[illegible]	[illegible]	[illegible]	[illegible]	[illegible]	[illegible]	[illegible]	[illegible]	[illegible]	[illegible]	[illegible]	[illegible]	[illegible]	[illegible]	[illegible]	[illegible]
neg_1967	[illegible]	[illegible]	[illegible]	[illegible]	[illegible]	[illegible]	[illegible]	[illegible]	[illegible]	[illegible]	[illegible]	[illegible]	[illegible]	[illegible]	[illegible]	[illegible]	[illegible]	[illegible]	[illegible]	[illegible]	[illegible]	[illegible]	[illegible]	[illegible]	[illegible]	[illegible]	[illegible]	[illegible]	[illegible]	[illegible]	[illegible]	[illegible]	[illegible]	[illegible]	[illegible]	[illegible]	[illegible]	[illegible]
neg_1968	[illegible]	[illegible]	[illegible]	[illegible]	[illegible]	[illegible]	[illegible]	[illegible]	[illegible]	[illegible]	[illegible]	[illegible]	[illegible]	[illegible]	[illegible]	[illegible]	[illegible]	[illegible]	[illegible]	[illegible]	[illegible]	[illegible]	[illegible]	[illegible]	[illegible]	[illegible]	[illegible]	[illegible]	[illegible]	[illegible]	[illegible]	[illegible]	[illegible]	[illegible]	[illegible]	[illegible]	[illegible]	[illegible]
neg_1975	[illegible]	[illegible]	[illegible]	[illegible]	[illegible]	[illegible]	[illegible]	[illegible]	[illegible]	[illegible]	[illegible]	[illegible]	[illegible]	[illegible]	[illegible]	[illegible]	[illegible]	[illegible]	[illegible]	[illegible]	[illegible]	[illegible]	[illegible]	[illegible]	[illegible]	[illegible]	[illegible]	[illegible]	[illegible]	[illegible]	[illegible]	[illegible]	[illegible]	[illegible]	[illegible]	[illegible]	[illegible]	[illegible]
neg_1979	[illegible]	[illegible]	[illegible]	[illegible]	[illegible]	[illegible]	[illegible]	[illegible]	[illegible]	[illegible]	[illegible]	[illegible]	[illegible]	[illegible]	[illegible]	[illegible]	[illegible]	[illegible]	[illegible]	[illegible]	[illegible]	[illegible]	[illegible]	[illegible]	[illegible]	[illegible]	[illegible]	[illegible]	[illegible]	[illegible]	[illegible]	[illegible]	[illegible]	[illegible]	[illegible]	[illegible]	[illegible]	[illegible]
neg_1996	[illegible]	[illegible]	[illegible]	[illegible]	[illegible]	[illegible]	[illegible]	[illegible]	[illegible]	[illegible]	[illegible]	[illegible]	[illegible]	[illegible]	[illegible]	[illegible]	[illegible]	[illegible]	[illegible]	[illegible]	[illegible]	[illegible]	[illegible]	[illegible]	[illegible]	[illegible]	[illegible]	[illegible]	[illegible]	[illegible]	[illegible]	[illegible]	[illegible]	[illegible]	[illegible]	[illegible]	[illegible]	[illegible]
neg_2	[illegible]	[illegible]	[illegible]	[illegible]	[illegible]	[illegible]	[illegible]	[illegible]	[illegible]	[illegible]	[illegible]	[illegible]	[illegible]	[illegible]	[illegible]	[illegible]	[illegible]	[illegible]	[illegible]	[illegible]	[illegible]	[illegible]	[illegible]	[illegible]	[illegible]	[illegible]	[illegible]	[illegible]	[illegible]	[illegible]	[illegible]	[illegible]	[illegible]	[illegible]	[illegible]	[illegible]	[illegible]	[illegible]
neg_2003	[illegible]	[illegible]	[illegible]	[illegible]	[illegible]	[illegible]	[illegible]	[illegible]	[illegible]	[illegible]	[illegible]	[illegible]	[illegible]	[illegible]	[illegible]	[illegible]	[illegible]	[illegible]	[illegible]	[illegible]	[illegible]	[illegible]	[illegible]	[illegible]	[illegible]	[illegible]	[illegible]	[illegible]	[illegible]	[illegible]	[illegible]	[illegible]	[illegible]	[illegible]	[illegible]	[illegible]	[illegible]	[illegible]
neg_2004	[illegible]	[illegible]	[illegible]	[illegible]	[illegible]	[illegible]	[illegible]	[illegible]	[illegible]	[illegible]	[illegible]	[illegible]	[illegible]	[illegible]	[illegible]	[illegible]	[illegible]	[illegible]	[illegible]	[illegible]	[illegible]	[illegible]	[illegible]	[illegible]	[illegible]	[illegible]	[illegible]	[illegible]	[illegible]	[illegible]	[illegible]	[illegible]	[illegible]	[illegible]	[illegible]	[illegible]	[illegible]	[illegible]

neg_2007	Acetamidopropanal	metab_7264	HMDB0012880	B(ii)	C18170	275.1253	3.0846	neg	2M+FA-H	C5H9NO2	0	[illegible]
neg_2021	N-Arachidonoyl Glycine	metab_7276	HMDB0241918;LMFA08020003;HMDB0005096	B(ii)	-	356.0995	3.0986	neg	M-H	C15H19NO9	0	[illegible]
neg_2028	Djenkolic acid	metab_7283	HMDB0251505	B(ii)	C08275	235.0224	3.1241	neg	M-H2O-H	C7H14N2O4S2	0	[illegible]
neg_2034	4-Nitrophenyl beta-D-xyloside	metab_7290	HMDB0245573	B(ii)	-	292.0443	3.1421	neg	M+Na-2H	C11H13NO7	0	[illegible]
neg_2038	5,6-Dihydro-5-hydroxy-6-methyl-2H-pyra	metab_7294	HMDB0034270	B(ii)	C09925	263.0499	3.1552	neg	M+Cl	C14H12O3	0	[illegible]
neg_2044	6-[4-(carboxymethyl)phenoxy]-3,4,5-trihyd	metab_7301	HMDB0132498	B(ii)	-	327.0727	3.1612	neg	M-H	C14H16O9	0	[illegible]
neg_2053	2-Hydroxy-3-Methylbutyric Acid	metab_7311	LMFA01050478;HMDB0000407;MJDBOTE0000595	B(i)	-	117.0551	3.192	neg	M-H	C5H10O3	67.1	[illegible]
neg_2058	(1R,6R)-6-hydroxy-2-succinylcyclohexa-2,4	metab_7316	HMDB0303951	B(ii)	C05817	285.062	3.2117	neg	M+FA-H	C11H12O6	0	[illegible]
neg_2059	5-Acetyl-3,4-dihydro-2H-pyrrole	metab_7317	HMDB0031308	B(ii)	-	156.0661	3.2158	neg	M+FA-H	C6H9NO	0	[illegible]
neg_2064	4,5-Dihydro-1-(3-(trifluoromethyl)phenyl)-	metab_7323	HMDB0247177	B(ii)	-	503.1643	3.2273	neg	2M+FA-H	C10H10F3N3	0	[illegible]
neg_2078	N-Acetyl-L-Tyrosine	metab_7336	HMDB0000866;HMDB0244966;MJDBOTE0000672	B(i)	C01657	222.0771	3.2607	neg	M-H	C11H13NO4	94.5	[illegible]
neg_2102	2-Amino-3-methylbenzoate	metab_7364	PW_C040581;HMDB0060680	B(ii)	C16571	196.0612	3.3692	neg	M+FA-H	C8H9NO2	0	[illegible]
neg_2108	Homovanillin	metab_7370	PW_C002411;HMDB0005175	B(ii)	C05581	211.0609	3.3944	neg	M+FA-H	C9H10O3	0	[illegible]
neg_2110	Tuliposide A	metab_7373	LMFA13010041;HMDB0303140	B(ii)	C08561	299.0776	3.399	neg	M+Na-2H	C11H18O8	0	[illegible]
neg_2111	2,6-Dimethoxy-1,4-benzoquinone	metab_7374	HMDB0029673	B(ii)	C10331	149.0238	3.4045	neg	M-H2O-H	C8H8O4	0	[illegible]
neg_2113	3,5-Dichloro-L-tyrosine	metab_7376	HMDB0244684	B(ii)	C03347	285.9454	3.4095	neg	M+K-2H	C9H9Cl2NO3	0	[illegible]
neg_2123	Piridronic acid	metab_7387	HMDB0256596	B(ii)	-	303.956	3.4482	neg	M+K-2H	C7H11NO6P2	0	[illegible]
neg_2124	3,4-Dihydroxybenzaldehyde	metab_7388	HMDB0059965;MJDBOTE0000419	B(i)	C16700	137.0238	3.4518	neg	M-H	C7H6O3	39.9	[illegible]
neg_2135	Butyl 4-aminobenzoate	metab_7400	HMDB0249473	B(ii)	C07875	431.2158	3.4915	neg	2M+FA-H	C11H15NO2	0	[illegible]
neg_2139	Umbelliferone	metab_7404	HMDB0029865;MJDBOTE0000399	B(ii)	C09315	485.0926	3.4981	neg	3M-H	C9H6O3	0	[illegible]
neg_2145	Carbamazepine-O-quinone	metab_7411	HMDB0060566;PW_C040556	B(ii)	C16606	303.0185	3.5276	neg	M+K-2H	C15H10N2O3	0	[illegible]
neg_215	Glucose lactate acetate	metab_7416	HMDB0252779	B(ii)	-	363.0354	0.7495	neg	M-H2O-H, M+	C11H18O11	0	[illegible]
neg_2155	1-Carboxyethyltyrosine	metab_7422	HMDB0242159	B(ii)	-	252.088	3.5765	neg	M-H	C12H15NO5	0	[illegible]
neg_2159	7-Methoxycoumarin-3-carbonyl azide	metab_7426	HMDB0247270	B(ii)	-	244.0365	3.5983	neg	M-H	C11H7N3O4	0	[illegible]
neg_216	Hydroxyphenylacetylglycine	metab_7427	HMDB0000735	B(ii)	C05596	208.0612	0.7495	neg	M-H, M+Cl	C10H11NO4	0	[illegible]
neg_2161	Dopaxanthin quinone	metab_7429	HMDB0012220	B(ii)	C17753	423.0611	3.6029	neg	M+Cl	C18H16N2O8	0	[illegible]
neg_2168	2-O-(6-Phospho-alpha-mannosyl)-D-glyc	metab_7436	HMDB0012152	B(ii)	C16699	369.0225	3.6465	neg	M+Na-2H	C9H17O12P	0	[illegible]
neg_2172	Shanzhiside	metab_7441	HMDB0258270	B(ii)	C17066	391.123	3.652	neg	M-H	C16H24O11	0	[illegible]
neg_2179	7,8-dihydromonapterin	metab_7447	HMDB0304238	B(ii)	C21008	290.065	3.6715	neg	M+Cl	C9H13N5O4	0	[illegible]
neg_2189	4-Hydroxybenzeneacetonitrile	metab_7458	HMDB0029757	B(ii)	C03766	178.0505	3.7201	neg	M+FA-H	C8H7NO	0	[illegible]
neg_2201	2,5-Dihydroxy-1H-pyrrole-3-sulfonic acid	metab_7473	HMDB0255400	B(ii)	-	402.978	3.7723	neg	2M+FA-H	C4H5NO5S	0	[illegible]
neg_2202	Gluconolactone	metab_7474	HMDB0000150;PW_C000097;MJDBOTE0000432	B(ii)	C00198	223.0458	3.7768	neg	M+FA-H	C6H10O6	0	[illegible]
neg_2205	16-Oxoestrone	metab_7477	LMST02010048;HMDB0000372	B(ii)	C14441	321.0883	3.7945	neg	M+K-2H	C18H20O3	0	[illegible]
neg_2206	3-Hydroxycinnamoylglycine sulfate	metab_7478	HMDB0304916	B(ii)	-	322.0029	3.7991	neg	M+Na-2H	C11H11NO7S	0	[illegible]
neg_2208	4-methoxy-3-(sulfooxy)benzoic acid	metab_7480	HMDB0140929	B(ii)	-	228.9809	3.8032	neg	M-H2O-H	C8H8O7S	0	[illegible]
neg_2216	Quercetin 3-sulfate	metab_7488	HMDB0303771	B(ii)	C00616	380.9956	3.8364	neg	M-H	C15H10O10S	0	[illegible]
neg_2219	Bolton-Hunter reagent	metab_7491	HMDB0246176	B(ii)	-	308.078	3.8455	neg	M+FA-H	C13H13NO5	0	[illegible]
neg_2220	Esculetin	metab_7493	HMDB0030819;MJDBOTE0000366	B(i)	C09263	177.0188	3.85	neg	M-H	C9H6O4	78.7	[illegible]
neg_2223	Biotin	metab_7496	HMDB0000030;PW_C000020;MJDBOTE0000823	B(i)	C00120	243.081	3.8661	neg	M-H	C10H16N2O3S	77	[illegible]
neg_2235	2-Isopropylmalic acid	metab_7508	HMDB0000402	B(ii)	C02504	197.0427	3.9099	neg	M+Na-2H	C7H12O5	0	[illegible]
neg_2238	Asn Ile Thr Glu	metab_7511	-	B(i)	-	474.2215	3.9145	neg	M-H	C19H33N5O9	60	[illegible]
neg_2239	3-Methyl-1-(2,4,6-trihydroxyphenyl)-1-but	metab_7512	HMDB0033798	B(ii)	C07350	255.089	3.928	neg	M+FA-H	C11H14O4	0	[illegible]
neg_2247	4-Hydroxy-3-methoxybenzenemethanol	metab_7520	HMDB0032012	B(ii)	C06317	153.0551	3.9648	neg	M-H	C8H10O3	0	[illegible]
neg_2258	Gabazine	metab_7532	HMDB0252568	B(ii)	C13796	332.1257	4.0031	neg	M+FA-H	C15H17N3O3	0	[illegible]
neg_2259	Gamma-L-Glutamyl-L-pipecolic acid	metab_7533	HMDB0038614	B(ii)	-	257.1144	4.0082	neg	M-H	C11H18N2O5	0	[illegible]
neg_2272	Methyl 2,6-dimethyl-5-nitro-4-[2-(trifluorc	metab_7548	HMDB0247310	B(ii)	C13758	337.0836	4.0487	neg	M-H2O-H	C16H15F3N2O4	0	[illegible]
neg_2273	6-Hydroxymelatonin	metab_7549	HMDB0004081;PW_C002052	B(ii)	C05643	283.084	4.0532	neg	M+Cl	C13H16N2O3	0	[illegible]
neg_2280	Aciclovir	metab_7557	HMDB0014925;MJDBOTE0001429	B(ii)	C06810	260.0544	4.0816	neg	M+Cl	C8H11N5O3	0	[illegible]
neg_229	Verbascose	metab_7567	HMDB0259784	B(ii)	C08252	863.2474	0.67	neg	M-H, M+Cl	C30H52O26	0	[illegible]
neg_2290	Feruloyltartaric acid	metab_7568	HMDB0303700	B(ii)	-	325.057	4.1228	neg	M-H	C14H14O9	0	[illegible]
neg_2300	Salannin	metab_7580	HMDB0257456	B(ii)	C08780	633.2424	4.1485	neg	M+K-2H	C34H44O9	0	[illegible]
neg_2304	Heliotridine	metab_7584	HMDB0253071	B(ii)	C10321	200.0925	4.1737	neg	M+FA-H	C8H13NO2	0	[illegible]
neg_2318	Taraxacoside	metab_7599	HMDB0030055	B(ii)	-	443.1204	4.237	neg	M+FA-H	C18H22O10	0	[illegible]
neg_2326	Isoschaftoside	metab_7608	LMPK12110243	B(i)	-	563.1409	4.2787	neg	M-H	C26H28O14	72.3	[illegible]
neg_233	D-Arabinono-1,4-lactone	metab_7612	HMDB0302833	B(ii)	C00652	147.0293	1.0124	neg	M-H2O-H, M-I	C5H8O5	0	[illegible]
neg_2335	N(1)-Acetylsulfamethoxazole	metab_7617	HMDB0255280	B(ii)	-	635.1275	4.3542	neg	2M+FA-H	C12H13N3O4S	0	[illegible]
neg_2338	Salicyluric Acid	metab_7620	HMDB0000840	B(i)	C07588	194.0455	4.3811	neg	M-H	C9H9NO4	52.4	[illegible]
neg_2352	Clothianidin	metab_7636	HMDB0250370	B(i)	C18508	248.0016	4.4176	neg	M-H	C6H8ClN5O2S	70.7	[illegible]
neg_2355	2,4-Dihydroxybenzoic acid	metab_7639	HMDB0001856;HMDB0029666	B(ii)	C00230	153.0188	4.4414	neg	M-H	C7H6O4	0	[illegible]
neg_2358	8-[(Aminomethyl)sulfanyl]-6-sulfanyloctanc	metab_7642	PW_C008526;HMDB0013639	B(ii)	-	272.0544	4.4571	neg	M+Cl	C9H19NO2S2	0	[illegible]
neg_2360	Efavirenz	metab_7645	PW_C009112;HMDB0014763;MJDBOTE0001689	B(ii)	C08088	349.9978	4.4611	neg	M+Cl	C14H9ClF3NO2	0	[illegible]
neg_2361	(R)-Menthone 8-thioacetate	metab_7646	HMDB0303595	B(ii)	-	265.0654	4.4647	neg	M+K-2H	C12H20O2S	0	[illegible]
neg_2369	Acetyl-DL-Leucine	metab_7654	HMDB0011756	B(i)	C02710	172.0974	4.5034	neg	M-H	C8H15NO3	67	[illegible]
neg_2378	Isopropyl 4-hydroxybenzoate	metab_7664	HMDB0253675	B(ii)	C20343	225.0768	4.5328	neg	M+FA-H	C10H12O3	0	[illegible]
neg_2381	Arginylaspartic acid	metab_7668	HMDB0028705	B(ii)	-	577.265	4.5445	neg	2M-H	C10H19N5O5	0	[illegible]
neg_2392	Ruberythric acid	metab_7678	HMDB0257353	B(ii)	-	533.1314	4.5925	neg	M-H	C25H26O13	0	[illegible]
neg_2397	Phenylpropionylglycine	metab_7683	HMDB0000860	B(ii)	-	228.0641	4.6202	neg	M+Na-2H	C11H13NO3	0	[illegible]
neg_2398	Glycine, N-[3-(4-hydroxy-3-methoxypheny	metab_7684	HMDB0244468	B(ii)	-	272.0544	4.6248	neg	M+Na-2H	C12H13NO5	0	[illegible]
neg_2399	Sesartemin	metab_7685	HMDB0038931	B(ii)	C10884	411.1411	4.6283	neg	M-H2O-H	C23H26O8	0	[illegible]
neg_2404	N-Eicosapentaenoyl Tryptophan	metab_7692	HMDB0242081	B(ii)	-	509.2768	4.6649	neg	M+Na-2H	C31H40N2O3	0	[illegible]
neg_2425	6-Thioinosine-5'-monophosphate	metab_7715	HMDB0060416;PW_C040504	B(ii)	C04646	345.0031	4.7463	neg	M-H2O-H	C10H13N4O7PS	0	[illegible]
neg_2426	Tyramine glucuronide	metab_7716	HMDB0010328	B(ii)	C03033	294.0988	4.7555	neg	M-H2O-H	C14H19NO7	0	[illegible]
neg_2428	2-[3-Methoxy-2-propoxy-5-[5-(3,4,5-trim	metab_7718	HMDB0253852	B(ii)	-	491.1782	4.7605	neg	M-H2O-H	C25H34O9S	0	[illegible]
neg_2440	Thiamylal	metab_7731	HMDB0015285	B(ii)	C07846	275.0831	4.8213	neg	M+Na-2H	C12H18N2O2S	0	[illegible]
neg_2449	1-Acetyl-3,27-dihydroxywitha-5,24-dienoli	metab_7740	HMDB0033729	B(ii)	-	707.3647	4.8475	neg	M+FA-H	C36H54O11	0	[illegible]
neg_2451	Corchoionoside B	metab_7743	HMDB0030975	B(ii)	-	399.1668	4.8546	neg	M-H	C19H28O9	0	[illegible]
neg_2452	Rutin	metab_7744	HMDB0257021;HMDB0003249;LMPK12112098	B(i)	C05625	609.148	4.8586	neg	M-H	C27H30O16	53	[illegible]
neg_2455	3-butenylglucosinolate	metab_7747	HMDB0304115;HMDB0038427	B(ii)	C08415	408.9876	4.8929	neg	M+K-2H	C11H18NO9S2-	0	[illegible]
neg_2457	Ferulic Acid	metab_7749	HMDB0000954;MJDBOTE0000325	B(i)	C01494	193.0502	4.896	neg	M-H	C10H10O4	93.2	[illegible]
neg_2462	2,2':5',2''-Terthiophene	metab_7754	HMDB0245378	B(ii)	C08460	292.9763	4.9207	neg	M+FA-H	C12H8S3	0	[illegible]
neg_247	3-[(3-(2-Carboxyethyl)-4-methylpyrrol-2-\	metab_7761	HMDB0258526	B(ii)	-	333.0638	1.1473	neg	M-H, M+K-2H	C17H16N2O3	0	[illegible]
neg_2472	4-Hydroxy-4-(methylnitrosoamino)-1-(3-p	metab_7764	HMDB0062404	B(ii)	C19566	222.0897	4.9763	neg	M-H	C10H13N3O3	0	[illegible]
neg_2473	Quercetin 3-O-glucoside	metab_7765	HMDB0037362	B(i)	C05623	463.0895	4.985	neg	M-H	C21H20O12	80.7	[illegible]
neg_2474	Phaseolic acid	metab_7766	LMFA01060200;HMDB0031897	B(ii)	C10483	261.1346	4.99	neg	M-H	C12H22O6	0	[illegible]
neg_2476	1-Deoxy-1-morpholino-D-fructose	metab_7768	HMDB0243858	B(ii)	-	270.0963	4.9936	neg	M+Na-2H	C10H19NO6	0	[illegible]
neg_248	Oxoglutaric acid	metab_7772	HMDB0000208;PW_C000134	B(ii)	C00026	191.0193	1.0602	neg	M+Cl, M+FA-H	C5H6O5	0	[illegible]
neg_2485	Indolelactic Acid	metab_7778	HMDB0000671	B(i)	C02043	204.0663	5.03	neg	M-H	C11H11NO3	83.9	[illegible]
neg_2486	Estriol	metab_7779	PW_C000100;HMDB0000347;HMDB0000153;MJDBOT	B(ii)	C05141	863.5031	5.03	neg	3M-H	C18H24O3	0	[illegible]
neg_2501	Acetylbalchanolide	metab_7797	HMDB0036693	B(ii)	-	329.1148	5.1111	neg	M+K-2H	C17H24O4	0	[illegible]
neg_2508	4-Methylcatechol	metab_7804	HMDB0000873;MJDBOTE0000523	B(i)	C06730	123.0445	5.1388	neg	M-H	C7H8O2	40.2	[illegible]
neg_2519	9-Oxo-nonanoic acid	metab_7816	LMFA01060160;HMDB0094711	B(ii)	C16322	217.108	5.2175	neg	M+FA-H	C9H16O3	0	[illegible]
neg_2529	Budralazine	metab_7827	HMDB0249426	B(ii)	-	285.1347	5.2707	neg	M+FA-H	C14H16N4	0	[illegible]
neg_2539	2,4-Diacetylphloroglucinol	metab_7837	-	B(i)	C21896	209.0453	5.3327	neg	M-H	C10H10O5	38.1	[illegible]
neg_2541	Lumichrome	metab_7840	HMDB0254199	B(i)	C01727	241.0732	5.3413	neg	M-H	C12H10N4O2	76.6	[illegible]
neg_2546	Pelargonic acid	metab_7845	LMFA01010009;HMDB0000847	B(ii)	C01601	203.1285	5.3655	neg	M+FA-H	C9H18O2	0	[illegible]
neg_2581	Isorhamnetin 4'-O-glucuronide	metab_7884	HMDB0041757	B(ii)	-	473.0745	5.5168	neg	M-H2O-H	C22H20O13	0	[illegible]
neg_2588	5-hydroxy-2-oxo-4-ureido-2,5-dihydro-1I	metab_7891	HMDB0304214	B(ii)	-	182.009	5.5523	neg	M-H2O-H	C5H5N4O5-	0	[illegible]
neg_259	Galactonolactone	metab_7893	HMDB0002541	B(ii)	C03383	177.04	1.227	neg	M-H2O-H, M-I	C6H10O6	0	[illegible]
neg_260	Fluvoxamino acid	metab_7905	HMDB0060950	B(ii)	-	299.0987	0.6621	neg	M-H2O-H, M+	C14H17F3N2O3	0	[illegible]
neg_2617	THIARABINE	metab_7924	HMDB0258974	B(ii)	-	240.0434	5.7519	neg	M-H2O-H	C9H13N3O4S	0	[illegible]
neg_2624	Linocinnamarin	metab_7932	HMDB0030678	B(ii)	-	321.0985	5.7889	neg	M-H2O-H	C16H20O8	0	[illegible]
neg_2634	(5E,7E)-Undeca-2,5,7-trienedioylcarnitine	metab_7942	HMDB0241202	B(ii)	-	352.1771	5.8432	neg	M-H	C18H27NO6	0	[illegible]
neg_2663	Resolvin E2	metab_7974	LMFA03140011;HMDB0257163	B(ii)	C18173	355.1879	5.9193	neg	M+Na-2H	C20H30O4	0	[illegible]
neg_2664	(2E,8E)-10-hydroxydeca-2,8-dien-4-ynoyl	metab_7975	HMDB0241109	B(ii)	-	368.172	5.9193	neg	M+FA-H	C17H25NO5	0	[illegible]
neg_2668	PS(14:0/14:1(9Z))	metab_7979	LMGP03010094;HMDB0012331;PW_C007861	B(ii)	C02737	714.3706	5.9269	neg	M+K-2H	C34H64NO10P	0	[illegible]
neg_2682	Sufentanil	metab_7994	PW_C009183;HMDB0014846	B(ii)	C08022	407.1753	5.9577	neg	M+Na-2H	C22H30N2O2S	0	[illegible]
neg_2695	N-Succinyl-L,L-2,6-diaminopimelate	metab_8008	HMDB0012267	B(ii)	C04421	289.1057	5.9885	neg	M-H	C11H18N2O7	0	[illegible]
neg_2708	Alogliptin	metab_8023	HMDB0248177	B(ii)	-	338.1614	6.0207	neg	M-H	C18H21N5O2	0	[illegible]
neg_2721	2-Mercaptobenzothiazole	metab_8036	HMDB0030524	B(i)	C14437	165.9785	6.0454	neg	M-H	C7H5NS2	93.7	[illegible]
neg_2722	Nitrosobenzene	metab_8037	HMDB0255658	B(ii)	C06876	152.0347	6.05	neg	M+FA-H	C6H5NO	0	[illegible]
neg_2723	Gingerol	metab_8038	HMDB0005783	B(ii)	C10462	339.1816	6.05	neg	M+FA-H	C17H26O4	0	[illegible]
neg_2732	1,2-Cyclohexanediol, 1-methyl-4-(1-methy	metab_8048	HMDB0256048	B(ii)	C18020	215.1286	6.0868	neg	M+FA-H	C10H18O2	0	[illegible]
neg_2733	Epothilone C	metab_8049	HMDB0251875	B(ii)	C15694	522.2489	6.0868	neg	M+FA-H	C26H39NO5S	0	[illegible]
neg_2740	Kessyl glycol	metab_8057	HMDB0037197	B(ii)	-	299.1866	6.1149	neg	M+FA-H	C15H26O3	0	[illegible]
neg_2749	Houttuynin	metab_8066	HMDB0034561	B(ii)	C16947	225.1859	6.138	neg	M-H	C14H26O2	0	[illegible]
neg_2752	24-Epibrassinolide	metab_8069	HMDB0034081;HMDB0041130	B(ii)	C11049	501.3236	6.146	neg	M+Na-2H	C28H48O6	0	[illegible]
neg_2756	Gamma-linolenyl carnitine	metab_8073	HMDB0006318;LMFA07070112	B(ii)	-	466.3177	6.162	neg	M+FA-H	C25H43NO4	0	[illegible]
neg_2765	9,10,13-TriHOME	metab_8083	HMDB0004710	B(ii)	C14835	329.2336	6.191	neg	M-H	C18H34O5	0	[illegible]
neg_2767	Rocuronium	metab_8085	HMDB0014866	B(ii)	C07556	566.3479	6.191	neg	M+K-2H	C32H53N2O4+	0	[illegible]
neg_2769	3H-Pyrido(1,2-c)pyrimidin-3-one, 4-(2-(bis	metab_8087	HMDB0243618	B(ii)	-	414.2769	6.199	neg	M+FA-H	C23H35N3O	0	[illegible]
neg_277	Uridine	metab_8088	HMDB0000296;PW_C000202;MJDBOTE0000788	B(i)	C00299	243.0625	1.395	neg	M-H, M+FA-H	C9H12N2O6	93	[illegible]
neg_2774	Tetranor 12-HETE	metab_8093	HMDB0060055	B(ii)	-	247.1704	6.2145	neg	M-H2O-H	C16H26O3	0	[illegible]
neg_278	2-Furamide	metab_8098	HMDB0015684;HMDB0245133	B(ii)	C07637	110.0241	1.395	neg	M-H, M+FA-H	C5H5NO2	0	[illegible]
neg_2780	Octadecenoylcarnitine	metab_8099	HMDB0094687	B(ii)	-	470.3496	6.2225	neg	M+FA-H	C25H47NO4	0	[illegible]
neg_2781	Leu-Arg-Asn-Arg	metab_8100	HMDB0304803	B(ii)	-	578.3116	6.2225	neg	M+Na-2H	C22H43N11O6	0	[illegible]
neg_2785	(10E,12E,14E)-16-Hydroxy-9-oxooctadeca	metab_8104	HMDB0241556	B(ii)	-	488.2427	6.2385	neg	M+K-2H	C25H41NO6	0	[illegible]
neg_2788	Inecalcitol	metab_8107	LMST03020649;HMDB0242665	B(ii)	-	421.2715	6.2465	neg	M+Na-2H	C26H40O3	0	[illegible]
neg_2791	Persenone B	metab_8111	HMDB0035959;LMFA05000719	B(ii)	-	419.2558	6.2625	neg	M+K-2H	C23H42O4	0	[illegible]
neg_2792	LysoPS(18:2(9Z,12Z)/0:0)	metab_8112	HMDB0240604	B(ii)	-	520.269	6.2625	neg	M-H	C24H44NO9P	0	[illegible]
neg_2796	Trans-Dodec-2-enoic acid	metab_8116	PW_C006736;HMDB0010729	B(ii)	-	243.1601	6.2785	neg	M+FA-H	C12H22O2	0	[illegible]
neg_2799	1-Methoxy-4-(2-propenyl)benzene	metab_8119	HMDB0034121	B(ii)	C10452	193.0865	6.2865	neg	M+FA-H	C10H12O	0	[illegible]
neg_2801	2,4,5,7alpha-Tetrahydro-1,4,4,7a-tetrameth	metab_8122	HMDB0036684	B(ii)	-	237.1495	6.2945	neg	M+FA-H	C13H20O	0	[illegible]
neg_2804	Tetranor-12(R)-HETE	metab_8125	-	B(i)	-	265.181	6.3025	neg	M-H	C16H26O3	54.8	[illegible]
neg_2811	Phaeophorbide b	metab_8132	HMDB0031149	B(ii)	-	651.2477	6.3265	neg	M+FA-H	C35H34N4O6	0	[illegible]
neg_2813	PG(16:0/0:0)[U]	metab_8134	-	B(i)	-	483.2737	6.3345	neg	M-H	C22H45O9P	63.1	[illegible]
neg_2815	LysoPE(14:0/0:0)	metab_8136	HMDB0011500	B(ii)	-	446.232	6.3425	neg	M+Na-2H	C19H40NO7P	0	[illegible]
neg_2817	2-Stearoylglycerophosphoglycerol	metab_8138	HMDB0061703	B(ii)	-	547.2814	6.3425	neg	M+Cl	C24H49O9P	0	[illegible]
neg_2819	4-(5H-Dibenzo[a,d]cyclohepten-5-ylidene)	metab_8140	HMDB0248686	B(ii)	-	396.2219	6.3505	neg	M-H	C25H27N5	0	[illegible]
neg_282	Hydroxykynurenine	metab_8141	HMDB0000732	B(i)	C02794	223.0724	1.283	neg	M-H2O-H, M-I	C10H12N2O4	91.1	[illegible]
neg_2828	Dihomo-alpha-linolenic acid	metab_8150	HMDB0060039	B(ii)	C16522	327.2287	6.3665	neg	M+Na-2H	C20H34O2	0	[illegible]
neg_2829	Cafestol	metab_8151	HMDB0035710	B(ii)	C09066	361.2001	6.3665	neg	M+FA-H	C20H28O3	0	[illegible]
neg_2830	Edrophonium	metab_8153	HMDB0015145	B(ii)	C06976	377.2449	6.3665	neg	2M+FA-H	C10H16NO+	0	[illegible]
neg_2836	Ajmalicine	metab_8159	HMDB0248072	B(ii)	C09024	387.1483	6.3825	neg	M+Cl	C21H24N2O3	0	[illegible]
neg_2840	Eriojaposide A	metab_8164	HMDB0038028	B(ii)	C17888	547.2363	6.3905	neg	M+FA-H	C24H38O11	0	[illegible]
neg_2844	Tanacetol B	metab_8168	HMDB0035075	B(ii)	-	295.1915	6.4145	neg	M-H	C17H28O4	0	[illegible]
neg_2845	PE(20:5/0:0)	metab_8169	-	B(i)	-	498.2633	6.4145	neg	M-H	C25H42NO7P	92.3	[illegible]
neg_2846	18-Nitrooctadeca-9,12-dienoic acid	metab_8170	HMDB0257646	B(ii)	-	306.2076	6.4225	neg	M-H2O-H	C18H31NO4	0	[illegible]
neg_2847	5,12-Naphthacenedione, 10-((4-aminotetra	metab_8171	HMDB0246848	B(ii)	-	574.1888	6.4225	neg	M+FA-H	C27H31NO10	0	[illegible]
neg_2848	PI(TXB2/20:4(8Z,11Z,14Z,17Z))	metab_8172	HMDB0277626	B(ii)	-	971.5171	6.4225	neg	M-H	C49H81O17P	0	[illegible]
neg_2849	PE(LTE4/20:1(11Z))	metab_8173	HMDB0261957	B(ii)	-	949.5357	6.4305	neg	M+Na-2H	C48H85N2O11PS	0	[illegible]
neg_2851	PE(18:3/0:0)	metab_8176	-	B(i)	-	474.2636	6.4385	neg	M-H	C23H42NO7P	90.7	[illegible]
neg_2853	N-Eicosapentaenoyl Glutamine	metab_8178	HMDB0242069	B(ii)	-	429.2762	6.4545	neg	M-H	C25H38N2O4	0	[illegible]
neg_2854	(R)-1-O-[b-D-Glucopyranosyl-(1->6)-b-D	metab_8179	HMDB0032799	B(ii)	-	505.2018	6.4545	neg	M+Cl	C20H38O12	0	[illegible]
neg_2858	10'-Apo-beta-carotenal	metab_8182	HMDB0059605	B(ii)	-	811.5621	6.4625	neg	2M+Hac-H	C27H36O	0	[illegible]
neg_2862	PE(22:2(13Z,16Z)/18:2(9Z,11E)+=O(13))	metab_8187	HMDB0283974	B(ii)	-	830.5358	6.4785	neg	M+Na-2H	C45H80NO9P	0	[illegible]
neg_2864	1-Methyl-2-undecylquinolin-4(1H)-one	metab_8189	HMDB0243927	B(ii)	-	358.2391	6.4865	neg	M+FA-H	C21H31NO	0	[illegible]
neg_2871	3-((((-Oxoheptyl)amino)acetyl)amino)methy	metab_8196	HMDB0258446	B(ii)	-	403.2605	6.502	neg	M-H2O-H	C23H38N2O5	0	[illegible]
neg_2874	PE(20:3(8Z,11Z,14Z)/P-16:0)	metab_8198	HMDB0009378	B(ii)	C00350	770.5341	6.5095	neg	M+FA-H	C41H76NO7P	0	[illegible]
neg_2885	SM(d18:1/14:1(9Z))	metab_8207	HMDB0240612	B(ii)	-	717.521	6.5929	neg	M+FA-H	C37H73N2O6P	0	[illegible]
neg_2889	Triton X-100	metab_8209	HMDB0259285	B(ii)	C11623	249.1859	6.6217	neg	M-H	C16H26O2	0	[illegible]
neg_2890	PC(18:1(12Z)-O(9S,10R)/17:0)	metab_8211	HMDB0286160	B(ii)	-	832.5747	6.6217	neg	M+FA-H	C43H82NO9P	0	[illegible]
neg_2891	Delimotecan	metab_8212	HMDB0250970	B(ii)	-	619.2579	6.6297	neg	M-H	C31H36N6O8	0	[illegible]
neg_2896	N-Ethyl trans-2-cis-6-nonadienamide	metab_8216	LMFA08020193;HMDB0032273	B(ii)	C06551	407.2921	6.6537	neg	2M+FA-H	C11H19NO	0	[illegible]
neg_29	Vanilloylglycine	metab_8220	HMDB0060026	B(ii)	-	224.0564	3.0766	neg	M+Cl, 2M-H, M	C10H11NO5	0	[illegible]
neg_2903	PI(TXB2/20:1(11Z))	metab_8224	HMDB0277106	B(ii)	-	977.5631	6.6617	neg	M-H	C49H87O17P	0	[illegible]
neg_2905	Propafenone glucuronide	metab_8226	HMDB0247645	B(ii)	-	552.2053	6.6697	neg	M+Cl	C27H35NO9	0	[illegible]
neg_2906	Eicosapentaenoic Acid	metab_8227	HMDB0001999;PW_C001337	B(i)	C06428	301.2175	6.6777	neg	M-H	C20H30O2	90.7	[illegible]
neg_2909	Canavalioside	metab_8230	HMDB0036317	B(ii)	-	591.2628	6.6777	neg	M+FA-H	C26H42O12	0	[illegible]
neg_2913	DG(20:4(6E,8Z,11Z,14Z)-OH(5S)/0:0/2:0)	metab_8235	HMDB0296870	B(ii)	-	931.5821	6.6937	neg	2M+Hac-H	C25H40O6	0	[illegible]
neg_2914	(-)-trans-Carveol	metab_8236	HMDB0003450;LMPR0102090005	B(ii)	C00964	349.2388	6.7016	neg	2M+FA-H	C10H16O	0	[illegible]
neg_2915	PE(18:1(9Z)/0:0)	metab_8237	LMGP02050004	B(i)	-	478.2946	6.7095	neg	M-H	C23H46NO7P	97.7	[illegible]
neg_2917	13,14-Dihydro PGF-1a	metab_8239	HMDB0005076	B(ii)	-	357.265	6.7175	neg	M-H	C20H38O5	0	[illegible]
neg_2918	Ganosporeric acid A	metab_8240	HMDB0033022;LMST01010393	B(ii)	C16526	547.2364	6.7175	neg	M+Na-2H	C30H38O8	0	[illegible]
neg_2929	N-Oleoyl Alanine	metab_8252	LMFA08020124;HMDB0241953	B(ii)	-	352.286	6.7892	neg	M-H	C21H39NO3	0	[illegible]
neg_2935	PE(17:0/0:0)	metab_8259	LMGP02050030;HMDB0061691	B(i)	-	466.2946	6.8052	neg	M-H	C22H46NO7P	88.4	[illegible]
neg_2945	N-Stearoyl Proline	metab_8268	LMFA08020119;HMDB0241948	B(ii)	-	402.3018	6.837	neg	M+Na-2H	C23H43NO3	0	[illegible]

neg_2948	PL	metab_8270	HMDB0302594	B(ii)	C18210	579.2624	6.8449	neg	M+K-2H	C28H46O10	0
neg_2952	N-Stearoyl Threonine	metab_8273	HMDB0241950	B(ii)	-	384.3122	6.8769	neg	M-H	C22H43NO4	0
neg_2956	C-2 Ceramide	metab_8277	-	B(i)	-	340.286	6.8929	neg	M-H	C20H39NO3	37.4
neg_2958	(Z)-2-decenal	metab_8279	HMDB0303875	B(ii)	-	367.2856	6.9009	neg	2M+Hac-H	C10H18O	0
neg_2968	DG(18:3(9Z,12Z,15Z)/20:1(11Z)/0:0)	metab_8288	PW_C003362;HMDB0007311	B(ii)	C00165	681.4869	6.941	neg	M+K-2H	C41H72O5	0
neg_2978	(-)-Aspidospermine	metab_8298	HMDB0030361	B(ii)	C09042	391.1801	6.9649	neg	M+K-2H	C22H30N2O2	0
neg_2982	SM(d18:0/PGJ2)	metab_8303	HMDB0290468	B(ii)	-	763.5388	6.9729	neg	M-H2O-H	C43H79N2O8P	0
neg_2985	PC(18:1(11Z)/18:3(9Z,12Z,15Z))	metab_8306	PW_C004123;HMDB0008074;LMGP01012151	B(ii)	C00157	826.5633	6.9889	neg	M+FA-H	C44H80NO8P	0
neg_2987	PE(18:1(9Z)/18:2(9Z,12Z))	metab_8308	LMGP02010048;HMDB0009060;PW_C005108	B(ii)	C00350	740.5261	7.0049	neg	M-H	C41H76NO8P	0
neg_2989	LysoPE(P-18:0/0:0)	metab_8310	HMDB0240598	B(ii)	-	464.3154	7.0129	neg	M-H	C23H48NO6P	0
neg_2995	11-HydroxyNonadecanoylcarnitine	metab_8316	HMDB0241561	B(ii)	-	494.3261	7.0373	neg	M+K-2H	C26H51NO5	0
neg_3	Vanillic acid 4-beta-D-glucoside	metab_8321	HMDB0302469	B(ii)	-	329.0884	3.0046	neg	M-H, M+FA-H,	C14H18O9	0
neg_3013	15-hydroxyicosanoic acid	metab_8335	HMDB0061665	B(ii)	-	327.2908	7.1315	neg	M-H	C20H40O3	0
neg_3027	15-Hydroxynorandrostene-3,17-dione gluc	metab_8349	LMST05010033;HMDB0010353	B(ii)	C03033	445.1887	7.2081	neg	M-H2O-H	C24H32O9	0
neg_3033	TG(18:2(9Z,12Z)/18:2(9Z,12Z)/20:1(11Z))	metab_8356	HMDB0005473	B(ii)	C00422	945.7269	7.2341	neg	M+K-2H	C59H104O6	0
neg_3035	PE(18:3(9Z,12Z,15Z)/16:0)	metab_8358	PW_C005202;HMDB0009154;LMGP02010047	B(ii)	C00350	734.4796	7.2522	neg	M+Na-2H	C39H72NO8P	0
neg_306	Gluconic Acid	metab_8384	HMDB0000625;HMDB0303730;MJDBOTE0001669	B(i)	C00257	195.0507	0.6065	neg	M-H, M+K-2H	C6H12O7	96.6
neg_308	2'-Deoxyuridine	metab_8404	HMDB0000012	B(i)	C00526	227.0673	1.8356	neg	M-H, M+FA-H	C9H12N2O5	52.3
neg_3093	6-Aminoindazole	metab_8418	-	B(i)	-	132.0561	7.6184	neg	M-H	C7H7N3	52.7
neg_31	2,4-Dinitrophenylhydrazone	metab_8425	HMDB0302826	B(ii)	-	599.1361	2.6774	neg	M-H2O-H, M-I	C9H10N4O6	0
neg_3113	LysoPE(0:0/18:4(6Z,9Z,12Z,15Z))	metab_8437	HMDB0011480	B(ii)	-	518.2535	7.6765	neg	M+FA-H	C23H40NO7P	0
neg_3118	Lappaol F	metab_8441	HMDB0302071	B(ii)	-	749.2322	7.6765	neg	M+Cl	C40H42O12	0
neg_3123	4-Decan-4-Ylbenzenesulfonic Acid	metab_8447	-	B(i)	-	297.1531	7.6925	neg	M-H	C16H26O3S	85
neg_3132	Candicine	metab_8457	HMDB0303388	B(ii)	C10575	405.2764	7.7045	neg	2M+FA-H	C11H18NO+	0
neg_3141	9(S)-HpODE	metab_8466	HMDB0006940	B(i)	C14827	311.2231	7.7085	neg	M-H	C18H32O4	67.2
neg_319	(+)-marmesin	metab_8519	HMDB0030786;HMDB0303942	B(ii)	C09276	281.0604	1.9533	neg	M+Na-2H, M+	C14H14O4	0
neg_32	Gamma-Glutamylphenylalanine	metab_8530	HMDB0000594	B(ii)	-	293.1149	3.5199	neg	M-H2O-H, M-I	C14H18N2O5	0
neg_3200	Phosphoric acid	metab_8532	PW_C001441;HMDB0001429	B(i)	C00009	96.9689	8.1479	neg	M-H	H3O4P	43.8
neg_325	Gentisic acid	metab_8586	HMDB0059999;HMDB0000152	B(i)	C00628	153.0188	1.9693	neg	M-H, M+Na-2I	C7H6O4	80.5
neg_3301	(2R,4Z)-4-Hepten-2-ol	metab_8642	LMFA05000550;HMDB0041320	B(ii)	-	287.2229	7.7045	neg	2M+Hac-H	C7H14O	0
neg_3302	3-Isopropyl-2-methoxy-5-methylpyrazine	metab_8643	HMDB0029742	B(ii)	-	203.0598	7.7045	neg	M+K-2H	C9H14N2O	0
neg_3318	1-(9Z,12Z,15Z-octadecatrienoyl)-glycero-3	metab_8658	LMGP10050035;HMDB0062320	B(ii)	-	431.2211	7.6845	neg	M-H	C21H37O7P	0
neg_333	Azelaic Acid	metab_8671	HMDB0000784;LMFA01170054;MJDBOTE0000344	B(i)	C08261	187.0972	5.802	neg	M-H, M+Na-2I	C9H16O4	75.7
neg_3330	1-Nonadecanoyl-glycero-3-phosphoethan	metab_8672	HMDB0243971;LMGP02050028	B(ii)	-	1035.6665	7.6685	neg	2M+FA-H	C24H50NO7P	0
neg_3333	PI(18:1(9Z)/0:0)	metab_8675	HMDB0256522;LMGP06050005;HMDB0061693	B(ii)	-	597.3064	7.6685	neg	M-H	C27H51O12P	0
neg_3335	LysoPC(20:3(8Z,11Z,14Z)/0:0)	metab_8677	HMDB0010394	B(ii)	-	590.348	7.6685	neg	M+FA-H	C28H52NO7P	0
neg_3337	PS(18:1(9Z)/0:0)	metab_8679	HMDB0240603;LMGP03050001	B(i)	-	522.2849	7.6685	neg	M-H	C24H46NO9P	90
neg_3340	PI(16:2(9Z,12Z)/6 keto-PGF1alpha)	metab_8683	HMDB0276264	B(ii)	-	919.4778	7.6605	neg	M-H	C45H77O17P	0
neg_3357	Endomorphin-2	metab_8699	HMDB0005774	B(ii)	C15891	608.2239	7.6334	neg	M+K-2H	C32H37N5O5	0
neg_3359	Peridinin	metab_8701	HMDB0256341;LMPR01070007	B(ii)	-	667.3097	7.6254	neg	M+K-2H	C39H50O7	0
neg_3360	Boc-val-pro-arg-mca	metab_8703	HMDB0249335	B(ii)	-	608.32	7.6254	neg	M-H2O-H	C31H45N7O7	0
neg_3362	Trans-5,6-Dihydro-5,6-dihydroxy-7,12-din	metab_8705	HMDB0060519	B(ii)	C19607	625.2649	7.6104	neg	2M+FA-H	C20H18O2	0
neg_3366	Indinavir	metab_8709	HMDB0014369	B(ii)	C07051	634.3358	7.6003	neg	M+Na-2H	C36H47N5O4	0
neg_339	12-[Methyl-(4-nitro-2,1,3-benzoxadiazol-7	metab_8735	HMDB0255480	B(ii)	-	475.2921	5.9076	neg	M-H, M+FA-H	C25H40N4O5	0
neg_3400	PE(18:3(9Z,12Z,15Z)/18:2(9Z,12Z))	metab_8748	HMDB0009159;PW_C005207;LMGP02010721	B(ii)	C00350	736.4949	7.4737	neg	M-H	C41H72NO8P	0
neg_3413	Nalfurafine	metab_8761	HMDB0255431	B(ii)	-	513.1762	7.3607	neg	M+K-2H	C28H32N2O5	0
neg_342	5-Pentyltetrahydro-2-oxo-3-furancarboxyl	metab_8768	HMDB0030989	B(ii)	-	199.0972	5.9638	neg	M-H, M+Na-2I	C10H16O4	0
neg_3427	DG(8:0/a-13:0/0:0)	metab_8776	HMDB0092911;PW_C064412	B(ii)	C00165	887.686	7.2602	neg	2M+Hac-H	C24H46O5	0
neg_343	Sativic acid	metab_8779	LMFA02000148;HMDB0302836	B(ii)	-	347.2443	5.9835	neg	M-H, M+Na-2I	C18H36O6	0
neg_3439	(5E)-7-[3,5-Dihydroxy-2-(3-hydroxyoctyl)c	metab_8788	HMDB0241879	B(ii)	-	534.319	7.2081	neg	M+Cl	C27H49NO7	0
neg_3441	PE(20:0/0:0)	metab_8791	HMDB0011511;LMGP02050012	B(i)	-	508.3412	7.2021	neg	M-H	C25H52NO7P	33.7
neg_3473	N-Stearoyl Isoleucine	metab_8823	HMDB0241944	B(ii)	-	396.3489	7.0532	neg	M-H	C24H47NO3	0
neg_3475	SM(d18:1/16:0)	metab_8825	LMSP03010003;HMDB0010169	B(ii)	-	747.5682	7.0373	neg	M+FA-H	C39H79N2O6P	0
neg_3477	LysoPC(20:3(5Z,8Z,11Z)/0:0)	metab_8827	HMDB0010393	B(ii)	-	590.3479	7.0373	neg	M+FA-H	C28H52NO7P	0
neg_3480	Stearaldehyde	metab_8831	LMFA06000098;HMDB0002384	B(ii)	C01838	313.2752	7.0373	neg	M+FA-H	C18H36O	0
neg_3482	DG(20:5(7Z,9Z,11E,13E,17Z)-3OH(5,6,15)/i	metab_8833	HMDB0300527	B(ii)	-	791.57	7.0213	neg	M+FA-H	C45H78O8	0
neg_3483	7-[(1R,2R,5S)-5-Hydroxy-2-[(1E,3S)-3-Hy	metab_8834	HMDB0241888	B(ii)	-	532.3032	7.0213	neg	M+Cl	C27H47NO7	0
neg_3502	3-Methylbut-2-enoylcarnitine	metab_8855	HMDB0241659	B(ii)	-	485.2834	6.957	neg	2M-H	C12H21NO4	0
neg_3520	PE(18:0/0:0)	metab_8873	LMGP02050001	B(i)	-	480.3103	6.9009	neg	M-H	C23H48NO7P	91.1
neg_3523	N-Oleoyl Leucine	metab_8876	LMFA08020116;HMDB0241962	B(i)	-	394.3333	6.8929	neg	M-H	C24H45NO3	61.5
neg_3529	N-Palmitoyl Leucine	metab_8881	LMFA08020115;HMDB0241928	B(ii)	-	368.3172	6.8689	neg	M-H	C22H43NO3	0
neg_3532	N-Oleoyl Phenylalanine	metab_8885	HMDB0062336;LMFA08020092	B(i)	-	428.3177	6.8609	neg	M-H	C27H43NO3	40.5
neg_3536	1,3-Propanediol, 2,2-diethyl-, dicarbamate	metab_8888	HMDB0254691	B(ii)	-	217.1191	6.8449	neg	M-H	C9H18N2O4	0
neg_3537	Pantetheine	metab_8889	PW_C001887;HMDB0003426	B(ii)	C00831	277.1229	6.8449	neg	M-H	C11H22N2O4S	0
neg_3539	Pyropheophorbide a	metab_8891	HMDB0256993	B(ii)	C18064	533.2568	6.837	neg	M-H	C33H34N4O3	0
neg_3541	LysoPS(18:0/0:0)	metab_8894	HMDB0240606	B(ii)	-	546.2825	6.829	neg	M+Na-2H	C24H48NO9P	0
neg_3543	N-Palmitoyl Valine	metab_8896	LMFA08020120;HMDB0241935	B(ii)	-	354.3017	6.829	neg	M-H	C21H41NO3	0
neg_3547	Taurochenodesoxycholic acid	metab_8900	PW_C000759;HMDB0000951	B(ii)	C05465	520.2673	6.8052	neg	M+Na-2H	C26H45NO6S	0
neg_3549	PE(16:0/0:0)	metab_8902	LMGP02050002;HMDB0011503	B(i)	-	452.2791	6.8052	neg	M-H	C21H44NO7P	97.9
neg_3561	N-Stearoyl Glutamine	metab_8914	HMDB0241941;LMFA08020130	B(ii)	-	411.3236	6.7892	neg	M-H	C23H44N2O4	0
neg_3563	16-Hydroxyhexadecanoic acid	metab_8916	HMDB0006294	B(ii)	C18218	271.2281	6.7892	neg	M-H	C16H32O3	0
neg_3567	Gymnodimine	metab_8920	HMDB0041430	B(ii)	C20025	488.3151	6.7493	neg	M-H2O-H	C32H45NO4	0
neg_3568	N-Docosahexaenoyl Glutamine	metab_8921	LMFA08020129;HMDB0242012	B(ii)	-	971.6129	6.7413	neg	2M+Hac-H	C27H40N2O4	0
neg_3569	Cis-Hydroxy Perhexiline	metab_8922	HMDB0060644	B(ii)	-	338.2704	6.7413	neg	M+FA-H	C19H35NO	0
neg_357	DG(PGD2/2:0/0:0)	metab_8923	HMDB0296908	B(ii)	-	449.2555	6.2705	neg	M-H2O-H, M+	C25H40O8	0
neg_3571	N-Linoleoyl Phenylalanine	metab_8925	HMDB0241982	B(ii)	-	426.3023	6.7254	neg	M-H	C27H41NO3	0
neg_3572	Pinolenic Acid	metab_8926	HMDB0256562;LMFA01030344	B(i)	-	323.223	6.7254	neg	M+FA-H	C18H30O2	48
neg_3574	N-Palmitoyl Threonine	metab_8928	LMFA08020107;HMDB0241933	B(ii)	-	356.2809	6.7175	neg	M-H	C20H39NO4	0
neg_3575	N-Palmitoyl Glycine	metab_8929	HMDB0013034;LMFA08020079	B(i)	-	312.2547	6.7175	neg	M-H	C18H35NO3	43.5
neg_3577	Petrosaspongiolide m	metab_8931	HMDB0256357	B(ii)	-	979.5795	6.7016	neg	2M+Hac-H	C27H40O6	0
neg_3579	LysoPE(18:1(9Z)/0:0)	metab_8933	HMDB0011506	B(ii)	-	957.5973	6.6937	neg	2M-H	C23H46NO7P	0
neg_3583	3-Ketosphingosine	metab_8937	HMDB0245914;LMSP01010002	B(ii)	C06121	342.2651	6.6937	neg	M+FA-H	C18H35NO2	0
neg_3585	Dmg-mino	metab_8939	HMDB0251539;LMPK07000009	B(ii)	C12011	578.2203	6.6857	neg	M+Na-2H	C27H35N5O8	0
neg_3587	12-Hydroxydodecanoylcarnitine	metab_8941	HMDB0241208	B(ii)	-	340.2498	6.6857	neg	M-H2O-H	C19H37NO5	0
neg_3588	Eriojaposide B	metab_8942	HMDB0038029	B(ii)	C17889	515.2458	6.6777	neg	M-H	C25H40O11	0
neg_3589	N-Oleoyl Asparagine	metab_8943	LMFA08020137;HMDB0241955	B(ii)	-	395.2921	6.6777	neg	M-H	C22H40N2O4	0
neg_3590	2,4,12-Octadecatrienoic acid piperidide	metab_8945	HMDB0032034	B(ii)	-	390.3019	6.6777	neg	M+FA-H	C23H39NO	0
neg_3592	Myristoylglycine	metab_8947	HMDB0013250	B(ii)	-	284.2232	6.6777	neg	M-H	C16H31NO3	0
neg_3594	Beta-tocotrienol	metab_8949	HMDB0304597;LMPR02020055;HMDB0030554	B(ii)	C14154	409.308	6.6697	neg	M-H	C28H42O2	0
neg_3597	N-Docosahexaenoyl Proline	metab_8952	HMDB0242020	B(ii)	-	424.2863	6.6617	neg	M-H	C27H39NO3	0
neg_3598	22-Hydroxydocosanoic acid	metab_8953	HMDB0304091;HMDB0302963	B(ii)	C19623	393.2764	6.6617	neg	M+K-2H	C22H44O3	0
neg_36	Isoketocamphoric acid	metab_8955	HMDB0301824	B(ii)	-	215.0923	4.41	neg	M-H, M+Na-2I	C10H16O5	0
neg_3604	PS(20:0/20:0)	metab_8959	PW_C060933;LMGP03010863;HMDB0112523	B(ii)	C02737	884.5765	6.6457	neg	M+K-2H	C46H90NO10P	0
neg_3605	(1R)-5-[2-[(1S,7As)-1-[(1S)-1-(3-hydroxy-	metab_8960	HMDB0242365	B(ii)	-	399.287	6.6457	neg	M-H2O-H	C26H42O4	0
neg_3609	2-Hydroxymyristic Acid	metab_8962	HMDB0002261;LMFA01050484	B(i)	-	243.1965	6.6377	neg	M-H	C14H28O3	63.2
neg_3618	1-Oleoylglycerophosphoserine	metab_8970	HMDB0061694	B(ii)	-	544.267	6.5864	neg	M+Na-2H	C24H46NO9P	0
neg_3621	PE(18:2/0:0)	metab_8973	-	B(i)	-	476.2789	6.5724	neg	M-H	C23H44NO7P	97.9
neg_3623	5,9,11-trihydroxyprosta-6E,14Z-dien-1-oat	metab_8975	HMDB0062413	B(ii)	-	576.2047	6.5489	neg	M+K-2H	C30H37NO8	0
neg_3629	(-)-3-(4-(2-(Phenoxazin-10-yl)ethoxy)phe	metab_8981	HMDB0242198	B(ii)	-	464.1752	6.5175	neg	M+FA-H	C25H25NO5	0
neg_3630	N-Palmitoyl Serine	metab_8983	HMDB0241932;LMFA08020101	B(ii)	-	364.2497	6.5175	neg	M+Na-2H	C19H37NO4	0
neg_3633	PE(16:1/0:0)	metab_8986	-	B(i)	-	450.2634	6.5095	neg	M-H	C21H42NO7P	96.9
neg_3634	13,16,19-Docosatrienoic acid	metab_8987	HMDB0002823;HMDB0244522;LMFA01030407	B(ii)	C16534	355.2603	6.502	neg	M+Na-2H	C22H38O2	0
neg_3636	(23R)-1alpha-Hydroxy-25,27-didehydrovita	metab_8988	HMDB0242503;LMST03020600	B(ii)	-	447.251	6.4945	neg	M+Na-2H	C27H38O4	0
neg_3637	N-Eicosapentaenoyl Serine	metab_8989	HMDB0242079	B(ii)	-	388.25	6.4945	neg	M-H	C23H35NO4	0
neg_3640	Lysopc(18:3)	metab_8993	HMDB0010387;HMDB0010388	B(i)	C04230	552.2879	6.4865	neg	M+Cl	C26H48NO7P	36.7
neg_3645	Homophytanic acid	metab_8998	HMDB0002337;LMPR0104010038	B(ii)	-	361.2864	6.4705	neg	M+Cl	C21H42O2	0
neg_3647	PS(22:1(13Z)/18:1(11Z))	metab_9000	HMDB0112742;PW_C061166	B(ii)	C02737	880.5486	6.4625	neg	M+K-2H	C46H86NO10P	0
neg_365	Trihydroxystearic acid	metab_9003	HMDB0302290	B(ii)	-	331.2493	6.3585	neg	M-H2O-H, M-I	C18H36O5	0
neg_3650	LysoPC(18:3(6Z,9Z,12Z)/0:0)	metab_9004	HMDB0010387	B(ii)	C04230	562.3164	6.4545	neg	M+FA-H	C26H48NO7P	0
neg_3656	PI(6 keto-PGF1alpha/22:3(10Z,13Z,16Z))	metab_9010	HMDB0277824	B(ii)	-	1015.582	6.4305	neg	M-H	C52H89O17P	0
neg_3658	PC(17:2(9Z,12Z)/0:0)	metab_9012	HMDB0256157;LMGP01050127	B(ii)	-	526.2947	6.4225	neg	M+Na-2H	C25H48NO7P	0
neg_366	PS(15:0/22:2(13Z,16Z))	metab_9014	HMDB0116749;LMGP03010158;PW_C060734	B(ii)	C02737	800.5475	6.3505	neg	M-H, M+Na-2I	C43H80NO10P	0
neg_3660	Deltaline	metab_9015	HMDB0250984	B(ii)	C08679	542.2512	6.4145	neg	M+Cl	C27H41NO8	0
neg_3661	MG(0:0/20:3(6,8,11)-OH(5)/0:0)	metab_9016	HMDB0260564	B(ii)	-	395.2802	6.4145	neg	M-H	C23H40O5	0
neg_3662	Retinyl ester	metab_9017	PW_C001935;HMDB0003598	B(ii)	C02075	301.2175	6.4145	neg	M-H	C20H30O2	0
neg_3666	N-Myristoyl Serine	metab_9020	HMDB0242059	B(ii)	-	336.2183	6.4065	neg	M+Na-2H	C17H33NO4	0
neg_3674	(+)-gamma-Hydroxy-L-homoarginine	metab_9028	HMDB0301838	B(ii)	C08286	467.2588	6.3825	neg	2M+Hac-H	C7H16N4O3	0
neg_3679	LysoPC(18:4(6Z,9Z,12Z,15Z)/0:0)	metab_9030	HMDB0010389	B(ii)	-	560.301	6.3745	neg	M+FA-H	C26H46NO7P	0
neg_3680	PE(18:4/0:0)	metab_9032	-	B(i)	-	472.2481	6.3745	neg	M-H	C23H40NO7P	42.3
neg_3685	N-Lauroyl Proline	metab_9037	HMDB0242039	B(ii)	-	342.2287	6.3665	neg	M+FA-H	C17H31NO3	0
neg_369	DG(20:4(8Z,11Z,14Z,17Z)/24:1(15Z)/0:0)	metab_9042	PW_C003609;HMDB0007558	B(ii)	C00165	747.5676	6.3425	neg	M+Na-2H, M+	C47H82O5	0
neg_3693	Pectenotoxin 1	metab_9046	HMDB0033490	B(ii)	C16871	919.4777	6.3505	neg	M+FA-H	C47H70O15	0
neg_3694	PE-NMe(18:2(9Z,12Z)/20:3(5Z,8Z,11Z))	metab_9047	HMDB0113179;PW_C059302	B(ii)	C01241	824.5475	6.3505	neg	M+FA-H	C44H78NO8P	0
neg_3695	DG(22:0/20:5(5Z,8Z,11Z,14Z,17Z)/0:0)	metab_9048	HMDB0007607;PW_C003658	B(ii)	C00165	733.5514	6.3505	neg	M+Cl	C45H78O5	0
neg_3698	Tuberose lactone	metab_9051	HMDB0303314	B(ii)	-	433.2589	6.3505	neg	2M+FA-H	C12H18O2	0
neg_3699	9(S)-HpOTrE	metab_9052	-	B(i)	C16321	309.2074	6.3505	neg	M-H	C18H30O4	75.7
neg_3706	L-a-Lysophosphatidylserine	metab_9060	-	B(i)	-	524.3006	6.3425	neg	M-H	C24H48NO9P	81.2
neg_3708	2-Geranyl-2',3,4,4'-tetrahydroxydihydrocha	metab_9062	HMDB0032005	B(ii)	-	431.1844	6.3425	neg	M+Na-2H	C25H30O5	0
neg_3711	7-[(1R,2R,3R)-3-Hydroxy-2-[(1E,3S)-3-Hy	metab_9066	HMDB0241871	B(ii)	-	534.2849	6.3345	neg	M+K-2H	C27H47NO7	0
neg_3713	Pangamic acid	metab_9068	HMDB0029949	B(ii)	-	481.2736	6.3265	neg	M+FA-H	C20H40N2O8	0
neg_3717	LysoPG(16:0/0:0)	metab_9072	HMDB0240601	B(ii)	-	505.2582	6.3185	neg	M+Na-2H	C22H45O9P	0
neg_3719	3a,12b-Dihydroxy-5b-cholanoic acid	metab_9074	HMDB0000411	B(ii)	C17646	413.2665	6.3105	neg	M+Na-2H	C24H40O4	0
neg_372	Leukotriene B4 ethanolamide	metab_9075	HMDB0002304	B(ii)	-	378.2651	6.5568	neg	M-H2O-H, M-I	C22H37NO4	0
neg_3722	Tetradecanedioic acid	metab_9078	LMFA01170018;HMDB0000872	B(i)	C11002	257.1759	6.3105	neg	M-H	C14H26O4	88
neg_3725	(11E)-Octadec-11-enedioylcarnitine	metab_9081	HMDB0241538	B(ii)	-	492.2741	6.3025	neg	M+K-2H	C25H45NO6	0
neg_3727	12S-HHT	metab_9083	HMDB0012535	B(ii)	C20388	325.2022	6.3025	neg	M+FA-H	C17H28O3	0
neg_3728	Isokobusone	metab_9084	HMDB0036791	B(ii)	C16977	267.1603	6.3025	neg	M+FA-H	C14H22O2	0
neg_3732	Palmitic Acid ethyl ester	metab_9089	HMDB0029811	B(i)	-	283.2644	6.2865	neg	M-H	C18H36O2	39.6
neg_3736	Embelin	metab_9093	HMDB0251767;LMPK15050006	B(ii)	C10342	293.1762	6.2785	neg	M-H	C17H26O4	0
neg_3737	PA(PGF1alpha/2:0)	metab_9094	HMDB0266580	B(ii)	-	573.2458	6.2705	neg	M+Na-2H	C25H45O11P	0
neg_3745	Dodecyl Hydrogen Sulfate	metab_9103	-	B(i)	-	265.1482	6.2545	neg	M-H	C12H26O4S	61.6
neg_3746	3-Hexen-1-ol	metab_9104	HMDB0030003;HMDB0302313;HMDB0302264	B(ii)	C08492	259.1915	6.2545	neg	2M+Hac-H	C6H12O	0
neg_3747	Murnac-tripeptide	metab_9105	HMDB0254953	B(ii)	-	685.2629	6.2465	neg	M+Na-2H	C26H44N6O14	0
neg_3753	Epothilone D	metab_9110	HMDB0251876;LMPK04000001	B(ii)	C12039	490.2586	6.2385	neg	M-H	C27H41NO5S	0
neg_3756	(Z)-Farnesol	metab_9113	HMDB0059849	B(ii)	C03220	267.1967	6.2385	neg	M+FA-H	C15H26O	0
neg_3768	Ethyl Myristate	metab_9126	HMDB0034153	B(i)	-	255.233	6.2145	neg	M-H	C16H32O2	58
neg_3777	(6Z,9Z)-Octadeca-6,9-dienoylcarnitine	metab_9136	HMDB0241540	B(ii)	-	468.334	6.191	neg	M+FA-H	C25H45NO4	0
neg_3780	Jasmonic acid	metab_9140	HMDB0032797;LMFA02020001	B(ii)	C08491	209.118	6.191	neg	M-H	C12H18O3	0
neg_3792	LysoPS(16:0/0:0)	metab_9153	HMDB0240605	B(ii)	-	518.2534	6.162	neg	M+Na-2H	C22H44NO9P	0
neg_3794	LysoPA(P-16:0/0:0)	metab_9155	HMDB0011154	B(ii)	C15646	393.2401	6.162	neg	M-H	C19H39O6P	0
neg_3795	1-Methyl-3-(2-oxopropylidene)indol-2-one	metab_9156	HMDB0258628	B(ii)	-	246.0773	6.162	neg	M+FA-H	C12H11NO2	0
neg_3796	Capsicoside A1	metab_9157	HMDB0303762	B(ii)	-	639.377	6.154	neg	M+FA-H	C33H54O9	0
neg_3798	9,10,13-Trihydroxystearic acid	metab_9159	HMDB0030935	B(ii)	-	331.2494	6.154	neg	M-H	C18H36O5	0
neg_3800	Diethyl Phthalate	metab_9163	HMDB0094660	B(i)	C14175	221.0817	6.146	neg	M-H	C12H14O4	63.2
neg_3825	DG(8:0/12:0/0:0)	metab_9190	PW_C062170;HMDB0092903	B(ii)	C00165	437.2666	6.0868	neg	M+K-2H	C23H44O5	0
neg_3831	Ceranapril	metab_9197	HMDB0249824	B(ii)	-	461.1839	6.0662	neg	M+Na-2H	C21H33N2O6P	0
neg_3838	7-Epi-12-hydroxyjasmonic acid	metab_9204	HMDB0303749	B(ii)	-	225.1131	6.05	neg	M-H	C12H18O4	0
neg_3853	O-Desmethylcarvedilol	metab_9220	HMDB0013949	B(ii)	-	427.1437	6.0207	neg	M+Cl	C23H24N2O4	0
neg_3855	9,10,16-trihydroxy palmitic acid	metab_9222	LMFA01050101	B(i)	-	303.218	6.0207	neg	M-H	C16H32O5	41.2
neg_3857	Capsidiol	metab_9224	HMDB0002352;LMPR0103250002	B(ii)	C09627	273.1248	6.0152	neg	M+K-2H	C15H24O2	0
neg_3865	Isoflupredone acetate	metab_9233	HMDB0253640	B(ii)	C14636	455.1602	5.994	neg	M+Cl	C23H29FO6	0
neg_3869	Pregnanetriol 3a-O-b-D-glucuronide	metab_9237	HMDB0247360	B(ii)	-	493.2825	5.9885	neg	M-H2O-H	C27H44O9	0
neg_3870	Menthyl pyrrolidone carboxylate	metab_9238	HMDB0032368	B(ii)	C03962	317.1973	5.9885	neg	M-H	C16H30O6	0
neg_3871	Germacrone	metab_9239	HMDB0252704	B(ii)	C16966	255.1142	5.9885	neg	M+K-2H	C15H22O	0
neg_3883	Tricetin	metab_9252	HMDB0029620;LMPK12110847	B(i)	C10192	301.0357	5.9739	neg	M-H	C15H10O7	44.3
neg_3885	Heptylmalonic acid	metab_9254	HMDB0059719	B(ii)	-	463.2561	5.9638	neg	2M+Hac-H	C10H18O4	0
neg_3892	N-Benzyl-9-(tetrahydro-2H-pyran-2-YL)a	metab_9262	HMDB0245639	B(ii)	-	354.1564	5.9532	neg	M+FA-H	C17H19N5O	0
neg_39	(1R)-Glutathionyl-(2R)-hydroxy-1,2-dihydr	metab_9270	HMDB0060299	B(ii)	C14793	450.1353	3.5169	neg	M-H2O-H, M-I	C20H25N3O7S	0
neg_3900	Lavoltidine	metab_9272	HMDB0254182	B(ii)	C11805	358.2242	5.9366	neg	M-H	C19H29N5O2	0
neg_3902	Diosgenin	metab_9274	HMDB0242671;LMST01080037	B(ii)	C08898	459.3087	5.93	neg	M+FA-H	C27H42O3	0
neg_3903	Abscisic Acid	metab_9275	HMDB0243505;HMDB0036093;HMDB0035140;MJDB	B(i)	C06082	263.1291	5.93	neg	M-H	C15H20O4	84.3
neg_3908	Pentisomide	metab_9280	HMDB0256288	B(ii)	-	356.2085	5.9193	neg	M+K-2H	C19H33N3O	0
neg_3909	Undecylenic acid	metab_9281	HMDB0033724;LMFA01030036	B(ii)	C13910	229.1444	5.9193	neg	M+FA-H	C11H20O2	0
neg_3922	Senkyunolide	metab_9296	HMDB0258235	B(ii)	C17853	237.1133	5.8874	neg	M+FA-H	C12H16O2	0
neg_3934	1-Naphthyl sulfate	metab_9309	HMDB0243963	B(ii)	-	223.007	5.8625	neg	M-H	C10H8O4S	0
neg_3966	Diacetoxyscirpenol	metab_9342	HMDB0035104	B(ii)	C09662	401.1361	5.7368	neg	M+Cl	C19H26O7	0

[illegible]

neg_3970	Ethyl 2-hydroxy-3-(3-indolyl)propanoate gl	metab_9347	HMDB0034873	B(ii)	-	394.1515	5.716	neg	M-H	C19H25NO8	0	58.1	1.93897587	143884-93-9	0.004672707	3.689	3.508	3.524	3.509	3.528	3.426	4.747	4.756	4.788	4.714	4.74	4.764	5.274	5.326	5.302	5.332	5.275	5.298	5.498	5.518	5.507	5.507	5.513	5.479	6.192	6.194	6.214	6.194	6.194	6.201	5.501	5.497	5.499
neg_3976	3-[[5-Methyl-2-(1-methylethyl)cyclohexyl]c	metab_9353	HMDB0036133	B(ii)	-	275.1868	5.6911	neg	M+FA-H	C13H26O3	0	38.5	1.70744846	87061-04-9	0.010354216	5.232	5.188	5.187	5.176	5.198	5.181	4.626	4.673	4.685	4.684	4.692	4.698	4.79	4.831	4.824	4.824	4.827	4.828	4.811	4.813	4.81	4.814	4.811	4.813	4.716	4.713	4.71	4.71	4.708	4.703	4.744	4.735	4.738
neg_398	PS(22:2(13Z,16Z)/15:0)	metab_9357	LMGP03010760;HMDB0112763;PW_C061191	B(ii)	C02737	800.5481	7.1951	neg	M-H, M+Na-2	C43H80NO10P	0	41.4	4.17860383	-	0.089987111	6.523	6.258	5.714	5.721	5.713	5.709	5.627	6.789	7.014	7.081	7.119	7.115	7.008	7.208	7.389	7.444	7.439	7.442	7.363	7.33	7.377	7.389	7.398	7.403	7.198	7.11	7.035	6.999	7.006	6.983	6.802	6.871	6.872
neg_3994	3-Hydroxymelatonin	metab_9373	HMDB0060742	B(ii)	-	499.2558	5.5523	neg	2M-H	C13H18N2O3	0	58	-0.8966204	-	0.00916458	3.035	3.06	2.98	3.095	3.05	3.004	5.276	5.308	5.322	5.299	5.314	5.314	5.943	6	6	6.009	5.993	6.002	5.919	5.903	5.908	5.905	5.907	5.906	6.168	6.179	6.216	6.189	6.185	6.185	5.798	5.792	5.799
neg_4003	Azimsulfuron	metab_9385	HMDB0033145	B(ii)	C18438	405.087	5.5168	neg	M-H2O-H	C13H16N10O5S	0	62.9	5.23187919	120162-55-2	0.002579412	7.447	7.41	7.414	7.416	7.395	7.408	4.873	4.892	4.933	4.901	4.875	4.909	4.995	5.021	5.046	5.011	5.023	5.02	5.072	5.136	5.093	5.097	5.098	5.052	4.426	4.371	4.434	4.352	4.473	4.467	6.972	6.972	6.97
neg_4012	4,1-Benzoxazepine	metab_9393	HMDB0246595	B(ii)	-	190.0506	5.4885	neg	M+FA-H	C9H7NO	0	54.9	-2.5905412	-	0.002101322	6.005	5.958	5.961	5.962	5.938	5.946	6.621	6.655	6.664	6.685	6.672	6.67	6.702	6.74	6.749	6.763	6.754	6.75	6.752	6.757	6.753	6.754	6.749	6.745	5.705	5.687	5.708	5.714	5.694	5.712	6.469	6.471	6.469
neg_4023	Lignan	metab_9405	HMDB0031452;HMDB0254088	B(ii)	C10871	439.1732	5.4369	neg	M-H2O-H	C25H30O8	0	35.8	-6.5506445	-	0.013898549	3.524	2.84	2.823	2.83	3.086	3.065	5.765	5.811	5.801	5.829	5.829	5.812	5.945	5.973	5.998	5.998	5.99	5.973	5.873	5.897	5.862	5.891	5.868	5.854	5.987	5.967	5.973	5.968	5.959	5.965	5.393	5.404	5.394
neg_4027	Quercetin 3-galactoside	metab_9409	HMDB0030775	B(ii)	C10073	463.0895	5.4257	neg	M-H	C21H20O12	0	73.1	2.69427419	482-36-0	0.011008151	3.536	2.892	3.219	3.533	2.891	2.887	6.177	6.218	6.221	6.258	6.241	6.235	5.454	5.522	5.517	5.522	5.532	5.517	5.608	5.618	5.582	5.62	5.591	5.602	4.853	4.824	4.827	4.834	4.825	4.828	5.592	5.601	5.593
neg_4036	Nintedanib	metab_9419	HMDB0255615	B(ii)	-	576.2007	5.3787	neg	M+K-2H	C31H33N5O4	0	34.7	-2.1741241	-	0.007921439	5.735	5.713	5.714	5.74	5.704	5.701	6.581	6.609	6.621	6.644	6.63	6.628	6.66	6.703	6.711	6.713	6.712	6.716	6.728	6.732	6.726	6.729	6.712	6.72	5.243	5.205	5.222	5.231	5.222	5.212	6.388	6.394	6.389
neg_4037	Trovafloxacin	metab_9420	HMDB0014823	B(ii)	C07664	453.0581	5.3787	neg	M+K-2H	C20H15F3N4O3	0	68.3	-0.3187067	147059-72-1	0.008859691	2.863	2.833	2.833	2.841	2.832	2.829	5.097	5.169	5.156	5.204	5.164	5.181	6.232	6.287	6.29	6.289	6.29	6.286	6.183	6.194	6.191	6.2	6.181	6.189	5.351	5.355	5.355	5.357	5.342	5.35	5.657	5.656	5.65
neg_4045	Citramalic Acid	metab_9429	HMDB0000426	B(i)	C00815	147.0295	5.3499	neg	M-H	C5H8O5	61.1	0	-2.9332492	597-44-4;230	0.013787224	5.245	5.191	5.212	5.214	5.209	5.208	4.654	4.692	4.69	4.726	4.703	4.679	4.728	4.765	4.779	4.826	4.74	4.727	4.756	4.739	4.777	4.771	4.783	4.743	4.345	4.295	4.342	4.288	4.31	4.293	4.93	4.937	4.926
neg_4054	Penitrem B	metab_9439	HMDB0242736;HMDB0256239	B(ii)	C20731	604.3009	5.3042	neg	M+Na-2H	C37H45NO5	0	67.9	-6.1451079	11076-67-8	0.01301549	6.473	6.44	6.456	6.451	6.453	6.466	2.889	2.936	3.431	3.441	2.954	3.098	3.052	3.094	3.087	3.087	3.089	3.091	3.074	3.075	3.073	3.076	3.074	3.075	2.978	2.976	2.972	2.973	2.971	2.966	6.248	6.258	6.249
neg_4059	6b-Angeloyl-3b,8b,9b-trihydroxy-7(11)-er	metab_9444	HMDB0041228	B(ii)	-	425.1829	5.2707	neg	M+FA-H	C20H28O7	0	51.9	3.17935416	171422-89-2	0.003810318	5.5	5.47	5.47	5.477	5.469	5.466	5.446	5.46	5.443	5.483	5.463	5.472	5.735	5.803	5.794	5.816	5.8	5.796	5.714	5.727	5.73	5.733	5.729	5.724	5.94	5.936	5.926	5.923	5.92	5.929	5.586	5.585	5.583
neg_4061	Methionyl-Aspartate	metab_9447	HMDB0028969	B(ii)	-	309.0773	5.2671	neg	M+FA-H	C9H16N2O5S	0	70.2	4.34009827	-	0.020165469	2.645	2.615	2.615	2.622	2.614	2.611	3.371	3.181	2.588	2.737	2.981	3	5.256	5.317	5.337	5.329	5.301	5.312	5.744	5.738	5.747	5.74	5.745	5.724	2.618	2.615	2.612	2.612	2.61	2.605	5.177	5.161	5.162
neg_4066	N-lactoyl-Tryptophan	metab_9452	HMDB0062178	B(ii)	-	275.1042	5.2463	neg	M-H	C14H16N2O4	0	40.9	1.83519018	-	0.010953993	2.49	2.46	2.46	2.507	2.459	2.456	4.884	4.864	4.756	4.82	4.839	4.815	5.364	5.373	5.452	5.493	5.413	5.243	5.22	5.089	5.178	5.138	5.166	5.102	4.6	4.727	4.681	4.797	4.669	4.756	4.766	4.774	4.766
neg_4069	Taraxinic acid glucosyl ester	metab_9455	HMDB0035249	B(ii)	-	469.1729	5.222	neg	M+FA-H	C21H28O9	0	67.6	3.18654566	75911-14-7	0.015394943	5.617	5.559	5.559	5.574	5.559	5.561	6.095	6.131	6.141	6.169	6.149	6.15	6.377	6.426	6.436	6.436	6.425	6.432	6.379	6.38	6.369	6.374	6.373	6.374	6.588	6.592	6.58	6.588	6.579	6.583	6.291	6.298	6.285

neg_4072	Glu-val-phe	metab_9459	-	B(i)	-	374.1728	5.2023	neg	M-H2O-H	C19H27N3O6	36.1	0	1.69619268	-	0.013575237	6.553	6.516	6.524	6.52	6.531	6.524	5.564	5.601	5.62	5.616	5.616	5.608	4.993	5.041	5.041	5.056	5.056	5.06	5.497	5.489	5.494	5.475	5.501	5.494	2.593	2.591	2.588	2.588	2.586	2.581	6.168	6.167	6.157
neg_4077	7-Methylinosine	metab_9463	HMDB0003950	B(ii)	C05276	304.0808	5.1746	neg	M+Na-2H	C11H15N4O5+	0	47.9	6.49932548	20245-33-4	0.030910712	4.958	4.928	4.928	4.936	4.927	4.924	5.223	5.221	5.246	5.26	5.241	5.277	5.724	5.758	5.725	5.773	5.749	5.737	5.63	5.643	5.626	5.634	5.627	5.637	4.946	4.928	4.943	4.956	4.938	4.933	5.209	5.209	5.185
neg_4084	3,4-Methylenedioxybenzaldehyde	metab_9471	HMDB0032612	B(ii)	C10812	149.0239	5.1429	neg	M-H	C8H6O3	0	35.5	-3.7239448	120-57-0	0.00726727	3.344	3.46	3.604	3.404	3.528	3.557	5.116	5.108	5.16	5.18	5.1	5.122	4.829	4.969	4.789	4.858	4.893	4.897	4.912	4.938	4.849	4.96	5.004	4.942	4.557	4.54	4.441	4.472	4.505	4.523	4.718	4.724	4.718
neg_4089	1-(3-Methyl-2-butenoyl)-6-apiosylglucose	metab_9475	HMDB0039952	B(ii)	-	375.1317	5.1197	neg	M-H2O-H	C16H26O11	0	74.1	5.20975911	467242-32-6	0.003109821	4.679	4.436	4.296	4.2	4.113	4.149	5.603	5.636	5.776	5.866	5.818	5.749	6.006	6.01	5.979	6.004	6.007	6.045	6.112	6.001	6.132	6.149	6.038	6.106	5.997	6.005	6.026	6.079	6.013	6.017	5.707	5.707	5.705
neg_410	PE(P-18:0/PGE1)	metab_9488	HMDB0285366	B(ii)	-	800.548	7.6529	neg	M-H, M+Na-2H	C43H80NO10P	0	85.6	4.04635211	-	0.037866673	6.275	6.124	5.303	5.311	5.302	5.299	5.216	6.5	6.764	6.806	6.817	6.796	6.515	6.916	7.031	7.057	7.057	7.083	6.995	7.015	7.018	7.059	7.04	7.069	6.92	6.852	6.732	6.755	6.73	6.761	6.543	6.571	6.572
neg_4110	Salsoline-1-carboxylate	metab_9500	HMDB0013067	B(ii)	-	236.0929	5.0163	neg	M-H	C12H15NO4	0	61.7	0.39059603	31758-50-6	0.014507436	4.871	4.819	4.805	4.824	4.801	4.83	6.257	6.264	6.284	6.299	6.28	6.306	6.754	6.787	6.766	6.794	6.78	6.774	6.665	6.662	6.65	6.665	6.655	6.659	6.012	6.009	6.015	6.029	6.015	6.009	6.338	6.337	6.327
neg_4114	Protoapigenone	metab_9504	HMDB0256871	B(ii)	-	331.0441	4.9936	neg	M+FA-H	C15H10O6	0	43.7	-6.6093534	-	0.005319562	5.329	5.253	5.207	5.262	5.194	5.242	2.248	2.295	2.307	2.786	2.313	2.319	2.411	2.649	2.548	2.446	2.448	2.474	2.432	2.434	2.432	2.435	2.432	2.434	2.337	2.335	2.331	2.331	2.33	2.325	4.958	4.954	4.955
neg_412	LysoPI(0:0/18:0)	metab_9510	HMDB0061704	B(ii)	-	599.3218	7.6685	neg	M-H, M+Na-2H	C27H53O12P	0	86.1	2.73254014	1913287-87-2	0.059996008	6.133	6.282	6.345	6.263	6.271	6.262	6.301	6.303	6.677	6.807	6.821	6.772	6.558	6.485	6.662	6.736	6.559	6.562	6.623	6.489	6.542	6.602	6.589	6.61	6.507	6.561	6.607	6.48	6.524	6.491	6.706	6.749	6.704
neg_4120	6-Methoxymellein	metab_9511	HMDB0038510	B(ii)	C02381	207.0659	4.9763	neg	M-H	C11H12O4	0	49.5	-2.0261753	13410-15-6	0.00516205	3.325	2.361	2.441	2.54	2.793	2.75	4.974	4.981	4.995	4.985	5.013	5.006	5.324	5.369	5.385	5.384	5.376	5.372	5.36	5.339	5.322	5.346	5.338	5.331	5.673	5.686	5.713	5.694	5.684	5.679	5.224	5.22	5.221
neg_4121	Suberic Acid	metab_9512	HMDB0000893;LMFA01170001;MJDBOTE0000630	B(i)	C08278	173.0814	4.9733	neg	M-H	C8H14O4	77.2	0	-2.8701341	505-48-6	0.010525169	4.978	4.919	4.892	4.911	4.885	4.892	4.744	4.735	4.785	4.795	4.76	4.791	4.793	4.802	4.873	4.864	4.858	4.846	4.867	4.791	4.776	4.812	4.826	4.817	5.33	5.343	5.357	5.354	5.329	5.31	5.095	5.086	5.088
neg_4123	1-(3-Ethyl-2,4-dihydroxy-6-methoxypheny	metab_9514	-	B(i)	-	237.1132	4.9677	neg	M-H	C13H18O4	75.1	0	-0.031508	-	0.008198446	2.521	2.492	2.491	2.499	2.531	2.487	4.698	4.666	4.68	4.718	4.714	4.679	5.016	5.004	5.014	5.155	5.071	5.031	5.095	5.053	5.041	4.999	5.046	5.094	5.374	5.356	5.364	5.413	5.378	5.35	4.975	4.969	4.976
neg_4125	Phaseic acid	metab_9516	HMDB0302844	B(ii)	C09707	279.124	4.953	neg	M-H	C15H20O5	0	44.8	0.76650933	24394-14-7	0.006942825	4.831	4.764	4.81	4.798	4.758	4.815	4.423	4.471	4.483	4.481	4.489	4.495	4.587	4.628	4.622	4.622	4.624	4.625	4.609	4.61	4.608	4.611	4.609	4.61	4.513	4.51	4.507	4.507	4.505	4.5	4.539	4.532	4.536
neg_4135	6-[3-(carboxymethyl)phenoxy]-3,4,5-trihyc	metab_9527	HMDB0125161	B(ii)	-	309.0621	4.9025	neg	M-H2O-H	C14H16O9	0	81.7	1.44936274	-	0.008428008	7.374	7.336	7.316	7.314	7.293	7.306	4.263	4.095	3.878	4.111	3.797	3.865	3.102	2.53	2.523	2.523	2.526	2.527	2.51	2.725	2.509	2.981	2.646	2.512	2.682	3.383	2.96	3.023	3.064	3.129	7.032	7.025	7.027
neg_4137	Dihydrocitrinone	metab_9528	HMDB0251297	B(ii)	-	287.054	4.896	neg	M+Na-2H	C13H14O6	0	47.2	0.93809576	-	0.025499509	5.041	5.066	5.047	4.989	4.981	4.97	2.856	2.583	0.5931	0.5919	2.563	0.6023	0.6729	0.7059	0.7004	0.7007	2.68	0.7035	2.649	0.6913	0.6894	2.603	0.6901	0.6914	2.606	0.6139	2.937	2.559	2.7	2.534	4.833	4.813	4.814
neg_4140	2-Methoxy-4-vinylphenol	metab_9532	HMDB0013744	B(i)	C17883	149.0602	4.8929	neg	M-H	C9H10O2	91.9	0	-4.1217836	7786-61-0	0.013660933	5.284	5.266	5.245	5.238	5.208	5.228	1.019	1.062	1.073	2.168	1.079	1.084	2.231	1.208	1.201	1.202	1.204	1.205	1.189	1.191	2.239	1.191	1.189	1.191	1.1	1.098	1.095	1.095	1.094	1.089	4.918	4.907	4.909
neg_4143	Cartap	metab_9535	HMDB0249690	B(ii)	C11080	533.1315	4.8702	neg	2M+Hac-H	C7H15N3O2S2	0	30.6	-7.2768356	15263-53-3	0.010002928	5.549	5.532	5.521	5.515	5.457	5.484	3.476	2.918	3.039	3.537	2.833	2.839	4.661	4.555	4.741	4.664	4.705	4.719	4.363	4.078	4.18	4.134	4.321	4.365	4.271	3.777	4.31	4.32	4.249	4.318	5.18	5.172	5.173
neg_4149	Methyl (3x,10R)-dihydroxy-11-dodecene-6	metab_9541	HMDB0040896	B(ii)	-	443.157	4.851	neg	M+FA-H	C19H26O9	0	73	2.86374778	152141-43-0	0.0064171	3.01	3.446	3.121	2.988	3.266	2.976	6.733	6.754	6.761	6.746	6.762	6.769	7.168	7.192	7.207	7.212	7.209	7.201	7.114	7.105	7.084	7.104	7.108	7.095	7.362	7.371	7.395	7.379	7.366	7.366	6.971	6.966	6.967
neg_4150	Actarit	metab_9543	HMDB0247976	B(ii)	-	192.0662	4.851	neg	M-H	C10H11NO3	0	42.8	-2.1520894	-	0.004720412	5.268	5.217	5.18	5.187	5.158	5.165	5.835	5.867	5.884	5.876	5.879	5.904	6.072	6.108	6.124	6.123	6.118	6.128	6.069	6.069	6.046	6.061	6.059	6.061	5.606	5.6	5.603	5.596	5.581	5.576	5.767	5.766	5.77
neg_4151	Tranexamic Acid	metab_9544	HMDB0014447;PW_C008836;MJDBOTE0000780	B(ii)	C12535	202.1081	4.851	neg	M+FA-H	C8H15NO2	0	71.4	-2.1653877	1197-18-8	0.011264145	5.047	5.003	4.971	4.995	4.973	4.982	6.513	6.527	6.536	6.526	6.54	6.549	6.891	6.921	6.923	6.933	6.929	6.92	6.809	6.796	6.781	6.799	6.799	6.783	6.104	6.111	6.138	6.118	6.113	6.109	6.47	6.461	6.462
neg_4155	3-Hydroxy-2-oxoindole	metab_9548	HMDB0240741	B(ii)	C11130	148.0398	4.8475	neg	M-H	C8H7NO2	0	44.5	-3.836081	-	0.007931804	4.439	4.424	4.398	4.424	4.38	4.387	5.202	5.263	5.258	5.256	5.243	5.271	5.273	5.322	5.306	5.284	5.279	5.332	5.324	5.365	5.339	5.341	5.348	5.348	4.627	4.616	4.632	4.632	4.674	4.625	5.059	5.054	5.06
neg_4159	L-Hypoglycin A	metab_9552	HMDB0029427	B(ii)	C08287	178.0266	4.833	neg	M+K-2H	C7H11NO2	0	45.4	-6.8047362	156-56-9	0.022674876	4.721	4.723	4.751	4.7	4.639	4.71	3.239	3.355	3.19	3.351	3.354	3.287	3.105	3.382	3.267	3.306	3.221	3.265	2.979	3.208	3.213	3.495	3.344	3.37	3.337	2.942	3.208	3.108	3.327	3.217	4.409	4.408	4.425
neg_4163	Daphnoretin	metab_9556	HMDB0250858	B(ii)	C09216	389.0089	4.8213	neg	M+K-2H	C19H12O7	0	50.5	5.69340566	2034-69-7	0.026046531	6.061	6.104	6.117	6.109	6.135	6.134	2.806	2.853	2.865	2.864	2.872	2.878	2.969	3.011	3.004	3.004	3.007	3.008	2.991	2.993	2.99	2.993	2.991	2.993	2.895	2.893	2.89	2.89	2.888	2.883	5.769	5.788	5.79
neg_4167	Coumarinic acid	metab_9560	HMDB0041592;HMDB0304296	B(ii)	C05838	387.1093	4.8086	neg	2M+Hac-H	C9H8O3	0	53.4	2.2321849	495-79-4	0.01658127	5.659	5.618	5.604	5.586	5.589	5.6	2.649	2.696	2.708	2.707	2.715	2.721	3.486	3.328	3.408	3.612	3.221	3.516	2.834	3.331	3.002	3.127	2.834	2.904	2.738	2.736	2.733	2.733	2.731	2.726	5.3	5.288	5.3
neg_4173	Violanthin	metab_9567	HMDB0302837;LMPK12110219	B(i)	C10196	577.1582	4.7737	neg	M-H	C27H30O14	59.9	0	3.37763593	40581-17-7	0.013557005	5.717	5.686	5.657	5.686	5.662	5.679	2.938	2.985	2.998	2.996	3.004	3.01	3.504	4.303	3.598	4.107	4.061	4.015	3.123	3.125	3.192	3.126	3.123	3.125	3.028	3.025	3.022	3.022	3.02	3.015	5.334	5.323	5.325
neg_4179	Alcesefoliside	metab_9573	-	B(i)	-	755.2067	4.7555	neg	M-H	C33H40O20	77.1	0	3.55727862	-	0.005757315	6.474	6.433	6.423	6.437	6.41	6.415	6.066	6.114	6.126	6.124	6.132	6.138	6.23	6.272	6.265	6.265	6.267	6.269	6.252	6.253	6.251	6.254	6.252	6.253	6.156	6.154	6.15	6.15	6.149	6.143	6.181	6.176	6.178
neg_4185	5-Hydroxy-2-[2-methyl-3-(trifluoromethyl	metab_9578	HMDB0246804	B(ii)	-	357.071	4.7418	neg	M+FA-H	C14H11F3N2O3	0	64.9	1.82773224	-	0.0217101	5.683	5.687	5.689	5.672	5.676	5.693	5.371	5.419	5.431	5.429	5.437	5.443	5.535	5.576	5.569	5.57	5.572	5.573	5.556	5.558	5.556	5.559	5.556	5.558	5.461	5.458	5.455	5.455	5.453	5.448	5.497	5.48	5.482
neg_4204	Lotaustralin	metab_9600	HMDB0033865	B(ii)	C08334	260.1143	4.6699	neg	M-H	C11H19NO6	0	58.2	1.19094097	534-67-8	0.003990349	2.625	3.175	2.595	3.025	2.594	3.065	5.865	5.905	5.902	5.881	5.887	5.901	5.891	5.929	5.928	5.933	5.939	5.935	5.865	5.862	5.849	5.842	5.873	5.842	4.994	4.993	5.022	5	4.98	4.988	5.552	5.548	5.55
neg_4205	Omadacycline	metab_9601	HMDB0255962	B(ii)	-	577.265	4.6649	neg	M+Na-2H	C29H40N4O7	0	88.5	1.08514397	-	0.00568723	3.158	3.128	3.128	3.135	3.127	3.124	3.087	3.088	3.1	3.403	3.107	3.113	4.449	4.32	4.388	4.41	4.258	4.464	4.647	4.491	4.539	4.577	4.544	4.515	5.675	5.711	5.723	5.732	5.72	5.686	4.994	4.996	4.991
neg_4210	7-Hydroxyflumequine	metab_9607	HMDB0247260	B(ii)	-	298.0473	4.6405	neg	M+Na-2H	C14H12FNO4	0	54.3	-8.7149263	-	0.004915613	2.725	2.695	3.514	2.703	3.168	3.63	5.611	5.641	5.652	5.641	5.641	5.659	5.601	5.646	5.653	5.645	5.64	5.653	5.645	5.636	5.633	5.622	5.636	5.625	5.104	5.113	5.107	5.107	5.088	5.099	5.17	5.174	5.171
neg_4216	N-Acetyl-D-phenylalanine	metab_9613	-	B(i)	C05620	206.082	4.6202	neg	M-H	C11H13NO3	86.3	0	-1.2636828	10172-89-1	0.00067085	6.99	6.964	6.954	6.928	6.934	6.935	5.597	5.62	5.639	5.619	5.622	5.649	5.755	5.784	5.802	5.796	5.8	5.805	5.642	5.628	5.618	5.632	5.639	5.618	5.512	5.511	5.529	5.508	5.498	5.507	6.63	6.63	6.63
neg_4217	4-Hydroxynonenal	metab_9614	LMFA06000051;HMDB0245269;HMDB0004362	B(ii)	C21642	201.1129	4.6071	neg	M+FA-H	C9H16O2	0	42.3	-1.9465637	75899-68-2	0.02413225	4.663	4.591	4.607	4.635	4.607	4.589	4.293	4.332	4.275	4.288	4.317	4.297	4.019	4.067	4.142	4.042	3.984	4.087	4.267	4.3	4.281	4.218	4.276	4.23	5.404	5.423	5.416	5.434	5.417	5.426	4.904	4.922	4.923
neg_4218	8-Amino-7-oxononanoic acid	metab_9615	HMDB0240687;LMFA01060168	B(ii)	C01092	186.1131	4.6031	neg	M-H	C9H17NO3	0	45.7	-2.3927267	682799-71-9	0.00962409	5.414	5.385	5.398	5.369	5.376	5.37	4.955	4.989	5.007	5.007	5.007	5.033	5.722	5.75	5.761	5.756	5.76	5.755	5.533	5.537	5.522	5.539	5.526	5.529	5.375	5.378	5.362	5.367	5.356	5.35	5.467	5.474	5.475
neg_4221	Quercetin 3-O-xylosyl-rutinoside	metab_9619	HMDB0301695	B(ii)	-	741.1912	4.5803	neg	M-H	C32H38O20	0	72	3.79461914	129235-39-8	0.01410965	6.85	6.875	6.878	6.858	6.842	6.857	6.409	6.457	6.469	6.467	6.475	6.481	6.573	6.614	6.607	6.608	6.61	6.611	6.594	6.596	6.594	6.597	6.594	6.596	6.499	6.496	6.493	6.493	6.491	6.486	6.519	6.53	6.52

neg_4222	Glutamylphenylalanine	metab_9620	HMDB0029156	B(ii)	-	275.1042	4.5803	neg	M-H2O-H	C14H18N2O5	0	43.3	1.56107515	20556-22-3	0.008570433	5.643	5.642	5.622	5.604	5.602	5.61	5.249	5.299	5.275	5.319	5.298	5.306	5.429	5.461	5.447	5.438	5.418	5.437	5.306	5.273	5.255	5.294	5.273	5.244	5.193	5.203	5.188	5.192	5.216	5.183	5.467	5.473	5.474
neg_4228	Visnagin	metab_9626	HMDB0259840;LMPK13110003	B(ii)	C09049	519.1283	4.5636	neg	2M+Hac-H	C13H10O4	0	31.4	-2.9241133	82-57-5	0.011339152	5.453	5.429	5.437	5.389	5.421	5.415	5.031	5.056	5.074	5.086	5.023	5.07	5.779	5.794	5.799	5.823	5.788	5.828	5.936	5.953	5.924	5.949	5.927	5.935	5.173	5.117	5.116	5.138	5.101	5.124	5.241	5.233	5.242
neg_4233	Edulitine	metab_9632	HMDB0030179	B(ii)	-	250.0722	4.5445	neg	M+FA-H	C11H11NO3	0	61.9	0.36092175	15272-24-9	0.018699409	5.077	5.072	5.016	4.994	5.002	5.016	2.569	2.544	2.581	2.64	3.256	2.511	2.949	2.644	3.192	2.69	2.771	3.188	3.047	2.944	2.623	2.627	3.097	2.858	2.838	2.526	2.563	3.179	2.521	2.516	4.629	4.615	4.63
neg_4234	6-Hydroxyhexanoic Acid	metab_9633	HMDB0012843	B(i)	C06103	131.0708	4.5445	neg	M-H	C6H12O3	72.4	0	-4.5819593	1191-25-9	0.002946386	6.387	6.361	6.356	6.341	6.338	6.342	6.18	6.214	6.221	6.218	6.216	6.238	6.075	6.104	6.112	6.11	6.106	6.109	6.115	6.124	6.108	6.104	6.111	6.108	5.699	5.701	5.693	5.705	5.688	5.682	6.259	6.26	6.262
neg_4246	3-Methyldioxyindole	metab_9646	HMDB0004186	B(ii)	C05834	385.1412	4.5034	neg	2M+Hac-H	C9H9NO2	0	34.7	2.23276605	3040-34-4	0.002756535	4.882	4.858	4.854	4.823	4.824	4.834	5.804	5.801	5.842	5.805	5.804	5.834	5.826	5.854	5.887	5.872	5.888	5.867	5.857	5.913	5.878	5.86	5.863	5.893	4.638	4.614	4.647	4.581	4.615	4.602	5.563	5.561	5.561
neg_4248	Parvaquone	metab_9648	HMDB0256137	B(ii)	-	291.0779	4.4959	neg	M+Cl	C16H16O3	0	70.5	-5.6395092	-	0.026740949	2.712	3.097	3.011	2.321	2.313	2.96	5.468	5.52	5.636	5.651	5.675	5.624	5.96	5.93	5.954	5.948	5.951	5.981	5.998	5.937	6.011	5.992	5.943	5.982	5.998	6.008	6.028	6.048	5.976	5.982	5.255	5.242	5.265
neg_4250	L-beta-aspartyl-L-phenylalanine	metab_9651	HMDB0011167	B(ii)	-	279.0991	4.4794	neg	M-H	C13H16N2O5	0	39.2	1.56939113	-	0.002410525	2.677	2.647	2.647	2.655	2.646	2.643	2.642	2.608	2.62	2.618	2.626	2.632	4.841	4.877	4.899	4.915	4.892	4.876	4.825	4.837	4.827	4.829	4.832	4.844	5.398	5.398	5.383	5.405	5.381	5.388	4.764	4.763	4.765
neg_4252	Menthyl ethylene glycol carbonate	metab_9653	HMDB0036141	B(ii)	-	289.1661	4.4738	neg	M+FA-H	C13H24O4	0	51.5	1.97882674	156324-78-6	0.001625934	2.617	2.587	2.615	2.613	2.586	2.583	3.575	3.901	3.784	3.855	3.606	3.921	2.663	2.705	2.698	2.698	2.739	2.705	3.534	3.15	3.35	3.011	3.024	3.339	6.543	6.551	6.539	6.553	6.54	6.54	5.902	5.901	5.901
neg_4256	Vicenin	metab_9657	HMDB0030708;HMDB0302244	B(ii)	C10195	625.1434	4.4611	neg	M-H	C27H30O17	0	52.2	3.73418187	23666-13-9	0.005116902	5.779	5.749	5.749	5.757	5.748	5.745	6.334	6.351	6.368	6.359	6.364	6.389	6.087	6.117	6.116	6.126	6.118	6.132	6.336	6.347	6.323	6.336	6.33	6.3	5.762	5.75	5.756	5.754	5.756	5.76	5.995	5.994	5.998
neg_4257	Dihydroxylysinonorleucine	metab_9658	HMDB0251345	B(ii)	-	488.2161	4.4571	neg	M+K-2H	C18H37N5O8	0	41.5	7.3594506	-	0.016715146	5.384	5.379	5.397	5.352	5.356	5.364	3.775	3.686	3.888	3.699	3.659	3.934	3.109	3.15	3.143	3.144	3.146	3.466	3.266	3.132	3.13	3.133	3.45	3.241	3.035	3.032	3.029	3.029	3.027	3.022	5.007	5.019	5.02
neg_4265	B-D-Xylopyranosyl-(1->4)-a-L-rhamnopyr	metab_9666	HMDB0041221	B(ii)	-	465.0982	4.4176	neg	M+K-2H	C16H28O13	0	47.4	-7.8678217	-	0.002364734	6.596	6.566	6.561	6.553	6.542	6.55	5.166	5.159	5.232	5.205	5.162	5.218	4.94	4.927	4.932	5.001	4.987	4.947	5.331	5.323	5.358	5.33	5.352	5.318	5.506	5.527	5.502	5.543	5.516	5.511	6.182	6.181	6.18
neg_4266	10-Acetoxytoxol	metab_9667	HMDB0030287	B(ii)	-	321.0985	4.4176	neg	M+FA-H	C15H16O5	0	50	1.8724661	75680-15-8	0.000606869	2.739	2.709	2.709	2.716	2.708	2.705	6.024	6.056	6.069	6.07	6.063	6.09	6.662	6.697	6.697	6.706	6.702	6.705	6.811	6.837	6.813	6.81	6.811	6.817	6.149	6.141	6.139	6.152	6.138	6.14	6.313	6.313	6.313
neg_4267	N-Lactoylvaline	metab_9668	HMDB0062181	B(ii)	-	170.0818	4.4176	neg	M-H2O-H	C8H15NO4	0	65.1	-2.2916736	21753-44-6	0.008443921	4.127	4.197	4.222	4.132	4.193	4.162	5.878	5.909	5.923	5.934	5.894	5.979	6.18	6.106	6.145	6.163	6.153	6.121	6.102	6.068	6.021	6.154	5.998	6.053	5.519	5.474	5.518	5.478	5.505	5.493	5.732	5.738	5.732
neg_4276	3-(6-((4-(Trifluoromethoxy)phenyl)amino)p	metab_9678	HMDB0252899	B(ii)	-	373.0944	4.3978	neg	M-H	C18H13F3N4O2	0	82.2	6.86435262	-	0.01784171	5.008	5.003	5.184	5.153	5.109	5.106	2.113	2.16	2.172	2.171	2.438	2.185	2.276	2.4	2.695	2.311	2.313	2.314	2.578	2.299	2.297	2.3	2.297	2.299	2.202	2.2	2.196	2.197	2.195	2.19	4.835	4.822	4.822
neg_4291	4-Benzofuranacetamide, N-methyl-N-(7-(:	metab_9695	HMDB0249570	B(ii)	-	441.2366	4.3	neg	M+FA-H	C24H32N2O3	0	69.7	-7.3997336	-	0.004841721	5.959	5.901	5.859	5.87	5.861	5.873	5.884	5.87	5.892	5.866	5.914	5.882	5.582	5.734	5.651	5.66	5.691	5.692	5.654	5.639	5.652	5.57	5.635	5.569	5.472	5.469	5.466	5.466	5.464	5.459	5.718	5.717	5.714
neg_4297	Fasoracetam	metab_9701	HMDB0252174	B(ii)	C13311	241.1195	4.2747	neg	M+FA-H	C10H16N2O2	0	51.1	0.48409196	110958-19-5	0.014289285	5.646	5.605	5.595	5.616	5.588	5.548	5.14	5.19	5.119	5.183	5.162	5.144	5.22	5.279	5.245	5.291	5.283	5.303	5.059	5.152	5.133	5.152	5.146	5.11	5.333	5.346	5.307	5.335	5.335	5.371	5.416	5.417	5.427
neg_4298	DIMBOA trihexose	metab_9702	HMDB0304554	B(ii)	-	170.0819	4.2697	neg	M-H2O-H	C8H15NO4	0	68.3	-2.1728606	-	0.003202093	4.436	4.409	4.361	4.377	4.388	4.377	6.151	6.142	6.17	6.161	6.198	6.166	6.279	6.391	6.377	6.372	6.396	6.39	6.284	6.319	6.325	6.257	6.321	6.277	5.721	5.763	5.706	5.744	5.718	5.721	5.997	5.995	5.994
neg_430	LysoPI(16:0/0:0)	metab_9705	HMDB0061695	B(ii)	-	593.2751	7.6685	neg	M-H, M+Na-2I	C25H49O12P	0	77.5	7.48845127	1425501-12-7	0.036906542	7.31	7.648	7.684	7.662	7.678	7.656	7.682	7.25	7.201	7.165	7.178	7.199	7.402	6.827	6.733	6.727	6.72	6.731	6.704	6.941	6.957	6.965	6.956	6.976	6.882	7.054	7.059	7.07	7.065	7.089	6.326	6.326	6.298
neg_4323	12-Hydroxyjasmonic acid glucoside	metab_9731	HMDB0301837	B(ii)	C08558	387.1666	4.1485	neg	M-H	C18H28O9	0	51.6	1.3341219	-	0.009486758	5.869	5.843	5.816	5.817	5.822	5.814	4.494	4.582	4.544	4.603	4.535	4.593	4.408	4.421	4.51	4.449	4.495	4.518	4.508	4.511	4.426	4.407	4.504	4.32	3.565	3.757	3.498	3.796	3.623	3.7	5.342	5.335	5.343
neg_4334	Arecaidine	metab_9742	HMDB0030352	B(ii)	C10128	178.0266	4.1228	neg	M+K-2H	C7H11NO2	0	31.6	-6.8058532	499-04-7	0.017942392	5.085	5.062	5.05	5.055	5.045	5.057	4.461	4.508	4.52	4.519	4.526	4.533	4.624	4.666	4.659	4.659	4.662	4.663	4.646	4.648	4.645	4.648	4.646	4.648	4.55	4.548	4.544	4.545	4.543	4.538	4.584	4.57	4.571
neg_4340	(2S)-2-Amino-3-[4-[(2S)-2-amino-3-(4-h	metab_9749	HMDB0260141	B(ii)	-	415.0446	4.0949	neg	M+K-2H	C18H19ClN2O5	0	35	-5.9905235	-	0.026300327	3.41	3.418	3.436	3.334	3.321	3.441	4.103	4.201	4.063	4.312	4.316	4.265	5.389	5.416	5.405	5.433	5.407	5.464	5.484	5.382	5.466	5.469	5.447	5.456	4.588	4.644	4.616	4.626	4.56	4.515	5.233	5.232	5.252
neg_4366	Corchoionol C 9-glucoside	metab_9776	HMDB0029772	B(ii)	-	431.1931	4.0168	neg	M+FA-H	C19H30O8	0	58.9	2.18400811	-	0.015744681	6.419	6.377	6.368	6.343	6.362	6.36	4.861	4.906	4.881	4.903	4.87	4.895	5.801	5.859	5.85	5.857	5.848	5.858	5.393	5.4	5.391	5.364	5.403	5.412	4.822	4.84	4.877	4.876	4.844	4.776	6.128	6.116	6.117
neg_4374	Methionylhydroxyproline	metab_9785	HMDB0028974	B(ii)	-	243.081	3.9865	neg	M-H2O-H	C10H18N2O4S	0	32.3	0.58918955	148305-57-1	0.00506383	4.627	4.654	4.638	4.587	4.596	4.628	5.489	5.465	5.508	5.497	5.457	5.49	5.557	5.646	5.581	5.574	5.597	5.615	5.649	5.609	5.573	5.608	5.598	5.595	5.743	5.694	5.715	5.705	5.723	5.693	4.844	4.84	4.841
neg_4391	Isovaleric Acid	metab_9803	LMFA01020181;HMDB0000718;MJDBOTE0000751	B(i)	C08262	101.06	3.9205	neg	M-H	C5H10O2	32.3	0	-7.480657	503-74-2	0.017627377	4.004	3.955	4.026	4.177	4.247	4.105	3.287	3.416	3.408	3.296	3.402	3.383	3	2.253	2.746	2.831	2.642	2.533	2.091	2.229	2.197	2.196	2.091	2.49	1.996	1.993	1.99	1.99	1.988	1.983	3.87	3.869	3.856
neg_4398	2-[(2,6-Dioxocyclohexyl)methyl]cyclohexan	metab_9810	HMDB0248526	B(ii)	-	281.1009	3.8953	neg	M+FA-H	C13H16O4	0	37.6	-9.1621899	-	0.010230848	6.025	5.997	5.978	5.968	5.959	5.967	4.03	4.024	3.948	4.011	3.913	3.932	2.419	2.974	2.626	2.695	3.125	2.353	2.982	2.253	2.479	2.332	2.869	2.444	3.158	0.5754	0.6824	1.039	0.8357	1.106	5.016	5.007	5.009
neg_4405	Proline betaine	metab_9818	HMDB0004827	B(ii)	C10172	188.0924	3.8716	neg	M+FA-H	C7H13NO2	0	50.6	-2.8053587	471-87-4	0.005831944	4.652	4.618	4.574	4.552	4.587	4.587	5.98	6.012	6	6.018	6.011	6.031	6.429	6.428	6.454	6.443	6.449	6.464	6.349	6.359	6.36	6.34	6.334	6.345	5.784	5.799	5.8	5.822	5.814	5.816	6.021	6.016	6.017
neg_4406	Prenyl glucoside	metab_9819	HMDB0031876	B(ii)	C09808	293.1245	3.8661	neg	M+FA-H	C11H20O6	0	58.4	1.22545135	117861-55-9	0.00850021	6.18	6.147	6.137	6.122	6.119	6.114	3.423	3.308	3.465	3.406	3.288	3.387	3.586	3.38	3.454	3.575	3.61	3.674	3.458	3.607	3.358	3.442	3.336	3.444	3.849	3.599	3.825	3.968	3.761	3.81	5.793	5.787	5.786
neg_4410	Sorbinil	metab_9824	HMDB0258388	B(ii)	-	273.0078	3.85	neg	M+K-2H	C11H9FN2O3	0	40.3	-2.2047031	-	0.007456789	5.69	5.676	5.653	5.64	5.635	5.644	2.445	2.492	2.504	2.503	2.511	2.517	2.608	2.65	2.643	2.643	2.645	2.647	2.63	2.631	2.629	2.632	2.63	2.631	3.438	3.366	3.445	3.245	3.426	3.299	5.247	5.246	5.241
neg_4413	Pilaralisib	metab_9827	HMDB0256547	B(ii)	-	585.1312	3.8409	neg	M+FA-H	C25H25ClN6O4S	0	48.9	-3.1288686	-	0.022758741	3.771	3.741	3.741	3.748	3.74	3.736	4.022	3.972	3.713	3.949	3.776	4.045	4.531	4.251	4.557	4.329	4.573	4.518	4.414	4.254	4.342	4.465	4.237	3.925	6.412	6.441	6.449	6.463	6.43	6.456	4.842	4.827	4.823
neg_4415	(1S,2S,3S,4R)-3-(1-Acetamido-2-ethylbuty	metab_9829	HMDB0242447	B(ii)	-	373.2097	3.8409	neg	M+FA-H	C15H28N4O4	0	74.6	1.47818795	-	0.022408415	5.165	5.207	5.176	5.145	5.178	5.151	4.826	4.873	4.885	4.884	4.892	4.898	4.99	5.031	5.024	5.025	5.027	5.028	5.011	5.013	5.01	5.014	5.011	5.013	4.916	4.913	4.91	4.91	4.908	4.903	4.952	4.935	4.952
neg_4420	N6-Methyl-2'-deoxyadenosine	metab_9835	HMDB0255300	B(ii)	C03795	246.0985	3.8323	neg	M-H2O-H	C11H15N5O3	0	34.7	-4.4533264	2002-35-9	0.020765661	2.597	2.622	2.567	2.681	2.566	2.563	4.71	4.724	4.73	4.718	4.733	4.799	4.843	4.818	4.846	4.871	4.819	4.894	4.88	4.888	4.878	4.893	4.754	4.856	4.016	4.004	3.92	3.898	3.997	3.946	4.515	4.5	4.516
neg_4432	2-Chloro-1-[(2S,3S,5S,10S,13S)-3-hydroxy	metab_9848	HMDB0247839	B(ii)	-	474.2215	3.7991	neg	M+K-2H	C25H40ClNO3	0	66.7	7.32314258	-	0.004159158	6.388	6.384	6.36	6.321	6.345	6.31	5.853	5.901	5.913	5.911	5.919	5.925	6.017	6.058	6.051	6.052	6.054	6.055	6.039	6.04	6.038	6.041	6.038	6.04	5.943	5.94	5.937	5.937	5.935	5.93	5.966	5.963	5.964
neg_4443	Sarmentosin	metab_9858	HMDB0030697	B(ii)	C08340	256.0805	3.7456	neg	M-H2O-H	C11H17NO7	0	31.7	-7.820315	71933-54-5	0.021756308	3.104	3.074	3.074	3.082	3.073	3.07	4.534	4.618	4.581	4.618	4.622	4.567	5.068	5.032	5.09	5.039	5.103	5.114	4.941	4.971	4.991	4.943	4.999	4.964	3.077	3.66	3.639	3.667	3.865	3.681	4.304	4.287	4.289
neg_4445	(3R-(3alpha,4alpha(2R*,3R*),5beta,6beta))-	metab_9859	HMDB0260151	B(ii)	-	346.1623	3.7314	neg	M+Na-2H	C17H27NO5	0	36.3	-3.8346031	-	0.004790162	5.435	5.357	5.366	5.369	5.344	5.336	2.657	2.705	2.717	2.715	2.723	2.729	2.821	2.862	2.855	2.856	2.858	2.859	2.842	2.844	2.841	2.845	2.842	2.844	2.747	2.744	2.741	2.741	2.739	2.734	4.93	4.929	4.926
neg_4453	6-Cyano-7-nitroquinoxaline-2,3-dione	metab_9868	HMDB0250384	B(ii)	C13668	268.9709	3.7045	neg	M+K-2H	C9H4N4O4	0	41.1	-4.3061118	115066-14-3	0.033760728	4.374	4.318	4.299	4.208	4.31	4.211	3.225	3.478	3.479	3.408	3.486	3.458	2.528	2.569	2.563	2.563	2.565	2.566	2.55	2.551	2.549	2.552	2.55	2.551	4.853	4.638	4.934	4.871	4.79	4.821	4.444	4.47	4.446
neg_4455	2-[4-(sulfooxy)phenyl]acetic acid	metab_9870	HMDB0132500	B(ii)	-	266.9739	3.6989	neg	M+Cl	C8H8O6S	0	54.3	1.38226657	-	0.002991746	4.531	4.498	4.488	4.428	4.454	4.444	3.014	2.572	3.001	3.026	2.483	2.489	3.633	3.407	3.206	3.289	2.618	3.579	2.602	2.692	2.601	3.404	3.147	2.737	5.821	5.834	5.832	5.839	5.844	5.833	5.305	5.303	5.304

neg_4456	Skimmin	metab_9871	HMDB0258334	B(ii)	-	369.0831	3.6944	neg	M+FA-H	C15H16O8	0	39.8	1.27885815	-	0.010804077	6.083	6.042	6.028	6.007	6.011	6.009	2.064	2.111	2.123	2.546	2.648	2.135	3.345	2.267	2.868	3.262	2.964	2.783	3.981	3.789	3.391	2.982	2.786	2.805	2.153	2.15	2.147	2.147	2.145	2.14	5.644	5.644	5.636
neg_4459	Vaccinoside	metab_9874	HMDB0034733	B(ii)	-	581.153	3.6848	neg	M+FA-H	C25H28O13	0	55.3	3.35479062	36138-58-6	0.008154299	6.094	6.044	6.049	6.012	6.022	6.03	2.281	2.328	2.34	2.339	2.346	2.353	2.444	2.485	2.478	2.479	2.481	2.482	2.466	2.467	2.465	2.468	2.466	2.467	2.37	2.368	2.364	2.365	2.363	2.358	5.698	5.691	5.693
neg_4469	8,8'-Diferulic acid	metab_9885	HMDB0247469;HMDB0000954	B(ii)	C01494	367.082	3.6565	neg	M-H2O-H	C20H18O8	0	47.6	-0.8277546	537-98-4	0.014696553	5.356	5.173	5.158	5.123	5.122	5.107	4.499	4.546	4.559	4.557	4.565	4.571	4.663	4.704	4.697	4.698	4.7	4.701	4.684	4.686	4.684	4.687	4.684	4.686	4.589	4.648	4.649	4.619	4.62	4.626	5.297	5.309	5.307
neg_4472	5-Hydroxylysinonorleucine	metab_9889	HMDB0246805	B(ii)	-	312.157	3.652	neg	M+Na-2H	C12H25N3O5	0	44.8	9.91371841	-	0.001963515	5.441	5.384	5.386	5.346	5.356	5.371	0.1385	0.1519	0.1555	0.1551	0.1574	0.1593	0.1896	0.2047	0.2022	0.2023	0.2032	0.2036	0.1974	0.1979	0.1971	0.1982	0.1974	0.198	4.19	4.153	4.188	4.228	4.143	4.085	4.977	4.976	4.977
neg_4474	N-[[(2S,3S,4R,5R)-5-(6-Aminopurin-9-yl)-	metab_9891	HMDB0257719	B(ii)	-	373.1048	3.6465	neg	M+Cl	C13H18N6O5	0	49.7	4.60512051	-	0.006790564	2.651	2.22	2.264	2.267	2.011	2.926	5.341	5.383	5.423	5.395	5.391	5.419	5.523	5.555	5.562	5.576	5.569	5.574	5.521	5.552	5.533	5.562	5.524	5.533	6.186	6.178	6.178	6.187	6.18	6.184	5.672	5.671	5.677
neg_448	1-(9Z-Nonadecenoyl)-glycero-3-phosphoe	metab_9897	LMGP02050019;HMDB0243799	B(ii)	-	492.3099	6.7972	neg	M-H, M+FA-H	C24H48NO7P	0	75.3	0.74005901	-	0.012675397	5.783	5.662	5.703	5.715	5.709	5.67	6.002	6.093	6.083	6.083	6.108	6.126	6.052	6.119	6.129	6.135	6.119	6.147	6.227	6.2	6.2	6.219	6.197	6.211	6.18	6.135	6.137	6.143	6.132	6.143	6.132	6.134	6.124
neg_4487	Bindarit	metab_9905	HMDB0249216	B(ii)	-	345.1206	3.5928	neg	M+Na-2H	C19H20N2O3	0	30.9	-4.5081144	-	0.048975147	4.89	4.806	4.72	4.66	4.65	4.603	2.392	2.44	2.452	2.45	2.458	2.464	2.555	2.597	2.59	2.59	2.593	2.594	2.577	2.579	2.576	2.579	2.577	2.579	2.482	2.479	2.476	2.476	2.474	2.469	4.369	4.371	4.406
neg_449	N-Linoleoyl Threonine	metab_9908	HMDB0241985	B(ii)	-	380.2809	6.7095	neg	M-H, M+Na-2I	C22H39NO4	0	39.8	0.7932617	-	0.007066647	7.038	7.01	6.995	6.996	6.989	6.991	5.516	5.489	5.532	5.595	5.569	5.515	4.481	4.724	4.763	4.782	4.445	4.589	4.886	4.791	5.006	4.663	4.904	4.801	5.248	5.297	5.253	5.288	5.174	5.208	6.585	6.591	6.587
neg_4499	Tafamidis	metab_9917	HMDB0258677	B(ii)	-	287.9611	3.5321	neg	M-H2O-H	C14H7Cl2NO3	0	56.5	-4.4195209	-	0.018486238	2.623	2.593	2.593	2.6	2.592	2.589	4.453	4.441	4.541	4.468	4.439	4.511	5.349	5.401	5.407	5.387	5.416	5.383	5.176	5.15	5.17	5.191	5.172	5.162	5.189	5.18	5.165	5.22	5.178	5.205	4.968	4.982	4.983
neg_4500	4-Oxo-1,4-dihydroquinoline-3-carboxylic a	metab_9920	HMDB0246559	B(ii)	-	188.0349	3.5321	neg	M-H	C10H7NO3	0	43.9	-2.0510558	-	0.030823387	4.822	4.751	4.74	4.728	4.778	4.74	5.669	5.704	5.729	5.701	5.715	5.72	6.437	6.455	6.479	6.49	6.491	6.489	6.279	6.275	6.284	6.308	6.269	6.281	6.304	6.287	6.294	6.316	6.29	6.3	6.109	6.088	6.113
neg_4504	Caffeic acid sulfate	metab_9924	HMDB0041706;HMDB0041708;HMDB0304903	B(ii)	-	256.9764	3.5199	neg	M-H	C9H6O7S-2	0	42.4	1.07742948	151481-52-6;	0.003976464	5.437	5.365	5.362	5.384	5.404	5.351	4.538	4.594	4.621	4.585	4.615	4.622	5.061	5.103	5.163	5.134	5.105	5.117	5.157	5.112	5.165	5.158	5.118	5.113	5.012	5.018	5.019	5	4.98	5.033	5.27	5.267	5.271
neg_4508	4-Oxo-1-(3-pyridyl)-1-butanone	metab_9928	HMDB0062406	B(ii)	C19567	208.0611	3.5103	neg	M+FA-H	C9H9NO2	0	52	-2.6584468	76014-80-7	0.017715951	2.572	2.694	2.835	2.902	2.415	2.275	4.604	4.648	4.67	4.644	4.672	4.681	5.208	5.249	5.278	5.265	5.265	5.273	5.188	5.161	5.198	5.217	5.153	5.175	4.801	4.767	4.804	4.785	4.785	4.802	4.877	4.867	4.882
neg_4511	UDP-D-apiose	metab_9932	HMDB0012301	B(ii)	C01623	517.0307	3.4981	neg	M-H2O-H	C14H22N2O16P2	0	43	7.58501577	20230-91-5	0.045803385	5.82	5.77	5.804	5.821	5.859	5.796	1.503	1.549	1.561	2.093	1.567	1.573	1.662	1.703	2.219	1.697	1.699	1.7	1.684	1.685	2.209	1.686	1.709	1.685	1.59	1.588	1.584	1.585	1.583	1.578	5.174	5.18	5.211
neg_4512	Dihydroferulic acid 4-O-glucuronide	metab_9933	HMDB0041723	B(ii)	-	417.1049	3.4981	neg	M+FA-H	C16H20O10	0	41.9	2.74853768	-	0.009079312	7.183	7.124	7.117	7.127	7.165	7.118	5.156	5.206	5.233	5.19	5.194	5.234	5.005	5.052	5.048	5.028	5.089	5.037	5.179	5.13	5.182	5.227	5.129	5.17	4.842	4.853	4.905	4.799	4.776	4.863	6.691	6.684	6.691
neg_4519	P-Coumaric acid glucuronide	metab_9940	HMDB0240550	B(ii)	-	385.0784	3.4782	neg	M+FA-H	C15H16O9	0	32.9	2.18484898	-	0.007097054	5.839	5.769	5.745	5.753	5.796	5.752	5.154	5.202	5.214	5.212	5.22	5.226	5.318	5.359	5.353	5.353	5.355	5.357	5.34	5.341	5.339	5.342	5.34	5.341	5.244	5.242	5.238	5.238	5.237	5.231	5.264	5.264	5.269
neg_4526	N-Acetylserotonin	metab_9947	PW_C000956;HMDB0001238	B(i)	C00978	217.0982	3.4518	neg	M-H	C12H14N2O2	63.9	0	-0.4131572	1210-83-9	0.025712871	3.671	3.655	3.534	3.388	3.726	3.308	4.586	4.636	4.68	4.672	4.656	4.747	5.117	5.101	5.113	5.126	5.118	5.125	5.037	5.035	5.062	5.055	5.005	5.027	5.156	5.13	5.123	5.113	5.142	5.132	4.755	4.754	4.773
neg_4532	2-(2-Hydroxyethoxy)-6-(2H-tetrazol-5-yl):	metab_9954	HMDB0249493	B(ii)	-	359.0558	3.4375	neg	M+Cl	C16H12N4O4	0	43.9	1.65463315	-	0.071926144	4.817	4.773	4.72	4.777	4.778	4.653	4.387	4.441	4.458	4.445	4.442	4.558	4.163	4.484	4.205	4.394	4.294	4.282	4.408	4.354	4.31	4.413	4.335	4.247	4.598	4.592	4.576	4.602	4.582	4.507	4.585	4.586	4.639
neg_4541	Zierin	metab_9964	HMDB0301769	B(ii)	C08345	310.0939	3.4095	neg	M-H	C14H17NO7	0	40.6	2.12985012	645-02-3	0.00616619	4.405	4.445	4.475	4.525	4.546	4.533	5.257	5.26	5.284	5.26	5.236	5.322	5.341	5.361	5.38	5.361	5.355	5.384	5.157	5.19	5.156	5.184	5.143	5.176	5.214	5.195	5.197	5.206	5.206	5.246	5.048	5.052	5.053
neg_4542	Glycerol glutamate	metab_9965	HMDB0252840	B(ii)	-	272.0181	3.4095	neg	M+K-2H	C8H13NO7	0	61.1	1.3913625	-	0.006616611	3.515	3.485	3.485	3.493	3.484	3.481	3.398	3.65	3.823	3.752	3.715	3.906	4.748	4.773	4.762	4.742	4.751	4.781	4.238	4.216	4.027	4.28	4.184	4.258	5.462	5.462	5.456	5.469	5.479	5.466	4.844	4.839	4.843
neg_4543	Xanthurenic Acid	metab_9966	PW_C000703;HMDB0000881;MJDBOTE0001103	B(i)	C02470	204.03	3.4045	neg	M-H	C10H7NO4	98.7	0	-1.2108736	59-00-7	0.009908956	4.742	4.65	4.688	4.704	4.69	4.687	5.619	5.662	5.698	5.672	5.676	5.718	6.275	6.292	6.311	6.303	6.307	6.309	5.933	5.934	5.947	5.967	5.917	5.945	7.004	6.991	6.984	6.991	6.997	6.992	6.445	6.441	6.45
neg_4556	Cyclohexyladenosine	metab_9980	HMDB0250660	B(ii)	-	370.1514	3.356	neg	M+Na-2H	C16H23N5O4	0	30.2	4.99892479	-	0.011119801	4.648	4.634	4.594	4.589	4.608	4.634	4.98	4.986	5.044	5.04	4.992	5.042	5.628	5.649	5.648	5.644	5.641	5.686	5.536	5.553	5.566	5.566	5.537	5.56	5.495	5.478	5.474	5.469	5.486	5.506	5.338	5.329	5.33
neg_4557	Homoanserine	metab_9981	HMDB0005767	B(ii)	-	253.1308	3.356	neg	M-H	C11H18N4O3	0	46.7	0.89951541	20314-38-9	0.004384251	5.337	5.147	5.071	5.092	5.07	5.117	2.398	2.545	2.986	2.456	2.512	2.47	2.669	2.637	2.669	2.762	2.645	3.508	2.583	2.584	2.981	3.504	3.341	2.996	2.487	2.485	2.481	2.482	2.48	2.475	4.961	4.962	4.965
neg_4565	FAD	metab_9990	HMDB0252293;HMDB0001248;PW_C000964	B(i)	C00016	784.1531	3.311	neg	M-H	C27H33N9O15P2	74	0	4.15426442	146-14-5	0.014218517	6.351	6.312	6.32	6.311	6.329	6.316	5.637	5.678	5.725	5.693	5.643	5.724	5.456	5.52	5.498	5.527	5.426	5.491	5.522	5.563	5.554	5.495	5.508	5.571	5.592	5.601	5.575	5.541	5.615	5.594	6.036	6.035	6.046
neg_4566	N-hydroxy-L-tryptophan	metab_9991	HMDB0255152	B(ii)	C19716	265.0835	3.311	neg	M+FA-H	C11H12N2O3	0	40.4	2.08609028	-	0.002921691	4.128	4.098	4.098	4.105	4.097	4.094	4.13	4.253	4.183	4.069	4.198	4.275	4.678	4.688	4.687	4.691	4.646	4.717	4.614	4.64	4.669	4.617	4.616	4.587	5.601	5.579	5.591	5.581	5.598	5.609	4.994	4.992	4.993
neg_4581	D-Galactaric acid	metab_10008	HMDB0000639	B(i)	C00879	209.03	3.2607	neg	M-H	C6H10O8	81	0	-1.1989809	526-99-8	0.006162507	5.585	5.532	5.537	5.537	5.523	5.533	3.077	3.314	3.293	3.352	3.346	3.185	3.02	2.8	2.837	2.839	2.752	2.732	3.381	3.285	3.262	3.053	3.075	2.952	2.149	1.949	2.253	1.349	2.022	2.193	5.016	5.016	5.02
neg_4592	Glycyl-Histidine	metab_10019	HMDB0028843	B(ii)	-	249.0383	3.2389	neg	M+K-2H	C8H12N4O3	0	65.4	-5.875417	-	0.008589258	2.866	2.48	2.38	2.845	2.379	2.376	4.679	4.69	4.738	4.745	4.71	4.733	4.453	4.515	4.526	4.522	4.525	4.501	4.441	4.45	4.453	4.421	4.405	4.453	5.069	5.035	5.061	5.035	5.074	5.032	4.579	4.578	4.585
neg_4593	3-(4-Hydroxyphenyl)lactate	metab_10020	HMDB0000755	B(i)	C03672	181.0502	3.2389	neg	M-H	C9H10O4	77.4	0	-2.2781015	306-23-0	0.00089594	5.02	4.986	4.988	4.954	4.952	4.949	5.836	5.873	5.903	5.903	5.896	5.905	5.72	5.762	5.777	5.757	5.773	5.785	5.712	5.725	5.73	5.728	5.722	5.735	6.219	6.209	6.22	6.203	6.219	6.21	5.85	5.849	5.849
neg_4599	Glutamyltyrosine	metab_10026	HMDB0028831	B(ii)	-	291.0992	3.2223	neg	M-H2O-H	C14H18N2O6	0	38.6	1.7322841	3422-39-7	0.004762812	5.046	5.02	5.037	5.011	5.024	5.004	5.229	5.244	5.281	5.264	5.272	5.289	5.107	5.131	5.144	5.142	5.123	5.125	5.144	5.178	5.167	5.186	5.178	5.182	5.367	5.343	5.371	5.352	5.377	5.356	5.218	5.214	5.215
neg_4600	Methyl n-acetylanthranilate	metab_10029	HMDB0032388	B(ii)	C06332	192.0663	3.2223	neg	M-H	C10H11NO3	0	40.6	-1.8118567	2719-8-6	0.007994238	4.873	4.831	4.822	4.828	4.815	4.808	5.915	5.948	5.979	5.964	5.978	5.987	6.016	6.058	6.057	6.059	6.054	6.08	5.917	5.941	5.94	5.931	5.94	5.949	5.159	5.148	5.156	5.153	5.154	5.163	5.674	5.674	5.68
neg_4605	N-Acetyl-DL-Valine	metab_10034	HMDB0011757	B(i)	-	158.0818	3.2071	neg	M-H	C7H13NO3	41.3	0	-3.1622923	96-81-1	0.00594708	5.631	5.585	5.591	5.587	5.578	5.587	5.585	5.618	5.658	5.646	5.634	5.658	5.758	5.791	5.79	5.787	5.795	5.825	5.684	5.695	5.695	5.704	5.693	5.704	5.527	5.506	5.52	5.509	5.519	5.524	5.679	5.682	5.684
neg_4612	Aspartame	metab_10042	HMDB0001894;MJDBOTE0001640	B(i)	C11045	647.2594	3.1683	neg	2M+Hac-H	C14H18N2O5	64	0	4.04557278	22839-47-0	0.017292777	4.651	4.621	4.62	4.628	4.62	4.616	4.565	4.581	4.788	4.759	4.723	4.835	5.589	5.645	5.671	5.654	5.642	5.673	5.839	5.862	5.861	5.863	5.858	5.866	4.911	4.902	4.999	4.945	4.918	4.952	5.13	5.139	5.124
neg_462	13(S)-HpODE	metab_10050	HMDB0003871	B(i)	C04717	311.2231	6.3105	neg	M-H, M+Na-2I	C18H32O4	42.5	0	0.94940645	33964-75-9	0.002692918	6.986	6.936	6.915	6.916	6.92	6.916	6.56	6.611	6.642	6.646	6.638	6.642	6.559	6.624	6.632	6.624	6.625	6.633	6.619	6.6	6.6	6.591	6.591	6.603	7.135	7.123	7.115	7.123	7.116	7.113	6.99	6.99	6.988
neg_4620	N-Acetyldopamine	metab_10051	HMDB0041943	B(ii)	-	194.0819	3.1612	neg	M-H	C10H13NO3	0	33.5	-1.7519268	2494-12-4	0.005315538	5.324	5.288	5.279	5.273	5.275	5.247	6.076	6.103	6.118	6.127	6.123	6.131	6.119	6.162	6.166	6.155	6.163	6.165	6.303	6.313	6.297	6.311	6.313	6.308	5.586	5.579	5.584	5.568	5.57	5.572	5.954	5.954	5.958
neg_4626	Lamivudine sulfoxide	metab_10057	HMDB0060599;PW_C040661	B(ii)	-	266.0205	3.1552	neg	M+Na-2H	C8H11N3O4S	0	46.2	-4.6794528	-	0.002365036	5.868	5.855	5.852	5.824	5.83	5.818	6.051	6.083	6.106	6.119	6.109	6.111	6.092	6.134	6.134	6.121	6.136	6.138	6.141	6.151	6.152	6.167	6.163	6.153	6.013	5.988	6.005	5.994	6.01	5.994	6.044	6.042	6.044
neg_4631	Nicotinic Acid Mononucleotide	metab_10063	PW_C000885;HMDB0001132	B(i)	C01185	334.0332	3.1421	neg	M-H	C11H14NO9P	45.3	0	-0.5088349	321-02-8	0.005202912	5.172	5.174	5.171	5.149	5.173	5.146	2.134	2	1.852	2.394	1.504	2.037	2.461	1.64	2.158	2.359	1.636	2.199	2.576	1.622	2.019	1.623	1.621	2.104	1.96	2.227	2.405	2.092	2.329	2.912	4.648	4.652	4.648
neg_4634	(2R)-2-[[(2S,3R)-3-Amino-2-hydroxy-4-pl	metab_10066	HMDB0242556	B(ii)	-	345.1209	3.1361	neg	M+K-2H	C16H24N2O4	0	40.7	-4.3559389	-	0.000664531	5.626	5.586	5.561	5.536	5.545	5.544	2.334	2.381	2.394	2.392	2.4	2.406	2.497	2.539	2.532	2.532	2.534	2.992	2.519	2.52	2.518	2.521	2.519	2.521	2.836	2.421	2.418	2.941	2.416	2.673	4.859	4.859	4.86

neg_4638	(Z)-N-Coumaroyl-5-hydroxyanthranilic aci	metab_10070	HMDB0038577	B(ii)	C08472	298.0724	3.1241	neg	M-H	C16H13NO5	0	39.9	1.16083147	108605-70-5	0.032645561
neg_465	Corchorifatty acid D	metab_10082	HMDB0033243	B(ii)	-	307.1917	6.2545	neg	M-H2O-H, M-I	C18H28O4	0	48.8	0.65673032	-	0.005415106
neg_4650	4-Allylpyrocatechol sulfate	metab_10083	HMDB0304934	B(ii)	-	505.0473	3.0926	neg	2M+FA-H	C9H10O5S	0	59.5	-1.5397538	-	0.018266577
neg_4653	Vanillylmandelic acid	metab_10086	PW_C000197;HMDB0304531;HMDB0000291;HMDB0	B(ii)	C05584	197.0453	3.0926	neg	M-H	C9H10O5	0	53.1	-1.261667	55-10-7;1324	0.002935165
neg_4667	Prolylhydroxyproline	metab_10100	HMDB0256781;HMDB0006695	B(ii)	-	227.1036	3.0846	neg	M-H	C10H16N2O4	0	59.2	-0.4524955	18684-24-7	0.001810655
neg_4671	3'-Sialyllactose	metab_10105	HMDB0000825	B(ii)	-	668.1832	3.0766	neg	M+Cl	C23H39NO19	0	65.7	3.48835406	35890-38-1	0.015885539
neg_468	Tetranorprostanedioic acid	metab_10112	HMDB0258926	B(ii)	-	283.1916	6.2385	neg	M-H, M+Na-2I	C16H28O4	0	50.6	0.56742171	-	0.002390892
neg_4680	5-Acetylamino-6-formylamino-3-methylur	metab_10113	HMDB0011105;PW_C006748	B(ii)	C16365	271.0704	3.0766	neg	M+FA-H	C8H10N4O4	0	36.2	8.89690818	85438-96-6	0.005027182
neg_4682	Dihydromaleimide beta-D-glucoside	metab_10115	HMDB0030277	B(ii)	-	242.067	3.0766	neg	M-H2O-H	C10H15NO7	0	41.9	0.095242	26696-59-3	0.013047258
neg_4683	L-Tryptophan	metab_10116	PW_C000741;HMDB0000929;MJDBOTE0000426	B(i)	C00078	203.0823	3.0766	neg	M-H	C11H12N2O2	97.4	0	-1.4318557	73-22-3	0.000881023
neg_4684	2-Methylglutaric Acid	metab_10117	HMDB0000422;MJDBOTE0000600	B(i)	-	145.0501	3.0766	neg	M-H	C6H10O4	85.3	0	-3.7904568	617-62-9	0.006496956
neg_4686	2',5'-Dideoxyadenosine	metab_10119	HMDB0245554	B(ii)	-	270.0776	3.0685	neg	M+Cl	C10H13N5O2	0	68.2	5.29711677	-	0.002493328
neg_4706	Simmondsin	metab_10140	HMDB0030394	B(ii)	-	356.1356	3.0285	neg	M-H2O-H	C16H25NO9	0	51	1.23470942	51771-52-9	0.003729779
neg_471	LysoPA(0:0/16:0)	metab_10144	HMDB0007849	B(ii)	-	431.2218	6.162	neg	M-H2O-H, M+	C19H39O7P	0	45	9.25617971	-	0.023497663
neg_4717	Vanillic acid	metab_10151	HMDB0004815;HMDB0000484;HMDB0240266	B(ii)	C06672	189.0165	3.0125	neg	M+Na-2H	C8H8O4	0	34	-2.7685355	121-34-6;394	0.004415683
neg_4718	2-Pyrocatechuic Acid	metab_10152	HMDB0000397;MJDBOTE0000462	B(i)	C00196	153.0188	3.0125	neg	M-H	C7H6O4	92.4	0	-3.5219109	303-38-8	0.002601893
neg_472	Xi-7-Hydroxyhexadecanedioic acid	metab_10154	HMDB0037830	B(ii)	-	301.2022	6.162	neg	M-H, M+Na-2I	C16H30O5	0	56	0.56308369	-	0.008404924
neg_4723	Clofibryl glucuronide	metab_10158	HMDB0250343	B(ii)	-	427.0193	3.0046	neg	M+K-2H	C16H19ClO9	0	31.7	-2.6275659	-	0.014258398
neg_4724	Ketamine	metab_10159	HMDB0015352	B(ii)	C07525	274.0395	3.0046	neg	M+K-2H	C13H16ClNO	0	57.1	-4.957991	6740-88-1	0.008187157
neg_4726	N-(2-Pyrimidinyl)formamide	metab_10161	HMDB0252442	B(ii)	-	122.0367	3.0046	neg	M-H	C5H5N3O	0	37.5	5.95404219	-	0.012575838
neg_473	Traumatic Acid	metab_10164	HMDB0000933;LMFA01170002	B(i)	C16308	227.1287	6.1149	neg	M-H, M+Na-2I	C12H20O4	83.1	0	-0.6467236	6402-36-4	0.010424645
neg_4732	Ritipenem	metab_10167	HMDB0257244	B(ii)	-	333.0413	2.9966	neg	M+FA-H	C10H12N2O6S	0	44.4	5.33982424	-	0.013085879
neg_4734	Glycyl-Phenylalanine	metab_10169	HMDB0028848	B(ii)	-	221.093	2.9966	neg	M-H	C11H14N2O3	0	35.6	-0.8918018	-	0.009855504
neg_4744	Glucocochlearin	metab_10178	HMDB0302326	B(ii)	C08407	420.0613	2.9806	neg	M+FA-H	C11H21NO9S2	0	38.8	-7.1602857	499-24-1	0.007379681
neg_4745	2,3-dinor-6-oxoprostaglandin F1alpha	metab_10179	HMDB0062618	B(ii)	-	379.1516	2.9806	neg	M+K-2H	C18H30O6	0	53.8	-3.5832112	64700-71-6	0.032767079
neg_4758	10-Formyldihydrofolate	metab_10192	HMDB0006485	B(ii)	C03204	470.1439	2.9646	neg	M-H	C20H21N7O7	0	49.9	2.06654454	28459-40-7	0.165732942
neg_4760	Vanillic acid 4-O-sulfate	metab_10194	HMDB0041788	B(ii)	-	246.9919	2.9646	neg	M-H	C8H8O7S	0	46.8	0.38342582	71235-86-4	0.008563834
neg_4761	Gallic acid	metab_10195	HMDB0005807	B(ii)	C01424	169.0137	2.9646	neg	M-H	C7H6O5	0	60.6	-3.4009236	149-91-7	0.011038349
neg_4766	Libenzapril	metab_10200	HMDB0254077	B(ii)	-	400.1257	2.9487	neg	M+K-2H	C18H25N3O5	0	30.4	-6.398999	-	0.012746686
neg_4767	Beta-D-ribosylnicotinate	metab_10201	HMDB0304540;HMDB0006809	B(ii)	C05841	254.0672	2.9487	neg	M-H	C11H13NO6	0	62.3	0.75741793	4013-6-3	0.013262592

neg_4638	3	2.757	2.757	2.797	3.128	2.752	5.107	5.157	5.159	5.174	5.175	5.14	4.817	4.827	4.797	4.87	4.878	4.86	4.873	4.834	4.868	4.874	4.886	4.88	5.158	5.093	5.023	5.142	5.115	5.052	4.765	4.741	4.766
neg_465	6.201	6.128	6.107	6.11	6.108	6.092	4.52	4.642	4.755	4.758	4.772	4.775	4.3	4.653	4.692	4.626	4.639	4.709	4.566	4.481	4.632	4.422	4.553	4.591	4.806	4.852	4.805	4.85	4.843	4.818	5.87	5.866	5.87
neg_4650	5.735	5.724	5.747	5.737	5.757	5.736	5.92	5.935	5.968	5.976	5.975	5.962	5.997	6.059	6.04	6.023	6.043	6.045	6.009	5.985	6.029	6.01	6.011	6.014	5.881	5.907	5.905	5.877	5.889	5.894	5.92	5.933	5.934
neg_4653	5.304	5.292	5.304	5.294	5.282	5.268	6.233	6.265	6.288	6.293	6.295	6.287	6.116	6.149	6.141	6.137	6.149	6.158	6.156	6.158	6.19	6.187	6.179	6.174	6.097	6.064	6.092	6.065	6.068	6.075	5.978	5.976	5.978
neg_4667	5.658	5.656	5.648	5.632	5.607	5.622	5.216	5.232	5.239	5.252	5.272	5.245	5.348	5.388	5.383	5.392	5.382	5.375	5.11	5.134	5.124	5.133	5.127	5.113	5.957	5.936	5.962	5.941	5.951	5.936	5.669	5.667	5.669
neg_4671	5.897	5.91	5.939	5.952	5.941	5.948	5.936	5.97	5.962	6.005	5.995	5.983	6.177	6.217	6.229	6.217	6.226	6.234	5.973	5.946	5.958	5.941	5.942	5.942	5.995	5.993	6.004	5.986	5.992	6.01	5.988	5.999	6
neg_468	6.129	6.073	6.059	6.056	6.071	6.053	5.247	5.32	5.329	5.404	5.366	5.361	4.57	4.733	4.705	4.739	4.694	4.718	4.9	4.833	4.831	4.892	4.825	4.833	5.075	5.036	5.036	5.031	5.031	5.019	5.873	5.873	5.875
neg_4680	6.123	6.124	6.138	6.105	6.107	6.109	6.156	6.191	6.195	6.222	6.218	6.216	6.277	6.313	6.315	6.312	6.317	6.325	6.218	6.217	6.216	6.227	6.218	6.225	6.18	6.17	6.18	6.173	6.179	6.176	6.226	6.222	6.223
neg_4682	4.447	4.446	4.4	4.438	4.389	4.437	4.576	4.603	4.619	4.58	4.605	4.603	4.932	4.993	4.969	4.981	5	5.009	4.822	4.793	4.781	4.794	4.795	4.807	4.979	4.977	4.974	4.982	4.989	4.955	4.786	4.785	4.795
neg_4683	6.428	6.445	6.462	6.444	6.424	6.43	6.438	6.47	6.461	6.514	6.51	6.492	6.613	6.65	6.65	6.653	6.646	6.658	6.483	6.482	6.477	6.488	6.486	6.486	6.471	6.455	6.451	6.459	6.46	6.459	6.506	6.507	6.507
neg_4684	4.941	4.974	4.983	4.937	4.955	4.956	4.959	4.993	5.024	4.993	5.017	5.001	4.961	5.006	5.007	4.982	5.011	5.015	5.007	5.017	5.018	5.003	5.008	4.987	5.076	5.083	5.085	5.069	5.074	5.056	5.043	5.044	5.038
neg_4686	4.555	4.561	4.533	4.562	4.559	4.553	5.314	5.361	5.393	5.389	5.4	5.368	5.072	5.093	5.088	5.08	5.097	5.119	5.3	5.299	5.282	5.298	5.267	5.285	5.229	5.244	5.247	5.25	5.25	5.229	5.318	5.318	5.316
neg_4706	4.649	4.595	4.606	4.594	4.595	4.59	5.709	5.746	5.755	5.762	5.752	5.766	5.586	5.615	5.645	5.622	5.641	5.638	6.269	6.267	6.267	6.262	6.265	6.278	5.595	5.578	5.581	5.591	5.6	5.584	5.777	5.774	5.775
neg_471	5.558	5.601	5.557	5.548	5.577	5.569	5.659	5.831	5.881	5.873	5.835	5.855	5.686	5.541	5.522	5.545	5.539	5.52	5.528	5.597	5.619	5.619	5.611	5.63	5.484	5.221	5.187	5.227	5.264	5.241	4.986	4.968	4.969
neg_4717	4.147	4.132	4.161	4.169	4.149	4.159	4.803	4.841	4.848	4.828	4.84	4.86	4.944	4.972	4.995	4.988	5.014	5.009	4.841	4.89	4.848	4.848	4.834	4.897	4.319	4.264	4.282	4.197	4.296	4.294	4.667	4.663	4.664
neg_4718	5.853	5.824	5.84	5.809	5.816	5.815	6.571	6.597	6.603	6.614	6.616	6.617	6.856	6.889	6.896	6.892	6.894	6.897	6.853	6.848	6.839	6.846	6.846	6.852	6.251	6.251	6.255	6.252	6.247	6.246	6.544	6.542	6.543
neg_472	6.349	6.306	6.296	6.288	6.291	6.271	4.888	5.021	5.085	5.16	5.071	5.088	4.908	4.994	5.003	5.053	5.004	5.033	5.096	4.905	5.01	5.001	4.951	4.997	5.261	5.225	5.25	5.281	5.231	5.19	6.086	6.08	6.087
neg_4723	3.294	3.264	3.264	3.272	3.263	3.26	5.067	5.064	5.075	5.07	5.092	5.075	5.321	5.422	5.411	5.415	5.397	5.39	5.162	5.141	5.16	5.168	5.187	5.181	3.267	3.264	3.261	3.261	3.343	3.254	4.893	4.903	4.904
neg_4724	5.728	5.705	5.704	5.694	5.692	5.693	3.668	3.862	3.26	3.448	3.413	3.369	3.364	3.406	3.399	3.399	3.402	3.403	3.386	3.387	3.385	3.388	3.386	3.388	3.841	3.589	3.961	3.868	3.469	3.857	5.201	5.194	5.196
neg_4726	2.009	1.979	1.979	1.986	1.978	1.975	5.015	5.037	5.047	5.036	5.035	5.063	5.144	5.179	5.202	5.195	5.191	5.19	5.068	5.064	5.048	5.039	5.046	5.058	4.765	4.741	4.758	4.738	4.752	4.734	4.882	4.873	4.883
neg_473	6.348	6.315	6.313	6.306	6.312	6.298	4.061	4.212	4.287	4.412	4.178	4.266	4.098	4.096	4.389	4.416	4.382	4.235	4.328	4.266	4.313	4.325	4.314	4.174	5.065	5.055	5.068	5.043	5.062	5.044	6.038	6.031	6.039
neg_4732	5.174	5.164	5.189	5.165	5.173	5.177	5.142	5.158	5.209	5.232	5.198	5.186	5.26	5.284	5.29	5.305	5.3	5.294	5.235	5.216	5.22	5.215	5.226	5.235	5.239	5.217	5.231	5.243	5.249	5.245	5.254	5.244	5.245
neg_4734	5.315	5.311	5.327	5.29	5.302	5.3	4.812	4.816	4.801	4.826	4.841	4.846	4.965	5.001	5.018	5.023	5.051	5.02	4.919	4.944	4.898	4.939	4.961	4.926	5.022	5.016	5.049	5.043	5.012	5.022	5.082	5.081	5.074
neg_4744	5.766	5.732	5.759	5.742	5.778	5.768	5.997	6.016	6.025	6.032	6.018	6.039	6.007	6.048	6.056	6.061	6.046	6.038	6.065	6.043	6.054	6.047	6.047	6.066	6.355	6.375	6.369	6.364	6.351	6.377	6.15	6.157	6.154
neg_4745	3.644	3.664	3.614	3.654	3.652	3.643	4.709	4.702	4.75	4.729	4.768	4.755	5.313	5.333	5.305	5.306	5.371	5.357	5.339	5.33	5.328	5.332	5.31	5.314	4.955	4.958	5.015	4.929	4.932	4.954	4.852	4.828	4.853
neg_4758	3.607	3.312	3.727	3.395	3.558	3.521	4.788	4.769	4.735	4.665	4.777	4.823	4.662	4.749	4.825	4.779	4.772	4.775	4.766	4.769	4.75	4.754	4.816	4.789	5.137	5.172	5.154	5.095	5.139	5.114	3.034	2.914	2.915
neg_4760	5.794	5.768	5.766	5.743	5.751	5.753	5.551	5.56	5.563	5.573	5.569	5.591	5.73	5.766	5.774	5.765	5.762	5.776	5.681	5.662	5.659	5.671	5.681	5.669	5.741	5.744	5.749	5.748	5.746	5.75	5.749	5.742	5.743
neg_4761	4.262	4.03	3.916	3.812	3.797	3.778	4.717	4.738	4.755	4.749	4.774	4.738	5.552	5.582	5.605	5.589	5.588	5.598	5.105	5.106	5.084	5.116	5.091	5.091	4.747	4.742	4.738	4.74	4.744	4.714	4.847	4.838	4.839
neg_4766	0.5961	1.772	0.1146	0.1163	0.1938	0.1136	5.025	5.072	5.037	5.06	5.051	5.084	5.406	5.431	5.432	5.413	5.446	5.416	5.85	5.855	5.841	5.852	5.839	5.871	5.243	5.244	5.212	5.266	5.23	5.258	5.323	5.322	5.313
neg_4767	4.352	4.336	4.317	4.293	4.379	4.327	2.836	2.737	2.749	2.747	2.755	2.761	3.441	3.431	3.273	3.271	3.483	2.891	2.878	2.94	2.873	3.471	3.384	3.391	4.905	4.902	4.909	4.922	4.9	4.882	4.49	4.479	4.48

neg_4783	Mergepta	metab_10218	HMDB0254467	B(ii)	-	473.1145	2.918	neg	2M-H	C7H15N3O2S2	0	65	1.39468587	-	0.067386936	1.403	2.597	1.504	1.381	1.373	1.37	4.45	4.157	3.81	4.071	3.511	4.29	3.529	4.106	3.501	3.506	1.484	1.485	1.469	1.47	1.468	1.471	1.469	1.47	5.433	5.438	5.429	5.414	5.406	5.43	4.446	4.443	4.393
neg_4788	Urolithin B 3-O-glucuronide	metab_10222	HMDB0041787	B(ii)	-	409.055	2.91	neg	M+Na-2H	C19H16O9	0	38.3	2.38678026	-	0.015237804	0.5819	2.769	1.499	2.633	0.5592	2.614	5.085	5.058	5.106	5.097	5.124	5.119	5.85	5.884	5.894	5.867	5.886	5.885	5.773	5.779	5.774	5.761	5.758	5.774	5.015	5.033	5.066	5.038	5.042	5.029	5.333	5.32	5.328
neg_4796	Fleroxacin N-oxide	metab_10231	HMDB0252300	B(ii)	-	366.1059	2.8956	neg	M-H2O-H	C17H18F3N3O4	0	44.3	-3.0146968	-	0.008314977	6.21	6.184	6.203	6.148	6.169	6.19	3.321	3.349	3.714	3.677	3.553	3.595	3.368	3.41	3.403	3.403	3.406	3.407	3.39	3.392	3.389	3.392	3.39	3.392	3.674	3.292	3.687	4.091	3.287	3.294	5.607	5.6	5.601
neg_4797	Saligenin-Beta-D-Glucopyranoside	metab_10232	HMDB0003546;MJDBOTE0001478	B(i)	C01451	285.0983	2.8956	neg	M-H	C13H18O7	83.9	0	1.20515449	138-52-3	0.016870062	2.712	2.748	2.723	2.869	2.661	2.68	5.405	5.427	5.445	5.446	5.446	5.465	6.06	6.103	6.097	6.086	6.109	6.096	5.755	5.743	5.749	5.765	5.747	5.767	5.01	5.058	5.062	5.003	5.03	5.026	5.462	5.449	5.45
neg_48	Sorbitan laurate	metab_10235	HMDB0029885;LMFA07011017	B(ii)	-	327.2181	6.0722	neg	M-H, M+Na-2	C18H34O6	0	41.5	1.25434678	1338-39-2	0.009600028	6.969	6.935	6.922	6.918	6.924	6.909	6.301	6.328	6.36	6.383	6.347	6.366	5.975	6.034	6.029	6.05	6.046	6.059	6.175	6.141	6.16	6.134	6.15	6.141	7.141	7.109	7.105	7.112	7.105	7.104	7.001	6.994	7.001
neg_480	Decanedioic acid	metab_10236	HMDB0000792;LMFA01170006	B(i)	C08277	201.1129	5.994	neg	M-H, M+Na-2	C10H18O4	69.1	0	-1.6859902	111-20-6	0.010717722	5.339	5.305	5.298	5.291	5.29	5.263	5.224	5.267	5.327	5.342	5.293	5.327	5.182	5.22	5.259	5.258	5.254	5.238	5.269	5.252	5.262	5.286	5.262	5.265	5.15	5.091	5.098	5.106	5.086	5.101	5.476	5.467	5.468
neg_4806	Benzyl sulfate	metab_10243	HMDB0034782	B(ii)	-	233.0126	2.8816	neg	M+FA-H	C7H8O4S	0	78	0.19498331	-	0.006990679	6.447	6.411	6.434	6.383	6.402	6.409	7.15	7.164	7.181	7.176	7.189	7.197	7.344	7.374	7.392	7.364	7.385	7.374	7.353	7.348	7.346	7.354	7.346	7.357	7.643	7.651	7.647	7.636	7.657	7.646	7.439	7.433	7.435
neg_4810	Aconine	metab_10247	HMDB0247960	B(ii)	C19990	544.2753	2.87	neg	M+FA-H	C25H41NO9	0	48.9	-2.1103376	509-20-6	0.018587601	5.458	5.417	5.447	5.441	5.438	5.474	4.737	4.785	4.797	4.795	4.803	4.809	4.901	4.942	4.936	4.936	4.938	4.939	4.923	4.924	4.922	4.925	4.923	4.924	4.827	4.824	4.821	4.821	4.819	4.814	4.847	4.861	4.862
neg_4815	Ginnalin B	metab_10252	HMDB0252723	B(ii)	-	315.0727	2.87	neg	M-H	C13H16O9	0	43.4	1.80769849	-	0.00394083	4.507	4.43	4.374	4.408	4.51	4.372	5.432	5.44	5.456	5.441	5.469	5.467	6.365	6.407	6.424	6.385	6.411	6.401	6.167	6.168	6.169	6.167	6.165	6.182	5.68	5.698	5.67	5.67	5.689	5.673	5.823	5.82	5.82
neg_4817	4-Hydroxyhippuric Acid	metab_10254	HMDB0013678	B(i)	-	194.0455	2.87	neg	M-H	C9H9NO4	30.3	0	-1.9215966	2482-25-9	0.00897277	4.727	4.695	4.717	4.681	4.691	4.685	5.065	5.068	5.078	5.076	5.103	5.115	5.42	5.439	5.483	5.436	5.472	5.46	5.249	5.253	5.251	5.272	5.258	5.285	4.944	4.949	4.911	4.898	4.956	4.939	5.094	5.087	5.088
neg_4820	Imidazolone A	metab_10258	HMDB0253421	B(ii)	-	299.1363	2.862	neg	M-H2O-H	C12H22N4O6	0	38.6	0.64609238	-	0.002796723	5.716	5.569	5.563	5.528	5.519	5.537	2.8	2.847	2.859	2.858	2.866	2.872	2.963	3.005	2.998	3.222	3.001	3.317	2.985	2.986	2.984	2.987	2.985	2.987	2.889	2.887	2.883	2.884	2.882	2.877	5.39	5.391	5.392
neg_4834	Aminohippuric acid	metab_10272	HMDB0001867	B(i)	D06890	193.0614	2.8346	neg	M-H	C9H10N2O3	62.6	0	-2.2996784	61-78-9	0.007061608	6.029	6.013	6.027	6.001	5.999	6.01	4.325	4.379	4.303	4.311	4.357	4.308	4.8	4.822	4.866	4.824	4.851	4.853	4.895	4.913	4.864	4.871	4.911	4.918	4.175	4.059	4.108	3.985	4.116	4.199	5.593	5.587	5.589
neg_4835	Fluorescein 5-maleimide	metab_10273	HMDB0252363	B(ii)	-	472.0702	2.8291	neg	M+FA-H	C24H13NO7	0	68.4	6.63406762	-	0.024403498	4.692	4.645	4.659	4.687	4.662	4.712	3.243	3.236	3.248	3.247	3.255	3.261	3.352	3.394	3.387	3.387	3.39	3.391	3.374	3.376	3.373	3.376	3.374	3.376	3.278	3.276	3.272	3.273	3.271	3.266	4.57	4.552	4.571
neg_4837	(E)-3-(2,3-Dihydroxyphenyl)-2-propenoic a	metab_10275	HMDB0032057	B(ii)	C12623	225.0402	2.8291	neg	M+FA-H	C9H8O4	0	63.9	-1.292379	38481-04-8	0.012818661	4.758	4.662	4.673	4.669	4.617	4.614	4.319	4.36	4.44	4.442	4.488	4.423	3.849	3.776	4.057	3.824	3.964	4.002	4.265	4.187	4.348	4.304	4.287	4.41	4.434	4.427	4.444	4.464	4.354	4.418	4.621	4.62	4.63
neg_4842	4-Hydroxychalcone	metab_10280	HMDB0032584	B(ii)	C14231	447.1633	2.8175	neg	2M-H	C15H12O2	0	72.7	6.98251223	20426-12-4	0.002836741	4.48	4.378	4.349	4.368	4.33	4.412	5.406	5.456	5.457	5.449	5.465	5.47	5.109	5.126	5.215	5.066	5.138	5.15	5.328	5.336	5.359	5.369	5.356	5.374	5.4	5.394	5.37	5.367	5.393	5.383	5.433	5.431	5.434
neg_4844	6-Hydroxy flavin adenine dinucleotide	metab_10282	HMDB0011612	B(ii)	-	822.129	2.8105	neg	M+Na-2H	C27H33N9O16P2	0	42.4	2.90565472	52301-43-6	0.007083427	5.68	5.637	5.719	5.667	5.653	5.724	3.64	3.687	3.699	3.698	3.705	3.712	3.803	3.845	3.838	3.838	3.841	3.842	3.825	3.827	3.824	3.827	3.825	3.827	3.729	3.727	3.723	3.724	3.722	3.717	3.75	3.749	3.755
neg_4846	Tigemonam	metab_10284	HMDB0259088	B(ii)	-	482.0266	2.8105	neg	M+FA-H	C12H15N5O9S2	0	45.1	-6.246697	-	0.010622387	6.476	6.479	6.532	6.455	6.504	6.518	4.899	5.009	4.963	4.997	4.936	5.007	3.729	4.188	4.303	3.966	4.152	3.726	4.027	3.427	4.045	3.913	3.653	3.427	3.33	3.328	3.324	3.324	3.323	3.318	6.033	6.04	6.041
neg_4848	Vidarabine	metab_10286	HMDB0014340	B(ii)	C07195	266.0895	2.8105	neg	M-H	C10H13N5O4	0	43.5	0.13901586	24356-66-9	0.018114737	4.779	4.79	4.828	4.756	4.776	4.784	4.142	4.19	4.202	4.2	4.208	4.214	4.306	4.348	4.341	4.341	4.343	4.345	4.328	4.329	4.327	4.33	4.328	4.33	4.232	4.23	4.226	4.226	4.225	4.22	4.265	4.252	4.266
neg_4850	1-Methyl-5-nitro-1H-imidazole-2-methan	metab_10289	HMDB0247667	B(ii)	-	156.0409	2.8105	neg	M-H	C5H7N3O3	0	32.8	-3.8313508	-	0.190970076	2.83	2.8	2.8	2.807	2.799	2.795	4.595	4.666	4.742	4.688	4.734	4.678	4.686	4.709	4.762	4.668	4.758	4.781	4.817	4.767	4.867	4.834	4.807	4.872	3.55	3.78	3.576	3.937	3.758	3.614	2.823	2.974	2.978
neg_4852	Alpelisib	metab_10291	HMDB0304878	B(ii)	-	486.1388	2.7975	neg	M+FA-H	C19H22F3N5O2S	0	41.7	-9.134502	-	0.024652343	4.675	4.578	4.642	4.549	4.445	4.555	4.782	4.94	4.924	4.81	4.939	4.816	5.109	5.18	5.158	5.115	5.182	5.106	5.11	5.156	5.132	5.112	5.133	5.222	4.986	5.07	5.031	5.04	4.973	4.994	4.975	4.975	4.994
neg_4859	(5R)-3-Ethylsulfanyl-6-(1-hydroxyethyl)-7-	metab_10297	HMDB0257542	B(ii)	-	274.0234	2.7895	neg	M-H	C10H13NO4S2	0	62.6	7.5587543	-	0.005054247	5.004	5.021	5.082	4.954	5.032	5.077	5.891	5.933	5.957	5.926	5.941	5.921	5.878	5.913	5.981	5.892	5.95	5.954	5.868	5.888	5.915	5.847	5.898	5.938	5.877	5.91	5.908	5.865	5.912	5.932	5.779	5.782	5.783
neg_4862	5,7-Dimethoxyflavone	metab_10301	HMDB0036620;LMPK12110188	B(ii)	C10029	623.1949	2.7825	neg	2M+Hac-H	C17H14O4	0	44.9	4.71824615	21392-57-4	0.016922874	5.354	5.338	5.393	5.293	5.31	5.337	4.884	4.949	4.952	4.905	4.869	4.928	3.879	4.439	4.134	4.554	4.203	4.229	4.386	3.996	3.898	4.183	3.704	4.182	3.304	3.302	3.299	3.299	3.297	3.532	5.605	5.592	5.605
neg_4864	Xanthosine	metab_10303	PW_C000203;HMDB0000299;MJDBOTE0001063	B(ii)	C01762	321.0266	2.7825	neg	M+K-2H	C10H12N4O6	0	60.7	8.09939176	146-80-5	0.012041733	3.744	3.718	4.11	3.775	4.018	4.118	5.101	5.145	5.183	5.12	5.155	5.15	5.107	5.144	5.168	5.091	5.182	5.188	5.073	5.108	5.113	5.104	5.089	5.164	5.119	5.122	5.108	5.044	5.12	5.115	4.974	4.982	4.983
neg_4868	(2R,3R)-3-[[(2S)-1-[4-(Diaminomethyliden	metab_10307	HMDB0251665	B(ii)	-	402.2	2.7746	neg	M+FA-H	C15H27N5O5	0	91.1	1.65631782	-	0.030858121	4.853	4.936	4.912	4.914	4.91	4.87	3.879	3.926	3.938	3.937	3.945	3.951	4.043	4.084	4.077	4.078	4.08	4.081	4.064	4.066	4.063	4.067	4.064	4.066	4.031	3.966	4.04	3.995	4.082	4.007	4.493	4.493	4.516
neg_4873	Histidyltryptophan	metab_10313	HMDB0028896	B(ii)	-	386.1462	2.7655	neg	M+FA-H	C17H19N5O3	0	33.4	-2.2120167	23403-90-9	0.004648248	2.985	2.955	2.955	3.317	2.954	2.951	5.356	5.381	5.36	5.394	5.385	5.378	5.458	5.448	5.485	5.453	5.474	5.477	5.621	5.616	5.608	5.632	5.616	5.624	5.425	5.387	5.397	5.424	5.393	5.378	5.421	5.417	5.421

neg_4874	Sumiki's acid	metab_10314	HMDB0002432	B(ii)	C20448	343.0665	2.7655	neg	2M+Hac-H	C6H6O4	0	31.7	-2.1199305	6338-41-6	0.002765432	5.579	5.548	5.542	5.517	5.53	5.503	4.887	4.958	4.952	4.953	4.977	4.966	4.652	4.614	4.572	4.602	4.567	4.614	4.804	4.78	4.856	4.869	4.762	4.904	3.203	3.002	2.999	3.585	2.998	2.992	4.736	4.738	4.736
neg_4876	Luteolin 7-glucuronide	metab_10315	HMDB0240541;LMPK12110644	B(ii)	C03515	443.0643	2.7593	neg	M-H2O-H	C21H18O12	0	35	5.03273497	-	0.193990492	3.755	3.725	3.725	3.732	3.724	3.721	5.002	4.968	5.058	5.028	5.098	4.961	4.623	4.646	4.679	4.713	4.716	4.742	4.79	4.758	4.867	4.786	4.813	4.843	3.796	3.92	3.955	3.965	4.066	4.036	3.748	3.92	3.861
neg_4886	2-Imidazolidinone, 4-hydroxy-1-(5-nitro-2	metab_10325	HMDB0246460	B(ii)	-	266.9605	2.7453	neg	M+K-2H	C6H6N4O4S	0	42.5	4.14212841	-	0.01100432	4.831	4.801	4.801	4.808	4.8	4.796	5.508	5.559	5.588	5.58	5.581	5.556	5.401	5.427	5.479	5.427	5.458	5.441	5.538	5.538	5.57	5.519	5.518	5.573	4.803	4.876	4.876	4.86	4.829	4.855	5.133	5.142	5.14
neg_4897	Epicylindrospermopsin	metab_10335	HMDB0247246	B(ii)	-	436.0928	2.7299	neg	M+Na-2H	C15H21N5O7S	0	30.2	4.69984539	-	0.026366209	1.899	1.869	1.869	1.877	1.868	1.865	2.927	2.243	1.842	3.565	1.848	2.638	4.516	4.333	4.502	4.36	4.371	4.286	3.712	3.896	3.705	3.749	4.033	3.69	5.303	5.23	5.26	5.246	5.21	5.286	4.681	4.658	4.666
neg_4898	(6-Aminoquinolin-2-yl) N-(2,5-dihydroxyp	metab_10336	HMDB0260166	B(ii)	-	361.0797	2.7299	neg	M+FA-H	C14H12N4O5	0	49.7	2.25123384	-	0.043907605	3.241	1.936	2.798	1.943	2.918	3.063	5.255	5.299	5.283	5.298	5.319	5.283	5.249	5.276	5.29	5.267	5.251	5.301	5.216	5.226	5.202	5.188	5.235	5.208	1.938	3.146	3.105	3.299	3.646	3.891	4.894	4.896	4.861
neg_49	Adenosine 5'-Monophosphate	metab_10338	HMDB0000045;MJDBOTE0000497	B(i)	C00020	346.0562	0.7336	neg	M-H, M+FA-H,	C10H14N5O7P	93.5	0	1.2171288	61-19-8	0.005352382	6.927	6.922	6.906	6.916	6.891	6.922	6.77	6.776	6.809	6.811	6.8	6.804	6.829	6.855	6.848	6.85	6.847	6.853	6.619	6.605	6.603	6.592	6.617	6.603	6.373	6.365	6.35	6.361	6.362	6.359	6.805	6.809	6.81
neg_4900	Azimexon	metab_10340	HMDB0248804	B(ii)	-	239.1149	2.7299	neg	M+FA-H	C9H14N4O	0	30.5	-0.0814023	-	0.041065622	4.872	4.752	4.697	4.635	4.672	4.687	2.716	2.763	2.775	2.774	2.782	2.788	2.879	2.921	2.914	2.914	2.917	2.918	3.026	2.902	2.9	2.903	2.901	2.975	2.805	2.803	2.799	2.8	2.798	2.793	4.64	4.67	4.672
neg_4902	6-Hydroxynicotinic Acid	metab_10341	HMDB0002658;MJDBOTE0000658	B(i)	C01020	138.019	2.7299	neg	M-H	C6H5NO3	83.2	0	-4.4740509	5006-66-6	0.006551903	4.463	4.438	4.346	4.214	4.324	4.375	2.932	2.674	3.079	3.252	2.551	2.84	2.977	3.055	2.611	3.019	2.59	2.449	2.797	2.434	3.209	2.574	2.432	2.943	3.139	2.984	2.331	3.044	2.329	2.422	4.288	4.288	4.293
neg_4911	3,5-Dihydroxyphenylglycine	metab_10349	HMDB0243702	B(ii)	-	228.0513	2.7173	neg	M+FA-H	C8H9NO4	0	31.7	-0.3984305	-	0.009553522	4.266	4.214	4.181	4.221	4.154	4.17	4.168	4.231	4.208	4.165	4.132	4.208	4.887	4.881	4.918	4.905	4.891	4.933	4.775	4.737	4.754	4.704	4.773	4.739	5.246	5.208	5.221	5.227	5.203	5.211	4.893	4.885	4.886
neg_4914	Arabinosylhypoxanthine	metab_10351	HMDB0003040	B(ii)	-	249.063	2.7093	neg	M-H2O-H	C10H12N4O5	0	48.8	0.22492114	7013-16-3	0.004437468	4.784	4.806	4.794	4.753	4.788	4.802	4.885	4.959	4.978	4.935	4.988	4.912	4.841	4.88	4.945	4.925	4.879	4.91	4.921	4.948	4.939	4.903	4.921	4.946	5.195	5.194	5.201	5.162	5.202	5.199	5.075	5.072	5.076
neg_4923	(R)-Methysticin	metab_10361	HMDB0033590	B(ii)	C09952	309.0556	2.7013	neg	M+Cl	C15H14O5	0	37.4	7.5078978	495-85-2	0.004717617	5.414	5.591	5.61	5.574	5.621	5.627	2.258	2.568	2.664	2.36	1.608	2.531	1.508	1.548	2.162	1.681	1.544	1.545	2.288	1.531	1.528	1.531	1.529	2.202	3.707	3.868	3.904	3.876	3.978	3.954	4.859	4.858	4.855
neg_4930	Hexahydropyrrolo[1,2-a]pyrazine-1,4-dione	metab_10368	HMDB0253141	B(ii)	-	353.1472	2.6934	neg	2M+FA-H	C7H10N2O2	0	35.1	1.6216009	-	0.009217476	5.035	5.048	5.045	5.013	5.044	5.056	3.459	3.496	3.734	3.703	3.5	3.681	3.16	3.201	3.194	3.195	3.504	3.198	3.181	3.183	3.181	3.184	3.181	3.183	5.144	5.124	5.161	5.119	5.122	5.144	4.953	4.946	4.947
neg_4933	2-Pyrrolidinone	metab_10371	HMDB0002039	B(ii)	C11118	229.1193	2.6934	neg	2M+Hac-H	C4H7NO	0	36.3	-0.6281773	616-45-5	0.010089116	3.775	3.679	3.712	3.592	3.647	3.657	3.976	4.003	3.893	3.955	4.033	4.042	2.871	3.043	3.323	2.906	2.908	2.909	4.85	4.85	4.831	4.818	4.795	4.81	4.677	4.682	4.685	4.673	4.694	4.659	4.49	4.482	4.483
neg_4934	Zalcitabine	metab_10372	HMDB0015078;HMDB0037019;PW_C009386;HMDB0	B(ii)	C07207	192.0774	2.6934	neg	M-H2O-H	C9H13N3O3	0	63.1	-1.9261308	5718-77-4;74	0.022545902	5.042	4.967	4.909	4.868	4.883	4.908	2.282	2.774	3.325	3.195	3.32	2.354	3.646	3.681	3.563	3.537	3.502	3.536	3.617	3.387	4.009	3.779	3.51	3.818	2.371	2.708	3.23	2.366	3.228	2.747	5.118	5.101	5.119

neg_4944	3-Hydroxyindolin-2-one-sulfate	metab_10382	HMDB0304928	B(ii)	-	227.9971	2.6854	neg	M-H	C8H7NO5S	0	32.8	-0.3749603	-	0.006732095	3.651	3.523	3.509	3.507	3.688	3.531	4.724	4.752	4.759	4.755	4.739	4.702	4.964	4.979	5.022	4.973	4.966	4.989	4.852	4.842	4.828	4.775	4.825	4.848	4.519	4.475	4.503	4.537	4.512	4.491	4.574	4.571	4.568
neg_4962	5'-Carboxy meloxicam	metab_10401	HMDB0060779	B(ii)	-	401.987	2.6698	neg	M+Na-2H	C14H11N3O6S2	0	48.2	9.01198145	130262-93-0	0.019796886	4.141	4.111	4.111	4.119	4.11	4.107	5.398	5.446	5.468	5.482	5.439	5.449	5.657	5.686	5.667	5.673	5.668	5.648	5.776	5.786	5.734	5.744	5.768	5.75	5.64	5.614	5.617	5.618	5.597	5.634	4.149	4.134	4.135
neg_4965	4,6-Dihydroxy-2-quinolinecarboxylic acid	metab_10404	HMDB0033528	B(ii)	C08480	204.0299	2.6698	neg	M-H	C10H7NO4	0	65.3	-1.5642427	-	0.003689294	4.376	4.317	4.276	4.312	4.293	4.277	5.082	5.127	5.123	5.151	5.138	5.119	5.253	5.278	5.309	5.288	5.262	5.285	5.211	5.201	5.189	5.187	5.203	5.177	4.826	4.836	4.839	4.827	4.829	4.82	4.902	4.899	4.901
neg_4973	(2S)-1-[2-[[(2S)-Pyrrolidine-2-carbonyl]am	metab_10413	HMDB0260136	B(ii)	-	314.1363	2.6619	neg	M+FA-H	C12H19N3O4	0	43	1.98122378	-	0.02428205	4.714	4.655	4.569	4.498	4.556	4.605	3.026	3.074	3.086	3.084	3.092	3.098	3.19	3.231	3.224	3.225	3.227	3.228	3.212	3.213	3.211	3.214	3.212	3.213	3.411	3.406	3.11	3.418	3.109	3.36	4.349	4.368	4.35
neg_4975	Cytosine deoxyribonucleoside	metab_10415	HMDB0250725;HMDB0000014	B(ii)	C00881	272.0893	2.6619	neg	M+FA-H	C9H13N3O4	0	71.5	2.22538117	951-77-9	0.037037584	4.051	4.028	4.075	4.023	4.02	4.045	4.659	4.612	4.559	4.671	4.47	4.641	4.6	4.689	4.525	4.591	4.507	4.518	4.39	4.444	4.18	4.362	4.275	4.296	4.282	4.122	4.162	4.211	4.116	4.126	4.252	4.224	4.253
neg_4978	Hydromorphone-3-glucoside	metab_10418	HMDB0061144	B(ii)	-	484.1408	2.6539	neg	M+K-2H	C23H29NO8	0	33.1	6.39620456	-	0.012982764	4.146	3.865	3.916	3.769	3.617	3.586	5.415	5.444	5.455	5.473	5.448	5.479	6.405	6.428	6.417	6.428	6.43	6.425	6.368	6.377	6.34	6.355	6.362	6.347	5.455	5.439	5.461	5.453	5.445	5.465	5.688	5.687	5.678
neg_4980	Mivazerol	metab_10421	HMDB0254769	B(ii)	-	216.0777	2.6539	neg	M-H	C11H11N3O2	0	48	-0.7740639	-	0.009418974	3.139	3.465	3.432	3.053	3.359	3.276	3.334	2.9	3.036	3.247	3.229	3.359	3.591	3.863	3.491	3.857	3.487	3.825	3.875	3.55	3.4	3.786	3.502	3.406	4.654	4.675	4.663	4.602	4.676	4.635	4.16	4.152	4.154
neg_4982	Ellagic acid acetyl-arabinoside	metab_10423	HMDB0301706	B(ii)	-	521.0613	2.6459	neg	M+FA-H	C21H16O13	0	45	8.40088477	476-66-4	0.028057063	2.407	2.377	2.377	2.385	2.376	2.373	5.363	5.476	5.509	5.426	5.491	5.376	5.695	5.76	5.854	5.732	5.8	5.823	5.732	5.777	5.797	5.703	5.828	5.794	5.238	5.256	5.321	5.169	5.257	5.366	3.556	3.535	3.536
neg_4987	Seselin	metab_10428	HMDB0034257	B(ii)	C09312	263.0499	2.6459	neg	M+Cl	C14H12O3	0	30.3	7.96238218	523-59-1	0.004558041	5.37	5.367	5.369	5.34	5.354	5.356	4.001	3.86	3.927	4.01	3.85	4.167	2.648	2.781	2.803	3.184	3.076	2.876	3.573	3.599	3.606	3.642	3.739	3.412	4.884	4.847	4.875	4.889	4.863	4.883	5.055	5.054	5.051
neg_499	Neopterin	metab_10431	PW_C000673;HMDB0000845;HMDB0000727;HMDB0	B(ii)	C05926	298.0794	2.1356	neg	M+Cl, M+FA-H	C9H11N5O4	0	31	0.18726776	2009-64-5;22	0.00480558	5.601	5.41	5.318	5.368	5.303	5.267	5.149	5.195	5.217	5.244	5.262	5.201	5.161	5.188	5.19	5.186	5.182	5.213	5.204	5.213	5.187	5.195	5.19	5.211	4.221	4.08	4.192	4.226	4.129	4.15	5.085	5.085	5.081
neg_5002	N-Oleoyl Glycine	metab_10445	LMFA08020082;HMDB0241916;HMDB0013631	B(ii)	-	465.129	2.6223	neg	M+Na-2H	C20H21FN6O5	0	32.7	-3.1988019	2601-90-3	0.00799415	3.729	3.699	3.699	3.707	3.698	3.695	4.702	4.725	4.666	4.755	4.716	4.718	4.225	3.817	4.167	4.22	4.102	4.231	5.715	5.709	5.675	5.706	5.722	5.69	6.339	6.316	6.292	6.355	6.312	6.312	5.584	5.578	5.579
neg_5010	Betanin	metab_10454	HMDB0029408	B(ii)	C08540	595.1437	2.6063	neg	M+FA-H	C24H26N2O13	0	51	3.68896782	7659-95-2	0.00914892	5.531	5.529	5.528	5.529	5.475	5.497	4.072	3.909	3.807	4.129	3.82	3.969	3.727	3.295	3.303	3.567	3.271	3.306	3.508	3.257	3.586	3.599	3.401	3.257	3.475	3.162	3.153	3.154	3.255	3.147	5.157	5.156	5.164
neg_5024	Pyrazosulfuron-ethyl	metab_10469	HMDB0256958	B(ii)	C18444	395.0758	2.5983	neg	M-H2O-H	C14H18N6O7S	0	35.3	-5.1116989	93697-74-6	0.007705464	3.816	3.892	4.183	4	3.929	4.095	3.7	4.231	4.503	3.658	4.085	4.033	4.491	4.383	4.824	4.676	4.899	4.88	4.592	4.89	4.819	4.705	4.919	4.822	5.636	5.811	5.753	5.498	5.739	5.816	5.058	5.055	5.052
neg_5028	DTDP-4-acetamido-4,6-dideoxy-D-galacto	metab_10473	HMDB0012222	B(ii)	-	634.1088	2.5904	neg	M+FA-H	C18H29N3O15P2	0	59.1	5.4518085	-	0.009727614	6.37	6.348	6.341	6.343	6.341	6.317	4.588	4.601	4.692	4.591	4.592	4.679	3.577	3.397	3.233	3.233	3.236	3.237	3.352	3.942	3.637	3.299	3.797	3.222	5.37	5.34	5.348	5.369	5.368	5.375	5.935	5.942	5.943
neg_503	Spongothymidine	metab_10475	MJDBOTE0000613	B(i)	C16744	257.0781	2.1963	neg	M-H, M+FA-H	C10H14N2O6	32.6	0	0.89037093	605-23-2	0.001828619	5.769	5.688	5.67	5.713	5.684	5.657	4.453	4.563	4.546	4.476	4.578	4.506	3.854	4.009	4.106	3.933	3.857	3.899	4.252	4.224	4.081	4.265	4.122	4.082	4.737	4.806	4.811	4.728	4.831	4.768	5.438	5.437	5.439
neg_5036	4,5-seco-dopa	metab_10482	HMDB0304164	B(ii)	C17758	210.0405	2.5833	neg	M-H2O-H	C9H11NO6	0	42.8	-1.1315091	-	0.007290757	4.036	4.01	3.859	3.847	3.898	3.938	4.621	4.666	4.681	4.647	4.654	4.685	5.763	5.797	5.787	5.788	5.794	5.78	5.515	5.534	5.496	5.505	5.524	5.507	4.574	4.591	4.551	4.605	4.598	4.548	4.968	4.962	4.964
neg_5062	Nona-2,6-dienedioyl-CoA	metab_10510	HMDB0301523	B(ii)	-	465.5832	2.5513	neg	M-2H	C30H46N7O19P3S	0	74.1	3.05167686	-	0.009168914	6.07	6.079	6.061	6.068	6.071	6.071	3.814	3.775	4.094	3.839	3.807	3.975	4.076	4.24	4.033	4.017	4.137	4.272	4.052	3.889	3.867	4.055	4.182	3.476	4.763	4.771	4.766	4.757	4.809	4.73	5.825	5.832	5.825
neg_5063	1,5-Naphthalene diisocyanate	metab_10511	HMDB0244232	B(ii)	C19464	246.9922	2.5513	neg	M+K-2H	C12H6N2O2	0	44.2	3.29505536	3173-72-6	0.007201462	4.55	4.481	4.568	4.52	4.491	4.403	6.089	6.132	6.142	6.14	6.133	6.144	6.144	6.175	6.15	6.159	6.171	6.155	6.19	6.206	6.172	6.183	6.18	6.179	5.287	5.306	5.273	5.306	5.298	5.29	5.655	5.649	5.651
neg_5064	3-Chloro-L-Tyrosine	metab_10512	HMDB0001885;MJDBOTE0000598	B(i)	-	214.0275	2.5513	neg	M-H	C9H10ClNO3	94.6	0	-0.8935101	7423-93-0	0.0057863	2.52	2.49	2.49	2.498	2.489	2.486	2.481	3.103	3.019	3.053	2.844	2.475	3.044	2.608	2.601	2.602	2.604	2.727	3.144	2.607	3.123	2.906	3.134	2.652	5.527	5.513	5.486	5.549	5.509	5.499	4.567	4.564	4.562
neg_508	Aminofructose 6-phosphate	metab_10529	HMDB0060436	B(ii)	C12214	258.0387	2.2214	neg	M-H, 2M-H	C6H14NO8P	0	31.8	1.15551618	-	0.006921642	2.526	2.496	2.495	2.503	2.495	2.491	4.338	4.362	4.349	4.323	4.377	4.371	5.238	5.255	5.233	5.22	5.224	5.229	5.098	5.081	5.063	5.096	5.066	5.08	6.105	6.083	6.069	6.1	6.085	6.074	5.246	5.241	5.247
neg_5080	3-O-Methyl-a-methyldopa	metab_10530	HMDB0060747	B(ii)	-	232.0591	2.5274	neg	M+Na-2H	C10H13NO4	0	45.7	-0.1212538	-	0.004075646	5.579	5.546	5.551	5.548	5.556	5.541	6.15	6.193	6.213	6.19	6.201	6.204	6.276	6.31	6.261	6.281	6.306	6.274	6.225	6.247	6.199	6.204	6.212	6.211	5.956	5.943	5.93	5.96	5.967	5.948	5.917	5.914	5.915
neg_5086	4-Acetamido-2-amino-6-nitrotoluene	metab_10536	HMDB0060384	B(ii)	C16420	254.0785	2.5118	neg	M+FA-H	C9H11N3O3	0	33.2	1.2560712	-	0.006622959	4.081	4.053	4.028	3.954	3.968	4.033	4.848	4.909	4.877	4.851	4.854	4.866	5.565	5.615	5.586	5.576	5.593	5.583	5.362	5.388	5.351	5.357	5.362	5.348	5.785	5.798	5.779	5.796	5.801	5.788	5.216	5.21	5.212
neg_5087	L-arginino-succinate	metab_10537	HMDB0304399	B(ii)	-	288.1093	2.5118	neg	M-H	C10H17N4O6-	0	38.1	6.06668927	-	0.018929252	5.402	5.372	5.371	5.379	5.37	5.367	5.787	5.832	5.829	5.817	5.829	5.837	5.588	5.621	5.615	5.607	5.625	5.617	5.532	5.577	5.539	5.542	5.558	5.545	5.664	5.668	5.675	5.671	5.684	5.671	5.409	5.394	5.395
neg_5088	3-Phenyl-2-thioxoimidazolidin-4-one	metab_10538	HMDB0245971	B(ii)	-	237.0339	2.5118	neg	M+FA-H	C9H8N2OS	0	37.4	-0.0623844	-	0.004701409	5.707	5.679	5.693	5.677	5.677	5.686	4.021	3.979	4.044	4.011	4.003	4.022	4.114	4.169	4.172	4.122	4.183	4.11	4.291	4.359	4.293	4.319	4.317	4.281	5.275	5.284	5.275	5.265	5.296	5.267	5.42	5.419	5.423
neg_5092	15-Demethyl plumieride	metab_10543	HMDB0253582	B(ii)	-	437.1062	2.5038	neg	M-H2O-H	C20H24O12	0	81.1	-5.9408396	-	0.017005934	5.002	5.021	5.02	5.043	5.046	5.034	5.91	5.938	5.968	5.956	5.943	5.968	5.957	5.993	5.952	5.953	5.987	5.95	5.957	5.951	5.951	5.954	5.928	5.925	6.162	6.102	6.098	6.163	6.123	6.093	5.565	5.578	5.566
neg_5103	4-[10,15,20-Tris(4-sulfophenyl)-21,22,23,2	metab_10556	HMDB0260011	B(ii)	-	467.0364	2.4729	neg	M-2H	C44H32N4O12S4	0	39.6	-2.7040236	-	0.002788703	6.449	6.434	6.444	6.422	6.429	6.431	5.19	4.021	3.83	3.064	3.323	3.078	4.29	3.211	3.204	3.204	3.207	3.208	3.191	3.192	3.19	3.193	3.191	3.193	3.749	3.62	4.014	3.832	3.282	3.311	6.062	6.064	6.065
neg_5106	Glucosyl 6-hydroxy-2,6-dimethyl-2E,7-octa	metab_10559	HMDB0035822	B(ii)	-	381.1311	2.4729	neg	M+Cl	C16H26O8	0	39	-3.0172786	191548-38-6	0.026236081	4.636	4.606	4.606	4.613	4.605	4.602	4.604	4.627	4.613	4.599	4.636	4.657	5.441	5.485	5.439	5.449	5.471	5.429	5.026	5.042	5.002	5.024	4.984	5.02	5.379	5.377	5.365	5.367	5.38	5.38	4.65	4.649	4.629
neg_5110	5'-(3',4'-Dihydroxyphenyl)-gamma-valerola	metab_10564	HMDB0029191	B(ii)	-	269.0103	2.4649	neg	M-H2O-H	C11H12O7S	0	45.5	-7.8697905	-	0.004851428	4.825	4.811	4.873	4.884	4.878	4.89	5.183	5.198	5.225	5.229	5.205	5.256	5.245	5.278	5.242	5.279	5.278	5.236	5.231	5.22	5.199	5.233	5.189	5.18	5.056	5.065	5.011	5.067	5.06	5.038	4.951	4.954	4.955
neg_5113	Cyclocalopin D	metab_10567	HMDB0039813	B(ii)	-	551.1493	2.4569	neg	M+Cl	C23H32O13	0	57.5	-8.5646389	-	0.000659412	4.836	4.84	4.886	4.716	4.981	4.969	5.939	6.009	6.043	5.995	6.017	6.013	6.136	6.17	6.127	6.143	6.18	6.137	6.018	6.063	6.003	5.995	6.028	6.027	5.448	5.47	5.428	5.465	5.513	5.478	5.521	5.521	5.521
neg_5117	Isopropylmaleic acid	metab_10571	HMDB0012241	B(ii)	C02631	139.0395	2.4569	neg	M-H2O-H	C7H10O4	0	34.3	-3.8665078	44976-69-4	0.00811677	3.302	3.259	3.436	3.508	3.079	3.161	4.832	4.904	4.879	4.872	4.918	4.96	5.275	5.304	5.301	5.299	5.307	5.316	5.074	5.081	5.064	5.091	5.072	5.078	4.817	4.77	4.764	4.774	4.775	4.765	4.588	4.593	4.594
neg_5123	Zanamivir	metab_10578	HMDB0014698	B(ii)	C08095	353.1093	2.4489	neg	M+Na-2H	C12H20N4O7	0	37.5	4.17451483	139110-80-8	0.019843715	3.071	3.041	3.128	3.361	3.373	3.036	5.077	5.168	5.174	5.167	5.172	5.187	5.371	5.391	5.366	5.384	5.404	5.401	5.224	5.289	5.252	5.266	5.241	5.259	4.902	4.895	4.938	4.931	4.964	4.924	4.566	4.574	4.557
neg_5128	(1S,2R,4R)-p-Menth-8-ene-2,10-diol 2-glu	metab_10583	HMDB0039055	B(ii)	-	369.131	2.4409	neg	M+K-2H	C16H28O7	0	39.9	-3.4275989	402593-53-7	0.001617721	5.046	5.057	5.022	5.054	5.06	5.009	6.001	6.04	6.05	6.041	6.055	6.058	6.432	6.469	6.458	6.459	6.458	6.462	6.6	6.595	6.586	6.59	6.585	6.589	7.261	7.252	7.248	7.261	7.257	7.248	6.587	6.587	6.588
neg_5145	N-(5-Amino-2-hydroxybenzoyl)glycine	metab_10602	HMDB0061683	B(ii)	-	209.0565	2.4249	neg	M-H	C9H10N2O4	0	60.6	-1.3029454	-	0.009906026	5.38	5.435	5.417	5.425	5.424	5.392	2.24	2.287	2.318	2.297	2.346	2.452	2.816	3.404	3.616	3.325	2.891	3.026	3.51	3.015	3.029	3.524	3.483	3.105	3.981	3.785	3.989	4.011	3.887	3.988	4.721	4.714	4.722
neg_5147	6,8-Dihydroxypurine	metab_10604	HMDB0001182	B(ii)	-	151.0255	2.4249	neg	M-H	C5H4N4O2	0	35.3	-4.1019177	13231-00-0	0.002695473	4.995	5.06	5.081	5.057	5.057	5.104	4.491	4.639	4.653	4.563	4.545	4.483	4.341	4.295	4.329	4.321	4.342	4.363	4.223	4.245	4.236	4.25	4.228	4.217	4.39	4.46	4.512	4.375	4.466	4.493	4.922	4.923	4.924
neg_515	Inosine	metab_10607	HMDB0000195;HMDB0247301;PW_C000127;MJDBO	B(i)	C00294	267.074	1.9613	neg	M-H, M+Cl	C10H12N4O5	98.4	0	1.82832952	58-63-9	0.002822523	7.224	7.186	7.177	7.186	7.16	7.159	7.294	7.321	7.343	7.357	7.329	7.358	7.351	7.381	7.395	7.384	7.388	7.381	7.428	7.419	7.4	7.426	7.429	7.407	7.141	7.136	7.159	7.156	7.132	7.132	7.27	7.267	7.269
neg_5158	1-Methyluric acid	metab_10615	PW_C001777;HMDB0003099	B(i)	C16359	181.0363	2.4088	neg	M-H	C6H6N4O3	58.1	0	-2.2851861	708-79-2	0.063465493	1.117	1.659	1.089	1.956	1.088	1.885	5.139	5.185	5.167	5.162	5.17	5.187	5.34	5.374	5.384	5.365	5.361	5.37	5.333	5.335	5.295	5.307	5.309	5.304	5.551	5.541	5.532	5.53	5.535	5.54	2.911	2.86	2.869

neg_5162	1,3-Benzodioxole-5,6-diamine	metab_10620	HMDB0244157	B(ii)	-	197.0564	2.4009	neg	M+FA-H	C7H8N2O2	0	32.9	-2.2346839	-	0.012998623	3.485	2.832	3.345	3.197	3.149	3.335	5.309	5.366	5.342	5.348	5.355	5.391	5.376	5.397	5.411	5.398	5.392	5.405	5.556	5.571	5.537	5.569	5.542	5.556	5.685	5.676	5.683	5.683	5.684	5.683	5.134	5.124	5.134
neg_5175	Xylobiose	metab_10633	HMDB0029894	B(ii)	C01630	327.0944	2.3703	neg	M+FA-H	C10H18O9	0	87.9	4.1101817	6860-47-5	0.028920352	2.837	3.2	3.113	3.186	2.739	2.735	4.612	4.582	4.561	4.595	4.579	4.621	4.87	4.92	4.905	4.873	4.881	4.904	5.019	4.953	4.944	5.005	4.893	4.886	5.271	5.243	5.236	5.268	5.24	5.254	4.624	4.603	4.625
neg_5181	Oligoadenylate	metab_10640	HMDB0255951	B(ii)	-	501.5779	2.3623	neg	M-2H	C30H38N15O19P3	0	80.2	2.26698769	-	0.005087471	6.215	6.217	6.206	6.204	6.196	6.199	3.822	4.088	4.209	4.022	4.022	3.849	4.079	4.213	4.381	4.226	4.225	4.367	4.155	4.265	3.925	4.232	4.285	4.263	3.058	3.344	3.052	3.321	3.051	3.68	5.911	5.907	5.908
neg_5184	N-Acetyl-L-glutamate 5-semialdehyde	metab_10643	HMDB0006488	B(ii)	C01250	218.0669	2.3623	neg	M+FA-H	C7H11NO4	0	35.6	-0.7451066	13074-21-0	0.011031776	3.215	2.931	2.13	2.639	2.637	3.003	5.381	5.432	5.429	5.413	5.42	5.438	5.498	5.536	5.535	5.52	5.518	5.54	5.504	5.502	5.499	5.52	5.5	5.503	4.354	4.321	4.363	4.316	4.328	4.296	5.116	5.115	5.124
neg_5187	8-Acetoxypinoresinol	metab_10646	HMDB0033280	B(ii)	C10544	437.119	2.3543	neg	M+Na-2H	C22H24O8	0	36.2	-6.7362322	81426-14-4	0.02035535	4.327	4.277	4.069	4.317	4.271	4.218	4.601	5.083	5.159	4.825	5.121	4.707	5.189	5.212	5.221	5.239	5.281	5.261	5.299	5.276	5.307	5.262	5.274	5.276	5.714	5.856	5.85	5.695	5.841	5.848	5.122	5.121	5.137
neg_5188	(1S,2R,4R,8S)-p-Menthane-2,8,9-triol 2-glu	metab_10647	HMDB0039977	B(ii)	-	385.1625	2.3543	neg	M+Cl	C16H30O8	0	56.3	-2.6932792	378254-07-0	0.004921963	2.644	2.374	3.331	2.355	2.661	2.778	6.361	6.405	6.411	6.414	6.418	6.421	6.156	6.186	6.18	6.172	6.168	6.187	7.084	7.071	7.061	7.073	7.062	7.061	6.311	6.278	6.279	6.303	6.283	6.28	6.306	6.305	6.309
neg_5189	Glycinexylidide	metab_10648	PW_C040579;HMDB0060679	B(ii)	C16569	223.1087	2.3543	neg	M+FA-H	C10H14N2O	0	43.3	-0.4695111	18865-38-8	0.004759606	2.731	2.701	2.701	2.709	2.7	2.697	5.641	5.68	5.688	5.684	5.698	5.693	5.445	5.486	5.472	5.48	5.48	5.488	6.297	6.288	6.274	6.289	6.284	6.272	5.587	5.559	5.55	5.571	5.56	5.554	5.543	5.542	5.546
neg_520	Adenylosuccinate	metab_10659	HMDB0248010;HMDB0000536	B(i)	C03794	462.0681	2.3863	neg	M-2H, M-H	C14H18N5O11P	87.2	0	2.9068672	19046-78-7	0.004548703	7.929	7.912	7.906	7.906	7.897	7.901	6.778	6.819	6.833	6.818	6.818	6.839	6.698	6.722	6.718	6.715	6.714	6.72	6.325	6.326	6.313	6.311	6.305	6.296	6.13	6.131	6.118	6.134	6.128	6.124	7.537	7.54	7.541
neg_5200	4,5-Diacetyl-6,7-dichloro-3',6'-dihydroxysp	metab_10660	HMDB0258000	B(ii)	-	518.9856	2.3217	neg	M+Cl	C24H14Cl2O7	0	44.4	9.47172499	-	0.008316373	6.038	6.054	6.074	6.057	6.092	6.097	5	5.034	5.091	5.028	5.04	5.056	4.778	4.718	4.712	4.684	4.689	4.688	3.475	3.442	3.162	3.939	3.176	3.589	3.067	3.065	3.093	3.15	3.06	3.054	5.831	5.836	5.838
neg_5205	1H-Imidazole-1-ethanol, 2-nitro-alpha-((2	metab_10665	HMDB0249723	B(ii)	-	318.0318	2.3081	neg	M-H2O-H	C9H9F6N3O4	0	32.1	-0.1276409	-	0.026140507	4.542	4.528	4.537	4.591	4.589	4.447	5.125	5.212	5.226	5.209	5.223	5.197	5.219	5.207	5.177	5.209	5.21	5.2	5.26	5.208	5.22	5.224	5.202	5.205	4.946	5.036	5.053	4.935	5.022	5.085	4.958	4.977	4.978
neg_5211	Quinol glucuronide	metab_10672	HMDB0259508	B(ii)	-	571.1327	2.2759	neg	2M-H	C12H14O8	0	38.2	3.90988072	-	0.004408474	5.896	5.878	5.862	5.861	5.858	5.852	4.652	4.731	4.669	4.637	4.655	4.728	4.65	4.821	4.911	4.627	4.783	4.771	4.033	4.319	3.457	3.189	4.031	3.958	4.835	4.82	4.901	4.857	4.821	4.863	5.484	5.487	5.488
neg_5212	Fe(II)-nicotianamine	metab_10673	HMDB0304350	B(ii)	-	284.1255	2.2759	neg	M-H2O-H	C12H21N3O6	0	37.6	0.84410422	-	0.004514027	6.161	6.118	6.1	6.124	6.095	6.091	2.548	2.595	2.608	2.606	2.614	2.62	2.711	2.753	2.746	2.746	2.749	2.75	2.733	2.735	2.732	3.039	2.733	2.735	2.638	2.635	2.632	2.632	2.63	2.625	5.768	5.767	5.771
neg_5219	(1R)-Hydroxy-(2R)-glutathionyl-1,2-dihydr	metab_10680	HMDB0060300	B(ii)	C14791	488.0862	2.2325	neg	M+K-2H	C20H25N3O7S	0	85	-8.239311	-	0.0716296	0.4672	0.4477	1.496	1.076	2.227	1.906	4.903	5.018	5.027	4.964	5.025	4.976	4.414	4.417	4.406	4.33	4.309	4.477	5.697	5.713	5.696	5.673	5.7	5.701	3.674	4.187	4.108	4.105	3.697	4.005	4.677	4.732	4.731
neg_5224	Phenyl-Alanine	metab_10686	HMDB0256428;HMDB0000159	B(ii)	C00079	146.0606	2.2214	neg	M-H2O-H	C9H11NO2	0	33.7	-3.4448382	63-91-2	0.002453506	3.993	3.745	3.657	3.675	3.523	3.653	4.702	4.74	4.741	4.726	4.757	4.754	5.432	5.474	5.459	5.47	5.444	5.462	5.375	5.374	5.351	5.373	5.35	5.362	6.136	6.112	6.093	6.131	6.119	6.106	5.484	5.485	5.486
neg_5226	4-Nitrophenyl 4-guanidinobenzoate	metab_10688	HMDB0246537	B(ii)	-	337.0362	2.2174	neg	M+K-2H	C14H12N4O4	0	36.5	5.74103578	-	0.013633074	4.753	4.769	4.642	4.681	4.712	4.639	4.331	4.729	4.775	4.509	4.795	4.465	5.01	5.023	5.006	5.053	5.027	5.01	4.923	4.856	4.908	4.929	4.924	4.968	5.548	5.614	5.603	5.542	5.622	5.618	5.067	5.056	5.059
neg_5227	L-Agaridoxin	metab_10689	HMDB0029445	B(ii)	-	275.0655	2.2174	neg	M+Na-2H	C11H14N2O5	0	36.2	2.03813387	-	0.000890064	3.532	3.387	3.101	3.424	3.161	3.322	5.152	5.183	5.171	5.194	5.186	5.206	5.922	5.949	5.934	5.945	5.919	5.927	5.841	5.828	5.811	5.827	5.815	5.816	6.524	6.495	6.48	6.517	6.508	6.485	5.883	5.882	5.883
neg_5228	5-Hydroxyindoleacetate	metab_10690	HMDB0000763	B(i)	C05635	190.0506	2.2174	neg	M-H	C10H9NO3	84.1	0	-1.8640525	54-16-0	0.006050833	4.555	4.47	4.402	4.449	4.409	4.387	5.422	5.46	5.473	5.468	5.479	5.494	6.078	6.116	6.107	6.11	6.097	6.104	6.015	6.011	5.991	6.012	5.992	6.007	6.746	6.721	6.706	6.74	6.732	6.716	6.103	6.107	6.108
neg_5244	6-Amino-2-methyl-2H-1,3-benzoxazin-4(	metab_10708	HMDB0247711	B(ii)	-	223.0724	2.1532	neg	M+FA-H	C9H10N2O2	0	33.9	-0.377664	-	0.008230958	2.417	2.387	2.387	2.394	2.386	2.649	3.046	3.195	3.206	3.152	3.287	3.162	3.875	3.957	3.963	3.769	3.954	4.071	3.69	3.567	3.882	3.684	3.691	3.855	5.126	5.156	5.178	5.123	5.177	5.175	4.465	4.458	4.459
neg_5248	Deoxyinosine	metab_10712	HMDB0000071;PW_C000050;MJDBOTE0001081	B(i)	C05512	251.0787	2.1467	neg	M-H	C10H12N4O4	89.4	0	0.29744552	890-38-0	0.006546216	5.466	5.411	5.375	5.417	5.391	5.357	4.643	4.685	4.665	4.666	4.699	4.677	4.57	4.676	4.662	4.623	4.627	4.581	4.835	4.812	4.825	4.874	4.826	4.833	4.805	4.797	4.792	4.794	4.841	4.801	5.15	5.154	5.155
neg_5256	Ciadox	metab_10721	HMDB0250601	B(ii)	-	316.0691	2.1038	neg	M+FA-H	C12H9N5O3	0	36.6	1.22801667	-	0.002792942	4.197	4.259	4.231	4.154	4.175	4.127	5.859	5.894	5.884	5.894	5.897	5.909	5.591	5.603	5.629	5.617	5.602	5.613	5.792	5.79	5.771	5.792	5.782	5.793	5.413	5.404	5.395	5.421	5.404	5.398	5.473	5.47	5.471
neg_5259	Deoxyguanosine	metab_10724	PW_C000058;HMDB0000085;MJDBOTE0000696	B(i)	C00330	266.0898	2.0926	neg	M-H	C10H13N5O4	88.8	0	1.15032827	961-07-9	0.005058777	5.303	5.273	5.209	5.255	5.247	5.177	3.772	3.674	3.53	3.555	3.707	3.617	2.48	2.522	2.515	2.515	2.517	2.519	2.502	3.018	2.501	2.604	3.15	2.971	4.081	4.023	3.863	4.013	4.025	3.981	4.817	4.813	4.814
neg_5263	HBOA trihexose	metab_10729	HMDB0304552	B(ii)	C08283	202.1082	2.0734	neg	M+FA-H	C8H15NO2	0	57.8	-1.85878	-	0.006876312	4.662	4.641	4.644	4.651	4.641	4.608	4.77	4.812	4.827	4.826	4.885	4.843	5.282	5.321	5.322	5.329	5.307	5.317	5.238	5.235	5.209	5.235	5.227	5.231	5.369	5.44	5.432	5.398	5.409	5.427	5.103	5.104	5.109
neg_5272	B-D-Glucuronopyranosyl-(1->3)-a-D-gala	metab_10739	HMDB0039728	B(ii)	-	497.1154	2.0255	neg	M-H2O-H	C18H28O17	0	73.9	1.16845666	-	0.015032991	3.528	3.719	3.682	3.535	3.579	3.62	5.451	5.46	5.453	5.494	5.52	5.508	5.47	5.54	5.533	5.527	5.514	5.507	5.676	5.638	5.622	5.663	5.632	5.645	4.596	4.598	4.7	4.614	4.632	4.576	5.033	5.021	5.029
neg_5284	Cytidylyl-(3',5')-guanosine	metab_10751	HMDB0245649	B(ii)	-	587.1277	1.9933	neg	M-H	C19H25N8O12P	0	71.2	3.37904175	-	0.000658318	6.517	6.482	6.448	6.467	6.447	6.431	4.025	3.769	4.072	4.204	4.033	3.791	3.484	3.17	3.148	4.082	3.367	3.117	3.712	4.367	3.142	3.103	3.666	3.828	4.529	4.654	4.588	4.432	4.642	4.577	6.019	6.019	6.02
neg_5286	Melatonin glucuronide	metab_10753	HMDB0060830	B(ii)	-	429.1273	1.9933	neg	M+Na-2H	C19H24N2O8	0	44.6	-1.5410398	-	0.004478038	5.562	5.525	5.498	5.529	5.467	5.466	6.675	6.709	6.703	6.715	6.722	6.732	6.697	6.747	6.745	6.739	6.733	6.725	6.886	6.879	6.851	6.885	6.869	6.868	6.092	6.087	6.096	6.109	6.082	6.082	6.382	6.382	6.379
neg_5298	4-Methoxyindoxyl sulfate	metab_10766	HMDB0304935	B(ii)	-	290.0345	1.9773	neg	M+FA-H	C9H11NO5S	0	52.9	2.04170294	-	0.007097304	5.405	5.363	5.365	5.344	5.328	5.316	4.991	5.021	5.035	5.035	5.039	5.028	4.999	5.026	5.047	5.021	5.01	5.025	5.012	5.024	4.982	4.974	4.998	4.984	5.639	5.657	5.682	5.673	5.662	5.653	5.373	5.367	5.368
neg_5301	2-Ketobutyric acid	metab_10771	PW_C000003;HMDB0000005	B(ii)	C00109	203.0558	1.9773	neg	2M-H	C4H6O3	0	39.7	-1.6076203	600-18-0	0.003241199	5.235	5.166	5.136	5.139	5.109	5.101	4.59	4.6	4.693	4.686	4.67	4.652	3.673	3.679	3.701	3.697	3.647	3.786	4.113	4.066	4.127	4.155	4.119	4.222	4.688	4.674	4.717	4.711	4.707	4.676	4.886	4.884	4.887
neg_5322	Guanosine	metab_10793	HMDB0000133;PW_C000087;MJDBOTE0000372	B(i)	C00387	282.0848	1.9693	neg	M-H	C10H13N5O5	98.8	0	1.29546821	118-00-3	0.007554676	7.207	7.172	7.165	7.175	7.148	7.144	6.54	6.56	6.582	6.599	6.567	6.602	6.529	6.562	6.573	6.559	6.563	6.56	6.5	6.49	6.476	6.502	6.495	6.485	6.41	6.409	6.434	6.432	6.41	6.406	6.947	6.941	6.942
neg_5326	2,6-diamino-4-hydroxy-5-formamidopyrin	metab_10797	HMDB0011657;HMDB0304046	B(ii)	C04744	150.0416	1.9693	neg	M-H2O-H	C5H7N5O2	0	61.9	-3.4167721	77440-13-2	0.007210653	5.41	5.397	5.394	5.389	5.357	5.386	4.723	4.742	4.771	4.776	4.757	4.792	4.708	4.731	4.749	4.713	4.746	4.737	4.672	4.665	4.653	4.683	4.659	4.644	4.572	4.582	4.59	4.567	4.569	4.569	5.15	5.145	5.15
neg_5329	P-Benzosemiquinone	metab_10800	HMDB0256033	B(ii)	C02389	109.0289	1.9693	neg	M-H	C6H6O2	0	66.8	-5.7598162	-	0.008178554	4.892	4.723	4.616	4.622	4.561	4.524	6.076	6.118	6.137	6.159	6.15	6.169	6.21	6.259	6.261	6.266	6.262	6.257	6.221	6.213	6.188	6.211	6.208	6.208	6.166	6.161	6.169	6.163	6.146	6.135	5.932	5.938	5.932
neg_533	Picraquassioside D	metab_10801	-	B(i)	-	301.0934	2.4649	neg	M-H, M+Cl	C13H18O8	47.9	0	1.53132179	-	0.001582279	2.763	2.901	2.733	2.74	2.732	2.729	5.897	5.944	5.955	5.945	5.965	5.971	6.466	6.504	6.485	6.486	6.496	6.491	6.18	6.188	6.169	6.183	6.171	6.172	5.58	5.551	5.553	5.577	5.558	5.551	5.645	5.645	5.646
neg_5333	UDP-N-acetyl-D-mannosamine	metab_10805	HMDB0013112	B(ii)	C01170	652.083	1.9613	neg	M+FA-H	C17H27N3O17P2	0	38.2	5.37737131	-	0.008110706	6.918	6.883	6.885	6.872	6.874	6.864	4.027	4.116	4.167	3.766	4.046	4.113	2.869	2.91	2.904	2.904	2.906	3.23	2.891	3.554	2.89	3.527	3.459	2.892	4.002	4.268	4.24	4.152	4.192	4.399	6.486	6.49	6.483
neg_5339	2''-O-alpha-L-Rhamnosyl-6-C-quinovopyr	metab_10811	LMPK12110521;HMDB0301973	B(ii)	-	559.1435	1.9613	neg	M-H2O-H	C27H30O14	0	46.6	-3.7888389	-	0.007493094	5.599	5.569	5.569	5.576	5.568	5.565	5.891	5.934	5.983	5.953	5.932	5.953	5.84	5.883	5.895	5.893	5.901	5.928	5.841	5.838	5.815	5.815	5.851	5.83	5.774	5.776	5.802	5.798	5.753	5.772	5.599	5.598	5.593
neg_5343	Natura	metab_10816	HMDB0255470	B(ii)	-	501.1311	1.9613	neg	M-H2O-H	C27H24N2O9	0	38.2	1.5448878	-	0.007834224	3.172	3.142	3.409	3.15	3.141	3.138	5.339	5.36	5.216	5.063	5.013	5.265	5.048	5.085	5.177	5.146	5.12	5.054	4.354	4.782	3.24	4.069	4.758	4.414	6.397	6.399	6.403	6.379	6.406	6.409	5.604	5.604	5.598
neg_5354	N-Acetylmuramate	metab_10828	HMDB0060493	B(ii)	C02713	292.1044	1.9613	neg	M-H	C11H19NO8	0	34.8	1.99286554	61633-75-8	0.01271907	4.33	4.3	4.299	4.307	4.299	4.295	4.74	4.772	4.817	4.801	4.784	4.824	5.184	5.207	5.223	5.201	5.205	5.227	5.18	5.172	5.138	5.162	5.162	5.155	4.332	4.3	4.312	4.324	4.351	4.323	4.612	4.621	4.622

neg_5355	Ala-Gly-OH	metab_10829	-	B(i)	-	253.0466	1.9613	neg	M-H	C10H10N2O6	40.8	0	-0.1424652	-	0.018472552	5.087	5.074	5.062	5.063	5.08	5.012	3.811	3.784	3.881	3.823	3.845	3.659	2.639	2.68	2.673	2.674	2.676	2.677	2.66	3.038	2.659	2.829	2.996	3.159	2.565	2.707	2.559	2.559	2.562	2.552	4.576	4.589	4.59
neg_5356	2-Hydroxyacetaminophen sulfate	metab_10830	HMDB0062547	B(ii)	-	246.0079	1.9613	neg	M-H	C8H9NO6S	0	38.8	0.44186103	53446-14-3	0.005567018	4.271	4.241	4.241	4.248	4.24	4.237	4.285	4.334	4.345	4.355	4.317	4.383	4.888	4.941	4.951	4.942	4.939	4.975	4.539	4.54	4.532	4.499	4.547	4.518	4.351	4.319	4.374	4.365	4.314	4.35	4.268	4.263	4.265
neg_5360	(2R,3S)-2,3-dimethylmalate	metab_10835	HMDB0303969	B(ii)	C03652	205.0351	1.9613	neg	M+FA-H	C6H8O5-2	0	36.2	-1.4369529	-	0.006445907	5.66	5.629	5.61	5.597	5.593	5.6	5.324	5.347	5.387	5.39	5.392	5.378	5.749	5.768	5.777	5.773	5.777	5.779	5.79	5.798	5.768	5.812	5.799	5.78	6.01	6.015	6.026	6.02	6.009	6.006	5.838	5.833	5.839
neg_5361	N-Acetyl-L-Glutamic Acid	metab_10836	HMDB0001138;MJDBOTE0000638	B(i)	C00624	188.0562	1.9613	neg	M-H	C7H11NO5	34.2	0	-1.4337678	1188-37-0	0.00969532	4.505	4.519	4.467	4.481	4.464	4.529	3.96	4.06	4.082	4.227	4.078	4.101	4.484	4.546	4.521	4.476	4.509	4.533	4.792	4.805	4.811	4.796	4.825	4.814	4.272	4.266	4.358	4.269	4.262	4.226	4.408	4.407	4.4
neg_5365	Guanosine diphosphate adenosine	metab_10840	HMDB0001501	B(ii)	-	713.0877	1.9533	neg	M+Na-2H	C20H26N10O14P2	0	43.6	3.59632555	65331-78-4	0.003020234	5.617	5.593	5.593	5.622	5.575	5.573	5.23	5.277	5.289	5.288	5.295	5.302	5.393	5.435	5.428	5.428	5.431	5.432	5.415	5.416	5.414	5.417	5.415	5.417	5.319	5.317	5.313	5.314	5.312	5.307	5.343	5.342	5.34
neg_5390	FAPy-adenine	metab_10866	HMDB0004816	B(ii)	C06502	134.0466	1.8436	neg	M-H2O-H	C5H7N5O	0	44.4	-3.865683	5122-36-1	0.007056907	5.654	5.645	5.64	5.623	5.613	5.645	4.921	4.905	4.912	4.939	4.877	4.885	4.916	4.95	4.961	4.897	4.93	4.932	4.859	4.859	4.872	4.863	4.833	4.862	4.746	4.737	4.738	4.75	4.725	4.742	5.305	5.31	5.311
neg_5392	2-Hydroxychlorpropamide	metab_10868	HMDB0014024	B(ii)	-	629.0555	1.8356	neg	2M+FA-H	C10H13ClN2O4S	0	47.1	0.68583344	36892-36-1	0.003857683	7.017	6.983	6.984	6.969	6.967	6.97	4.233	4.227	3.728	3.96	4.349	4.03	3.793	3.352	4.072	3.345	3.783	3.781	3.401	3.559	3.331	3.334	3.505	3.378	3.479	3.342	3.507	3.437	3.981	3.441	6.625	6.624	6.622
neg_5397	Canavanine	metab_10873	HMDB0002706	B(ii)	C00308	213.0378	1.8356	neg	M+K-2H	C5H12N4O3	0	33.6	-9.8511939	543-38-4	0.022486027	4.204	3.936	4.008	3.895	4.056	4.014	4.733	4.736	4.757	4.791	4.695	4.778	4.023	4.059	4.095	4.014	4.04	4.048	4.265	4.272	4.08	4.21	4.282	4.22	4.628	4.643	4.657	4.655	4.629	4.672	4.276	4.293	4.276
neg_5398	Aminoadipic acid	metab_10874	LMFA01170098;HMDB0302754;HMDB0000510;PW_C	B(ii)	C00956	206.068	1.8356	neg	M+FA-H	C6H11NO4	0	47.8	6.02248834	542-32-5;111	0.011282625	4.931	4.883	4.869	4.885	4.864	4.879	0.3329	0.7004	0.3658	0.3649	0.3694	0.3729	1.367	0.4546	0.4501	0.4503	0.6529	1.656	0.4418	0.4428	0.4413	0.4433	0.4418	0.4429	2.386	0.3818	2.328	0.3799	0.3789	0.7946	4.365	4.373	4.365
neg_5407	3',5'-Cyclic GMP	metab_10885	HMDB0001314	B(i)	C00942	344.0407	1.8124	neg	M-H	C10H12N5O7P	77.9	0	1.54842869	7665-99-8	0.00971937	5.649	5.617	5.589	5.581	5.591	5.618	2.655	3.174	3.134	2.167	2.301	2.945	1.548	2.986	3	1.549	0.6098	0.0494	0.0476	2.121	2.943	3.307	3.023	2.756	4.695	4.67	4.722	4.678	4.745	4.673	5.297	5.289	5.29
neg_5422	5'-Thymidylic Acid	metab_10902	HMDB0001227	B(i)	C00364	321.0499	1.7578	neg	M-H	C10H15N2O8P	97.9	0	1.70457569	365-07-1	0.009848462	6.508	6.478	6.465	6.459	6.446	6.463	5.384	5.423	5.448	5.446	5.432	5.427	5.096	5.064	5.035	5.048	5.091	5.085	5.473	5.449	5.424	5.472	5.455	5.474	6.371	6.37	6.373	6.369	6.366	6.364	6.252	6.244	6.245
neg_5429	(6S)-Tetrahydro-L-biopterin	metab_10909	HMDB0257928	B(ii)	-	262.0936	1.7278	neg	M+Na-2H	C9H15N5O3	0	31.6	6.02461231	-	0.002877178	1.578	1.549	1.549	1.556	1.548	1.545	2.315	2.618	2.093	2.415	2.18	2.663	4.616	4.666	4.677	4.67	4.666	4.653	3.951	4.089	4.023	3.987	4.084	3.924	5.4	5.414	5.428	5.395	5.425	5.398	4.702	4.702	4.7
neg_5433	S-(6-Purinyl)cysteine	metab_10914	HMDB0244484	B(ii)	-	260.0238	1.7213	neg	M+Na-2H	C8H9N5O2S	0	31.8	6.13290012	-	0.005395707	3.946	4.043	4.009	4.035	3.89	3.905	1.406	1.451	1.467	1.461	1.469	1.475	1.564	1.604	1.598	1.598	1.6	1.601	2.25	2.831	1.584	1.587	1.585	1.587	6.205	6.2	6.196	6.19	6.194	6.192	5.484	5.479	5.48
neg_5436	Valaciclovir	metab_10917	HMDB0014716	B(ii)	C07184	359.1257	1.7076	neg	M+Cl	C13H20N6O4	0	41.8	5.23371747	124832-27-5	0.015007209	3.937	4.1	3.906	3.939	3.854	3.987	4.346	4.256	4.34	4.331	4.343	4.304	4.618	4.647	4.609	4.546	4.684	4.691	4.694	4.632	4.615	4.661	4.654	4.673	5.916	5.921	5.921	5.914	5.917	5.91	5.235	5.223	5.225
neg_5445	5-Fluorodeoxyuridine monophosphate	metab_10927	HMDB0060394	B(ii)	C04242	347.0039	1.6791	neg	M+Na-2H	C9H12FN2O8P	0	45.3	-7.0276268	-	0.020437735	5.34	5.337	5.338	5.331	5.34	5.351	5.595	5.59	5.629	5.614	5.61	5.607	5.704	5.739	5.715	5.715	5.741	5.717	5.707	5.717	5.702	5.713	5.709	5.732	5.473	5.475	5.452	5.466	5.479	5.452	5.508	5.507	5.492
neg_5462	Cidofovir	metab_10946	HMDB0014513	B(ii)	C06909	260.0455	1.6344	neg	M-H2O-H	C8H14N3O6P	0	59.4	4.86343503	113852-37-2	0.00638069	5.218	5.224	5.189	5.23	5.192	5.213	5.453	5.463	5.497	5.49	5.504	5.5	5.595	5.612	5.608	5.61	5.625	5.622	5.577	5.606	5.574	5.603	5.591	5.586	5.201	5.204	5.187	5.198	5.224	5.202	5.38	5.375	5.376
neg_5463	Dopamine 3-O-sulfate	metab_10947	HMDB0006275	B(ii)	C13690	232.0285	1.6279	neg	M-H	C8H11NO5S	0	47.3	-0.0905012	51317-41-0	0.002866052	4.827	4.807	4.763	4.79	4.762	4.756	5.226	5.267	5.284	5.279	5.275	5.248	4.884	4.825	4.929	4.838	4.911	4.861	4.988	4.998	4.967	4.947	4.983	4.994	5.695	5.686	5.687	5.68	5.689	5.683	5.255	5.253	5.254
neg_5464	Pyrroline hydroxycarboxylic acid	metab_10948	PW_C001057;HMDB0001369	B(ii)	C04281	257.0782	1.6243	neg	2M-H	C5H7NO3	0	47.4	0.94940486	22573-88-2	0.00461767	5.679	5.653	5.633	5.639	5.601	5.641	5.108	5.125	5.151	5.164	5.143	5.158	5.277	5.279	5.291	5.282	5.312	5.307	5.124	5.103	5.139	5.128	5.143	5.127	5.659	5.64	5.669	5.662	5.664	5.662	5.504	5.5	5.504
neg_5478	Phosphoribosylformylglycinamidine	metab_10963	HMDB0000999;HMDB0002305	B(ii)	C04640	334.0414	1.5586	neg	M+Na-2H	C8H16N3O8P	0	37.6	-2.4586083	37721-04-3;6	0.022805837	4.789	4.842	4.779	4.899	4.859	4.76	4.294	4.191	3.906	4.288	3.972	4.31	4.543	4.594	4.564	4.411	4.592	4.632	4.786	4.63	4.627	4.782	4.689	4.721	4.097	3.896	4.193	4.113	4.287	4.35	5.236	5.221	5.218
neg_5481	Thymidine	metab_10967	HMDB0000273;PW_C000187;MJDBOTE0000448	B(ii)	C00214	279.0392	1.5471	neg	M+K-2H	C10H14N2O5	0	31.5	1.32098643	50-89-5	0.020148026	6.517	6.495	6.477	6.494	6.464	6.484	6.286	6.324	6.331	6.319	6.34	6.343	6.375	6.399	6.41	6.402	6.427	6.416	6.327	6.318	6.307	6.333	6.326	6.322	6.284	6.283	6.278	6.279	6.298	6.283	6.364	6.348	6.35
neg_5482	L-DOPA 3'-glucoside	metab_10968	HMDB0029452	B(ii)	-	404.1219	1.5391	neg	M+FA-H	C15H21NO9	0	62.4	5.75860885	2275-95-8	0.009964242	4.477	4.267	4.184	4.457	4.345	4.302	4.028	4.179	4.074	4.085	4.008	4.085	4.152	3.993	4.05	3.863	4.319	4.232	4.134	4.063	4.219	4.109	4.137	4.254	4.878	4.829	4.829	4.922	4.768	4.864	5.955	5.954	5.962
neg_5483	Glu-Thr	metab_10969	HMDB0028829	B(i)	-	229.0829	1.5391	neg	M-H2O-H	C9H16N2O6	63.1	0	-0.2893691	6875-80-5	0.009223919	5.009	5.045	4.972	5.03	4.901	4.942	4.077	4.144	4.051	4.048	4.094	4.174	4.292	4.259	4.385	4.136	4.339	4.278	4.132	4.097	3.953	4.117	4.059	4.063	4.904	4.904	4.933	4.929	4.932	4.942	4.769	4.768	4.775
neg_5485	Liriodenine	metab_10971	HMDB0301844	B(ii)	C09567	310.0252	1.5311	neg	M+Cl	C17H9NO3	0	49.6	-9.0486504	475-75-2	0.03809567	3.175	3.145	3.145	3.153	3.144	3.141	3.593	3.618	3.822	3.936	3.602	3.585	3.698	3.561	3.721	3.517	3.746	3.959	3.244	3.245	3.243	3.246	3.244	3.245	4.673	4.697	4.661	4.605	4.734	4.591	4.126	4.154	4.155
neg_5492	[(2R,3S,4R,5R)-5-(4-Amino-2-oxopyrimidir	metab_10979	HMDB0260221	B(ii)	-	651.0995	1.483	neg	M+K-2H	C20H31N4O16P	0	50.3	5.88169561	-	0.00219834	6.958	6.93	6.913	6.918	6.897	6.913	6.316	6.364	6.376	6.374	6.382	6.388	6.48	6.521	6.515	6.515	6.517	6.519	6.502	6.503	6.501	6.504	6.502	6.503	6.406	6.404	6.4	6.4	6.399	6.393	6.429	6.428	6.427
neg_5494	8-Hydroxy-7-methylguanine	metab_10981	HMDB0006037	B(ii)	-	180.0524	1.483	neg	M-H	C6H7N5O2	0	56.9	-1.5110821	1688-85-3	0.281688643	0.8592	2.041	0.8334	1.578	0.8326	0.8298	5.32	5.381	5.336	5.384	5.325	5.356	5.412	5.41	5.435	5.387	5.438	5.387	5.383	5.414	5.342	5.315	5.389	5.379	5.147	5.138	5.074	5.094	5.109	5.092	2.933	2.933	2.697
neg_5497	Hexafluoroacetylacetone	metab_10984	HMDB0253132	B(ii)	-	206.9885	1.467	neg	M-H	C5H2F6O2	0	31.4	-0.5518083	-	0.020595951	4.493	4.478	4.419	4.448	4.378	4.435	4.899	4.951	4.964	4.965	4.984	4.99	4.933	4.928	4.945	4.936	4.948	4.953	5.106	5.061	5.069	5.105	5.071	5.087	4.709	4.726	4.698	4.683	4.716	4.757	4.805	4.804	4.82
neg_5502	7-[(7S)-7-Amino-5-azaspiro[2.4]heptan-5	metab_10991	HMDB0251635	B(ii)	-	430.0774	1.443	neg	M+Na-2H	C19H18ClF2N3O3	0	41.7	5.63054055	-	0.015001372	5.608	5.631	5.599	5.628	5.594	5.615	5.364	5.362	5.408	5.394	5.382	5.4	5.36	5.403	5.427	5.396	5.402	5.368	5.314	5.256	5.269	5.316	5.327	5.31	4.859	4.779	4.803	4.743	4.71	4.837	5.27	5.269	5.258
neg_5503	2-Amino-4-oxo-6-(1',2'-dioxoprolyl)-7,8-(	metab_10992	HMDB0001410	B(ii)	C05255	312.0593	1.443	neg	M+FA-H	C9H9N5O5	0	44.6	2.80133413	-	0.005703148	5.826	5.773	5.754	5.76	5.746	5.728	4.004	3.932	3.772	4.082	3.908	4.238	4.135	3.964	4.184	4.163	4.238	3.975	3.946	4.015	3.843	4.099	3.892	3.93	3.595	3.876	3.969	3.73	3.978	3.86	6.064	6.06	6.065
neg_5513	Tyrosylglycine	metab_11002	HMDB0259337;HMDB0029105	B(ii)	-	219.0773	1.419	neg	M-H2O-H	C11H14N2O4	0	30.2	-0.8813669	673-08-5	0.009900319	5.065	4.956	4.933	4.976	4.948	4.951	2.748	3.178	3.102	3.513	3.295	2.819	4.021	4.119	4.075	4.109	4.149	4.22	3.816	3.707	3.834	4.065	3.637	3.657	2.951	3.518	3.575	3.398	3.289	3.288	5.064	5.071	5.072
neg_5517	Reproterol	metab_11006	HMDB0257155	B(ii)	-	424.1373	1.395	neg	M+Cl	C18H23N5O5	0	51.3	-5.2775062	-	0.018089792	3.814	4.137	4.179	4.357	4.199	4.231	4.851	4.854	4.893	4.808	4.948	4.879	4.78	4.81	4.79	4.85	4.781	4.8	4.221	3.618	3.927	4.18	4.081	4.135	5.979	5.965	5.963	5.975	5.991	5.969	5.395	5.395	5.381
neg_5522	Sinapic acid 4-O-sulfate	metab_11011	HMDB0060020	B(ii)	-	349.0218	1.387	neg	M+FA-H	C11H12O8S	0	58.2	-5.5449603	2283338-09-ξ	0.0021474	5.418	5.413	5.416	5.444	5.439	5.403	4.757	4.629	4.81	4.746	4.751	4.801	4.851	4.833	4.822	4.821	4.824	4.806	4.582	4.605	4.531	4.551	4.583	4.535	4.436	4.434	4.43	4.43	4.429	4.423	5.035	5.036	5.037
neg_5527	Xanthomicrol	metab_11016	HMDB0037600;LMPK12111447	B(ii)	C14476	389.0859	1.371	neg	M+FA-H	C18H16O7	0	42.2	-5.3981827	16545-23-6	0.055199531	3.146	3.223	3.624	3.721	3.746	3.178	5.239	5.267	5.343	5.355	5.317	5.309	5.694	5.7	5.729	5.746	5.736	5.748	5.833	5.748	5.796	5.874	5.8	5.895	6.231	6.224	6.215	6.215	6.229	6.244	4.884	4.886	4.843
neg_5528	Benzo[b]thiophene-2-carboxylic acid	metab_11017	HMDB0249004	B(ii)	-	355.0113	1.371	neg	2M-H	C9H6O2S	0	31.4	2.4320119	-	0.003151373	4.208	3.939	3.722	4.023	4.142	3.944	5.405	5.447	5.482	5.469	5.481	5.448	5.234	5.235	5.255	5.248	5.249	5.256	5.261	5.271	5.28	5.276	5.234	5.29	5.981	5.968	5.96	5.984	5.972	5.965	5.566	5.567	5.568
neg_5532	Floridin	metab_11022	HMDB0303624	B(ii)	C11754	460.0674	1.355	neg	M+FA-H	C19H17N3O4S2	0	64	7.65852445	-	0.04762571	3.337	3.307	3.307	3.315	3.306	3.303	3.22	3.268	3.28	3.278	3.286	3.292	3.384	3.425	3.418	3.419	3.421	3.422	3.406	3.407	3.405	3.408	3.406	3.407	5.883	5.874	5.877	5.867	5.876	5.857	5.365	5.329	5.331

neg_5537	Methylisocitric acid	metab_11027	HMDB0006471	B(ii)	C04593	205.0351	1.347	neg	M-H	C7H10O7	0	58.4	-1.1688943	-	0.006127745	5.63	5.517	5.499	5.49	5.468	5.472	5.234	5.282	5.34	5.344	5.384	5.33	5.347	5.342	5.345	5.314	5.354	5.392	5.445	5.408	5.502	5.491	5.437	5.493	5.595	5.572	5.583	5.566	5.556	5.569	5.803	5.804	5.808
neg_5551	3-Methoxyphenol sulfate	metab_11041	HMDB0304914	B(ii)	-	249.0076	1.307	neg	M+FA-H	C7H8O5S	0	53.7	0.92124314	-	0.002335728	4.428	4.356	4.319	4.25	4.14	4.09	5.837	5.878	5.893	5.903	5.9	5.905	6.52	6.545	6.558	6.556	6.556	6.56	6.381	6.374	6.365	6.36	6.369	6.379	6.266	6.26	6.256	6.27	6.264	6.256	6.089	6.089	6.091
neg_5552	Ricinine	metab_11042	HMDB0042006	B(ii)	C01526	209.0566	1.307	neg	M+FA-H	C8H8N2O2	0	67.9	-1.2082329	524-40-3	0.0009793	4.35	4.166	3.865	3.99	3.931	3.988	6.327	6.369	6.367	6.366	6.376	6.389	6.82	6.839	6.868	6.864	6.856	6.855	6.752	6.744	6.742	6.743	6.739	6.755	7.123	7.12	7.12	7.125	7.127	7.124	6.689	6.69	6.69
neg_5557	2-Hydroxynicotinic acid	metab_11047	HMDB0059710	B(ii)	-	277.0447	1.291	neg	2M-H	C6H5NO3	0	41.4	-6.9268869	609-71-2	0.036774934	3.446	3.416	3.416	3.423	3.415	3.411	3.469	3.73	3.388	4.063	3.395	3.749	4.887	4.897	4.93	4.976	4.937	4.987	4.658	4.655	4.603	4.488	4.654	4.669	5.099	5.128	5.097	5.09	5.127	5.099	3.971	3.999	4
neg_5562	Flunixin	metab_11052	HMDB0252340	B(i)	-	277.0567	1.283	neg	M-H2O-H	C14H11F3N2O2	41.4	0	-9.1330568	-	0.016599669	4.977	4.956	4.994	4.918	4.967	4.977	3.025	3.493	3.808	3.447	3.998	3.446	4.423	4.388	4.519	4.479	4.672	4.65	5.427	5.418	5.386	5.431	5.409	5.396	0.5508	0.5491	0.5466	0.8513	0.5455	0.5418	4.823	4.81	4.811
neg_5567	Alpha-CYANO-3-HYDROXYCINNAMIC AC	metab_11057	-	B(i)	-	188.0349	1.275	neg	M-H	C10H7NO3	34.4	0	-2.4463518	-	0.012424118	3.442	3.63	3.769	3.563	3.632	3.461	2.787	2.753	3.088	3.144	3.041	2.688	3.083	2.893	2.815	2.907	2.817	2.818	2.802	2.803	2.801	2.804	2.802	2.803	4.879	4.904	4.886	4.896	4.877	4.869	3.711	3.71	3.701
neg_5573	1-(gamma-Glutamylamino)cyclopropaneca	metab_11064	HMDB0031701	B(ii)	-	275.0889	1.259	neg	M+FA-H	C9H14N2O5	0	31.5	1.65518829	171437-78-8	0.009911548	3.802	4.076	4.126	4.035	4.059	4.055	4.434	4.549	4.516	4.573	4.604	4.495	4.578	4.688	4.672	4.786	4.765	4.698	4.622	4.533	4.621	4.551	4.492	4.511	3.6	3.595	3.548	3.529	3.7	3.422	4.363	4.362	4.37
neg_5578	8-Hydroxyacyclovir	metab_11068	HMDB0247439	B(ii)	-	286.0796	1.251	neg	M+FA-H	C8H11N5O4	0	48.1	1.42192406	-	0.000949286	5.337	5.293	5.297	5.328	5.299	5.292	5.206	5.254	5.266	5.264	5.272	5.278	5.37	5.411	5.405	5.405	5.407	5.408	5.392	5.393	5.391	5.394	5.392	5.393	5.296	5.293	5.29	5.29	5.288	5.283	5.466	5.465	5.465
neg_5579	1-[(3R,4R,5R)-3-Fluoro-3,4-dihydroxy-5-(	metab_11069	HMDB0257564	B(ii)	-	275.0712	1.251	neg	M-H	C10H13FN2O6	0	38.4	9.77157703	-	0.004697148	4.43	4.316	4.335	4.433	4.366	4.383	5.018	5.066	5.081	5.078	5.1	5.086	5.45	5.476	5.49	5.481	5.491	5.49	5.219	5.189	5.174	5.194	5.189	5.214	4.723	4.757	4.735	4.732	4.779	4.815	5.029	5.028	5.032
neg_5584	Threonylhistidine	metab_11072	HMDB0029063	B(ii)	-	237.0993	1.243	neg	M-H2O-H	C10H16N4O4	0	30.1	0.00585542	55831-93-1	0.053759646	4.893	4.856	4.965	4.62	4.812	4.642	2.9	2.947	2.96	2.958	2.966	2.972	3.064	3.105	3.098	3.098	3.101	3.102	3.085	3.087	3.084	3.088	3.085	3.087	2.99	2.987	2.984	2.984	2.982	2.977	4.251	4.291	4.293
neg_5585	3,5-dihydroxy-4-(sulfooxy)benzoic acid	metab_11073	HMDB0126639	B(ii)	-	230.9583	1.243	neg	M-H2O-H	C7H6O8S	0	48.1	-8.662091	-	0.009714139	5.141	5.109	5.097	5.088	5.101	5.138	4.56	4.639	4.585	4.591	4.545	4.57	4.747	4.784	4.765	4.787	4.778	4.727	4.635	4.708	4.699	4.696	4.685	4.703	4.412	4.383	4.436	4.424	4.368	4.468	4.671	4.67	4.663
neg_5587	6-Aminopurin-8-one	metab_11075	HMDB0258004	B(ii)	-	194.0315	1.243	neg	M+FA-H	C5H3N5O	0	32.4	-3.3580359	-	0.007053774	5.302	5.543	5.601	5.608	5.621	5.634	5.953	5.987	5.993	5.991	6.007	6.018	6.058	6.087	6.108	6.092	6.091	6.079	6.059	6.069	6.056	6.052	6.065	6.069	5.95	5.949	5.954	5.952	5.953	5.953	5.852	5.851	5.846
neg_5595	Dopamine	metab_11083	HMDB0000073;PW_C000052;MJDBSJD0000070	B(i)	C03758	152.0711	1.227	neg	M-H	C8H11NO2	41.2	0	-3.8162604	51-61-6;62-3	0.01776156	4.233	4.212	4.121	4.137	4.007	4.022	4.538	4.562	4.677	4.727	4.764	4.761	4.805	4.83	4.854	4.854	4.882	4.872	5.243	5.253	5.253	5.254	5.292	5.262	5.163	5.144	5.122	5.089	5.085	5.096	4.888	4.902	4.889
neg_5603	6-(4-carboxy-2,6-dihydroxyphenoxy)-3,4,5	metab_11091	HMDB0126636	B(ii)	-	391.0502	1.2111	neg	M+FA-H	C13H14O11	0	40.4	-4.7915754	-	0.100022204	4.445	4.415	4.414	4.422	4.414	4.41	5.133	5.195	5.244	5.215	5.245	5.18	5.03	5.014	5.079	5.029	4.961	4.981	4.854	4.962	4.998	4.893	4.921	4.955	4.417	4.415	4.411	4.412	4.41	4.405	4.504	4.437	4.521
neg_5607	GSK264220A	metab_11095	-	B(i)	-	362.121	1.2031	neg	M-H	C17H21N3O4S	43.7	0	8.33120515	-	0.018402586	5.469	5.329	5.366	5.314	5.341	5.286	3.244	3.927	4.197	3.514	4.324	4.015	4.36	4.255	4.343	4.526	4.343	4.249	4.304	4.253	4.701	4.671	4.596	4.515	4.326	4.234	4.463	3.89	4.177	4.373	5.952	5.965	5.966
neg_5629	9-beta-d-Arabinofuranosylguanine	metab_11117	HMDB0246240	B(ii)	-	264.074	1.1553	neg	M-H2O-H	C10H13N5O5	0	37.7	0.76974379	-	0.061292339	5.095	5.055	5.078	5.057	5.082	5.097	3.013	3.644	3.153	2.998	3.471	3.669	3.104	3.145	3.138	3.139	3.141	3.146	3.126	3.127	3.125	3.128	3.126	3.127	3.152	3.027	3.026	3.024	3.022	3.017	4.236	4.188	4.235
neg_5635	DIMBOA-Glc	metab_11123	HMDB0029710	B(ii)	-	408.0711	1.1314	neg	M+Cl	C15H19NO10	0	31	2.06273993	113565-32-5	0.0266448	1.726	0.0578	2.12	2.069	0.0577	0.0572	5.112	5.172	5.217	5.124	5.215	5.125	5.65	5.68	5.734	5.643	5.674	5.675	5.509	5.524	5.497	5.467	5.513	5.495	5.463	5.434	5.462	5.427	5.448	5.453	5.288	5.287	5.307
neg_5639	3,5-Dichloro-2,6-dihydroxybenzoic acid	metab_11127	HMDB0242164	B(ii)	-	266.9466	1.1234	neg	M+FA-H	C7H4Cl2O4	0	41.3	-1.0797246	-	0.020624918	3.756	4.529	4.752	4.729	4.866	4.917	6.107	6.125	6.141	6.152	6.131	6.165	6.185	6.21	6.202	6.21	6.203	6.193	6.296	6.305	6.29	6.285	6.281	6.298	6.512	6.526	6.513	6.51	6.527	6.5	5.089	5.104	5.105
neg_564	3,4-Dihydroxymandelic Acid	metab_11128	PW_C001240;HMDB0001866	B(i)	C05580	183.0295	2.6459	neg	M-H2O-H, M-I	C8H8O5	78.4	0	-2.2947345	775-01-9	0.005715171	5.725	5.698	5.684	5.691	5.68	5.657	6.754	6.807	6.818	6.811	6.837	6.823	6.71	6.743	6.74	6.729	6.734	6.742	6.728	6.739	6.733	6.737	6.736	6.735	6.73	6.72	6.718	6.727	6.719	6.715	6.433	6.429	6.434
neg_5641	(2S)-3-(4H-Imidazol-4-yl)-2-[[(2S)-5-oxo	metab_11130	HMDB0256374	B(ii)	-	301.0683	1.1155	neg	M+Cl	C11H14N4O4	0	31.3	-9.9474444	-	0.019788164	5.498	5.454	5.456	5.42	5.459	5.448	4.885	4.848	4.922	4.973	4.984	4.957	3.224	3.381	3.105	3.432	3.838	3.116	4	3.146	3.757	3.629	3.053	3.79	4.628	4.486	4.453	4.654	4.603	4.771	4.945	4.945	4.96
neg_5644	3-Dehydroquinic acid	metab_11133	HMDB0012710	B(ii)	C00944	235.0459	1.0996	neg	M+FA-H	C7H10O6	0	45.4	-0.1862732	10534-44-8	0.004451011	4.61	4.655	4.63	4.607	4.659	4.612	5.288	5.245	5.248	5.287	5.313	5.302	5.235	5.317	5.312	5.31	5.356	5.323	5.402	5.362	5.345	5.369	5.354	5.365	5.339	5.366	5.334	5.367	5.366	5.36	5.158	5.155	5.156
neg_5646	5,6,8-Trihydroxy-2-methylbenzo[g]chrome	metab_11135	HMDB0033911	B(ii)	C17671	303.0532	1.0917	neg	M+FA-H	C14H10O5	0	55	8.3594814	3566-98-1	0.012135541	4.658	4.643	4.717	4.706	4.702	4.652	5.618	5.65	5.677	5.69	5.669	5.659	5.817	5.861	5.869	5.914	5.909	5.875	5.955	5.96	5.999	5.933	5.971	5.949	5.458	5.482	5.474	5.422	5.47	5.433	5.722	5.721	5.731
neg_5651	Daucic acid	metab_11139	HMDB0031665	B(ii)	-	203.0193	1.0757	neg	M-H	C7H8O7	0	30.2	-1.9804032	34098-52-7	0.278418951	5.074	5.066	5.057	5.056	5.042	5.052	2.758	2.805	2.817	2.816	2.824	2.83	2.921	2.963	2.956	2.956	2.959	2.96	2.943	2.945	2.942	2.945	2.943	2.945	2.847	2.845	2.842	2.842	2.982	2.835	3.985	3.98	3.75
neg_5652	DTDP-4-dehydro-6-deoxy-beta-L-manno	metab_11140	HMDB0304343	B(ii)	C00688	591.0662	1.0679	neg	M+FA-H	C16H24N2O15P2	0	45.5	5.13220073	-	0.028038589	4.025	3.995	3.995	4.003	3.994	3.991	4.682	4.726	4.431	4.794	4.565	4.796	4.093	4.171	4.107	4.107	4.643	4.459	4.094	4.095	4.093	4.096	4.094	4.095	3.998	3.995	3.992	3.992	3.991	3.985	5.172	5.151	5.152
neg_5661	5'-Guanylic Acid	metab_11150	HMDB0001397;MJDBOTE0000666	B(i)	C00144	362.0513	1.0522	neg	M-H	C10H14N5O8P	92.5	0	1.66854204	85-32-5	0.003501155	7.242	7.22	7.227	7.205	7.207	7.22	6.438	6.468	6.481	6.485	6.487	6.495	6.537	6.567	6.578	6.555	6.575	6.573	6.521	6.524	6.524	6.515	6.52	6.521	6.785	6.787	6.78	6.777	6.781	6.768	6.955	6.954	6.957
neg_5662	3-(2-Hydroxyethyl)histidine	metab_11151	HMDB0245773	B(ii)	-	244.0939	1.0522	neg	M+FA-H	C8H13N3O3	0	36.1	0.03816834	-	0.056497729	4.937	4.863	4.772	4.82	4.847	4.823	3.885	3.932	3.944	3.943	3.95	3.956	4.048	4.09	4.083	4.083	4.086	4.087	4.07	4.071	4.069	4.072	4.07	4.072	3.974	3.972	3.968	3.969	3.967	3.962	3.995	4.038	3.997
neg_5667	2-Phenylethanol glucuronide	metab_11156	HMDB0010350	B(ii)	C03033	319.0786	1.0363	neg	M+Na-2H	C14H18O7	0	61.3	-4.3195296	-	0.011737088	5.613	5.585	5.566	5.572	5.562	5.573	5.794	5.816	5.846	5.875	5.859	5.865	5.916	5.933	5.956	5.951	5.948	5.942	6.061	6.052	6.068	6.056	6.071	6.052	6.26	6.264	6.274	6.258	6.268	6.254	5.928	5.931	5.938
neg_5668	Uric Acid	metab_11157	PW_C000195;HMDB0000289;MJDBOTE0001097	B(i)	C00366	167.0206	1.0363	neg	M-H	C5H4N4O3	92.8	0	-3.0177251	69-93-2	0.026326738	5.309	6.06	6.243	6.265	6.315	6.363	7.661	7.684	7.694	7.692	7.693	7.706	7.719	7.743	7.756	7.75	7.756	7.751	7.839	7.847	7.83	7.825	7.843	7.836	7.981	7.98	7.972	7.973	7.979	7.966	6.818	6.818	6.798
neg_5682	Maltohexaose	metab_11170	HMDB0012253	B(ii)	C01936	989.3234	0.9887	neg	M-H	C36H62O31	0	83.5	3.21248604	34620-77-4	0.028512142	4.505	4.429	3.937	4.146	4.151	4.309	5.716	5.817	5.77	5.732	5.836	5.767	5.862	5.89	5.949	5.876	5.965	5.964	5.949	5.947	5.99	5.899	5.925	5.87	5.783	5.906	5.837	5.866	5.856	5.83	5.842	5.841	5.863
neg_5684	ADP-ribose 2'-phosphate	metab_11171	HMDB0006336	B(ii)	C03246	620.0218	0.9887	neg	M-H2O-H	C15H24N5O17P3	0	50	2.49952608	4197-14-2	0.008445813	4.626	4.665	4.554	4.64	4.558	4.583	4.586	3.841	4.367	4.077	4.309	4.354	5.058	5.09	5.122	4.927	4.961	5.048	4.929	4.748	4.914	5.008	5.033	5.06	3.883	4.419	4.412	4.278	4.445	4.567	4.771	4.77	4.764
neg_5690	Guanosine diphosphate	metab_11178	HMDB0001201;PW_C000936	B(ii)	C00035	442.018	0.9807	neg	M-H	C10H15N5O11P2	0	93.1	2.24021611	146-91-8	0.214478099	5.076	5.118	5.012	5.055	5.09	5.041	4.109	4.324	4.379	3.826	4.325	4.291	5.251	5.188	5.289	5.175	5.194	5.336	5.079	4.689	5.052	4.805	4.967	4.963	3.82	3.817	3.814	3.814	3.812	3.807	4.97	4.796	4.972
neg_5694	2,2-Difluoro-n-(2-hydroxyethyl)-3-(2-nitro	metab_11182	HMDB0253829	B(ii)	-	245.0475	0.9727	neg	M-H2O-H	C8H10F2N4O4	0	35	-6.2064701	-	0.010656561	4.96	4.94	4.861	4.928	4.955	4.906	6.136	6.156	6.176	6.186	6.17	6.17	6.492	6.516	6.505	6.514	6.526	6.51	6.569	6.56	6.583	6.572	6.569	6.574	6.281	6.263	6.261	6.271	6.279	6.255	6.375	6.374	6.383
neg_5701	2-(3-Mercaptopropyl)pentanedioic acid	metab_11191	HMDB0245244	B(ii)	-	187.0416	0.9332	neg	M-H2O-H	C8H14O4S	0	50	-9.1536486	-	0.010040535	3.869	3.948	3.916	3.826	3.932	3.88	5.682	5.715	5.719	5.733	5.718	5.711	6.281	6.295	6.291	6.293	6.298	6.277	6.292	6.284	6.302	6.286	6.284	6.294	6.269	6.258	6.261	6.261	6.254	6.245	6.009	6.008	6.016
neg_5706	5-hydroxyindole thiazolidine carboxylate	metab_11196	HMDB0304217	B(ii)	-	313.0431	0.9253	neg	M+Cl	C13H14N2O3S	0	38.2	4.34079618	-	0.027865053	4.596	4.638	4.679	4.601	4.76	4.792	6.227	6.254	6.253	6.248	6.248	6.261	6.086	6.098	6.129	6.115	6.101	6.103	6.162	6.17	6.129	6.123	6.15	6.136	4.889	4.834	4.866	4.97	4.913	4.987	4.942	4.92	4.922
neg_5717	Deoxyguanylic Acid	metab_11205	HMDB0001044	B(i)	C00362	693.1213	0.9013	neg	2M-H	C10H14N5O7P	89.7	0	3.4216882	902-04-5	0.089747097	5.14	5.156	5.237	5.096	5.125	5.208	3.851	3.33	4.112	3.578	3.9	3.866	4.128	3.888	3.481	3.481	4.255	3.485	3.468	3.469	3.467	3.47	3.468	3.469	3.372	3.37	3.761	3.405	3.726	3.36	4.08	4.146	4.081
neg_5719	ADP	metab_11207	HMDB0001341	B(i)	C00008	426.0232	0.9013	neg	M-H	C10H15N5O10P2	88.6	0	2.56682587	58-64-0	0.013929219	6.025	6.027	5.997	5.999	5.989	6.011	6.268	6.283	6.31	6.302	6.328	6.311	6.452	6.468	6.484	6.478	6.483	6.498	6.264	6.264	6.247	6.255	6.256	6.265	5.871	5.873	5.865	5.865	5.86	5.875	6.158	6.168	6.169

neg_5725	Glutarimide	metab_11213	HMDB0252816	B(ii)	C07275	158.0453	0.8935	neg	M+FA-H	C5H7NO2	0	52.9	-5.3075354	1121-89-7	0.001250699	2.972	3.731	2.58	2.587	3.021	2.916	4.432	4.41	4.418	4.543	4.633	4.485	5.369	5.4	5.416	5.395	5.405	5.411	5.409	5.418	5.406	5.426	5.407	5.42	5.836	5.821	5.82	5.821	5.817	5.819	5.174	5.175	5.174
neg_5727	Glucotropaeolin	metab_11214	HMDB0038419	B(ii)	C02153	444.0232	0.8855	neg	M+Cl	C14H19NO9S2	0	55	8.97423195	499-26-3	0.036020173	5.498	5.477	5.509	5.423	5.466	5.485	4.31	4.255	4.304	4.444	4.501	4.538	5.065	5.021	5.009	5.06	4.994	5.095	4.598	3.814	4.685	4.381	4.619	4.594	4.679	4.859	4.76	4.874	4.818	4.82	5.169	5.196	5.197
neg_5731	2-(3-Methylpiperidin-1-yl)[1,3]thiazolo[4,5-	metab_11218	HMDB0244922	B(ii)	-	545.1737	0.8775	neg	2M+FA-H	C11H14N4OS	0	44.6	-4.2359493	-	0.009438269	5.239	5.209	5.209	5.216	5.208	5.205	5.583	5.564	5.589	5.646	5.645	5.646	5.776	5.846	5.877	5.823	5.849	5.828	5.805	5.757	5.791	5.736	5.745	5.802	5.31	5.255	5.286	5.228	5.204	5.271	5.421	5.428	5.421
neg_5745	N-[(4-Amino-2-methylpyrimidin-5-yl)meth	metab_11230	HMDB0258148	B(ii)	-	753.2334	0.8451	neg	2M+FA-H	C15H22N4O2S2	0	37.4	-2.3588496	-	0.052082438	3.767	3.737	3.737	3.744	3.736	3.732	6.002	5.971	6.009	6.021	6.057	6.025	6.072	6.125	6.097	6.143	6.14	6.142	6.024	5.987	5.979	5.988	6.036	6.033	3.926	3.737	3.733	4.184	3.732	3.727	5.046	5.085	5.046
neg_5752	Rafabegron	metab_11236	HMDB0257097	B(ii)	-	447.1365	0.8133	neg	M+FA-H	C21H23ClN2O4	0	45.7	9.15935633	-	0.093184641	4.24	4.21	4.21	4.217	4.209	4.206	5.224	5.203	5.292	5.185	5.261	5.307	5.197	5.256	5.243	5.248	5.207	5.183	5.237	5.351	5.19	5.283	5.272	5.305	4.213	4.21	4.207	4.207	4.205	4.2	4.233	4.304	4.306
neg_5753	Isopropyl tartaric acid	metab_11237	HMDB0302348	B(ii)	-	429.1257	0.8133	neg	2M+FA-H	C7H12O6	0	56.4	1.89522354	-	0.012433468	3.444	3.414	3.414	3.421	3.413	3.409	6.448	6.471	6.492	6.478	6.482	6.495	6.389	6.436	6.423	6.442	6.43	6.421	6.485	6.477	6.458	6.445	6.474	6.467	3.416	3.511	3.41	3.902	3.409	3.605	5.738	5.737	5.728
neg_5785	Uric acid lactate	metab_11264	HMDB0259719	B(ii)	-	301.0429	0.7655	neg	M+FA-H	C8H8N4O6	0	35.9	1.3532448	-	0.043401246	3.739	3.709	4.267	4.239	4.478	4.536	5.749	5.765	5.772	5.787	5.784	5.778	5.732	5.749	5.769	5.741	5.758	5.725	5.667	5.674	5.637	5.647	5.653	5.677	5.093	5.048	5.036	5.044	5.09	5.032	4.877	4.876	4.844
neg_5796	Aceglutamide	metab_11275	HMDB0006029;MJDBOTE0000339	B(i)	-	187.072	0.7575	neg	M-H	C7H12N2O4	46	0	-2.2213422	2490-97-3	0.007175861	5.133	5.098	5.085	5.095	5.07	5.078	4.818	4.833	4.919	4.869	4.899	4.794	5.033	5.039	5.07	5.019	5.08	5.064	4.703	4.754	4.731	4.699	4.673	4.732	4.725	4.783	4.738	4.76	4.765	4.775	5.043	5.048	5.049
neg_5806	Uridine-5'-Monophosphate	metab_11286	HMDB0000288;MJDBOTE0000717	B(i)	C00105	647.0666	0.7416	neg	2M-H	C9H13N2O9P	95.3	0	3.34087944	58-97-9	0.070458385	4.564	4.392	4.066	4.01	4.444	3.964	3.881	4.064	3.941	3.939	3.947	3.953	4.045	4.086	4.079	4.08	4.082	4.083	4.314	4.317	4.216	4.069	4.067	4.22	5.583	5.594	5.556	5.595	5.589	5.577	4.559	4.504	4.557
neg_581	(2R,3S)-Piscidic acid	metab_11289	HMDB0030809	B(ii)	-	277.0334	2.7093	neg	M+Na-2H, 2M	C11H12O7	0	30.9	1.63370536	35388-57-9	0.012640275	4.84	4.852	4.794	4.751	4.82	4.858	5.132	5.202	5.203	5.205	5.229	5.177	4.859	4.82	4.83	4.827	4.817	4.814	4.908	4.965	5.001	4.97	5.011	4.968	3.747	3.611	3.774	3.604	3.874	3.989	4.641	4.651	4.642
neg_5814	Geniposide	metab_11293	HMDB0252679;MJDBOTE0000901	B(ii)	C09781	369.1168	0.7416	neg	M-H2O-H	C17H24O10	0	50.3	-5.8860824	24512-63-8	0.015271349	4.846	4.375	4.121	4.363	4.239	4.011	3.928	3.976	3.988	3.986	3.994	4	4.092	4.133	4.127	4.127	4.129	4.131	4.114	4.115	4.113	4.116	4.114	4.115	4.018	4.016	4.012	4.012	4.011	4.005	5.275	5.265	5.278
neg_5815	Inosine 5'-Phosphate	metab_11294	HMDB0000175	B(i)	C00130	347.0404	0.7416	neg	M-H	C10H13N4O8P	85	0	1.56905832	131-99-7	0.00736322	6.058	6.057	6.048	6.031	6.042	6.05	6.75	6.756	6.776	6.785	6.777	6.776	6.807	6.84	6.837	6.833	6.831	6.834	6.989	6.976	6.975	6.988	6.965	6.97	7.157	7.151	7.129	7.149	7.143	7.139	6.847	6.853	6.848
neg_5816	Pseudouridine 5'-phosphate	metab_11295	HMDB0001271	B(ii)	C01168	323.029	0.7416	neg	M-H	C9H13N2O9P	0	79.4	1.24815789	1157-60-4	0.006338556	7.46	7.436	7.423	7.424	7.42	7.434	6.984	7.004	7.019	7.026	7.029	7.026	6.989	7.029	7.029	7.021	7.018	7.032	7.142	7.131	7.131	7.131	7.134	7.136	7.617	7.621	7.613	7.615	7.615	7.611	7.375	7.379	7.381
neg_5824	Elsamitrucin	metab_11302	HMDB0251754	B(ii)	-	688.1745	0.7336	neg	M+Cl	C33H35NO13	0	39.9	-8.7882451	-	0.009966876	5.19	5.16	5.16	5.168	5.159	5.156	5.741	5.74	5.693	5.727	5.774	5.724	5.644	5.718	5.78	5.664	5.734	5.7	5.64	5.61	5.704	5.638	5.668	5.654	5.231	5.161	5.226	5.31	5.301	5.246	5.522	5.515	5.523
neg_5846	Biflorin	metab_11323	HMDB0002336	B(ii)	C08996	399.0957	0.7016	neg	M+FA-H	C16H18O9	0	69.1	6.83992127	89701-85-9	0.014643508	5.854	5.813	5.801	5.79	5.795	5.797	5.492	5.539	5.551	5.55	5.557	5.564	5.655	5.697	5.69	5.69	5.693	5.694	5.677	5.679	5.676	5.679	5.677	5.679	6.453	6.449	6.448	6.461	6.451	6.444	5.613	5.613	5.602
neg_5865	Glutamyl pyruvate	metab_11341	HMDB0252814	B(ii)	-	262.0571	0.678	neg	M+FA-H	C8H11NO6	0	76.5	1.11304924	-	0.038410523	5.221	5.21	5.213	5.193	5.219	5.217	4.658	4.706	4.718	4.716	4.724	4.73	4.822	4.863	4.856	4.857	4.859	4.86	4.843	4.845	4.843	4.846	4.843	4.845	4.748	4.745	4.742	4.742	4.74	4.735	4.768	4.797	4.798
neg_5872	Hyaluronan biosynthesis, precursor 1	metab_11347	HMDB0060071	B(ii)	C04794	757.2185	0.67	neg	2M-H	C14H21NO11	0	90.9	3.71864218	-	0.024943969	5.768	5.792	5.745	5.754	5.637	5.772	4.726	4.746	4.54	4.935	4.362	4.896	4.56	4.567	4.254	4.33	4.243	4.245	4.527	5.005	4.948	4.657	4.874	4.812	4.132	4.13	4.126	4.126	4.125	4.119	5.406	5.425	5.408
neg_5875	4,5-Dihydro-4-((1-piperazinyl)acetyl)-7-(3-	metab_11350	HMDB0250308	B(ii)	-	451.1233	0.67	neg	M+Cl	C20H19F3N6O	0	70.7	-8.1277038	-	0.003899071	4.252	4.182	4.013	4.159	4.249	4.139	5.952	5.947	5.985	5.961	5.956	5.98	5.76	5.793	5.766	5.774	5.783	5.777	6.2	6.197	6.2	6.2	6.189	6.181	5.391	5.391	5.343	5.421	5.411	5.41	5.534	5.533	5.536
neg_5882	Threonolactone	metab_11356	HMDB0000940	B(ii)	-	163.0242	0.67	neg	M+FA-H	C4H6O4	0	49	-5.0428329	21730-93-8	0.009412893	5.155	5.109	5.112	5.089	5.092	5.117	5.071	5.08	5.112	5.106	5.117	5.093	5.031	4.996	4.998	5.035	5.032	5.043	5.064	5.038	5.031	5.066	5.103	5.065	5.106	5.109	5.106	5.096	5.104	5.115	5.099	5.092	5.099
neg_5886	Olprinone	metab_11358	HMDB0255959	B(ii)	-	545.1735	0.6621	neg	2M+FA-H	C14H10N4O	0	40.7	8.75416802	-	0.008804693	4	3.97	3.97	3.977	4.01	3.966	5.935	5.943	5.958	5.978	5.969	5.968	6.053	6.077	6.065	6.063	6.077	6.088	5.968	5.965	6.007	5.985	6	5.963	5.22	5.033	5.193	5.164	5.083	5.162	5.621	5.627	5.629
neg_5895	Uridine diphosphate-N-acetylglucosamine	metab_11368	PW_C000196;HMDB0000290	B(i)	C00043	606.0761	0.6541	neg	M-H	C17H27N3O17P2	90.6	0	2.94166917	528-04-1	0.002934759	6.661	6.639	6.606	6.615	6.617	6.628	6.874	6.867	6.914	6.943	6.899	6.933	6.92	6.947	6.934	6.952	6.966	6.943	6.962	6.917	6.957	6.932	6.951	6.923	7.113	7.116	7.102	7.083	7.091	7.086	6.927	6.93	6.928

neg_59	Xanthine	metab_11373	HMDB0000292;PW_C000198;MJDBOTE0000403	B(i)	C00385	151.0256	1.291	neg	M-H, 2M+FA-	C5H4N4O2	88.9	0	-3.5114729	69-89-6	0.001909451
neg_5901	5-Aminolevulinic acid	metab_11376	PW_C000894;HMDB0001149	B(ii)	C00430	176.0559	0.6541	neg	M+FA-H	C5H9NO3	0	78	-4.4235712	106-60-5	0.009605135
neg_5908	1,1,1-Trifluoro-2,4-pentanedione	metab_11380	HMDB0246204	B(ii)	-	367.065	0.6461	neg	2M+Hac-H	C5H5F3O2	0	38.8	9.31074427	-	0.006151721
neg_5914	D-Digitoxose	metab_11387	HMDB0251279	B(ii)	C21045	185.0216	0.6461	neg	M+K-2H	C6H12O4	0	48.7	-3.8421748	-	0.009504615
neg_5917	Trans-cinnamoyl-beta-D-glucoside	metab_11390	HMDB0304505	B(ii)	C04164	679.2184	0.6382	neg	2M+Hac-H	C15H18O7	0	58.2	-9.544502	-	0.010087261
neg_5919	SA-beta-Gal	metab_11392	HMDB0257432	B(ii)	-	601.1394	0.6382	neg	M+K-2H	C26H32N2O12	0	76.5	-8.315698	-	0.008597466
neg_5920	1-Amino-2-methylanthraquinone	metab_11394	HMDB0243816	B(ii)	C19320	519.1573	0.6382	neg	2M+FA-H	C15H11NO2	0	32	2.47762988	82-28-0	0.025086966
neg_5925	Serylasparagine	metab_11399	HMDB0029034	B(ii)	-	200.0673	0.6382	neg	M-H2O-H	C7H13N3O5	0	36.1	-1.7678176	81466-41-3	0.013625458
neg_5929	Trehalose	metab_11402	HMDB0000975;PW_C000776	B(i)	C01083	387.115	0.6303	neg	M+FA-H	C12H22O11	98.2	0	1.72682279	99-20-7	0.001548229
neg_593	Phenylalanylphenylalanine	metab_11403	HMDB0013302	B(ii)	-	333.1209	2.7825	neg	M-H, M+Na-2	C18H20N2O3	0	54.1	-3.7641297	2577-40-4	0.009605644
neg_5931	Ethyl glucuronide	metab_11405	HMDB0010325	B(ii)	-	221.0664	0.6303	neg	M-H	C8H14O7	0	62.7	-1.4133049	17685-04-0	0.010602933
neg_5935	Hydroxytyrosyl acetate	metab_11407	HMDB0253300	B(ii)	-	537.1684	0.6223	neg	2M+Hac-H	C11H13NO5	0	43.2	-8.7826935	-	0.004267295
neg_5936	Lusitanicoside	metab_11408	HMDB0034120	B(ii)	C10474	487.1789	0.6223	neg	M+FA-H	C21H30O10	0	57.3	-7.2142256	499-35-4	0.002294931
neg_5937	4-(2-Nitroethyl)phenyl primeveroside	metab_11409	HMDB0031742	B(ii)	-	482.1289	0.6223	neg	M+Na-2H	C19H27NO12	0	50.1	1.9909301	-	0.007134633
neg_5938	Setipiprant	metab_11410	HMDB0258261	B(ii)	-	439.0867	0.6223	neg	M+K-2H	C24H19FN2O3	0	34.8	0.42729491	-	0.005844746
neg_5940	Palatinose	metab_11413	HMDB0256083	B(ii)	C01742	377.0861	0.6223	neg	M+Cl	C12H22O11	0	89.9	1.44680423	13718-94-0	0.001197919
neg_5944	N-Acetyl-D-mannosamine 6-phosphate	metab_11415	PW_C000875;HMDB0001121	B(ii)	C04257	300.0491	0.6145	neg	M-H	C8H16NO9P	0	65.9	0.46558333	873185-52-5	0.019319228
neg_5949	10-EdAM	metab_11419	HMDB0244267	B(ii)	-	488.1632	0.6065	neg	M+Na-2H	C22H25N7O5	0	44.4	-6.8540894	-	0.002505961
neg_5952	Glycerophosphoinositol	metab_11423	HMDB0011649	B(ii)	C01225	333.0597	0.6065	neg	M-H	C9H19O11P	0	62.6	1.4664613	129830-95-1	0.00580804
neg_5961	2-Hydroxy-L-methionine	metab_11431	HMDB0246915	B(ii)	-	164.0381	0.5986	neg	M-H	C5H11NO3S	0	51.6	-3.330761	-	0.004888327
neg_5966	3-Hydroxyflavone	metab_11436	HMDB0031816;MJDBOTE0000444	B(ii)	C01495	219.0452	0.5906	neg	M-H2O-H	C15H10O3	0	37.9	0.14760402	577-85-5	0.016642549
neg_5976	N-(L-Arginino)succinate	metab_11445	HMDB0000052	B(i)	C03406	289.1156	0.5828	neg	M-H	C10H18N4O6	45.7	0	0.81990739	2387-71-5	0.004942588
neg_5977	Iminoerythrose 4-phosphate	metab_11446	HMDB0060483	B(ii)	C12215	244.0228	0.5828	neg	M+FA-H	C4H10NO6P	0	56.8	0.28391091	-	0.037203961
neg_5978	1-(2-Hydroxyethyl)-5-nitropyrrole-2-carbc	metab_11447	HMDB0255761	B(ii)	-	220.0322	0.5828	neg	M+Na-2H	C7H9N3O4	0	37.4	-8.9761622	-	0.031820998
neg_5979	Citrulline	metab_11448	PW_C000723;HMDB0000904;MJDBOTE0000481	B(i)	C00327	174.0878	0.5828	neg	M-H	C6H13N3O3	71.2	0	-3.4628901	372-75-8	0.01269792
neg_5981	Dimethylglycine	metab_11450	PW_C000062;HMDB0000092;MJDBOTE0000579	B(i)	C01026	102.0553	0.5828	neg	M-H	C4H9NO2	56.2	0	-7.0538412	1118-68-9	0.008597252
neg_5991	Hydroxymethylcytosine	metab_11459	HMDB0253278	B(ii)	-	186.0516	0.5753	neg	M+FA-H	C5H7N3O2	0	49.4	-3.1841964	-	0.016095455
neg_5992	5-Aminoisoquinolin-1(2H)-one	metab_11460	HMDB0246750	B(ii)	-	181.0381	0.5753	neg	M+Na-2H	C9H8N2O	0	31.8	-1.6096433	-	0.02803855
neg_5996	Histidylglutamine	metab_11463	HMDB0028883	B(ii)	-	328.1283	0.5675	neg	M+FA-H	C11H17N5O4	0	52.3	7.05188366	224638-13-5	0.039780448
neg_5997	1-O-Caffeoylglucose	metab_11464	HMDB0036937	B(ii)	C10433	323.0782	0.5675	neg	M-H2O-H	C15H18O9	0	40.9	2.94705049	14364-08-0	0.037685592
neg_5999	Serylcysteine	metab_11466	HMDB0029036	B(ii)	-	207.0446	0.5675	neg	M-H	C6H12N2O4S	0	60.9	0.4362508	109481-41-6	0.003437099
neg_60	Succinic Acid	metab_11468	LMFA01170043;HMDB0000254;PW_C000174;MJDBO	B(i)	C00042	117.0187	1.363	neg	M-H2O-H, M-	C4H6O4	73.1	0	-5.0576227	110-15-6	0.004406361
neg_6004	Taurine	metab_11474	HMDB0000251;PW_C000171;MJDBOTE0000787	B(i)	C00245	124.0068	0.5675	neg	M-H	C2H7NO3S	93.5	0	-4.8697507	107-35-7	0.00337258
neg_6015	N-(2,3-Dihydroxypropyl)valine	metab_11486	HMDB0254994	B(ii)	-	228.0641	0.5597	neg	M+K-2H	C8H17NO4	0	31	-1.1364043	-	0.003182807
neg_6019	2-Amino-4-[carbamimidoyl(methyl)amino]l	metab_11490	HMDB0255374	B(ii)	-	209.0808	0.5597	neg	M+Cl	C6H14N4O2	0	75.8	-1.3501415	-	0.038515473
neg_6021	L-Histidine	metab_11493	PW_C000116;HMDB0000177;MJDBOTE0000427	B(i)	C00135	190.0385	0.5597	neg	M+Cl	C6H9N3O2	96.8	0	-2.6713666	71-00-1	0.058276048
neg_6025	Maleamic Acid	metab_11497	HMDB0254310	B(i)	C01596	114.019	0.5597	neg	M-H	C4H5NO3	46.8	0	-5.4618303	557-24-4	0.007626087
neg_6031	8-Hydroxy-3,4,5-trimethyl-6-oxo-4,6-dihy	metab_11503	HMDB0249887	B(ii)	-	295.0816	0.5518	neg	M+FA-H	C13H14O5	0	49.4	-2.8052563	-	0.001350287
neg_6069	Asparaginyl-Gamma-glutamate	metab_11540	HMDB0028745	B(ii)	-	281.0871	0.5359	neg	M+Na-2H	C9H16N4O5	0	30.8	1.22236923	-	0.028602534
neg_6072	DL-Lanthionine	metab_11544	HMDB0251521	B(ii)	-	245.0005	0.5359	neg	M+K-2H	C6H12N2O4S	0	34.2	0.76162905	-	0.01815474
neg_6114	Carboxyifosfamide	metab_11589	PW_C040491;HMDB0060692	B(ii)	C16557	328.9622	0.5199	neg	M+K-2H	C7H15Cl2N2O4P	0	42.7	-3.6529361	53459-52-2	0.072928576
neg_6164	(2S)-2-Amino-3-[(2S,3R)-2-amino-3-[(2S)	metab_11643	HMDB0257991	B(ii)	-	368.1429	0.496	neg	M-H	C16H23N3O7	0	38.2	-9.248581	-	0.017150794
neg_6169	N-Acetyl-S-benzyl-L-cysteine	metab_11648	HMDB0255061	B(ii)	-	290.0245	0.496	neg	M+K-2H	C12H15NO3S	0	30.9	-5.5087089	-	0.025652887
neg_617	4-Methylumbelliferone glucuronide	metab_11649	HMDB0240464	B(ii)	C11584	397.0759	2.9333	neg	M+Cl, M+FA-H	C16H16O9	0	43.7	-4.812937	6160-80-1	0.007218289
neg_6194	Narceine	metab_11674	HMDB0030246	B(ii)	C09591	482.124	0.4835	neg	M+K-2H	C23H27NO8	0	33.7	3.95801519	131-28-2	0.012924625
neg_6196	Thiamphenicol glycinate	metab_11676	HMDB0258973	B(ii)	-	457.0211	0.4835	neg	M+FA-H	C14H18Cl2N2O6S	0	36.6	-8.1657596	-	0.018641697
neg_6209	C.I. Solvent Red 80	metab_11687	HMDB0037521	B(ii)	C19214	345.0642	0.4775	neg	M+K-2H	C18H16N2O3	0	34.4	-1.6915043	6358-53-8	0.019754332
neg_6224	3,4,5-trihydroxy-6-{4-[(E)-2-(3-hydroxy-5	metab_11704	HMDB0128507	B(ii)	-	587.141	0.468	neg	M+Na-2H	C26H30O14	0	37.3	4.94220015	-	0.028735567
neg_6229	DTDP-D-galactose	metab_11709	HMDB0006876	B(ii)	C02097	601.0214	0.4631	neg	M+K-2H	C16H26N2O16P2	0	34.9	-5.2770584	5817-97-0	0.063030681
neg_629	Fluprostenol	metab_11773	HMDB0252405	B(ii)	-	457.1839	3.0046	neg	M-H, M+Na-2	C23H29F3O6	0	58.9	-0.9166346	-	0.006593675
neg_63	(2E,6E)-2,6-bis(2-phenylhydrazono)cyclohe	metab_11784	-	B(i)	-	363.108	1.6188	neg	M-H, M+FA-H,	C18H14N4O2	30.2	0	-6.0519553	-	0.003980445
neg_630	Osmundalactone	metab_11785	HMDB0031303	B(ii)	-	109.0289	3.0125	neg	M-H2O-H, 3M	C6H8O3	0	51.3	-5.0269093	69308-39-0	0.010162702
neg_6304	Phosphate	metab_11790	HMDB0001429;PW_C001104	B(i)	C00009	96.9689	0.0556	neg	M-H	H3O4P	97.3	0	-7.8202172	7664-38-2;14	0.005270131
neg_632	3'-Methoxyfukiic acid	metab_11807	HMDB0029498	B(ii)	-	307.0424	3.0205	neg	M+Na-2H, M+	C12H14O8	0	30.7	-4.1094056	-	0.017443323
neg_638	Vanillin	metab_11856	HMDB0012308;HMDB0245834;MJDBOTE0000374	B(i)	C00755	151.0395	3.0685	neg	M-H2O-H, M-	C8H8O3	90.9	0	-3.6992992	121-33-5	0.015397702
neg_641	(4aS,5aS,12aS)-7-chloro-4-(dimethylaminc	metab_11860	HMDB0144089	B(ii)	-	463.0933	3.0766	neg	M-H, M+FA-H	C21H21ClN2O8	0	40.7	4.07177199	-	0.01089605
neg_645	Oxaprozin glucuronide	metab_11864	HMDB0255967	B(ii)	-	468.1282	3.0846	neg	M-H, M+Na-2	C24H23NO9	0	51.5	-3.7582879	-	0.002030597
neg_646	2,3,4,5-Tetrahydroxy-6-(1,2,3,4-tetrahydro	metab_11865	HMDB0255397	B(ii)	-	333.0621	3.0685	neg	M+Cl, 3M-H	C10H18O10	0	71.9	8.93398566	-	0.008487649
neg_649	Danielone	metab_11868	HMDB0031704	B(ii)	C10674	211.0609	3.0605	neg	M-H2O-H, M-	C10H12O5	0	39.9	-1.3751355	90426-22-5	0.013246636
neg_653	Methidathion	metab_11873	HMDB0254527	B(ii)	C14431	346.9617	3.1552	neg	M-H, M+FA-H	C6H11N2O4PS3	0	48.7	5.36947912	950-37-8	0.011268335
neg_663	7-[2,5-Dihydroxy-2-[3-hydroxy-4-[3-(trifl	metab_11884	HMDB0260400	B(ii)	-	457.1842	3.2526	neg	M-H, M+Na-2	C23H29F3O6	0	61.4	-0.328469	-	0.00437541
neg_665	Caffeic acid 4-O-glucuronide	metab_11886	HMDB0041707	B(ii)	-	355.0677	3.2643	neg	M-H, M+Na-2	C15H16O10	0	33.4	1.81751814	-	0.001699772
neg_677	Gamma-Glu-Leu	metab_11899	HMDB0011171	B(i)	-	259.1304	3.4411	neg	M-H, M+Na-2	C11H20N2O5	68.8	0	1.58798477	2566-39-4	0.01180451
neg_690	5-Methoxytryptophan	metab_11914	HMDB0002339	B(ii)	-	279.0991	3.6156	neg	M-H2O-H, M+	C12H14N2O3	0	46.5	1.76638101	2504-22-5	0.008489338
neg_705	Galactosylhydroxylysine	metab_11931	HMDB0246151;HMDB0242167;HMDB0000600	B(ii)	C05547	359.1254	3.8283	neg	M+Cl, M+K-2H	C12H24N2O8	0	48	8.50934736	32448-36-5	0.008593163
neg_712	Secologanate	metab_11939	HMDB0304481	B(ii)	C01957	419.122	3.9755	neg	M-H, M+FA-H	C16H22O10	0	60.5	6.72975794	-	0.008666845
neg_722	Inosine 2'-phosphate	metab_11950	HMDB0011681	B(ii)	-	393.0448	4.1096	neg	M-H, M+FA-H	C10H13N4O8P	0	36.1	-1.4652288	-	0.016973676
neg_754	L-Asparagine	metab_11985	HMDB0000168;HMDB0251512;PW_C000110;MJDBO	B(i)	C00152	131.0456	0.5597	neg	M-H2O-H, M-	C4H8N2O3	73.9	0	-4.4382073	70-47-3	0.003975438
neg_769	3,4,5-trihydroxy-6-({7-oxo-7H-furo[3,2-g]	metab_12001	HMDB0129424	B(ii)	-	377.0495	4.8929	neg	M-H, M+Na-2	C17H14O10	0	31	-5.1740266	-	0.017681042
neg_771	N-Acetyl-D-tryptophan	metab_12004	HMDB0255052	B(ii)	C03137	245.0933	4.9071	neg	M-H, M+Na-2	C13H14N2O3	0	60	0.53022801	2280-1-5	0.008171947
neg_774	4-Hydroxystyrene	metab_12007	HMDB0004072	B(ii)	C05627	165.0552	4.8586	neg	M-H, M+FA-H	C8H8O	0	48.4	-4.6498929	2628-17-3	0.003348826
neg_787	2,3-Dihydroxypropanamide	metab_12021	HMDB0245395	B(ii)	-	140.0112	0.5597	neg	M-H, M+Cl	C3H7NO3	0	32.5	-7.1521072	-	0.003364887
neg_798	2-Amino-3-Methylsuccinic Acid	metab_12031	HMDB0000191	B(i)	C00049	146.0453	0.5753	neg	M-H2O-H, M-	C5H9NO4	90.7	0	-4.154824	56-84-8	0.004752978
neg_800	Oleuropein	metab_12035	HMDB0035872;MJDBOTE0001617	B(ii)	C09794	539.1763	5.4541	neg	M-H, M+Na-2	C25H32O13	0	59.7	-1.2495961	32619-42-4	0.005343455
neg_808	Aesculin	metab_12043	HMDB0030820	B(ii)	C09264	321.0612	0.5518	neg	M-H2O-H, M-	C15H16O9	0	52.4	-1.0774998	531-75-9	0.158077036
neg_817	(S)-10,16-Dihydroxyhexadecanoic acid	metab_12052	HMDB0037798	B(ii)	C08285	287.2229	6.3825	neg	M-H2O-H, M-	C16H32O4	0	75.3	0.53959443	69232-67-3	0.00139345
neg_819	13(S)-Hydroperoxylinolenic acid	metab_12054	HMDB0301803	B(ii)	C04785	291.1968	6.3745	neg	M-H2O-H, M+	C18H30O4	0	34.6	0.77591002	67597-26-6	0.00555961
neg_820	LysoPC(20:5(5Z,8Z,11Z,14Z,17Z)/0:0)	metab_12056	HMDB0010397	B(ii)	C04230	586.3161	6.3985	neg	M-H2O-H, M+	C28H48NO7P	0	44.9	1.95956843	-	0.015241226
neg_874	Alpha-Hydroxyisobutyric Acid	metab_12115	HMDB0242161;HMDB0000729;MJDBOTE0000667	B(i)	-	103.0394	0.3583	neg	M-H	C4H8O3	71.1	0	-6.7425992	594-61-6	0.003928603
neg_930	(2R)-2-Acetamido-6-hydroxy-2-(sulfanylm	metab_12175	HMDB0257715	B(ii)	-	268.0429	0.488	neg	M+Cl	C9H15NO4S	0	45.1	5.78074996	-	0.006344081
neg_961	Arkofix	metab_12206	HMDB0248602	B(ii)	-	215.0074	0.5359	neg	M+K-2H	C5H10N2O5	0	38	-0.9177677	-	0.014563448
neg_981	Histamine	metab_12226	HMDB0000870;PW_C000693;MJDBOTE0000422	B(i)	C00388	110.0717	0.5518	neg	M-H	C5H9N3	89.2	0	-5.7575567	51-45-6	0.00213376
neg_982	Urocanic Acid	metab_12227	PW_C000205;HMDB0062562;HMDB0000301;MJDBO	B(i)	C00785	137.0351	0.5518	neg	M-H	C6H6N2O2	93.8	0	-4.3467818	104-98-3;346	0.004383912
neg_986	O-Phospho-L-serine	metab_12231	HMDB0001721;MJDBOTE0000487	B(i)	C01005	184.0013	0.5597	neg	M-H	C3H8NO6P	80.7	0	-1.7587305	17885-08-4;4	0.045304026
neg_989	N-Gluconyl ethanolamine	metab_12234	HMDB0032293	B(ii)	-	284.0992	0.5597	neg	M+FA-H	C8H17NO7	0	43.7	1.9210281	686298-93-1	0.005678572
neg_4493	2-Hydroxycarbamazepine	metab_12246	PW_C040553;HMDB0060651	B(ii)	C16576	549.1744	3.5593	neg	2M+FA-H	C15H12N2O2	0	48.8	-7.0655782	68011-66-5	0.016176604
neg_1439	3-Hydroxybutanoic Acid	metab_12247	HMDB0000442;HMDB0000011;MJDBOTE0001066	B(i)	C01089	103.0394	1.8356	neg	M-H	C4H8O3	67.4	0	-6.6721696	6168-83-8;62	0.001558191
neg_2233	3-Methyl-2-Oxovaleric Acid	metab_12248	HMDB0000491;PW_C000373	B(i)	C00671	129.0551	3.8998	neg	M-H	C6H10O3	52	0	-4.6490678	24809-08-3;1	0.005470229
neg_4011	4-Nitrophenol	metab_12249	HMDB0041950;HMDB0001232;MJDBOTE0000622	B(i)	C00870	138.0191	5.493	neg	M-H	C6H5NO3	95	0	-4.389269	98-95-3;100-	0.012732527
neg_3600	9,10-Dihydroxystearic acid	metab_12250	HMDB0303981;HMDB0302281;LMFA02000142	B(ii)	C19622	315.2541	6.6617	neg	M-H	C18H36O4	0	50.6	0.12258887	120-87-6	0.00693159
neg_5734	Angelicin	metab_12251	HMDB0033930;HMDB0000852;MJDBOTE0000961	B(ii)	C09060	231.0317	0.8775	neg	M+FA-H	C11H6O3	0	36.9	9.78589381	523-50-2;83-	0.006980174
neg_1004	Aspartic Acid	metab_12252	HMDB0000191;HMDB0006483;MJDBOTE0000530	B(i)	C00049	132.0296	0.5753	neg	M-H	C4H7NO4	88.9	0	-4.6304732	1783-96-6;61	0.006154335
neg_788	Cystathionine	metab_12253	HMDB0303077;HMDB0000099	B(i)	C02291	221.06	0.5675	neg	M-H, M+K-2H	C7H14N2O4S	57.1	0	-0.4624607	535-34-2;56-	0.00723991
neg_5797	Cytidine monophosphate	metab_12254	PW_C000064;HMDB0240312;HMDB0000095	B(ii)	C00055;C05822	691.1056	0.7495	neg	2M+FA-H	C9H14N3O8P	0	45.7	5.68871972	63-37-6;84-5	0.078198429
neg_5707	D-Galactose	metab_12255	HMDB0250761;HMDB0000143;HMDB0003449;PW_C	B(i)	C00124	201.0377	0.9253	neg	M+Na-2H	C6H12O6	40.6	0	-1.8815372	59-23-4;1025	0.024610792
neg_5942	D-Glucuronic Acid	metab_12256	HMDB0006355;PW_C000082;HMDB0000127;MJDBO	B(i)	C00191	193.0353	0.6223	neg	M-H	C6H10O7	86.3	0	-0.571347	6556-12-3;32	0.004974947
neg_167	D-Glycerate 3-phosphate	metab_12257	HMDB0060180;HMDB0000807	B(i)	C00197	184.9852	0.6461	neg	M-H2O-H, M-	C3H7O7P	51.8	0	-2.4559134	820-11-1;344	0.008648563
neg_4331	D-Tartaric Acid	metab_12258	HMDB0029878;HMDB0000956;MJDBOTE0000633	B(i)	C00898	149.0086	4.1334	neg	M-H	C4H6O6	64.9	0	-3.9910992	87-69-4;147-	0.009047743
neg_1197	GDP-Glucose	metab_12259	HMDB0003351;HMDB0001163	B(i)	C00394	604.0718	0.949	neg	M-H	C16H25N5O16P2	74.9	0	3.18357055	5750-57-2;31	0.119629947
neg_5980	Glycerol 3-Phosphate	metab_12260	HMDB0252849;HMDB0000126;PW_C000081	B(i)	C00093	171.0059	0.5828	neg	M-H	C3H9O6P	79.1	0	-3.0595465	57-03-4;1798	0.004857316
neg_3675	Hepoxilin A3	metab_12261	LMFA03090005;HMDB0062619;HMDB0004688	B(ii)	C14808	317.2123	6.3825	neg	M-H2O-H	C20H32O4	0	78.9	0.1520147	85589-24-8	0.012194124
neg_3677	Hexadecanedioic acid	metab_12262	LMFA01170022;HMDB0000712;HMDB0304377;HMDI	B(ii)	C19615	285.2073	6.3825	neg	M-H	C16H30O4	0	67	0.43086618	42150-38-9;5	0.000264671
neg_28	Homogentisic Acid	metab_12263	HMDB0001336;PW_C000084;HMDB0000130;MJDBO	B(i)	C00544	167.0345	2.7453	neg	M-H, 2M-H, M	C8H8O4	83.7	0	-3.104031	102-32-9;451	0.009023542
neg_4759	Kaempferol	metab_12264	LMPK12110003;HMDB0005801;HMDB0032008;MJDB	B(ii)	C05903	331.0463	2.9646	neg	M+FA-H	C15H10O6	0	46.4	1.29806685	520-18-3;807	0.019728404
neg_1574	Kynurenine	metab_12265	HMDB0000684;MJDBOTE0001164	B(i)	C00328	207.0773	2.2174	neg	M-H	C10H12N2O3	89.8	0	-1.2415889	2922-83-0;34	0.004574242
neg_16	L-Lactic acid	metab_12266	HMDB0001311;HMDB0000190;PW_C000122	B(ii)	C00186	225.0614	0.6065	neg	2M+FA-H, 2M-	C3H6O3	0	51.6	-1.051831	79-33-4;1032	0.007073418
neg_202	Malic acid	metab_12267	PW_C000592;HMDB0000156	B(i)	C00149	133.0137	0.7655	neg	M-H2O-H, M-	C4H6O5	85.1	0	-4.3910609	6915-15-7;97	0.00557948
neg_5780	Malonic Acid	metab_12268	PW_C000543;HMDB0000691;LMFA01170041	B(i)	C00383	103.003	0.7734	neg	M-H	C3H4O4	38.8	0	-7.0234275	141-82-2	0.021198818
neg_5	Maltotriose	metab_12269	HMDB0001262;HMDB0038852;MJDBOTE0000606	B(i)	C01835	539.1397	0.6461	neg	M+Cl, 2M-H, M	C18H32O16	91.9	0	2.59790533	6118-87-2;11	0.002387422
neg_4664	N-Acetylneuraminic acid	metab_12270	PW_C000155;HMDB0000230;HMDB0246275;HMDB0	B(ii)	C00270	290.0885	3.0846	neg	M-H2O-H	C11H19NO9	0	42.4	1.14184977	131-48-6;193	0.002149729
neg_1210	NADP	metab_12271	HMDB0000221;PW_C000143;HMDB0000217	B(ii)	C00006	742.0705	0.9887	neg	M-H	C21H28N7O17P3	0	96.5	3.16148497	53-59-8;53-5	0.10405209
neg_926	Necatorine	metab_12272	HMDB0039035;HMDB0031330	B(ii)	C07561	299.0229	0.4835	neg	M+Cl	C15H8N2O3	0	62.2	0.07636071	89915-35-5;5	0.024122507
neg_5928	Oxalic Acid	metab_12273	HMDB0040196;HMDB0302914;HMDB0303019;HMDE	B(i)	C00209	88.9872	0.6382	neg	M-H	C2H2O4	38	0	-9.0617028	2050-60-4;14	0.003250976
neg_1935	Phenylacetylglutamine	metab_12274	HMDB0062645;HMDB0006344;PW_C002728;MJDBO	B(ii)	C04148	301.057	2.9487	neg	M+K-2H	C13H16N2O4	0	58.5	-9.9821139	28047-15-6	0.015701411
neg_2805	Prostaglandin J2	metab_12275	PW_C001687;HMDB0004238;HMDB0247117;HMDB0	B(ii)	C05957	379.2106	6.3025	neg	M+FA-H	C20H30O4	0	44.2	-6.0182928	60203-57-8;8	0.004229174
neg_5588	Pyrocatechol	metab_12276	PW_C000764;HMDB0000957;MJDBOTE0000665	B(i)	C00090	109.0289	1.243	neg	M-H	C6H6O2	85.7	0	-5.8330941	120-80-9	0.029308358
neg_4181	Quercetin 3-O-sophoroside	metab_12277	HMDB0304729	B(ii)	C08549	289.0832	4.7463	neg	M-H2O-H	C14H16N2O6	0	67.3	0.78880882	18609-17-1	0.009056817
neg_33	Riboflavin	metab_12278	HMDB0247501;HMDB0001520;PW_C000165;HMDB0	B(i)	C00255	375.1317	3.8077	neg	M+Cl, M+FA-H	C17H20N4O6	98.1	0	1.84928457	83-88-5;146-	0.015419715
neg_1662	Trans-Cinnamic Acid	metab_12279	HMDB0000567;HMDB0000930;MJDBOTE0000400	B(i)	C00423	147.0445	2.4569	neg	M-H	C9H8O2	34.4	0	-4.1330469	140-10-3;102	0.020709708
neg_5746	UDP-D-Galactose	metab_12280	HMDB0000302;HMDB0012305	B(i)	C00052	565.0493	0.8451	neg	M-H	C15H24N2O17P2	91.9	0	2.72979054	1955-26-6;29	0.006255447
